# Next Generation Sequencing Analysis of Human Platelet PolyA+ mRNAs and rRNA-Depleted Total RNA

**DOI:** 10.1371/journal.pone.0081809

**Published:** 2013-12-11

**Authors:** Antheia Kissopoulou, Jon Jonasson, Tomas L. Lindahl, Abdimajid Osman

**Affiliations:** Department of Clinical and Experimental Medicine, University of Linköping, Linköping, Sweden; Duke-NUS, Singapore

## Abstract

**Background:**

Platelets are small anucleate cells circulating in the blood vessels where they play a key role in hemostasis and thrombosis. Here, we compared platelet RNA-Seq results obtained from polyA+ mRNA and rRNA-depleted total RNA.

**Materials and Methods:**

We used purified, CD45 depleted, human blood platelets collected by apheresis from three male and one female healthy blood donors. The Illumina HiSeq 2000 platform was employed to sequence cDNA converted either from oligo(dT) isolated polyA+ RNA or from rRNA-depleted total RNA. The reads were aligned to the GRCh37 reference assembly with the TopHat/Cufflinks alignment package using Ensembl annotations. A *de novo* assembly of the platelet transcriptome using the Trinity software package and RSEM was also performed. The bioinformatic tools HTSeq and DESeq from Bioconductor were employed for further statistical analyses of read counts.

**Results:**

Consistent with previous findings our data suggests that mitochondrially expressed genes comprise a substantial fraction of the platelet transcriptome. We also identified high transcript levels for protein coding genes related to the cytoskeleton function, chemokine signaling, cell adhesion, aggregation, as well as receptor interaction between cells. Certain transcripts were particularly abundant in platelets compared with other cell and tissue types represented by RNA-Seq data from the Illumina Human Body Map 2.0 project. Irrespective of the different library preparation and sequencing protocols, there was good agreement between samples from the 4 individuals. Eighteen differentially expressed genes were identified in the two sexes at 10% false discovery rate using DESeq.

**Conclusion:**

The present data suggests that platelets may have a unique transcriptome profile characterized by a relative over-expression of mitochondrially encoded genes and also of genomic transcripts related to the cytoskeleton function, chemokine signaling and surface components compared with other cell and tissue types. The *in vivo* functional significance of the non-mitochondrial transcripts remains to be shown.

## Background

**Figure 1:**
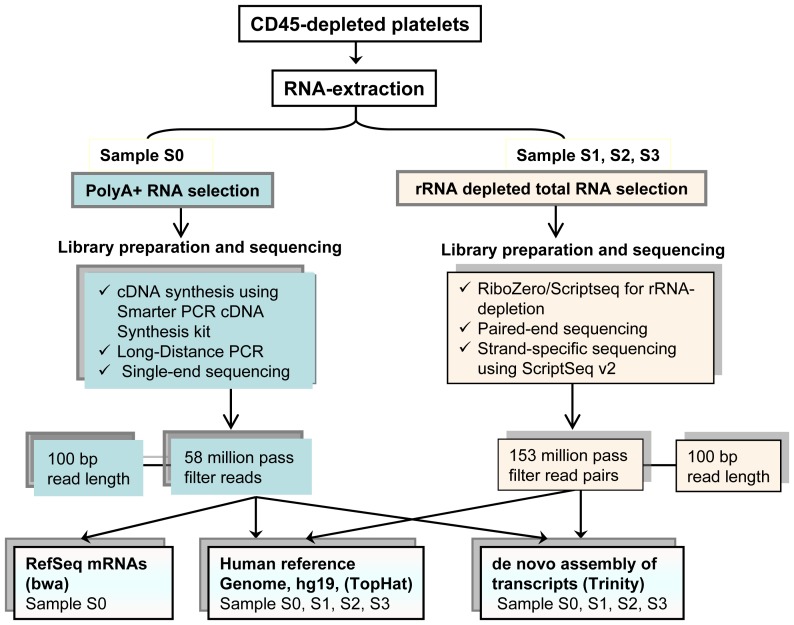
Schematic presentation of experimental plan used in this study. Samples from 4 platelet donors were investigated. One sample (S0) was used for isolation of polyA+ transcripts. The 3 other samples (S1, S2, and S3) were used for analysis of total RNA after depletion of ribosomal RNA (rRNA).

Produced by bone marrow megakaryocytes, platelets are small anucleate elements of the blood that play a pivotal role in hemostasis. They are involved in fibrinolysis and repair of the vessel wall, while circulating in the blood as sentinels of vascular integrity. Platelets lack genomic DNA but retain the ability for protein synthesis from cytoplasmic mRNA [1]. Platelet mRNA was first isolated and converted to a cDNA library more than two decades ago [2]. In recent years, several studies utilizing genome-wide techniques for gene expression profiling, such as microarrays and Serial Analysis of Gene Expression (SAGE) in concert with computer-assisted bioinformatics, have reported that thousands of gene transcripts are present in human platelets [3]–[7]. While microarrays and SAGE have made significant contributions to the characterization of the platelet transcriptome, they also have serious limitations. Hybridization-based approaches rely on probe-target binding of selected sequences and do not detect novel transcripts or unknown genes. In contrast, SAGE uses sequence tags from individual mRNAs and has an advantage over microarrays by detecting unknown genes but does not provide information on splice isoforms and is biased toward short tags, which cannot be uniquely mapped to the human genome [8]. Recently, mass sequencing of transcripts (RNA-Seq) by next generation sequencing (NGS) technologies has emerged as a powerful approach for quantitative transcript discovery [9]–[13]. RNA-Seq has clear advantages over other approaches [14] and shows higher levels of reproducibility for both technical and biological replicates [15]. Two recently published studies used NGS technology to characterize the platelet transcriptome [16]–[17]. One of these used cDNA from poly(dT) isolated mRNA and the other cDNA from ribosomal RNA-depleted total RNA. Both studies used relatively short reads (≤50 base pairs) for alignment to the human genome. In this context, we now report results from both polyA+ mRNA and rRNA-depleted total RNA approaches utilizing 100 bp long sequencing reads for investigating the transcriptional profile of unstimulated human platelets (Fig. 1). We have also for the first time applied a *de novo* assembly of platelet transcripts to confirm the reference-guided alignments. We believe that our data may provide important clues for understanding the elusive platelet transcriptome and its role in the coagulation system and hemostasis.

## Results

### Mapping of polyA+ mRNA (Sample S0)

**Figure 2:**
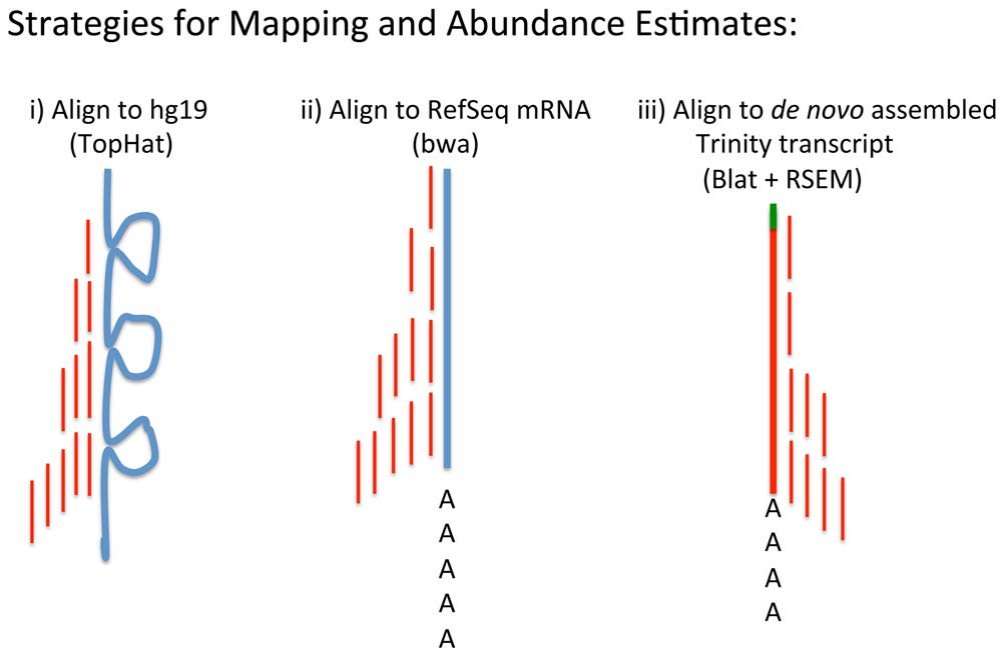
Mapping strategies and abundance estimates. i) Alignment of reads (short red lines) to the human reference genome hg19 (thick blue line) using the TopHat program that aligns RNA-Seq reads to the genome while also attending to splice junction reads. Abundance estimates obtained by counting the number of reads that map within the coordinates defining the corresponding gene with RefSeq annotations; ii) Alignment of reads (short red lines) to human reference (RefSeq) mRNA (thick blue line with polyA tail) using the bwa software for abundance estimates; iii) Alignment of reads (short red lines) to a *de novo* assembled transcript reported by Trinity (thick red line with polyA tail and green SMARTer IIA oligonucleotide as 5′-leader sequence) using Blat for identification and RSEM for abundance estimates.

We tried three mapping strategies for polyA+ mRNA (Fig. 2).

**Figure 3:**
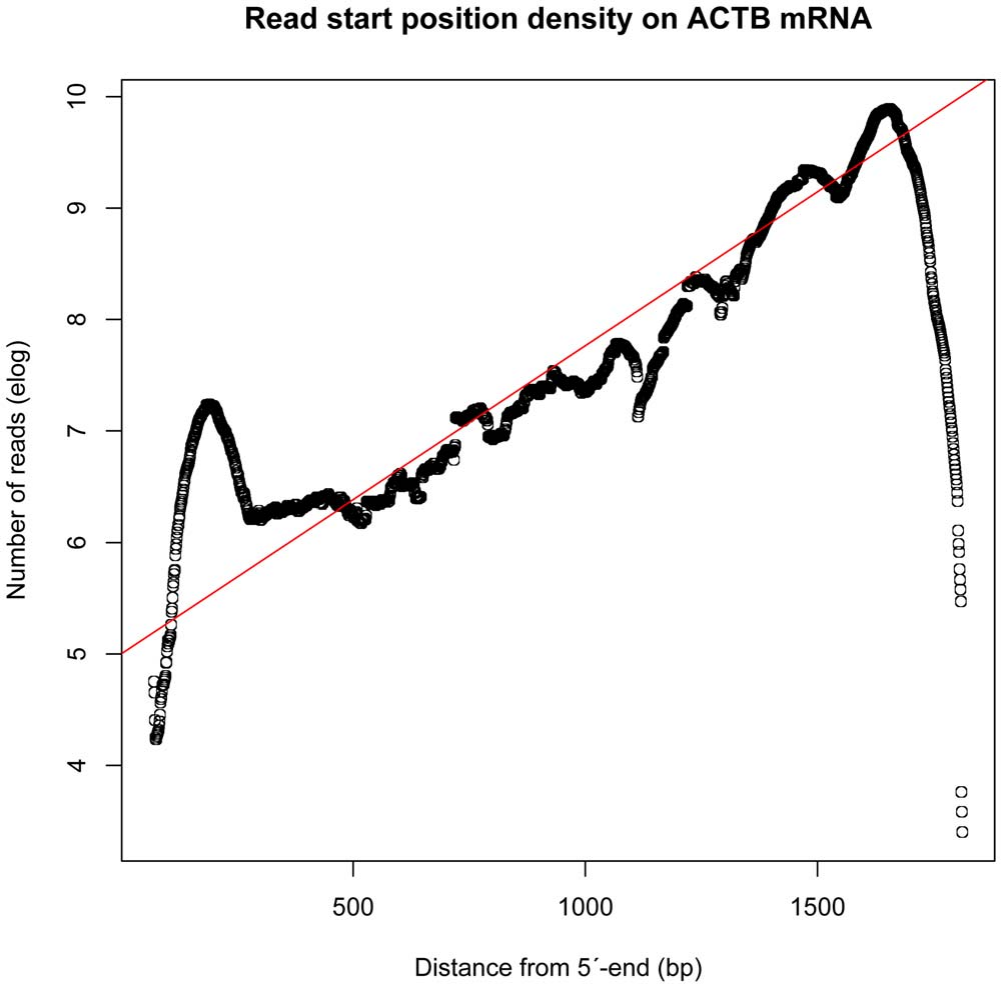
Read start position density on *ACTB* mRNA. The horizontal axis shows the distance in nucleotides (bp) from the 5′-end of *ACTB* mRNA, and the vertical axis shows the natural logarithm of the number of uniquely mapped reads. The fitted red line calculated over the transcript body ignoring both ends corresponds to exponential decay of approximately 50% per 250 bp upstreams fom the polyA-site in the 3′-UTR. Correlation coefficient: 0.93, Slope: 0.0027638, Std error: 0.0002751, t value: -10.05, p-value: 4.70e-08 ***. (Statistics and graph generated by the R-program).

First, the 58,155,680 cleaned sequenced single-end reads with no strand-specificity were mapped to the human reference genome (GRCh37/hg19) using TopHat software (http://tophat.cbcb.umd.edu/) in order to identify exon-exon splice junctions (Fig. 2 i). This resulted in 35,322,009 (60.7%) of uniquely mapped ∼100 bp long single-end reads. The aligned sequencing reads and the Homo_sapiens.GRCh37.71.gtf features were used to estimate the coverage of known genes and transcripts with the aid of bedtools-2.17.0 (http://code.google.com/p/bedtools/). A strong bias towards the 3′-UTR end of transcripts was clearly evident, which can be expected due to the library construction involving oligo-dT primed cDNA in the library preparation procedure (Fig. 3). The uniquely mapped read localizations on the different chromosomes are shown in Table 1. Top 30 loci are shown in Table 2. The HTSeq counts are shown in Table S1 in File S1.

**Table 1:** Distribution of mapped reads for samples S0, S1, S2 and S3.

Transcript info		No. Mapped reads per sample			
Chr	Length (bp)	S0	S1	S2	S3
1	249250621	1417879	5604828	6082062	5836619
2	243199373	839205	4828700	4942088	5293788
3	198022430	376751	4053306	3561770	5497609
4	191154276	1574321	5304616	5313861	8582878
5	180915260	987877	2476361	2751025	2864982
6	171115067	510420	3116547	3325548	3279675
7	159138663	534153	2881230	2900407	3260889
8	146364022	202649	1788536	1718024	1966160
9	141213431	182805	2898002	2193803	4043810
10	135534747	228845	2687946	2569453	2952239
11	135006516	915079	2259841	1890737	2206632
12	133851895	396073	2337950	2332903	2698125
13	115169878	733311	1026903	1066507	1135743
14	107349540	165457	6691970	4061007	12098431
15	102531392	1039909	5280432	5780695	5035062
16	90354753	168779	946256	807193	892257
17	81195210	440380	1934478	1881603	2076841
18	78077248	92840	1184820	1263978	1233671
19	59128983	257403	860224	632489	900418
20	63025520	425316	1327116	1295269	1404632
21	48129895	92487	741307	599255	624149
22	51304566	148511	750870	567420	726189
X	155270560	1153342	7951615	6776104	6929134
Y	59373566	21311	51658	4949	50979
MT	16569	22416906	6781716	9016861	7198049
Sum:		35322009	76000000	73335011	88788961

**Table 2:** TopHat alignment of PolyA + mRNA to genome.

Ensembl id.	Gene	Locus	NRC*	Rank
ENSG00000210082	MT-RNR2	MT:1671–3229	10000000	MT
ENSG00000211459	MT-RNR1	MT:648–1601	5000000	MT
ENSG00000205542	TMSB4X	X:12993226–12995346	1000000	1
ENSG00000166710	B2M	15:45003674–45011075	862880	2
ENSG00000198888	MT-ND1	MT:3306–4262	833782	MT
ENSG00000163736	PPBP	4:74852754–74853914	555955	3
ENSG00000198712	MT-CO2	MT:7585–8269	534277	MT
ENSG00000163737	PF4	4:74844540–74848796	437842	4
ENSG00000198763	MT-ND2	MT:4469–5511	407889	MT
ENSG00000198886	MT-ND4	MT:10469–12137	355599	MT
ENSG00000198899	MT-ATP6	MT:8365–9990	303773	MT
ENSG00000198938	MT-CO3	MT:8365–9990	303743	MT
ENSG00000198786	MT-ND5	MT:12336–14148	287825	MT
ENSG00000198804	MT-CO1	MT:5903–7445	282378	MT
ENSG00000198727	MT-CYB	MT:14746–15887	217548	MT
ENSG00000187514	PTMA	2:232571605–232578251	210648	5
ENSG00000161570	CCL5	17:34195970–34212867	185274	6
ENSG00000228474	OST4	2:27265231–27294641	180079	7
ENSG00000198695	MT-ND6	MT:14148–14673	148341	MT
ENSG00000198840	MT-ND3	MT:10058–10404	105928	MT
ENSG00000212907	MT-ND4L	MT:10469–12137	98894	MT
ENSG00000075624	ACTB	7:5566781–5603415	91079	8
ENSG00000127920	GNG11	7:93220884–93567791	85225	9
ENSG00000204592	HLA-E	6:30457244–30461982	82263	10
ENSG00000087086	FTL	19:49468558–49470135	81047	11
ENSG00000158710	TAGLN2	1:159887897–159895522	77614	12
ENSG00000120885	CLU	8:27454434–27472548	72310	13
ENSG00000168497	SDPR	2:192699027–193060435	71863	14
ENSG00000150681	RGS18	1:192127586–192154945	65222	15
ENSG00000163041	H3F3A	1:226249552–226259702	63326	16

*NRC =  Normalized Read Counts calculated from transcript length (x) as NRC =  read_count*(1+e-0.0027638x).

**Figure 4:**
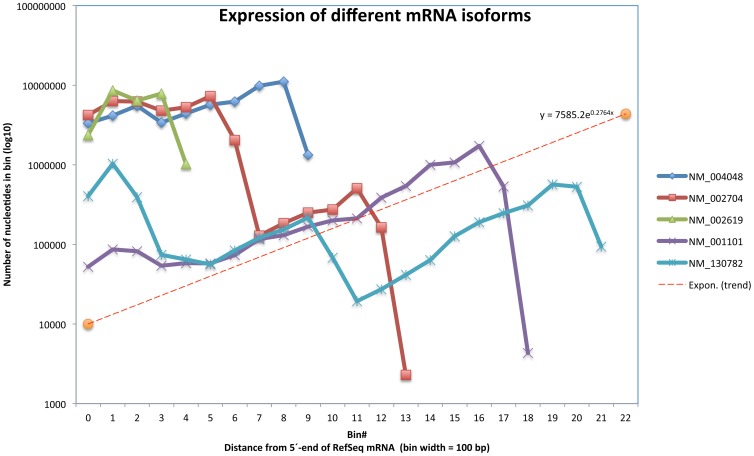
Mapping of S0 (poly(dT) selected transcripts) against RefSeq mRNA. The horizontal axis shows the distance in nucleotides from the 5′-end of the transcript (bin length  = 100 bp), and the vertical logarithmic axis shows the sum of uniquely mapped reads to each position of the bin. The slope of the dotted line corresponds to the exponential decay function derived in Fig. 3. The sudden “drops” correspond to polyA-sites. As seen in the figure NM_002704 (*PPBP*) has two polyA-sites which correspond to the known polyA-sites at positions 708 and 1307, respectively. The abundance of the longer *PPBP* transcript appears to be hundred-fold lower than that of the shorter transcript.

Second, to check the quality of the TopHat alignments the reads were also mapped against RefSeq mRNAs (Fig. 2 ii) using bwa (http://bio-bwa.sourceforge.net/) and samtools (http://samtools.sourceforge.net/) giving similar results (data not shown). PolyA-sites and the expression level of individual transcripts were visualized by plotting log coverage against the distance from the 5′-end of the RefSeq mRNA sequences (Fig. 4). Additional data is shown in Table S2 in File S1.

**Figure 5:**
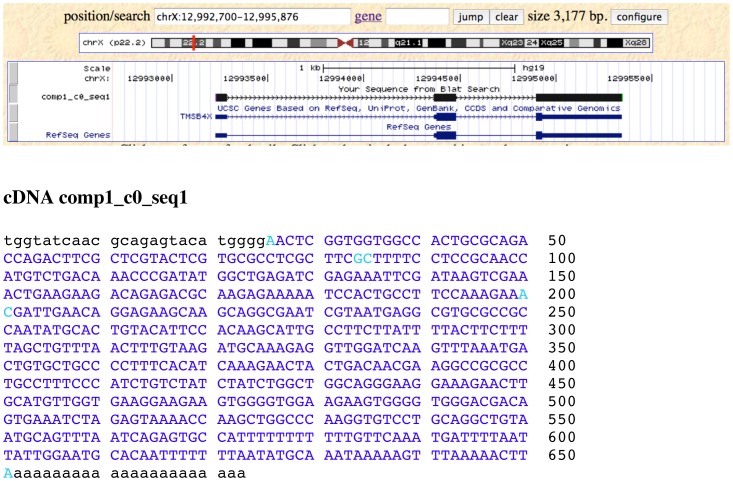
Snapshot of UCSC Browser Blat alignment of *de novo* assembled transcript variant comp1_c0_seq1 mapping to TMSB4X. The 5′-leader sequence matches the SMARTer IIA oligonucleotide. The Trinity *de novo* assembled nucleotide sequence is identical to the GRCh37/hg19 reference. Part of the polyA tail is also included. Splice junctions are marked in turquoise.

Finally, a detailed analysis of transcripts and assignment of mRNA isoforms was performed by *de novo* assembly of transcripts using Trinity RNA-Seq software from (http://trinityrnaseq.sourceforge.net/) followed by quantification of transcripts with RSEM (RNA-Seq by Expectation-Maximization) (Fig. 2 iii). Identification of the *de novo* assembled transcripts was achieved by Blat and BLAST searches using the UCSC Browser and the NCBI Genome databases, respectively. Table 3a shows the Top 25 out of 9077 reported *de novo* assembled polyA+ genomic transcripts with identification to locus; excluding the mitochondrial genome for clarity. Full-length transcripts could be identified by the presence of a SMARTer IIA 5′-leader sequence and a 3′-end polyA tail (Fig 5). The magnitude of expression of *de novo* assembled polyA+ transcripts correlated well (Spearman's rho  = 0.83) with the length normalized coverage by TopHat alignment of polyA+ cDNA reads to the human genome (compare Tables 2 and 3a).

**Table 3:** de novo assembly of platelet transcripts.

Table 3a. Trinity/RSEM					Table 3b. Trinity/RSEM				
de novo assembly of PolyA+ mRNA transcripts (MT-RNA excluded)					de novo assembly of rRNA-depleted total RNA transcripts (MT-RNA excluded)				
Rank	Gene	Length	FPKM¶	NRC*	Rank§	Gene	Length_mean	FPKM¶ _mean	FPKM_sd
1	TMSB4X	673	67846	1399035	ncrna	7SLRNA	349	295364	120832
2	B2M	992	28110	827815	1	TMSB4X	646	145005	15806
3	PPBP	1789	10067	524473	2	B2M	990	64093	8783
4	PF4	1035	13004	398344	3	PPBP	2296	22402	12048
5	OST4	470	19998	291396	ncrna	7SK-RNA	330	9981	2495
6	CCL5	777	6289	148021	ncrna	LSU-rRNA	1032	8029	6510
7	FTH1	961	5068	144939	4	FTH1	955	8008	1385
8	SERF2	598	6988	129001	ncrna	RNA45S5	307	6586	4582
9	PTMA	1036	4101	125730	5	PF4	1201	6367	3351
10	H3F3B	1087	3849	123435	6	SMARCA5	510	5991	1417
11	SH3BGRL3	781	4037	95446	7	OST4	492	5765	1800
12	ACTB	974	3058	88556	8	PF4V1	530	3609	505
13	FTL	914	3053	83361	9	C21orf7	1527	3207	912
14	TAGLN2	1414	1866	76941	10	ACTB	1680	2991	637
15	GNG11	876	2804	73644	11	MYL6	700	2705	678
16	PTMA	320	6581	62019	12	CCL5	777	2569	370
17	RGS18	4238	485	61271	13	GNG11	1004	2493	1019
18	C21orf7	1518	1341	59276	14	HSMAR1	1756	2456	344
19	SDPR	2554	774	58065	15	RGS18	4443	2432	160
20	TUBB1	3109	612	56270	16	H3F3A	588	2370	736
21	MYL6	696	2509	53370	17	HIST1H2AC	1744	2280	424
22	CLU	1769	939	48350	18	MYL12A	1316	2278	552
23	HLA-E	1492	1025	44539	19	EFCAB13	608	2234	704
24	GPX1	907	1301	35274	20	MORC3	714	2225	834
25	RGS10	1001	1136	33743	21	PTMA	1232	2179	667

¶ Fragments Per Kilobase of transcript per Million mapped reads.

*NRC =  Normalized Read Count calculated from transcript length (x) as NRC =  read_count*(1+e-0.0027638x).

§ Ranking of protein coding transcripts only.

### Mapping of rRNA-depleted total RNA (Samples S1, S2 and S3)

**Figure 6:**
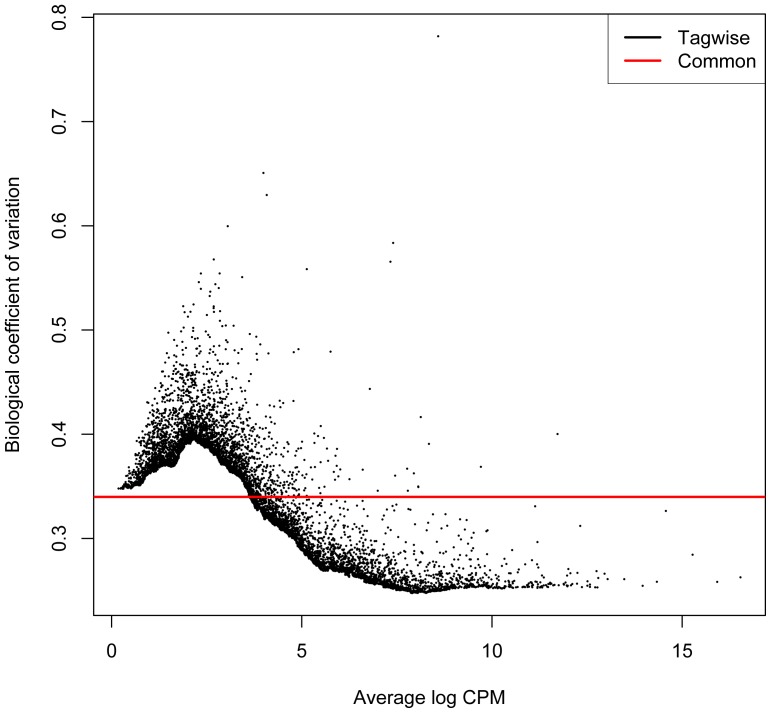
Biological coefficient of variation of samples S1, S2 and S3 as estimated by TopHat/HTSeq/edgeR software. As expected the more highly expressed genes show much lower dispersion estimates than the mean value. “CPM” represents counts per million.

**Figure 7:**
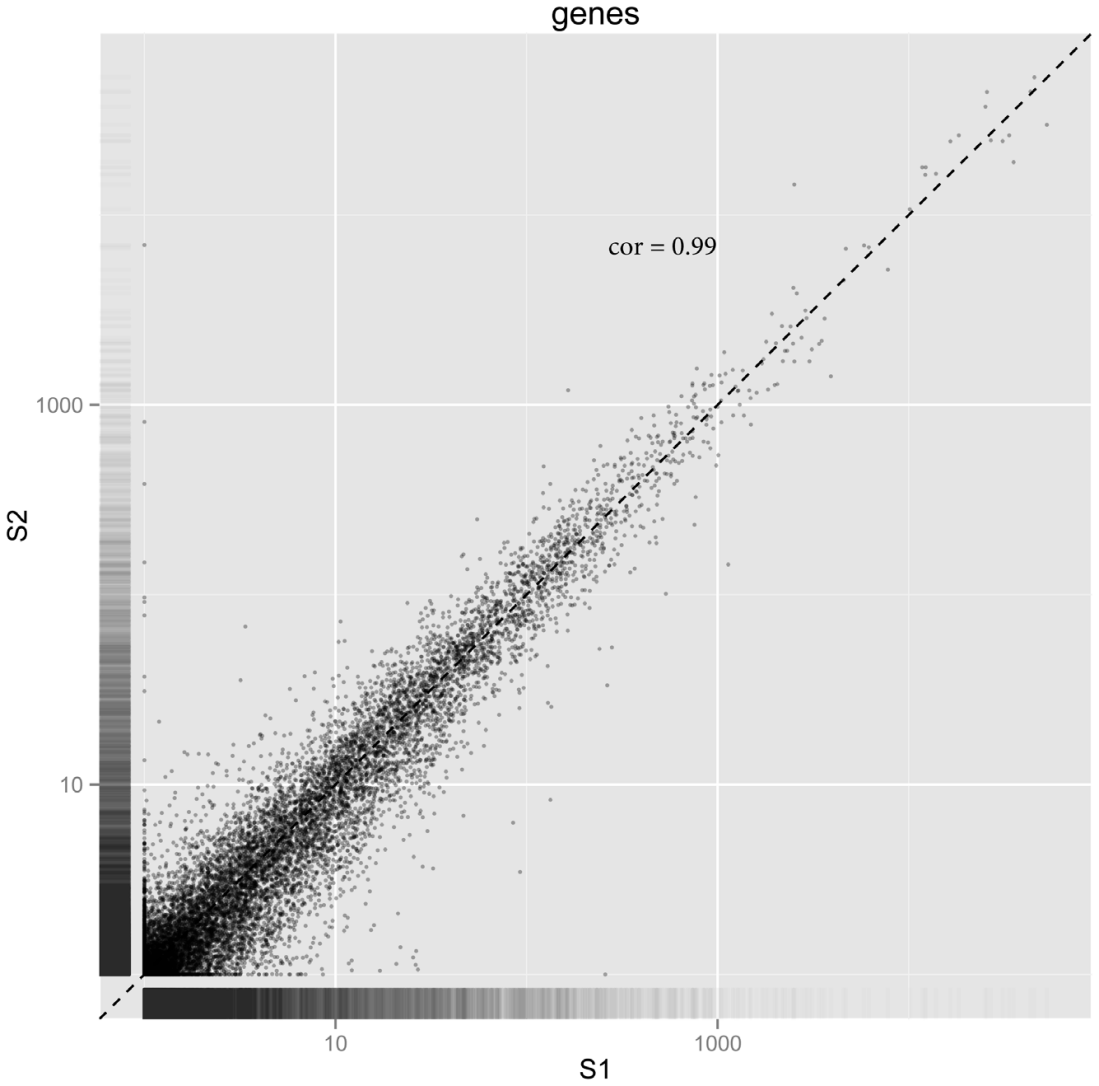
Plot showing the magnitude of FPKM gene expression in rRNA-depleted total RNA in pair-wise comparisons between sample S1 and sample S2. Each dot represents a S1/S2 pair for a gene that had detectable expression in both samples. Pearson's correlation coefficient  = 0.99. (TopHat/Cufflinks/Cuffdiff/CummeRbund software).

The three barcoded rRNA-depleted total RNA libraries (S1,S2 and S3) resulted in 153 million pass filter strand-specific read pairs (QC data in Fig. S1 in File S1) which were mapped to the human reference genome (GRCh37/hg19) using TopHat. The uniquely mapped read localizations on the different chromosomes are shown in Table 1. The aligned sequencing reads were assigned to the Homo_sapiens.GRCh37.71.gtf features as described above. Top 30 loci are shown in Table 4. A full table of HTSeq counts is presented in Table S1 in File S1. The biological coefficient of variation as estimated by the edgeR software (http://www.bioconductor.org/) is shown in figure 6. There was a linear dependence between FPKM (Fragments Per Kilobase of transcript per Million mapped reads)-values in samples S1, S2 and S3. Figure 7 shows a pair-wise comparison of S1 (male) and S2 (female) rendering a Pearson's correlation coefficient of 0.99. These results were confirmed by *de novo* assembly using the Trinity software (Table 3b).

**Table 4:** TopHat/Cufflinks alignment of rRNA-depleted total RNA to genome (excluding ncrna).

Ensembl id.	Gene	Locus	S1_FPKM¶	S2_FPKM¶	S3_FPKM¶	Rank
ENSG00000205542	TMSB4X	X:12993226–12995346	34973	28506	46120	1
ENSG00000163736	PPBP	4:74852754–74853914	25489	23607	37832	2
ENSG00000198804	MT-CO1	MT:5903–7445	23594	35045	27213	MT
ENSG00000198888	MT-ND1	MT:3306–4262	17087	24640	18055	MT
ENSG00000198938	MT-CO3	MT:8365–9990	16415	22715	15566	MT
ENSG00000198840	MT-ND3	MT:10058–10404	15273	22805	14332	MT
ENSG00000198886	MT-ND4	MT:10469–12137	14039	22467	9924	MT
ENSG00000198899	MT-ATP6	MT:8365–9990	12643	15608	11442	MT
ENSG00000198727	MT-CYB	MT:14746–15887	13017	15645	10847	MT
ENSG00000166710	B2M	15:45003674–45011075	9394	11022	16484	3
ENSG00000212907	MT-ND4L	MT:10469–12137	9394	18469	8991	MT
ENSG00000198786	MT-ND5	MT:12336–14148	10900	16518	8191	MT
ENSG00000198712	MT-CO2	MT:7585–8269	11460	15423	8156	MT
ENSG00000198763	MT-ND2	MT:4469–5511	11304	16506	6650	MT
ENSG00000228253	MT-ATP8	MT:8365–9990	12611	10792	9831	MT
ENSG00000163737	PF4	4:74844540–74848796	5352	5933	9990	4
ENSG00000228474	OST4	2:27265231–27294641	7326	4882	6268	5
ENSG00000180573	HIST1H2AC	6:26124372–26139344	5539	6458	3635	6
ENSG00000150681	RGS18	1:192127586–192154945	4310	6219	2841	7
ENSG00000075624	ACTB	7:5566781–5603415	3375	2548	3199	8
ENSG00000167996	FTH1	11:61717292–61735132	3044	2459	2413	9
ENSG00000198695	MT-ND6	MT:14148–14673	2741	3190	1528	MT
ENSG00000127920	GNG11	7:93220884–93567791	2369	1585	2850	10
ENSG00000168497	SDPR	2:192699027–193060435	1809	2832	2040	11
ENSG00000154146	NRGN	11:124609809–124636392	2807	1574	2154	12
ENSG00000101608	MYL12A	18:3247478–3261848	2719	1959	1755	13
ENSG00000180596	HIST1H2BC	6:26115100–26124154	2897	1822	1525	14
ENSG00000104904	OAZ1	19:2252251–2273487	2003	1762	1954	15
ENSG00000163041	H3F3A	1:226249551–226259702	1910	1233	1314	16
ENSG00000161570	CCL5	17:34195970–34212867	1437	1760	1236	17

¶ Fragments Per Kilobase of transcript per Million mapped reads.

**Figure 8:**
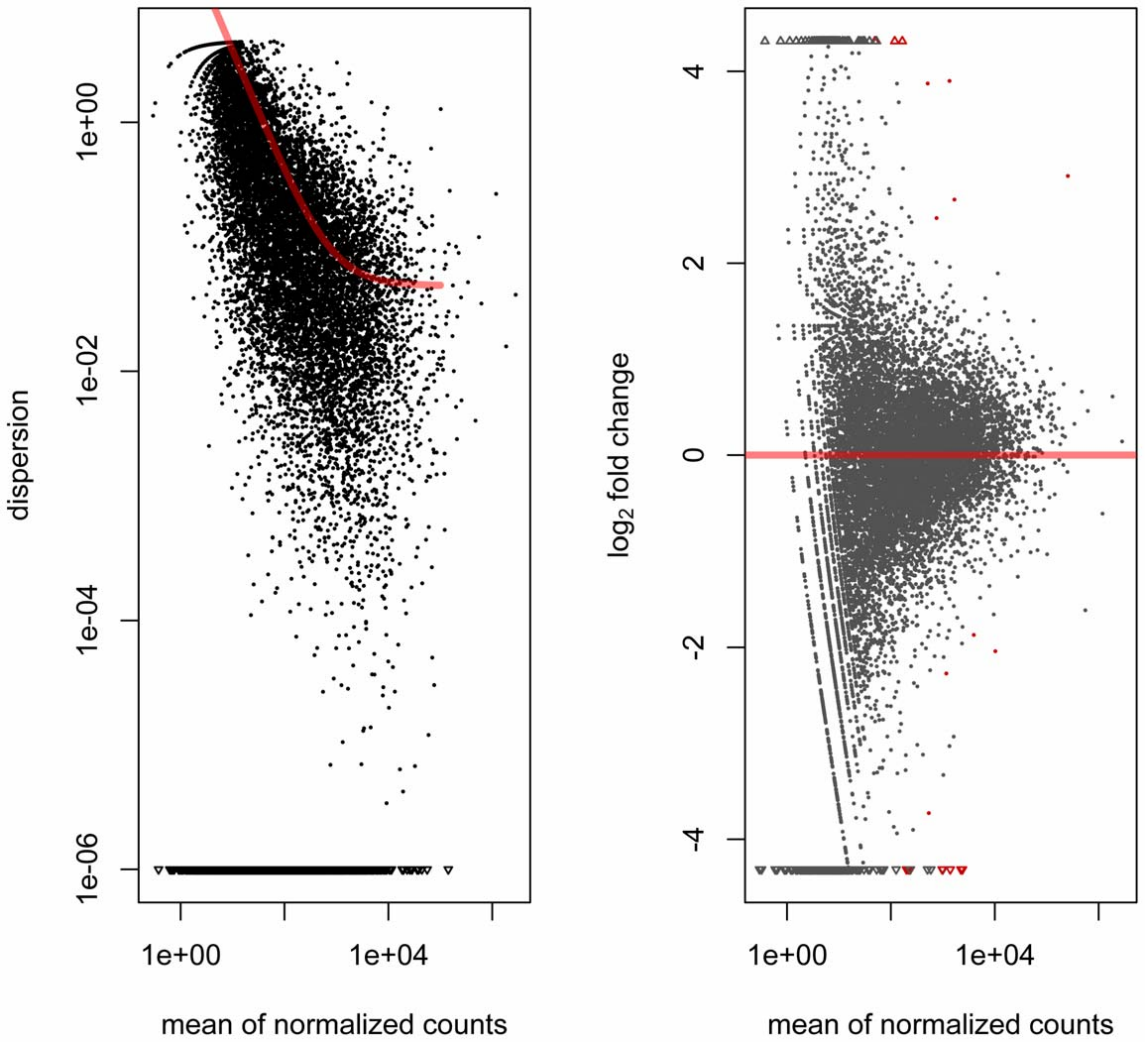
Graphs showing the dispersion and log_2_ fold change, respectively, when comparing the two male samples S1 and S3 with the female sample S2 using DESeq. The “dispersion” on the y-axis in the left-hand plot represents the square of the coefficient of biological variation, and the red “hockey-stick” line is a fitted curve through the estimates of the dispersion value for each gene. In the right-hand plot, the horizontal red line represents equal expression in male and female samples. Red dots represent differentially expressed genes at 10% FDR, and red triangles represent red dots that lie outside the graph (above or below). The identity of the differentially expressed genes and the corrresponding log_2_ fold changes can be found in Table 5 (columns 2 and 8, respectively).

Further analyses to reveal differential expression (DE) were performed with Cufflinks and the bioinformatic tools HTseq and DESeq from Bioconductor (http://www.bioconductor.org/), which uses the R statistical programming language. Figure 8 shows dispersion and log_2_ fold change when comparing the two male samples S1 and S3 with the female sample S2 using DESeq. Eighteen differentially expressed genes were identified between the two sexes at 10% false discovery rate (FDR) using DESeq (Fig. 8, red dots). Not all of these genes were located on the Y chromosome (Table 5.).

**Table 5:** Significantly differentially expressed genes in male and female platelets at 10% FDR as estimated by DESeq.

Ensembl id.	gene	locus	baseMean	baseMeanA	baseMeanB	FC*	log2 FC*	pval¶	padj§
ENSG00000183878	UTY	Y:15360259–15592553	1511.7	2265.7	3.5	0.002	−9.3	7.6E-27	1.4E-22
ENSG00000198692	EIF1AY	Y:22737611–22755040	618.0	925.3	3.5	0.004	−8.0	4.4E-19	4.0E-15
ENSG00000210082	MT-RNR2	MT:1671–3229	160159.6	50966.7	378545.5	7.427	2.9	3.5E-13	2.2E-09
ENSG00000116117	PARD3B	2:205410516–206484886	843.0	153.2	2222.5	14.51	3.9	1.2E-12	5.7E-09
ENSG00000154620	TMSB4Y	Y:15815447–15817904	1407.2	2107.9	5.6	0.003	−8.5	1.9E-12	6.9E-09
ENSG00000196565	HBG2	11:5274420–5667019	635.7	908.0	91.0	0.100	−3.3	4.1E-10	1.3E-06
ENSG00000067048	DDX3Y	Y:15016019–15032390	884.4	1323.8	5.6	0.004	−7.9	1.2E-09	3.2E-06
ENSG00000100362	PVALB	22:37196728–37215523	209.1	296.0	35.3	0.119	−3.1	7.0E-09	1.6E-05
ENSG00000113658	SMAD5	5:135468534–135524435	1050.2	384.3	2382.0	6.198	2.6	9.4E-09	1.9E-05
ENSG00000135426	TESPA1	12:55341802–55378530	321.0	59.3	844.6	14.25	3.8	1.7E-08	3.2E-05
ENSG00000077984	CST7	20:24929866–24940564	140.5	208.9	3.5	0.017	−5.9	2.6E-08	4.4E-05
ENSG00000118946	PCDH17	13:58205944–58303445	74.2	0.88	220.8	251.4	8.0	6.0E-07	9.2E-04
ENSG00000248527	MTATP6P1	1:569076–569756	7122.7	3712.0	13944.1	3.756	1.9	1.2E-06	1.7E-03
ENSG00000012817	KDM5D	Y:21865751–21906825	149.8	224.4	0.71	0.003	−8.3	1.5E-06	2.0E-03
ENSG00000114374	USP9Y	Y:14813160–14972764	142.5	213.4	0.71	0.003	−8.2	2.4E-06	2.9E-03
ENSG00000185736	ADARB2	10:1228073–1779670	79.2	118.8	0.00	0.000	-Inf	1.53E-05	1.7E-02
ENSG00000229308	AC010084.1	Y:3904538–3968361	340.5	492.35	36.7	0.075	−3.75	1.47E-05	1.7E-02
ENSG00000240356	RPL23AP7	2:114368079–114384667	6531.0	8750.85	2091.0	0.239	−2.07	1.62E-05	1.7E-02

*Fold change;

¶ P-value;

§ Adjusted P-value.

### Differential expression at the gene level in polyA + mRNA vs total RNA

**Figure 9:**
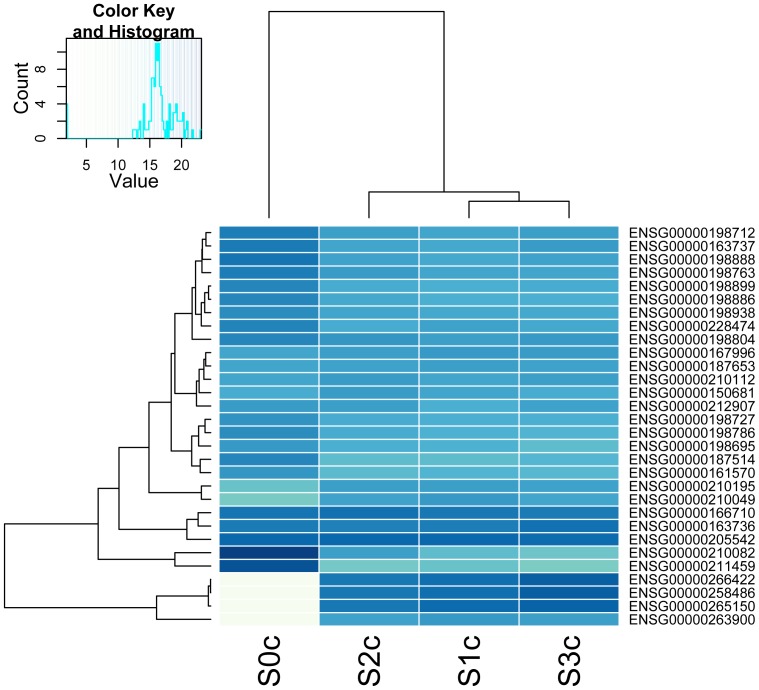
Heatmap showing normalized levels of expression for the 30 most highly expressed gene transcripts across mRNA and rRNA-depleted total RNA samples from the 4 different patients. Nearly all differences of intensity for a given gene are likely to represent preparation artefacts, i.e. due to the poly(dT) enrichment and rRNA-depletion, respectively. Sample names have a ‘C’ added to indicate that the intensities represent length- and method-adjusted counts (TopHat/bedtools/DESeq and “in-house” software).

**Figure 10:**
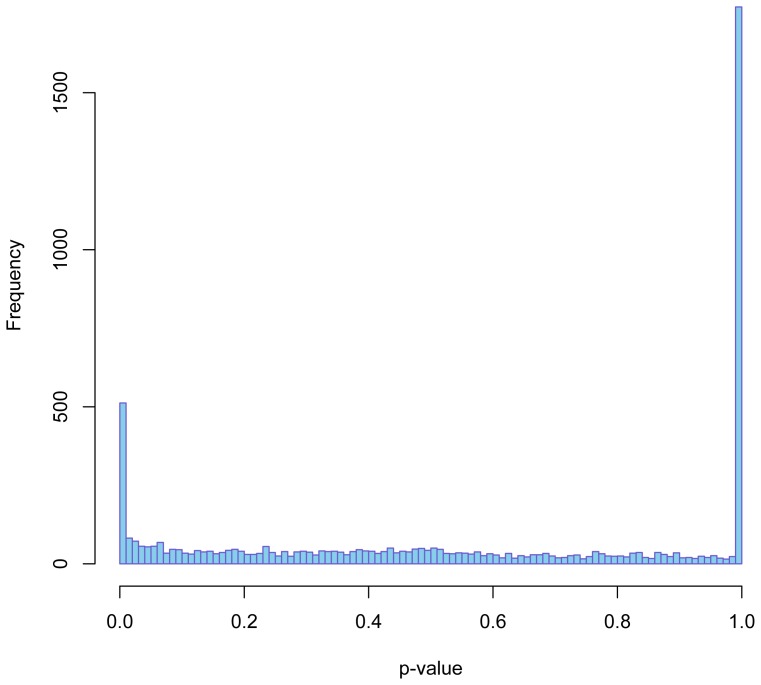
Histogram of p-values from the call to negative binomial test with DESeq comparing the length- and method-adjusted counts of polyA + mRNA sample S0 with the rRNA-depleted total RNA samples S1, S2 and S3. Most of the circa 500 remaining significant differences after length- and method-adjusted normalization presumably represent preparation artefacts, i.e. due to the poly(dT) enrichment and rRNA-depletion, respectively. However, protein coding transcripts lacking a polyA-tail should also appear as differentially expressed. Note that omission of the length- and method-adjusted normalization yields a couple of thousand “differentially expressed” genes (TopHat/bedtools/DESeq and “in-house” software).

Gene expression levels in total RNA samples are conventionally measured as RPKM (Reads Per Kilobase of transcript per Million mapped reads) or FPKM values assuming a rectangular distribution of reads covering the transcript coordinates, i.e. these measures are proportional to the number of reads divided by transcript lengths. The distribution of reads covering the transcript coordinates using oligo(dT) isolated mRNA is very different as it fits an exponential decay function from the 3′-end polyA site towards the 5′-end. (Fig. 3 and Fig. 4). This makes RPKM and FPKM estimates less appropriate for comparison of gene expression levels in polyA + mRNA. Consequently, both transcript lengths and library preparation method ought to be taken into account. Otherwise, false differences will emerge. Adjusted bedcoverage data for the most abundant transcript of each gene is presented in Table S3 in File S1 where columns S0, S1, S2, and S3 represent raw counts and columns S0_adj, and S1_adj to S3_adj represent Normalized Read Counts (NRC) and normalized FPKM figures, respectively (see Table 3a for definition of NRC used in this context). Table 3a demonstrates the fallaciousness of FPKM-values if used on poly(dT) selected transcripts. Figure 9 shows a heatmap of such normalized levels of expression for the 30 most highly expressed genes across the samples from the 4 different patients. Altogether circa 500 differentially expressed genes were identified at 10% FDR comparing mRNA vs. totRNA using DESeq (Fig. 10). A full table of mRNA vs. totRNA comparisons is provided in Table S4 in File S1. As expected, most of this “DE”, which primarily should represent preparation method and mapping artefacts, was observed for non-coding transcripts, which were not present in the polyA+ mRNA preparation, and mitochondrial rRNA transcripts which were more abundant in the polyA+ mRNA sample (Table 6). However, coding transcripts that lack a polyA-tail should also appear as differentially expressed.

**Table 6:** Significant DEφ among the most abundant transcripts in polyA+ mRNA versus rRNA-depleted total RNA.

Ensembl id.	gene	locus	baseMean	baseMeanA	baseMeanB	FC*	log2 FC*	pval¶	padj^§^
ENSG00000210082	MT-RNR2	MT:1671–3229∶1	16931163	66886248	279467	0.004	−7.9	4.0E-23	2.8E-20
ENSG00000266422	RN7SL593P	14:50053298–50053594∶1	6392989	0	8523986	Inf	Inf	5.7E-09	3.1E-07
ENSG00000258486	RN7SL1	14:50053297–50053596∶1	6329059	0	8438746	Inf	Inf	5.8E-09	3.1E-07
ENSG00000211459	MT-RNR1	MT:648–1601∶1	6194782	24607232	57298	0.002	−8.75	3.8E-27	4.0E-24
ENSG00000265150	RN7SL2	14:50329271–50329567∶−1	6165927	0	8221236	Inf	Inf	3.2E-12	4.1E-10
ENSG00000198888	MT-ND1	MT:3307–4262∶1	1690338	5336127	475075	0.089	−3.5	6.8E-06	1.9E-04
ENSG00000163737	PF4	4:74846794–74847841∶−1	1375290	3880989	540057	0.139	−2.9	1.7E-04	3.3E-03
ENSG00000198763	MT-ND2	MT:4470–5511∶1	1269252	3375664	567114	0.168	−2.6	5.4E-04	8.8E-03
ENSG00000198712	MT-CO2	MT:7586–8269∶1	1191008	3025844	579396	0.191	−2.4	1.3E-03	1.8E-02
ENSG00000228474	OST4	2:27293340–27294641∶−1	896875	2240352	449050	0.2	−2.3	1.7E-03	2.3E-02
ENSG00000198899	MT-ATP6	MT:8527–9207∶1	770469	2046623	345084	0.169	−2.6	5.8E-04	9.4E-03
ENSG00000198886	MT-ND4	MT:10760–12137∶1	768789	2051607	341183	0.166	−2.6	5.2E-04	8.6E-03
ENSG00000198938	MT-CO3	MT:9207–9990∶1	728578	1653616	420232	0.254	−2.0	6.4E-03	6.8E-02
ENSG00000187514	PTMA	2:232571605–232578251∶1	634893	2086609	150988	0.072	−3.8	1.5E-06	5.0E-05
ENSG00000198786	MT-ND5	MT:12337–14148∶1	605680	1589548	277724	0.175	−2.5	7.2E-04	1.1E-02
ENSG00000263900	AC006483.1	7:5567734–5567817∶−1	506347	0	675129	Inf	Inf	5.6E-27	5.0E-24
ENSG00000210195	MT-TT	MT:15888–15953∶1	466133	82374	594053	7.21	2.9	1.1E-03	1.6E-02
ENSG00000210049	MT-TF	MT:577–647∶1	464726	42641	605420	14.20	3.8	2.6E-05	6.2E-04
ENSG00000161570	CCL5	17:34198495–34207797∶-1	392953	1050080	173911	0.166	−2.6	5.5E-04	9.0E-03
ENSG00000198695	MT-ND6	MT:14149–14673∶−1	390765	897940	221707	0.247	−2.0	5.5E-03	6.0E-02
ENSG00000209082	MT-TL1	MT:3230–3304∶1	337949	774474	192441	0.248	−2.0	5.9E-03	6.4E-02
ENSG00000140264	SERF2	15:44069285–44094787∶1	252646	889675	40303	0.045	−4.5	2.8E-08	1.3E-06
ENSG00000156265	MAP3K7CL	21:30449792–30548210∶1	240370	0	320494	Inf	Inf	1.4E-16	3.8E-14
ENSG00000210196	MT-TP	MT:15956–16023∶−1	238130	41112	303802	7.39	2.9	9.9E-04	1.5E-02
ENSG00000087086	FTL	19:49468558–49470135∶1	167208	518399	50145	0.097	−3.4	1.7E-05	4.2E-04
ENSG00000210077	MT-TV	MT:1602–1670∶1	136962	329425	72807	0.221	−2.2	3.3E-03	3.9E-02
ENSG00000142669	SH3BGRL3	1:26605667–26608007∶1	136382	503776	13917	0.028	−5.2	2.3E-10	1.9E-08
ENSG00000169756	LIMS1	2:109150857–109303702∶1	124932	283811	71972	0.254	−2.0	7.1E-03	7.4E-02
ENSG00000101608	MYL12A	18:3247479–3256234∶1	122263	3261	161930	49.66	5.6	2.5E-06	7.9E-05
ENSG00000248527	MTATP6P1	1:569076–569756∶1	122224	401658	29079	0.072	−3.8	2.0E-06	6.2E-05

φ Differential Expression;

*Fold change;

¶ P-value; §Adjusted P-value.

### The platelet transcriptome

**Figure 11:**
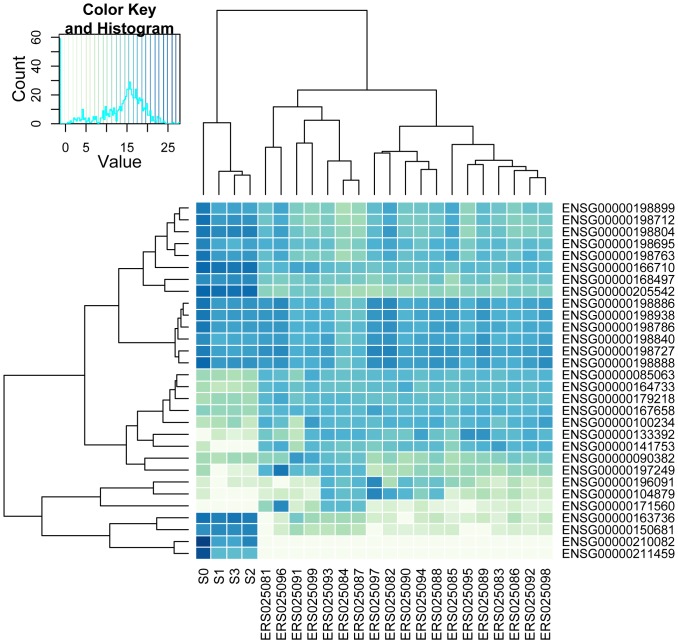
The platelet transcriptome data compared with RNASeq data from Illumina's Human BodyMap 2.0 project. The integrated platelet data from samples S0, S1, S2, and S3 represent counts obtained with TopHat, Ensembl annotations, and the HTSeq-counts program. The Illumina codes are as follows. ERS025098 adipose, ERS025092 adrenal, ERS025085 brain, ERS025088 breast, ERS025089 colon, ERS025082 heart, ERS025081 kidney, ERS025096 liver, ERS025099 lung, ERS025086 lymphnode, ERS025084 mixture, ERS025087 mixture, ERS025093 mixture, ERS025083 ovary, ERS025095 prostate, ERS025097 skeletal_muscle, ERS025094 testes, ERS025090 thyroid, ERS025091 white_blood_cell.

**Figure 12:**
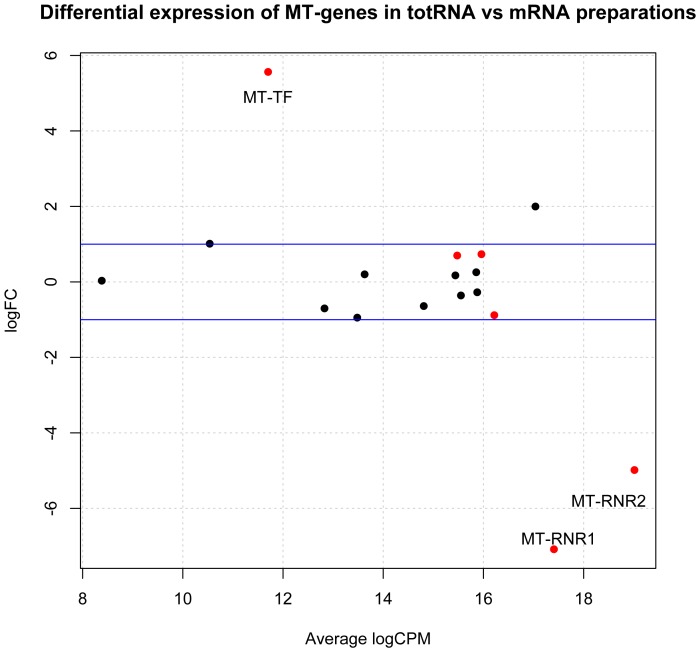
Differential expression of mitochondrial (MT)-genes in total RNA vs mRNA preparations. The figure shows that apart from MT-RNR1, MT-RNR2 and MT-TF, mitochondrially encoded gene expression levels were rather similar in rRNA-depleted total RNA and polyA + mRNA preparations (TopHat/HTSeq/edgeR software). “FC” denotes fold change whereas “CPM” represents counts per million.

The platelet transcriptome data was then compared with RNASeq data from Illumina's Human BodyMap 2.0 project. The Illumina data, generated on HiSeq 2000 instruments, consists of 16 human tissue types, including adrenal, adipose, brain, breast, colon, heart, kidney, liver, lung, lymph, ovary, prostate, skeletal muscle, testes, thyroid, and white blood cells. The heatmap in figure 11 summarizes expression for this data integrated with the platelet raw data counts obtained with the HTSeq-counts program. The dendrogram at the top clearly shows that the platelet expression profile is unique because the samples S0,S1,S2 and S3 forms a cluster of its own from root level. As expected, the polyA+ mRNA sample S0 profile shows some DE when compared with the rRNA-depleted total RNA samples S1, S2, and S3. Thus, the present data suggests that platelets may have a unique transcriptome profile characterized by a relative over-expression of many mitochondrially encoded genes. Apart from MT-RNR1, MT-RNR2 and MT-TF, mitochondrially encoded gene expression levels were rather similar in totRNA and mRNA preparations (Fig. 12 and Table 7).

**Table 7:** Read count table for mitochondrially encoded genes for samples S0, S1, S2 and S3.

Ensembl id.	gene	locus	length	S0	S1	S2	S3
ENSG00000210049	MT-TF	MT:577–647∶1	71	4716	72194	45984	42484
ENSG00000211459	MT-RNR1	MT:648–1601∶1	954	4626427	98775	54719	51139
ENSG00000210077	MT-TV	MT:1602–1670∶1	69	36340	10399	6856	1215
ENSG00000210082	MT-RNR2	MT:1671–3229∶1	1559	13296885	250572	1137529	153605
ENSG00000209082	MT-TL1	MT:3230–3304∶1	75	86075	20917	13706	19721
ENSG00000198888	MT-ND1	MT:3307–4262∶1	956	1003620	480131	557853	659073
ENSG00000210100	MT-TI	MT:4263–4331∶1	69	19538	21026	11319	7338
ENSG00000210107	MT-TQ	MT:4329–4400∶−1	72	26650	31551	37167	27844
ENSG00000210112	MT-TM	MT:4402–4469∶1	68	53547	40780	53844	59771
ENSG00000198763	MT-ND2	MT:4470–5511∶1	1042	643951	625223	928642	622441
ENSG00000210117	MT-TW	MT:5512–5579∶1	68	7355	19999	8161	3780
ENSG00000210127	MT-TA	MT:5587–5655∶−1	69	6825	13478	5840	3956
ENSG00000210135	MT-TN	MT:5657–5729∶−1	73	8457	11923	4449	4815
ENSG00000210140	MT-TC	MT:5761–5826∶−1	66	12786	7746	3790	3592
ENSG00000210144	MT-TY	MT:5826–5891∶−1	66	8401	8967	4896	4065
ENSG00000198804	MT-CO1	MT:5904–7445∶1	1542	414158	1257943	1669453	1633848
ENSG00000210151	MT-TS1	MT:7446–7514∶−1	69	32507	12569	11010	9894
ENSG00000210154	MT-TD	MT:7518–7585∶1	68	7787	7045	6504	4022
ENSG00000198712	MT-CO2	MT:7586–8269∶1	684	529645	397862	485662	598293
ENSG00000210156	MT-TK	MT:8295–8364∶1	70	16629	14388	13232	15576
ENSG00000228253	MT-ATP8	MT:8366–8572∶1	207	61948	66353	72109	65706
ENSG00000198899	MT-ATP6	MT:8527–9207∶1	681	357851	277472	294047	304636
ENSG00000198938	MT-CO3	MT:9207–9990∶1	784	298920	387019	407105	435081
ENSG00000210164	MT-TG	MT:9991–10058∶1	68	6289	11269	13275	11525
ENSG00000198840	MT-ND3	MT:10059–10404∶1	346	86378	92634	121847	146128
ENSG00000210174	MT-TR	MT:10405–10469∶1	65	8430	11341	17249	19014
ENSG00000212907	MT-ND4L	MT:10470–10766∶1	297	111257	118428	203231	207225
ENSG00000198886	MT-ND4	MT:10760–12137∶1	1378	404373	502675	664748	575461
ENSG00000210176	MT-TH	MT:12138–12206∶1	69	10272	14951	7898	7056
ENSG00000210184	MT-TS2	MT:12207–12265∶1	59	8641	6673	7713	13231
ENSG00000210191	MT-TL2	MT:12266–12336∶1	71	8013	8112	9209	12015
ENSG00000198786	MT-ND5	MT:12337–14148∶1	1812	318124	585691	707224	571473
ENSG00000198695	MT-ND6	MT:14149–14673∶−1	525	146565	167234	175374	85557
ENSG00000210194	MT-TE	MT:14674–14742∶−1	69	7417	14089	15081	13333
ENSG00000198727	MT-CYB	MT:14747–15887∶1	1141	220495	447899	453336	452116
ENSG00000210195	MT-TT	MT:15888–15953∶1	66	9053	52084	45617	48793
ENSG00000210196	MT-TP	MT:15956–16023	68	4530	31223	21717	24467
Sum				22910855	6198635	8297396	6919289
Sum without rRNA				4987543	5849288	7105148	6714545

As shown in Figure 11 transcripts from some nuclear genes, particularly TMSB4X, were also more abundant in human platelets as compared to the other cells and tissues. TMSB4X plays a role in regulation of actin polymerization, and is involved in cell proliferation, migration, and differentiation [18]. Furthermore, several genes involved in signal transduction, including chemokines were also abundantly expressed, particularly *PPBP*. The protein encoded by this gene is a platelet-derived growth factor that belongs to the CXC chemokine family, and is a potent chemoattractant for neutrophils [19]. *B2M* (beta-2-microglogulin gene) encodes a serum protein found in association with the major histocompatibility complex (MHC) class I heavy chain on the surface of nearly all nucleated cells [20]. The PF4 chemokine is released from the alpha granules of activated platelets in the form of a homotetramer, which has high affinity for heparin and is involved in platelet aggregation [21]. ACTB is a major constituent of the contractile apparatus and one of the two nonmuscle cytoskeletal actins [22].

The full table of platelet RNASeq data integrated in Illumina's Human BodyMap 2.0 project is available in Table S5 in File S1.

### Functional classification of platelet coding transcripts

**Figure 13:**
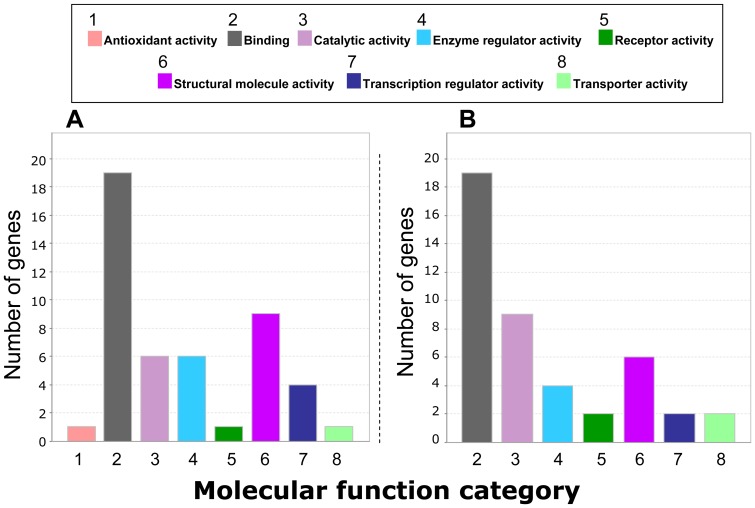
Classification of the proteins coded by the most abundant (top 50) coding transcripts of human platelets. Bars represent molecular function categories generated by the PANTHER gene ontology classification web-based tool. A) Sequencing was performed on polyA+ enriched RNA, whereas in B) rRNA-depleted total RNA was analyzed.

We used the web-based PANTHER software (http://www.pantherdb.org/about.jsp) [23] to classify proteins coded by the top 50 platelet genes using either polyA+ or rRNA-depleted total RNA transcripts mapped against the reference genome. The corresponding genes were grouped into clusters representing gene ontology (GO) categories of molecular functions (Fig. 13). A major finding with this analysis was that the molecular function groups of the top 50 platelet genes for polyA+ enriched RNA (Fig. 13A) correlated remarkably well with those of rRNA-depleted total RNA (Fig. 13B) despite the two distinct approaches and different donors. Among the molecular function GO groups shown in Figure 13, the category b*inding* (GO:0005488) seems to dominate in each top 50 list. As shown in Table 8 most of the genes in this category belong to the *protein binding* subgroup, a class that is expected to play a prominent role in platelet functions. Another noticeable category is the *structural molecule activity* group. This category entails structural constituents of the cytoskeleton, and critical functions concerning cell motility and organization.

**Table 8:** The function of the proteins coded by top 50 platelet genes, as provided by PANTHER gene ontology classification web-based tool.

Nr.	Molecular function category (GO term)	Sub category (GO term)	Number of genes
1	Antioxidant activity (GO:0016209)	n.a.*	–
2	Binding (GO:0005488)	Calcium ion binding (GO:0005509)	2
		Nucleic acid binding (GO:0003676)	6
		Protein binding (GO:0005515)	14
3	Catalytic activity (GO:0003824)	Hydrolase activity (GO:0016787)	2
		Ligase activity (GO:0016874)	2
		Oxidoreductase activity (GO:0016491)	1
		Transferase activity (GO:0016740)	1
4	Enzyme regulator activity (GO:0030234)	Enzyme activator activity (GO:0008047)	1
		Enzyme inhibitor activity (GO:0004857)	2
		Kinase regulator activity (GO:0019207)	1
		Small GTPase regulator activity (GO:0005083)	4
5	Receptor activity (GO:0004872)	n.a.*	–
6	Structural molecule activity (GO:0005198)	Structural constituent of cytoskeleton (GO:0005200)	9
7	Transcription regulator activity (GO:0030528)	Transcription cofactor activity (GO:0003712)	1
		Transcription factor activity (GO:0003700)	4
8	Transporter activity (GO:0005215)	n.a.*	–

*Not available because of too few genes.

## Discussion

In the present study we have compared results of RNA-Seq mapping of polyA+ transcripts in purified blood platelets with those obtained with rRNA-depleted total RNA from healthy blood donors against the set of chromosomes of the Human Feb. 2009 (GRCh37/hg19) assembly (http://www.ncbi.nlm.nih.gov/projects/genome/assembly/grc/). Based on four individuals, the present data show an apparently unique transcriptome profile as compared with other tissues of the Illumina bodymap 2.0 project.

In a typical RNA-Seq experiment, reads are sampled from RNA extracts and either mapped back to a reference genome or used for *de novo* assembly. Alignment and assembly of short or inaccurate reads poses a problem, which we have avoided by using 100 bp high quality Illumina reads. How closely the cDNA sequencing reflects the original RNA population is supposedly mainly determined in the library preparation step. As expected, our mapping of polyA+ reads showed a substantial bias for the 3′-end of gene transcripts due to the selection of mRNA using oligo-dT during the RNA extraction procedure and the following cDNA preparation step [24]. This 3′-UTR bias follows an exponential decay function. After length correction of coverage figures using that function for mRNA and FPKM-values for total RNA, we obtained a reasonably good agreement between quantitative estimates from mapping of polyA+ mRNA and rRNA-depleted total RNA reads to the human genome GRCh37/hg19. It is a notoriously difficult problem to assign reads to a particular isoform if there are many transcript variants with overlaps between them. Very high coverage figures are needed for satisfactory results. This is one of the reasons why RNA-Seq with low coverage has many of the same limitations as other RNA expression analysis pipelines.

Obviously, mapping of reads against the human genome and also mapping against the human exome both rely on the accuracy of gene and transcript annotations. In order to fully characterize the platelet transcriptome without reference to previous results, including the possibility to detect and fully characterize novel transcripts, we also performed a *de novo* assembly of transcripts using Trinity RNA-Seq software (http://trinityrnaseq.sourceforge.net/). This software will extract full-length transcripts for alternatively spliced isoforms based on the generation and analysis of de Bruijn graphs. RSEM software with the bowtie aligner (http://bowtie-bio.sourceforge.net/) was used for mapping the RNA-Seq reads back to the reported transcripts for abundance estimation. Identification of the transcripts was achieved by Blat and BLAST searches using the UCSC Browser and the NCBI Genome databases, respectively. These data fully supported our results obtained by mapping the reads to the human genome and exome, respectively, using gtf.guided assembly. However, even if transcript abundance figures agreed only the most abundant transcripts could be reliably reconstructed by the *de novo* assembly approach; presumably due to insufficient amounts of reads that were available.

When we started this study there was no published RNA-Seq data on platelet gene expression although microarray based as well as SAGE and real-time PCR methods have been used in the past. However, two studies using RNA-Seq by NGS were published during the progress of this study. One of these studies was reported by Rowley et al. who used polyA+ enriched RNA to characterize the transcriptomes of human and mouse platelets [17]. In contrast, Bray et al. utilized rRNA-depleted total RNA and found that their data correlated with those of previously reported microarray transcriptome data at least for the well-expressed mRNAs [16]. Both studies used relatively short sequence reads (≤50 bp for Rowley et al. and ≤40 bp for Bray et al.). The present study employed different strategies for library preparation in addition to the longer (100 bp) read length used for mapping. It is thus expected that there might exist some discrepancies between the current and the previously reported platelet transcript data. A notable difference is the “missed” NGRN transcripts (i.e. an at least tenfold lower amount) in our study when compared to the data of Rowley et al., which possibly could be due to differences of the sample preparation method. However, it should also be kept in mind that when adopting available NGS software for the RNA-seq analyses even small changes in parameter settings can produce a remarkably different result. We used settings of the Bowtie program allowing only 2 mismatches when aligning 100 bp reads to the reference sequence. Context sequencing errors (CSE) that are supposedly specific for the sequencing platform could obviously affect the read counts under such circumstances but a >10-fold reduction seems unlikely because reads from the reverse strand in the mRNA sample S0 should not have been affected to the same extent. One could also speculate whether RNA editing might influence the mapping of our platelet RNA transcripts. Adenosine to inosine (A>I) RNA editing occurs widely across the human transcriptome in certain tissues, especially in the brain [25]. Although there is no data available regarding RNA editing in platelets, we cannot exclude that possibility. However, RNA editing of protein-coding regions appears to be relatively rare events, and may thus have had limited impact on the mapping of cDNA from platelet transcripts.

The relative frequency of reads mapping uniquely to genes involved in platelet function and our molecular function protein classification by PANTHER software is consistent with but does not prove the notion that at least some mRNAs in platelets are not merely remnants from the megakaryocytes without function, but rather reflect an important role of mRNA in the physiological function of platelets. In this regard, it is not surprising that many of the proteins coded by top 50 platelet genes represent key platelet functions such as structural constituent of cytoskeleton, protein binding, calcium binding and signal transduction. On the other hand transcripts of some genes encoding prominent platelet receptors were missing or present with few sequence reads, suggesting that no further synthesis of such proteins is needed after platelet formation in the bone marrow. We also searched for transcript signal for tissue factor (TF) since this protein's eventual presence and function in platelets has been debated for years. However, we could not detect any transcripts encoding TF. Interestingly, Schwertz et al. reported that resting platelets contain TF pre-mRNA that, upon activation, is spliced into mature mRNA, indicating that only activated platelets express mature TF mRNA transcripts [26].

Simultaneously, we have confirmed the dominant frequency of mitochondrially expressed genes comprising the platelet mRNA pool. Specifically in our polyA+ mRNA study, 22,416,906 out of 35,322,009 uniquely mapped reads represent MT-transcripts, apparently related to persistent MT-transcription in the absence of nuclear-derived transcription. This is not unexpected as platelets are metabolically adapted to rapidly expend large amounts of energy required for aggregation, granule release, and clot retraction.

## Conclusions

This study demonstrates that human platelets carry a unique signature of well-defined and highly abundant coding transcripts that are expressed at similar levels among individuals. However, the *in vivo* functional significance of nuclearly encoded platelet mRNAs remains to be shown. Future studies need to focus on establishing the biological and biochemical functions of the identified genes in the physiological and pathological regulation of platelets. The desired end point would be to define a platelet mRNA profile that is directly associated with athero-thrombotic disease, which could eventually lead to the identification of novel targets for anti-thrombotic agents.

## Methods

### Ethical statement

The Regional Ethical Review Board in Linköping (EPN; http://www.epn.se/start/startpage.aspx; Linköping, Sweden) granted an ethical permission for this study (permission number M74-09). Informed written consent was obtained from all participants involved in this study.

### Platelet preparation

Non-irradiated apheresis platelets were collected from healthy blood donors. Platelets were collected by COBE Spectra system (Gambro BCT) as previously described [27] and were used on the same collection day. Residual leukocytes were depleted with anti-CD45 conjugated Dynabeads, according to the manufacturer's recommendations (Pan Leukocyte; Invitrogen, Carlsbad, CA). The platelet suspension, with a volume of 30 mL and a platelet count of 1.4×10^9^ cells/mL, was centrifuged at 800 g for 8 min and the supernatant was discarded. The platelet pellet was processed for leukocyte depletion, as recommended by the supplier of the Pan Leukocyte reagent (Invitrogen, Carlsbad, CA). Since leukocytes possess magnitudes of order more mRNA than platelets, the purification of platelets is a pivotal step. The leukocyte removal was performed at room temperature. Approximately 70–75% of the original platelets were recovered after the leukocyte depletion. To investigate the level of leukocyte contamination, we determined the level of CD45 (*PTPRC*) transcript in multiple platelet preparations (n = 6) by qPCR using TaqMan Gene Expression Assay for this gene according to the recommendations of the supplier (Assay ID: Hs00894713_m1; Applied Bioystems, Carlsbad, CA, USA).

### RNA extraction and cDNA synthesis

The different strategies used for RNA-extraction, library preparation, sequencing and mapping are graphically depicted in Figure 1. For isolation of total RNA, we employed the miRVana RNA Extraction Kit as recommended by the supplier (Life Technologies). Isolation of polyA+ mRNA and synthesis of cDNA were performed by the method described by Rox et al. [22], with the exception that we used Smarter PCR cDNA Synthesis kit for the cDNA synthesis (Clontech, Mountain View, CA, USA). Briefly, the leukocyte-depleted platelet suspension was centrifuged at 1000 g for 10 min and the supernatant was discarded. PolyA+ mRNA was isolated from the platelet cell pellet by using Dynabeads Oligo(dT)_25_ according to the instruction of the manufacturer (Invitrogen, Carlsbad, CA, USA). To synthesize the first-strand cDNA, 3.5 µL of polyA+ mRNA was combined with 1 µL of 3′-Smart CDS primer II A (12 µM). After mixing the tube, the sample was incubated at 72 C for 3 min and then at 42 C for 2 min. This was followed by the addition of a master mix containing 2 µL of 5 First-Strand Buffer, 0.25 µL DTT (100 mM), 1 µL dNTP mix (10 mM), 1 µL SMARTer IIA oligonucleotide (12 µM), 0.25 µL RNase inhibitor, and 1 µL of SMARTScribe Reverse Transcriptase (100 U). The reverse transcription was run by incubating the tube at 42 C for 1 h before the reaction was terminated at 70 C for 10 min. The sample was then diluted with 90 µL of TE-buffer (10 nM Tris, 1 nM EDTA, pH 8.0). To run Long-Distance PCR, 10 µL of the diluted and reverse-transcribed platelet cDNA was mixed with 10 µL 10 Advantage 2 PCR buffer, 2 µL 50 dNTP mix (10 mM), 2 µL 5′PCR primer IIA (12 µM), 2 µL 50 Advantage 2 polymerase mix, and 74 µL of deionized water to a final volume of 100 µL. The sample was then incubated in a thermal cycler running a PCR program containing 95 C for 1 min, and then 20 cycles of 95 C for 15 s, 65 C for 30 s, and 68 C for 3 min. The synthesized platelet cDNA was purified with QIAquick PCR purification kit according to the manufacturer's instructions (Qiagen, Hilden, Germany), and the amount of cDNA was estimated on a Nanodrop spectrophotometer (ND1000; Saveen & Werner, Limhamn, Sweden).

### Illumina HiSeq2000 sequencing

The cDNA obtained from the platelet polyA+ mRNA sample was shotgun sequenced (1×100 bp single read module) with the Illumina HiSeq 2000® instrument (Illumina, San Diego, CA, USA) by using a customer sequencing service (Eurofins MWG Operon, Ebersberg, Germany) which also included nebulization and end repair of cDNA, ligation of adaptors, gel purification, PCR amplification and library purification. The number of raw sequencing reads was 65,111,491. Filtering to remove bad quality bases and reads resulted in 58,155,680 reads (89.3%). These sequences were then mapped against the set of chromosomes of the Human Feb. 2009 (GRCh37/hg19) assembly. Initially, the mapping was conducted using the software TopHat 1.2.0 (http://tophat.cbcb.umd.edu). The post-processing of the mapping results was conducted using SamTools 0.1.12 a (http://samtools.sourceforge.net) and custom made Ruby 1.8.7 software. Bowtie http://bowtie-bio.sourceforge.net/ and bwa http://bio-bwa.sourceforge.net/ were used for aligning to de novo assembled transcripts and RefSeq mRNA respectively.

RNA samples from three platelet donors were prepared for total RNA sequencing. For these samples, ribosomal RNA was depleted with Ribo-Zero (Epicentre, Madison, WI, USA) and strand specific barcoded RNA-sequencing libraries were prepared using ScriptSeq v2 (Epicentre) according to manufacturers instructions. The barcoded libraries were run on a single lane paired end 100 bp on a HiSeq2000® (Illumina, San Diego, CA, USA), which resulted in 153 million pass filter read pairs. QC data can be found in Figure S1 in File S1. The TopHat2 software was used with the bowtie aligner.

### Submission of the sequencing data to public repository

The complete sequencing data is publicly available at The European Nucleotide Archive (http://www.ebi.ac.uk/ena/) under the accession numbers E-MTAB-715 (polyA+ transcripts) and E-MTAB-1846 (total RNA transcripts). Both accession numbers are cross-referenced to one another.

### Assembly of reads and bioinformatics

*de novo* assembly of transcripts was performed using the Trinity RNA-Seq software (http://trinityrnaseq.sourceforge.net).

## Supporting Information

File S1 **Contents**: Table S1: HTSeq raw counts per gene in samples S0, S1, S2, and S3. Table S2: Mapping of S0 (poly(dT) selected transcripts) against RefSeq mRNA. Table S3: Length and method (NRC/FPKM) adjusted counts per gene as represented by the most abundant transcript in samples S0, S1, S2, and S3. Table S4: Differential expression of genes in polyA+ mRNA (A) versus rRNA-depleted total RNA (B). Table S5: The S0, S1, S2 and S3 RNA-Seq data integrated with Illumina's Human BodyMap 2.0 project raw data. Figure S1: QC report.
(PDF)
