# Supplementary material for: Next Generation Sequencing Analysis of Human Platelet PolyA+ mRNAs and rRNA-Depleted Total RNA
Source: PLoS One. 2013 Dec 11;8(12):e81809. doi: 10.1371/journal.pone.0081809 (PMC3859545; doi:10.1371/journal.pone.0081809)
Supplement: File S1 — Contents: Table S1: HTSeq raw counts per gene in samples S0, S1, S2, and S3. Table S2: Mapping of S0 (poly(dT) selected transcripts) against RefSeq mRNA. Table S3: Length and method (NRC/FPKM) adjusted counts per gene as represented by the most abundant transcript in samples S0, S1, S2, and S3. Table S4: Differential expression of genes in polyA+ mRNA (A) versus rRNA-depleted total RNA (B). Table S5: The S0, S1, S2 and S3 RNA-Seq data integrated with Illumina's Human BodyMap 2.0 project raw data. Figure S1: QC report. (PDF) [file pone.0081809.s001.pdf]

SUPPLEMENTARY DATA FILE

Next Generation Sequencing Analysis of Human Platelet polyA+ mRNAs and rRNA-depleted total RNA

Antheia Kissopoulou, Jon Jonasson, Tomas L. Lindahl, Abdimajid Osman

SUPPLEMENTARY TABLES

-Table S1. HTSeq raw counts per gene in samples S0, S1, S2, and S3

-Table S2. Mapping of S0 (poly(dT) selected transcripts) against RefSeq mRNA

-Table S3. Length and method (NRC/FPKM) adjusted counts per gene as represented by the most abundant transcript in samples S0, S1, S2, and S3

-Table S4. Differential expression of genes in polyA+ mRNA (A) versus rRNA-depleted total RNA (B)

-Table S5. The S0, S1, S2 and S3 RNA-Seq data integrated with Illumina's Human BodyMap 2.0 project raw data.

SUPPLEMENTARY FIGURE

-Figure S1. QC report

Table S1. HTSeq raw counts per gene in samples S0, S1, S2, and S3

id	S0	S1	S2	S3
ENSG00000000003	1	40	27	40
ENSG00000000005	0	0	6	0
ENSG00000000419	33	121	279	266
ENSG00000000457	1	170	88	115
ENSG00000000460	51	851	759	930
ENSG00000000938	75	16	16	6
ENSG00000000971	0	0	10	7
ENSG00000001036	63	175	271	280
ENSG00000001084	8	26	13	23
ENSG00000001167	5	135	129	81
ENSG00000001460	2	60	28	50
ENSG00000001461	136	548	443	1148
ENSG00000001497	18	19	17	9
ENSG00000001561	6	187	170	610
ENSG00000001617	0	0	0	0
ENSG00000001626	0	0	0	0
ENSG00000001629	49	4393	4448	6117
ENSG00000001630	0	0	0	0
ENSG00000001631	37	2650	2834	3464
ENSG00000002016	0	33	16	21
ENSG00000002079	0	0	0	0
ENSG00000002330	66	216	107	203
ENSG00000002549	81	363	202	159
ENSG00000002586	9518	10780	5165	11607
ENSG00000002587	1	0	0	0
ENSG00000002726	0	0	0	0
ENSG00000002745	0	0	0	0
ENSG00000002746	0	0	0	2
ENSG00000002822	9	273	114	151
ENSG00000002834	138	1315	930	1041
ENSG00000002919	18	54	63	83
ENSG00000002933	1	0	0	0
ENSG00000003056	82	931	1351	1004
ENSG00000003096	7	106	87	203
ENSG00000003137	0	0	0	0
ENSG00000003147	9	2959	2329	3002
ENSG00000003249	3	0	0	0
ENSG00000003393	5	402	238	450
ENSG00000003400	13	5	32	23
ENSG00000003402	677	2221	3466	2918
ENSG00000003436	493	9712	12768	14105
ENSG00000003509	0	20	19	36
ENSG00000003756	31	1006	789	956
ENSG00000003987	0	0	0	1

ENSG00000003989	0	0	2	0
ENSG00000004059	307	341	274	527
ENSG00000004139	0	0	0	0
ENSG00000004142	28	68	29	46
ENSG00000004399	1	0	0	9
ENSG00000004455	49	180	84	154
ENSG00000004468	3	15	28	53
ENSG00000004478	8	0	3	3
ENSG00000004487	21	2034	2617	4410
ENSG00000004534	25	1024	827	1898
ENSG00000004660	6	8	4	6
ENSG00000004700	46	1196	1635	2410
ENSG00000004766	6	880	999	1358
ENSG00000004776	0	19	1	6
ENSG00000004777	0	0	0	0
ENSG00000004779	47	1	20	42
ENSG00000004799	9	19	0	3
ENSG00000004809	1	0	13	7
ENSG00000004838	0	0	0	0
ENSG00000004846	0	18	10	19
ENSG00000004848	0	0	0	0
ENSG00000004864	6	410	279	353
ENSG00000004866	444	2569	2005	2781
ENSG00000004897	144	4694	6353	6345
ENSG00000004939	3	58	10	43
ENSG00000004948	0	0	0	10
ENSG00000004961	5	0	16	23
ENSG00000004975	7	0	3	3
ENSG00000005001	0	0	0	0
ENSG00000005007	60	371	102	247
ENSG00000005020	3482	16945	24076	28205
ENSG00000005022	218	1381	471	1376
ENSG00000005059	24	106	49	19
ENSG00000005073	0	0	0	0
ENSG00000005075	55	35	44	71
ENSG00000005100	24	645	336	694
ENSG00000005102	2	4	3	3
ENSG00000005108	3	0	0	0
ENSG00000005156	3	19	5	7
ENSG00000005175	204	1020	1166	821
ENSG00000005187	12	51	364	61
ENSG00000005189	33	716	432	344
ENSG00000005194	46	25	43	70
ENSG00000005206	0	23	9	3
ENSG00000005238	303	349	324	227
ENSG00000005243	1	17	43	16

ENSG00000005249	16503	107332	139849	152835
ENSG00000005302	99	75	97	180
ENSG00000005339	113	644	369	630
ENSG00000005379	2	0	0	0
ENSG00000005381	2	0	0	0
ENSG00000005421	0	0	0	0
ENSG00000005436	8	137	155	262
ENSG00000005448	8	0	2	0
ENSG00000005469	5	272	172	294
ENSG00000005471	0	0	0	0
ENSG00000005483	577	8857	8758	9467
ENSG00000005486	70	25	49	35
ENSG00000005513	1	0	0	0
ENSG00000005700	37	1844	1426	2121
ENSG00000005801	1	58	11	31
ENSG00000005810	448	5527	6206	6532
ENSG00000005812	82	1824	2171	1339
ENSG00000005844	56	27	42	58
ENSG00000005882	1	177	89	112
ENSG00000005884	0	0	0	0
ENSG00000005889	22	320	364	206
ENSG00000005893	118	1802	2641	3092
ENSG00000005955	200	899	813	1048
ENSG00000005961	21633	25822	24224	32915
ENSG00000005981	0	0	0	0
ENSG00000006007	745	2375	1902	2918
ENSG00000006015	22	81	18	16
ENSG00000006016	0	0	2	2
ENSG00000006025	0	0	5	4
ENSG00000006042	0	0	0	3
ENSG00000006047	0	0	0	0
ENSG00000006059	0	0	0	0
ENSG00000006062	1	0	0	0
ENSG00000006071	0	0	0	0
ENSG00000006074	1	0	5	10
ENSG00000006075	53	0	0	8
ENSG00000006114	37	620	484	687
ENSG00000006116	0	0	0	0
ENSG00000006118	0	0	0	0
ENSG00000006125	310	6843	6438	5661
ENSG00000006128	0	0	0	0
ENSG00000006194	10	0	5	3
ENSG00000006210	2	16	0	0
ENSG00000006282	5	0	0	3
ENSG00000006283	0	0	0	0
ENSG00000006327	0	0	0	0

ENSG00000006377	0	0	0	0
ENSG00000006432	1	0	0	3
ENSG00000006451	31	40	61	61
ENSG00000006453	0	0	1	0
ENSG00000006459	273	2195	2120	793
ENSG00000006468	0	0	0	5
ENSG00000006530	4	67	50	107
ENSG00000006534	18	0	1	0
ENSG00000006555	0	0	1	0
ENSG00000006576	184	4069	3875	2955
ENSG00000006606	0	0	0	0
ENSG00000006607	0	531	406	839
ENSG00000006611	0	0	0	0
ENSG00000006625	30	41	26	35
ENSG00000006634	18	381	211	572
ENSG00000006638	960	634	349	444
ENSG00000006652	681	19752	19285	10062
ENSG00000006659	0	0	0	0
ENSG00000006695	2	35	27	37
ENSG00000006704	0	0	0	0
ENSG00000006712	10	18	14	40
ENSG00000006715	414	2498	4728	4152
ENSG00000006740	2	0	4	0
ENSG00000006744	4	0	6	45
ENSG00000006747	0	21	0	6
ENSG00000006756	6	8	7	8
ENSG00000006757	4	0	0	8
ENSG00000006788	0	0	3	3
ENSG00000006831	460	2720	2959	2665
ENSG00000006837	8	313	285	469
ENSG00000007001	2	0	2	11
ENSG00000007038	0	0	0	0
ENSG00000007047	21	160	155	340
ENSG00000007062	2	19	13	31
ENSG00000007080	4	0	1	0
ENSG00000007129	3	35	2	15
ENSG00000007168	210	2445	2333	2197
ENSG00000007171	0	0	1	0
ENSG00000007174	2	0	0	0
ENSG00000007202	32	137	159	214
ENSG00000007216	0	0	0	0
ENSG00000007237	32	37	5	0
ENSG00000007255	40	0	7	4
ENSG00000007264	6	0	1	4
ENSG00000007306	0	0	0	0
ENSG00000007312	5	0	0	0

ENSG00000007314	1	27	0	10
ENSG00000007341	108	2490	2712	4141
ENSG00000007350	2	0	2	11
ENSG00000007372	1	0	0	0
ENSG00000007376	9	8	0	1
ENSG00000007384	0	0	0	0
ENSG00000007392	9	54	109	175
ENSG00000007402	2	0	0	0
ENSG00000007516	0	0	0	0
ENSG00000007520	2	47	10	35
ENSG00000007541	1	28	7	9
ENSG00000007545	4	71	21	42
ENSG00000007866	1	0	0	0
ENSG00000007908	9	0	0	3
ENSG00000007923	1	37	49	70
ENSG00000007933	0	0	0	1
ENSG00000007944	50	261	103	11
ENSG00000007952	1	0	0	0
ENSG00000007968	5	0	0	3
ENSG00000008018	151	232	352	353
ENSG00000008056	0	11	0	0
ENSG00000008083	1133	6880	4858	5153
ENSG00000008086	26	265	153	112
ENSG00000008118	0	0	0	0
ENSG00000008128	3	0	1	2
ENSG00000008130	25	24	10	2
ENSG00000008196	0	0	0	0
ENSG00000008197	0	0	0	0
ENSG00000008226	0	0	1	0
ENSG00000008256	2	0	14	29
ENSG00000008277	0	692	562	1308
ENSG00000008282	57	589	487	538
ENSG00000008283	4	0	1	0
ENSG00000008294	32	2648	2217	3670
ENSG00000008300	0	0	0	0
ENSG00000008311	0	62	63	72
ENSG00000008323	0	0	0	0
ENSG00000008324	46	12	4	4
ENSG00000008382	8	23	4	21
ENSG00000008394	13	16	32	25
ENSG00000008405	8	137	111	127
ENSG00000008438	3	12	0	0
ENSG00000008441	12	0	0	0
ENSG00000008513	301	637	341	282
ENSG00000008516	0	0	0	0
ENSG00000008517	304	0	3	1

ENSG00000008710	0	0	0	1
ENSG00000008735	0	0	0	0
ENSG00000008838	9	55	54	82
ENSG00000008853	9	0	3	4
ENSG00000008869	6	1555	1162	1414
ENSG00000008952	674	1964	1708	1941
ENSG00000008988	2113	2332	1667	2935
ENSG00000009307	1438	12174	16987	19420
ENSG00000009335	28	825	825	843
ENSG00000009413	36	773	1114	1555
ENSG00000009694	0	0	0	3
ENSG00000009709	0	0	0	0
ENSG00000009724	1	0	0	0
ENSG00000009765	0	0	0	0
ENSG00000009780	9	51	26	51
ENSG00000009790	79	0	1	0
ENSG00000009830	1	14	1	6
ENSG00000009844	44	1420	1239	1592
ENSG00000009950	0	0	0	0
ENSG00000009954	67	836	785	1175
ENSG00000010017	72	14663	14389	22107
ENSG00000010030	1	0	0	0
ENSG00000010072	5	93	39	38
ENSG00000010165	9	0	3	0
ENSG00000010219	16	2	9	31
ENSG00000010244	200	894	1082	1176
ENSG00000010256	80	20	9	18
ENSG00000010270	193	420	1062	1599
ENSG00000010278	1935	18690	6599	15381
ENSG00000010282	0	0	0	0
ENSG00000010292	51	238	107	70
ENSG00000010295	0	33	12	12
ENSG00000010310	0	0	0	0
ENSG00000010318	64	377	878	1360
ENSG00000010319	0	0	0	1
ENSG00000010322	19	18	18	8
ENSG00000010327	3	24	17	20
ENSG00000010361	4	23	10	13
ENSG00000010379	1	0	0	0
ENSG00000010404	1482	3685	5433	5355
ENSG00000010438	0	0	0	0
ENSG00000010539	6	14	6	2
ENSG00000010610	11	0	1	0
ENSG00000010626	5	54	12	61
ENSG00000010671	1117	5752	3849	3177
ENSG00000010704	9	29	55	14

ENSG00000010803	9	726	614	994
ENSG00000010810	852	2324	1694	2634
ENSG00000010818	22	243	355	103
ENSG00000010932	0	0	0	0
ENSG00000011007	43	91	109	218
ENSG00000011009	35	12	14	20
ENSG00000011021	23	2	23	23
ENSG00000011028	0	0	0	1
ENSG00000011052	0	0	0	0
ENSG00000011083	0	0	0	1
ENSG00000011105	212	2274	1850	1788
ENSG00000011114	57	580	705	900
ENSG00000011132	9	26	9	4
ENSG00000011143	2	0	19	39
ENSG00000011198	58	715	627	579
ENSG00000011201	1	39	14	31
ENSG00000011243	1	69	39	39
ENSG00000011258	525	13344	9970	9903
ENSG00000011260	39	686	600	861
ENSG00000011275	117	5189	2932	3804
ENSG00000011295	20	187	412	182
ENSG00000011304	289	885	522	574
ENSG00000011332	0	0	0	0
ENSG00000011347	0	0	0	1
ENSG00000011376	1	0	10	19
ENSG00000011405	22	1732	2051	2089
ENSG00000011422	1	0	2	0
ENSG00000011426	0	462	536	645
ENSG00000011451	0	0	0	1
ENSG00000011454	196	8663	7148	11858
ENSG00000011465	0	0	0	0
ENSG00000011478	0	0	2	3
ENSG00000011485	2	62	20	40
ENSG00000011523	162	243	573	160
ENSG00000011566	3	452	720	913
ENSG00000011590	0	0	0	0
ENSG00000011600	164	84	41	13
ENSG00000011638	392	709	362	251
ENSG00000011677	0	0	0	2
ENSG00000012048	5	1490	1379	1867
ENSG00000012061	67	162	69	64
ENSG00000012124	31	162	99	89
ENSG00000012171	0	0	0	0
ENSG00000012174	1	49	106	123
ENSG00000012211	0	15	3	2
ENSG00000012223	6	71	0	7

ENSG00000012232	4	0	4	13
ENSG00000012504	0	0	4	1
ENSG00000012660	27	643	978	1132
ENSG00000012779	21	0	3	3
ENSG00000012817	5	386	1	375
ENSG00000012822	245	2056	1662	1324
ENSG00000012963	11	136	125	170
ENSG00000012983	770	5816	8131	5113
ENSG00000013016	1546	6911	4927	6972
ENSG00000013275	90	129	88	189
ENSG00000013288	4	25	7	18
ENSG00000013293	0	0	0	0
ENSG00000013297	1	0	1	2
ENSG00000013306	42	12	8	25
ENSG00000013364	8	10	16	15
ENSG00000013374	94	224	277	294
ENSG00000013375	4	87	147	84
ENSG00000013392	0	0	4	0
ENSG00000013441	103	1687	1779	2455
ENSG00000013503	5	76	67	74
ENSG00000013523	2	0	2	1
ENSG00000013561	102	781	1089	957
ENSG00000013563	22	166	156	202
ENSG00000013573	5	33	7	6
ENSG00000013583	73	279	120	179
ENSG00000013588	0	0	0	2
ENSG00000013619	1	17	11	3
ENSG00000013725	86	0	0	23
ENSG00000013810	399	613	183	294
ENSG00000014123	28	112	102	110
ENSG00000014138	1	34	39	42
ENSG00000014164	2	20	29	29
ENSG00000014216	444	3112	1570	2083
ENSG00000014257	1	0	0	0
ENSG00000014641	36	49	29	91
ENSG00000014824	34	738	869	814
ENSG00000014914	7	13	0	4
ENSG00000014919	11	13	24	42
ENSG00000015133	281	895	368	738
ENSG00000015153	270	451	697	403
ENSG00000015171	142	1706	1901	2525
ENSG00000015285	58	134	44	55
ENSG00000015413	0	0	0	0
ENSG00000015475	213	607	716	1531
ENSG00000015479	844	8417	9315	8868
ENSG00000015520	0	0	0	0

ENSG00000015532	85	169	68	260
ENSG00000015568	0	1	53	74
ENSG00000015592	4	0	1	0
ENSG00000015676	23	16	29	46
ENSG00000016082	0	0	0	0
ENSG00000016391	0	0	0	1
ENSG00000016402	21	0	0	4
ENSG00000016490	3	0	0	0
ENSG00000016602	0	0	0	0
ENSG00000016864	17	54	62	80
ENSG00000017260	98	13354	12788	10131
ENSG00000017373	0	0	0	0
ENSG00000017427	0	0	0	0
ENSG00000017483	5	29	13	17
ENSG00000017621	26	29	54	77
ENSG00000017797	447	3248	3832	4097
ENSG00000018189	11	74	92	76
ENSG00000018236	0	0	0	0
ENSG00000018280	90	46	16	37
ENSG00000018408	14	16	19	18
ENSG00000018510	22	882	975	1100
ENSG00000018607	0	0	0	0
ENSG00000018610	15	44	38	47
ENSG00000018625	0	0	0	0
ENSG00000018699	14	9888	7678	10312
ENSG00000018869	11	215	71	75
ENSG00000019102	879	352	401	205
ENSG00000019144	0	0	0	1
ENSG00000019169	0	0	0	0
ENSG00000019186	0	0	0	0
ENSG00000019485	2	0	13	7
ENSG00000019505	0	0	0	0
ENSG00000019549	1	0	0	0
ENSG00000019582	1042	463	571	417
ENSG00000019991	1	0	11	16
ENSG00000019995	52	320	327	446
ENSG00000020129	0	0	0	1
ENSG00000020181	0	0	0	0
ENSG00000020219	0	0	0	0
ENSG00000020256	1	15	6	6
ENSG00000020426	41	516	746	1270
ENSG00000020577	57	163	87	338
ENSG00000020633	105	0	1	0
ENSG00000020922	17	1420	1565	2195
ENSG00000021300	2	0	5	9
ENSG00000021355	3383	19861	18106	27064

ENSG00000021461	1	0	0	0
ENSG00000021488	0	0	0	3
ENSG00000021574	41	3604	3152	4284
ENSG00000021645	1	0	1	0
ENSG00000021762	3	0	0	6
ENSG00000021776	18	1233	1170	1544
ENSG00000021826	0	0	2	0
ENSG00000021852	0	0	0	0
ENSG00000022267	1443	5290	5186	6705
ENSG00000022277	228	608	510	710
ENSG00000022355	0	9	0	0
ENSG00000022556	2	0	3	0
ENSG00000022567	27	3156	1327	3455
ENSG00000022840	424	2706	1641	2234
ENSG00000022976	1	0	13	28
ENSG00000023041	4	23	21	12
ENSG00000023171	0	0	1	10
ENSG00000023191	42	189	110	247
ENSG00000023228	42	1925	2223	3170
ENSG00000023287	139	522	618	459
ENSG00000023318	44	1062	1157	1272
ENSG00000023330	12	131	116	175
ENSG00000023445	51	48	35	6
ENSG00000023516	31	327	548	311
ENSG00000023572	3	28	19	43
ENSG00000023608	8	33	68	54
ENSG00000023697	367	3540	4113	2556
ENSG00000023734	350	2632	1862	2056
ENSG00000023839	0	0	3	0
ENSG00000023892	20	0	1	2
ENSG00000023902	1720	4261	3070	2999
ENSG00000023909	705	3837	4478	3833
ENSG00000024048	47	3074	2875	3477
ENSG00000024422	67	109	86	133
ENSG00000024526	6	175	234	436
ENSG00000024862	106	224	191	108
ENSG00000025039	25	52	74	21
ENSG00000025156	2	12	15	30
ENSG00000025293	176	2768	1829	3014
ENSG00000025423	0	0	4	0
ENSG00000025434	10	45	0	4
ENSG00000025708	1	1	3	16
ENSG00000025770	2	0	0	0
ENSG00000025772	14	0	8	15
ENSG00000025796	79	2272	3303	4908
ENSG00000025800	105	251	178	269

ENSG00000026025	567	640	432	366
ENSG00000026036	0	0	0	0
ENSG00000026103	1	8	7	6
ENSG00000026297	83	69	103	76
ENSG00000026508	111	880	823	319
ENSG00000026559	0	0	0	0
ENSG00000026652	7	21	54	69
ENSG00000026751	12	0	0	0
ENSG00000026950	91	732	490	740
ENSG00000027001	7	1077	236	293
ENSG00000027075	26	27	24	8
ENSG00000027644	0	0	0	0
ENSG00000027697	97	1552	2114	1009
ENSG00000027847	4	0	5	2
ENSG00000027869	15	0	10	11
ENSG00000028116	54	4700	4266	6296
ENSG00000028137	24	0	1	0
ENSG00000028203	87	1961	1833	2016
ENSG00000028277	117	10	5	7
ENSG00000028310	12	54	32	59
ENSG00000028528	93	952	1157	1826
ENSG00000028839	219	3360	4486	3597
ENSG00000029153	4	1280	1228	1683
ENSG00000029363	119	442	637	603
ENSG00000029364	20	505	502	634
ENSG00000029534	514	2352	1793	2499
ENSG00000029559	0	0	0	0
ENSG00000029639	8	16	7	16
ENSG00000029725	45	1970	2412	2732
ENSG00000029993	12	0	22	46
ENSG00000030066	5	214	169	257
ENSG00000030110	21	14	8	22
ENSG00000030304	0	0	0	0
ENSG00000030419	6	0	0	0
ENSG00000030582	48	55	23	54
ENSG00000031003	138	27902	49998	47663
ENSG00000031081	3	1	10	19
ENSG00000031544	0	0	0	0
ENSG00000031691	2	65	34	25
ENSG00000031698	88	101	70	107
ENSG00000031823	12	114	37	71
ENSG00000032219	79	817	737	1281
ENSG00000032389	11	9	5	27
ENSG00000032444	8	120	22	62
ENSG00000032742	64	1015	1038	1459
ENSG00000033011	0	40	15	19

ENSG00000033030	1	293	147	259
ENSG00000033050	13	0	12	6
ENSG00000033100	4	21	21	13
ENSG00000033122	0	0	0	0
ENSG00000033170	395	9779	5810	7683
ENSG00000033178	9	613	660	608
ENSG00000033327	29	226	190	89
ENSG00000033627	67	4212	2400	3605
ENSG00000033800	92	2865	2460	3041
ENSG00000033867	19	462	558	891
ENSG00000034053	10	472	293	732
ENSG00000034063	1	0	2	6
ENSG00000034152	1380	2529	1288	1610
ENSG00000034239	0	0	0	0
ENSG00000034510	2796	2253	2125	2972
ENSG00000034533	0	27	12	11
ENSG00000034677	39	1937	1877	2699
ENSG00000034693	8	825	573	848
ENSG00000034713	3888	11075	8704	16584
ENSG00000034971	0	0	0	0
ENSG00000035115	26	113	46	24
ENSG00000035141	28	0	13	1
ENSG00000035403	12315	88141	109233	114154
ENSG00000035499	5	256	209	282
ENSG00000035664	117	149	118	146
ENSG00000035681	13	228	162	265
ENSG00000035687	26	344	310	363
ENSG00000035720	6	0	3	3
ENSG00000035862	13	47	60	43
ENSG00000035928	121	977	1264	1941
ENSG00000036054	190	1895	2848	2437
ENSG00000036257	55	2951	2067	2964
ENSG00000036448	60	0	2	25
ENSG00000036473	0	0	0	0
ENSG00000036530	1	0	0	0
ENSG00000036549	37	1365	1607	1993
ENSG00000036565	1	0	1	0
ENSG00000036672	0	0	0	0
ENSG00000036828	0	0	0	0
ENSG00000037042	9	0	6	84
ENSG00000037241	28	12	39	61
ENSG00000037280	1	0	2	1
ENSG00000037474	3	765	561	988
ENSG00000037637	29	181	175	241
ENSG00000037749	3	42	86	69
ENSG00000037757	4	17	1	1

ENSG00000037897	1	0	1	1
ENSG00000037965	0	0	0	0
ENSG00000038002	3	20	24	36
ENSG00000038210	134	446	434	473
ENSG00000038219	145	239	245	335
ENSG00000038274	151	1622	1806	1144
ENSG00000038295	0	0	0	0
ENSG00000038358	0	0	3	7
ENSG00000038382	18	485	376	416
ENSG00000038427	14	12	7	4
ENSG00000038532	16	1175	633	1011
ENSG00000038945	2	0	0	0
ENSG00000039068	0	0	6	0
ENSG00000039123	27	1224	1458	1580
ENSG00000039139	0	0	6	0
ENSG00000039319	9	86	71	67
ENSG00000039523	3	8	5	2
ENSG00000039537	0	0	0	0
ENSG00000039560	1	0	1	0
ENSG00000039600	0	0	0	0
ENSG00000039650	12	5	10	23
ENSG00000039987	7	27	33	27
ENSG00000040199	3	273	136	196
ENSG00000040275	16	377	331	394
ENSG00000040341	460	6578	7142	5815
ENSG00000040487	16	70	20	16
ENSG00000040531	339	417	315	206
ENSG00000040608	0	0	0	0
ENSG00000040633	35	73	14	18
ENSG00000040731	0	10	8	20
ENSG00000040933	15	12	9	33
ENSG00000041353	3594	68130	103832	81154
ENSG00000041357	157	694	497	673
ENSG00000041515	0	0	0	10
ENSG00000041802	61	98	94	144
ENSG00000041880	10	1	5	1
ENSG00000041982	0	0	0	0
ENSG00000041988	5	4	0	2
ENSG00000042062	73	287	224	182
ENSG00000042088	6	83	84	85
ENSG00000042286	1	0	0	1
ENSG00000042304	0	0	0	0
ENSG00000042317	2	264	364	56
ENSG00000042429	6	437	369	498
ENSG00000042445	3	0	3	0
ENSG00000042493	54	15	2	0

ENSG00000042753	505	947	482	1137
ENSG00000042781	0	0	0	0
ENSG00000042813	0	0	1	2
ENSG00000042832	20	9	6	36
ENSG00000042980	0	22	2	0
ENSG00000043039	0	0	0	0
ENSG00000043093	235	6589	9109	6533
ENSG00000043143	13	12	59	109
ENSG00000043355	0	0	0	0
ENSG00000043462	236	1068	1198	1149
ENSG00000043514	0	45	94	141
ENSG00000043591	0	0	0	0
ENSG00000044012	0	0	0	0
ENSG00000044090	1	10	7	3
ENSG00000044115	132	1242	1653	2407
ENSG00000044446	11	119	74	122
ENSG00000044459	300	10391	9888	11509
ENSG00000044524	0	0	0	0
ENSG00000044574	42	186	87	127
ENSG00000046604	0	0	5	5
ENSG00000046647	4	19	22	34
ENSG00000046651	450	2669	2785	2756
ENSG00000046653	3	0	8	2
ENSG00000046774	0	0	0	0
ENSG00000046889	1	22	4	6
ENSG00000047056	9	1454	1347	2139
ENSG00000047188	38	1900	2312	2144
ENSG00000047230	2	925	626	954
ENSG00000047249	14	470	351	524
ENSG00000047315	55	566	474	682
ENSG00000047346	31	1384	1551	1659
ENSG00000047365	20	55	93	84
ENSG00000047410	110	748	688	979
ENSG00000047457	0	0	1	0
ENSG00000047578	1	21	39	61
ENSG00000047579	18	0	40	40
ENSG00000047597	485	5459	4921	3424
ENSG00000047617	45	3965	2960	4463
ENSG00000047621	4	405	248	440
ENSG00000047634	17	31	65	164
ENSG00000047644	164	568	1074	1008
ENSG00000047648	1319	5507	5934	6160
ENSG00000047662	4	0	8	8
ENSG00000047849	36	175	72	100
ENSG00000047932	11	43	64	77
ENSG00000047936	0	0	0	0

ENSG00000048028	20	11	16	16
ENSG00000048052	108	4193	3138	5437
ENSG00000048140	3	0	0	6
ENSG00000048162	6	0	2	0
ENSG00000048342	2	0	3	0
ENSG00000048392	6	87	96	85
ENSG00000048405	51	531	389	867
ENSG00000048462	1	0	0	0
ENSG00000048471	43	474	292	484
ENSG00000048540	0	0	0	0
ENSG00000048544	176	52	257	222
ENSG00000048545	0	0	0	0
ENSG00000048649	685	5663	6332	8716
ENSG00000048707	44	1050	513	1200
ENSG00000048740	1865	7086	8251	7118
ENSG00000048828	351	6066	3935	5924
ENSG00000048991	84	3178	4327	4667
ENSG00000049089	1	0	0	0
ENSG00000049130	21	1363	1813	941
ENSG00000049167	4	127	108	166
ENSG00000049192	18	3723	1919	2239
ENSG00000049239	4	0	1	0
ENSG00000049245	101	1424	3295	4324
ENSG00000049246	15	232	352	888
ENSG00000049247	2	0	1	14
ENSG00000049249	0	0	1	0
ENSG00000049283	0	0	0	0
ENSG00000049319	0	0	0	0
ENSG00000049323	3580	30361	29138	38278
ENSG00000049449	2	0	12	10
ENSG00000049540	1	0	0	1
ENSG00000049541	47	205	173	283
ENSG00000049618	112	926	532	862
ENSG00000049656	36	93	145	191
ENSG00000049759	13	185	135	114
ENSG00000049768	0	0	0	0
ENSG00000049769	4	19	17	44
ENSG00000049860	8	80	127	236
ENSG00000049883	0	17	25	81
ENSG00000050030	0	0	0	0
ENSG00000050130	96	3021	3304	2254
ENSG00000050165	0	0	0	0
ENSG00000050327	0	0	0	0
ENSG00000050344	3	0	29	9
ENSG00000050393	2681	14094	10770	12499
ENSG00000050405	35	102	80	132

ENSG00000050426	32	222	161	243
ENSG00000050438	0	0	17	53
ENSG00000050555	0	0	0	0
ENSG00000050628	22	209	336	474
ENSG00000050730	7	0	5	31
ENSG00000050748	22	645	951	1303
ENSG00000050767	0	0	9	0
ENSG00000050820	0	0	0	6
ENSG00000051009	2	0	0	0
ENSG00000051108	8	4	35	39
ENSG00000051128	0	0	0	0
ENSG00000051180	0	12	1	7
ENSG00000051341	0	626	921	1166
ENSG00000051382	437	12834	13284	9539
ENSG00000051523	10	8	11	14
ENSG00000051596	1	0	6	15
ENSG00000051620	17	0	21	26
ENSG00000051825	16	3205	3777	4610
ENSG00000052126	91	1449	1568	2042
ENSG00000052344	0	0	0	0
ENSG00000052723	16	64	80	102
ENSG00000052749	6	0	1	3
ENSG00000052795	6	69	76	102
ENSG00000052802	5	0	12	8
ENSG00000052841	10	377	416	545
ENSG00000052850	0	0	0	0
ENSG00000053108	112	250	99	160
ENSG00000053254	303	415	558	560
ENSG00000053328	0	141	54	33
ENSG00000053371	324	170	309	245
ENSG00000053372	20	0	1	1
ENSG00000053438	0	0	0	0
ENSG00000053501	22	18	2	0
ENSG00000053524	2	3373	1986	3397
ENSG00000053702	0	0	0	0
ENSG00000053747	1	16	4	14
ENSG00000053770	39	906	955	1011
ENSG00000053900	7	612	314	382
ENSG00000053918	0	0	0	0
ENSG00000054116	89	186	123	228
ENSG00000054118	186	227	235	308
ENSG00000054148	48	0	1	0
ENSG00000054179	0	0	0	0
ENSG00000054219	0	0	16	11
ENSG00000054267	282	1121	839	1263
ENSG00000054277	2	24	34	41

ENSG00000054282	260	2252	2925	2871
ENSG00000054356	152	17	29	14
ENSG00000054392	0	0	1	10
ENSG00000054523	89	1139	1212	363
ENSG00000054598	8	8	19	25
ENSG00000054611	62	427	208	456
ENSG00000054654	69	168	51	83
ENSG00000054690	0	0	0	0
ENSG00000054793	928	6471	6718	5093
ENSG00000054796	0	0	0	0
ENSG00000054803	0	0	0	0
ENSG00000054938	0	0	0	0
ENSG00000054965	10	76	56	60
ENSG00000054967	0	0	0	0
ENSG00000054983	5	115	67	95
ENSG00000055044	74	303	577	855
ENSG00000055070	281	477	397	633
ENSG00000055118	0	0	0	0
ENSG00000055130	44	704	873	931
ENSG00000055147	24	1141	808	522
ENSG00000055163	133	0	23	46
ENSG00000055208	700	4433	7831	5497
ENSG00000055211	151	759	669	1002
ENSG00000055332	57	199	338	319
ENSG00000055483	8	16	11	26
ENSG00000055609	999	31124	28059	35771
ENSG00000055732	0	0	0	0
ENSG00000055813	5	3	3	5
ENSG00000055917	176	2752	3236	3062
ENSG00000055950	27	0	3	13
ENSG00000055955	0	0	0	0
ENSG00000055957	0	0	0	0
ENSG00000056050	24	39	57	99
ENSG00000056097	189	1118	1383	1520
ENSG00000056277	1	106	71	89
ENSG00000056291	0	0	0	0
ENSG00000056487	0	0	0	0
ENSG00000056558	4	0	1	0
ENSG00000056586	489	5440	8850	7140
ENSG00000056661	0	0	2	6
ENSG00000056678	0	0	0	0
ENSG00000056736	1	0	0	0
ENSG00000056972	5	0	0	8
ENSG00000056998	0	0	0	0
ENSG00000057019	50	721	550	496
ENSG00000057149	0	0	0	0

ENSG00000057252	4	399	574	763
ENSG00000057294	38	236	169	374
ENSG00000057468	1	11	45	148
ENSG00000057593	0	0	0	0
ENSG00000057608	263	8000	9272	11832
ENSG00000057657	8	0	0	0
ENSG00000057663	18	699	659	1010
ENSG00000057704	7	21	8	0
ENSG00000057757	84	207	164	203
ENSG00000057935	6	175	265	374
ENSG00000058056	2	219	142	332
ENSG00000058063	132	2113	2787	2413
ENSG00000058085	0	0	0	0
ENSG00000058091	161	1584	1830	1987
ENSG00000058262	17	150	209	264
ENSG00000058272	637	4266	4756	4895
ENSG00000058335	0	0	0	4
ENSG00000058404	0	0	0	0
ENSG00000058453	0	0	0	2
ENSG00000058600	13	90	32	66
ENSG00000058668	18	89	42	66
ENSG00000058673	339	4588	5850	3354
ENSG00000058729	1	0	18	7
ENSG00000058799	33	36	44	108
ENSG00000058804	4	1398	1494	1052
ENSG00000058866	113	2895	2741	3728
ENSG00000059122	2	0	8	1
ENSG00000059145	25	98	50	75
ENSG00000059377	395	2180	1943	1735
ENSG00000059378	9	18	63	47
ENSG00000059573	0	10	10	15
ENSG00000059588	6	285	178	223
ENSG00000059691	6	0	1	6
ENSG00000059728	168	3868	3708	1721
ENSG00000059758	67	4105	5581	5976
ENSG00000059769	3	74	28	22
ENSG00000059804	1625	12611	14502	12733
ENSG00000059915	1	0	0	0
ENSG00000060069	5	232	119	131
ENSG00000060138	1115	1867	889	2242
ENSG00000060140	2	9	8	5
ENSG00000060237	670	3345	2347	3095
ENSG00000060303	0	0	0	0
ENSG00000060339	408	4396	3837	6608
ENSG00000060491	2	0	4	0
ENSG00000060558	287	586	652	569

ENSG00000060566	0	0	0	0
ENSG00000060642	2	0	14	3
ENSG00000060656	0	0	0	2
ENSG00000060688	20	378	236	374
ENSG00000060709	0	0	0	0
ENSG00000060718	0	12	0	4
ENSG00000060749	19	400	413	311
ENSG00000060762	144	400	307	757
ENSG00000060971	8	0	3	12
ENSG00000060982	32	302	227	323
ENSG00000061273	25	12	7	3
ENSG00000061337	0	0	0	0
ENSG00000061455	2	0	0	0
ENSG00000061492	0	0	0	0
ENSG00000061656	0	0	0	0
ENSG00000061676	209	11173	11353	11929
ENSG00000061794	25	2626	2456	3507
ENSG00000061918	2011	17498	20530	23309
ENSG00000061936	17	46	48	79
ENSG00000061938	9	80	17	80
ENSG00000061987	2	695	753	1132
ENSG00000062038	0	0	0	0
ENSG00000062096	0	0	0	0
ENSG00000062194	341	11699	10266	12885
ENSG00000062282	0	0	0	3
ENSG00000062370	0	0	4	10
ENSG00000062485	50	110	82	152
ENSG00000062524	0	0	4	0
ENSG00000062582	20	0	0	8
ENSG00000062598	39	372	158	378
ENSG00000062650	102	2980	2044	3173
ENSG00000062716	111	2381	1857	2863
ENSG00000062725	16	574	715	739
ENSG00000062822	0	175	48	117
ENSG00000063015	0	0	0	3
ENSG00000063046	237	1016	962	1162
ENSG00000063127	0	0	2	5
ENSG00000063169	0	0	0	0
ENSG00000063176	0	4	1	19
ENSG00000063177	736	49	18	62
ENSG00000063180	0	0	2	2
ENSG00000063241	2	0	0	1
ENSG00000063244	55	367	205	281
ENSG00000063245	10	119	48	119
ENSG00000063322	30	58	17	45
ENSG00000063438	0	0	0	2

ENSG00000063515	0	0	0	0
ENSG00000063587	12	5	38	57
ENSG00000063601	3	45	86	120
ENSG00000063660	0	0	0	0
ENSG00000063761	1	0	1	14
ENSG00000063854	23	94	45	75
ENSG00000063978	39	409	252	357
ENSG00000064012	16	62	51	77
ENSG00000064042	0	7	4	6
ENSG00000064102	9	318	290	399
ENSG00000064115	92	2028	1422	2323
ENSG00000064195	0	0	0	0
ENSG00000064199	4	78	66	59
ENSG00000064201	155	146	121	311
ENSG00000064205	0	0	0	0
ENSG00000064218	0	0	0	0
ENSG00000064225	171	1496	1362	2922
ENSG00000064270	0	0	0	0
ENSG00000064300	0	0	0	0
ENSG00000064309	0	0	1	2
ENSG00000064313	15	1876	1754	2200
ENSG00000064393	938	4711	4180	5010
ENSG00000064419	28	4455	3007	4097
ENSG00000064489	0	0	0	0
ENSG00000064490	13	15	10	11
ENSG00000064545	0	0	0	0
ENSG00000064547	2	12	1	1
ENSG00000064601	14081	22290	18890	31713
ENSG00000064607	9	195	119	192
ENSG00000064651	7	2122	1615	2281
ENSG00000064652	170	1	188	555
ENSG00000064655	0	0	0	0
ENSG00000064666	251	957	358	1696
ENSG00000064687	34	3	23	20
ENSG00000064692	0	0	0	0
ENSG00000064703	5	81	49	106
ENSG00000064726	106	1512	2103	1904
ENSG00000064763	11	1925	1327	2676
ENSG00000064787	3	0	0	0
ENSG00000064835	0	0	0	0
ENSG00000064886	13	0	0	0
ENSG00000064932	4	29	6	15
ENSG00000064933	18	2110	3069	3740
ENSG00000064961	114	1155	913	1904
ENSG00000064989	0	15	6	13
ENSG00000064995	44	60	49	86

ENSG00000064999	39	367	134	248
ENSG00000065000	53	219	93	259
ENSG00000065029	6	18	40	27
ENSG00000065054	6	36	3	5
ENSG00000065057	2	2	1	3
ENSG00000065060	180	619	462	308
ENSG00000065135	66	1235	1510	1259
ENSG00000065150	35	116	78	117
ENSG00000065154	10	305	114	166
ENSG00000065183	15	41	50	26
ENSG00000065243	37	5535	7354	8153
ENSG00000065268	1	0	0	1
ENSG00000065308	25	37	59	102
ENSG00000065320	0	0	0	0
ENSG00000065325	0	0	0	0
ENSG00000065328	1	433	248	709
ENSG00000065357	24	35	72	76
ENSG00000065361	1	0	0	4
ENSG00000065371	0	0	0	0
ENSG00000065413	11	11	11	15
ENSG00000065427	114	157	209	252
ENSG00000065457	107	140	225	237
ENSG00000065485	25	636	331	741
ENSG00000065491	2	21	15	24
ENSG00000065518	383	574	463	480
ENSG00000065526	78	634	910	737
ENSG00000065534	8304	19947	20590	17108
ENSG00000065548	108	194	190	202
ENSG00000065559	29	3868	4400	6593
ENSG00000065600	25	146	194	158
ENSG00000065609	0	0	0	0
ENSG00000065613	89	4689	3970	5232
ENSG00000065615	99	704	1017	914
ENSG00000065618	0	0	0	0
ENSG00000065621	5	18	17	20
ENSG00000065665	3	19	22	33
ENSG00000065675	174	2620	1916	2612
ENSG00000065717	1	0	0	0
ENSG00000065802	9	19	8	3
ENSG00000065809	269	1417	1131	1094
ENSG00000065833	35	317	1130	983
ENSG00000065882	80	9018	4504	5590
ENSG00000065883	33	887	742	1165
ENSG00000065911	210	2570	1935	2518
ENSG00000065923	1	968	269	602
ENSG00000065970	59	586	129	261

ENSG00000065978	2554	3815	3837	7393
ENSG00000065989	8	19	0	0
ENSG00000066027	280	941	1204	864
ENSG00000066032	0	0	0	0
ENSG00000066044	135	584	646	607
ENSG00000066056	0	0	4	4
ENSG00000066084	6	154	117	163
ENSG00000066117	5	169	114	118
ENSG00000066135	10	44	22	32
ENSG00000066136	41	366	289	574
ENSG00000066185	270	1122	874	1145
ENSG00000066230	0	0	0	0
ENSG00000066248	0	0	2	0
ENSG00000066279	7	598	492	620
ENSG00000066294	1266	5107	6336	9583
ENSG00000066322	49	177	74	109
ENSG00000066336	8	6	8	4
ENSG00000066379	15	0	5	8
ENSG00000066382	21	308	143	205
ENSG00000066405	0	0	0	0
ENSG00000066422	22	1666	1930	2234
ENSG00000066427	33	1035	1104	1464
ENSG00000066455	15	322	479	408
ENSG00000066468	0	0	0	4
ENSG00000066557	7	540	407	418
ENSG00000066583	8	162	300	368
ENSG00000066629	0	0	0	0
ENSG00000066651	3	235	213	259
ENSG00000066654	262	1348	2554	672
ENSG00000066697	616	2909	3794	3787
ENSG00000066735	0	0	0	0
ENSG00000066739	15	342	292	508
ENSG00000066777	61	4973	5120	6888
ENSG00000066813	0	0	0	0
ENSG00000066827	2	99	129	246
ENSG00000066855	10	255	196	185
ENSG00000066923	1	19	2	0
ENSG00000066926	19	310	164	176
ENSG00000066933	13	1295	1305	1774
ENSG00000067048	32	1833	8	2675
ENSG00000067057	203	944	375	1351
ENSG00000067064	86	579	353	384
ENSG00000067066	188	1379	1074	1058
ENSG00000067082	382	616	359	106
ENSG00000067113	0	8	9	25
ENSG00000067141	0	0	3	0

ENSG00000067167	2109	23446	27879	29405
ENSG00000067177	0	0	4	7
ENSG00000067182	6	35	12	22
ENSG00000067191	16	92	41	79
ENSG00000067208	27	2127	1664	2631
ENSG00000067221	0	0	0	2
ENSG00000067225	12462	57310	44300	61037
ENSG00000067248	84	1911	2532	3002
ENSG00000067334	89	350	353	466
ENSG00000067365	480	658	827	1533
ENSG00000067369	31	63	148	128
ENSG00000067445	1	11	1	0
ENSG00000067533	21	26	41	50
ENSG00000067560	4616	18821	18404	22098
ENSG00000067596	24	67	105	220
ENSG00000067601	14	113	70	75
ENSG00000067606	1	0	0	0
ENSG00000067646	14	216	0	440
ENSG00000067704	5	215	182	214
ENSG00000067715	1	0	5	0
ENSG00000067798	1	0	7	6
ENSG00000067829	168	53	49	70
ENSG00000067836	38	134	65	106
ENSG00000067840	3	87	2	1
ENSG00000067842	0	0	0	0
ENSG00000067900	998	7982	9136	9584
ENSG00000067955	17	386	397	405
ENSG00000067992	125	1088	1731	1681
ENSG00000068001	0	0	0	0
ENSG00000068024	1	8	9	33
ENSG00000068028	50	0	31	0
ENSG00000068078	0	0	3	0
ENSG00000068079	40	0	3	3
ENSG00000068097	3	14	8	1
ENSG00000068120	60	58	19	35
ENSG00000068137	0	0	0	0
ENSG00000068305	141	2362	2275	2836
ENSG00000068308	207	765	400	357
ENSG00000068323	125	208	129	148
ENSG00000068354	129	294	180	233
ENSG00000068366	207	4399	6824	7568
ENSG00000068383	112	2720	2879	3644
ENSG00000068394	21	0	2	7
ENSG00000068400	107	193	97	204
ENSG00000068438	9	0	1	1
ENSG00000068489	37	109	71	112

ENSG00000068615	38	127	188	21
ENSG00000068650	67	724	322	452
ENSG00000068654	2	39	39	38
ENSG00000068697	20	72	75	93
ENSG00000068724	6	27	22	38
ENSG00000068745	63	99	106	129
ENSG00000068781	0	37	2	18
ENSG00000068784	20	1521	1004	1614
ENSG00000068793	56	420	433	771
ENSG00000068796	6407	94060	119556	152841
ENSG00000068831	1520	1325	696	1188
ENSG00000068878	197	2591	2459	2222
ENSG00000068885	10	463	482	639
ENSG00000068903	210	343	252	314
ENSG00000068912	25	317	354	552
ENSG00000068971	1	66	2	14
ENSG00000068976	5	0	2	0
ENSG00000068985	1	0	0	0
ENSG00000069011	0	0	0	0
ENSG00000069018	0	0	0	0
ENSG00000069020	873	2644	3426	2989
ENSG00000069122	2	0	9	2
ENSG00000069188	0	0	1	1
ENSG00000069206	0	0	1	0
ENSG00000069248	16	858	625	841
ENSG00000069275	517	2346	2574	4288
ENSG00000069329	127	1223	889	1155
ENSG00000069345	117	930	985	841
ENSG00000069399	2	0	0	0
ENSG00000069424	9	14	5	20
ENSG00000069431	11	183	202	209
ENSG00000069482	4	0	0	0
ENSG00000069493	3	230	95	154
ENSG00000069509	33	2180	4117	3495
ENSG00000069535	26	38	90	354
ENSG00000069667	9	5	22	26
ENSG00000069696	0	0	0	0
ENSG00000069702	5	5	0	0
ENSG00000069712	1	53	62	145
ENSG00000069764	0	0	2	0
ENSG00000069812	0	0	0	0
ENSG00000069849	38	123	125	213
ENSG00000069869	41	1457	1593	1734
ENSG00000069943	4	439	399	519
ENSG00000069956	96	2004	2763	2063
ENSG00000069966	1075	3259	2341	4883

ENSG00000069974	76	28	15	42
ENSG00000069998	4	0	0	0
ENSG00000070010	410	843	789	1023
ENSG00000070018	0	456	496	788
ENSG00000070019	0	19	0	0
ENSG00000070031	0	0	0	0
ENSG00000070047	2	67	15	23
ENSG00000070061	10	21	29	21
ENSG00000070081	236	3002	3146	3920
ENSG00000070087	3	4	2	5
ENSG00000070159	0	0	0	0
ENSG00000070182	76	1712	979	2266
ENSG00000070190	12520	50947	65083	59437
ENSG00000070193	0	0	0	0
ENSG00000070214	612	5214	5341	4949
ENSG00000070269	46	400	281	596
ENSG00000070366	10	125	61	130
ENSG00000070367	81	2570	3304	3351
ENSG00000070371	0	0	0	0
ENSG00000070388	0	0	0	0
ENSG00000070404	1	0	0	1
ENSG00000070413	116	364	161	277
ENSG00000070423	3	0	0	1
ENSG00000070444	9	40	24	15
ENSG00000070476	4	0	11	11
ENSG00000070495	6	22	8	13
ENSG00000070501	16	52	287	298
ENSG00000070526	1	0	0	0
ENSG00000070540	167	2469	2138	3055
ENSG00000070601	0	0	1	0
ENSG00000070610	26	98	94	71
ENSG00000070614	275	1979	856	1346
ENSG00000070669	1	0	4	0
ENSG00000070718	60	226	247	116
ENSG00000070729	0	0	0	0
ENSG00000070731	0	0	0	1
ENSG00000070748	0	0	0	0
ENSG00000070756	9591	12494	10687	16119
ENSG00000070759	7	280	159	291
ENSG00000070761	6	18	5	8
ENSG00000070770	49	242	210	231
ENSG00000070778	0	0	1	0
ENSG00000070785	12	0	14	47
ENSG00000070808	0	9	0	0
ENSG00000070814	11	30	1	12
ENSG00000070831	2901	25921	31403	28463

ENSG00000070882	2	35	108	156
ENSG00000070886	0	0	0	0
ENSG00000070915	0	0	0	0
ENSG00000070950	20	766	548	1205
ENSG00000070961	39	322	285	424
ENSG00000070985	0	0	0	0
ENSG00000071051	1022	10037	9200	8273
ENSG00000071054	229	880	770	552
ENSG00000071073	25	0	4	0
ENSG00000071082	1907	1228	749	1755
ENSG00000071127	5133	16983	13835	16358
ENSG00000071189	19	3408	3764	4794
ENSG00000071203	0	0	0	0
ENSG00000071205	331	15748	22026	26754
ENSG00000071242	10	24	13	9
ENSG00000071243	99	484	452	439
ENSG00000071246	39	181	77	217
ENSG00000071282	4	27	5	5
ENSG00000071462	85	0	22	50
ENSG00000071537	82	1763	1382	1265
ENSG00000071539	12	115	37	40
ENSG00000071553	337	244	217	341
ENSG00000071564	6	0	2	8
ENSG00000071575	5	0	2	8
ENSG00000071626	166	179	104	157
ENSG00000071655	6	3	8	12
ENSG00000071677	0	0	0	0
ENSG00000071794	33	1296	1136	1171
ENSG00000071859	127	219	194	283
ENSG00000071889	72	53	54	149
ENSG00000071894	7	41	6	7
ENSG00000071909	30	963	381	40
ENSG00000071967	5	60	111	30
ENSG00000071991	0	0	8	1
ENSG00000071994	26	23	7	15
ENSG00000072041	0	0	12	3
ENSG00000072042	3037	13672	11587	9834
ENSG00000072062	130	72	70	238
ENSG00000072071	0	0	1	5
ENSG00000072080	0	7	28	12
ENSG00000072110	8047	19214	11511	23601
ENSG00000072121	4	95	63	85
ENSG00000072133	0	0	1	0
ENSG00000072134	1	0	0	0
ENSG00000072135	11160	17281	10495	10196
ENSG00000072163	2	11	1	0

ENSG00000072182	0	0	0	0
ENSG00000072195	0	0	0	0
ENSG00000072201	3	32	2	2
ENSG00000072210	12	0	21	16
ENSG00000072274	15	275	355	558
ENSG00000072310	0	0	0	3
ENSG00000072315	0	0	0	0
ENSG00000072364	43	913	1120	1365
ENSG00000072401	17	19	18	24
ENSG00000072415	23	1940	1576	2619
ENSG00000072422	1544	6609	4841	6409
ENSG00000072444	0	0	2	2
ENSG00000072501	81	1502	1907	3476
ENSG00000072506	30	0	1	8
ENSG00000072518	13	35	42	33
ENSG00000072571	10	351	368	437
ENSG00000072609	12	16	13	66
ENSG00000072657	1	1135	1093	1001
ENSG00000072682	0	0	0	0
ENSG00000072694	4	0	0	0
ENSG00000072736	12	1185	1453	1692
ENSG00000072756	75	3575	3922	4896
ENSG00000072778	125	162	203	298
ENSG00000072786	113	153	88	126
ENSG00000072803	60	1388	1916	2433
ENSG00000072818	29	12	5	12
ENSG00000072832	0	0	0	0
ENSG00000072840	0	0	0	0
ENSG00000072849	84	810	770	876
ENSG00000072858	145	536	389	928
ENSG00000072864	15	454	435	711
ENSG00000072952	178	706	310	917
ENSG00000072954	0	0	1	0
ENSG00000072958	40	59	30	81
ENSG00000073008	2	0	0	0
ENSG00000073009	217	817	834	935
ENSG00000073050	46	81	64	155
ENSG00000073060	0	21	6	2
ENSG00000073067	0	0	0	0
ENSG00000073111	11	66	72	26
ENSG00000073146	2	0	14	21
ENSG00000073150	0	0	0	0
ENSG00000073169	0	0	1	1
ENSG00000073282	7	86	107	127
ENSG00000073331	2	26	38	49
ENSG00000073350	0	0	1	11

ENSG00000073417	4	129	239	307
ENSG00000073464	149	959	1127	903
ENSG00000073536	0	0	0	6
ENSG00000073578	300	1025	742	1065
ENSG00000073584	315	1035	681	833
ENSG00000073598	0	0	0	0
ENSG00000073605	6	95	38	111
ENSG00000073614	212	683	628	671
ENSG00000073670	1	0	0	0
ENSG00000073711	2	51	22	17
ENSG00000073712	5	100	38	83
ENSG00000073734	0	0	0	0
ENSG00000073737	1	0	0	1
ENSG00000073754	0	0	0	0
ENSG00000073756	0	0	3	7
ENSG00000073792	1294	2722	1959	2501
ENSG00000073803	45	100	61	86
ENSG00000073849	354	1568	3235	2804
ENSG00000073861	19	0	0	0
ENSG00000073905	0	0	0	0
ENSG00000073910	2	662	529	924
ENSG00000073921	60	26368	20829	23963
ENSG00000073969	13	73	54	148
ENSG00000074047	0	0	0	0
ENSG00000074054	25	1283	1057	1918
ENSG00000074071	85	161	45	78
ENSG00000074181	2	0	0	0
ENSG00000074201	21	10012	8149	12760
ENSG00000074211	1	0	0	0
ENSG00000074219	0	0	0	0
ENSG00000074266	9	3031	2101	4146
ENSG00000074276	0	0	0	0
ENSG00000074317	0	0	0	0
ENSG00000074319	65	259	405	327
ENSG00000074356	42	419	297	398
ENSG00000074370	2051	2311	1196	2951
ENSG00000074410	0	0	0	0
ENSG00000074416	4483	5034	2998	3831
ENSG00000074527	0	0	5	0
ENSG00000074582	4	0	16	28
ENSG00000074590	3	0	3	2
ENSG00000074603	102	1888	1912	2584
ENSG00000074621	8	27	5	5
ENSG00000074657	3	0	5	2
ENSG00000074660	4	161	125	212
ENSG00000074695	136	882	1255	686

ENSG00000074696	2	0	17	11
ENSG00000074706	238	3627	3852	3004
ENSG00000074755	33	1491	891	1321
ENSG00000074771	0	0	0	0
ENSG00000074800	1873	5506	2135	5497
ENSG00000074803	0	0	0	0
ENSG00000074842	15	0	0	1
ENSG00000074855	2	23	4	4
ENSG00000074935	0	0	4	5
ENSG00000074964	0	0	0	0
ENSG00000074966	20	1508	454	1230
ENSG00000075035	0	0	1	0
ENSG00000075043	0	0	0	0
ENSG00000075073	0	0	0	0
ENSG00000075089	20	213	228	368
ENSG00000075131	3	524	634	851
ENSG00000075142	28	138	113	218
ENSG00000075151	1331	12131	14166	20835
ENSG00000075188	10	46	39	70
ENSG00000075213	0	0	0	0
ENSG00000075218	5	0	0	6
ENSG00000075223	2	28	14	18
ENSG00000075234	19	0	10	3
ENSG00000075239	16	0	42	45
ENSG00000075240	1	0	6	0
ENSG00000075275	0	0	0	0
ENSG00000075290	0	0	0	0
ENSG00000075292	81	4304	4853	5499
ENSG00000075303	6	164	242	264
ENSG00000075336	2	0	3	12
ENSG00000075340	3	0	1	3
ENSG00000075388	0	0	0	0
ENSG00000075391	11	491	188	480
ENSG00000075399	46	90	37	102
ENSG00000075407	31	8	60	138
ENSG00000075413	142	2243	2775	3132
ENSG00000075415	216	568	449	598
ENSG00000075420	10	130	111	275
ENSG00000075426	5	0	0	0
ENSG00000075429	0	0	0	0
ENSG00000075461	0	0	0	0
ENSG00000075539	65	2306	1671	4351
ENSG00000075568	17	885	639	867
ENSG00000075618	1	0	0	1
ENSG00000075624	89881	159711	103695	175492
ENSG00000075643	0	0	2	0

ENSG00000075651	1	16	15	22
ENSG00000075673	0	0	1	0
ENSG00000075702	0	43	7	29
ENSG00000075711	95	4762	3932	6009
ENSG00000075785	526	1440	1540	2137
ENSG00000075790	152	1576	2608	2289
ENSG00000075826	1	40	2	1
ENSG00000075856	35	182	114	175
ENSG00000075884	43	1075	997	1894
ENSG00000075886	0	0	0	0
ENSG00000075891	0	0	0	0
ENSG00000075914	10	0	4	1
ENSG00000075945	1000	9619	7177	10291
ENSG00000075975	9	20	36	27
ENSG00000076003	391	1576	1473	829
ENSG00000076043	140	241	263	302
ENSG00000076053	15	101	103	113
ENSG00000076067	3	19	39	85
ENSG00000076108	141	163	146	127
ENSG00000076201	3	0	3	3
ENSG00000076242	20	204	155	201
ENSG00000076248	3	0	18	9
ENSG00000076258	0	0	6	3
ENSG00000076321	9	115	111	133
ENSG00000076344	0	0	0	0
ENSG00000076351	0	0	0	0
ENSG00000076356	26	94	55	62
ENSG00000076382	0	6	8	21
ENSG00000076513	206	1676	1618	1732
ENSG00000076554	6	54	44	59
ENSG00000076555	3	86	18	32
ENSG00000076604	1	0	0	0
ENSG00000076641	57	248	146	286
ENSG00000076650	10	419	371	951
ENSG00000076662	59	0	0	1
ENSG00000076685	230	2138	2597	1550
ENSG00000076706	3	0	0	0
ENSG00000076716	0	0	1	3
ENSG00000076770	284	10683	10144	11292
ENSG00000076826	0	0	0	0
ENSG00000076864	0	0	0	0
ENSG00000076924	5	17	16	27
ENSG00000076928	1	1	2	0
ENSG00000076944	662	615	319	550
ENSG00000076984	4	0	4	3
ENSG00000077009	0	0	0	0

ENSG00000077044	459	1839	1123	1889
ENSG00000077063	0	0	0	2
ENSG00000077080	0	0	0	0
ENSG00000077092	1	0	4	4
ENSG00000077097	137	1695	2061	2132
ENSG00000077147	32	3163	3274	4110
ENSG00000077150	1	2	7	8
ENSG00000077152	4	10	0	8
ENSG00000077157	4	212	127	157
ENSG00000077232	15	344	263	411
ENSG00000077235	13	147	96	162
ENSG00000077238	5	0	1	3
ENSG00000077254	519	7287	8977	9737
ENSG00000077264	0	0	0	1
ENSG00000077274	0	0	0	0
ENSG00000077279	0	0	0	0
ENSG00000077312	12	61	9	11
ENSG00000077327	0	11	6	0
ENSG00000077348	26	0	1	0
ENSG00000077380	365	1479	1243	1586
ENSG00000077420	104	268	169	185
ENSG00000077454	2	2	13	24
ENSG00000077458	30	370	300	334
ENSG00000077463	1	22	0	0
ENSG00000077498	0	0	0	0
ENSG00000077514	13	11	16	18
ENSG00000077522	0	0	0	0
ENSG00000077549	1829	3807	2555	4239
ENSG00000077585	182	294	920	630
ENSG00000077616	0	0	0	5
ENSG00000077684	11	41	16	34
ENSG00000077713	33	784	669	1046
ENSG00000077721	66	250	357	299
ENSG00000077782	1	0	0	0
ENSG00000077800	0	0	0	0
ENSG00000077809	758	2960	2819	2885
ENSG00000077935	0	18	27	32
ENSG00000077942	0	0	0	2
ENSG00000077943	0	0	0	0
ENSG00000077984	200	300	5	411
ENSG00000078018	12	325	337	353
ENSG00000078043	18	2213	1797	2024
ENSG00000078053	14	0	0	0
ENSG00000078061	171	171	96	132
ENSG00000078070	1	558	460	840
ENSG00000078081	0	17	0	5

ENSG00000078098	0	0	1	0
ENSG00000078114	0	14	5	1
ENSG00000078124	328	2360	1821	2623
ENSG00000078140	940	4580	5865	7202
ENSG00000078142	52	13513	12819	18437
ENSG00000078177	12	133	201	184
ENSG00000078237	5	0	2	0
ENSG00000078246	4	0	1	6
ENSG00000078269	84	180	211	26
ENSG00000078295	0	17	0	2
ENSG00000078304	199	1022	1089	1733
ENSG00000078319	4	257	210	350
ENSG00000078328	0	0	0	0
ENSG00000078369	1227	5521	4656	9884
ENSG00000078399	0	0	0	0
ENSG00000078401	0	0	0	0
ENSG00000078403	33	2427	1756	3230
ENSG00000078487	14	8	54	54
ENSG00000078549	0	0	0	0
ENSG00000078579	0	0	0	0
ENSG00000078589	114	739	1055	531
ENSG00000078596	1020	1898	1742	2358
ENSG00000078618	1348	9202	11639	12489
ENSG00000078668	3236	29096	19229	16710
ENSG00000078674	142	3343	3563	4202
ENSG00000078687	127	304	285	596
ENSG00000078699	3	168	209	387
ENSG00000078725	0	0	0	0
ENSG00000078747	77	4493	3443	5894
ENSG00000078795	0	1	18	9
ENSG00000078804	64	66	65	132
ENSG00000078808	2	319	285	545
ENSG00000078814	2	0	0	0
ENSG00000078898	0	0	0	0
ENSG00000078900	0	0	0	0
ENSG00000078902	462	645	344	486
ENSG00000078967	9	13	30	58
ENSG00000079101	0	0	0	8
ENSG00000079102	4	34	14	40
ENSG00000079112	0	0	3	0
ENSG00000079134	10	300	229	349
ENSG00000079150	3	0	2	8
ENSG00000079156	15	43	115	84
ENSG00000079215	0	0	0	0
ENSG00000079246	280	1160	1008	1410
ENSG00000079257	80	281	632	832

ENSG00000079263	32	0	0	0
ENSG00000079277	78	357	442	529
ENSG00000079308	451	3732	2584	1970
ENSG00000079313	0	4	3	9
ENSG00000079332	107	1987	1916	2674
ENSG00000079335	10	1381	1801	2806
ENSG00000079337	2	0	0	1
ENSG00000079385	0	0	0	0
ENSG00000079387	107	1609	1653	1643
ENSG00000079393	0	0	0	0
ENSG00000079432	57	156	42	71
ENSG00000079435	0	0	0	5
ENSG00000079459	92	137	286	343
ENSG00000079462	5	0	2	0
ENSG00000079482	106	6213	5638	9331
ENSG00000079557	0	0	1	0
ENSG00000079616	38	13	106	80
ENSG00000079689	0	0	0	0
ENSG00000079691	16	1920	1442	2690
ENSG00000079739	67	204	317	410
ENSG00000079785	39	184	137	180
ENSG00000079805	40	335	161	240
ENSG00000079819	17	95	145	219
ENSG00000079841	0	0	0	3
ENSG00000079931	0	0	0	0
ENSG00000079950	700	6644	5405	2649
ENSG00000079974	14	103	10	46
ENSG00000079999	22	43	29	31
ENSG00000080007	0	92	73	105
ENSG00000080031	0	0	0	0
ENSG00000080166	5	1	4	0
ENSG00000080189	23	49	40	47
ENSG00000080200	2	63	60	72
ENSG00000080224	0	0	0	0
ENSG00000080293	0	0	0	0
ENSG00000080298	56	505	689	937
ENSG00000080345	71	1534	1480	1756
ENSG00000080371	112	1865	2232	2079
ENSG00000080493	0	6	23	23
ENSG00000080503	796	4382	3918	5117
ENSG00000080511	0	0	0	0
ENSG00000080546	14	86	121	102
ENSG00000080561	0	0	0	0
ENSG00000080572	0	0	0	0
ENSG00000080573	0	0	0	0
ENSG00000080603	30	313	170	335

ENSG00000080608	32	434	373	347
ENSG00000080618	0	0	0	0
ENSG00000080644	0	0	3	0
ENSG00000080709	0	0	0	0
ENSG00000080802	43	457	449	565
ENSG00000080815	16	1051	781	1323
ENSG00000080819	4	10	36	21
ENSG00000080822	31	333	235	265
ENSG00000080823	1	0	3	0
ENSG00000080824	1615	3367	2636	4696
ENSG00000080839	9	8444	5866	9057
ENSG00000080845	28	71	27	56
ENSG00000080854	4	9	12	9
ENSG00000080910	0	0	0	0
ENSG00000080947	5	0	0	0
ENSG00000080986	3	111	121	177
ENSG00000081014	12	720	1061	1315
ENSG00000081019	17	68	48	80
ENSG00000081026	1	263	63	250
ENSG00000081041	21	19	4	15
ENSG00000081051	0	0	0	0
ENSG00000081052	0	0	0	1
ENSG00000081059	74	0	0	0
ENSG00000081087	84	916	1226	1422
ENSG00000081138	0	0	0	0
ENSG00000081148	0	53	45	85
ENSG00000081154	2202	10544	15038	19991
ENSG00000081177	0	13	18	16
ENSG00000081181	37	1680	1711	937
ENSG00000081189	1347	4998	6966	7385
ENSG00000081237	158	85	484	136
ENSG00000081248	0	0	0	0
ENSG00000081277	0	0	0	0
ENSG00000081307	14	70	82	151
ENSG00000081320	62	226	260	196
ENSG00000081377	1792	16226	14679	21131
ENSG00000081386	4	1	38	49
ENSG00000081479	0	0	1	0
ENSG00000081665	26	76	147	307
ENSG00000081692	0	0	0	2
ENSG00000081721	6	108	55	64
ENSG00000081760	2	48	21	23
ENSG00000081791	10	19	21	20
ENSG00000081800	0	0	0	0
ENSG00000081803	1	6	10	12
ENSG00000081818	0	0	0	0

ENSG00000081842	0	0	1	0
ENSG00000081853	0	0	0	0
ENSG00000081870	134	236	289	670
ENSG00000081913	5	348	410	542
ENSG00000081923	0	10	14	55
ENSG00000081985	3	8	15	10
ENSG00000082014	8	0	1	0
ENSG00000082068	17	79	54	67
ENSG00000082074	8589	19783	25102	30328
ENSG00000082126	0	0	0	1
ENSG00000082146	98	401	607	529
ENSG00000082153	208	1172	1074	986
ENSG00000082175	0	0	0	0
ENSG00000082196	0	38	7	42
ENSG00000082212	23	2434	3256	5230
ENSG00000082213	9	187	196	276
ENSG00000082258	9	247	218	319
ENSG00000082269	1	283	282	300
ENSG00000082293	0	0	0	0
ENSG00000082397	179	3153	1732	2281
ENSG00000082438	9	29	45	37
ENSG00000082458	3	36	5	22
ENSG00000082482	0	0	0	0
ENSG00000082497	0	0	0	0
ENSG00000082512	5	0	9	26
ENSG00000082515	23	99	303	525
ENSG00000082516	6	13	32	58
ENSG00000082556	0	0	0	0
ENSG00000082641	11	128	22	28
ENSG00000082684	0	0	0	0
ENSG00000082701	265	6243	5270	8466
ENSG00000082781	2194	5231	4264	5457
ENSG00000082805	27	2075	2141	3170
ENSG00000082898	120	14613	19727	29477
ENSG00000082929	0	0	0	0
ENSG00000082996	127	8615	7796	8785
ENSG00000083067	2	0	0	2
ENSG00000083093	8	193	160	286
ENSG00000083097	1	399	612	864
ENSG00000083099	51	32	43	22
ENSG00000083123	3	156	79	190
ENSG00000083168	851	3245	2549	2287
ENSG00000083223	120	8755	9026	10556
ENSG00000083290	16	496	259	376
ENSG00000083307	0	0	0	0
ENSG00000083312	324	4402	9192	5025

ENSG00000083444	27	39	78	148
ENSG00000083454	2	0	0	0
ENSG00000083457	265	215	164	571
ENSG00000083520	17	347	504	725
ENSG00000083535	4	1600	1760	2836
ENSG00000083544	30	5663	4836	7869
ENSG00000083622	0	0	0	0
ENSG00000083635	14	130	132	287
ENSG00000083642	121	1751	2345	2469
ENSG00000083720	9	846	708	939
ENSG00000083750	9	87	60	68
ENSG00000083782	0	0	0	0
ENSG00000083799	99	1147	863	1332
ENSG00000083807	2	0	0	0
ENSG00000083812	0	0	0	0
ENSG00000083814	3	0	0	2
ENSG00000083817	0	0	1	0
ENSG00000083828	16	295	248	663
ENSG00000083838	0	0	0	1
ENSG00000083842	0	0	0	0
ENSG00000083844	1	4	10	11
ENSG00000083845	375	73	42	207
ENSG00000083857	0	0	0	0
ENSG00000083896	104	139	190	169
ENSG00000083937	80	401	235	438
ENSG00000084070	116	216	109	173
ENSG00000084072	283	682	483	851
ENSG00000084073	71	3815	2757	3520
ENSG00000084090	57	102	103	123
ENSG00000084092	20	80	20	45
ENSG00000084093	272	633	828	542
ENSG00000084110	2	0	2	3
ENSG00000084112	314	1431	1354	2131
ENSG00000084207	283	105	21	95
ENSG00000084234	45	139	110	310
ENSG00000084444	0	41	16	6
ENSG00000084453	1	0	0	0
ENSG00000084463	113	476	315	417
ENSG00000084623	133	80	24	85
ENSG00000084628	0	0	0	0
ENSG00000084636	0	0	0	0
ENSG00000084652	11	180	89	159
ENSG00000084674	0	0	0	0
ENSG00000084676	601	4009	5556	5828
ENSG00000084693	343	25878	19618	11541
ENSG00000084710	1	0	3	0

ENSG00000084731	375	999	406	487
ENSG00000084733	896	8680	9131	11103
ENSG00000084734	4	0	20	8
ENSG00000084754	154	242	343	297
ENSG00000084764	0	0	1	0
ENSG00000084774	1	0	1	3
ENSG00000085063	65	225	258	159
ENSG00000085117	1374	770	674	748
ENSG00000085185	21	13	38	39
ENSG00000085224	710	4531	3336	5333
ENSG00000085231	93	307	452	376
ENSG00000085265	21	0	1	2
ENSG00000085274	6	34	31	17
ENSG00000085276	17	40	56	63
ENSG00000085365	60	1333	1649	1176
ENSG00000085377	4	1	23	58
ENSG00000085382	2	623	629	899
ENSG00000085415	13	82	154	216
ENSG00000085433	3	631	606	819
ENSG00000085449	80	1435	1530	1730
ENSG00000085465	0	0	0	1
ENSG00000085491	21	313	268	556
ENSG00000085511	13	368	488	582
ENSG00000085514	12	14	0	0
ENSG00000085552	0	2	0	0
ENSG00000085563	0	27	24	29
ENSG00000085644	4	0	3	7
ENSG00000085662	118	298	155	303
ENSG00000085719	135	297	430	653
ENSG00000085721	8	119	105	188
ENSG00000085733	5045	21688	14746	21446
ENSG00000085741	85	132	105	96
ENSG00000085760	31	237	248	369
ENSG00000085788	13	243	407	518
ENSG00000085831	1	0	0	0
ENSG00000085832	175	11342	9364	10633
ENSG00000085840	0	12	16	30
ENSG00000085871	12	408	217	379
ENSG00000085872	3	33	5	11
ENSG00000085978	2	1690	681	1337
ENSG00000085982	1	82	77	94
ENSG00000085998	5	0	2	0
ENSG00000085999	1	22	0	0
ENSG00000086015	1	27	22	42
ENSG00000086061	100	269	198	344
ENSG00000086062	213	1129	655	890

ENSG00000086065	138	2603	2690	4116
ENSG00000086102	138	2209	1337	1964
ENSG00000086159	1	0	0	0
ENSG00000086189	1524	490	739	428
ENSG00000086200	117	758	1166	2603
ENSG00000086205	0	0	0	0
ENSG00000086232	7792	22508	20638	13829
ENSG00000086288	3	17	7	10
ENSG00000086289	14	365	317	640
ENSG00000086300	50	22	14	8
ENSG00000086475	19	15	61	65
ENSG00000086504	36	99	58	69
ENSG00000086506	436	230	32	96
ENSG00000086544	0	0	11	34
ENSG00000086548	0	0	0	4
ENSG00000086570	0	0	0	0
ENSG00000086589	73	178	146	101
ENSG00000086598	73	2336	2625	4502
ENSG00000086619	13	372	470	552
ENSG00000086666	1238	25944	18393	18230
ENSG00000086696	0	0	0	0
ENSG00000086712	9	217	227	282
ENSG00000086717	0	0	2	2
ENSG00000086730	32	94	54	58
ENSG00000086758	66	2108	1973	3785
ENSG00000086827	5	112	136	209
ENSG00000086848	0	8	8	17
ENSG00000086967	0	0	0	0
ENSG00000086991	0	0	0	0
ENSG00000087008	4	0	0	4
ENSG00000087053	157	1569	1756	1956
ENSG00000087074	362	868	626	483
ENSG00000087076	2	0	1	0
ENSG00000087077	9	11	5	0
ENSG00000087085	0	0	0	0
ENSG00000087086	74469	22750	20109	24033
ENSG00000087087	19	0	15	14
ENSG00000087088	115	82	19	10
ENSG00000087095	378	27318	26947	34955
ENSG00000087111	69	111	45	109
ENSG00000087116	0	0	0	0
ENSG00000087128	0	0	0	0
ENSG00000087152	110	509	380	520
ENSG00000087157	17	103	38	127
ENSG00000087191	326	258	162	474
ENSG00000087206	479	24675	24698	28602

ENSG00000087237	408	220	147	439
ENSG00000087245	0	0	0	0
ENSG00000087250	0	0	0	0
ENSG00000087253	1	0	2	20
ENSG00000087258	261	526	339	544
ENSG00000087263	45	133	170	147
ENSG00000087266	15	0	2	3
ENSG00000087269	14	478	371	525
ENSG00000087274	421	1850	1388	3167
ENSG00000087299	4	42	133	87
ENSG00000087301	24	1560	1960	2207
ENSG00000087302	264	734	635	1095
ENSG00000087303	28	332	336	673
ENSG00000087338	63	542	419	603
ENSG00000087365	262	246	103	154
ENSG00000087448	23	71	100	82
ENSG00000087460	34804	41807	34169	41210
ENSG00000087470	603	6468	7489	10588
ENSG00000087494	0	0	0	1
ENSG00000087495	96	1035	806	1473
ENSG00000087502	37	293	465	588
ENSG00000087510	0	0	0	2
ENSG00000087586	6	1739	1017	1582
ENSG00000087589	48	244	157	161
ENSG00000087842	18	15	27	13
ENSG00000087884	12	18	24	25
ENSG00000087903	126	200	114	207
ENSG00000087916	0	0	0	0
ENSG00000087995	1	0	5	17
ENSG00000088002	0	0	6	0
ENSG00000088035	0	182	175	302
ENSG00000088038	11	137	91	189
ENSG00000088053	1388	2023	1880	2304
ENSG00000088179	61	1732	1724	2332
ENSG00000088205	97	77	59	57
ENSG00000088247	22	34	28	45
ENSG00000088256	0	0	2	0
ENSG00000088280	2	52	25	73
ENSG00000088298	13	130	25	88
ENSG00000088305	11	70	109	127
ENSG00000088320	0	0	0	0
ENSG00000088325	7	245	122	196
ENSG00000088340	0	0	0	0
ENSG00000088356	31	54	10	7
ENSG00000088367	1	0	1	4
ENSG00000088386	0	0	0	0

ENSG00000088387	7	64	22	14
ENSG00000088448	287	2612	2289	2612
ENSG00000088451	11	13	76	142
ENSG00000088538	0	0	1	30
ENSG00000088543	1	0	0	0
ENSG00000088682	5	14	18	31
ENSG00000088726	7918	6866	3769	5292
ENSG00000088727	16	56	34	139
ENSG00000088756	0	0	2	2
ENSG00000088766	146	649	743	1073
ENSG00000088782	0	0	0	0
ENSG00000088808	1	11	1	4
ENSG00000088812	1	94	67	95
ENSG00000088826	3716	7346	5589	4285
ENSG00000088827	0	0	0	0
ENSG00000088832	4665	9851	9001	8609
ENSG00000088833	173	162	77	136
ENSG00000088836	13	14	4	0
ENSG00000088854	9	631	537	948
ENSG00000088876	0	0	3	0
ENSG00000088881	0	0	0	0
ENSG00000088882	0	0	4	8
ENSG00000088888	253	464	188	414
ENSG00000088899	1	0	4	1
ENSG00000088926	0	0	0	0
ENSG00000088930	121	942	1039	1240
ENSG00000088970	10	119	331	353
ENSG00000088986	17945	18848	12398	14949
ENSG00000088992	93	148	61	108
ENSG00000089006	151	1594	1031	1889
ENSG00000089009	948	281	260	416
ENSG00000089012	0	0	0	0
ENSG00000089022	3	327	362	612
ENSG00000089041	6	0	0	0
ENSG00000089048	45	197	61	108
ENSG00000089050	9	0	1	0
ENSG00000089053	2856	8520	6463	9461
ENSG00000089057	12	264	104	269
ENSG00000089060	3	0	1	4
ENSG00000089063	120	712	476	1572
ENSG00000089091	0	0	18	37
ENSG00000089094	5	55	17	43
ENSG00000089101	0	0	1	0
ENSG00000089116	0	0	0	0
ENSG00000089123	13	648	671	881
ENSG00000089127	39	0	2	3

ENSG00000089154	6	39	12	20
ENSG00000089157	502	92	73	199
ENSG00000089159	24	0	4	5
ENSG00000089163	0	0	0	0
ENSG00000089169	2	0	0	0
ENSG00000089177	29	88	228	335
ENSG00000089195	19	9	12	15
ENSG00000089199	0	0	1	0
ENSG00000089220	107	30	23	32
ENSG00000089225	0	0	0	0
ENSG00000089234	30	3122	2687	4081
ENSG00000089248	329	229	166	272
ENSG00000089250	0	0	0	0
ENSG00000089280	20	17	23	26
ENSG00000089289	256	1809	1886	1320
ENSG00000089327	3113	1778	3200	4626
ENSG00000089335	4	78	21	38
ENSG00000089351	5	0	6	4
ENSG00000089356	54	0	7	9
ENSG00000089472	2	23	26	71
ENSG00000089486	777	701	590	952
ENSG00000089505	2	0	4	7
ENSG00000089558	0	0	0	0
ENSG00000089597	45	182	138	270
ENSG00000089639	60	523	558	1110
ENSG00000089682	86	1103	794	1056
ENSG00000089685	6	4	1	11
ENSG00000089692	0	0	0	0
ENSG00000089693	32	5	9	7
ENSG00000089723	1	0	0	0
ENSG00000089737	220	151	194	228
ENSG00000089775	3	40	48	40
ENSG00000089818	4	0	19	37
ENSG00000089820	18	37	10	14
ENSG00000089847	0	0	2	0
ENSG00000089876	5	25	22	10
ENSG00000089902	70	1291	1078	1295
ENSG00000089916	98	3935	2277	4937
ENSG00000090006	4	25	24	40
ENSG00000090013	46	111	11	43
ENSG00000090020	97	630	469	1011
ENSG00000090054	7	228	295	622
ENSG00000090060	309	5820	5217	9062
ENSG00000090061	108	497	359	369
ENSG00000090097	2	0	0	8
ENSG00000090104	0	10	0	5

ENSG00000090238	311	819	460	281
ENSG00000090263	36	0	2	4
ENSG00000090266	248	15	21	20
ENSG00000090273	61	92	180	317
ENSG00000090316	30	101	29	30
ENSG00000090339	0	0	0	0
ENSG00000090372	250	1457	1053	1540
ENSG00000090376	60	7859	7274	8342
ENSG00000090382	46	110	205	385
ENSG00000090402	0	0	0	0
ENSG00000090432	9	6	6	13
ENSG00000090447	0	0	0	2
ENSG00000090470	29	63	24	45
ENSG00000090487	52	276	183	200
ENSG00000090512	0	0	0	0
ENSG00000090520	14	121	46	80
ENSG00000090530	1	0	9	37
ENSG00000090534	0	0	0	0
ENSG00000090539	0	0	0	0
ENSG00000090554	3	0	0	0
ENSG00000090565	39	347	141	230
ENSG00000090581	17	13	25	80
ENSG00000090612	8	14	35	98
ENSG00000090615	20	24	39	51
ENSG00000090621	52	26	22	30
ENSG00000090659	1	0	0	0
ENSG00000090661	1	5	8	23
ENSG00000090674	0	7	1	9
ENSG00000090686	333	5306	6017	8569
ENSG00000090776	138	533	334	203
ENSG00000090857	7	31	26	96
ENSG00000090861	15	452	428	559
ENSG00000090863	87	1586	860	1257
ENSG00000090889	6	203	147	250
ENSG00000090905	174	439	475	628
ENSG00000090920	0	0	0	0
ENSG00000090924	2	39	12	5
ENSG00000090932	0	0	0	0
ENSG00000090971	0	0	0	0
ENSG00000090975	350	2217	1294	2205
ENSG00000090989	23	151	257	270
ENSG00000091009	98	1000	725	847
ENSG00000091010	0	0	0	0
ENSG00000091039	642	4811	8468	5603
ENSG00000091073	0	9	6	3
ENSG00000091106	9	0	0	0

ENSG00000091127	3	1410	1672	2482
ENSG00000091128	0	0	0	0
ENSG00000091129	0	0	0	0
ENSG00000091136	0	0	2	3
ENSG00000091137	3	50	46	83
ENSG00000091138	0	0	0	0
ENSG00000091140	30	244	240	254
ENSG00000091157	20	3321	3507	4342
ENSG00000091164	221	1015	1482	1124
ENSG00000091181	0	0	0	3
ENSG00000091262	2	0	0	0
ENSG00000091317	1095	14432	10313	16418
ENSG00000091409	237	2045	2120	3201
ENSG00000091428	0	15	8	18
ENSG00000091436	30	0	2	14
ENSG00000091482	0	0	0	0
ENSG00000091483	13	10	33	46
ENSG00000091490	70	588	1333	198
ENSG00000091513	0	0	0	2
ENSG00000091527	170	1223	1256	1200
ENSG00000091536	0	0	0	0
ENSG00000091542	119	777	239	663
ENSG00000091583	0	0	0	0
ENSG00000091592	5	8	3	0
ENSG00000091622	1	0	0	0
ENSG00000091640	21	63	10	34
ENSG00000091651	5	44	7	22
ENSG00000091656	0	0	0	0
ENSG00000091664	0	0	0	0
ENSG00000091704	0	0	0	0
ENSG00000091732	3	321	160	273
ENSG00000091831	0	0	0	0
ENSG00000091844	5	0	29	6
ENSG00000091879	0	0	2	1
ENSG00000091947	11	0	0	0
ENSG00000091972	0	0	6	7
ENSG00000091986	5	20	19	20
ENSG00000092009	2	0	1	0
ENSG00000092010	394	99	70	125
ENSG00000092020	102	1234	921	1506
ENSG00000092036	3	27	10	19
ENSG00000092051	0	0	0	0
ENSG00000092054	0	0	0	0
ENSG00000092067	1	0	0	1
ENSG00000092068	0	15	6	10
ENSG00000092094	48	61	45	63

ENSG00000092096	3	27	37	18
ENSG00000092098	0	0	6	0
ENSG00000092108	117	7063	6868	10376
ENSG00000092140	21	681	517	751
ENSG00000092148	54	1829	2121	2734
ENSG00000092199	969	5631	4987	5033
ENSG00000092200	2	0	2	0
ENSG00000092201	125	174	325	653
ENSG00000092203	284	356	267	227
ENSG00000092208	4	100	34	65
ENSG00000092295	0	0	0	0
ENSG00000092330	92	194	210	276
ENSG00000092345	0	0	0	0
ENSG00000092377	0	0	0	1
ENSG00000092421	0	0	2	0
ENSG00000092439	15	4460	3852	5502
ENSG00000092445	0	0	0	0
ENSG00000092470	3	106	115	194
ENSG00000092529	0	0	0	0
ENSG00000092531	1962	23890	25984	38607
ENSG00000092607	0	0	0	0
ENSG00000092621	65	129	125	784
ENSG00000092758	0	0	0	0
ENSG00000092820	128	0	2	0
ENSG00000092841	40564	26669	13613	25636
ENSG00000092847	296	2100	1133	1850
ENSG00000092850	0	0	0	0
ENSG00000092853	19	231	150	176
ENSG00000092871	6	89	286	186
ENSG00000092929	158	1329	504	1005
ENSG00000092931	23	1978	891	1711
ENSG00000092964	62	445	537	660
ENSG00000092969	18	531	1252	724
ENSG00000092978	7	117	101	111
ENSG00000093000	68	962	811	745
ENSG00000093009	1	0	3	5
ENSG00000093010	67	235	73	133
ENSG00000093072	9	21	30	6
ENSG00000093100	0	0	0	0
ENSG00000093134	0	0	0	0
ENSG00000093144	35	32	41	50
ENSG00000093167	263	7797	7951	19424
ENSG00000093183	14	50	56	67
ENSG00000093217	3	0	0	6
ENSG00000094631	33	269	184	261
ENSG00000094661	0	0	0	0

ENSG00000094755	0	0	0	0
ENSG00000094796	0	0	0	0
ENSG00000094804	11	76	55	42
ENSG00000094841	18	1000	472	799
ENSG00000094880	3	100	179	280
ENSG00000094914	12	0	6	12
ENSG00000094916	37	342	252	306
ENSG00000094963	17	61	11	4
ENSG00000094975	35	7221	5092	5845
ENSG00000095002	5	359	354	327
ENSG00000095015	35	704	607	927
ENSG00000095059	44	390	147	252
ENSG00000095066	5	0	2	6
ENSG00000095110	0	0	1	6
ENSG00000095139	73	1799	1582	2067
ENSG00000095203	0	0	0	2
ENSG00000095209	2	116	154	159
ENSG00000095261	9	136	213	252
ENSG00000095303	12494	45885	39550	51402
ENSG00000095319	4	49	12	18
ENSG00000095321	303	1704	972	1187
ENSG00000095370	8	2	2	6
ENSG00000095380	12	40	36	55
ENSG00000095383	1	0	0	6
ENSG00000095397	0	0	0	0
ENSG00000095464	0	0	0	0
ENSG00000095485	12	118	91	149
ENSG00000095539	0	0	0	4
ENSG00000095564	5	1697	1483	2278
ENSG00000095574	23	81	142	133
ENSG00000095585	4	2	0	1
ENSG00000095587	0	0	0	0
ENSG00000095596	0	0	0	0
ENSG00000095627	0	33	2	0
ENSG00000095637	18	59	177	78
ENSG00000095713	0	0	0	0
ENSG00000095739	43	144	123	43
ENSG00000095752	0	0	0	0
ENSG00000095777	0	0	0	0
ENSG00000095787	555	4331	5840	4939
ENSG00000095794	180	765	936	1263
ENSG00000095906	4	0	3	6
ENSG00000095917	0	0	0	0
ENSG00000095932	2	0	4	4
ENSG00000095951	15	112	146	130
ENSG00000095970	1	0	0	0

ENSG00000095981	0	0	0	0
ENSG00000096006	0	0	0	5
ENSG00000096060	31	77	120	154
ENSG00000096063	30	321	352	319
ENSG00000096070	4	18	18	3
ENSG00000096080	2	0	12	12
ENSG00000096088	0	0	0	0
ENSG00000096092	8	56	22	89
ENSG00000096093	14	301	175	242
ENSG00000096150	0	0	0	0
ENSG00000096155	0	0	0	0
ENSG00000096171	0	0	0	0
ENSG00000096264	0	0	0	0
ENSG00000096384	539	183	111	187
ENSG00000096395	0	0	0	0
ENSG00000096401	171	1190	1234	1515
ENSG00000096433	0	0	2	0
ENSG00000096654	0	0	18	14
ENSG00000096696	4	8	0	8
ENSG00000096717	5	252	157	314
ENSG00000096746	345	1689	1431	1147
ENSG00000096872	111	1465	1501	2279
ENSG00000096968	230	7595	10198	8775
ENSG00000096996	5	0	0	0
ENSG00000097007	2	0	6	2
ENSG00000097021	169	125	144	324
ENSG00000097033	375	5602	7143	7870
ENSG00000097046	1	55	112	75
ENSG00000097096	0	10	44	55
ENSG00000099139	14	0	0	0
ENSG00000099194	1	0	5	6
ENSG00000099203	0	34	31	28
ENSG00000099204	891	4034	5122	3251
ENSG00000099219	5	354	321	346
ENSG00000099246	54	573	846	1211
ENSG00000099250	2	1	0	7
ENSG00000099251	0	0	3	7
ENSG00000099256	229	2081	2534	2064
ENSG00000099260	9	24	15	11
ENSG00000099282	8	779	418	843
ENSG00000099284	3	0	0	3
ENSG00000099290	5	89	81	221
ENSG00000099308	7	15	42	43
ENSG00000099326	1	0	13	5
ENSG00000099330	9	29	4	0
ENSG00000099331	51	935	382	761

ENSG00000099337	254	455	251	318
ENSG00000099338	2	0	11	8
ENSG00000099341	62	92	21	55
ENSG00000099364	0	2	4	16
ENSG00000099365	4	0	11	8
ENSG00000099377	0	0	0	0
ENSG00000099381	2	0	1	3
ENSG00000099385	6	0	3	7
ENSG00000099399	0	0	0	0
ENSG00000099617	0	0	0	0
ENSG00000099622	332	724	367	543
ENSG00000099624	26	0	5	13
ENSG00000099625	0	0	0	0
ENSG00000099715	3	1	4	0
ENSG00000099721	0	0	0	0
ENSG00000099725	4	25	0	48
ENSG00000099769	0	0	0	0
ENSG00000099783	134	3590	3545	7172
ENSG00000099785	5955	8339	5097	7830
ENSG00000099795	40	13	16	53
ENSG00000099797	57	15	40	86
ENSG00000099800	24	0	0	1
ENSG00000099804	22	142	62	72
ENSG00000099810	4	102	240	198
ENSG00000099812	0	0	0	0
ENSG00000099814	0	0	0	0
ENSG00000099817	343	384	282	366
ENSG00000099821	4	0	0	0
ENSG00000099822	0	0	0	0
ENSG00000099834	0	0	0	0
ENSG00000099840	0	0	0	0
ENSG00000099849	0	0	0	0
ENSG00000099860	4	0	3	0
ENSG00000099864	0	0	0	0
ENSG00000099866	0	0	0	0
ENSG00000099869	0	0	0	0
ENSG00000099875	30	28	10	21
ENSG00000099889	0	0	0	0
ENSG00000099899	3	0	0	0
ENSG00000099901	11	0	15	7
ENSG00000099904	0	0	0	0
ENSG00000099910	0	15	4	1
ENSG00000099917	9	157	55	93
ENSG00000099937	0	0	0	5
ENSG00000099940	578	1628	1690	1749
ENSG00000099942	227	7592	6442	6653

ENSG00000099949	7	0	20	13
ENSG00000099953	0	0	1	0
ENSG00000099954	18	76	75	102
ENSG00000099956	8	33	189	373
ENSG00000099957	1	0	0	0
ENSG00000099958	4	0	1	1
ENSG00000099960	0	0	0	0
ENSG00000099968	16	246	292	417
ENSG00000099974	0	3	1	0
ENSG00000099977	2	55	15	44
ENSG00000099984	0	0	0	0
ENSG00000099985	0	0	0	0
ENSG00000099991	26	32	62	196
ENSG00000099992	3	0	2	0
ENSG00000099994	1	0	0	0
ENSG00000099995	244	863	420	652
ENSG00000099998	0	0	0	0
ENSG00000099999	19	19	34	33
ENSG00000100003	6	50	9	1
ENSG00000100012	0	0	0	0
ENSG00000100014	9	0	7	12
ENSG00000100023	4	35	53	55
ENSG00000100024	7	0	2	17
ENSG00000100027	17	12	11	10
ENSG00000100028	172	213	147	331
ENSG00000100029	4	0	2	0
ENSG00000100030	586	6964	7608	7575
ENSG00000100031	4	0	21	11
ENSG00000100033	1	0	0	0
ENSG00000100034	12	0	3	11
ENSG00000100036	0	0	1	0
ENSG00000100038	0	0	5	0
ENSG00000100053	0	0	0	0
ENSG00000100055	26	8	1	4
ENSG00000100056	8	42	7	8
ENSG00000100058	20	30	26	62
ENSG00000100060	238	88	147	155
ENSG00000100065	2	0	0	1
ENSG00000100068	0	0	2	0
ENSG00000100075	21	14	24	21
ENSG00000100077	433	4350	3566	8067
ENSG00000100078	0	0	0	1
ENSG00000100079	99	21	1	0
ENSG00000100083	11	46	20	14
ENSG00000100084	5	7	2	19
ENSG00000100092	11	10	3	4

ENSG00000100095	0	0	0	0
ENSG00000100097	432	153	46	30
ENSG00000100099	35	732	371	698
ENSG00000100100	6	0	3	0
ENSG00000100101	0	0	0	0
ENSG00000100104	0	0	0	1
ENSG00000100105	6	0	19	4
ENSG00000100106	17	48	20	16
ENSG00000100109	10	40	9	15
ENSG00000100116	0	37	22	30
ENSG00000100121	0	0	0	0
ENSG00000100122	4	0	0	0
ENSG00000100124	20	32	9	17
ENSG00000100129	27	6	5	1
ENSG00000100138	408	274	184	455
ENSG00000100139	7	0	0	0
ENSG00000100142	38	0	0	20
ENSG00000100146	0	0	0	0
ENSG00000100147	0	38	28	72
ENSG00000100150	37	659	517	1005
ENSG00000100151	0	0	0	0
ENSG00000100154	36	213	59	76
ENSG00000100156	0	0	0	0
ENSG00000100162	2	0	1	0
ENSG00000100167	1	0	0	0
ENSG00000100170	0	0	1	2
ENSG00000100181	8418	8322	8267	11066
ENSG00000100191	0	0	3	0
ENSG00000100196	1	7	9	8
ENSG00000100197	0	0	0	0
ENSG00000100201	382	3793	4175	4065
ENSG00000100206	0	1154	930	1179
ENSG00000100207	82	620	343	548
ENSG00000100209	12	0	19	17
ENSG00000100211	12	25	24	30
ENSG00000100216	82	24	20	42
ENSG00000100218	21	163	123	57
ENSG00000100219	18	177	145	307
ENSG00000100220	34	83	16	21
ENSG00000100221	111	524	464	485
ENSG00000100225	1127	4061	3612	4502
ENSG00000100226	34	50	111	155
ENSG00000100227	54	66	107	80
ENSG00000100228	3	0	0	0
ENSG00000100234	11	147	23	714
ENSG00000100239	11	583	407	691

ENSG00000100241	2	31	20	13
ENSG00000100242	1	4	2	2
ENSG00000100243	670	3496	2929	3930
ENSG00000100246	11	149	63	87
ENSG00000100249	0	0	0	0
ENSG00000100253	0	0	0	0
ENSG00000100258	1	23	1	2
ENSG00000100263	0	0	0	0
ENSG00000100266	327	8259	5892	5451
ENSG00000100271	2	8	44	33
ENSG00000100276	0	0	0	0
ENSG00000100280	55	461	312	525
ENSG00000100281	56	87	105	124
ENSG00000100284	3	0	0	0
ENSG00000100285	4	45	0	0
ENSG00000100288	0	0	0	0
ENSG00000100290	1	0	0	0
ENSG00000100292	87	0	1	0
ENSG00000100294	2	0	0	0
ENSG00000100296	23	104	94	92
ENSG00000100297	18	12	17	17
ENSG00000100298	1	0	2	0
ENSG00000100299	9	71	15	19
ENSG00000100300	46	12	9	13
ENSG00000100302	0	0	0	0
ENSG00000100304	4	0	7	2
ENSG00000100307	27	38	8	19
ENSG00000100311	123	231	209	150
ENSG00000100312	5	31	2	25
ENSG00000100314	0	0	2	9
ENSG00000100316	2316	364	70	210
ENSG00000100319	4	41	26	94
ENSG00000100320	1	13	3	21
ENSG00000100321	1	0	0	0
ENSG00000100324	5	0	3	3
ENSG00000100325	22	150	95	130
ENSG00000100330	18	662	338	454
ENSG00000100335	194	263	208	302
ENSG00000100336	1	0	2	1
ENSG00000100341	0	0	0	0
ENSG00000100342	4	0	2	0
ENSG00000100344	0	0	0	0
ENSG00000100345	3535	38305	12738	35832
ENSG00000100346	0	0	0	0
ENSG00000100347	11	0	2	6
ENSG00000100348	142	8	14	10

ENSG00000100350	0	0	0	0
ENSG00000100351	11082	59422	55676	58934
ENSG00000100353	134	144	138	345
ENSG00000100354	99	289	328	374
ENSG00000100359	20	121	70	168
ENSG00000100360	9	7	3	1
ENSG00000100362	9532	86	50	935
ENSG00000100364	58	45	19	49
ENSG00000100365	30	18	6	9
ENSG00000100368	9	20	3	12
ENSG00000100372	33	297	210	271
ENSG00000100373	0	0	0	0
ENSG00000100376	3	22	64	84
ENSG00000100379	1	0	0	0
ENSG00000100380	371	1961	1994	3057
ENSG00000100385	25	0	0	0
ENSG00000100387	129	71	91	65
ENSG00000100393	227	3090	2273	3198
ENSG00000100395	59	198	69	44
ENSG00000100399	10	48	45	51
ENSG00000100401	14	130	23	49
ENSG00000100403	5	162	58	196
ENSG00000100410	50	127	163	163
ENSG00000100412	10	96	26	105
ENSG00000100413	4	3	1	4
ENSG00000100416	0	41	51	96
ENSG00000100417	14	10	4	12
ENSG00000100418	36	263	177	241
ENSG00000100422	37	17	3	0
ENSG00000100425	6	19	43	47
ENSG00000100426	22	76	83	140
ENSG00000100427	32	257	269	401
ENSG00000100429	0	0	0	0
ENSG00000100433	2	0	0	2
ENSG00000100439	291	1237	677	1022
ENSG00000100441	7	0	37	56
ENSG00000100442	85	4296	2692	4119
ENSG00000100445	2	0	14	9
ENSG00000100448	1	0	1	0
ENSG00000100450	52	0	1	0
ENSG00000100453	54	0	1	0
ENSG00000100461	52	1257	1526	3651
ENSG00000100462	2	69	76	144
ENSG00000100473	0	48	320	84
ENSG00000100478	78	589	724	994
ENSG00000100479	1	5039	3147	5931

ENSG00000100483	2	16	11	21
ENSG00000100485	42	3760	4349	5665
ENSG00000100490	506	3960	3795	2176
ENSG00000100503	752	4429	5773	6498
ENSG00000100504	1011	6841	5494	7291
ENSG00000100505	14	103	42	30
ENSG00000100519	42	306	406	550
ENSG00000100522	1	40	23	29
ENSG00000100523	5	184	170	184
ENSG00000100526	11	303	521	809
ENSG00000100528	35	213	209	507
ENSG00000100532	322	1674	1809	1858
ENSG00000100554	394	1045	709	1285
ENSG00000100557	1	0	0	0
ENSG00000100558	0	0	2	2
ENSG00000100564	2	73	26	56
ENSG00000100565	0	0	0	0
ENSG00000100567	163	397	194	306
ENSG00000100568	332	3213	3502	4101
ENSG00000100575	24	49	52	37
ENSG00000100577	5	6	0	0
ENSG00000100578	21	588	599	868
ENSG00000100580	177	1357	1317	2458
ENSG00000100583	0	0	1	13
ENSG00000100591	42	226	138	190
ENSG00000100592	1442	10176	10363	13694
ENSG00000100593	0	0	0	0
ENSG00000100596	33	605	603	858
ENSG00000100599	17	177	47	99
ENSG00000100600	153	601	517	948
ENSG00000100601	3	0	5	14
ENSG00000100603	321	2114	1134	1216
ENSG00000100604	0	0	0	1
ENSG00000100605	29	16	51	29
ENSG00000100612	242	1294	1035	514
ENSG00000100614	1316	18296	25149	16275
ENSG00000100625	2	0	0	1
ENSG00000100626	0	0	0	0
ENSG00000100628	2	0	0	0
ENSG00000100629	74	1608	1382	2308
ENSG00000100632	125	156	102	192
ENSG00000100644	122	3236	4126	3521
ENSG00000100647	56	440	389	367
ENSG00000100650	214	397	259	198
ENSG00000100652	0	0	0	0
ENSG00000100664	791	3199	2796	2391

ENSG00000100665	0	0	0	0
ENSG00000100678	109	1925	1435	1665
ENSG00000100697	104	1855	1967	2061
ENSG00000100711	256	770	561	586
ENSG00000100714	8	137	77	183
ENSG00000100721	35	0	2	1
ENSG00000100722	20	952	667	840
ENSG00000100726	1	0	1	0
ENSG00000100731	8	565	387	680
ENSG00000100739	0	0	0	0
ENSG00000100744	23	155	191	226
ENSG00000100749	40	4025	3246	5284
ENSG00000100764	83	119	169	336
ENSG00000100767	1	0	1	0
ENSG00000100784	4	521	553	755
ENSG00000100796	65	4519	3375	4333
ENSG00000100802	5	1	1	14
ENSG00000100804	36	7	14	33
ENSG00000100811	902	1599	1664	1466
ENSG00000100813	34	129	78	139
ENSG00000100814	45	167	142	227
ENSG00000100815	334	3891	5167	5803
ENSG00000100823	41	160	114	61
ENSG00000100836	328	207	216	206
ENSG00000100842	0	0	0	0
ENSG00000100852	291	3613	5517	4650
ENSG00000100865	21	16	4	9
ENSG00000100867	0	0	5	0
ENSG00000100883	27	760	899	1124
ENSG00000100884	0	0	0	0
ENSG00000100888	34	246	201	315
ENSG00000100889	34	59	44	37
ENSG00000100890	0	0	0	0
ENSG00000100897	102	147	116	117
ENSG00000100902	21	167	126	155
ENSG00000100906	109	92	79	81
ENSG00000100908	2	0	0	4
ENSG00000100911	363	8	7	25
ENSG00000100916	1	176	180	362
ENSG00000100918	3	0	5	0
ENSG00000100926	0	0	0	0
ENSG00000100934	95	1306	1393	1695
ENSG00000100938	92	298	200	225
ENSG00000100941	48	2804	2137	3688
ENSG00000100949	8	0	2	5
ENSG00000100968	0	0	0	0

ENSG00000100979	71	284	172	90
ENSG00000100982	64	128	56	88
ENSG00000100983	95	165	191	128
ENSG00000100985	0	0	0	0
ENSG00000100987	0	0	0	0
ENSG00000100991	14	93	86	95
ENSG00000100994	841	3494	2393	4125
ENSG00000100997	4	56	38	73
ENSG00000101000	6	0	0	0
ENSG00000101003	1	310	311	479
ENSG00000101004	0	0	4	10
ENSG00000101017	83	79	164	196
ENSG00000101019	4	583	431	716
ENSG00000101040	143	8108	5900	10036
ENSG00000101049	0	0	1	5
ENSG00000101052	18	48	62	90
ENSG00000101057	20	131	18	17
ENSG00000101074	0	0	0	0
ENSG00000101076	0	0	0	0
ENSG00000101079	70	680	531	591
ENSG00000101082	2029	6044	4962	6959
ENSG00000101084	4	33	30	10
ENSG00000101096	370	816	478	1903
ENSG00000101098	0	0	0	0
ENSG00000101104	0	0	6	7
ENSG00000101109	1995	14352	16262	16613
ENSG00000101115	0	0	0	0
ENSG00000101126	116	1272	1067	793
ENSG00000101132	26	35	17	31
ENSG00000101134	0	0	0	0
ENSG00000101138	14	62	17	28
ENSG00000101144	0	0	0	0
ENSG00000101146	20	0	25	13
ENSG00000101150	71	69	65	127
ENSG00000101152	17	381	210	279
ENSG00000101158	168	473	401	385
ENSG00000101160	35	148	97	121
ENSG00000101161	56	76	46	68
ENSG00000101162	36286	182307	201866	223031
ENSG00000101166	38	229	253	373
ENSG00000101180	0	0	0	0
ENSG00000101181	7	0	2	5
ENSG00000101182	969	349	206	428
ENSG00000101187	0	0	1	0
ENSG00000101188	0	0	0	0
ENSG00000101189	4	9	2	1

ENSG00000101190	2	79	28	52
ENSG00000101191	36	93	109	92
ENSG00000101193	144	220	185	290
ENSG00000101194	0	0	0	0
ENSG00000101197	0	0	0	0
ENSG00000101198	0	0	0	0
ENSG00000101199	0	23	3	3
ENSG00000101200	0	0	0	0
ENSG00000101203	0	0	0	0
ENSG00000101204	0	0	0	0
ENSG00000101210	0	0	0	0
ENSG00000101213	2	0	0	0
ENSG00000101216	2	0	8	15
ENSG00000101220	7	15	3	9
ENSG00000101222	0	0	0	0
ENSG00000101224	24	32	3	5
ENSG00000101230	1	0	0	0
ENSG00000101236	433	2235	3183	5094
ENSG00000101246	9	39	32	46
ENSG00000101247	3	34	25	55
ENSG00000101251	0	0	0	3
ENSG00000101255	1	0	0	1
ENSG00000101265	49	22	13	14
ENSG00000101266	64	498	347	562
ENSG00000101276	0	0	0	0
ENSG00000101278	0	0	0	9
ENSG00000101280	0	0	1	0
ENSG00000101282	0	0	0	0
ENSG00000101290	289	2290	2286	4937
ENSG00000101292	0	0	0	0
ENSG00000101294	6	183	147	264
ENSG00000101298	32	185	89	76
ENSG00000101306	0	0	0	1
ENSG00000101307	3	0	0	0
ENSG00000101310	18	359	283	343
ENSG00000101311	0	0	7	33
ENSG00000101321	0	0	0	0
ENSG00000101323	0	0	0	0
ENSG00000101327	0	0	0	0
ENSG00000101331	1	0	0	1
ENSG00000101333	50	2281	2348	3553
ENSG00000101335	261	7631	3201	9453
ENSG00000101336	47	1	7	0
ENSG00000101337	44	140	239	237
ENSG00000101342	1	0	0	0
ENSG00000101343	26	239	194	255

ENSG00000101346	0	16	0	0
ENSG00000101347	395	94	42	31
ENSG00000101349	0	17	0	0
ENSG00000101350	13	469	324	709
ENSG00000101353	1	57	39	52
ENSG00000101361	67	26	21	33
ENSG00000101363	4	11	4	6
ENSG00000101365	62	38	33	36
ENSG00000101367	423	5360	5015	6008
ENSG00000101384	1	16	12	22
ENSG00000101391	2	70	81	148
ENSG00000101400	2	0	0	0
ENSG00000101405	0	0	0	0
ENSG00000101407	0	4	6	4
ENSG00000101412	90	216	86	82
ENSG00000101413	7	808	417	667
ENSG00000101417	63	54	34	62
ENSG00000101421	205	785	384	632
ENSG00000101425	2	0	0	8
ENSG00000101435	1	0	0	0
ENSG00000101438	0	0	0	0
ENSG00000101439	3217	2601	2546	2617
ENSG00000101440	0	0	0	0
ENSG00000101441	0	0	0	0
ENSG00000101442	3	8	0	5
ENSG00000101443	0	0	0	0
ENSG00000101444	14	0	0	11
ENSG00000101445	7	0	4	0
ENSG00000101446	0	0	0	0
ENSG00000101447	44	333	198	180
ENSG00000101448	0	0	0	2
ENSG00000101452	2	16	8	22
ENSG00000101457	89	122	102	145
ENSG00000101460	9	30	14	40
ENSG00000101463	1	0	0	0
ENSG00000101464	0	72	89	128
ENSG00000101470	4532	1597	987	1623
ENSG00000101473	51	181	70	117
ENSG00000101474	28	152	20	60
ENSG00000101489	1	0	0	0
ENSG00000101493	3	14	2	10
ENSG00000101542	2	0	12	9
ENSG00000101544	23	22	38	23
ENSG00000101546	12	99	8	18
ENSG00000101557	70	1320	1722	2160
ENSG00000101558	1307	7433	8287	6482

ENSG00000101574	41	249	191	170
ENSG00000101577	116	593	658	541
ENSG00000101596	51	1990	2483	3158
ENSG00000101605	105	2507	1635	2691
ENSG00000101608	22118	135391	80609	130295
ENSG00000101624	2	155	83	136
ENSG00000101638	19	0	0	13
ENSG00000101639	6	4078	4335	5669
ENSG00000101654	27	160	68	112
ENSG00000101665	0	0	0	3
ENSG00000101670	0	0	0	0
ENSG00000101680	1	0	0	0
ENSG00000101695	36	0	1	0
ENSG00000101745	359	20221	19601	24914
ENSG00000101746	0	0	0	0
ENSG00000101751	4	2824	3061	4540
ENSG00000101752	59	3015	2581	2605
ENSG00000101773	53	1287	1447	2095
ENSG00000101782	6353	26528	28470	22268
ENSG00000101811	3	0	4	5
ENSG00000101812	0	0	0	0
ENSG00000101825	0	0	0	0
ENSG00000101842	0	0	1	0
ENSG00000101843	38	203	138	148
ENSG00000101844	35	210	167	245
ENSG00000101846	6	31	92	46
ENSG00000101849	89	126	114	98
ENSG00000101850	0	0	0	0
ENSG00000101856	13568	58131	74790	80789
ENSG00000101868	12	922	992	1296
ENSG00000101871	2	0	5	4
ENSG00000101882	28	90	83	173
ENSG00000101883	0	0	0	0
ENSG00000101888	111	1034	1650	1480
ENSG00000101890	0	0	1	0
ENSG00000101892	0	0	0	0
ENSG00000101898	0	0	0	0
ENSG00000101901	32	327	280	418
ENSG00000101911	20	106	163	212
ENSG00000101916	1	0	1	1
ENSG00000101928	7	56	14	24
ENSG00000101935	18	0	92	131
ENSG00000101938	0	0	0	0
ENSG00000101940	249	402	271	353
ENSG00000101945	23	83	39	21
ENSG00000101951	0	0	0	0

ENSG00000101955	0	0	0	0
ENSG00000101958	0	0	10	19
ENSG00000101966	74	554	513	640
ENSG00000101972	670	6008	8207	6103
ENSG00000101974	16	608	814	1153
ENSG00000101977	0	11	13	33
ENSG00000101981	0	0	0	0
ENSG00000101986	4	125	16	38
ENSG00000101997	2	24	2	4
ENSG00000102001	2	4	11	15
ENSG00000102003	9	0	1	0
ENSG00000102007	33	89	24	52
ENSG00000102010	0	0	0	0
ENSG00000102021	0	0	0	0
ENSG00000102024	21	425	475	347
ENSG00000102030	75	10	27	36
ENSG00000102032	0	130	39	33
ENSG00000102034	11	59	24	19
ENSG00000102038	1	26	6	11
ENSG00000102043	3	32	32	39
ENSG00000102048	0	0	0	1
ENSG00000102053	20	172	244	466
ENSG00000102054	132	1228	967	1117
ENSG00000102055	0	0	0	0
ENSG00000102057	1	0	0	0
ENSG00000102069	0	0	0	0
ENSG00000102076	0	0	0	0
ENSG00000102078	5	0	1	4
ENSG00000102080	0	0	0	0
ENSG00000102081	53	759	885	927
ENSG00000102096	21	0	13	0
ENSG00000102098	0	51	78	112
ENSG00000102100	7	0	4	1
ENSG00000102103	24	0	1	4
ENSG00000102104	0	0	0	0
ENSG00000102109	1	0	5	4
ENSG00000102119	224	649	514	909
ENSG00000102125	9	0	7	17
ENSG00000102128	0	0	1	0
ENSG00000102144	823	4335	4137	5509
ENSG00000102145	339	354	132	194
ENSG00000102158	61	759	625	1351
ENSG00000102172	258	2117	2003	2630
ENSG00000102174	17	177	63	232
ENSG00000102178	2404	3310	2773	2973
ENSG00000102181	6	308	520	836

ENSG00000102189	80	2859	3055	3799
ENSG00000102195	0	0	0	0
ENSG00000102218	30	504	784	623
ENSG00000102221	7	19	61	27
ENSG00000102225	98	286	231	463
ENSG00000102226	65	86	58	109
ENSG00000102230	336	4614	4400	3577
ENSG00000102239	0	0	0	0
ENSG00000102241	50	40	39	47
ENSG00000102243	0	0	0	0
ENSG00000102245	48	628	1046	688
ENSG00000102265	13244	3781	3850	3225
ENSG00000102271	2	0	0	1
ENSG00000102287	7	16	50	7
ENSG00000102290	0	0	0	0
ENSG00000102302	1	0	0	0
ENSG00000102309	107	73	138	56
ENSG00000102312	0	0	0	0
ENSG00000102313	0	0	0	0
ENSG00000102316	751	3141	2247	2744
ENSG00000102317	349	292	193	375
ENSG00000102349	2	0	18	35
ENSG00000102359	0	3	2	0
ENSG00000102362	652	4834	3914	4235
ENSG00000102383	5	605	317	479
ENSG00000102384	0	1012	999	1097
ENSG00000102385	1	0	4	0
ENSG00000102387	11	85	35	15
ENSG00000102390	27	0	12	15
ENSG00000102393	1429	4294	6810	5362
ENSG00000102401	1183	4970	6552	4869
ENSG00000102409	254	797	761	652
ENSG00000102445	0	0	0	0
ENSG00000102452	0	0	0	0
ENSG00000102466	0	0	0	0
ENSG00000102468	68	172	180	189
ENSG00000102471	1	0	4	1
ENSG00000102524	428	1315	924	1518
ENSG00000102531	12	4244	3331	6375
ENSG00000102539	0	14	0	0
ENSG00000102543	0	32	52	54
ENSG00000102547	4	26	37	62
ENSG00000102554	1	0	0	0
ENSG00000102572	1015	8779	6988	8219
ENSG00000102575	3	0	0	0
ENSG00000102580	66	9061	8369	13208

ENSG00000102595	12	3663	4602	4979
ENSG00000102606	28	806	584	648
ENSG00000102678	0	0	0	0
ENSG00000102683	0	0	0	0
ENSG00000102699	20	802	383	634
ENSG00000102710	24	2052	1405	2201
ENSG00000102738	30	158	145	236
ENSG00000102743	27	603	560	812
ENSG00000102753	329	2752	3645	3828
ENSG00000102755	7	83	56	98
ENSG00000102760	311	1350	1028	1109
ENSG00000102763	462	3233	5295	4852
ENSG00000102780	8	770	1032	1026
ENSG00000102781	37	664	1399	1933
ENSG00000102786	40	813	1365	1190
ENSG00000102794	0	0	0	0
ENSG00000102796	4	0	6	5
ENSG00000102802	0	0	0	0
ENSG00000102804	10655	54182	45546	31630
ENSG00000102805	8	16	26	38
ENSG00000102837	1	0	0	19
ENSG00000102854	0	0	0	0
ENSG00000102858	6	234	102	142
ENSG00000102870	4	15	1	2
ENSG00000102871	8	25	8	4
ENSG00000102878	0	0	0	0
ENSG00000102879	60	51	33	67
ENSG00000102882	79	109	73	98
ENSG00000102886	2	0	0	0
ENSG00000102890	1	0	0	0
ENSG00000102891	0	0	0	0
ENSG00000102893	1089	24536	23812	24466
ENSG00000102897	224	911	945	574
ENSG00000102898	419	1229	927	1166
ENSG00000102900	19	87	29	60
ENSG00000102901	509	1197	490	582
ENSG00000102904	0	20	57	86
ENSG00000102908	270	4668	8083	4884
ENSG00000102910	40	670	587	759
ENSG00000102921	27	45	87	74
ENSG00000102924	0	0	0	0
ENSG00000102931	37	137	123	203
ENSG00000102934	0	0	0	0
ENSG00000102935	0	0	0	0
ENSG00000102962	0	0	0	0
ENSG00000102967	2	5	0	0

ENSG00000102970	0	0	0	0
ENSG00000102974	414	2543	2350	3647
ENSG00000102977	105	125	44	86
ENSG00000102978	10	26	4	32
ENSG00000102981	27	18	6	15
ENSG00000102984	6	0	23	42
ENSG00000102996	0	0	0	0
ENSG00000103005	43	14	24	19
ENSG00000103018	32	16	49	87
ENSG00000103021	0	0	1	0
ENSG00000103023	0	0	0	0
ENSG00000103024	15	4	1	1
ENSG00000103034	2	0	0	0
ENSG00000103035	44	323	274	385
ENSG00000103037	0	0	6	7
ENSG00000103042	3	4	11	6
ENSG00000103043	20	35	37	47
ENSG00000103044	0	0	4	3
ENSG00000103047	3	68	45	79
ENSG00000103051	16	84	40	64
ENSG00000103056	1	0	0	0
ENSG00000103061	3	0	29	44
ENSG00000103064	1	67	25	22
ENSG00000103066	7	32	19	19
ENSG00000103067	0	0	0	0
ENSG00000103089	0	0	0	0
ENSG00000103091	4	538	324	694
ENSG00000103111	31	17	55	87
ENSG00000103121	596	1191	1155	1066
ENSG00000103126	39	333	170	442
ENSG00000103145	78	32	36	56
ENSG00000103148	296	1254	288	1770
ENSG00000103150	3	9	10	14
ENSG00000103152	7	25	4	3
ENSG00000103154	0	0	0	0
ENSG00000103160	161	1570	2327	1067
ENSG00000103168	0	29	2	0
ENSG00000103174	3	7	6	4
ENSG00000103175	12	201	50	95
ENSG00000103184	251	1923	924	1455
ENSG00000103187	2829	5662	2990	6159
ENSG00000103194	462	3347	1766	6382
ENSG00000103196	0	0	0	1
ENSG00000103197	8	5	5	13
ENSG00000103199	2	0	0	0
ENSG00000103200	0	0	0	0

ENSG00000103202	213	286	81	566
ENSG00000103222	82	2345	1385	1551
ENSG00000103226	5	86	63	50
ENSG00000103227	0	0	3	0
ENSG00000103241	0	0	0	0
ENSG00000103245	0	0	0	2
ENSG00000103248	2	0	3	2
ENSG00000103249	0	36	4	34
ENSG00000103253	0	0	0	0
ENSG00000103254	0	0	0	0
ENSG00000103257	34	169	50	111
ENSG00000103260	0	0	0	3
ENSG00000103264	3	2	5	14
ENSG00000103266	21	153	46	113
ENSG00000103269	0	0	0	0
ENSG00000103274	2	9	33	56
ENSG00000103275	153	129	192	309
ENSG00000103310	0	0	0	0
ENSG00000103313	12	19	0	1
ENSG00000103316	51	54	59	29
ENSG00000103319	9	103	62	43
ENSG00000103326	0	0	3	0
ENSG00000103335	1	0	1	5
ENSG00000103342	82	1052	1106	1464
ENSG00000103343	2	0	5	6
ENSG00000103351	5	32	49	44
ENSG00000103353	5	0	16	34
ENSG00000103355	0	0	0	0
ENSG00000103356	0	0	0	0
ENSG00000103363	2413	322	97	230
ENSG00000103365	19	51	37	50
ENSG00000103375	0	0	0	0
ENSG00000103381	69	43	29	28
ENSG00000103404	179	2224	1941	2734
ENSG00000103415	34	0	6	8
ENSG00000103423	5	2	10	20
ENSG00000103426	0	0	0	0
ENSG00000103429	94	474	284	520
ENSG00000103449	0	10	0	0
ENSG00000103460	0	0	0	0
ENSG00000103472	4	276	289	401
ENSG00000103479	106	1517	1157	1485
ENSG00000103485	9	0	0	0
ENSG00000103489	1	169	47	144
ENSG00000103490	43	0	1	5
ENSG00000103494	1	37	13	22

ENSG00000103495	2	701	448	632
ENSG00000103496	98	21	34	44
ENSG00000103502	97	150	124	104
ENSG00000103507	39	92	44	131
ENSG00000103510	17	3	41	62
ENSG00000103512	310	2907	2562	2606
ENSG00000103522	0	23	11	43
ENSG00000103528	7	0	0	0
ENSG00000103534	0	0	0	1
ENSG00000103540	51	512	473	505
ENSG00000103544	21	1388	800	1075
ENSG00000103546	0	0	0	0
ENSG00000103549	35	162	58	147
ENSG00000103550	6	22	0	8
ENSG00000103569	2	17	0	7
ENSG00000103591	70	839	592	828
ENSG00000103599	0	72	37	61
ENSG00000103642	28	131	127	143
ENSG00000103647	0	0	0	0
ENSG00000103653	330	655	220	532
ENSG00000103657	313	6263	5439	6813
ENSG00000103671	49	216	233	262
ENSG00000103707	1	0	2	2
ENSG00000103710	0	0	0	0
ENSG00000103723	0	0	0	0
ENSG00000103740	209	839	915	1282
ENSG00000103742	0	0	0	0
ENSG00000103769	7707	80431	71096	110931
ENSG00000103811	10	17	0	4
ENSG00000103832	0	0	0	0
ENSG00000103852	0	376	143	261
ENSG00000103855	0	0	0	0
ENSG00000103876	640	962	407	930
ENSG00000103888	2	165	145	209
ENSG00000103932	2	0	0	0
ENSG00000103942	282	1940	1301	3541
ENSG00000103966	8	0	8	10
ENSG00000103978	16	699	810	1072
ENSG00000103994	256	355	442	577
ENSG00000103995	2	257	236	284
ENSG00000104043	1	626	1259	945
ENSG00000104044	0	0	0	0
ENSG00000104047	15	882	942	1504
ENSG00000104055	0	0	0	0
ENSG00000104059	4	0	18	16
ENSG00000104064	53	774	825	1093

ENSG00000104067	3	19	136	96
ENSG00000104081	5	0	2	0
ENSG00000104093	12	171	214	408
ENSG00000104112	0	0	0	0
ENSG00000104129	5	0	1	0
ENSG00000104131	904	791	876	1236
ENSG00000104133	17	3567	3720	4025
ENSG00000104140	0	0	0	0
ENSG00000104142	6	0	8	10
ENSG00000104147	0	12	2	1
ENSG00000104154	0	25	7	31
ENSG00000104164	2808	14156	10748	10772
ENSG00000104177	5	71	123	165
ENSG00000104205	8	0	1	1
ENSG00000104213	0	0	0	0
ENSG00000104218	165	2165	1450	2503
ENSG00000104219	52	776	961	1599
ENSG00000104221	0	0	5	14
ENSG00000104228	38	888	790	1324
ENSG00000104231	770	6121	6202	7990
ENSG00000104237	0	0	0	0
ENSG00000104267	14218	46896	25963	81535
ENSG00000104290	2	0	3	0
ENSG00000104299	4	35	14	32
ENSG00000104312	9	153	118	162
ENSG00000104313	0	0	0	0
ENSG00000104320	56	508	640	763
ENSG00000104321	0	59	28	14
ENSG00000104324	89	667	879	807
ENSG00000104325	36	252	311	338
ENSG00000104327	0	0	1	0
ENSG00000104331	73	707	1199	1075
ENSG00000104332	0	0	0	0
ENSG00000104341	222	2186	1906	1137
ENSG00000104343	103	1991	2853	1608
ENSG00000104356	8	19	20	45
ENSG00000104361	1	144	104	134
ENSG00000104365	11	192	120	239
ENSG00000104368	0	0	0	0
ENSG00000104369	0	0	0	0
ENSG00000104371	0	0	1	0
ENSG00000104375	46	4206	5185	7457
ENSG00000104381	10	476	263	178
ENSG00000104388	81	230	522	886
ENSG00000104408	304	974	798	1150
ENSG00000104412	95	3732	3468	7357

ENSG00000104413	0	0	0	0
ENSG00000104415	0	0	2	6
ENSG00000104419	29	0	14	6
ENSG00000104427	3	0	3	1
ENSG00000104432	17	569	495	498
ENSG00000104435	3	0	0	0
ENSG00000104442	27	351	269	356
ENSG00000104447	16	192	268	242
ENSG00000104450	4	92	129	232
ENSG00000104472	47	32	71	81
ENSG00000104490	7	0	1	0
ENSG00000104497	16	160	264	305
ENSG00000104499	0	0	0	0
ENSG00000104517	95	2631	3388	4288
ENSG00000104518	11	0	9	9
ENSG00000104522	15	25	18	25
ENSG00000104524	0	0	0	0
ENSG00000104529	273	259	95	156
ENSG00000104537	0	0	0	0
ENSG00000104549	8	57	124	138
ENSG00000104611	2	0	4	11
ENSG00000104613	19	205	209	415
ENSG00000104626	121	185	121	228
ENSG00000104635	1	0	0	0
ENSG00000104643	2	0	21	29
ENSG00000104660	71	676	815	858
ENSG00000104671	15	184	113	236
ENSG00000104679	11	0	1	0
ENSG00000104687	96	2460	2075	2222
ENSG00000104689	0	0	0	0
ENSG00000104691	3	39	41	31
ENSG00000104695	98	766	774	640
ENSG00000104714	704	731	572	386
ENSG00000104722	1	0	0	0
ENSG00000104723	0	0	0	13
ENSG00000104725	0	0	0	0
ENSG00000104728	6	31	71	98
ENSG00000104731	0	58	7	10
ENSG00000104738	23	132	85	95
ENSG00000104755	0	0	0	0
ENSG00000104756	126	598	841	559
ENSG00000104760	0	0	0	0
ENSG00000104763	1893	26271	22613	18718
ENSG00000104765	2602	9904	13043	10937
ENSG00000104774	0	39	65	136
ENSG00000104783	0	6	0	3

ENSG00000104804	1	0	2	7
ENSG00000104805	560	1720	1076	2642
ENSG00000104808	2	0	0	0
ENSG00000104812	7	70	32	57
ENSG00000104814	10	0	0	6
ENSG00000104818	0	0	0	0
ENSG00000104823	123	0	6	5
ENSG00000104824	93	453	608	563
ENSG00000104825	29	15	16	12
ENSG00000104826	0	0	0	0
ENSG00000104827	0	0	0	0
ENSG00000104833	1	0	0	0
ENSG00000104835	1	0	0	0
ENSG00000104848	0	0	4	0
ENSG00000104852	4	70	1	2
ENSG00000104853	0	82	18	58
ENSG00000104856	3	0	10	14
ENSG00000104859	3	5	5	4
ENSG00000104863	1	0	0	4
ENSG00000104866	0	0	0	0
ENSG00000104870	5	0	0	0
ENSG00000104872	238	386	112	175
ENSG00000104879	1	0	0	0
ENSG00000104880	3	0	5	3
ENSG00000104881	1	0	0	0
ENSG00000104883	0	0	0	0
ENSG00000104884	5	0	2	8
ENSG00000104885	0	131	108	286
ENSG00000104886	125	155	65	94
ENSG00000104888	0	0	0	0
ENSG00000104889	4	0	0	8
ENSG00000104892	2	0	0	0
ENSG00000104894	127	109	297	258
ENSG00000104897	40	233	170	188
ENSG00000104899	0	0	1	0
ENSG00000104901	0	0	0	0
ENSG00000104903	179	511	339	309
ENSG00000104904	15193	36862	27580	37212
ENSG00000104907	212	85	163	135
ENSG00000104915	27	0	9	11
ENSG00000104918	1	0	0	0
ENSG00000104921	7	0	0	0
ENSG00000104936	1	0	3	5
ENSG00000104938	0	8	1	0
ENSG00000104941	0	20	0	0
ENSG00000104946	14	31	12	53

ENSG00000104951	2	0	0	0
ENSG00000104953	0	0	0	0
ENSG00000104957	1	0	3	7
ENSG00000104960	5	36	21	7
ENSG00000104964	97	684	516	437
ENSG00000104967	0	0	0	0
ENSG00000104969	28	8	3	12
ENSG00000104970	0	0	0	0
ENSG00000104972	10	0	0	0
ENSG00000104973	1	46	9	8
ENSG00000104974	35	0	3	0
ENSG00000104976	5	0	2	1
ENSG00000104979	169	25	26	72
ENSG00000104980	2	0	0	6
ENSG00000104983	0	0	0	0
ENSG00000104998	36	87	30	79
ENSG00000105011	13	99	19	75
ENSG00000105048	6	29	4	15
ENSG00000105053	133	87	60	84
ENSG00000105058	1081	1875	1497	2104
ENSG00000105063	21	1041	650	1741
ENSG00000105072	0	45	10	61
ENSG00000105085	11	53	9	3
ENSG00000105088	0	0	0	0
ENSG00000105122	13	10	0	0
ENSG00000105127	10	33	35	64
ENSG00000105131	0	0	0	6
ENSG00000105135	0	0	0	0
ENSG00000105136	5	50	6	21
ENSG00000105137	0	0	0	0
ENSG00000105141	0	0	0	0
ENSG00000105143	0	0	0	0
ENSG00000105146	2	0	1	0
ENSG00000105171	25	93	36	65
ENSG00000105173	0	0	6	8
ENSG00000105176	37	378	388	607
ENSG00000105185	29	0	10	1
ENSG00000105186	19	661	514	573
ENSG00000105193	952	0	0	10
ENSG00000105197	8	3	1	8
ENSG00000105198	0	0	0	0
ENSG00000105202	81	0	3	3
ENSG00000105204	1	45	9	21
ENSG00000105205	12	0	6	5
ENSG00000105219	0	0	1	0
ENSG00000105220	581	751	611	731

ENSG00000105221	51	152	124	308
ENSG00000105223	8	37	15	16
ENSG00000105227	1	25	1	1
ENSG00000105229	17	206	112	80
ENSG00000105245	0	0	1	0
ENSG00000105246	1	0	0	0
ENSG00000105248	1	1	12	9
ENSG00000105251	0	0	0	0
ENSG00000105254	62	313	243	534
ENSG00000105255	0	0	0	0
ENSG00000105258	59	39	21	28
ENSG00000105261	0	0	0	0
ENSG00000105270	0	0	0	0
ENSG00000105278	1	0	0	0
ENSG00000105281	14	25	5	16
ENSG00000105287	45	9	30	39
ENSG00000105289	0	0	0	0
ENSG00000105290	1	0	0	2
ENSG00000105298	25	15	2	21
ENSG00000105321	0	66	30	66
ENSG00000105323	323	834	356	645
ENSG00000105325	2	16	21	29
ENSG00000105327	7	133	69	47
ENSG00000105329	136	12153	4656	10088
ENSG00000105339	70	1180	744	1031
ENSG00000105341	15	0	1	2
ENSG00000105352	0	0	0	0
ENSG00000105355	88	102	67	145
ENSG00000105357	0	0	0	0
ENSG00000105364	3	0	0	0
ENSG00000105366	3	0	0	3
ENSG00000105369	15	0	0	0
ENSG00000105370	0	0	0	0
ENSG00000105371	0	0	4	13
ENSG00000105372	4487	634	288	550
ENSG00000105373	32	71	7	8
ENSG00000105374	359	0	0	0
ENSG00000105376	0	70	10	15
ENSG00000105379	74	1	4	16
ENSG00000105383	3	0	0	0
ENSG00000105388	0	0	0	0
ENSG00000105392	1	0	0	0
ENSG00000105393	93	47	9	25
ENSG00000105397	23	0	12	22
ENSG00000105398	0	0	0	0
ENSG00000105401	167	817	595	1071

ENSG00000105402	612	1328	1555	1940
ENSG00000105404	317	972	663	642
ENSG00000105409	1	0	1	0
ENSG00000105419	0	0	0	2
ENSG00000105426	115	427	280	1063
ENSG00000105427	1	0	0	0
ENSG00000105428	0	0	0	0
ENSG00000105429	3	11	0	3
ENSG00000105438	119	215	109	191
ENSG00000105443	79	49	111	132
ENSG00000105447	5	0	0	1
ENSG00000105464	7	0	0	6
ENSG00000105467	0	0	0	0
ENSG00000105472	9	16	3	3
ENSG00000105479	1	0	0	0
ENSG00000105483	1089	3858	3623	3791
ENSG00000105486	31	34	19	67
ENSG00000105492	0	0	1	0
ENSG00000105497	120	402	457	322
ENSG00000105499	77	201	124	423
ENSG00000105501	0	0	0	0
ENSG00000105507	1091	2434	2332	2015
ENSG00000105509	0	0	0	0
ENSG00000105514	4	75	46	45
ENSG00000105516	2	0	0	9
ENSG00000105518	4	17	0	0
ENSG00000105519	0	0	0	0
ENSG00000105520	1	0	0	0
ENSG00000105523	0	0	0	0
ENSG00000105538	0	30	0	0
ENSG00000105549	0	0	0	0
ENSG00000105550	0	0	0	0
ENSG00000105552	4	0	0	1
ENSG00000105556	1	6	6	9
ENSG00000105559	0	8	0	0
ENSG00000105568	99	73	41	100
ENSG00000105576	80	242	141	196
ENSG00000105583	631	54	90	116
ENSG00000105605	0	0	0	0
ENSG00000105607	5	0	0	1
ENSG00000105609	1	0	0	0
ENSG00000105610	0	0	0	5
ENSG00000105612	6	11	6	28
ENSG00000105613	2	0	2	0
ENSG00000105617	0	0	1	0
ENSG00000105618	6	24	2	3

ENSG00000105619	3	15	7	2
ENSG00000105639	73	90	141	309
ENSG00000105640	1127	7	17	20
ENSG00000105641	1	0	0	0
ENSG00000105642	0	0	0	0
ENSG00000105643	15	0	1	0
ENSG00000105647	0	0	0	0
ENSG00000105649	0	0	0	0
ENSG00000105650	1	0	0	0
ENSG00000105655	2	0	0	0
ENSG00000105656	1	0	5	0
ENSG00000105662	1	32	9	19
ENSG00000105663	4	0	10	4
ENSG00000105664	0	0	0	0
ENSG00000105668	2	13	0	0
ENSG00000105669	27	115	76	84
ENSG00000105671	1	21	14	23
ENSG00000105672	2	0	0	0
ENSG00000105675	0	0	0	0
ENSG00000105676	5	23	20	7
ENSG00000105677	14	0	1	4
ENSG00000105679	0	0	0	0
ENSG00000105694	1	0	0	0
ENSG00000105695	0	0	3	4
ENSG00000105696	0	0	0	0
ENSG00000105697	0	0	0	0
ENSG00000105698	1127	3547	1485	3751
ENSG00000105699	20	88	38	68
ENSG00000105700	83	206	161	275
ENSG00000105701	130	4577	2261	4698
ENSG00000105705	20	7	19	51
ENSG00000105707	6	9	6	14
ENSG00000105708	1	2	8	28
ENSG00000105711	296	912	674	933
ENSG00000105717	35	233	117	237
ENSG00000105722	0	8	5	3
ENSG00000105723	32	28	31	41
ENSG00000105726	2	0	14	5
ENSG00000105732	4	0	1	0
ENSG00000105737	1	35	33	51
ENSG00000105738	3	86	38	73
ENSG00000105750	3	18	44	63
ENSG00000105755	18	0	3	3
ENSG00000105767	0	0	0	0
ENSG00000105771	10	18	27	45
ENSG00000105778	8	341	281	536

ENSG00000105784	0	0	5	9
ENSG00000105792	1	0	0	4
ENSG00000105793	4	241	219	420
ENSG00000105808	2	0	0	0
ENSG00000105810	190	854	1599	577
ENSG00000105819	31	437	281	612
ENSG00000105821	661	1361	1053	1629
ENSG00000105825	16	221	144	144
ENSG00000105829	728	4948	4784	5496
ENSG00000105835	47	373	447	388
ENSG00000105849	30	11	24	23
ENSG00000105851	143	2155	2214	2236
ENSG00000105852	0	0	0	0
ENSG00000105854	13	33	62	109
ENSG00000105855	2	1	12	27
ENSG00000105856	94	3054	3500	1924
ENSG00000105865	1	0	11	30
ENSG00000105866	10	68	76	72
ENSG00000105875	7	17	19	16
ENSG00000105877	2	0	5	10
ENSG00000105879	12	224	167	174
ENSG00000105880	0	0	0	0
ENSG00000105887	13518	89737	131813	118974
ENSG00000105889	3	90	302	133
ENSG00000105894	1	0	1	13
ENSG00000105926	20	2747	2945	3480
ENSG00000105928	1	0	0	0
ENSG00000105929	0	0	1	0
ENSG00000105939	112	1270	1286	1561
ENSG00000105948	0	17	50	65
ENSG00000105953	19	331	244	511
ENSG00000105954	0	0	0	0
ENSG00000105963	2	0	0	0
ENSG00000105967	5	156	264	646
ENSG00000105968	184	1055	629	747
ENSG00000105971	301	965	1587	1323
ENSG00000105974	63	113	173	119
ENSG00000105976	0	0	0	0
ENSG00000105982	1	39	37	13
ENSG00000105983	13	2949	2649	3383
ENSG00000105988	0	0	0	0
ENSG00000105989	0	0	0	0
ENSG00000105991	0	0	0	6
ENSG00000105993	4535	9056	9954	7959
ENSG00000105996	0	0	1	0
ENSG00000105997	0	0	0	0

ENSG00000106003	2	0	0	0
ENSG00000106004	0	0	0	0
ENSG00000106006	0	0	0	0
ENSG00000106009	1	0	1	0
ENSG00000106012	25	42	47	152
ENSG00000106013	0	29	24	26
ENSG00000106018	3	58	24	17
ENSG00000106025	1	0	5	6
ENSG00000106028	70	7	12	7
ENSG00000106031	0	0	0	0
ENSG00000106034	49	633	683	670
ENSG00000106038	0	0	0	0
ENSG00000106049	1	269	429	450
ENSG00000106052	889	4306	5774	4983
ENSG00000106066	14	11	11	36
ENSG00000106069	8	0	0	1
ENSG00000106070	42	1008	954	2040
ENSG00000106077	42	89	27	42
ENSG00000106078	0	0	0	0
ENSG00000106080	9	14	23	88
ENSG00000106086	36	140	266	267
ENSG00000106089	143	309	216	291
ENSG00000106100	13	70	60	69
ENSG00000106105	43	327	388	527
ENSG00000106113	0	13	0	0
ENSG00000106123	1	0	0	0
ENSG00000106125	0	0	0	0
ENSG00000106128	0	0	0	0
ENSG00000106133	0	0	0	0
ENSG00000106144	177	831	680	1016
ENSG00000106153	537	525	248	499
ENSG00000106178	0	0	0	0
ENSG00000106211	847	361	177	201
ENSG00000106236	0	0	0	0
ENSG00000106244	286	833	887	1562
ENSG00000106245	158	637	287	492
ENSG00000106246	0	0	5	1
ENSG00000106258	1	0	1	4
ENSG00000106261	63	527	338	718
ENSG00000106263	41	161	218	315
ENSG00000106266	21	32	16	17
ENSG00000106268	67	22	7	20
ENSG00000106278	0	0	0	0
ENSG00000106290	0	18	10	41
ENSG00000106299	8	74	156	196
ENSG00000106302	0	0	0	0

ENSG00000106304	0	0	0	0
ENSG00000106305	4	57	101	66
ENSG00000106327	0	10	0	2
ENSG00000106328	0	0	0	0
ENSG00000106330	21	0	2	14
ENSG00000106331	0	0	0	0
ENSG00000106333	3	0	0	0
ENSG00000106336	0	0	0	3
ENSG00000106341	4	0	0	0
ENSG00000106344	26	51	88	91
ENSG00000106346	10	282	226	413
ENSG00000106348	12	13	10	22
ENSG00000106351	4	13	2	2
ENSG00000106355	87	25	31	60
ENSG00000106366	224	777	838	1088
ENSG00000106367	18	21	45	69
ENSG00000106384	0	0	0	0
ENSG00000106392	225	2961	3771	2211
ENSG00000106397	6	0	3	2
ENSG00000106399	25	10	4	9
ENSG00000106400	107	162	27	128
ENSG00000106404	5	45	12	12
ENSG00000106410	0	0	0	0
ENSG00000106415	58	933	876	903
ENSG00000106436	0	0	0	0
ENSG00000106443	177	1896	1806	2897
ENSG00000106459	15	393	200	238
ENSG00000106460	4	375	629	989
ENSG00000106462	48	1708	2018	3141
ENSG00000106477	161	252	237	286
ENSG00000106479	5	0	1	0
ENSG00000106483	0	0	0	0
ENSG00000106484	329	1476	1241	1318
ENSG00000106511	1	28	1	4
ENSG00000106524	17	471	362	387
ENSG00000106526	0	67	85	122
ENSG00000106536	2	0	2	6
ENSG00000106537	37	1036	1046	1385
ENSG00000106538	0	0	0	0
ENSG00000106540	0	0	0	0
ENSG00000106541	0	0	0	0
ENSG00000106546	25	342	691	249
ENSG00000106554	17	115	121	224
ENSG00000106560	24	0	4	8
ENSG00000106565	8	0	0	0
ENSG00000106571	3	0	0	0

ENSG00000106588	34	153	78	157
ENSG00000106591	14	9	7	4
ENSG00000106603	62	23	84	73
ENSG00000106605	38	0	5	1
ENSG00000106608	3	30	52	91
ENSG00000106609	201	1746	2106	1846
ENSG00000106610	13	55	47	112
ENSG00000106615	249	8755	6253	9212
ENSG00000106617	26	351	352	368
ENSG00000106624	1	0	0	1
ENSG00000106628	14	20	1	2
ENSG00000106631	0	0	0	0
ENSG00000106633	0	0	0	0
ENSG00000106635	161	422	156	399
ENSG00000106636	44	56	25	50
ENSG00000106638	0	0	2	4
ENSG00000106648	1	0	0	0
ENSG00000106665	235	2117	536	1524
ENSG00000106682	70	347	323	326
ENSG00000106683	27	99	56	159
ENSG00000106686	3	0	5	18
ENSG00000106688	0	0	9	3
ENSG00000106689	0	0	0	0
ENSG00000106692	3	13	21	60
ENSG00000106701	5	478	550	495
ENSG00000106714	0	0	0	0
ENSG00000106723	24	386	461	435
ENSG00000106733	219	543	443	603
ENSG00000106771	64	2013	1708	2941
ENSG00000106772	17	294	384	328
ENSG00000106780	37	19	177	284
ENSG00000106785	38	0	8	11
ENSG00000106789	4	0	0	3
ENSG00000106799	1	18	5	1
ENSG00000106803	85	18	28	49
ENSG00000106804	4	503	274	602
ENSG00000106809	1	0	13	30
ENSG00000106819	0	0	0	0
ENSG00000106823	0	0	1	2
ENSG00000106829	75	548	502	632
ENSG00000106852	0	0	0	0
ENSG00000106853	2	53	24	38
ENSG00000106868	720	22028	18003	27903
ENSG00000106927	0	0	1	1
ENSG00000106948	19	0	0	8
ENSG00000106952	0	0	0	0

ENSG00000106976	60	286	138	428
ENSG00000106991	5	16	8	17
ENSG00000106992	2	0	1	0
ENSG00000106993	6	123	49	55
ENSG00000107014	2	1	5	4
ENSG00000107018	4	0	12	29
ENSG00000107020	18	0	6	12
ENSG00000107021	219	405	275	378
ENSG00000107036	13	290	440	461
ENSG00000107077	15	556	668	1400
ENSG00000107099	54	500	369	692
ENSG00000107104	5	37	15	43
ENSG00000107105	0	0	0	0
ENSG00000107130	0	0	0	0
ENSG00000107140	1	0	9	11
ENSG00000107147	0	0	0	0
ENSG00000107159	0	0	0	0
ENSG00000107164	14	26	46	85
ENSG00000107165	0	0	0	0
ENSG00000107175	21	51	25	20
ENSG00000107185	8	21	93	86
ENSG00000107186	1	26	12	35
ENSG00000107187	0	0	0	0
ENSG00000107201	15	33	43	59
ENSG00000107223	412	72	70	219
ENSG00000107242	138	364	417	812
ENSG00000107249	0	0	1	2
ENSG00000107262	220	321	269	325
ENSG00000107263	16	0	0	0
ENSG00000107281	0	0	0	1
ENSG00000107282	0	0	0	0
ENSG00000107290	273	3850	4236	3547
ENSG00000107295	0	0	2	5
ENSG00000107317	10	0	0	0
ENSG00000107331	0	0	0	0
ENSG00000107338	0	0	0	0
ENSG00000107341	553	1025	699	818
ENSG00000107362	18	749	510	847
ENSG00000107371	7	11	8	34
ENSG00000107372	156	748	519	292
ENSG00000107404	0	0	5	0
ENSG00000107438	7017	12924	8986	10018
ENSG00000107443	1	41	22	42
ENSG00000107447	0	0	0	6
ENSG00000107485	63	50	72	10
ENSG00000107518	0	0	1	0

ENSG00000107521	394	337	165	188
ENSG00000107537	12	248	229	464
ENSG00000107551	26	0	6	7
ENSG00000107554	5	100	112	149
ENSG00000107560	9	72	199	142
ENSG00000107562	1	0	3	1
ENSG00000107566	16	148	66	156
ENSG00000107581	210	560	426	587
ENSG00000107593	0	0	0	0
ENSG00000107611	1	0	19	14
ENSG00000107614	13	235	194	184
ENSG00000107618	0	0	0	0
ENSG00000107623	0	0	0	0
ENSG00000107625	27	520	452	493
ENSG00000107643	13	675	748	819
ENSG00000107651	19	399	348	423
ENSG00000107669	556	8212	9940	12646
ENSG00000107672	7	159	62	75
ENSG00000107679	38	191	82	214
ENSG00000107719	2	0	1	0
ENSG00000107731	0	0	0	0
ENSG00000107736	9	27	6	5
ENSG00000107738	597	768	559	602
ENSG00000107742	3	0	0	1
ENSG00000107745	1271	4556	5339	5564
ENSG00000107758	57	1563	1533	2053
ENSG00000107771	193	1662	1646	1965
ENSG00000107779	0	0	0	10
ENSG00000107789	11	1504	1933	1517
ENSG00000107796	1	39	48	82
ENSG00000107798	520	9873	5710	10873
ENSG00000107807	0	0	0	0
ENSG00000107815	0	0	0	0
ENSG00000107816	73	67	69	20
ENSG00000107819	140	493	714	438
ENSG00000107821	0	0	0	0
ENSG00000107829	19	35	45	81
ENSG00000107831	0	0	0	0
ENSG00000107833	5	0	1	0
ENSG00000107854	46	1022	1051	1357
ENSG00000107859	0	0	0	0
ENSG00000107862	43	219	215	357
ENSG00000107863	2328	19042	27067	20016
ENSG00000107864	1	115	86	80
ENSG00000107872	12	4	2	6
ENSG00000107874	12	18	4	10

ENSG00000107882	10	127	22	34
ENSG00000107890	13	35	21	37
ENSG00000107897	7	38	44	41
ENSG00000107902	15	0	1	4
ENSG00000107929	39	479	388	699
ENSG00000107937	22	200	79	91
ENSG00000107938	1	138	98	126
ENSG00000107949	31	53	73	92
ENSG00000107951	3	87	186	237
ENSG00000107954	6	0	0	0
ENSG00000107957	57	368	168	188
ENSG00000107959	36	394	461	424
ENSG00000107960	15	122	114	112
ENSG00000107968	128	723	878	492
ENSG00000107984	0	12	6	20
ENSG00000108001	1	0	3	0
ENSG00000108010	571	2599	1300	3471
ENSG00000108018	0	0	0	0
ENSG00000108021	112	2594	3172	3495
ENSG00000108039	856	6169	5455	3959
ENSG00000108055	187	5351	7559	7241
ENSG00000108061	1330	19695	28238	25241
ENSG00000108064	45	49	76	117
ENSG00000108091	36	511	664	794
ENSG00000108094	12	2686	2418	4100
ENSG00000108100	1305	5684	5263	7574
ENSG00000108106	1	0	1	1
ENSG00000108107	837	230	56	144
ENSG00000108175	38	99	59	64
ENSG00000108176	0	0	0	0
ENSG00000108179	1279	1077	854	1118
ENSG00000108187	145	432	538	793
ENSG00000108219	105	307	212	374
ENSG00000108231	0	0	0	0
ENSG00000108239	0	23	11	35
ENSG00000108242	0	0	0	0
ENSG00000108244	1	28	1	0
ENSG00000108255	0	0	0	0
ENSG00000108256	26	319	332	341
ENSG00000108262	7	3	16	23
ENSG00000108264	60	2008	1709	2745
ENSG00000108270	36	84	59	121
ENSG00000108272	5	11	0	8
ENSG00000108278	26	39	23	36
ENSG00000108292	66	0	3	8
ENSG00000108294	195	32	27	54

ENSG00000108296	29	27	8	16
ENSG00000108298	1487	379	232	577
ENSG00000108306	3	492	437	604
ENSG00000108309	1	0	0	1
ENSG00000108312	34	84	46	73
ENSG00000108342	0	0	0	0
ENSG00000108344	3	30	17	21
ENSG00000108349	30	134	185	202
ENSG00000108352	0	0	1	0
ENSG00000108370	146	292	571	332
ENSG00000108375	6	113	108	45
ENSG00000108379	1	0	0	0
ENSG00000108381	0	0	0	0
ENSG00000108384	25	830	434	616
ENSG00000108387	353	1537	291	1892
ENSG00000108389	6	26	24	34
ENSG00000108395	9	1176	1118	2101
ENSG00000108405	1401	1439	1271	1816
ENSG00000108406	24	238	240	334
ENSG00000108417	0	0	0	0
ENSG00000108423	3	176	91	164
ENSG00000108424	248	863	551	960
ENSG00000108433	35	95	34	88
ENSG00000108439	2	0	3	12
ENSG00000108442	0	0	0	0
ENSG00000108443	35	867	856	1178
ENSG00000108448	40	172	225	127
ENSG00000108452	0	0	0	0
ENSG00000108465	33	0	4	7
ENSG00000108468	24	0	2	16
ENSG00000108469	35	159	20	137
ENSG00000108474	2	0	10	10
ENSG00000108479	0	0	0	0
ENSG00000108506	1	440	727	1323
ENSG00000108509	12	336	124	241
ENSG00000108510	167	5421	6167	6816
ENSG00000108511	0	0	0	0
ENSG00000108515	0	99	50	29
ENSG00000108516	1	0	2	0
ENSG00000108518	3073	2570	1199	2539
ENSG00000108523	335	243	277	306
ENSG00000108528	42	29	21	47
ENSG00000108551	3	0	0	0
ENSG00000108556	1	13	14	17
ENSG00000108557	0	0	2	0
ENSG00000108559	23	231	229	310

ENSG00000108561	12	20	4	9
ENSG00000108576	378	1473	1325	1519
ENSG00000108578	13	58	49	175
ENSG00000108582	9	140	244	365
ENSG00000108587	47	3579	3040	5605
ENSG00000108588	15	763	1086	1408
ENSG00000108590	27	132	368	189
ENSG00000108591	18	0	5	11
ENSG00000108592	14	0	3	17
ENSG00000108599	27	1139	1361	1829
ENSG00000108602	0	0	0	0
ENSG00000108604	77	114	50	87
ENSG00000108622	588	1197	1189	638
ENSG00000108639	8	37	1	3
ENSG00000108641	0	0	0	0
ENSG00000108651	9	344	318	661
ENSG00000108654	289	1600	1207	1624
ENSG00000108666	9	0	15	6
ENSG00000108669	36	0	4	22
ENSG00000108671	119	301	247	225
ENSG00000108679	17	42	27	32
ENSG00000108684	0	0	0	0
ENSG00000108688	0	0	0	0
ENSG00000108691	3	0	1	0
ENSG00000108700	0	0	0	0
ENSG00000108702	0	0	0	0
ENSG00000108733	1	4	7	4
ENSG00000108753	0	0	0	0
ENSG00000108759	0	0	0	0
ENSG00000108771	6	0	1	2
ENSG00000108773	4	0	0	6
ENSG00000108774	154	212	208	150
ENSG00000108784	1	0	0	2
ENSG00000108785	1	0	0	0
ENSG00000108786	3	0	0	2
ENSG00000108788	20	54	24	64
ENSG00000108797	0	0	0	0
ENSG00000108798	57	0	2	0
ENSG00000108799	22	778	734	1179
ENSG00000108813	0	0	0	0
ENSG00000108819	10	80	60	137
ENSG00000108821	0	0	0	0
ENSG00000108823	0	0	0	0
ENSG00000108825	0	0	0	0
ENSG00000108826	45	32	43	49
ENSG00000108828	23	23	25	31

ENSG00000108829	35	387	265	574
ENSG00000108830	0	0	0	0
ENSG00000108839	2119	5122	2514	5163
ENSG00000108840	62	1283	721	753
ENSG00000108846	2487	11027	10039	11738
ENSG00000108848	230	2038	2030	3196
ENSG00000108849	0	0	0	0
ENSG00000108852	0	0	0	0
ENSG00000108854	5	96	173	201
ENSG00000108861	235	620	863	1334
ENSG00000108878	0	0	0	0
ENSG00000108883	255	1295	1529	2163
ENSG00000108924	1	0	0	0
ENSG00000108932	2	0	2	0
ENSG00000108946	1419	27331	45022	28951
ENSG00000108947	0	0	0	0
ENSG00000108950	0	0	0	0
ENSG00000108953	4087	32202	22546	32152
ENSG00000108958	0	0	0	0
ENSG00000108960	17393	69960	86821	62661
ENSG00000108961	0	0	0	4
ENSG00000108963	1	0	0	0
ENSG00000108984	5	36	26	6
ENSG00000109016	58	103	88	83
ENSG00000109046	109	616	518	594
ENSG00000109047	0	0	0	0
ENSG00000109061	0	0	0	0
ENSG00000109062	738	543	317	259
ENSG00000109063	0	0	0	8
ENSG00000109065	8	50	9	12
ENSG00000109066	63	285	151	175
ENSG00000109072	0	0	0	0
ENSG00000109079	8	26	29	65
ENSG00000109083	10	110	22	26
ENSG00000109084	8	0	0	0
ENSG00000109089	27	189	36	115
ENSG00000109099	1	0	0	0
ENSG00000109101	0	0	0	0
ENSG00000109103	55	11	19	17
ENSG00000109107	18	11	31	22
ENSG00000109111	55	222	122	275
ENSG00000109113	19	47	9	51
ENSG00000109118	44	76	70	86
ENSG00000109132	0	0	0	0
ENSG00000109133	13	366	499	521
ENSG00000109158	0	0	0	0

ENSG00000109163	0	0	2	1
ENSG00000109171	345	11830	13806	13870
ENSG00000109180	172	1508	1464	1923
ENSG00000109181	0	0	0	0
ENSG00000109182	0	0	4	0
ENSG00000109184	14	5701	5140	8475
ENSG00000109189	3	50	37	71
ENSG00000109193	0	0	0	0
ENSG00000109205	0	0	0	0
ENSG00000109208	0	0	0	0
ENSG00000109220	25	225	157	118
ENSG00000109255	0	0	0	0
ENSG00000109265	11	517	255	358
ENSG00000109270	79	448	439	536
ENSG00000109272	3943	42497	53355	230633
ENSG00000109320	32	206	168	461
ENSG00000109321	0	0	0	0
ENSG00000109323	93	2960	3221	3887
ENSG00000109332	501	4630	4733	4892
ENSG00000109339	20	788	574	613
ENSG00000109381	59	8360	6095	9655
ENSG00000109390	662	478	202	348
ENSG00000109424	0	0	0	0
ENSG00000109436	9	68	64	56
ENSG00000109445	5	23	10	19
ENSG00000109452	43	4540	5370	8281
ENSG00000109458	6	966	853	457
ENSG00000109466	14	319	323	365
ENSG00000109471	0	0	0	0
ENSG00000109472	0	0	0	0
ENSG00000109475	1978	364	214	417
ENSG00000109501	0	0	0	0
ENSG00000109511	0	0	0	0
ENSG00000109519	38	3	23	37
ENSG00000109534	3	0	0	0
ENSG00000109536	66	138	103	184
ENSG00000109572	1014	34573	38620	58880
ENSG00000109576	0	0	0	8
ENSG00000109586	10	109	130	73
ENSG00000109606	30	196	182	262
ENSG00000109610	0	0	0	0
ENSG00000109618	12	538	689	658
ENSG00000109625	0	0	0	0
ENSG00000109654	2	13	2	29
ENSG00000109667	2	0	8	19
ENSG00000109670	40	12168	8809	16568

ENSG00000109674	1	3016	2411	3731
ENSG00000109680	4	119	144	311
ENSG00000109684	1	0	0	0
ENSG00000109685	31	329	446	550
ENSG00000109686	0	25	4	0
ENSG00000109689	67	333	412	705
ENSG00000109705	0	0	0	0
ENSG00000109736	1	16	8	4
ENSG00000109738	0	0	12	11
ENSG00000109743	4	0	3	5
ENSG00000109756	48	1496	1546	2181
ENSG00000109758	0	0	0	0
ENSG00000109762	0	94	72	79
ENSG00000109771	0	0	0	0
ENSG00000109775	11	163	88	124
ENSG00000109787	824	2787	3870	2426
ENSG00000109790	367	323	7699	7064
ENSG00000109794	0	0	0	0
ENSG00000109805	6	508	730	920
ENSG00000109814	4	43	4	7
ENSG00000109819	0	0	0	0
ENSG00000109832	0	0	0	0
ENSG00000109846	0	0	0	0
ENSG00000109851	0	0	0	0
ENSG00000109854	573	1946	441	300
ENSG00000109861	90	667	655	557
ENSG00000109881	18	12	6	7
ENSG00000109906	105	43	66	103
ENSG00000109911	5	59	30	41
ENSG00000109917	13	0	9	3
ENSG00000109919	65	581	208	401
ENSG00000109920	34	1719	896	1563
ENSG00000109927	20	17	30	45
ENSG00000109929	14	507	798	736
ENSG00000109943	11	7	6	0
ENSG00000109944	2	33	28	35
ENSG00000109956	1	0	0	0
ENSG00000109971	687	578	586	943
ENSG00000109991	0	0	0	0
ENSG00000110002	53	575	752	2791
ENSG00000110011	97	461	190	409
ENSG00000110013	588	4421	4754	7654
ENSG00000110025	9	0	11	11
ENSG00000110031	36	79	22	37
ENSG00000110042	1	0	0	0
ENSG00000110046	0	0	0	4

ENSG00000110047	467	2150	1174	1902
ENSG00000110048	24	232	185	308
ENSG00000110057	0	0	0	0
ENSG00000110060	9	23	12	4
ENSG00000110063	17	0	1	4
ENSG00000110066	34	1803	1326	2020
ENSG00000110074	7	0	0	16
ENSG00000110075	87	7993	7506	8676
ENSG00000110076	0	0	1	0
ENSG00000110077	22	30	7	0
ENSG00000110079	0	0	2	0
ENSG00000110080	4	443	151	283
ENSG00000110090	177	1364	1727	1979
ENSG00000110092	4	174	49	82
ENSG00000110104	1	9	3	0
ENSG00000110107	35	103	70	109
ENSG00000110108	89	63	51	75
ENSG00000110148	0	0	0	1
ENSG00000110169	0	0	0	0
ENSG00000110171	1	0	1	0
ENSG00000110172	18	150	191	277
ENSG00000110195	1	0	9	1
ENSG00000110200	42	191	79	109
ENSG00000110203	2	0	0	0
ENSG00000110218	345	5200	5462	3863
ENSG00000110237	0	0	0	0
ENSG00000110243	0	0	0	0
ENSG00000110244	0	0	0	0
ENSG00000110245	0	0	0	0
ENSG00000110274	4	0	10	9
ENSG00000110315	68	459	270	242
ENSG00000110318	4	1	16	47
ENSG00000110321	4168	30914	44188	38408
ENSG00000110324	21	7	13	30
ENSG00000110328	0	0	0	0
ENSG00000110330	210	2706	4385	3517
ENSG00000110344	4	164	94	103
ENSG00000110347	0	0	0	0
ENSG00000110367	134	3689	4004	4267
ENSG00000110375	0	0	0	0
ENSG00000110395	332	1899	2049	1563
ENSG00000110400	3	0	4	19
ENSG00000110422	340	27078	22298	27432
ENSG00000110427	0	0	0	0
ENSG00000110429	5	41	141	99
ENSG00000110435	2	1123	743	1259

ENSG00000110436	0	0	0	0
ENSG00000110442	27	14	16	15
ENSG00000110446	4	0	0	1
ENSG00000110448	0	0	0	0
ENSG00000110455	121	291	152	79
ENSG00000110484	0	0	0	0
ENSG00000110492	25	36	23	20
ENSG00000110497	1	101	80	146
ENSG00000110514	559	3130	1665	2148
ENSG00000110536	2	0	0	5
ENSG00000110583	13	5	4	19
ENSG00000110619	25	31	30	28
ENSG00000110628	1	0	0	0
ENSG00000110651	10	0	0	0
ENSG00000110660	0	39	14	7
ENSG00000110665	478	884	503	852
ENSG00000110675	0	0	0	0
ENSG00000110680	0	0	0	0
ENSG00000110693	2	18	5	36
ENSG00000110696	1551	6437	5919	6376
ENSG00000110697	2	26	10	9
ENSG00000110700	1259	78	76	113
ENSG00000110711	35	59	11	17
ENSG00000110713	24	1215	1115	2009
ENSG00000110717	83	0	1	8
ENSG00000110719	1	0	2	6
ENSG00000110721	1	151	161	222
ENSG00000110723	1	0	1	6
ENSG00000110756	24	934	616	1438
ENSG00000110768	34	407	533	817
ENSG00000110777	6	0	0	0
ENSG00000110786	0	0	1	0
ENSG00000110799	689	1616	842	1993
ENSG00000110801	7	0	8	2
ENSG00000110811	0	0	0	0
ENSG00000110841	16	196	97	220
ENSG00000110844	0	0	0	0
ENSG00000110848	92	72	666	503
ENSG00000110851	64	128	196	198
ENSG00000110852	28	0	12	5
ENSG00000110871	17	49	65	96
ENSG00000110876	28	20	5	10
ENSG00000110880	1258	32906	38810	36117
ENSG00000110881	0	0	0	0
ENSG00000110887	2	0	0	0
ENSG00000110888	1	106	103	146

ENSG00000110900	0	0	0	0
ENSG00000110906	208	1335	868	1133
ENSG00000110911	3	41	82	88
ENSG00000110917	188	514	615	1619
ENSG00000110921	0	0	0	0
ENSG00000110925	5	98	65	39
ENSG00000110931	20	16	21	55
ENSG00000110934	4551	20916	16440	24859
ENSG00000110944	0	0	0	0
ENSG00000110955	274	607	274	539
ENSG00000110958	344	2565	3390	4652
ENSG00000110975	0	0	0	0
ENSG00000110987	2	19	16	49
ENSG00000111011	228	483	910	1025
ENSG00000111012	0	0	0	0
ENSG00000111046	0	0	0	0
ENSG00000111049	0	0	0	0
ENSG00000111052	1	0	1	1
ENSG00000111057	40	60	49	41
ENSG00000111058	0	0	0	4
ENSG00000111077	0	0	0	0
ENSG00000111087	1	0	0	0
ENSG00000111110	4	0	59	34
ENSG00000111142	85	864	753	846
ENSG00000111144	86	201	137	83
ENSG00000111145	903	7337	7854	7260
ENSG00000111181	0	0	0	0
ENSG00000111186	4	0	11	12
ENSG00000111196	421	1410	2254	2228
ENSG00000111199	0	0	0	0
ENSG00000111203	8	11	12	18
ENSG00000111206	0	49	6	27
ENSG00000111215	17	259	414	997
ENSG00000111218	1	0	0	0
ENSG00000111224	11	257	247	324
ENSG00000111229	825	3412	2409	4101
ENSG00000111231	65	281	189	680
ENSG00000111237	209	558	645	846
ENSG00000111241	0	0	0	0
ENSG00000111245	0	0	0	0
ENSG00000111247	20	60	42	54
ENSG00000111249	1	0	0	0
ENSG00000111252	370	2195	2317	2575
ENSG00000111254	3	9	12	27
ENSG00000111261	0	0	1	19
ENSG00000111262	0	0	0	0

ENSG00000111266	4	41	67	190
ENSG00000111269	92	324	687	1086
ENSG00000111271	2	12	6	15
ENSG00000111275	20	21	16	11
ENSG00000111276	23	45	54	31
ENSG00000111291	0	0	0	4
ENSG00000111300	8	1000	559	944
ENSG00000111305	0	0	0	5
ENSG00000111319	1	0	1	0
ENSG00000111321	2	0	1	0
ENSG00000111325	0	0	0	2
ENSG00000111328	303	4411	6389	5598
ENSG00000111331	8	0	3	0
ENSG00000111335	29	0	5	0
ENSG00000111339	0	0	0	2
ENSG00000111341	3	21	0	0
ENSG00000111344	0	0	0	0
ENSG00000111348	15291	25311	28147	34228
ENSG00000111358	21	675	421	508
ENSG00000111361	22	72	59	151
ENSG00000111364	11	17	12	5
ENSG00000111371	17	584	628	799
ENSG00000111404	1	0	1	0
ENSG00000111405	0	0	0	2
ENSG00000111412	10	0	8	9
ENSG00000111424	463	754	373	609
ENSG00000111432	0	0	0	0
ENSG00000111445	5	37	52	105
ENSG00000111450	3	77	72	97
ENSG00000111452	1	0	0	0
ENSG00000111481	550	1741	1448	1504
ENSG00000111490	0	0	2	0
ENSG00000111530	29	341	311	395
ENSG00000111536	0	0	0	0
ENSG00000111537	1	0	0	0
ENSG00000111540	10	1101	871	1094
ENSG00000111554	382	6530	6034	13454
ENSG00000111581	18	2183	1922	2498
ENSG00000111596	67	930	792	768
ENSG00000111602	0	12	5	12
ENSG00000111605	42	618	505	633
ENSG00000111615	31	257	398	280
ENSG00000111639	237	108	109	85
ENSG00000111640	4062	17537	8421	21373
ENSG00000111641	8	0	5	2
ENSG00000111642	390	858	639	596

ENSG00000111644	30356	8455	4797	6811
ENSG00000111647	21	344	430	303
ENSG00000111652	22	13	32	92
ENSG00000111653	201	702	754	518
ENSG00000111664	0	0	0	0
ENSG00000111665	0	0	0	0
ENSG00000111666	5	425	232	246
ENSG00000111667	10	0	4	7
ENSG00000111669	1677	1319	1036	1952
ENSG00000111670	74	647	692	743
ENSG00000111671	9	0	1	6
ENSG00000111674	422	937	970	1170
ENSG00000111676	20	98	42	86
ENSG00000111678	1892	1069	338	637
ENSG00000111679	351	256	136	241
ENSG00000111684	13	203	104	156
ENSG00000111696	7	0	30	71
ENSG00000111700	0	0	0	0
ENSG00000111701	0	0	0	0
ENSG00000111704	1	0	0	0
ENSG00000111707	73	296	363	442
ENSG00000111711	388	1770	2624	2224
ENSG00000111713	9	11	19	94
ENSG00000111716	521	1768	1499	3330
ENSG00000111725	14	100	33	62
ENSG00000111726	105	1215	860	865
ENSG00000111727	6	119	80	93
ENSG00000111728	0	0	0	2
ENSG00000111729	8	0	3	3
ENSG00000111731	20	3496	2699	3482
ENSG00000111732	2	0	0	0
ENSG00000111737	14	69	54	33
ENSG00000111752	12	25	221	141
ENSG00000111775	856	99	51	177
ENSG00000111780	0	0	0	0
ENSG00000111783	0	18	0	0
ENSG00000111785	1	2385	2052	3552
ENSG00000111786	77	107	143	89
ENSG00000111788	1	0	0	0
ENSG00000111790	417	7603	8353	5949
ENSG00000111796	187	19	20	16
ENSG00000111799	0	0	3	0
ENSG00000111801	8	44	18	16
ENSG00000111802	131	700	872	547
ENSG00000111816	0	0	0	0
ENSG00000111817	1130	7211	8243	5031

ENSG00000111832	303	585	333	1046
ENSG00000111834	0	0	1	1
ENSG00000111837	0	14	0	1
ENSG00000111843	97	155	79	131
ENSG00000111845	12	466	367	825
ENSG00000111846	0	0	1	6
ENSG00000111850	8	32	23	44
ENSG00000111859	28	85	10	6
ENSG00000111860	34	344	474	484
ENSG00000111863	3	0	0	0
ENSG00000111875	64	772	722	758
ENSG00000111877	7	56	64	112
ENSG00000111879	0	0	1	3
ENSG00000111880	13	327	502	712
ENSG00000111885	405	3731	6443	4429
ENSG00000111886	3	0	2	6
ENSG00000111897	105	1796	2041	1517
ENSG00000111906	21	82	50	32
ENSG00000111907	0	0	0	0
ENSG00000111911	54	192	195	251
ENSG00000111912	272	1795	2805	1781
ENSG00000111913	732	3154	1913	1416
ENSG00000111961	8	0	5	1
ENSG00000111962	14	0	26	112
ENSG00000111971	0	0	0	0
ENSG00000111981	2	0	0	0
ENSG00000112029	5	12	17	10
ENSG00000112031	606	1686	1466	5182
ENSG00000112033	1	0	2	1
ENSG00000112038	0	12	27	12
ENSG00000112039	4	0	1	1
ENSG00000112041	0	0	0	0
ENSG00000112053	2	5	15	21
ENSG00000112062	310	1729	1392	1260
ENSG00000112077	4	33	24	18
ENSG00000112078	3382	13546	14493	12766
ENSG00000112079	207	3173	3230	5480
ENSG00000112081	925	3618	1977	2324
ENSG00000112096	7066	70122	67227	88430
ENSG00000112110	175	450	179	209
ENSG00000112115	0	0	0	0
ENSG00000112116	0	0	0	0
ENSG00000112118	24	5	14	28
ENSG00000112130	178	404	303	395
ENSG00000112137	398	58	143	213
ENSG00000112139	1	0	0	0

ENSG00000112144	3	0	8	16
ENSG00000112146	241	1259	1883	2481
ENSG00000112149	0	0	3	4
ENSG00000112159	19	731	533	885
ENSG00000112164	0	0	0	0
ENSG00000112167	12	1	23	68
ENSG00000112175	0	0	0	0
ENSG00000112182	40	41	78	12
ENSG00000112183	0	0	0	0
ENSG00000112186	0	0	0	0
ENSG00000112195	8	85	63	34
ENSG00000112200	133	2664	3217	2272
ENSG00000112208	9	57	94	117
ENSG00000112210	2	656	641	744
ENSG00000112212	1	0	0	1
ENSG00000112214	0	0	0	0
ENSG00000112218	0	0	0	4
ENSG00000112232	0	167	370	486
ENSG00000112234	48	3046	1096	2331
ENSG00000112237	14	259	321	489
ENSG00000112238	0	0	0	0
ENSG00000112242	413	1145	1061	1098
ENSG00000112245	309	5263	7443	5996
ENSG00000112246	0	0	0	0
ENSG00000112249	18	1603	1556	1455
ENSG00000112273	0	0	0	0
ENSG00000112276	0	0	0	1
ENSG00000112280	0	0	0	0
ENSG00000112282	3	187	73	116
ENSG00000112290	19	755	559	762
ENSG00000112293	0	0	4	23
ENSG00000112294	3	15	25	8
ENSG00000112297	19	23	5	1
ENSG00000112299	20	1281	819	499
ENSG00000112303	11	5	10	8
ENSG00000112304	67	186	266	336
ENSG00000112305	68	517	543	751
ENSG00000112306	2015	562	248	1222
ENSG00000112308	859	4716	7465	4730
ENSG00000112309	0	2	19	1
ENSG00000112312	2	106	62	168
ENSG00000112319	0	0	0	0
ENSG00000112320	1	22	7	6
ENSG00000112333	0	0	0	0
ENSG00000112335	1403	6513	5700	13381
ENSG00000112337	0	0	0	0

ENSG00000112339	19	301	361	671
ENSG00000112343	21	4	15	17
ENSG00000112357	1	58	51	74
ENSG00000112365	3	269	166	478
ENSG00000112367	14	604	513	598
ENSG00000112378	1	0	0	0
ENSG00000112379	1	0	0	0
ENSG00000112394	1	50	61	68
ENSG00000112406	123	799	1214	683
ENSG00000112414	1	0	15	1
ENSG00000112419	242	1168	1369	1149
ENSG00000112425	2	66	49	87
ENSG00000112459	0	0	0	0
ENSG00000112461	0	0	0	0
ENSG00000112462	0	0	0	0
ENSG00000112473	32	15	21	27
ENSG00000112474	0	0	0	0
ENSG00000112486	2	0	1	0
ENSG00000112493	0	0	0	0
ENSG00000112494	0	0	0	0
ENSG00000112499	0	0	1	0
ENSG00000112511	34	58	32	47
ENSG00000112514	266	24	34	63
ENSG00000112530	1	0	1	2
ENSG00000112531	512	7243	9619	12751
ENSG00000112539	0	0	0	0
ENSG00000112541	0	0	0	0
ENSG00000112559	0	0	0	0
ENSG00000112561	5	0	2	0
ENSG00000112562	0	0	2	0
ENSG00000112576	4400	6809	4491	6908
ENSG00000112578	3	0	0	0
ENSG00000112584	15	320	252	415
ENSG00000112592	4	22	10	32
ENSG00000112599	0	0	1	0
ENSG00000112619	0	0	0	0
ENSG00000112624	69	1937	1705	1284
ENSG00000112640	1	1	36	19
ENSG00000112651	20	5	21	58
ENSG00000112655	51	213	124	351
ENSG00000112658	66	47	74	115
ENSG00000112659	4	14	7	3
ENSG00000112664	266	658	611	1139
ENSG00000112667	5	0	1	0
ENSG00000112679	1483	5444	3153	4598
ENSG00000112685	14	588	374	758

ENSG00000112695	522	373	277	328
ENSG00000112697	42	660	709	921
ENSG00000112699	3	26	30	28
ENSG00000112701	86	11458	13864	17606
ENSG00000112706	0	0	0	0
ENSG00000112715	0	16	5	16
ENSG00000112739	74	290	303	527
ENSG00000112742	7	314	338	521
ENSG00000112759	2	0	14	13
ENSG00000112761	0	0	0	0
ENSG00000112763	38	95	108	68
ENSG00000112769	3	89	5	1
ENSG00000112773	7	4	7	18
ENSG00000112782	2	20	6	0
ENSG00000112787	54	167	72	142
ENSG00000112796	0	0	0	4
ENSG00000112799	40	0	9	0
ENSG00000112812	0	0	0	0
ENSG00000112818	0	0	0	1
ENSG00000112837	0	0	0	2
ENSG00000112851	1012	26044	34246	41458
ENSG00000112852	0	0	0	0
ENSG00000112855	0	5	6	8
ENSG00000112874	1	0	3	4
ENSG00000112877	1	5	1	2
ENSG00000112893	112	11649	14106	17685
ENSG00000112902	8	68	16	12
ENSG00000112936	0	27	0	2
ENSG00000112941	9	130	168	266
ENSG00000112964	5	20	79	120
ENSG00000112972	4	1432	1104	1538
ENSG00000112977	1009	2416	1967	4575
ENSG00000112981	0	0	0	0
ENSG00000112983	7	0	21	52
ENSG00000112984	0	18	8	11
ENSG00000112992	271	4251	4459	5273
ENSG00000112996	10	33	33	78
ENSG00000113013	57	357	409	522
ENSG00000113048	10	33	9	30
ENSG00000113068	64	185	77	207
ENSG00000113070	1	8	2	13
ENSG00000113073	0	0	0	0
ENSG00000113083	8	95	80	307
ENSG00000113088	2	0	0	0
ENSG00000113100	0	58	6	10
ENSG00000113108	1	0	0	4

ENSG00000113119	2	0	1	4
ENSG00000113140	40882	110799	123726	130569
ENSG00000113141	184	127	125	178
ENSG00000113161	2	101	77	163
ENSG00000113163	61	379	522	706
ENSG00000113194	12	34	105	94
ENSG00000113196	0	0	0	0
ENSG00000113205	0	0	0	0
ENSG00000113209	0	0	0	0
ENSG00000113211	0	0	0	0
ENSG00000113212	0	0	0	0
ENSG00000113231	4	0	7	7
ENSG00000113240	15	247	258	198
ENSG00000113248	0	0	0	0
ENSG00000113249	0	10	1	5
ENSG00000113262	0	0	0	0
ENSG00000113263	6	0	0	2
ENSG00000113269	404	862	712	1167
ENSG00000113272	15	9	16	25
ENSG00000113273	2	117	177	174
ENSG00000113282	403	2262	2900	3034
ENSG00000113296	0	0	1	0
ENSG00000113300	27	1128	1129	1524
ENSG00000113302	0	0	0	0
ENSG00000113303	0	0	0	0
ENSG00000113312	156	652	768	1004
ENSG00000113318	24	549	484	525
ENSG00000113319	12	172	730	847
ENSG00000113327	0	0	0	0
ENSG00000113328	638	8902	8934	6480
ENSG00000113356	890	2294	1338	2214
ENSG00000113360	6	1164	1191	2480
ENSG00000113361	5	39	190	28
ENSG00000113368	5	121	211	391
ENSG00000113369	103	2011	3836	2374
ENSG00000113384	82	327	340	268
ENSG00000113387	442	4990	2638	4275
ENSG00000113389	3	0	66	65
ENSG00000113391	68	1010	1160	1503
ENSG00000113396	0	14	1	0
ENSG00000113407	34	226	125	166
ENSG00000113430	0	0	0	0
ENSG00000113441	281	4264	6534	5065
ENSG00000113448	255	8757	11341	7925
ENSG00000113456	26	118	95	80
ENSG00000113460	16	245	173	265

ENSG00000113492	0	0	0	0
ENSG00000113494	0	0	0	0
ENSG00000113504	1	0	3	1
ENSG00000113520	0	0	0	0
ENSG00000113522	105	1620	1462	1401
ENSG00000113525	0	0	0	0
ENSG00000113532	15	1201	778	2037
ENSG00000113552	21	0	3	1
ENSG00000113555	0	0	0	0
ENSG00000113558	2981	15116	7881	20332
ENSG00000113569	9	653	490	594
ENSG00000113575	83	504	589	461
ENSG00000113578	0	0	1	0
ENSG00000113580	173	616	1002	606
ENSG00000113583	18	296	354	383
ENSG00000113593	13	91	157	508
ENSG00000113594	0	0	13	4
ENSG00000113595	32	348	359	312
ENSG00000113597	9	259	346	524
ENSG00000113600	0	0	2	19
ENSG00000113615	12	698	721	1175
ENSG00000113621	5	15	18	53
ENSG00000113638	304	10253	9595	10596
ENSG00000113643	43	5334	5185	8991
ENSG00000113645	135	51	111	525
ENSG00000113648	129	342	167	315
ENSG00000113649	37	182	189	275
ENSG00000113657	0	33	5	14
ENSG00000113658	865	478	3376	833
ENSG00000113712	123	1565	1517	2022
ENSG00000113716	1	10	8	4
ENSG00000113719	138	698	221	636
ENSG00000113721	0	0	1	0
ENSG00000113722	0	0	0	0
ENSG00000113732	5076	6140	7111	8870
ENSG00000113734	2	0	4	2
ENSG00000113739	0	0	0	0
ENSG00000113742	440	3142	5546	2386
ENSG00000113749	5	0	0	0
ENSG00000113758	116	654	274	437
ENSG00000113761	25	1360	710	932
ENSG00000113763	0	0	0	0
ENSG00000113790	0	9	12	9
ENSG00000113805	0	0	0	0
ENSG00000113810	252	2199	2854	3616
ENSG00000113811	111	108	181	379

ENSG00000113812	8	445	175	311
ENSG00000113838	0	27	15	5
ENSG00000113845	38	5346	3400	4954
ENSG00000113851	1090	8180	10392	9464
ENSG00000113889	0	0	0	0
ENSG00000113905	0	0	1	0
ENSG00000113916	10	38	10	0
ENSG00000113924	3632	5259	3425	6257
ENSG00000113946	4	70	75	73
ENSG00000113966	1	0	6	7
ENSG00000113971	11	932	888	1411
ENSG00000114013	10	0	1	0
ENSG00000114019	0	0	0	0
ENSG00000114021	17	11	31	74
ENSG00000114023	41	36	27	51
ENSG00000114026	10	211	144	176
ENSG00000114030	75	1005	1057	1017
ENSG00000114054	19	78	144	257
ENSG00000114062	54	4934	4564	6410
ENSG00000114098	466	7201	7099	8560
ENSG00000114107	6	9649	9693	17247
ENSG00000114113	0	0	0	0
ENSG00000114115	3	0	0	0
ENSG00000114120	17	254	252	345
ENSG00000114124	0	0	0	0
ENSG00000114125	87	158	144	242
ENSG00000114126	53	1126	1006	1538
ENSG00000114127	159	3901	4679	4552
ENSG00000114166	244	1398	3048	2174
ENSG00000114200	0	0	0	0
ENSG00000114204	0	0	2	0
ENSG00000114209	2622	14837	15839	13143
ENSG00000114248	0	0	0	0
ENSG00000114251	0	0	0	0
ENSG00000114268	2	59	34	47
ENSG00000114270	0	0	0	0
ENSG00000114279	2	2	2	0
ENSG00000114302	74	215	353	467
ENSG00000114315	9	11	1	0
ENSG00000114316	116	2485	4737	5499
ENSG00000114331	196	5252	4841	6909
ENSG00000114346	2	43	68	57
ENSG00000114349	0	0	0	0
ENSG00000114353	1830	2071	1245	2546
ENSG00000114354	69	809	819	890
ENSG00000114374	7	313	1	413

ENSG00000114378	0	0	0	0
ENSG00000114383	100	164	48	165
ENSG00000114388	12	0	9	4
ENSG00000114391	721	156	137	223
ENSG00000114395	8	0	1	3
ENSG00000114405	4	0	0	1
ENSG00000114416	83	1348	810	1345
ENSG00000114423	12	7	69	51
ENSG00000114439	323	2644	2151	2097
ENSG00000114446	30	72	123	124
ENSG00000114450	58	2387	1886	2686
ENSG00000114455	6	0	3	5
ENSG00000114473	10	115	92	68
ENSG00000114480	23	659	610	766
ENSG00000114487	92	10080	9418	11509
ENSG00000114491	5	12	65	74
ENSG00000114503	32	100	91	138
ENSG00000114520	22	1003	907	1268
ENSG00000114529	8	208	216	286
ENSG00000114541	1878	10236	14006	11607
ENSG00000114544	7	0	2	10
ENSG00000114547	0	0	0	0
ENSG00000114554	0	0	0	0
ENSG00000114573	222	2136	1367	1523
ENSG00000114626	224	537	405	267
ENSG00000114631	0	0	0	0
ENSG00000114638	0	0	0	0
ENSG00000114646	0	0	0	0
ENSG00000114648	47	185	38	125
ENSG00000114650	5	242	62	144
ENSG00000114654	3	2	3	0
ENSG00000114656	8	0	5	5
ENSG00000114670	14	347	253	726
ENSG00000114686	15	53	134	162
ENSG00000114698	25	229	607	282
ENSG00000114735	4	0	0	0
ENSG00000114737	5	0	3	0
ENSG00000114738	128	32	103	159
ENSG00000114739	4	0	4	4
ENSG00000114742	36	207	373	354
ENSG00000114744	30	1	17	21
ENSG00000114745	44	111	68	96
ENSG00000114757	0	0	0	0
ENSG00000114767	6	0	0	0
ENSG00000114770	187	274	540	359
ENSG00000114771	0	0	0	0

ENSG00000114779	170	68	86	130
ENSG00000114784	534	2726	2940	3002
ENSG00000114786	0	0	0	0
ENSG00000114790	0	29	17	13
ENSG00000114796	159	2066	2490	1813
ENSG00000114805	186	1292	2148	1642
ENSG00000114812	1	0	0	0
ENSG00000114841	0	12	4	3
ENSG00000114850	84	621	634	797
ENSG00000114853	1	0	0	5
ENSG00000114854	0	0	0	0
ENSG00000114857	288	524	1087	1366
ENSG00000114859	0	0	0	0
ENSG00000114861	348	1104	961	800
ENSG00000114867	32	130	122	256
ENSG00000114902	250	1095	568	1937
ENSG00000114904	131	1925	1684	2561
ENSG00000114923	0	0	0	0
ENSG00000114933	27	303	379	332
ENSG00000114942	394	294	168	545
ENSG00000114948	0	0	0	0
ENSG00000114956	361	393	137	193
ENSG00000114978	1132	23166	23252	19899
ENSG00000114982	611	3432	4040	3652
ENSG00000114988	4	38	29	17
ENSG00000114993	0	0	0	0
ENSG00000114999	4	320	151	195
ENSG00000115008	0	0	0	0
ENSG00000115009	0	0	0	0
ENSG00000115020	57	1242	1659	2157
ENSG00000115041	0	0	0	0
ENSG00000115042	3	0	2	3
ENSG00000115053	280	102	110	144
ENSG00000115073	34	53	23	49
ENSG00000115084	29	2046	2862	2658
ENSG00000115085	6	0	0	0
ENSG00000115091	821	15675	16598	17686
ENSG00000115107	0	1	0	7
ENSG00000115109	1	0	26	39
ENSG00000115112	2	0	0	0
ENSG00000115128	173	286	169	300
ENSG00000115129	4	24	17	4
ENSG00000115137	1	0	34	17
ENSG00000115138	0	63	16	53
ENSG00000115145	15	1125	941	1077
ENSG00000115155	0	0	0	0

ENSG00000115159	283	7542	11455	8429
ENSG00000115163	3	0	9	12
ENSG00000115165	34	6	3	23
ENSG00000115170	271	3588	3470	3087
ENSG00000115183	0	25	39	125
ENSG00000115194	0	0	0	0
ENSG00000115204	29	129	48	74
ENSG00000115207	8	513	389	199
ENSG00000115211	10	0	1	1
ENSG00000115216	111	963	718	696
ENSG00000115221	0	0	2	0
ENSG00000115226	0	0	0	0
ENSG00000115232	13	121	79	137
ENSG00000115233	12	236	218	288
ENSG00000115234	171	188	123	225
ENSG00000115239	4	599	545	726
ENSG00000115241	101	150	116	253
ENSG00000115252	4	0	17	44
ENSG00000115255	0	0	0	0
ENSG00000115257	0	0	0	0
ENSG00000115263	0	0	0	0
ENSG00000115266	0	0	1	0
ENSG00000115267	19	36	65	48
ENSG00000115268	814	116	64	229
ENSG00000115271	32	131	227	298
ENSG00000115274	6	67	13	7
ENSG00000115275	8	0	0	0
ENSG00000115282	5	0	5	4
ENSG00000115286	6	0	4	15
ENSG00000115289	8	29	13	0
ENSG00000115290	771	4427	5628	7105
ENSG00000115295	8	242	123	183
ENSG00000115297	0	0	0	0
ENSG00000115306	153	2360	677	2975
ENSG00000115307	120	159	60	121
ENSG00000115310	2190	9500	5538	8093
ENSG00000115317	39	112	56	84
ENSG00000115318	46	233	142	459
ENSG00000115325	236	323	78	250
ENSG00000115339	2	0	0	0
ENSG00000115350	32	24	11	2
ENSG00000115353	0	0	0	0
ENSG00000115355	321	3212	3055	3364
ENSG00000115361	0	0	1	0
ENSG00000115363	12	0	23	9
ENSG00000115364	11	25	35	60

ENSG00000115365	12	26	79	61
ENSG00000115368	13	22	51	61
ENSG00000115380	0	0	0	3
ENSG00000115386	0	0	0	0
ENSG00000115392	23	14607	12387	22650
ENSG00000115414	1	11	2	0
ENSG00000115415	82	267	435	258
ENSG00000115419	51	16748	15401	18589
ENSG00000115421	9	570	476	637
ENSG00000115423	0	188	54	57
ENSG00000115425	14	69	11	19
ENSG00000115446	3	39	10	16
ENSG00000115457	35	54	51	261
ENSG00000115459	10	178	138	257
ENSG00000115461	0	0	0	1
ENSG00000115464	329	8936	10261	11428
ENSG00000115468	0	0	0	0
ENSG00000115474	0	0	3	6
ENSG00000115484	48	518	481	665
ENSG00000115486	9	0	8	11
ENSG00000115488	0	0	0	0
ENSG00000115504	177	3942	3043	3315
ENSG00000115507	0	0	0	0
ENSG00000115514	21	72	64	95
ENSG00000115520	58	1144	1077	1106
ENSG00000115523	748	70	60	126
ENSG00000115524	239	1542	1370	1521
ENSG00000115525	23	190	153	105
ENSG00000115526	0	0	0	0
ENSG00000115539	22	13	22	30
ENSG00000115540	152	2106	1594	1469
ENSG00000115541	44	57	51	33
ENSG00000115548	85	1263	1219	1026
ENSG00000115556	84	235	100	293
ENSG00000115561	1261	2418	1999	1424
ENSG00000115568	2	12	26	47
ENSG00000115590	0	0	0	0
ENSG00000115592	0	0	0	0
ENSG00000115593	0	0	0	0
ENSG00000115594	0	27	0	4
ENSG00000115596	0	0	0	0
ENSG00000115598	0	0	0	0
ENSG00000115602	2	16	63	6
ENSG00000115604	3	30	326	32
ENSG00000115607	5	0	3	0
ENSG00000115616	0	23	0	0

ENSG00000115641	294	978	335	347
ENSG00000115648	0	0	0	0
ENSG00000115649	216	148	129	102
ENSG00000115652	736	8026	12102	14052
ENSG00000115657	4	0	4	0
ENSG00000115661	102	38	55	91
ENSG00000115665	0	0	0	0
ENSG00000115677	127	706	533	1000
ENSG00000115685	48	155	224	328
ENSG00000115687	10	103	62	91
ENSG00000115694	4	44	12	31
ENSG00000115705	0	0	0	7
ENSG00000115718	0	0	0	0
ENSG00000115738	271	59	70	21
ENSG00000115750	9	13	45	31
ENSG00000115756	320	953	464	751
ENSG00000115758	5656	42406	34086	42789
ENSG00000115760	83	9062	8863	12080
ENSG00000115761	15	1185	821	1105
ENSG00000115762	551	1374	1496	1808
ENSG00000115806	22	52	19	49
ENSG00000115808	104	1633	2038	1612
ENSG00000115816	54	63	74	107
ENSG00000115825	16	3760	3849	5023
ENSG00000115827	6	1089	652	1168
ENSG00000115828	0	0	2	3
ENSG00000115839	154	1531	1837	1778
ENSG00000115840	3	8	79	115
ENSG00000115841	12	2648	2789	3399
ENSG00000115844	0	0	0	0
ENSG00000115850	0	0	0	0
ENSG00000115866	265	686	590	968
ENSG00000115875	77	87	134	99
ENSG00000115884	0	0	0	0
ENSG00000115896	84	923	998	1116
ENSG00000115902	2	44	50	82
ENSG00000115904	127	3244	4267	3697
ENSG00000115919	14	65	30	10
ENSG00000115934	0	0	0	0
ENSG00000115935	8197	26968	28988	36571
ENSG00000115942	6	977	639	1104
ENSG00000115944	227	836	499	613
ENSG00000115946	17	43	16	38
ENSG00000115947	25	3552	3128	4716
ENSG00000115956	3553	16478	29692	24796
ENSG00000115963	0	0	0	0

ENSG00000115966	106	3366	2784	3693
ENSG00000115970	7	772	557	815
ENSG00000115977	4	5	22	85
ENSG00000115993	519	3943	3496	2661
ENSG00000115998	6	53	53	96
ENSG00000116001	266	4437	4045	4937
ENSG00000116005	14	10	20	36
ENSG00000116014	0	0	1	0
ENSG00000116016	23	39	30	10
ENSG00000116017	5	0	4	14
ENSG00000116030	204	1110	784	740
ENSG00000116031	3	0	0	0
ENSG00000116032	0	0	0	0
ENSG00000116035	0	0	0	0
ENSG00000116039	0	0	0	0
ENSG00000116044	570	3693	2892	2349
ENSG00000116062	8	88	62	71
ENSG00000116095	35	103	167	224
ENSG00000116096	0	0	3	0
ENSG00000116106	5	0	10	6
ENSG00000116117	19	129	3150	396
ENSG00000116120	12	297	246	319
ENSG00000116127	3	559	676	665
ENSG00000116128	0	0	11	4
ENSG00000116132	0	0	0	0
ENSG00000116133	0	0	6	0
ENSG00000116138	74	5760	6203	6798
ENSG00000116141	0	0	0	0
ENSG00000116147	0	0	0	0
ENSG00000116151	0	0	0	7
ENSG00000116157	0	0	4	58
ENSG00000116161	64	481	358	495
ENSG00000116171	900	2998	5539	6026
ENSG00000116176	0	0	0	0
ENSG00000116183	0	0	1	1
ENSG00000116191	16	75	153	109
ENSG00000116194	0	0	0	0
ENSG00000116198	37	323	207	334
ENSG00000116199	233	1630	3555	3628
ENSG00000116205	6	0	11	18
ENSG00000116209	241	1567	1131	1462
ENSG00000116212	2	0	4	6
ENSG00000116213	4	0	2	2
ENSG00000116218	0	0	0	0
ENSG00000116221	77	186	91	113
ENSG00000116237	15	99	37	112

ENSG00000116251	573	103	209	438
ENSG00000116254	0	0	0	0
ENSG00000116260	58	192	121	108
ENSG00000116266	297	4877	6155	4841
ENSG00000116273	11	48	30	13
ENSG00000116285	3	0	2	0
ENSG00000116288	6825	13866	10161	12049
ENSG00000116299	48	121	39	57
ENSG00000116329	0	0	0	0
ENSG00000116337	218	719	161	347
ENSG00000116350	119	2977	3311	3781
ENSG00000116353	1	0	4	9
ENSG00000116396	0	0	1	0
ENSG00000116406	13	954	719	912
ENSG00000116455	5	0	3	0
ENSG00000116459	250	762	597	1149
ENSG00000116473	833	6965	8887	7885
ENSG00000116478	119	497	618	515
ENSG00000116489	563	10445	10193	15046
ENSG00000116497	8	49	67	65
ENSG00000116514	13	900	1311	1684
ENSG00000116521	13	38	11	28
ENSG00000116525	4	12	6	10
ENSG00000116539	69	6150	6127	8395
ENSG00000116544	1	12	1	0
ENSG00000116560	121	584	566	523
ENSG00000116574	60	420	613	263
ENSG00000116580	29	5737	4096	6602
ENSG00000116584	20	24	19	13
ENSG00000116586	257	226	59	145
ENSG00000116604	43	255	181	268
ENSG00000116641	1	609	548	584
ENSG00000116649	14	0	0	0
ENSG00000116652	24	0	1	0
ENSG00000116661	1	0	0	0
ENSG00000116663	12	0	0	0
ENSG00000116667	120	686	971	817
ENSG00000116668	51	8206	6137	7910
ENSG00000116670	6	0	1	4
ENSG00000116675	8	13106	9332	15341
ENSG00000116678	830	9186	7970	9786
ENSG00000116679	617	9200	8286	10291
ENSG00000116685	190	279	451	848
ENSG00000116688	823	8881	4499	615
ENSG00000116690	0	0	1	0
ENSG00000116691	3	0	0	0

ENSG00000116698	154	646	691	979
ENSG00000116701	97	181	30	37
ENSG00000116703	0	0	0	0
ENSG00000116704	59	664	613	670
ENSG00000116711	9	4354	5660	6707
ENSG00000116717	3847	3161	2873	2220
ENSG00000116721	0	0	0	0
ENSG00000116726	0	0	0	0
ENSG00000116729	8	89	60	73
ENSG00000116731	73	802	806	993
ENSG00000116741	24	72	30	20
ENSG00000116745	0	0	0	0
ENSG00000116747	221	4595	6198	7599
ENSG00000116748	0	0	0	0
ENSG00000116750	44	826	747	1129
ENSG00000116752	23	131	101	174
ENSG00000116754	238	416	309	371
ENSG00000116761	0	0	4	16
ENSG00000116771	3	0	1	1
ENSG00000116774	0	0	0	0
ENSG00000116783	0	0	0	0
ENSG00000116785	0	0	0	0
ENSG00000116786	3	56	48	123
ENSG00000116791	6	30	8	7
ENSG00000116793	97	1525	1922	2680
ENSG00000116809	1	0	3	8
ENSG00000116815	25	205	309	318
ENSG00000116819	0	0	0	0
ENSG00000116824	47	0	0	0
ENSG00000116830	9	37	16	42
ENSG00000116833	0	0	5	1
ENSG00000116852	14	0	0	0
ENSG00000116857	119	217	141	201
ENSG00000116863	11	17	8	14
ENSG00000116871	32	37	18	23
ENSG00000116874	2	0	9	15
ENSG00000116882	0	0	0	0
ENSG00000116883	1	0	0	0
ENSG00000116885	1	0	0	0
ENSG00000116898	25	50	9	17
ENSG00000116903	1	24	49	32
ENSG00000116906	56	667	570	769
ENSG00000116918	103	210	195	312
ENSG00000116922	1	0	6	1
ENSG00000116954	64	219	147	103
ENSG00000116957	2	615	585	1248

ENSG00000116962	79	592	408	731
ENSG00000116977	206	3571	3965	2645
ENSG00000116981	0	0	0	0
ENSG00000116983	0	0	0	0
ENSG00000116984	135	3152	3125	3024
ENSG00000116985	15	75	128	202
ENSG00000116990	5	0	2	0
ENSG00000116991	1	28	0	5
ENSG00000116996	0	0	0	0
ENSG00000117000	8	3908	2470	3500
ENSG00000117009	1	22	6	4
ENSG00000117010	2	0	13	43
ENSG00000117013	28	0	14	19
ENSG00000117016	2	15	20	22
ENSG00000117020	147	13218	16491	16536
ENSG00000117036	6	7	11	9
ENSG00000117054	193	3343	2038	2899
ENSG00000117069	0	0	0	0
ENSG00000117090	36	0	0	1
ENSG00000117091	89	12	22	11
ENSG00000117114	0	0	1	7
ENSG00000117115	3	20	3	6
ENSG00000117118	36	86	30	37
ENSG00000117122	0	0	0	0
ENSG00000117133	23	35	17	4
ENSG00000117139	34	685	548	764
ENSG00000117143	7	36	31	35
ENSG00000117148	0	0	0	0
ENSG00000117151	30	279	471	371
ENSG00000117152	0	0	0	0
ENSG00000117153	41	348	401	317
ENSG00000117154	0	0	0	0
ENSG00000117155	1401	15653	23317	18496
ENSG00000117174	11	114	50	111
ENSG00000117215	0	0	0	0
ENSG00000117222	64	161	200	155
ENSG00000117226	10	41	63	89
ENSG00000117228	92	0	6	4
ENSG00000117242	1	0	1	3
ENSG00000117245	1	19	6	3
ENSG00000117262	1	0	98	193
ENSG00000117266	0	0	0	0
ENSG00000117280	107	17	4	15
ENSG00000117281	24	0	7	0
ENSG00000117289	1978	3352	2182	1203
ENSG00000117298	452	2876	1792	3300

ENSG00000117305	103	185	75	150
ENSG00000117308	4	22	2	0
ENSG00000117318	3	0	0	1
ENSG00000117322	0	0	0	4
ENSG00000117335	155	1418	2503	3745
ENSG00000117360	16	274	202	343
ENSG00000117362	219	207	136	200
ENSG00000117385	10	90	51	86
ENSG00000117394	0	0	1	8
ENSG00000117395	15	19	3	0
ENSG00000117399	6	18	2	5
ENSG00000117400	114	1134	754	1350
ENSG00000117407	0	0	0	0
ENSG00000117408	14	21	2	7
ENSG00000117410	222	25	30	59
ENSG00000117411	5	7	1	1
ENSG00000117419	5	438	247	445
ENSG00000117425	0	0	0	0
ENSG00000117448	25	0	1	0
ENSG00000117450	81	103	67	84
ENSG00000117461	2	125	156	350
ENSG00000117472	0	0	0	0
ENSG00000117475	142	2245	3121	1693
ENSG00000117477	2	4	5	17
ENSG00000117479	1	0	0	0
ENSG00000117480	1	0	3	0
ENSG00000117481	10	48	13	33
ENSG00000117500	74	1293	1832	1338
ENSG00000117501	0	0	0	0
ENSG00000117505	368	3543	5077	2986
ENSG00000117507	0	0	0	0
ENSG00000117519	1	0	1	2
ENSG00000117523	1346	3648	4340	3939
ENSG00000117525	0	0	0	0
ENSG00000117528	23	510	1081	1115
ENSG00000117533	32	406	486	587
ENSG00000117543	3	97	41	56
ENSG00000117560	0	0	1	0
ENSG00000117569	19	1704	1915	2114
ENSG00000117586	651	15324	17105	7088
ENSG00000117592	7874	27346	33078	44957
ENSG00000117593	4	12	63	99
ENSG00000117594	0	0	0	0
ENSG00000117595	0	0	0	2
ENSG00000117597	3	1	4	30
ENSG00000117598	0	0	0	0

ENSG00000117600	0	0	2	0
ENSG00000117601	0	0	0	0
ENSG00000117602	35	4043	5749	15514
ENSG00000117614	286	464	692	901
ENSG00000117616	50	804	627	1014
ENSG00000117620	6	967	766	665
ENSG00000117625	296	1413	2496	1467
ENSG00000117632	38	29	17	59
ENSG00000117640	1245	2982	1427	3156
ENSG00000117643	0	0	0	0
ENSG00000117650	2	0	13	12
ENSG00000117676	35	0	2	1
ENSG00000117682	7	35	43	83
ENSG00000117691	1327	1458	753	3176
ENSG00000117697	53	143	87	117
ENSG00000117707	0	10	0	1
ENSG00000117713	232	312	284	196
ENSG00000117724	19	381	279	387
ENSG00000117748	58	0	67	67
ENSG00000117751	52	125	122	171
ENSG00000117758	17	120	153	184
ENSG00000117791	0	0	4	4
ENSG00000117834	1	0	0	0
ENSG00000117859	28	580	704	999
ENSG00000117862	45	199	247	299
ENSG00000117868	46	11677	8825	14709
ENSG00000117877	1	0	8	2
ENSG00000117899	126	305	273	542
ENSG00000117906	3	0	14	55
ENSG00000117971	0	0	0	0
ENSG00000117983	0	0	0	0
ENSG00000117984	341	299	122	68
ENSG00000118004	0	0	0	0
ENSG00000118007	74	3601	3724	4995
ENSG00000118017	0	0	0	0
ENSG00000118046	10	62	36	63
ENSG00000118058	243	821	696	742
ENSG00000118094	0	0	0	0
ENSG00000118096	0	311	242	528
ENSG00000118113	0	0	2	6
ENSG00000118137	0	0	0	0
ENSG00000118156	0	0	0	0
ENSG00000118160	0	0	0	0
ENSG00000118162	1	0	2	0
ENSG00000118181	1357	841	435	1851
ENSG00000118193	18	1880	1880	2812

ENSG00000118194	0	0	0	0
ENSG00000118197	23	207	180	235
ENSG00000118200	1	0	0	0
ENSG00000118217	40	1275	1164	1683
ENSG00000118231	0	0	0	0
ENSG00000118242	0	0	0	0
ENSG00000118245	0	0	0	0
ENSG00000118246	2	0	4	10
ENSG00000118257	9	56	16	21
ENSG00000118260	104	1295	1095	1371
ENSG00000118263	165	798	742	1082
ENSG00000118271	0	0	0	0
ENSG00000118276	63	2595	2331	3518
ENSG00000118292	9	0	4	9
ENSG00000118298	1	0	0	0
ENSG00000118307	0	36	39	25
ENSG00000118308	700	6845	4852	9060
ENSG00000118322	1	0	0	2
ENSG00000118363	120	663	405	612
ENSG00000118369	1	0	1	0
ENSG00000118402	0	0	0	0
ENSG00000118407	1	0	1	0
ENSG00000118412	40	526	857	1017
ENSG00000118418	79	206	163	259
ENSG00000118420	5	32	37	59
ENSG00000118432	0	0	0	0
ENSG00000118434	0	0	0	0
ENSG00000118454	6	3250	3135	4959
ENSG00000118473	0	0	3	1
ENSG00000118482	151	1325	1650	1505
ENSG00000118491	0	0	0	0
ENSG00000118492	4	62	154	125
ENSG00000118495	2	0	4	2
ENSG00000118496	1	141	253	160
ENSG00000118503	14	50	22	35
ENSG00000118507	196	3083	2986	3718
ENSG00000118508	3437	5308	4660	3370
ENSG00000118513	2	135	89	138
ENSG00000118514	0	0	0	0
ENSG00000118515	0	0	1	2
ENSG00000118518	10	110	154	78
ENSG00000118520	0	29	1	0
ENSG00000118523	1	0	6	6
ENSG00000118526	0	0	0	0
ENSG00000118557	1	33	64	111
ENSG00000118564	44	617	631	966

ENSG00000118579	81	357	217	332
ENSG00000118596	15	257	668	406
ENSG00000118600	8	20	39	28
ENSG00000118620	15	565	1546	2568
ENSG00000118640	3147	781	319	659
ENSG00000118655	15	356	161	272
ENSG00000118680	3214	9136	6867	8252
ENSG00000118689	834	1216	1280	777
ENSG00000118690	1	103	56	128
ENSG00000118702	0	0	0	0
ENSG00000118705	1582	2652	1298	4402
ENSG00000118707	5	0	3	2
ENSG00000118729	0	0	1	0
ENSG00000118733	3	4	27	11
ENSG00000118762	5	1614	1251	1621
ENSG00000118777	0	0	1	9
ENSG00000118785	0	0	0	2
ENSG00000118804	0	0	2	0
ENSG00000118816	2156	2406	2744	2109
ENSG00000118849	16	36	74	18
ENSG00000118855	5094	36468	39260	39756
ENSG00000118873	103	1066	1336	1637
ENSG00000118894	2	25	1	5
ENSG00000118898	0	0	10	10
ENSG00000118900	229	706	448	874
ENSG00000118903	0	0	0	0
ENSG00000118922	17	168	492	236
ENSG00000118939	7	0	6	2
ENSG00000118946	1	1	313	2
ENSG00000118960	24	68	32	70
ENSG00000118961	10	67	46	134
ENSG00000118965	1	3	19	28
ENSG00000118971	26	0	16	27
ENSG00000118972	0	0	0	0
ENSG00000118976	0	0	1	1
ENSG00000118985	10	48	95	163
ENSG00000118990	0	0	0	0
ENSG00000118997	5	88	41	65
ENSG00000119004	16	486	408	616
ENSG00000119013	137	217	259	327
ENSG00000119041	9	114	137	186
ENSG00000119042	4	195	99	305
ENSG00000119048	987	1474	1648	841
ENSG00000119121	1	0	0	0
ENSG00000119125	0	0	0	0
ENSG00000119138	442	1559	2012	1676

ENSG00000119139	242	2237	2342	2922
ENSG00000119147	1	0	0	0
ENSG00000119185	37	280	538	311
ENSG00000119203	19	413	412	528
ENSG00000119227	1	0	0	0
ENSG00000119231	62	1280	1356	1051
ENSG00000119242	2052	4201	1903	4779
ENSG00000119280	525	6088	3395	3768
ENSG00000119283	0	0	0	0
ENSG00000119285	6	143	234	136
ENSG00000119314	289	2979	3885	3642
ENSG00000119318	326	9313	6124	9623
ENSG00000119321	37	272	235	261
ENSG00000119326	415	3813	2995	3702
ENSG00000119328	44	99	98	83
ENSG00000119333	0	1	9	16
ENSG00000119335	820	3048	3286	2684
ENSG00000119383	167	785	405	522
ENSG00000119392	29	1987	771	1922
ENSG00000119396	540	4423	4669	4055
ENSG00000119397	70	1163	1156	1513
ENSG00000119401	3	0	1	5
ENSG00000119402	50	149	201	427
ENSG00000119403	8	19	5	19
ENSG00000119408	17	89	34	62
ENSG00000119411	2	0	0	0
ENSG00000119414	248	3328	4150	3177
ENSG00000119421	29	98	41	60
ENSG00000119431	3	0	0	2
ENSG00000119440	0	0	0	0
ENSG00000119446	36	505	871	558
ENSG00000119457	0	0	0	0
ENSG00000119471	17	179	408	403
ENSG00000119487	41	1836	1846	3149
ENSG00000119508	28	186	144	301
ENSG00000119509	16	475	552	830
ENSG00000119514	11	146	105	105
ENSG00000119522	8	39	114	122
ENSG00000119523	1	78	9	13
ENSG00000119535	4	12	2	0
ENSG00000119537	2	19	33	37
ENSG00000119541	176	744	517	541
ENSG00000119547	0	14	0	0
ENSG00000119559	16	29	26	31
ENSG00000119574	4	9	6	6
ENSG00000119596	42	174	155	146

ENSG00000119599	1	18	0	2
ENSG00000119608	0	0	0	0
ENSG00000119614	0	0	0	0
ENSG00000119616	14	90	67	90
ENSG00000119630	2	0	0	0
ENSG00000119632	1434	644	298	621
ENSG00000119636	74	102	274	143
ENSG00000119638	20	231	272	472
ENSG00000119640	88	184	166	172
ENSG00000119650	6	0	0	0
ENSG00000119655	381	696	671	829
ENSG00000119660	0	0	0	0
ENSG00000119661	7	135	244	294
ENSG00000119669	12	9	5	5
ENSG00000119673	1	0	0	0
ENSG00000119681	3	0	2	0
ENSG00000119682	14	181	92	176
ENSG00000119684	28048	27375	19167	24903
ENSG00000119685	7	751	509	708
ENSG00000119686	0	31	5	3
ENSG00000119688	9	13	7	5
ENSG00000119689	26	948	901	405
ENSG00000119698	0	0	2	5
ENSG00000119699	0	0	0	0
ENSG00000119703	1	0	0	0
ENSG00000119705	53	12	6	14
ENSG00000119707	117	295	247	388
ENSG00000119711	10	3	36	35
ENSG00000119714	1	0	0	0
ENSG00000119715	0	0	3	0
ENSG00000119718	216	427	532	638
ENSG00000119720	75	98	20	70
ENSG00000119723	2	47	12	12
ENSG00000119725	1	0	4	9
ENSG00000119729	34	352	257	324
ENSG00000119737	0	0	3	1
ENSG00000119760	14	161	81	104
ENSG00000119771	0	0	0	0
ENSG00000119772	41	64	52	72
ENSG00000119777	22	1	28	12
ENSG00000119778	41	8947	8590	10389
ENSG00000119782	314	325	145	324
ENSG00000119787	3	572	419	574
ENSG00000119801	4783	83888	77810	45259
ENSG00000119812	5	17	50	28
ENSG00000119820	24	254	235	292

ENSG00000119844	528	2438	3007	3711
ENSG00000119862	1340	18198	18803	12946
ENSG00000119865	0	0	5	5
ENSG00000119866	16	0	9	8
ENSG00000119878	62	135	86	100
ENSG00000119888	0	0	7	15
ENSG00000119899	90	1327	1109	2387
ENSG00000119900	55	849	1224	1044
ENSG00000119906	19	1041	1149	1596
ENSG00000119912	8	2210	1543	2693
ENSG00000119913	0	0	0	0
ENSG00000119915	0	0	0	0
ENSG00000119917	38	28	27	32
ENSG00000119919	0	0	0	0
ENSG00000119922	25	75	21	29
ENSG00000119927	1	30	9	0
ENSG00000119929	12	32	11	11
ENSG00000119938	0	0	0	0
ENSG00000119943	3	3	1	0
ENSG00000119946	0	0	1	0
ENSG00000119950	36	81	126	54
ENSG00000119953	222	1247	856	1107
ENSG00000119965	26	348	196	250
ENSG00000119969	5	360	264	295
ENSG00000119973	0	0	0	0
ENSG00000119977	3	0	4	6
ENSG00000119979	95	310	285	352
ENSG00000119986	1	0	0	0
ENSG00000120008	104	2359	2629	2174
ENSG00000120029	12	1343	1427	2171
ENSG00000120049	1	0	9	2
ENSG00000120051	4	52	40	30
ENSG00000120053	8	0	5	0
ENSG00000120054	0	0	0	0
ENSG00000120055	0	0	0	0
ENSG00000120057	0	0	0	0
ENSG00000120063	981	9175	11577	9888
ENSG00000120068	1	0	0	0
ENSG00000120071	17	445	310	743
ENSG00000120075	0	0	0	0
ENSG00000120087	11	0	0	0
ENSG00000120088	0	0	0	0
ENSG00000120093	1	0	0	0
ENSG00000120094	0	0	0	0
ENSG00000120129	1	0	0	0
ENSG00000120137	60	2183	3315	4002

ENSG00000120149	0	0	0	0
ENSG00000120156	0	0	5	0
ENSG00000120158	47	853	614	603
ENSG00000120159	258	1644	2151	2011
ENSG00000120160	0	0	4	1
ENSG00000120162	8	24	10	6
ENSG00000120210	0	0	0	0
ENSG00000120211	0	0	0	0
ENSG00000120215	3	7	4	6
ENSG00000120217	0	14	77	11
ENSG00000120235	0	0	0	0
ENSG00000120242	0	0	0	0
ENSG00000120251	0	0	1	0
ENSG00000120253	9	413	290	726
ENSG00000120254	0	132	102	123
ENSG00000120256	0	0	6	3
ENSG00000120262	10	0	1	0
ENSG00000120265	560	4118	3595	5725
ENSG00000120278	0	0	8	3
ENSG00000120279	629	3513	4221	4873
ENSG00000120280	13	0	5	1
ENSG00000120289	0	0	0	0
ENSG00000120306	12	0	5	1
ENSG00000120314	6	1	4	26
ENSG00000120318	3	0	4	0
ENSG00000120322	0	0	0	0
ENSG00000120324	0	0	0	0
ENSG00000120327	0	0	0	0
ENSG00000120328	0	0	0	0
ENSG00000120329	0	0	0	0
ENSG00000120332	0	0	0	0
ENSG00000120333	48	194	130	167
ENSG00000120334	6	0	26	25
ENSG00000120337	1	127	52	22
ENSG00000120341	0	0	0	0
ENSG00000120370	2	18	20	19
ENSG00000120436	0	0	0	0
ENSG00000120437	5	19	40	26
ENSG00000120438	77	1328	1263	1269
ENSG00000120440	0	0	0	0
ENSG00000120451	38	1195	994	195
ENSG00000120457	0	0	0	0
ENSG00000120458	6	0	4	5
ENSG00000120471	2	0	1	0
ENSG00000120498	0	0	0	0
ENSG00000120500	1	0	0	0

ENSG00000120509	17	30	17	18
ENSG00000120519	8	749	1026	877
ENSG00000120526	6	127	111	111
ENSG00000120533	85	341	166	186
ENSG00000120539	7	1360	1482	1994
ENSG00000120549	2	8	3	20
ENSG00000120555	0	0	0	7
ENSG00000120563	0	0	0	0
ENSG00000120586	0	0	0	0
ENSG00000120594	315	9082	9569	11326
ENSG00000120616	38	363	308	539
ENSG00000120645	0	0	0	0
ENSG00000120647	6	432	366	621
ENSG00000120656	29	238	208	354
ENSG00000120658	0	0	0	5
ENSG00000120659	0	0	0	0
ENSG00000120662	6	969	1242	1664
ENSG00000120664	0	62	14	37
ENSG00000120669	0	0	0	0
ENSG00000120675	2112	2320	1841	1813
ENSG00000120685	19	164	169	228
ENSG00000120686	57	114	181	282
ENSG00000120688	211	606	419	344
ENSG00000120690	4539	25659	27649	17801
ENSG00000120693	0	0	2	1
ENSG00000120694	52	170	152	177
ENSG00000120696	1	0	7	13
ENSG00000120697	1	0	11	29
ENSG00000120699	33	16	25	37
ENSG00000120705	40	289	133	186
ENSG00000120708	31	0	1	36
ENSG00000120709	8	189	138	47
ENSG00000120725	7	108	136	239
ENSG00000120727	1743	24754	22996	22577
ENSG00000120729	0	0	0	0
ENSG00000120733	89	1121	758	934
ENSG00000120738	14	0	5	5
ENSG00000120742	594	2644	2936	1935
ENSG00000120756	1	23	75	103
ENSG00000120784	5	0	1	47
ENSG00000120798	5	200	182	362
ENSG00000120800	10	230	168	197
ENSG00000120802	60	578	618	628
ENSG00000120805	17	128	186	179
ENSG00000120820	0	0	0	1
ENSG00000120832	2	29	19	26

ENSG00000120833	44	316	258	117
ENSG00000120837	61	1609	1441	1893
ENSG00000120860	427	2656	2012	3384
ENSG00000120868	28	986	1138	1571
ENSG00000120875	2	0	0	0
ENSG00000120885	68010	61522	52216	66981
ENSG00000120889	2	0	0	0
ENSG00000120896	1	0	0	0
ENSG00000120899	72	334	168	361
ENSG00000120903	93	451	130	293
ENSG00000120907	0	0	0	0
ENSG00000120910	9	340	348	493
ENSG00000120913	9	0	0	0
ENSG00000120915	34	177	38	115
ENSG00000120925	19	385	291	569
ENSG00000120937	0	0	0	0
ENSG00000120942	5	0	0	0
ENSG00000120948	54	180	216	265
ENSG00000120949	6	0	0	0
ENSG00000120952	0	0	0	0
ENSG00000120963	126	325	251	253
ENSG00000120992	250	1307	1706	2209
ENSG00000121005	0	11	31	22
ENSG00000121022	79	273	278	257
ENSG00000121039	28	149	286	123
ENSG00000121053	0	0	0	0
ENSG00000121057	1	0	2	9
ENSG00000121058	11	48	53	36
ENSG00000121060	11	83	56	126
ENSG00000121064	16	0	56	31
ENSG00000121067	343	343	303	248
ENSG00000121068	0	0	0	0
ENSG00000121073	43	108	88	161
ENSG00000121075	0	4	0	0
ENSG00000121083	1687	4917	3305	3923
ENSG00000121089	0	0	0	0
ENSG00000121101	10	97	122	266
ENSG00000121104	21	36	38	64
ENSG00000121152	3	361	430	658
ENSG00000121207	0	0	0	0
ENSG00000121210	61	623	551	489
ENSG00000121211	0	113	648	824
ENSG00000121236	0	0	0	0
ENSG00000121270	0	0	1	2
ENSG00000121274	21	92	115	167
ENSG00000121281	0	0	2	10

ENSG00000121289	3	226	300	547
ENSG00000121297	5	0	0	1
ENSG00000121310	6	0	0	0
ENSG00000121314	0	0	0	0
ENSG00000121316	9	0	0	2
ENSG00000121318	3	0	6	2
ENSG00000121335	0	0	0	0
ENSG00000121350	8	12	59	34
ENSG00000121351	1	0	0	0
ENSG00000121361	0	0	0	0
ENSG00000121377	0	0	0	0
ENSG00000121380	1	0	0	3
ENSG00000121381	0	0	0	0
ENSG00000121388	0	0	0	0
ENSG00000121390	41	1276	1122	1754
ENSG00000121406	4	10	5	10
ENSG00000121410	0	0	0	0
ENSG00000121413	7	49	28	59
ENSG00000121417	12	57	103	143
ENSG00000121440	0	0	0	0
ENSG00000121446	0	0	2	0
ENSG00000121454	0	0	1	0
ENSG00000121481	24	474	624	525
ENSG00000121486	27	554	499	496
ENSG00000121542	8	503	331	428
ENSG00000121552	42	1	3	9
ENSG00000121570	9	12	80	49
ENSG00000121577	25	42	10	26
ENSG00000121578	7	3	34	26
ENSG00000121579	322	2045	1858	1369
ENSG00000121594	0	0	0	0
ENSG00000121621	2	1302	1261	1740
ENSG00000121634	0	0	0	0
ENSG00000121644	21	260	262	344
ENSG00000121653	0	0	0	0
ENSG00000121671	4	16	10	17
ENSG00000121680	2	0	4	6
ENSG00000121690	1	0	1	0
ENSG00000121691	53	77	60	240
ENSG00000121716	1	20	113	183
ENSG00000121741	46	8242	7893	8825
ENSG00000121742	0	0	0	0
ENSG00000121743	0	0	0	0
ENSG00000121749	1742	8306	9114	8273
ENSG00000121753	0	0	0	0
ENSG00000121764	0	23	5	5

ENSG00000121766	2158	6648	5831	7820
ENSG00000121769	1	0	0	9
ENSG00000121774	163	630	451	569
ENSG00000121775	282	266	101	146
ENSG00000121797	23	41	32	6
ENSG00000121807	1	0	1	0
ENSG00000121848	613	1905	1764	1997
ENSG00000121851	142	111	26	47
ENSG00000121853	0	0	0	0
ENSG00000121858	24	0	3	16
ENSG00000121864	26	347	310	499
ENSG00000121871	0	0	0	0
ENSG00000121879	66	926	1423	1320
ENSG00000121892	77	4266	4080	5544
ENSG00000121895	1	0	24	39
ENSG00000121897	5	0	10	16
ENSG00000121898	4	0	12	29
ENSG00000121900	0	0	0	0
ENSG00000121903	0	0	0	0
ENSG00000121904	1	0	0	0
ENSG00000121905	0	0	0	0
ENSG00000121931	48	46	122	73
ENSG00000121933	2	0	1	15
ENSG00000121940	6	191	232	306
ENSG00000121957	0	269	339	453
ENSG00000121964	327	14573	15501	20720
ENSG00000121966	31	85	42	31
ENSG00000121988	5	508	520	473
ENSG00000121989	40	32177	19093	45786
ENSG00000122008	49	1836	3202	3170
ENSG00000122012	1	48	19	7
ENSG00000122025	1	34	9	4
ENSG00000122026	7099	736	441	1270
ENSG00000122033	139	102	119	135
ENSG00000122034	196	643	557	697
ENSG00000122035	4	0	1	0
ENSG00000122042	46	274	217	201
ENSG00000122043	0	0	0	0
ENSG00000122068	220	1301	1284	1325
ENSG00000122085	512	1410	773	1091
ENSG00000122121	6	0	0	0
ENSG00000122122	21	0	3	1
ENSG00000122126	2	136	108	288
ENSG00000122133	0	0	0	0
ENSG00000122136	0	0	0	0
ENSG00000122140	7	0	1	0

ENSG00000122145	0	0	0	0
ENSG00000122176	0	0	0	0
ENSG00000122180	0	0	0	0
ENSG00000122188	1	0	0	0
ENSG00000122194	0	0	0	0
ENSG00000122203	351	1101	1255	1795
ENSG00000122218	507	3114	2941	2860
ENSG00000122223	23	189	88	146
ENSG00000122224	3	0	0	0
ENSG00000122254	0	0	0	0
ENSG00000122257	738	1745	1056	1135
ENSG00000122299	293	3095	3278	3787
ENSG00000122304	0	0	0	0
ENSG00000122335	80	803	875	538
ENSG00000122359	486	821	504	913
ENSG00000122367	0	0	0	0
ENSG00000122375	0	0	0	0
ENSG00000122376	14	189	416	422
ENSG00000122378	5	0	0	6
ENSG00000122386	0	17	4	9
ENSG00000122390	5	4	37	45
ENSG00000122406	1210	188	214	236
ENSG00000122417	43	870	755	910
ENSG00000122420	0	0	0	0
ENSG00000122432	21	298	290	245
ENSG00000122435	9	126	79	120
ENSG00000122477	0	11	25	33
ENSG00000122481	28	79	110	131
ENSG00000122482	80	495	837	907
ENSG00000122483	296	8364	10380	9191
ENSG00000122484	4	2694	1977	5223
ENSG00000122490	49	95	77	74
ENSG00000122497	1	135	315	604
ENSG00000122507	5	1332	1084	1320
ENSG00000122512	2	121	129	235
ENSG00000122515	109	112	86	173
ENSG00000122543	0	0	3	1
ENSG00000122545	10658	35319	42868	48740
ENSG00000122547	2	0	0	0
ENSG00000122548	0	0	0	0
ENSG00000122550	6	350	234	223
ENSG00000122557	61	4278	2855	7783
ENSG00000122565	511	5350	3760	3392
ENSG00000122566	1462	3619	3195	5013
ENSG00000122574	0	0	5	5
ENSG00000122584	0	0	0	0

ENSG00000122585	0	0	0	0
ENSG00000122591	125	2775	3802	2874
ENSG00000122592	0	0	0	0
ENSG00000122641	0	0	0	0
ENSG00000122642	5	40	75	87
ENSG00000122643	20576	61863	69342	58088
ENSG00000122644	8	33	37	24
ENSG00000122674	2	295	201	335
ENSG00000122678	1	10	0	0
ENSG00000122679	33	0	10	70
ENSG00000122687	17	0	0	0
ENSG00000122691	1	0	7	1
ENSG00000122692	50	624	660	1014
ENSG00000122694	43	13	8	32
ENSG00000122696	17	0	39	59
ENSG00000122705	173	1803	1620	2591
ENSG00000122707	2	298	447	310
ENSG00000122711	6	0	0	0
ENSG00000122718	0	0	0	0
ENSG00000122728	0	0	0	0
ENSG00000122729	5	5	27	47
ENSG00000122733	0	0	1	0
ENSG00000122735	0	0	0	0
ENSG00000122741	37	165	150	227
ENSG00000122756	0	0	0	0
ENSG00000122778	6	51	107	125
ENSG00000122779	110	4178	4925	5516
ENSG00000122783	610	1012	814	1368
ENSG00000122786	2403	5873	5395	7848
ENSG00000122787	0	0	1	0
ENSG00000122824	0	0	0	0
ENSG00000122852	0	0	0	0
ENSG00000122859	0	0	0	0
ENSG00000122861	0	0	0	0
ENSG00000122862	7423	37538	42914	55471
ENSG00000122863	0	0	0	0
ENSG00000122870	0	0	1	0
ENSG00000122872	0	0	0	0
ENSG00000122873	20	25	1	3
ENSG00000122877	0	0	0	0
ENSG00000122882	12	351	344	558
ENSG00000122884	7	378	291	360
ENSG00000122912	6	956	735	757
ENSG00000122952	4	35	16	11
ENSG00000122958	74	2368	2167	2119
ENSG00000122965	3	24	3	4

ENSG00000122966	1	311	241	352
ENSG00000122970	48	1202	1029	1395
ENSG00000122971	3	0	0	0
ENSG00000122986	7	0	5	3
ENSG00000123009	0	0	0	0
ENSG00000123064	0	0	0	0
ENSG00000123066	146	1802	2049	2114
ENSG00000123080	34	216	136	260
ENSG00000123091	7991	55394	57438	79373
ENSG00000123094	1	14	11	5
ENSG00000123095	0	0	0	0
ENSG00000123096	3	0	0	0
ENSG00000123104	312	5579	6031	5790
ENSG00000123106	609	8180	5708	10482
ENSG00000123119	4	35	4	4
ENSG00000123124	528	5089	7385	6586
ENSG00000123130	12	30	64	97
ENSG00000123131	3	14	14	10
ENSG00000123136	23	0	5	8
ENSG00000123143	42	68	18	57
ENSG00000123144	590	853	523	793
ENSG00000123146	57	136	93	75
ENSG00000123154	0	19	17	18
ENSG00000123159	20	47	21	75
ENSG00000123165	0	0	0	0
ENSG00000123171	0	0	0	0
ENSG00000123178	7	191	355	366
ENSG00000123179	10	0	10	16
ENSG00000123191	7	66	41	30
ENSG00000123200	321	1111	1285	1248
ENSG00000123201	0	0	0	0
ENSG00000123213	2	202	216	254
ENSG00000123219	27	1234	2195	2911
ENSG00000123240	110	412	593	574
ENSG00000123243	0	0	0	0
ENSG00000123268	29	708	947	1366
ENSG00000123297	0	0	0	0
ENSG00000123307	0	0	0	3
ENSG00000123329	24	19	3	2
ENSG00000123338	74	1	43	63
ENSG00000123342	1	0	0	0
ENSG00000123349	1946	1103	671	1522
ENSG00000123352	140	1356	1562	2116
ENSG00000123353	8	109	34	25
ENSG00000123358	1	8	3	0
ENSG00000123360	0	0	0	0

ENSG00000123364	0	0	0	0
ENSG00000123374	38	567	314	339
ENSG00000123384	5	0	0	0
ENSG00000123388	0	0	0	0
ENSG00000123395	1	0	1	0
ENSG00000123405	1653	3517	3238	1907
ENSG00000123407	0	0	0	0
ENSG00000123411	6	1	2	11
ENSG00000123415	22	0	0	10
ENSG00000123416	227	5075	3386	6141
ENSG00000123427	4	0	13	7
ENSG00000123444	1	30	13	12
ENSG00000123447	0	0	0	0
ENSG00000123453	1	0	0	0
ENSG00000123454	0	0	0	0
ENSG00000123472	153	516	458	723
ENSG00000123473	5	2437	2183	2166
ENSG00000123485	22	63	80	56
ENSG00000123496	0	0	1	0
ENSG00000123500	35	106	163	320
ENSG00000123505	2172	37512	26134	18606
ENSG00000123545	7	0	11	7
ENSG00000123552	1	1375	1556	1739
ENSG00000123560	0	0	1	5
ENSG00000123561	0	0	0	0
ENSG00000123562	35	230	295	360
ENSG00000123569	0	0	0	0
ENSG00000123570	8	57	79	47
ENSG00000123572	0	0	0	0
ENSG00000123575	29	439	413	354
ENSG00000123576	0	0	0	0
ENSG00000123584	0	0	0	0
ENSG00000123594	0	0	0	0
ENSG00000123595	17	29	60	38
ENSG00000123600	13	3077	2338	3824
ENSG00000123607	4	153	166	219
ENSG00000123609	181	384	496	321
ENSG00000123610	2	0	3	0
ENSG00000123612	0	8	1	7
ENSG00000123636	46	284	407	493
ENSG00000123643	1	0	8	8
ENSG00000123684	51	1868	1391	1639
ENSG00000123685	14	0	0	0
ENSG00000123689	0	0	0	0
ENSG00000123700	0	0	0	0
ENSG00000123728	26	192	413	284

ENSG00000123737	17	428	175	388
ENSG00000123739	4265	11612	15429	14402
ENSG00000123810	31	2	12	22
ENSG00000123815	7	9	15	22
ENSG00000123836	4	1	37	26
ENSG00000123838	0	0	0	0
ENSG00000123843	0	0	0	0
ENSG00000123870	0	34	53	101
ENSG00000123892	25	181	171	244
ENSG00000123901	0	0	0	0
ENSG00000123908	65	1066	784	984
ENSG00000123933	15	11	12	8
ENSG00000123965	2	322	154	271
ENSG00000123975	20	215	109	42
ENSG00000123977	0	0	0	0
ENSG00000123983	3	482	463	517
ENSG00000123989	0	0	0	0
ENSG00000123992	25	10	5	2
ENSG00000123999	0	0	0	0
ENSG00000124003	0	0	0	0
ENSG00000124006	1	0	3	8
ENSG00000124019	3	29	24	2
ENSG00000124067	0	0	0	0
ENSG00000124074	3	13	9	0
ENSG00000124089	0	0	0	0
ENSG00000124091	0	0	0	0
ENSG00000124092	0	0	0	0
ENSG00000124097	18	0	16	6
ENSG00000124098	279	259	277	212
ENSG00000124102	0	0	0	0
ENSG00000124103	0	0	0	0
ENSG00000124104	16	93	28	30
ENSG00000124107	5	0	0	0
ENSG00000124116	237	357	28	104
ENSG00000124120	133	407	303	259
ENSG00000124126	36	15	13	14
ENSG00000124134	0	0	0	0
ENSG00000124140	0	0	0	0
ENSG00000124143	0	0	0	0
ENSG00000124145	117	1150	1030	1239
ENSG00000124151	354	3296	3167	3734
ENSG00000124155	6	4	28	27
ENSG00000124157	0	0	0	0
ENSG00000124159	0	0	1	0
ENSG00000124160	9	48	25	38
ENSG00000124164	465	8207	7935	11543

ENSG00000124171	1	0	0	0
ENSG00000124172	10871	25222	35622	17785
ENSG00000124177	224	1789	1619	2145
ENSG00000124181	6	0	2	0
ENSG00000124191	0	0	0	0
ENSG00000124193	1963	28151	15902	15473
ENSG00000124194	0	0	0	0
ENSG00000124196	0	0	0	0
ENSG00000124198	116	1374	1803	2056
ENSG00000124201	145	322	183	167
ENSG00000124203	2	0	3	0
ENSG00000124205	0	0	0	0
ENSG00000124207	31	3561	3640	3870
ENSG00000124208	0	0	0	0
ENSG00000124209	66	174	329	342
ENSG00000124212	0	10	2	0
ENSG00000124214	351	2000	1720	2112
ENSG00000124215	18	19	53	32
ENSG00000124216	1	0	0	0
ENSG00000124217	0	0	0	5
ENSG00000124222	269	104	255	273
ENSG00000124224	2	62	124	151
ENSG00000124225	0	0	0	0
ENSG00000124226	26	103	76	61
ENSG00000124227	0	0	0	0
ENSG00000124228	41	0	12	29
ENSG00000124232	0	0	0	0
ENSG00000124233	0	0	0	0
ENSG00000124237	0	0	0	0
ENSG00000124243	12	762	1126	1014
ENSG00000124249	0	0	0	0
ENSG00000124251	0	0	0	0
ENSG00000124253	0	0	0	0
ENSG00000124256	6	0	0	0
ENSG00000124257	29	40	20	23
ENSG00000124260	0	0	0	0
ENSG00000124275	8	534	153	533
ENSG00000124279	1	22	33	47
ENSG00000124299	14	0	11	13
ENSG00000124302	6	136	91	46
ENSG00000124313	0	0	0	0
ENSG00000124333	997	13814	14563	9446
ENSG00000124334	0	0	0	0
ENSG00000124343	0	0	2	4
ENSG00000124356	51	123	120	182
ENSG00000124357	78	4	1	8

ENSG00000124370	2	0	7	2
ENSG00000124374	0	0	0	3
ENSG00000124380	47	103	88	133
ENSG00000124383	31	0	28	53
ENSG00000124391	0	0	0	0
ENSG00000124399	0	0	0	0
ENSG00000124406	78	27659	28369	35596
ENSG00000124422	180	4076	2617	2855
ENSG00000124429	0	0	2	0
ENSG00000124440	4	0	0	0
ENSG00000124444	2	0	2	1
ENSG00000124449	0	0	0	0
ENSG00000124459	4	125	131	292
ENSG00000124466	0	0	0	0
ENSG00000124467	0	0	0	0
ENSG00000124469	1	0	0	17
ENSG00000124479	0	0	0	0
ENSG00000124486	177	4563	6182	3624
ENSG00000124490	0	0	0	5
ENSG00000124491	18026	153717	151208	230255
ENSG00000124493	0	0	0	0
ENSG00000124496	6	53	13	12
ENSG00000124507	1	13	0	0
ENSG00000124508	6	137	9	69
ENSG00000124523	3	1297	858	1412
ENSG00000124529	19	169	257	176
ENSG00000124532	37	1214	1520	2598
ENSG00000124535	782	2323	2569	1925
ENSG00000124541	16	159	113	66
ENSG00000124549	3	0	0	1
ENSG00000124557	0	0	0	0
ENSG00000124562	104	8	16	56
ENSG00000124564	3	0	6	0
ENSG00000124568	0	0	0	0
ENSG00000124570	978	2095	1479	2249
ENSG00000124571	3	824	596	746
ENSG00000124574	0	0	2	5
ENSG00000124575	5	25	4	13
ENSG00000124578	0	0	0	0
ENSG00000124587	16	16	14	9
ENSG00000124588	203	169	60	247
ENSG00000124593	12	0	5	12
ENSG00000124596	83	96	120	125
ENSG00000124602	0	0	0	0
ENSG00000124608	1	0	0	2
ENSG00000124610	1	0	1	2

ENSG00000124613	0	0	1	0
ENSG00000124614	952	416	111	270
ENSG00000124615	0	0	1	0
ENSG00000124635	2330	11957	14653	13331
ENSG00000124641	13	58	40	81
ENSG00000124657	362	1214	791	580
ENSG00000124659	5	0	0	2
ENSG00000124664	0	0	0	0
ENSG00000124678	0	0	0	0
ENSG00000124688	156	419	370	174
ENSG00000124693	90	574	270	422
ENSG00000124701	0	0	0	0
ENSG00000124702	77	148	108	202
ENSG00000124713	2	0	0	6
ENSG00000124721	1	0	0	0
ENSG00000124731	11	26	53	20
ENSG00000124733	697	1347	907	1544
ENSG00000124743	0	0	0	0
ENSG00000124749	0	0	0	0
ENSG00000124762	2447	5865	3847	2651
ENSG00000124766	45	110	71	25
ENSG00000124767	102	140	83	137
ENSG00000124772	1674	938	1035	1032
ENSG00000124780	0	0	0	0
ENSG00000124782	10	666	613	875
ENSG00000124783	295	4101	4759	5939
ENSG00000124784	19	32	148	129
ENSG00000124785	7	89	0	5
ENSG00000124786	9	89	76	90
ENSG00000124787	1	0	11	18
ENSG00000124788	3	153	137	57
ENSG00000124789	16	1911	1788	2761
ENSG00000124795	976	11255	9013	12666
ENSG00000124802	19	53	18	32
ENSG00000124812	0	0	0	0
ENSG00000124813	8	46	0	0
ENSG00000124818	0	2	0	6
ENSG00000124827	0	0	0	0
ENSG00000124831	2800	2891	3008	2339
ENSG00000124835	0	0	1	0
ENSG00000124839	0	0	0	0
ENSG00000124875	1	19	7	38
ENSG00000124882	6	0	45	28
ENSG00000124900	0	0	0	0
ENSG00000124915	0	0	0	0
ENSG00000124920	0	0	0	0

ENSG00000124935	0	0	0	0
ENSG00000124939	0	0	0	0
ENSG00000124942	59	201	113	71
ENSG00000125037	13215	40774	24266	41554
ENSG00000125046	0	4	2	0
ENSG00000125084	0	0	0	0
ENSG00000125089	0	0	0	2
ENSG00000125107	34	708	745	1158
ENSG00000125122	5	55	8	24
ENSG00000125124	11	112	120	63
ENSG00000125144	0	0	0	0
ENSG00000125148	293	203	85	86
ENSG00000125149	1	5	28	64
ENSG00000125166	25	49	84	100
ENSG00000125170	2	0	2	6
ENSG00000125207	0	0	0	0
ENSG00000125245	76	9	26	12
ENSG00000125246	4	47	7	8
ENSG00000125247	0	7	18	20
ENSG00000125249	159	1134	2082	1310
ENSG00000125255	0	0	0	0
ENSG00000125257	1397	26207	37516	54044
ENSG00000125266	71	64	126	220
ENSG00000125285	0	0	0	1
ENSG00000125304	51	2263	1981	2664
ENSG00000125319	1	183	82	173
ENSG00000125337	5	0	0	0
ENSG00000125347	46	0	7	3
ENSG00000125351	74	88	68	56
ENSG00000125352	20	53	3	0
ENSG00000125354	1723	6037	6080	8250
ENSG00000125355	0	0	0	5
ENSG00000125356	3905	1451	968	1548
ENSG00000125363	0	0	0	0
ENSG00000125375	409	501	614	408
ENSG00000125378	0	0	0	0
ENSG00000125384	5	0	0	0
ENSG00000125385	0	0	0	0
ENSG00000125386	31	1085	663	1088
ENSG00000125388	210	1167	1217	2011
ENSG00000125398	0	0	0	0
ENSG00000125409	0	17	0	2
ENSG00000125414	0	0	0	0
ENSG00000125430	2	0	2	0
ENSG00000125434	3	0	3	12
ENSG00000125445	19	135	182	222

ENSG00000125447	29	108	123	201
ENSG00000125449	128	122	33	59
ENSG00000125450	10	95	40	95
ENSG00000125454	1	0	0	5
ENSG00000125457	177	717	411	768
ENSG00000125458	5	85	37	20
ENSG00000125459	0	0	2	5
ENSG00000125462	1	0	0	0
ENSG00000125482	31	63	27	61
ENSG00000125484	5	18	25	8
ENSG00000125485	0	20	12	12
ENSG00000125492	0	0	0	0
ENSG00000125498	1	0	0	0
ENSG00000125503	15	132	50	124
ENSG00000125505	5	0	10	4
ENSG00000125508	0	0	0	0
ENSG00000125510	0	0	0	0
ENSG00000125514	0	0	0	0
ENSG00000125520	7	0	0	1
ENSG00000125522	0	0	0	0
ENSG00000125531	1	0	6	0
ENSG00000125533	0	0	0	0
ENSG00000125534	291	866	1117	1433
ENSG00000125538	2	20	10	9
ENSG00000125551	0	0	0	0
ENSG00000125571	0	0	0	0
ENSG00000125611	28	0	0	0
ENSG00000125618	1	0	2	5
ENSG00000125629	31	569	553	316
ENSG00000125630	3	83	48	58
ENSG00000125631	0	0	0	0
ENSG00000125633	6	71	167	278
ENSG00000125637	7	29	9	5
ENSG00000125648	5	0	0	0
ENSG00000125650	5	3	1	0
ENSG00000125651	53	87	30	50
ENSG00000125652	16	0	5	9
ENSG00000125656	19	21	4	16
ENSG00000125657	2	0	0	0
ENSG00000125675	5	16	11	19
ENSG00000125676	873	8511	8355	10409
ENSG00000125686	24	682	677	1042
ENSG00000125691	1875	211	126	195
ENSG00000125695	0	0	0	0
ENSG00000125703	12	1624	1923	2848
ENSG00000125726	0	0	0	0

ENSG00000125730	2	0	0	1
ENSG00000125731	0	0	0	2
ENSG00000125733	15	289	160	181
ENSG00000125734	221	257	190	156
ENSG00000125735	0	0	0	4
ENSG00000125740	1	0	1	0
ENSG00000125741	7	0	0	6
ENSG00000125743	237	0	7	9
ENSG00000125744	246	529	325	348
ENSG00000125746	2	24	5	19
ENSG00000125753	633	1205	579	1193
ENSG00000125755	43	220	137	112
ENSG00000125772	303	5435	7709	4471
ENSG00000125775	12	44	12	9
ENSG00000125779	182	1246	955	946
ENSG00000125780	1	0	0	0
ENSG00000125787	0	0	0	0
ENSG00000125788	0	0	0	0
ENSG00000125798	0	0	0	0
ENSG00000125804	0	1	0	0
ENSG00000125810	8	0	3	8
ENSG00000125812	5	113	62	93
ENSG00000125813	0	0	0	0
ENSG00000125814	86	667	803	1245
ENSG00000125815	0	0	0	0
ENSG00000125816	0	0	0	0
ENSG00000125817	6	36	10	29
ENSG00000125818	234	298	356	559
ENSG00000125820	0	0	0	0
ENSG00000125821	85	381	205	205
ENSG00000125823	0	0	0	0
ENSG00000125826	32	79	29	45
ENSG00000125827	350	1265	1919	1774
ENSG00000125831	0	0	0	0
ENSG00000125834	1	8	10	4
ENSG00000125835	45	39	23	30
ENSG00000125841	2	0	0	2
ENSG00000125843	337	191	158	175
ENSG00000125844	13	0	1	24
ENSG00000125845	0	0	1	0
ENSG00000125846	2	97	196	173
ENSG00000125848	0	0	0	0
ENSG00000125850	0	0	0	0
ENSG00000125851	0	0	0	0
ENSG00000125861	0	0	0	0
ENSG00000125863	245	1567	1126	1448

ENSG00000125864	5	0	7	2
ENSG00000125868	1001	2472	2865	4560
ENSG00000125869	214	903	542	276
ENSG00000125870	142	196	111	188
ENSG00000125871	114	106	248	198
ENSG00000125872	0	0	0	0
ENSG00000125875	199	512	476	448
ENSG00000125877	59	29	7	6
ENSG00000125878	2	0	1	2
ENSG00000125879	0	0	0	0
ENSG00000125885	4	124	135	192
ENSG00000125888	0	0	0	0
ENSG00000125895	0	0	0	0
ENSG00000125898	694	263	165	135
ENSG00000125899	0	0	0	0
ENSG00000125900	0	0	0	0
ENSG00000125901	1	0	0	0
ENSG00000125903	0	0	0	0
ENSG00000125910	4	0	0	0
ENSG00000125912	0	0	0	0
ENSG00000125931	0	0	1	0
ENSG00000125944	245	744	661	1386
ENSG00000125945	0	56	18	14
ENSG00000125952	10902	56705	51189	61869
ENSG00000125954	0	0	0	0
ENSG00000125962	16	276	368	377
ENSG00000125965	0	0	0	0
ENSG00000125966	0	42	0	5
ENSG00000125967	30	76	7	8
ENSG00000125968	1	0	1	0
ENSG00000125970	150	1472	853	1575
ENSG00000125971	8509	18404	19106	20396
ENSG00000125975	0	0	0	0
ENSG00000125977	270	2802	2145	4406
ENSG00000125991	98	20	16	33
ENSG00000125995	260	55	10	11
ENSG00000125997	0	0	0	0
ENSG00000125998	0	0	0	0
ENSG00000125999	0	0	0	0
ENSG00000126001	6	0	2	16
ENSG00000126003	17	26	22	12
ENSG00000126005	229	275	81	215
ENSG00000126010	0	0	9	12
ENSG00000126012	7	68	55	99
ENSG00000126016	4	13	30	24
ENSG00000126062	25	21	16	9

ENSG00000126067	74	42	53	70
ENSG00000126070	7	714	682	883
ENSG00000126088	281	448	273	347
ENSG00000126091	668	2029	1261	2093
ENSG00000126106	0	16	12	2
ENSG00000126107	5	46	6	8
ENSG00000126214	4	2	4	2
ENSG00000126215	0	0	1	2
ENSG00000126216	15	284	258	794
ENSG00000126217	258	871	476	498
ENSG00000126218	0	0	0	0
ENSG00000126226	15	95	107	91
ENSG00000126231	0	0	2	1
ENSG00000126233	0	0	0	0
ENSG00000126243	7	0	0	0
ENSG00000126246	0	0	0	6
ENSG00000126247	2868	2066	1553	2622
ENSG00000126249	0	0	0	4
ENSG00000126251	1	0	0	4
ENSG00000126254	25	26	12	23
ENSG00000126259	0	0	0	0
ENSG00000126261	40	3307	2992	5139
ENSG00000126262	1	0	0	0
ENSG00000126264	639	123	160	274
ENSG00000126266	0	0	0	0
ENSG00000126267	3374	1441	1030	1441
ENSG00000126337	0	0	0	0
ENSG00000126351	18	30	57	44
ENSG00000126353	6	0	0	0
ENSG00000126368	5	0	0	12
ENSG00000126391	93	143	74	60
ENSG00000126432	1522	1991	435	1739
ENSG00000126453	1	0	1	3
ENSG00000126456	15	24	31	38
ENSG00000126457	8	39	3	23
ENSG00000126458	248	188	70	172
ENSG00000126460	9	43	15	23
ENSG00000126461	2	0	1	14
ENSG00000126464	2	41	4	18
ENSG00000126467	1	0	0	0
ENSG00000126500	0	0	0	0
ENSG00000126522	1	0	0	4
ENSG00000126524	61	5025	3594	8779
ENSG00000126542	0	0	1	0
ENSG00000126545	0	0	0	0
ENSG00000126549	0	0	0	0

ENSG00000126550	0	0	1	0
ENSG00000126561	80	243	70	187
ENSG00000126562	0	0	0	0
ENSG00000126581	246	1785	2095	2944
ENSG00000126583	0	20	0	0
ENSG00000126602	0	0	5	9
ENSG00000126603	0	0	0	0
ENSG00000126653	406	178	202	187
ENSG00000126698	446	801	524	877
ENSG00000126705	6	16	5	6
ENSG00000126709	29	119	58	63
ENSG00000126733	1	0	0	0
ENSG00000126746	13	163	184	158
ENSG00000126749	17	0	5	9
ENSG00000126752	0	0	0	0
ENSG00000126756	312	355	236	245
ENSG00000126759	19	0	0	0
ENSG00000126767	14	22	14	16
ENSG00000126768	177	56	54	99
ENSG00000126773	31	242	460	413
ENSG00000126775	17	122	189	204
ENSG00000126777	196	1952	2157	2517
ENSG00000126778	0	0	0	0
ENSG00000126785	0	0	0	5
ENSG00000126787	17	733	511	952
ENSG00000126790	3	2	9	34
ENSG00000126803	13	51	54	45
ENSG00000126804	53	708	1075	931
ENSG00000126814	4	43	20	19
ENSG00000126821	5	31	37	29
ENSG00000126822	6	134	47	38
ENSG00000126838	0	0	0	0
ENSG00000126856	31	18	25	65
ENSG00000126858	17	2198	3263	4514
ENSG00000126860	31	23	27	25
ENSG00000126861	0	0	0	0
ENSG00000126870	254	1010	501	902
ENSG00000126878	9	25	9	10
ENSG00000126882	40	119	61	85
ENSG00000126883	61	271	249	363
ENSG00000126890	0	0	0	0
ENSG00000126895	0	0	0	0
ENSG00000126903	694	778	986	722
ENSG00000126934	23	217	143	178
ENSG00000126945	81	841	806	877
ENSG00000126947	0	39	7	6

ENSG00000126950	0	0	0	0
ENSG00000126952	0	0	0	0
ENSG00000126953	9	0	10	0
ENSG00000126970	49	147	144	199
ENSG00000127022	66	2812	3001	4213
ENSG00000127054	15	56	0	10
ENSG00000127074	0	0	0	0
ENSG00000127080	0	0	13	12
ENSG00000127081	0	0	29	37
ENSG00000127083	0	0	0	0
ENSG00000127084	90	257	200	184
ENSG00000127124	9	17	3	8
ENSG00000127125	150	456	305	465
ENSG00000127129	0	0	0	0
ENSG00000127152	14	0	1	1
ENSG00000127184	652	182	102	241
ENSG00000127191	1	8	5	12
ENSG00000127220	0	0	0	0
ENSG00000127241	5	0	3	12
ENSG00000127249	66	495	274	618
ENSG00000127252	222	1103	600	1015
ENSG00000127311	3	169	139	232
ENSG00000127314	2109	114188	121798	157213
ENSG00000127318	0	0	0	0
ENSG00000127324	0	0	0	0
ENSG00000127325	8	67	244	284
ENSG00000127328	25	1094	1184	1552
ENSG00000127329	0	14	0	1
ENSG00000127334	28	72	107	42
ENSG00000127337	8	156	112	109
ENSG00000127362	0	0	0	0
ENSG00000127364	0	0	0	0
ENSG00000127366	0	0	0	0
ENSG00000127377	3	0	0	0
ENSG00000127399	1	0	1	0
ENSG00000127412	0	0	0	0
ENSG00000127415	0	0	1	1
ENSG00000127418	1	0	1	0
ENSG00000127419	3	0	10	15
ENSG00000127423	13	171	168	91
ENSG00000127445	22	83	44	62
ENSG00000127452	8	1	12	16
ENSG00000127463	2	8	12	22
ENSG00000127472	6	0	5	1
ENSG00000127481	207	1250	1070	1245
ENSG00000127483	191	6275	5402	10504

ENSG00000127507	7	135	52	120
ENSG00000127511	52	294	128	106
ENSG00000127515	0	0	0	0
ENSG00000127526	887	2464	1958	2258
ENSG00000127527	429	909	689	512
ENSG00000127528	14	0	2	0
ENSG00000127529	0	0	0	0
ENSG00000127530	0	0	0	0
ENSG00000127533	769	311	511	487
ENSG00000127540	4	8	0	0
ENSG00000127554	4	3	0	2
ENSG00000127561	0	0	0	0
ENSG00000127564	0	0	1	0
ENSG00000127578	0	0	0	0
ENSG00000127580	0	0	0	0
ENSG00000127585	1	0	0	0
ENSG00000127586	0	0	0	0
ENSG00000127588	0	0	0	0
ENSG00000127589	0	0	0	0
ENSG00000127603	350	4931	4588	5478
ENSG00000127616	35	69	66	141
ENSG00000127663	15	595	197	511
ENSG00000127666	6	0	3	2
ENSG00000127688	1	38	72	106
ENSG00000127720	37	977	970	1169
ENSG00000127743	0	0	0	0
ENSG00000127774	0	0	13	13
ENSG00000127780	0	0	0	0
ENSG00000127804	5	124	79	172
ENSG00000127824	15571	16091	9350	11871
ENSG00000127831	665	1984	1060	2195
ENSG00000127837	14	48	12	5
ENSG00000127838	967	875	648	1020
ENSG00000127863	0	20	28	56
ENSG00000127870	117	2004	2331	1995
ENSG00000127884	60	31	7	20
ENSG00000127903	0	0	0	0
ENSG00000127914	810	2626	3676	1824
ENSG00000127920	79676	70960	41424	104051
ENSG00000127922	317	277	155	278
ENSG00000127928	5	10	6	0
ENSG00000127946	8	30	11	8
ENSG00000127947	4849	59829	69840	60263
ENSG00000127948	2	7	2	0
ENSG00000127951	54	0	25	17
ENSG00000127952	61	225	100	100

ENSG00000127954	1	0	0	0
ENSG00000127955	9	176	320	272
ENSG00000127957	0	256	172	322
ENSG00000127980	5	839	1052	1163
ENSG00000127989	1	30	9	7
ENSG00000127990	1	0	0	0
ENSG00000127993	10	1	15	3
ENSG00000127995	7	466	441	668
ENSG00000128000	7	234	228	303
ENSG00000128011	3	0	0	0
ENSG00000128016	7	14	8	3
ENSG00000128039	1	0	4	0
ENSG00000128040	0	0	0	0
ENSG00000128045	0	0	0	0
ENSG00000128050	14	53	34	5
ENSG00000128052	1	41	3	7
ENSG00000128059	6	27	23	60
ENSG00000128159	0	0	1	9
ENSG00000128165	0	0	0	0
ENSG00000128185	13	13	3	12
ENSG00000128191	10	107	37	65
ENSG00000128203	9	0	1	0
ENSG00000128218	3	0	0	0
ENSG00000128228	5	6	0	0
ENSG00000128242	0	0	0	0
ENSG00000128245	2821	19269	16279	16040
ENSG00000128250	0	12	0	0
ENSG00000128253	0	0	0	0
ENSG00000128254	6	56	17	22
ENSG00000128262	1	0	0	0
ENSG00000128266	2844	14583	10427	18260
ENSG00000128268	4	12	10	14
ENSG00000128271	0	0	0	0
ENSG00000128272	6224	19475	16840	17794
ENSG00000128274	0	0	8	5
ENSG00000128276	0	0	0	0
ENSG00000128283	0	0	0	0
ENSG00000128284	31	61	4	8
ENSG00000128285	0	0	0	0
ENSG00000128294	2507	6077	4835	6232
ENSG00000128298	1	0	0	7
ENSG00000128309	211	776	359	638
ENSG00000128310	0	0	0	0
ENSG00000128311	1166	713	202	367
ENSG00000128313	1	0	0	0
ENSG00000128322	0	0	0	0

ENSG00000128335	18	0	3	0
ENSG00000128340	46	25	41	56
ENSG00000128342	0	0	0	0
ENSG00000128346	1	0	0	0
ENSG00000128383	38	0	0	3
ENSG00000128394	26	0	12	21
ENSG00000128408	0	0	3	0
ENSG00000128422	0	0	0	0
ENSG00000128438	0	0	0	0
ENSG00000128463	60	80	56	101
ENSG00000128482	0	0	0	0
ENSG00000128487	298	4479	4745	6729
ENSG00000128510	0	0	0	0
ENSG00000128512	1	0	0	6
ENSG00000128513	22	889	617	854
ENSG00000128519	0	0	0	0
ENSG00000128524	661	854	768	1010
ENSG00000128534	86	259	385	473
ENSG00000128536	0	0	1	0
ENSG00000128563	33	156	219	312
ENSG00000128564	0	0	0	0
ENSG00000128567	2	0	4	4
ENSG00000128573	0	0	0	3
ENSG00000128578	67	501	442	481
ENSG00000128581	5	18	7	4
ENSG00000128585	57	5287	4006	5300
ENSG00000128590	2	93	35	68
ENSG00000128591	0	0	0	0
ENSG00000128594	2	0	3	1
ENSG00000128595	379	1665	3498	3022
ENSG00000128596	0	20	13	31
ENSG00000128602	0	49	8	23
ENSG00000128604	6	0	1	0
ENSG00000128606	0	0	5	0
ENSG00000128607	11	2378	3454	5223
ENSG00000128609	1437	17481	20754	19406
ENSG00000128610	0	0	0	0
ENSG00000128617	1	1	2	0
ENSG00000128626	7	4	0	0
ENSG00000128641	1	0	0	2
ENSG00000128645	0	0	0	0
ENSG00000128652	0	0	0	0
ENSG00000128654	6	134	102	196
ENSG00000128655	0	46	20	9
ENSG00000128656	1	50	33	109
ENSG00000128683	2	0	1	2

ENSG00000128692	0	35	30	51
ENSG00000128694	3	17	49	75
ENSG00000128699	39	165	149	141
ENSG00000128708	125	4768	2889	5658
ENSG00000128709	0	0	0	0
ENSG00000128710	0	0	0	0
ENSG00000128713	0	0	0	0
ENSG00000128714	0	0	0	0
ENSG00000128731	79	1267	750	1203
ENSG00000128739	8	0	3	9
ENSG00000128789	1	0	0	1
ENSG00000128791	126	3084	3874	2464
ENSG00000128802	2	0	0	0
ENSG00000128805	5	41	9	9
ENSG00000128815	13	0	1	0
ENSG00000128829	17	213	240	371
ENSG00000128833	6	58	66	171
ENSG00000128849	0	0	0	0
ENSG00000128872	3	0	18	48
ENSG00000128881	12	899	667	831
ENSG00000128886	0	0	0	0
ENSG00000128891	5	25	12	21
ENSG00000128908	44	1560	1238	1847
ENSG00000128915	23	812	561	794
ENSG00000128917	0	0	0	0
ENSG00000128918	12	92	92	106
ENSG00000128923	174	604	786	817
ENSG00000128928	30	13	8	14
ENSG00000128944	2	117	108	250
ENSG00000128951	34	84	60	115
ENSG00000128965	0	0	0	0
ENSG00000128973	9	9	14	17
ENSG00000128989	622	11013	12909	14905
ENSG00000129003	59	9748	9106	12440
ENSG00000129007	14	0	7	3
ENSG00000129009	0	0	0	0
ENSG00000129028	0	0	4	0
ENSG00000129038	0	0	0	0
ENSG00000129048	0	0	0	0
ENSG00000129055	76	45	41	127
ENSG00000129071	146	519	394	670
ENSG00000129083	36	473	632	992
ENSG00000129084	201	79	72	107
ENSG00000129103	52	3	30	55
ENSG00000129116	17	0	8	35
ENSG00000129128	121	2157	3073	2752

ENSG00000129151	0	0	0	0
ENSG00000129152	0	0	0	0
ENSG00000129158	7	68	58	79
ENSG00000129159	0	19	0	1
ENSG00000129167	6	0	1	4
ENSG00000129170	0	0	0	0
ENSG00000129173	1	14	5	20
ENSG00000129187	56	1201	1173	1290
ENSG00000129194	0	0	0	0
ENSG00000129195	0	0	0	2
ENSG00000129197	8	25	0	5
ENSG00000129204	3	0	15	3
ENSG00000129214	0	0	0	0
ENSG00000129219	1	0	0	0
ENSG00000129221	0	0	0	0
ENSG00000129226	0	0	2	0
ENSG00000129235	83	56	124	63
ENSG00000129244	2	12	0	2
ENSG00000129245	11	60	51	71
ENSG00000129250	8	17	22	33
ENSG00000129255	5	0	6	3
ENSG00000129270	14	0	2	9
ENSG00000129277	31	0	0	0
ENSG00000129282	0	0	0	0
ENSG00000129292	888	4598	4826	3315
ENSG00000129295	322	735	509	673
ENSG00000129315	16	61	65	92
ENSG00000129317	49	414	168	248
ENSG00000129347	11	0	16	20
ENSG00000129351	59	172	52	122
ENSG00000129353	1948	9730	5677	7215
ENSG00000129354	166	403	154	426
ENSG00000129355	5745	4503	3564	4713
ENSG00000129422	6	0	6	2
ENSG00000129437	0	0	0	0
ENSG00000129450	2	0	0	1
ENSG00000129451	0	0	0	0
ENSG00000129455	0	0	0	0
ENSG00000129460	7	0	6	8
ENSG00000129465	2	0	0	3
ENSG00000129467	0	0	0	0
ENSG00000129472	38	758	664	910
ENSG00000129473	204	1244	523	454
ENSG00000129474	0	0	0	0
ENSG00000129480	4	0	11	10
ENSG00000129484	5	51	93	145

ENSG00000129493	13	723	629	686
ENSG00000129514	1	0	1	0
ENSG00000129515	531	8502	8540	9832
ENSG00000129518	125	249	207	234
ENSG00000129521	252	2092	1927	1771
ENSG00000129534	83	1864	1977	1882
ENSG00000129535	2	0	5	0
ENSG00000129538	36	7	36	65
ENSG00000129559	574	72	56	119
ENSG00000129562	4873	7935	5047	10110
ENSG00000129566	11	62	93	459
ENSG00000129595	1	0	5	5
ENSG00000129596	1	0	1	14
ENSG00000129625	119	934	1441	2316
ENSG00000129636	252	4816	6460	6276
ENSG00000129646	0	1	13	72
ENSG00000129654	0	0	0	0
ENSG00000129657	2504	6334	5025	3755
ENSG00000129667	2	83	16	54
ENSG00000129673	1	0	1	4
ENSG00000129675	43	531	710	383
ENSG00000129680	229	518	515	910
ENSG00000129682	32	326	231	159
ENSG00000129691	151	4498	4180	5713
ENSG00000129696	2	41	25	44
ENSG00000129744	0	0	0	0
ENSG00000129749	1	0	0	0
ENSG00000129757	48	11	2	6
ENSG00000129810	6	100	118	149
ENSG00000129816	0	0	0	0
ENSG00000129824	198	0	0	4
ENSG00000129845	0	0	0	0
ENSG00000129862	0	0	0	0
ENSG00000129864	0	0	0	0
ENSG00000129873	0	0	0	0
ENSG00000129910	0	0	0	0
ENSG00000129911	5	39	10	29
ENSG00000129925	70	114	49	161
ENSG00000129932	0	3	0	0
ENSG00000129933	30	28	43	45
ENSG00000129946	0	0	0	0
ENSG00000129951	0	0	0	0
ENSG00000129965	0	0	0	0
ENSG00000129968	132	864	655	1201
ENSG00000129988	0	0	0	0
ENSG00000129990	9	0	0	0

ENSG00000129991	2	0	4	5
ENSG00000129993	59	109	77	121
ENSG00000130005	12	0	3	27
ENSG00000130021	12	8	53	61
ENSG00000130023	1	1194	801	1766
ENSG00000130024	11	57	112	127
ENSG00000130032	4	0	0	0
ENSG00000130035	0	10	2	1
ENSG00000130037	0	0	0	0
ENSG00000130038	2	40	8	9
ENSG00000130045	2	0	0	0
ENSG00000130052	52	248	164	114
ENSG00000130054	0	0	0	0
ENSG00000130055	3	0	0	3
ENSG00000130066	16379	68117	43952	72427
ENSG00000130119	227	453	347	831
ENSG00000130147	5	0	1	7
ENSG00000130150	28	151	93	69
ENSG00000130158	4	9	0	1
ENSG00000130159	0	0	1	0
ENSG00000130164	5	0	2	2
ENSG00000130165	63	25	13	6
ENSG00000130167	0	0	0	0
ENSG00000130173	0	0	0	0
ENSG00000130175	8	110	48	122
ENSG00000130176	27	127	132	141
ENSG00000130177	282	2945	2188	1774
ENSG00000130182	0	0	0	0
ENSG00000130193	0	0	0	0
ENSG00000130201	46	6	38	55
ENSG00000130202	16	80	24	33
ENSG00000130203	0	0	0	0
ENSG00000130204	0	0	0	0
ENSG00000130208	1	0	0	0
ENSG00000130222	3	0	3	6
ENSG00000130224	0	0	50	31
ENSG00000130226	0	0	0	0
ENSG00000130227	506	6926	7130	6698
ENSG00000130234	0	0	0	0
ENSG00000130244	17	0	2	11
ENSG00000130254	101	316	283	493
ENSG00000130255	1767	144	36	196
ENSG00000130270	0	0	0	0
ENSG00000130283	0	0	0	0
ENSG00000130287	0	12	0	0
ENSG00000130294	0	0	0	0

ENSG00000130299	5	82	5	2
ENSG00000130300	0	0	0	0
ENSG00000130303	25	0	4	3
ENSG00000130304	1	0	3	2
ENSG00000130305	4	0	4	0
ENSG00000130307	0	9	0	0
ENSG00000130309	1	14	4	8
ENSG00000130311	23	82	110	221
ENSG00000130312	40	1	10	31
ENSG00000130313	16	25	6	21
ENSG00000130332	17	0	3	3
ENSG00000130338	6	65	52	46
ENSG00000130340	271	763	742	580
ENSG00000130347	3	17	36	56
ENSG00000130348	10	46	6	10
ENSG00000130349	4	13	63	77
ENSG00000130363	2	22	16	28
ENSG00000130368	1	3	3	0
ENSG00000130377	0	0	1	0
ENSG00000130382	0	0	2	33
ENSG00000130383	0	0	0	0
ENSG00000130385	0	0	0	0
ENSG00000130396	3	60	103	27
ENSG00000130402	241	609	257	593
ENSG00000130413	1	198	62	124
ENSG00000130414	66	1124	925	1310
ENSG00000130427	0	0	0	0
ENSG00000130429	14273	11695	7082	13766
ENSG00000130433	0	0	0	0
ENSG00000130449	9	267	213	197
ENSG00000130475	8	15	22	40
ENSG00000130477	0	0	0	3
ENSG00000130479	6	3	2	9
ENSG00000130487	2	0	0	0
ENSG00000130489	0	0	0	0
ENSG00000130508	0	0	0	0
ENSG00000130511	1	0	3	4
ENSG00000130513	0	0	0	0
ENSG00000130517	36	36	74	46
ENSG00000130518	1	0	0	0
ENSG00000130520	34	42	43	109
ENSG00000130522	118	68	51	24
ENSG00000130528	1	0	1	0
ENSG00000130529	46	27	85	50
ENSG00000130538	0	0	0	0
ENSG00000130540	0	0	0	0

ENSG00000130544	5	0	4	12
ENSG00000130545	0	0	0	0
ENSG00000130558	0	0	0	0
ENSG00000130559	7	744	395	684
ENSG00000130560	77	137	127	138
ENSG00000130561	3	0	0	3
ENSG00000130584	1	0	1	0
ENSG00000130589	0	0	0	3
ENSG00000130590	2	0	0	0
ENSG00000130592	160	17	6	6
ENSG00000130595	4	36	7	39
ENSG00000130598	9	0	0	0
ENSG00000130600	59	695	84	111
ENSG00000130612	0	0	0	0
ENSG00000130635	0	0	2	0
ENSG00000130638	103	4731	2779	4319
ENSG00000130640	18	12	12	11
ENSG00000130643	0	0	0	0
ENSG00000130649	1	0	0	2
ENSG00000130653	0	0	0	0
ENSG00000130656	0	0	0	0
ENSG00000130669	1	0	2	3
ENSG00000130675	0	0	0	0
ENSG00000130684	0	0	8	21
ENSG00000130695	3	512	501	761
ENSG00000130699	8	73	50	92
ENSG00000130700	0	0	0	0
ENSG00000130701	0	0	0	0
ENSG00000130702	3	0	9	0
ENSG00000130703	179	1005	661	746
ENSG00000130706	44	86	52	28
ENSG00000130707	11	19	8	3
ENSG00000130711	0	0	0	0
ENSG00000130713	5	0	0	1
ENSG00000130714	0	857	527	719
ENSG00000130717	19	71	30	88
ENSG00000130720	0	0	0	0
ENSG00000130723	65	341	288	476
ENSG00000130724	352	323	93	174
ENSG00000130725	17	37	17	34
ENSG00000130726	25	41	13	12
ENSG00000130731	53	35	37	9
ENSG00000130733	6	1	12	5
ENSG00000130734	4	50	6	20
ENSG00000130741	82	372	320	432
ENSG00000130748	1	0	4	8

ENSG00000130749	40	195	130	238
ENSG00000130751	0	0	0	0
ENSG00000130755	2928	2593	2343	2460
ENSG00000130758	0	4	7	11
ENSG00000130762	0	0	0	0
ENSG00000130764	38	66	22	55
ENSG00000130766	5	7	3	0
ENSG00000130768	0	0	0	0
ENSG00000130770	329	328	269	592
ENSG00000130772	14	16	6	0
ENSG00000130775	54	30	7	4
ENSG00000130779	1145	3439	7364	8500
ENSG00000130783	2	99	181	316
ENSG00000130787	1	0	0	0
ENSG00000130803	23	72	54	72
ENSG00000130810	0	0	0	0
ENSG00000130811	48	198	40	81
ENSG00000130812	0	0	1	0
ENSG00000130813	22	36	46	35
ENSG00000130816	709	6015	4248	7507
ENSG00000130818	13	92	82	156
ENSG00000130821	1	0	4	2
ENSG00000130822	0	0	0	1
ENSG00000130826	68	220	134	260
ENSG00000130827	0	0	0	0
ENSG00000130829	0	0	0	0
ENSG00000130830	5640	22984	19295	20113
ENSG00000130844	4	0	3	5
ENSG00000130856	31	2531	1841	2553
ENSG00000130876	0	0	0	0
ENSG00000130881	0	0	0	4
ENSG00000130921	48	43	13	10
ENSG00000130935	14	56	58	129
ENSG00000130939	53	1264	1044	1209
ENSG00000130940	0	5	0	12
ENSG00000130943	0	0	0	0
ENSG00000130948	174	680	459	1002
ENSG00000130950	0	0	0	0
ENSG00000130956	21	170	858	899
ENSG00000130957	0	0	0	0
ENSG00000130958	425	7302	5060	4319
ENSG00000130962	0	118	61	134
ENSG00000130985	158	261	193	300
ENSG00000130988	0	0	1	0
ENSG00000130997	0	165	133	226
ENSG00000131002	13	818	4	2482

ENSG00000131007	0	0	0	0
ENSG00000131009	0	0	0	0
ENSG00000131013	42	623	982	1125
ENSG00000131015	0	0	0	0
ENSG00000131016	0	0	1	0
ENSG00000131018	50	134	239	191
ENSG00000131019	0	0	0	0
ENSG00000131023	14	1185	1175	1668
ENSG00000131037	3	0	7	2
ENSG00000131042	16	0	0	0
ENSG00000131043	8	25	10	32
ENSG00000131044	0	0	0	3
ENSG00000131050	0	0	0	0
ENSG00000131051	813	24921	19831	28291
ENSG00000131055	0	0	0	0
ENSG00000131059	0	0	0	0
ENSG00000131061	0	99	11	13
ENSG00000131067	0	0	0	0
ENSG00000131068	0	0	0	0
ENSG00000131069	104	112	76	144
ENSG00000131080	0	0	1	0
ENSG00000131089	10	102	121	103
ENSG00000131094	0	0	0	0
ENSG00000131095	51	165	53	243
ENSG00000131096	0	0	0	0
ENSG00000131097	0	0	0	0
ENSG00000131100	822	6311	5054	8066
ENSG00000131115	5	33	11	27
ENSG00000131116	6	19	45	116
ENSG00000131126	0	0	0	1
ENSG00000131127	3	125	204	210
ENSG00000131142	0	0	0	0
ENSG00000131143	2420	1559	1003	1407
ENSG00000131148	31	43	35	79
ENSG00000131149	78	2244	2176	3856
ENSG00000131152	0	0	0	0
ENSG00000131153	3	0	0	0
ENSG00000131165	172	252	102	150
ENSG00000131171	4052	57109	50801	82506
ENSG00000131174	490	466	298	745
ENSG00000131183	0	0	0	0
ENSG00000131187	4	0	5	5
ENSG00000131188	31	231	110	254
ENSG00000131196	229	294	130	219
ENSG00000131203	5	18	3	3
ENSG00000131233	0	0	0	0

ENSG00000131236	6654	13568	16394	17594
ENSG00000131238	27	9	49	55
ENSG00000131242	10	0	3	3
ENSG00000131263	28	582	860	552
ENSG00000131264	0	0	0	0
ENSG00000131269	6	132	222	325
ENSG00000131323	6	3	4	13
ENSG00000131351	7	16	18	16
ENSG00000131355	0	0	3	0
ENSG00000131368	30	1	33	38
ENSG00000131370	11	547	446	870
ENSG00000131373	3	136	92	119
ENSG00000131374	85	4037	4077	5104
ENSG00000131375	13	817	660	1186
ENSG00000131378	32	55	25	28
ENSG00000131379	4	0	13	17
ENSG00000131381	6	22	24	58
ENSG00000131386	0	0	0	0
ENSG00000131389	154	2302	1624	1761
ENSG00000131398	0	0	0	0
ENSG00000131400	0	0	0	0
ENSG00000131401	21	0	1	2
ENSG00000131408	57	324	153	283
ENSG00000131409	0	0	0	0
ENSG00000131435	0	0	0	0
ENSG00000131437	9	64	92	119
ENSG00000131446	34	69	40	85
ENSG00000131459	1	0	0	0
ENSG00000131462	3	0	26	53
ENSG00000131467	34	119	231	343
ENSG00000131469	1369	396	115	246
ENSG00000131470	0	15	8	23
ENSG00000131471	1	0	0	0
ENSG00000131473	31	70	84	138
ENSG00000131475	30	10	16	20
ENSG00000131477	1	0	1	0
ENSG00000131480	0	0	0	0
ENSG00000131482	0	0	0	0
ENSG00000131484	0	0	1	0
ENSG00000131495	177	29	31	37
ENSG00000131503	1	0	23	19
ENSG00000131504	3137	24287	15461	23991
ENSG00000131507	99	1746	1937	1335
ENSG00000131508	277	4836	4387	6319
ENSG00000131538	0	0	0	0
ENSG00000131548	0	0	0	0

ENSG00000131558	83	1010	663	997
ENSG00000131584	0	49	4	3
ENSG00000131591	0	0	0	0
ENSG00000131620	0	0	0	0
ENSG00000131626	87	906	656	1120
ENSG00000131634	139	266	78	239
ENSG00000131650	0	0	0	0
ENSG00000131652	4	0	0	0
ENSG00000131653	13	151	85	187
ENSG00000131668	0	0	0	0
ENSG00000131669	130	5	67	149
ENSG00000131686	0	0	0	0
ENSG00000131697	0	0	2	8
ENSG00000131711	86	524	621	553
ENSG00000131721	0	0	0	0
ENSG00000131724	5	12	15	19
ENSG00000131725	368	17272	19766	27101
ENSG00000131730	0	0	1	0
ENSG00000131732	15	16	7	3
ENSG00000131737	0	0	0	0
ENSG00000131738	0	0	0	0
ENSG00000131746	0	0	0	0
ENSG00000131747	101	1088	852	1089
ENSG00000131748	8	72	43	57
ENSG00000131759	6	0	1	0
ENSG00000131771	0	0	0	0
ENSG00000131773	4	0	3	4
ENSG00000131778	1220	5339	7009	6612
ENSG00000131779	37	26	35	29
ENSG00000131781	54	412	575	570
ENSG00000131788	7	11	47	25
ENSG00000131791	95	602	908	434
ENSG00000131795	122	360	355	316
ENSG00000131797	0	0	0	0
ENSG00000131808	0	0	0	0
ENSG00000131828	31	173	47	80
ENSG00000131831	2	0	2	0
ENSG00000131844	7	83	105	143
ENSG00000131845	1	3	8	4
ENSG00000131848	12	27	22	21
ENSG00000131849	0	0	2	0
ENSG00000131864	0	0	0	0
ENSG00000131871	9	0	13	17
ENSG00000131873	86	609	537	346
ENSG00000131876	12	0	18	11
ENSG00000131885	0	0	0	0

ENSG00000131899	2	0	9	7
ENSG00000131910	0	0	0	0
ENSG00000131914	0	0	0	0
ENSG00000131931	14	101	80	68
ENSG00000131941	0	0	0	0
ENSG00000131943	7	0	4	2
ENSG00000131944	5	0	1	0
ENSG00000131951	0	139	73	120
ENSG00000131966	661	4822	6464	3782
ENSG00000131969	4	0	2	0
ENSG00000131979	28	79	32	45
ENSG00000131981	173	285	178	213
ENSG00000131982	0	0	0	0
ENSG00000132000	1	0	1	7
ENSG00000132002	38	0	35	68
ENSG00000132003	1	0	0	0
ENSG00000132004	54	18	6	17
ENSG00000132005	25	9	14	13
ENSG00000132010	0	0	0	0
ENSG00000132016	2	0	0	2
ENSG00000132017	0	28	1	14
ENSG00000132024	2	0	2	3
ENSG00000132026	0	0	0	0
ENSG00000132031	0	0	4	0
ENSG00000132109	15	0	38	23
ENSG00000132122	0	44	25	69
ENSG00000132128	22	211	71	101
ENSG00000132130	0	0	0	0
ENSG00000132139	0	0	0	0
ENSG00000132141	3	112	113	142
ENSG00000132142	6	236	365	441
ENSG00000132153	19	0	7	46
ENSG00000132155	59	424	532	496
ENSG00000132164	5	13	15	10
ENSG00000132170	0	0	1	0
ENSG00000132182	7	16	13	28
ENSG00000132185	3	0	0	0
ENSG00000132196	2	0	1	3
ENSG00000132199	0	29	29	24
ENSG00000132204	0	34	22	0
ENSG00000132205	1	0	2	0
ENSG00000132207	0	0	0	0
ENSG00000132254	5	10	7	2
ENSG00000132256	22	81	147	70
ENSG00000132259	0	0	0	4
ENSG00000132274	40	77	101	106

ENSG00000132275	40	15	27	34
ENSG00000132286	32	43	102	83
ENSG00000132294	57	679	794	719
ENSG00000132297	9	0	8	11
ENSG00000132300	16	740	554	788
ENSG00000132305	66	359	303	565
ENSG00000132313	15	39	46	63
ENSG00000132321	1	0	0	0
ENSG00000132323	11	1478	1065	1722
ENSG00000132326	6	0	4	0
ENSG00000132329	72	42	64	119
ENSG00000132330	1	0	0	0
ENSG00000132334	57	0	2	9
ENSG00000132341	147	567	311	415
ENSG00000132356	80	7056	7793	7279
ENSG00000132357	29	362	486	347
ENSG00000132359	24	214	203	379
ENSG00000132361	0	0	1	4
ENSG00000132376	131	269	171	336
ENSG00000132382	1	0	0	1
ENSG00000132383	8769	20817	15394	13363
ENSG00000132386	25	11	1	9
ENSG00000132388	119	4524	3376	4292
ENSG00000132394	0	26	16	45
ENSG00000132405	6	8337	5028	10702
ENSG00000132406	8	369	204	239
ENSG00000132423	1	0	2	7
ENSG00000132424	120	302	484	526
ENSG00000132429	0	0	0	0
ENSG00000132432	337	588	236	222
ENSG00000132434	6	0	22	56
ENSG00000132436	3	36	44	37
ENSG00000132437	0	0	0	0
ENSG00000132446	0	0	0	0
ENSG00000132463	175	1059	1491	699
ENSG00000132464	3	18	27	7
ENSG00000132465	23	21	4	2
ENSG00000132466	66	992	1724	1913
ENSG00000132467	16	29	5	15
ENSG00000132470	0	0	0	0
ENSG00000132471	1342	3435	2172	2925
ENSG00000132475	4996	7279	5578	5878
ENSG00000132478	52	458	382	597
ENSG00000132481	0	0	0	0
ENSG00000132485	92	59	60	110
ENSG00000132498	0	0	1	0

ENSG00000132507	107	287	110	286
ENSG00000132510	15	74	35	35
ENSG00000132514	3	0	0	0
ENSG00000132517	0	0	0	0
ENSG00000132518	0	0	0	0
ENSG00000132522	0	1	2	9
ENSG00000132530	55	23	16	11
ENSG00000132535	5	158	230	448
ENSG00000132541	5	6	18	34
ENSG00000132549	40	4030	4117	4706
ENSG00000132554	0	0	10	11
ENSG00000132561	0	0	0	3
ENSG00000132563	0	0	7	9
ENSG00000132570	17	71	48	91
ENSG00000132581	73	145	139	155
ENSG00000132589	171	164	144	266
ENSG00000132591	8	0	7	6
ENSG00000132600	3	8	7	6
ENSG00000132603	28	19	14	25
ENSG00000132604	32	1909	1080	2364
ENSG00000132612	35	15	18	77
ENSG00000132613	3	218	17	25
ENSG00000132622	1	0	0	0
ENSG00000132623	6	49	15	12
ENSG00000132631	0	0	0	0
ENSG00000132635	8	0	0	0
ENSG00000132639	11	12	3	8
ENSG00000132640	25	324	551	386
ENSG00000132646	14	0	24	43
ENSG00000132661	6	0	0	0
ENSG00000132664	6	37	56	105
ENSG00000132669	25	192	81	79
ENSG00000132670	650	32675	17928	33915
ENSG00000132671	0	0	0	0
ENSG00000132676	52	1346	945	1245
ENSG00000132677	1	0	0	0
ENSG00000132680	4	367	202	430
ENSG00000132681	1	0	0	1
ENSG00000132688	1	0	0	0
ENSG00000132692	0	0	0	0
ENSG00000132693	0	0	0	0
ENSG00000132694	0	0	1	0
ENSG00000132698	0	0	0	0
ENSG00000132702	0	0	0	0
ENSG00000132703	0	0	0	0
ENSG00000132704	0	0	0	0

ENSG00000132716	36	45	39	28
ENSG00000132718	18	318	182	222
ENSG00000132740	0	0	3	0
ENSG00000132744	4	0	0	0
ENSG00000132746	0	0	0	0
ENSG00000132749	0	15	5	17
ENSG00000132763	0	0	0	3
ENSG00000132768	1	0	0	2
ENSG00000132773	9	9	15	13
ENSG00000132780	71	160	84	169
ENSG00000132781	4	10	14	13
ENSG00000132792	45	66	86	87
ENSG00000132793	0	0	0	0
ENSG00000132801	2	5	9	2
ENSG00000132819	96	2098	1037	918
ENSG00000132821	0	0	0	0
ENSG00000132823	89	109	199	68
ENSG00000132824	1831	9888	9892	6937
ENSG00000132825	12	138	85	57
ENSG00000132832	1	0	0	0
ENSG00000132837	0	0	3	1
ENSG00000132840	0	0	0	1
ENSG00000132842	73	1870	1514	2058
ENSG00000132846	0	0	2	5
ENSG00000132849	5	37	31	20
ENSG00000132854	0	0	0	0
ENSG00000132855	0	0	0	0
ENSG00000132872	0	0	0	0
ENSG00000132874	0	0	0	0
ENSG00000132879	3	0	1	0
ENSG00000132881	0	0	0	0
ENSG00000132906	59	78	83	176
ENSG00000132911	0	0	0	0
ENSG00000132912	151	3307	2948	3153
ENSG00000132915	0	0	1	2
ENSG00000132932	0	9	2	2
ENSG00000132938	0	0	0	0
ENSG00000132950	114	1705	2148	2533
ENSG00000132952	5	112	148	184
ENSG00000132953	5	346	219	389
ENSG00000132958	0	0	9	13
ENSG00000132963	507	801	712	617
ENSG00000132964	2	1878	1444	2820
ENSG00000132965	29	0	1	2
ENSG00000132967	22	210	258	273
ENSG00000132970	273	3060	2353	4932

ENSG00000132972	0	0	131	0
ENSG00000132975	1	0	0	0
ENSG00000133019	3	0	26	67
ENSG00000133020	0	0	0	0
ENSG00000133026	3	30	46	40
ENSG00000133027	0	0	0	0
ENSG00000133028	1	2	55	16
ENSG00000133030	49	228	89	246
ENSG00000133048	0	0	0	6
ENSG00000133055	0	0	0	0
ENSG00000133056	1	0	0	0
ENSG00000133059	42	117	102	181
ENSG00000133063	0	0	1	0
ENSG00000133065	2	0	0	0
ENSG00000133067	1	0	0	0
ENSG00000133069	370	1786	973	686
ENSG00000133083	0	0	0	0
ENSG00000133101	0	0	0	0
ENSG00000133103	17	2265	1698	2908
ENSG00000133104	161	1235	1549	1044
ENSG00000133105	0	0	0	0
ENSG00000133106	99	3350	2307	4632
ENSG00000133107	0	0	0	0
ENSG00000133110	0	0	0	0
ENSG00000133111	2	0	6	8
ENSG00000133112	7247	18775	12909	31029
ENSG00000133114	9	499	554	643
ENSG00000133115	0	0	0	0
ENSG00000133116	3	0	0	0
ENSG00000133119	6	11	15	2
ENSG00000133121	0	0	10	10
ENSG00000133124	0	0	0	0
ENSG00000133131	3	0	4	7
ENSG00000133134	8	15	2	0
ENSG00000133135	0	0	4	0
ENSG00000133136	0	0	2	0
ENSG00000133138	17	430	475	476
ENSG00000133142	236	24	109	153
ENSG00000133149	0	0	0	0
ENSG00000133169	35	58	3	42
ENSG00000133193	30	1058	925	1027
ENSG00000133195	1	43	6	16
ENSG00000133216	1	0	0	0
ENSG00000133226	340	402	323	389
ENSG00000133243	35	393	212	477
ENSG00000133246	6	0	1	0

ENSG00000133247	1	0	1	3
ENSG00000133250	0	22	0	0
ENSG00000133256	22	0	10	23
ENSG00000133265	14	0	0	0
ENSG00000133275	12	135	80	142
ENSG00000133302	427	3845	3717	4837
ENSG00000133313	108	25	34	72
ENSG00000133315	0	0	3	2
ENSG00000133316	3	2	0	5
ENSG00000133317	803	1963	1163	769
ENSG00000133318	1734	12383	10455	10493
ENSG00000133321	345	165	173	114
ENSG00000133328	7	18	1	12
ENSG00000133392	0	15	4	1
ENSG00000133393	235	1947	1996	1814
ENSG00000133398	59	27	47	40
ENSG00000133401	25	125	127	146
ENSG00000133422	4	24	13	45
ENSG00000133424	0	0	2	2
ENSG00000133433	0	0	0	0
ENSG00000133454	16	39	12	14
ENSG00000133460	39	3	35	23
ENSG00000133466	0	14	4	13
ENSG00000133475	0	0	0	0
ENSG00000133477	0	0	0	0
ENSG00000133488	0	0	0	0
ENSG00000133519	0	0	0	0
ENSG00000133561	31	2	5	5
ENSG00000133574	213	40	16	7
ENSG00000133597	5	35	0	1
ENSG00000133606	2507	13387	13321	10426
ENSG00000133612	3	0	7	21
ENSG00000133619	0	0	0	0
ENSG00000133624	1	318	155	403
ENSG00000133627	279	3483	2573	3111
ENSG00000133636	0	16	0	0
ENSG00000133639	215	202	210	41
ENSG00000133640	0	0	0	0
ENSG00000133641	4	53	69	58
ENSG00000133657	8	688	524	532
ENSG00000133661	0	0	0	0
ENSG00000133665	0	0	0	0
ENSG00000133678	4	1	22	13
ENSG00000133687	3	54	133	30
ENSG00000133703	102	755	800	706
ENSG00000133704	11	591	564	731

ENSG00000133706	69	405	461	704
ENSG00000133710	0	0	0	0
ENSG00000133731	18	106	232	203
ENSG00000133739	212	955	1055	1270
ENSG00000133740	1	21	5	27
ENSG00000133742	31	87	36	110
ENSG00000133773	55	127	27	38
ENSG00000133789	64	143	44	49
ENSG00000133794	7	1	25	22
ENSG00000133800	0	0	8	5
ENSG00000133805	4	9	12	13
ENSG00000133808	0	0	2	0
ENSG00000133812	6	27	17	31
ENSG00000133816	3	12	2	14
ENSG00000133818	13	74	54	108
ENSG00000133835	25	420	354	515
ENSG00000133858	95	2019	1909	2564
ENSG00000133863	5	139	109	90
ENSG00000133872	864	2771	2837	3352
ENSG00000133874	9	34	22	41
ENSG00000133878	0	0	0	1
ENSG00000133884	30	36	15	3
ENSG00000133895	7	0	2	0
ENSG00000133935	2	0	4	5
ENSG00000133937	0	0	0	0
ENSG00000133943	5	60	27	45
ENSG00000133958	0	0	4	12
ENSG00000133961	97	1439	1292	1832
ENSG00000133962	11	1474	1290	3683
ENSG00000133980	0	0	0	0
ENSG00000133983	24	1	6	8
ENSG00000133985	11	0	4	14
ENSG00000133997	12	54	57	86
ENSG00000134001	49	74	37	56
ENSG00000134007	0	0	0	0
ENSG00000134013	1	0	0	0
ENSG00000134014	9	499	206	551
ENSG00000134020	2	0	0	0
ENSG00000134028	0	0	0	0
ENSG00000134030	34	615	244	463
ENSG00000134042	0	0	17	44
ENSG00000134046	132	828	974	1048
ENSG00000134049	8	0	26	50
ENSG00000134056	61	85	104	173
ENSG00000134057	1	3352	2945	4812
ENSG00000134058	7	396	396	454

ENSG00000134061	2	0	0	0
ENSG00000134070	34	580	410	239
ENSG00000134072	0	43	40	71
ENSG00000134077	29	156	122	264
ENSG00000134086	182	446	402	232
ENSG00000134107	5	0	2	6
ENSG00000134108	731	6173	9683	5567
ENSG00000134109	33	366	337	339
ENSG00000134115	0	0	0	0
ENSG00000134121	0	0	2	3
ENSG00000134138	0	0	0	0
ENSG00000134146	8	91	142	311
ENSG00000134152	16	365	444	514
ENSG00000134153	12	46	56	97
ENSG00000134160	6	0	19	17
ENSG00000134183	1	0	0	0
ENSG00000134184	5	0	21	0
ENSG00000134186	99	52	49	42
ENSG00000134193	0	0	0	0
ENSG00000134198	277	3821	806	2884
ENSG00000134200	0	0	0	0
ENSG00000134201	101	0	9	301
ENSG00000134202	35	263	260	122
ENSG00000134207	0	0	0	3
ENSG00000134215	67	3538	4818	5137
ENSG00000134216	0	0	0	0
ENSG00000134222	43	557	74	388
ENSG00000134240	0	0	0	0
ENSG00000134242	3	1305	1446	1528
ENSG00000134243	101	2944	2205	3991
ENSG00000134245	0	0	2	4
ENSG00000134247	0	6	0	0
ENSG00000134248	169	58	33	74
ENSG00000134249	0	0	0	0
ENSG00000134250	7	72	166	198
ENSG00000134253	0	29	0	0
ENSG00000134255	22	306	214	267
ENSG00000134256	0	0	0	0
ENSG00000134258	0	0	0	0
ENSG00000134259	0	0	0	0
ENSG00000134262	3	18	17	51
ENSG00000134265	282	1842	2487	1825
ENSG00000134278	41	514	741	506
ENSG00000134283	85	6886	5377	5650
ENSG00000134285	2	0	10	7
ENSG00000134287	1195	4513	4184	5289

ENSG00000134291	967	3956	4653	7916
ENSG00000134294	145	4250	5052	3365
ENSG00000134297	525	1539	2206	1419
ENSG00000134308	897	7091	6456	7419
ENSG00000134313	237	3750	4286	3943
ENSG00000134317	935	3621	3364	6454
ENSG00000134318	1601	9954	12923	12443
ENSG00000134321	22	89	202	93
ENSG00000134323	3	0	8	8
ENSG00000134324	13	109	60	95
ENSG00000134326	13	39	88	5
ENSG00000134330	9	44	33	40
ENSG00000134333	85	537	393	560
ENSG00000134339	0	0	0	0
ENSG00000134343	0	0	0	0
ENSG00000134352	660	16672	24943	13924
ENSG00000134363	0	0	0	0
ENSG00000134365	0	0	0	0
ENSG00000134369	5	0	0	2
ENSG00000134371	61	2514	2137	3572
ENSG00000134375	21	0	5	4
ENSG00000134376	0	0	0	0
ENSG00000134389	0	0	0	0
ENSG00000134398	0	0	0	0
ENSG00000134419	498	128	112	134
ENSG00000134438	0	0	0	0
ENSG00000134440	112	649	351	455
ENSG00000134443	0	0	0	0
ENSG00000134444	2	2457	2058	2551
ENSG00000134452	98	322	356	274
ENSG00000134453	111	139	128	93
ENSG00000134460	1	0	0	0
ENSG00000134461	2	0	1	0
ENSG00000134463	0	0	1	1
ENSG00000134470	0	80	36	42
ENSG00000134480	26	374	354	378
ENSG00000134489	0	0	0	9
ENSG00000134490	14	35	63	50
ENSG00000134504	4	0	2	4
ENSG00000134508	42	80	27	44
ENSG00000134516	26	198	74	120
ENSG00000134531	0	0	0	3
ENSG00000134532	1	0	0	0
ENSG00000134533	2	0	2	1
ENSG00000134538	0	0	1	4
ENSG00000134539	59	8	2	0

ENSG00000134545	4	0	0	0
ENSG00000134548	694	4920	9956	11162
ENSG00000134551	0	0	0	0
ENSG00000134569	0	0	1	1
ENSG00000134571	0	0	0	0
ENSG00000134574	10	37	28	24
ENSG00000134575	45	36	30	127
ENSG00000134588	0	0	0	0
ENSG00000134590	133	58	58	85
ENSG00000134594	34	0	44	65
ENSG00000134595	0	0	0	0
ENSG00000134597	33	0	10	13
ENSG00000134602	323	4503	6903	4720
ENSG00000134612	0	0	0	0
ENSG00000134627	3	108	72	63
ENSG00000134640	0	0	0	0
ENSG00000134644	67	321	271	402
ENSG00000134668	801	907	509	364
ENSG00000134684	54	170	75	145
ENSG00000134686	25	85	45	50
ENSG00000134690	2	12	17	35
ENSG00000134697	22	249	243	334
ENSG00000134698	14	72	84	82
ENSG00000134709	2	53	17	12
ENSG00000134716	0	0	0	0
ENSG00000134717	18	149	134	104
ENSG00000134744	35	3271	3764	5553
ENSG00000134748	11	81	33	48
ENSG00000134755	0	0	0	1
ENSG00000134757	1	0	0	0
ENSG00000134758	65	1309	1260	1153
ENSG00000134759	7	34	112	92
ENSG00000134760	0	0	0	0
ENSG00000134762	0	0	0	0
ENSG00000134765	0	0	0	0
ENSG00000134769	2	49	8	4
ENSG00000134775	0	0	0	0
ENSG00000134779	322	581	904	1142
ENSG00000134780	0	0	0	0
ENSG00000134802	1	1	5	17
ENSG00000134809	41	22	1	0
ENSG00000134812	0	0	1	0
ENSG00000134815	2	0	4	20
ENSG00000134817	0	0	0	1
ENSG00000134824	29	536	201	324
ENSG00000134825	17	0	3	5

ENSG00000134827	0	0	0	3
ENSG00000134830	0	0	0	0
ENSG00000134851	42	14545	14473	18556
ENSG00000134852	12	330	288	384
ENSG00000134853	155	223	337	246
ENSG00000134864	2	0	4	32
ENSG00000134871	10	0	18	191
ENSG00000134873	0	0	1	4
ENSG00000134874	0	0	8	2
ENSG00000134882	688	7435	4975	5465
ENSG00000134884	37	170	83	95
ENSG00000134897	111	321	319	441
ENSG00000134899	31	25	45	76
ENSG00000134900	509	6296	5733	5599
ENSG00000134901	0	0	10	9
ENSG00000134905	17	57	65	65
ENSG00000134909	364	4120	3321	3777
ENSG00000134910	12	231	230	175
ENSG00000134917	0	0	0	0
ENSG00000134940	0	0	0	0
ENSG00000134954	99	155	91	67
ENSG00000134955	0	0	0	0
ENSG00000134962	0	0	1	0
ENSG00000134970	19	619	877	1456
ENSG00000134982	28	1758	1811	2686
ENSG00000134986	131	704	1819	1525
ENSG00000134987	8	53	52	52
ENSG00000134996	502	4608	2956	4643
ENSG00000135002	19	20	18	12
ENSG00000135018	76	19608	15707	31349
ENSG00000135040	15	598	798	1051
ENSG00000135045	17	77	236	192
ENSG00000135046	79	109	30	54
ENSG00000135047	0	11	0	0
ENSG00000135048	7	52	34	24
ENSG00000135049	82	4402	4375	3976
ENSG00000135052	3	13	3	14
ENSG00000135063	0	0	0	0
ENSG00000135069	2	0	0	0
ENSG00000135070	565	5805	6354	6953
ENSG00000135074	2	14	2	0
ENSG00000135077	6	1	20	11
ENSG00000135083	152	263	304	409
ENSG00000135090	2524	7733	7603	8329
ENSG00000135093	2	124	50	68
ENSG00000135094	0	0	0	0

ENSG00000135097	0	0	0	0
ENSG00000135100	0	0	0	0
ENSG00000135108	8	0	2	9
ENSG00000135111	1	20	0	0
ENSG00000135114	11	0	0	4
ENSG00000135116	0	0	0	0
ENSG00000135119	0	10	2	5
ENSG00000135124	6	5	20	40
ENSG00000135127	0	0	1	1
ENSG00000135144	4	0	0	0
ENSG00000135148	212	929	963	661
ENSG00000135164	34	248	509	501
ENSG00000135175	0	0	0	0
ENSG00000135185	1	3	5	0
ENSG00000135205	28	277	376	604
ENSG00000135211	154	441	441	247
ENSG00000135213	11	14	10	8
ENSG00000135218	709	15832	22599	19294
ENSG00000135220	0	0	0	0
ENSG00000135222	0	0	0	0
ENSG00000135226	85	16	25	25
ENSG00000135241	64	218	216	204
ENSG00000135245	0	0	0	0
ENSG00000135248	9	8	13	6
ENSG00000135249	0	377	326	422
ENSG00000135250	83	260	319	288
ENSG00000135253	1	25	10	4
ENSG00000135269	110	0	10	0
ENSG00000135272	36	5	23	13
ENSG00000135297	8	328	263	479
ENSG00000135298	0	0	0	0
ENSG00000135299	3	71	64	35
ENSG00000135312	0	0	0	0
ENSG00000135314	0	0	0	0
ENSG00000135315	276	8798	11180	9844
ENSG00000135316	171	1301	1257	2166
ENSG00000135317	4	2021	2144	3029
ENSG00000135318	0	0	0	0
ENSG00000135324	4	97	78	20
ENSG00000135333	0	0	0	0
ENSG00000135334	762	3030	3700	1926
ENSG00000135336	38	475	453	604
ENSG00000135338	60	1163	1388	2314
ENSG00000135341	27	359	347	406
ENSG00000135346	0	0	0	0
ENSG00000135355	0	0	0	0

ENSG00000135362	13	791	423	749
ENSG00000135363	338	922	1214	1204
ENSG00000135365	36	1957	4491	4206
ENSG00000135372	16	9	23	41
ENSG00000135373	0	0	0	0
ENSG00000135374	0	0	0	0
ENSG00000135378	2	0	0	0
ENSG00000135387	62	3137	2604	4515
ENSG00000135390	471	146	119	290
ENSG00000135392	2	19	10	18
ENSG00000135404	1830	1351	955	974
ENSG00000135406	0	0	0	0
ENSG00000135407	3	0	2	5
ENSG00000135409	3	0	0	0
ENSG00000135413	0	0	0	0
ENSG00000135414	2	0	4	0
ENSG00000135423	1	0	2	0
ENSG00000135424	0	0	0	3
ENSG00000135426	49	52	1197	151
ENSG00000135436	0	0	0	0
ENSG00000135437	0	0	0	0
ENSG00000135439	6	0	8	26
ENSG00000135441	224	68	19	14
ENSG00000135443	0	0	0	0
ENSG00000135446	1	6	5	28
ENSG00000135447	0	0	0	0
ENSG00000135451	1	0	1	7
ENSG00000135452	7	56	14	51
ENSG00000135454	0	0	0	0
ENSG00000135457	10	244	491	643
ENSG00000135469	0	0	0	0
ENSG00000135472	0	0	0	0
ENSG00000135473	1	0	4	2
ENSG00000135476	0	0	4	2
ENSG00000135477	0	0	2	0
ENSG00000135480	0	0	0	0
ENSG00000135482	2	0	4	0
ENSG00000135486	366	392	554	698
ENSG00000135502	0	0	0	0
ENSG00000135503	12	31	36	13
ENSG00000135506	16	258	187	301
ENSG00000135517	0	0	0	0
ENSG00000135519	0	0	0	0
ENSG00000135521	10	0	28	74
ENSG00000135525	1	38	36	56
ENSG00000135535	667	2934	7470	12409

ENSG00000135537	1	466	343	493
ENSG00000135540	1	11	5	3
ENSG00000135541	7	279	25	312
ENSG00000135547	1	0	4	0
ENSG00000135549	4	0	1	7
ENSG00000135569	0	0	0	0
ENSG00000135577	0	0	0	0
ENSG00000135587	1	0	0	0
ENSG00000135596	6	0	6	19
ENSG00000135597	17	2477	2249	2269
ENSG00000135604	115	676	714	840
ENSG00000135605	51	5637	3723	4824
ENSG00000135617	10	0	1	0
ENSG00000135622	1	0	2	3
ENSG00000135624	72	49	18	35
ENSG00000135625	0	0	0	0
ENSG00000135631	0	0	0	0
ENSG00000135632	4	6	0	0
ENSG00000135636	2	2	12	1
ENSG00000135637	3	0	1	2
ENSG00000135638	0	0	0	0
ENSG00000135643	17	51	59	160
ENSG00000135655	174	4260	5039	4338
ENSG00000135677	14	172	279	229
ENSG00000135678	15	0	37	24
ENSG00000135679	91	2739	2932	3088
ENSG00000135686	12	18	16	37
ENSG00000135697	1	0	0	5
ENSG00000135698	9	0	17	30
ENSG00000135702	0	0	0	0
ENSG00000135709	556	3977	2507	2666
ENSG00000135720	18	130	104	94
ENSG00000135722	0	2	0	0
ENSG00000135723	93	105	81	192
ENSG00000135736	2	0	0	0
ENSG00000135740	0	0	4	0
ENSG00000135744	0	0	0	0
ENSG00000135747	1	0	0	0
ENSG00000135749	0	0	29	18
ENSG00000135750	0	0	0	0
ENSG00000135763	1	0	1	2
ENSG00000135766	12	49	30	47
ENSG00000135773	0	0	0	1
ENSG00000135775	12	360	354	484
ENSG00000135776	32	191	286	260
ENSG00000135778	9	0	2	11

ENSG00000135801	8	14	65	76
ENSG00000135821	1366	8902	12358	5900
ENSG00000135823	65	1132	925	878
ENSG00000135824	0	0	0	0
ENSG00000135828	21	56	66	97
ENSG00000135829	99	911	1013	1265
ENSG00000135835	0	0	0	0
ENSG00000135837	175	7155	7059	11249
ENSG00000135838	123	684	472	769
ENSG00000135842	114	111	430	284
ENSG00000135845	30	100	39	30
ENSG00000135847	15	175	405	540
ENSG00000135862	18	167	1478	496
ENSG00000135870	102	1480	1558	1204
ENSG00000135898	637	50	298	155
ENSG00000135899	222	82	17	21
ENSG00000135900	7	48	18	32
ENSG00000135902	0	0	0	0
ENSG00000135903	0	0	0	0
ENSG00000135905	30	1783	1175	1783
ENSG00000135912	0	20	7	12
ENSG00000135913	36	1741	1095	1823
ENSG00000135914	0	0	0	0
ENSG00000135916	7	1	2	1
ENSG00000135917	0	0	0	0
ENSG00000135919	138	1284	930	423
ENSG00000135924	145	903	496	828
ENSG00000135925	0	0	0	0
ENSG00000135926	2494	8235	7408	7467
ENSG00000135929	0	0	0	2
ENSG00000135930	60	99	40	58
ENSG00000135931	0	0	0	2
ENSG00000135932	464	7006	6882	5536
ENSG00000135940	282	46	47	117
ENSG00000135945	24	761	751	976
ENSG00000135951	15	107	381	318
ENSG00000135953	5	88	30	20
ENSG00000135956	115	330	186	271
ENSG00000135960	0	0	0	9
ENSG00000135966	26	0	38	39
ENSG00000135968	212	791	1061	1455
ENSG00000135972	10	13	80	75
ENSG00000135973	0	0	0	0
ENSG00000135974	37	196	140	167
ENSG00000135976	20	998	1287	1581
ENSG00000135999	12	356	288	313

ENSG00000136002	0	10	0	0
ENSG00000136003	808	4191	3240	3421
ENSG00000136010	1	1	13	23
ENSG00000136011	1	0	3	1
ENSG00000136014	1	0	6	1
ENSG00000136021	344	10525	11840	12044
ENSG00000136026	4	0	0	4
ENSG00000136040	5	230	309	273
ENSG00000136044	26	1186	480	1259
ENSG00000136045	44	0	34	40
ENSG00000136048	49	214	193	214
ENSG00000136051	57	1150	1030	1520
ENSG00000136052	2	51	33	28
ENSG00000136059	1	43	0	0
ENSG00000136068	1	22	7	17
ENSG00000136098	3	86	62	152
ENSG00000136099	0	0	1	0
ENSG00000136100	80	1581	1468	2192
ENSG00000136104	39	4452	3500	5450
ENSG00000136108	62	1060	1034	964
ENSG00000136110	0	0	0	0
ENSG00000136111	18	30	29	31
ENSG00000136114	0	0	5	6
ENSG00000136122	2	66	53	60
ENSG00000136141	8	664	729	1372
ENSG00000136143	3	27	70	100
ENSG00000136144	7	0	20	9
ENSG00000136146	127	1049	772	704
ENSG00000136147	33	43	43	91
ENSG00000136149	10	0	0	2
ENSG00000136152	5	719	1285	1097
ENSG00000136153	0	17	27	28
ENSG00000136155	0	0	0	0
ENSG00000136156	18683	65070	78706	66601
ENSG00000136158	4	46	26	14
ENSG00000136159	3	0	9	0
ENSG00000136160	0	0	1	0
ENSG00000136161	33	45	42	17
ENSG00000136167	755	328	123	77
ENSG00000136169	8	4341	3118	4183
ENSG00000136193	37	30	39	83
ENSG00000136197	5	0	1	3
ENSG00000136205	465	800	878	356
ENSG00000136206	0	0	0	0
ENSG00000136213	52	59	52	66
ENSG00000136231	612	19384	13132	25263

ENSG00000136235	0	0	0	2
ENSG00000136237	11	136	40	44
ENSG00000136238	5154	4360	3238	5042
ENSG00000136240	110	532	1068	1146
ENSG00000136243	5	291	320	907
ENSG00000136244	0	0	0	0
ENSG00000136247	101	567	588	523
ENSG00000136250	17	210	90	145
ENSG00000136261	37	278	75	237
ENSG00000136267	3	0	1	15
ENSG00000136270	6	0	0	0
ENSG00000136271	10	0	4	8
ENSG00000136273	12	96	135	143
ENSG00000136274	0	0	0	0
ENSG00000136275	0	0	0	0
ENSG00000136279	1215	2231	1127	2831
ENSG00000136280	37	249	250	273
ENSG00000136286	6	6	2	6
ENSG00000136295	42	613	223	649
ENSG00000136297	0	0	0	0
ENSG00000136305	0	54	45	15
ENSG00000136315	2	0	0	0
ENSG00000136319	36	233	417	241
ENSG00000136327	0	0	0	0
ENSG00000136352	0	0	0	0
ENSG00000136367	0	0	0	0
ENSG00000136371	12	0	7	10
ENSG00000136378	0	0	0	1
ENSG00000136379	1	0	6	10
ENSG00000136381	36	836	934	1042
ENSG00000136383	0	0	3	0
ENSG00000136404	639	6023	5311	2911
ENSG00000136425	3	0	0	2
ENSG00000136436	104	565	328	591
ENSG00000136444	6	0	6	1
ENSG00000136448	77	786	390	615
ENSG00000136449	2	15	11	8
ENSG00000136450	51	476	260	374
ENSG00000136451	198	409	664	518
ENSG00000136457	0	0	0	0
ENSG00000136463	7	0	18	22
ENSG00000136478	59	829	651	1087
ENSG00000136485	178	200	190	256
ENSG00000136487	0	0	0	0
ENSG00000136488	0	0	0	0
ENSG00000136490	19	4	26	9

ENSG00000136492	1	1259	1282	1643
ENSG00000136504	9	271	191	390
ENSG00000136514	4	0	8	3
ENSG00000136518	7	37	13	30
ENSG00000136521	35	144	63	104
ENSG00000136522	28	463	530	788
ENSG00000136527	588	1193	1307	2014
ENSG00000136531	1	2	26	24
ENSG00000136535	0	0	0	0
ENSG00000136536	47	1510	1574	1634
ENSG00000136541	0	0	0	0
ENSG00000136542	0	0	2	1
ENSG00000136546	0	9	0	0
ENSG00000136560	62	914	1067	1518
ENSG00000136573	0	0	0	0
ENSG00000136574	0	0	0	0
ENSG00000136603	44	485	529	250
ENSG00000136628	75	402	629	985
ENSG00000136630	3	28	0	0
ENSG00000136631	35	329	305	323
ENSG00000136634	1	0	0	0
ENSG00000136636	0	245	151	375
ENSG00000136643	22	3139	2078	2708
ENSG00000136653	84	0	2	1
ENSG00000136682	11	312	164	317
ENSG00000136688	0	0	0	0
ENSG00000136689	8	0	0	0
ENSG00000136694	0	0	0	0
ENSG00000136695	0	32	0	0
ENSG00000136696	0	0	2	0
ENSG00000136697	0	0	0	0
ENSG00000136698	0	0	0	0
ENSG00000136699	4	0	1	3
ENSG00000136709	25	1645	1610	1819
ENSG00000136710	42	34	21	52
ENSG00000136715	4	146	88	216
ENSG00000136717	11	0	5	0
ENSG00000136718	26	0	7	13
ENSG00000136720	1	0	2	1
ENSG00000136731	126	3864	4035	4875
ENSG00000136732	27	14	19	32
ENSG00000136738	106	1017	1377	1549
ENSG00000136750	5	18	0	3
ENSG00000136754	945	15833	14148	14711
ENSG00000136758	274	2718	2467	3208
ENSG00000136770	164	2002	1742	2280

ENSG00000136783	38	1715	643	680
ENSG00000136802	5	0	19	18
ENSG00000136805	0	0	0	0
ENSG00000136807	48	104	58	75
ENSG00000136810	563	1136	766	955
ENSG00000136811	55	458	469	841
ENSG00000136813	66	5630	6778	9994
ENSG00000136816	7	47	34	61
ENSG00000136819	583	320	273	457
ENSG00000136824	47	204	205	352
ENSG00000136826	9	0	0	0
ENSG00000136827	23	113	89	73
ENSG00000136828	2	14	28	59
ENSG00000136830	0	11	23	15
ENSG00000136834	0	0	0	0
ENSG00000136839	0	0	0	0
ENSG00000136840	9	11	3	5
ENSG00000136842	306	493	840	791
ENSG00000136848	0	1	0	1
ENSG00000136854	15	181	43	167
ENSG00000136856	66	0	26	32
ENSG00000136859	0	0	0	0
ENSG00000136861	880	1333	1796	1362
ENSG00000136866	0	0	0	2
ENSG00000136867	3	20	16	6
ENSG00000136868	9	4	38	31
ENSG00000136869	4	0	3	6
ENSG00000136870	8	16	75	92
ENSG00000136872	0	0	0	2
ENSG00000136874	85	624	934	1119
ENSG00000136875	34	65	40	51
ENSG00000136877	14	0	16	4
ENSG00000136878	107	305	139	461
ENSG00000136881	0	0	0	0
ENSG00000136883	0	0	0	0
ENSG00000136888	1239	1360	1229	1836
ENSG00000136891	18	363	613	472
ENSG00000136895	0	0	8	4
ENSG00000136897	9	43	29	19
ENSG00000136908	47	73	33	73
ENSG00000136918	0	0	0	0
ENSG00000136925	18	122	179	136
ENSG00000136928	0	0	0	1
ENSG00000136929	1797	6321	23808	14654
ENSG00000136930	103	22	9	39
ENSG00000136931	0	0	0	0

ENSG00000136932	3	32	11	12
ENSG00000136933	11	65	21	59
ENSG00000136935	4	552	764	1473
ENSG00000136936	17	25	42	30
ENSG00000136937	14	238	194	299
ENSG00000136938	2189	2696	2475	4588
ENSG00000136939	0	0	0	0
ENSG00000136940	30	288	365	261
ENSG00000136942	925	156	32	56
ENSG00000136943	0	0	0	0
ENSG00000136944	0	0	0	0
ENSG00000136950	61	0	3	1
ENSG00000136960	0	0	0	8
ENSG00000136982	0	239	134	234
ENSG00000136986	46	482	528	730
ENSG00000136997	3	0	1	2
ENSG00000136999	0	0	0	0
ENSG00000137033	1	11	0	4
ENSG00000137038	12	7	2	4
ENSG00000137040	13	159	57	80
ENSG00000137054	11	19	7	5
ENSG00000137055	14	257	243	287
ENSG00000137070	0	0	0	0
ENSG00000137073	240	15129	13313	22550
ENSG00000137074	8	7	19	18
ENSG00000137075	160	2037	2135	2206
ENSG00000137076	17328	61389	55721	90527
ENSG00000137077	1	0	0	0
ENSG00000137078	1	0	0	0
ENSG00000137080	0	0	0	0
ENSG00000137090	0	0	0	0
ENSG00000137094	1	0	0	0
ENSG00000137098	2	6	7	4
ENSG00000137100	56	44	19	42
ENSG00000137101	3	11	10	0
ENSG00000137103	1	0	0	0
ENSG00000137106	22	124	124	247
ENSG00000137124	4	0	1	2
ENSG00000137133	38	28	15	30
ENSG00000137135	5	0	4	0
ENSG00000137142	0	0	0	0
ENSG00000137145	520	12032	14296	9746
ENSG00000137154	2250	392	173	324
ENSG00000137161	691	917	885	1224
ENSG00000137166	0	0	0	0
ENSG00000137168	7	7	0	0

ENSG00000137171	4	0	0	0
ENSG00000137177	18	400	278	490
ENSG00000137185	0	12	2	3
ENSG00000137193	30	100	116	12
ENSG00000137197	0	0	0	0
ENSG00000137198	1724	3349	2611	3136
ENSG00000137200	4	33	1	7
ENSG00000137203	0	0	0	1
ENSG00000137204	0	0	0	0
ENSG00000137207	315	393	345	342
ENSG00000137210	79	0	48	106
ENSG00000137216	10	46	66	105
ENSG00000137218	1	0	2	0
ENSG00000137221	8	10	11	20
ENSG00000137225	23	110	101	245
ENSG00000137251	0	0	0	0
ENSG00000137252	0	0	0	0
ENSG00000137259	1	17	4	0
ENSG00000137261	0	0	0	0
ENSG00000137265	4	0	1	0
ENSG00000137266	112	961	413	1161
ENSG00000137267	0	0	1	3
ENSG00000137269	2	0	17	23
ENSG00000137270	0	0	0	0
ENSG00000137273	0	0	0	0
ENSG00000137274	1	20	0	0
ENSG00000137275	23	243	142	239
ENSG00000137285	0	0	0	0
ENSG00000137288	20	13	2	26
ENSG00000137309	58	7	18	20
ENSG00000137310	1	195	130	152
ENSG00000137312	122	453	297	311
ENSG00000137313	0	0	0	0
ENSG00000137321	0	0	0	0
ENSG00000137331	15	0	1	0
ENSG00000137332	0	0	0	0
ENSG00000137337	8	22	13	26
ENSG00000137338	0	4	4	3
ENSG00000137343	0	7	2	4
ENSG00000137345	0	0	0	0
ENSG00000137364	24	48	91	192
ENSG00000137384	0	0	0	0
ENSG00000137392	0	0	0	0
ENSG00000137393	9	22	10	23
ENSG00000137394	0	0	0	0
ENSG00000137397	0	0	0	0

ENSG00000137403	0	0	0	0
ENSG00000137404	42	0	14	20
ENSG00000137409	47	78	50	133
ENSG00000137411	2	1	8	11
ENSG00000137413	28	36	49	42
ENSG00000137414	55	541	761	601
ENSG00000137434	0	0	0	0
ENSG00000137440	0	0	0	0
ENSG00000137441	29	0	1	0
ENSG00000137449	55	615	1000	567
ENSG00000137460	0	0	4	7
ENSG00000137462	1	0	17	7
ENSG00000137463	3	25	12	1
ENSG00000137473	0	0	0	0
ENSG00000137474	0	0	0	0
ENSG00000137478	24	3973	3500	5214
ENSG00000137486	845	1598	924	1031
ENSG00000137491	0	0	0	0
ENSG00000137492	133	1200	1074	1523
ENSG00000137494	6	248	184	223
ENSG00000137496	5	0	10	0
ENSG00000137497	7	253	57	164
ENSG00000137500	38	1311	1716	2695
ENSG00000137501	6	0	15	1
ENSG00000137502	436	2378	3939	2180
ENSG00000137504	17	147	151	141
ENSG00000137507	286	92	82	58
ENSG00000137509	12	60	60	94
ENSG00000137513	3	261	227	356
ENSG00000137522	7	93	84	116
ENSG00000137547	17	41	38	15
ENSG00000137558	0	0	0	3
ENSG00000137561	0	0	0	0
ENSG00000137563	3	24	93	120
ENSG00000137571	0	0	0	0
ENSG00000137573	0	0	0	0
ENSG00000137574	46	300	435	499
ENSG00000137575	589	7911	9591	7706
ENSG00000137601	127	6067	6418	7839
ENSG00000137628	19	528	620	660
ENSG00000137634	0	0	0	0
ENSG00000137642	10	0	15	3
ENSG00000137648	0	0	1	0
ENSG00000137656	10	64	45	63
ENSG00000137672	57	1327	1550	1380
ENSG00000137673	0	0	0	0

ENSG00000137674	0	0	4	6
ENSG00000137675	0	0	0	0
ENSG00000137691	0	0	0	0
ENSG00000137692	9	66	75	120
ENSG00000137693	0	2	2	5
ENSG00000137699	0	0	0	0
ENSG00000137700	2	0	0	0
ENSG00000137707	0	0	0	0
ENSG00000137709	0	0	0	0
ENSG00000137710	152	1370	1122	1326
ENSG00000137713	13	339	437	380
ENSG00000137714	18	0	9	11
ENSG00000137720	16	23	9	18
ENSG00000137726	0	20	3	1
ENSG00000137727	0	0	0	8
ENSG00000137731	0	0	0	0
ENSG00000137745	0	0	0	0
ENSG00000137747	34	57	79	64
ENSG00000137752	79	14	27	32
ENSG00000137757	8	0	0	0
ENSG00000137760	10	25	9	9
ENSG00000137764	5	1565	1371	2009
ENSG00000137766	0	0	0	0
ENSG00000137767	63	117	41	43
ENSG00000137770	15	425	738	1044
ENSG00000137776	1820	15775	11845	25431
ENSG00000137801	3419	58927	59151	79046
ENSG00000137802	1	0	2	0
ENSG00000137804	20	779	1313	1589
ENSG00000137806	1	0	1	2
ENSG00000137807	1	409	373	851
ENSG00000137808	0	0	0	0
ENSG00000137809	0	0	0	0
ENSG00000137812	14	2069	1969	2346
ENSG00000137814	12	370	228	506
ENSG00000137815	245	248	277	331
ENSG00000137817	90	266	177	274
ENSG00000137818	5766	725	286	1672
ENSG00000137819	0	0	0	0
ENSG00000137821	0	21	53	52
ENSG00000137822	181	1746	1422	1916
ENSG00000137824	1	44	1	3
ENSG00000137825	0	0	0	0
ENSG00000137831	268	691	795	1135
ENSG00000137834	0	20	0	1
ENSG00000137841	18	10	4	26

ENSG00000137842	2	454	302	497
ENSG00000137843	0	0	0	0
ENSG00000137845	236	7348	8834	9398
ENSG00000137857	11	12	3	11
ENSG00000137860	0	0	0	0
ENSG00000137868	0	0	0	0
ENSG00000137869	9	78	107	42
ENSG00000137871	18	1052	1219	1562
ENSG00000137872	0	0	0	0
ENSG00000137875	0	0	0	0
ENSG00000137876	88	100	35	56
ENSG00000137877	1	0	0	0
ENSG00000137878	10	1584	2142	1279
ENSG00000137880	6	0	0	0
ENSG00000137936	1	0	0	0
ENSG00000137941	130	963	1441	939
ENSG00000137942	246	2454	3264	2438
ENSG00000137944	22	153	215	255
ENSG00000137947	297	4519	3533	3053
ENSG00000137948	0	0	0	0
ENSG00000137955	5	87	67	129
ENSG00000137959	14	49	19	12
ENSG00000137960	0	11	2	3
ENSG00000137962	2	22	14	20
ENSG00000137965	27	181	288	293
ENSG00000137968	0	0	0	0
ENSG00000137970	7	84	14	79
ENSG00000137975	0	0	0	0
ENSG00000137976	0	0	0	2
ENSG00000137992	24	230	239	376
ENSG00000137996	39	283	247	336
ENSG00000138002	1	79	105	143
ENSG00000138018	7	3080	2953	2683
ENSG00000138028	0	0	0	0
ENSG00000138029	35	1323	1028	2046
ENSG00000138030	2	21	4	9
ENSG00000138031	57	1147	428	677
ENSG00000138032	37	2091	1414	1412
ENSG00000138035	8	745	908	1293
ENSG00000138036	6	552	206	241
ENSG00000138039	0	12	2	72
ENSG00000138041	259	5562	5905	5101
ENSG00000138050	51	1055	739	735
ENSG00000138061	7	0	1	0
ENSG00000138068	0	0	0	0
ENSG00000138069	124	4058	3068	4837

ENSG00000138071	1417	8763	10216	10617
ENSG00000138073	32	29	9	8
ENSG00000138074	1	0	0	3
ENSG00000138075	0	0	0	0
ENSG00000138078	33	177	350	339
ENSG00000138079	0	0	8	1
ENSG00000138080	8	49	13	25
ENSG00000138081	61	2306	2863	3917
ENSG00000138083	0	0	0	0
ENSG00000138085	87	82	36	176
ENSG00000138092	2	34	20	16
ENSG00000138095	16	996	693	1243
ENSG00000138100	0	0	0	0
ENSG00000138101	11	171	86	153
ENSG00000138107	193	942	626	871
ENSG00000138109	0	0	0	0
ENSG00000138111	4	0	3	1
ENSG00000138115	0	0	1	6
ENSG00000138119	22	0	0	0
ENSG00000138131	0	0	0	0
ENSG00000138134	1	41	41	72
ENSG00000138135	0	0	0	0
ENSG00000138136	0	0	0	0
ENSG00000138138	0	116	99	135
ENSG00000138152	0	0	0	0
ENSG00000138160	3	441	429	649
ENSG00000138161	0	0	2	0
ENSG00000138162	1	25	1	5
ENSG00000138166	6	20	11	9
ENSG00000138172	13	0	0	0
ENSG00000138175	28	54	46	47
ENSG00000138180	0	118	91	160
ENSG00000138182	204	4757	5649	5143
ENSG00000138185	33	0	10	0
ENSG00000138190	32	2689	2832	4048
ENSG00000138193	1	0	1	1
ENSG00000138207	0	0	0	0
ENSG00000138231	9	34	40	78
ENSG00000138246	21	574	380	454
ENSG00000138271	0	0	0	1
ENSG00000138279	340	3280	3146	3505
ENSG00000138286	58	1122	986	1243
ENSG00000138293	22208	211965	176538	250330
ENSG00000138294	0	0	0	7
ENSG00000138297	18	40	19	45
ENSG00000138303	3	139	177	275

ENSG00000138308	0	0	0	0
ENSG00000138311	1	0	0	0
ENSG00000138315	0	0	0	2
ENSG00000138316	0	0	0	0
ENSG00000138326	1435	365	243	858
ENSG00000138336	1	506	770	502
ENSG00000138346	2	2230	3143	4110
ENSG00000138347	0	0	0	0
ENSG00000138356	0	0	0	1
ENSG00000138363	25	34	11	19
ENSG00000138375	12	43	173	164
ENSG00000138376	171	7852	6207	10380
ENSG00000138378	10	100	34	52
ENSG00000138379	0	0	0	0
ENSG00000138380	18	913	689	804
ENSG00000138381	23	66	111	86
ENSG00000138382	22	280	330	460
ENSG00000138385	140	366	261	354
ENSG00000138386	43	1084	1121	1536
ENSG00000138395	1	0	0	0
ENSG00000138398	187	393	518	582
ENSG00000138399	9	2266	2028	2234
ENSG00000138400	0	0	0	0
ENSG00000138411	1	43	8	3
ENSG00000138413	8	71	57	130
ENSG00000138430	46	445	542	833
ENSG00000138433	260	483	262	447
ENSG00000138434	563	5838	8305	6689
ENSG00000138435	0	0	0	0
ENSG00000138439	6	89	35	77
ENSG00000138442	3	31	66	78
ENSG00000138443	630	2712	2724	3529
ENSG00000138448	17	548	474	791
ENSG00000138449	4947	45966	84803	31405
ENSG00000138459	10	263	345	239
ENSG00000138463	0	12	12	6
ENSG00000138468	68	1991	3403	2447
ENSG00000138472	0	17	0	1
ENSG00000138483	2	0	0	0
ENSG00000138495	525	660	388	635
ENSG00000138496	41	553	478	401
ENSG00000138587	19	102	34	40
ENSG00000138592	125	2171	3006	3800
ENSG00000138593	181	2950	2890	2681
ENSG00000138594	990	12542	15062	14829
ENSG00000138600	16	792	563	714

ENSG00000138604	5	864	1208	1340
ENSG00000138606	0	0	0	0
ENSG00000138613	426	191	402	318
ENSG00000138614	9	224	95	174
ENSG00000138615	0	0	0	0
ENSG00000138617	2	0	3	0
ENSG00000138621	17	9	8	9
ENSG00000138622	0	0	0	0
ENSG00000138623	2	30	78	40
ENSG00000138629	26	0	2	3
ENSG00000138639	3	22	16	40
ENSG00000138640	8	534	299	735
ENSG00000138641	15	45	164	143
ENSG00000138642	36	245	225	289
ENSG00000138646	10	0	20	19
ENSG00000138650	0	0	0	0
ENSG00000138653	0	0	0	0
ENSG00000138658	0	805	807	1192
ENSG00000138660	4	150	118	128
ENSG00000138663	64	1078	912	1392
ENSG00000138668	136	545	426	584
ENSG00000138669	0	172	158	144
ENSG00000138670	2	15	51	12
ENSG00000138674	207	2498	1808	3392
ENSG00000138675	4	12	2	10
ENSG00000138678	1	0	0	1
ENSG00000138684	0	0	0	0
ENSG00000138685	38	340	958	660
ENSG00000138686	7	189	254	231
ENSG00000138688	85	2343	2067	2554
ENSG00000138696	1	17	18	12
ENSG00000138698	177	3778	3906	3118
ENSG00000138709	315	4571	4590	7041
ENSG00000138722	650	19431	25107	41805
ENSG00000138735	2125	62755	77437	69740
ENSG00000138738	0	844	1141	3645
ENSG00000138741	0	0	0	0
ENSG00000138744	9	0	3	2
ENSG00000138750	13	3493	3503	5607
ENSG00000138755	1	0	4	0
ENSG00000138756	31	3605	5806	3932
ENSG00000138757	695	9086	9308	10788
ENSG00000138758	2989	13277	18236	9924
ENSG00000138759	0	8	0	0
ENSG00000138760	8	0	43	63
ENSG00000138764	18	45	109	10

ENSG00000138767	171	5267	3650	5611
ENSG00000138768	34	1186	1166	1511
ENSG00000138769	0	0	12	5
ENSG00000138771	0	0	2	0
ENSG00000138772	211	574	744	1089
ENSG00000138777	51	7720	7910	10765
ENSG00000138778	43	736	1038	1130
ENSG00000138780	1	74	37	70
ENSG00000138785	10	225	175	235
ENSG00000138792	0	0	14	14
ENSG00000138794	363	600	465	434
ENSG00000138795	12	0	0	0
ENSG00000138796	141	635	350	497
ENSG00000138798	589	24200	19882	20065
ENSG00000138801	113	2445	1450	2715
ENSG00000138802	11	1928	1270	1941
ENSG00000138813	0	0	0	0
ENSG00000138814	151	604	818	614
ENSG00000138821	19	740	683	736
ENSG00000138823	0	0	2	3
ENSG00000138829	0	0	0	0
ENSG00000138834	5	331	242	464
ENSG00000138835	85	776	591	239
ENSG00000138867	119	695	540	686
ENSG00000138892	0	0	0	0
ENSG00000138942	12	0	3	7
ENSG00000138944	0	0	0	0
ENSG00000138964	44	20	2	8
ENSG00000139044	0	0	0	0
ENSG00000139053	38	58	25	50
ENSG00000139055	6	0	17	14
ENSG00000139083	136	1932	1335	1969
ENSG00000139112	123	247	490	141
ENSG00000139116	7	0	0	6
ENSG00000139117	27	509	699	573
ENSG00000139131	1	14	18	23
ENSG00000139132	82	16863	11790	15721
ENSG00000139133	20	927	1165	975
ENSG00000139144	0	0	0	0
ENSG00000139146	53	460	412	476
ENSG00000139151	0	0	2	0
ENSG00000139154	20	2937	2716	4315
ENSG00000139155	1	2	6	22
ENSG00000139160	10	0	37	3
ENSG00000139163	5	432	579	494
ENSG00000139168	120	534	257	1116

ENSG00000139173	0	12	27	38
ENSG00000139174	2	88	45	50
ENSG00000139178	4	25	10	19
ENSG00000139180	27	0	1	11
ENSG00000139182	1	9	5	17
ENSG00000139187	37	44	24	22
ENSG00000139190	3	0	6	9
ENSG00000139192	12	67	64	49
ENSG00000139193	3	2	21	12
ENSG00000139194	0	0	0	0
ENSG00000139197	1	0	2	6
ENSG00000139200	0	0	0	0
ENSG00000139209	0	0	0	0
ENSG00000139211	295	1196	1284	661
ENSG00000139218	346	2116	2077	2283
ENSG00000139219	0	0	0	1
ENSG00000139220	0	0	1	0
ENSG00000139223	0	0	0	0
ENSG00000139233	26	7	2	14
ENSG00000139239	1	0	2	3
ENSG00000139263	0	0	0	0
ENSG00000139266	7	0	6	9
ENSG00000139269	0	0	0	0
ENSG00000139278	4	0	13	6
ENSG00000139287	2	42	24	17
ENSG00000139289	12	67	36	70
ENSG00000139291	8	27	19	14
ENSG00000139292	0	0	0	0
ENSG00000139304	0	0	1	0
ENSG00000139318	97	0	27	46
ENSG00000139323	431	7563	6258	7053
ENSG00000139324	1	262	383	369
ENSG00000139329	0	0	0	0
ENSG00000139330	0	0	0	0
ENSG00000139343	48	77	1	1
ENSG00000139344	0	0	0	0
ENSG00000139350	68	1343	1241	1600
ENSG00000139351	2	57	15	26
ENSG00000139352	0	0	0	0
ENSG00000139354	3	50	52	90
ENSG00000139364	0	0	0	3
ENSG00000139370	3	1312	892	1397
ENSG00000139372	96	366	362	536
ENSG00000139405	4	0	3	20
ENSG00000139410	2	0	0	0
ENSG00000139428	7	0	0	0

ENSG00000139433	225	186	240	286
ENSG00000139436	32	808	607	813
ENSG00000139437	17	56	27	49
ENSG00000139438	0	0	0	0
ENSG00000139445	0	0	0	0
ENSG00000139496	26	675	697	760
ENSG00000139505	30	80	206	225
ENSG00000139508	0	126	133	100
ENSG00000139514	24	165	79	147
ENSG00000139515	0	0	0	0
ENSG00000139517	9	306	283	421
ENSG00000139531	23	27	53	33
ENSG00000139537	11	0	0	2
ENSG00000139540	0	0	0	0
ENSG00000139546	8	0	0	1
ENSG00000139547	1	0	0	2
ENSG00000139549	0	0	0	0
ENSG00000139567	0	15	0	4
ENSG00000139572	0	0	0	0
ENSG00000139574	0	0	0	0
ENSG00000139579	37	9	6	25
ENSG00000139597	197	1863	2128	2140
ENSG00000139610	0	0	5	0
ENSG00000139613	75	227	196	318
ENSG00000139618	11	582	498	644
ENSG00000139620	2	5	22	40
ENSG00000139624	54	43	101	90
ENSG00000139625	2	0	6	3
ENSG00000139626	3	0	1	2
ENSG00000139629	3	0	0	0
ENSG00000139631	2	0	0	3
ENSG00000139636	1	0	2	4
ENSG00000139637	19	24	2	11
ENSG00000139641	11	16	25	44
ENSG00000139644	2551	5491	5839	4500
ENSG00000139645	5	5	15	24
ENSG00000139648	0	0	0	0
ENSG00000139651	2	14	5	1
ENSG00000139656	0	0	0	0
ENSG00000139668	14	61	39	78
ENSG00000139675	2	0	0	0
ENSG00000139679	8	15	6	4
ENSG00000139684	77	127	120	207
ENSG00000139687	56	1544	1787	1534
ENSG00000139697	173	2301	2230	2339
ENSG00000139714	6	0	4	8

ENSG00000139718	19	36	5	32
ENSG00000139719	11	0	1	10
ENSG00000139722	70	943	621	491
ENSG00000139725	0	418	186	353
ENSG00000139726	62	229	102	184
ENSG00000139734	19	522	411	590
ENSG00000139737	4	383	322	558
ENSG00000139746	22	863	993	1270
ENSG00000139767	0	0	0	0
ENSG00000139780	0	0	0	0
ENSG00000139793	170	2497	2170	3388
ENSG00000139797	0	0	0	0
ENSG00000139800	0	0	0	0
ENSG00000139826	19	192	323	479
ENSG00000139832	6	8	12	23
ENSG00000139835	40	206	145	135
ENSG00000139842	38	78	95	151
ENSG00000139865	0	0	4	0
ENSG00000139874	0	0	0	0
ENSG00000139880	1	0	0	0
ENSG00000139890	0	3	0	0
ENSG00000139899	0	0	0	3
ENSG00000139908	26	0	0	16
ENSG00000139910	1	0	0	5
ENSG00000139914	0	0	0	0
ENSG00000139915	0	0	3	19
ENSG00000139921	78	1459	1550	2331
ENSG00000139926	0	0	0	1
ENSG00000139946	529	2092	3465	1391
ENSG00000139970	94	640	1138	859
ENSG00000139971	2	5	13	17
ENSG00000139973	0	0	0	0
ENSG00000139974	32	468	398	369
ENSG00000139977	5	50	59	49
ENSG00000139985	0	0	0	0
ENSG00000139988	0	0	0	0
ENSG00000139990	73	734	688	523
ENSG00000139998	1	0	1	0
ENSG00000140006	3	51	68	90
ENSG00000140009	1	0	1	2
ENSG00000140015	0	0	0	3
ENSG00000140022	1528	11495	13580	9784
ENSG00000140025	7	183	132	235
ENSG00000140030	16	0	1	4
ENSG00000140043	1	0	0	0
ENSG00000140044	4	0	0	0

ENSG00000140057	0	0	0	0
ENSG00000140067	0	0	0	0
ENSG00000140090	2	0	97	31
ENSG00000140092	4	0	2	2
ENSG00000140093	0	0	0	0
ENSG00000140104	0	0	0	0
ENSG00000140105	139	75	71	84
ENSG00000140107	0	0	0	0
ENSG00000140153	6	960	1307	2846
ENSG00000140157	4	85	84	94
ENSG00000140181	47	86	86	435
ENSG00000140199	17	515	344	414
ENSG00000140254	111	0	9	0
ENSG00000140259	42	172	77	127
ENSG00000140262	58	586	707	783
ENSG00000140263	36	38	36	40
ENSG00000140264	56665	21812	9085	18518
ENSG00000140265	72	446	221	274
ENSG00000140274	0	0	0	0
ENSG00000140279	1	0	0	3
ENSG00000140280	21	0	1	1
ENSG00000140284	0	0	9	6
ENSG00000140285	0	0	30	47
ENSG00000140287	0	0	0	0
ENSG00000140297	0	0	0	0
ENSG00000140299	1181	13605	13600	13120
ENSG00000140307	284	379	696	304
ENSG00000140319	2692	2487	1723	3283
ENSG00000140320	13	35	24	11
ENSG00000140323	0	0	0	0
ENSG00000140326	3	43	6	14
ENSG00000140332	30	29	29	29
ENSG00000140350	356	865	464	928
ENSG00000140365	26	25	3	20
ENSG00000140367	710	13504	9382	14597
ENSG00000140368	11	13	1	0
ENSG00000140374	1184	28173	11005	12504
ENSG00000140379	55	90	15	14
ENSG00000140382	9	885	374	710
ENSG00000140386	32	363	350	464
ENSG00000140391	8	81	58	62
ENSG00000140395	28	46	29	61
ENSG00000140396	265	5191	7504	6318
ENSG00000140398	0	0	0	0
ENSG00000140400	9	30	11	37
ENSG00000140403	4	46	1	6

ENSG00000140406	12	0	8	7
ENSG00000140416	1770	11763	16221	20935
ENSG00000140443	111	294	351	300
ENSG00000140450	45	450	684	182
ENSG00000140451	1	0	1	2
ENSG00000140455	34	2079	1674	2712
ENSG00000140459	22	20	3	18
ENSG00000140463	1	0	11	3
ENSG00000140464	21	14	1	10
ENSG00000140465	0	0	0	0
ENSG00000140470	0	0	1	1
ENSG00000140471	1	560	194	1491
ENSG00000140474	12	39	21	49
ENSG00000140478	0	0	0	0
ENSG00000140479	356	4413	4522	6815
ENSG00000140481	0	0	0	0
ENSG00000140488	0	0	0	0
ENSG00000140497	149	143	123	130
ENSG00000140505	0	0	0	0
ENSG00000140506	0	0	0	0
ENSG00000140511	1	0	1	0
ENSG00000140519	0	0	0	0
ENSG00000140521	3	7	24	21
ENSG00000140522	0	0	0	0
ENSG00000140525	4	253	403	623
ENSG00000140526	32	4895	3814	5327
ENSG00000140527	0	0	0	0
ENSG00000140534	0	59	36	55
ENSG00000140538	0	0	1	0
ENSG00000140543	2	0	21	28
ENSG00000140545	2	0	0	1
ENSG00000140548	1	29	3	7
ENSG00000140553	162	583	446	715
ENSG00000140557	0	0	0	2
ENSG00000140563	6	1630	1910	2893
ENSG00000140564	107	1431	1074	1013
ENSG00000140575	100	333	202	225
ENSG00000140577	8	0	18	28
ENSG00000140598	9	357	424	695
ENSG00000140600	2	38	79	10
ENSG00000140612	115	2739	3295	4277
ENSG00000140623	0	0	0	0
ENSG00000140632	52	441	559	1055
ENSG00000140650	3	15	56	67
ENSG00000140675	0	0	0	0
ENSG00000140678	25	176	66	74

ENSG00000140682	150	196	238	423
ENSG00000140688	1	0	2	2
ENSG00000140691	8	74	21	37
ENSG00000140694	41	1646	1799	2763
ENSG00000140718	4	1572	899	1286
ENSG00000140740	80	578	291	596
ENSG00000140743	54	649	325	942
ENSG00000140749	72	4	7	0
ENSG00000140750	2	34	110	144
ENSG00000140795	0	0	0	1
ENSG00000140798	0	0	0	0
ENSG00000140807	0	0	0	0
ENSG00000140829	14	98	47	80
ENSG00000140830	263	2832	2411	2011
ENSG00000140832	0	0	0	0
ENSG00000140835	0	0	0	1
ENSG00000140836	13	0	21	33
ENSG00000140839	0	0	0	0
ENSG00000140848	104	224	154	62
ENSG00000140853	64	336	240	307
ENSG00000140854	44	293	116	94
ENSG00000140859	160	1699	1055	1301
ENSG00000140873	0	0	1	0
ENSG00000140876	0	0	5	12
ENSG00000140905	4	18	34	36
ENSG00000140931	94	420	244	398
ENSG00000140932	115	344	131	259
ENSG00000140937	0	0	1	0
ENSG00000140939	0	0	0	1
ENSG00000140941	3323	22218	21604	21404
ENSG00000140943	9	72	36	75
ENSG00000140945	0	0	3	4
ENSG00000140948	5	7	10	6
ENSG00000140950	4	0	1	2
ENSG00000140955	0	0	0	0
ENSG00000140961	2	0	0	2
ENSG00000140968	39	11	12	4
ENSG00000140983	5	98	9	49
ENSG00000140986	1	0	0	0
ENSG00000140987	7	0	3	9
ENSG00000140988	1147	94	23	54
ENSG00000140990	107	72	42	107
ENSG00000140992	34	73	81	61
ENSG00000140993	1	0	9	11
ENSG00000140995	8	34	0	0
ENSG00000141002	91	287	127	289

ENSG00000141012	2	0	1	4
ENSG00000141013	0	0	7	3
ENSG00000141026	6	1	13	222
ENSG00000141027	1114	8535	6430	9521
ENSG00000141028	0	0	0	0
ENSG00000141030	158	1775	1575	1985
ENSG00000141034	15	6	41	27
ENSG00000141040	1	0	2	3
ENSG00000141052	0	0	0	0
ENSG00000141068	12	254	115	216
ENSG00000141076	3	235	267	345
ENSG00000141084	11	41	14	24
ENSG00000141086	0	0	0	0
ENSG00000141096	0	0	0	0
ENSG00000141098	2	15	14	17
ENSG00000141101	10	0	1	2
ENSG00000141127	67	1214	1413	1596
ENSG00000141140	8	147	80	93
ENSG00000141141	52	603	467	542
ENSG00000141150	0	0	0	0
ENSG00000141161	0	0	18	58
ENSG00000141179	54	345	420	392
ENSG00000141194	0	0	0	0
ENSG00000141198	11	705	1363	1447
ENSG00000141200	0	0	0	0
ENSG00000141219	6	138	144	277
ENSG00000141232	17	0	22	11
ENSG00000141252	18	355	195	451
ENSG00000141255	0	0	1	1
ENSG00000141258	2	0	2	7
ENSG00000141279	127	2649	4084	3017
ENSG00000141293	75	35	13	25
ENSG00000141294	0	0	0	0
ENSG00000141295	0	0	1	1
ENSG00000141298	186	6341	5925	8629
ENSG00000141314	1	0	0	3
ENSG00000141316	0	0	0	0
ENSG00000141337	9	38	40	32
ENSG00000141338	0	0	0	0
ENSG00000141349	23	5	16	15
ENSG00000141367	114	4569	4402	4910
ENSG00000141371	0	0	0	0
ENSG00000141376	29	353	294	513
ENSG00000141378	9	10	24	10
ENSG00000141380	45	1598	1360	1381
ENSG00000141384	3	612	387	559

ENSG00000141385	5	134	177	257
ENSG00000141391	0	0	8	15
ENSG00000141401	26	70	39	29
ENSG00000141404	3	0	1	4
ENSG00000141424	4	30	57	31
ENSG00000141425	6	1732	1472	2195
ENSG00000141428	7	47	54	53
ENSG00000141429	141	1007	1245	1543
ENSG00000141431	34	4	30	4
ENSG00000141433	1	0	0	0
ENSG00000141434	1	0	1	1
ENSG00000141437	0	0	0	0
ENSG00000141441	1	0	1	3
ENSG00000141446	45	1579	2430	2182
ENSG00000141447	11	561	505	590
ENSG00000141448	0	0	0	0
ENSG00000141449	0	7	3	12
ENSG00000141452	72	1947	1572	1394
ENSG00000141456	5	0	2	11
ENSG00000141458	3	131	110	263
ENSG00000141469	8	0	24	23
ENSG00000141480	187	157	124	169
ENSG00000141485	0	0	0	0
ENSG00000141497	3	0	2	3
ENSG00000141499	4	0	0	0
ENSG00000141503	129	2418	1149	1406
ENSG00000141504	28	0	1	3
ENSG00000141505	2	0	0	0
ENSG00000141506	15	0	8	9
ENSG00000141510	39	0	4	0
ENSG00000141519	0	0	0	0
ENSG00000141522	1	103	38	87
ENSG00000141524	7	0	1	5
ENSG00000141526	3	32	9	12
ENSG00000141527	0	0	0	0
ENSG00000141540	0	0	0	0
ENSG00000141542	0	0	1	0
ENSG00000141543	10	25	14	26
ENSG00000141551	20	38	19	37
ENSG00000141552	445	93	35	45
ENSG00000141556	10	302	66	134
ENSG00000141560	8	96	13	20
ENSG00000141562	38	35	51	72
ENSG00000141564	5	238	159	348
ENSG00000141568	6	161	94	176
ENSG00000141569	8	46	2	0

ENSG00000141570	0	0	1	0
ENSG00000141574	2	0	0	0
ENSG00000141576	20	0	0	0
ENSG00000141577	1	0	3	4
ENSG00000141579	0	0	0	0
ENSG00000141580	91	485	387	530
ENSG00000141582	2	1	5	2
ENSG00000141622	1	0	1	0
ENSG00000141627	54	1244	1041	1782
ENSG00000141639	0	0	0	0
ENSG00000141642	6	0	6	3
ENSG00000141644	25	87	66	84
ENSG00000141646	21	376	218	348
ENSG00000141655	0	1051	403	752
ENSG00000141664	119	2836	3044	4972
ENSG00000141665	9	51	24	55
ENSG00000141668	0	0	0	0
ENSG00000141682	11	13	22	13
ENSG00000141696	3	0	0	0
ENSG00000141698	2	39	13	16
ENSG00000141699	92	417	149	221
ENSG00000141720	60	275	276	407
ENSG00000141736	0	0	1	0
ENSG00000141738	0	0	0	0
ENSG00000141741	42	21	15	33
ENSG00000141744	0	0	0	0
ENSG00000141748	0	0	0	0
ENSG00000141750	0	0	0	0
ENSG00000141753	5	0	0	0
ENSG00000141756	0	0	3	0
ENSG00000141759	298	512	300	642
ENSG00000141837	1	0	0	0
ENSG00000141854	1422	835	391	486
ENSG00000141858	13	0	4	6
ENSG00000141867	191	214	149	286
ENSG00000141873	207	573	312	319
ENSG00000141905	26	0	23	34
ENSG00000141933	2	0	6	1
ENSG00000141934	0	0	0	0
ENSG00000141946	0	0	0	0
ENSG00000141956	1	0	5	3
ENSG00000141959	18	51	62	111
ENSG00000141965	0	88	40	93
ENSG00000141968	8	103	40	42
ENSG00000141971	2	0	0	7
ENSG00000141977	2	0	0	0

ENSG00000141979	0	0	0	0
ENSG00000141985	33	49	9	21
ENSG00000141994	0	0	0	0
ENSG00000142002	19	31	32	49
ENSG00000142025	0	0	0	0
ENSG00000142039	13	58	10	17
ENSG00000142046	7107	1697	1673	544
ENSG00000142065	14	127	19	21
ENSG00000142082	37	53	18	32
ENSG00000142089	377	97	94	101
ENSG00000142102	0	0	0	0
ENSG00000142149	0	0	0	0
ENSG00000142156	0	0	0	0
ENSG00000142163	0	0	0	0
ENSG00000142166	25	225	245	220
ENSG00000142168	964	1466	1510	1429
ENSG00000142173	0	0	0	0
ENSG00000142178	1	0	0	0
ENSG00000142182	0	0	0	0
ENSG00000142185	0	0	0	0
ENSG00000142186	3	30	33	43
ENSG00000142188	13	141	131	115
ENSG00000142192	2537	17342	13125	14516
ENSG00000142197	8	790	536	798
ENSG00000142207	7	0	6	7
ENSG00000142208	13	253	214	314
ENSG00000142224	1	0	0	0
ENSG00000142227	666	2749	1479	1169
ENSG00000142230	42	183	196	242
ENSG00000142233	0	0	0	0
ENSG00000142235	0	0	0	2
ENSG00000142252	5	0	0	0
ENSG00000142273	0	0	0	0
ENSG00000142279	0	0	0	0
ENSG00000142303	0	0	0	0
ENSG00000142319	0	0	0	0
ENSG00000142327	64	141	156	302
ENSG00000142330	0	0	0	0
ENSG00000142347	30	78	32	20
ENSG00000142396	13	0	2	3
ENSG00000142405	3	11	3	0
ENSG00000142408	2	0	0	0
ENSG00000142409	3	6	2	5
ENSG00000142444	15	31	18	31
ENSG00000142449	0	0	0	0
ENSG00000142453	2	19	12	12

ENSG00000142459	5	59	21	35
ENSG00000142484	13	0	3	3
ENSG00000142494	0	0	0	0
ENSG00000142507	102	0	5	0
ENSG00000142511	0	0	0	0
ENSG00000142512	6	0	0	0
ENSG00000142513	0	0	0	0
ENSG00000142515	0	0	0	0
ENSG00000142528	1	0	1	2
ENSG00000142530	0	1	2	0
ENSG00000142534	3219	2510	1230	2965
ENSG00000142538	0	0	0	0
ENSG00000142539	0	0	0	0
ENSG00000142541	2704	398	154	623
ENSG00000142544	0	0	0	0
ENSG00000142546	115	114	66	137
ENSG00000142549	0	0	0	0
ENSG00000142552	0	0	0	0
ENSG00000142556	0	57	22	18
ENSG00000142583	0	0	0	10
ENSG00000142599	113	4102	3817	7564
ENSG00000142606	0	0	1	0
ENSG00000142609	12	94	11	3
ENSG00000142611	0	0	0	0
ENSG00000142615	0	0	0	0
ENSG00000142619	0	0	1	0
ENSG00000142621	1	18	1	0
ENSG00000142623	0	0	0	0
ENSG00000142627	0	0	0	0
ENSG00000142632	2	0	1	0
ENSG00000142634	73	24	2	1
ENSG00000142655	1	1	8	4
ENSG00000142657	1562	4283	2201	3082
ENSG00000142661	0	0	0	0
ENSG00000142669	46612	4971	5303	6269
ENSG00000142675	0	0	0	0
ENSG00000142676	1210	282	148	327
ENSG00000142677	0	0	0	0
ENSG00000142684	0	0	0	0
ENSG00000142686	14	32	19	12
ENSG00000142687	34	789	592	848
ENSG00000142694	22	208	142	57
ENSG00000142698	0	0	0	0
ENSG00000142700	0	0	0	0
ENSG00000142731	2	46	65	78
ENSG00000142733	2	0	0	0

ENSG00000142748	0	5	2	0
ENSG00000142751	5	3	3	4
ENSG00000142765	4	0	0	0
ENSG00000142784	87	709	358	598
ENSG00000142789	0	0	0	0
ENSG00000142794	25	195	405	480
ENSG00000142798	0	0	0	0
ENSG00000142856	882	2899	5956	5926
ENSG00000142864	312	629	647	913
ENSG00000142867	17	291	152	127
ENSG00000142871	0	0	0	0
ENSG00000142875	762	23828	27991	26334
ENSG00000142892	18	776	1084	1032
ENSG00000142910	1	0	0	1
ENSG00000142920	1	0	0	0
ENSG00000142937	1786	86	88	174
ENSG00000142945	0	0	7	34
ENSG00000142949	136	553	169	279
ENSG00000142959	0	0	0	0
ENSG00000142961	679	2148	940	1717
ENSG00000142973	0	0	0	0
ENSG00000143001	0	0	0	0
ENSG00000143006	0	0	0	0
ENSG00000143013	47	65	40	33
ENSG00000143028	0	0	0	0
ENSG00000143032	0	0	0	0
ENSG00000143033	57	174	196	218
ENSG00000143036	0	0	0	0
ENSG00000143061	1	0	0	0
ENSG00000143067	0	0	0	0
ENSG00000143079	3	116	92	75
ENSG00000143093	0	0	0	0
ENSG00000143105	0	0	0	0
ENSG00000143106	54	53	54	52
ENSG00000143107	0	0	0	0
ENSG00000143110	148	0	1	4
ENSG00000143119	33	0	3	11
ENSG00000143125	0	0	0	0
ENSG00000143126	0	0	0	0
ENSG00000143127	0	0	0	1
ENSG00000143140	0	0	4	1
ENSG00000143147	1	0	1	0
ENSG00000143149	60	166	425	517
ENSG00000143153	32	130	250	149
ENSG00000143155	38	299	238	185
ENSG00000143156	7	2696	1687	2540

ENSG00000143157	94	495	547	412
ENSG00000143158	151	788	675	797
ENSG00000143162	287	1555	2708	1614
ENSG00000143164	286	3682	4224	4853
ENSG00000143167	9	1	4	39
ENSG00000143171	0	0	0	0
ENSG00000143178	12	7	8	10
ENSG00000143179	2	13	13	32
ENSG00000143183	44	278	284	281
ENSG00000143184	0	23	0	0
ENSG00000143185	6	0	0	0
ENSG00000143190	115	348	390	445
ENSG00000143194	21	241	11	165
ENSG00000143195	2	109	8	75
ENSG00000143196	0	0	0	0
ENSG00000143198	113	584	1494	1325
ENSG00000143199	2	17	5	24
ENSG00000143207	23	3410	2306	3805
ENSG00000143217	0	0	0	0
ENSG00000143222	98	69	77	185
ENSG00000143224	7	0	1	9
ENSG00000143226	278	1112	2404	2729
ENSG00000143228	3	78	50	62
ENSG00000143248	2	0	3	9
ENSG00000143252	64	29	23	23
ENSG00000143256	50	47	115	175
ENSG00000143257	2	0	0	7
ENSG00000143258	14	0	16	34
ENSG00000143278	0	0	1	2
ENSG00000143294	97	90	86	104
ENSG00000143297	0	0	0	0
ENSG00000143303	6	0	4	0
ENSG00000143314	26	0	0	0
ENSG00000143315	4	0	3	4
ENSG00000143318	0	0	0	0
ENSG00000143319	79	35	42	42
ENSG00000143320	1	0	0	0
ENSG00000143321	3911	6779	7026	10438
ENSG00000143322	3	83	184	273
ENSG00000143324	105	1841	2260	1772
ENSG00000143333	51	29	19	32
ENSG00000143337	16	271	223	280
ENSG00000143340	0	0	0	0
ENSG00000143341	0	0	6	3
ENSG00000143344	2	36	2	10
ENSG00000143353	1158	16954	13792	17585

ENSG00000143355	0	0	1	0
ENSG00000143363	1820	10787	10525	9251
ENSG00000143365	0	0	0	0
ENSG00000143367	0	0	0	0
ENSG00000143368	39	186	131	134
ENSG00000143369	34	81	44	113
ENSG00000143373	1	10	4	11
ENSG00000143374	8	110	60	152
ENSG00000143375	0	0	0	0
ENSG00000143376	21	232	396	359
ENSG00000143379	14	122	103	165
ENSG00000143382	0	0	0	0
ENSG00000143384	123	732	792	595
ENSG00000143387	0	0	2	3
ENSG00000143390	5	0	3	12
ENSG00000143393	21	92	71	126
ENSG00000143398	3	243	183	336
ENSG00000143401	91	178	219	214
ENSG00000143409	1024	6687	5398	5650
ENSG00000143412	20	39	31	32
ENSG00000143416	2	3	10	10
ENSG00000143418	146	8205	6415	7060
ENSG00000143420	278	710	675	1501
ENSG00000143429	4	0	0	0
ENSG00000143434	0	0	0	4
ENSG00000143436	2	28	15	6
ENSG00000143437	81	889	558	794
ENSG00000143442	13	86	46	70
ENSG00000143443	1	0	5	1
ENSG00000143450	8	0	3	16
ENSG00000143452	0	36	9	13
ENSG00000143457	4	74	68	104
ENSG00000143458	19	68	54	70
ENSG00000143466	9	0	0	0
ENSG00000143469	0	0	0	0
ENSG00000143473	0	0	0	0
ENSG00000143476	2	544	351	688
ENSG00000143479	4	25	13	6
ENSG00000143486	11	2	7	18
ENSG00000143493	5	598	358	481
ENSG00000143494	0	0	0	0
ENSG00000143498	3	88	70	132
ENSG00000143499	122	723	777	778
ENSG00000143502	0	0	0	0
ENSG00000143507	2	0	7	5
ENSG00000143512	0	0	0	0

ENSG00000143514	47	1176	2011	3212
ENSG00000143515	18	103	146	233
ENSG00000143520	0	0	0	0
ENSG00000143536	0	0	0	0
ENSG00000143537	0	0	0	0
ENSG00000143543	346	757	568	842
ENSG00000143545	625	430	192	281
ENSG00000143546	846	431	90	292
ENSG00000143549	6987	30075	23114	37626
ENSG00000143552	7	118	68	111
ENSG00000143553	80	223	183	433
ENSG00000143554	1	13	0	1
ENSG00000143556	0	0	0	0
ENSG00000143569	50	348	446	571
ENSG00000143570	25	48	77	86
ENSG00000143575	26	0	11	6
ENSG00000143578	1	15	2	8
ENSG00000143590	0	0	0	0
ENSG00000143595	60	1034	552	1008
ENSG00000143603	11	41	16	28
ENSG00000143612	202	845	484	970
ENSG00000143614	19	509	265	318
ENSG00000143621	55	57	63	108
ENSG00000143622	806	11874	14405	12414
ENSG00000143624	5	1	17	55
ENSG00000143627	0	0	0	0
ENSG00000143630	0	0	0	3
ENSG00000143631	0	0	0	0
ENSG00000143632	0	0	0	0
ENSG00000143633	26	144	49	62
ENSG00000143641	27	210	83	264
ENSG00000143643	1	585	685	1049
ENSG00000143653	33	469	1120	1598
ENSG00000143669	281	5306	6904	6023
ENSG00000143674	0	0	0	13
ENSG00000143702	55	807	758	668
ENSG00000143727	1213	5322	5261	4396
ENSG00000143740	3	0	1	15
ENSG00000143742	465	4225	3871	4084
ENSG00000143748	8	164	194	302
ENSG00000143751	29	312	675	199
ENSG00000143753	15	105	147	155
ENSG00000143756	9	55	29	34
ENSG00000143761	4117	9981	6234	11638
ENSG00000143768	26	2	1	31
ENSG00000143771	33	42	84	39

ENSG00000143772	69	224	60	123
ENSG00000143774	322	742	380	1062
ENSG00000143776	118	3630	3622	3907
ENSG00000143786	28	67	29	65
ENSG00000143793	10	0	2	6
ENSG00000143797	237	32189	18252	30250
ENSG00000143799	106	32	26	80
ENSG00000143801	7	6	7	19
ENSG00000143811	297	1995	858	1868
ENSG00000143815	130	1045	833	818
ENSG00000143816	0	0	0	0
ENSG00000143819	4	0	15	22
ENSG00000143839	0	0	0	0
ENSG00000143842	1	0	0	0
ENSG00000143845	0	0	1	0
ENSG00000143847	0	0	0	0
ENSG00000143850	0	65	27	48
ENSG00000143851	22	61	51	112
ENSG00000143858	0	0	0	0
ENSG00000143862	11	71	8	16
ENSG00000143867	0	0	0	0
ENSG00000143869	0	0	0	0
ENSG00000143870	10	282	265	438
ENSG00000143878	4	0	28	8
ENSG00000143882	0	1	0	0
ENSG00000143889	173	4061	3188	3681
ENSG00000143891	278	795	469	327
ENSG00000143919	1	320	308	547
ENSG00000143921	0	0	0	0
ENSG00000143924	34	651	606	759
ENSG00000143933	2516	24343	18894	29450
ENSG00000143942	3	0	2	5
ENSG00000143947	3040	1131	984	1979
ENSG00000143951	24	864	954	1511
ENSG00000143952	15	3434	3688	4105
ENSG00000143954	0	0	0	0
ENSG00000143970	21	1399	1013	1349
ENSG00000143971	0	65	62	92
ENSG00000143977	127	7	14	14
ENSG00000143994	0	0	3	0
ENSG00000143995	2628	11564	20177	11214
ENSG00000144010	0	0	0	0
ENSG00000144015	0	0	0	0
ENSG00000144021	36	114	18	16
ENSG00000144026	2	81	19	15
ENSG00000144028	32	130	50	95

ENSG00000144029	60	51	34	65
ENSG00000144031	0	0	0	0
ENSG00000144034	67	334	237	403
ENSG00000144035	6	5	2	12
ENSG00000144036	4	9215	9705	13679
ENSG00000144040	16	87	93	134
ENSG00000144043	28	23	20	45
ENSG00000144045	0	0	0	0
ENSG00000144048	17	14	29	33
ENSG00000144057	0	2253	1012	2693
ENSG00000144061	0	0	2	1
ENSG00000144063	7	203	108	148
ENSG00000144115	0	0	0	0
ENSG00000144118	357	2725	2163	3143
ENSG00000144119	0	0	0	0
ENSG00000144120	0	0	0	0
ENSG00000144130	0	0	0	0
ENSG00000144134	37	57	14	101
ENSG00000144136	8	49	49	35
ENSG00000144140	0	0	0	0
ENSG00000144152	0	0	0	0
ENSG00000144158	0	0	0	0
ENSG00000144161	7	130	83	146
ENSG00000144182	4	93	18	34
ENSG00000144188	0	5	0	0
ENSG00000144191	0	0	0	0
ENSG00000144199	1	0	0	0
ENSG00000144214	1	0	0	2
ENSG00000144218	7	46	19	32
ENSG00000144224	730	2175	1824	2094
ENSG00000144227	0	0	0	0
ENSG00000144228	37	895	1036	873
ENSG00000144229	0	0	0	0
ENSG00000144230	0	0	0	0
ENSG00000144231	6	6	30	72
ENSG00000144233	12	43	58	42
ENSG00000144278	0	14	6	4
ENSG00000144283	153	575	703	552
ENSG00000144285	2	0	12	0
ENSG00000144290	0	0	0	3
ENSG00000144306	62	459	727	740
ENSG00000144320	19	2041	2613	3317
ENSG00000144331	1	13	1	16
ENSG00000144339	2	6	1	9
ENSG00000144354	0	68	114	110
ENSG00000144355	0	0	0	0

ENSG00000144357	14	2934	2695	3454
ENSG00000144362	0	0	3	3
ENSG00000144366	0	0	6	13
ENSG00000144369	0	0	9	12
ENSG00000144381	62	336	225	459
ENSG00000144395	7	181	97	162
ENSG00000144401	11	0	13	23
ENSG00000144406	0	17	0	0
ENSG00000144407	0	83	62	37
ENSG00000144410	0	12	0	0
ENSG00000144426	8	1023	911	1626
ENSG00000144445	9	1385	1714	2761
ENSG00000144451	18	416	616	1063
ENSG00000144452	0	1	0	3
ENSG00000144455	14	383	316	607
ENSG00000144460	0	8	14	2
ENSG00000144468	313	24562	17441	24323
ENSG00000144476	0	0	0	0
ENSG00000144481	0	0	0	0
ENSG00000144485	3	0	2	0
ENSG00000144488	0	0	0	0
ENSG00000144504	23	102	125	181
ENSG00000144524	30	53	28	66
ENSG00000144535	3	198	170	209
ENSG00000144550	1	0	0	0
ENSG00000144554	8	145	189	251
ENSG00000144559	2	647	442	662
ENSG00000144560	74	1107	1417	1338
ENSG00000144566	90	1741	1784	1629
ENSG00000144567	225	596	799	588
ENSG00000144579	1165	982	338	605
ENSG00000144580	19	443	415	800
ENSG00000144583	2	0	4	12
ENSG00000144589	4	0	0	0
ENSG00000144591	23	0	2	0
ENSG00000144596	0	30	13	4
ENSG00000144597	24	744	1362	419
ENSG00000144619	1	55	8	4
ENSG00000144635	74	1029	977	1237
ENSG00000144642	1	21	4	0
ENSG00000144644	0	0	0	0
ENSG00000144645	3	0	7	0
ENSG00000144647	39	106	72	45
ENSG00000144648	109	155	48	46
ENSG00000144649	2806	39	125	50
ENSG00000144655	17	57	20	12

ENSG00000144659	9	0	7	0
ENSG00000144668	31	30	29	55
ENSG00000144671	0	0	0	0
ENSG00000144674	119	2428	3030	3502
ENSG00000144677	4061	6681	7166	9917
ENSG00000144681	3	59	92	89
ENSG00000144711	26	21	8	2
ENSG00000144712	0	8	0	0
ENSG00000144713	1789	129	73	242
ENSG00000144724	0	21	22	30
ENSG00000144730	1	0	0	1
ENSG00000144736	4	445	310	457
ENSG00000144741	11	269	212	290
ENSG00000144744	80	1412	1246	1181
ENSG00000144746	14432	37851	34065	32218
ENSG00000144747	164	690	703	784
ENSG00000144749	0	0	4	1
ENSG00000144771	2	0	2	11
ENSG00000144785	0	0	0	0
ENSG00000144791	88	415	429	780
ENSG00000144792	3	0	17	18
ENSG00000144802	5	28	42	65
ENSG00000144810	3	0	0	7
ENSG00000144815	23	36	227	245
ENSG00000144820	0	11	2	1
ENSG00000144821	0	0	1	0
ENSG00000144824	1	10	7	0
ENSG00000144827	22	266	141	201
ENSG00000144834	0	0	0	0
ENSG00000144837	1	0	1	1
ENSG00000144840	35	148	179	277
ENSG00000144843	21	57	20	46
ENSG00000144847	0	0	1	4
ENSG00000144848	68	557	495	871
ENSG00000144852	0	0	0	0
ENSG00000144857	0	0	0	0
ENSG00000144867	4	1	16	3
ENSG00000144868	41	29	53	87
ENSG00000144891	0	0	0	1
ENSG00000144893	9688	20286	14026	22332
ENSG00000144895	42	447	479	609
ENSG00000144908	0	0	0	0
ENSG00000144909	15	125	139	256
ENSG00000144935	2	478	456	782
ENSG00000144959	0	84	45	122
ENSG00000144962	3	0	5	17

ENSG00000145002	0	0	0	0
ENSG00000145012	1290	5248	8804	8982
ENSG00000145014	6	0	10	44
ENSG00000145016	48	145	124	282
ENSG00000145020	19	19	7	26
ENSG00000145022	333	457	424	428
ENSG00000145029	5	15	9	14
ENSG00000145040	0	0	0	0
ENSG00000145041	12	163	97	127
ENSG00000145050	7	2	23	47
ENSG00000145063	0	0	0	0
ENSG00000145075	0	0	0	0
ENSG00000145087	0	0	0	0
ENSG00000145088	37	1526	1836	1678
ENSG00000145103	0	0	0	0
ENSG00000145107	0	0	0	1
ENSG00000145113	0	0	0	0
ENSG00000145147	0	0	0	0
ENSG00000145191	15	0	7	33
ENSG00000145192	0	0	0	0
ENSG00000145194	1	0	0	2
ENSG00000145198	0	0	0	0
ENSG00000145214	7	8	6	4
ENSG00000145216	169	13332	10224	15694
ENSG00000145217	0	0	0	0
ENSG00000145220	39	80	93	154
ENSG00000145241	20	475	563	626
ENSG00000145242	0	0	0	0
ENSG00000145244	0	11	4	21
ENSG00000145246	3	18	37	43
ENSG00000145247	21	11	1	17
ENSG00000145248	0	0	0	0
ENSG00000145283	0	0	0	8
ENSG00000145284	0	0	0	0
ENSG00000145287	222	52	22	13
ENSG00000145293	19	141	305	492
ENSG00000145309	0	0	0	0
ENSG00000145321	0	0	0	0
ENSG00000145331	3	17	8	18
ENSG00000145332	79	5705	9008	17832
ENSG00000145335	1281	9908	9529	10014
ENSG00000145337	0	92	66	245
ENSG00000145348	3	2048	1755	2025
ENSG00000145349	65	229	304	751
ENSG00000145354	82	294	319	240
ENSG00000145358	0	0	0	0

ENSG00000145362	0	1	13	15
ENSG00000145365	6	33	6	0
ENSG00000145375	3	197	184	276
ENSG00000145384	0	0	0	4
ENSG00000145386	2	21	26	37
ENSG00000145388	17	136	72	78
ENSG00000145390	6	36	121	108
ENSG00000145391	5	0	5	2
ENSG00000145414	0	32	18	22
ENSG00000145416	17	0	5	2
ENSG00000145423	0	0	0	0
ENSG00000145425	2232	1777	1056	2875
ENSG00000145428	0	0	0	0
ENSG00000145431	265	3368	5265	3088
ENSG00000145439	14	1568	1017	1171
ENSG00000145451	0	0	2	0
ENSG00000145476	1	15	18	7
ENSG00000145491	7	33	20	9
ENSG00000145494	258	131	66	125
ENSG00000145495	526	10076	9419	8914
ENSG00000145506	0	0	0	0
ENSG00000145526	0	0	0	0
ENSG00000145536	0	0	0	0
ENSG00000145545	3	11	16	33
ENSG00000145555	38	95	27	67
ENSG00000145569	6	33	88	27
ENSG00000145592	1533	375	401	817
ENSG00000145604	0	24	3	9
ENSG00000145623	0	8	0	0
ENSG00000145626	0	0	0	11
ENSG00000145632	1	0	5	6
ENSG00000145642	0	0	0	0
ENSG00000145649	63	0	1	0
ENSG00000145675	307	1580	1550	767
ENSG00000145681	0	0	0	0
ENSG00000145685	259	3316	4502	5484
ENSG00000145687	311	2322	3698	4417
ENSG00000145692	0	0	0	0
ENSG00000145700	3	63	127	240
ENSG00000145703	1707	11285	12720	13603
ENSG00000145708	0	54	12	12
ENSG00000145715	20	12718	9331	17555
ENSG00000145721	0	0	0	0
ENSG00000145723	4	17	32	37
ENSG00000145725	21	386	346	493
ENSG00000145730	13	106	310	320

ENSG00000145734	170	1798	1866	2541
ENSG00000145736	0	215	33	85
ENSG00000145740	100	2928	3157	3915
ENSG00000145741	464	1912	1061	2664
ENSG00000145743	100	3200	4554	5945
ENSG00000145757	0	20	23	8
ENSG00000145777	0	0	0	0
ENSG00000145779	354	2913	1395	2128
ENSG00000145780	4	55	78	39
ENSG00000145781	15	376	264	338
ENSG00000145782	141	595	850	823
ENSG00000145794	0	0	1	2
ENSG00000145808	1	0	0	2
ENSG00000145817	43	191	307	277
ENSG00000145819	27	421	154	286
ENSG00000145824	0	0	0	0
ENSG00000145826	0	0	0	6
ENSG00000145832	0	0	0	0
ENSG00000145833	136	319	313	401
ENSG00000145835	0	0	0	0
ENSG00000145839	0	0	8	0
ENSG00000145850	0	0	0	0
ENSG00000145860	8	6	33	48
ENSG00000145861	0	0	0	0
ENSG00000145863	0	0	0	0
ENSG00000145864	1	1	12	7
ENSG00000145868	12	10	82	102
ENSG00000145879	0	0	0	0
ENSG00000145882	61	449	137	302
ENSG00000145888	0	0	0	0
ENSG00000145901	155	207	187	203
ENSG00000145907	100	775	595	948
ENSG00000145908	0	0	1	0
ENSG00000145911	1	0	2	0
ENSG00000145912	17	0	0	2
ENSG00000145916	54	140	17	15
ENSG00000145919	1	0	2	1
ENSG00000145920	0	0	0	0
ENSG00000145934	0	0	0	0
ENSG00000145936	26	11	83	75
ENSG00000145945	8	18	13	49
ENSG00000145949	9	94	45	103
ENSG00000145965	0	0	0	0
ENSG00000145975	0	0	0	0
ENSG00000145979	17	59	36	76
ENSG00000145982	229	791	1003	621

ENSG00000145990	101	263	374	379
ENSG00000145996	38	1713	2583	4213
ENSG00000146001	0	0	0	0
ENSG00000146005	0	0	0	0
ENSG00000146006	0	0	0	0
ENSG00000146007	1251	1926	2459	2163
ENSG00000146013	0	0	0	0
ENSG00000146021	2	25	17	36
ENSG00000146038	1	0	17	46
ENSG00000146039	0	0	0	0
ENSG00000146047	0	0	0	0
ENSG00000146049	0	0	0	0
ENSG00000146054	0	0	0	0
ENSG00000146063	20	0	20	18
ENSG00000146066	143	14	26	22
ENSG00000146067	15	147	108	129
ENSG00000146070	29	132	113	75
ENSG00000146072	11	53	56	89
ENSG00000146083	53	72	32	58
ENSG00000146085	5	112	67	40
ENSG00000146090	0	0	0	0
ENSG00000146094	276	320	166	262
ENSG00000146109	12	0	1	5
ENSG00000146112	462	454	247	475
ENSG00000146122	0	0	0	0
ENSG00000146143	2	130	83	90
ENSG00000146147	0	0	0	5
ENSG00000146151	0	0	0	0
ENSG00000146166	0	0	0	0
ENSG00000146192	21	0	3	3
ENSG00000146197	0	0	0	0
ENSG00000146205	1	0	1	0
ENSG00000146215	0	0	0	0
ENSG00000146216	0	0	0	0
ENSG00000146221	0	0	0	0
ENSG00000146223	113	46	45	61
ENSG00000146232	3	0	0	0
ENSG00000146233	0	0	1	0
ENSG00000146242	1	0	0	2
ENSG00000146243	0	20	24	9
ENSG00000146247	166	2424	3424	4505
ENSG00000146250	0	0	0	0
ENSG00000146263	8	533	301	363
ENSG00000146267	0	0	0	0
ENSG00000146276	0	0	0	0
ENSG00000146278	55	370	392	598

ENSG00000146281	3	24	35	41
ENSG00000146282	34	800	790	1338
ENSG00000146285	3	0	0	0
ENSG00000146350	3	556	540	671
ENSG00000146352	0	3	0	0
ENSG00000146360	0	0	0	0
ENSG00000146373	7	541	469	457
ENSG00000146374	0	0	0	0
ENSG00000146376	3480	24485	32538	26199
ENSG00000146378	0	0	0	0
ENSG00000146383	0	0	0	0
ENSG00000146385	0	0	0	0
ENSG00000146386	224	269	231	719
ENSG00000146399	0	11	6	2
ENSG00000146409	0	15	0	3
ENSG00000146410	0	0	2	7
ENSG00000146411	7	3	7	1
ENSG00000146414	9	2075	1554	2592
ENSG00000146416	1044	11946	7387	8762
ENSG00000146425	104	153	205	382
ENSG00000146426	3	228	169	477
ENSG00000146433	0	1967	1732	1216
ENSG00000146453	56	29	6	0
ENSG00000146457	176	523	597	605
ENSG00000146463	16	7276	7329	10782
ENSG00000146469	14	56	33	32
ENSG00000146476	17	42	115	53
ENSG00000146477	0	0	1	0
ENSG00000146521	0	0	0	0
ENSG00000146530	0	32	26	25
ENSG00000146535	398	659	211	527
ENSG00000146540	558	518	339	641
ENSG00000146555	0	32	20	145
ENSG00000146556	6	61	19	16
ENSG00000146574	8	455	200	621
ENSG00000146576	17	44	14	38
ENSG00000146587	9	134	119	165
ENSG00000146592	7	0	81	2
ENSG00000146618	0	0	0	0
ENSG00000146648	0	17	0	0
ENSG00000146666	0	0	0	0
ENSG00000146670	1	0	0	0
ENSG00000146674	0	0	1	0
ENSG00000146676	83	477	532	597
ENSG00000146677	1	0	0	0
ENSG00000146678	0	0	0	0

ENSG00000146700	0	0	0	1
ENSG00000146701	233	190	205	280
ENSG00000146707	15	66	29	94
ENSG00000146722	0	0	12	4
ENSG00000146729	38	186	476	580
ENSG00000146731	62	225	137	215
ENSG00000146733	3	7	13	24
ENSG00000146755	0	0	0	0
ENSG00000146757	3	121	222	123
ENSG00000146776	25	450	392	586
ENSG00000146802	1	0	27	38
ENSG00000146809	0	0	0	0
ENSG00000146826	0	0	1	1
ENSG00000146828	4	11	3	0
ENSG00000146830	3	0	1	3
ENSG00000146833	13	78	69	111
ENSG00000146834	2745	2441	1480	2209
ENSG00000146839	0	0	0	0
ENSG00000146842	7	723	500	854
ENSG00000146856	9	97	170	282
ENSG00000146857	1	0	0	0
ENSG00000146858	20	588	515	239
ENSG00000146859	2948	7468	9568	2990
ENSG00000146872	86	6111	5176	8046
ENSG00000146904	4	38	79	38
ENSG00000146909	9	275	100	114
ENSG00000146910	0	0	0	2
ENSG00000146918	8	306	356	534
ENSG00000146926	0	0	0	0
ENSG00000146938	0	0	0	0
ENSG00000146950	0	0	0	0
ENSG00000146955	0	0	0	0
ENSG00000146963	644	900	829	553
ENSG00000146966	0	0	2	0
ENSG00000147003	0	0	1	3
ENSG00000147010	294	366	380	345
ENSG00000147027	0	0	4	0
ENSG00000147036	333	2133	2503	749
ENSG00000147041	0	0	0	2
ENSG00000147044	3	11	17	49
ENSG00000147050	25	308	488	345
ENSG00000147059	1	0	5	15
ENSG00000147065	2726	11971	7779	11898
ENSG00000147081	0	0	0	0
ENSG00000147082	0	0	12	7
ENSG00000147099	38	189	253	302

ENSG00000147100	0	0	0	0
ENSG00000147113	3	0	13	8
ENSG00000147117	0	0	2	0
ENSG00000147118	5	30	4	6
ENSG00000147119	1	18	17	1
ENSG00000147121	8	24	14	24
ENSG00000147123	155	80	37	59
ENSG00000147124	10	108	129	118
ENSG00000147127	1	0	0	0
ENSG00000147130	14	5	13	8
ENSG00000147133	30	396	515	876
ENSG00000147138	0	14	85	22
ENSG00000147140	151	750	491	701
ENSG00000147144	0	0	1	0
ENSG00000147145	191	734	614	415
ENSG00000147155	7	0	10	21
ENSG00000147160	0	0	0	0
ENSG00000147162	11	180	191	148
ENSG00000147164	90	377	252	291
ENSG00000147166	3	17	0	1
ENSG00000147168	71	0	0	0
ENSG00000147174	21	68	49	32
ENSG00000147180	7	122	171	150
ENSG00000147183	0	0	0	0
ENSG00000147202	24	198	208	148
ENSG00000147206	307	79	28	149
ENSG00000147223	0	0	0	0
ENSG00000147224	10	0	35	40
ENSG00000147231	0	0	0	0
ENSG00000147234	0	0	0	0
ENSG00000147246	0	0	0	0
ENSG00000147251	37	3963	3675	5542
ENSG00000147255	0	0	0	0
ENSG00000147256	0	0	0	0
ENSG00000147257	0	0	0	0
ENSG00000147262	0	0	0	0
ENSG00000147274	75	204	247	229
ENSG00000147316	8	49	42	107
ENSG00000147324	8	11	2	7
ENSG00000147364	14	101	44	83
ENSG00000147378	1	0	0	0
ENSG00000147380	0	0	0	0
ENSG00000147381	0	0	0	0
ENSG00000147382	10	63	20	24
ENSG00000147383	4	3	22	24
ENSG00000147394	2779	17417	15610	16149

ENSG00000147400	462	3393	3089	3884
ENSG00000147402	0	0	0	0
ENSG00000147403	1706	733	628	528
ENSG00000147408	2	0	4	0
ENSG00000147416	71	641	549	231
ENSG00000147419	78	218	280	248
ENSG00000147421	27	336	481	833
ENSG00000147432	0	0	0	0
ENSG00000147434	0	0	0	0
ENSG00000147437	0	0	3	5
ENSG00000147439	5	0	5	14
ENSG00000147443	984	1167	941	1694
ENSG00000147454	33	151	195	96
ENSG00000147457	14	16	8	9
ENSG00000147459	88	3457	3009	4555
ENSG00000147465	0	0	0	0
ENSG00000147471	55	478	395	652
ENSG00000147475	5	0	10	7
ENSG00000147481	0	0	0	0
ENSG00000147485	0	3	0	0
ENSG00000147488	0	0	1	4
ENSG00000147509	0	0	0	0
ENSG00000147526	1364	5009	4768	4745
ENSG00000147533	287	1019	989	1048
ENSG00000147535	174	413	391	313
ENSG00000147536	1	15	10	4
ENSG00000147548	252	1145	1161	1466
ENSG00000147570	0	0	0	0
ENSG00000147571	0	0	2	0
ENSG00000147573	0	0	2	0
ENSG00000147576	6	32	29	77
ENSG00000147586	4	0	2	9
ENSG00000147588	0	0	0	0
ENSG00000147592	15	649	762	1003
ENSG00000147596	0	0	0	0
ENSG00000147601	37	52	180	174
ENSG00000147604	1034	890	702	1202
ENSG00000147606	0	2	14	5
ENSG00000147613	0	0	0	0
ENSG00000147614	0	0	0	16
ENSG00000147642	0	16	0	0
ENSG00000147647	4	21	36	31
ENSG00000147649	571	3012	2683	3710
ENSG00000147650	205	2923	3841	1783
ENSG00000147654	29	120	218	239
ENSG00000147655	0	0	0	0

ENSG00000147669	27	248	101	177
ENSG00000147676	0	0	0	0
ENSG00000147677	263	480	447	786
ENSG00000147679	17	52	12	22
ENSG00000147684	453	346	266	266
ENSG00000147687	15	585	400	527
ENSG00000147689	0	0	0	0
ENSG00000147697	0	4	8	4
ENSG00000147724	0	0	0	0
ENSG00000147753	0	0	0	0
ENSG00000147761	0	0	0	0
ENSG00000147789	7	13	9	5
ENSG00000147799	0	0	0	0
ENSG00000147804	193	40	3	21
ENSG00000147813	4	7	0	3
ENSG00000147852	1	0	3	2
ENSG00000147853	436	987	929	1324
ENSG00000147854	79	2922	2706	2797
ENSG00000147862	534	1489	1799	2862
ENSG00000147869	0	0	0	0
ENSG00000147872	39	77	105	37
ENSG00000147873	0	0	0	0
ENSG00000147874	19	3132	3139	3659
ENSG00000147883	34	28	159	46
ENSG00000147885	0	0	0	0
ENSG00000147889	0	0	0	0
ENSG00000147894	3	63	140	65
ENSG00000147896	0	0	0	0
ENSG00000147905	51	2407	2325	3046
ENSG00000147912	0	0	0	0
ENSG00000147926	0	0	0	0
ENSG00000147955	5	0	9	8
ENSG00000147996	2	45	187	211
ENSG00000148019	22	19	18	3
ENSG00000148053	0	0	0	0
ENSG00000148057	13	10	11	22
ENSG00000148082	0	0	4	0
ENSG00000148090	2	193	94	165
ENSG00000148110	21	120	193	146
ENSG00000148120	135	1421	899	882
ENSG00000148123	0	0	0	0
ENSG00000148136	0	0	0	0
ENSG00000148143	0	128	26	100
ENSG00000148153	36	118	68	112
ENSG00000148154	4	44	42	70
ENSG00000148156	0	0	0	0

ENSG00000148158	10	51	59	48
ENSG00000148175	2800	26516	36946	36906
ENSG00000148180	574	5318	2186	4489
ENSG00000148187	6	258	239	275
ENSG00000148200	2	19	1	0
ENSG00000148204	0	0	1	0
ENSG00000148215	0	0	0	0
ENSG00000148218	26	28	10	60
ENSG00000148219	6	0	9	12
ENSG00000148225	2	38	59	115
ENSG00000148229	478	1109	923	1215
ENSG00000148248	104	406	226	273
ENSG00000148288	2	0	0	0
ENSG00000148290	127	25	12	34
ENSG00000148291	4	0	0	0
ENSG00000148296	3	18	0	0
ENSG00000148297	2	8	2	3
ENSG00000148300	5	0	15	22
ENSG00000148303	1056	86	139	340
ENSG00000148308	68	173	111	181
ENSG00000148331	4	0	0	4
ENSG00000148334	16	16	10	11
ENSG00000148335	12	0	1	4
ENSG00000148337	48	92	74	149
ENSG00000148339	0	0	17	12
ENSG00000148341	2	94	24	77
ENSG00000148343	223	687	544	378
ENSG00000148344	0	0	0	0
ENSG00000148346	7637	5115	2925	2796
ENSG00000148356	3	3	5	7
ENSG00000148357	0	0	0	0
ENSG00000148358	14	790	649	920
ENSG00000148362	7	0	0	0
ENSG00000148377	0	0	0	0
ENSG00000148384	0	0	0	0
ENSG00000148386	0	0	0	0
ENSG00000148396	23	130	55	78
ENSG00000148399	0	18	16	15
ENSG00000148400	7	46	15	55
ENSG00000148408	0	0	2	0
ENSG00000148411	5	0	3	7
ENSG00000148426	220	837	861	650
ENSG00000148429	2	676	974	1067
ENSG00000148444	39	34	16	9
ENSG00000148450	14	2	11	12
ENSG00000148459	1	327	464	367

ENSG00000148468	0	0	0	0
ENSG00000148481	16	4606	3912	5946
ENSG00000148482	0	0	2	0
ENSG00000148483	0	0	0	5
ENSG00000148484	2013	119909	35641	48287
ENSG00000148488	85	1318	1350	2619
ENSG00000148498	1281	10938	11522	15272
ENSG00000148513	0	0	0	1
ENSG00000148516	14	30	40	58
ENSG00000148541	0	0	4	0
ENSG00000148572	19	32	33	12
ENSG00000148584	0	0	0	0
ENSG00000148600	3	16	18	4
ENSG00000148602	0	0	0	0
ENSG00000148604	0	0	0	0
ENSG00000148606	6	151	74	96
ENSG00000148634	38	2038	2635	3392
ENSG00000148655	4	70	42	98
ENSG00000148660	41	146	244	233
ENSG00000148671	0	0	0	0
ENSG00000148672	51	277	318	553
ENSG00000148677	0	0	0	0
ENSG00000148680	0	0	0	0
ENSG00000148688	18	6	35	99
ENSG00000148690	242	2207	2015	3415
ENSG00000148700	2567	12389	14693	16071
ENSG00000148702	0	0	0	0
ENSG00000148704	0	0	0	0
ENSG00000148719	15	203	81	300
ENSG00000148730	262	1254	1549	1328
ENSG00000148734	0	0	0	0
ENSG00000148735	0	0	0	0
ENSG00000148737	27	0	7	9
ENSG00000148773	7	283	261	602
ENSG00000148795	3	0	0	0
ENSG00000148798	1	0	1	0
ENSG00000148803	5	0	0	0
ENSG00000148814	3	0	9	6
ENSG00000148824	0	8	0	0
ENSG00000148826	0	0	0	0
ENSG00000148828	0	0	0	0
ENSG00000148832	18	36	34	56
ENSG00000148834	3550	7024	4057	4752
ENSG00000148835	6	81	41	73
ENSG00000148840	5	0	3	3
ENSG00000148841	0	0	2	2

ENSG00000148842	35	450	236	302
ENSG00000148843	9	171	330	641
ENSG00000148848	83	370	328	546
ENSG00000148908	31767	70726	45744	66179
ENSG00000148925	4	1644	1813	2259
ENSG00000148926	0	0	0	0
ENSG00000148935	20	24	39	42
ENSG00000148942	0	0	0	0
ENSG00000148943	38	2650	3937	4072
ENSG00000148948	1	0	20	5
ENSG00000148950	13	198	143	163
ENSG00000148965	0	0	0	0
ENSG00000148985	6	0	0	0
ENSG00000149016	0	0	0	0
ENSG00000149021	0	0	0	0
ENSG00000149043	0	0	1	0
ENSG00000149050	0	0	3	0
ENSG00000149054	0	0	5	9
ENSG00000149084	98	1007	926	1274
ENSG00000149089	45	268	337	573
ENSG00000149090	0	0	0	0
ENSG00000149091	213	279	155	348
ENSG00000149100	56	232	193	348
ENSG00000149115	0	0	0	0
ENSG00000149124	3	18	16	5
ENSG00000149131	203	35	56	25
ENSG00000149133	0	0	0	0
ENSG00000149136	55	27	0	1
ENSG00000149150	1	124	31	76
ENSG00000149177	428	23703	29482	28910
ENSG00000149179	80	181	89	136
ENSG00000149182	62	153	82	43
ENSG00000149187	23	372	213	420
ENSG00000149196	31	37	107	187
ENSG00000149201	0	25	34	35
ENSG00000149212	54	742	1230	331
ENSG00000149218	3235	21679	21319	25608
ENSG00000149231	189	1405	1651	1198
ENSG00000149243	43	50	30	28
ENSG00000149256	0	0	0	0
ENSG00000149257	3	0	2	3
ENSG00000149260	4	0	0	0
ENSG00000149262	5	65	173	287
ENSG00000149269	60	1	17	37
ENSG00000149273	2148	122	105	95
ENSG00000149289	21	4098	3115	4492

ENSG00000149292	2	0	0	5
ENSG00000149294	0	0	0	0
ENSG00000149295	0	0	0	0
ENSG00000149300	0	0	0	0
ENSG00000149305	0	0	1	0
ENSG00000149308	99	4178	4439	4866
ENSG00000149311	85	1838	1918	2781
ENSG00000149313	44	189	256	238
ENSG00000149328	0	0	0	0
ENSG00000149346	4	454	658	1218
ENSG00000149357	7339	8670	5020	10925
ENSG00000149380	0	0	0	0
ENSG00000149403	4	35	20	40
ENSG00000149418	0	0	0	0
ENSG00000149428	1	0	7	15
ENSG00000149435	0	0	0	0
ENSG00000149443	0	0	0	0
ENSG00000149451	0	0	0	0
ENSG00000149452	0	0	0	0
ENSG00000149474	0	0	1	9
ENSG00000149476	41	63	26	62
ENSG00000149480	49	288	112	118
ENSG00000149483	45	901	724	1028
ENSG00000149485	293	271	519	504
ENSG00000149488	0	0	0	0
ENSG00000149489	1	0	2	2
ENSG00000149499	2	0	6	17
ENSG00000149503	0	23	8	9
ENSG00000149506	0	0	0	0
ENSG00000149507	0	0	0	0
ENSG00000149516	3	0	2	6
ENSG00000149527	0	0	0	0
ENSG00000149531	55	336	125	281
ENSG00000149532	15	14	16	35
ENSG00000149534	0	0	0	0
ENSG00000149541	10	0	0	14
ENSG00000149547	15	0	18	33
ENSG00000149548	15	295	239	300
ENSG00000149554	3	122	57	92
ENSG00000149557	8	0	2	0
ENSG00000149564	2913	2863	1818	1952
ENSG00000149571	0	0	3	0
ENSG00000149573	1	0	0	0
ENSG00000149575	1	0	0	0
ENSG00000149577	5	3	1	0
ENSG00000149582	1	0	0	0

ENSG00000149591	3	0	5	0
ENSG00000149596	0	0	0	0
ENSG00000149599	0	0	0	0
ENSG00000149600	1322	1823	708	1164
ENSG00000149609	0	0	0	0
ENSG00000149633	0	0	0	0
ENSG00000149634	0	0	7	4
ENSG00000149635	0	0	0	2
ENSG00000149636	6	35	18	7
ENSG00000149639	2	0	3	9
ENSG00000149646	0	0	0	0
ENSG00000149651	0	0	0	0
ENSG00000149654	0	0	0	3
ENSG00000149656	0	0	0	0
ENSG00000149657	47	143	110	198
ENSG00000149658	7	27	16	11
ENSG00000149679	1	0	0	3
ENSG00000149716	6	51	22	20
ENSG00000149735	0	0	0	0
ENSG00000149742	0	0	0	0
ENSG00000149743	17	0	13	14
ENSG00000149761	3	31	13	41
ENSG00000149781	2880	12381	5122	13235
ENSG00000149782	2	9	20	24
ENSG00000149792	57	390	156	174
ENSG00000149798	4	0	0	0
ENSG00000149806	1075	407	161	217
ENSG00000149809	10	6	4	5
ENSG00000149823	12	0	2	6
ENSG00000149922	0	0	0	0
ENSG00000149923	189	261	120	182
ENSG00000149925	6057	4329	2173	4793
ENSG00000149926	0	15	0	0
ENSG00000149927	0	0	0	0
ENSG00000149929	20	0	11	11
ENSG00000149930	17	116	77	80
ENSG00000149932	4200	1793	998	1758
ENSG00000149948	0	0	8	19
ENSG00000149968	0	0	0	0
ENSG00000149970	1	10	1	13
ENSG00000149972	0	0	0	2
ENSG00000150045	55	21	22	0
ENSG00000150048	0	0	0	2
ENSG00000150051	0	0	0	0
ENSG00000150054	44	3278	4671	6208
ENSG00000150076	169	2669	3019	5158

ENSG00000150086	0	0	0	0
ENSG00000150093	8618	86283	133122	153858
ENSG00000150165	0	0	0	0
ENSG00000150175	0	0	0	0
ENSG00000150201	0	0	0	0
ENSG00000150244	0	0	0	0
ENSG00000150261	0	0	0	0
ENSG00000150269	0	0	0	0
ENSG00000150275	0	0	2	0
ENSG00000150276	1	0	0	0
ENSG00000150281	1	0	0	0
ENSG00000150316	102	83	82	87
ENSG00000150337	0	16	0	0
ENSG00000150347	8	38	2	2
ENSG00000150361	0	0	0	0
ENSG00000150394	0	0	0	0
ENSG00000150401	0	23	18	7
ENSG00000150403	38	275	340	409
ENSG00000150433	7	0	15	30
ENSG00000150455	14	122	55	74
ENSG00000150456	21	54	368	206
ENSG00000150457	27	273	275	367
ENSG00000150459	405	1723	1114	1565
ENSG00000150471	0	145	199	123
ENSG00000150477	32	813	1025	1148
ENSG00000150510	2	0	0	0
ENSG00000150526	0	0	0	0
ENSG00000150527	2	0	3	10
ENSG00000150540	15	0	9	11
ENSG00000150551	0	0	0	0
ENSG00000150556	0	0	0	6
ENSG00000150593	115	557	532	263
ENSG00000150594	44	260	254	178
ENSG00000150625	1	0	2	1
ENSG00000150627	0	28	21	21
ENSG00000150628	0	0	0	0
ENSG00000150630	57	1322	1186	1404
ENSG00000150636	4	116	106	112
ENSG00000150637	3053	66935	91073	67704
ENSG00000150656	0	0	0	4
ENSG00000150667	4	281	265	353
ENSG00000150672	2	140	121	235
ENSG00000150676	0	1	2	5
ENSG00000150681	63803	432729	559443	426566
ENSG00000150687	7	0	0	4
ENSG00000150712	378	5913	4944	7363

ENSG00000150722	1	0	2	12
ENSG00000150732	0	0	0	0
ENSG00000150750	0	0	0	0
ENSG00000150753	114	214	84	153
ENSG00000150756	13	50	105	106
ENSG00000150760	8	983	1252	2179
ENSG00000150764	7	23	19	3
ENSG00000150768	14	122	162	284
ENSG00000150773	0	14	0	8
ENSG00000150776	56	34	88	149
ENSG00000150779	293	240	100	319
ENSG00000150782	16	1	4	13
ENSG00000150783	0	0	0	0
ENSG00000150787	34	138	19	80
ENSG00000150867	3666	49784	45117	43143
ENSG00000150873	0	0	0	0
ENSG00000150893	0	0	0	0
ENSG00000150907	159	246	319	145
ENSG00000150938	2	34	10	11
ENSG00000150961	13	222	185	113
ENSG00000150967	13	62	1	17
ENSG00000150977	121	615	397	762
ENSG00000150990	1	9	0	5
ENSG00000150991	4503	17519	11450	15284
ENSG00000150995	149	1643	1660	1611
ENSG00000151005	0	0	0	0
ENSG00000151006	0	0	1	0
ENSG00000151012	14	207	390	249
ENSG00000151014	1	2	22	42
ENSG00000151023	5249	21415	28340	30215
ENSG00000151025	0	0	0	2
ENSG00000151033	1	0	0	0
ENSG00000151062	4	0	4	0
ENSG00000151065	1	0	8	9
ENSG00000151067	0	14	0	0
ENSG00000151079	0	0	0	0
ENSG00000151090	639	1553	2363	1745
ENSG00000151092	21	354	318	488
ENSG00000151093	5	0	2	1
ENSG00000151116	39	397	448	345
ENSG00000151117	5	18	11	6
ENSG00000151131	43	332	186	239
ENSG00000151135	40	386	393	405
ENSG00000151136	7	1091	6	510
ENSG00000151148	184	1606	811	975
ENSG00000151150	61	199	257	168

ENSG00000151151	4	5	39	48
ENSG00000151164	5	312	451	493
ENSG00000151176	0	0	0	0
ENSG00000151208	0	0	0	0
ENSG00000151224	0	0	0	0
ENSG00000151229	8	382	844	943
ENSG00000151233	1	405	281	300
ENSG00000151239	5	18	47	52
ENSG00000151240	0	0	0	0
ENSG00000151247	345	8642	7596	8497
ENSG00000151276	1	86	43	182
ENSG00000151287	4	28	47	9
ENSG00000151292	56	23041	21448	38486
ENSG00000151303	0	22	8	2
ENSG00000151304	18	1219	628	1530
ENSG00000151320	0	0	3	15
ENSG00000151322	0	0	0	4
ENSG00000151327	445	1666	2473	2022
ENSG00000151332	4	135	86	130
ENSG00000151338	22	230	319	434
ENSG00000151348	13	222	314	235
ENSG00000151353	18	86	80	77
ENSG00000151360	0	0	0	0
ENSG00000151364	0	0	0	0
ENSG00000151365	0	0	0	2
ENSG00000151366	20	0	0	8
ENSG00000151376	2	3	6	6
ENSG00000151379	0	0	0	0
ENSG00000151388	0	0	2	0
ENSG00000151413	2	8	28	26
ENSG00000151414	212	14215	15115	13612
ENSG00000151418	0	0	0	0
ENSG00000151422	145	8141	9703	11877
ENSG00000151445	7	81	56	46
ENSG00000151458	8	79	128	45
ENSG00000151461	205	3671	3793	4641
ENSG00000151465	31	462	569	913
ENSG00000151466	708	45305	34803	46305
ENSG00000151468	25	39	82	101
ENSG00000151470	32	125	150	172
ENSG00000151474	3	0	1	5
ENSG00000151475	0	0	0	0
ENSG00000151490	1	0	0	3
ENSG00000151491	11	0	6	23
ENSG00000151498	75	310	104	168
ENSG00000151500	37	49	31	56

ENSG00000151502	235	1035	461	738
ENSG00000151503	20	508	262	373
ENSG00000151532	7	215	193	191
ENSG00000151552	19	26	9	8
ENSG00000151553	44	1664	1529	2006
ENSG00000151572	1	0	0	0
ENSG00000151575	89	2312	4542	3026
ENSG00000151576	8	7	4	8
ENSG00000151577	0	0	0	0
ENSG00000151611	2	32	37	20
ENSG00000151612	2	327	347	618
ENSG00000151615	0	0	0	0
ENSG00000151617	0	0	0	0
ENSG00000151623	2	15	0	8
ENSG00000151631	0	0	0	0
ENSG00000151632	0	0	1	4
ENSG00000151640	0	0	0	0
ENSG00000151650	0	0	0	0
ENSG00000151651	0	0	0	0
ENSG00000151655	0	0	0	0
ENSG00000151657	56	524	433	775
ENSG00000151665	36	364	378	541
ENSG00000151687	4	2116	1439	2449
ENSG00000151689	6	53	39	49
ENSG00000151690	374	1955	1802	1769
ENSG00000151692	1	11	7	0
ENSG00000151693	161	19111	16486	24566
ENSG00000151694	4	998	848	1701
ENSG00000151702	649	11672	7714	13496
ENSG00000151704	3	0	3	0
ENSG00000151715	1	0	0	0
ENSG00000151718	3	0	13	83
ENSG00000151725	70	807	1381	634
ENSG00000151726	7	37	38	35
ENSG00000151729	1	0	0	0
ENSG00000151743	22	1177	983	1017
ENSG00000151746	90	566	562	622
ENSG00000151748	2038	4378	3857	3537
ENSG00000151773	28	112	195	647
ENSG00000151778	7	19	5	0
ENSG00000151779	173	7093	9143	9252
ENSG00000151789	559	2378	4302	2997
ENSG00000151790	0	0	0	5
ENSG00000151806	10	98	137	169
ENSG00000151812	0	0	0	0
ENSG00000151834	0	0	1	0

ENSG00000151835	5	153	118	151
ENSG00000151838	415	2216	4562	7070
ENSG00000151846	5	1	4	2
ENSG00000151849	10	222	250	284
ENSG00000151876	5	126	147	266
ENSG00000151881	3	9	26	47
ENSG00000151882	2	0	3	13
ENSG00000151883	33	557	794	1044
ENSG00000151892	0	0	0	0
ENSG00000151893	72	997	978	1324
ENSG00000151914	7	106	192	153
ENSG00000151917	1	0	4	7
ENSG00000151923	29	471	413	492
ENSG00000151929	3	0	2	0
ENSG00000151948	0	3	1	1
ENSG00000151952	0	0	0	0
ENSG00000151962	0	0	0	0
ENSG00000151963	0	0	0	0
ENSG00000151967	1	0	6	2
ENSG00000152022	24	129	95	68
ENSG00000152034	0	0	0	0
ENSG00000152042	0	13	1	0
ENSG00000152049	1	0	0	11
ENSG00000152056	3	26	34	50
ENSG00000152061	6862	37582	43240	48955
ENSG00000152076	0	0	0	0
ENSG00000152078	17	574	510	393
ENSG00000152082	3	34	14	20
ENSG00000152086	0	0	0	0
ENSG00000152092	0	0	0	0
ENSG00000152093	0	0	0	0
ENSG00000152102	51	271	258	347
ENSG00000152104	0	0	0	0
ENSG00000152117	1	0	1	1
ENSG00000152127	177	2874	3222	2894
ENSG00000152128	47	112	49	93
ENSG00000152133	36	100	127	153
ENSG00000152137	0	0	0	0
ENSG00000152147	5	0	2	0
ENSG00000152154	0	0	0	0
ENSG00000152192	0	0	0	0
ENSG00000152193	7	1021	675	1164
ENSG00000152207	0	0	0	0
ENSG00000152208	0	0	0	0
ENSG00000152213	9	0	2	0
ENSG00000152214	0	0	0	0

ENSG00000152217	5	0	1	8
ENSG00000152219	38	0	53	77
ENSG00000152223	4	317	99	145
ENSG00000152229	2815	12680	11989	12133
ENSG00000152234	204	74	77	144
ENSG00000152240	29	659	732	1361
ENSG00000152242	17	81	274	131
ENSG00000152253	17	141	219	390
ENSG00000152254	0	9	16	18
ENSG00000152256	286	12383	8311	8052
ENSG00000152266	0	0	0	0
ENSG00000152268	0	0	0	0
ENSG00000152270	4	0	18	39
ENSG00000152284	0	0	0	0
ENSG00000152291	938	3607	4095	4411
ENSG00000152292	2	0	0	1
ENSG00000152315	0	0	0	0
ENSG00000152332	213	2221	4792	4325
ENSG00000152348	31	146	220	278
ENSG00000152359	9	113	110	127
ENSG00000152377	0	0	0	0
ENSG00000152380	2	51	33	60
ENSG00000152382	8	20	31	43
ENSG00000152402	0	5	0	4
ENSG00000152404	37	645	905	1845
ENSG00000152409	15	100	109	73
ENSG00000152413	0	112	66	235
ENSG00000152422	13	242	162	283
ENSG00000152430	0	0	1	0
ENSG00000152433	0	0	0	0
ENSG00000152439	4	0	17	9
ENSG00000152443	4	37	71	85
ENSG00000152454	0	40	12	22
ENSG00000152455	1	609	434	622
ENSG00000152457	15	93	51	163
ENSG00000152463	0	0	0	0
ENSG00000152464	3	0	5	8
ENSG00000152465	36	35	144	46
ENSG00000152467	0	0	0	0
ENSG00000152475	1	0	0	0
ENSG00000152484	384	12610	16886	15168
ENSG00000152487	9	0	5	2
ENSG00000152492	39	30	38	10
ENSG00000152495	9	0	0	0
ENSG00000152503	0	0	0	0
ENSG00000152518	50	142	22	12

ENSG00000152520	70	5181	3820	5606
ENSG00000152527	0	0	0	1
ENSG00000152556	39	243	221	370
ENSG00000152558	214	4371	6508	4698
ENSG00000152578	0	0	0	0
ENSG00000152580	0	2	18	0
ENSG00000152582	2	30	9	30
ENSG00000152583	10	47	5	14
ENSG00000152591	0	0	0	0
ENSG00000152592	0	0	0	0
ENSG00000152595	0	0	1	2
ENSG00000152601	15437	98905	153607	101082
ENSG00000152611	1	29	16	21
ENSG00000152620	59	435	599	365
ENSG00000152642	74	727	738	828
ENSG00000152661	0	19	34	6
ENSG00000152669	0	0	0	0
ENSG00000152670	0	5	7	7
ENSG00000152672	1	21	0	0
ENSG00000152683	18	682	725	964
ENSG00000152684	21	13	32	65
ENSG00000152689	52	1237	1722	1657
ENSG00000152700	25	365	209	248
ENSG00000152705	0	0	0	0
ENSG00000152726	8	0	1	0
ENSG00000152749	25	451	575	392
ENSG00000152760	17	191	73	172
ENSG00000152763	1	2083	1255	3710
ENSG00000152766	2	19	0	11
ENSG00000152767	2	19	0	1
ENSG00000152778	14	99	61	53
ENSG00000152779	0	0	0	0
ENSG00000152782	2	40	25	10
ENSG00000152784	2	0	0	0
ENSG00000152785	0	0	0	0
ENSG00000152795	98	503	518	553
ENSG00000152804	168	475	473	352
ENSG00000152818	563	8915	11329	14803
ENSG00000152822	0	0	0	0
ENSG00000152894	0	0	2	0
ENSG00000152904	63	1189	1081	814
ENSG00000152910	0	0	0	0
ENSG00000152926	114	1204	2022	1004
ENSG00000152931	0	0	0	0
ENSG00000152932	52	337	353	610
ENSG00000152936	0	0	3	0

ENSG00000152939	1	0	0	4
ENSG00000152942	12	244	328	393
ENSG00000152944	13	292	397	388
ENSG00000152952	205	3331	4821	4612
ENSG00000152953	0	0	0	1
ENSG00000152954	0	0	0	0
ENSG00000152969	2	0	0	0
ENSG00000152977	0	0	0	0
ENSG00000152990	0	80	33	70
ENSG00000153002	0	0	1	0
ENSG00000153006	183	184	233	250
ENSG00000153012	0	0	2	1
ENSG00000153015	71	733	437	1116
ENSG00000153029	10	59	36	96
ENSG00000153037	8	0	9	13
ENSG00000153044	2	364	405	591
ENSG00000153046	589	5209	4078	6869
ENSG00000153048	22	61	12	28
ENSG00000153060	0	0	0	0
ENSG00000153064	1140	8526	11483	15054
ENSG00000153066	3	277	183	234
ENSG00000153071	5388	18355	19766	14829
ENSG00000153086	0	0	0	0
ENSG00000153093	0	0	0	0
ENSG00000153094	4	0	0	1
ENSG00000153107	6	199	177	460
ENSG00000153113	422	2420	3935	2945
ENSG00000153130	57	844	1149	1197
ENSG00000153132	0	1	8	13
ENSG00000153140	15	156	178	183
ENSG00000153147	172	78658	58670	105026
ENSG00000153157	0	0	9	27
ENSG00000153162	624	6704	7278	5398
ENSG00000153165	0	13	102	73
ENSG00000153179	31	218	130	117
ENSG00000153187	227	1743	1907	1925
ENSG00000153201	16	1564	1154	1874
ENSG00000153207	537	4447	2859	4793
ENSG00000153208	0	180	106	57
ENSG00000153214	75	3563	3681	3877
ENSG00000153230	0	0	0	0
ENSG00000153233	0	0	0	1
ENSG00000153234	12	35	33	23
ENSG00000153237	10	387	414	236
ENSG00000153246	0	0	0	0
ENSG00000153250	64	90	327	179

ENSG00000153253	2	0	0	0
ENSG00000153266	0	0	0	0
ENSG00000153283	46	131	322	972
ENSG00000153291	0	0	0	3
ENSG00000153292	0	0	0	0
ENSG00000153294	0	0	0	0
ENSG00000153303	0	0	0	0
ENSG00000153310	90	970	1150	1800
ENSG00000153317	1736	22887	24352	37648
ENSG00000153339	20	1353	1572	2197
ENSG00000153347	359	2754	2774	3271
ENSG00000153363	84	136	137	170
ENSG00000153391	26	30	39	34
ENSG00000153395	1	21	5	13
ENSG00000153404	0	0	0	0
ENSG00000153406	19	32	18	35
ENSG00000153443	2	1	3	4
ENSG00000153446	0	0	0	0
ENSG00000153485	5	0	1	0
ENSG00000153487	67	241	234	162
ENSG00000153495	0	0	13	29
ENSG00000153498	0	0	0	0
ENSG00000153531	0	0	0	0
ENSG00000153551	14	387	209	215
ENSG00000153558	0	0	22	32
ENSG00000153560	108	1689	1433	1884
ENSG00000153561	513	5448	7215	2239
ENSG00000153563	3	0	0	0
ENSG00000153574	71	114	124	271
ENSG00000153575	7	387	357	489
ENSG00000153666	4	54	73	94
ENSG00000153684	0	0	0	0
ENSG00000153707	13	78	135	164
ENSG00000153714	77	242	199	228
ENSG00000153721	4	41	83	81
ENSG00000153767	8	338	168	348
ENSG00000153774	30	110	94	143
ENSG00000153779	0	0	0	0
ENSG00000153786	27	346	356	318
ENSG00000153789	0	0	0	0
ENSG00000153790	13	29	78	131
ENSG00000153802	0	0	0	0
ENSG00000153814	17	14	8	14
ENSG00000153815	3059	7822	5859	5970
ENSG00000153820	0	0	0	0
ENSG00000153822	0	0	0	0

ENSG00000153823	16	0	0	0
ENSG00000153827	279	5405	5316	5319
ENSG00000153832	0	0	3	4
ENSG00000153879	14	101	10	14
ENSG00000153885	2	0	0	0
ENSG00000153896	8	5	9	3
ENSG00000153898	3	0	10	5
ENSG00000153902	2	0	0	1
ENSG00000153904	45	565	152	209
ENSG00000153914	182	449	409	460
ENSG00000153922	42	747	735	1195
ENSG00000153923	1	6	1	0
ENSG00000153930	0	13	4	3
ENSG00000153933	6	3417	3824	6027
ENSG00000153936	75	601	1053	600
ENSG00000153944	36	150	279	252
ENSG00000153956	1	0	1	0
ENSG00000153975	10	543	628	804
ENSG00000153976	0	0	0	0
ENSG00000153982	8	329	236	411
ENSG00000153989	17	121	347	513
ENSG00000153993	0	18	22	42
ENSG00000154001	114	276	329	359
ENSG00000154007	0	0	0	0
ENSG00000154016	1	0	0	0
ENSG00000154025	0	0	0	0
ENSG00000154027	7	23	68	6
ENSG00000154035	7	0	2	0
ENSG00000154040	0	0	0	0
ENSG00000154059	12	238	277	249
ENSG00000154065	0	0	0	1
ENSG00000154079	5	21	13	24
ENSG00000154080	0	0	0	0
ENSG00000154096	0	0	0	0
ENSG00000154099	0	0	1	6
ENSG00000154102	1	0	0	0
ENSG00000154114	21	1021	1283	1476
ENSG00000154118	0	0	0	0
ENSG00000154122	138	283	125	389
ENSG00000154124	37	251	428	264
ENSG00000154127	395	2401	3596	3070
ENSG00000154133	1	0	0	0
ENSG00000154134	0	0	3	0
ENSG00000154143	0	0	0	0
ENSG00000154144	62	221	279	319
ENSG00000154146	8916	52908	27893	45904

ENSG00000154153	9	79	48	64
ENSG00000154162	3	0	0	0
ENSG00000154165	0	0	0	0
ENSG00000154174	21	410	430	407
ENSG00000154175	0	19	0	0
ENSG00000154188	780	10522	16464	15921
ENSG00000154198	0	0	0	0
ENSG00000154217	36	1368	1717	3434
ENSG00000154222	33	107	163	214
ENSG00000154227	0	0	4	0
ENSG00000154229	144	2183	1992	3278
ENSG00000154237	11	147	68	160
ENSG00000154240	1	0	20	20
ENSG00000154252	0	0	0	0
ENSG00000154258	11	93	108	34
ENSG00000154262	26	314	323	213
ENSG00000154263	1	0	39	42
ENSG00000154265	0	510	764	863
ENSG00000154269	0	0	3	2
ENSG00000154274	0	0	0	0
ENSG00000154277	0	0	0	0
ENSG00000154305	36	264	205	308
ENSG00000154309	2	8	10	28
ENSG00000154310	544	3912	3594	3426
ENSG00000154316	0	0	0	0
ENSG00000154319	2	0	0	0
ENSG00000154328	12	7	0	0
ENSG00000154330	32	27	14	127
ENSG00000154342	0	0	0	0
ENSG00000154358	0	0	0	0
ENSG00000154359	3	20	63	57
ENSG00000154370	6	0	0	2
ENSG00000154380	7	12	28	77
ENSG00000154415	0	0	0	0
ENSG00000154429	51	1120	1446	1024
ENSG00000154438	0	0	0	0
ENSG00000154447	2	90	59	180
ENSG00000154451	39	39	94	73
ENSG00000154473	62	187	122	151
ENSG00000154478	0	0	0	0
ENSG00000154479	4	20	3	45
ENSG00000154485	0	0	0	5
ENSG00000154493	0	0	0	0
ENSG00000154511	1	0	9	10
ENSG00000154518	101	68	61	97
ENSG00000154529	0	0	0	0

ENSG00000154537	0	0	0	0
ENSG00000154545	0	0	0	0
ENSG00000154548	0	0	0	0
ENSG00000154553	0	0	0	0
ENSG00000154556	58	113	296	356
ENSG00000154582	155	191	268	315
ENSG00000154589	13	4	0	5
ENSG00000154608	0	83	42	89
ENSG00000154611	0	0	2	0
ENSG00000154620	768	4345	8	2776
ENSG00000154639	1	0	3	0
ENSG00000154640	60	64	66	40
ENSG00000154642	19	371	702	561
ENSG00000154645	0	0	1	0
ENSG00000154646	0	0	0	0
ENSG00000154654	1	0	1	7
ENSG00000154655	4	507	674	915
ENSG00000154678	0	0	0	0
ENSG00000154710	1	0	0	0
ENSG00000154719	7	117	51	106
ENSG00000154721	5	135	31	168
ENSG00000154723	793	775	465	1055
ENSG00000154727	61	942	1419	1599
ENSG00000154734	1	0	0	0
ENSG00000154736	0	0	0	0
ENSG00000154743	1	281	312	418
ENSG00000154760	5	35	29	26
ENSG00000154764	0	0	0	3
ENSG00000154767	10	0	5	15
ENSG00000154768	4	0	1	0
ENSG00000154781	65	55	47	61
ENSG00000154783	1	8	1	0
ENSG00000154803	2	21	3	3
ENSG00000154813	50	313	202	157
ENSG00000154814	9	1690	1139	1647
ENSG00000154822	39	121	96	107
ENSG00000154832	21	0	7	11
ENSG00000154839	2	29	16	30
ENSG00000154845	32	806	1226	897
ENSG00000154856	154	0	11	3
ENSG00000154864	2	48	25	75
ENSG00000154874	1	117	96	91
ENSG00000154889	14	13	26	23
ENSG00000154898	2	13	59	76
ENSG00000154914	0	0	2	0
ENSG00000154917	313	2187	1383	1893

ENSG00000154920	1	0	17	4
ENSG00000154928	3	43	36	1
ENSG00000154930	23	33	23	25
ENSG00000154945	8	47	128	244
ENSG00000154957	2	0	3	3
ENSG00000154975	0	0	0	0
ENSG00000154978	210	1111	787	678
ENSG00000154997	0	0	2	4
ENSG00000155008	6	3696	3265	5349
ENSG00000155011	0	0	0	0
ENSG00000155016	1	0	3	3
ENSG00000155026	0	0	2	4
ENSG00000155034	0	0	0	5
ENSG00000155052	0	0	0	0
ENSG00000155066	0	0	0	0
ENSG00000155070	0	0	0	0
ENSG00000155085	67	1578	1442	1982
ENSG00000155087	0	0	0	0
ENSG00000155090	56	370	153	145
ENSG00000155093	7	0	1	2
ENSG00000155096	469	10810	14897	15032
ENSG00000155097	43	1308	1042	1496
ENSG00000155099	2325	9969	13256	9217
ENSG00000155100	5	57	55	74
ENSG00000155111	293	1927	2896	2553
ENSG00000155115	1018	3174	2877	3864
ENSG00000155130	82	53	31	35
ENSG00000155158	342	3172	6196	3978
ENSG00000155189	60	920	798	1461
ENSG00000155229	8	175	38	76
ENSG00000155249	0	0	0	0
ENSG00000155252	3	0	0	3
ENSG00000155254	6	0	10	4
ENSG00000155256	0	32	0	6
ENSG00000155265	2	0	0	0
ENSG00000155269	0	0	0	0
ENSG00000155275	0	1	6	6
ENSG00000155282	0	0	6	6
ENSG00000155287	17	16	4	4
ENSG00000155304	4	271	427	378
ENSG00000155307	13	80	108	65
ENSG00000155313	37	3238	3820	6924
ENSG00000155324	0	0	5	2
ENSG00000155329	144	137	286	306
ENSG00000155330	115	1267	1189	715
ENSG00000155363	7	0	5	2

ENSG00000155366	913	499	302	253
ENSG00000155367	3	0	4	5
ENSG00000155368	159	23	9	49
ENSG00000155380	15	193	89	42
ENSG00000155393	2	236	179	314
ENSG00000155428	0	0	0	0
ENSG00000155438	30	7	47	105
ENSG00000155463	45	0	9	8
ENSG00000155465	49	0	1	0
ENSG00000155495	0	0	0	0
ENSG00000155506	174	375	356	427
ENSG00000155508	28	619	724	1086
ENSG00000155511	0	0	0	0
ENSG00000155530	0	159	53	248
ENSG00000155542	17	112	81	97
ENSG00000155545	4	53	100	94
ENSG00000155561	6	357	247	578
ENSG00000155592	0	0	2	18
ENSG00000155621	17	63	78	107
ENSG00000155622	0	0	0	10
ENSG00000155629	120	202	132	180
ENSG00000155636	6	129	114	107
ENSG00000155640	245	1237	1368	1241
ENSG00000155657	4	104	32	51
ENSG00000155659	5	38	82	14
ENSG00000155660	1	545	353	505
ENSG00000155666	0	24	9	90
ENSG00000155714	0	0	0	0
ENSG00000155719	0	0	0	0
ENSG00000155729	101	785	490	828
ENSG00000155744	43	817	807	927
ENSG00000155749	43	422	438	697
ENSG00000155754	0	93	51	116
ENSG00000155755	0	0	1	8
ENSG00000155760	0	0	0	0
ENSG00000155761	0	0	0	0
ENSG00000155792	3	0	63	42
ENSG00000155816	0	0	2	0
ENSG00000155827	55	37	45	90
ENSG00000155833	0	0	0	0
ENSG00000155846	3	0	1	0
ENSG00000155849	207	4703	2212	2792
ENSG00000155850	4	22	25	20
ENSG00000155858	2	0	6	4
ENSG00000155868	15	36	24	24
ENSG00000155875	0	0	0	0

ENSG00000155876	20	36	50	65
ENSG00000155886	0	0	0	0
ENSG00000155890	0	0	0	0
ENSG00000155893	2	68	27	36
ENSG00000155897	0	0	0	0
ENSG00000155903	16	1603	1493	2281
ENSG00000155906	5	184	267	305
ENSG00000155918	0	0	0	0
ENSG00000155926	318	687	231	390
ENSG00000155957	110	25	143	140
ENSG00000155959	66	329	644	775
ENSG00000155961	7	0	4	0
ENSG00000155962	12	37	17	11
ENSG00000155966	2	144	137	115
ENSG00000155970	1	20	105	75
ENSG00000155974	2	17	29	62
ENSG00000155975	35	5818	4650	5959
ENSG00000155980	0	0	1	4
ENSG00000155984	818	1768	1085	1043
ENSG00000156006	0	0	0	0
ENSG00000156009	0	0	0	0
ENSG00000156011	323	4876	5030	9055
ENSG00000156017	10	2940	2767	4003
ENSG00000156026	118	2410	1996	2865
ENSG00000156030	17	218	85	82
ENSG00000156042	0	29	27	35
ENSG00000156049	0	0	2	1
ENSG00000156050	1	0	1	1
ENSG00000156052	1925	16832	18525	25304
ENSG00000156076	0	0	0	0
ENSG00000156096	0	0	0	0
ENSG00000156097	0	0	0	0
ENSG00000156103	0	0	0	0
ENSG00000156110	17	213	154	299
ENSG00000156113	2	29	6	10
ENSG00000156127	3	0	0	1
ENSG00000156136	208	2536	3175	1332
ENSG00000156140	0	28	17	9
ENSG00000156150	0	0	0	0
ENSG00000156162	3	672	621	851
ENSG00000156170	27	724	883	1173
ENSG00000156171	29	116	115	111
ENSG00000156172	3	96	100	84
ENSG00000156194	0	0	0	0
ENSG00000156206	520	2865	2246	5378
ENSG00000156218	3	50	92	67

ENSG00000156219	0	0	0	0
ENSG00000156222	0	0	0	0
ENSG00000156232	28	1053	939	1322
ENSG00000156234	0	0	0	0
ENSG00000156239	4	16	4	14
ENSG00000156253	3	0	20	32
ENSG00000156256	126	180	127	170
ENSG00000156261	99	703	661	732
ENSG00000156265	57961	204796	152459	95874
ENSG00000156269	1	4	0	1
ENSG00000156273	187	2130	2455	1912
ENSG00000156282	0	0	0	0
ENSG00000156284	0	0	0	0
ENSG00000156298	2	0	0	0
ENSG00000156299	7	0	2	6
ENSG00000156304	23	94	136	194
ENSG00000156313	3	32	41	96
ENSG00000156345	0	0	0	0
ENSG00000156374	1	424	380	575
ENSG00000156381	14	532	211	487
ENSG00000156384	11	0	30	67
ENSG00000156395	1	0	0	0
ENSG00000156398	2	0	1	0
ENSG00000156411	1323	2979	1710	3545
ENSG00000156413	0	0	0	0
ENSG00000156414	1	53	39	70
ENSG00000156427	0	0	0	0
ENSG00000156453	2	0	0	0
ENSG00000156463	0	0	0	2
ENSG00000156466	0	0	0	0
ENSG00000156467	726	1606	1018	1667
ENSG00000156469	5	87	45	134
ENSG00000156471	80	998	577	1157
ENSG00000156475	17	0	4	0
ENSG00000156482	437	1188	626	1327
ENSG00000156486	0	0	0	0
ENSG00000156500	20	136	136	208
ENSG00000156502	2	271	174	266
ENSG00000156504	491	2981	4822	4804
ENSG00000156508	2053	3616	3659	4038
ENSG00000156509	0	21	32	54
ENSG00000156510	0	0	0	0
ENSG00000156515	1054	7446	4238	6540
ENSG00000156521	5	0	1	0
ENSG00000156531	25	187	289	463
ENSG00000156535	39	20701	3482	5236

ENSG00000156564	0	0	0	0
ENSG00000156574	0	0	0	0
ENSG00000156575	0	0	0	0
ENSG00000156587	239	330	319	185
ENSG00000156599	21	233	222	186
ENSG00000156603	5	0	2	4
ENSG00000156639	365	1859	1476	2155
ENSG00000156642	1480	13545	13425	13361
ENSG00000156650	35	288	148	269
ENSG00000156671	9	334	258	276
ENSG00000156675	51	0	16	12
ENSG00000156687	0	0	0	0
ENSG00000156689	46	734	1897	696
ENSG00000156697	15	0	10	8
ENSG00000156709	13	88	27	36
ENSG00000156711	4	3	3	3
ENSG00000156735	31	80	150	131
ENSG00000156738	27	0	0	1
ENSG00000156750	1	0	0	0
ENSG00000156755	0	0	0	0
ENSG00000156787	15	8131	7753	9369
ENSG00000156795	9	9	22	35
ENSG00000156802	16	2286	2109	3620
ENSG00000156804	7	0	0	0
ENSG00000156831	11	380	362	453
ENSG00000156853	45	47	45	44
ENSG00000156858	36	61	36	72
ENSG00000156860	88	234	166	272
ENSG00000156869	2	35	81	25
ENSG00000156873	17	0	5	4
ENSG00000156875	31	745	848	735
ENSG00000156876	20	320	379	446
ENSG00000156885	0	0	0	0
ENSG00000156886	0	0	0	0
ENSG00000156920	0	0	0	5
ENSG00000156925	0	0	0	0
ENSG00000156928	3	219	179	122
ENSG00000156931	117	9599	8195	8869
ENSG00000156958	20	1769	2189	3208
ENSG00000156959	0	0	0	0
ENSG00000156966	0	0	0	0
ENSG00000156968	0	0	0	1
ENSG00000156970	2	82	216	401
ENSG00000156973	12	126	39	78
ENSG00000156976	236	2245	3416	3231
ENSG00000156983	5	36	2	12

ENSG00000156990	10	0	3	3
ENSG00000157005	0	0	0	0
ENSG00000157014	78	98	85	139
ENSG00000157017	4	20	0	0
ENSG00000157020	48	11	24	35
ENSG00000157021	0	0	0	0
ENSG00000157036	3	49	34	86
ENSG00000157045	331	914	850	1646
ENSG00000157060	0	0	0	0
ENSG00000157064	0	0	2	3
ENSG00000157077	1	15	1	0
ENSG00000157087	6	11	16	37
ENSG00000157093	0	0	0	0
ENSG00000157103	0	0	0	0
ENSG00000157106	67	2886	2617	3033
ENSG00000157107	12	3295	3027	5162
ENSG00000157110	1	18	0	1
ENSG00000157111	0	0	0	0
ENSG00000157119	0	0	0	0
ENSG00000157131	0	0	0	0
ENSG00000157150	0	0	0	0
ENSG00000157152	0	15	1	0
ENSG00000157168	2	0	0	0
ENSG00000157181	10	578	998	1652
ENSG00000157184	2	15	8	17
ENSG00000157191	132	257	154	220
ENSG00000157193	6	282	172	299
ENSG00000157211	0	0	0	0
ENSG00000157212	1	42	28	71
ENSG00000157214	1	0	2	0
ENSG00000157216	368	292	156	99
ENSG00000157219	0	0	0	3
ENSG00000157224	0	8	3	22
ENSG00000157227	0	0	0	1
ENSG00000157240	1	0	0	0
ENSG00000157259	96	74	93	136
ENSG00000157303	343	912	565	718
ENSG00000157306	0	0	0	0
ENSG00000157315	1	0	1	0
ENSG00000157322	0	0	0	0
ENSG00000157326	1	10	3	3
ENSG00000157330	0	0	0	0
ENSG00000157335	0	0	0	0
ENSG00000157343	0	0	0	0
ENSG00000157349	10	9	6	17
ENSG00000157350	5	56	7	35

ENSG00000157353	1	0	1	3
ENSG00000157358	0	0	0	0
ENSG00000157368	0	0	0	0
ENSG00000157379	28	20	6	0
ENSG00000157388	1	29	11	19
ENSG00000157399	0	9	8	16
ENSG00000157404	0	0	2	5
ENSG00000157423	0	0	2	1
ENSG00000157426	10	148	141	287
ENSG00000157429	0	0	2	3
ENSG00000157445	1	0	2	5
ENSG00000157450	5	235	345	515
ENSG00000157456	4	0	11	10
ENSG00000157470	1	0	0	0
ENSG00000157483	4	117	28	148
ENSG00000157500	182	5898	5921	8744
ENSG00000157502	0	0	0	0
ENSG00000157510	6	30	11	3
ENSG00000157514	5333	14255	11139	9555
ENSG00000157538	178	728	1046	674
ENSG00000157540	146	6110	5302	6813
ENSG00000157542	0	0	0	0
ENSG00000157551	0	7	1	1
ENSG00000157554	7	49	19	20
ENSG00000157557	38	87	97	86
ENSG00000157570	1526	2648	1567	1329
ENSG00000157578	0	0	1	4
ENSG00000157593	6	0	0	0
ENSG00000157600	476	3039	2323	2742
ENSG00000157601	47	4	8	5
ENSG00000157613	2	0	2	0
ENSG00000157617	0	0	14	24
ENSG00000157625	111	1100	1429	939
ENSG00000157637	10	52	48	68
ENSG00000157653	0	0	8	8
ENSG00000157654	0	0	0	0
ENSG00000157657	6	0	21	57
ENSG00000157680	13	89	129	162
ENSG00000157693	1	0	3	6
ENSG00000157703	0	0	4	0
ENSG00000157734	3	13	9	20
ENSG00000157741	18	1263	1187	1956
ENSG00000157764	13	2429	2779	3208
ENSG00000157765	0	0	2	0
ENSG00000157766	0	0	0	0
ENSG00000157778	50	31	20	4

ENSG00000157782	0	0	0	0
ENSG00000157796	1	18	34	52
ENSG00000157800	0	50	23	64
ENSG00000157823	10	11	9	19
ENSG00000157827	7	24	73	77
ENSG00000157828	0	0	0	0
ENSG00000157833	1	0	0	0
ENSG00000157837	1043	12809	10681	10121
ENSG00000157851	0	0	0	0
ENSG00000157856	0	0	2	0
ENSG00000157869	7	130	120	205
ENSG00000157870	53	64	56	32
ENSG00000157873	33	84	74	72
ENSG00000157881	1	16	5	1
ENSG00000157884	0	0	0	0
ENSG00000157890	0	0	0	0
ENSG00000157895	19	0	2	5
ENSG00000157911	9	4	10	8
ENSG00000157916	33	97	96	140
ENSG00000157927	10	11	8	9
ENSG00000157933	48	258	103	358
ENSG00000157950	0	0	0	0
ENSG00000157954	155	362	252	182
ENSG00000157965	0	0	0	0
ENSG00000157978	1394	3796	2349	2713
ENSG00000157985	9	0	7	8
ENSG00000157992	0	0	0	0
ENSG00000157999	0	0	1	0
ENSG00000158006	10	43	5	12
ENSG00000158008	0	21	0	0
ENSG00000158014	0	0	0	0
ENSG00000158019	181	161	300	322
ENSG00000158022	0	0	0	0
ENSG00000158023	0	0	3	8
ENSG00000158042	12	0	21	77
ENSG00000158050	1	0	0	0
ENSG00000158055	0	0	0	0
ENSG00000158062	586	1998	1188	891
ENSG00000158077	0	0	0	0
ENSG00000158079	0	19	1	15
ENSG00000158089	2	17	8	13
ENSG00000158092	40	257	290	205
ENSG00000158104	2	0	6	0
ENSG00000158106	0	0	0	0
ENSG00000158109	286	371	458	379
ENSG00000158113	0	33	6	17

ENSG00000158122	38	176	126	91
ENSG00000158125	0	0	0	0
ENSG00000158156	2	0	1	3
ENSG00000158158	6	70	31	30
ENSG00000158161	5	946	734	1311
ENSG00000158163	2	36	0	0
ENSG00000158164	151	216	174	295
ENSG00000158169	0	318	83	185
ENSG00000158186	9	0	1	3
ENSG00000158195	587	654	481	770
ENSG00000158201	19	162	221	246
ENSG00000158220	0	0	0	0
ENSG00000158234	3	494	227	484
ENSG00000158246	0	0	0	0
ENSG00000158258	0	0	2	0
ENSG00000158270	2	14	8	5
ENSG00000158286	3	0	0	0
ENSG00000158290	121	2522	2707	2466
ENSG00000158292	0	0	0	0
ENSG00000158296	0	0	0	0
ENSG00000158301	3	18	5	7
ENSG00000158315	5	0	3	0
ENSG00000158321	10	83	69	279
ENSG00000158352	54	412	233	441
ENSG00000158373	1163	4949	2268	3026
ENSG00000158402	2	156	146	261
ENSG00000158406	2251	10924	4972	3847
ENSG00000158411	44	2727	4068	7476
ENSG00000158417	169	307	143	217
ENSG00000158423	0	0	0	0
ENSG00000158427	5	49	16	14
ENSG00000158428	0	0	0	0
ENSG00000158435	11	694	342	805
ENSG00000158445	1	0	1	0
ENSG00000158457	7032	12567	7419	8730
ENSG00000158458	3	0	3	0
ENSG00000158467	6	228	319	415
ENSG00000158470	4	240	154	206
ENSG00000158473	6	0	3	9
ENSG00000158477	0	0	0	0
ENSG00000158480	3	6	17	6
ENSG00000158481	15	24	18	11
ENSG00000158482	0	13	2	8
ENSG00000158483	3	0	1	3
ENSG00000158485	0	0	0	0
ENSG00000158486	0	0	1	3

ENSG00000158488	12	0	9	28
ENSG00000158497	0	0	0	0
ENSG00000158516	2	0	0	0
ENSG00000158517	1	0	0	0
ENSG00000158525	15	64	8	2
ENSG00000158526	129	72	39	96
ENSG00000158528	1	33	27	56
ENSG00000158545	6	105	59	138
ENSG00000158552	242	773	506	528
ENSG00000158553	0	0	0	0
ENSG00000158555	3	0	1	2
ENSG00000158560	13	208	198	459
ENSG00000158571	0	0	2	6
ENSG00000158578	18	137	130	51
ENSG00000158604	184	464	440	708
ENSG00000158615	12	249	165	141
ENSG00000158623	6	91	74	112
ENSG00000158636	19	2139	1595	2679
ENSG00000158639	0	0	0	0
ENSG00000158669	38	239	168	110
ENSG00000158683	1	11	0	3
ENSG00000158691	0	20	27	30
ENSG00000158710	74560	83556	58321	92462
ENSG00000158711	87	1017	1430	1241
ENSG00000158714	5	0	2	0
ENSG00000158715	40	96	152	68
ENSG00000158716	28	68	13	25
ENSG00000158717	4	13	22	24
ENSG00000158731	0	0	0	0
ENSG00000158747	0	0	0	0
ENSG00000158748	0	0	0	0
ENSG00000158764	0	0	4	0
ENSG00000158769	907	2750	1574	1940
ENSG00000158773	35	89	42	59
ENSG00000158786	0	0	0	0
ENSG00000158792	0	0	3	1
ENSG00000158793	118	197	239	207
ENSG00000158796	455	992	665	848
ENSG00000158805	1	60	44	209
ENSG00000158806	4	20	4	6
ENSG00000158813	0	0	0	0
ENSG00000158815	0	0	0	0
ENSG00000158816	0	0	0	0
ENSG00000158825	6	0	0	0
ENSG00000158828	5	156	173	121
ENSG00000158850	20	30	18	37

ENSG00000158856	2128	6169	3483	4727
ENSG00000158859	3	0	0	0
ENSG00000158863	0	29	0	5
ENSG00000158864	64	69	28	42
ENSG00000158865	0	0	0	0
ENSG00000158869	3696	5985	10428	9778
ENSG00000158874	0	0	0	0
ENSG00000158882	2	0	6	38
ENSG00000158887	8	21	15	30
ENSG00000158901	0	0	0	0
ENSG00000158941	5	18	7	13
ENSG00000158955	1	0	0	0
ENSG00000158966	1	393	125	325
ENSG00000158985	1688	20953	23499	10097
ENSG00000158987	3	0	10	0
ENSG00000159023	394	10901	11171	6979
ENSG00000159055	1	0	1	2
ENSG00000159063	13	616	339	450
ENSG00000159069	93	612	739	1489
ENSG00000159079	1	0	0	1
ENSG00000159082	4	453	347	414
ENSG00000159086	10	309	251	368
ENSG00000159110	6	11	44	72
ENSG00000159111	18	0	12	44
ENSG00000159123	0	0	0	0
ENSG00000159128	14	76	63	116
ENSG00000159131	20	37	74	62
ENSG00000159140	395	800	834	818
ENSG00000159147	1	92	74	149
ENSG00000159164	1	0	0	12
ENSG00000159166	1	0	0	0
ENSG00000159167	0	19	0	0
ENSG00000159173	0	0	0	0
ENSG00000159176	316	396	442	630
ENSG00000159182	0	0	0	0
ENSG00000159184	0	0	0	0
ENSG00000159186	0	0	0	0
ENSG00000159189	0	0	0	0
ENSG00000159197	1	0	3	0
ENSG00000159199	41	0	1	0
ENSG00000159200	25	25	40	80
ENSG00000159202	196	240	228	248
ENSG00000159208	0	0	0	0
ENSG00000159210	26	17	13	28
ENSG00000159212	0	0	0	0
ENSG00000159214	0	0	0	0

ENSG00000159216	77	1567	1768	1448
ENSG00000159217	0	0	0	0
ENSG00000159224	0	0	0	0
ENSG00000159228	36	129	71	43
ENSG00000159231	0	0	0	4
ENSG00000159239	3	0	0	0
ENSG00000159247	0	0	1	0
ENSG00000159248	0	0	0	0
ENSG00000159251	0	0	0	0
ENSG00000159256	91	54151	45960	67513
ENSG00000159259	0	36	70	63
ENSG00000159261	0	0	0	0
ENSG00000159263	0	0	1	0
ENSG00000159267	3	0	18	4
ENSG00000159289	0	0	0	0
ENSG00000159307	0	0	0	0
ENSG00000159314	20	16	4	4
ENSG00000159322	1	133	122	232
ENSG00000159335	96	303	247	268
ENSG00000159337	0	0	0	0
ENSG00000159339	1846	1907	765	1220
ENSG00000159346	2410	15141	16028	14750
ENSG00000159348	2219	2629	2205	3139
ENSG00000159352	133	287	139	210
ENSG00000159363	3	17	20	17
ENSG00000159374	137	117	70	133
ENSG00000159377	171	90	71	150
ENSG00000159387	0	0	0	0
ENSG00000159388	3	0	5	0
ENSG00000159398	0	0	0	0
ENSG00000159399	2	0	3	0
ENSG00000159403	0	0	0	0
ENSG00000159409	0	0	1	0
ENSG00000159423	0	0	0	0
ENSG00000159433	0	31	24	45
ENSG00000159445	10	39	21	36
ENSG00000159450	1	0	17	2
ENSG00000159455	0	0	0	0
ENSG00000159459	36	2739	2789	3774
ENSG00000159461	375	5307	4925	2289
ENSG00000159479	34	62	49	59
ENSG00000159495	0	0	0	0
ENSG00000159496	11	0	1	2
ENSG00000159516	0	0	0	0
ENSG00000159527	0	0	0	0
ENSG00000159556	0	0	0	0

ENSG00000159579	23	140	77	125
ENSG00000159588	3	0	1	6
ENSG00000159592	178	3092	2147	2806
ENSG00000159593	172	2258	2372	2304
ENSG00000159596	10	10	131	56
ENSG00000159618	12	0	0	0
ENSG00000159625	116	97	55	182
ENSG00000159640	0	1	0	0
ENSG00000159648	23	0	6	1
ENSG00000159650	0	0	0	0
ENSG00000159658	340	1446	1820	2614
ENSG00000159674	9	0	0	0
ENSG00000159685	0	0	0	0
ENSG00000159692	210	361	137	266
ENSG00000159708	0	0	1	2
ENSG00000159712	0	16	8	13
ENSG00000159713	9	0	0	0
ENSG00000159714	0	0	0	3
ENSG00000159720	424	418	302	468
ENSG00000159723	0	0	0	0
ENSG00000159733	3	0	2	1
ENSG00000159753	1	0	0	5
ENSG00000159761	1	43	12	8
ENSG00000159763	0	0	0	0
ENSG00000159784	0	55	4	7
ENSG00000159788	4	15	4	11
ENSG00000159792	5	41	11	46
ENSG00000159840	3417	2257	1516	2434
ENSG00000159842	18	182	41	103
ENSG00000159860	0	0	1	0
ENSG00000159871	0	0	0	0
ENSG00000159873	9	42	52	99
ENSG00000159882	0	0	5	2
ENSG00000159884	0	89	29	20
ENSG00000159885	0	0	0	5
ENSG00000159899	0	10	0	1
ENSG00000159904	0	0	0	0
ENSG00000159905	2	0	7	13
ENSG00000159915	0	0	3	1
ENSG00000159917	2	56	25	23
ENSG00000159921	4	0	12	29
ENSG00000159958	0	0	0	0
ENSG00000159961	0	0	0	0
ENSG00000160007	51	356	287	508
ENSG00000160013	402	653	531	352
ENSG00000160014	12696	42030	24029	47293

ENSG00000160049	20	24	17	38
ENSG00000160050	53	18	41	84
ENSG00000160051	1	0	2	0
ENSG00000160055	30	110	37	58
ENSG00000160058	371	1863	1776	2157
ENSG00000160062	0	0	0	0
ENSG00000160072	0	13	1	0
ENSG00000160075	297	317	207	367
ENSG00000160087	12	25	64	51
ENSG00000160094	0	955	548	1578
ENSG00000160097	0	0	0	0
ENSG00000160111	64	172	81	20
ENSG00000160113	0	0	0	0
ENSG00000160117	3	0	0	1
ENSG00000160124	4	14	15	62
ENSG00000160131	61	939	990	1718
ENSG00000160145	138	1612	1153	1793
ENSG00000160161	0	0	0	0
ENSG00000160172	1	20	3	14
ENSG00000160179	2	0	0	0
ENSG00000160180	0	0	0	0
ENSG00000160181	0	0	0	0
ENSG00000160182	0	0	0	0
ENSG00000160183	2	0	0	0
ENSG00000160185	10	0	3	1
ENSG00000160188	68	154	95	110
ENSG00000160190	94	1342	1363	1732
ENSG00000160191	5	0	0	0
ENSG00000160193	2	0	0	0
ENSG00000160194	35	13	2	1
ENSG00000160199	11	50	63	117
ENSG00000160200	0	0	0	0
ENSG00000160201	61	53	92	152
ENSG00000160202	0	0	0	0
ENSG00000160207	0	0	2	6
ENSG00000160208	10	0	8	1
ENSG00000160209	13	0	4	8
ENSG00000160211	76	704	265	357
ENSG00000160213	91	0	20	7
ENSG00000160214	4	0	0	0
ENSG00000160216	35	217	207	327
ENSG00000160218	89	928	574	804
ENSG00000160219	38	338	252	211
ENSG00000160221	96	42	24	35
ENSG00000160223	0	0	0	0
ENSG00000160224	0	0	0	0

ENSG00000160226	0	0	0	0
ENSG00000160229	3	44	151	357
ENSG00000160233	0	0	0	0
ENSG00000160255	55	0	1	1
ENSG00000160256	2	0	4	9
ENSG00000160271	33	81	44	23
ENSG00000160282	1	0	0	0
ENSG00000160284	2	0	0	0
ENSG00000160285	4	0	1	19
ENSG00000160293	2	6	3	8
ENSG00000160294	3	53	32	22
ENSG00000160298	120	222	116	76
ENSG00000160299	26	2465	749	1733
ENSG00000160305	27	69	27	59
ENSG00000160307	125	123	7	41
ENSG00000160310	613	908	998	1234
ENSG00000160318	3	0	0	0
ENSG00000160321	0	0	0	0
ENSG00000160323	0	0	0	0
ENSG00000160325	0	0	0	0
ENSG00000160326	5	0	0	0
ENSG00000160336	9	43	64	135
ENSG00000160339	0	0	0	0
ENSG00000160345	2	0	1	0
ENSG00000160349	0	0	0	0
ENSG00000160352	43	125	381	418
ENSG00000160360	0	0	0	0
ENSG00000160392	3	35	6	36
ENSG00000160396	0	0	0	2
ENSG00000160401	0	0	0	0
ENSG00000160404	4	0	1	0
ENSG00000160408	18	0	25	31
ENSG00000160410	37	107	60	125
ENSG00000160439	0	0	4	0
ENSG00000160445	104	364	133	306
ENSG00000160446	19	74	30	43
ENSG00000160447	0	0	2	5
ENSG00000160460	0	0	0	0
ENSG00000160469	0	0	4	3
ENSG00000160471	0	0	0	0
ENSG00000160472	0	0	0	0
ENSG00000160505	0	0	0	0
ENSG00000160539	0	0	3	0
ENSG00000160551	563	18740	18301	28461
ENSG00000160563	7	17	59	67
ENSG00000160570	18	68	11	12

ENSG00000160584	47	372	360	406
ENSG00000160588	29	797	1746	1936
ENSG00000160593	44	49	6	5
ENSG00000160602	3	0	0	0
ENSG00000160606	0	0	0	0
ENSG00000160613	58	88	95	257
ENSG00000160633	6	734	409	902
ENSG00000160654	17	0	2	0
ENSG00000160678	0	0	3	0
ENSG00000160679	22	101	73	82
ENSG00000160683	0	0	0	0
ENSG00000160685	48	369	453	684
ENSG00000160688	25	90	35	53
ENSG00000160691	59	131	60	77
ENSG00000160695	18	40	53	112
ENSG00000160703	33	113	45	121
ENSG00000160710	133	545	419	502
ENSG00000160712	79	117	96	95
ENSG00000160714	585	1544	1297	1806
ENSG00000160716	0	0	0	0
ENSG00000160741	34	6	9	18
ENSG00000160746	12	962	800	851
ENSG00000160752	8	71	29	59
ENSG00000160753	12	12	5	10
ENSG00000160766	1	0	0	4
ENSG00000160767	99	108	35	42
ENSG00000160781	0	0	0	0
ENSG00000160783	21	0	3	0
ENSG00000160785	18	38	30	13
ENSG00000160789	489	4133	1647	2732
ENSG00000160791	4	0	0	0
ENSG00000160796	19	452	277	494
ENSG00000160799	47	45	9	13
ENSG00000160801	0	0	0	0
ENSG00000160803	26	0	56	68
ENSG00000160808	0	0	0	0
ENSG00000160813	6	94	23	29
ENSG00000160818	24	0	2	4
ENSG00000160828	3	30	35	72
ENSG00000160838	4	0	2	12
ENSG00000160844	0	0	0	0
ENSG00000160856	9	0	0	0
ENSG00000160862	0	0	0	0
ENSG00000160867	0	0	0	0
ENSG00000160868	0	0	0	0
ENSG00000160870	0	0	0	0

ENSG00000160877	3	50	26	38
ENSG00000160882	0	0	0	0
ENSG00000160883	8	0	1	4
ENSG00000160886	0	0	0	0
ENSG00000160888	15	34	5	7
ENSG00000160908	23	495	287	569
ENSG00000160917	6	0	3	10
ENSG00000160932	38	0	2	8
ENSG00000160948	2238	759	406	582
ENSG00000160949	10	0	3	2
ENSG00000160951	0	0	0	0
ENSG00000160953	0	0	11	7
ENSG00000160957	0	0	0	0
ENSG00000160959	9	0	2	7
ENSG00000160961	2	0	9	16
ENSG00000160963	0	0	0	0
ENSG00000160972	1	1	3	19
ENSG00000160973	0	0	0	0
ENSG00000160991	2024	1220	656	954
ENSG00000160993	1	0	0	0
ENSG00000160994	0	0	0	0
ENSG00000160999	1	53	32	28
ENSG00000161010	101	349	238	125
ENSG00000161011	1014	4642	3841	3238
ENSG00000161013	154	4874	3261	5957
ENSG00000161016	1280	61	11	116
ENSG00000161021	7	137	25	79
ENSG00000161031	2	8	0	0
ENSG00000161036	0	0	1	12
ENSG00000161040	8	1360	1115	1749
ENSG00000161048	8	214	111	255
ENSG00000161055	0	0	0	0
ENSG00000161057	31	37	149	150
ENSG00000161082	0	0	0	0
ENSG00000161091	3	0	9	5
ENSG00000161103	0	0	0	0
ENSG00000161132	0	0	0	0
ENSG00000161133	0	0	0	0
ENSG00000161149	0	0	1	1
ENSG00000161179	3	0	7	8
ENSG00000161180	0	0	0	0
ENSG00000161202	25	91	52	75
ENSG00000161203	2359	6751	4721	6358
ENSG00000161204	6	0	2	4
ENSG00000161217	66	358	307	545
ENSG00000161243	1	0	1	0

ENSG00000161249	1	0	0	0
ENSG00000161265	41	0	1	13
ENSG00000161267	1	0	0	0
ENSG00000161270	0	0	0	7
ENSG00000161277	3	0	0	0
ENSG00000161281	6	0	1	0
ENSG00000161298	3	37	44	58
ENSG00000161326	13	57	39	59
ENSG00000161328	0	0	0	0
ENSG00000161381	0	9	0	0
ENSG00000161395	1	22	1	0
ENSG00000161405	3	0	5	0
ENSG00000161509	3	0	0	0
ENSG00000161513	0	0	2	0
ENSG00000161526	42	37	54	84
ENSG00000161533	25	254	684	875
ENSG00000161542	40	216	93	126
ENSG00000161544	0	0	0	0
ENSG00000161547	186	331	138	279
ENSG00000161551	11	223	238	321
ENSG00000161558	1	0	0	3
ENSG00000161570	170313	86132	79181	70565
ENSG00000161572	0	0	0	0
ENSG00000161573	0	0	0	0
ENSG00000161574	0	0	0	0
ENSG00000161583	0	0	0	0
ENSG00000161594	0	0	0	0
ENSG00000161609	4	0	6	7
ENSG00000161610	3	0	0	0
ENSG00000161618	10	37	5	5
ENSG00000161634	0	0	0	0
ENSG00000161638	15	24	16	17
ENSG00000161640	0	0	0	0
ENSG00000161642	40	18	14	21
ENSG00000161643	1	0	0	0
ENSG00000161647	1	66	39	31
ENSG00000161649	1	4	0	0
ENSG00000161652	0	0	0	0
ENSG00000161653	0	0	0	0
ENSG00000161654	128	316	317	280
ENSG00000161664	2	22	0	0
ENSG00000161671	41	113	105	131
ENSG00000161677	0	0	0	11
ENSG00000161681	0	0	0	0
ENSG00000161682	0	0	0	0
ENSG00000161692	2	34	13	28

ENSG00000161714	5	7	3	4
ENSG00000161791	29	95	38	62
ENSG00000161798	0	0	0	0
ENSG00000161800	1	253	186	308
ENSG00000161807	0	0	0	0
ENSG00000161813	67	6884	6251	10364
ENSG00000161835	0	0	0	0
ENSG00000161847	0	0	0	0
ENSG00000161849	0	0	0	0
ENSG00000161850	0	0	0	0
ENSG00000161860	0	0	0	0
ENSG00000161888	0	10	15	29
ENSG00000161896	0	0	0	0
ENSG00000161904	3	0	41	21
ENSG00000161905	3	0	0	0
ENSG00000161911	11896	13301	14194	17182
ENSG00000161912	1	58	17	26
ENSG00000161914	0	0	1	0
ENSG00000161920	13	0	5	7
ENSG00000161921	114	149	130	241
ENSG00000161929	20	0	0	0
ENSG00000161939	1	0	0	0
ENSG00000161940	1	0	2	1
ENSG00000161944	3	0	0	0
ENSG00000161955	4	36	8	8
ENSG00000161956	0	0	3	6
ENSG00000161958	0	0	0	0
ENSG00000161960	1	0	0	2
ENSG00000161970	192	77	13	36
ENSG00000161973	0	0	0	0
ENSG00000161980	34	19	20	50
ENSG00000161981	10	0	0	3
ENSG00000161992	0	0	0	0
ENSG00000161996	0	0	0	0
ENSG00000161999	0	142	121	165
ENSG00000162004	0	0	0	0
ENSG00000162006	0	0	0	0
ENSG00000162009	0	0	0	0
ENSG00000162032	0	68	19	18
ENSG00000162039	0	0	3	2
ENSG00000162040	0	0	0	0
ENSG00000162062	0	0	0	0
ENSG00000162063	2	0	0	3
ENSG00000162065	2	8	8	11
ENSG00000162066	2	1	6	4
ENSG00000162068	0	0	0	0

ENSG00000162069	0	0	0	0
ENSG00000162073	0	0	2	16
ENSG00000162076	2	0	3	7
ENSG00000162078	11	0	4	0
ENSG00000162086	4	16	29	14
ENSG00000162104	1	0	3	1
ENSG00000162105	7	0	1	24
ENSG00000162129	2	0	4	14
ENSG00000162139	0	0	0	0
ENSG00000162144	12	58	40	49
ENSG00000162148	18	31	8	8
ENSG00000162174	27	207	33	204
ENSG00000162188	0	1	3	1
ENSG00000162191	134	137	11	39
ENSG00000162194	53	40	1	6
ENSG00000162222	28	213	92	148
ENSG00000162227	0	12	0	3
ENSG00000162231	13	13	22	24
ENSG00000162236	21	154	197	290
ENSG00000162241	38	131	28	17
ENSG00000162244	737	312	144	497
ENSG00000162267	0	9	0	0
ENSG00000162290	44	272	286	327
ENSG00000162298	0	18	9	6
ENSG00000162300	29	32	7	11
ENSG00000162302	9	0	0	0
ENSG00000162337	0	0	1	3
ENSG00000162341	0	0	0	0
ENSG00000162344	0	0	0	0
ENSG00000162365	0	0	0	0
ENSG00000162366	1230	3747	2928	1870
ENSG00000162367	1806	12433	9330	5842
ENSG00000162368	3307	29572	35801	32123
ENSG00000162373	1	0	0	0
ENSG00000162374	1	0	0	0
ENSG00000162377	1	0	0	0
ENSG00000162378	38	243	328	332
ENSG00000162383	0	0	0	0
ENSG00000162384	22	22	13	22
ENSG00000162385	46	10	23	24
ENSG00000162390	0	0	5	0
ENSG00000162391	0	0	0	0
ENSG00000162396	2	0	0	0
ENSG00000162398	1	0	0	0
ENSG00000162399	0	0	0	0
ENSG00000162402	18	671	604	712

ENSG00000162407	34	187	58	44
ENSG00000162408	5	178	85	151
ENSG00000162409	0	0	1	0
ENSG00000162413	4	0	1	3
ENSG00000162415	0	0	1	6
ENSG00000162419	35	175	71	129
ENSG00000162426	0	0	0	0
ENSG00000162430	147	184	166	332
ENSG00000162433	0	0	1	0
ENSG00000162434	1150	5729	6746	4865
ENSG00000162437	4	147	182	142
ENSG00000162438	0	0	0	0
ENSG00000162441	229	7771	8717	15943
ENSG00000162444	15	0	2	6
ENSG00000162456	0	0	0	0
ENSG00000162458	3	0	2	0
ENSG00000162460	0	0	0	0
ENSG00000162461	0	0	0	0
ENSG00000162482	0	0	0	0
ENSG00000162490	4	0	0	0
ENSG00000162493	0	0	0	0
ENSG00000162494	0	0	0	0
ENSG00000162496	927	1872	472	1183
ENSG00000162510	0	0	0	0
ENSG00000162511	546	1895	2017	1542
ENSG00000162512	0	0	0	0
ENSG00000162517	175	292	254	428
ENSG00000162520	1	148	31	12
ENSG00000162521	35	1706	1509	2519
ENSG00000162522	0	0	0	0
ENSG00000162526	0	0	1	0
ENSG00000162542	3	0	3	2
ENSG00000162543	1	0	1	0
ENSG00000162545	2	0	0	0
ENSG00000162551	0	0	2	0
ENSG00000162552	0	0	0	0
ENSG00000162571	0	0	0	0
ENSG00000162572	0	0	0	0
ENSG00000162576	0	0	0	0
ENSG00000162585	5	18	8	10
ENSG00000162591	1	0	0	2
ENSG00000162592	0	0	0	0
ENSG00000162594	0	0	0	6
ENSG00000162595	0	0	0	0
ENSG00000162598	34	59	37	252
ENSG00000162599	10	25	12	8

ENSG00000162600	3	810	804	1330
ENSG00000162601	14	508	1004	1176
ENSG00000162604	20	416	343	879
ENSG00000162607	205	3184	3244	4266
ENSG00000162613	158	573	647	517
ENSG00000162614	8946	9860	6339	11220
ENSG00000162616	6	126	335	295
ENSG00000162618	1	41	8	5
ENSG00000162620	0	0	0	3
ENSG00000162621	0	0	0	0
ENSG00000162623	29	0	15	21
ENSG00000162624	0	0	0	0
ENSG00000162627	0	0	0	0
ENSG00000162630	0	0	0	0
ENSG00000162631	0	0	0	0
ENSG00000162636	7	332	207	388
ENSG00000162639	18	80	63	81
ENSG00000162641	0	0	0	0
ENSG00000162642	71	133	180	218
ENSG00000162643	0	0	0	0
ENSG00000162645	80	0	16	8
ENSG00000162650	1	7	3	0
ENSG00000162654	79	94	210	138
ENSG00000162664	217	254	328	341
ENSG00000162669	0	449	912	537
ENSG00000162670	0	0	0	0
ENSG00000162676	0	0	0	0
ENSG00000162685	0	0	0	0
ENSG00000162687	6	707	1154	239
ENSG00000162688	12	445	473	432
ENSG00000162692	1	0	0	0
ENSG00000162694	25	65	74	84
ENSG00000162695	7	1126	951	1236
ENSG00000162699	0	0	0	0
ENSG00000162702	18	100	54	48
ENSG00000162704	6826	21738	16229	23400
ENSG00000162706	0	0	0	0
ENSG00000162711	6	0	0	6
ENSG00000162714	1	23	19	12
ENSG00000162722	11721	7218	8255	6608
ENSG00000162723	0	0	0	0
ENSG00000162727	0	0	0	0
ENSG00000162728	0	0	0	0
ENSG00000162729	0	0	0	0
ENSG00000162733	0	0	0	5
ENSG00000162734	190	316	394	562

ENSG00000162735	24	62	23	40
ENSG00000162736	12	76	72	82
ENSG00000162738	0	0	0	0
ENSG00000162739	3	0	13	12
ENSG00000162745	3	0	0	0
ENSG00000162746	1	0	0	0
ENSG00000162747	13	7	3	5
ENSG00000162753	1	3	0	5
ENSG00000162755	0	0	0	0
ENSG00000162757	0	0	0	0
ENSG00000162761	0	0	0	0
ENSG00000162763	0	0	0	0
ENSG00000162769	15	939	871	1170
ENSG00000162771	0	0	0	0
ENSG00000162772	31	60	120	111
ENSG00000162775	2	29	15	66
ENSG00000162777	99	183	118	183
ENSG00000162779	1	14	11	5
ENSG00000162782	0	0	3	0
ENSG00000162783	9	0	9	8
ENSG00000162804	117	75	44	20
ENSG00000162813	11	19	1	6
ENSG00000162814	0	0	0	3
ENSG00000162817	7	8	6	1
ENSG00000162819	823	8005	7373	5719
ENSG00000162825	1	0	0	8
ENSG00000162836	1	26	15	51
ENSG00000162840	2	0	0	0
ENSG00000162843	0	0	5	1
ENSG00000162849	3	64	71	25
ENSG00000162851	7	611	570	789
ENSG00000162852	3593	42948	37066	29451
ENSG00000162869	12	614	586	744
ENSG00000162873	2	0	1	0
ENSG00000162877	0	0	0	0
ENSG00000162878	0	0	1	0
ENSG00000162881	0	0	8	4
ENSG00000162882	2	0	0	0
ENSG00000162885	1	34	39	78
ENSG00000162888	0	0	0	0
ENSG00000162889	332	2494	1277	2446
ENSG00000162891	0	0	0	0
ENSG00000162892	5	0	0	0
ENSG00000162894	19	0	0	0
ENSG00000162896	1	0	0	0
ENSG00000162897	0	0	0	0

ENSG00000162909	1247	5664	5065	5445
ENSG00000162910	23	40	7	9
ENSG00000162913	1	0	0	0
ENSG00000162923	406	8591	9440	6902
ENSG00000162924	39	678	772	819
ENSG00000162927	2	10	31	49
ENSG00000162928	4	50	95	81
ENSG00000162929	65	3942	3948	5559
ENSG00000162931	0	0	0	0
ENSG00000162944	0	30	5	39
ENSG00000162946	3	0	16	25
ENSG00000162947	0	0	0	0
ENSG00000162949	1	0	4	2
ENSG00000162951	0	0	0	0
ENSG00000162959	0	24	27	14
ENSG00000162961	27	73	42	27
ENSG00000162971	2	133	115	106
ENSG00000162972	5	12	51	65
ENSG00000162975	0	0	0	0
ENSG00000162976	10	66	68	100
ENSG00000162980	289	1162	1366	1525
ENSG00000162981	0	0	0	0
ENSG00000162989	2	0	1	2
ENSG00000162992	0	0	0	0
ENSG00000162994	41	2981	1402	3463
ENSG00000162997	2	0	2	3
ENSG00000162998	0	0	1	1
ENSG00000162999	38	901	1400	1262
ENSG00000163001	8	16	17	67
ENSG00000163002	4	1398	886	1374
ENSG00000163006	1	797	620	1141
ENSG00000163009	0	0	5	3
ENSG00000163012	0	0	0	0
ENSG00000163013	1	0	0	0
ENSG00000163016	0	0	0	0
ENSG00000163017	0	0	1	0
ENSG00000163026	7	0	10	7
ENSG00000163029	36	707	744	909
ENSG00000163032	1	0	0	0
ENSG00000163040	0	0	0	0
ENSG00000163041	61667	53533	30778	36202
ENSG00000163046	0	0	0	8
ENSG00000163050	23	86	107	39
ENSG00000163053	0	4	0	0
ENSG00000163060	0	0	0	0
ENSG00000163064	0	0	0	0

ENSG00000163067	1	0	3	2
ENSG00000163069	0	0	1	1
ENSG00000163071	0	0	0	0
ENSG00000163072	5	0	11	6
ENSG00000163075	0	23	0	1
ENSG00000163081	0	0	0	0
ENSG00000163082	0	0	0	0
ENSG00000163083	1	0	0	0
ENSG00000163092	262	330	888	385
ENSG00000163093	0	0	0	0
ENSG00000163098	0	0	0	0
ENSG00000163104	9	412	447	451
ENSG00000163106	0	0	2	2
ENSG00000163110	347	6662	7004	6495
ENSG00000163113	12	98	130	144
ENSG00000163114	0	0	0	0
ENSG00000163116	0	82	121	142
ENSG00000163121	46	82	32	50
ENSG00000163125	23	524	1023	1264
ENSG00000163126	0	0	1	0
ENSG00000163131	200	80	29	9
ENSG00000163132	0	0	0	0
ENSG00000163138	2	69	72	111
ENSG00000163141	2	0	22	35
ENSG00000163145	0	0	0	0
ENSG00000163154	61	0	18	37
ENSG00000163155	1	0	0	9
ENSG00000163156	173	431	744	1850
ENSG00000163157	0	0	0	0
ENSG00000163159	18	119	56	146
ENSG00000163161	15	0	30	27
ENSG00000163162	21	101	85	75
ENSG00000163166	32	68	46	100
ENSG00000163170	11	14	16	10
ENSG00000163171	138	402	650	724
ENSG00000163191	416	0	5	0
ENSG00000163202	0	0	0	0
ENSG00000163206	0	0	0	0
ENSG00000163207	0	0	0	0
ENSG00000163209	0	0	0	0
ENSG00000163214	6	40	91	90
ENSG00000163216	0	0	0	0
ENSG00000163217	0	0	1	2
ENSG00000163218	0	0	0	0
ENSG00000163219	56	73	33	53
ENSG00000163220	248	630	193	385

ENSG00000163221	54	13	3	18
ENSG00000163235	0	0	0	0
ENSG00000163239	2	22	0	0
ENSG00000163249	7	3167	4291	5852
ENSG00000163251	0	0	7	0
ENSG00000163254	0	0	0	0
ENSG00000163257	30	86	84	77
ENSG00000163263	1	0	0	0
ENSG00000163273	0	0	0	0
ENSG00000163281	29	788	860	761
ENSG00000163283	0	0	0	0
ENSG00000163285	0	0	0	6
ENSG00000163286	0	0	0	0
ENSG00000163288	0	0	0	0
ENSG00000163291	4	109	113	37
ENSG00000163293	0	0	0	0
ENSG00000163295	0	0	0	0
ENSG00000163297	196	3552	3944	2665
ENSG00000163312	13	1379	1212	1616
ENSG00000163319	14	88	94	78
ENSG00000163320	1173	7099	12272	7641
ENSG00000163322	72	1807	2432	3014
ENSG00000163328	27	455	305	491
ENSG00000163331	0	0	0	0
ENSG00000163344	97	55	13	18
ENSG00000163346	7745	1098	1049	378
ENSG00000163347	0	0	0	0
ENSG00000163348	56	18	11	13
ENSG00000163349	440	3994	4926	3957
ENSG00000163352	0	0	0	0
ENSG00000163354	0	0	0	0
ENSG00000163357	0	0	0	0
ENSG00000163359	61	351	277	592
ENSG00000163362	0	0	0	0
ENSG00000163364	0	0	0	3
ENSG00000163374	89	8864	8392	19944
ENSG00000163376	1	0	25	3
ENSG00000163377	0	0	0	10
ENSG00000163378	1	301	191	267
ENSG00000163380	1	0	0	5
ENSG00000163382	16	29	0	0
ENSG00000163386	54	311	277	307
ENSG00000163389	3	11	21	12
ENSG00000163393	0	52	44	37
ENSG00000163394	0	0	0	0
ENSG00000163395	0	0	0	0

ENSG00000163399	56	395	208	386
ENSG00000163406	12	318	215	231
ENSG00000163412	59	2770	206	1241
ENSG00000163421	50	170	9	32
ENSG00000163424	0	0	0	0
ENSG00000163428	62	211	188	187
ENSG00000163430	538	7635	9875	23728
ENSG00000163431	0	0	0	0
ENSG00000163435	0	0	0	0
ENSG00000163440	0	0	0	0
ENSG00000163444	133	359	409	510
ENSG00000163449	2	0	8	10
ENSG00000163453	0	0	0	0
ENSG00000163462	0	0	0	2
ENSG00000163463	241	79	43	66
ENSG00000163464	1	20	0	16
ENSG00000163466	5582	4902	4194	4336
ENSG00000163467	0	0	0	0
ENSG00000163468	55	451	550	848
ENSG00000163472	1	5	0	1
ENSG00000163479	130	167	42	63
ENSG00000163481	3	0	1	5
ENSG00000163482	1	0	1	0
ENSG00000163485	0	0	0	0
ENSG00000163486	50	380	369	273
ENSG00000163491	0	0	4	1
ENSG00000163492	0	0	0	5
ENSG00000163497	0	0	0	0
ENSG00000163499	0	0	0	0
ENSG00000163501	0	0	0	0
ENSG00000163507	15	520	365	567
ENSG00000163508	23	0	0	0
ENSG00000163510	70	536	413	502
ENSG00000163512	6	162	288	233
ENSG00000163513	97	1320	1372	1790
ENSG00000163515	0	0	0	0
ENSG00000163516	12	6	17	14
ENSG00000163517	0	0	0	0
ENSG00000163518	0	0	0	0
ENSG00000163519	1	0	0	0
ENSG00000163520	0	0	0	0
ENSG00000163521	7	9	3	9
ENSG00000163527	30	6416	7653	8175
ENSG00000163528	4	0	4	0
ENSG00000163530	0	0	0	8
ENSG00000163531	1	0	0	0

ENSG00000163534	2	0	0	0
ENSG00000163535	13	226	154	291
ENSG00000163536	52	185	320	486
ENSG00000163539	5	4515	3140	5018
ENSG00000163541	66	6	23	53
ENSG00000163545	0	0	0	0
ENSG00000163554	3	104	18	38
ENSG00000163558	8	64	116	192
ENSG00000163563	410	227	202	90
ENSG00000163564	109	17	32	4
ENSG00000163565	179	244	239	154
ENSG00000163568	6	0	3	5
ENSG00000163576	137	15	15	0
ENSG00000163577	5	12	55	43
ENSG00000163581	0	0	0	0
ENSG00000163584	45	19	6	3
ENSG00000163586	0	0	0	0
ENSG00000163590	440	3315	4238	4572
ENSG00000163596	5	251	227	391
ENSG00000163597	12	23	43	80
ENSG00000163599	0	0	0	0
ENSG00000163600	1	0	0	0
ENSG00000163602	4035	13251	17007	9979
ENSG00000163605	120	982	1265	727
ENSG00000163606	1	5	72	14
ENSG00000163607	15	50	45	30
ENSG00000163608	14	251	134	191
ENSG00000163611	13	350	381	557
ENSG00000163612	0	0	0	0
ENSG00000163617	22	70	71	120
ENSG00000163618	0	0	0	0
ENSG00000163623	0	0	0	0
ENSG00000163624	1	7	0	0
ENSG00000163625	10	272	404	761
ENSG00000163626	9	0	4	7
ENSG00000163629	3	120	161	294
ENSG00000163630	0	0	0	0
ENSG00000163631	0	0	4	2
ENSG00000163632	0	0	0	0
ENSG00000163633	48	147	95	222
ENSG00000163634	272	1391	1477	2553
ENSG00000163635	44	269	328	422
ENSG00000163636	297	2258	2117	2180
ENSG00000163637	1	53	40	106
ENSG00000163638	0	0	0	0
ENSG00000163644	69	91	172	109

ENSG00000163645	0	49	39	46
ENSG00000163646	0	0	0	0
ENSG00000163655	115	4910	4418	5860
ENSG00000163659	13	128	284	224
ENSG00000163660	12	72	116	139
ENSG00000163661	0	0	0	0
ENSG00000163666	0	32	11	8
ENSG00000163673	0	0	0	0
ENSG00000163681	1227	10357	10888	12163
ENSG00000163682	1784	267	297	594
ENSG00000163683	29	0	10	6
ENSG00000163684	0	0	0	0
ENSG00000163686	2	0	0	0
ENSG00000163687	15	13	0	6
ENSG00000163689	2	6	18	38
ENSG00000163694	6	0	6	3
ENSG00000163697	0	0	4	1
ENSG00000163701	0	0	3	0
ENSG00000163702	0	0	2	1
ENSG00000163703	29	75	31	95
ENSG00000163704	32	11	7	3
ENSG00000163705	0	0	3	0
ENSG00000163710	0	0	0	0
ENSG00000163714	71	250	210	232
ENSG00000163719	74	121	118	108
ENSG00000163728	3	25	114	120
ENSG00000163734	161	180	148	256
ENSG00000163735	5228	20283	43121	34316
ENSG00000163736	488277	935649	779433	2004551
ENSG00000163737	368439	118229	124200	204968
ENSG00000163738	480	2716	4980	3704
ENSG00000163739	0	0	0	3
ENSG00000163743	296	1609	3767	2828
ENSG00000163746	0	0	0	0
ENSG00000163749	0	13	11	27
ENSG00000163751	1	2	2	0
ENSG00000163754	49	102	104	138
ENSG00000163755	8	190	74	104
ENSG00000163762	0	0	4	4
ENSG00000163781	12	754	728	1091
ENSG00000163785	13	621	920	1713
ENSG00000163788	87	3417	3142	3245
ENSG00000163792	0	0	0	0
ENSG00000163793	0	0	0	0
ENSG00000163794	0	0	0	0
ENSG00000163795	2	0	2	5

ENSG00000163798	30	393	415	700
ENSG00000163803	1	21	1	1
ENSG00000163806	0	0	2	0
ENSG00000163807	103	388	283	270
ENSG00000163808	11	1017	1019	1443
ENSG00000163810	0	0	0	0
ENSG00000163811	31	711	680	948
ENSG00000163812	166	508	262	456
ENSG00000163814	0	0	0	0
ENSG00000163815	0	0	0	0
ENSG00000163817	0	0	0	0
ENSG00000163818	30	116	49	58
ENSG00000163820	32	282	118	307
ENSG00000163823	4	0	4	0
ENSG00000163825	0	0	0	0
ENSG00000163827	1	0	0	0
ENSG00000163832	102	64	46	59
ENSG00000163833	11	117	25	237
ENSG00000163840	34	354	230	223
ENSG00000163848	170	2829	2535	3210
ENSG00000163864	22	532	106	338
ENSG00000163866	186	253	229	271
ENSG00000163867	29	1011	952	1064
ENSG00000163870	4	100	25	19
ENSG00000163872	30	2282	2302	3680
ENSG00000163873	0	0	0	18
ENSG00000163874	0	0	0	0
ENSG00000163875	72	365	175	262
ENSG00000163877	3	0	13	4
ENSG00000163879	0	0	0	2
ENSG00000163882	22	68	71	78
ENSG00000163884	0	0	0	0
ENSG00000163885	0	0	0	0
ENSG00000163888	0	0	0	0
ENSG00000163898	158	2695	1878	2815
ENSG00000163900	49	130	118	133
ENSG00000163902	322	1197	1220	1349
ENSG00000163904	26	2258	1105	1376
ENSG00000163909	0	0	0	1
ENSG00000163913	20	143	169	218
ENSG00000163914	0	0	0	1
ENSG00000163915	0	0	0	0
ENSG00000163918	5	135	157	299
ENSG00000163923	5	0	0	0
ENSG00000163930	81	301	181	170
ENSG00000163931	161	65	27	55

ENSG00000163932	950	1202	619	920
ENSG00000163933	6	103	157	166
ENSG00000163935	12	76	37	72
ENSG00000163938	46	177	194	265
ENSG00000163939	166	1779	1748	2487
ENSG00000163945	0	0	10	20
ENSG00000163946	41	5953	5540	8168
ENSG00000163947	195	4178	1617	4319
ENSG00000163950	380	1730	1490	1633
ENSG00000163956	8	2	9	30
ENSG00000163958	0	0	0	0
ENSG00000163959	3	0	2	0
ENSG00000163960	130	9862	18726	24511
ENSG00000163961	380	379	304	494
ENSG00000163964	50	210	88	245
ENSG00000163975	0	16	0	4
ENSG00000163982	0	0	0	0
ENSG00000163993	7	26	6	2
ENSG00000163995	0	0	0	0
ENSG00000164002	0	0	10	12
ENSG00000164007	0	0	0	0
ENSG00000164008	112	348	291	543
ENSG00000164010	33	153	265	239
ENSG00000164011	4	16	1	0
ENSG00000164022	101	200	231	414
ENSG00000164023	0	0	2	5
ENSG00000164024	33	908	589	640
ENSG00000164031	66	1029	1213	1567
ENSG00000164032	875	2382	1084	1399
ENSG00000164035	7	36	238	521
ENSG00000164037	1	722	495	432
ENSG00000164038	1	61	105	195
ENSG00000164039	3	0	0	0
ENSG00000164040	21	91	84	84
ENSG00000164045	0	0	7	10
ENSG00000164047	5	7	0	6
ENSG00000164048	3	0	5	2
ENSG00000164049	0	0	0	0
ENSG00000164050	0	0	0	0
ENSG00000164051	4	0	0	5
ENSG00000164053	0	42	173	336
ENSG00000164054	218	441	380	417
ENSG00000164056	4	42	40	42
ENSG00000164061	0	0	0	0
ENSG00000164062	23	9	12	13
ENSG00000164066	6	1093	672	1004

ENSG00000164068	8	15	16	11
ENSG00000164070	0	45	62	71
ENSG00000164073	2	530	508	633
ENSG00000164074	12	2187	2221	2695
ENSG00000164076	0	0	0	0
ENSG00000164077	4	0	0	0
ENSG00000164078	0	0	0	0
ENSG00000164080	15	407	232	444
ENSG00000164081	13	58	8	21
ENSG00000164082	0	0	1	1
ENSG00000164086	21	38	22	39
ENSG00000164087	14	0	8	19
ENSG00000164088	96	157	64	152
ENSG00000164089	0	0	0	0
ENSG00000164091	209	785	556	688
ENSG00000164093	0	0	0	0
ENSG00000164096	4003	8437	9908	12279
ENSG00000164099	0	0	0	0
ENSG00000164100	1	836	493	1053
ENSG00000164104	293	685	653	382
ENSG00000164105	14	96	19	37
ENSG00000164106	0	0	4	4
ENSG00000164107	0	0	0	0
ENSG00000164109	3	14	5	40
ENSG00000164111	688	56	75	94
ENSG00000164112	0	0	0	0
ENSG00000164113	0	0	0	0
ENSG00000164114	3	0	17	18
ENSG00000164116	1354	7545	10122	13058
ENSG00000164117	3	283	330	213
ENSG00000164118	109	2985	4229	1641
ENSG00000164120	563	2065	1514	1710
ENSG00000164122	1	0	0	0
ENSG00000164123	0	0	3	0
ENSG00000164124	0	0	0	0
ENSG00000164125	2	27	8	5
ENSG00000164128	0	0	0	4
ENSG00000164129	0	0	0	0
ENSG00000164134	24	705	932	979
ENSG00000164136	8	19	18	6
ENSG00000164142	0	0	2	0
ENSG00000164144	20	297	262	268
ENSG00000164151	14	153	208	373
ENSG00000164161	0	0	0	0
ENSG00000164162	2	29	66	147
ENSG00000164163	16	387	328	540

ENSG00000164164	20	837	1207	1686
ENSG00000164167	42	25	15	27
ENSG00000164168	10	85	110	235
ENSG00000164169	2	77	97	105
ENSG00000164171	216	3031	2111	2199
ENSG00000164172	12	16	25	21
ENSG00000164175	0	0	0	0
ENSG00000164176	0	0	1	0
ENSG00000164180	5	234	135	99
ENSG00000164181	1000	47347	36800	42365
ENSG00000164182	19	0	28	45
ENSG00000164185	0	0	0	0
ENSG00000164187	7	405	477	499
ENSG00000164188	0	0	0	0
ENSG00000164190	174	2737	2708	3271
ENSG00000164197	0	33	6	26
ENSG00000164199	0	0	4	2
ENSG00000164209	21	389	276	258
ENSG00000164211	24	242	2391	1299
ENSG00000164219	8	317	280	369
ENSG00000164220	0	0	0	0
ENSG00000164221	7	17	75	81
ENSG00000164236	3	127	143	205
ENSG00000164237	53	352	121	133
ENSG00000164241	1	0	0	0
ENSG00000164244	20	151	133	141
ENSG00000164251	0	0	7	4
ENSG00000164252	22	4374	3471	4050
ENSG00000164253	43	1598	1506	2068
ENSG00000164256	0	0	0	0
ENSG00000164258	214	1260	1133	1594
ENSG00000164265	0	0	0	0
ENSG00000164266	2	0	0	3
ENSG00000164270	2	13	3	28
ENSG00000164283	5	17	28	13
ENSG00000164284	0	155	38	64
ENSG00000164287	0	0	0	0
ENSG00000164291	37	529	269	425
ENSG00000164292	16	25211	17373	20012
ENSG00000164294	0	0	0	0
ENSG00000164296	2	0	4	3
ENSG00000164299	0	0	0	0
ENSG00000164300	1	106	94	101
ENSG00000164303	0	0	0	0
ENSG00000164304	2	0	0	7
ENSG00000164305	179	3613	3357	2287

ENSG00000164306	106	1165	991	1015
ENSG00000164307	14	435	582	631
ENSG00000164308	30	109	48	73
ENSG00000164309	1	0	3	0
ENSG00000164318	1	0	0	1
ENSG00000164323	28	209	401	535
ENSG00000164325	0	0	0	0
ENSG00000164326	0	0	0	0
ENSG00000164327	6	939	1442	2277
ENSG00000164329	26	3051	2825	3203
ENSG00000164330	3	19	15	8
ENSG00000164331	11	412	198	434
ENSG00000164332	33	27	34	53
ENSG00000164334	0	0	0	0
ENSG00000164338	3	13	17	21
ENSG00000164342	0	34	9	7
ENSG00000164344	0	0	0	0
ENSG00000164346	92	55	46	59
ENSG00000164347	3	645	533	762
ENSG00000164362	0	0	0	0
ENSG00000164363	0	0	0	0
ENSG00000164366	61	239	147	125
ENSG00000164379	0	0	0	0
ENSG00000164385	0	0	1	0
ENSG00000164393	0	0	2	4
ENSG00000164398	11	1	5	7
ENSG00000164399	0	0	0	0
ENSG00000164400	0	0	0	0
ENSG00000164402	4	0	0	3
ENSG00000164403	0	0	0	0
ENSG00000164404	0	0	0	0
ENSG00000164405	197	42	25	125
ENSG00000164406	1	0	0	0
ENSG00000164411	0	0	0	0
ENSG00000164414	0	0	2	0
ENSG00000164418	0	0	0	0
ENSG00000164430	8	349	214	550
ENSG00000164434	0	0	0	0
ENSG00000164438	0	0	0	0
ENSG00000164440	3	15	5	5
ENSG00000164442	11	8	38	47
ENSG00000164451	4	1	12	21
ENSG00000164458	0	0	0	0
ENSG00000164463	484	4056	4674	2615
ENSG00000164465	3	79	42	86
ENSG00000164466	5	222	104	88

ENSG00000164483	33	38	8	0
ENSG00000164484	0	0	2	0
ENSG00000164485	0	0	0	0
ENSG00000164488	0	0	0	0
ENSG00000164494	6	39	47	127
ENSG00000164500	1	0	0	0
ENSG00000164506	100	3173	3346	4067
ENSG00000164508	0	0	0	0
ENSG00000164509	0	0	0	0
ENSG00000164512	964	553	871	409
ENSG00000164520	0	0	0	0
ENSG00000164530	0	0	0	0
ENSG00000164532	0	0	0	0
ENSG00000164535	8	88	30	47
ENSG00000164542	0	0	5	4
ENSG00000164543	107	797	1334	908
ENSG00000164548	200	637	907	743
ENSG00000164556	0	0	0	0
ENSG00000164574	226	950	712	965
ENSG00000164576	8	63	41	28
ENSG00000164587	2625	174	62	94
ENSG00000164588	0	0	0	0
ENSG00000164591	0	0	0	0
ENSG00000164597	6	990	1056	1452
ENSG00000164600	0	0	0	0
ENSG00000164603	4	178	291	329
ENSG00000164604	0	0	0	0
ENSG00000164609	257	353	373	424
ENSG00000164610	18	38	12	15
ENSG00000164611	36	30	43	55
ENSG00000164615	163	260	309	464
ENSG00000164616	0	0	0	0
ENSG00000164619	0	0	3	0
ENSG00000164620	1	48	27	10
ENSG00000164621	30	0	11	2
ENSG00000164626	1	0	0	0
ENSG00000164627	1	0	14	4
ENSG00000164631	4	38	26	23
ENSG00000164638	0	0	0	0
ENSG00000164645	0	0	0	0
ENSG00000164647	0	0	1	0
ENSG00000164649	10	0	29	24
ENSG00000164651	0	0	0	0
ENSG00000164654	7	122	105	277
ENSG00000164659	18	742	576	474
ENSG00000164663	7	186	286	341

ENSG00000164669	0	0	0	0
ENSG00000164674	2	0	6	2
ENSG00000164675	9	267	292	409
ENSG00000164683	3	16	0	0
ENSG00000164684	2	25	16	5
ENSG00000164687	29	52	19	17
ENSG00000164690	0	0	0	2
ENSG00000164691	9	0	0	3
ENSG00000164692	0	0	1	1
ENSG00000164694	0	0	1	0
ENSG00000164695	24	46	35	37
ENSG00000164707	0	0	2	0
ENSG00000164708	0	14	0	0
ENSG00000164713	121	75	34	21
ENSG00000164715	1	37	62	71
ENSG00000164729	0	0	0	0
ENSG00000164733	37	35	136	54
ENSG00000164736	2	0	0	0
ENSG00000164740	1	0	0	1
ENSG00000164741	19	55	109	91
ENSG00000164742	0	0	0	0
ENSG00000164743	0	0	0	0
ENSG00000164744	0	0	0	0
ENSG00000164746	0	0	0	0
ENSG00000164749	0	0	0	0
ENSG00000164751	10	10	17	9
ENSG00000164754	297	2259	2426	2189
ENSG00000164756	0	0	1	0
ENSG00000164758	4	0	0	3
ENSG00000164761	1	0	1	0
ENSG00000164764	0	0	0	0
ENSG00000164776	0	4	15	27
ENSG00000164778	0	0	0	0
ENSG00000164794	0	0	0	0
ENSG00000164796	0	0	1	0
ENSG00000164808	13	3766	2469	4841
ENSG00000164815	3	1929	1527	2139
ENSG00000164816	0	0	0	0
ENSG00000164818	0	0	8	6
ENSG00000164821	7	0	0	2
ENSG00000164822	0	0	0	0
ENSG00000164823	44	442	560	435
ENSG00000164825	0	0	0	0
ENSG00000164828	4	99	13	18
ENSG00000164830	67	894	1402	1748
ENSG00000164841	0	0	0	6

ENSG00000164845	1	0	0	0
ENSG00000164849	0	0	0	8
ENSG00000164850	0	0	0	0
ENSG00000164853	0	0	0	0
ENSG00000164855	0	0	0	1
ENSG00000164867	0	0	0	0
ENSG00000164871	0	0	0	0
ENSG00000164877	2	81	78	22
ENSG00000164879	4	30	13	44
ENSG00000164880	2	16	1	9
ENSG00000164885	3	0	1	2
ENSG00000164889	2	21	15	32
ENSG00000164893	0	0	0	0
ENSG00000164896	1	0	1	5
ENSG00000164897	0	87	11	32
ENSG00000164898	2	0	0	0
ENSG00000164900	0	0	0	0
ENSG00000164902	46	37	83	65
ENSG00000164904	3	12	7	9
ENSG00000164916	26	23	55	117
ENSG00000164919	1264	808	501	1415
ENSG00000164920	0	0	0	0
ENSG00000164924	11763	195708	170031	185600
ENSG00000164929	0	0	1	4
ENSG00000164930	0	0	14	9
ENSG00000164932	1	0	0	1
ENSG00000164933	6	24	167	278
ENSG00000164934	7	442	347	498
ENSG00000164935	59	165	129	73
ENSG00000164938	36	432	546	383
ENSG00000164941	10	3266	3332	4135
ENSG00000164944	29	404	435	577
ENSG00000164946	0	6	4	2
ENSG00000164949	0	0	0	0
ENSG00000164951	15	83	113	73
ENSG00000164953	16	607	395	838
ENSG00000164961	13	172	163	214
ENSG00000164967	39	1	1	5
ENSG00000164970	3	0	0	0
ENSG00000164972	1	0	0	0
ENSG00000164975	12	215	171	255
ENSG00000164976	0	23	6	11
ENSG00000164978	56	72	13	25
ENSG00000164983	13	524	528	247
ENSG00000164985	580	1596	1383	1653
ENSG00000164989	21	338	637	635

ENSG00000165006	126	1425	994	1255
ENSG00000165023	0	0	0	0
ENSG00000165025	732	1880	1339	2398
ENSG00000165028	19	233	115	183
ENSG00000165029	0	0	1	0
ENSG00000165030	4	18	9	7
ENSG00000165046	1	40	24	57
ENSG00000165055	10	17	3	11
ENSG00000165059	0	0	0	0
ENSG00000165060	20	67	75	118
ENSG00000165061	0	0	0	0
ENSG00000165066	0	0	0	0
ENSG00000165071	91	448	260	242
ENSG00000165072	0	0	0	0
ENSG00000165076	0	0	0	0
ENSG00000165078	6	81	59	71
ENSG00000165084	0	0	0	3
ENSG00000165091	31	15	11	24
ENSG00000165092	5	33	3	9
ENSG00000165097	17	235	515	868
ENSG00000165102	18	76	105	127
ENSG00000165105	4	1	11	13
ENSG00000165113	63	575	617	1366
ENSG00000165115	6	372	285	430
ENSG00000165118	3	0	1	1
ENSG00000165119	2223	14645	17478	13683
ENSG00000165120	0	0	0	0
ENSG00000165121	35	1044	912	1156
ENSG00000165124	0	0	0	0
ENSG00000165125	0	0	0	0
ENSG00000165131	0	0	0	0
ENSG00000165138	1	0	1	1
ENSG00000165140	11	0	0	0
ENSG00000165152	0	0	2	14
ENSG00000165156	51	371	407	435
ENSG00000165164	0	0	2	0
ENSG00000165168	76	386	367	304
ENSG00000165169	256	1260	2680	5098
ENSG00000165171	0	0	0	0
ENSG00000165175	14	205	134	98
ENSG00000165178	0	0	0	0
ENSG00000165181	0	0	0	1
ENSG00000165182	0	0	0	0
ENSG00000165185	0	0	4	6
ENSG00000165186	0	0	0	2
ENSG00000165188	0	0	0	0

ENSG00000165192	0	0	0	0
ENSG00000165194	0	0	0	0
ENSG00000165195	5	33	18	26
ENSG00000165197	1	0	1	2
ENSG00000165202	0	0	0	0
ENSG00000165204	0	0	0	0
ENSG00000165209	76	337	337	367
ENSG00000165215	0	0	0	0
ENSG00000165219	127	2363	1864	2920
ENSG00000165233	1150	3130	1373	904
ENSG00000165238	0	0	0	0
ENSG00000165240	6	2298	1882	3541
ENSG00000165244	229	3748	7504	4079
ENSG00000165246	1	0	0	0
ENSG00000165259	1	0	0	6
ENSG00000165264	79	79	39	63
ENSG00000165269	0	0	0	0
ENSG00000165271	3	0	0	4
ENSG00000165272	0	0	0	0
ENSG00000165275	29	405	163	275
ENSG00000165280	228	1310	1274	1395
ENSG00000165282	3	38	6	5
ENSG00000165283	35	24	13	15
ENSG00000165288	219	6514	8302	8175
ENSG00000165300	0	0	2	2
ENSG00000165304	0	561	387	660
ENSG00000165309	244	4733	4703	4526
ENSG00000165312	3	0	32	5
ENSG00000165322	6	713	704	859
ENSG00000165323	0	0	0	4
ENSG00000165325	0	0	0	0
ENSG00000165338	1	606	411	654
ENSG00000165349	0	0	0	0
ENSG00000165355	11	81	81	167
ENSG00000165359	1	577	735	771
ENSG00000165366	2	311	465	742
ENSG00000165370	0	0	0	0
ENSG00000165376	1	0	0	1
ENSG00000165379	0	0	0	0
ENSG00000165383	0	0	2	0
ENSG00000165388	0	0	0	0
ENSG00000165389	462	1203	1633	1613
ENSG00000165390	0	0	0	0
ENSG00000165392	4	930	602	1033
ENSG00000165406	410	1338	1615	1309
ENSG00000165409	1	1	27	14

ENSG00000165410	2	67	59	231
ENSG00000165416	84	350	540	900
ENSG00000165417	89	1275	1963	1395
ENSG00000165424	14	13	13	19
ENSG00000165434	162	880	1677	558
ENSG00000165443	0	0	0	0
ENSG00000165449	0	0	0	0
ENSG00000165457	1	0	0	0
ENSG00000165458	45	312	144	178
ENSG00000165462	0	0	0	0
ENSG00000165471	0	0	0	0
ENSG00000165474	0	0	0	0
ENSG00000165475	560	1826	658	1680
ENSG00000165476	57	1848	3773	2694
ENSG00000165478	0	0	0	0
ENSG00000165480	1	6316	4341	6869
ENSG00000165487	28	1293	1265	1556
ENSG00000165490	1	33	21	44
ENSG00000165494	171	956	828	829
ENSG00000165495	0	0	0	0
ENSG00000165496	0	0	0	0
ENSG00000165501	4	452	264	369
ENSG00000165502	931	638	396	568
ENSG00000165506	1	0	24	22
ENSG00000165507	20	40	9	4
ENSG00000165509	0	0	0	0
ENSG00000165511	45	110	121	134
ENSG00000165512	127	498	528	454
ENSG00000165516	30	181	276	265
ENSG00000165521	1	56	66	45
ENSG00000165525	891	9183	8579	11540
ENSG00000165526	6	2	2	0
ENSG00000165527	51	6	57	35
ENSG00000165533	1	131	179	153
ENSG00000165548	0	0	0	0
ENSG00000165553	0	0	0	0
ENSG00000165555	0	0	4	4
ENSG00000165556	0	0	0	0
ENSG00000165566	1	0	0	0
ENSG00000165568	9	561	203	203
ENSG00000165572	2	0	6	7
ENSG00000165583	0	0	0	0
ENSG00000165584	0	0	0	0
ENSG00000165588	0	0	0	0
ENSG00000165591	3	14	42	49
ENSG00000165606	0	0	0	0

ENSG00000165609	15	41	28	40
ENSG00000165617	1	0	8	8
ENSG00000165621	0	0	0	0
ENSG00000165623	0	0	0	0
ENSG00000165626	150	235	296	83
ENSG00000165629	155	1692	1802	2358
ENSG00000165630	7	33	38	29
ENSG00000165632	91	45	42	62
ENSG00000165633	0	0	1	0
ENSG00000165637	123	985	805	955
ENSG00000165643	0	0	0	0
ENSG00000165644	4	14	1	10
ENSG00000165646	199	2485	2580	2525
ENSG00000165650	32	366	633	1084
ENSG00000165655	1	0	0	0
ENSG00000165659	115	106	96	150
ENSG00000165660	14	189	265	404
ENSG00000165661	5	0	27	44
ENSG00000165669	633	2486	1393	1250
ENSG00000165671	142	2644	2738	3045
ENSG00000165672	22	74	87	169
ENSG00000165675	22	54	61	114
ENSG00000165678	170	914	705	1014
ENSG00000165682	3957	13925	8821	13728
ENSG00000165684	0	0	0	0
ENSG00000165685	0	0	0	0
ENSG00000165688	6	1	3	1
ENSG00000165689	5	0	1	8
ENSG00000165694	0	0	0	0
ENSG00000165695	2	0	0	0
ENSG00000165698	43	75	98	42
ENSG00000165699	7	118	42	75
ENSG00000165702	5013	11935	12073	7881
ENSG00000165704	16	865	1120	1357
ENSG00000165714	0	71	0	0
ENSG00000165716	28	686	357	265
ENSG00000165724	0	0	1	0
ENSG00000165730	0	8	5	26
ENSG00000165731	1	96	62	82
ENSG00000165732	541	3595	2622	3570
ENSG00000165733	37	293	355	468
ENSG00000165752	4	0	0	0
ENSG00000165757	5	126	48	82
ENSG00000165762	0	0	0	0
ENSG00000165775	457	632	610	828
ENSG00000165782	43	43	59	30

ENSG00000165792	1	0	2	10
ENSG00000165794	0	0	0	0
ENSG00000165795	16	3	7	0
ENSG00000165799	0	0	0	0
ENSG00000165801	0	0	1	0
ENSG00000165802	0	0	0	0
ENSG00000165804	1	16	8	21
ENSG00000165805	25	149	158	153
ENSG00000165806	6	60	79	35
ENSG00000165807	2	19	33	53
ENSG00000165810	2	0	1	0
ENSG00000165813	58	198	263	178
ENSG00000165816	0	0	0	0
ENSG00000165819	8	379	212	382
ENSG00000165821	3	74	27	57
ENSG00000165828	0	0	0	0
ENSG00000165832	1	353	322	482
ENSG00000165837	0	0	0	0
ENSG00000165841	0	0	4	3
ENSG00000165861	7	26	46	50
ENSG00000165862	0	0	0	0
ENSG00000165863	0	0	0	0
ENSG00000165868	0	0	0	0
ENSG00000165874	0	0	0	0
ENSG00000165879	8	57	13	6
ENSG00000165886	0	20	0	1
ENSG00000165887	0	0	0	0
ENSG00000165891	0	0	10	3
ENSG00000165895	0	39	38	30
ENSG00000165898	24	62	42	91
ENSG00000165899	1	59	167	151
ENSG00000165905	0	0	0	0
ENSG00000165912	0	0	0	0
ENSG00000165914	516	6274	4165	6523
ENSG00000165915	1	9	3	7
ENSG00000165916	60	50	16	54
ENSG00000165917	0	0	0	0
ENSG00000165923	0	11	29	47
ENSG00000165929	992	11865	15968	16303
ENSG00000165934	97	1619	2349	2120
ENSG00000165935	0	0	7	8
ENSG00000165943	10	37	15	19
ENSG00000165948	130	134	55	59
ENSG00000165949	86	82	44	27
ENSG00000165953	0	0	0	0
ENSG00000165959	2953	303	358	122

ENSG00000165966	0	7	6	18
ENSG00000165970	0	0	0	0
ENSG00000165972	0	0	0	0
ENSG00000165973	0	0	0	0
ENSG00000165983	20	143	126	177
ENSG00000165985	0	0	1	0
ENSG00000165995	1	0	0	0
ENSG00000165996	3	775	560	1317
ENSG00000165997	25	345	624	259
ENSG00000166002	10	0	0	1
ENSG00000166004	82	1292	1433	1750
ENSG00000166006	0	0	4	0
ENSG00000166007	0	0	0	0
ENSG00000166008	0	0	0	0
ENSG00000166012	70	290	460	566
ENSG00000166013	0	0	0	0
ENSG00000166016	0	0	1	3
ENSG00000166024	15	126	114	195
ENSG00000166025	25	212	373	384
ENSG00000166033	0	0	0	0
ENSG00000166035	472	769	853	1666
ENSG00000166037	82	1137	1555	2250
ENSG00000166046	8	20	25	21
ENSG00000166049	0	0	0	0
ENSG00000166068	3	14	11	10
ENSG00000166069	0	29	1	0
ENSG00000166073	0	0	1	0
ENSG00000166086	1062	30237	14672	41364
ENSG00000166090	7	0	0	0
ENSG00000166091	2648	2536	1845	2800
ENSG00000166104	0	0	0	0
ENSG00000166105	0	0	0	0
ENSG00000166106	0	0	0	0
ENSG00000166111	0	0	0	0
ENSG00000166118	0	0	0	0
ENSG00000166123	0	18	0	0
ENSG00000166126	0	0	1	0
ENSG00000166128	213	4545	6714	3525
ENSG00000166130	18	94	124	173
ENSG00000166133	0	11	6	7
ENSG00000166135	25	84	91	208
ENSG00000166136	38	0	0	0
ENSG00000166140	9	52	18	24
ENSG00000166143	0	0	0	0
ENSG00000166145	1	0	1	0
ENSG00000166147	0	0	18	29

ENSG00000166148	50	1221	1235	607
ENSG00000166152	0	0	0	0
ENSG00000166153	11	116	268	320
ENSG00000166157	0	0	1	2
ENSG00000166159	0	0	0	0
ENSG00000166160	0	0	0	0
ENSG00000166164	82	240	176	341
ENSG00000166165	37	56	11	18
ENSG00000166166	0	0	0	0
ENSG00000166167	67	412	301	404
ENSG00000166169	26	74	41	31
ENSG00000166170	69	196	336	232
ENSG00000166171	265	325	238	269
ENSG00000166173	0	0	0	0
ENSG00000166181	57	645	506	828
ENSG00000166183	0	0	0	0
ENSG00000166188	2	0	1	3
ENSG00000166189	3	0	1	0
ENSG00000166192	0	0	3	2
ENSG00000166197	45	65	79	134
ENSG00000166199	13	51	118	58
ENSG00000166200	75	2272	2282	3454
ENSG00000166206	0	0	0	0
ENSG00000166211	1	0	0	0
ENSG00000166220	0	0	0	0
ENSG00000166224	6	0	3	1
ENSG00000166225	3	154	115	142
ENSG00000166226	86	3425	4708	4960
ENSG00000166228	3	0	0	1
ENSG00000166233	46	2307	1545	1990
ENSG00000166246	0	0	0	0
ENSG00000166250	11	0	2	0
ENSG00000166257	0	0	0	0
ENSG00000166260	25	166	242	218
ENSG00000166261	0	13	1	0
ENSG00000166262	46	498	382	888
ENSG00000166263	2	1548	823	1169
ENSG00000166265	10	97	76	22
ENSG00000166266	48	4922	4347	5520
ENSG00000166268	0	0	3	9
ENSG00000166272	450	1090	561	846
ENSG00000166275	123	135	92	82
ENSG00000166278	0	0	0	0
ENSG00000166289	2	0	0	0
ENSG00000166292	0	0	0	0
ENSG00000166295	141	203	178	259

ENSG00000166311	626	241	55	97
ENSG00000166313	8	0	0	0
ENSG00000166317	0	0	0	0
ENSG00000166321	1	30	3	0
ENSG00000166323	0	27	45	55
ENSG00000166326	28	0	32	32
ENSG00000166329	0	0	0	0
ENSG00000166333	5	9142	4398	10898
ENSG00000166337	951	2080	1065	2157
ENSG00000166340	2663	3754	3185	4083
ENSG00000166341	1	9	1	4
ENSG00000166342	0	0	0	0
ENSG00000166343	0	0	10	6
ENSG00000166347	5	0	6	3
ENSG00000166348	2	22	36	51
ENSG00000166349	2	29	22	31
ENSG00000166351	0	0	0	0
ENSG00000166352	2	69	33	59
ENSG00000166359	0	0	0	0
ENSG00000166363	0	0	0	0
ENSG00000166368	0	0	0	0
ENSG00000166377	20	4189	4913	4402
ENSG00000166387	4	0	0	0
ENSG00000166391	0	0	0	0
ENSG00000166394	1	0	0	1
ENSG00000166396	0	0	0	0
ENSG00000166398	11	76	35	37
ENSG00000166401	8	0	0	0
ENSG00000166402	0	0	0	1
ENSG00000166405	2	67	45	50
ENSG00000166407	0	0	0	0
ENSG00000166408	0	0	0	0
ENSG00000166411	5	95	91	70
ENSG00000166415	0	0	10	5
ENSG00000166426	0	0	0	0
ENSG00000166428	2	0	2	0
ENSG00000166432	4	0	18	29
ENSG00000166435	4	14	47	71
ENSG00000166436	5	0	21	35
ENSG00000166439	11	910	621	1153
ENSG00000166441	2585	378	211	872
ENSG00000166444	3	0	0	2
ENSG00000166446	7	406	387	205
ENSG00000166448	0	0	0	0
ENSG00000166450	0	0	0	1
ENSG00000166451	6	50	230	382

ENSG00000166452	78	284	148	247
ENSG00000166454	30	113	151	173
ENSG00000166455	0	1	1	2
ENSG00000166471	23	591	855	1063
ENSG00000166473	0	0	0	0
ENSG00000166477	16	116	119	233
ENSG00000166478	32	139	177	206
ENSG00000166479	3	172	321	503
ENSG00000166482	0	0	0	0
ENSG00000166483	62	435	586	848
ENSG00000166484	6	0	0	9
ENSG00000166492	0	0	0	0
ENSG00000166501	3610	11823	10595	13401
ENSG00000166503	38	897	599	787
ENSG00000166507	0	0	5	7
ENSG00000166508	83	72	115	83
ENSG00000166509	0	0	0	0
ENSG00000166510	16	499	363	569
ENSG00000166523	1	0	1	0
ENSG00000166526	21	66	46	108
ENSG00000166527	4	0	0	0
ENSG00000166529	0	0	0	7
ENSG00000166530	0	0	0	0
ENSG00000166532	21	402	654	296
ENSG00000166535	4	0	3	4
ENSG00000166546	0	0	0	0
ENSG00000166548	7	34	7	24
ENSG00000166557	759	931	520	712
ENSG00000166558	0	0	0	0
ENSG00000166562	10	40	30	44
ENSG00000166569	0	0	0	5
ENSG00000166573	4	0	2	13
ENSG00000166575	4	1130	1086	2332
ENSG00000166578	5	0	2	0
ENSG00000166579	14	165	159	136
ENSG00000166582	12	74	16	75
ENSG00000166589	1	0	0	0
ENSG00000166592	0	0	0	0
ENSG00000166595	121	55	29	63
ENSG00000166596	0	0	1	0
ENSG00000166598	83	412	358	545
ENSG00000166603	0	0	0	0
ENSG00000166619	49	19	59	31
ENSG00000166634	0	0	2	17
ENSG00000166664	0	0	0	0
ENSG00000166667	0	0	0	0

ENSG00000166669	50	1983	1429	1613
ENSG00000166670	0	0	0	0
ENSG00000166676	1	0	0	0
ENSG00000166681	11403	13405	6386	13097
ENSG00000166682	0	0	0	0
ENSG00000166685	5	89	54	39
ENSG00000166689	0	0	1	3
ENSG00000166693	0	0	0	0
ENSG00000166704	1	40	19	46
ENSG00000166707	0	0	0	0
ENSG00000166710	761109	1791246	2132689	1593066
ENSG00000166716	24	153	131	206
ENSG00000166734	108	6927	6495	8673
ENSG00000166736	0	0	0	0
ENSG00000166741	1	0	2	1
ENSG00000166743	0	0	0	0
ENSG00000166747	106	3971	1553	4141
ENSG00000166748	0	0	0	0
ENSG00000166750	154	3	13	10
ENSG00000166762	1	0	4	6
ENSG00000166763	0	0	1	0
ENSG00000166770	82	419	186	1223
ENSG00000166780	3	0	17	34
ENSG00000166783	192	988	1259	1731
ENSG00000166787	0	0	0	0
ENSG00000166788	7	0	89	103
ENSG00000166793	0	0	0	0
ENSG00000166794	43	130	83	203
ENSG00000166796	35	4	39	20
ENSG00000166797	42	23	39	88
ENSG00000166800	1	0	0	0
ENSG00000166801	3	0	27	32
ENSG00000166803	3	39	100	91
ENSG00000166813	0	0	0	2
ENSG00000166816	0	0	1	0
ENSG00000166819	0	0	0	0
ENSG00000166821	0	0	1	0
ENSG00000166822	23	48	82	47
ENSG00000166823	0	0	0	0
ENSG00000166825	1	0	0	6
ENSG00000166828	0	0	0	0
ENSG00000166831	303	730	611	1201
ENSG00000166833	4	39	32	55
ENSG00000166839	3	0	3	11
ENSG00000166840	20	5	27	21
ENSG00000166845	17	261	613	270

ENSG00000166847	77	90	32	86
ENSG00000166848	858	2077	1961	2139
ENSG00000166851	1	0	2	0
ENSG00000166855	8	1115	1256	1839
ENSG00000166856	0	0	0	0
ENSG00000166860	4	0	0	7
ENSG00000166862	0	0	0	0
ENSG00000166863	0	0	0	0
ENSG00000166866	1	0	0	0
ENSG00000166869	0	0	0	0
ENSG00000166881	0	134	73	113
ENSG00000166884	0	0	0	0
ENSG00000166886	0	0	1	1
ENSG00000166887	84	521	452	482
ENSG00000166888	80	217	114	146
ENSG00000166889	23	423	775	1105
ENSG00000166896	18	753	312	615
ENSG00000166897	0	0	0	0
ENSG00000166900	3	0	8	8
ENSG00000166902	9	0	2	0
ENSG00000166908	4	31	30	3
ENSG00000166912	140	3294	2950	6238
ENSG00000166913	1525	1749	1485	1852
ENSG00000166917	0	0	0	0
ENSG00000166920	0	0	0	0
ENSG00000166922	1	15	4	18
ENSG00000166923	0	0	0	0
ENSG00000166924	0	0	0	0
ENSG00000166925	26	347	147	120
ENSG00000166926	0	0	0	0
ENSG00000166927	228	0	2	0
ENSG00000166928	3	0	1	0
ENSG00000166930	0	0	0	0
ENSG00000166938	10	141	67	132
ENSG00000166946	227	769	852	735
ENSG00000166947	21	1	18	18
ENSG00000166948	0	0	0	0
ENSG00000166949	18	113	75	71
ENSG00000166959	0	0	0	0
ENSG00000166960	0	0	0	0
ENSG00000166961	0	0	0	0
ENSG00000166963	870	2125	2110	1588
ENSG00000166965	0	19	4	1
ENSG00000166971	0	1	10	32
ENSG00000166974	1055	18326	18584	21529
ENSG00000166979	89	461	196	367

ENSG00000166984	0	0	0	0
ENSG00000166986	16	58	40	46
ENSG00000166987	77	225	62	89
ENSG00000166997	1	0	6	16
ENSG00000167004	309	4471	4353	6225
ENSG00000167005	227	3232	3404	2969
ENSG00000167011	0	0	0	0
ENSG00000167014	0	0	0	0
ENSG00000167034	0	0	0	0
ENSG00000167037	3	0	0	0
ENSG00000167046	0	0	0	0
ENSG00000167065	3	0	7	2
ENSG00000167074	0	0	2	2
ENSG00000167077	13	34	7	9
ENSG00000167080	0	0	0	0
ENSG00000167081	222	1147	1069	1680
ENSG00000167083	11	0	0	0
ENSG00000167085	20	16	5	1
ENSG00000167088	50	82	52	93
ENSG00000167094	0	0	0	0
ENSG00000167098	0	0	0	0
ENSG00000167100	14	456	307	875
ENSG00000167103	0	0	0	4
ENSG00000167104	0	0	0	0
ENSG00000167105	0	0	0	0
ENSG00000167106	2	0	0	0
ENSG00000167107	5	0	0	5
ENSG00000167110	384	625	321	585
ENSG00000167112	115	625	199	247
ENSG00000167113	45	40	36	101
ENSG00000167114	41	159	41	59
ENSG00000167117	2	0	0	0
ENSG00000167118	11	2	2	3
ENSG00000167123	3	11	4	2
ENSG00000167130	9	71	54	45
ENSG00000167131	0	4	10	4
ENSG00000167136	1	0	0	0
ENSG00000167139	0	0	0	0
ENSG00000167157	0	0	0	0
ENSG00000167165	0	0	0	0
ENSG00000167173	38	26	9	16
ENSG00000167178	0	0	0	0
ENSG00000167182	87	156	81	100
ENSG00000167183	0	0	0	0
ENSG00000167186	20	30	36	45
ENSG00000167191	0	0	0	0

ENSG00000167193	96	192	180	161
ENSG00000167194	0	0	0	0
ENSG00000167195	0	0	0	0
ENSG00000167196	2	136	258	585
ENSG00000167202	3	22	24	45
ENSG00000167207	1	0	1	0
ENSG00000167208	35	24	19	49
ENSG00000167210	78	584	507	450
ENSG00000167216	0	0	13	8
ENSG00000167220	28	494	360	651
ENSG00000167230	0	0	0	1
ENSG00000167232	29	448	226	318
ENSG00000167236	0	0	0	0
ENSG00000167244	6	105	15	29
ENSG00000167257	57	398	619	787
ENSG00000167258	192	1185	1334	1472
ENSG00000167261	12	0	3	1
ENSG00000167264	6	121	92	125
ENSG00000167272	21	0	1	9
ENSG00000167280	1	0	10	5
ENSG00000167281	1	0	0	0
ENSG00000167283	4966	2980	1990	2423
ENSG00000167286	37	0	0	0
ENSG00000167291	1	0	1	0
ENSG00000167302	1	0	0	1
ENSG00000167306	0	0	0	6
ENSG00000167311	0	0	0	0
ENSG00000167315	12	0	3	2
ENSG00000167323	634	2019	1124	1372
ENSG00000167325	27	462	304	391
ENSG00000167332	0	0	0	0
ENSG00000167333	2	10	1	2
ENSG00000167346	0	0	0	0
ENSG00000167355	0	0	0	2
ENSG00000167359	0	0	0	0
ENSG00000167360	0	0	0	0
ENSG00000167363	3	485	72	71
ENSG00000167371	13	33	31	8
ENSG00000167377	0	0	0	0
ENSG00000167378	8	0	1	9
ENSG00000167380	31	127	68	101
ENSG00000167383	2	0	10	12
ENSG00000167384	2	13	6	5
ENSG00000167390	0	0	0	0
ENSG00000167393	3	165	77	246
ENSG00000167394	2	0	4	8

ENSG00000167395	6	30	6	3
ENSG00000167397	267	45	48	53
ENSG00000167414	1620	371	877	519
ENSG00000167419	0	0	0	0
ENSG00000167434	0	0	0	0
ENSG00000167447	2	25	3	0
ENSG00000167459	0	0	0	0
ENSG00000167460	12527	53727	48687	66495
ENSG00000167461	308	11388	12462	15844
ENSG00000167468	4580	8001	4028	6404
ENSG00000167470	31	41	15	5
ENSG00000167476	6	526	503	269
ENSG00000167483	12	0	0	0
ENSG00000167487	1	0	0	0
ENSG00000167491	402	1312	911	1898
ENSG00000167494	0	0	0	0
ENSG00000167508	2	0	1	3
ENSG00000167513	1	0	0	2
ENSG00000167515	33	0	2	1
ENSG00000167522	312	1162	745	1203
ENSG00000167523	18	0	8	14
ENSG00000167524	0	0	0	8
ENSG00000167525	9	0	4	2
ENSG00000167526	2151	152	62	140
ENSG00000167528	6	14	11	4
ENSG00000167531	0	0	0	0
ENSG00000167535	2	10	8	4
ENSG00000167536	1	10	4	0
ENSG00000167543	0	0	4	0
ENSG00000167548	5	36	19	53
ENSG00000167549	0	0	0	0
ENSG00000167550	1	0	0	0
ENSG00000167552	125	36	53	38
ENSG00000167553	359	3514	1972	3361
ENSG00000167554	5	18	9	12
ENSG00000167555	24	77	104	217
ENSG00000167562	3	37	85	61
ENSG00000167565	9	0	0	11
ENSG00000167566	2	24	0	2
ENSG00000167578	21	0	6	23
ENSG00000167580	0	0	0	0
ENSG00000167588	0	0	2	2
ENSG00000167595	12	0	10	20
ENSG00000167600	0	0	1	5
ENSG00000167601	0	0	0	0
ENSG00000167604	1	0	0	0

ENSG00000167608	22	0	3	11
ENSG00000167612	0	0	0	0
ENSG00000167613	17	30	1	1
ENSG00000167614	0	0	0	0
ENSG00000167615	6	8	41	38
ENSG00000167617	0	0	0	0
ENSG00000167618	1	0	0	0
ENSG00000167619	0	0	0	0
ENSG00000167625	7	0	1	1
ENSG00000167632	443	1652	1429	2606
ENSG00000167633	0	0	0	0
ENSG00000167634	0	0	0	0
ENSG00000167635	40	430	396	242
ENSG00000167637	1	13	14	37
ENSG00000167641	97	503	64	195
ENSG00000167642	930	3149	1499	2252
ENSG00000167644	1	43	4	32
ENSG00000167645	310	715	466	485
ENSG00000167646	8	12	3	5
ENSG00000167653	0	0	0	0
ENSG00000167654	0	0	0	1
ENSG00000167656	0	0	0	0
ENSG00000167657	27	212	67	75
ENSG00000167658	237	592	296	734
ENSG00000167664	4	0	0	0
ENSG00000167670	51	99	21	75
ENSG00000167671	139	686	596	783
ENSG00000167674	7	118	38	86
ENSG00000167676	0	19	0	4
ENSG00000167680	1	0	0	0
ENSG00000167685	5	103	20	14
ENSG00000167693	24	22	36	21
ENSG00000167695	1	0	1	0
ENSG00000167699	27	82	45	5
ENSG00000167700	0	0	0	0
ENSG00000167701	0	0	0	0
ENSG00000167702	0	0	0	4
ENSG00000167703	0	0	2	3
ENSG00000167705	102	105	81	89
ENSG00000167711	0	0	0	0
ENSG00000167716	2	0	5	10
ENSG00000167720	0	17	14	28
ENSG00000167721	9	18	9	16
ENSG00000167723	1	19	42	16
ENSG00000167733	1	0	3	1
ENSG00000167740	519	630	614	615

ENSG00000167741	1	0	0	0
ENSG00000167747	5	0	0	0
ENSG00000167748	1	0	0	0
ENSG00000167749	0	0	0	0
ENSG00000167751	0	0	0	0
ENSG00000167754	0	0	0	0
ENSG00000167755	0	0	0	0
ENSG00000167757	0	0	0	0
ENSG00000167759	0	0	0	0
ENSG00000167765	0	0	1	0
ENSG00000167766	17	181	66	185
ENSG00000167767	0	0	0	0
ENSG00000167768	2	0	0	0
ENSG00000167769	0	0	0	0
ENSG00000167770	78	25	2	15
ENSG00000167771	0	0	0	0
ENSG00000167772	0	0	0	0
ENSG00000167774	0	0	0	0
ENSG00000167775	2	0	0	0
ENSG00000167778	22	10	0	2
ENSG00000167779	0	0	0	0
ENSG00000167780	0	0	0	0
ENSG00000167785	0	0	3	0
ENSG00000167791	0	0	0	0
ENSG00000167792	24	6	6	13
ENSG00000167797	97	46	20	33
ENSG00000167798	0	0	0	0
ENSG00000167799	0	0	0	0
ENSG00000167800	0	0	0	0
ENSG00000167807	0	0	0	0
ENSG00000167815	29	0	5	23
ENSG00000167822	0	0	0	0
ENSG00000167825	0	0	0	0
ENSG00000167840	0	0	0	0
ENSG00000167842	19	31	65	75
ENSG00000167850	1	0	0	0
ENSG00000167851	18	0	1	0
ENSG00000167858	0	0	0	0
ENSG00000167861	2	0	0	0
ENSG00000167862	11	24	2	2
ENSG00000167863	358	393	486	783
ENSG00000167874	8	16	1	1
ENSG00000167880	0	0	0	0
ENSG00000167881	21	43	104	134
ENSG00000167889	0	0	0	9
ENSG00000167895	8	10	2	4

ENSG00000167900	2	0	3	0
ENSG00000167904	8	290	427	398
ENSG00000167910	0	0	0	0
ENSG00000167912	53	26	34	26
ENSG00000167914	2	38	24	23
ENSG00000167916	0	0	0	0
ENSG00000167920	5	0	0	0
ENSG00000167925	7	0	8	3
ENSG00000167930	35	23	6	23
ENSG00000167941	0	0	0	0
ENSG00000167945	0	0	0	0
ENSG00000167962	0	9	5	17
ENSG00000167964	0	0	0	0
ENSG00000167965	1	19	5	1
ENSG00000167967	0	0	2	0
ENSG00000167968	0	0	0	0
ENSG00000167969	7	0	0	0
ENSG00000167970	0	0	0	0
ENSG00000167971	0	0	1	0
ENSG00000167972	94	171	70	186
ENSG00000167977	4	20	31	49
ENSG00000167978	47	248	94	207
ENSG00000167981	0	0	5	0
ENSG00000167984	7	37	28	25
ENSG00000167985	516	131	107	142
ENSG00000167986	56	159	136	227
ENSG00000167987	27	48	34	57
ENSG00000167992	1	0	0	1
ENSG00000167994	0	0	0	0
ENSG00000167995	0	7782	5157	2494
ENSG00000167996	3468	304661	204760	254948
ENSG00000168000	20	47	69	70
ENSG00000168002	1004	898	406	912
ENSG00000168003	56	125	181	64
ENSG00000168004	0	10	1	0
ENSG00000168005	0	0	1	10
ENSG00000168010	18	26	0	5
ENSG00000168014	136	468	283	607
ENSG00000168016	10	13	18	39
ENSG00000168026	0	0	3	4
ENSG00000168028	1400	602	363	1617
ENSG00000168032	0	0	1	8
ENSG00000168036	54	878	800	838
ENSG00000168038	23	187	376	532
ENSG00000168040	0	176	82	66
ENSG00000168056	0	0	1	7

ENSG00000168060	8	0	0	0
ENSG00000168061	0	0	0	0
ENSG00000168062	0	0	0	0
ENSG00000168065	0	3	3	0
ENSG00000168066	176	294	164	321
ENSG00000168067	190	1772	1459	1649
ENSG00000168070	0	0	0	0
ENSG00000168071	2	0	1	0
ENSG00000168077	0	0	0	0
ENSG00000168078	1	36	21	33
ENSG00000168079	0	0	0	0
ENSG00000168081	11	0	0	0
ENSG00000168090	108	96	40	90
ENSG00000168092	74	1040	1384	1525
ENSG00000168096	1	19	4	0
ENSG00000168101	21	5	1	12
ENSG00000168116	251	3102	3202	3107
ENSG00000168118	1843	7900	7528	7034
ENSG00000168122	0	0	0	0
ENSG00000168124	0	0	0	0
ENSG00000168126	0	1	12	1
ENSG00000168129	0	0	0	0
ENSG00000168131	0	0	0	0
ENSG00000168135	0	0	3	17
ENSG00000168137	78	1153	854	1529
ENSG00000168140	0	0	0	0
ENSG00000168143	0	0	0	0
ENSG00000168148	0	0	0	0
ENSG00000168152	0	31	24	57
ENSG00000168158	0	0	0	0
ENSG00000168159	55	35	14	62
ENSG00000168172	360	9813	16824	20111
ENSG00000168175	367	1303	1641	1102
ENSG00000168209	0	0	0	0
ENSG00000168214	85	361	437	502
ENSG00000168216	79	3369	2959	4875
ENSG00000168228	0	235	236	231
ENSG00000168229	21	139	67	165
ENSG00000168234	11	350	598	398
ENSG00000168237	12	24	4	10
ENSG00000168242	0	29	0	3
ENSG00000168243	8	0	0	0
ENSG00000168246	6	26	14	9
ENSG00000168255	21	4	43	47
ENSG00000168256	353	376	178	555
ENSG00000168259	90	653	535	822

ENSG00000168260	0	0	0	0
ENSG00000168263	0	0	0	0
ENSG00000168264	24	104	129	79
ENSG00000168267	0	0	0	0
ENSG00000168268	3	0	5	5
ENSG00000168269	0	0	0	0
ENSG00000168273	13	0	4	3
ENSG00000168274	2841	8503	3932	4292
ENSG00000168275	16	18	3	12
ENSG00000168280	1	0	0	4
ENSG00000168282	0	71	81	66
ENSG00000168283	85	611	697	565
ENSG00000168286	321	634	388	237
ENSG00000168288	30	218	173	143
ENSG00000168291	71	185	209	347
ENSG00000168297	239	234	723	464
ENSG00000168298	1	303	112	109
ENSG00000168300	1424	54420	52327	56557
ENSG00000168301	4	0	8	9
ENSG00000168303	15	29	3	2
ENSG00000168306	0	0	0	0
ENSG00000168309	9	15	7	4
ENSG00000168310	436	1318	2263	1580
ENSG00000168314	2	0	0	14
ENSG00000168329	49	0	0	0
ENSG00000168333	0	0	0	0
ENSG00000168334	0	0	0	0
ENSG00000168348	0	0	0	0
ENSG00000168350	4	0	0	0
ENSG00000168356	0	136	88	88
ENSG00000168367	0	0	0	0
ENSG00000168374	426	3743	3193	3454
ENSG00000168379	0	0	0	0
ENSG00000168384	0	0	0	0
ENSG00000168385	2045	11710	14779	18580
ENSG00000168386	4	14	22	17
ENSG00000168389	0	7	18	59
ENSG00000168393	2	0	0	0
ENSG00000168394	15	147	111	43
ENSG00000168395	117	292	401	600
ENSG00000168397	19	143	31	61
ENSG00000168398	0	0	0	0
ENSG00000168404	6	101	31	35
ENSG00000168405	905	1975	2421	2974
ENSG00000168411	7	103	59	95
ENSG00000168412	0	0	0	0

ENSG00000168418	0	0	0	0
ENSG00000168421	3	0	0	0
ENSG00000168427	0	0	0	0
ENSG00000168434	10	56	30	81
ENSG00000168438	62	171	330	590
ENSG00000168439	58	174	260	339
ENSG00000168447	0	0	0	0
ENSG00000168452	0	0	0	0
ENSG00000168453	0	0	0	0
ENSG00000168454	0	0	5	1
ENSG00000168461	10399	33828	47057	34515
ENSG00000168468	0	0	0	0
ENSG00000168476	1	0	0	0
ENSG00000168477	11	122	82	127
ENSG00000168481	0	0	0	0
ENSG00000168484	1	0	0	0
ENSG00000168487	0	0	0	1
ENSG00000168488	3	105	39	93
ENSG00000168490	0	0	0	0
ENSG00000168491	0	0	0	0
ENSG00000168495	2	0	9	2
ENSG00000168496	8	19	6	5
ENSG00000168497	71854	267531	349797	417617
ENSG00000168502	0	92	66	114
ENSG00000168505	0	0	0	0
ENSG00000168509	0	0	0	0
ENSG00000168515	0	0	0	0
ENSG00000168517	1	420	171	185
ENSG00000168522	25	55	102	117
ENSG00000168528	0	0	0	0
ENSG00000168530	0	0	0	0
ENSG00000168538	10	626	678	1024
ENSG00000168539	0	0	0	0
ENSG00000168542	0	0	0	0
ENSG00000168546	1	0	1	0
ENSG00000168556	67	32	100	49
ENSG00000168564	326	606	800	321
ENSG00000168566	125	850	704	1035
ENSG00000168569	6	0	0	2
ENSG00000168575	14	833	497	809
ENSG00000168582	0	0	0	0
ENSG00000168589	1	0	0	0
ENSG00000168591	31	54	22	25
ENSG00000168594	0	0	0	0
ENSG00000168610	2208	3971	4491	5960
ENSG00000168612	6	0	19	28

ENSG00000168614	3	319	334	678
ENSG00000168615	187	5219	7535	9224
ENSG00000168619	0	0	0	9
ENSG00000168621	0	0	0	0
ENSG00000168630	0	0	0	0
ENSG00000168631	0	0	0	0
ENSG00000168634	2	0	0	0
ENSG00000168646	0	0	0	0
ENSG00000168653	3226	2588	1614	2684
ENSG00000168658	0	0	2	0
ENSG00000168661	3	0	5	17
ENSG00000168671	0	0	0	0
ENSG00000168672	0	0	5	0
ENSG00000168675	32	312	347	379
ENSG00000168676	0	0	0	2
ENSG00000168679	0	0	4	0
ENSG00000168685	67	860	1247	1005
ENSG00000168701	6	14	2	6
ENSG00000168702	0	0	0	5
ENSG00000168703	0	0	0	0
ENSG00000168710	125	269	181	234
ENSG00000168724	123	717	527	687
ENSG00000168734	457	777	330	570
ENSG00000168743	0	0	0	1
ENSG00000168746	0	0	0	0
ENSG00000168748	2	17	0	0
ENSG00000168754	0	0	0	0
ENSG00000168757	0	0	0	0
ENSG00000168758	0	0	0	0
ENSG00000168763	1	0	2	4
ENSG00000168765	242	540	259	400
ENSG00000168769	175	4739	4738	6347
ENSG00000168772	67	441	457	833
ENSG00000168778	0	104	61	108
ENSG00000168779	0	0	0	0
ENSG00000168781	1	0	3	0
ENSG00000168785	171	355	387	225
ENSG00000168787	0	0	0	0
ENSG00000168792	3	0	9	2
ENSG00000168795	1	0	7	1
ENSG00000168802	159	234	183	231
ENSG00000168803	21	692	426	1589
ENSG00000168806	5	5	6	4
ENSG00000168807	22	223	126	190
ENSG00000168811	0	0	0	0
ENSG00000168813	8	168	185	175

ENSG00000168818	17	706	464	969
ENSG00000168824	1	0	0	3
ENSG00000168826	2	208	175	344
ENSG00000168827	9	110	121	222
ENSG00000168828	0	0	0	0
ENSG00000168830	0	0	0	0
ENSG00000168843	0	0	0	0
ENSG00000168852	1	1238	609	915
ENSG00000168872	15	25	16	24
ENSG00000168874	1	0	0	0
ENSG00000168875	0	0	0	0
ENSG00000168876	26	44	55	23
ENSG00000168878	1	0	0	0
ENSG00000168883	304	1345	906	866
ENSG00000168884	19	106	22	86
ENSG00000168887	560	615	515	443
ENSG00000168890	1	0	3	0
ENSG00000168894	468	125	100	138
ENSG00000168899	369	29	0	7
ENSG00000168903	0	0	1	0
ENSG00000168904	267	891	881	1177
ENSG00000168906	52	94	135	241
ENSG00000168907	0	0	0	0
ENSG00000168913	3	0	0	0
ENSG00000168916	1	0	0	0
ENSG00000168917	3	0	1	1
ENSG00000168918	60	75	38	88
ENSG00000168924	6	15	0	9
ENSG00000168925	0	0	0	0
ENSG00000168928	0	0	0	0
ENSG00000168930	0	0	0	0
ENSG00000168936	20	79	39	34
ENSG00000168938	1	0	0	4
ENSG00000168939	3	13	32	12
ENSG00000168944	17	291	385	399
ENSG00000168952	4	0	1	1
ENSG00000168955	0	0	0	0
ENSG00000168958	293	2076	1933	2525
ENSG00000168959	0	0	0	0
ENSG00000168961	71	0	0	2
ENSG00000168967	0	0	0	0
ENSG00000168970	0	0	0	0
ENSG00000168992	0	0	0	0
ENSG00000168993	0	0	0	0
ENSG00000168994	34	117	92	151
ENSG00000168995	2	0	0	0

ENSG00000169006	0	0	0	0
ENSG00000169016	18	35	79	87
ENSG00000169018	9	276	155	170
ENSG00000169019	44	22	14	22
ENSG00000169020	343	188	77	224
ENSG00000169021	97	114	65	128
ENSG00000169026	0	0	0	0
ENSG00000169031	0	0	0	0
ENSG00000169032	56	441	415	403
ENSG00000169035	0	0	0	0
ENSG00000169040	0	0	0	0
ENSG00000169045	450	1287	1357	1317
ENSG00000169047	140	621	305	237
ENSG00000169057	245	687	711	842
ENSG00000169059	0	0	0	0
ENSG00000169062	668	672	630	344
ENSG00000169064	0	33	7	34
ENSG00000169067	0	0	0	0
ENSG00000169071	0	0	0	0
ENSG00000169075	0	0	0	0
ENSG00000169083	173	262	323	319
ENSG00000169084	7	189	141	285
ENSG00000169085	0	0	1	11
ENSG00000169087	4	67	76	195
ENSG00000169093	15	0	7	8
ENSG00000169100	384	26	27	81
ENSG00000169105	0	0	0	0
ENSG00000169116	3	7	0	0
ENSG00000169118	8	5	9	1
ENSG00000169122	37	91	38	16
ENSG00000169126	0	26	15	292
ENSG00000169129	196	488	229	110
ENSG00000169131	3	0	3	4
ENSG00000169136	13	0	6	4
ENSG00000169139	116	828	1138	1484
ENSG00000169154	0	0	0	0
ENSG00000169155	23	58	29	26
ENSG00000169164	0	0	0	0
ENSG00000169169	10	0	0	1
ENSG00000169174	0	0	0	0
ENSG00000169180	37	300	404	484
ENSG00000169181	0	0	2	0
ENSG00000169184	0	0	2	0
ENSG00000169188	17	21	16	20
ENSG00000169189	36	89	21	71
ENSG00000169193	6	5962	3684	5446

ENSG00000169194	0	0	0	0
ENSG00000169202	0	0	0	0
ENSG00000169203	0	0	0	0
ENSG00000169208	0	0	0	0
ENSG00000169213	0	0	0	0
ENSG00000169214	0	0	0	0
ENSG00000169217	34	74	27	48
ENSG00000169218	0	0	0	0
ENSG00000169220	3	0	0	2
ENSG00000169221	39	42	15	14
ENSG00000169223	33	178	90	243
ENSG00000169224	144	1827	3265	1677
ENSG00000169228	0	0	0	0
ENSG00000169230	5	2	16	39
ENSG00000169231	3	17	2	1
ENSG00000169239	7	5	33	33
ENSG00000169241	772	634	472	543
ENSG00000169242	1	0	0	0
ENSG00000169245	1	0	0	4
ENSG00000169246	4	4	7	9
ENSG00000169247	115	2294	2152	1617
ENSG00000169248	1	51	6	25
ENSG00000169249	36	134	149	184
ENSG00000169251	22	741	607	802
ENSG00000169252	2	0	0	0
ENSG00000169253	0	0	0	0
ENSG00000169255	0	0	2	6
ENSG00000169258	0	0	0	0
ENSG00000169260	0	0	0	0
ENSG00000169271	15	102	119	29
ENSG00000169282	1	0	28	47
ENSG00000169288	26	39	85	91
ENSG00000169291	44	191	100	37
ENSG00000169297	0	0	2	0
ENSG00000169299	13	589	397	724
ENSG00000169302	0	0	1	0
ENSG00000169306	0	98	41	44
ENSG00000169313	756	23159	26940	17726
ENSG00000169314	16	12	9	7
ENSG00000169325	0	0	0	0
ENSG00000169327	0	0	0	0
ENSG00000169330	0	16	11	19
ENSG00000169340	0	0	0	0
ENSG00000169344	0	0	0	0
ENSG00000169347	0	0	0	0
ENSG00000169359	4	2236	2511	2576

ENSG00000169371	13	35	46	138
ENSG00000169372	10	19	37	52
ENSG00000169375	66	582	552	513
ENSG00000169379	60	1160	1793	3614
ENSG00000169385	1	0	0	2
ENSG00000169393	0	0	0	0
ENSG00000169397	0	0	0	3
ENSG00000169398	390	23815	20320	28407
ENSG00000169402	0	0	0	0
ENSG00000169403	240	244	123	116
ENSG00000169410	24	9	9	22
ENSG00000169413	9	19	0	1
ENSG00000169418	0	0	0	0
ENSG00000169427	0	0	0	0
ENSG00000169429	0	2	4	3
ENSG00000169432	1	22	11	35
ENSG00000169435	0	0	0	2
ENSG00000169436	0	0	0	1
ENSG00000169439	1	0	0	0
ENSG00000169442	921	0	11	1
ENSG00000169446	17	296	702	661
ENSG00000169469	0	0	0	0
ENSG00000169474	0	0	0	0
ENSG00000169484	0	0	0	0
ENSG00000169488	0	0	0	0
ENSG00000169490	735	3859	3098	2115
ENSG00000169495	0	0	0	0
ENSG00000169499	88	902	746	235
ENSG00000169504	697	22760	25370	27030
ENSG00000169507	0	0	0	0
ENSG00000169508	35	365	275	134
ENSG00000169509	0	0	0	0
ENSG00000169515	0	0	0	0
ENSG00000169519	9	1621	1867	2494
ENSG00000169548	0	0	0	0
ENSG00000169550	0	0	0	0
ENSG00000169551	0	0	0	0
ENSG00000169554	1337	9553	10738	9055
ENSG00000169562	0	0	0	0
ENSG00000169564	754	1894	1526	3029
ENSG00000169567	202	32	29	14
ENSG00000169570	1	168	145	170
ENSG00000169575	0	0	0	0
ENSG00000169583	4	0	0	0
ENSG00000169592	0	14	8	13
ENSG00000169594	0	0	0	0

ENSG00000169598	2	67	9	41
ENSG00000169599	30	225	112	187
ENSG00000169604	0	0	0	2
ENSG00000169605	0	0	0	0
ENSG00000169607	5	126	172	210
ENSG00000169609	42	13	12	30
ENSG00000169612	25	116	52	64
ENSG00000169618	0	0	0	0
ENSG00000169621	29	15344	19502	24023
ENSG00000169627	3	11	0	0
ENSG00000169629	0	103	68	89
ENSG00000169635	0	0	0	0
ENSG00000169641	19	4	39	54
ENSG00000169660	4	18	1	0
ENSG00000169662	0	0	0	0
ENSG00000169668	3	15	10	10
ENSG00000169676	0	0	0	0
ENSG00000169679	12	428	407	559
ENSG00000169682	7	0	1	0
ENSG00000169683	0	0	0	2
ENSG00000169684	0	133	165	173
ENSG00000169688	0	0	0	0
ENSG00000169689	39	10	3	18
ENSG00000169692	1	0	6	3
ENSG00000169696	3	0	1	7
ENSG00000169704	458	3643	2610	1866
ENSG00000169710	0	0	1	6
ENSG00000169714	166	99	126	196
ENSG00000169715	11	12	2	0
ENSG00000169717	0	0	0	0
ENSG00000169718	9	0	3	15
ENSG00000169727	3	24	7	8
ENSG00000169733	20	36	15	32
ENSG00000169738	18	0	4	1
ENSG00000169740	11	25	8	21
ENSG00000169744	11	80	11	1
ENSG00000169750	0	0	0	0
ENSG00000169752	1	0	1	0
ENSG00000169756	45441	75040	111983	99492
ENSG00000169758	0	0	0	2
ENSG00000169760	35	186	414	314
ENSG00000169762	56	569	545	659
ENSG00000169763	0	0	0	0
ENSG00000169764	770	6511	7573	9399
ENSG00000169777	0	0	0	0
ENSG00000169783	0	0	0	0

ENSG00000169789	0	0	0	0
ENSG00000169800	0	0	0	0
ENSG00000169807	0	0	0	0
ENSG00000169811	0	0	0	0
ENSG00000169813	376	1159	1000	1210
ENSG00000169814	5	0	13	0
ENSG00000169826	15	1547	2554	3017
ENSG00000169836	0	0	0	1
ENSG00000169840	0	0	0	0
ENSG00000169849	0	0	0	0
ENSG00000169851	0	0	0	0
ENSG00000169855	2	50	41	64
ENSG00000169856	0	0	0	0
ENSG00000169857	9	64	95	43
ENSG00000169860	239	1482	2909	1110
ENSG00000169861	0	0	0	0
ENSG00000169862	47	53	102	138
ENSG00000169871	28	40	26	15
ENSG00000169876	0	0	0	0
ENSG00000169877	25	0	0	5
ENSG00000169884	0	0	0	0
ENSG00000169885	0	0	0	0
ENSG00000169891	336	5170	4438	7328
ENSG00000169894	0	0	0	0
ENSG00000169895	53	350	345	501
ENSG00000169896	230	1131	412	435
ENSG00000169900	0	0	0	0
ENSG00000169902	2	827	657	1050
ENSG00000169903	0	0	0	0
ENSG00000169905	73	583	703	693
ENSG00000169906	0	0	0	0
ENSG00000169908	25	417	162	218
ENSG00000169914	1	45	283	319
ENSG00000169918	1	0	2	3
ENSG00000169919	61	33	92	155
ENSG00000169925	568	1319	669	586
ENSG00000169926	47	121	98	67
ENSG00000169933	0	0	0	0
ENSG00000169946	6	640	644	1370
ENSG00000169951	2	0	1	0
ENSG00000169953	0	0	0	0
ENSG00000169955	11	10	12	26
ENSG00000169957	2	47	28	62
ENSG00000169962	0	0	0	0
ENSG00000169964	2	0	0	3
ENSG00000169967	107	857	742	751

ENSG00000169972	2	0	0	0
ENSG00000169976	233	161	16	43
ENSG00000169981	7	17	1	4
ENSG00000169989	0	0	0	0
ENSG00000169991	2	0	0	0
ENSG00000169992	0	0	0	8
ENSG00000169994	0	0	0	0
ENSG00000170004	63	566	338	733
ENSG00000170006	14	107	93	48
ENSG00000170011	0	0	0	4
ENSG00000170017	27	243	464	508
ENSG00000170027	147	1443	1320	2107
ENSG00000170035	1309	4794	5114	6260
ENSG00000170037	54	122	99	293
ENSG00000170043	4342	2440	1414	2925
ENSG00000170044	0	0	0	0
ENSG00000170049	0	0	0	0
ENSG00000170054	0	0	0	0
ENSG00000170074	0	0	2	0
ENSG00000170075	0	0	0	0
ENSG00000170085	2	0	37	89
ENSG00000170088	3	129	107	127
ENSG00000170089	1	1	2	24
ENSG00000170091	1	0	0	0
ENSG00000170092	0	0	0	1
ENSG00000170099	0	0	0	0
ENSG00000170100	219	235	290	116
ENSG00000170113	57	696	651	656
ENSG00000170122	0	0	0	0
ENSG00000170128	0	0	0	0
ENSG00000170142	109	902	604	384
ENSG00000170144	343	1154	1710	1315
ENSG00000170145	13	227	160	178
ENSG00000170152	0	0	0	0
ENSG00000170153	0	0	1	0
ENSG00000170160	0	32	64	14
ENSG00000170161	0	0	0	2
ENSG00000170162	0	0	0	0
ENSG00000170165	0	0	0	0
ENSG00000170166	0	0	0	0
ENSG00000170175	256	105	121	100
ENSG00000170178	0	0	0	0
ENSG00000170180	3	13	6	30
ENSG00000170185	16	108	217	166
ENSG00000170190	29	70	9	37
ENSG00000170191	0	0	7	1

ENSG00000170209	0	0	0	0
ENSG00000170214	0	0	0	0
ENSG00000170215	0	0	0	0
ENSG00000170217	0	0	0	0
ENSG00000170222	10	0	23	20
ENSG00000170231	1	17	7	4
ENSG00000170234	34	1987	2440	2432
ENSG00000170236	0	53	40	53
ENSG00000170242	470	3468	3606	3829
ENSG00000170248	40	971	722	1074
ENSG00000170255	0	0	0	0
ENSG00000170260	1	0	2	1
ENSG00000170262	0	0	0	0
ENSG00000170264	1	0	5	7
ENSG00000170265	2	32	15	12
ENSG00000170266	38	387	235	250
ENSG00000170270	42	154	41	89
ENSG00000170271	3786	13210	14441	11610
ENSG00000170275	184	973	613	1003
ENSG00000170276	0	0	0	0
ENSG00000170279	0	0	0	0
ENSG00000170289	0	0	0	0
ENSG00000170290	0	0	0	0
ENSG00000170291	21	17	1	9
ENSG00000170293	0	0	0	1
ENSG00000170296	23	60	13	20
ENSG00000170298	4	0	0	0
ENSG00000170310	63	46	49	121
ENSG00000170312	7	32	34	88
ENSG00000170315	1602	9939	7186	10547
ENSG00000170322	12	0	11	4
ENSG00000170323	144	208	23	56
ENSG00000170324	0	0	0	0
ENSG00000170325	3	0	5	1
ENSG00000170340	13	223	223	161
ENSG00000170345	1	0	2	0
ENSG00000170348	53	434	385	418
ENSG00000170356	0	0	0	0
ENSG00000170364	4	20	22	15
ENSG00000170365	175	334	295	475
ENSG00000170367	0	0	0	0
ENSG00000170369	0	0	0	0
ENSG00000170370	0	0	0	0
ENSG00000170373	0	0	0	0
ENSG00000170374	0	0	0	0
ENSG00000170379	0	0	0	2

ENSG00000170381	0	0	0	0
ENSG00000170382	0	12	0	0
ENSG00000170385	11	980	1944	805
ENSG00000170390	0	310	193	304
ENSG00000170396	1	54	23	9
ENSG00000170409	0	0	0	1
ENSG00000170412	1	0	0	0
ENSG00000170417	2	576	730	260
ENSG00000170419	0	7	0	0
ENSG00000170421	190	52	80	71
ENSG00000170423	0	0	0	0
ENSG00000170425	54	187	219	117
ENSG00000170426	0	0	0	0
ENSG00000170430	16	0	11	14
ENSG00000170439	0	0	0	0
ENSG00000170442	0	0	0	0
ENSG00000170445	11	0	2	5
ENSG00000170448	3	274	200	243
ENSG00000170454	0	0	0	0
ENSG00000170456	4	171	109	151
ENSG00000170458	35	13	3	1
ENSG00000170464	1	0	2	0
ENSG00000170465	0	0	0	0
ENSG00000170468	0	0	0	0
ENSG00000170469	0	0	5	1
ENSG00000170471	18	595	474	615
ENSG00000170473	140	79	50	148
ENSG00000170476	2	0	0	0
ENSG00000170477	0	0	0	0
ENSG00000170482	1	0	0	0
ENSG00000170484	0	0	0	0
ENSG00000170485	0	0	0	0
ENSG00000170486	0	0	0	0
ENSG00000170498	0	0	0	0
ENSG00000170500	0	0	0	0
ENSG00000170502	6	4	6	10
ENSG00000170509	1	0	1	0
ENSG00000170515	125	2	31	24
ENSG00000170516	0	0	0	0
ENSG00000170522	29	22	51	50
ENSG00000170523	0	0	0	0
ENSG00000170525	27	223	196	115
ENSG00000170537	0	0	0	0
ENSG00000170540	148	2195	2308	1515
ENSG00000170542	196	842	664	848
ENSG00000170545	8	0	3	0

ENSG00000170549	0	0	0	0
ENSG00000170558	0	0	1	0
ENSG00000170561	0	0	0	0
ENSG00000170571	21	1559	1418	1924
ENSG00000170577	0	0	0	0
ENSG00000170579	1	3	38	7
ENSG00000170581	27	34	25	44
ENSG00000170584	8	0	12	20
ENSG00000170590	0	0	0	0
ENSG00000170604	1	14	0	0
ENSG00000170605	0	0	0	0
ENSG00000170606	34	1050	1155	1861
ENSG00000170608	0	0	0	0
ENSG00000170613	0	0	0	0
ENSG00000170615	0	0	0	0
ENSG00000170616	0	0	0	0
ENSG00000170619	10	29	5	7
ENSG00000170624	0	0	0	0
ENSG00000170627	1	39	18	43
ENSG00000170629	2	43	24	31
ENSG00000170631	2	0	0	1
ENSG00000170632	29	44	47	47
ENSG00000170633	61	976	567	939
ENSG00000170634	46	39	181	210
ENSG00000170638	11	0	1	4
ENSG00000170647	0	0	1	0
ENSG00000170653	37	58	72	127
ENSG00000170667	0	0	0	0
ENSG00000170677	1	64	31	40
ENSG00000170681	19	83	134	144
ENSG00000170683	0	0	0	0
ENSG00000170684	2	0	0	0
ENSG00000170688	0	0	0	0
ENSG00000170689	1	0	1	0
ENSG00000170703	0	0	0	0
ENSG00000170727	0	0	0	3
ENSG00000170734	9	0	6	7
ENSG00000170743	1	0	0	0
ENSG00000170745	0	0	0	0
ENSG00000170748	0	0	0	0
ENSG00000170759	336	4335	5121	5931
ENSG00000170775	0	0	0	0
ENSG00000170776	181	891	827	600
ENSG00000170777	0	0	0	0
ENSG00000170779	4	36	22	33
ENSG00000170782	0	0	0	0

ENSG00000170786	0	0	0	0
ENSG00000170788	0	0	0	0
ENSG00000170790	0	0	0	0
ENSG00000170791	55	96	68	133
ENSG00000170801	0	0	0	0
ENSG00000170802	214	4812	5332	4422
ENSG00000170803	0	0	0	0
ENSG00000170807	1	0	1	0
ENSG00000170819	0	0	0	0
ENSG00000170820	0	0	1	0
ENSG00000170827	0	0	0	0
ENSG00000170832	57	1436	1488	1892
ENSG00000170835	0	36	6	13
ENSG00000170836	4	123	195	292
ENSG00000170837	5	0	0	0
ENSG00000170846	37	61	51	73
ENSG00000170848	0	0	0	0
ENSG00000170852	84	796	1251	528
ENSG00000170854	6	0	3	3
ENSG00000170855	16	46	10	8
ENSG00000170858	0	0	0	0
ENSG00000170860	119	101	175	506
ENSG00000170866	4	0	0	0
ENSG00000170871	82	336	617	578
ENSG00000170873	276	3164	3419	3286
ENSG00000170876	3	6	17	8
ENSG00000170881	11	51	70	57
ENSG00000170889	484	96	50	76
ENSG00000170890	0	0	0	0
ENSG00000170891	1	0	4	0
ENSG00000170892	14	0	7	10
ENSG00000170893	0	0	0	0
ENSG00000170899	3	17	6	1
ENSG00000170903	3	20	32	42
ENSG00000170906	689	32	19	91
ENSG00000170909	0	0	0	0
ENSG00000170915	2	37	10	14
ENSG00000170917	11	149	78	89
ENSG00000170919	9	0	27	63
ENSG00000170920	0	0	0	0
ENSG00000170921	1	56	18	28
ENSG00000170923	0	0	0	0
ENSG00000170925	0	0	0	0
ENSG00000170927	0	0	1	0
ENSG00000170929	0	0	0	0
ENSG00000170935	10	63	230	119

ENSG00000170941	0	0	0	0
ENSG00000170946	9	161	119	142
ENSG00000170948	0	0	0	0
ENSG00000170949	24	163	402	466
ENSG00000170950	0	0	0	0
ENSG00000170953	0	0	17	0
ENSG00000170954	3	0	1	0
ENSG00000170955	0	0	0	0
ENSG00000170956	2	9	1	0
ENSG00000170959	0	0	5	0
ENSG00000170961	0	0	0	0
ENSG00000170962	7	245	200	168
ENSG00000170965	0	0	0	0
ENSG00000170967	0	0	0	0
ENSG00000170983	0	0	0	0
ENSG00000170989	4	0	1	0
ENSG00000171004	0	0	0	0
ENSG00000171014	0	0	0	0
ENSG00000171016	0	0	1	0
ENSG00000171017	0	0	0	0
ENSG00000171033	161	1189	1764	2192
ENSG00000171044	3	6	7	10
ENSG00000171045	0	0	1	0
ENSG00000171049	0	0	1	5
ENSG00000171051	11	0	3	2
ENSG00000171053	0	0	0	0
ENSG00000171054	0	0	0	0
ENSG00000171055	155	896	856	871
ENSG00000171056	7	0	2	0
ENSG00000171060	0	0	0	0
ENSG00000171067	25	14	57	68
ENSG00000171084	0	0	1	0
ENSG00000171094	0	0	0	0
ENSG00000171097	2	0	0	1
ENSG00000171100	31	1568	1317	3114
ENSG00000171101	7	0	0	0
ENSG00000171102	0	0	0	0
ENSG00000171103	0	99	197	263
ENSG00000171105	13	57	44	72
ENSG00000171109	6	861	515	1039
ENSG00000171115	21	0	3	0
ENSG00000171116	0	0	0	0
ENSG00000171119	0	0	0	0
ENSG00000171121	0	2	3	4
ENSG00000171124	0	0	0	0
ENSG00000171126	0	0	0	0

ENSG00000171129	0	0	2	0
ENSG00000171130	1	0	2	4
ENSG00000171132	0	0	1	6
ENSG00000171133	0	0	1	0
ENSG00000171135	6	0	1	0
ENSG00000171136	0	0	0	0
ENSG00000171148	711	1281	823	1858
ENSG00000171150	29	233	380	280
ENSG00000171155	3	0	44	39
ENSG00000171159	541	835	489	867
ENSG00000171160	0	0	0	0
ENSG00000171161	270	561	410	285
ENSG00000171163	2	0	7	4
ENSG00000171169	7	0	5	8
ENSG00000171174	0	19	5	14
ENSG00000171180	0	0	2	0
ENSG00000171189	1	0	0	1
ENSG00000171195	0	0	0	0
ENSG00000171199	0	0	0	0
ENSG00000171201	0	0	0	0
ENSG00000171202	12	0	1	1
ENSG00000171204	30	43	55	129
ENSG00000171206	110	416	295	200
ENSG00000171208	7	0	11	7
ENSG00000171209	0	0	0	0
ENSG00000171217	0	0	0	0
ENSG00000171219	0	0	0	0
ENSG00000171222	10	24	22	27
ENSG00000171223	8	0	2	2
ENSG00000171224	1	0	0	0
ENSG00000171227	0	0	0	0
ENSG00000171234	0	0	0	0
ENSG00000171236	2	0	1	0
ENSG00000171241	13	189	71	159
ENSG00000171243	0	0	0	0
ENSG00000171246	0	0	0	0
ENSG00000171262	16	288	250	379
ENSG00000171282	0	0	0	0
ENSG00000171291	2	0	2	5
ENSG00000171295	0	0	2	0
ENSG00000171298	4	0	0	0
ENSG00000171302	14	96	48	46
ENSG00000171303	0	0	0	0
ENSG00000171307	8	36	9	40
ENSG00000171310	27	168	127	185
ENSG00000171311	22	666	214	649

ENSG00000171314	290	441	333	484
ENSG00000171316	10	57	50	72
ENSG00000171320	4	0	37	30
ENSG00000171345	0	0	0	0
ENSG00000171346	0	0	0	0
ENSG00000171357	0	0	0	0
ENSG00000171360	0	0	0	0
ENSG00000171365	2	0	1	3
ENSG00000171368	0	0	0	0
ENSG00000171385	487	855	582	1348
ENSG00000171388	0	21	0	0
ENSG00000171396	0	0	0	0
ENSG00000171401	0	0	0	0
ENSG00000171402	0	0	0	0
ENSG00000171403	0	0	0	0
ENSG00000171405	0	0	0	0
ENSG00000171408	2	0	6	4
ENSG00000171421	23	0	7	4
ENSG00000171425	16	0	1	0
ENSG00000171428	4	55	19	9
ENSG00000171431	1	0	0	0
ENSG00000171433	18	64	7	5
ENSG00000171435	0	0	0	0
ENSG00000171443	11	58	41	38
ENSG00000171444	5	0	0	1
ENSG00000171446	0	0	0	0
ENSG00000171448	14	70	159	157
ENSG00000171450	0	0	0	0
ENSG00000171451	35	436	546	277
ENSG00000171453	13	0	0	0
ENSG00000171456	21	200	250	358
ENSG00000171459	0	0	0	0
ENSG00000171462	0	0	0	0
ENSG00000171466	7	1156	821	1560
ENSG00000171467	16	10	43	47
ENSG00000171469	3	86	42	71
ENSG00000171471	6	198	90	211
ENSG00000171475	257	502	412	325
ENSG00000171476	78	0	0	4
ENSG00000171478	0	0	0	0
ENSG00000171481	0	0	0	0
ENSG00000171483	0	0	0	0
ENSG00000171484	0	0	0	0
ENSG00000171487	0	0	0	0
ENSG00000171488	38	393	747	516
ENSG00000171489	0	0	0	0

ENSG00000171490	266	1251	460	1431
ENSG00000171492	881	6765	6414	5904
ENSG00000171495	0	0	0	0
ENSG00000171496	0	0	0	0
ENSG00000171497	22	324	205	344
ENSG00000171501	0	0	0	0
ENSG00000171502	34	2922	2378	4218
ENSG00000171503	3	45	49	114
ENSG00000171505	0	0	0	0
ENSG00000171509	0	0	2	0
ENSG00000171517	0	27	7	19
ENSG00000171522	8	66	11	9
ENSG00000171530	95	7	46	57
ENSG00000171532	0	0	0	0
ENSG00000171533	0	0	1	1
ENSG00000171540	0	0	0	0
ENSG00000171551	0	0	0	0
ENSG00000171552	826	5557	4469	3772
ENSG00000171557	0	0	0	0
ENSG00000171560	0	0	0	0
ENSG00000171561	1	0	0	0
ENSG00000171564	0	0	1	0
ENSG00000171566	17	49	22	53
ENSG00000171570	0	0	0	0
ENSG00000171574	0	0	2	5
ENSG00000171587	0	0	0	0
ENSG00000171595	0	0	0	0
ENSG00000171596	1	0	0	0
ENSG00000171603	1	0	8	23
ENSG00000171604	15	0	0	0
ENSG00000171606	14	50	19	32
ENSG00000171608	27	0	5	0
ENSG00000171611	1616	3205	1856	1862
ENSG00000171612	2	14	24	19
ENSG00000171617	4	0	2	2
ENSG00000171621	42	58	50	38
ENSG00000171631	0	0	0	0
ENSG00000171634	718	4731	4663	5420
ENSG00000171643	5	0	0	0
ENSG00000171649	6	11	15	46
ENSG00000171657	4	0	0	0
ENSG00000171658	6	0	11	17
ENSG00000171659	0	0	0	0
ENSG00000171671	0	0	0	0
ENSG00000171680	0	0	0	0
ENSG00000171681	143	4664	4973	6487

ENSG00000171695	0	0	0	0
ENSG00000171700	15	21	2	5
ENSG00000171703	26	21	8	33
ENSG00000171711	0	0	0	0
ENSG00000171714	0	5	74	0
ENSG00000171720	106	191	141	206
ENSG00000171722	0	0	0	0
ENSG00000171723	1	50	49	109
ENSG00000171724	0	0	0	0
ENSG00000171729	1	0	0	0
ENSG00000171735	3235	7688	5962	12302
ENSG00000171747	0	0	0	0
ENSG00000171757	1	222	133	165
ENSG00000171759	0	0	0	0
ENSG00000171763	1	0	33	32
ENSG00000171766	4	57	91	71
ENSG00000171772	0	0	0	0
ENSG00000171773	0	0	0	0
ENSG00000171777	11	0	0	0
ENSG00000171786	0	0	0	0
ENSG00000171790	0	0	0	0
ENSG00000171791	24	0	2	0
ENSG00000171792	8	34	7	14
ENSG00000171793	5	110	60	123
ENSG00000171794	0	0	0	0
ENSG00000171798	1	0	0	0
ENSG00000171804	0	0	0	0
ENSG00000171806	7	0	2	18
ENSG00000171811	0	0	0	0
ENSG00000171812	0	0	0	0
ENSG00000171813	0	0	0	0
ENSG00000171815	0	0	0	0
ENSG00000171817	0	0	1	6
ENSG00000171819	0	0	0	0
ENSG00000171823	0	0	0	0
ENSG00000171824	13	210	140	198
ENSG00000171827	9	158	62	68
ENSG00000171840	36	9	4	27
ENSG00000171843	305	2530	1942	1710
ENSG00000171847	0	0	0	0
ENSG00000171848	25	155	388	238
ENSG00000171853	30	177	59	115
ENSG00000171855	0	0	0	0
ENSG00000171858	939	66	16	18
ENSG00000171860	4	13	9	2
ENSG00000171861	2	11	13	21

ENSG00000171862	94	2202	3897	3704
ENSG00000171863	884	137	144	344
ENSG00000171864	0	0	0	0
ENSG00000171865	6	10	13	8
ENSG00000171867	32	204	226	588
ENSG00000171872	0	0	0	0
ENSG00000171873	0	0	0	0
ENSG00000171877	0	0	0	0
ENSG00000171885	0	0	0	0
ENSG00000171889	0	0	0	0
ENSG00000171903	0	0	0	0
ENSG00000171914	4	0	21	90
ENSG00000171916	7	0	0	11
ENSG00000171928	100	3383	2780	3108
ENSG00000171931	0	0	1	0
ENSG00000171936	0	0	0	0
ENSG00000171940	11	37	48	45
ENSG00000171942	0	0	0	0
ENSG00000171943	1	6	22	11
ENSG00000171944	0	0	0	0
ENSG00000171951	0	0	0	0
ENSG00000171953	4	8	32	21
ENSG00000171954	0	0	0	0
ENSG00000171956	0	0	0	0
ENSG00000171960	24	55	14	23
ENSG00000171962	2	0	6	12
ENSG00000171970	0	0	2	4
ENSG00000171984	5	34	13	29
ENSG00000171987	0	0	0	0
ENSG00000171988	120	3287	4215	3822
ENSG00000171989	0	0	0	0
ENSG00000171992	6	0	3	1
ENSG00000171999	0	0	0	0
ENSG00000172000	4	0	0	1
ENSG00000172005	5	0	0	0
ENSG00000172006	11	0	3	4
ENSG00000172007	5	1	24	53
ENSG00000172009	0	0	1	6
ENSG00000172014	0	0	14	0
ENSG00000172016	0	0	0	0
ENSG00000172020	0	17	0	0
ENSG00000172023	4	10	4	8
ENSG00000172031	0	0	0	0
ENSG00000172037	9	30	39	72
ENSG00000172046	3	17	29	22
ENSG00000172053	76	44	5	19

ENSG00000172057	396	1478	1012	1279
ENSG00000172058	0	0	0	0
ENSG00000172059	57	346	359	277
ENSG00000172061	0	0	0	0
ENSG00000172062	3	240	86	78
ENSG00000172070	4	15	46	51
ENSG00000172071	2	149	160	266
ENSG00000172073	0	0	0	0
ENSG00000172081	73	125	29	49
ENSG00000172086	64	165	184	170
ENSG00000172113	13	0	0	0
ENSG00000172115	344	3956	4169	5091
ENSG00000172116	9	0	0	0
ENSG00000172123	0	105	79	100
ENSG00000172137	1	0	12	5
ENSG00000172139	0	87	116	113
ENSG00000172146	0	0	0	0
ENSG00000172148	0	0	0	0
ENSG00000172150	0	0	0	0
ENSG00000172154	0	0	0	0
ENSG00000172155	0	0	0	0
ENSG00000172156	0	0	0	0
ENSG00000172159	3097	15818	12801	7428
ENSG00000172164	292	3092	3209	4398
ENSG00000172167	0	383	273	350
ENSG00000172171	2	17	11	10
ENSG00000172172	24	85	75	60
ENSG00000172175	18	15	31	26
ENSG00000172179	0	0	0	0
ENSG00000172183	38	0	6	0
ENSG00000172186	0	0	0	0
ENSG00000172188	0	0	0	0
ENSG00000172197	99	130	330	719
ENSG00000172199	0	0	0	0
ENSG00000172201	0	0	1	0
ENSG00000172208	0	0	0	0
ENSG00000172209	0	0	0	0
ENSG00000172215	0	0	0	0
ENSG00000172216	19	0	0	0
ENSG00000172232	1	0	0	0
ENSG00000172236	0	0	0	0
ENSG00000172238	0	0	0	0
ENSG00000172239	27	98	176	317
ENSG00000172243	24	0	5	2
ENSG00000172244	0	4	61	60
ENSG00000172247	0	0	0	0

ENSG00000172250	0	0	0	0
ENSG00000172260	1	0	5	6
ENSG00000172262	43	1744	1736	2695
ENSG00000172264	15	38	36	45
ENSG00000172269	2	3	16	42
ENSG00000172270	95	905	542	830
ENSG00000172273	53	237	198	345
ENSG00000172283	0	0	0	0
ENSG00000172288	0	0	0	0
ENSG00000172289	0	0	0	0
ENSG00000172292	2	1838	1125	629
ENSG00000172294	0	0	0	0
ENSG00000172296	0	0	2	0
ENSG00000172297	0	0	0	0
ENSG00000172301	2	0	0	0
ENSG00000172315	6	9	12	9
ENSG00000172318	0	0	0	0
ENSG00000172320	0	0	0	0
ENSG00000172322	368	225	274	96
ENSG00000172324	0	0	0	0
ENSG00000172331	26	113	139	233
ENSG00000172332	0	0	0	0
ENSG00000172336	8	0	0	2
ENSG00000172339	1	0	0	0
ENSG00000172340	19	271	258	560
ENSG00000172342	0	0	0	0
ENSG00000172345	2	0	0	4
ENSG00000172346	0	0	0	0
ENSG00000172348	0	0	0	0
ENSG00000172349	220	340	280	832
ENSG00000172350	0	0	0	0
ENSG00000172352	0	0	0	0
ENSG00000172354	29	114	72	103
ENSG00000172361	4	0	7	11
ENSG00000172362	0	0	0	0
ENSG00000172365	0	0	0	0
ENSG00000172366	3	0	0	0
ENSG00000172367	0	0	0	0
ENSG00000172375	23	50	47	73
ENSG00000172377	0	0	0	0
ENSG00000172379	0	0	0	0
ENSG00000172380	12	24	5	20
ENSG00000172381	0	0	0	0
ENSG00000172382	12	26	8	71
ENSG00000172399	0	0	0	0
ENSG00000172403	0	0	5	3

ENSG00000172404	0	3	0	0
ENSG00000172409	4	0	3	3
ENSG00000172410	0	12	2	0
ENSG00000172421	0	0	0	0
ENSG00000172425	0	0	0	0
ENSG00000172426	147	49	82	98
ENSG00000172428	392	39	7	18
ENSG00000172432	409	3720	2663	2075
ENSG00000172456	4	288	270	297
ENSG00000172457	0	0	0	0
ENSG00000172458	2	0	1	0
ENSG00000172459	0	0	0	0
ENSG00000172460	2	15	0	0
ENSG00000172461	0	0	0	0
ENSG00000172464	0	0	0	0
ENSG00000172465	14	17	40	74
ENSG00000172466	661	4536	5982	2268
ENSG00000172468	0	0	0	0
ENSG00000172469	3	265	276	385
ENSG00000172476	0	0	13	30
ENSG00000172478	0	0	0	0
ENSG00000172482	0	0	0	0
ENSG00000172487	0	0	0	0
ENSG00000172489	0	0	0	0
ENSG00000172493	118	2405	2007	2318
ENSG00000172497	0	0	10	8
ENSG00000172500	51	10	16	23
ENSG00000172508	0	0	0	0
ENSG00000172519	0	0	0	0
ENSG00000172524	0	0	0	0
ENSG00000172530	5	1095	263	1249
ENSG00000172531	68	290	147	277
ENSG00000172534	57	117	68	121
ENSG00000172538	0	0	0	0
ENSG00000172543	1224	628	279	804
ENSG00000172548	0	0	0	0
ENSG00000172551	0	0	0	0
ENSG00000172554	0	0	0	0
ENSG00000172568	0	0	0	0
ENSG00000172572	91	4555	4944	5028
ENSG00000172575	22	3	2	5
ENSG00000172578	549	3853	4499	3595
ENSG00000172586	36	0	26	30
ENSG00000172590	23	0	0	4
ENSG00000172594	1	25	0	0
ENSG00000172602	0	0	0	0

ENSG00000172613	3	0	3	5
ENSG00000172638	22	46	7	4
ENSG00000172640	0	0	0	0
ENSG00000172650	0	0	0	0
ENSG00000172653	17	93	18	29
ENSG00000172660	9	225	125	335
ENSG00000172661	26	406	199	295
ENSG00000172663	28	24	9	11
ENSG00000172667	724	1477	2746	2751
ENSG00000172671	0	503	380	790
ENSG00000172673	8	0	0	0
ENSG00000172680	0	0	0	0
ENSG00000172687	2	0	20	21
ENSG00000172689	0	0	0	0
ENSG00000172716	0	104	54	92
ENSG00000172717	0	0	0	0
ENSG00000172724	1	0	0	0
ENSG00000172725	16	160	109	218
ENSG00000172728	2	39	28	40
ENSG00000172731	1	0	0	0
ENSG00000172732	14	30	8	15
ENSG00000172733	0	0	5	0
ENSG00000172738	0	4	14	26
ENSG00000172742	0	0	0	0
ENSG00000172746	0	0	0	0
ENSG00000172748	0	8	7	40
ENSG00000172752	0	8	0	0
ENSG00000172757	2800	8508	4225	9403
ENSG00000172765	10	932	1137	1971
ENSG00000172766	66	2842	2096	2907
ENSG00000172769	0	0	0	0
ENSG00000172771	0	0	2	0
ENSG00000172772	0	0	0	0
ENSG00000172774	0	0	0	0
ENSG00000172775	187	4999	4157	5482
ENSG00000172780	1	0	0	0
ENSG00000172782	0	0	0	0
ENSG00000172785	40	562	885	1722
ENSG00000172789	0	0	0	0
ENSG00000172794	3848	7811	5704	7987
ENSG00000172795	238	4851	8678	5037
ENSG00000172799	3	0	0	0
ENSG00000172803	0	162	143	83
ENSG00000172809	875	739	247	821
ENSG00000172817	0	0	16	0
ENSG00000172818	0	0	0	0

ENSG00000172819	47	436	241	503
ENSG00000172824	0	0	1	0
ENSG00000172828	1	0	0	0
ENSG00000172830	0	0	1	0
ENSG00000172831	27	16	6	13
ENSG00000172840	5	114	85	101
ENSG00000172845	88	1432	1566	1261
ENSG00000172850	0	0	0	0
ENSG00000172867	0	0	0	0
ENSG00000172869	44	2711	2451	3064
ENSG00000172878	0	0	4	9
ENSG00000172888	12	34	20	25
ENSG00000172889	59	182	151	125
ENSG00000172890	32	52	12	18
ENSG00000172893	37	48	93	171
ENSG00000172900	0	0	0	0
ENSG00000172901	0	0	0	3
ENSG00000172912	0	0	0	0
ENSG00000172915	27	594	760	729
ENSG00000172922	39	6	9	32
ENSG00000172927	745	1498	1062	857
ENSG00000172932	1	31	21	45
ENSG00000172935	0	0	0	0
ENSG00000172936	27	0	4	2
ENSG00000172938	0	0	0	0
ENSG00000172939	14	375	244	473
ENSG00000172940	0	0	0	0
ENSG00000172943	6	237	292	454
ENSG00000172954	5	1469	1001	1493
ENSG00000172955	5	0	7	1
ENSG00000172965	2804	5914	4637	5497
ENSG00000172967	1	0	47	25
ENSG00000172969	0	0	0	0
ENSG00000172971	0	0	0	0
ENSG00000172974	1	1	3	0
ENSG00000172977	1	41	10	36
ENSG00000172985	0	0	0	0
ENSG00000172986	0	0	0	0
ENSG00000172987	0	2	5	12
ENSG00000172992	222	857	737	1091
ENSG00000172995	0	0	0	0
ENSG00000173011	3	0	0	5
ENSG00000173013	0	0	0	0
ENSG00000173020	17	94	122	143
ENSG00000173039	78	105	66	70
ENSG00000173040	0	0	1	1

ENSG00000173041	3	1398	1287	1997
ENSG00000173064	122	2097	1356	2354
ENSG00000173065	112	127	115	179
ENSG00000173068	1	0	0	0
ENSG00000173077	0	0	0	0
ENSG00000173080	0	0	0	0
ENSG00000173083	123	2656	3235	3148
ENSG00000173085	35	425	294	275
ENSG00000173088	0	15	28	36
ENSG00000173093	0	0	0	0
ENSG00000173110	5	0	0	0
ENSG00000173113	159	129	31	72
ENSG00000173114	3	0	0	1
ENSG00000173120	53	300	553	754
ENSG00000173124	0	0	3	0
ENSG00000173137	0	0	0	0
ENSG00000173141	68	76	18	56
ENSG00000173145	29	61	120	190
ENSG00000173153	2	26	16	34
ENSG00000173156	0	0	0	0
ENSG00000173157	0	0	0	2
ENSG00000173163	20	51	61	131
ENSG00000173166	2	18	21	13
ENSG00000173171	8	0	0	0
ENSG00000173175	0	0	1	1
ENSG00000173193	82	423	389	248
ENSG00000173198	27	82	89	118
ENSG00000173200	4	234	194	264
ENSG00000173207	27	2	33	62
ENSG00000173208	2	0	0	0
ENSG00000173209	12	256	250	331
ENSG00000173210	4714	7960	7805	10300
ENSG00000173212	167	201	184	171
ENSG00000173213	0	0	0	0
ENSG00000173214	5	800	529	574
ENSG00000173218	74	577	476	357
ENSG00000173221	43	26	13	5
ENSG00000173226	8	169	194	246
ENSG00000173227	0	0	0	0
ENSG00000173230	220	1958	2092	2447
ENSG00000173231	0	0	0	0
ENSG00000173237	0	0	0	0
ENSG00000173239	0	0	0	0
ENSG00000173250	0	0	0	0
ENSG00000173253	0	0	0	0
ENSG00000173258	0	0	2	1

ENSG00000173261	0	0	0	0
ENSG00000173262	1	0	139	2
ENSG00000173264	73	111	85	104
ENSG00000173267	1	0	0	0
ENSG00000173269	0	0	0	2
ENSG00000173272	2	0	3	5
ENSG00000173273	14	257	269	343
ENSG00000173275	2	45	11	35
ENSG00000173276	6	0	9	10
ENSG00000173281	65	290	163	75
ENSG00000173285	0	0	0	0
ENSG00000173295	1	23	0	5
ENSG00000173302	0	0	0	0
ENSG00000173320	0	0	1	12
ENSG00000173327	13	52	39	77
ENSG00000173334	2	0	3	5
ENSG00000173335	0	0	0	0
ENSG00000173338	0	0	0	0
ENSG00000173349	0	18	8	12
ENSG00000173357	0	0	0	0
ENSG00000173366	0	0	0	0
ENSG00000173369	2	0	0	0
ENSG00000173372	5	0	0	0
ENSG00000173376	0	0	4	0
ENSG00000173389	0	0	0	0
ENSG00000173391	1	0	0	0
ENSG00000173401	0	0	0	2
ENSG00000173402	45	235	129	201
ENSG00000173404	0	0	0	0
ENSG00000173406	0	0	2	0
ENSG00000173409	12	126	140	179
ENSG00000173418	13	106	205	218
ENSG00000173421	0	0	0	0
ENSG00000173431	0	0	0	0
ENSG00000173432	0	0	0	0
ENSG00000173436	53	35	8	3
ENSG00000173442	1	39	12	19
ENSG00000173451	4	13	30	55
ENSG00000173452	0	0	0	0
ENSG00000173456	0	13	3	0
ENSG00000173457	18	0	11	11
ENSG00000173464	0	0	0	0
ENSG00000173465	6	0	0	5
ENSG00000173467	0	0	0	0
ENSG00000173473	387	2744	2342	3402
ENSG00000173480	2	0	8	0

ENSG00000173482	1	46	5	7
ENSG00000173486	91	14	37	53
ENSG00000173503	0	0	0	0
ENSG00000173511	21	52	31	36
ENSG00000173517	7	478	492	677
ENSG00000173530	0	0	0	0
ENSG00000173531	1	0	2	1
ENSG00000173535	0	0	0	0
ENSG00000173540	0	0	0	0
ENSG00000173542	1405	20465	36894	20512
ENSG00000173545	5	0	1	6
ENSG00000173546	0	0	0	0
ENSG00000173548	2	0	0	2
ENSG00000173557	0	0	0	0
ENSG00000173559	17	45	117	11
ENSG00000173566	4	0	0	0
ENSG00000173567	0	0	4	1
ENSG00000173572	0	0	0	0
ENSG00000173575	1374	5863	5466	6896
ENSG00000173578	1	0	0	0
ENSG00000173581	1	0	7	3
ENSG00000173585	0	0	0	0
ENSG00000173588	11	454	306	373
ENSG00000173597	0	0	2	0
ENSG00000173598	103	662	469	461
ENSG00000173599	0	0	0	0
ENSG00000173610	0	0	0	0
ENSG00000173611	7	1432	1291	1902
ENSG00000173612	0	0	0	0
ENSG00000173614	5	614	518	1024
ENSG00000173621	0	0	0	0
ENSG00000173626	2750	6712	6634	5624
ENSG00000173627	0	0	0	0
ENSG00000173638	1	0	0	0
ENSG00000173641	0	0	0	0
ENSG00000173653	2	0	2	0
ENSG00000173660	10207	8940	7140	17361
ENSG00000173662	0	0	0	0
ENSG00000173673	0	0	0	0
ENSG00000173674	284	1631	1942	2846
ENSG00000173678	0	0	0	0
ENSG00000173679	0	0	0	0
ENSG00000173681	30	1706	1523	1901
ENSG00000173692	82	721	827	1074
ENSG00000173698	0	0	3	0
ENSG00000173699	0	0	0	0

ENSG00000173702	0	0	0	0
ENSG00000173705	0	0	0	0
ENSG00000173706	1	0	7	16
ENSG00000173714	2	0	0	2
ENSG00000173715	2	36	19	30
ENSG00000173726	118	642	329	740
ENSG00000173727	19	12	2	7
ENSG00000173728	0	0	0	0
ENSG00000173744	30	569	768	558
ENSG00000173757	276	1097	916	1034
ENSG00000173762	0	0	0	0
ENSG00000173769	0	30	2	6
ENSG00000173786	13	19	16	32
ENSG00000173801	13	12	0	4
ENSG00000173805	0	0	0	0
ENSG00000173809	0	0	1	3
ENSG00000173810	0	0	0	0
ENSG00000173811	0	0	0	0
ENSG00000173812	12464	20315	18995	19191
ENSG00000173818	11	0	13	5
ENSG00000173821	154	201	222	198
ENSG00000173825	1	0	0	0
ENSG00000173826	0	0	0	0
ENSG00000173838	0	0	0	0
ENSG00000173846	4	0	0	0
ENSG00000173848	45	671	405	406
ENSG00000173852	153	5312	5491	6797
ENSG00000173862	0	0	0	0
ENSG00000173867	0	0	0	0
ENSG00000173868	2	0	2	0
ENSG00000173875	12	8	28	27
ENSG00000173876	0	0	0	0
ENSG00000173889	14	12675	8335	13576
ENSG00000173890	3	25	9	3
ENSG00000173894	0	0	0	0
ENSG00000173898	0	0	0	0
ENSG00000173905	55	2027	1864	2751
ENSG00000173908	0	0	0	0
ENSG00000173914	3	88	56	98
ENSG00000173915	946	500	520	801
ENSG00000173917	0	0	0	0
ENSG00000173918	0	0	0	0
ENSG00000173926	5	33	22	19
ENSG00000173928	8	2	2	4
ENSG00000173930	1	0	4	5
ENSG00000173933	12	26	29	29

ENSG00000173947	0	0	0	0
ENSG00000173950	6	20	18	23
ENSG00000173954	0	0	0	0
ENSG00000173960	474	5381	4913	7422
ENSG00000173966	0	0	0	0
ENSG00000173976	0	0	0	0
ENSG00000173988	0	60	49	39
ENSG00000173991	0	12	1	0
ENSG00000173992	299	149	54	36
ENSG00000174004	4	33	8	23
ENSG00000174007	17	0	10	3
ENSG00000174010	0	11	29	35
ENSG00000174013	5	0	7	17
ENSG00000174015	0	0	0	0
ENSG00000174016	0	0	0	0
ENSG00000174021	333	135	122	121
ENSG00000174028	0	1	29	45
ENSG00000174032	65	858	1660	826
ENSG00000174038	0	0	0	0
ENSG00000174059	6	0	10	9
ENSG00000174080	0	0	0	0
ENSG00000174083	15	27	47	53
ENSG00000174093	6	130	140	158
ENSG00000174099	533	2459	3828	2738
ENSG00000174100	14	0	22	22
ENSG00000174106	5	223	267	254
ENSG00000174109	5	2	5	1
ENSG00000174111	0	35	28	93
ENSG00000174123	2	0	2	0
ENSG00000174125	5	0	8	4
ENSG00000174130	1	13	12	25
ENSG00000174132	2	0	11	70
ENSG00000174136	1	0	0	0
ENSG00000174137	0	0	0	0
ENSG00000174145	0	0	0	0
ENSG00000174151	6	16	10	2
ENSG00000174156	0	0	0	0
ENSG00000174165	3	0	0	8
ENSG00000174171	1	0	0	0
ENSG00000174173	37	51	54	48
ENSG00000174175	2907	12738	11152	20773
ENSG00000174177	0	0	1	0
ENSG00000174194	0	0	0	1
ENSG00000174196	0	0	0	0
ENSG00000174197	21	3062	4023	4380
ENSG00000174206	2	0	0	2

ENSG00000174225	0	0	0	0
ENSG00000174226	0	0	0	2
ENSG00000174227	18	74	52	64
ENSG00000174231	120	547	335	567
ENSG00000174233	15	192	95	382
ENSG00000174236	0	0	0	0
ENSG00000174238	23	190	89	143
ENSG00000174243	24	97	70	78
ENSG00000174255	0	0	1	0
ENSG00000174276	0	0	0	0
ENSG00000174279	0	0	0	0
ENSG00000174282	161	451	407	491
ENSG00000174292	0	0	0	0
ENSG00000174306	16	68	58	55
ENSG00000174307	2	0	1	0
ENSG00000174325	0	0	0	0
ENSG00000174326	0	0	0	0
ENSG00000174327	0	0	0	0
ENSG00000174332	0	0	0	0
ENSG00000174339	0	0	0	0
ENSG00000174343	0	0	0	0
ENSG00000174348	0	0	0	0
ENSG00000174353	3	60	16	60
ENSG00000174358	0	0	0	0
ENSG00000174365	83	30	11	13
ENSG00000174368	0	0	7	25
ENSG00000174370	0	0	0	2
ENSG00000174371	0	42	73	102
ENSG00000174373	3	1043	1129	1776
ENSG00000174374	4	0	4	5
ENSG00000174384	0	0	0	0
ENSG00000174403	1	0	0	0
ENSG00000174405	29	383	456	486
ENSG00000174407	0	0	0	0
ENSG00000174408	0	0	0	0
ENSG00000174417	3	80	296	67
ENSG00000174418	0	0	0	2
ENSG00000174428	2	15	6	16
ENSG00000174429	0	0	0	0
ENSG00000174437	84	2239	1853	2708
ENSG00000174442	20	836	838	1346
ENSG00000174444	2665	4431	2993	4706
ENSG00000174446	42	105	71	105
ENSG00000174448	0	0	0	0
ENSG00000174450	0	0	0	0
ENSG00000174453	0	0	0	0

ENSG00000174456	817	5146	5070	3853
ENSG00000174460	0	0	0	0
ENSG00000174469	4	27	22	37
ENSG00000174473	0	0	0	0
ENSG00000174482	3	0	0	0
ENSG00000174483	0	0	0	3
ENSG00000174485	80	15367	19191	21786
ENSG00000174495	0	0	0	0
ENSG00000174498	0	0	0	0
ENSG00000174500	5	0	2	0
ENSG00000174501	96	830	1107	1673
ENSG00000174502	0	0	0	0
ENSG00000174514	4	0	10	0
ENSG00000174516	0	0	0	0
ENSG00000174521	0	0	0	0
ENSG00000174527	0	0	0	0
ENSG00000174529	2	0	0	2
ENSG00000174547	14	0	1	3
ENSG00000174562	0	0	0	0
ENSG00000174564	0	0	5	7
ENSG00000174567	0	0	0	0
ENSG00000174572	0	0	0	0
ENSG00000174574	700	1805	1729	1158
ENSG00000174576	0	0	1	0
ENSG00000174579	28	311	278	186
ENSG00000174586	1	23	3	1
ENSG00000174595	0	0	0	0
ENSG00000174599	0	20	0	0
ENSG00000174600	2	21	0	0
ENSG00000174606	18	57	244	387
ENSG00000174607	0	45	4	4
ENSG00000174611	0	9	1	0
ENSG00000174628	3	0	8	25
ENSG00000174640	6	30	28	124
ENSG00000174652	9	23	18	51
ENSG00000174667	0	0	0	0
ENSG00000174669	0	0	0	0
ENSG00000174672	0	0	0	0
ENSG00000174677	0	0	0	0
ENSG00000174678	0	19	0	0
ENSG00000174680	0	0	1	0
ENSG00000174684	0	0	0	0
ENSG00000174695	297	6216	7788	8062
ENSG00000174697	1	0	0	0
ENSG00000174705	0	39	1	16
ENSG00000174715	0	0	0	0

ENSG00000174718	70	242	389	195
ENSG00000174720	139	375	330	576
ENSG00000174721	0	0	0	0
ENSG00000174738	100	1276	746	512
ENSG00000174740	0	0	0	0
ENSG00000174744	21	0	9	12
ENSG00000174748	2335	4409	2536	3646
ENSG00000174749	21	43	96	58
ENSG00000174775	5	0	0	3
ENSG00000174776	0	111	114	219
ENSG00000174780	179	347	419	922
ENSG00000174788	24	351	157	198
ENSG00000174791	0	0	0	0
ENSG00000174792	0	0	0	0
ENSG00000174796	7	78	43	48
ENSG00000174799	96	2658	2406	3107
ENSG00000174804	0	0	0	0
ENSG00000174807	0	0	0	0
ENSG00000174808	0	0	5	3
ENSG00000174827	2	15	5	1
ENSG00000174837	127	1059	1679	1110
ENSG00000174839	18	944	1059	1351
ENSG00000174840	6	14	37	28
ENSG00000174842	1	357	250	443
ENSG00000174844	458	306	251	471
ENSG00000174851	3	0	0	0
ENSG00000174871	0	0	0	0
ENSG00000174876	0	0	0	0
ENSG00000174885	0	0	0	0
ENSG00000174886	84	10	4	0
ENSG00000174891	154	3552	4339	7025
ENSG00000174898	1	0	9	9
ENSG00000174899	4	0	20	22
ENSG00000174903	69	2933	2245	4011
ENSG00000174912	0	2	2	10
ENSG00000174914	0	0	0	0
ENSG00000174915	59	96	81	115
ENSG00000174917	13	43	0	1
ENSG00000174928	0	129	78	160
ENSG00000174930	0	0	0	0
ENSG00000174937	0	0	0	0
ENSG00000174938	0	0	0	0
ENSG00000174939	0	0	0	0
ENSG00000174942	0	0	0	0
ENSG00000174943	176	62	25	14
ENSG00000174944	2	1	6	0

ENSG00000174945	0	0	0	0
ENSG00000174946	1	0	0	0
ENSG00000174948	0	0	0	0
ENSG00000174950	0	0	0	0
ENSG00000174951	2	0	2	0
ENSG00000174953	82	324	541	726
ENSG00000174957	0	0	0	0
ENSG00000174963	0	0	0	0
ENSG00000174970	0	0	0	0
ENSG00000174977	0	0	2	0
ENSG00000174982	0	0	0	0
ENSG00000174989	1	76	87	140
ENSG00000174990	119	20	45	57
ENSG00000174992	0	0	0	0
ENSG00000174996	0	3	0	1
ENSG00000175003	0	0	0	0
ENSG00000175018	0	0	0	0
ENSG00000175029	69	518	300	468
ENSG00000175040	65	212	150	136
ENSG00000175048	2	0	5	15
ENSG00000175054	8	375	391	523
ENSG00000175061	52	42	92	105
ENSG00000175063	71	168	498	409
ENSG00000175065	0	0	1	0
ENSG00000175066	1	69	60	113
ENSG00000175073	62	816	1397	543
ENSG00000175077	0	0	0	0
ENSG00000175084	0	0	0	0
ENSG00000175087	8	23	110	52
ENSG00000175093	2	0	17	22
ENSG00000175097	0	0	0	0
ENSG00000175104	10	96	110	154
ENSG00000175105	15	130	164	119
ENSG00000175106	0	0	37	23
ENSG00000175110	6	0	8	15
ENSG00000175115	461	1117	810	1408
ENSG00000175121	0	0	0	0
ENSG00000175130	23	0	0	0
ENSG00000175137	134	251	249	363
ENSG00000175143	0	0	0	0
ENSG00000175147	0	0	0	5
ENSG00000175155	250	3356	2797	2306
ENSG00000175161	41	73	181	499
ENSG00000175164	38	142	88	147
ENSG00000175166	345	1138	783	899
ENSG00000175170	0	0	0	0

ENSG00000175175	0	37	11	11
ENSG00000175182	5	33	20	13
ENSG00000175183	0	0	0	2
ENSG00000175189	0	0	0	0
ENSG00000175193	23	428	547	613
ENSG00000175197	8	13	9	9
ENSG00000175198	2	2325	1686	2942
ENSG00000175202	0	0	0	0
ENSG00000175203	373	1967	997	1440
ENSG00000175206	1	3	3	8
ENSG00000175213	4	44	1	0
ENSG00000175215	585	1723	1075	1273
ENSG00000175216	103	5186	4023	4656
ENSG00000175220	75	47	44	104
ENSG00000175221	49	147	95	117
ENSG00000175224	113	243	139	180
ENSG00000175229	0	0	0	0
ENSG00000175262	1	0	2	0
ENSG00000175264	0	0	0	0
ENSG00000175265	0	23	0	0
ENSG00000175267	0	0	3	9
ENSG00000175274	65	19	44	155
ENSG00000175279	1	0	4	0
ENSG00000175283	0	0	0	5
ENSG00000175287	0	0	1	2
ENSG00000175294	174	155	88	65
ENSG00000175302	0	0	0	0
ENSG00000175305	1	106	113	94
ENSG00000175309	33	88	76	50
ENSG00000175311	0	0	0	0
ENSG00000175315	0	0	0	0
ENSG00000175318	0	0	0	0
ENSG00000175319	0	0	0	0
ENSG00000175322	0	2	12	21
ENSG00000175324	297	2321	2099	3794
ENSG00000175325	0	0	0	0
ENSG00000175329	0	0	0	0
ENSG00000175334	269	0	7	18
ENSG00000175336	0	0	0	0
ENSG00000175344	0	0	0	0
ENSG00000175348	332	1148	1072	1209
ENSG00000175352	1	0	11	0
ENSG00000175354	71	636	563	1051
ENSG00000175356	0	0	0	0
ENSG00000175376	9	4	12	23
ENSG00000175387	802	14380	13660	13084

ENSG00000175390	203	44	41	65
ENSG00000175393	0	0	0	0
ENSG00000175395	29	477	777	663
ENSG00000175398	0	0	2	0
ENSG00000175414	5	0	3	0
ENSG00000175416	23	181	33	113
ENSG00000175426	1	11	12	5
ENSG00000175445	3	76	8	7
ENSG00000175449	4	87	148	92
ENSG00000175455	57	871	1144	1254
ENSG00000175463	8	7	2	0
ENSG00000175467	21	35	7	4
ENSG00000175470	456	2178	1638	2700
ENSG00000175471	664	15340	14970	16548
ENSG00000175482	0	33	12	26
ENSG00000175485	0	0	0	0
ENSG00000175489	26	0	0	0
ENSG00000175497	0	80	63	0
ENSG00000175505	0	0	0	0
ENSG00000175509	3	25	4	27
ENSG00000175513	0	0	0	0
ENSG00000175514	0	0	0	0
ENSG00000175518	1	0	0	1
ENSG00000175520	0	0	0	0
ENSG00000175535	0	0	0	0
ENSG00000175536	0	0	9	1
ENSG00000175538	181	295	174	263
ENSG00000175544	2	0	0	1
ENSG00000175548	0	35	119	143
ENSG00000175550	93	421	167	223
ENSG00000175556	1	0	0	3
ENSG00000175564	2	0	0	0
ENSG00000175567	2115	726	438	1415
ENSG00000175573	180	46	27	21
ENSG00000175575	12	37	17	22
ENSG00000175581	24	8	21	32
ENSG00000175582	629	13979	15025	23226
ENSG00000175591	0	0	1	0
ENSG00000175592	378	0	3	10
ENSG00000175595	7	147	167	236
ENSG00000175600	0	34	16	43
ENSG00000175602	39	642	236	364
ENSG00000175604	0	0	0	0
ENSG00000175606	1067	2120	2413	692
ENSG00000175611	6	0	6	2
ENSG00000175619	0	0	0	0

ENSG00000175634	6	117	36	163
ENSG00000175643	11	28	21	41
ENSG00000175646	0	0	0	0
ENSG00000175658	0	0	0	0
ENSG00000175662	35	651	328	596
ENSG00000175664	0	0	0	0
ENSG00000175676	0	0	0	0
ENSG00000175691	0	0	4	3
ENSG00000175697	0	0	2	0
ENSG00000175699	0	0	0	0
ENSG00000175701	98	80	19	71
ENSG00000175707	1	0	0	0
ENSG00000175711	1	0	0	7
ENSG00000175718	0	0	0	0
ENSG00000175727	650	530	350	541
ENSG00000175728	0	0	0	0
ENSG00000175730	3	0	0	0
ENSG00000175741	0	0	0	0
ENSG00000175745	0	0	0	0
ENSG00000175746	398	7860	10819	6529
ENSG00000175749	1	0	0	0
ENSG00000175756	44	91	13	22
ENSG00000175764	0	0	22	35
ENSG00000175766	0	0	0	0
ENSG00000175768	0	0	6	1
ENSG00000175772	0	0	0	0
ENSG00000175773	1	0	0	9
ENSG00000175779	10	86	34	41
ENSG00000175782	18	28	24	59
ENSG00000175785	1	0	0	0
ENSG00000175787	3	12	4	16
ENSG00000175792	19	49	27	34
ENSG00000175793	0	0	0	0
ENSG00000175800	0	0	0	0
ENSG00000175806	69	220	147	194
ENSG00000175809	0	0	0	0
ENSG00000175820	0	0	0	0
ENSG00000175826	460	234	219	259
ENSG00000175827	0	0	0	0
ENSG00000175832	0	0	0	0
ENSG00000175841	0	0	0	0
ENSG00000175854	1629	594	265	398
ENSG00000175857	26	27	13	4
ENSG00000175866	6	46	13	4
ENSG00000175868	0	0	0	0
ENSG00000175873	3	84	57	174

ENSG00000175874	0	0	0	1
ENSG00000175877	0	0	0	0
ENSG00000175879	0	0	0	0
ENSG00000175886	0	0	0	2
ENSG00000175893	14	731	401	547
ENSG00000175894	0	0	0	0
ENSG00000175895	92	962	1236	736
ENSG00000175898	1	0	3	1
ENSG00000175899	7	43	17	22
ENSG00000175906	0	0	0	0
ENSG00000175911	3	0	0	4
ENSG00000175920	0	0	0	0
ENSG00000175928	0	0	0	0
ENSG00000175931	304	991	360	525
ENSG00000175938	115	25	46	58
ENSG00000175946	0	0	0	0
ENSG00000175967	0	0	0	0
ENSG00000175970	11	103	34	81
ENSG00000175984	396	5859	5405	6120
ENSG00000175985	4	9	9	9
ENSG00000176007	0	0	0	0
ENSG00000176009	0	0	0	0
ENSG00000176014	7	65	14	49
ENSG00000176018	0	0	2	0
ENSG00000176020	0	0	0	0
ENSG00000176022	3	25	0	3
ENSG00000176024	0	17	12	24
ENSG00000176029	0	0	0	0
ENSG00000176034	0	9	7	18
ENSG00000176040	0	0	1	3
ENSG00000176043	0	0	0	0
ENSG00000176046	0	0	0	3
ENSG00000176049	7	54	42	60
ENSG00000176054	0	0	0	0
ENSG00000176055	0	0	0	0
ENSG00000176058	1	16	0	0
ENSG00000176075	0	0	0	0
ENSG00000176076	0	0	0	0
ENSG00000176082	0	0	0	0
ENSG00000176083	4	0	0	0
ENSG00000176087	74	58	30	68
ENSG00000176092	0	0	0	0
ENSG00000176095	60	171	138	66
ENSG00000176101	48	56	19	13
ENSG00000176102	22	711	987	1360
ENSG00000176105	11	2310	1837	2595

ENSG00000176108	27	189	91	227
ENSG00000176115	0	0	0	0
ENSG00000176124	102	580	693	539
ENSG00000176125	0	0	1	0
ENSG00000176134	0	0	0	0
ENSG00000176136	0	0	0	0
ENSG00000176142	1	111	61	91
ENSG00000176148	2	126	154	152
ENSG00000176153	0	0	0	0
ENSG00000176155	160	159	151	287
ENSG00000176160	0	59	12	34
ENSG00000176165	0	0	0	0
ENSG00000176170	299	666	333	229
ENSG00000176171	34	293	234	856
ENSG00000176177	30	297	338	176
ENSG00000176182	2	3	0	1
ENSG00000176183	0	0	0	0
ENSG00000176194	0	0	0	0
ENSG00000176198	0	0	0	0
ENSG00000176200	4	0	0	0
ENSG00000176204	0	11	2	16
ENSG00000176208	11	1003	812	1210
ENSG00000176209	30	99	77	123
ENSG00000176219	0	0	0	0
ENSG00000176222	0	3	3	2
ENSG00000176225	2	170	126	244
ENSG00000176227	0	0	0	0
ENSG00000176230	0	0	0	0
ENSG00000176231	0	0	0	0
ENSG00000176232	0	0	0	0
ENSG00000176236	0	0	0	0
ENSG00000176239	0	0	0	0
ENSG00000176243	0	0	0	0
ENSG00000176244	0	1	0	0
ENSG00000176246	0	0	0	0
ENSG00000176248	0	3	7	21
ENSG00000176253	0	0	0	0
ENSG00000176256	0	0	0	0
ENSG00000176261	153	1370	1195	2195
ENSG00000176268	0	0	1	0
ENSG00000176269	0	0	0	0
ENSG00000176273	192	678	451	460
ENSG00000176274	145	82	109	147
ENSG00000176281	0	0	0	0
ENSG00000176289	0	0	0	0
ENSG00000176290	0	0	0	0

ENSG00000176293	7	0	8	27
ENSG00000176294	0	0	0	0
ENSG00000176299	0	0	0	0
ENSG00000176302	0	0	0	0
ENSG00000176305	0	0	0	0
ENSG00000176312	0	0	0	0
ENSG00000176318	0	0	0	0
ENSG00000176320	6	0	0	0
ENSG00000176340	270	4	9	18
ENSG00000176343	0	0	0	0
ENSG00000176349	0	0	0	0
ENSG00000176354	0	0	0	3
ENSG00000176358	0	0	0	0
ENSG00000176366	0	0	0	0
ENSG00000176371	11	0	7	5
ENSG00000176378	0	0	0	0
ENSG00000176381	0	0	0	0
ENSG00000176383	0	15	1	0
ENSG00000176386	19	29	15	68
ENSG00000176387	0	0	0	0
ENSG00000176390	32	1436	1865	2519
ENSG00000176393	21	21	0	8
ENSG00000176395	0	0	0	0
ENSG00000176396	5	20	2	4
ENSG00000176399	0	0	0	0
ENSG00000176401	5	0	0	0
ENSG00000176402	0	0	0	0
ENSG00000176406	1	1	0	0
ENSG00000176407	564	2159	2355	2125
ENSG00000176410	5	0	0	0
ENSG00000176422	1	0	2	0
ENSG00000176428	0	0	0	0
ENSG00000176435	0	0	0	0
ENSG00000176438	0	8	1	0
ENSG00000176444	5	126	110	357
ENSG00000176454	6	1	0	2
ENSG00000176463	249	265	119	89
ENSG00000176472	16	0	3	6
ENSG00000176473	1	0	1	3
ENSG00000176476	11	12	8	7
ENSG00000176485	19	0	0	8
ENSG00000176490	4	31	1	26
ENSG00000176495	0	0	0	0
ENSG00000176510	0	0	0	0
ENSG00000176515	0	0	0	0
ENSG00000176531	0	0	0	0

ENSG00000176532	0	0	0	0
ENSG00000176533	22	36	7	19
ENSG00000176540	0	0	0	0
ENSG00000176542	162	973	1868	1416
ENSG00000176547	0	0	0	5
ENSG00000176555	0	0	0	0
ENSG00000176563	2	21	59	30
ENSG00000176566	0	0	0	0
ENSG00000176567	0	0	0	0
ENSG00000176571	0	0	0	0
ENSG00000176584	0	0	0	0
ENSG00000176593	25	19	64	85
ENSG00000176595	1	0	0	0
ENSG00000176597	0	0	0	5
ENSG00000176601	0	0	0	0
ENSG00000176605	0	0	0	0
ENSG00000176619	2	0	3	12
ENSG00000176623	48	375	510	1019
ENSG00000176624	10	199	257	186
ENSG00000176635	0	0	0	0
ENSG00000176641	0	12	7	2
ENSG00000176654	0	0	0	0
ENSG00000176658	3	11	11	19
ENSG00000176659	38	111	259	148
ENSG00000176678	0	0	0	0
ENSG00000176679	0	0	0	0
ENSG00000176681	1	15	5	13
ENSG00000176692	0	0	0	0
ENSG00000176695	0	0	0	0
ENSG00000176697	16	100	113	95
ENSG00000176700	7	3	18	12
ENSG00000176714	1	0	0	4
ENSG00000176715	8	0	11	15
ENSG00000176716	0	0	0	0
ENSG00000176720	1	0	0	0
ENSG00000176723	0	0	0	4
ENSG00000176728	7	217	3	547
ENSG00000176731	19	41	32	50
ENSG00000176732	3	21	3	7
ENSG00000176734	0	0	0	0
ENSG00000176742	0	0	0	0
ENSG00000176746	0	0	0	0
ENSG00000176748	0	0	0	0
ENSG00000176749	4	0	0	0
ENSG00000176752	0	0	0	0
ENSG00000176753	0	0	0	0

ENSG00000176754	0	0	0	0
ENSG00000176761	0	0	0	0
ENSG00000176769	0	0	0	1
ENSG00000176771	0	0	0	0
ENSG00000176774	0	0	0	0
ENSG00000176782	0	0	0	0
ENSG00000176783	25825	111219	135385	117502
ENSG00000176787	0	0	0	0
ENSG00000176788	34	16	24	8
ENSG00000176797	0	0	0	0
ENSG00000176798	0	0	0	0
ENSG00000176809	6	101	68	75
ENSG00000176812	0	0	0	0
ENSG00000176824	1	48	7	4
ENSG00000176826	0	0	0	0
ENSG00000176834	98	0	3	5
ENSG00000176840	0	0	0	0
ENSG00000176842	0	0	0	0
ENSG00000176845	2	0	0	0
ENSG00000176853	19	509	338	551
ENSG00000176855	0	0	0	0
ENSG00000176857	0	0	0	0
ENSG00000176868	0	0	0	0
ENSG00000176871	131	1614	660	2326
ENSG00000176879	0	0	0	0
ENSG00000176882	0	0	0	0
ENSG00000176884	0	0	0	0
ENSG00000176887	0	0	0	0
ENSG00000176890	3	145	157	305
ENSG00000176893	0	0	0	0
ENSG00000176894	2	0	0	2
ENSG00000176895	0	0	0	0
ENSG00000176896	4	434	262	474
ENSG00000176900	0	0	0	0
ENSG00000176903	637	3723	3263	1513
ENSG00000176904	0	0	0	0
ENSG00000176907	3	8	0	3
ENSG00000176909	3	0	0	0
ENSG00000176912	1	0	6	19
ENSG00000176915	60	319	306	291
ENSG00000176919	0	0	0	0
ENSG00000176920	0	0	0	0
ENSG00000176922	0	0	0	2
ENSG00000176923	0	8	25	0
ENSG00000176925	0	0	0	0
ENSG00000176927	27	154	100	181

ENSG00000176928	0	16	20	33
ENSG00000176933	0	0	0	0
ENSG00000176937	0	0	0	0
ENSG00000176945	0	0	0	0
ENSG00000176946	9	48	11	26
ENSG00000176951	0	0	0	0
ENSG00000176953	144	236	231	163
ENSG00000176956	0	0	0	0
ENSG00000176970	0	0	0	0
ENSG00000176971	0	0	0	0
ENSG00000176973	169	105	19	50
ENSG00000176974	2	0	2	0
ENSG00000176978	13	65	25	54
ENSG00000176979	0	0	0	0
ENSG00000176984	0	0	0	0
ENSG00000176986	24	50	54	58
ENSG00000176988	0	0	4	14
ENSG00000176994	3	178	171	117
ENSG00000176998	0	0	0	0
ENSG00000177000	149	219	87	124
ENSG00000177023	0	0	0	0
ENSG00000177025	0	0	11	14
ENSG00000177030	21	6	19	50
ENSG00000177034	2	4	19	21
ENSG00000177042	7	0	12	20
ENSG00000177045	0	0	1	6
ENSG00000177047	4	0	2	0
ENSG00000177051	6	0	6	2
ENSG00000177054	3	44	45	58
ENSG00000177058	3	1522	1122	1568
ENSG00000177076	27	2277	2301	1076
ENSG00000177082	5	0	10	1
ENSG00000177084	2	20	17	44
ENSG00000177096	22	80	50	53
ENSG00000177098	0	0	0	0
ENSG00000177103	0	0	0	0
ENSG00000177105	576	46	124	85
ENSG00000177106	2	0	0	0
ENSG00000177108	0	0	0	0
ENSG00000177112	3	47	9	14
ENSG00000177119	2812	49620	81306	58289
ENSG00000177125	133	2464	2998	2159
ENSG00000177133	0	0	0	0
ENSG00000177138	0	22	0	0
ENSG00000177143	0	0	0	0
ENSG00000177144	0	0	0	1

ENSG00000177150	5	374	277	427
ENSG00000177151	0	0	0	0
ENSG00000177156	4639	6660	4323	4894
ENSG00000177169	22	13	18	9
ENSG00000177173	0	0	8	7
ENSG00000177174	0	0	0	0
ENSG00000177181	0	55	11	8
ENSG00000177182	0	0	1	0
ENSG00000177186	0	0	0	0
ENSG00000177189	105	2532	2498	3262
ENSG00000177191	0	0	0	0
ENSG00000177192	0	0	0	1
ENSG00000177197	0	1	0	0
ENSG00000177200	1249	29897	36430	35707
ENSG00000177201	0	0	0	0
ENSG00000177202	0	0	0	0
ENSG00000177212	1	0	0	0
ENSG00000177225	2	0	0	6
ENSG00000177233	0	0	0	0
ENSG00000177234	0	0	0	0
ENSG00000177236	0	0	0	1
ENSG00000177238	0	0	0	0
ENSG00000177239	2	26	3	15
ENSG00000177243	0	0	0	0
ENSG00000177257	0	0	0	0
ENSG00000177261	0	0	0	0
ENSG00000177272	262	1498	2844	1094
ENSG00000177275	0	0	0	0
ENSG00000177283	0	0	0	0
ENSG00000177291	0	0	0	0
ENSG00000177294	0	0	0	0
ENSG00000177300	0	0	0	0
ENSG00000177301	16	15	10	33
ENSG00000177302	17	173	98	83
ENSG00000177303	0	0	0	3
ENSG00000177306	0	0	0	0
ENSG00000177311	221	1251	1763	1117
ENSG00000177324	4697	28346	47372	34076
ENSG00000177335	0	0	0	0
ENSG00000177337	90	90	192	70
ENSG00000177338	0	0	0	0
ENSG00000177340	1	0	0	0
ENSG00000177350	0	0	0	0
ENSG00000177352	1	2	2	1
ENSG00000177354	0	0	0	0
ENSG00000177359	3	777	628	39

ENSG00000177363	0	0	0	0
ENSG00000177369	0	0	0	0
ENSG00000177370	101	301	132	134
ENSG00000177374	0	0	0	0
ENSG00000177380	1	11	1	3
ENSG00000177383	7	0	3	0
ENSG00000177398	0	0	0	0
ENSG00000177400	0	0	0	0
ENSG00000177406	141	343	241	324
ENSG00000177409	33	78	30	35
ENSG00000177410	4322	9360	4026	5377
ENSG00000177414	0	0	0	0
ENSG00000177418	0	0	0	0
ENSG00000177425	134	1276	1832	1569
ENSG00000177426	4	0	1	0
ENSG00000177427	12	38	6	1
ENSG00000177432	8	93	95	49
ENSG00000177447	0	0	0	11
ENSG00000177452	0	0	0	0
ENSG00000177453	0	0	0	0
ENSG00000177455	0	0	0	0
ENSG00000177459	0	0	0	0
ENSG00000177462	2	0	1	1
ENSG00000177463	13	719	621	939
ENSG00000177464	0	0	0	0
ENSG00000177465	2	0	0	0
ENSG00000177468	0	0	0	0
ENSG00000177469	52	53	10	16
ENSG00000177476	0	0	0	0
ENSG00000177479	82	449	341	541
ENSG00000177483	1	30	12	12
ENSG00000177485	12	122	264	259
ENSG00000177489	0	0	0	0
ENSG00000177494	1	0	0	0
ENSG00000177504	0	0	0	0
ENSG00000177508	374	78	36	66
ENSG00000177511	0	0	0	0
ENSG00000177519	0	0	0	0
ENSG00000177535	0	0	0	0
ENSG00000177542	3	0	0	0
ENSG00000177548	0	0	1	3
ENSG00000177551	0	0	0	0
ENSG00000177553	0	0	0	0
ENSG00000177556	382	53	44	128
ENSG00000177558	4	0	6	4
ENSG00000177565	888	9968	13740	7700

ENSG00000177570	0	9	8	6
ENSG00000177575	5	28	19	12
ENSG00000177576	4	24	25	17
ENSG00000177586	0	0	0	0
ENSG00000177590	0	0	0	0
ENSG00000177595	0	13	2	0
ENSG00000177596	0	0	0	0
ENSG00000177599	0	1	0	5
ENSG00000177600	2977	430	111	489
ENSG00000177602	0	0	0	1
ENSG00000177606	0	0	2	2
ENSG00000177613	7	36	7	15
ENSG00000177614	0	0	0	0
ENSG00000177627	0	0	1	0
ENSG00000177628	14	1	10	29
ENSG00000177640	0	0	0	1
ENSG00000177646	7	27	13	40
ENSG00000177663	150	471	262	396
ENSG00000177666	12	9	14	46
ENSG00000177669	0	0	0	1
ENSG00000177673	0	0	0	0
ENSG00000177674	224	60	106	103
ENSG00000177675	2	255	88	218
ENSG00000177679	0	0	0	0
ENSG00000177683	24	49	202	276
ENSG00000177684	0	0	0	0
ENSG00000177685	1	0	0	1
ENSG00000177688	0	14	11	3
ENSG00000177689	0	0	0	0
ENSG00000177692	2	13	2	10
ENSG00000177693	0	0	0	0
ENSG00000177694	2	1689	1412	1956
ENSG00000177697	688	836	1648	1864
ENSG00000177699	0	0	0	0
ENSG00000177700	225	15	63	66
ENSG00000177706	2	0	0	0
ENSG00000177707	0	0	2	4
ENSG00000177710	0	0	0	0
ENSG00000177721	14	17	3	4
ENSG00000177725	0	0	0	0
ENSG00000177728	5	106	8	19
ENSG00000177731	297	1243	607	1206
ENSG00000177732	40	71	15	30
ENSG00000177733	79	217	364	333
ENSG00000177736	0	0	0	0
ENSG00000177738	25	38	63	58

ENSG00000177752	0	0	6	5
ENSG00000177757	0	0	0	0
ENSG00000177764	0	0	0	0
ENSG00000177770	0	0	0	0
ENSG00000177776	0	0	0	0
ENSG00000177788	0	0	0	0
ENSG00000177791	0	0	0	0
ENSG00000177800	0	0	0	0
ENSG00000177803	0	0	0	0
ENSG00000177807	1	0	0	0
ENSG00000177820	0	0	0	0
ENSG00000177822	0	0	0	0
ENSG00000177830	6	0	4	4
ENSG00000177839	0	0	0	0
ENSG00000177842	1	20	1	2
ENSG00000177853	14	239	241	300
ENSG00000177854	7	15	6	3
ENSG00000177855	0	0	1	0
ENSG00000177868	678	564	1011	541
ENSG00000177873	2	34	6	4
ENSG00000177875	0	21	0	0
ENSG00000177879	146	1023	1089	1429
ENSG00000177885	364	1361	1087	1482
ENSG00000177888	0	327	338	417
ENSG00000177889	45	168	115	176
ENSG00000177910	0	0	0	0
ENSG00000177917	102	657	569	407
ENSG00000177932	0	0	10	7
ENSG00000177938	0	0	0	0
ENSG00000177943	0	0	0	0
ENSG00000177946	2	0	0	3
ENSG00000177947	18	1	4	81
ENSG00000177951	109	149	63	92
ENSG00000177954	6931	1512	829	3074
ENSG00000177963	127	303	113	266
ENSG00000177971	18	9	1	5
ENSG00000177981	1462	2637	2328	1747
ENSG00000177984	0	0	0	0
ENSG00000177989	0	0	0	0
ENSG00000177990	5	2191	1592	2433
ENSG00000177992	0	0	0	0
ENSG00000177993	15	0	12	27
ENSG00000177994	1	0	0	0
ENSG00000178015	0	0	0	0
ENSG00000178021	0	0	0	0
ENSG00000178026	1	0	0	0

ENSG00000178028	5	0	1	1
ENSG00000178031	0	0	0	0
ENSG00000178033	78	1496	1890	1016
ENSG00000178035	29	0	6	3
ENSG00000178038	0	0	0	0
ENSG00000178053	3	0	2	0
ENSG00000178055	0	0	0	0
ENSG00000178057	2330	1359	978	1703
ENSG00000178074	30	129	395	373
ENSG00000178075	0	22	0	0
ENSG00000178078	5	0	0	0
ENSG00000178081	0	14	3	0
ENSG00000178082	0	0	0	0
ENSG00000178084	0	0	0	2
ENSG00000178093	0	0	0	0
ENSG00000178096	2	0	5	13
ENSG00000178104	45	184	183	319
ENSG00000178105	34	312	460	541
ENSG00000178107	0	0	0	0
ENSG00000178110	1	0	3	14
ENSG00000178115	0	0	5	15
ENSG00000178125	0	0	0	0
ENSG00000178127	26	2	5	3
ENSG00000178130	0	0	0	0
ENSG00000178146	0	0	9	4
ENSG00000178149	0	0	5	5
ENSG00000178150	1	0	0	2
ENSG00000178162	16	85	46	16
ENSG00000178163	41	562	454	439
ENSG00000178171	0	0	2	1
ENSG00000178172	0	0	0	0
ENSG00000178175	3	0	2	0
ENSG00000178177	13	698	983	1219
ENSG00000178184	30	256	125	96
ENSG00000178187	0	0	0	0
ENSG00000178188	3	0	0	0
ENSG00000178193	0	0	0	0
ENSG00000178199	0	0	0	0
ENSG00000178201	0	0	8	5
ENSG00000178202	3	0	9	6
ENSG00000178206	0	0	0	0
ENSG00000178209	2	1	4	11
ENSG00000178217	0	0	0	0
ENSG00000178222	0	0	0	0
ENSG00000178226	6	0	0	1
ENSG00000178229	1	0	2	1

ENSG00000178233	0	0	0	0
ENSG00000178234	6	20	32	2
ENSG00000178235	0	0	0	0
ENSG00000178243	0	0	0	0
ENSG00000178248	0	0	0	0
ENSG00000178252	5	22	0	5
ENSG00000178257	0	0	0	0
ENSG00000178279	0	0	0	0
ENSG00000178287	0	0	0	0
ENSG00000178295	0	27	56	87
ENSG00000178297	0	0	1	0
ENSG00000178301	0	0	7	12
ENSG00000178307	73	172	127	145
ENSG00000178338	1	34	33	23
ENSG00000178342	0	0	0	0
ENSG00000178343	0	0	0	0
ENSG00000178358	0	0	0	0
ENSG00000178363	0	0	0	0
ENSG00000178372	0	0	0	0
ENSG00000178381	1128	1199	997	2245
ENSG00000178385	3	85	50	41
ENSG00000178386	0	0	0	0
ENSG00000178394	0	0	0	0
ENSG00000178395	0	0	0	0
ENSG00000178397	6	1	32	35
ENSG00000178401	0	0	0	0
ENSG00000178403	0	0	0	0
ENSG00000178404	0	0	0	0
ENSG00000178409	0	0	3	0
ENSG00000178412	0	0	0	0
ENSG00000178425	48	133	160	113
ENSG00000178429	1	2	0	0
ENSG00000178430	0	0	0	0
ENSG00000178440	0	1	9	28
ENSG00000178445	3	0	13	9
ENSG00000178449	528	480	107	166
ENSG00000178457	0	0	0	0
ENSG00000178458	14	34	25	8
ENSG00000178460	0	132	187	77
ENSG00000178462	3	85	64	110
ENSG00000178464	3	0	0	0
ENSG00000178467	1	0	7	8
ENSG00000178473	0	0	0	0
ENSG00000178498	3	0	0	0
ENSG00000178502	0	0	4	2
ENSG00000178503	0	0	0	0

ENSG00000178522	3	0	7	0
ENSG00000178531	0	0	0	0
ENSG00000178537	25	143	66	140
ENSG00000178538	2	11	0	0
ENSG00000178550	0	0	0	0
ENSG00000178556	0	0	0	0
ENSG00000178562	40	216	288	167
ENSG00000178567	23	174	88	120
ENSG00000178568	0	0	0	0
ENSG00000178573	25	0	7	0
ENSG00000178585	102	779	380	702
ENSG00000178586	0	0	0	0
ENSG00000178591	0	0	0	0
ENSG00000178596	0	0	0	0
ENSG00000178597	0	0	0	0
ENSG00000178602	0	0	0	0
ENSG00000178605	11	0	0	1
ENSG00000178607	1	626	363	559
ENSG00000178623	0	0	0	0
ENSG00000178631	0	0	0	0
ENSG00000178636	0	0	0	4
ENSG00000178642	0	0	0	0
ENSG00000178645	0	0	0	0
ENSG00000178654	0	0	0	0
ENSG00000178660	0	0	0	0
ENSG00000178662	1	0	0	0
ENSG00000178665	1	31	1	18
ENSG00000178685	0	0	0	0
ENSG00000178690	0	0	0	0
ENSG00000178691	115	3636	3710	5890
ENSG00000178694	3	34	37	12
ENSG00000178695	61	15	36	12
ENSG00000178700	3	0	1	3
ENSG00000178715	1	0	0	0
ENSG00000178718	3	0	0	0
ENSG00000178719	6	0	1	3
ENSG00000178722	0	0	0	0
ENSG00000178723	0	0	0	0
ENSG00000178726	5	6	20	6
ENSG00000178732	81	803	586	1045
ENSG00000178734	0	0	1	0
ENSG00000178741	57	233	303	482
ENSG00000178750	0	0	0	0
ENSG00000178752	3	0	16	10
ENSG00000178761	6	82	61	73
ENSG00000178762	1	0	0	0

ENSG00000178764	7	43	18	13
ENSG00000178772	0	0	0	0
ENSG00000178773	0	0	0	0
ENSG00000178776	0	0	0	0
ENSG00000178789	0	7	0	0
ENSG00000178795	2	9	0	1
ENSG00000178796	0	0	0	0
ENSG00000178802	12	0	0	3
ENSG00000178803	0	0	3	9
ENSG00000178804	0	0	0	0
ENSG00000178809	2	0	0	1
ENSG00000178814	0	7	7	0
ENSG00000178821	0	0	0	0
ENSG00000178826	0	0	0	0
ENSG00000178828	0	0	0	0
ENSG00000178836	0	0	0	0
ENSG00000178852	462	51503	63183	99603
ENSG00000178860	9	65	14	10
ENSG00000178863	0	0	0	0
ENSG00000178878	1	0	0	5
ENSG00000178882	0	0	0	0
ENSG00000178894	0	0	0	0
ENSG00000178896	95	168	81	56
ENSG00000178904	35	588	721	531
ENSG00000178913	265	777	751	487
ENSG00000178917	0	0	1	1
ENSG00000178919	0	0	0	0
ENSG00000178921	2	0	0	2
ENSG00000178922	1	402	114	244
ENSG00000178927	60	331	90	152
ENSG00000178928	0	0	0	0
ENSG00000178934	0	0	0	0
ENSG00000178935	5	9	1	5
ENSG00000178947	0	0	0	0
ENSG00000178950	10	151	98	143
ENSG00000178951	78	24	28	45
ENSG00000178952	30	14	9	23
ENSG00000178965	0	0	0	3
ENSG00000178966	13	111	151	92
ENSG00000178971	1	0	1	13
ENSG00000178972	1	31	0	0
ENSG00000178974	8	186	129	153
ENSG00000178977	21	0	0	0
ENSG00000178980	1597	289	131	362
ENSG00000178982	265	78	30	117
ENSG00000178988	22	18	25	30

ENSG00000178996	33	179	195	202
ENSG00000178997	0	0	0	0
ENSG00000178999	1	0	7	2
ENSG00000179002	0	0	0	0
ENSG00000179008	0	0	0	2
ENSG00000179010	192	759	176	301
ENSG00000179021	26	416	381	259
ENSG00000179023	0	0	0	0
ENSG00000179028	0	0	0	0
ENSG00000179029	85	201	103	279
ENSG00000179031	0	0	0	0
ENSG00000179038	0	0	0	0
ENSG00000179041	6	0	0	0
ENSG00000179044	0	0	0	0
ENSG00000179046	0	0	0	0
ENSG00000179051	29	383	256	519
ENSG00000179055	0	0	0	0
ENSG00000179057	6	0	0	0
ENSG00000179058	0	0	0	0
ENSG00000179059	0	0	0	0
ENSG00000179061	0	0	0	0
ENSG00000179066	0	0	0	0
ENSG00000179071	0	0	0	0
ENSG00000179073	0	0	0	0
ENSG00000179082	0	0	0	0
ENSG00000179083	5	0	7	3
ENSG00000179085	126	93	20	98
ENSG00000179088	1	0	3	1
ENSG00000179091	9	0	12	6
ENSG00000179094	2	0	1	0
ENSG00000179097	0	0	24	10
ENSG00000179101	16	54	74	43
ENSG00000179104	0	36	11	17
ENSG00000179111	0	0	0	0
ENSG00000179115	19	1603	475	1351
ENSG00000179119	18	76	88	63
ENSG00000179131	0	0	0	0
ENSG00000179133	0	0	0	0
ENSG00000179134	51	30	20	31
ENSG00000179136	0	0	0	0
ENSG00000179141	0	0	0	0
ENSG00000179142	0	0	0	0
ENSG00000179144	390	0	2	5
ENSG00000179148	0	0	0	0
ENSG00000179151	5	88	83	120
ENSG00000179152	22	510	463	724

ENSG00000179157	0	0	0	0
ENSG00000179163	3	0	7	10
ENSG00000179165	383	26	13	11
ENSG00000179168	0	0	0	0
ENSG00000179170	0	0	0	0
ENSG00000179172	0	0	0	0
ENSG00000179178	0	0	0	0
ENSG00000179195	589	4217	4976	4515
ENSG00000179213	0	0	0	0
ENSG00000179218	31	475	216	653
ENSG00000179219	0	0	0	0
ENSG00000179222	20	32	22	27
ENSG00000179240	1	0	1	0
ENSG00000179241	29	291	126	228
ENSG00000179242	0	0	0	0
ENSG00000179253	0	0	0	0
ENSG00000179256	0	0	0	0
ENSG00000179262	90	0	7	5
ENSG00000179270	0	0	0	0
ENSG00000179271	5	0	3	0
ENSG00000179277	1	21	11	11
ENSG00000179284	0	0	0	0
ENSG00000179292	0	0	0	0
ENSG00000179294	1	0	0	0
ENSG00000179295	109	2638	3463	4424
ENSG00000179296	0	0	5	0
ENSG00000179299	0	0	2	1
ENSG00000179300	0	0	0	1
ENSG00000179304	0	0	0	0
ENSG00000179314	2	0	0	0
ENSG00000179331	0	0	0	0
ENSG00000179335	48	72	33	41
ENSG00000179342	0	0	0	0
ENSG00000179344	1	16	3	0
ENSG00000179348	351	530	338	218
ENSG00000179361	86	598	293	276
ENSG00000179362	0	0	0	0
ENSG00000179363	3	0	4	3
ENSG00000179364	37	130	55	252
ENSG00000179381	0	0	0	0
ENSG00000179387	5	140	334	406
ENSG00000179388	0	0	0	1
ENSG00000179397	0	39	43	48
ENSG00000179399	8	386	185	372
ENSG00000179403	0	0	0	0
ENSG00000179406	0	15	14	28

ENSG00000179407	0	0	0	0
ENSG00000179409	2	0	0	1
ENSG00000179412	0	0	0	0
ENSG00000179420	0	0	0	0
ENSG00000179428	0	0	0	0
ENSG00000179431	0	0	0	0
ENSG00000179443	0	0	0	0
ENSG00000179447	0	0	0	0
ENSG00000179449	0	0	0	0
ENSG00000179452	0	0	0	0
ENSG00000179454	51	634	664	549
ENSG00000179455	0	22	1	2
ENSG00000179456	23	174	151	98
ENSG00000179460	0	0	0	0
ENSG00000179467	0	0	0	0
ENSG00000179468	0	0	0	0
ENSG00000179476	1	63	17	16
ENSG00000179477	0	0	0	1
ENSG00000179520	0	0	1	1
ENSG00000179523	22	7	41	24
ENSG00000179526	158	394	135	386
ENSG00000179528	0	0	3	3
ENSG00000179532	0	0	5	11
ENSG00000179542	0	0	0	0
ENSG00000179546	0	0	0	0
ENSG00000179562	8	56	61	20
ENSG00000179564	0	0	0	0
ENSG00000179571	0	0	0	0
ENSG00000179577	0	0	0	0
ENSG00000179580	0	0	0	0
ENSG00000179583	5	0	1	1
ENSG00000179588	2	0	35	36
ENSG00000179593	0	0	0	0
ENSG00000179598	2	0	0	3
ENSG00000179600	0	0	0	0
ENSG00000179603	0	0	6	3
ENSG00000179604	0	13	3	2
ENSG00000179611	0	0	0	5
ENSG00000179615	0	0	0	0
ENSG00000179626	0	0	0	0
ENSG00000179627	0	0	0	1
ENSG00000179630	11	16	17	14
ENSG00000179632	123	1133	720	1286
ENSG00000179636	0	0	0	0
ENSG00000179639	11	0	4	21
ENSG00000179673	0	0	0	0

ENSG00000179674	0	0	0	0
ENSG00000179676	0	0	0	3
ENSG00000179695	0	0	0	0
ENSG00000179698	2	0	0	0
ENSG00000179709	0	0	0	0
ENSG00000179715	252	709	660	951
ENSG00000179743	75	73	40	33
ENSG00000179750	0	0	1	9
ENSG00000179751	0	0	0	0
ENSG00000179755	0	0	0	0
ENSG00000179761	46	83	32	68
ENSG00000179766	2	0	2	2
ENSG00000179772	0	0	0	0
ENSG00000179774	0	0	0	0
ENSG00000179776	0	0	2	5
ENSG00000179796	0	0	0	0
ENSG00000179799	0	0	0	0
ENSG00000179813	0	0	0	0
ENSG00000179817	0	0	0	0
ENSG00000179818	12	516	312	446
ENSG00000179820	17	1	18	25
ENSG00000179826	0	0	0	2
ENSG00000179832	10	6	22	23
ENSG00000179833	142	190	433	265
ENSG00000179837	10	7	7	26
ENSG00000179840	0	0	0	0
ENSG00000179841	0	0	1	0
ENSG00000179846	0	0	0	0
ENSG00000179855	188	127	112	237
ENSG00000179859	15	0	0	0
ENSG00000179862	0	0	0	0
ENSG00000179869	1	0	4	16
ENSG00000179873	0	0	0	0
ENSG00000179886	0	0	0	0
ENSG00000179889	31	400	696	889
ENSG00000179899	0	0	9	5
ENSG00000179902	1	0	0	0
ENSG00000179909	7	182	68	59
ENSG00000179912	69	622	619	793
ENSG00000179913	0	0	0	0
ENSG00000179914	3	0	0	0
ENSG00000179915	0	0	0	0
ENSG00000179918	73	81	46	91
ENSG00000179919	0	0	0	0
ENSG00000179921	5	0	0	0
ENSG00000179922	2	0	0	0

ENSG00000179930	0	0	0	0
ENSG00000179933	313	658	489	1109
ENSG00000179934	0	0	0	0
ENSG00000179935	0	0	0	0
ENSG00000179938	0	0	0	0
ENSG00000179941	0	102	99	92
ENSG00000179943	3	0	1	2
ENSG00000179950	29	0	2	3
ENSG00000179954	0	0	0	0
ENSG00000179958	24	0	1	0
ENSG00000179965	0	0	0	3
ENSG00000179967	0	0	4	5
ENSG00000179978	0	0	0	0
ENSG00000179979	1	0	1	10
ENSG00000179981	17	31	18	33
ENSG00000179988	6	32	8	14
ENSG00000179994	0	0	0	0
ENSG00000179997	0	0	0	0
ENSG00000180008	11	284	308	254
ENSG00000180011	32	90	132	135
ENSG00000180015	8	56	14	210
ENSG00000180016	0	0	0	0
ENSG00000180019	0	0	0	0
ENSG00000180035	1	0	5	0
ENSG00000180042	0	0	0	0
ENSG00000180043	0	0	0	0
ENSG00000180044	0	68	29	51
ENSG00000180053	0	0	0	0
ENSG00000180061	0	0	0	0
ENSG00000180066	0	0	0	0
ENSG00000180068	0	0	0	0
ENSG00000180071	0	0	0	0
ENSG00000180081	0	0	0	0
ENSG00000180083	0	0	0	0
ENSG00000180089	5	0	1	4
ENSG00000180090	0	0	0	0
ENSG00000180096	34	35	24	74
ENSG00000180098	50	146	135	196
ENSG00000180104	50	67	45	116
ENSG00000180105	0	0	0	0
ENSG00000180113	3	0	2	4
ENSG00000180116	0	0	2	1
ENSG00000180138	0	0	0	0
ENSG00000180139	0	0	0	0
ENSG00000180150	0	0	0	0
ENSG00000180152	0	0	0	14

ENSG00000180155	0	0	0	0
ENSG00000180172	0	0	0	0
ENSG00000180176	0	0	0	0
ENSG00000180178	0	0	0	0
ENSG00000180182	4	264	403	552
ENSG00000180185	13	0	5	9
ENSG00000180189	0	0	0	0
ENSG00000180190	368	4330	4574	4182
ENSG00000180198	19	0	3	18
ENSG00000180205	0	0	0	0
ENSG00000180209	35	0	0	6
ENSG00000180210	0	0	0	0
ENSG00000180211	0	0	0	0
ENSG00000180219	0	0	0	0
ENSG00000180221	0	0	0	0
ENSG00000180228	15	65	46	41
ENSG00000180229	6	94	62	111
ENSG00000180230	0	0	0	0
ENSG00000180233	112	2116	2633	2710
ENSG00000180245	0	0	4	2
ENSG00000180251	6	0	3	2
ENSG00000180257	1	73	40	101
ENSG00000180259	0	0	0	0
ENSG00000180263	14	137	34	52
ENSG00000180264	0	0	0	0
ENSG00000180269	0	0	0	0
ENSG00000180279	0	0	0	0
ENSG00000180284	5	766	570	1364
ENSG00000180287	0	0	0	0
ENSG00000180304	69	38	41	48
ENSG00000180305	0	0	0	0
ENSG00000180316	0	0	0	0
ENSG00000180318	0	0	0	0
ENSG00000180329	7	35	15	6
ENSG00000180332	0	0	0	0
ENSG00000180336	0	0	0	0
ENSG00000180340	0	0	0	0
ENSG00000180344	0	0	0	0
ENSG00000180346	4	0	15	5
ENSG00000180347	0	0	0	0
ENSG00000180353	222	298	251	464
ENSG00000180354	11084	40458	48087	44050
ENSG00000180357	6	0	0	6
ENSG00000180370	140	2360	2389	4445
ENSG00000180376	93	4667	4023	6594
ENSG00000180383	0	0	0	0

ENSG00000180385	9	47	26	21
ENSG00000180386	0	0	0	0
ENSG00000180389	6	0	2	1
ENSG00000180398	199	632	1503	1978
ENSG00000180409	0	0	0	0
ENSG00000180422	0	0	0	0
ENSG00000180423	1	15	2	4
ENSG00000180424	0	0	2	0
ENSG00000180425	10	11	38	65
ENSG00000180432	0	0	0	0
ENSG00000180433	0	0	0	0
ENSG00000180437	0	0	0	0
ENSG00000180438	0	0	0	0
ENSG00000180440	0	0	1	0
ENSG00000180447	0	0	0	0
ENSG00000180448	352	2654	1494	2179
ENSG00000180458	0	0	0	0
ENSG00000180475	0	0	0	0
ENSG00000180479	0	0	13	11
ENSG00000180481	1	0	2	2
ENSG00000180483	0	0	0	0
ENSG00000180488	13	2878	2091	2848
ENSG00000180509	2	12	0	0
ENSG00000180525	0	0	0	0
ENSG00000180530	131	14525	15806	38266
ENSG00000180532	0	0	0	0
ENSG00000180535	0	0	0	0
ENSG00000180537	0	0	0	0
ENSG00000180539	0	0	0	0
ENSG00000180543	1	5	3	0
ENSG00000180549	0	0	0	0
ENSG00000180573	41486	203937	256633	167646
ENSG00000180574	0	0	0	1
ENSG00000180581	0	25	11	13
ENSG00000180592	0	0	0	0
ENSG00000180596	892	28272	18403	16679
ENSG00000180610	0	0	0	0
ENSG00000180611	1	0	0	0
ENSG00000180613	0	0	0	0
ENSG00000180616	0	0	0	0
ENSG00000180626	0	0	7	24
ENSG00000180628	2025	64351	80177	76296
ENSG00000180636	0	0	0	0
ENSG00000180638	0	0	0	0
ENSG00000180644	33	17	1	0
ENSG00000180658	0	0	0	0

ENSG00000180660	1	0	0	0
ENSG00000180662	0	0	0	0
ENSG00000180663	0	0	0	0
ENSG00000180667	51	869	1864	896
ENSG00000180672	10	11	5	6
ENSG00000180673	0	0	0	1
ENSG00000180694	275	4266	6813	4217
ENSG00000180697	0	0	0	0
ENSG00000180708	0	0	0	0
ENSG00000180712	23	12	21	60
ENSG00000180714	0	0	0	0
ENSG00000180720	0	0	0	0
ENSG00000180723	0	0	0	0
ENSG00000180725	0	0	0	0
ENSG00000180730	0	0	0	0
ENSG00000180739	4	0	0	2
ENSG00000180745	0	0	0	0
ENSG00000180747	10	78	176	95
ENSG00000180758	0	0	0	0
ENSG00000180764	0	0	1	0
ENSG00000180767	0	0	6	0
ENSG00000180769	1	0	2	0
ENSG00000180770	0	0	0	0
ENSG00000180771	1406	2266	2735	2098
ENSG00000180772	0	0	0	0
ENSG00000180773	8	162	73	48
ENSG00000180776	10	3465	2882	4232
ENSG00000180777	0	0	0	0
ENSG00000180785	0	0	0	0
ENSG00000180787	1	0	10	6
ENSG00000180801	0	0	0	0
ENSG00000180803	0	0	0	0
ENSG00000180806	0	0	0	0
ENSG00000180815	0	0	1	11
ENSG00000180817	46	76	69	118
ENSG00000180818	0	0	0	0
ENSG00000180822	6	0	9	31
ENSG00000180828	0	0	0	0
ENSG00000180834	0	0	3	2
ENSG00000180846	0	0	0	0
ENSG00000180855	0	1	8	33
ENSG00000180861	0	0	0	0
ENSG00000180867	0	25	19	33
ENSG00000180869	1	0	0	0
ENSG00000180871	18	18	2	2
ENSG00000180872	0	0	0	0

ENSG00000180875	0	0	0	0
ENSG00000180878	0	0	0	0
ENSG00000180879	4649	1648	843	1482
ENSG00000180881	2	364	346	536
ENSG00000180884	52	430	551	156
ENSG00000180891	5	0	0	0
ENSG00000180900	0	0	0	1
ENSG00000180901	84	193	87	299
ENSG00000180902	0	0	1	1
ENSG00000180909	0	0	0	0
ENSG00000180910	0	0	0	0
ENSG00000180913	0	0	1	0
ENSG00000180914	838	2120	2496	949
ENSG00000180917	7	98	88	61
ENSG00000180919	0	0	0	0
ENSG00000180921	1	0	0	0
ENSG00000180926	0	0	0	0
ENSG00000180929	0	0	0	0
ENSG00000180934	0	0	0	0
ENSG00000180938	0	8	10	16
ENSG00000180953	132	60	44	60
ENSG00000180957	74	2466	2875	5230
ENSG00000180964	120	1070	1141	1193
ENSG00000180974	1	0	0	0
ENSG00000180979	39	83	97	133
ENSG00000180987	0	0	0	0
ENSG00000180988	1	0	2	3
ENSG00000180992	30	13	13	19
ENSG00000180998	3	27	31	17
ENSG00000180999	0	0	1	0
ENSG00000181001	0	0	0	0
ENSG00000181004	7	53	61	15
ENSG00000181007	2	1	3	4
ENSG00000181009	0	0	0	0
ENSG00000181013	0	0	0	0
ENSG00000181016	269	1839	1557	1678
ENSG00000181017	0	0	0	0
ENSG00000181019	1	0	5	2
ENSG00000181023	0	0	0	0
ENSG00000181026	2	0	0	1
ENSG00000181027	35	15	6	0
ENSG00000181029	0	0	4	0
ENSG00000181031	17	58	50	37
ENSG00000181035	2	0	0	3
ENSG00000181036	7	0	1	0
ENSG00000181038	45	5	9	28

ENSG00000181039	9	2	1	7
ENSG00000181045	1	0	0	0
ENSG00000181061	1053	7350	8211	12317
ENSG00000181072	0	0	11	9
ENSG00000181074	0	0	0	0
ENSG00000181085	0	0	0	0
ENSG00000181090	10	254	149	375
ENSG00000181092	0	0	0	0
ENSG00000181097	0	0	0	0
ENSG00000181101	0	0	0	0
ENSG00000181104	3923	8385	10922	7781
ENSG00000181109	0	0	0	0
ENSG00000181123	0	0	0	0
ENSG00000181126	0	0	0	0
ENSG00000181135	1	10	11	11
ENSG00000181143	0	0	0	0
ENSG00000181163	327	1752	1722	2789
ENSG00000181171	0	0	4	0
ENSG00000181191	1	0	30	24
ENSG00000181192	25	378	325	517
ENSG00000181195	0	0	0	0
ENSG00000181201	0	0	0	0
ENSG00000181211	0	0	0	0
ENSG00000181214	0	0	0	0
ENSG00000181215	0	0	0	0
ENSG00000181218	0	0	7	15
ENSG00000181220	11	34	46	19
ENSG00000181222	71	575	321	410
ENSG00000181227	0	0	0	0
ENSG00000181234	1	0	0	0
ENSG00000181240	0	0	0	0
ENSG00000181260	0	0	0	0
ENSG00000181264	0	0	0	5
ENSG00000181273	0	0	0	0
ENSG00000181274	4	21	4	0
ENSG00000181282	0	0	0	0
ENSG00000181284	0	0	0	0
ENSG00000181291	0	0	0	0
ENSG00000181296	0	0	0	0
ENSG00000181315	0	0	2	8
ENSG00000181322	3	32	10	12
ENSG00000181323	0	0	0	0
ENSG00000181325	0	0	0	0
ENSG00000181333	28	54	16	11
ENSG00000181350	2	51	35	35
ENSG00000181355	0	0	0	0

ENSG00000181358	0	12	0	0
ENSG00000181359	0	0	0	0
ENSG00000181371	0	0	0	0
ENSG00000181374	0	0	0	0
ENSG00000181378	0	0	0	0
ENSG00000181381	21	174	273	269
ENSG00000181392	0	0	0	2
ENSG00000181395	0	0	0	0
ENSG00000181396	4	0	14	18
ENSG00000181404	3	12	8	7
ENSG00000181408	0	0	0	0
ENSG00000181409	13	169	89	58
ENSG00000181418	0	0	0	0
ENSG00000181433	0	0	0	0
ENSG00000181444	1	0	0	0
ENSG00000181449	0	0	0	0
ENSG00000181450	6	215	333	269
ENSG00000181458	382	1319	1536	1035
ENSG00000181464	0	0	0	0
ENSG00000181467	1293	6696	8129	5463
ENSG00000181472	31	151	190	88
ENSG00000181481	12	66	43	108
ENSG00000181495	0	0	0	0
ENSG00000181499	0	0	0	0
ENSG00000181511	0	0	0	0
ENSG00000181513	0	0	5	5
ENSG00000181514	0	0	0	0
ENSG00000181518	0	0	0	0
ENSG00000181523	0	0	0	0
ENSG00000181524	0	0	0	0
ENSG00000181541	0	0	0	0
ENSG00000181544	6	151	344	466
ENSG00000181552	0	0	0	0
ENSG00000181555	117	7168	6930	9596
ENSG00000181562	0	0	0	0
ENSG00000181577	3	0	1	8
ENSG00000181585	0	0	0	0
ENSG00000181588	1	0	0	0
ENSG00000181609	0	0	0	0
ENSG00000181610	13	12	18	24
ENSG00000181616	0	0	0	0
ENSG00000181617	0	0	0	0
ENSG00000181619	3	85	54	62
ENSG00000181625	0	0	0	0
ENSG00000181626	1	0	1	0
ENSG00000181631	2	0	4	0

ENSG00000181634	0	0	0	0
ENSG00000181638	2	0	0	0
ENSG00000181649	0	0	0	0
ENSG00000181652	0	0	0	0
ENSG00000181656	9	58	37	142
ENSG00000181666	8	85	30	47
ENSG00000181689	0	0	0	0
ENSG00000181690	53	215	407	260
ENSG00000181693	0	0	0	0
ENSG00000181698	0	0	0	0
ENSG00000181704	250	1477	1817	1758
ENSG00000181705	0	0	0	0
ENSG00000181718	0	0	0	0
ENSG00000181722	11	223	222	456
ENSG00000181733	0	0	0	0
ENSG00000181741	0	0	0	0
ENSG00000181744	127	1480	1753	909
ENSG00000181751	79	590	806	682
ENSG00000181752	0	0	0	0
ENSG00000181754	1	0	0	0
ENSG00000181761	0	0	0	0
ENSG00000181767	0	0	0	0
ENSG00000181773	1	0	0	0
ENSG00000181778	0	0	0	0
ENSG00000181780	0	0	0	0
ENSG00000181781	0	0	0	0
ENSG00000181784	0	0	0	0
ENSG00000181785	0	0	0	0
ENSG00000181786	0	0	0	0
ENSG00000181788	945	4818	3589	2652
ENSG00000181789	24	273	113	232
ENSG00000181790	0	0	0	0
ENSG00000181791	0	0	0	0
ENSG00000181798	0	0	8	0
ENSG00000181800	0	0	0	0
ENSG00000181803	0	0	0	0
ENSG00000181804	236	10876	8725	14253
ENSG00000181817	95	0	9	3
ENSG00000181819	0	0	0	0
ENSG00000181826	0	646	453	1141
ENSG00000181827	20	237	409	427
ENSG00000181830	4	12	0	0
ENSG00000181837	0	0	0	0
ENSG00000181847	13	0	0	0
ENSG00000181852	28	19	55	54
ENSG00000181856	0	0	0	0

ENSG00000181867	0	0	0	0
ENSG00000181873	1	0	2	0
ENSG00000181885	4	0	0	0
ENSG00000181894	7	20	8	11
ENSG00000181896	24	39	58	80
ENSG00000181903	0	0	0	0
ENSG00000181904	26	62	90	128
ENSG00000181908	0	0	0	0
ENSG00000181915	10	41	41	40
ENSG00000181924	24	0	0	0
ENSG00000181927	0	0	0	0
ENSG00000181929	37	188	140	210
ENSG00000181935	0	0	0	0
ENSG00000181938	1	0	0	1
ENSG00000181939	0	0	0	0
ENSG00000181943	0	0	0	0
ENSG00000181950	0	0	0	0
ENSG00000181958	0	0	0	0
ENSG00000181961	0	0	0	0
ENSG00000181963	0	0	0	0
ENSG00000181965	0	0	0	0
ENSG00000181977	0	0	0	0
ENSG00000181982	1	0	0	0
ENSG00000181984	0	0	0	0
ENSG00000181991	17	17	8	7
ENSG00000181995	11	30	17	12
ENSG00000181997	0	0	0	0
ENSG00000182000	0	0	0	0
ENSG00000182004	79	0	7	11
ENSG00000182010	0	11	49	71
ENSG00000182013	0	0	0	0
ENSG00000182021	7	0	4	3
ENSG00000182022	2	0	0	0
ENSG00000182035	0	0	0	0
ENSG00000182040	0	0	0	0
ENSG00000182048	87	292	546	349
ENSG00000182050	0	0	0	0
ENSG00000182053	0	0	0	0
ENSG00000182054	83	284	159	326
ENSG00000182057	0	18	9	27
ENSG00000182070	0	0	1	0
ENSG00000182077	0	0	0	5
ENSG00000182083	0	0	0	0
ENSG00000182087	4	112	18	27
ENSG00000182093	952	2825	6132	17652
ENSG00000182095	6	41	5	22

ENSG00000182103	0	0	0	0
ENSG00000182107	0	0	0	0
ENSG00000182108	0	68	30	19
ENSG00000182109	0	0	0	0
ENSG00000182111	0	0	0	0
ENSG00000182117	999	969	526	909
ENSG00000182118	6	58	70	45
ENSG00000182132	0	0	0	0
ENSG00000182134	6	158	100	129
ENSG00000182141	6	139	98	74
ENSG00000182149	192	1071	854	523
ENSG00000182150	18	1089	1068	1871
ENSG00000182154	10	0	4	18
ENSG00000182156	0	0	0	0
ENSG00000182158	24	47	64	118
ENSG00000182162	3	0	0	0
ENSG00000182165	113	150	129	443
ENSG00000182168	1	0	0	0
ENSG00000182170	0	0	0	0
ENSG00000182173	9	0	0	1
ENSG00000182175	0	0	0	0
ENSG00000182177	0	0	0	0
ENSG00000182179	1488	1405	816	1332
ENSG00000182180	42	222	96	102
ENSG00000182183	6	0	0	0
ENSG00000182185	2	22	46	17
ENSG00000182187	0	0	0	0
ENSG00000182195	11	0	4	0
ENSG00000182196	0	0	0	0
ENSG00000182197	4	63	65	43
ENSG00000182199	33	0	1	20
ENSG00000182208	241	1163	977	533
ENSG00000182217	0	0	0	2
ENSG00000182218	0	0	0	0
ENSG00000182220	540	2189	2952	3348
ENSG00000182223	0	0	0	1
ENSG00000182224	4	38	34	29
ENSG00000182230	0	0	3	1
ENSG00000182240	4	0	3	13
ENSG00000182242	0	0	0	0
ENSG00000182247	8	0	1	13
ENSG00000182253	252	1780	1160	2401
ENSG00000182255	0	0	0	3
ENSG00000182256	0	0	0	0
ENSG00000182257	0	0	0	0
ENSG00000182261	0	0	0	0

ENSG00000182263	0	0	4	11
ENSG00000182264	1	12	3	0
ENSG00000182271	0	0	0	0
ENSG00000182272	0	0	0	1
ENSG00000182287	1277	14965	18020	21555
ENSG00000182307	9	0	0	9
ENSG00000182308	0	0	0	0
ENSG00000182310	3	0	0	0
ENSG00000182315	0	0	0	0
ENSG00000182318	0	0	0	0
ENSG00000182319	0	0	0	0
ENSG00000182324	0	0	0	0
ENSG00000182325	0	0	3	0
ENSG00000182326	1	0	0	4
ENSG00000182327	0	0	0	0
ENSG00000182329	0	19	0	0
ENSG00000182330	0	0	0	0
ENSG00000182333	2	0	5	0
ENSG00000182334	0	0	0	0
ENSG00000182346	0	0	0	0
ENSG00000182347	0	0	0	0
ENSG00000182348	0	0	0	0
ENSG00000182351	0	0	0	0
ENSG00000182352	0	0	0	0
ENSG00000182356	0	0	0	0
ENSG00000182359	15	35	27	20
ENSG00000182362	10	0	13	7
ENSG00000182365	0	0	0	0
ENSG00000182366	0	0	0	0
ENSG00000182368	0	0	0	0
ENSG00000182372	229	632	527	831
ENSG00000182376	0	0	0	0
ENSG00000182378	26	111	1	1
ENSG00000182379	0	0	0	0
ENSG00000182383	0	1	0	0
ENSG00000182389	3	528	260	240
ENSG00000182393	3	0	0	0
ENSG00000182397	1	0	6	0
ENSG00000182400	93	2567	3032	2769
ENSG00000182404	0	0	0	0
ENSG00000182405	0	0	2	3
ENSG00000182415	0	0	0	0
ENSG00000182446	41	470	352	573
ENSG00000182447	0	0	1	0
ENSG00000182450	0	0	0	0
ENSG00000182459	0	0	0	0

ENSG00000182463	6	0	1	7
ENSG00000182472	3	0	6	0
ENSG00000182473	16	140	125	168
ENSG00000182477	0	0	0	0
ENSG00000182481	16	55	38	78
ENSG00000182484	7	148	35	233
ENSG00000182487	4	4	1	0
ENSG00000182489	0	0	1	2
ENSG00000182492	0	0	0	0
ENSG00000182500	1221	683	548	566
ENSG00000182502	0	0	3	0
ENSG00000182504	5	22	50	75
ENSG00000182508	0	0	1	0
ENSG00000182511	31	23	23	23
ENSG00000182512	253	328	159	195
ENSG00000182518	158	149	174	208
ENSG00000182521	1	0	0	0
ENSG00000182531	0	0	0	5
ENSG00000182533	0	0	0	4
ENSG00000182534	31	84	30	80
ENSG00000182541	0	0	5	13
ENSG00000182544	37	0	3	8
ENSG00000182545	0	0	0	0
ENSG00000182551	1002	6382	3903	5542
ENSG00000182552	39	269	221	235
ENSG00000182557	87	123	27	29
ENSG00000182565	0	0	1	0
ENSG00000182566	0	0	0	0
ENSG00000182568	135	759	1023	825
ENSG00000182572	0	0	6	5
ENSG00000182574	0	0	0	0
ENSG00000182575	0	0	3	8
ENSG00000182578	21	0	7	19
ENSG00000182580	0	0	0	0
ENSG00000182583	0	0	0	0
ENSG00000182584	0	0	1	0
ENSG00000182585	0	0	3	2
ENSG00000182586	0	0	0	0
ENSG00000182591	0	0	0	0
ENSG00000182600	0	0	0	0
ENSG00000182601	0	0	0	0
ENSG00000182606	10	70	76	114
ENSG00000182611	2	5	10	11
ENSG00000182612	0	0	0	0
ENSG00000182613	0	0	0	0
ENSG00000182621	3	46	37	38

ENSG00000182625	0	0	0	0
ENSG00000182628	165	1196	882	1986
ENSG00000182631	0	0	0	0
ENSG00000182632	0	0	0	2
ENSG00000182634	0	0	0	0
ENSG00000182636	0	0	5	18
ENSG00000182645	0	0	1	0
ENSG00000182646	0	0	0	0
ENSG00000182648	49	24	6	27
ENSG00000182652	0	0	0	0
ENSG00000182667	0	0	0	0
ENSG00000182670	225	4829	4965	4725
ENSG00000182674	0	0	2	4
ENSG00000182676	0	0	0	0
ENSG00000182685	0	0	0	0
ENSG00000182687	0	0	0	0
ENSG00000182698	0	0	0	0
ENSG00000182700	11	18	68	45
ENSG00000182704	0	0	0	0
ENSG00000182707	0	0	0	0
ENSG00000182712	0	0	0	0
ENSG00000182718	189	10	17	26
ENSG00000182722	0	0	0	0
ENSG00000182732	918	3512	3408	3333
ENSG00000182742	0	0	0	0
ENSG00000182747	38	182	184	333
ENSG00000182749	84	103	88	69
ENSG00000182752	1	37	37	41
ENSG00000182759	1	0	0	0
ENSG00000182768	2	0	6	8
ENSG00000182771	1	0	17	33
ENSG00000182774	0	0	0	0
ENSG00000182776	0	0	0	0
ENSG00000182782	0	0	0	0
ENSG00000182783	0	0	0	0
ENSG00000182791	0	0	0	0
ENSG00000182793	1	0	1	0
ENSG00000182795	68	484	244	398
ENSG00000182796	3	0	9	3
ENSG00000182798	0	0	0	0
ENSG00000182809	8	0	0	7
ENSG00000182810	2	0	1	0
ENSG00000182814	68	96	71	199
ENSG00000182816	0	0	0	0
ENSG00000182824	0	0	0	0
ENSG00000182827	22	57	130	171

ENSG00000182831	57	368	537	356
ENSG00000182836	0	0	0	0
ENSG00000182841	0	5	1	0
ENSG00000182851	3	0	0	0
ENSG00000182853	4	3	1	0
ENSG00000182854	0	0	0	0
ENSG00000182858	4	0	3	10
ENSG00000182866	52	0	0	0
ENSG00000182870	0	0	0	0
ENSG00000182871	0	0	0	1
ENSG00000182872	16	37	8	26
ENSG00000182873	0	0	0	0
ENSG00000182885	0	0	0	0
ENSG00000182888	0	0	0	0
ENSG00000182890	0	0	0	0
ENSG00000182896	1	0	0	0
ENSG00000182898	0	0	0	0
ENSG00000182899	3099	3643	2052	3124
ENSG00000182901	0	0	0	0
ENSG00000182902	0	0	0	0
ENSG00000182903	8	74	69	127
ENSG00000182909	0	0	1	0
ENSG00000182912	3	0	0	0
ENSG00000182916	0	0	0	0
ENSG00000182919	20	850	600	1026
ENSG00000182921	0	0	0	0
ENSG00000182923	48	484	372	422
ENSG00000182931	16	0	7	11
ENSG00000182934	191	1062	1107	1368
ENSG00000182938	0	0	0	0
ENSG00000182944	44	583	292	537
ENSG00000182950	0	0	0	0
ENSG00000182952	27	1362	528	950
ENSG00000182957	36	47	21	49
ENSG00000182963	1	15	0	0
ENSG00000182965	0	0	0	0
ENSG00000182968	0	0	0	0
ENSG00000182973	31	1354	1265	1482
ENSG00000182974	0	0	0	0
ENSG00000182979	4	21	15	56
ENSG00000182983	1	0	3	7
ENSG00000182985	1	15	0	0
ENSG00000182986	5	51	46	58
ENSG00000182993	0	0	0	0
ENSG00000183010	0	0	0	1
ENSG00000183011	658	157	110	309

ENSG00000183016	0	0	0	0
ENSG00000183018	0	0	1	0
ENSG00000183019	223	512	115	199
ENSG00000183020	12	0	11	12
ENSG00000183022	0	0	0	0
ENSG00000183023	7	0	1	0
ENSG00000183024	0	0	0	0
ENSG00000183032	0	0	3	0
ENSG00000183034	0	0	0	0
ENSG00000183035	0	0	0	0
ENSG00000183036	0	0	0	0
ENSG00000183038	0	0	0	0
ENSG00000183044	252	806	636	1797
ENSG00000183048	0	0	0	0
ENSG00000183049	42	0	15	40
ENSG00000183054	0	5	0	0
ENSG00000183055	0	1	1	0
ENSG00000183060	2	0	3	3
ENSG00000183066	3	0	2	0
ENSG00000183067	0	0	0	0
ENSG00000183072	0	0	0	0
ENSG00000183077	4	0	3	8
ENSG00000183086	0	0	0	0
ENSG00000183087	0	0	0	0
ENSG00000183090	25	0	0	2
ENSG00000183091	4	10	14	23
ENSG00000183092	0	0	0	0
ENSG00000183098	0	0	71	0
ENSG00000183103	0	0	0	3
ENSG00000183111	0	0	0	0
ENSG00000183114	0	0	0	0
ENSG00000183117	0	0	0	1
ENSG00000183122	0	0	0	0
ENSG00000183128	0	0	0	0
ENSG00000183130	0	0	0	0
ENSG00000183134	2	0	0	0
ENSG00000183137	31	1234	1121	2401
ENSG00000183145	1	0	0	0
ENSG00000183146	0	0	0	0
ENSG00000183148	0	0	0	0
ENSG00000183150	0	5	3	0
ENSG00000183153	0	0	0	0
ENSG00000183154	0	0	1	0
ENSG00000183155	187	284	173	118
ENSG00000183160	0	0	0	0
ENSG00000183161	0	0	8	4

ENSG00000183166	0	0	1	0
ENSG00000183169	0	0	0	0
ENSG00000183171	0	0	0	0
ENSG00000183172	451	226	177	408
ENSG00000183185	0	0	0	0
ENSG00000183186	0	0	0	0
ENSG00000183196	0	0	0	0
ENSG00000183199	0	1	0	0
ENSG00000183206	0	0	1	0
ENSG00000183207	28	46	34	29
ENSG00000183208	1	0	0	0
ENSG00000183214	0	0	0	0
ENSG00000183230	2	5	52	24
ENSG00000183239	0	0	0	0
ENSG00000183242	0	0	0	0
ENSG00000183246	0	0	0	0
ENSG00000183248	0	0	0	0
ENSG00000183249	0	0	0	0
ENSG00000183250	0	0	0	0
ENSG00000183251	0	0	4	1
ENSG00000183255	891	8663	6363	7647
ENSG00000183258	120	100	57	155
ENSG00000183260	0	0	0	0
ENSG00000183269	0	0	0	0
ENSG00000183273	1	0	0	0
ENSG00000183281	4	14	32	18
ENSG00000183283	3405	5591	9749	9661
ENSG00000183287	0	0	0	0
ENSG00000183291	182	821	849	1468
ENSG00000183292	3	0	0	0
ENSG00000183298	0	0	0	0
ENSG00000183303	0	0	0	0
ENSG00000183304	0	0	0	0
ENSG00000183305	0	0	0	0
ENSG00000183307	0	0	0	0
ENSG00000183308	0	37	25	12
ENSG00000183309	0	20	2	14
ENSG00000183310	0	0	0	0
ENSG00000183311	0	0	0	0
ENSG00000183313	0	0	0	0
ENSG00000183317	0	0	0	0
ENSG00000183318	0	0	0	2
ENSG00000183323	10	179	159	401
ENSG00000183324	0	4	0	0
ENSG00000183336	0	0	0	0
ENSG00000183337	108	335	271	105

ENSG00000183340	28	73	86	51
ENSG00000183346	0	0	0	6
ENSG00000183347	0	0	2	2
ENSG00000183354	193	1067	1451	1759
ENSG00000183378	0	0	0	0
ENSG00000183379	0	0	0	0
ENSG00000183385	0	0	0	0
ENSG00000183386	345	59	66	11
ENSG00000183389	0	0	0	0
ENSG00000183395	1	0	4	2
ENSG00000183396	0	0	0	0
ENSG00000183397	0	0	0	0
ENSG00000183401	13	71	18	25
ENSG00000183405	4	0	0	0
ENSG00000183421	0	0	0	0
ENSG00000183423	0	0	0	0
ENSG00000183426	0	0	6	6
ENSG00000183431	46	0	53	68
ENSG00000183432	0	0	0	0
ENSG00000183434	0	0	0	0
ENSG00000183439	1	0	1	0
ENSG00000183444	13	0	7	11
ENSG00000183454	0	0	2	10
ENSG00000183458	0	0	0	0
ENSG00000183461	0	0	0	0
ENSG00000183463	0	0	0	0
ENSG00000183470	0	0	0	0
ENSG00000183473	0	0	0	0
ENSG00000183474	0	284	200	250
ENSG00000183475	12	104	89	58
ENSG00000183476	0	0	0	0
ENSG00000183479	0	0	0	0
ENSG00000183484	1	0	4	1
ENSG00000183486	20	26	2	0
ENSG00000183495	17	32	49	61
ENSG00000183496	4	17	17	5
ENSG00000183504	0	0	0	0
ENSG00000183506	0	0	1	0
ENSG00000183508	530	6039	6411	4720
ENSG00000183513	44	56	63	194
ENSG00000183514	0	0	0	0
ENSG00000183520	32	57	49	18
ENSG00000183527	5	34	33	38
ENSG00000183530	9	96	114	208
ENSG00000183531	0	0	0	8
ENSG00000183535	0	0	0	0

ENSG00000183542	0	0	0	0
ENSG00000183549	0	0	0	0
ENSG00000183558	17	333	322	230
ENSG00000183559	0	0	0	0
ENSG00000183560	0	0	0	0
ENSG00000183562	6	0	1	0
ENSG00000183566	0	0	0	0
ENSG00000183569	1	0	0	0
ENSG00000183570	0	0	5	24
ENSG00000183571	1	0	0	0
ENSG00000183574	0	0	0	0
ENSG00000183576	130	8287	6985	7348
ENSG00000183578	0	0	0	0
ENSG00000183579	2	59	15	26
ENSG00000183580	1	0	4	0
ENSG00000183586	0	0	0	0
ENSG00000183597	200	2058	1391	1803
ENSG00000183598	0	0	3	0
ENSG00000183604	0	145	83	74
ENSG00000183605	2	0	8	5
ENSG00000183607	0	0	0	0
ENSG00000183615	0	0	0	0
ENSG00000183617	32	17	2	3
ENSG00000183621	9	104	228	183
ENSG00000183624	15	16	3	12
ENSG00000183625	1	0	1	0
ENSG00000183628	0	0	0	1
ENSG00000183629	0	0	0	0
ENSG00000183631	0	0	0	0
ENSG00000183632	0	0	0	0
ENSG00000183638	0	0	0	0
ENSG00000183640	0	0	0	0
ENSG00000183643	0	0	0	1
ENSG00000183644	0	0	0	0
ENSG00000183647	1	10	0	0
ENSG00000183648	284	226	197	273
ENSG00000183653	0	0	0	0
ENSG00000183654	0	0	0	0
ENSG00000183655	1	0	6	1
ENSG00000183657	0	0	0	0
ENSG00000183662	0	0	0	0
ENSG00000183663	0	0	0	0
ENSG00000183665	2	0	1	1
ENSG00000183666	0	36	17	25
ENSG00000183668	0	0	0	0
ENSG00000183671	21	126	146	45

ENSG00000183674	0	0	0	0
ENSG00000183675	0	0	0	0
ENSG00000183678	0	0	0	0
ENSG00000183682	0	0	0	0
ENSG00000183684	12	0	8	12
ENSG00000183688	507	1364	928	1845
ENSG00000183690	316	4048	4992	2573
ENSG00000183691	1	0	2	0
ENSG00000183695	0	0	0	0
ENSG00000183696	61	33	56	20
ENSG00000183704	0	0	0	0
ENSG00000183706	0	0	0	0
ENSG00000183709	0	0	0	0
ENSG00000183715	0	0	0	2
ENSG00000183718	1	0	3	9
ENSG00000183722	37	278	90	251
ENSG00000183723	17	72	69	142
ENSG00000183726	4550	17670	12056	21562
ENSG00000183729	0	0	0	0
ENSG00000183733	0	0	0	0
ENSG00000183734	0	0	0	0
ENSG00000183735	27	1298	1934	1805
ENSG00000183741	45	89	96	108
ENSG00000183742	6	71	290	212
ENSG00000183747	0	0	0	0
ENSG00000183748	0	0	0	1
ENSG00000183751	2	0	0	0
ENSG00000183753	0	0	0	0
ENSG00000183760	0	0	0	0
ENSG00000183762	2	0	0	5
ENSG00000183763	3	13	2	3
ENSG00000183765	4	638	671	730
ENSG00000183770	0	0	0	0
ENSG00000183773	1	0	0	0
ENSG00000183775	0	0	1	0
ENSG00000183778	0	0	0	0
ENSG00000183779	3	0	0	0
ENSG00000183780	0	0	0	0
ENSG00000183783	0	0	0	0
ENSG00000183784	3	0	4	3
ENSG00000183785	53	116	116	190
ENSG00000183791	0	0	0	0
ENSG00000183793	0	0	0	0
ENSG00000183795	0	0	0	0
ENSG00000183798	0	0	0	0
ENSG00000183801	0	0	0	0

ENSG00000183807	0	0	0	0
ENSG00000183808	17	120	186	187
ENSG00000183813	58	1380	1587	1165
ENSG00000183814	1	598	396	657
ENSG00000183822	0	0	0	0
ENSG00000183826	1	187	183	131
ENSG00000183828	1	0	0	0
ENSG00000183831	0	18	35	69
ENSG00000183833	0	0	0	0
ENSG00000183837	0	0	0	0
ENSG00000183840	0	0	0	0
ENSG00000183844	1	0	0	0
ENSG00000183850	0	217	174	404
ENSG00000183853	1	0	0	0
ENSG00000183856	0	0	1	3
ENSG00000183862	0	0	0	0
ENSG00000183864	32	33	1	8
ENSG00000183873	0	0	0	0
ENSG00000183876	0	0	0	0
ENSG00000183878	66	3878	5	3808
ENSG00000183888	0	0	0	0
ENSG00000183889	0	0	0	0
ENSG00000183891	24	14	45	27
ENSG00000183900	0	0	0	0
ENSG00000183908	0	0	0	0
ENSG00000183909	0	0	0	0
ENSG00000183911	0	0	0	0
ENSG00000183914	7	25	79	67
ENSG00000183918	72	376	461	690
ENSG00000183921	0	0	0	0
ENSG00000183929	0	0	0	0
ENSG00000183935	0	1	0	1
ENSG00000183938	0	0	0	2
ENSG00000183941	0	86	29	20
ENSG00000183943	56	71	76	37
ENSG00000183955	25	73	196	215
ENSG00000183960	0	23	15	6
ENSG00000183963	19	100	58	98
ENSG00000183971	0	0	0	0
ENSG00000183977	0	0	1	1
ENSG00000183978	292	32	35	91
ENSG00000183979	0	0	0	0
ENSG00000183981	0	0	0	0
ENSG00000184005	0	0	2	0
ENSG00000184007	9430	33697	36338	30377
ENSG00000184009	13991	26037	17223	30413

ENSG00000184012	0	0	0	0
ENSG00000184014	13	22	65	19
ENSG00000184022	0	0	0	0
ENSG00000184029	0	0	0	0
ENSG00000184032	0	0	0	0
ENSG00000184033	0	0	0	0
ENSG00000184040	0	0	0	3
ENSG00000184047	2	41	10	11
ENSG00000184055	0	0	0	0
ENSG00000184056	6	0	16	15
ENSG00000184058	0	0	0	0
ENSG00000184060	29	0	0	0
ENSG00000184068	3	0	2	0
ENSG00000184076	1654	302	319	421
ENSG00000184083	2	22	27	21
ENSG00000184084	0	0	0	0
ENSG00000184100	0	0	0	0
ENSG00000184106	85	85	33	84
ENSG00000184108	0	0	0	0
ENSG00000184110	0	0	1	6
ENSG00000184111	0	0	0	0
ENSG00000184113	1124	1279	566	555
ENSG00000184115	3	0	2	3
ENSG00000184117	18	0	0	0
ENSG00000184139	0	0	0	6
ENSG00000184140	0	0	0	0
ENSG00000184144	0	0	0	0
ENSG00000184148	0	0	0	0
ENSG00000184154	28	9	42	42
ENSG00000184155	0	0	0	0
ENSG00000184156	0	0	0	0
ENSG00000184160	1	0	0	0
ENSG00000184162	4	0	0	0
ENSG00000184163	0	0	0	0
ENSG00000184164	1	0	0	1
ENSG00000184166	0	0	0	0
ENSG00000184178	1892	11523	8377	15564
ENSG00000184182	1517	1920	1513	1355
ENSG00000184185	0	0	0	0
ENSG00000184188	0	0	0	0
ENSG00000184194	0	0	0	0
ENSG00000184203	50	450	570	655
ENSG00000184205	67	149	163	100
ENSG00000184206	0	0	7	0
ENSG00000184207	23	3	11	15
ENSG00000184208	7	10	3	0

ENSG00000184209	61	157	84	101
ENSG00000184210	1	0	0	8
ENSG00000184216	48	44	58	71
ENSG00000184220	15	59	22	57
ENSG00000184221	0	0	0	0
ENSG00000184224	5	0	5	3
ENSG00000184226	96	407	659	418
ENSG00000184227	0	0	0	0
ENSG00000184232	0	0	0	0
ENSG00000184247	0	0	0	0
ENSG00000184254	3	0	2	1
ENSG00000184258	0	0	1	0
ENSG00000184260	0	751	237	576
ENSG00000184261	0	0	0	0
ENSG00000184270	1	125	55	170
ENSG00000184271	0	0	0	0
ENSG00000184274	0	0	0	0
ENSG00000184276	0	0	0	0
ENSG00000184277	43	232	354	204
ENSG00000184281	1	29	3	9
ENSG00000184292	2	0	0	0
ENSG00000184293	2	0	0	0
ENSG00000184302	0	0	0	0
ENSG00000184303	0	0	0	0
ENSG00000184304	0	30	18	121
ENSG00000184305	1	156	57	106
ENSG00000184307	0	0	0	0
ENSG00000184313	1	0	0	0
ENSG00000184319	1399	4692	1262	6598
ENSG00000184321	0	0	0	0
ENSG00000184324	0	0	0	0
ENSG00000184330	0	0	0	0
ENSG00000184343	3	0	0	4
ENSG00000184344	0	0	0	0
ENSG00000184345	0	0	0	0
ENSG00000184347	0	0	0	2
ENSG00000184348	16	100	183	58
ENSG00000184349	3	41	19	56
ENSG00000184350	0	0	0	0
ENSG00000184351	0	0	0	0
ENSG00000184357	5	19	32	41
ENSG00000184361	0	0	0	1
ENSG00000184363	0	0	0	0
ENSG00000184368	0	0	2	2
ENSG00000184371	15	41	11	2
ENSG00000184374	0	0	0	0

ENSG00000184378	1	0	4	0
ENSG00000184381	0	0	0	8
ENSG00000184384	3	24	13	20
ENSG00000184385	0	0	0	0
ENSG00000184388	0	0	0	0
ENSG00000184389	0	0	0	0
ENSG00000184394	0	0	0	0
ENSG00000184402	7	34	34	21
ENSG00000184408	0	0	6	0
ENSG00000184414	1	0	0	0
ENSG00000184423	0	0	0	0
ENSG00000184428	1	0	0	0
ENSG00000184432	82	472	570	638
ENSG00000184434	0	0	0	0
ENSG00000184436	5	0	1	0
ENSG00000184441	1	0	0	0
ENSG00000184445	13	807	960	1173
ENSG00000184451	0	0	0	0
ENSG00000184454	0	0	0	0
ENSG00000184459	0	0	0	0
ENSG00000184465	2	0	17	43
ENSG00000184470	6	0	0	0
ENSG00000184471	0	0	0	0
ENSG00000184478	0	0	0	0
ENSG00000184481	42	362	324	327
ENSG00000184486	0	0	0	0
ENSG00000184489	26	156	61	136
ENSG00000184490	0	0	0	0
ENSG00000184492	0	0	0	0
ENSG00000184494	0	0	0	0
ENSG00000184497	0	0	0	0
ENSG00000184500	745	12718	13302	27152
ENSG00000184502	0	0	0	0
ENSG00000184507	0	0	0	0
ENSG00000184508	10	14	8	6
ENSG00000184515	1	0	1	0
ENSG00000184517	4	103	22	27
ENSG00000184523	0	0	0	0
ENSG00000184524	0	0	0	0
ENSG00000184530	0	0	1	0
ENSG00000184544	0	0	0	0
ENSG00000184545	0	0	0	0
ENSG00000184551	0	0	0	0
ENSG00000184557	2	0	2	0
ENSG00000184560	0	0	0	0
ENSG00000184564	0	0	0	0

ENSG00000184566	1	0	0	0
ENSG00000184571	0	0	0	0
ENSG00000184574	479	571	667	996
ENSG00000184575	20	422	556	564
ENSG00000184584	7	0	0	3
ENSG00000184586	0	0	0	0
ENSG00000184588	4	0	6	17
ENSG00000184596	0	0	0	0
ENSG00000184599	0	0	0	0
ENSG00000184601	0	0	0	0
ENSG00000184602	3417	20508	14092	24416
ENSG00000184608	0	0	0	0
ENSG00000184611	0	0	7	0
ENSG00000184612	0	0	0	0
ENSG00000184613	2	0	0	0
ENSG00000184616	0	0	0	0
ENSG00000184617	0	0	0	0
ENSG00000184619	0	0	0	1
ENSG00000184624	0	0	0	0
ENSG00000184634	15	264	140	208
ENSG00000184635	8	163	175	113
ENSG00000184640	132	177	212	712
ENSG00000184647	0	0	0	0
ENSG00000184650	0	0	0	0
ENSG00000184659	0	0	0	0
ENSG00000184661	1	65	68	143
ENSG00000184669	0	0	0	0
ENSG00000184672	0	0	0	2
ENSG00000184674	31	23	8	34
ENSG00000184675	1	5	12	20
ENSG00000184677	3	27	3	15
ENSG00000184678	4644	26647	40289	19406
ENSG00000184682	0	0	0	0
ENSG00000184697	0	0	0	0
ENSG00000184698	0	0	0	0
ENSG00000184702	102	6039	2612	5841
ENSG00000184708	62	451	562	975
ENSG00000184709	0	0	0	0
ENSG00000184716	0	2	22	4
ENSG00000184719	2	25	62	58
ENSG00000184724	0	0	0	0
ENSG00000184730	1	17	10	4
ENSG00000184731	0	0	0	0
ENSG00000184735	0	0	0	0
ENSG00000184741	0	0	0	0
ENSG00000184743	70	1942	3632	2971

ENSG00000184750	0	0	0	0
ENSG00000184752	58	21	7	6
ENSG00000184761	0	0	0	0
ENSG00000184774	0	0	0	0
ENSG00000184779	1	0	1	0
ENSG00000184785	6	12	9	31
ENSG00000184786	0	0	0	0
ENSG00000184787	162	30	61	102
ENSG00000184788	6	2	0	0
ENSG00000184789	0	0	0	0
ENSG00000184792	95	1352	2227	681
ENSG00000184795	0	0	0	0
ENSG00000184809	0	0	0	0
ENSG00000184811	0	0	0	0
ENSG00000184814	0	0	0	0
ENSG00000184825	1	0	16	22
ENSG00000184828	3	0	0	0
ENSG00000184831	4	33	20	30
ENSG00000184838	70	8	259	280
ENSG00000184840	316	325	135	342
ENSG00000184844	0	0	0	0
ENSG00000184845	0	0	2	0
ENSG00000184856	0	0	0	0
ENSG00000184857	1	0	13	4
ENSG00000184860	0	0	0	0
ENSG00000184863	48	37260	34020	48015
ENSG00000184867	1	0	1	0
ENSG00000184879	0	0	0	0
ENSG00000184881	0	0	0	0
ENSG00000184886	2	8	3	6
ENSG00000184887	446	509	313	667
ENSG00000184895	0	0	0	0
ENSG00000184897	3	18	104	44
ENSG00000184898	23	64	132	95
ENSG00000184900	1690	2603	1640	3606
ENSG00000184903	66	650	262	932
ENSG00000184905	0	0	0	0
ENSG00000184906	0	0	0	0
ENSG00000184908	2	0	0	0
ENSG00000184909	0	0	0	0
ENSG00000184911	0	0	0	0
ENSG00000184916	0	0	2	0
ENSG00000184922	11	0	5	16
ENSG00000184923	0	0	1	0
ENSG00000184924	60	234	93	193
ENSG00000184925	0	0	0	0

ENSG00000184933	0	0	0	0
ENSG00000184937	0	0	3	0
ENSG00000184939	1	0	1	17
ENSG00000184945	0	0	0	0
ENSG00000184949	1	1	0	3
ENSG00000184954	0	0	0	0
ENSG00000184956	0	13	0	0
ENSG00000184961	0	0	0	0
ENSG00000184967	4	0	1	0
ENSG00000184979	2	0	7	3
ENSG00000184983	9368	11427	6843	9361
ENSG00000184984	0	0	0	0
ENSG00000184985	0	0	0	0
ENSG00000184986	0	0	0	0
ENSG00000184988	0	20	26	20
ENSG00000184990	18	3	1	0
ENSG00000184991	0	0	0	0
ENSG00000184992	12	0	2	13
ENSG00000184995	0	0	0	0
ENSG00000184999	0	0	0	0
ENSG00000185000	2	0	1	0
ENSG00000185002	0	0	0	0
ENSG00000185008	0	0	0	0
ENSG00000185009	32	187	234	386
ENSG00000185010	22	636	1098	1434
ENSG00000185013	0	0	0	0
ENSG00000185015	135	1663	3127	3460
ENSG00000185019	21	23	32	44
ENSG00000185020	0	0	0	0
ENSG00000185022	2	17	6	0
ENSG00000185024	34	67	88	150
ENSG00000185028	0	0	0	0
ENSG00000185031	0	0	0	0
ENSG00000185033	8	359	252	434
ENSG00000185037	0	0	0	0
ENSG00000185038	0	0	0	0
ENSG00000185040	1	0	0	0
ENSG00000185041	0	0	0	0
ENSG00000185043	60	42	34	40
ENSG00000185044	2	0	5	1
ENSG00000185046	1	0	1	0
ENSG00000185049	1	0	0	3
ENSG00000185052	342	4461	3504	6174
ENSG00000185053	0	0	0	0
ENSG00000185055	0	0	0	0
ENSG00000185056	0	0	3	8

ENSG00000185065	2	0	3	0
ENSG00000185068	423	636	601	610
ENSG00000185069	0	0	0	0
ENSG00000185070	0	0	0	0
ENSG00000185074	0	0	0	0
ENSG00000185078	0	0	0	0
ENSG00000185085	4	0	2	0
ENSG00000185087	0	0	0	2
ENSG00000185088	457	2806	983	1490
ENSG00000185090	0	0	0	0
ENSG00000185097	0	0	0	0
ENSG00000185100	0	0	0	2
ENSG00000185101	0	0	0	0
ENSG00000185104	19	841	1308	2358
ENSG00000185105	0	0	0	0
ENSG00000185112	0	0	0	0
ENSG00000185115	270	605	239	452
ENSG00000185122	2	28	6	9
ENSG00000185127	11	110	137	140
ENSG00000185128	0	18	11	1
ENSG00000185129	263	315	346	250
ENSG00000185130	9	143	170	50
ENSG00000185133	0	0	0	0
ENSG00000185149	0	0	0	0
ENSG00000185155	1	0	0	0
ENSG00000185156	0	0	0	4
ENSG00000185158	6	38	90	206
ENSG00000185162	0	0	0	0
ENSG00000185163	6	0	0	0
ENSG00000185164	1	44	13	47
ENSG00000185168	0	0	0	0
ENSG00000185176	0	0	0	0
ENSG00000185177	0	0	1	0
ENSG00000185182	0	0	0	0
ENSG00000185186	1	0	0	0
ENSG00000185187	2	0	0	4
ENSG00000185189	6	0	3	6
ENSG00000185198	0	0	0	0
ENSG00000185201	878	132	145	74
ENSG00000185203	0	0	0	0
ENSG00000185215	26	0	3	3
ENSG00000185219	4	0	11	4
ENSG00000185220	4	0	25	24
ENSG00000185221	0	0	0	4
ENSG00000185222	119	992	620	806
ENSG00000185231	0	0	0	0

ENSG00000185236	294	855	649	1001
ENSG00000185238	53	579	1035	1379
ENSG00000185245	2132	6932	4263	7301
ENSG00000185246	9	364	246	351
ENSG00000185247	0	0	0	0
ENSG00000185250	2	0	7	12
ENSG00000185252	0	0	0	0
ENSG00000185254	0	0	0	0
ENSG00000185261	0	47	98	79
ENSG00000185262	209	624	296	159
ENSG00000185264	0	0	0	0
ENSG00000185267	1	36	48	85
ENSG00000185269	0	0	0	0
ENSG00000185271	0	0	0	0
ENSG00000185272	0	0	4	0
ENSG00000185274	0	0	0	1
ENSG00000185275	0	0	1	0
ENSG00000185278	2	37	41	34
ENSG00000185290	0	0	0	0
ENSG00000185291	5	0	1	0
ENSG00000185294	0	0	0	0
ENSG00000185296	0	0	0	0
ENSG00000185298	6	42	13	15
ENSG00000185303	0	0	0	0
ENSG00000185304	0	0	0	0
ENSG00000185305	235	7431	10173	7141
ENSG00000185306	0	0	0	0
ENSG00000185313	0	0	0	1
ENSG00000185319	0	0	0	0
ENSG00000185324	2	0	8	21
ENSG00000185332	0	0	0	0
ENSG00000185338	0	0	0	0
ENSG00000185339	0	0	0	0
ENSG00000185340	625	888	506	887
ENSG00000185344	9	209	349	635
ENSG00000185345	8	0	3	33
ENSG00000185347	0	0	0	0
ENSG00000185352	0	0	0	0
ENSG00000185359	1	97	23	20
ENSG00000185361	604	491	189	334
ENSG00000185372	0	0	0	0
ENSG00000185379	3	0	0	0
ENSG00000185385	0	0	0	0
ENSG00000185386	0	0	0	0
ENSG00000185390	0	0	0	0
ENSG00000185404	20	42	69	85

ENSG00000185414	0	0	2	0
ENSG00000185418	67	3603	3684	4899
ENSG00000185420	508	4014	2929	4001
ENSG00000185432	149	1202	342	772
ENSG00000185433	0	27	9	9
ENSG00000185436	5	0	0	0
ENSG00000185437	0	0	0	31
ENSG00000185442	1	0	4	2
ENSG00000185448	0	0	0	0
ENSG00000185453	1	0	6	0
ENSG00000185467	0	0	0	0
ENSG00000185475	10	7	9	12
ENSG00000185477	10	13	6	4
ENSG00000185479	0	0	0	0
ENSG00000185480	6	922	732	1388
ENSG00000185482	1	15	4	18
ENSG00000185483	1	0	2	8
ENSG00000185485	0	35	14	49
ENSG00000185495	0	23	0	0
ENSG00000185499	0	0	1	0
ENSG00000185504	2	0	3	1
ENSG00000185507	8	3	3	1
ENSG00000185513	7	68	36	55
ENSG00000185515	19	30	64	75
ENSG00000185518	0	22	0	0
ENSG00000185519	0	0	0	0
ENSG00000185522	0	0	1	2
ENSG00000185523	0	0	3	3
ENSG00000185527	5	0	0	0
ENSG00000185532	121	4674	5716	7437
ENSG00000185551	0	0	1	0
ENSG00000185554	0	0	0	0
ENSG00000185559	2	15	10	6
ENSG00000185561	0	0	0	0
ENSG00000185565	0	23	0	0
ENSG00000185567	1	0	0	0
ENSG00000185585	0	0	2	18
ENSG00000185591	100	572	660	563
ENSG00000185594	0	0	2	0
ENSG00000185596	41	101	43	71
ENSG00000185607	0	0	0	0
ENSG00000185608	15	0	0	1
ENSG00000185610	6	0	8	12
ENSG00000185614	118	353	169	165
ENSG00000185615	0	0	0	0
ENSG00000185619	45	338	197	239

ENSG00000185621	31	399	420	471
ENSG00000185624	46	360	391	949
ENSG00000185627	184	251	160	326
ENSG00000185630	2140	4978	5359	7881
ENSG00000185631	0	0	0	0
ENSG00000185633	0	0	0	0
ENSG00000185634	1	0	12	16
ENSG00000185638	0	0	0	0
ENSG00000185640	0	0	0	0
ENSG00000185641	3	13	2	6
ENSG00000185650	141	95	32	71
ENSG00000185651	1100	4406	3450	4063
ENSG00000185652	0	0	0	0
ENSG00000185658	204	12679	10234	18576
ENSG00000185662	0	0	0	0
ENSG00000185664	4	7	17	2
ENSG00000185666	3	0	1	3
ENSG00000185668	0	0	0	0
ENSG00000185669	1	0	0	0
ENSG00000185670	4	15	3	1
ENSG00000185674	0	0	0	0
ENSG00000185681	0	0	0	0
ENSG00000185684	19	19	20	13
ENSG00000185686	0	0	0	0
ENSG00000185689	0	0	0	0
ENSG00000185697	7	32	62	70
ENSG00000185700	0	0	0	0
ENSG00000185701	0	0	0	0
ENSG00000185710	0	0	1	1
ENSG00000185716	39	60	88	230
ENSG00000185721	31	148	81	152
ENSG00000185722	3	302	235	229
ENSG00000185728	34	586	649	631
ENSG00000185730	0	0	0	0
ENSG00000185736	71	281	0	119
ENSG00000185737	0	0	0	0
ENSG00000185739	0	0	1	0
ENSG00000185742	0	10	62	3
ENSG00000185745	6	19	37	69
ENSG00000185751	0	0	0	0
ENSG00000185753	18	0	16	18
ENSG00000185758	0	0	0	0
ENSG00000185760	0	0	21	29
ENSG00000185761	0	0	0	0
ENSG00000185774	0	0	0	0
ENSG00000185775	0	0	0	0

ENSG00000185787	1927	8017	5981	6157
ENSG00000185792	0	0	0	2
ENSG00000185798	0	0	0	0
ENSG00000185800	44	155	175	140
ENSG00000185803	1	0	1	0
ENSG00000185808	13	33	17	57
ENSG00000185811	114	152	123	90
ENSG00000185813	5	14	9	6
ENSG00000185818	0	0	0	0
ENSG00000185821	0	0	0	0
ENSG00000185823	0	0	0	0
ENSG00000185825	2562	4826	2750	2905
ENSG00000185829	3	35	9	14
ENSG00000185834	0	0	0	0
ENSG00000185837	0	0	0	0
ENSG00000185838	1	0	0	0
ENSG00000185839	0	0	0	0
ENSG00000185842	6	3357	3401	4423
ENSG00000185847	0	0	0	0
ENSG00000185860	0	0	0	0
ENSG00000185862	157	0	18	32
ENSG00000185863	0	0	0	0
ENSG00000185864	9	2	45	116
ENSG00000185869	3	0	9	28
ENSG00000185873	0	0	0	1
ENSG00000185875	4	72	52	39
ENSG00000185880	102	630	1111	392
ENSG00000185883	0	0	2	2
ENSG00000185885	31	0	2	4
ENSG00000185888	0	0	0	0
ENSG00000185894	0	0	0	0
ENSG00000185896	329	2098	1611	2232
ENSG00000185897	2	1	26	0
ENSG00000185899	0	0	0	0
ENSG00000185900	2	0	18	33
ENSG00000185903	0	0	0	0
ENSG00000185904	0	0	2	8
ENSG00000185905	21	110	49	86
ENSG00000185909	90	153	140	51
ENSG00000185915	0	0	0	0
ENSG00000185917	2	9	12	20
ENSG00000185920	4	37	1	21
ENSG00000185924	0	0	0	0
ENSG00000185926	0	0	0	0
ENSG00000185928	4	26	2	0
ENSG00000185933	0	0	0	0

ENSG00000185940	0	0	0	0
ENSG00000185942	1	0	2	5
ENSG00000185945	0	0	0	0
ENSG00000185946	38	580	337	445
ENSG00000185947	43	59	110	64
ENSG00000185950	98	176	232	253
ENSG00000185955	0	0	1	0
ENSG00000185958	0	0	0	0
ENSG00000185960	2	0	1	0
ENSG00000185962	0	0	0	0
ENSG00000185963	2549	6389	5055	6028
ENSG00000185966	0	0	0	0
ENSG00000185972	0	0	0	0
ENSG00000185973	5	514	381	553
ENSG00000185974	0	0	0	0
ENSG00000185978	0	0	0	0
ENSG00000185982	0	0	0	0
ENSG00000185985	0	0	0	0
ENSG00000185986	2	1	7	0
ENSG00000185988	0	0	0	0
ENSG00000185989	976	5118	3488	6807
ENSG00000185990	0	0	0	0
ENSG00000186001	14	9151	6623	9806
ENSG00000186007	0	0	0	0
ENSG00000186008	0	0	0	0
ENSG00000186009	0	0	0	0
ENSG00000186010	0	0	1	0
ENSG00000186017	7	664	615	702
ENSG00000186019	2	147	61	71
ENSG00000186020	33	256	188	135
ENSG00000186026	0	0	1	0
ENSG00000186038	0	0	0	0
ENSG00000186047	2	0	1	0
ENSG00000186049	0	0	0	0
ENSG00000186051	0	0	0	0
ENSG00000186056	0	0	0	0
ENSG00000186063	685	2185	3090	2448
ENSG00000186073	2	94	58	99
ENSG00000186074	8	0	0	0
ENSG00000186075	0	0	0	0
ENSG00000186076	0	0	0	0
ENSG00000186081	1	0	0	0
ENSG00000186082	0	0	0	0
ENSG00000186086	0	0	0	0
ENSG00000186088	190	13340	15435	20688
ENSG00000186090	0	0	0	0

ENSG00000186092	0	0	0	0
ENSG00000186094	0	0	0	0
ENSG00000186103	0	0	0	0
ENSG00000186104	1	39	53	43
ENSG00000186105	0	13	18	8
ENSG00000186106	2	362	264	303
ENSG00000186111	106	1248	510	1118
ENSG00000186113	0	0	0	0
ENSG00000186115	0	0	0	0
ENSG00000186117	0	0	0	0
ENSG00000186118	0	0	0	0
ENSG00000186119	0	0	0	0
ENSG00000186124	0	0	0	0
ENSG00000186130	2	85	69	55
ENSG00000186132	5	57	52	73
ENSG00000186136	0	0	0	0
ENSG00000186141	7	653	445	701
ENSG00000186143	0	0	0	0
ENSG00000186146	0	0	0	0
ENSG00000186148	0	0	0	0
ENSG00000186150	0	0	0	0
ENSG00000186152	0	0	0	0
ENSG00000186153	546	588	682	495
ENSG00000186160	0	0	0	0
ENSG00000186162	468	560	547	300
ENSG00000186163	0	0	0	0
ENSG00000186166	2	17	7	3
ENSG00000186174	3	0	0	0
ENSG00000186184	226	512	504	515
ENSG00000186185	1	3	3	9
ENSG00000186187	8	126	223	384
ENSG00000186188	0	0	0	0
ENSG00000186190	36	23	25	148
ENSG00000186191	12	0	6	2
ENSG00000186193	1	0	2	1
ENSG00000186197	0	0	0	0
ENSG00000186198	0	14	2	3
ENSG00000186204	0	0	0	0
ENSG00000186205	8	0	22	49
ENSG00000186207	0	0	0	0
ENSG00000186212	0	0	0	0
ENSG00000186222	13	0	3	0
ENSG00000186223	0	0	0	0
ENSG00000186226	0	0	0	0
ENSG00000186230	0	0	0	4
ENSG00000186231	0	0	0	0

ENSG00000186232	0	0	0	0
ENSG00000186234	0	0	0	0
ENSG00000186235	0	0	3	1
ENSG00000186244	0	0	0	0
ENSG00000186260	7	95	102	78
ENSG00000186265	4	64	17	16
ENSG00000186268	0	0	0	0
ENSG00000186272	1	0	4	15
ENSG00000186275	2	30	20	36
ENSG00000186280	0	0	0	0
ENSG00000186281	0	0	0	0
ENSG00000186283	5	0	0	0
ENSG00000186288	0	0	0	0
ENSG00000186297	0	0	2	0
ENSG00000186298	93	503	506	503
ENSG00000186300	6	0	11	8
ENSG00000186301	0	0	0	0
ENSG00000186306	0	0	0	0
ENSG00000186310	6	125	130	131
ENSG00000186312	7	2	7	11
ENSG00000186314	21	2817	2123	4005
ENSG00000186318	17	102	67	68
ENSG00000186322	0	0	2	0
ENSG00000186326	0	0	0	0
ENSG00000186328	0	0	0	0
ENSG00000186329	3	2	34	37
ENSG00000186334	0	0	0	0
ENSG00000186335	0	0	0	0
ENSG00000186340	0	0	0	0
ENSG00000186350	24	0	0	0
ENSG00000186352	0	0	3	0
ENSG00000186354	0	0	0	0
ENSG00000186364	0	0	4	0
ENSG00000186367	0	0	0	0
ENSG00000186369	0	0	0	0
ENSG00000186376	5	1	34	32
ENSG00000186377	0	0	0	1
ENSG00000186393	0	0	0	0
ENSG00000186395	24	0	0	0
ENSG00000186399	1	0	3	11
ENSG00000186400	0	0	0	0
ENSG00000186407	8	0	0	0
ENSG00000186409	48	445	287	501
ENSG00000186416	5	1	12	24
ENSG00000186417	0	0	0	0
ENSG00000186431	0	0	0	0

ENSG00000186432	124	936	1183	896
ENSG00000186439	2	0	0	2
ENSG00000186440	0	0	0	0
ENSG00000186442	0	0	0	0
ENSG00000186446	0	0	7	3
ENSG00000186448	4	53	50	53
ENSG00000186451	0	0	0	0
ENSG00000186452	2	0	0	4
ENSG00000186453	2	0	1	0
ENSG00000186458	0	0	0	0
ENSG00000186462	0	0	0	0
ENSG00000186466	0	19	2	6
ENSG00000186468	144	198	79	120
ENSG00000186469	72	11	19	2
ENSG00000186470	1629	4877	4492	1785
ENSG00000186471	1	0	0	0
ENSG00000186472	0	0	3	4
ENSG00000186474	0	0	0	0
ENSG00000186479	0	0	0	0
ENSG00000186480	1245	6731	6322	2628
ENSG00000186481	1	9	14	16
ENSG00000186487	3	0	0	13
ENSG00000186493	0	0	0	0
ENSG00000186496	2	0	19	3
ENSG00000186501	116	77	38	83
ENSG00000186508	0	0	0	0
ENSG00000186509	0	0	0	0
ENSG00000186510	0	0	0	0
ENSG00000186513	0	0	0	0
ENSG00000186517	69	0	1	5
ENSG00000186522	4	0	10	3
ENSG00000186523	0	0	0	4
ENSG00000186526	0	0	2	0
ENSG00000186529	2	0	0	0
ENSG00000186530	0	0	0	0
ENSG00000186532	97	22	35	70
ENSG00000186543	0	0	0	0
ENSG00000186562	0	0	0	0
ENSG00000186564	1	19	3	5
ENSG00000186566	63	294	579	766
ENSG00000186567	1	0	0	0
ENSG00000186572	0	0	0	0
ENSG00000186575	10	97	64	160
ENSG00000186577	56	10	17	36
ENSG00000186579	0	0	0	0
ENSG00000186583	0	0	2	0

ENSG00000186591	1604	7721	8588	3103
ENSG00000186594	44	228	115	138
ENSG00000186599	0	0	0	0
ENSG00000186603	0	0	0	0
ENSG00000186615	3	0	0	0
ENSG00000186625	23	661	281	440
ENSG00000186628	0	16	2	6
ENSG00000186635	53	995	283	398
ENSG00000186638	0	253	213	258
ENSG00000186642	41	313	156	393
ENSG00000186645	0	0	0	0
ENSG00000186648	0	0	0	0
ENSG00000186652	0	0	0	0
ENSG00000186654	5	0	3	0
ENSG00000186660	91	553	691	542
ENSG00000186665	10	0	32	28
ENSG00000186666	0	0	0	2
ENSG00000186675	0	0	0	0
ENSG00000186676	1	0	4	2
ENSG00000186678	0	0	0	0
ENSG00000186684	0	0	0	0
ENSG00000186687	41	204	299	356
ENSG00000186704	0	5	0	0
ENSG00000186710	0	0	0	0
ENSG00000186714	2	0	42	22
ENSG00000186715	0	0	0	2
ENSG00000186716	500	1170	637	848
ENSG00000186723	0	0	0	0
ENSG00000186732	0	0	0	0
ENSG00000186743	0	0	0	0
ENSG00000186765	0	0	0	0
ENSG00000186766	0	0	0	0
ENSG00000186767	2	119	68	65
ENSG00000186777	0	16	25	54
ENSG00000186787	118	42	47	216
ENSG00000186788	0	0	0	0
ENSG00000186790	0	0	0	0
ENSG00000186792	32	14	52	20
ENSG00000186795	0	0	0	0
ENSG00000186803	0	0	0	0
ENSG00000186806	3	0	11	10
ENSG00000186807	0	0	0	0
ENSG00000186810	4	0	0	0
ENSG00000186812	57	231	129	212
ENSG00000186814	40	174	67	65
ENSG00000186815	24	207	81	227

ENSG00000186818	4	0	0	0
ENSG00000186825	0	0	0	0
ENSG00000186827	0	0	0	0
ENSG00000186831	0	0	0	0
ENSG00000186832	0	0	0	0
ENSG00000186834	245	762	699	553
ENSG00000186838	0	0	0	0
ENSG00000186842	0	0	0	0
ENSG00000186844	0	0	0	0
ENSG00000186847	0	0	0	0
ENSG00000186854	1	0	1	0
ENSG00000186860	0	0	2	1
ENSG00000186862	0	0	0	0
ENSG00000186866	3	6	0	2
ENSG00000186867	0	0	0	0
ENSG00000186868	0	0	0	0
ENSG00000186871	0	25	4	22
ENSG00000186881	0	0	0	0
ENSG00000186886	0	0	0	0
ENSG00000186889	133	153	179	101
ENSG00000186891	0	0	0	0
ENSG00000186895	0	0	0	0
ENSG00000186897	0	0	0	0
ENSG00000186907	0	0	0	0
ENSG00000186908	4	417	400	576
ENSG00000186910	0	0	0	0
ENSG00000186912	0	0	0	0
ENSG00000186918	7	0	2	0
ENSG00000186919	0	0	1	7
ENSG00000186924	0	0	0	0
ENSG00000186925	0	0	0	0
ENSG00000186930	0	0	0	0
ENSG00000186940	0	0	0	0
ENSG00000186943	0	0	0	0
ENSG00000186951	14	162	66	92
ENSG00000186952	0	189	207	344
ENSG00000186960	0	0	0	0
ENSG00000186965	0	0	0	0
ENSG00000186967	0	0	0	0
ENSG00000186970	0	0	0	0
ENSG00000186971	0	0	0	0
ENSG00000186973	0	0	0	0
ENSG00000186976	2	481	515	289
ENSG00000186977	0	0	0	0
ENSG00000186980	0	0	0	0
ENSG00000186994	0	0	0	0

ENSG00000186998	0	0	0	0
ENSG00000187003	0	0	0	0
ENSG00000187005	0	0	0	0
ENSG00000187008	36	190	262	428
ENSG00000187010	119	564	3	1366
ENSG00000187012	0	0	0	0
ENSG00000187013	0	0	0	0
ENSG00000187017	0	0	0	0
ENSG00000187021	0	0	0	0
ENSG00000187024	0	0	1	0
ENSG00000187026	0	0	0	0
ENSG00000187033	0	0	0	0
ENSG00000187037	2	0	1	2
ENSG00000187045	0	0	0	0
ENSG00000187048	0	0	1	1
ENSG00000187049	6	0	6	0
ENSG00000187051	122	371	210	486
ENSG00000187054	0	0	0	0
ENSG00000187060	0	0	0	0
ENSG00000187066	0	0	0	0
ENSG00000187068	2	0	0	4
ENSG00000187079	0	9	5	3
ENSG00000187080	0	19	5	7
ENSG00000187082	0	0	0	0
ENSG00000187091	1	0	0	0
ENSG00000187094	0	0	0	0
ENSG00000187097	62	382	577	232
ENSG00000187098	259	1524	1496	980
ENSG00000187103	0	0	0	0
ENSG00000187105	0	10	12	5
ENSG00000187109	31824	94950	133914	159042
ENSG00000187116	5	0	0	3
ENSG00000187118	28	1011	1017	1363
ENSG00000187122	0	0	0	0
ENSG00000187123	0	1	1	15
ENSG00000187134	3	89	69	157
ENSG00000187135	0	0	0	0
ENSG00000187140	0	0	0	0
ENSG00000187144	0	51	27	6
ENSG00000187145	92	63	19	18
ENSG00000187147	15	108	6	10
ENSG00000187151	0	0	0	0
ENSG00000187156	1	0	0	0
ENSG00000187164	44	0	58	73
ENSG00000187166	0	0	0	0
ENSG00000187170	0	0	0	0

ENSG00000187172	0	3	2	0
ENSG00000187173	0	0	0	0
ENSG00000187175	0	0	0	0
ENSG00000187180	0	0	0	0
ENSG00000187185	1	0	2	0
ENSG00000187186	0	0	0	0
ENSG00000187187	0	21	10	16
ENSG00000187189	20	49	50	23
ENSG00000187191	0	0	0	0
ENSG00000187193	57	25	0	8
ENSG00000187210	25	276	517	467
ENSG00000187223	0	0	0	0
ENSG00000187229	0	0	0	0
ENSG00000187231	314	3471	3607	4091
ENSG00000187238	0	0	0	0
ENSG00000187239	170	129	87	186
ENSG00000187240	4	137	121	130
ENSG00000187242	0	0	0	0
ENSG00000187243	0	0	0	0
ENSG00000187244	0	0	0	0
ENSG00000187257	89	1959	1398	2126
ENSG00000187258	0	0	0	0
ENSG00000187260	0	0	3	0
ENSG00000187266	188	332	245	414
ENSG00000187268	1	0	15	0
ENSG00000187272	0	0	0	0
ENSG00000187288	1	0	0	0
ENSG00000187323	0	4	0	0
ENSG00000187325	12	32	73	81
ENSG00000187372	0	0	0	0
ENSG00000187391	14	113	38	72
ENSG00000187398	0	0	0	0
ENSG00000187416	0	0	0	0
ENSG00000187446	105	298	256	477
ENSG00000187456	0	0	2	2
ENSG00000187461	0	0	0	0
ENSG00000187472	0	0	4	0
ENSG00000187474	0	0	1	0
ENSG00000187475	1	4	288	206
ENSG00000187479	0	0	0	0
ENSG00000187481	0	0	0	0
ENSG00000187483	0	0	0	0
ENSG00000187486	1	14	1	2
ENSG00000187492	0	0	2	0
ENSG00000187498	1	25	0	25
ENSG00000187504	0	0	0	0

ENSG00000187510	0	0	0	0
ENSG00000187513	13	0	12	11
ENSG00000187514	187450	108797	59094	104269
ENSG00000187516	0	0	0	0
ENSG00000187522	21	227	318	523
ENSG00000187527	8	60	26	20
ENSG00000187531	5	63	3	2
ENSG00000187533	0	0	0	0
ENSG00000187534	1	0	0	0
ENSG00000187535	3	38	21	45
ENSG00000187536	0	0	0	7
ENSG00000187537	0	0	0	0
ENSG00000187545	0	0	0	0
ENSG00000187546	0	1	0	0
ENSG00000187550	0	0	0	0
ENSG00000187553	0	0	0	0
ENSG00000187554	405	141	58	53
ENSG00000187555	116	1741	1981	3137
ENSG00000187556	20	15	15	12
ENSG00000187559	0	0	0	0
ENSG00000187566	0	0	1	0
ENSG00000187569	0	0	0	0
ENSG00000187581	0	0	0	0
ENSG00000187583	0	0	0	0
ENSG00000187589	0	0	0	0
ENSG00000187595	8	102	11	24
ENSG00000187600	0	0	0	0
ENSG00000187601	3	0	0	1
ENSG00000187605	0	38	133	153
ENSG00000187607	1	0	0	5
ENSG00000187608	18	0	2	5
ENSG00000187609	8	0	2	2
ENSG00000187612	0	0	0	0
ENSG00000187616	0	0	0	0
ENSG00000187621	13	244	279	1048
ENSG00000187624	0	0	0	1
ENSG00000187626	0	0	5	3
ENSG00000187627	0	1	6	5
ENSG00000187630	15	0	3	2
ENSG00000187634	0	0	0	0
ENSG00000187642	0	0	0	0
ENSG00000187650	2	45	7	5
ENSG00000187653	26149	24942	18239	27200
ENSG00000187657	0	0	0	0
ENSG00000187658	0	0	0	0
ENSG00000187664	0	0	0	0

ENSG00000187667	3340	8164	13870	18374
ENSG00000187672	48	970	1003	1480
ENSG00000187676	0	183	136	262
ENSG00000187678	0	12	0	0
ENSG00000187682	0	0	0	0
ENSG00000187686	0	0	0	0
ENSG00000187688	5	0	0	0
ENSG00000187689	0	0	0	0
ENSG00000187690	0	0	0	0
ENSG00000187695	0	0	0	3
ENSG00000187699	17331	80832	106688	74337
ENSG00000187701	0	0	0	0
ENSG00000187713	21	47	7	19
ENSG00000187714	7	0	3	0
ENSG00000187715	8	75	50	138
ENSG00000187720	0	0	0	0
ENSG00000187721	0	0	0	0
ENSG00000187726	0	0	0	0
ENSG00000187730	5	128	10	14
ENSG00000187733	0	0	0	0
ENSG00000187735	445	3160	3492	3656
ENSG00000187736	0	0	1	1
ENSG00000187741	213	29	96	158
ENSG00000187742	11240	8444	4918	12957
ENSG00000187747	0	0	0	0
ENSG00000187753	0	40	1	1
ENSG00000187754	0	0	0	0
ENSG00000187758	0	0	0	0
ENSG00000187762	0	0	0	0
ENSG00000187763	0	0	0	0
ENSG00000187764	2321	18824	17085	38140
ENSG00000187766	0	0	0	0
ENSG00000187772	0	0	0	1
ENSG00000187773	0	0	0	0
ENSG00000187775	0	0	0	0
ENSG00000187778	32	0	2	9
ENSG00000187783	0	0	0	0
ENSG00000187786	0	0	0	0
ENSG00000187790	4	555	473	786
ENSG00000187791	0	0	0	0
ENSG00000187792	0	2	3	15
ENSG00000187796	0	0	0	0
ENSG00000187800	390	2187	1589	1568
ENSG00000187801	0	0	3	0
ENSG00000187806	0	0	2	4
ENSG00000187808	0	0	0	0

ENSG00000187811	0	0	0	0
ENSG00000187812	0	0	1	0
ENSG00000187815	0	6	7	29
ENSG00000187821	0	0	0	0
ENSG00000187823	0	0	0	0
ENSG00000187824	0	20	8	22
ENSG00000187833	0	0	0	0
ENSG00000187837	1746	2722	3966	4002
ENSG00000187838	0	0	0	0
ENSG00000187840	42	57	15	22
ENSG00000187847	0	0	0	0
ENSG00000187848	0	0	0	0
ENSG00000187855	3	0	0	2
ENSG00000187857	0	0	0	0
ENSG00000187860	5	0	13	9
ENSG00000187862	0	0	0	0
ENSG00000187866	4	0	24	30
ENSG00000187867	0	0	0	0
ENSG00000187870	0	0	0	0
ENSG00000187871	0	0	0	0
ENSG00000187889	0	0	0	0
ENSG00000187893	0	0	0	0
ENSG00000187900	0	0	0	0
ENSG00000187902	0	0	0	0
ENSG00000187904	0	0	0	0
ENSG00000187905	0	0	0	0
ENSG00000187908	0	0	0	0
ENSG00000187912	0	0	0	0
ENSG00000187918	0	0	0	0
ENSG00000187922	0	0	0	0
ENSG00000187939	0	0	0	0
ENSG00000187942	0	0	0	0
ENSG00000187944	2	62	8	49
ENSG00000187950	0	0	4	5
ENSG00000187951	20	270	220	403
ENSG00000187952	0	0	0	0
ENSG00000187953	8	0	9	10
ENSG00000187954	0	27	9	12
ENSG00000187955	0	21	4	0
ENSG00000187957	0	0	0	0
ENSG00000187959	0	0	0	0
ENSG00000187961	0	0	0	0
ENSG00000187969	0	0	0	0
ENSG00000187979	0	0	0	0
ENSG00000187980	0	0	0	0
ENSG00000187984	1	0	2	0

ENSG00000187987	0	0	1	0
ENSG00000187988	0	0	0	0
ENSG00000187990	254	1601	10625	6963
ENSG00000187994	22	42	6	7
ENSG00000187997	0	0	0	4
ENSG00000187999	0	0	0	0
ENSG00000188000	0	0	0	0
ENSG00000188001	3	0	2	5
ENSG00000188002	14	20	6	8
ENSG00000188004	4	7	4	4
ENSG00000188010	5	70	114	168
ENSG00000188011	0	0	0	0
ENSG00000188013	4	183	75	134
ENSG00000188015	0	0	0	0
ENSG00000188021	40	61	149	194
ENSG00000188026	76	267	87	110
ENSG00000188029	0	0	0	0
ENSG00000188032	1	0	0	0
ENSG00000188033	7	0	20	31
ENSG00000188037	0	0	1	0
ENSG00000188038	0	0	0	0
ENSG00000188039	0	0	0	0
ENSG00000188042	127	0	2	0
ENSG00000188050	0	0	0	0
ENSG00000188051	0	0	0	0
ENSG00000188056	0	0	4	16
ENSG00000188060	0	0	0	0
ENSG00000188064	0	0	0	0
ENSG00000188069	0	0	0	0
ENSG00000188070	1	0	1	0
ENSG00000188073	0	1	4	2
ENSG00000188076	2009	275	267	988
ENSG00000188078	0	0	0	0
ENSG00000188086	0	0	0	0
ENSG00000188089	0	0	0	0
ENSG00000188092	0	29	4	5
ENSG00000188095	0	0	0	0
ENSG00000188100	0	0	0	0
ENSG00000188101	0	0	0	0
ENSG00000188107	3	0	0	0
ENSG00000188112	1	0	0	0
ENSG00000188120	0	0	0	0
ENSG00000188124	0	0	0	0
ENSG00000188126	0	0	0	0
ENSG00000188130	1	0	0	0
ENSG00000188133	0	0	0	0

ENSG00000188152	0	0	0	0
ENSG00000188153	0	0	0	0
ENSG00000188155	0	0	0	0
ENSG00000188157	0	0	0	0
ENSG00000188158	0	0	0	0
ENSG00000188162	0	0	0	0
ENSG00000188163	0	0	5	1
ENSG00000188167	5	150	107	125
ENSG00000188171	16	89	104	190
ENSG00000188175	0	26	0	2
ENSG00000188176	0	0	0	0
ENSG00000188177	3646	1327	1805	1931
ENSG00000188185	0	83	100	40
ENSG00000188186	898	431	105	265
ENSG00000188191	1621	1031	760	1705
ENSG00000188199	0	0	0	0
ENSG00000188206	4	135	420	434
ENSG00000188211	1	0	0	0
ENSG00000188215	0	40	10	4
ENSG00000188219	0	0	0	0
ENSG00000188223	0	0	0	0
ENSG00000188227	0	0	1	0
ENSG00000188229	1071	2544	1864	2574
ENSG00000188234	0	0	0	0
ENSG00000188242	2	0	0	0
ENSG00000188243	395	44	59	139
ENSG00000188257	0	0	0	0
ENSG00000188263	0	0	0	0
ENSG00000188266	0	0	5	3
ENSG00000188269	0	0	0	0
ENSG00000188277	0	0	0	0
ENSG00000188279	0	0	0	0
ENSG00000188280	0	0	0	1
ENSG00000188282	0	0	20	0
ENSG00000188283	4	0	33	14
ENSG00000188290	0	0	0	0
ENSG00000188293	0	0	0	0
ENSG00000188295	2	0	4	0
ENSG00000188305	1	0	0	0
ENSG00000188306	1	0	1	0
ENSG00000188312	1	147	151	182
ENSG00000188313	22	261	323	235
ENSG00000188314	0	0	0	0
ENSG00000188315	15	15	23	8
ENSG00000188316	0	0	0	0
ENSG00000188321	14	153	80	118

ENSG00000188322	3	0	0	0
ENSG00000188324	0	0	0	0
ENSG00000188334	0	0	0	0
ENSG00000188338	0	0	0	0
ENSG00000188340	0	0	0	0
ENSG00000188342	24	677	585	867
ENSG00000188343	9	217	135	248
ENSG00000188352	13	6701	4125	5102
ENSG00000188365	0	0	0	0
ENSG00000188368	0	0	0	0
ENSG00000188372	4	8	18	19
ENSG00000188373	0	0	0	0
ENSG00000188375	0	0	0	0
ENSG00000188379	0	0	0	0
ENSG00000188383	0	0	0	0
ENSG00000188384	0	0	0	0
ENSG00000188385	0	0	0	0
ENSG00000188386	0	0	0	0
ENSG00000188388	0	0	0	0
ENSG00000188389	0	0	0	0
ENSG00000188393	0	0	0	0
ENSG00000188394	1	9	5	5
ENSG00000188396	0	0	0	0
ENSG00000188399	0	0	0	0
ENSG00000188403	0	0	0	0
ENSG00000188404	109	312	396	287
ENSG00000188408	0	0	0	0
ENSG00000188419	27	389	326	238
ENSG00000188424	0	1	0	3
ENSG00000188425	0	0	0	0
ENSG00000188428	99	709	587	645
ENSG00000188438	0	0	0	0
ENSG00000188439	0	0	0	0
ENSG00000188451	0	0	0	0
ENSG00000188452	11	0	0	0
ENSG00000188459	1	0	0	3
ENSG00000188460	0	0	0	0
ENSG00000188467	0	0	0	0
ENSG00000188474	0	0	0	0
ENSG00000188477	0	0	0	0
ENSG00000188483	4	10	1	0
ENSG00000188486	0	0	1	5
ENSG00000188487	0	0	0	0
ENSG00000188488	0	0	0	0
ENSG00000188493	5	0	0	1
ENSG00000188501	0	0	0	0

ENSG00000188505	2	0	0	0
ENSG00000188508	0	0	0	0
ENSG00000188511	1	0	5	6
ENSG00000188512	0	0	0	0
ENSG00000188517	9	42	92	63
ENSG00000188522	1	0	0	0
ENSG00000188523	0	0	0	0
ENSG00000188525	1	0	0	0
ENSG00000188529	57	209	193	290
ENSG00000188536	3293	33	14	25
ENSG00000188542	102	322	133	203
ENSG00000188549	63	539	221	361
ENSG00000188554	414	1772	1637	1874
ENSG00000188558	0	0	0	0
ENSG00000188559	5	96	248	399
ENSG00000188566	0	5	5	0
ENSG00000188573	0	0	0	0
ENSG00000188580	2	62	128	107
ENSG00000188581	0	0	0	0
ENSG00000188582	1	0	0	0
ENSG00000188585	0	0	0	0
ENSG00000188596	6	92	113	109
ENSG00000188599	8	271	334	303
ENSG00000188603	1	9	0	0
ENSG00000188610	0	0	2	6
ENSG00000188611	0	15	0	2
ENSG00000188612	1134	4995	4311	4667
ENSG00000188613	9	0	11	1
ENSG00000188620	0	0	0	0
ENSG00000188624	0	0	0	0
ENSG00000188626	3	0	8	2
ENSG00000188629	0	0	0	0
ENSG00000188636	1	0	0	0
ENSG00000188641	312	9256	14062	8373
ENSG00000188643	10	12	0	5
ENSG00000188646	0	0	0	0
ENSG00000188647	13	153	124	152
ENSG00000188649	0	0	8	7
ENSG00000188655	0	0	0	0
ENSG00000188656	0	0	0	0
ENSG00000188659	11	0	0	0
ENSG00000188660	0	0	0	0
ENSG00000188662	0	0	0	0
ENSG00000188668	0	0	0	0
ENSG00000188672	6	68	26	138
ENSG00000188674	0	0	0	0

ENSG00000188676	0	2	0	0
ENSG00000188677	1744	3954	3016	7574
ENSG00000188681	39	0	4	17
ENSG00000188686	0	0	0	0
ENSG00000188687	3	24	9	2
ENSG00000188690	24	125	165	169
ENSG00000188691	0	0	0	0
ENSG00000188693	0	0	0	0
ENSG00000188694	0	0	0	0
ENSG00000188706	0	0	11	20
ENSG00000188707	0	0	0	1
ENSG00000188710	0	0	0	0
ENSG00000188712	6	0	3	0
ENSG00000188716	0	0	0	0
ENSG00000188722	0	0	0	0
ENSG00000188725	15	91	87	104
ENSG00000188729	0	0	0	0
ENSG00000188730	0	0	0	0
ENSG00000188732	0	18	38	39
ENSG00000188735	39	51	73	73
ENSG00000188738	24	43	63	137
ENSG00000188739	2	0	0	4
ENSG00000188740	0	0	0	0
ENSG00000188747	0	0	0	0
ENSG00000188755	0	0	0	0
ENSG00000188760	0	0	0	0
ENSG00000188761	2	0	0	0
ENSG00000188763	0	0	0	0
ENSG00000188765	8	10	11	24
ENSG00000188766	0	2	0	0
ENSG00000188770	0	0	0	0
ENSG00000188771	0	0	0	0
ENSG00000188778	0	0	0	0
ENSG00000188779	0	0	0	8
ENSG00000188782	0	16	0	0
ENSG00000188783	0	0	0	0
ENSG00000188784	0	0	0	0
ENSG00000188785	0	0	12	17
ENSG00000188786	13	110	126	225
ENSG00000188800	4	0	11	13
ENSG00000188801	0	0	0	0
ENSG00000188803	0	0	0	0
ENSG00000188807	1	0	0	0
ENSG00000188811	2	90	14	17
ENSG00000188816	0	0	0	0
ENSG00000188817	0	0	0	0

ENSG00000188818	0	0	0	0
ENSG00000188820	27	1	30	30
ENSG00000188822	2	0	0	0
ENSG00000188825	1	14	9	24
ENSG00000188827	85	383	206	128
ENSG00000188828	5	3	28	26
ENSG00000188831	0	0	0	0
ENSG00000188833	0	0	0	0
ENSG00000188846	1190	1215	573	1009
ENSG00000188848	0	0	2	4
ENSG00000188850	0	0	0	0
ENSG00000188856	0	0	0	0
ENSG00000188859	0	0	0	0
ENSG00000188868	4	0	26	23
ENSG00000188869	0	0	0	0
ENSG00000188873	0	0	0	0
ENSG00000188877	0	0	0	0
ENSG00000188878	0	0	6	11
ENSG00000188883	0	0	0	0
ENSG00000188886	0	0	0	0
ENSG00000188888	0	0	0	0
ENSG00000188895	160	682	342	277
ENSG00000188897	1	0	2	1
ENSG00000188906	23	60	52	118
ENSG00000188909	0	0	0	0
ENSG00000188910	0	0	0	0
ENSG00000188916	11	14	53	40
ENSG00000188917	17	173	107	176
ENSG00000188921	3742	18394	13322	12422
ENSG00000188931	0	0	3	0
ENSG00000188933	0	0	0	4
ENSG00000188937	0	0	0	0
ENSG00000188938	48	41	101	132
ENSG00000188958	0	0	0	0
ENSG00000188959	0	0	0	0
ENSG00000188971	7	8	14	5
ENSG00000188976	0	0	7	7
ENSG00000188981	0	0	0	1
ENSG00000188984	0	0	0	0
ENSG00000188985	0	0	0	1
ENSG00000188986	17	140	73	141
ENSG00000188987	5	0	13	16
ENSG00000188991	0	0	0	0
ENSG00000188992	0	0	0	0
ENSG00000188993	0	0	0	0
ENSG00000188994	31	9940	10290	12066

ENSG00000188996	0	0	0	0
ENSG00000188997	1	18	2	0
ENSG00000189001	0	0	0	0
ENSG00000189002	0	0	1	3
ENSG00000189007	73	331	259	230
ENSG00000189011	0	135	24	44
ENSG00000189013	0	0	0	0
ENSG00000189014	0	0	1	0
ENSG00000189023	0	0	0	0
ENSG00000189030	0	0	0	0
ENSG00000189037	0	0	0	0
ENSG00000189042	4	62	71	145
ENSG00000189043	2882	1316	926	1791
ENSG00000189045	343	936	772	448
ENSG00000189046	7	0	0	0
ENSG00000189050	16	165	188	76
ENSG00000189051	0	0	0	0
ENSG00000189052	0	0	0	0
ENSG00000189056	0	0	0	5
ENSG00000189057	1	18	22	15
ENSG00000189058	2	0	0	4
ENSG00000189060	624	2300	1558	1751
ENSG00000189064	0	0	0	0
ENSG00000189067	212	59	31	9
ENSG00000189068	1	11	0	0
ENSG00000189077	4	0	5	3
ENSG00000189079	102	2539	2885	2679
ENSG00000189089	0	0	0	0
ENSG00000189090	0	0	0	0
ENSG00000189091	26	534	391	400
ENSG00000189093	0	0	0	0
ENSG00000189099	0	0	0	0
ENSG00000189108	0	0	0	0
ENSG00000189114	14	0	0	9
ENSG00000189120	0	0	0	0
ENSG00000189127	0	0	0	0
ENSG00000189129	0	0	0	0
ENSG00000189132	0	0	0	0
ENSG00000189134	4	0	3	12
ENSG00000189136	124	1225	1157	1082
ENSG00000189139	0	0	2	0
ENSG00000189143	0	0	0	0
ENSG00000189144	3	40	72	104
ENSG00000189145	0	0	0	0
ENSG00000189149	0	210	115	240
ENSG00000189152	0	0	0	0

ENSG00000189157	0	0	0	0
ENSG00000189159	69	31	48	56
ENSG00000189164	0	0	0	0
ENSG00000189166	0	0	0	0
ENSG00000189167	0	0	0	0
ENSG00000189169	0	0	0	0
ENSG00000189171	19	73	32	55
ENSG00000189180	224	1199	1473	1738
ENSG00000189181	0	0	0	0
ENSG00000189182	0	0	0	0
ENSG00000189184	0	0	0	0
ENSG00000189186	0	0	0	0
ENSG00000189190	4	9	9	7
ENSG00000189195	0	162	134	171
ENSG00000189196	1	3	1	2
ENSG00000189212	5	48	85	104
ENSG00000189221	0	0	2	0
ENSG00000189223	27	37	228	339
ENSG00000189227	2	0	0	2
ENSG00000189229	0	45	33	117
ENSG00000189233	2	0	0	0
ENSG00000189238	0	0	0	0
ENSG00000189241	97	415	283	390
ENSG00000189252	0	0	0	0
ENSG00000189253	0	0	0	0
ENSG00000189266	9	20	66	37
ENSG00000189269	0	0	2	12
ENSG00000189275	0	0	0	0
ENSG00000189280	1	0	1	2
ENSG00000189283	12	13	49	34
ENSG00000189292	35	5	13	17
ENSG00000189295	2	0	0	0
ENSG00000189298	0	0	1	5
ENSG00000189299	0	0	0	0
ENSG00000189306	21	0	0	5
ENSG00000189308	118	3609	4163	5404
ENSG00000189316	0	0	1	0
ENSG00000189319	3	3	0	5
ENSG00000189320	0	0	0	0
ENSG00000189325	0	0	0	0
ENSG00000189326	63	76	104	26
ENSG00000189332	0	0	1	0
ENSG00000189334	0	0	0	0
ENSG00000189337	6	33	38	91
ENSG00000189339	51	128	128	116
ENSG00000189343	2	0	0	0

ENSG00000189348	0	0	0	0
ENSG00000189350	0	0	0	0
ENSG00000189357	0	0	0	0
ENSG00000189362	8	84	99	150
ENSG00000189366	1	0	0	0
ENSG00000189367	0	0	0	0
ENSG00000189369	3	0	0	7
ENSG00000189372	0	0	0	0
ENSG00000189375	0	0	0	0
ENSG00000189376	7	0	26	9
ENSG00000189377	0	0	0	0
ENSG00000189393	0	0	0	0
ENSG00000189398	0	0	0	0
ENSG00000189401	0	0	0	0
ENSG00000189403	27141	40477	55125	51015
ENSG00000189409	0	0	0	0
ENSG00000189410	0	0	0	0
ENSG00000189419	0	5	0	0
ENSG00000189420	0	0	0	0
ENSG00000189423	0	31	5	9
ENSG00000189430	2	0	8	8
ENSG00000189431	0	0	0	0
ENSG00000189433	1	0	0	0
ENSG00000194270	0	0	0	0
ENSG00000194297	0	0	0	0
ENSG00000194647	0	0	0	0
ENSG00000194717	0	0	0	0
ENSG00000195024	0	0	0	0
ENSG00000195401	0	0	0	0
ENSG00000196071	14	126	301	164
ENSG00000196072	28	86	128	127
ENSG00000196074	12	132	177	178
ENSG00000196081	2	0	33	44
ENSG00000196082	0	0	0	0
ENSG00000196083	1	35	20	6
ENSG00000196085	0	0	0	0
ENSG00000196090	0	0	0	0
ENSG00000196091	1	0	2	5
ENSG00000196092	15	0	0	0
ENSG00000196095	0	0	0	2
ENSG00000196096	0	0	0	0
ENSG00000196098	0	0	0	0
ENSG00000196099	0	0	0	0
ENSG00000196101	0	0	0	0
ENSG00000196104	0	0	2	5
ENSG00000196109	0	0	0	0

ENSG00000196110	0	7	3	11
ENSG00000196114	0	0	0	0
ENSG00000196115	0	0	2	10
ENSG00000196116	5	49	54	119
ENSG00000196118	1	0	7	2
ENSG00000196119	0	0	0	0
ENSG00000196123	1	0	0	1
ENSG00000196126	19	0	1	0
ENSG00000196131	0	0	0	0
ENSG00000196132	0	0	0	0
ENSG00000196136	0	0	0	0
ENSG00000196138	1	0	2	0
ENSG00000196139	25	9	8	7
ENSG00000196141	8	88	61	68
ENSG00000196143	0	0	0	2
ENSG00000196150	17	86	26	12
ENSG00000196151	13	175	342	417
ENSG00000196152	2	192	83	118
ENSG00000196154	1137	43	29	42
ENSG00000196155	0	0	0	0
ENSG00000196156	0	0	0	0
ENSG00000196159	1	39	79	56
ENSG00000196166	0	0	0	0
ENSG00000196167	1	0	0	0
ENSG00000196169	0	0	0	0
ENSG00000196171	0	0	0	0
ENSG00000196172	4	47	86	104
ENSG00000196176	29	30	21	41
ENSG00000196177	8	320	156	380
ENSG00000196182	689	3500	1785	3497
ENSG00000196183	0	0	0	0
ENSG00000196184	0	0	0	0
ENSG00000196187	63	696	306	391
ENSG00000196188	1	14	0	4
ENSG00000196189	11	12	25	16
ENSG00000196193	0	0	0	0
ENSG00000196196	0	0	0	0
ENSG00000196199	153	271	276	367
ENSG00000196204	11	32	11	18
ENSG00000196205	3	85	165	174
ENSG00000196208	1	0	0	0
ENSG00000196209	4	0	1	0
ENSG00000196214	15	489	490	699
ENSG00000196218	0	0	5	6
ENSG00000196220	1	0	0	2
ENSG00000196224	0	0	0	0

ENSG00000196226	48	731	829	400
ENSG00000196227	55	130	65	140
ENSG00000196228	0	0	0	0
ENSG00000196230	1122	2853	2309	4597
ENSG00000196231	0	0	0	0
ENSG00000196233	153	3147	3486	3495
ENSG00000196235	80	69	78	151
ENSG00000196236	114	248	318	478
ENSG00000196240	0	0	0	0
ENSG00000196242	0	0	0	0
ENSG00000196243	0	0	0	0
ENSG00000196247	3	34	27	39
ENSG00000196248	0	0	0	0
ENSG00000196260	0	0	0	0
ENSG00000196262	3657	7515	6265	11819
ENSG00000196263	2	12	20	22
ENSG00000196266	0	0	0	0
ENSG00000196267	2	17	8	13
ENSG00000196268	14	18	67	48
ENSG00000196273	2	0	0	1
ENSG00000196274	0	0	0	0
ENSG00000196275	1	0	2	0
ENSG00000196277	0	0	0	0
ENSG00000196284	7	270	143	134
ENSG00000196289	0	0	0	0
ENSG00000196290	3	0	7	23
ENSG00000196295	13	639	739	1158
ENSG00000196296	2	0	0	2
ENSG00000196299	0	0	0	0
ENSG00000196301	0	0	0	0
ENSG00000196302	0	0	0	0
ENSG00000196305	10	171	186	244
ENSG00000196312	1	24	1	2
ENSG00000196313	6	168	195	355
ENSG00000196323	74	455	474	403
ENSG00000196326	0	195	29	259
ENSG00000196329	32	0	0	8
ENSG00000196331	143	2332	2481	778
ENSG00000196335	0	107	90	88
ENSG00000196337	0	0	0	0
ENSG00000196338	0	0	0	0
ENSG00000196341	0	0	0	0
ENSG00000196344	0	0	0	0
ENSG00000196345	7	0	3	3
ENSG00000196350	0	0	0	0
ENSG00000196352	253	901	1291	1302

ENSG00000196353	17	479	178	226
ENSG00000196355	0	0	24	2
ENSG00000196357	1	9	5	10
ENSG00000196358	2	0	0	0
ENSG00000196361	0	0	0	0
ENSG00000196363	4	37	22	25
ENSG00000196364	0	0	0	0
ENSG00000196365	1	4	11	5
ENSG00000196366	0	0	0	0
ENSG00000196367	11	895	460	816
ENSG00000196368	0	0	0	0
ENSG00000196369	0	63	81	118
ENSG00000196371	0	0	3	5
ENSG00000196372	2	0	0	0
ENSG00000196374	2	47	5	10
ENSG00000196376	0	0	2	0
ENSG00000196378	2	0	2	0
ENSG00000196381	0	0	2	0
ENSG00000196383	0	0	1	0
ENSG00000196387	4	70	13	17
ENSG00000196388	0	0	1	0
ENSG00000196390	0	0	0	0
ENSG00000196391	0	0	0	0
ENSG00000196395	0	0	0	0
ENSG00000196396	152	537	370	298
ENSG00000196400	0	0	0	0
ENSG00000196403	0	0	0	0
ENSG00000196405	478	1484	371	719
ENSG00000196406	0	0	0	0
ENSG00000196407	408	285	206	207
ENSG00000196408	0	0	0	0
ENSG00000196409	0	0	2	0
ENSG00000196411	0	0	5	1
ENSG00000196415	0	0	0	0
ENSG00000196417	3	76	67	145
ENSG00000196418	3	2059	1910	4633
ENSG00000196419	222	650	454	757
ENSG00000196420	0	0	0	0
ENSG00000196421	0	0	0	0
ENSG00000196422	2	0	8	2
ENSG00000196427	0	0	0	0
ENSG00000196428	10	44	74	73
ENSG00000196431	2	0	6	3
ENSG00000196433	0	4	0	0
ENSG00000196436	0	0	2	0
ENSG00000196437	0	233	381	627

ENSG00000196440	0	199	174	138
ENSG00000196449	2	0	5	0
ENSG00000196453	0	0	0	2
ENSG00000196455	0	160	119	182
ENSG00000196456	3	0	0	3
ENSG00000196458	1	0	17	56
ENSG00000196459	795	11229	11631	8433
ENSG00000196460	0	0	0	0
ENSG00000196465	24	37	16	42
ENSG00000196466	0	0	0	1
ENSG00000196468	0	0	0	0
ENSG00000196470	26	237	134	63
ENSG00000196472	0	0	0	0
ENSG00000196475	0	0	0	0
ENSG00000196476	45	47	32	79
ENSG00000196482	0	0	0	4
ENSG00000196497	0	0	0	0
ENSG00000196498	56	562	300	571
ENSG00000196502	25	160	21	29
ENSG00000196503	0	0	0	0
ENSG00000196504	228	881	790	1070
ENSG00000196505	5	680	427	571
ENSG00000196507	100	157	87	154
ENSG00000196510	5	50	34	66
ENSG00000196511	6	11	32	43
ENSG00000196517	0	7	0	6
ENSG00000196526	810	2417	2007	1712
ENSG00000196531	979	1147	883	1793
ENSG00000196532	13	59	27	26
ENSG00000196533	4	0	0	1
ENSG00000196534	0	0	0	0
ENSG00000196535	13	157	22	66
ENSG00000196539	0	0	0	0
ENSG00000196542	0	0	0	0
ENSG00000196544	206	111	116	197
ENSG00000196547	177	1865	1264	2177
ENSG00000196549	0	0	14	4
ENSG00000196550	0	0	5	6
ENSG00000196553	0	0	0	0
ENSG00000196557	0	0	0	0
ENSG00000196559	0	0	0	0
ENSG00000196562	4	10	7	2
ENSG00000196564	0	0	0	0
ENSG00000196565	5935	2799	129	231
ENSG00000196566	0	0	0	0
ENSG00000196569	4	17	16	9

ENSG00000196570	0	0	0	0
ENSG00000196576	1	42	3	18
ENSG00000196578	0	0	0	0
ENSG00000196581	0	0	0	0
ENSG00000196584	69	607	553	301
ENSG00000196586	7	65	32	62
ENSG00000196588	19	337	227	618
ENSG00000196589	0	0	0	0
ENSG00000196591	98	386	549	470
ENSG00000196593	0	0	0	0
ENSG00000196597	0	96	231	338
ENSG00000196600	0	0	0	0
ENSG00000196604	0	0	0	0
ENSG00000196605	36	47	35	36
ENSG00000196610	0	0	0	0
ENSG00000196611	38	540	215	435
ENSG00000196616	0	0	0	0
ENSG00000196620	0	0	0	0
ENSG00000196622	0	0	0	0
ENSG00000196628	290	659	569	220
ENSG00000196632	0	29	5	11
ENSG00000196634	1	0	0	0
ENSG00000196636	13	1	20	49
ENSG00000196639	1	0	0	0
ENSG00000196642	14	0	18	60
ENSG00000196644	0	0	0	0
ENSG00000196646	1	0	13	17
ENSG00000196648	0	8	0	0
ENSG00000196652	5	8	30	56
ENSG00000196653	1	0	0	0
ENSG00000196655	37	75	74	63
ENSG00000196656	2	0	0	2
ENSG00000196659	2	35	23	14
ENSG00000196660	0	0	0	2
ENSG00000196661	0	0	0	0
ENSG00000196663	293	952	187	380
ENSG00000196664	1	14	0	0
ENSG00000196666	0	0	0	0
ENSG00000196668	502	79	251	124
ENSG00000196670	4	10	16	29
ENSG00000196674	0	0	0	0
ENSG00000196678	7	314	374	172
ENSG00000196683	479	105	311	346
ENSG00000196684	1	0	0	0
ENSG00000196689	0	0	0	0
ENSG00000196693	126	585	434	483

ENSG00000196696	0	0	0	0
ENSG00000196700	0	0	0	1
ENSG00000196704	131	413	493	506
ENSG00000196705	68	589	931	785
ENSG00000196711	0	0	1	0
ENSG00000196712	14	1393	1249	1541
ENSG00000196715	556	2385	2390	1825
ENSG00000196724	2	166	126	215
ENSG00000196730	11	72	45	47
ENSG00000196734	0	0	0	0
ENSG00000196735	5	0	1	0
ENSG00000196737	0	0	0	0
ENSG00000196739	0	0	0	0
ENSG00000196741	3	0	16	12
ENSG00000196743	5	0	0	4
ENSG00000196747	3	49	280	115
ENSG00000196748	0	0	0	0
ENSG00000196754	0	0	0	0
ENSG00000196756	73	23	10	1
ENSG00000196757	1	0	10	10
ENSG00000196758	0	0	0	0
ENSG00000196767	0	0	0	0
ENSG00000196772	0	0	0	0
ENSG00000196774	0	0	0	2
ENSG00000196776	696	10507	10766	12419
ENSG00000196777	0	0	0	0
ENSG00000196778	0	0	0	0
ENSG00000196781	13	19	16	10
ENSG00000196782	250	974	1258	1124
ENSG00000196787	677	4446	6664	4745
ENSG00000196792	129	12525	13332	15377
ENSG00000196793	0	0	1	0
ENSG00000196796	0	0	0	0
ENSG00000196800	0	0	0	0
ENSG00000196805	0	0	0	0
ENSG00000196810	8	0	9	12
ENSG00000196811	0	0	0	0
ENSG00000196812	2	0	3	4
ENSG00000196814	16	276	212	299
ENSG00000196821	383	1952	1202	2027
ENSG00000196826	0	0	0	0
ENSG00000196832	0	0	0	0
ENSG00000196834	0	0	0	0
ENSG00000196839	24	0	0	0
ENSG00000196843	13	81	36	30
ENSG00000196844	0	0	0	0

ENSG00000196850	14	36	70	50
ENSG00000196859	0	0	0	0
ENSG00000196860	0	0	3	10
ENSG00000196861	0	0	0	0
ENSG00000196862	0	0	2	9
ENSG00000196865	40	3531	2618	3943
ENSG00000196866	0	0	0	0
ENSG00000196867	1	34	27	38
ENSG00000196872	0	0	0	0
ENSG00000196873	0	13	10	15
ENSG00000196876	1	0	2	0
ENSG00000196878	0	0	0	0
ENSG00000196890	0	0	0	0
ENSG00000196893	0	0	0	0
ENSG00000196900	0	0	0	0
ENSG00000196911	38	4372	5316	9876
ENSG00000196912	2	121	246	353
ENSG00000196914	1712	20593	23238	22148
ENSG00000196917	0	0	5	5
ENSG00000196922	11	33	26	47
ENSG00000196923	413	889	356	630
ENSG00000196924	1745	40169	20245	49690
ENSG00000196927	1	0	1	0
ENSG00000196932	0	0	0	2
ENSG00000196933	0	0	0	0
ENSG00000196934	0	0	0	0
ENSG00000196935	1	17	26	48
ENSG00000196936	0	0	0	0
ENSG00000196937	5	404	357	540
ENSG00000196943	1	0	7	0
ENSG00000196944	0	0	0	0
ENSG00000196946	0	0	0	0
ENSG00000196950	17	162	123	89
ENSG00000196951	7	52	18	25
ENSG00000196954	209	1452	2789	1507
ENSG00000196960	0	0	0	0
ENSG00000196961	11	82	39	101
ENSG00000196963	0	0	0	0
ENSG00000196966	3	22	8	10
ENSG00000196967	5	7	5	14
ENSG00000196968	7	15	22	49
ENSG00000196970	0	0	0	0
ENSG00000196972	0	0	0	0
ENSG00000196975	89	92	149	173
ENSG00000196976	24	2	1	6
ENSG00000196979	0	0	0	0

ENSG00000196981	3	24	20	39
ENSG00000196987	0	0	0	0
ENSG00000196990	0	0	0	0
ENSG00000196993	0	3	0	0
ENSG00000196998	74	14	26	23
ENSG00000197006	417	1439	1182	1685
ENSG00000197008	1	128	37	55
ENSG00000197013	180	450	546	328
ENSG00000197015	0	0	0	0
ENSG00000197016	9	95	92	97
ENSG00000197019	3	0	0	0
ENSG00000197020	5	170	277	236
ENSG00000197021	17	35	15	27
ENSG00000197023	0	0	0	0
ENSG00000197024	0	34	37	31
ENSG00000197037	3	0	18	14
ENSG00000197038	0	0	0	0
ENSG00000197043	103	30	7	6
ENSG00000197044	6	0	9	8
ENSG00000197045	14	316	333	523
ENSG00000197046	0	0	0	0
ENSG00000197047	0	0	0	0
ENSG00000197050	4	17	20	26
ENSG00000197054	0	0	0	0
ENSG00000197056	2	668	415	652
ENSG00000197057	5	0	0	0
ENSG00000197061	759	184	198	185
ENSG00000197062	2	0	24	23
ENSG00000197063	129	426	310	435
ENSG00000197067	0	0	0	0
ENSG00000197070	2	0	8	9
ENSG00000197071	0	0	0	0
ENSG00000197077	2	1	0	0
ENSG00000197079	0	0	0	0
ENSG00000197081	2	8	9	18
ENSG00000197083	0	0	1	0
ENSG00000197084	0	0	0	0
ENSG00000197085	0	0	0	0
ENSG00000197092	0	0	0	0
ENSG00000197093	2	0	0	0
ENSG00000197099	0	0	0	0
ENSG00000197102	35	617	414	814
ENSG00000197106	1	0	0	0
ENSG00000197110	0	0	0	0
ENSG00000197111	735	1056	755	1020
ENSG00000197114	0	29	0	2

ENSG00000197119	0	0	0	1
ENSG00000197121	1	173	138	174
ENSG00000197122	164	2162	1153	1647
ENSG00000197123	0	0	0	0
ENSG00000197124	3	15	15	18
ENSG00000197125	0	0	0	0
ENSG00000197128	1	7	17	53
ENSG00000197134	2	208	291	478
ENSG00000197136	2	13	2	5
ENSG00000197140	0	1686	1547	1289
ENSG00000197142	6	75	88	129
ENSG00000197146	0	0	0	0
ENSG00000197147	587	5056	7137	4986
ENSG00000197149	0	0	3	7
ENSG00000197150	1	0	1	0
ENSG00000197153	15	38	39	24
ENSG00000197157	42	237	148	287
ENSG00000197161	0	0	0	0
ENSG00000197162	1	0	0	10
ENSG00000197165	2	1	0	4
ENSG00000197168	1	50	7	11
ENSG00000197170	26	125	173	194
ENSG00000197171	0	0	0	0
ENSG00000197172	0	0	0	0
ENSG00000197176	0	0	0	0
ENSG00000197177	0	0	0	0
ENSG00000197180	15	5	0	9
ENSG00000197181	0	0	4	0
ENSG00000197182	1	20	0	7
ENSG00000197183	136	302	316	346
ENSG00000197185	0	0	0	0
ENSG00000197191	0	0	0	0
ENSG00000197208	0	0	0	0
ENSG00000197210	0	0	0	0
ENSG00000197213	0	0	0	1
ENSG00000197214	0	0	0	0
ENSG00000197217	15	115	176	158
ENSG00000197223	132	306	214	250
ENSG00000197226	130	498	318	585
ENSG00000197233	0	0	0	0
ENSG00000197238	5	0	9	19
ENSG00000197241	0	0	0	0
ENSG00000197245	0	0	0	0
ENSG00000197249	41	0	1	1
ENSG00000197251	0	0	0	0
ENSG00000197253	0	0	0	0

ENSG00000197254	0	0	0	0
ENSG00000197256	18	14	23	23
ENSG00000197258	1	15	4	0
ENSG00000197261	0	0	0	0
ENSG00000197262	0	0	0	0
ENSG00000197263	0	0	0	0
ENSG00000197265	5	97	73	226
ENSG00000197272	0	0	0	0
ENSG00000197273	0	0	0	0
ENSG00000197275	0	0	29	27
ENSG00000197279	1	0	0	0
ENSG00000197283	1	0	9	15
ENSG00000197284	0	0	0	0
ENSG00000197291	0	0	0	4
ENSG00000197296	0	0	0	0
ENSG00000197299	4	119	201	348
ENSG00000197301	0	0	9	28
ENSG00000197302	34	126	205	327
ENSG00000197308	0	0	0	0
ENSG00000197309	0	0	0	0
ENSG00000197312	183	464	530	818
ENSG00000197320	0	0	0	0
ENSG00000197321	109	1071	686	776
ENSG00000197322	0	0	0	0
ENSG00000197323	38	3525	3410	5424
ENSG00000197324	333	578	492	586
ENSG00000197329	10	57	119	106
ENSG00000197332	0	0	0	0
ENSG00000197334	0	0	0	0
ENSG00000197343	114	170	237	280
ENSG00000197345	25	0	1	0
ENSG00000197353	4	0	0	0
ENSG00000197355	0	0	0	0
ENSG00000197358	20	18	35	145
ENSG00000197360	0	0	0	3
ENSG00000197361	0	0	0	0
ENSG00000197362	2	73	51	90
ENSG00000197363	8	0	1	0
ENSG00000197364	0	0	0	0
ENSG00000197372	4	159	220	429
ENSG00000197375	0	0	0	4
ENSG00000197376	0	0	0	0
ENSG00000197380	1	0	0	0
ENSG00000197381	26	622	636	835
ENSG00000197385	0	0	0	0
ENSG00000197386	165	2756	1878	3175

ENSG00000197403	0	0	0	0
ENSG00000197405	7	6	2	1
ENSG00000197406	0	0	0	0
ENSG00000197408	0	0	0	0
ENSG00000197409	41	100	58	42
ENSG00000197410	0	0	0	0
ENSG00000197414	0	0	0	0
ENSG00000197415	1055	6480	5130	8920
ENSG00000197416	0	0	0	0
ENSG00000197417	6	0	0	0
ENSG00000197421	0	0	0	0
ENSG00000197426	0	0	0	0
ENSG00000197428	0	0	0	0
ENSG00000197429	21	106	44	64
ENSG00000197430	0	0	0	0
ENSG00000197437	0	0	0	0
ENSG00000197442	422	19737	17163	24223
ENSG00000197444	0	0	0	0
ENSG00000197445	7	0	0	0
ENSG00000197446	25	16	10	15
ENSG00000197448	316	678	486	1064
ENSG00000197451	40	248	130	218
ENSG00000197454	0	0	0	0
ENSG00000197457	0	0	0	0
ENSG00000197459	924	5998	6105	6530
ENSG00000197461	1103	4279	3625	2978
ENSG00000197462	2	0	0	3
ENSG00000197463	0	0	0	0
ENSG00000197465	0	0	13	6
ENSG00000197467	0	0	0	0
ENSG00000197468	0	0	0	0
ENSG00000197471	14	0	6	8
ENSG00000197472	0	0	3	9
ENSG00000197475	0	0	0	2
ENSG00000197476	0	0	0	0
ENSG00000197479	0	0	0	0
ENSG00000197483	0	0	0	0
ENSG00000197487	0	0	0	0
ENSG00000197496	0	0	3	0
ENSG00000197497	17	31	24	17
ENSG00000197498	22	41	97	82
ENSG00000197502	0	0	0	0
ENSG00000197503	0	0	0	4
ENSG00000197506	0	0	2	0
ENSG00000197520	0	0	0	0
ENSG00000197530	12	30	4	6

ENSG00000197532	0	0	0	0
ENSG00000197535	97	2375	2591	2317
ENSG00000197536	12	21	9	17
ENSG00000197540	4	0	0	0
ENSG00000197548	13	91	170	191
ENSG00000197549	0	23	0	0
ENSG00000197550	0	0	36	30
ENSG00000197555	15	292	172	381
ENSG00000197557	6	76	256	162
ENSG00000197558	0	0	0	0
ENSG00000197561	0	0	0	2
ENSG00000197562	0	0	0	5
ENSG00000197563	12	6812	5705	7499
ENSG00000197565	1	0	0	0
ENSG00000197566	2	14	25	28
ENSG00000197568	12	23	2	6
ENSG00000197575	0	0	0	0
ENSG00000197576	0	0	0	0
ENSG00000197579	27	39	58	62
ENSG00000197580	1	0	1	5
ENSG00000197582	13	1	775	1540
ENSG00000197584	1	0	0	0
ENSG00000197585	0	0	0	0
ENSG00000197586	11	36	49	87
ENSG00000197587	0	0	0	0
ENSG00000197588	0	0	0	0
ENSG00000197591	0	6	0	0
ENSG00000197594	0	0	0	0
ENSG00000197595	0	0	0	0
ENSG00000197599	0	0	0	0
ENSG00000197601	42	1324	1486	1712
ENSG00000197603	19	2855	1795	2877
ENSG00000197604	0	0	0	0
ENSG00000197608	1	42	51	55
ENSG00000197614	6	0	0	3
ENSG00000197616	3	0	0	1
ENSG00000197617	0	0	0	0
ENSG00000197619	4	78	32	63
ENSG00000197620	11	24	20	18
ENSG00000197622	58	706	443	576
ENSG00000197627	0	0	0	0
ENSG00000197629	31	0	17	2
ENSG00000197632	1	0	0	0
ENSG00000197635	1	0	3	0
ENSG00000197641	0	0	0	0
ENSG00000197644	0	0	0	0

ENSG00000197646	7	47	59	91
ENSG00000197647	1	0	1	0
ENSG00000197651	0	0	0	0
ENSG00000197653	53	4	10	33
ENSG00000197658	0	0	0	0
ENSG00000197665	0	0	0	0
ENSG00000197670	0	0	1	0
ENSG00000197674	0	0	0	0
ENSG00000197681	0	0	0	0
ENSG00000197683	0	0	0	0
ENSG00000197692	0	0	0	0
ENSG00000197694	43	245	84	245
ENSG00000197696	1	0	0	0
ENSG00000197697	7	4	83	55
ENSG00000197701	1	0	12	9
ENSG00000197702	0	42	0	0
ENSG00000197704	0	0	0	0
ENSG00000197705	3	0	0	0
ENSG00000197706	0	0	0	0
ENSG00000197712	18	83	105	188
ENSG00000197713	5	114	133	202
ENSG00000197714	0	43	22	18
ENSG00000197721	78	877	510	446
ENSG00000197723	0	0	0	0
ENSG00000197724	15	129	101	160
ENSG00000197727	0	0	0	0
ENSG00000197728	2174	40	75	63
ENSG00000197734	0	0	0	0
ENSG00000197744	83	63	35	112
ENSG00000197745	3	0	0	0
ENSG00000197746	4247	9886	9791	15784
ENSG00000197747	250	62	69	47
ENSG00000197748	0	35	21	38
ENSG00000197753	0	0	1	0
ENSG00000197756	1632	336	165	600
ENSG00000197757	0	0	0	0
ENSG00000197763	0	0	13	42
ENSG00000197766	2	0	5	0
ENSG00000197768	0	0	0	0
ENSG00000197769	5	0	0	0
ENSG00000197771	27	117	64	89
ENSG00000197774	2	16	5	0
ENSG00000197775	0	0	13	8
ENSG00000197776	9	7669	9364	12224
ENSG00000197779	43	249	230	303
ENSG00000197780	19	202	76	117

ENSG00000197782	3	31	53	58
ENSG00000197785	1	0	0	0
ENSG00000197786	0	0	0	0
ENSG00000197790	0	0	0	0
ENSG00000197794	0	0	0	0
ENSG00000197798	39	449	312	296
ENSG00000197805	0	2	0	0
ENSG00000197808	9	51	20	38
ENSG00000197813	0	0	18	14
ENSG00000197815	0	0	0	0
ENSG00000197816	0	0	1	1
ENSG00000197818	1	87	125	174
ENSG00000197822	4	35	0	2
ENSG00000197825	0	0	0	0
ENSG00000197826	0	0	0	0
ENSG00000197830	1	0	0	0
ENSG00000197837	22	3	178	38
ENSG00000197838	0	0	0	0
ENSG00000197841	3	41	7	22
ENSG00000197844	0	0	0	1
ENSG00000197846	220	10751	8701	6245
ENSG00000197847	0	0	0	0
ENSG00000197848	0	0	0	0
ENSG00000197849	0	0	0	0
ENSG00000197851	0	0	0	0
ENSG00000197852	363	584	483	167
ENSG00000197857	4	99	290	328
ENSG00000197858	39	112	31	86
ENSG00000197859	0	0	0	0
ENSG00000197860	27	576	433	644
ENSG00000197863	1	135	77	98
ENSG00000197866	0	0	0	0
ENSG00000197870	0	0	0	0
ENSG00000197872	24	57	5	0
ENSG00000197879	427	972	796	863
ENSG00000197880	1	0	0	0
ENSG00000197882	0	0	0	0
ENSG00000197885	2	15	30	54
ENSG00000197887	0	0	0	0
ENSG00000197888	0	0	0	0
ENSG00000197889	0	0	1	0
ENSG00000197891	0	0	0	0
ENSG00000197892	4	371	223	435
ENSG00000197893	0	0	0	0
ENSG00000197894	117	818	545	749
ENSG00000197901	0	0	0	0

ENSG00000197903	7644	13887	17339	13600
ENSG00000197905	0	0	0	1
ENSG00000197910	0	0	0	0
ENSG00000197912	1	61	46	39
ENSG00000197914	9	2	5	0
ENSG00000197915	0	0	0	0
ENSG00000197919	0	0	0	0
ENSG00000197921	0	0	0	0
ENSG00000197927	0	0	1	2
ENSG00000197928	31	58	21	75
ENSG00000197930	0	4	30	38
ENSG00000197932	6	0	0	4
ENSG00000197933	0	545	332	652
ENSG00000197934	0	15	0	0
ENSG00000197935	0	21	15	0
ENSG00000197937	58	130	106	221
ENSG00000197938	0	0	0	0
ENSG00000197943	20	689	399	869
ENSG00000197948	2	16	6	8
ENSG00000197951	6	41	10	3
ENSG00000197953	0	0	2	0
ENSG00000197955	0	0	0	0
ENSG00000197956	749	27	44	45
ENSG00000197958	1369	292	201	530
ENSG00000197959	2680	35788	47936	51348
ENSG00000197961	5	443	372	592
ENSG00000197965	0	0	3	5
ENSG00000197969	276	25886	23811	37058
ENSG00000197971	2146	4330	5775	4209
ENSG00000197976	19	19	15	10
ENSG00000197977	0	0	0	0
ENSG00000197978	0	0	1	0
ENSG00000197980	0	0	3	2
ENSG00000197982	27	51	9	27
ENSG00000197984	0	0	0	0
ENSG00000197989	20	996	521	1104
ENSG00000197990	0	0	0	0
ENSG00000197991	3	18	23	5
ENSG00000197992	21	26	11	34
ENSG00000197993	6	5	8	7
ENSG00000198000	78	208	296	306
ENSG00000198001	108	1188	1181	1775
ENSG00000198003	1	0	0	0
ENSG00000198010	3	0	1	0
ENSG00000198015	10	93	124	96
ENSG00000198018	0	38	11	8

ENSG00000198019	0	0	0	0
ENSG00000198021	0	0	0	0
ENSG00000198022	0	0	0	0
ENSG00000198026	3	9	4	12
ENSG00000198028	0	0	0	0
ENSG00000198033	0	0	0	0
ENSG00000198034	1196	657	574	1191
ENSG00000198035	0	2	0	6
ENSG00000198039	5	187	133	110
ENSG00000198040	0	0	5	5
ENSG00000198042	3	0	25	49
ENSG00000198046	2	1	9	10
ENSG00000198049	0	0	0	0
ENSG00000198053	4	51	9	19
ENSG00000198054	0	0	0	0
ENSG00000198055	128	571	391	656
ENSG00000198056	8	835	615	679
ENSG00000198060	8	75	75	103
ENSG00000198062	0	0	0	0
ENSG00000198064	0	0	0	0
ENSG00000198074	0	0	21	16
ENSG00000198075	3	84	94	144
ENSG00000198077	0	0	0	0
ENSG00000198081	78	390	1027	626
ENSG00000198082	0	0	0	0
ENSG00000198083	0	0	0	0
ENSG00000198087	117	4398	4393	5337
ENSG00000198088	3	114	211	234
ENSG00000198089	30	500	281	426
ENSG00000198090	0	0	0	0
ENSG00000198092	0	0	0	0
ENSG00000198093	0	15	3	1
ENSG00000198099	0	0	0	0
ENSG00000198100	0	0	0	0
ENSG00000198104	0	0	0	0
ENSG00000198105	4	167	199	238
ENSG00000198106	4	40	17	43
ENSG00000198108	0	0	0	5
ENSG00000198113	42	126	33	20
ENSG00000198121	1	148	219	390
ENSG00000198125	0	0	0	0
ENSG00000198128	0	0	4	0
ENSG00000198129	0	0	0	0
ENSG00000198130	66	361	292	360
ENSG00000198131	9	89	45	115
ENSG00000198133	5	0	0	0

ENSG00000198134	0	0	0	0
ENSG00000198142	0	0	0	0
ENSG00000198143	0	0	0	0
ENSG00000198146	14	125	126	84
ENSG00000198150	2	0	1	0
ENSG00000198153	0	0	0	0
ENSG00000198155	5	510	962	1217
ENSG00000198156	1	0	0	5
ENSG00000198157	52	29	39	36
ENSG00000198160	452	6678	6700	8377
ENSG00000198161	0	0	0	0
ENSG00000198162	5399	19842	17954	21679
ENSG00000198168	5536	14606	27435	10412
ENSG00000198169	2	12	5	9
ENSG00000198171	19	39	6	27
ENSG00000198173	0	0	0	0
ENSG00000198176	11	114	47	63
ENSG00000198178	1	0	3	0
ENSG00000198182	0	0	0	10
ENSG00000198183	0	0	0	0
ENSG00000198185	3	2	31	86
ENSG00000198189	34	286	234	162
ENSG00000198198	6	38	24	21
ENSG00000198203	0	0	0	0
ENSG00000198205	0	0	3	0
ENSG00000198208	0	0	0	0
ENSG00000198211	0	0	0	0
ENSG00000198216	0	0	0	0
ENSG00000198217	0	0	0	0
ENSG00000198218	10	57	50	74
ENSG00000198221	1	0	1	0
ENSG00000198223	4	44	0	1
ENSG00000198225	2	0	0	2
ENSG00000198229	0	0	0	0
ENSG00000198231	59	351	499	691
ENSG00000198237	0	127	23	84
ENSG00000198242	186	161	107	276
ENSG00000198246	15	0	1	2
ENSG00000198250	0	0	0	0
ENSG00000198251	0	0	0	0
ENSG00000198252	34	732	797	1086
ENSG00000198258	3113	883	388	1297
ENSG00000198261	0	0	0	0
ENSG00000198265	273	3949	4616	3964
ENSG00000198270	2	503	341	621
ENSG00000198271	0	0	0	0

ENSG00000198276	10	0	2	9
ENSG00000198277	0	0	0	0
ENSG00000198283	0	0	0	0
ENSG00000198284	0	0	0	0
ENSG00000198286	1	0	3	0
ENSG00000198298	2	0	5	0
ENSG00000198300	1	0	0	0
ENSG00000198301	61	380	331	426
ENSG00000198305	0	0	0	0
ENSG00000198307	0	0	0	0
ENSG00000198312	0	1	2	0
ENSG00000198315	1	0	11	16
ENSG00000198324	19	4	4	4
ENSG00000198326	1	0	0	0
ENSG00000198327	0	0	1	2
ENSG00000198331	5	0	0	0
ENSG00000198336	14	16	3	5
ENSG00000198339	65	56	172	97
ENSG00000198342	0	34	12	56
ENSG00000198346	1	10	7	11
ENSG00000198353	0	0	0	0
ENSG00000198354	0	0	0	0
ENSG00000198355	4	23	6	4
ENSG00000198356	111	466	133	346
ENSG00000198358	0	0	1	0
ENSG00000198363	192	11178	10764	19604
ENSG00000198366	13	93	91	41
ENSG00000198367	0	0	1	0
ENSG00000198369	69	275	140	181
ENSG00000198373	27	261	110	183
ENSG00000198374	7	55	66	40
ENSG00000198380	24	3868	3114	5255
ENSG00000198382	21	278	261	401
ENSG00000198384	0	2	0	4
ENSG00000198388	0	0	0	0
ENSG00000198390	0	0	0	0
ENSG00000198393	0	0	0	0
ENSG00000198398	0	0	0	0
ENSG00000198399	325	1473	1484	1620
ENSG00000198400	0	0	7	0
ENSG00000198406	0	0	11	10
ENSG00000198408	152	3808	4433	4513
ENSG00000198414	5	28	25	47
ENSG00000198416	0	0	0	0
ENSG00000198417	5	0	0	0
ENSG00000198420	12	0	18	19

ENSG00000198429	2	0	0	2
ENSG00000198431	389	2442	3580	2025
ENSG00000198434	0	0	0	0
ENSG00000198435	0	0	1	0
ENSG00000198440	2	4	2	0
ENSG00000198443	0	0	0	5
ENSG00000198444	0	0	0	0
ENSG00000198445	0	0	0	0
ENSG00000198452	0	51	24	7
ENSG00000198453	6	18	32	51
ENSG00000198454	0	0	0	0
ENSG00000198455	0	0	1	4
ENSG00000198457	0	0	0	0
ENSG00000198464	15	15	27	25
ENSG00000198466	3	135	116	190
ENSG00000198467	83	118	79	27
ENSG00000198468	3	0	1	0
ENSG00000198471	0	0	0	0
ENSG00000198477	0	0	4	1
ENSG00000198478	4457	70441	103447	97191
ENSG00000198482	5	80	81	136
ENSG00000198483	0	0	0	0
ENSG00000198488	0	0	0	0
ENSG00000198491	0	0	0	0
ENSG00000198492	379	327	272	288
ENSG00000198496	37	70	28	316
ENSG00000198498	25	76	59	90
ENSG00000198502	4	15	0	0
ENSG00000198513	248	1372	1582	972
ENSG00000198515	0	0	2	3
ENSG00000198517	4	0	15	5
ENSG00000198518	62	329	320	426
ENSG00000198520	1	0	0	0
ENSG00000198521	14	113	97	138
ENSG00000198522	10	14	5	13
ENSG00000198523	0	0	2	2
ENSG00000198526	0	0	0	0
ENSG00000198535	0	0	1	0
ENSG00000198538	25	243	100	46
ENSG00000198542	0	7	0	0
ENSG00000198546	10	0	3	16
ENSG00000198547	1	15	2	0
ENSG00000198551	1	60	3	9
ENSG00000198553	66	28	66	55
ENSG00000198554	5	748	698	910
ENSG00000198555	0	0	0	0

ENSG00000198556	14	55	40	72
ENSG00000198558	0	0	2	7
ENSG00000198561	7	112	64	61
ENSG00000198563	3	0	6	7
ENSG00000198566	0	0	0	0
ENSG00000198569	0	0	0	0
ENSG00000198570	0	0	0	0
ENSG00000198573	0	0	0	0
ENSG00000198574	11	0	0	0
ENSG00000198576	0	0	0	0
ENSG00000198580	0	0	0	0
ENSG00000198585	27	15	6	0
ENSG00000198586	3230	70893	92708	87975
ENSG00000198589	790	40785	40374	35694
ENSG00000198590	0	0	5	6
ENSG00000198597	0	0	0	0
ENSG00000198598	0	16	0	2
ENSG00000198601	0	0	0	0
ENSG00000198604	261	2829	3412	4000
ENSG00000198610	7	29	6	68
ENSG00000198612	65	270	213	299
ENSG00000198618	0	48	33	34
ENSG00000198624	117	11	48	50
ENSG00000198625	53	318	318	361
ENSG00000198626	675	488	799	284
ENSG00000198632	0	25	0	0
ENSG00000198633	0	0	1	3
ENSG00000198642	18	255	105	210
ENSG00000198643	0	0	0	0
ENSG00000198646	99	552	544	773
ENSG00000198648	66	7778	6640	10868
ENSG00000198650	0	0	0	0
ENSG00000198657	0	0	0	0
ENSG00000198658	0	0	0	0
ENSG00000198663	12	285	143	190
ENSG00000198668	6765	19657	18747	22824
ENSG00000198670	0	0	1	0
ENSG00000198671	0	0	0	0
ENSG00000198673	7	309	195	429
ENSG00000198674	0	0	0	0
ENSG00000198677	218	2367	2166	2033
ENSG00000198678	0	0	0	0
ENSG00000198680	5	0	4	1
ENSG00000198681	0	0	0	0
ENSG00000198682	104	586	644	1569
ENSG00000198683	0	0	0	0

ENSG00000198685	33	11	4	3
ENSG00000198689	11	344	183	564
ENSG00000198690	2	138	114	166
ENSG00000198691	0	0	0	0
ENSG00000198692	320	1382	5	1765
ENSG00000198695	120179	55838	71912	28825
ENSG00000198700	11	298	166	285
ENSG00000198703	0	0	0	0
ENSG00000198704	0	0	0	0
ENSG00000198707	60	811	898	982
ENSG00000198711	0	0	0	0
ENSG00000198712	464183	150710	195808	267117
ENSG00000198715	4	9	4	25
ENSG00000198718	22	2140	1765	2762
ENSG00000198719	0	0	0	0
ENSG00000198720	0	0	1	0
ENSG00000198721	17	98	19	20
ENSG00000198722	3	961	778	1206
ENSG00000198723	3	0	0	0
ENSG00000198727	208639	84469	84699	87419
ENSG00000198728	18	33	48	61
ENSG00000198729	15	11	24	12
ENSG00000198730	398	864	854	1151
ENSG00000198732	2	0	0	0
ENSG00000198734	0	3	8	14
ENSG00000198736	65	25	4	42
ENSG00000198738	0	0	0	0
ENSG00000198739	0	0	0	0
ENSG00000198740	53	1127	965	993
ENSG00000198742	12	279	237	310
ENSG00000198743	19	62	94	66
ENSG00000198744	35	14	62	57
ENSG00000198746	1	0	0	1
ENSG00000198750	0	0	0	0
ENSG00000198752	143	2975	3663	5613
ENSG00000198753	2	129	35	66
ENSG00000198754	0	0	0	0
ENSG00000198755	814	26	22	33
ENSG00000198756	0	0	1	0
ENSG00000198758	0	0	0	0
ENSG00000198759	0	10	9	4
ENSG00000198763	386207	126410	159175	121250
ENSG00000198765	0	0	0	0
ENSG00000198768	2	0	0	8
ENSG00000198771	71	25	18	7
ENSG00000198774	0	0	0	0

ENSG00000198780	4	67	90	76
ENSG00000198783	6	27	3	8
ENSG00000198785	1	0	0	0
ENSG00000198786	285914	113770	120947	103226
ENSG00000198787	0	0	0	0
ENSG00000198788	0	0	0	0
ENSG00000198791	80	281	369	622
ENSG00000198792	2	25	37	62
ENSG00000198793	3	166	52	71
ENSG00000198794	0	0	0	0
ENSG00000198795	4	31	8	31
ENSG00000198796	0	0	0	0
ENSG00000198797	0	0	0	0
ENSG00000198798	0	0	0	0
ENSG00000198799	1	260	371	488
ENSG00000198804	278453	481651	670492	713035
ENSG00000198805	251	2301	3297	1826
ENSG00000198807	0	0	0	0
ENSG00000198812	0	0	0	0
ENSG00000198814	134	1522	2520	3923
ENSG00000198815	19	4155	3210	3812
ENSG00000198816	25	54	10	23
ENSG00000198818	284	1071	1347	1134
ENSG00000198821	6	13	4	0
ENSG00000198822	0	0	0	0
ENSG00000198824	34	101	113	68
ENSG00000198825	8	236	178	235
ENSG00000198826	5	238	284	275
ENSG00000198829	139	364	1639	1358
ENSG00000198830	1162	1906	596	934
ENSG00000198832	3	0	0	0
ENSG00000198833	1174	5151	5201	7285
ENSG00000198835	0	0	0	0
ENSG00000198836	3245	5448	4965	3421
ENSG00000198837	7	0	3	9
ENSG00000198838	0	2	19	35
ENSG00000198839	16	363	226	292
ENSG00000198840	76520	34087	49234	60953
ENSG00000198841	1	0	0	0
ENSG00000198842	0	0	0	1
ENSG00000198843	446	5468	5827	6779
ENSG00000198844	15	0	2	2
ENSG00000198846	61	73	88	43
ENSG00000198848	0	0	0	0
ENSG00000198851	39	0	0	0
ENSG00000198853	0	11	3	0

ENSG00000198854	0	0	0	0
ENSG00000198855	1	0	2	0
ENSG00000198856	16	70	115	229
ENSG00000198857	0	0	0	0
ENSG00000198858	2543	4473	3495	3571
ENSG00000198860	10	153	97	112
ENSG00000198862	7	621	483	600
ENSG00000198863	12	22	3	14
ENSG00000198865	25	58	68	46
ENSG00000198868	0	0	0	0
ENSG00000198870	0	0	0	0
ENSG00000198873	611	2644	2445	2031
ENSG00000198874	3	118	74	182
ENSG00000198876	107	1085	1166	1015
ENSG00000198877	0	0	0	0
ENSG00000198879	18	302	76	96
ENSG00000198881	0	0	0	0
ENSG00000198883	0	0	0	0
ENSG00000198885	2	0	0	0
ENSG00000198886	347883	138501	187895	189829
ENSG00000198887	69	927	970	1323
ENSG00000198888	778359	172883	218095	297272
ENSG00000198889	0	0	0	0
ENSG00000198890	2	0	3	8
ENSG00000198892	4	0	3	1
ENSG00000198894	8	26	41	26
ENSG00000198898	2410	37672	43758	46641
ENSG00000198899	263632	66347	59218	109939
ENSG00000198900	542	4365	3038	3838
ENSG00000198901	5	264	192	235
ENSG00000198908	5	3	32	28
ENSG00000198909	17	37	24	26
ENSG00000198910	0	0	0	0
ENSG00000198911	60	231	324	498
ENSG00000198912	115	538	325	763
ENSG00000198914	0	0	0	0
ENSG00000198915	2	0	0	0
ENSG00000198917	10	0	2	7
ENSG00000198918	1862	634	495	1589
ENSG00000198919	8	134	248	298
ENSG00000198920	7	159	162	122
ENSG00000198923	0	0	0	0
ENSG00000198924	2196	2852	2822	2754
ENSG00000198925	210	306	282	323
ENSG00000198929	30	74	225	358
ENSG00000198930	0	0	0	0

ENSG00000198931	82	0	4	7
ENSG00000198932	2	0	1	3
ENSG00000198933	0	0	0	10
ENSG00000198934	3	22	5	5
ENSG00000198937	16	0	0	0
ENSG00000198938	272528	119274	145990	170729
ENSG00000198939	0	19	5	7
ENSG00000198944	0	0	0	0
ENSG00000198945	15	82	76	140
ENSG00000198946	0	0	0	0
ENSG00000198947	16	261	193	208
ENSG00000198948	3239	38268	55887	47578
ENSG00000198951	72	118	135	188
ENSG00000198952	32	152	91	189
ENSG00000198954	3	35	38	59
ENSG00000198959	5	28	1	2
ENSG00000198960	431	4182	4581	3575
ENSG00000198961	230	2421	4603	2135
ENSG00000198963	0	0	0	0
ENSG00000198964	175	3962	5542	7303
ENSG00000198965	0	0	0	0
ENSG00000198967	0	0	0	0
ENSG00000198972	0	0	0	0
ENSG00000198973	0	0	0	0
ENSG00000198974	0	0	0	0
ENSG00000198975	0	0	0	0
ENSG00000198976	0	0	0	0
ENSG00000198981	0	0	0	0
ENSG00000198982	0	0	0	0
ENSG00000198983	0	0	0	0
ENSG00000198984	0	0	0	0
ENSG00000198986	0	0	0	0
ENSG00000198987	0	0	0	0
ENSG00000198995	0	0	0	0
ENSG00000198997	0	0	0	0
ENSG00000199001	0	0	0	0
ENSG00000199004	0	0	0	0
ENSG00000199005	0	0	0	0
ENSG00000199012	0	0	0	0
ENSG00000199015	0	0	0	0
ENSG00000199017	0	0	0	0
ENSG00000199020	0	0	0	0
ENSG00000199023	0	0	0	0
ENSG00000199024	3	0	3	4
ENSG00000199025	0	0	0	0
ENSG00000199030	0	0	0	0

ENSG00000199031	0	0	0	0
ENSG00000199032	0	0	0	0
ENSG00000199035	0	0	0	0
ENSG00000199036	0	0	0	0
ENSG00000199038	0	0	0	0
ENSG00000199043	0	0	0	0
ENSG00000199047	0	0	0	0
ENSG00000199051	0	0	0	0
ENSG00000199053	0	0	0	0
ENSG00000199058	0	0	0	0
ENSG00000199059	0	0	0	0
ENSG00000199060	0	0	0	0
ENSG00000199065	0	0	0	0
ENSG00000199066	0	0	0	0
ENSG00000199069	0	0	0	0
ENSG00000199071	0	0	0	0
ENSG00000199072	0	0	0	0
ENSG00000199075	0	0	0	0
ENSG00000199077	0	0	0	0
ENSG00000199080	0	0	0	0
ENSG00000199082	0	0	0	0
ENSG00000199085	0	0	0	0
ENSG00000199088	0	0	0	0
ENSG00000199089	0	0	0	0
ENSG00000199090	0	0	0	0
ENSG00000199092	0	0	0	0
ENSG00000199094	0	0	0	0
ENSG00000199095	0	0	0	0
ENSG00000199097	0	0	0	0
ENSG00000199102	0	0	0	0
ENSG00000199104	0	0	0	0
ENSG00000199107	0	0	0	0
ENSG00000199109	0	0	0	0
ENSG00000199121	0	0	0	0
ENSG00000199122	0	0	0	0
ENSG00000199127	0	0	0	0
ENSG00000199130	0	0	0	0
ENSG00000199132	0	0	0	0
ENSG00000199133	0	0	0	0
ENSG00000199135	0	0	1	0
ENSG00000199143	0	0	0	0
ENSG00000199145	0	0	0	0
ENSG00000199149	0	0	0	0
ENSG00000199150	0	0	0	0
ENSG00000199151	0	0	0	0
ENSG00000199153	0	0	0	0

ENSG00000199156	0	0	0	0
ENSG00000199157	0	0	0	0
ENSG00000199158	0	0	0	0
ENSG00000199161	0	0	0	0
ENSG00000199165	8	0	3	4
ENSG00000199168	0	0	0	0
ENSG00000199169	0	0	0	0
ENSG00000199172	0	0	0	0
ENSG00000199177	0	0	0	0
ENSG00000199179	0	0	0	0
ENSG00000199180	0	0	0	0
ENSG00000199187	0	0	0	0
ENSG00000199196	0	0	0	0
ENSG00000199197	0	0	0	0
ENSG00000199200	0	0	0	0
ENSG00000199201	0	0	0	0
ENSG00000199202	0	0	0	0
ENSG00000199203	0	0	0	0
ENSG00000199204	0	0	0	0
ENSG00000199212	0	0	0	0
ENSG00000199214	0	0	0	0
ENSG00000199217	0	0	0	0
ENSG00000199218	0	0	0	0
ENSG00000199219	0	0	0	0
ENSG00000199220	0	0	0	0
ENSG00000199222	0	0	0	0
ENSG00000199223	0	0	0	0
ENSG00000199224	0	0	0	0
ENSG00000199226	0	0	0	0
ENSG00000199231	0	0	0	0
ENSG00000199235	0	0	0	0
ENSG00000199237	0	0	0	0
ENSG00000199240	0	0	0	0
ENSG00000199241	0	0	0	0
ENSG00000199245	0	0	0	0
ENSG00000199246	0	0	0	0
ENSG00000199248	0	0	0	0
ENSG00000199251	0	0	0	0
ENSG00000199260	0	0	0	0
ENSG00000199262	0	0	0	0
ENSG00000199263	0	0	0	0
ENSG00000199266	0	0	0	0
ENSG00000199270	0	0	0	0
ENSG00000199272	0	0	0	0
ENSG00000199273	0	0	0	0
ENSG00000199276	0	0	0	0

ENSG00000199279	0	0	0	0
ENSG00000199282	0	0	0	0
ENSG00000199283	0	0	0	0
ENSG00000199285	0	0	0	0
ENSG00000199286	0	0	0	0
ENSG00000199287	0	0	0	0
ENSG00000199289	0	0	0	0
ENSG00000199290	0	0	0	0
ENSG00000199291	0	0	0	0
ENSG00000199293	0	0	0	0
ENSG00000199295	0	0	0	0
ENSG00000199298	0	0	0	0
ENSG00000199299	0	0	0	0
ENSG00000199301	0	0	0	0
ENSG00000199303	0	0	0	0
ENSG00000199306	0	0	0	0
ENSG00000199308	0	0	0	0
ENSG00000199311	0	0	0	0
ENSG00000199313	0	0	0	0
ENSG00000199315	0	0	0	0
ENSG00000199318	0	0	0	0
ENSG00000199319	0	0	0	0
ENSG00000199321	0	0	0	0
ENSG00000199322	0	0	0	0
ENSG00000199325	0	0	0	0
ENSG00000199326	0	0	0	0
ENSG00000199327	0	0	0	0
ENSG00000199331	0	0	0	0
ENSG00000199332	0	0	0	0
ENSG00000199334	0	0	0	0
ENSG00000199335	9	0	12	3
ENSG00000199337	0	0	0	0
ENSG00000199347	0	0	3	0
ENSG00000199348	0	0	0	0
ENSG00000199349	0	0	0	0
ENSG00000199350	0	0	0	0
ENSG00000199352	0	0	0	0
ENSG00000199354	1	0	0	0
ENSG00000199357	0	0	0	0
ENSG00000199360	0	0	0	0
ENSG00000199361	0	0	0	0
ENSG00000199362	0	0	0	0
ENSG00000199363	0	0	0	0
ENSG00000199364	0	0	0	0
ENSG00000199366	0	0	0	0
ENSG00000199368	0	0	0	0

ENSG00000199370	0	0	0	0
ENSG00000199373	0	0	0	0
ENSG00000199377	0	0	0	0
ENSG00000199378	0	0	0	0
ENSG00000199381	0	0	0	0
ENSG00000199382	0	0	0	0
ENSG00000199385	0	0	0	0
ENSG00000199390	0	0	0	0
ENSG00000199392	0	0	0	0
ENSG00000199394	0	0	0	0
ENSG00000199395	0	0	0	0
ENSG00000199396	0	0	0	0
ENSG00000199398	0	0	0	0
ENSG00000199400	0	0	0	0
ENSG00000199402	0	0	0	0
ENSG00000199404	0	0	0	0
ENSG00000199405	0	0	0	0
ENSG00000199407	0	0	0	0
ENSG00000199409	0	0	0	0
ENSG00000199410	0	0	0	0
ENSG00000199411	0	0	0	0
ENSG00000199415	0	0	0	0
ENSG00000199420	0	0	0	0
ENSG00000199422	0	0	0	0
ENSG00000199424	0	0	0	0
ENSG00000199426	0	0	0	0
ENSG00000199432	0	0	0	0
ENSG00000199436	0	0	0	0
ENSG00000199437	0	0	0	0
ENSG00000199438	0	0	0	0
ENSG00000199440	0	0	0	0
ENSG00000199442	0	0	0	4
ENSG00000199444	0	0	0	0
ENSG00000199446	0	0	0	0
ENSG00000199448	0	0	0	0
ENSG00000199450	0	0	0	0
ENSG00000199452	0	0	0	0
ENSG00000199453	0	0	0	0
ENSG00000199454	0	0	0	0
ENSG00000199455	0	0	0	0
ENSG00000199458	0	0	0	0
ENSG00000199459	0	0	0	0
ENSG00000199460	0	0	0	0
ENSG00000199461	0	0	0	0
ENSG00000199466	0	0	0	0
ENSG00000199468	0	0	0	0

ENSG00000199469	0	0	0	0
ENSG00000199470	0	0	0	0
ENSG00000199471	0	0	0	0
ENSG00000199472	0	0	0	0
ENSG00000199473	0	0	0	0
ENSG00000199474	0	0	0	0
ENSG00000199475	0	0	0	0
ENSG00000199476	0	0	0	0
ENSG00000199477	0	0	0	0
ENSG00000199480	0	0	0	0
ENSG00000199482	0	0	0	0
ENSG00000199483	0	0	0	0
ENSG00000199487	0	0	0	0
ENSG00000199488	0	0	0	0
ENSG00000199489	0	0	0	0
ENSG00000199490	0	0	0	0
ENSG00000199492	0	0	0	0
ENSG00000199497	0	0	0	0
ENSG00000199506	0	0	0	0
ENSG00000199508	0	0	0	0
ENSG00000199509	0	0	0	0
ENSG00000199512	0	0	0	0
ENSG00000199514	0	0	0	0
ENSG00000199515	0	0	0	0
ENSG00000199516	0	0	0	0
ENSG00000199520	0	0	0	0
ENSG00000199523	0	0	0	0
ENSG00000199525	0	0	0	0
ENSG00000199529	0	0	0	0
ENSG00000199530	1	0	0	0
ENSG00000199535	0	0	0	0
ENSG00000199536	0	0	0	0
ENSG00000199537	0	0	0	0
ENSG00000199540	0	0	0	0
ENSG00000199545	0	0	0	0
ENSG00000199546	0	0	0	0
ENSG00000199550	0	0	0	0
ENSG00000199551	0	0	0	0
ENSG00000199552	0	0	0	0
ENSG00000199554	0	0	0	0
ENSG00000199556	0	0	0	0
ENSG00000199562	1	0	3	0
ENSG00000199564	0	0	0	0
ENSG00000199565	0	0	0	0
ENSG00000199566	0	0	0	0
ENSG00000199567	0	0	0	0

ENSG00000199568	0	0	0	0
ENSG00000199570	0	0	0	4
ENSG00000199571	0	0	0	0
ENSG00000199572	0	0	0	0
ENSG00000199574	0	0	0	0
ENSG00000199575	0	0	0	0
ENSG00000199577	0	0	0	0
ENSG00000199580	0	0	0	0
ENSG00000199584	0	0	0	0
ENSG00000199585	0	0	0	0
ENSG00000199591	0	0	0	0
ENSG00000199592	0	0	0	0
ENSG00000199593	0	0	0	0
ENSG00000199594	0	0	0	0
ENSG00000199595	0	0	0	0
ENSG00000199598	0	0	0	0
ENSG00000199601	0	0	0	0
ENSG00000199603	0	0	0	0
ENSG00000199605	0	0	0	0
ENSG00000199609	0	0	0	0
ENSG00000199620	0	0	0	0
ENSG00000199622	0	0	0	0
ENSG00000199626	0	0	0	0
ENSG00000199627	0	0	0	0
ENSG00000199629	0	13	0	0
ENSG00000199630	0	0	0	0
ENSG00000199631	0	0	0	0
ENSG00000199633	0	0	0	0
ENSG00000199634	0	0	0	0
ENSG00000199635	0	0	0	0
ENSG00000199636	0	0	0	2
ENSG00000199638	0	0	0	0
ENSG00000199640	0	0	0	0
ENSG00000199641	0	0	0	0
ENSG00000199643	0	0	0	0
ENSG00000199645	0	0	0	0
ENSG00000199646	0	0	0	0
ENSG00000199652	0	0	0	0
ENSG00000199654	0	0	0	0
ENSG00000199663	0	0	0	0
ENSG00000199664	0	0	0	0
ENSG00000199666	0	0	0	0
ENSG00000199667	0	0	0	0
ENSG00000199668	0	0	0	0
ENSG00000199672	0	0	0	0
ENSG00000199673	0	0	0	0

ENSG00000199674	0	0	1	0
ENSG00000199675	0	0	0	0
ENSG00000199676	0	0	0	0
ENSG00000199677	0	0	0	0
ENSG00000199678	0	0	0	12
ENSG00000199683	0	0	0	0
ENSG00000199687	0	0	0	0
ENSG00000199691	0	0	2	0
ENSG00000199695	0	0	0	0
ENSG00000199697	0	0	0	0
ENSG00000199698	0	0	0	0
ENSG00000199700	0	0	0	0
ENSG00000199701	0	0	0	0
ENSG00000199702	0	0	0	0
ENSG00000199704	0	0	0	0
ENSG00000199705	0	0	0	0
ENSG00000199709	0	0	0	0
ENSG00000199710	0	0	4	0
ENSG00000199711	0	0	0	0
ENSG00000199712	0	0	0	0
ENSG00000199713	0	0	0	0
ENSG00000199715	0	0	0	0
ENSG00000199716	0	0	0	0
ENSG00000199719	0	0	0	0
ENSG00000199727	0	0	2	0
ENSG00000199728	0	0	0	0
ENSG00000199730	0	0	0	6
ENSG00000199731	0	0	0	0
ENSG00000199732	0	0	0	0
ENSG00000199733	0	0	0	0
ENSG00000199735	0	0	0	0
ENSG00000199739	0	0	0	0
ENSG00000199740	0	0	0	0
ENSG00000199744	0	0	0	0
ENSG00000199751	0	0	0	0
ENSG00000199753	0	0	0	0
ENSG00000199755	0	0	0	0
ENSG00000199756	0	0	0	0
ENSG00000199762	0	0	0	0
ENSG00000199764	0	0	0	0
ENSG00000199765	0	0	0	0
ENSG00000199767	0	0	0	0
ENSG00000199769	0	0	0	0
ENSG00000199771	0	0	0	0
ENSG00000199773	0	0	0	0
ENSG00000199780	0	0	0	0

ENSG00000199781	0	0	0	0
ENSG00000199782	0	0	0	0
ENSG00000199783	0	0	0	0
ENSG00000199784	0	0	0	0
ENSG00000199785	0	0	0	0
ENSG00000199786	0	0	0	0
ENSG00000199787	0	0	0	0
ENSG00000199788	0	0	0	0
ENSG00000199789	0	0	0	0
ENSG00000199790	0	0	0	0
ENSG00000199791	0	0	0	0
ENSG00000199792	0	0	0	0
ENSG00000199796	0	0	0	0
ENSG00000199797	0	0	0	0
ENSG00000199798	0	0	0	0
ENSG00000199801	0	0	0	0
ENSG00000199803	0	0	0	0
ENSG00000199804	0	0	0	0
ENSG00000199805	0	0	0	0
ENSG00000199806	0	0	0	0
ENSG00000199809	0	0	0	0
ENSG00000199812	0	0	0	0
ENSG00000199814	0	0	0	0
ENSG00000199815	0	0	0	0
ENSG00000199824	0	0	0	0
ENSG00000199827	0	0	0	0
ENSG00000199831	0	0	0	0
ENSG00000199832	0	0	0	0
ENSG00000199833	0	0	0	0
ENSG00000199836	0	0	0	0
ENSG00000199837	0	0	0	0
ENSG00000199839	0	0	0	0
ENSG00000199840	0	0	0	0
ENSG00000199843	0	0	0	0
ENSG00000199845	0	0	0	0
ENSG00000199846	0	0	0	0
ENSG00000199849	0	0	0	0
ENSG00000199851	0	0	0	0
ENSG00000199855	0	0	0	0
ENSG00000199856	0	0	0	0
ENSG00000199857	0	0	0	0
ENSG00000199858	0	0	0	0
ENSG00000199859	0	0	0	0
ENSG00000199862	0	0	0	0
ENSG00000199865	0	0	0	0
ENSG00000199866	0	0	0	0

ENSG00000199867	0	0	0	0
ENSG00000199870	0	0	0	0
ENSG00000199872	0	0	0	0
ENSG00000199874	0	0	0	0
ENSG00000199875	0	0	0	0
ENSG00000199876	0	0	0	0
ENSG00000199878	0	0	0	0
ENSG00000199879	0	0	0	0
ENSG00000199880	0	0	0	0
ENSG00000199881	0	0	0	0
ENSG00000199883	0	0	0	0
ENSG00000199884	0	0	0	0
ENSG00000199885	0	0	0	0
ENSG00000199886	0	0	0	0
ENSG00000199890	0	0	0	0
ENSG00000199892	0	0	0	0
ENSG00000199893	0	0	0	0
ENSG00000199894	0	0	0	0
ENSG00000199895	0	0	0	0
ENSG00000199899	0	0	0	0
ENSG00000199900	0	0	0	0
ENSG00000199901	0	0	0	0
ENSG00000199903	0	0	0	0
ENSG00000199905	0	0	0	0
ENSG00000199906	0	0	0	0
ENSG00000199910	0	0	0	0
ENSG00000199911	0	0	0	0
ENSG00000199912	0	0	0	0
ENSG00000199913	0	0	0	0
ENSG00000199914	0	0	0	0
ENSG00000199916	0	0	0	0
ENSG00000199920	0	0	0	0
ENSG00000199921	0	0	0	0
ENSG00000199924	0	0	0	0
ENSG00000199927	0	0	0	0
ENSG00000199929	0	0	3	0
ENSG00000199932	0	0	0	0
ENSG00000199933	0	0	0	0
ENSG00000199934	0	0	0	0
ENSG00000199936	0	0	0	0
ENSG00000199938	0	0	0	0
ENSG00000199940	0	0	0	0
ENSG00000199942	0	0	0	0
ENSG00000199944	0	0	0	0
ENSG00000199949	0	0	0	0
ENSG00000199953	0	0	0	0

ENSG00000199959	0	0	0	0
ENSG00000199960	0	0	0	0
ENSG00000199961	0	0	0	0
ENSG00000199962	0	0	0	0
ENSG00000199963	0	0	0	0
ENSG00000199964	0	0	0	0
ENSG00000199968	0	0	0	0
ENSG00000199970	0	0	0	0
ENSG00000199971	0	0	0	0
ENSG00000199975	0	0	0	0
ENSG00000199977	0	0	0	0
ENSG00000199979	0	0	0	0
ENSG00000199980	0	0	0	0
ENSG00000199985	0	0	0	0
ENSG00000199986	0	0	0	0
ENSG00000199990	0	1	2	0
ENSG00000199994	0	0	0	0
ENSG00000200000	0	0	0	0
ENSG00000200003	0	0	0	0
ENSG00000200008	0	0	0	0
ENSG00000200011	0	0	0	0
ENSG00000200013	0	0	0	0
ENSG00000200016	0	0	0	0
ENSG00000200021	0	0	0	0
ENSG00000200024	0	0	0	0
ENSG00000200026	0	0	0	0
ENSG00000200028	0	0	0	0
ENSG00000200029	0	0	0	0
ENSG00000200033	0	0	0	0
ENSG00000200034	0	0	0	0
ENSG00000200036	0	0	0	0
ENSG00000200037	0	0	0	0
ENSG00000200040	0	0	0	0
ENSG00000200041	0	0	0	0
ENSG00000200042	0	0	0	0
ENSG00000200047	0	0	0	0
ENSG00000200048	0	0	0	0
ENSG00000200049	0	0	0	0
ENSG00000200051	0	0	0	0
ENSG00000200052	0	0	0	0
ENSG00000200057	0	0	0	0
ENSG00000200058	0	0	0	0
ENSG00000200059	0	0	0	0
ENSG00000200060	0	0	0	0
ENSG00000200062	0	0	0	0
ENSG00000200063	0	0	0	0

ENSG00000200064	0	0	0	0
ENSG00000200065	0	0	0	0
ENSG00000200066	0	0	0	0
ENSG00000200070	0	0	0	0
ENSG00000200072	0	0	0	0
ENSG00000200075	0	0	0	0
ENSG00000200079	0	0	0	0
ENSG00000200084	0	0	0	0
ENSG00000200085	0	0	4	8
ENSG00000200086	0	0	0	0
ENSG00000200087	0	0	2	5
ENSG00000200089	0	0	0	0
ENSG00000200090	0	0	0	0
ENSG00000200091	0	0	0	0
ENSG00000200095	0	0	0	0
ENSG00000200097	0	0	0	0
ENSG00000200101	0	0	0	0
ENSG00000200102	0	0	0	0
ENSG00000200105	0	0	0	0
ENSG00000200106	0	0	0	0
ENSG00000200107	0	0	0	0
ENSG00000200108	0	0	0	0
ENSG00000200112	0	0	0	0
ENSG00000200113	0	0	0	0
ENSG00000200114	0	0	0	0
ENSG00000200115	0	0	0	0
ENSG00000200118	0	0	0	0
ENSG00000200120	0	0	0	0
ENSG00000200121	0	0	0	0
ENSG00000200127	0	0	0	0
ENSG00000200130	0	0	0	0
ENSG00000200131	0	0	0	0
ENSG00000200132	0	0	0	0
ENSG00000200135	0	0	0	0
ENSG00000200138	0	0	0	0
ENSG00000200139	0	0	0	0
ENSG00000200141	0	0	0	0
ENSG00000200142	0	0	0	0
ENSG00000200146	0	0	0	0
ENSG00000200150	0	0	0	0
ENSG00000200151	0	0	0	0
ENSG00000200152	0	0	0	0
ENSG00000200153	0	0	0	0
ENSG00000200154	0	0	0	0
ENSG00000200156	0	0	0	0
ENSG00000200161	0	0	0	0

ENSG00000200162	0	0	0	0
ENSG00000200163	0	0	0	0
ENSG00000200164	0	0	0	0
ENSG00000200168	0	0	0	0
ENSG00000200169	0	0	0	0
ENSG00000200170	0	0	0	0
ENSG00000200171	0	0	0	0
ENSG00000200174	0	0	0	0
ENSG00000200175	0	0	0	0
ENSG00000200176	0	0	0	0
ENSG00000200179	0	0	0	0
ENSG00000200181	0	0	0	0
ENSG00000200182	0	0	0	0
ENSG00000200183	0	0	0	0
ENSG00000200184	0	0	0	0
ENSG00000200185	0	0	0	0
ENSG00000200189	0	0	0	0
ENSG00000200191	0	0	0	0
ENSG00000200197	0	0	0	0
ENSG00000200198	0	0	0	0
ENSG00000200201	0	0	0	0
ENSG00000200204	0	0	0	0
ENSG00000200206	0	0	0	0
ENSG00000200208	0	0	0	0
ENSG00000200209	0	0	0	0
ENSG00000200211	0	0	0	0
ENSG00000200213	0	0	0	0
ENSG00000200215	0	0	0	0
ENSG00000200216	0	0	0	0
ENSG00000200217	0	0	0	0
ENSG00000200218	0	0	0	0
ENSG00000200220	0	0	0	0
ENSG00000200222	0	0	0	0
ENSG00000200224	0	0	0	0
ENSG00000200225	0	0	0	0
ENSG00000200227	0	0	0	0
ENSG00000200228	0	0	0	0
ENSG00000200229	0	0	0	0
ENSG00000200231	0	0	0	0
ENSG00000200235	0	0	0	0
ENSG00000200237	0	0	0	0
ENSG00000200238	0	0	0	0
ENSG00000200241	0	0	0	0
ENSG00000200243	0	0	0	0
ENSG00000200246	0	0	0	0
ENSG00000200247	0	0	0	0

ENSG00000200248	0	0	0	0
ENSG00000200250	0	0	0	0
ENSG00000200252	0	0	0	0
ENSG00000200253	0	0	0	0
ENSG00000200254	0	0	0	0
ENSG00000200256	0	0	0	0
ENSG00000200257	0	0	0	0
ENSG00000200259	0	0	0	0
ENSG00000200261	0	0	0	0
ENSG00000200262	0	0	0	0
ENSG00000200267	0	0	0	0
ENSG00000200269	0	0	0	0
ENSG00000200270	0	0	0	0
ENSG00000200274	0	0	0	0
ENSG00000200275	0	0	0	0
ENSG00000200278	0	21	23	11
ENSG00000200279	0	0	0	0
ENSG00000200280	0	0	0	0
ENSG00000200281	0	0	0	0
ENSG00000200283	0	0	0	0
ENSG00000200285	0	0	0	0
ENSG00000200287	0	0	0	0
ENSG00000200288	0	0	0	0
ENSG00000200291	0	0	0	0
ENSG00000200293	0	0	0	0
ENSG00000200294	0	0	0	0
ENSG00000200295	0	0	0	0
ENSG00000200296	0	0	0	0
ENSG00000200298	0	0	0	0
ENSG00000200301	0	0	0	0
ENSG00000200303	0	0	0	0
ENSG00000200304	0	0	0	0
ENSG00000200305	0	0	0	0
ENSG00000200309	0	0	0	0
ENSG00000200310	0	0	0	0
ENSG00000200312	0	3155	3162	7097
ENSG00000200313	0	0	0	0
ENSG00000200314	0	0	0	0
ENSG00000200318	0	0	0	0
ENSG00000200319	0	0	0	0
ENSG00000200320	0	0	0	0
ENSG00000200325	0	0	0	0
ENSG00000200326	0	0	0	0
ENSG00000200327	0	0	0	0
ENSG00000200331	0	0	0	0
ENSG00000200332	0	0	0	0

ENSG00000200334	0	0	0	0
ENSG00000200336	0	0	0	0
ENSG00000200338	0	0	0	0
ENSG00000200340	0	0	0	0
ENSG00000200343	0	0	0	0
ENSG00000200344	0	0	0	0
ENSG00000200345	0	0	0	0
ENSG00000200347	0	0	0	0
ENSG00000200350	0	0	1	0
ENSG00000200351	0	0	0	0
ENSG00000200354	0	0	0	0
ENSG00000200355	0	0	0	0
ENSG00000200356	0	0	0	0
ENSG00000200360	0	0	0	0
ENSG00000200361	0	0	0	0
ENSG00000200366	0	0	0	0
ENSG00000200367	0	0	0	0
ENSG00000200369	0	0	0	0
ENSG00000200370	0	0	0	0
ENSG00000200372	0	0	0	0
ENSG00000200376	0	0	0	0
ENSG00000200377	0	0	0	0
ENSG00000200378	0	0	0	0
ENSG00000200379	0	0	0	0
ENSG00000200381	0	0	0	0
ENSG00000200385	0	0	0	0
ENSG00000200388	0	0	0	0
ENSG00000200389	0	0	0	0
ENSG00000200390	0	0	0	0
ENSG00000200391	0	0	0	0
ENSG00000200393	0	0	0	0
ENSG00000200394	0	0	0	0
ENSG00000200397	0	0	0	0
ENSG00000200398	0	0	0	0
ENSG00000200403	0	0	0	0
ENSG00000200406	0	0	0	0
ENSG00000200407	0	0	0	0
ENSG00000200408	0	0	0	0
ENSG00000200411	0	0	0	0
ENSG00000200413	0	0	0	0
ENSG00000200418	0	0	0	0
ENSG00000200419	0	0	0	0
ENSG00000200421	0	0	0	0
ENSG00000200422	0	0	0	0
ENSG00000200424	0	0	0	0
ENSG00000200427	0	0	0	0

ENSG00000200428	0	0	0	0
ENSG00000200431	0	0	0	0
ENSG00000200432	0	0	0	0
ENSG00000200434	0	0	0	0
ENSG00000200436	0	0	0	0
ENSG00000200437	0	0	0	0
ENSG00000200442	0	0	0	0
ENSG00000200443	0	0	0	0
ENSG00000200444	0	0	0	0
ENSG00000200446	0	0	0	0
ENSG00000200448	0	0	0	0
ENSG00000200455	0	0	0	0
ENSG00000200456	0	0	0	0
ENSG00000200462	0	0	0	0
ENSG00000200463	0	0	0	0
ENSG00000200468	0	0	0	0
ENSG00000200469	0	0	0	0
ENSG00000200471	0	0	0	0
ENSG00000200472	0	0	0	0
ENSG00000200473	0	0	0	0
ENSG00000200475	0	0	0	0
ENSG00000200478	0	0	0	0
ENSG00000200480	0	0	0	0
ENSG00000200483	0	0	0	0
ENSG00000200484	0	0	0	0
ENSG00000200485	0	0	0	0
ENSG00000200486	0	0	0	0
ENSG00000200487	0	0	0	0
ENSG00000200488	0	1393	994	625
ENSG00000200492	0	0	0	0
ENSG00000200494	0	0	0	0
ENSG00000200495	0	0	0	0
ENSG00000200496	0	0	0	0
ENSG00000200502	0	16	2	2
ENSG00000200503	0	0	0	0
ENSG00000200506	0	0	0	0
ENSG00000200508	0	0	0	0
ENSG00000200510	0	0	0	0
ENSG00000200516	0	0	0	0
ENSG00000200520	0	0	0	0
ENSG00000200521	0	0	0	0
ENSG00000200522	0	0	0	0
ENSG00000200525	0	0	0	0
ENSG00000200526	0	0	0	0
ENSG00000200527	0	0	0	0
ENSG00000200528	0	0	0	0

ENSG00000200530	0	0	0	0
ENSG00000200534	0	0	0	0
ENSG00000200536	0	0	0	0
ENSG00000200537	0	0	0	0
ENSG00000200538	0	0	0	0
ENSG00000200543	0	0	0	0
ENSG00000200544	0	0	0	0
ENSG00000200545	0	0	0	0
ENSG00000200547	0	0	0	0
ENSG00000200550	0	0	0	0
ENSG00000200552	0	0	0	0
ENSG00000200553	0	0	0	0
ENSG00000200554	0	0	0	0
ENSG00000200555	0	0	0	0
ENSG00000200556	0	0	0	0
ENSG00000200558	0	0	0	0
ENSG00000200560	0	0	0	0
ENSG00000200563	0	0	0	0
ENSG00000200564	0	0	0	0
ENSG00000200566	0	0	0	0
ENSG00000200570	0	0	0	0
ENSG00000200571	0	0	0	0
ENSG00000200572	0	0	0	0
ENSG00000200575	0	0	0	0
ENSG00000200579	0	0	0	0
ENSG00000200587	0	0	0	0
ENSG00000200591	0	0	0	0
ENSG00000200593	0	0	0	0
ENSG00000200594	0	0	0	0
ENSG00000200597	0	0	0	0
ENSG00000200600	0	0	0	0
ENSG00000200601	0	0	0	0
ENSG00000200605	0	0	0	0
ENSG00000200606	0	0	0	0
ENSG00000200608	0	0	0	0
ENSG00000200610	0	0	0	0
ENSG00000200612	0	0	0	0
ENSG00000200613	0	0	0	0
ENSG00000200615	0	0	0	0
ENSG00000200616	0	0	0	0
ENSG00000200619	0	0	0	0
ENSG00000200620	0	0	0	0
ENSG00000200622	0	0	0	0
ENSG00000200623	0	0	0	0
ENSG00000200624	0	0	0	0
ENSG00000200626	0	0	0	0

ENSG00000200629	0	0	0	0
ENSG00000200630	0	0	0	0
ENSG00000200632	0	0	0	0
ENSG00000200635	0	0	0	0
ENSG00000200636	0	0	0	0
ENSG00000200637	0	0	0	0
ENSG00000200638	0	0	0	0
ENSG00000200645	0	0	0	0
ENSG00000200646	0	0	0	0
ENSG00000200648	0	0	0	0
ENSG00000200649	7	0	0	0
ENSG00000200650	0	0	0	0
ENSG00000200651	0	0	0	0
ENSG00000200652	0	0	0	0
ENSG00000200653	0	0	0	0
ENSG00000200654	0	0	0	0
ENSG00000200656	0	0	0	0
ENSG00000200658	0	0	0	0
ENSG00000200661	0	0	0	0
ENSG00000200662	0	0	0	0
ENSG00000200664	0	0	0	0
ENSG00000200665	0	0	0	0
ENSG00000200670	0	0	0	0
ENSG00000200673	0	0	0	0
ENSG00000200674	0	0	0	0
ENSG00000200677	0	0	0	0
ENSG00000200680	0	0	0	0
ENSG00000200681	0	0	0	0
ENSG00000200683	0	0	0	0
ENSG00000200685	0	0	0	0
ENSG00000200686	0	0	0	0
ENSG00000200687	0	0	0	0
ENSG00000200688	0	0	0	0
ENSG00000200693	17	0	0	0
ENSG00000200701	0	0	0	0
ENSG00000200702	0	0	0	0
ENSG00000200703	0	0	0	0
ENSG00000200706	0	0	0	0
ENSG00000200708	0	0	0	0
ENSG00000200709	0	0	0	0
ENSG00000200710	0	0	0	0
ENSG00000200711	0	0	0	0
ENSG00000200713	0	0	0	0
ENSG00000200714	0	0	0	0
ENSG00000200718	0	0	0	0
ENSG00000200719	0	0	0	0

ENSG00000200720	0	0	0	0
ENSG00000200726	0	0	0	0
ENSG00000200728	0	0	0	0
ENSG00000200729	0	0	0	0
ENSG00000200731	0	0	0	0
ENSG00000200732	0	0	0	0
ENSG00000200733	0	0	0	0
ENSG00000200735	0	0	0	0
ENSG00000200737	0	0	0	0
ENSG00000200738	0	0	0	0
ENSG00000200741	0	0	0	0
ENSG00000200742	0	41	2	3
ENSG00000200745	0	0	0	0
ENSG00000200750	0	0	0	0
ENSG00000200752	0	0	0	0
ENSG00000200753	0	0	0	0
ENSG00000200754	0	0	0	0
ENSG00000200755	0	0	0	0
ENSG00000200756	0	0	0	0
ENSG00000200757	0	0	0	0
ENSG00000200759	0	0	0	0
ENSG00000200761	0	0	0	0
ENSG00000200763	0	0	0	0
ENSG00000200764	0	0	0	0
ENSG00000200769	0	0	0	0
ENSG00000200774	0	0	0	0
ENSG00000200779	0	0	0	0
ENSG00000200783	0	0	0	0
ENSG00000200785	0	0	0	0
ENSG00000200786	0	0	0	0
ENSG00000200788	0	0	0	0
ENSG00000200789	0	0	0	0
ENSG00000200790	0	0	0	0
ENSG00000200792	0	0	0	0
ENSG00000200794	0	0	0	0
ENSG00000200795	0	0	0	0
ENSG00000200796	0	0	0	0
ENSG00000200799	0	0	1	0
ENSG00000200800	0	0	0	0
ENSG00000200801	0	0	0	0
ENSG00000200806	0	0	0	0
ENSG00000200807	0	0	0	0
ENSG00000200812	0	0	0	0
ENSG00000200814	0	0	0	0
ENSG00000200815	0	0	0	0
ENSG00000200816	0	0	1	0

ENSG00000200817	0	0	0	0
ENSG00000200818	0	0	0	0
ENSG00000200822	0	0	0	0
ENSG00000200823	0	0	0	0
ENSG00000200827	0	0	0	0
ENSG00000200829	0	0	0	0
ENSG00000200830	0	0	0	0
ENSG00000200831	0	0	0	0
ENSG00000200832	0	0	0	0
ENSG00000200833	0	0	0	0
ENSG00000200834	0	0	0	0
ENSG00000200837	0	0	0	0
ENSG00000200839	0	0	0	0
ENSG00000200840	0	0	0	0
ENSG00000200842	0	0	0	0
ENSG00000200843	0	0	0	0
ENSG00000200847	0	0	0	0
ENSG00000200849	0	0	0	0
ENSG00000200851	0	0	0	0
ENSG00000200852	0	0	0	0
ENSG00000200855	0	0	0	0
ENSG00000200857	0	0	0	0
ENSG00000200860	0	0	0	0
ENSG00000200867	0	0	0	0
ENSG00000200869	0	0	0	0
ENSG00000200871	0	0	0	0
ENSG00000200872	0	0	0	0
ENSG00000200873	0	0	0	0
ENSG00000200874	0	0	0	0
ENSG00000200875	0	0	0	0
ENSG00000200877	0	0	0	0
ENSG00000200879	0	0	0	0
ENSG00000200882	0	0	0	0
ENSG00000200883	0	0	0	0
ENSG00000200884	0	0	0	0
ENSG00000200885	0	0	0	0
ENSG00000200887	0	0	0	0
ENSG00000200888	0	0	0	0
ENSG00000200889	0	0	0	0
ENSG00000200890	0	0	0	0
ENSG00000200891	0	0	0	0
ENSG00000200893	0	0	0	0
ENSG00000200895	0	0	0	0
ENSG00000200897	0	0	0	0
ENSG00000200898	0	0	0	0
ENSG00000200902	0	0	0	0

ENSG00000200903	0	0	0	0
ENSG00000200906	0	0	0	0
ENSG00000200913	0	0	0	0
ENSG00000200914	0	0	0	0
ENSG00000200917	0	0	0	0
ENSG00000200922	0	0	0	0
ENSG00000200924	0	0	0	0
ENSG00000200926	0	0	0	0
ENSG00000200935	0	17	0	0
ENSG00000200941	0	0	0	0
ENSG00000200942	0	0	0	0
ENSG00000200949	0	0	0	0
ENSG00000200953	0	0	0	0
ENSG00000200954	0	0	0	0
ENSG00000200957	0	0	0	0
ENSG00000200959	0	0	0	0
ENSG00000200963	0	0	0	0
ENSG00000200966	0	0	0	0
ENSG00000200969	0	0	0	0
ENSG00000200972	0	0	0	0
ENSG00000200974	0	0	0	0
ENSG00000200975	0	0	0	0
ENSG00000200976	0	0	0	0
ENSG00000200980	0	0	0	0
ENSG00000200982	0	0	0	0
ENSG00000200983	0	0	0	0
ENSG00000200985	0	0	0	0
ENSG00000200986	0	0	0	0
ENSG00000200987	0	0	0	0
ENSG00000200991	0	0	0	0
ENSG00000200997	0	0	0	0
ENSG00000200998	0	0	0	0
ENSG00000200999	0	0	0	0
ENSG00000201000	0	0	0	0
ENSG00000201001	0	0	0	0
ENSG00000201003	0	0	0	0
ENSG00000201006	0	0	0	0
ENSG00000201009	0	0	0	0
ENSG00000201010	0	0	0	0
ENSG00000201012	0	0	0	0
ENSG00000201013	0	0	0	0
ENSG00000201014	0	0	0	0
ENSG00000201015	0	0	0	0
ENSG00000201016	0	0	0	0
ENSG00000201021	0	0	0	0
ENSG00000201023	0	0	0	0

ENSG00000201025	0	0	0	0
ENSG00000201026	0	0	0	0
ENSG00000201027	0	0	0	0
ENSG00000201028	0	0	0	0
ENSG00000201031	0	0	0	0
ENSG00000201032	0	0	0	0
ENSG00000201033	0	0	0	0
ENSG00000201034	0	0	0	0
ENSG00000201035	0	0	0	0
ENSG00000201036	0	0	0	0
ENSG00000201041	0	0	0	0
ENSG00000201042	0	0	0	0
ENSG00000201044	0	0	0	0
ENSG00000201047	0	0	0	0
ENSG00000201048	0	0	0	0
ENSG00000201050	0	0	0	0
ENSG00000201059	0	0	0	0
ENSG00000201061	0	0	0	1
ENSG00000201065	0	0	0	0
ENSG00000201066	0	0	0	0
ENSG00000201070	0	0	0	0
ENSG00000201071	0	0	0	0
ENSG00000201074	0	0	0	0
ENSG00000201075	0	0	0	0
ENSG00000201076	0	0	0	0
ENSG00000201077	0	0	0	0
ENSG00000201078	0	0	0	0
ENSG00000201080	0	0	0	0
ENSG00000201084	0	0	0	0
ENSG00000201085	0	0	0	0
ENSG00000201086	0	0	0	0
ENSG00000201088	0	0	0	0
ENSG00000201095	0	0	0	0
ENSG00000201096	0	0	0	0
ENSG00000201097	0	0	0	0
ENSG00000201098	0	16	32	43
ENSG00000201102	0	0	0	0
ENSG00000201103	0	0	0	0
ENSG00000201104	0	0	0	0
ENSG00000201105	0	0	0	0
ENSG00000201109	0	0	0	0
ENSG00000201113	0	0	0	0
ENSG00000201114	0	0	0	0
ENSG00000201118	0	0	0	0
ENSG00000201119	0	0	0	0
ENSG00000201121	0	0	0	0

ENSG00000201126	0	0	0	0
ENSG00000201129	0	0	0	0
ENSG00000201133	0	0	0	0
ENSG00000201134	0	0	0	0
ENSG00000201135	0	0	0	0
ENSG00000201136	0	0	0	0
ENSG00000201138	0	0	0	0
ENSG00000201140	0	0	0	0
ENSG00000201142	0	0	0	0
ENSG00000201143	0	0	0	0
ENSG00000201145	0	0	0	0
ENSG00000201148	0	0	0	0
ENSG00000201151	0	0	0	0
ENSG00000201153	0	0	0	0
ENSG00000201155	0	0	0	0
ENSG00000201157	0	0	0	0
ENSG00000201160	0	0	0	0
ENSG00000201161	0	0	0	0
ENSG00000201162	0	0	0	0
ENSG00000201164	0	0	0	0
ENSG00000201165	0	0	0	0
ENSG00000201168	0	0	0	0
ENSG00000201170	0	0	0	0
ENSG00000201176	0	0	0	0
ENSG00000201178	0	0	0	0
ENSG00000201179	0	0	0	0
ENSG00000201180	0	0	0	0
ENSG00000201182	0	0	0	0
ENSG00000201183	0	0	0	0
ENSG00000201184	0	0	0	0
ENSG00000201185	0	0	0	0
ENSG00000201186	0	0	0	0
ENSG00000201196	0	0	0	0
ENSG00000201198	0	0	0	0
ENSG00000201199	0	0	0	0
ENSG00000201201	0	0	0	0
ENSG00000201204	0	0	0	0
ENSG00000201207	0	0	0	0
ENSG00000201208	0	0	0	0
ENSG00000201209	0	0	0	0
ENSG00000201210	0	0	0	0
ENSG00000201216	0	0	0	0
ENSG00000201217	0	0	0	0
ENSG00000201218	0	0	0	0
ENSG00000201221	0	0	0	0
ENSG00000201223	0	0	0	0

ENSG00000201227	2	0	3	0
ENSG00000201228	0	0	0	0
ENSG00000201229	0	0	0	0
ENSG00000201231	0	0	0	0
ENSG00000201239	0	0	0	0
ENSG00000201240	0	0	0	0
ENSG00000201241	0	0	0	0
ENSG00000201242	0	0	0	0
ENSG00000201243	0	0	1	0
ENSG00000201245	0	0	0	0
ENSG00000201247	0	0	0	0
ENSG00000201253	0	0	0	0
ENSG00000201255	0	0	0	0
ENSG00000201260	0	0	0	0
ENSG00000201263	0	0	0	0
ENSG00000201264	0	0	0	0
ENSG00000201270	0	0	0	0
ENSG00000201271	0	0	0	0
ENSG00000201273	0	0	0	0
ENSG00000201274	0	0	0	0
ENSG00000201277	0	0	0	0
ENSG00000201279	0	0	0	0
ENSG00000201282	0	0	0	0
ENSG00000201283	0	0	0	0
ENSG00000201285	0	0	0	0
ENSG00000201287	0	0	0	0
ENSG00000201288	0	0	0	0
ENSG00000201289	0	0	0	0
ENSG00000201291	0	0	0	0
ENSG00000201292	0	0	0	0
ENSG00000201294	0	0	0	0
ENSG00000201296	0	0	0	0
ENSG00000201297	0	0	0	0
ENSG00000201298	0	0	0	0
ENSG00000201300	0	0	0	0
ENSG00000201301	0	0	0	0
ENSG00000201302	0	0	0	0
ENSG00000201308	0	0	0	0
ENSG00000201309	0	0	0	0
ENSG00000201311	0	0	0	0
ENSG00000201312	0	0	0	0
ENSG00000201314	0	0	0	0
ENSG00000201315	0	0	0	0
ENSG00000201316	0	0	0	0
ENSG00000201317	0	0	0	0
ENSG00000201318	0	0	0	0

ENSG00000201321	0	0	0	0
ENSG00000201324	0	0	0	0
ENSG00000201325	0	0	0	0
ENSG00000201326	0	0	0	0
ENSG00000201329	0	0	0	0
ENSG00000201330	0	0	0	0
ENSG00000201331	0	0	0	0
ENSG00000201339	0	0	0	0
ENSG00000201340	0	0	0	0
ENSG00000201341	0	0	0	0
ENSG00000201342	0	0	0	0
ENSG00000201343	0	0	0	0
ENSG00000201346	0	0	0	0
ENSG00000201347	0	0	0	0
ENSG00000201348	0	0	0	0
ENSG00000201351	0	0	0	0
ENSG00000201354	0	0	0	0
ENSG00000201355	0	0	0	0
ENSG00000201356	0	0	0	0
ENSG00000201358	0	0	0	0
ENSG00000201361	0	0	0	0
ENSG00000201363	0	0	0	0
ENSG00000201364	0	0	0	0
ENSG00000201365	0	0	0	0
ENSG00000201367	0	0	0	0
ENSG00000201368	0	0	0	0
ENSG00000201370	0	0	0	0
ENSG00000201371	0	0	0	0
ENSG00000201372	0	0	0	0
ENSG00000201376	0	0	0	0
ENSG00000201377	0	0	0	0
ENSG00000201378	0	0	0	0
ENSG00000201379	0	0	0	0
ENSG00000201382	0	0	0	0
ENSG00000201384	0	0	0	0
ENSG00000201386	0	0	0	0
ENSG00000201388	0	0	0	0
ENSG00000201390	0	0	0	0
ENSG00000201392	0	0	0	0
ENSG00000201393	0	0	0	0
ENSG00000201394	0	0	0	0
ENSG00000201395	0	0	0	0
ENSG00000201398	0	0	0	0
ENSG00000201403	0	0	0	0
ENSG00000201405	0	0	0	0
ENSG00000201407	0	0	0	0

ENSG00000201410	0	0	0	0
ENSG00000201412	0	0	0	0
ENSG00000201413	0	0	0	0
ENSG00000201415	0	0	0	0
ENSG00000201416	0	0	0	0
ENSG00000201420	0	0	0	0
ENSG00000201421	0	0	0	0
ENSG00000201423	0	0	0	0
ENSG00000201425	0	0	0	0
ENSG00000201426	0	0	0	0
ENSG00000201428	0	1	0	1
ENSG00000201431	0	0	0	0
ENSG00000201432	0	0	0	0
ENSG00000201433	0	0	0	0
ENSG00000201435	0	0	0	0
ENSG00000201436	0	0	0	0
ENSG00000201439	0	0	0	0
ENSG00000201440	0	0	0	0
ENSG00000201441	0	0	0	0
ENSG00000201442	0	0	0	0
ENSG00000201443	0	0	0	0
ENSG00000201444	0	0	0	0
ENSG00000201447	0	0	0	0
ENSG00000201448	0	0	0	0
ENSG00000201450	0	0	0	0
ENSG00000201451	0	0	0	0
ENSG00000201452	0	0	0	0
ENSG00000201457	0	0	0	0
ENSG00000201458	0	0	0	0
ENSG00000201464	0	0	0	0
ENSG00000201465	0	0	0	0
ENSG00000201466	0	0	0	0
ENSG00000201467	0	0	0	0
ENSG00000201469	0	0	0	0
ENSG00000201470	0	0	0	0
ENSG00000201474	0	0	0	0
ENSG00000201476	0	0	0	0
ENSG00000201482	0	0	0	0
ENSG00000201483	0	0	0	0
ENSG00000201487	0	0	0	0
ENSG00000201489	0	0	0	0
ENSG00000201491	0	0	0	0
ENSG00000201492	0	0	0	0
ENSG00000201493	0	0	0	0
ENSG00000201496	0	0	0	0
ENSG00000201498	0	0	0	0

ENSG00000201499	0	0	0	0
ENSG00000201500	0	0	0	0
ENSG00000201501	0	0	0	0
ENSG00000201502	0	0	0	0
ENSG00000201509	0	0	0	0
ENSG00000201510	0	0	0	0
ENSG00000201511	0	0	0	0
ENSG00000201512	0	0	0	0
ENSG00000201516	0	0	0	0
ENSG00000201517	0	0	0	0
ENSG00000201518	0	0	0	0
ENSG00000201519	0	0	0	0
ENSG00000201523	0	0	0	0
ENSG00000201524	0	0	0	0
ENSG00000201527	0	0	0	0
ENSG00000201529	0	0	0	0
ENSG00000201532	0	0	0	0
ENSG00000201533	0	0	0	0
ENSG00000201535	0	0	0	0
ENSG00000201541	0	0	0	0
ENSG00000201542	0	0	0	0
ENSG00000201544	0	0	0	0
ENSG00000201545	0	0	0	0
ENSG00000201547	0	0	0	0
ENSG00000201548	0	0	0	0
ENSG00000201549	0	0	0	0
ENSG00000201550	0	0	0	0
ENSG00000201554	0	0	0	0
ENSG00000201555	0	0	0	0
ENSG00000201557	0	0	0	0
ENSG00000201558	0	0	0	0
ENSG00000201560	0	0	0	0
ENSG00000201563	0	0	0	0
ENSG00000201564	0	0	0	0
ENSG00000201565	0	0	0	0
ENSG00000201566	0	0	0	0
ENSG00000201567	0	0	0	0
ENSG00000201569	0	0	0	0
ENSG00000201570	0	0	0	0
ENSG00000201573	0	0	0	0
ENSG00000201574	0	0	0	0
ENSG00000201579	0	0	0	0
ENSG00000201581	0	0	0	0
ENSG00000201583	0	0	0	0
ENSG00000201584	0	0	0	0
ENSG00000201586	0	0	0	0

ENSG00000201588	0	0	0	0
ENSG00000201591	0	0	0	0
ENSG00000201592	0	0	0	0
ENSG00000201594	0	0	0	0
ENSG00000201595	0	0	0	0
ENSG00000201596	0	0	0	0
ENSG00000201598	0	0	0	0
ENSG00000201599	0	0	0	0
ENSG00000201600	0	0	0	0
ENSG00000201602	0	0	0	0
ENSG00000201604	0	0	0	0
ENSG00000201607	0	0	0	0
ENSG00000201608	0	0	0	0
ENSG00000201609	0	0	0	0
ENSG00000201610	0	0	0	0
ENSG00000201612	0	0	0	0
ENSG00000201613	0	0	0	0
ENSG00000201614	0	0	0	0
ENSG00000201616	0	0	0	0
ENSG00000201618	0	0	0	0
ENSG00000201619	0	0	0	0
ENSG00000201620	0	0	0	0
ENSG00000201622	0	0	0	0
ENSG00000201623	0	0	0	0
ENSG00000201624	0	0	0	0
ENSG00000201627	0	0	0	0
ENSG00000201628	0	0	0	0
ENSG00000201633	0	0	0	0
ENSG00000201634	0	0	0	0
ENSG00000201635	0	0	0	0
ENSG00000201638	0	0	0	0
ENSG00000201640	0	0	0	0
ENSG00000201641	0	0	0	0
ENSG00000201642	0	0	0	0
ENSG00000201643	0	0	0	0
ENSG00000201644	0	0	0	0
ENSG00000201648	0	0	1	0
ENSG00000201649	0	0	0	0
ENSG00000201654	0	0	0	0
ENSG00000201658	0	0	0	0
ENSG00000201659	0	0	0	0
ENSG00000201660	0	0	0	0
ENSG00000201662	0	0	0	1
ENSG00000201663	0	0	0	0
ENSG00000201665	0	0	0	0
ENSG00000201666	0	0	0	0

ENSG00000201668	0	0	0	0
ENSG00000201669	0	0	0	0
ENSG00000201671	0	0	0	0
ENSG00000201672	0	0	0	0
ENSG00000201674	0	0	0	0
ENSG00000201675	0	0	0	0
ENSG00000201676	0	0	0	0
ENSG00000201678	0	0	0	0
ENSG00000201679	0	0	0	0
ENSG00000201680	0	0	0	0
ENSG00000201683	0	0	0	0
ENSG00000201684	0	0	0	0
ENSG00000201686	0	0	0	0
ENSG00000201687	0	0	0	0
ENSG00000201689	0	0	0	0
ENSG00000201690	0	0	0	0
ENSG00000201692	0	0	0	0
ENSG00000201695	0	0	0	0
ENSG00000201699	2	33	2	1
ENSG00000201700	0	0	0	0
ENSG00000201701	0	0	0	0
ENSG00000201704	0	0	0	0
ENSG00000201707	0	0	0	0
ENSG00000201708	0	0	0	0
ENSG00000201709	0	0	0	0
ENSG00000201710	0	0	0	0
ENSG00000201711	0	0	0	0
ENSG00000201713	0	0	0	0
ENSG00000201715	0	0	0	0
ENSG00000201719	0	0	0	0
ENSG00000201723	0	0	0	0
ENSG00000201724	0	0	0	0
ENSG00000201725	0	0	0	0
ENSG00000201727	0	0	0	0
ENSG00000201728	0	0	0	0
ENSG00000201733	0	0	0	0
ENSG00000201736	0	0	0	0
ENSG00000201737	0	0	0	0
ENSG00000201741	0	0	0	0
ENSG00000201742	0	0	0	0
ENSG00000201744	0	0	0	0
ENSG00000201746	0	0	0	0
ENSG00000201747	0	0	0	0
ENSG00000201749	0	0	0	0
ENSG00000201750	0	0	0	0
ENSG00000201752	0	0	0	0

ENSG00000201754	0	0	0	0
ENSG00000201756	0	0	0	0
ENSG00000201758	0	0	0	0
ENSG00000201761	0	0	0	0
ENSG00000201763	0	0	0	0
ENSG00000201766	0	0	0	0
ENSG00000201770	0	9	0	1
ENSG00000201772	0	0	0	0
ENSG00000201774	0	0	0	0
ENSG00000201775	0	0	0	0
ENSG00000201778	0	0	0	0
ENSG00000201780	0	0	0	0
ENSG00000201782	0	0	0	0
ENSG00000201784	0	0	0	0
ENSG00000201785	0	0	0	0
ENSG00000201786	0	0	0	0
ENSG00000201788	0	0	0	0
ENSG00000201789	0	0	0	0
ENSG00000201790	0	0	0	0
ENSG00000201791	0	0	0	0
ENSG00000201792	0	0	0	0
ENSG00000201793	0	2	4	15
ENSG00000201794	0	0	0	0
ENSG00000201796	0	0	0	0
ENSG00000201800	0	0	0	0
ENSG00000201801	0	0	0	0
ENSG00000201805	0	0	0	0
ENSG00000201806	0	0	0	0
ENSG00000201807	0	0	0	0
ENSG00000201808	0	0	0	0
ENSG00000201809	0	0	0	0
ENSG00000201810	0	0	0	0
ENSG00000201811	0	0	0	0
ENSG00000201812	0	0	0	0
ENSG00000201813	0	0	0	0
ENSG00000201815	0	0	0	0
ENSG00000201816	0	0	0	0
ENSG00000201818	0	0	0	0
ENSG00000201820	0	0	0	0
ENSG00000201821	0	0	0	0
ENSG00000201822	0	0	0	0
ENSG00000201823	0	0	0	0
ENSG00000201825	0	0	0	0
ENSG00000201826	0	0	0	0
ENSG00000201827	0	0	0	0
ENSG00000201830	0	0	0	0

ENSG00000201831	0	0	0	0
ENSG00000201838	0	0	0	0
ENSG00000201839	0	0	0	0
ENSG00000201843	0	0	0	0
ENSG00000201846	0	0	0	0
ENSG00000201847	0	0	0	0
ENSG00000201850	0	0	0	0
ENSG00000201852	0	0	0	0
ENSG00000201853	0	0	0	0
ENSG00000201856	0	0	0	0
ENSG00000201860	0	0	0	0
ENSG00000201861	0	0	0	0
ENSG00000201862	0	0	0	0
ENSG00000201863	0	0	0	0
ENSG00000201865	0	0	0	0
ENSG00000201867	0	0	0	0
ENSG00000201868	0	0	0	0
ENSG00000201869	0	0	0	0
ENSG00000201875	0	0	0	0
ENSG00000201876	0	0	0	0
ENSG00000201881	0	0	0	0
ENSG00000201882	0	0	0	0
ENSG00000201884	0	0	0	0
ENSG00000201885	0	0	0	0
ENSG00000201892	0	0	0	0
ENSG00000201894	0	0	0	0
ENSG00000201896	0	0	0	0
ENSG00000201898	0	0	0	0
ENSG00000201899	0	0	0	0
ENSG00000201900	0	0	0	0
ENSG00000201901	0	0	0	0
ENSG00000201902	0	0	0	0
ENSG00000201907	0	0	0	0
ENSG00000201909	0	0	0	0
ENSG00000201910	0	0	0	0
ENSG00000201912	0	0	0	0
ENSG00000201913	0	0	0	0
ENSG00000201916	0	0	0	0
ENSG00000201919	0	0	0	0
ENSG00000201920	0	0	0	0
ENSG00000201922	0	0	0	0
ENSG00000201923	0	0	0	0
ENSG00000201925	0	0	0	0
ENSG00000201931	0	0	0	0
ENSG00000201933	0	0	0	0
ENSG00000201937	0	0	0	0

ENSG00000201938	0	0	0	0
ENSG00000201939	0	0	0	0
ENSG00000201942	0	0	0	0
ENSG00000201943	0	0	0	0
ENSG00000201944	0	0	0	0
ENSG00000201945	0	0	0	0
ENSG00000201950	0	0	0	0
ENSG00000201954	0	0	0	0
ENSG00000201955	0	0	0	0
ENSG00000201957	0	0	0	0
ENSG00000201959	0	0	0	0
ENSG00000201962	0	0	0	0
ENSG00000201965	0	0	0	0
ENSG00000201966	0	0	0	0
ENSG00000201967	0	0	0	0
ENSG00000201968	0	0	0	0
ENSG00000201969	0	0	0	0
ENSG00000201976	0	0	0	0
ENSG00000201980	0	0	0	0
ENSG00000201984	0	0	0	0
ENSG00000201987	0	0	0	0
ENSG00000201988	0	0	0	0
ENSG00000201990	0	0	0	0
ENSG00000201992	0	0	0	0
ENSG00000201998	0	0	1	5
ENSG00000201999	0	0	0	0
ENSG00000202000	0	0	0	0
ENSG00000202001	0	0	0	0
ENSG00000202008	0	0	0	0
ENSG00000202012	0	0	0	0
ENSG00000202014	0	0	0	0
ENSG00000202016	0	0	0	0
ENSG00000202017	0	0	0	0
ENSG00000202019	0	0	0	0
ENSG00000202021	0	0	0	0
ENSG00000202023	0	0	0	0
ENSG00000202024	0	0	0	0
ENSG00000202025	0	0	0	0
ENSG00000202026	0	0	0	0
ENSG00000202027	0	0	0	0
ENSG00000202029	0	0	0	0
ENSG00000202030	0	0	0	0
ENSG00000202031	0	0	0	0
ENSG00000202034	0	0	0	0
ENSG00000202035	0	0	0	0
ENSG00000202039	0	0	0	0

ENSG00000202041	0	0	0	0
ENSG00000202042	0	0	0	0
ENSG00000202044	0	0	0	0
ENSG00000202046	0	0	0	0
ENSG00000202047	0	0	0	0
ENSG00000202048	0	0	0	0
ENSG00000202050	0	0	0	0
ENSG00000202051	0	0	0	0
ENSG00000202054	0	0	0	0
ENSG00000202056	0	0	0	0
ENSG00000202058	0	0	0	0
ENSG00000202059	0	0	0	0
ENSG00000202060	0	0	0	0
ENSG00000202063	0	0	0	0
ENSG00000202064	0	0	0	0
ENSG00000202067	0	0	0	0
ENSG00000202069	0	0	0	0
ENSG00000202070	0	0	0	0
ENSG00000202071	0	0	0	0
ENSG00000202072	0	0	0	0
ENSG00000202074	0	0	0	0
ENSG00000202077	0	2	0	0
ENSG00000202078	5	55	42	45
ENSG00000202079	0	0	0	0
ENSG00000202081	0	0	0	0
ENSG00000202082	0	0	0	0
ENSG00000202089	0	0	0	0
ENSG00000202092	0	0	0	0
ENSG00000202093	0	0	0	0
ENSG00000202095	0	0	0	0
ENSG00000202099	0	0	0	0
ENSG00000202100	0	0	0	0
ENSG00000202103	0	0	0	0
ENSG00000202105	0	0	0	0
ENSG00000202107	0	0	0	0
ENSG00000202111	2	0	0	0
ENSG00000202112	0	0	0	0
ENSG00000202119	0	0	0	0
ENSG00000202124	0	0	0	0
ENSG00000202125	0	0	0	0
ENSG00000202129	0	0	0	0
ENSG00000202137	0	0	0	0
ENSG00000202140	0	0	0	0
ENSG00000202141	0	0	0	0
ENSG00000202142	0	0	0	0
ENSG00000202144	0	0	0	0

ENSG00000202146	0	0	0	0
ENSG00000202147	0	0	0	0
ENSG00000202150	0	0	0	0
ENSG00000202151	0	0	0	0
ENSG00000202157	0	0	0	0
ENSG00000202159	0	0	0	0
ENSG00000202160	0	0	0	0
ENSG00000202164	0	0	0	0
ENSG00000202167	0	0	0	0
ENSG00000202169	0	0	0	0
ENSG00000202172	0	0	0	0
ENSG00000202174	0	0	0	0
ENSG00000202175	0	0	0	0
ENSG00000202176	0	0	0	0
ENSG00000202177	0	0	0	0
ENSG00000202178	0	0	0	0
ENSG00000202181	0	0	0	0
ENSG00000202182	0	0	0	0
ENSG00000202183	0	0	0	0
ENSG00000202184	0	0	0	0
ENSG00000202186	0	0	0	0
ENSG00000202187	0	0	0	0
ENSG00000202188	0	0	0	0
ENSG00000202189	0	0	0	0
ENSG00000202190	0	0	0	0
ENSG00000202191	0	0	0	0
ENSG00000202193	0	0	0	0
ENSG00000202195	0	0	0	0
ENSG00000202198	0	46997	32301	42114
ENSG00000202199	0	0	0	0
ENSG00000202200	0	0	0	0
ENSG00000202205	0	0	0	0
ENSG00000202206	0	0	0	0
ENSG00000202211	0	0	0	0
ENSG00000202215	0	0	0	0
ENSG00000202216	0	0	0	0
ENSG00000202217	0	0	0	0
ENSG00000202220	0	0	0	0
ENSG00000202222	0	0	0	0
ENSG00000202224	0	0	0	0
ENSG00000202225	0	0	0	0
ENSG00000202227	0	0	0	0
ENSG00000202229	0	0	0	0
ENSG00000202231	0	0	0	0
ENSG00000202233	0	0	0	0
ENSG00000202237	0	0	0	0

ENSG00000202239	0	0	0	0
ENSG00000202240	0	0	0	0
ENSG00000202241	0	0	0	0
ENSG00000202242	0	0	0	0
ENSG00000202245	0	0	0	0
ENSG00000202248	0	0	0	0
ENSG00000202249	0	0	0	0
ENSG00000202250	0	0	0	0
ENSG00000202251	0	0	0	0
ENSG00000202252	0	0	0	0
ENSG00000202254	0	0	0	0
ENSG00000202255	0	0	0	0
ENSG00000202257	0	0	0	0
ENSG00000202259	0	0	0	0
ENSG00000202260	0	0	0	0
ENSG00000202261	0	0	0	0
ENSG00000202263	0	0	0	0
ENSG00000202264	0	0	0	0
ENSG00000202265	0	0	0	0
ENSG00000202268	2	0	8	2
ENSG00000202269	0	0	0	0
ENSG00000202270	0	0	0	0
ENSG00000202272	0	0	0	0
ENSG00000202273	0	0	0	0
ENSG00000202275	0	0	0	0
ENSG00000202276	0	0	0	0
ENSG00000202279	0	0	0	0
ENSG00000202281	0	0	0	0
ENSG00000202283	0	0	0	0
ENSG00000202285	0	0	0	0
ENSG00000202290	0	0	0	0
ENSG00000202293	0	0	0	0
ENSG00000202294	0	0	0	0
ENSG00000202296	0	0	0	0
ENSG00000202297	0	0	0	0
ENSG00000202300	0	0	0	0
ENSG00000202304	0	0	0	0
ENSG00000202306	0	0	0	0
ENSG00000202308	0	0	0	0
ENSG00000202309	0	0	0	0
ENSG00000202310	0	0	0	0
ENSG00000202313	0	0	0	0
ENSG00000202314	0	0	0	0
ENSG00000202317	0	0	0	0
ENSG00000202318	0	0	0	0
ENSG00000202322	0	0	0	0

ENSG00000202323	0	0	0	0
ENSG00000202324	0	0	0	0
ENSG00000202329	0	0	0	0
ENSG00000202331	0	0	0	0
ENSG00000202332	0	0	0	0
ENSG00000202334	0	0	0	0
ENSG00000202335	0	0	0	0
ENSG00000202336	0	0	0	0
ENSG00000202337	0	0	8	1
ENSG00000202339	0	0	0	0
ENSG00000202341	0	0	0	0
ENSG00000202343	0	0	1	0
ENSG00000202344	0	0	0	0
ENSG00000202345	0	0	0	0
ENSG00000202347	0	0	0	0
ENSG00000202350	0	0	0	0
ENSG00000202351	0	0	0	0
ENSG00000202354	1	16	32	30
ENSG00000202356	0	0	0	0
ENSG00000202357	0	0	0	0
ENSG00000202358	0	0	0	0
ENSG00000202360	2	0	4	19
ENSG00000202361	0	0	0	0
ENSG00000202363	0	0	0	0
ENSG00000202364	0	0	0	0
ENSG00000202368	0	0	0	0
ENSG00000202373	0	0	0	0
ENSG00000202374	0	14	2	5
ENSG00000202377	0	0	0	0
ENSG00000202379	0	0	0	0
ENSG00000202380	0	0	0	0
ENSG00000202382	0	0	1	0
ENSG00000202383	0	0	0	0
ENSG00000202385	0	0	0	0
ENSG00000202386	0	0	0	0
ENSG00000202388	0	0	0	0
ENSG00000202389	0	0	0	0
ENSG00000202392	0	0	0	0
ENSG00000202394	0	0	0	0
ENSG00000202395	0	0	0	0
ENSG00000202398	0	0	0	0
ENSG00000202399	0	0	2	0
ENSG00000202400	0	0	0	9
ENSG00000202402	0	0	0	0
ENSG00000202406	0	0	0	0
ENSG00000202407	0	0	0	0

ENSG00000202408	0	0	0	3
ENSG00000202410	0	0	0	0
ENSG00000202411	0	0	0	0
ENSG00000202412	0	0	0	0
ENSG00000202414	0	0	0	0
ENSG00000202415	0	0	3	0
ENSG00000202417	0	0	0	0
ENSG00000202422	0	0	0	0
ENSG00000202423	0	0	0	0
ENSG00000202427	0	0	0	0
ENSG00000202428	0	0	0	0
ENSG00000202429	0	0	0	0
ENSG00000202430	0	0	0	0
ENSG00000202431	0	0	0	0
ENSG00000202433	0	0	0	0
ENSG00000202434	0	0	0	0
ENSG00000202438	0	0	0	0
ENSG00000202440	0	0	0	0
ENSG00000202441	0	0	0	0
ENSG00000202444	0	0	0	0
ENSG00000202445	0	0	0	0
ENSG00000202449	0	0	0	0
ENSG00000202459	0	0	0	0
ENSG00000202461	0	0	0	0
ENSG00000202468	0	0	0	0
ENSG00000202469	0	0	0	0
ENSG00000202470	0	0	0	0
ENSG00000202471	0	0	0	0
ENSG00000202472	0	0	0	0
ENSG00000202473	0	0	0	0
ENSG00000202474	0	0	0	0
ENSG00000202476	0	0	0	0
ENSG00000202477	0	0	0	0
ENSG00000202478	0	0	0	0
ENSG00000202479	0	0	0	0
ENSG00000202482	0	0	0	0
ENSG00000202485	0	0	0	0
ENSG00000202487	0	0	0	0
ENSG00000202490	0	0	0	0
ENSG00000202491	0	0	0	0
ENSG00000202495	0	0	0	0
ENSG00000202496	0	0	0	0
ENSG00000202497	0	0	0	0
ENSG00000202498	0	0	0	0
ENSG00000202499	0	0	0	0
ENSG00000202502	0	0	0	0

ENSG00000202503	0	0	0	0
ENSG00000202508	0	0	0	0
ENSG00000202512	1	0	0	0
ENSG00000202513	0	0	0	0
ENSG00000202514	0	0	0	0
ENSG00000202515	0	0	0	0
ENSG00000202517	0	0	0	0
ENSG00000202521	0	0	0	0
ENSG00000202522	0	0	0	0
ENSG00000202523	0	0	0	0
ENSG00000202526	0	0	0	0
ENSG00000202528	0	0	0	0
ENSG00000202529	0	0	0	0
ENSG00000202532	0	0	0	0
ENSG00000202533	0	0	0	0
ENSG00000202534	0	0	0	0
ENSG00000202536	0	0	0	0
ENSG00000202537	0	0	0	0
ENSG00000202538	0	0	1	0
ENSG00000202542	0	0	0	0
ENSG00000202560	0	0	0	0
ENSG00000202566	0	0	1	0
ENSG00000202569	0	0	0	0
ENSG00000202601	0	0	0	0
ENSG00000202609	0	0	0	0
ENSG00000202641	0	0	0	0
ENSG00000203257	0	0	0	0
ENSG00000203258	0	0	0	0
ENSG00000203260	0	0	0	0
ENSG00000203262	0	0	0	0
ENSG00000203266	0	0	0	0
ENSG00000203268	0	0	0	0
ENSG00000203276	0	0	0	0
ENSG00000203279	1	0	0	0
ENSG00000203280	0	0	0	0
ENSG00000203281	0	0	0	0
ENSG00000203284	0	0	0	0
ENSG00000203285	0	0	0	0
ENSG00000203286	0	0	0	3
ENSG00000203288	1	0	3	0
ENSG00000203301	73	0	13	11
ENSG00000203305	0	0	0	0
ENSG00000203306	2	37	53	17
ENSG00000203307	0	0	0	0
ENSG00000203314	0	0	0	0
ENSG00000203315	0	0	0	0

ENSG00000203316	0	0	0	0
ENSG00000203317	0	0	0	0
ENSG00000203318	0	0	0	0
ENSG00000203321	0	0	0	0
ENSG00000203325	14	13	0	0
ENSG00000203326	4	701	671	1161
ENSG00000203327	0	0	0	0
ENSG00000203334	0	0	0	0
ENSG00000203335	0	0	1	0
ENSG00000203341	1	0	0	0
ENSG00000203343	0	0	0	0
ENSG00000203344	0	0	0	0
ENSG00000203346	0	0	0	0
ENSG00000203356	7	3	3	11
ENSG00000203362	0	0	0	0
ENSG00000203363	0	0	0	0
ENSG00000203364	1	0	0	0
ENSG00000203372	0	0	0	0
ENSG00000203377	0	0	0	0
ENSG00000203384	0	0	0	0
ENSG00000203386	0	0	0	4
ENSG00000203387	0	0	0	0
ENSG00000203392	0	0	0	0
ENSG00000203394	0	15	11	3
ENSG00000203395	49	40	70	64
ENSG00000203396	65	0	0	0
ENSG00000203397	0	0	0	0
ENSG00000203400	0	0	0	0
ENSG00000203401	0	0	0	0
ENSG00000203402	3	0	0	0
ENSG00000203403	0	0	0	0
ENSG00000203408	0	0	0	0
ENSG00000203411	0	0	0	0
ENSG00000203413	0	0	0	0
ENSG00000203414	0	0	1	0
ENSG00000203415	0	0	0	0
ENSG00000203416	0	0	0	0
ENSG00000203418	0	0	0	0
ENSG00000203421	0	0	0	0
ENSG00000203422	0	0	0	0
ENSG00000203423	0	0	0	0
ENSG00000203424	0	0	0	0
ENSG00000203425	0	0	0	0
ENSG00000203426	0	0	0	0
ENSG00000203427	0	0	0	0
ENSG00000203428	0	0	0	0

ENSG00000203430	0	0	0	0
ENSG00000203431	0	0	0	0
ENSG00000203432	0	0	0	0
ENSG00000203433	0	0	0	0
ENSG00000203434	0	0	0	0
ENSG00000203435	2	0	0	0
ENSG00000203436	0	0	0	0
ENSG00000203437	0	0	0	0
ENSG00000203438	0	0	0	0
ENSG00000203441	1	0	2	3
ENSG00000203442	0	0	0	0
ENSG00000203443	0	0	0	0
ENSG00000203446	0	0	0	2
ENSG00000203457	0	0	0	0
ENSG00000203462	0	0	0	3
ENSG00000203463	0	0	0	0
ENSG00000203465	0	0	0	0
ENSG00000203469	0	0	0	0
ENSG00000203471	0	0	0	0
ENSG00000203472	0	0	0	0
ENSG00000203473	0	0	0	0
ENSG00000203477	0	0	0	0
ENSG00000203478	0	0	0	0
ENSG00000203479	0	0	0	0
ENSG00000203480	0	0	0	0
ENSG00000203482	0	0	0	0
ENSG00000203483	0	0	0	0
ENSG00000203484	0	0	2	0
ENSG00000203485	389	2012	792	1158
ENSG00000203489	0	0	0	0
ENSG00000203492	0	0	0	0
ENSG00000203496	0	0	0	0
ENSG00000203497	54	46	9	26
ENSG00000203498	0	0	0	0
ENSG00000203499	0	0	0	0
ENSG00000203504	0	0	0	0
ENSG00000203505	0	0	0	0
ENSG00000203506	0	0	0	0
ENSG00000203507	0	0	0	0
ENSG00000203511	0	0	0	0
ENSG00000203512	0	0	0	0
ENSG00000203513	0	0	0	0
ENSG00000203518	0	0	0	0
ENSG00000203520	0	0	0	0
ENSG00000203523	0	0	0	0
ENSG00000203527	0	0	0	0

ENSG00000203531	0	0	0	0
ENSG00000203542	0	0	0	0
ENSG00000203543	6	0	0	0
ENSG00000203544	0	0	0	0
ENSG00000203546	1	0	3	0
ENSG00000203547	0	0	0	0
ENSG00000203560	0	0	0	0
ENSG00000203562	0	0	0	0
ENSG00000203563	0	0	0	0
ENSG00000203565	0	0	0	0
ENSG00000203572	0	0	0	0
ENSG00000203573	0	0	1	0
ENSG00000203574	0	0	0	0
ENSG00000203575	0	0	0	0
ENSG00000203576	0	0	0	0
ENSG00000203578	0	0	0	0
ENSG00000203581	0	0	0	0
ENSG00000203583	0	0	0	0
ENSG00000203585	0	0	0	2
ENSG00000203588	0	0	0	0
ENSG00000203589	0	0	0	0
ENSG00000203593	1	0	0	0
ENSG00000203594	0	0	0	0
ENSG00000203601	0	0	0	0
ENSG00000203602	0	0	13	0
ENSG00000203605	0	0	0	0
ENSG00000203606	0	0	0	0
ENSG00000203614	0	0	0	0
ENSG00000203615	0	0	0	0
ENSG00000203616	0	0	0	0
ENSG00000203618	0	0	0	0
ENSG00000203620	3	0	3	1
ENSG00000203623	0	0	0	0
ENSG00000203624	0	0	0	0
ENSG00000203630	0	0	0	0
ENSG00000203632	2	0	2	5
ENSG00000203635	1	0	0	0
ENSG00000203643	0	0	0	0
ENSG00000203644	4	104	79	95
ENSG00000203645	2	0	0	0
ENSG00000203647	0	0	0	0
ENSG00000203648	0	0	0	0
ENSG00000203650	1	0	5	3
ENSG00000203652	0	0	0	0
ENSG00000203659	0	2	7	9
ENSG00000203661	0	0	0	0

ENSG00000203663	0	0	19	10
ENSG00000203664	0	0	0	0
ENSG00000203666	142	413	986	1368
ENSG00000203667	0	106	142	206
ENSG00000203668	34	293	393	234
ENSG00000203684	0	0	0	0
ENSG00000203685	1	13	0	6
ENSG00000203688	0	0	0	0
ENSG00000203690	0	0	0	0
ENSG00000203691	0	0	0	0
ENSG00000203697	28	19	32	30
ENSG00000203705	36	124	248	165
ENSG00000203706	0	0	0	0
ENSG00000203709	0	9	5	21
ENSG00000203710	12	100	131	170
ENSG00000203711	0	0	0	0
ENSG00000203721	0	0	3	1
ENSG00000203722	0	0	0	0
ENSG00000203724	0	0	0	0
ENSG00000203727	0	0	0	0
ENSG00000203729	0	0	0	0
ENSG00000203730	0	0	0	0
ENSG00000203733	0	0	0	0
ENSG00000203734	0	0	0	0
ENSG00000203737	0	0	1	0
ENSG00000203739	4	94	54	135
ENSG00000203740	0	0	0	4
ENSG00000203747	118	1	1	0
ENSG00000203756	0	0	0	0
ENSG00000203757	0	0	0	0
ENSG00000203758	0	0	0	0
ENSG00000203760	5	0	5	4
ENSG00000203761	1	0	0	0
ENSG00000203772	0	0	0	0
ENSG00000203778	0	0	0	0
ENSG00000203780	1	0	1	2
ENSG00000203781	0	0	0	0
ENSG00000203782	0	0	0	0
ENSG00000203783	0	0	0	0
ENSG00000203784	0	0	0	0
ENSG00000203785	0	0	0	0
ENSG00000203786	0	13	0	0
ENSG00000203791	4	27	49	73
ENSG00000203795	0	0	0	0
ENSG00000203797	0	8	6	23
ENSG00000203799	1	0	5	13

ENSG00000203801	0	0	2	0
ENSG00000203804	1	0	4	0
ENSG00000203805	0	0	0	0
ENSG00000203808	0	0	0	0
ENSG00000203809	0	0	0	0
ENSG00000203811	0	0	0	0
ENSG00000203812	0	57	47	41
ENSG00000203813	4797	11964	13049	7658
ENSG00000203814	6	89	80	55
ENSG00000203815	0	0	0	0
ENSG00000203817	0	0	0	2
ENSG00000203818	0	0	0	0
ENSG00000203819	0	0	1	3
ENSG00000203825	0	0	0	0
ENSG00000203827	0	0	0	1
ENSG00000203832	0	0	4	9
ENSG00000203835	0	0	0	0
ENSG00000203836	0	0	7	24
ENSG00000203837	0	0	0	0
ENSG00000203843	0	0	0	0
ENSG00000203849	0	0	0	0
ENSG00000203852	0	0	0	0
ENSG00000203855	0	0	0	0
ENSG00000203857	0	0	0	0
ENSG00000203858	0	0	0	0
ENSG00000203859	0	0	0	0
ENSG00000203863	2	0	0	0
ENSG00000203864	0	0	0	0
ENSG00000203865	0	0	1	0
ENSG00000203867	0	0	0	2
ENSG00000203870	0	0	0	0
ENSG00000203871	0	0	6	0
ENSG00000203872	0	78	49	104
ENSG00000203875	103	129	56	151
ENSG00000203876	23	59	8	43
ENSG00000203877	0	0	0	0
ENSG00000203878	0	0	0	0
ENSG00000203879	478	2346	2003	2915
ENSG00000203880	51	136	121	115
ENSG00000203883	1	0	0	0
ENSG00000203886	0	0	0	23
ENSG00000203896	0	0	0	0
ENSG00000203897	0	0	0	0
ENSG00000203900	0	0	0	0
ENSG00000203902	0	0	0	0
ENSG00000203907	0	0	0	0

ENSG00000203908	0	0	0	0
ENSG00000203909	0	0	0	0
ENSG00000203910	0	0	0	0
ENSG00000203914	0	0	0	0
ENSG00000203923	0	0	0	0
ENSG00000203924	0	0	0	0
ENSG00000203926	0	0	0	0
ENSG00000203930	0	0	0	0
ENSG00000203933	0	0	0	0
ENSG00000203942	0	0	0	0
ENSG00000203943	2	0	0	0
ENSG00000203945	5	1	5	7
ENSG00000203950	48	69	20	10
ENSG00000203952	0	0	0	0
ENSG00000203963	0	0	3	25
ENSG00000203965	1	122	96	98
ENSG00000203970	0	0	0	0
ENSG00000203971	0	7	0	0
ENSG00000203972	0	0	0	0
ENSG00000203985	0	0	0	0
ENSG00000203987	0	0	0	0
ENSG00000203989	0	0	0	0
ENSG00000203993	1	1	0	2
ENSG00000203995	0	15	1	0
ENSG00000203999	0	0	0	0
ENSG00000204001	0	0	0	0
ENSG00000204003	0	0	0	0
ENSG00000204006	0	0	0	0
ENSG00000204007	0	0	0	0
ENSG00000204010	1	0	1	0
ENSG00000204011	0	0	0	0
ENSG00000204018	0	0	0	0
ENSG00000204019	0	0	0	0
ENSG00000204020	0	0	0	0
ENSG00000204021	0	0	0	10
ENSG00000204022	0	6	7	4
ENSG00000204025	0	0	0	0
ENSG00000204031	0	0	0	0
ENSG00000204033	0	0	0	0
ENSG00000204038	0	0	0	0
ENSG00000204044	0	0	0	0
ENSG00000204049	0	0	0	0
ENSG00000204052	0	0	0	0
ENSG00000204053	0	0	0	0
ENSG00000204054	120	285	156	79
ENSG00000204055	0	0	0	0

ENSG00000204060	0	0	0	0
ENSG00000204065	0	0	0	0
ENSG00000204070	28	21	21	13
ENSG00000204071	0	0	0	0
ENSG00000204072	3	1	6	11
ENSG00000204084	64	1504	1054	1367
ENSG00000204086	0	0	0	0
ENSG00000204091	0	0	0	0
ENSG00000204092	0	0	0	0
ENSG00000204099	0	0	0	0
ENSG00000204103	44	0	0	0
ENSG00000204104	9	0	7	14
ENSG00000204110	0	0	0	0
ENSG00000204113	0	0	0	0
ENSG00000204116	6	359	651	616
ENSG00000204117	0	0	0	0
ENSG00000204118	1	0	0	0
ENSG00000204119	0	0	0	0
ENSG00000204120	85	554	596	928
ENSG00000204121	0	0	0	0
ENSG00000204128	0	0	0	0
ENSG00000204130	22	3343	2935	4926
ENSG00000204131	8	0	0	12
ENSG00000204136	4248	7131	3885	9969
ENSG00000204138	51	815	717	1408
ENSG00000204140	0	0	0	0
ENSG00000204147	0	0	0	0
ENSG00000204148	0	0	0	0
ENSG00000204149	1	30	19	25
ENSG00000204150	0	0	8	16
ENSG00000204152	0	0	2	4
ENSG00000204160	4	0	2	1
ENSG00000204161	4	0	0	0
ENSG00000204164	0	0	4	5
ENSG00000204165	0	0	0	0
ENSG00000204169	0	0	0	0
ENSG00000204172	0	0	6	2
ENSG00000204173	0	0	0	0
ENSG00000204174	0	0	0	0
ENSG00000204175	0	0	0	0
ENSG00000204176	3	16	4	38
ENSG00000204177	0	0	4	2
ENSG00000204178	52	1058	765	1252
ENSG00000204179	0	0	0	0
ENSG00000204183	0	0	0	0
ENSG00000204186	6	154	179	204

ENSG00000204187	0	0	0	0
ENSG00000204188	0	0	0	0
ENSG00000204193	0	0	0	0
ENSG00000204194	0	0	0	0
ENSG00000204195	0	0	0	0
ENSG00000204196	0	0	0	0
ENSG00000204197	0	0	0	0
ENSG00000204209	151	67	35	64
ENSG00000204217	57	2377	1901	3900
ENSG00000204219	1	0	2	2
ENSG00000204220	6	0	9	16
ENSG00000204221	0	0	0	0
ENSG00000204227	3	46	5	24
ENSG00000204228	5	0	0	1
ENSG00000204231	7	41	6	3
ENSG00000204237	5	20	11	15
ENSG00000204241	2	0	0	0
ENSG00000204246	0	0	0	0
ENSG00000204248	1	0	0	0
ENSG00000204250	0	0	0	0
ENSG00000204252	11	0	0	0
ENSG00000204253	0	5	1	4
ENSG00000204256	250	378	370	425
ENSG00000204257	42	15	34	26
ENSG00000204261	132	296	313	257
ENSG00000204262	1	0	0	0
ENSG00000204264	602	1050	649	570
ENSG00000204267	14	70	122	31
ENSG00000204271	0	0	0	1
ENSG00000204272	211	186	124	339
ENSG00000204276	0	0	0	0
ENSG00000204277	2	0	0	0
ENSG00000204278	0	0	0	0
ENSG00000204279	0	0	0	0
ENSG00000204282	1	0	2	0
ENSG00000204283	0	0	1	4
ENSG00000204287	348	32	23	18
ENSG00000204290	0	0	0	0
ENSG00000204291	1	0	0	0
ENSG00000204293	0	0	0	0
ENSG00000204296	0	0	0	0
ENSG00000204300	0	0	0	0
ENSG00000204301	9	2	8	7
ENSG00000204304	104	670	300	327
ENSG00000204305	5	10	3	8
ENSG00000204306	0	0	0	0

ENSG00000204308	170	261	214	272
ENSG00000204310	1098	5229	2967	3595
ENSG00000204311	0	0	5	0
ENSG00000204314	38	40	20	15
ENSG00000204315	2	0	1	3
ENSG00000204316	0	0	0	0
ENSG00000204323	2299	3190	897	1091
ENSG00000204334	0	0	0	0
ENSG00000204335	0	0	0	0
ENSG00000204338	0	0	0	0
ENSG00000204344	265	13	71	46
ENSG00000204345	0	0	0	0
ENSG00000204347	0	0	0	0
ENSG00000204348	15	0	6	3
ENSG00000204351	15	0	7	6
ENSG00000204352	0	14	0	1
ENSG00000204356	35	16	11	4
ENSG00000204359	0	0	0	0
ENSG00000204361	0	0	0	0
ENSG00000204362	0	0	0	0
ENSG00000204363	0	0	0	0
ENSG00000204364	0	0	0	0
ENSG00000204365	1	0	3	0
ENSG00000204366	0	0	0	0
ENSG00000204368	0	0	0	0
ENSG00000204370	18	61	22	49
ENSG00000204371	32	0	4	28
ENSG00000204375	0	0	0	0
ENSG00000204376	0	0	0	0
ENSG00000204377	0	0	0	0
ENSG00000204379	0	0	0	0
ENSG00000204380	8	0	5	1
ENSG00000204381	0	0	0	2
ENSG00000204382	0	0	0	0
ENSG00000204385	0	0	0	0
ENSG00000204386	4	0	0	2
ENSG00000204387	196	388	234	451
ENSG00000204388	3	4	13	40
ENSG00000204389	6	25	11	35
ENSG00000204390	2	0	4	3
ENSG00000204392	38	0	1	10
ENSG00000204393	0	0	0	0
ENSG00000204394	4	0	2	1
ENSG00000204396	0	0	0	0
ENSG00000204397	915	1723	1831	2752
ENSG00000204398	0	0	0	0

ENSG00000204399	0	0	0	0
ENSG00000204403	1	0	0	0
ENSG00000204406	99	1911	2342	3448
ENSG00000204410	0	0	0	3
ENSG00000204414	0	0	0	0
ENSG00000204420	10679	5736	6048	7657
ENSG00000204421	12	0	2	0
ENSG00000204422	0	0	0	0
ENSG00000204424	929	425	129	439
ENSG00000204427	7	20	4	3
ENSG00000204428	654	208	153	140
ENSG00000204429	0	0	0	0
ENSG00000204434	0	0	0	0
ENSG00000204435	135	44	10	26
ENSG00000204437	0	0	0	0
ENSG00000204438	111	338	165	146
ENSG00000204439	6	56	74	64
ENSG00000204442	0	0	0	0
ENSG00000204444	4	0	2	2
ENSG00000204446	0	0	0	0
ENSG00000204449	0	0	0	0
ENSG00000204450	0	0	0	0
ENSG00000204455	0	0	0	0
ENSG00000204456	0	0	0	0
ENSG00000204460	0	0	0	0
ENSG00000204463	53	87	65	108
ENSG00000204464	0	0	0	0
ENSG00000204466	0	0	0	0
ENSG00000204469	7	177	38	120
ENSG00000204471	0	0	0	0
ENSG00000204472	1596	56	103	176
ENSG00000204475	19	0	3	0
ENSG00000204478	0	0	0	0
ENSG00000204479	0	0	0	0
ENSG00000204480	0	0	0	0
ENSG00000204481	0	0	0	0
ENSG00000204482	736	126	38	137
ENSG00000204485	0	0	0	0
ENSG00000204486	0	0	0	0
ENSG00000204487	0	0	0	0
ENSG00000204488	0	0	0	0
ENSG00000204490	0	0	0	0
ENSG00000204491	0	0	0	0
ENSG00000204495	0	0	0	0
ENSG00000204498	2	3	9	2
ENSG00000204501	0	0	0	0

ENSG00000204502	0	0	0	0
ENSG00000204503	0	0	0	0
ENSG00000204505	0	0	0	0
ENSG00000204508	0	0	0	0
ENSG00000204510	0	0	0	0
ENSG00000204511	0	0	2	4
ENSG00000204513	0	0	0	0
ENSG00000204514	9	27	8	27
ENSG00000204516	3	0	15	5
ENSG00000204518	0	0	0	0
ENSG00000204519	3	0	0	0
ENSG00000204520	3	0	5	4
ENSG00000204524	0	16	27	15
ENSG00000204525	2257	3615	10158	8369
ENSG00000204528	0	0	0	0
ENSG00000204529	6	12	1	0
ENSG00000204531	1	2	3	2
ENSG00000204532	0	0	0	0
ENSG00000204533	0	0	0	0
ENSG00000204536	1	0	1	5
ENSG00000204538	0	0	0	0
ENSG00000204539	0	0	0	0
ENSG00000204540	0	0	0	0
ENSG00000204542	0	0	0	0
ENSG00000204544	0	0	0	0
ENSG00000204547	0	0	0	0
ENSG00000204548	0	0	0	0
ENSG00000204555	0	0	0	0
ENSG00000204556	0	0	1	10
ENSG00000204560	1	26	13	48
ENSG00000204564	31	120	56	39
ENSG00000204566	0	0	0	0
ENSG00000204568	38	0	4	14
ENSG00000204569	81	124	78	109
ENSG00000204571	0	0	0	0
ENSG00000204572	0	0	0	0
ENSG00000204574	200	154	114	153
ENSG00000204576	35	47	56	38
ENSG00000204577	3	0	0	3
ENSG00000204580	1	0	0	0
ENSG00000204581	0	0	0	0
ENSG00000204583	0	0	0	0
ENSG00000204584	0	0	0	0
ENSG00000204588	0	0	0	0
ENSG00000204590	22	215	179	190
ENSG00000204592	80231	75458	73316	101087

ENSG00000204595	0	0	0	3
ENSG00000204599	7	6	6	0
ENSG00000204603	0	0	0	0
ENSG00000204604	4	20	15	36
ENSG00000204610	16	13	0	0
ENSG00000204611	12	275	155	190
ENSG00000204612	0	0	0	0
ENSG00000204613	83	245	169	187
ENSG00000204614	67	770	306	735
ENSG00000204616	1	0	0	0
ENSG00000204618	1	0	5	8
ENSG00000204619	74	169	113	168
ENSG00000204620	8	0	0	5
ENSG00000204622	0	0	0	0
ENSG00000204623	3	222	180	187
ENSG00000204624	0	0	0	0
ENSG00000204625	1	0	5	1
ENSG00000204628	1553	636	342	538
ENSG00000204632	24	0	0	0
ENSG00000204634	8	0	1	0
ENSG00000204637	0	0	0	0
ENSG00000204640	0	0	0	0
ENSG00000204642	347	513	423	603
ENSG00000204644	3	0	0	213
ENSG00000204645	0	0	0	0
ENSG00000204648	1	0	0	0
ENSG00000204650	2	0	0	0
ENSG00000204652	1	0	0	0
ENSG00000204653	0	0	0	0
ENSG00000204655	0	0	0	0
ENSG00000204657	0	0	0	0
ENSG00000204658	0	0	0	0
ENSG00000204659	0	0	0	0
ENSG00000204661	0	0	0	0
ENSG00000204662	0	0	0	0
ENSG00000204663	0	0	0	0
ENSG00000204666	0	0	0	0
ENSG00000204669	0	0	0	0
ENSG00000204670	0	0	0	0
ENSG00000204671	0	0	0	0
ENSG00000204673	9	1	1	7
ENSG00000204677	0	0	0	0
ENSG00000204681	5	58	16	31
ENSG00000204682	0	0	0	0
ENSG00000204683	0	0	0	0
ENSG00000204684	0	0	0	0

ENSG00000204685	0	2	16	18
ENSG00000204687	0	0	0	0
ENSG00000204688	0	0	0	0
ENSG00000204689	0	0	0	0
ENSG00000204690	0	0	0	0
ENSG00000204692	0	0	0	0
ENSG00000204694	0	0	0	0
ENSG00000204695	0	0	0	0
ENSG00000204697	0	0	0	0
ENSG00000204699	0	0	0	0
ENSG00000204700	0	0	0	0
ENSG00000204701	0	0	0	0
ENSG00000204702	0	0	0	0
ENSG00000204703	0	0	0	0
ENSG00000204704	0	0	0	0
ENSG00000204705	0	0	0	0
ENSG00000204706	0	0	0	0
ENSG00000204709	3	26	4	3
ENSG00000204710	238	17	42	116
ENSG00000204711	0	0	0	0
ENSG00000204713	31	50	18	30
ENSG00000204717	0	0	0	0
ENSG00000204718	0	0	0	0
ENSG00000204740	8	362	223	241
ENSG00000204745	0	66	59	84
ENSG00000204752	0	0	0	0
ENSG00000204754	0	0	0	0
ENSG00000204758	0	0	0	0
ENSG00000204764	2	3914	3851	6781
ENSG00000204767	0	0	0	0
ENSG00000204771	0	0	0	0
ENSG00000204775	0	0	0	0
ENSG00000204776	0	0	0	0
ENSG00000204778	0	0	0	0
ENSG00000204779	0	0	0	0
ENSG00000204780	0	0	0	0
ENSG00000204787	0	0	0	0
ENSG00000204788	0	0	0	0
ENSG00000204789	3	0	0	0
ENSG00000204790	0	127	69	95
ENSG00000204791	0	0	3	2
ENSG00000204792	0	0	0	0
ENSG00000204793	0	0	0	0
ENSG00000204794	1	0	0	0
ENSG00000204801	0	0	0	0
ENSG00000204802	1	0	0	3

ENSG00000204804	0	0	0	0
ENSG00000204805	0	0	0	0
ENSG00000204807	0	0	0	0
ENSG00000204813	0	0	0	0
ENSG00000204814	0	0	0	0
ENSG00000204815	0	0	0	0
ENSG00000204816	0	0	0	0
ENSG00000204818	0	0	0	0
ENSG00000204822	1	0	0	0
ENSG00000204828	0	0	0	0
ENSG00000204832	26	42	11	37
ENSG00000204837	0	0	0	0
ENSG00000204839	2	0	4	5
ENSG00000204842	76	10109	9044	18558
ENSG00000204843	37	186	58	282
ENSG00000204844	0	0	0	0
ENSG00000204848	0	0	0	0
ENSG00000204849	0	0	0	0
ENSG00000204850	0	0	0	0
ENSG00000204851	0	0	0	0
ENSG00000204852	0	13	15	64
ENSG00000204856	9	739	931	1437
ENSG00000204859	0	15	1	0
ENSG00000204860	0	0	0	0
ENSG00000204866	0	0	0	0
ENSG00000204869	0	0	0	1
ENSG00000204872	1655	1154	567	1137
ENSG00000204873	0	0	0	0
ENSG00000204876	0	0	0	0
ENSG00000204880	0	0	0	0
ENSG00000204882	0	0	0	0
ENSG00000204887	0	0	0	0
ENSG00000204889	0	0	0	0
ENSG00000204894	0	0	0	0
ENSG00000204897	0	0	0	0
ENSG00000204899	101	497	793	1145
ENSG00000204904	0	0	0	7
ENSG00000204909	6	24	60	43
ENSG00000204913	0	0	0	0
ENSG00000204915	0	0	0	0
ENSG00000204918	0	0	0	0
ENSG00000204919	0	0	0	0
ENSG00000204920	0	4	6	6
ENSG00000204922	1	15	0	1
ENSG00000204923	238	275	186	151
ENSG00000204928	0	0	0	2

ENSG00000204929	0	0	0	0
ENSG00000204930	1	0	0	0
ENSG00000204933	0	0	0	0
ENSG00000204934	0	0	0	0
ENSG00000204936	0	0	0	0
ENSG00000204941	0	0	0	0
ENSG00000204946	2	0	0	0
ENSG00000204947	1	47	104	181
ENSG00000204949	0	0	0	0
ENSG00000204950	0	0	0	0
ENSG00000204952	0	4	0	8
ENSG00000204954	1	0	6	0
ENSG00000204956	0	0	0	0
ENSG00000204957	0	0	0	0
ENSG00000204959	0	0	0	0
ENSG00000204960	0	0	0	0
ENSG00000204961	0	0	0	0
ENSG00000204962	0	0	0	0
ENSG00000204963	0	0	0	0
ENSG00000204965	0	0	0	0
ENSG00000204967	0	0	0	0
ENSG00000204969	0	0	0	0
ENSG00000204970	0	0	0	0
ENSG00000204971	0	0	0	0
ENSG00000204977	185	592	818	538
ENSG00000204978	0	0	0	0
ENSG00000204979	0	0	0	0
ENSG00000204982	0	0	0	0
ENSG00000204983	0	0	0	0
ENSG00000204989	0	0	0	0
ENSG00000204990	0	0	0	0
ENSG00000204991	0	0	0	0
ENSG00000204993	0	0	0	0
ENSG00000205002	14	0	0	0
ENSG00000205011	0	0	0	0
ENSG00000205015	0	0	0	0
ENSG00000205018	0	0	0	0
ENSG00000205020	1	0	0	0
ENSG00000205021	12	11	1	1
ENSG00000205022	0	0	0	0
ENSG00000205025	0	0	0	0
ENSG00000205029	0	0	0	0
ENSG00000205030	0	0	0	0
ENSG00000205035	0	0	0	0
ENSG00000205037	0	0	1	0
ENSG00000205038	298	23586	31521	36785

ENSG00000205041	0	0	0	0
ENSG00000205044	0	0	0	0
ENSG00000205045	1	0	0	0
ENSG00000205047	1	0	0	0
ENSG00000205054	0	0	0	4
ENSG00000205056	4	0	0	1
ENSG00000205057	0	0	0	0
ENSG00000205060	49	480	334	242
ENSG00000205076	0	0	0	0
ENSG00000205078	0	0	0	0
ENSG00000205081	0	0	1	9
ENSG00000205084	0	5	2	2
ENSG00000205085	0	14	14	10
ENSG00000205086	0	0	0	0
ENSG00000205089	0	0	0	0
ENSG00000205090	0	0	0	0
ENSG00000205097	0	0	0	0
ENSG00000205100	0	0	0	0
ENSG00000205105	0	1	0	0
ENSG00000205106	0	0	0	0
ENSG00000205108	0	0	0	0
ENSG00000205111	0	171	139	158
ENSG00000205116	0	0	0	0
ENSG00000205126	74	375	376	182
ENSG00000205129	0	37	1	0
ENSG00000205133	38	3661	5113	5667
ENSG00000205138	3	0	4	2
ENSG00000205143	0	0	0	0
ENSG00000205147	0	0	0	0
ENSG00000205148	0	0	0	0
ENSG00000205155	8	3	4	5
ENSG00000205174	0	0	0	0
ENSG00000205176	0	27	2	5
ENSG00000205177	0	0	0	0
ENSG00000205181	0	44	5	2
ENSG00000205184	0	0	0	0
ENSG00000205186	0	0	0	0
ENSG00000205189	6	230	150	164
ENSG00000205208	6	35	26	15
ENSG00000205209	0	0	1	2
ENSG00000205212	0	0	0	0
ENSG00000205213	2	67	199	167
ENSG00000205215	0	0	0	0
ENSG00000205217	0	0	0	0
ENSG00000205220	54	26	35	29
ENSG00000205221	0	0	0	1

ENSG00000205231	0	0	0	0
ENSG00000205233	0	0	0	0
ENSG00000205236	0	0	0	0
ENSG00000205238	0	0	0	0
ENSG00000205240	3	33	5	0
ENSG00000205246	5	1	168	584
ENSG00000205250	24	13	29	32
ENSG00000205266	0	0	0	0
ENSG00000205267	0	0	0	0
ENSG00000205268	2	781	891	1319
ENSG00000205269	10	141	332	505
ENSG00000205271	0	0	0	0
ENSG00000205274	0	0	0	0
ENSG00000205275	0	0	0	0
ENSG00000205277	1	0	0	0
ENSG00000205279	0	0	0	0
ENSG00000205281	0	0	0	0
ENSG00000205293	0	0	0	0
ENSG00000205300	0	0	0	0
ENSG00000205301	0	0	0	0
ENSG00000205302	117	492	793	1012
ENSG00000205307	0	0	0	0
ENSG00000205309	1053	1382	874	1369
ENSG00000205312	0	0	0	0
ENSG00000205318	0	0	0	0
ENSG00000205323	14	0	3	3
ENSG00000205325	0	0	0	0
ENSG00000205327	0	0	0	0
ENSG00000205328	0	0	0	0
ENSG00000205329	0	0	0	0
ENSG00000205330	0	0	0	0
ENSG00000205331	0	0	0	0
ENSG00000205333	0	0	0	0
ENSG00000205334	1	0	0	0
ENSG00000205336	11	0	4	5
ENSG00000205339	57	4405	4257	5682
ENSG00000205352	546	404	206	364
ENSG00000205356	15	3	5	18
ENSG00000205358	0	0	0	0
ENSG00000205359	0	0	4	1
ENSG00000205360	0	0	0	0
ENSG00000205361	0	0	0	0
ENSG00000205362	0	0	0	0
ENSG00000205363	0	0	0	0
ENSG00000205364	0	0	0	0
ENSG00000205396	0	0	0	0

ENSG00000205403	0	0	0	0
ENSG00000205409	0	0	0	0
ENSG00000205412	0	0	0	0
ENSG00000205413	54	167	448	544
ENSG00000205414	0	0	0	0
ENSG00000205420	0	0	0	0
ENSG00000205423	2	45	11	46
ENSG00000205424	0	0	0	0
ENSG00000205426	0	0	0	0
ENSG00000205433	0	0	0	0
ENSG00000205436	1	19	2	0
ENSG00000205439	0	0	0	0
ENSG00000205441	0	0	0	0
ENSG00000205442	0	0	0	0
ENSG00000205444	0	0	0	0
ENSG00000205445	0	0	0	0
ENSG00000205449	0	0	0	0
ENSG00000205452	0	0	0	0
ENSG00000205456	7	26	41	36
ENSG00000205457	11	0	3	14
ENSG00000205464	2	51	29	115
ENSG00000205476	0	0	0	0
ENSG00000205482	0	0	0	0
ENSG00000205485	3	5	11	20
ENSG00000205488	0	0	0	0
ENSG00000205494	0	0	0	0
ENSG00000205495	1	0	0	0
ENSG00000205496	0	0	0	0
ENSG00000205497	0	0	0	0
ENSG00000205500	0	0	0	0
ENSG00000205502	0	0	0	0
ENSG00000205505	0	0	0	0
ENSG00000205506	0	0	0	0
ENSG00000205508	0	0	0	0
ENSG00000205509	0	0	0	0
ENSG00000205517	0	0	0	0
ENSG00000205531	562	2962	3023	3276
ENSG00000205534	12	61	106	107
ENSG00000205537	0	0	0	0
ENSG00000205542	1089411	3227340	2670990	2625697
ENSG00000205544	40	26	18	42
ENSG00000205549	0	0	0	0
ENSG00000205559	0	0	1	0
ENSG00000205560	0	0	0	0
ENSG00000205562	0	0	0	0
ENSG00000205565	0	0	0	0

ENSG00000205571	1	14	39	72
ENSG00000205572	0	0	0	0
ENSG00000205578	0	1	0	0
ENSG00000205579	1	0	0	0
ENSG00000205580	0	0	0	0
ENSG00000205581	1663	5146	6177	11579
ENSG00000205583	1	1	6	10
ENSG00000205584	0	0	0	0
ENSG00000205592	0	0	0	1
ENSG00000205593	73	147	41	206
ENSG00000205595	0	0	0	0
ENSG00000205596	0	0	0	0
ENSG00000205609	0	0	0	0
ENSG00000205611	0	0	0	0
ENSG00000205622	0	0	0	0
ENSG00000205625	0	0	0	0
ENSG00000205628	0	0	0	2
ENSG00000205629	19	411	232	502
ENSG00000205632	0	0	0	0
ENSG00000205634	0	0	0	0
ENSG00000205636	0	0	0	0
ENSG00000205639	376	622	789	1146
ENSG00000205642	0	0	0	0
ENSG00000205643	2	0	0	0
ENSG00000205644	0	0	0	0
ENSG00000205649	0	0	0	0
ENSG00000205653	0	0	0	0
ENSG00000205659	20	11	26	48
ENSG00000205662	0	0	0	0
ENSG00000205663	0	112	112	0
ENSG00000205664	1	515	764	338
ENSG00000205667	0	0	3	8
ENSG00000205669	0	0	2	0
ENSG00000205670	29	21	16	37
ENSG00000205677	0	0	0	0
ENSG00000205678	0	0	0	0
ENSG00000205682	5	0	0	1
ENSG00000205683	43	142	119	111
ENSG00000205693	0	0	0	0
ENSG00000205695	0	0	0	0
ENSG00000205696	0	0	0	0
ENSG00000205702	0	0	0	0
ENSG00000205704	0	0	0	0
ENSG00000205707	61	718	883	1168
ENSG00000205710	0	0	0	0
ENSG00000205716	0	0	0	0

ENSG00000205718	0	0	0	0
ENSG00000205726	98	391	408	728
ENSG00000205730	4	0	2	2
ENSG00000205740	0	0	0	0
ENSG00000205744	30	0	1	0
ENSG00000205745	0	0	0	0
ENSG00000205746	0	0	0	5
ENSG00000205754	0	16	0	3
ENSG00000205755	0	0	0	0
ENSG00000205758	31	219	270	780
ENSG00000205763	6	13	8	3
ENSG00000205765	64	839	1151	972
ENSG00000205767	0	0	0	0
ENSG00000205771	7	25	16	31
ENSG00000205775	0	0	0	0
ENSG00000205777	0	0	0	0
ENSG00000205784	0	0	0	3
ENSG00000205786	2	0	0	0
ENSG00000205790	0	0	0	0
ENSG00000205791	7	0	9	4
ENSG00000205794	0	0	0	0
ENSG00000205795	1	44	4	0
ENSG00000205808	1	31	17	3
ENSG00000205809	3	0	0	0
ENSG00000205810	1	0	0	0
ENSG00000205821	0	0	0	0
ENSG00000205822	1	0	2	1
ENSG00000205830	0	0	0	0
ENSG00000205832	0	22	4	5
ENSG00000205835	0	0	0	0
ENSG00000205837	0	0	0	0
ENSG00000205838	0	0	0	0
ENSG00000205846	0	0	0	0
ENSG00000205847	0	0	0	0
ENSG00000205850	0	0	0	0
ENSG00000205853	0	0	0	2
ENSG00000205856	0	0	2	0
ENSG00000205857	0	0	0	0
ENSG00000205858	0	0	0	0
ENSG00000205861	0	0	0	0
ENSG00000205863	0	0	0	0
ENSG00000205864	0	0	0	0
ENSG00000205865	0	0	0	0
ENSG00000205866	0	0	0	0
ENSG00000205867	0	0	0	0
ENSG00000205869	0	0	0	0

ENSG00000205871	8	0	0	0
ENSG00000205873	12	40	22	30
ENSG00000205879	0	0	0	0
ENSG00000205882	0	0	0	0
ENSG00000205883	0	0	0	0
ENSG00000205884	0	0	0	0
ENSG00000205885	2	0	1	2
ENSG00000205890	0	0	0	0
ENSG00000205897	0	0	0	0
ENSG00000205898	0	0	2	0
ENSG00000205899	0	0	0	0
ENSG00000205903	0	0	0	0
ENSG00000205913	0	0	0	0
ENSG00000205916	0	0	0	0
ENSG00000205918	0	0	0	1
ENSG00000205922	0	0	0	0
ENSG00000205923	0	0	2	0
ENSG00000205927	0	0	0	0
ENSG00000205929	1	11	8	0
ENSG00000205930	45	27	83	108
ENSG00000205936	0	0	0	0
ENSG00000205937	75	198	117	312
ENSG00000205940	0	0	0	0
ENSG00000205944	0	0	0	0
ENSG00000205946	0	0	0	0
ENSG00000205955	1	0	0	0
ENSG00000205959	0	0	0	0
ENSG00000205971	0	0	0	0
ENSG00000205976	0	0	0	0
ENSG00000205978	0	0	0	0
ENSG00000205981	19	73	21	53
ENSG00000205989	0	0	0	0
ENSG00000206013	0	0	0	0
ENSG00000206014	0	0	0	0
ENSG00000206026	0	0	0	0
ENSG00000206028	0	0	0	0
ENSG00000206034	0	0	0	0
ENSG00000206042	0	0	0	0
ENSG00000206043	0	0	0	0
ENSG00000206044	0	0	0	0
ENSG00000206047	0	0	0	0
ENSG00000206052	27	259	170	400
ENSG00000206053	170	668	630	487
ENSG00000206062	0	0	0	0
ENSG00000206066	0	0	1	0
ENSG00000206069	0	0	0	0

ENSG00000206072	0	0	0	0
ENSG00000206073	0	0	0	0
ENSG00000206075	0	0	0	0
ENSG00000206077	1	0	0	0
ENSG00000206082	0	30	77	62
ENSG00000206090	0	0	0	0
ENSG00000206102	0	0	0	0
ENSG00000206104	0	0	0	0
ENSG00000206105	0	0	0	0
ENSG00000206106	0	0	0	0
ENSG00000206107	0	0	0	0
ENSG00000206113	0	0	0	0
ENSG00000206114	0	0	0	0
ENSG00000206120	0	0	8	3
ENSG00000206127	0	0	8	14
ENSG00000206129	40	34	22	25
ENSG00000206140	2	0	3	3
ENSG00000206142	0	0	0	0
ENSG00000206144	0	0	0	0
ENSG00000206145	20	21	1	4
ENSG00000206147	0	0	0	0
ENSG00000206149	26	753	471	758
ENSG00000206150	0	0	0	0
ENSG00000206159	0	11	0	7
ENSG00000206168	0	0	0	0
ENSG00000206171	0	0	0	0
ENSG00000206172	1048	21	12	15
ENSG00000206176	0	0	2	1
ENSG00000206177	16	20	4	15
ENSG00000206178	0	0	0	0
ENSG00000206181	0	0	1	0
ENSG00000206187	0	0	0	0
ENSG00000206190	1	0	0	0
ENSG00000206192	0	0	4	4
ENSG00000206195	0	0	5	17
ENSG00000206199	0	358	404	145
ENSG00000206203	0	0	0	0
ENSG00000206206	0	0	0	0
ENSG00000206208	0	0	0	0
ENSG00000206228	0	0	0	0
ENSG00000206232	0	0	0	0
ENSG00000206235	0	0	0	0
ENSG00000206237	0	0	0	0
ENSG00000206240	0	0	0	0
ENSG00000206245	0	0	0	0
ENSG00000206249	0	0	0	0

ENSG00000206252	0	0	0	0
ENSG00000206256	0	0	0	0
ENSG00000206258	0	0	0	0
ENSG00000206260	0	0	0	0
ENSG00000206262	0	0	0	0
ENSG00000206268	0	0	0	0
ENSG00000206279	0	0	0	0
ENSG00000206280	0	0	0	0
ENSG00000206281	0	0	0	0
ENSG00000206282	0	0	0	0
ENSG00000206283	0	0	0	0
ENSG00000206284	0	0	0	0
ENSG00000206285	0	0	0	0
ENSG00000206286	0	0	0	0
ENSG00000206287	0	0	0	0
ENSG00000206288	0	0	0	0
ENSG00000206289	0	0	0	0
ENSG00000206290	0	0	0	0
ENSG00000206291	0	0	0	0
ENSG00000206292	0	0	0	0
ENSG00000206296	0	0	0	0
ENSG00000206297	0	0	0	0
ENSG00000206298	0	0	0	0
ENSG00000206299	0	0	0	0
ENSG00000206301	0	0	0	0
ENSG00000206302	0	0	0	0
ENSG00000206305	0	0	0	0
ENSG00000206306	0	0	0	0
ENSG00000206308	0	0	0	0
ENSG00000206310	0	0	0	0
ENSG00000206312	0	0	0	0
ENSG00000206314	0	0	0	0
ENSG00000206315	0	0	0	0
ENSG00000206320	0	0	0	0
ENSG00000206324	0	0	0	0
ENSG00000206329	0	0	0	0
ENSG00000206331	0	0	0	0
ENSG00000206337	14	30	2	2
ENSG00000206338	0	0	0	0
ENSG00000206340	0	0	0	0
ENSG00000206341	143	12	4	67
ENSG00000206342	0	0	0	0
ENSG00000206344	23	94	28	46
ENSG00000206346	0	0	0	0
ENSG00000206353	0	0	0	0
ENSG00000206355	0	0	0	0

ENSG00000206356	0	0	0	0
ENSG00000206357	0	0	0	0
ENSG00000206366	0	0	0	0
ENSG00000206372	0	0	0	0
ENSG00000206376	0	0	0	0
ENSG00000206378	0	0	0	0
ENSG00000206379	0	0	0	0
ENSG00000206380	0	0	0	0
ENSG00000206383	0	0	0	0
ENSG00000206384	0	0	0	0
ENSG00000206394	0	0	0	0
ENSG00000206395	0	0	0	0
ENSG00000206396	0	0	0	0
ENSG00000206398	0	0	0	0
ENSG00000206401	0	0	0	0
ENSG00000206402	0	0	0	0
ENSG00000206403	0	0	0	0
ENSG00000206404	0	0	0	0
ENSG00000206406	0	0	0	0
ENSG00000206408	0	0	0	0
ENSG00000206409	0	0	0	0
ENSG00000206412	0	0	0	0
ENSG00000206417	0	0	3	4
ENSG00000206418	31	743	590	749
ENSG00000206422	0	0	0	0
ENSG00000206427	0	0	0	0
ENSG00000206428	0	0	0	0
ENSG00000206430	0	0	0	0
ENSG00000206432	0	0	0	0
ENSG00000206433	0	0	0	0
ENSG00000206435	0	0	0	0
ENSG00000206437	0	0	0	0
ENSG00000206439	0	0	0	0
ENSG00000206440	0	0	0	0
ENSG00000206444	0	0	0	0
ENSG00000206445	0	0	0	0
ENSG00000206446	0	0	0	0
ENSG00000206447	0	0	0	0
ENSG00000206448	0	0	0	0
ENSG00000206449	0	0	0	0
ENSG00000206450	0	0	0	0
ENSG00000206452	0	0	0	0
ENSG00000206454	0	0	0	0
ENSG00000206455	0	0	0	0
ENSG00000206457	0	0	0	0
ENSG00000206458	0	0	0	0

ENSG00000206459	0	0	0	0
ENSG00000206460	0	0	0	0
ENSG00000206461	0	0	0	0
ENSG00000206463	0	0	0	0
ENSG00000206465	0	0	0	0
ENSG00000206466	0	0	0	0
ENSG00000206467	0	0	0	0
ENSG00000206468	0	0	0	0
ENSG00000206470	0	0	0	0
ENSG00000206471	0	0	0	0
ENSG00000206472	0	0	0	0
ENSG00000206474	0	0	0	0
ENSG00000206476	0	0	0	0
ENSG00000206478	0	0	0	0
ENSG00000206480	0	0	0	0
ENSG00000206481	0	0	0	0
ENSG00000206483	0	0	0	0
ENSG00000206484	0	0	0	0
ENSG00000206485	0	0	0	0
ENSG00000206486	0	0	0	0
ENSG00000206487	0	0	0	0
ENSG00000206488	0	0	0	0
ENSG00000206489	0	0	0	0
ENSG00000206490	0	0	0	0
ENSG00000206491	0	0	0	0
ENSG00000206492	0	0	0	0
ENSG00000206493	0	0	0	0
ENSG00000206495	0	0	0	0
ENSG00000206500	0	0	0	0
ENSG00000206501	0	0	0	0
ENSG00000206502	0	0	0	0
ENSG00000206503	8299	8972	11071	11059
ENSG00000206504	0	0	0	0
ENSG00000206505	0	0	0	0
ENSG00000206506	0	0	0	0
ENSG00000206507	0	0	0	0
ENSG00000206508	0	0	0	0
ENSG00000206509	0	0	0	0
ENSG00000206510	0	0	0	0
ENSG00000206511	0	0	0	0
ENSG00000206512	0	0	0	0
ENSG00000206513	0	0	0	0
ENSG00000206515	0	0	0	0
ENSG00000206516	0	0	0	0
ENSG00000206517	0	0	0	0
ENSG00000206519	0	0	0	0

ENSG00000206522	0	0	0	0
ENSG00000206523	0	0	0	0
ENSG00000206524	0	0	0	0
ENSG00000206525	0	0	0	0
ENSG00000206526	0	0	0	0
ENSG00000206527	0	7	15	22
ENSG00000206530	7	430	329	851
ENSG00000206531	0	0	0	0
ENSG00000206532	0	0	0	0
ENSG00000206535	3	61	34	19
ENSG00000206536	0	0	0	3
ENSG00000206538	0	0	0	1
ENSG00000206549	6	18	152	14
ENSG00000206552	0	0	0	0
ENSG00000206557	0	0	0	0
ENSG00000206559	1	81	218	365
ENSG00000206560	1812	76424	80666	54061
ENSG00000206561	6	12	12	8
ENSG00000206562	7	668	590	653
ENSG00000206567	44	58	29	52
ENSG00000206573	488	862	698	869
ENSG00000206579	0	0	0	0
ENSG00000206582	0	0	0	0
ENSG00000206583	0	0	0	0
ENSG00000206585	0	0	0	0
ENSG00000206587	0	0	0	0
ENSG00000206588	0	0	0	0
ENSG00000206589	0	0	0	0
ENSG00000206590	0	0	0	0
ENSG00000206592	0	0	0	0
ENSG00000206593	0	0	0	0
ENSG00000206595	0	0	0	0
ENSG00000206596	0	0	0	0
ENSG00000206597	0	0	0	0
ENSG00000206598	0	0	0	0
ENSG00000206599	0	0	0	0
ENSG00000206600	0	0	2	1
ENSG00000206601	0	0	0	0
ENSG00000206602	0	0	0	0
ENSG00000206603	0	0	0	0
ENSG00000206604	0	0	0	0
ENSG00000206605	0	0	0	0
ENSG00000206606	0	0	0	0
ENSG00000206609	0	0	0	0
ENSG00000206610	0	0	0	0
ENSG00000206611	0	0	0	0

ENSG00000206612	0	0	0	0
ENSG00000206613	0	0	0	0
ENSG00000206614	0	0	0	0
ENSG00000206615	0	0	0	0
ENSG00000206616	0	0	0	0
ENSG00000206617	0	0	0	0
ENSG00000206618	0	0	0	0
ENSG00000206620	0	0	0	0
ENSG00000206621	0	0	0	0
ENSG00000206622	0	0	0	0
ENSG00000206623	0	0	0	0
ENSG00000206624	0	0	0	0
ENSG00000206625	0	33	50	13
ENSG00000206627	0	0	0	0
ENSG00000206629	0	0	0	0
ENSG00000206630	0	0	0	0
ENSG00000206631	0	0	0	0
ENSG00000206633	0	0	1	0
ENSG00000206634	0	1	6	8
ENSG00000206635	0	0	0	0
ENSG00000206636	0	0	0	0
ENSG00000206637	0	0	0	0
ENSG00000206638	0	0	0	0
ENSG00000206639	0	0	0	0
ENSG00000206640	0	0	0	0
ENSG00000206641	0	0	0	0
ENSG00000206644	0	0	0	0
ENSG00000206645	0	0	0	0
ENSG00000206646	0	0	0	0
ENSG00000206647	0	0	0	0
ENSG00000206649	0	0	0	0
ENSG00000206650	0	0	0	0
ENSG00000206651	0	0	0	0
ENSG00000206652	0	0	2	0
ENSG00000206653	0	0	0	0
ENSG00000206654	0	0	0	0
ENSG00000206656	0	0	0	0
ENSG00000206658	0	0	0	0
ENSG00000206659	0	0	0	0
ENSG00000206660	0	0	0	0
ENSG00000206661	0	0	0	0
ENSG00000206662	0	0	0	0
ENSG00000206663	0	0	0	0
ENSG00000206665	0	0	0	0
ENSG00000206668	0	0	1	0
ENSG00000206669	0	0	0	0

ENSG00000206671	0	0	0	0
ENSG00000206672	0	0	0	0
ENSG00000206673	0	0	0	0
ENSG00000206674	0	0	0	0
ENSG00000206675	0	0	3	0
ENSG00000206676	0	0	0	0
ENSG00000206677	0	0	0	0
ENSG00000206678	0	0	0	0
ENSG00000206679	0	0	0	0
ENSG00000206680	0	0	0	0
ENSG00000206681	0	0	0	0
ENSG00000206682	0	0	0	0
ENSG00000206684	0	0	0	0
ENSG00000206685	0	0	0	0
ENSG00000206686	0	0	0	0
ENSG00000206687	0	0	0	0
ENSG00000206688	0	0	0	0
ENSG00000206690	0	0	0	0
ENSG00000206692	0	0	0	0
ENSG00000206693	0	0	0	0
ENSG00000206694	0	0	0	0
ENSG00000206695	0	0	0	0
ENSG00000206696	0	0	0	0
ENSG00000206697	0	0	0	0
ENSG00000206698	0	0	0	0
ENSG00000206699	0	0	0	0
ENSG00000206700	0	0	0	0
ENSG00000206701	0	0	0	0
ENSG00000206702	1	0	0	0
ENSG00000206703	0	0	0	0
ENSG00000206704	0	0	0	0
ENSG00000206705	0	0	0	0
ENSG00000206706	0	0	0	0
ENSG00000206708	0	0	0	0
ENSG00000206709	0	0	0	0
ENSG00000206710	0	0	0	0
ENSG00000206711	0	0	0	0
ENSG00000206712	0	0	5	1
ENSG00000206713	0	0	0	0
ENSG00000206714	0	0	0	0
ENSG00000206715	0	0	0	0
ENSG00000206716	0	0	0	0
ENSG00000206717	0	0	0	0
ENSG00000206718	0	0	0	0
ENSG00000206719	0	0	0	0
ENSG00000206721	0	0	0	0

ENSG00000206722	0	0	0	0
ENSG00000206723	0	0	0	0
ENSG00000206724	0	0	0	0
ENSG00000206725	0	0	0	0
ENSG00000206726	0	0	0	0
ENSG00000206727	0	0	0	0
ENSG00000206728	0	0	0	0
ENSG00000206729	0	0	0	0
ENSG00000206730	0	0	0	0
ENSG00000206731	0	0	0	0
ENSG00000206732	0	0	0	0
ENSG00000206733	0	0	0	0
ENSG00000206734	0	0	0	0
ENSG00000206737	1	0	0	0
ENSG00000206738	0	0	1	0
ENSG00000206739	0	0	0	0
ENSG00000206741	0	0	0	0
ENSG00000206743	0	0	0	0
ENSG00000206744	0	0	0	0
ENSG00000206745	0	0	0	0
ENSG00000206746	0	0	0	0
ENSG00000206747	0	0	0	0
ENSG00000206749	0	0	0	0
ENSG00000206750	0	0	0	0
ENSG00000206751	0	0	0	0
ENSG00000206752	0	0	0	0
ENSG00000206753	0	0	0	0
ENSG00000206754	0	0	0	0
ENSG00000206755	0	0	0	0
ENSG00000206756	0	0	0	0
ENSG00000206758	0	0	0	0
ENSG00000206759	0	0	0	0
ENSG00000206760	0	0	0	0
ENSG00000206761	0	0	0	0
ENSG00000206762	0	0	0	0
ENSG00000206763	0	0	0	0
ENSG00000206764	0	0	0	0
ENSG00000206765	0	0	0	0
ENSG00000206766	0	0	0	0
ENSG00000206767	0	0	0	0
ENSG00000206768	0	0	0	0
ENSG00000206769	0	0	0	0
ENSG00000206770	0	0	0	0
ENSG00000206772	0	0	0	0
ENSG00000206774	0	0	0	0
ENSG00000206775	0	0	0	0

ENSG00000206776	0	0	0	0
ENSG00000206777	0	0	0	0
ENSG00000206778	0	0	0	0
ENSG00000206779	0	0	0	0
ENSG00000206780	0	0	0	0
ENSG00000206781	0	0	0	0
ENSG00000206782	0	0	0	0
ENSG00000206783	0	0	0	0
ENSG00000206784	0	0	1	0
ENSG00000206785	0	0	0	0
ENSG00000206786	0	0	0	0
ENSG00000206787	0	0	0	0
ENSG00000206788	0	0	0	0
ENSG00000206790	0	0	0	0
ENSG00000206791	0	0	0	0
ENSG00000206792	0	0	0	0
ENSG00000206794	0	0	0	0
ENSG00000206795	0	0	0	0
ENSG00000206796	0	0	0	0
ENSG00000206797	0	0	0	0
ENSG00000206799	0	0	0	0
ENSG00000206800	0	0	0	0
ENSG00000206801	0	0	0	0
ENSG00000206802	0	0	0	0
ENSG00000206803	0	0	0	0
ENSG00000206804	0	0	0	0
ENSG00000206805	0	0	0	0
ENSG00000206806	0	0	0	0
ENSG00000206807	0	0	0	0
ENSG00000206808	0	0	0	0
ENSG00000206811	0	0	0	0
ENSG00000206812	0	0	0	0
ENSG00000206813	0	0	0	0
ENSG00000206814	0	0	0	0
ENSG00000206815	0	0	0	0
ENSG00000206816	0	0	0	0
ENSG00000206817	0	0	0	0
ENSG00000206818	0	0	0	0
ENSG00000206819	0	0	0	0
ENSG00000206820	0	0	0	0
ENSG00000206822	0	0	0	0
ENSG00000206823	0	0	0	0
ENSG00000206824	0	0	0	0
ENSG00000206826	0	0	0	0
ENSG00000206827	0	0	0	0
ENSG00000206828	0	0	0	0

ENSG00000206830	0	0	0	0
ENSG00000206832	0	0	0	0
ENSG00000206833	0	0	0	0
ENSG00000206834	0	0	0	0
ENSG00000206835	0	0	0	0
ENSG00000206836	0	0	0	0
ENSG00000206838	0	0	0	0
ENSG00000206839	0	0	0	0
ENSG00000206840	0	0	0	0
ENSG00000206841	0	0	0	0
ENSG00000206842	0	0	0	0
ENSG00000206843	0	0	0	0
ENSG00000206844	0	0	0	0
ENSG00000206845	0	0	0	0
ENSG00000206846	1	0	0	0
ENSG00000206847	0	0	0	0
ENSG00000206848	0	0	0	0
ENSG00000206849	0	0	0	0
ENSG00000206850	0	0	0	0
ENSG00000206852	0	0	0	0
ENSG00000206853	0	0	0	0
ENSG00000206854	0	0	0	0
ENSG00000206855	0	0	0	0
ENSG00000206856	0	0	0	0
ENSG00000206857	0	0	0	0
ENSG00000206858	0	0	0	0
ENSG00000206859	0	0	0	0
ENSG00000206861	0	0	0	0
ENSG00000206862	0	0	0	0
ENSG00000206863	0	0	0	0
ENSG00000206864	0	0	0	0
ENSG00000206865	0	0	0	0
ENSG00000206866	0	0	0	0
ENSG00000206867	0	0	0	0
ENSG00000206869	0	0	0	0
ENSG00000206870	0	0	0	0
ENSG00000206871	0	0	0	0
ENSG00000206872	0	0	0	0
ENSG00000206874	0	0	0	0
ENSG00000206875	0	0	0	0
ENSG00000206877	0	0	0	0
ENSG00000206878	0	0	0	0
ENSG00000206880	0	0	0	0
ENSG00000206881	0	0	0	0
ENSG00000206882	0	0	0	0
ENSG00000206885	0	0	0	0

ENSG00000206886	0	0	0	0
ENSG00000206887	0	0	0	0
ENSG00000206888	0	0	0	0
ENSG00000206889	0	0	0	0
ENSG00000206891	0	0	0	0
ENSG00000206892	0	0	0	0
ENSG00000206895	0	0	0	0
ENSG00000206896	0	0	0	0
ENSG00000206897	0	0	0	1
ENSG00000206898	0	0	0	0
ENSG00000206899	0	15	27	14
ENSG00000206900	0	0	0	0
ENSG00000206901	0	0	0	0
ENSG00000206902	0	0	1	0
ENSG00000206903	0	0	0	0
ENSG00000206905	0	0	0	0
ENSG00000206906	0	0	0	0
ENSG00000206907	0	0	0	0
ENSG00000206908	0	0	0	0
ENSG00000206909	0	0	0	0
ENSG00000206910	0	0	0	0
ENSG00000206911	0	0	0	0
ENSG00000206912	0	0	0	0
ENSG00000206913	0	0	0	0
ENSG00000206914	0	0	0	0
ENSG00000206915	0	0	0	0
ENSG00000206917	0	0	0	0
ENSG00000206918	0	0	0	0
ENSG00000206920	1	0	0	0
ENSG00000206921	0	0	0	0
ENSG00000206922	0	0	0	0
ENSG00000206923	0	0	0	0
ENSG00000206924	0	0	0	0
ENSG00000206925	0	0	0	0
ENSG00000206926	0	0	0	0
ENSG00000206927	0	0	0	0
ENSG00000206929	0	0	0	0
ENSG00000206931	0	0	0	0
ENSG00000206932	0	50	76	24
ENSG00000206935	0	0	0	0
ENSG00000206936	0	0	0	0
ENSG00000206937	0	0	0	0
ENSG00000206938	0	0	0	0
ENSG00000206939	0	0	0	0
ENSG00000206941	0	0	0	0
ENSG00000206942	0	0	0	0

ENSG00000206944	0	0	0	0
ENSG00000206946	0	0	0	0
ENSG00000206947	0	0	0	0
ENSG00000206948	0	0	0	0
ENSG00000206949	0	0	0	0
ENSG00000206950	0	0	0	0
ENSG00000206951	1	0	0	0
ENSG00000206952	0	0	2	6
ENSG00000206954	0	0	0	0
ENSG00000206956	0	0	0	0
ENSG00000206957	0	0	0	0
ENSG00000206958	0	0	0	0
ENSG00000206959	0	0	0	0
ENSG00000206960	0	0	0	0
ENSG00000206961	0	0	0	0
ENSG00000206962	0	0	0	0
ENSG00000206963	0	0	0	0
ENSG00000206964	0	0	0	0
ENSG00000206965	3	21	26	18
ENSG00000206967	0	0	0	0
ENSG00000206968	0	0	0	0
ENSG00000206969	0	0	0	0
ENSG00000206970	0	0	0	0
ENSG00000206972	0	0	0	0
ENSG00000206973	0	0	0	0
ENSG00000206974	0	0	0	0
ENSG00000206975	0	0	5	0
ENSG00000206976	0	0	0	0
ENSG00000206977	0	0	0	0
ENSG00000206978	0	0	0	0
ENSG00000206979	0	0	0	0
ENSG00000206980	0	0	0	0
ENSG00000206981	0	0	0	0
ENSG00000206982	0	0	0	0
ENSG00000206983	0	0	0	0
ENSG00000206985	0	0	0	0
ENSG00000206987	0	0	0	0
ENSG00000206989	0	0	0	0
ENSG00000206990	0	0	0	0
ENSG00000206991	0	0	0	0
ENSG00000206992	0	0	3	0
ENSG00000206995	1	0	0	0
ENSG00000206996	0	0	0	0
ENSG00000206997	0	0	0	0
ENSG00000206998	0	0	0	0
ENSG00000206999	0	0	0	0

ENSG00000207000	0	0	0	0
ENSG00000207001	0	0	0	0
ENSG00000207002	0	0	0	0
ENSG00000207003	0	0	0	0
ENSG00000207004	0	0	0	0
ENSG00000207005	0	0	0	0
ENSG00000207007	0	0	0	0
ENSG00000207008	0	0	0	0
ENSG00000207009	0	0	0	0
ENSG00000207010	0	0	0	0
ENSG00000207011	0	0	0	0
ENSG00000207012	0	0	0	0
ENSG00000207013	0	0	0	0
ENSG00000207014	0	0	0	0
ENSG00000207016	0	0	0	0
ENSG00000207017	0	0	0	0
ENSG00000207019	0	0	0	0
ENSG00000207020	0	0	0	0
ENSG00000207021	0	0	0	0
ENSG00000207022	0	0	0	0
ENSG00000207023	0	0	0	0
ENSG00000207024	0	0	0	0
ENSG00000207025	0	0	0	0
ENSG00000207026	0	0	0	0
ENSG00000207027	0	0	0	0
ENSG00000207029	0	0	0	0
ENSG00000207031	0	0	0	0
ENSG00000207032	0	0	0	0
ENSG00000207033	0	0	0	0
ENSG00000207034	0	0	0	0
ENSG00000207036	0	0	0	0
ENSG00000207037	0	0	0	0
ENSG00000207039	0	0	0	0
ENSG00000207040	0	0	0	0
ENSG00000207041	0	38	36	9
ENSG00000207042	0	0	0	0
ENSG00000207044	0	0	0	0
ENSG00000207045	0	0	0	0
ENSG00000207046	0	0	0	0
ENSG00000207047	0	0	0	0
ENSG00000207049	0	0	0	0
ENSG00000207051	0	0	0	0
ENSG00000207052	0	0	0	0
ENSG00000207053	0	0	0	0
ENSG00000207056	0	0	0	0
ENSG00000207058	0	0	0	0

ENSG00000207060	0	0	0	0
ENSG00000207061	0	0	0	0
ENSG00000207062	0	0	0	0
ENSG00000207063	0	0	0	0
ENSG00000207065	0	0	0	0
ENSG00000207066	0	0	0	0
ENSG00000207067	0	0	0	0
ENSG00000207068	0	0	0	0
ENSG00000207069	0	0	0	0
ENSG00000207070	0	0	0	0
ENSG00000207071	0	0	0	0
ENSG00000207072	0	0	2	0
ENSG00000207073	0	0	0	0
ENSG00000207074	0	0	0	0
ENSG00000207075	0	0	0	0
ENSG00000207076	0	0	0	0
ENSG00000207077	0	0	0	0
ENSG00000207080	0	0	0	0
ENSG00000207081	0	0	0	0
ENSG00000207082	0	0	0	0
ENSG00000207083	0	0	0	0
ENSG00000207084	0	0	0	0
ENSG00000207086	0	0	0	0
ENSG00000207087	0	0	0	0
ENSG00000207088	0	0	7	28
ENSG00000207089	0	0	0	0
ENSG00000207090	0	0	0	0
ENSG00000207091	0	0	0	0
ENSG00000207092	0	0	0	0
ENSG00000207093	0	0	0	0
ENSG00000207094	0	0	0	0
ENSG00000207095	0	0	0	0
ENSG00000207097	0	0	0	0
ENSG00000207098	0	0	0	0
ENSG00000207099	0	0	0	0
ENSG00000207100	0	0	0	0
ENSG00000207101	2	0	1	2
ENSG00000207103	0	0	0	0
ENSG00000207104	0	0	0	0
ENSG00000207105	0	0	0	0
ENSG00000207106	0	0	0	0
ENSG00000207108	0	0	0	0
ENSG00000207109	0	0	0	0
ENSG00000207110	0	0	0	0
ENSG00000207112	0	0	0	0
ENSG00000207113	0	12	7	1

ENSG00000207114	0	0	0	0
ENSG00000207115	0	0	0	0
ENSG00000207116	0	0	0	0
ENSG00000207117	0	0	0	0
ENSG00000207118	0	0	0	0
ENSG00000207119	0	0	0	0
ENSG00000207121	0	0	0	0
ENSG00000207122	0	0	0	0
ENSG00000207123	0	0	0	0
ENSG00000207124	0	0	0	0
ENSG00000207127	0	0	0	0
ENSG00000207128	0	0	0	0
ENSG00000207129	0	0	0	0
ENSG00000207130	0	0	0	0
ENSG00000207131	0	0	0	0
ENSG00000207132	0	0	0	0
ENSG00000207133	0	0	0	0
ENSG00000207134	0	0	0	0
ENSG00000207135	0	0	0	0
ENSG00000207136	0	0	0	0
ENSG00000207137	0	0	0	0
ENSG00000207138	1	0	1	0
ENSG00000207139	0	0	0	0
ENSG00000207141	0	0	0	0
ENSG00000207142	0	0	1	0
ENSG00000207144	0	0	0	0
ENSG00000207145	0	0	0	0
ENSG00000207146	0	0	0	0
ENSG00000207147	0	0	0	0
ENSG00000207148	0	0	0	0
ENSG00000207149	0	0	0	0
ENSG00000207150	0	0	0	0
ENSG00000207151	0	0	0	0
ENSG00000207152	0	0	0	0
ENSG00000207153	0	0	0	0
ENSG00000207154	0	0	0	0
ENSG00000207155	0	0	0	0
ENSG00000207156	0	0	0	0
ENSG00000207157	0	0	0	0
ENSG00000207158	0	0	0	0
ENSG00000207159	0	0	0	0
ENSG00000207160	0	0	0	0
ENSG00000207161	0	0	0	0
ENSG00000207162	0	0	0	0
ENSG00000207163	0	0	0	0
ENSG00000207164	0	0	0	0

ENSG00000207165	0	0	0	0
ENSG00000207166	0	0	0	0
ENSG00000207167	0	0	0	0
ENSG00000207168	0	0	0	0
ENSG00000207169	0	0	0	0
ENSG00000207170	0	0	0	0
ENSG00000207171	0	0	0	0
ENSG00000207172	0	0	0	0
ENSG00000207173	0	0	0	0
ENSG00000207174	0	0	0	0
ENSG00000207175	0	0	0	0
ENSG00000207176	0	0	0	0
ENSG00000207177	0	0	0	0
ENSG00000207178	0	0	0	0
ENSG00000207180	0	0	0	0
ENSG00000207181	0	0	0	1
ENSG00000207182	0	0	0	0
ENSG00000207183	0	0	0	0
ENSG00000207184	0	0	2	0
ENSG00000207185	0	0	0	0
ENSG00000207186	0	0	0	0
ENSG00000207187	0	0	0	0
ENSG00000207188	0	0	0	0
ENSG00000207189	0	0	0	0
ENSG00000207190	0	0	0	0
ENSG00000207191	0	0	0	0
ENSG00000207192	0	0	0	0
ENSG00000207193	0	0	0	0
ENSG00000207194	0	0	0	0
ENSG00000207195	0	0	0	0
ENSG00000207196	0	0	0	0
ENSG00000207197	0	0	0	0
ENSG00000207198	0	0	0	0
ENSG00000207199	0	0	0	0
ENSG00000207200	0	0	0	0
ENSG00000207201	0	0	0	0
ENSG00000207202	0	0	0	0
ENSG00000207203	0	0	0	0
ENSG00000207204	0	0	0	0
ENSG00000207205	0	0	0	1
ENSG00000207206	0	0	0	0
ENSG00000207207	0	0	0	0
ENSG00000207208	0	0	0	0
ENSG00000207209	0	0	0	0
ENSG00000207210	0	0	0	0
ENSG00000207212	0	0	0	0

ENSG00000207213	0	0	0	0
ENSG00000207214	0	0	0	0
ENSG00000207215	0	0	0	0
ENSG00000207217	0	0	0	0
ENSG00000207218	0	0	0	0
ENSG00000207220	0	0	0	0
ENSG00000207221	0	0	0	0
ENSG00000207222	0	0	0	0
ENSG00000207223	0	0	0	0
ENSG00000207225	0	0	0	0
ENSG00000207227	0	0	0	0
ENSG00000207229	0	0	0	0
ENSG00000207231	0	0	0	0
ENSG00000207233	0	0	0	0
ENSG00000207234	0	0	0	0
ENSG00000207235	0	0	0	0
ENSG00000207236	0	0	0	0
ENSG00000207237	0	0	0	0
ENSG00000207240	0	0	0	0
ENSG00000207241	0	0	0	0
ENSG00000207242	0	0	0	0
ENSG00000207243	0	0	0	0
ENSG00000207244	0	0	0	0
ENSG00000207245	0	0	0	0
ENSG00000207247	0	0	0	0
ENSG00000207248	0	0	0	0
ENSG00000207249	0	0	0	0
ENSG00000207251	0	0	0	0
ENSG00000207252	0	0	0	0
ENSG00000207255	0	0	0	0
ENSG00000207256	0	0	0	0
ENSG00000207257	0	0	0	0
ENSG00000207258	0	0	0	0
ENSG00000207259	0	0	0	0
ENSG00000207260	0	0	0	0
ENSG00000207261	0	0	0	0
ENSG00000207262	0	0	0	0
ENSG00000207263	0	0	0	0
ENSG00000207264	0	0	0	0
ENSG00000207265	0	0	0	0
ENSG00000207266	0	0	0	0
ENSG00000207267	0	0	0	0
ENSG00000207268	0	0	0	0
ENSG00000207269	0	0	0	0
ENSG00000207270	0	0	0	0
ENSG00000207271	0	0	0	0

ENSG00000207274	0	0	0	0
ENSG00000207275	0	0	0	0
ENSG00000207276	4	19	7	6
ENSG00000207277	0	0	0	0
ENSG00000207278	0	0	0	0
ENSG00000207279	0	0	3	0
ENSG00000207280	0	0	0	0
ENSG00000207281	0	0	0	0
ENSG00000207282	0	0	0	0
ENSG00000207283	0	0	0	0
ENSG00000207286	0	0	0	0
ENSG00000207287	0	0	0	0
ENSG00000207289	0	0	0	0
ENSG00000207290	0	0	0	0
ENSG00000207291	0	0	0	0
ENSG00000207292	0	0	0	0
ENSG00000207293	0	0	0	0
ENSG00000207294	0	0	0	0
ENSG00000207295	0	0	0	0
ENSG00000207296	0	0	0	0
ENSG00000207297	0	0	0	0
ENSG00000207298	0	0	0	0
ENSG00000207299	0	0	0	0
ENSG00000207300	0	0	0	0
ENSG00000207302	0	0	0	0
ENSG00000207303	0	0	0	0
ENSG00000207304	0	0	0	0
ENSG00000207305	0	0	0	0
ENSG00000207306	0	0	0	0
ENSG00000207307	0	0	0	0
ENSG00000207308	0	11	0	0
ENSG00000207309	0	0	0	0
ENSG00000207310	0	0	0	0
ENSG00000207311	0	0	0	0
ENSG00000207312	0	0	0	0
ENSG00000207313	0	0	0	0
ENSG00000207314	0	0	0	0
ENSG00000207315	0	0	0	0
ENSG00000207316	0	0	0	0
ENSG00000207317	0	0	0	0
ENSG00000207318	0	0	0	0
ENSG00000207319	0	0	0	0
ENSG00000207320	0	0	0	0
ENSG00000207321	0	0	0	0
ENSG00000207322	0	0	0	0
ENSG00000207323	0	0	0	0

ENSG00000207325	0	0	0	0
ENSG00000207326	0	0	0	0
ENSG00000207327	0	0	0	0
ENSG00000207328	0	0	0	0
ENSG00000207329	0	0	0	0
ENSG00000207330	0	0	0	0
ENSG00000207331	0	0	0	0
ENSG00000207332	0	0	0	0
ENSG00000207333	0	0	0	0
ENSG00000207334	0	0	0	0
ENSG00000207336	0	0	0	0
ENSG00000207338	0	0	0	0
ENSG00000207339	0	0	0	0
ENSG00000207340	0	0	0	0
ENSG00000207341	0	0	0	0
ENSG00000207342	0	0	0	0
ENSG00000207343	0	0	0	0
ENSG00000207344	0	0	0	6
ENSG00000207345	0	0	0	0
ENSG00000207347	0	0	0	0
ENSG00000207349	0	0	0	0
ENSG00000207351	0	0	0	0
ENSG00000207352	0	0	0	0
ENSG00000207355	0	0	0	0
ENSG00000207356	0	0	0	0
ENSG00000207357	0	0	0	0
ENSG00000207359	7	20	11	6
ENSG00000207360	0	0	0	0
ENSG00000207361	0	0	0	0
ENSG00000207362	0	0	0	0
ENSG00000207363	0	0	0	0
ENSG00000207364	0	0	0	0
ENSG00000207365	0	0	0	0
ENSG00000207366	0	0	0	0
ENSG00000207367	0	0	1	0
ENSG00000207368	0	0	0	0
ENSG00000207369	0	0	0	0
ENSG00000207370	0	0	0	1
ENSG00000207371	0	0	0	0
ENSG00000207375	0	0	0	0
ENSG00000207378	0	0	0	0
ENSG00000207379	0	0	0	0
ENSG00000207380	0	0	0	0
ENSG00000207381	0	0	0	0
ENSG00000207382	0	0	0	0
ENSG00000207383	0	0	0	0

ENSG00000207384	0	0	0	0
ENSG00000207385	0	0	0	0
ENSG00000207386	0	0	0	0
ENSG00000207387	0	0	0	0
ENSG00000207389	0	0	0	0
ENSG00000207390	0	0	0	0
ENSG00000207391	0	0	0	0
ENSG00000207392	0	0	0	0
ENSG00000207393	0	0	0	0
ENSG00000207394	0	0	0	0
ENSG00000207395	0	0	0	0
ENSG00000207397	0	0	0	0
ENSG00000207399	0	0	0	0
ENSG00000207401	0	0	0	0
ENSG00000207402	0	0	0	0
ENSG00000207403	0	0	0	0
ENSG00000207404	0	0	0	0
ENSG00000207405	0	0	0	0
ENSG00000207406	0	0	0	0
ENSG00000207407	0	0	0	0
ENSG00000207408	0	0	0	0
ENSG00000207410	0	0	0	0
ENSG00000207411	0	0	0	0
ENSG00000207412	0	0	0	0
ENSG00000207414	0	0	0	0
ENSG00000207415	0	0	0	0
ENSG00000207416	0	0	0	0
ENSG00000207417	0	0	0	0
ENSG00000207418	0	0	0	0
ENSG00000207419	0	0	0	0
ENSG00000207420	0	0	0	0
ENSG00000207421	0	0	0	0
ENSG00000207422	0	0	0	0
ENSG00000207424	0	0	0	0
ENSG00000207425	1	0	2	3
ENSG00000207426	0	0	0	0
ENSG00000207427	0	0	0	0
ENSG00000207428	0	0	0	0
ENSG00000207430	0	0	0	0
ENSG00000207431	0	0	0	0
ENSG00000207432	0	0	0	0
ENSG00000207433	0	0	0	0
ENSG00000207434	0	0	0	0
ENSG00000207435	0	0	0	0
ENSG00000207437	0	0	0	0
ENSG00000207438	0	0	0	0

ENSG00000207439	0	0	0	0
ENSG00000207440	0	0	0	0
ENSG00000207441	0	0	0	0
ENSG00000207442	0	0	0	0
ENSG00000207443	0	0	0	0
ENSG00000207444	0	0	0	0
ENSG00000207445	0	0	0	0
ENSG00000207447	0	30	57	11
ENSG00000207448	0	0	0	0
ENSG00000207449	0	0	0	0
ENSG00000207450	0	0	0	0
ENSG00000207451	0	0	0	0
ENSG00000207452	0	0	0	0
ENSG00000207453	0	0	0	0
ENSG00000207454	0	0	0	0
ENSG00000207455	0	0	2	0
ENSG00000207456	0	0	0	0
ENSG00000207457	0	0	0	0
ENSG00000207458	0	0	0	0
ENSG00000207459	0	0	0	0
ENSG00000207460	0	0	0	0
ENSG00000207461	0	0	0	0
ENSG00000207462	0	0	0	0
ENSG00000207464	0	0	0	0
ENSG00000207465	0	0	0	0
ENSG00000207466	0	0	0	0
ENSG00000207467	0	0	0	0
ENSG00000207468	1	0	0	0
ENSG00000207471	0	0	0	0
ENSG00000207472	0	0	0	0
ENSG00000207473	0	0	0	0
ENSG00000207474	0	0	0	0
ENSG00000207475	0	0	0	0
ENSG00000207476	0	0	0	0
ENSG00000207477	0	0	0	0
ENSG00000207480	0	0	0	0
ENSG00000207481	0	0	0	0
ENSG00000207483	0	0	0	0
ENSG00000207484	0	0	0	0
ENSG00000207486	0	0	0	0
ENSG00000207487	0	0	0	0
ENSG00000207488	0	0	0	0
ENSG00000207490	0	0	0	0
ENSG00000207491	0	0	0	0
ENSG00000207492	0	0	0	0
ENSG00000207493	0	0	0	0

ENSG00000207494	0	0	0	0
ENSG00000207495	0	0	0	0
ENSG00000207496	0	0	0	0
ENSG00000207497	0	0	0	0
ENSG00000207498	2	0	0	0
ENSG00000207499	0	0	0	0
ENSG00000207500	0	0	0	0
ENSG00000207501	1	0	0	2
ENSG00000207502	0	0	0	0
ENSG00000207503	0	0	0	0
ENSG00000207504	0	0	0	0
ENSG00000207505	0	0	0	0
ENSG00000207507	0	0	0	0
ENSG00000207508	0	0	0	0
ENSG00000207511	0	0	0	0
ENSG00000207512	0	0	0	0
ENSG00000207513	0	0	0	0
ENSG00000207514	0	0	0	0
ENSG00000207515	0	0	0	0
ENSG00000207516	0	0	0	0
ENSG00000207518	0	0	0	0
ENSG00000207523	0	0	0	0
ENSG00000207524	0	4	12	6
ENSG00000207525	0	0	0	0
ENSG00000207546	0	0	0	0
ENSG00000207547	0	0	0	0
ENSG00000207548	0	0	0	0
ENSG00000207549	0	0	0	0
ENSG00000207550	0	0	0	0
ENSG00000207551	0	0	0	0
ENSG00000207552	0	0	0	0
ENSG00000207553	0	0	0	0
ENSG00000207554	0	0	0	0
ENSG00000207555	0	0	0	0
ENSG00000207556	0	0	0	0
ENSG00000207558	0	0	0	0
ENSG00000207559	0	0	0	0
ENSG00000207560	0	0	0	0
ENSG00000207561	1	0	0	2
ENSG00000207562	0	0	0	0
ENSG00000207563	0	0	0	0
ENSG00000207567	0	0	0	0
ENSG00000207568	0	0	0	0
ENSG00000207569	0	0	0	0
ENSG00000207570	0	0	0	0
ENSG00000207571	0	0	0	0

ENSG00000207572	0	0	0	0
ENSG00000207573	0	0	0	0
ENSG00000207574	0	0	0	0
ENSG00000207575	0	0	0	0
ENSG00000207577	0	0	0	0
ENSG00000207578	0	0	0	0
ENSG00000207579	0	0	0	0
ENSG00000207580	0	0	0	0
ENSG00000207581	0	0	0	0
ENSG00000207582	0	0	0	0
ENSG00000207583	0	0	0	0
ENSG00000207584	0	0	0	0
ENSG00000207585	0	0	0	0
ENSG00000207586	0	0	0	0
ENSG00000207587	0	0	0	0
ENSG00000207588	0	0	0	0
ENSG00000207589	0	0	0	0
ENSG00000207590	0	0	0	0
ENSG00000207594	0	0	0	0
ENSG00000207595	0	0	0	0
ENSG00000207596	0	0	0	0
ENSG00000207597	0	0	0	0
ENSG00000207598	0	0	0	0
ENSG00000207599	0	0	0	0
ENSG00000207600	0	0	0	0
ENSG00000207601	0	0	0	0
ENSG00000207602	0	0	0	0
ENSG00000207603	0	0	0	0
ENSG00000207604	0	0	0	0
ENSG00000207605	0	0	0	0
ENSG00000207606	0	0	0	0
ENSG00000207607	0	0	0	0
ENSG00000207608	0	0	0	0
ENSG00000207609	0	0	0	0
ENSG00000207610	0	0	0	0
ENSG00000207611	0	0	0	0
ENSG00000207612	0	0	0	0
ENSG00000207613	0	0	0	0
ENSG00000207614	0	0	0	0
ENSG00000207615	0	0	0	0
ENSG00000207616	0	0	0	0
ENSG00000207617	0	0	0	0
ENSG00000207618	0	0	0	0
ENSG00000207619	0	0	0	0
ENSG00000207620	0	0	0	0
ENSG00000207621	0	0	0	0

ENSG00000207622	0	0	0	0
ENSG00000207623	0	0	0	0
ENSG00000207624	0	0	0	0
ENSG00000207625	0	0	0	0
ENSG00000207626	0	0	0	0
ENSG00000207627	0	0	0	0
ENSG00000207628	0	0	0	0
ENSG00000207629	0	0	0	0
ENSG00000207630	0	0	0	0
ENSG00000207631	1	0	0	0
ENSG00000207632	0	0	0	0
ENSG00000207633	0	0	0	0
ENSG00000207634	0	0	0	0
ENSG00000207635	0	0	0	0
ENSG00000207636	0	0	0	0
ENSG00000207637	0	0	0	0
ENSG00000207638	0	0	0	0
ENSG00000207639	0	0	0	0
ENSG00000207641	0	0	0	0
ENSG00000207642	0	0	0	0
ENSG00000207643	0	0	0	0
ENSG00000207644	0	0	0	0
ENSG00000207645	0	0	0	0
ENSG00000207646	0	0	0	0
ENSG00000207647	0	0	0	0
ENSG00000207648	0	0	0	0
ENSG00000207649	0	0	0	0
ENSG00000207650	0	0	0	0
ENSG00000207651	0	0	0	0
ENSG00000207652	5	68	136	114
ENSG00000207653	0	0	0	0
ENSG00000207654	0	0	0	0
ENSG00000207656	0	0	0	0
ENSG00000207688	0	0	0	0
ENSG00000207689	0	0	0	0
ENSG00000207690	0	0	0	0
ENSG00000207691	0	0	0	0
ENSG00000207692	0	0	0	0
ENSG00000207693	0	0	0	0
ENSG00000207694	0	0	0	0
ENSG00000207695	0	0	0	0
ENSG00000207696	0	0	0	0
ENSG00000207697	0	0	1	0
ENSG00000207698	0	0	0	0
ENSG00000207699	0	0	0	0
ENSG00000207700	0	0	0	0

ENSG00000207701	0	0	0	0
ENSG00000207702	0	0	0	0
ENSG00000207703	0	0	0	0
ENSG00000207704	0	0	0	0
ENSG00000207705	0	0	0	0
ENSG00000207706	0	0	0	0
ENSG00000207707	0	0	0	0
ENSG00000207708	0	0	0	0
ENSG00000207709	0	0	0	0
ENSG00000207710	0	0	0	0
ENSG00000207711	0	0	0	0
ENSG00000207712	0	0	0	0
ENSG00000207713	0	0	0	0
ENSG00000207714	0	3	3	6
ENSG00000207715	0	0	0	0
ENSG00000207716	0	0	0	0
ENSG00000207717	0	0	0	0
ENSG00000207718	0	0	0	0
ENSG00000207719	0	0	0	0
ENSG00000207720	0	0	0	0
ENSG00000207721	0	0	0	0
ENSG00000207722	0	0	0	0
ENSG00000207723	0	0	0	0
ENSG00000207724	0	0	0	0
ENSG00000207725	0	0	0	0
ENSG00000207726	0	0	0	0
ENSG00000207727	0	0	0	0
ENSG00000207728	0	0	0	0
ENSG00000207729	0	0	0	0
ENSG00000207730	0	0	0	0
ENSG00000207731	0	0	0	0
ENSG00000207732	0	0	0	0
ENSG00000207733	0	0	0	0
ENSG00000207734	0	0	0	0
ENSG00000207735	0	0	0	0
ENSG00000207736	0	0	0	0
ENSG00000207737	0	0	0	0
ENSG00000207738	0	0	0	0
ENSG00000207739	0	0	0	0
ENSG00000207740	0	0	0	0
ENSG00000207741	0	0	0	0
ENSG00000207742	0	0	0	0
ENSG00000207743	0	0	0	0
ENSG00000207744	0	0	0	0
ENSG00000207745	0	0	0	0
ENSG00000207746	0	0	0	0

ENSG00000207747	0	0	0	0
ENSG00000207748	0	0	0	0
ENSG00000207749	0	0	0	0
ENSG00000207750	0	0	0	0
ENSG00000207751	0	0	0	0
ENSG00000207752	5	0	1	0
ENSG00000207753	0	0	0	0
ENSG00000207754	0	0	0	0
ENSG00000207755	0	0	0	0
ENSG00000207756	0	0	0	0
ENSG00000207757	0	0	0	0
ENSG00000207758	0	0	0	0
ENSG00000207759	0	0	0	0
ENSG00000207761	0	0	0	0
ENSG00000207762	0	0	0	0
ENSG00000207763	0	0	0	0
ENSG00000207764	0	0	0	0
ENSG00000207765	0	0	0	0
ENSG00000207766	0	0	0	0
ENSG00000207767	0	0	0	0
ENSG00000207768	0	0	0	0
ENSG00000207769	0	0	0	0
ENSG00000207770	0	0	0	0
ENSG00000207771	0	0	1	0
ENSG00000207773	0	0	0	0
ENSG00000207775	0	0	0	0
ENSG00000207776	0	0	0	0
ENSG00000207777	0	0	0	0
ENSG00000207778	0	0	0	0
ENSG00000207779	0	0	0	0
ENSG00000207780	0	0	0	0
ENSG00000207781	0	0	0	0
ENSG00000207782	0	0	0	0
ENSG00000207783	0	0	0	0
ENSG00000207784	0	0	0	0
ENSG00000207785	0	0	0	0
ENSG00000207786	0	0	0	0
ENSG00000207787	0	0	0	0
ENSG00000207788	0	0	0	0
ENSG00000207789	0	0	0	0
ENSG00000207790	0	0	0	0
ENSG00000207791	0	0	0	0
ENSG00000207792	0	0	0	0
ENSG00000207793	0	0	0	0
ENSG00000207794	0	0	0	0
ENSG00000207795	0	0	0	0

ENSG00000207797	0	0	0	0
ENSG00000207798	0	0	0	0
ENSG00000207799	0	0	0	0
ENSG00000207800	0	0	0	0
ENSG00000207802	0	0	0	0
ENSG00000207803	0	0	0	0
ENSG00000207804	0	0	0	0
ENSG00000207805	0	0	0	0
ENSG00000207806	0	0	0	0
ENSG00000207807	0	0	0	0
ENSG00000207808	0	0	1	1
ENSG00000207809	0	0	0	0
ENSG00000207810	0	0	0	0
ENSG00000207811	0	0	0	0
ENSG00000207812	0	0	0	0
ENSG00000207813	0	0	0	0
ENSG00000207814	0	0	0	0
ENSG00000207815	0	0	0	0
ENSG00000207816	0	0	0	0
ENSG00000207817	0	0	0	0
ENSG00000207818	0	0	0	0
ENSG00000207819	0	0	0	0
ENSG00000207820	0	0	0	0
ENSG00000207821	1	0	0	0
ENSG00000207825	0	0	0	0
ENSG00000207826	0	0	0	0
ENSG00000207827	0	0	0	0
ENSG00000207830	0	0	0	0
ENSG00000207832	0	0	0	0
ENSG00000207833	0	0	0	0
ENSG00000207834	0	0	0	0
ENSG00000207835	0	0	0	0
ENSG00000207836	0	0	0	0
ENSG00000207837	0	0	0	0
ENSG00000207838	0	0	0	0
ENSG00000207839	0	0	0	0
ENSG00000207844	0	0	0	0
ENSG00000207846	0	0	0	0
ENSG00000207849	0	0	0	0
ENSG00000207857	0	0	0	0
ENSG00000207858	0	0	0	0
ENSG00000207861	0	0	0	0
ENSG00000207862	0	0	0	0
ENSG00000207863	0	0	0	0
ENSG00000207864	0	12	0	0
ENSG00000207865	0	0	0	0

ENSG00000207866	0	0	0	0
ENSG00000207867	0	0	0	0
ENSG00000207868	0	0	0	0
ENSG00000207869	0	0	0	0
ENSG00000207870	0	0	0	0
ENSG00000207871	0	0	0	0
ENSG00000207873	0	0	0	0
ENSG00000207874	0	0	0	0
ENSG00000207875	0	0	0	0
ENSG00000207895	0	0	0	0
ENSG00000207922	0	0	0	0
ENSG00000207923	0	0	0	0
ENSG00000207924	0	0	0	0
ENSG00000207925	0	0	0	0
ENSG00000207926	0	0	0	0
ENSG00000207927	0	0	0	0
ENSG00000207928	0	0	0	0
ENSG00000207929	0	0	0	0
ENSG00000207930	0	0	0	0
ENSG00000207931	0	0	0	0
ENSG00000207932	0	0	0	0
ENSG00000207933	0	0	0	0
ENSG00000207934	0	0	0	0
ENSG00000207935	0	0	0	0
ENSG00000207936	0	0	0	0
ENSG00000207937	0	0	0	0
ENSG00000207938	0	0	0	0
ENSG00000207939	217	490	484	232
ENSG00000207940	0	0	0	0
ENSG00000207941	0	0	0	0
ENSG00000207942	0	0	0	0
ENSG00000207943	0	0	0	0
ENSG00000207944	0	0	0	0
ENSG00000207945	0	0	0	0
ENSG00000207946	0	0	0	0
ENSG00000207947	0	0	0	0
ENSG00000207948	0	0	0	0
ENSG00000207949	0	0	0	0
ENSG00000207950	0	0	0	0
ENSG00000207951	0	0	0	0
ENSG00000207952	0	0	0	0
ENSG00000207953	0	0	0	0
ENSG00000207954	0	0	0	0
ENSG00000207955	0	0	0	0
ENSG00000207956	0	0	0	0
ENSG00000207957	0	0	0	0

ENSG00000207958	0	0	0	0
ENSG00000207959	0	0	0	0
ENSG00000207960	0	0	0	0
ENSG00000207961	0	0	0	0
ENSG00000207962	0	0	0	0
ENSG00000207963	0	0	0	0
ENSG00000207964	0	0	0	0
ENSG00000207965	0	0	0	0
ENSG00000207966	0	0	0	0
ENSG00000207967	0	0	0	0
ENSG00000207968	0	0	0	0
ENSG00000207969	0	0	0	0
ENSG00000207970	0	0	0	0
ENSG00000207971	0	0	0	0
ENSG00000207972	0	0	0	0
ENSG00000207973	0	0	0	0
ENSG00000207974	0	0	0	0
ENSG00000207975	0	0	0	0
ENSG00000207976	0	0	0	0
ENSG00000207977	0	0	0	0
ENSG00000207978	0	0	0	0
ENSG00000207979	0	0	0	0
ENSG00000207980	0	0	4	0
ENSG00000207981	0	0	0	0
ENSG00000207982	0	0	0	0
ENSG00000207983	0	0	0	0
ENSG00000207984	0	0	0	0
ENSG00000207985	0	0	0	0
ENSG00000207986	0	0	0	0
ENSG00000207987	0	0	0	0
ENSG00000207988	0	0	0	0
ENSG00000207989	0	0	0	0
ENSG00000207990	0	0	0	0
ENSG00000207991	0	0	0	0
ENSG00000207992	0	0	0	0
ENSG00000207993	0	0	0	0
ENSG00000207994	0	0	0	0
ENSG00000207995	0	0	0	0
ENSG00000207996	0	0	0	0
ENSG00000207997	0	0	0	0
ENSG00000208000	0	0	0	0
ENSG00000208001	0	0	0	0
ENSG00000208002	0	0	0	0
ENSG00000208003	0	0	0	0
ENSG00000208004	0	0	0	0
ENSG00000208005	0	0	0	0

ENSG00000208006	0	0	0	0
ENSG00000208007	0	0	0	0
ENSG00000208008	0	0	0	0
ENSG00000208009	0	0	0	0
ENSG00000208010	0	0	0	0
ENSG00000208011	0	0	0	0
ENSG00000208012	0	0	0	0
ENSG00000208013	0	0	0	0
ENSG00000208014	0	0	0	0
ENSG00000208015	0	0	0	0
ENSG00000208016	0	0	0	0
ENSG00000208017	0	0	0	0
ENSG00000208018	0	0	0	0
ENSG00000208022	0	0	0	0
ENSG00000208023	0	0	0	0
ENSG00000208024	0	0	0	0
ENSG00000208025	0	0	0	0
ENSG00000208027	0	0	0	0
ENSG00000208028	0	0	0	0
ENSG00000208032	1	23	6	21
ENSG00000208033	0	0	0	0
ENSG00000208034	0	0	0	0
ENSG00000208035	0	0	0	0
ENSG00000208036	0	0	0	0
ENSG00000208037	0	0	0	0
ENSG00000208038	0	0	0	0
ENSG00000208308	0	0	0	0
ENSG00000208313	0	0	0	0
ENSG00000208317	0	0	0	0
ENSG00000208342	0	0	0	0
ENSG00000208506	0	0	0	0
ENSG00000208772	0	0	0	0
ENSG00000208797	0	0	0	0
ENSG00000208839	0	0	0	0
ENSG00000208883	0	0	0	0
ENSG00000208892	0	0	5	4
ENSG00000209042	0	0	0	0
ENSG00000209082	20	0	0	0
ENSG00000209418	0	0	2	0
ENSG00000209480	0	0	0	0
ENSG00000209482	0	0	0	0
ENSG00000209582	0	0	0	0
ENSG00000209645	0	0	0	0
ENSG00000209702	0	0	0	0
ENSG00000209707	0	0	2	1
ENSG00000209804	0	0	0	0

ENSG00000210049	626	10982	8832	9209
ENSG00000210077	701	0	0	0
ENSG00000210082	12991963	97103	536515	75394
ENSG00000210100	3	107	65	34
ENSG00000210107	8	13453	15186	6462
ENSG00000210112	71	9	18	46
ENSG00000210117	1	354	171	170
ENSG00000210127	7	2050	815	311
ENSG00000210135	12	0	0	10
ENSG00000210140	509	0	0	0
ENSG00000210144	2	411	231	208
ENSG00000210151	22	4744	3917	3052
ENSG00000210154	2	93	69	22
ENSG00000210156	6	0	0	0
ENSG00000210164	2	0	0	0
ENSG00000210174	0	0	0	0
ENSG00000210176	8	0	0	0
ENSG00000210181	0	0	0	0
ENSG00000210184	1	0	0	0
ENSG00000210191	1	0	0	0
ENSG00000210194	3	339	256	256
ENSG00000210195	1	571	359	319
ENSG00000210196	1224	26322	18538	21677
ENSG00000210678	0	0	0	0
ENSG00000210709	0	0	0	0
ENSG00000210741	0	0	0	0
ENSG00000210825	0	0	0	0
ENSG00000210839	0	0	0	0
ENSG00000210841	0	0	0	0
ENSG00000211137	0	0	0	0
ENSG00000211445	4	19	30	14
ENSG00000211448	0	0	3	5
ENSG00000211450	15	7	1	7
ENSG00000211451	2	4	11	8
ENSG00000211452	0	0	0	0
ENSG00000211454	0	0	1	0
ENSG00000211455	17	376	579	649
ENSG00000211456	157	4471	4482	4076
ENSG00000211459	4568273	15628	10834	13775
ENSG00000211460	35	541	538	979
ENSG00000211482	0	0	0	0
ENSG00000211483	0	0	0	0
ENSG00000211491	0	0	0	0
ENSG00000211495	0	0	0	0
ENSG00000211498	0	0	0	0
ENSG00000211499	0	0	0	0

ENSG00000211502	0	0	0	0
ENSG00000211508	0	0	0	0
ENSG00000211510	0	0	0	0
ENSG00000211513	0	0	2	0
ENSG00000211514	0	0	0	0
ENSG00000211515	0	0	0	0
ENSG00000211517	0	0	0	0
ENSG00000211518	0	0	0	0
ENSG00000211519	0	0	0	0
ENSG00000211520	0	0	0	0
ENSG00000211521	0	0	0	0
ENSG00000211524	0	0	0	0
ENSG00000211525	0	0	0	0
ENSG00000211526	0	0	0	0
ENSG00000211528	0	0	0	0
ENSG00000211530	0	0	0	0
ENSG00000211532	0	0	0	0
ENSG00000211534	0	0	0	0
ENSG00000211535	0	0	0	0
ENSG00000211538	0	0	0	0
ENSG00000211542	0	0	0	0
ENSG00000211543	0	0	0	0
ENSG00000211544	0	0	0	0
ENSG00000211553	0	0	0	0
ENSG00000211554	0	0	0	0
ENSG00000211556	0	0	0	0
ENSG00000211562	0	0	0	0
ENSG00000211563	0	0	0	0
ENSG00000211566	0	0	0	0
ENSG00000211568	0	0	0	0
ENSG00000211571	0	0	0	0
ENSG00000211573	0	0	0	0
ENSG00000211574	0	0	0	0
ENSG00000211575	0	0	0	0
ENSG00000211577	0	0	0	0
ENSG00000211578	0	0	0	0
ENSG00000211579	0	0	0	0
ENSG00000211580	0	0	0	0
ENSG00000211581	0	0	0	0
ENSG00000211582	0	0	0	0
ENSG00000211583	0	0	0	0
ENSG00000211584	31	11	51	43
ENSG00000211588	0	0	0	0
ENSG00000211589	0	0	0	0
ENSG00000211590	0	0	0	0
ENSG00000211591	0	0	0	0

ENSG00000211592	0	0	0	0
ENSG00000211593	0	0	0	0
ENSG00000211594	0	0	0	0
ENSG00000211595	0	0	0	0
ENSG00000211596	0	0	0	0
ENSG00000211597	0	0	0	0
ENSG00000211598	3	0	0	0
ENSG00000211599	0	0	0	0
ENSG00000211611	0	0	0	0
ENSG00000211619	0	0	0	0
ENSG00000211623	0	0	0	0
ENSG00000211625	0	0	0	0
ENSG00000211626	0	0	0	0
ENSG00000211630	0	0	0	0
ENSG00000211632	0	0	0	0
ENSG00000211633	0	0	0	0
ENSG00000211637	0	0	0	0
ENSG00000211638	0	0	0	0
ENSG00000211639	0	0	0	0
ENSG00000211640	0	0	0	0
ENSG00000211641	0	0	0	0
ENSG00000211642	0	0	0	0
ENSG00000211643	0	0	0	0
ENSG00000211644	1	0	0	0
ENSG00000211645	0	0	0	0
ENSG00000211647	0	0	0	0
ENSG00000211648	0	0	0	0
ENSG00000211649	0	0	0	0
ENSG00000211650	0	0	0	0
ENSG00000211651	0	0	0	0
ENSG00000211652	0	0	0	0
ENSG00000211653	0	0	0	0
ENSG00000211654	0	0	0	0
ENSG00000211655	0	0	0	0
ENSG00000211656	0	0	0	0
ENSG00000211657	0	0	0	0
ENSG00000211658	0	0	0	0
ENSG00000211659	0	0	0	0
ENSG00000211660	1	0	0	0
ENSG00000211661	0	0	0	0
ENSG00000211662	1	0	0	0
ENSG00000211663	1	0	0	0
ENSG00000211664	0	0	0	0
ENSG00000211665	0	0	0	0
ENSG00000211666	3	0	0	0
ENSG00000211667	0	0	0	0

ENSG00000211668	3	0	0	0
ENSG00000211669	0	0	0	0
ENSG00000211670	0	0	0	0
ENSG00000211671	0	0	0	0
ENSG00000211672	0	0	0	0
ENSG00000211673	0	0	0	0
ENSG00000211674	0	0	0	0
ENSG00000211675	0	0	0	0
ENSG00000211676	0	0	0	0
ENSG00000211677	10	0	0	0
ENSG00000211678	0	0	0	0
ENSG00000211679	4	0	2	0
ENSG00000211680	0	0	0	0
ENSG00000211681	0	0	0	0
ENSG00000211682	0	0	0	0
ENSG00000211683	0	0	0	0
ENSG00000211684	0	0	0	0
ENSG00000211685	1	0	0	0
ENSG00000211687	0	0	0	0
ENSG00000211688	0	0	0	0
ENSG00000211689	19	24	0	0
ENSG00000211690	0	0	0	0
ENSG00000211691	0	0	0	0
ENSG00000211692	0	0	0	0
ENSG00000211693	0	0	0	0
ENSG00000211694	1	0	0	0
ENSG00000211695	1	0	1	0
ENSG00000211696	0	0	0	0
ENSG00000211697	1	0	0	19
ENSG00000211698	11	0	1	0
ENSG00000211699	1	25	6	6
ENSG00000211701	0	0	0	0
ENSG00000211706	0	0	0	0
ENSG00000211707	1	0	0	0
ENSG00000211710	2	0	4	11
ENSG00000211713	0	0	0	0
ENSG00000211714	1	21	9	4
ENSG00000211715	0	0	0	0
ENSG00000211716	1	38	0	9
ENSG00000211717	0	0	0	0
ENSG00000211720	0	0	0	0
ENSG00000211721	1	0	2	0
ENSG00000211724	1	0	3	3
ENSG00000211725	1	0	0	6
ENSG00000211727	0	0	0	0
ENSG00000211728	0	0	0	0

ENSG00000211731	0	0	0	0
ENSG00000211732	0	0	0	0
ENSG00000211734	0	0	0	0
ENSG00000211739	0	0	0	0
ENSG00000211745	0	0	0	0
ENSG00000211746	1	0	0	0
ENSG00000211747	0	0	0	0
ENSG00000211749	0	0	0	0
ENSG00000211750	1	0	0	0
ENSG00000211751	8	1	31	71
ENSG00000211752	0	0	0	0
ENSG00000211753	232	228	777	836
ENSG00000211764	0	0	0	0
ENSG00000211765	0	0	0	0
ENSG00000211766	0	0	0	0
ENSG00000211767	3	0	0	0
ENSG00000211768	0	0	0	0
ENSG00000211769	0	0	0	0
ENSG00000211770	0	0	0	0
ENSG00000211771	0	0	0	0
ENSG00000211772	188	0	0	0
ENSG00000211776	0	0	0	0
ENSG00000211777	0	0	0	0
ENSG00000211778	0	0	0	0
ENSG00000211779	0	0	0	0
ENSG00000211780	0	0	0	0
ENSG00000211781	0	0	0	0
ENSG00000211782	0	0	0	0
ENSG00000211783	0	0	0	0
ENSG00000211784	0	0	0	0
ENSG00000211785	0	0	0	0
ENSG00000211786	0	0	0	0
ENSG00000211787	0	0	0	0
ENSG00000211788	0	0	1	0
ENSG00000211789	0	0	0	0
ENSG00000211790	1	0	0	0
ENSG00000211791	3	0	1	7
ENSG00000211792	0	0	0	0
ENSG00000211793	0	0	0	0
ENSG00000211794	1	0	0	0
ENSG00000211795	0	0	0	0
ENSG00000211796	0	0	0	0
ENSG00000211797	0	0	0	0
ENSG00000211798	0	0	0	0
ENSG00000211799	0	0	0	0
ENSG00000211800	1	0	0	0

ENSG00000211801	0	0	0	0
ENSG00000211802	0	0	0	0
ENSG00000211803	0	0	0	0
ENSG00000211804	0	0	0	0
ENSG00000211805	0	0	0	0
ENSG00000211806	0	0	0	0
ENSG00000211807	0	0	0	0
ENSG00000211808	0	0	0	0
ENSG00000211809	0	0	0	0
ENSG00000211810	0	0	0	0
ENSG00000211812	0	0	0	0
ENSG00000211813	0	0	0	0
ENSG00000211814	0	0	0	0
ENSG00000211815	0	0	0	0
ENSG00000211816	0	0	0	0
ENSG00000211817	0	0	0	0
ENSG00000211818	0	0	0	0
ENSG00000211819	0	0	0	0
ENSG00000211820	0	0	0	0
ENSG00000211821	0	0	0	0
ENSG00000211825	5	0	0	0
ENSG00000211826	0	0	0	0
ENSG00000211827	0	0	0	0
ENSG00000211828	0	0	0	0
ENSG00000211829	73	10	1	6
ENSG00000211831	0	0	0	0
ENSG00000211832	0	0	0	0
ENSG00000211833	0	0	0	0
ENSG00000211834	0	0	0	0
ENSG00000211835	0	0	0	0
ENSG00000211836	0	0	0	0
ENSG00000211837	0	0	0	0
ENSG00000211838	0	0	0	0
ENSG00000211839	0	0	0	0
ENSG00000211840	0	0	0	0
ENSG00000211841	0	0	0	0
ENSG00000211842	0	0	0	0
ENSG00000211843	0	0	0	0
ENSG00000211844	0	0	0	0
ENSG00000211845	0	0	0	0
ENSG00000211846	0	0	0	0
ENSG00000211847	0	0	0	0
ENSG00000211848	0	0	0	0
ENSG00000211849	0	0	0	0
ENSG00000211850	0	0	0	0
ENSG00000211851	0	0	0	0

ENSG00000211852	0	0	0	0
ENSG00000211853	0	0	0	0
ENSG00000211854	0	0	0	0
ENSG00000211855	0	0	0	0
ENSG00000211856	0	0	0	0
ENSG00000211857	0	0	0	0
ENSG00000211858	0	0	0	0
ENSG00000211859	0	0	0	0
ENSG00000211860	0	0	0	0
ENSG00000211861	0	0	0	0
ENSG00000211862	0	0	0	0
ENSG00000211863	0	0	0	0
ENSG00000211864	1	0	0	0
ENSG00000211865	0	0	0	0
ENSG00000211866	0	0	0	0
ENSG00000211867	0	0	0	0
ENSG00000211868	0	0	0	0
ENSG00000211869	0	0	0	0
ENSG00000211870	0	0	0	0
ENSG00000211871	0	0	0	0
ENSG00000211872	0	0	0	0
ENSG00000211873	0	0	0	0
ENSG00000211874	0	0	0	0
ENSG00000211875	0	0	0	0
ENSG00000211876	0	0	0	0
ENSG00000211877	0	0	0	0
ENSG00000211878	0	0	0	0
ENSG00000211879	0	0	0	0
ENSG00000211880	0	0	0	0
ENSG00000211881	0	0	0	0
ENSG00000211882	0	0	0	0
ENSG00000211883	0	0	0	5
ENSG00000211884	0	0	0	0
ENSG00000211885	0	0	0	0
ENSG00000211886	0	0	0	0
ENSG00000211887	0	0	0	0
ENSG00000211888	0	0	0	0
ENSG00000211890	4	0	0	0
ENSG00000211891	1	0	0	0
ENSG00000211892	4	35	0	0
ENSG00000211893	2	0	0	0
ENSG00000211895	28	8	0	2
ENSG00000211896	10	0	0	1
ENSG00000211897	10	0	2	0
ENSG00000211898	4	0	0	0
ENSG00000211899	28	0	0	0

ENSG00000211900	0	0	0	0
ENSG00000211904	0	0	0	0
ENSG00000211905	0	0	0	0
ENSG00000211907	0	0	0	0
ENSG00000211909	0	0	0	0
ENSG00000211911	0	0	0	0
ENSG00000211912	0	0	0	0
ENSG00000211914	0	0	0	0
ENSG00000211915	0	0	0	0
ENSG00000211917	0	0	0	0
ENSG00000211918	0	0	0	0
ENSG00000211920	0	0	0	0
ENSG00000211921	0	0	0	0
ENSG00000211923	0	0	0	0
ENSG00000211924	0	0	0	0
ENSG00000211925	0	0	0	0
ENSG00000211928	0	0	0	0
ENSG00000211930	0	0	0	0
ENSG00000211931	0	0	0	0
ENSG00000211933	0	0	0	0
ENSG00000211934	1	0	0	2
ENSG00000211935	24	234	437	132
ENSG00000211936	2	0	1	4
ENSG00000211937	0	0	0	0
ENSG00000211938	0	0	0	0
ENSG00000211939	2	0	0	0
ENSG00000211940	0	51	10	0
ENSG00000211941	0	0	0	0
ENSG00000211942	0	0	0	0
ENSG00000211943	0	0	0	0
ENSG00000211944	0	0	0	0
ENSG00000211945	0	0	0	0
ENSG00000211946	0	0	0	0
ENSG00000211947	1	0	0	0
ENSG00000211949	0	0	0	0
ENSG00000211950	0	0	0	0
ENSG00000211951	0	0	0	0
ENSG00000211952	0	0	0	0
ENSG00000211953	0	0	0	0
ENSG00000211955	1	0	0	0
ENSG00000211956	0	0	0	0
ENSG00000211957	0	0	0	0
ENSG00000211958	0	0	0	0
ENSG00000211959	0	0	0	0
ENSG00000211961	1	0	0	0
ENSG00000211962	0	0	0	0

ENSG00000211964	0	0	0	0
ENSG00000211965	0	0	0	0
ENSG00000211966	0	0	0	0
ENSG00000211967	0	0	0	0
ENSG00000211968	0	0	0	0
ENSG00000211970	0	0	0	0
ENSG00000211972	0	0	0	0
ENSG00000211973	0	0	0	0
ENSG00000211974	0	0	0	0
ENSG00000211976	0	0	0	0
ENSG00000211978	0	0	0	0
ENSG00000211979	0	0	0	0
ENSG00000211983	0	0	0	0
ENSG00000211984	0	0	0	0
ENSG00000211987	0	0	0	0
ENSG00000211990	0	0	0	0
ENSG00000211991	0	0	0	0
ENSG00000211995	0	0	0	0
ENSG00000211996	0	0	0	0
ENSG00000211997	0	0	0	0
ENSG00000212001	0	0	0	0
ENSG00000212007	0	0	0	0
ENSG00000212013	0	0	0	0
ENSG00000212014	0	0	0	0
ENSG00000212017	0	0	0	0
ENSG00000212022	0	0	0	0
ENSG00000212024	0	0	0	0
ENSG00000212025	0	0	0	0
ENSG00000212027	0	0	0	0
ENSG00000212030	0	0	0	0
ENSG00000212031	0	0	0	0
ENSG00000212032	0	0	0	0
ENSG00000212034	0	0	0	0
ENSG00000212036	0	0	0	0
ENSG00000212037	0	0	0	0
ENSG00000212039	0	0	0	0
ENSG00000212040	0	0	0	0
ENSG00000212044	0	0	0	0
ENSG00000212045	0	0	0	0
ENSG00000212049	0	0	0	0
ENSG00000212051	0	0	0	0
ENSG00000212054	0	0	0	0
ENSG00000212055	0	0	0	0
ENSG00000212057	0	0	0	0
ENSG00000212061	0	0	0	0
ENSG00000212063	0	0	0	0

ENSG00000212065	0	0	0	0
ENSG00000212066	0	0	0	0
ENSG00000212067	0	0	0	0
ENSG00000212070	0	0	0	0
ENSG00000212071	0	0	0	0
ENSG00000212072	0	0	0	0
ENSG00000212073	0	0	0	0
ENSG00000212075	0	0	0	0
ENSG00000212076	0	0	0	0
ENSG00000212082	0	0	0	0
ENSG00000212084	1	0	0	0
ENSG00000212085	0	0	0	0
ENSG00000212086	0	0	0	0
ENSG00000212089	0	0	0	0
ENSG00000212091	0	0	0	0
ENSG00000212093	0	0	0	0
ENSG00000212094	0	0	0	0
ENSG00000212095	0	0	0	0
ENSG00000212098	0	0	0	0
ENSG00000212099	0	0	0	0
ENSG00000212100	0	0	0	0
ENSG00000212101	0	0	0	0
ENSG00000212102	0	0	0	0
ENSG00000212104	0	0	0	0
ENSG00000212111	0	0	0	0
ENSG00000212114	0	0	0	0
ENSG00000212122	0	0	0	0
ENSG00000212123	0	0	0	0
ENSG00000212124	0	11	17	25
ENSG00000212125	0	0	0	3
ENSG00000212126	0	0	0	3
ENSG00000212127	10	0	36	28
ENSG00000212128	0	0	0	0
ENSG00000212133	0	0	0	0
ENSG00000212134	0	0	0	0
ENSG00000212135	0	0	0	0
ENSG00000212136	0	0	0	0
ENSG00000212138	0	0	0	0
ENSG00000212140	0	0	0	0
ENSG00000212144	0	0	0	0
ENSG00000212145	0	0	0	0
ENSG00000212146	0	0	0	0
ENSG00000212147	0	0	0	0
ENSG00000212148	0	0	0	0
ENSG00000212149	0	0	0	0
ENSG00000212153	0	0	0	0

ENSG00000212154	0	0	0	0
ENSG00000212156	0	0	0	0
ENSG00000212157	0	0	0	0
ENSG00000212158	0	0	0	0
ENSG00000212160	0	0	0	0
ENSG00000212161	0	0	0	0
ENSG00000212163	0	0	0	0
ENSG00000212165	0	0	0	0
ENSG00000212167	0	0	0	0
ENSG00000212168	0	0	0	0
ENSG00000212170	0	0	0	0
ENSG00000212171	0	0	0	0
ENSG00000212172	0	0	0	0
ENSG00000212174	0	0	0	0
ENSG00000212175	0	0	0	0
ENSG00000212176	0	0	0	0
ENSG00000212181	0	0	0	0
ENSG00000212182	0	0	0	0
ENSG00000212184	0	0	0	0
ENSG00000212186	0	0	0	0
ENSG00000212187	0	0	0	0
ENSG00000212189	0	0	0	0
ENSG00000212190	0	0	0	0
ENSG00000212191	0	0	0	0
ENSG00000212195	0	0	0	0
ENSG00000212197	0	0	0	0
ENSG00000212199	0	0	0	0
ENSG00000212204	0	0	0	0
ENSG00000212205	0	0	0	0
ENSG00000212206	2	0	3	1
ENSG00000212207	0	0	0	0
ENSG00000212211	0	0	0	0
ENSG00000212214	0	0	0	0
ENSG00000212215	0	0	0	0
ENSG00000212216	0	0	0	0
ENSG00000212219	0	0	0	0
ENSG00000212221	0	0	0	0
ENSG00000212224	282	237	201	110
ENSG00000212226	0	0	0	0
ENSG00000212228	0	0	0	0
ENSG00000212229	0	0	0	0
ENSG00000212230	0	0	0	0
ENSG00000212232	0	0	2	4
ENSG00000212237	0	0	0	0
ENSG00000212238	0	0	0	0
ENSG00000212240	0	0	0	0

ENSG00000212241	0	0	0	0
ENSG00000212242	1	0	0	0
ENSG00000212246	0	0	0	0
ENSG00000212247	0	0	0	0
ENSG00000212248	0	0	0	0
ENSG00000212249	0	0	0	0
ENSG00000212251	0	0	0	0
ENSG00000212254	0	0	0	0
ENSG00000212257	0	0	0	0
ENSG00000212258	0	0	0	0
ENSG00000212259	0	0	0	0
ENSG00000212260	0	0	0	0
ENSG00000212264	0	0	0	0
ENSG00000212265	0	0	0	0
ENSG00000212266	0	0	0	0
ENSG00000212269	0	0	0	0
ENSG00000212270	0	0	0	0
ENSG00000212273	0	0	0	0
ENSG00000212276	0	0	0	0
ENSG00000212277	0	0	0	0
ENSG00000212278	0	0	0	0
ENSG00000212279	0	0	2	1
ENSG00000212280	0	0	0	0
ENSG00000212282	0	0	0	0
ENSG00000212283	0	0	0	0
ENSG00000212284	0	0	0	0
ENSG00000212289	0	0	0	0
ENSG00000212290	0	0	0	0
ENSG00000212292	0	0	0	0
ENSG00000212293	0	0	0	0
ENSG00000212295	0	0	0	0
ENSG00000212296	0	0	0	0
ENSG00000212297	0	0	3	5
ENSG00000212298	0	0	0	0
ENSG00000212300	0	0	0	0
ENSG00000212302	0	0	0	0
ENSG00000212303	0	0	0	0
ENSG00000212304	0	0	2	0
ENSG00000212305	0	0	0	0
ENSG00000212306	0	0	0	0
ENSG00000212308	0	0	0	0
ENSG00000212309	0	0	0	0
ENSG00000212312	0	0	0	0
ENSG00000212314	0	0	0	0
ENSG00000212316	0	0	0	0
ENSG00000212319	0	0	0	0

ENSG00000212321	0	0	0	0
ENSG00000212324	0	0	0	0
ENSG00000212325	0	0	0	0
ENSG00000212327	0	0	0	0
ENSG00000212329	0	0	0	0
ENSG00000212330	0	0	0	0
ENSG00000212331	0	0	0	0
ENSG00000212332	0	0	0	0
ENSG00000212333	0	0	0	0
ENSG00000212335	0	0	0	0
ENSG00000212336	0	0	0	0
ENSG00000212338	0	0	0	0
ENSG00000212340	0	0	0	0
ENSG00000212342	0	0	0	0
ENSG00000212344	0	0	0	0
ENSG00000212345	0	0	0	0
ENSG00000212347	0	0	0	0
ENSG00000212348	0	0	0	0
ENSG00000212354	0	0	0	0
ENSG00000212358	0	0	0	0
ENSG00000212359	0	0	0	0
ENSG00000212360	0	0	0	0
ENSG00000212363	0	0	0	0
ENSG00000212365	0	0	0	0
ENSG00000212366	0	0	0	0
ENSG00000212367	0	0	0	0
ENSG00000212368	0	0	0	0
ENSG00000212370	0	0	0	0
ENSG00000212371	0	0	0	0
ENSG00000212373	0	0	0	0
ENSG00000212374	0	0	0	0
ENSG00000212377	0	0	0	0
ENSG00000212378	0	0	0	0
ENSG00000212379	0	0	0	0
ENSG00000212380	0	0	0	0
ENSG00000212381	0	0	0	0
ENSG00000212382	0	0	0	0
ENSG00000212383	0	0	0	0
ENSG00000212384	0	0	0	0
ENSG00000212385	0	0	0	0
ENSG00000212387	0	0	0	0
ENSG00000212388	0	0	0	0
ENSG00000212389	0	0	0	0
ENSG00000212391	0	0	0	0
ENSG00000212392	0	0	0	0
ENSG00000212395	0	0	0	0

ENSG00000212396	0	0	0	0
ENSG00000212397	0	0	0	0
ENSG00000212398	0	0	2	1
ENSG00000212402	0	0	0	0
ENSG00000212404	0	0	0	0
ENSG00000212407	0	0	0	0
ENSG00000212409	0	0	0	0
ENSG00000212410	0	0	0	0
ENSG00000212411	0	0	0	0
ENSG00000212413	0	0	0	0
ENSG00000212414	0	0	0	0
ENSG00000212415	0	0	0	0
ENSG00000212418	0	0	0	0
ENSG00000212420	0	0	0	0
ENSG00000212421	0	0	0	0
ENSG00000212422	0	0	0	0
ENSG00000212424	0	0	0	0
ENSG00000212425	0	0	0	0
ENSG00000212428	0	0	0	0
ENSG00000212429	0	0	0	0
ENSG00000212432	0	0	0	0
ENSG00000212433	0	0	0	0
ENSG00000212434	0	0	0	0
ENSG00000212440	0	0	0	0
ENSG00000212441	0	0	0	0
ENSG00000212442	0	0	0	0
ENSG00000212443	0	0	1	0
ENSG00000212445	0	0	0	0
ENSG00000212446	0	0	0	0
ENSG00000212447	0	0	0	0
ENSG00000212448	0	0	0	0
ENSG00000212450	0	0	0	0
ENSG00000212451	0	0	0	0
ENSG00000212452	0	0	0	0
ENSG00000212454	0	0	0	0
ENSG00000212455	0	0	0	0
ENSG00000212456	0	0	0	0
ENSG00000212457	0	0	0	0
ENSG00000212458	0	0	0	0
ENSG00000212459	0	0	0	0
ENSG00000212460	0	0	0	0
ENSG00000212461	0	0	0	0
ENSG00000212464	0	0	0	0
ENSG00000212466	0	0	0	0
ENSG00000212468	0	0	0	0
ENSG00000212469	0	0	0	0

ENSG00000212473	0	0	0	0
ENSG00000212475	0	0	0	0
ENSG00000212479	10	0	1	0
ENSG00000212482	0	0	0	0
ENSG00000212485	0	0	0	0
ENSG00000212487	0	0	0	0
ENSG00000212489	0	0	0	0
ENSG00000212490	0	0	0	0
ENSG00000212493	0	0	0	0
ENSG00000212495	0	0	0	0
ENSG00000212496	0	0	0	0
ENSG00000212497	0	0	0	0
ENSG00000212498	0	0	0	0
ENSG00000212499	0	0	0	0
ENSG00000212502	0	0	0	0
ENSG00000212505	0	0	0	0
ENSG00000212510	0	0	0	0
ENSG00000212511	0	0	0	0
ENSG00000212512	0	0	0	0
ENSG00000212516	0	0	0	0
ENSG00000212517	0	0	0	0
ENSG00000212518	0	0	0	0
ENSG00000212520	0	0	0	0
ENSG00000212521	0	0	0	0
ENSG00000212522	0	0	0	0
ENSG00000212525	0	0	0	0
ENSG00000212526	0	0	1	0
ENSG00000212527	0	0	0	0
ENSG00000212528	0	0	0	0
ENSG00000212529	0	0	0	0
ENSG00000212532	0	0	0	0
ENSG00000212533	0	0	0	0
ENSG00000212534	0	0	0	0
ENSG00000212535	0	0	0	0
ENSG00000212536	0	0	0	0
ENSG00000212538	0	0	0	0
ENSG00000212539	0	0	0	0
ENSG00000212541	0	0	0	0
ENSG00000212542	0	0	0	0
ENSG00000212544	0	0	0	0
ENSG00000212545	0	0	0	0
ENSG00000212546	0	0	0	0
ENSG00000212549	0	0	0	0
ENSG00000212550	0	0	0	0
ENSG00000212551	0	0	0	0
ENSG00000212552	0	0	0	0

ENSG00000212553	0	0	0	0
ENSG00000212555	0	0	0	0
ENSG00000212556	0	0	0	0
ENSG00000212558	0	0	0	0
ENSG00000212559	0	0	0	0
ENSG00000212560	0	0	0	0
ENSG00000212561	0	0	0	0
ENSG00000212564	0	0	0	0
ENSG00000212565	0	0	0	0
ENSG00000212567	0	0	0	0
ENSG00000212568	0	0	0	0
ENSG00000212569	0	0	0	0
ENSG00000212571	0	0	0	0
ENSG00000212572	0	0	0	0
ENSG00000212576	0	0	0	0
ENSG00000212579	0	0	0	0
ENSG00000212580	0	0	0	0
ENSG00000212581	0	0	0	0
ENSG00000212582	0	0	0	2
ENSG00000212584	0	0	0	0
ENSG00000212585	0	0	0	0
ENSG00000212586	0	0	0	0
ENSG00000212587	0	0	0	0
ENSG00000212588	0	0	0	0
ENSG00000212589	0	0	0	0
ENSG00000212590	0	0	0	0
ENSG00000212593	0	0	0	0
ENSG00000212594	0	0	0	0
ENSG00000212595	0	0	0	0
ENSG00000212597	0	0	0	0
ENSG00000212598	0	0	0	0
ENSG00000212599	0	0	0	0
ENSG00000212601	0	0	0	0
ENSG00000212604	0	0	0	0
ENSG00000212605	0	0	0	0
ENSG00000212607	0	0	0	0
ENSG00000212608	0	0	0	0
ENSG00000212609	0	0	0	0
ENSG00000212610	0	0	0	0
ENSG00000212611	0	0	0	0
ENSG00000212612	0	0	0	0
ENSG00000212615	0	0	0	0
ENSG00000212618	0	0	0	0
ENSG00000212620	0	0	0	0
ENSG00000212623	0	0	0	0
ENSG00000212624	0	0	0	0

ENSG00000212625	0	0	0	0
ENSG00000212626	0	0	0	0
ENSG00000212628	0	0	0	0
ENSG00000212643	0	0	0	0
ENSG00000212657	1	0	2	0
ENSG00000212658	3	0	1	3
ENSG00000212659	0	0	0	0
ENSG00000212663	0	0	0	0
ENSG00000212664	2	0	0	0
ENSG00000212670	1	0	0	0
ENSG00000212673	0	0	0	0
ENSG00000212694	800	230	142	142
ENSG00000212695	0	0	0	0
ENSG00000212710	0	0	0	0
ENSG00000212712	0	0	0	0
ENSG00000212717	0	0	0	0
ENSG00000212719	3	0	0	0
ENSG00000212721	0	0	0	0
ENSG00000212722	0	0	0	0
ENSG00000212724	0	0	0	0
ENSG00000212725	0	0	0	0
ENSG00000212734	0	0	0	0
ENSG00000212743	0	0	2	2
ENSG00000212747	15	42	25	12
ENSG00000212766	2	19	7	6
ENSG00000212768	0	0	0	0
ENSG00000212769	0	0	0	0
ENSG00000212789	0	0	0	2
ENSG00000212802	4	18	17	22
ENSG00000212807	0	0	0	0
ENSG00000212829	0	0	0	0
ENSG00000212855	0	0	0	0
ENSG00000212856	0	0	0	1
ENSG00000212857	0	0	0	0
ENSG00000212864	86	9	9	32
ENSG00000212866	0	0	0	0
ENSG00000212884	4	0	3	3
ENSG00000212899	0	0	0	0
ENSG00000212900	0	0	0	0
ENSG00000212901	0	0	0	0
ENSG00000212907	68674	9435	20520	17790
ENSG00000212916	2	64	33	47
ENSG00000212928	0	0	24	12
ENSG00000212930	0	0	0	0
ENSG00000212932	0	0	0	1
ENSG00000212933	0	0	0	0

ENSG00000212935	0	0	0	0
ENSG00000212938	0	0	0	0
ENSG00000212939	0	0	0	0
ENSG00000212950	0	0	0	0
ENSG00000212951	0	0	0	0
ENSG00000212952	0	0	0	0
ENSG00000212961	12	1	0	0
ENSG00000212978	8	0	1	0
ENSG00000212989	0	0	0	0
ENSG00000212993	0	0	0	0
ENSG00000212994	0	0	1	0
ENSG00000212997	0	0	0	0
ENSG00000212998	6	22	8	5
ENSG00000212999	0	39	147	66
ENSG00000213002	0	132	314	108
ENSG00000213003	0	0	0	0
ENSG00000213005	0	0	0	0
ENSG00000213013	0	0	0	0
ENSG00000213014	0	0	0	0
ENSG00000213015	106	12	16	24
ENSG00000213016	0	0	0	0
ENSG00000213018	1	0	0	0
ENSG00000213020	27	62	80	71
ENSG00000213022	0	0	0	0
ENSG00000213023	0	0	0	0
ENSG00000213024	47	1	3	18
ENSG00000213025	4	0	10	6
ENSG00000213026	0	0	0	0
ENSG00000213028	0	0	0	0
ENSG00000213029	0	0	0	0
ENSG00000213030	0	0	0	0
ENSG00000213032	0	0	0	0
ENSG00000213033	0	0	0	0
ENSG00000213035	1	0	0	0
ENSG00000213036	0	0	0	0
ENSG00000213041	0	0	0	0
ENSG00000213045	0	0	0	0
ENSG00000213046	0	0	0	0
ENSG00000213047	12	4292	2893	5104
ENSG00000213048	0	0	0	0
ENSG00000213049	0	0	0	0
ENSG00000213050	0	0	0	0
ENSG00000213051	0	0	0	0
ENSG00000213055	0	0	0	0
ENSG00000213057	0	0	0	0
ENSG00000213058	1	0	0	0

ENSG00000213060	0	0	0	0
ENSG00000213061	0	0	0	0
ENSG00000213062	0	0	0	0
ENSG00000213063	0	0	0	0
ENSG00000213064	21	226	258	219
ENSG00000213065	0	0	0	0
ENSG00000213066	2	374	206	343
ENSG00000213067	0	0	0	0
ENSG00000213068	0	0	0	0
ENSG00000213069	0	0	0	0
ENSG00000213070	0	0	0	0
ENSG00000213071	0	0	1	0
ENSG00000213073	1	0	0	0
ENSG00000213075	0	0	0	0
ENSG00000213076	0	0	0	0
ENSG00000213077	0	0	0	0
ENSG00000213078	0	0	0	0
ENSG00000213079	17	3741	2896	4274
ENSG00000213080	1	0	1	3
ENSG00000213081	0	0	0	0
ENSG00000213082	0	0	0	0
ENSG00000213083	0	0	0	2
ENSG00000213084	0	0	0	0
ENSG00000213085	31	219	158	176
ENSG00000213087	0	0	0	0
ENSG00000213088	1	0	0	0
ENSG00000213089	0	0	0	0
ENSG00000213090	0	1	10	67
ENSG00000213091	0	0	0	0
ENSG00000213096	1	115	226	419
ENSG00000213100	0	0	0	0
ENSG00000213104	0	0	0	0
ENSG00000213107	0	0	0	0
ENSG00000213108	0	0	0	0
ENSG00000213109	2	7	3	9
ENSG00000213110	0	0	0	0
ENSG00000213111	0	0	0	0
ENSG00000213113	3	31	0	18
ENSG00000213115	0	0	0	0
ENSG00000213117	0	0	0	0
ENSG00000213118	0	0	0	0
ENSG00000213120	0	0	0	0
ENSG00000213121	0	52	76	77
ENSG00000213122	0	0	0	0
ENSG00000213123	1	0	0	0
ENSG00000213126	0	0	0	0

ENSG00000213128	0	0	0	0
ENSG00000213130	0	0	0	0
ENSG00000213131	0	1	1	2
ENSG00000213132	0	0	0	0
ENSG00000213133	0	0	0	0
ENSG00000213135	0	0	0	0
ENSG00000213137	0	0	0	0
ENSG00000213139	0	0	0	0
ENSG00000213140	0	0	0	0
ENSG00000213144	0	0	6	8
ENSG00000213145	6	0	0	0
ENSG00000213147	0	0	0	0
ENSG00000213148	0	0	0	0
ENSG00000213149	0	0	0	9
ENSG00000213150	0	0	0	0
ENSG00000213152	1	0	0	0
ENSG00000213153	0	0	0	0
ENSG00000213155	0	0	0	0
ENSG00000213157	0	0	0	0
ENSG00000213158	0	0	0	0
ENSG00000213159	0	0	0	0
ENSG00000213160	1	0	4	19
ENSG00000213165	0	0	0	0
ENSG00000213167	0	0	0	0
ENSG00000213169	0	0	0	0
ENSG00000213170	0	0	0	0
ENSG00000213171	0	0	0	0
ENSG00000213172	0	0	0	0
ENSG00000213174	0	0	0	0
ENSG00000213176	0	0	0	0
ENSG00000213177	0	0	0	0
ENSG00000213178	0	0	0	2
ENSG00000213179	0	0	0	0
ENSG00000213180	0	0	0	0
ENSG00000213181	0	0	0	0
ENSG00000213182	0	0	0	0
ENSG00000213183	0	0	0	0
ENSG00000213184	0	0	0	0
ENSG00000213185	2	0	0	0
ENSG00000213186	5	61	19	21
ENSG00000213187	0	0	0	0
ENSG00000213188	0	0	0	0
ENSG00000213189	0	0	0	0
ENSG00000213190	17	480	299	208
ENSG00000213194	0	0	0	0
ENSG00000213197	0	0	0	0

ENSG00000213199	1	0	0	0
ENSG00000213201	0	0	0	0
ENSG00000213203	25	0	2	0
ENSG00000213204	1	10	5	1
ENSG00000213205	0	0	0	0
ENSG00000213209	0	0	0	0
ENSG00000213210	0	0	0	0
ENSG00000213211	0	0	0	0
ENSG00000213212	0	0	0	0
ENSG00000213213	0	0	1	3
ENSG00000213214	0	0	0	0
ENSG00000213215	0	0	0	0
ENSG00000213216	0	0	0	0
ENSG00000213218	0	0	0	0
ENSG00000213221	0	0	0	0
ENSG00000213222	0	0	0	0
ENSG00000213225	0	0	0	0
ENSG00000213226	0	0	0	0
ENSG00000213228	2	0	0	0
ENSG00000213231	0	0	0	0
ENSG00000213232	0	0	0	4
ENSG00000213233	0	0	0	0
ENSG00000213234	0	0	0	0
ENSG00000213235	0	1	0	3
ENSG00000213236	0	2	9	3
ENSG00000213237	11	0	0	0
ENSG00000213238	0	0	0	0
ENSG00000213239	0	0	0	0
ENSG00000213240	43	188	265	455
ENSG00000213244	0	0	0	0
ENSG00000213246	2504	8235	9734	5375
ENSG00000213247	0	0	0	0
ENSG00000213250	0	0	0	0
ENSG00000213252	0	0	0	0
ENSG00000213253	0	0	0	0
ENSG00000213260	0	5	5	4
ENSG00000213261	0	1	0	0
ENSG00000213262	1	0	0	0
ENSG00000213264	0	0	0	0
ENSG00000213265	1	0	0	0
ENSG00000213269	0	0	0	0
ENSG00000213270	0	0	0	0
ENSG00000213272	0	0	0	0
ENSG00000213275	0	0	0	0
ENSG00000213277	0	0	0	0
ENSG00000213279	2	0	0	0

ENSG00000213280	0	0	0	0
ENSG00000213281	79	1079	1785	1347
ENSG00000213283	0	0	0	0
ENSG00000213285	0	0	0	2
ENSG00000213287	0	0	0	0
ENSG00000213290	0	0	0	0
ENSG00000213291	0	0	0	0
ENSG00000213293	0	0	0	0
ENSG00000213295	0	0	0	0
ENSG00000213296	0	0	0	0
ENSG00000213297	0	0	0	0
ENSG00000213300	6	13	8	14
ENSG00000213301	0	0	0	0
ENSG00000213302	0	0	0	0
ENSG00000213303	0	0	0	0
ENSG00000213304	0	0	0	0
ENSG00000213305	0	0	0	0
ENSG00000213307	0	0	0	0
ENSG00000213309	0	0	0	0
ENSG00000213310	0	0	0	0
ENSG00000213312	0	0	0	0
ENSG00000213315	3	0	1	0
ENSG00000213316	0	0	0	0
ENSG00000213318	0	0	0	0
ENSG00000213326	0	0	0	0
ENSG00000213328	0	0	0	0
ENSG00000213331	0	0	0	1
ENSG00000213332	0	0	0	0
ENSG00000213333	3	0	2	0
ENSG00000213335	0	0	0	0
ENSG00000213337	3	4	5	0
ENSG00000213338	0	0	0	0
ENSG00000213339	11	0	0	0
ENSG00000213341	6	515	458	472
ENSG00000213343	0	0	0	0
ENSG00000213344	0	0	0	0
ENSG00000213347	2	6	6	7
ENSG00000213352	0	0	0	0
ENSG00000213355	0	0	0	0
ENSG00000213356	0	0	0	0
ENSG00000213358	0	0	0	0
ENSG00000213361	0	0	0	0
ENSG00000213362	2	0	0	2
ENSG00000213363	2	1	0	0
ENSG00000213365	0	0	0	0
ENSG00000213366	71	263	208	285

ENSG00000213368	0	0	0	0
ENSG00000213370	0	0	0	0
ENSG00000213371	0	0	0	0
ENSG00000213373	0	0	0	0
ENSG00000213375	0	0	0	0
ENSG00000213376	0	0	0	0
ENSG00000213380	0	0	3	3
ENSG00000213383	0	0	0	0
ENSG00000213384	0	0	0	1
ENSG00000213385	0	0	0	0
ENSG00000213386	0	0	0	0
ENSG00000213390	4	46	31	69
ENSG00000213391	0	0	0	0
ENSG00000213393	0	0	0	0
ENSG00000213394	2	0	0	0
ENSG00000213397	0	0	0	0
ENSG00000213398	0	0	3	0
ENSG00000213399	0	0	0	0
ENSG00000213400	0	0	0	0
ENSG00000213401	0	0	0	0
ENSG00000213402	23	0	2	4
ENSG00000213403	0	0	0	0
ENSG00000213406	0	0	0	0
ENSG00000213409	0	0	0	0
ENSG00000213410	0	0	0	0
ENSG00000213411	3	0	1	2
ENSG00000213412	0	0	2	0
ENSG00000213413	0	0	0	0
ENSG00000213414	0	0	0	0
ENSG00000213416	0	0	0	0
ENSG00000213417	0	0	0	0
ENSG00000213420	0	0	0	0
ENSG00000213421	0	0	0	0
ENSG00000213423	0	0	0	0
ENSG00000213424	0	0	0	0
ENSG00000213430	0	0	0	0
ENSG00000213431	0	0	0	1
ENSG00000213432	0	0	0	0
ENSG00000213433	2	0	1	0
ENSG00000213434	0	0	0	0
ENSG00000213435	0	0	0	0
ENSG00000213438	0	0	0	0
ENSG00000213439	0	0	0	0
ENSG00000213440	0	0	0	0
ENSG00000213442	6	0	0	5
ENSG00000213443	5	0	1	0

ENSG00000213445	9	11	12	24
ENSG00000213448	0	0	0	0
ENSG00000213449	0	0	0	0
ENSG00000213450	0	0	0	0
ENSG00000213451	0	0	0	0
ENSG00000213452	0	0	0	0
ENSG00000213453	2	7	2	10
ENSG00000213455	0	0	0	0
ENSG00000213461	0	0	0	0
ENSG00000213462	1508	1428	1872	578
ENSG00000213463	9	15	8	9
ENSG00000213465	118	8	10	25
ENSG00000213467	1	1	1	0
ENSG00000213468	0	59	91	121
ENSG00000213470	0	0	0	0
ENSG00000213471	0	0	0	0
ENSG00000213478	0	0	0	0
ENSG00000213480	0	0	0	0
ENSG00000213483	0	0	0	0
ENSG00000213484	0	0	0	0
ENSG00000213486	0	0	0	0
ENSG00000213487	0	0	0	0
ENSG00000213488	0	0	0	0
ENSG00000213489	0	0	0	0
ENSG00000213492	27	194	295	11
ENSG00000213493	0	0	0	0
ENSG00000213494	0	0	0	2
ENSG00000213495	0	0	0	0
ENSG00000213498	0	0	0	0
ENSG00000213500	0	0	0	0
ENSG00000213509	0	0	0	0
ENSG00000213512	0	0	0	1
ENSG00000213513	0	0	0	0
ENSG00000213514	0	0	0	0
ENSG00000213516	85	76	61	62
ENSG00000213519	0	0	0	0
ENSG00000213522	0	0	0	0
ENSG00000213523	41	36	13	14
ENSG00000213525	0	0	0	0
ENSG00000213526	0	0	0	0
ENSG00000213527	0	0	0	0
ENSG00000213529	0	0	0	0
ENSG00000213530	0	0	0	0
ENSG00000213533	4	0	4	3
ENSG00000213536	0	0	0	0
ENSG00000213538	0	0	0	0

ENSG00000213539	0	0	0	0
ENSG00000213540	0	0	0	0
ENSG00000213542	0	0	0	0
ENSG00000213543	1	0	4	17
ENSG00000213547	0	0	0	0
ENSG00000213548	0	0	0	0
ENSG00000213549	0	0	1	3
ENSG00000213551	48	1145	923	1038
ENSG00000213553	1	0	1	0
ENSG00000213556	0	0	0	0
ENSG00000213557	0	0	0	0
ENSG00000213558	0	0	0	0
ENSG00000213559	0	0	0	0
ENSG00000213560	0	0	0	0
ENSG00000213561	0	0	0	0
ENSG00000213563	4	0	10	4
ENSG00000213568	0	0	0	0
ENSG00000213569	0	0	0	0
ENSG00000213574	0	0	0	0
ENSG00000213578	0	0	0	0
ENSG00000213579	0	0	0	0
ENSG00000213585	69	358	474	695
ENSG00000213587	0	0	0	0
ENSG00000213588	0	0	0	3
ENSG00000213590	3	0	0	0
ENSG00000213592	0	0	0	0
ENSG00000213593	14	15	24	19
ENSG00000213594	0	0	0	0
ENSG00000213598	0	0	0	0
ENSG00000213599	0	0	0	0
ENSG00000213600	0	0	0	0
ENSG00000213601	0	0	0	0
ENSG00000213604	0	0	0	0
ENSG00000213605	0	0	0	0
ENSG00000213606	0	0	0	0
ENSG00000213607	0	0	0	0
ENSG00000213608	0	0	0	0
ENSG00000213609	7	0	0	0
ENSG00000213612	0	0	0	0
ENSG00000213613	0	0	0	0
ENSG00000213614	13	67	40	46
ENSG00000213619	17	0	9	5
ENSG00000213620	0	0	0	0
ENSG00000213621	0	0	0	0
ENSG00000213622	0	0	0	0
ENSG00000213625	5418	24151	31042	17283

ENSG00000213626	43	131	6	8
ENSG00000213630	0	0	0	0
ENSG00000213638	0	0	0	0
ENSG00000213639	327	3517	5142	4004
ENSG00000213640	0	0	0	0
ENSG00000213641	0	0	0	0
ENSG00000213642	0	0	0	0
ENSG00000213643	0	0	0	0
ENSG00000213644	0	0	0	0
ENSG00000213645	0	0	0	0
ENSG00000213648	0	0	0	0
ENSG00000213650	0	0	0	0
ENSG00000213652	0	0	0	0
ENSG00000213653	0	0	0	0
ENSG00000213654	1516	958	904	1233
ENSG00000213655	0	0	0	0
ENSG00000213657	0	0	5	5
ENSG00000213658	2	0	0	0
ENSG00000213659	0	41	2	5
ENSG00000213661	0	0	0	0
ENSG00000213663	0	0	0	0
ENSG00000213664	0	0	0	0
ENSG00000213667	0	0	0	0
ENSG00000213669	0	0	0	0
ENSG00000213671	1	0	0	0
ENSG00000213672	249	191	123	349
ENSG00000213673	0	0	0	0
ENSG00000213676	29	58	43	101
ENSG00000213683	0	0	0	0
ENSG00000213684	0	0	0	0
ENSG00000213686	0	0	0	0
ENSG00000213689	142	252	166	241
ENSG00000213690	0	0	0	0
ENSG00000213693	0	0	3	0
ENSG00000213694	1	0	0	0
ENSG00000213695	0	0	0	0
ENSG00000213697	0	0	1	0
ENSG00000213698	0	0	0	0
ENSG00000213699	1	0	0	0
ENSG00000213700	0	0	1	0
ENSG00000213701	0	0	0	0
ENSG00000213702	0	0	0	0
ENSG00000213703	0	0	0	0
ENSG00000213704	0	0	0	0
ENSG00000213706	0	0	0	0
ENSG00000213707	6	5	8	6

ENSG00000213708	0	0	0	0
ENSG00000213711	0	0	0	0
ENSG00000213713	0	0	0	0
ENSG00000213714	0	0	0	0
ENSG00000213716	0	0	0	0
ENSG00000213717	0	0	0	0
ENSG00000213719	3117	7047	4032	4348
ENSG00000213721	0	0	0	0
ENSG00000213722	96	69	107	38
ENSG00000213724	0	0	0	0
ENSG00000213726	1	0	0	0
ENSG00000213727	0	0	0	0
ENSG00000213729	0	0	0	0
ENSG00000213730	0	0	0	0
ENSG00000213731	0	0	0	0
ENSG00000213735	0	0	0	0
ENSG00000213736	0	0	0	0
ENSG00000213739	0	0	0	0
ENSG00000213740	0	0	0	0
ENSG00000213741	1927	42	26	44
ENSG00000213742	0	0	7	5
ENSG00000213744	0	0	0	0
ENSG00000213747	0	0	0	0
ENSG00000213750	0	0	0	0
ENSG00000213752	0	0	0	0
ENSG00000213753	38	87	36	142
ENSG00000213754	0	0	0	0
ENSG00000213755	0	0	0	0
ENSG00000213757	1	0	0	0
ENSG00000213759	37	23	37	60
ENSG00000213760	2	0	2	3
ENSG00000213761	0	0	0	0
ENSG00000213762	4	20	11	22
ENSG00000213763	0	1	0	12
ENSG00000213770	0	0	0	0
ENSG00000213771	0	0	0	0
ENSG00000213772	0	0	1	0
ENSG00000213774	0	0	0	0
ENSG00000213777	0	0	0	0
ENSG00000213778	0	0	0	0
ENSG00000213779	0	0	0	0
ENSG00000213780	0	0	0	0
ENSG00000213781	0	0	0	0
ENSG00000213782	0	0	0	0
ENSG00000213783	0	0	0	0
ENSG00000213785	0	0	0	0

ENSG00000213786	0	0	0	0
ENSG00000213787	0	0	0	0
ENSG00000213790	0	0	0	0
ENSG00000213791	0	0	0	0
ENSG00000213793	0	20	7	22
ENSG00000213798	0	0	0	0
ENSG00000213799	3	0	19	11
ENSG00000213801	0	0	0	0
ENSG00000213809	0	0	0	1
ENSG00000213816	0	0	0	0
ENSG00000213820	0	0	0	0
ENSG00000213822	0	0	0	0
ENSG00000213828	0	0	0	0
ENSG00000213830	0	0	0	0
ENSG00000213839	4	0	0	0
ENSG00000213842	0	0	1	0
ENSG00000213846	1	2	2	1
ENSG00000213849	1	0	0	0
ENSG00000213851	0	0	0	0
ENSG00000213853	0	0	0	0
ENSG00000213854	0	0	0	0
ENSG00000213856	0	0	0	0
ENSG00000213857	0	0	0	0
ENSG00000213859	2	21	2	2
ENSG00000213860	3	0	0	9
ENSG00000213862	0	0	0	0
ENSG00000213863	0	0	0	0
ENSG00000213864	0	0	0	0
ENSG00000213865	20	0	7	10
ENSG00000213866	0	0	0	2
ENSG00000213867	0	0	0	0
ENSG00000213871	0	0	1	0
ENSG00000213872	0	0	0	0
ENSG00000213873	0	0	0	0
ENSG00000213875	0	0	0	0
ENSG00000213876	0	0	0	0
ENSG00000213877	0	0	0	0
ENSG00000213880	0	0	0	0
ENSG00000213881	0	0	0	1
ENSG00000213882	0	0	0	0
ENSG00000213885	1	0	0	0
ENSG00000213886	0	0	0	0
ENSG00000213888	55	41	25	60
ENSG00000213889	346	179	73	184
ENSG00000213891	0	0	0	0
ENSG00000213892	0	0	0	0

ENSG00000213896	0	0	0	0
ENSG00000213900	0	0	0	0
ENSG00000213901	6	6	0	0
ENSG00000213903	2	9	3	9
ENSG00000213904	2	0	1	0
ENSG00000213906	0	0	3	0
ENSG00000213908	0	0	0	0
ENSG00000213911	0	0	0	0
ENSG00000213916	0	0	0	0
ENSG00000213917	0	0	0	0
ENSG00000213918	9	22	21	14
ENSG00000213920	4	0	0	0
ENSG00000213921	0	0	0	0
ENSG00000213922	0	0	0	0
ENSG00000213923	4	0	6	6
ENSG00000213924	0	0	0	0
ENSG00000213925	0	0	0	0
ENSG00000213926	0	0	0	0
ENSG00000213927	0	0	0	0
ENSG00000213928	0	5	0	0
ENSG00000213930	11	0	0	0
ENSG00000213931	9	152	18	51
ENSG00000213934	157	231	62	85
ENSG00000213935	0	0	0	0
ENSG00000213937	0	0	0	0
ENSG00000213938	0	0	0	0
ENSG00000213939	0	0	0	0
ENSG00000213940	0	0	0	0
ENSG00000213942	0	0	0	0
ENSG00000213943	0	0	0	0
ENSG00000213946	0	0	0	0
ENSG00000213949	11	60	90	224
ENSG00000213950	0	0	0	0
ENSG00000213953	0	0	0	0
ENSG00000213954	0	0	0	0
ENSG00000213956	0	0	0	0
ENSG00000213958	0	0	0	0
ENSG00000213959	0	0	0	0
ENSG00000213962	0	0	0	0
ENSG00000213963	0	0	1	7
ENSG00000213964	0	0	0	0
ENSG00000213965	30	178	149	252
ENSG00000213967	3	154	97	112
ENSG00000213970	0	0	0	0
ENSG00000213971	17	28	14	38
ENSG00000213972	0	0	0	0

ENSG00000213973	0	0	5	2
ENSG00000213976	1	0	2	24
ENSG00000213977	331	95	54	17
ENSG00000213979	0	0	0	0
ENSG00000213981	0	0	0	0
ENSG00000213983	3	1	13	16
ENSG00000213985	0	0	0	1
ENSG00000213987	0	0	0	0
ENSG00000213988	2	44	56	84
ENSG00000213994	0	0	1	0
ENSG00000213995	1	66	67	86
ENSG00000213996	0	0	0	0
ENSG00000213997	0	0	0	0
ENSG00000213999	0	0	0	1
ENSG00000214003	0	0	0	0
ENSG00000214006	0	0	0	0
ENSG00000214009	0	0	0	0
ENSG00000214012	0	0	0	0
ENSG00000214013	21	290	266	341
ENSG00000214014	0	0	0	0
ENSG00000214015	0	0	0	0
ENSG00000214016	0	1	0	0
ENSG00000214018	13	788	940	598
ENSG00000214019	0	0	1	0
ENSG00000214020	0	0	0	0
ENSG00000214021	2	1	11	3
ENSG00000214022	9	0	6	1
ENSG00000214024	0	0	0	0
ENSG00000214025	0	0	0	0
ENSG00000214026	9	17	10	12
ENSG00000214027	0	2	0	0
ENSG00000214029	2	9	0	2
ENSG00000214031	0	0	0	0
ENSG00000214035	0	0	0	0
ENSG00000214039	1	0	0	0
ENSG00000214041	0	0	0	0
ENSG00000214042	0	0	0	0
ENSG00000214043	0	0	0	0
ENSG00000214045	0	0	0	0
ENSG00000214046	472	556	439	738
ENSG00000214047	0	0	0	0
ENSG00000214049	0	0	0	0
ENSG00000214050	0	0	0	0
ENSG00000214051	0	0	0	0
ENSG00000214062	0	0	0	0
ENSG00000214063	0	27	1	1

ENSG00000214064	0	0	0	0
ENSG00000214067	0	0	0	0
ENSG00000214070	0	0	0	0
ENSG00000214071	0	0	0	0
ENSG00000214073	0	0	0	0
ENSG00000214074	0	0	2	0
ENSG00000214076	0	0	0	2
ENSG00000214077	0	3	10	1
ENSG00000214078	82	97	57	66
ENSG00000214081	0	0	0	0
ENSG00000214087	48	89	43	56
ENSG00000214089	0	0	0	0
ENSG00000214093	0	0	0	0
ENSG00000214097	0	0	0	0
ENSG00000214100	0	0	0	0
ENSG00000214102	1	0	17	2
ENSG00000214105	0	0	0	0
ENSG00000214106	10	0	2	1
ENSG00000214107	0	0	0	0
ENSG00000214108	0	0	0	2
ENSG00000214109	0	0	0	0
ENSG00000214110	0	0	1	0
ENSG00000214111	0	0	0	0
ENSG00000214113	19	53	10	0
ENSG00000214114	153	773	1039	1162
ENSG00000214117	0	0	0	0
ENSG00000214121	9	17	27	27
ENSG00000214124	0	0	0	0
ENSG00000214125	0	0	0	0
ENSG00000214128	0	0	0	0
ENSG00000214132	0	0	0	0
ENSG00000214135	1	26	38	17
ENSG00000214140	0	0	0	0
ENSG00000214141	0	0	0	0
ENSG00000214142	0	0	0	0
ENSG00000214144	0	0	0	0
ENSG00000214145	2	0	4	2
ENSG00000214146	0	0	0	0
ENSG00000214147	0	0	0	0
ENSG00000214160	14	10	13	11
ENSG00000214161	0	0	0	0
ENSG00000214164	0	0	0	0
ENSG00000214167	0	0	0	0
ENSG00000214174	72	187	179	121
ENSG00000214176	6	55	7	42
ENSG00000214178	0	0	0	0

ENSG00000214179	0	0	0	0
ENSG00000214181	0	0	1	0
ENSG00000214182	13	4	1	10
ENSG00000214184	0	13	1	0
ENSG00000214185	0	0	0	0
ENSG00000214188	0	0	0	10
ENSG00000214189	1	29	3	14
ENSG00000214190	0	0	0	0
ENSG00000214192	0	0	0	0
ENSG00000214193	1	1	1	5
ENSG00000214194	231	619	812	1021
ENSG00000214195	0	0	0	0
ENSG00000214198	0	28	28	32
ENSG00000214199	0	0	0	0
ENSG00000214200	0	0	0	0
ENSG00000214203	0	0	0	0
ENSG00000214204	0	0	0	0
ENSG00000214207	0	0	0	0
ENSG00000214210	0	0	0	0
ENSG00000214211	0	0	0	0
ENSG00000214212	30	0	0	5
ENSG00000214215	0	0	0	0
ENSG00000214216	0	0	0	0
ENSG00000214222	0	0	0	0
ENSG00000214223	0	0	0	0
ENSG00000214226	6	187	182	227
ENSG00000214237	1	0	0	0
ENSG00000214239	0	0	0	0
ENSG00000214243	0	0	0	0
ENSG00000214244	0	0	0	0
ENSG00000214245	0	0	0	0
ENSG00000214248	0	0	0	0
ENSG00000214249	0	0	0	0
ENSG00000214252	0	0	0	0
ENSG00000214253	345	319	117	201
ENSG00000214254	0	0	0	0
ENSG00000214255	0	0	0	0
ENSG00000214259	0	0	0	3
ENSG00000214262	1	0	2	0
ENSG00000214263	0	0	0	0
ENSG00000214264	0	0	0	0
ENSG00000214265	2	16	1	21
ENSG00000214266	0	0	0	0
ENSG00000214268	0	0	0	0
ENSG00000214269	0	0	0	0
ENSG00000214273	28	36	39	151

ENSG00000214274	0	0	0	0
ENSG00000214278	0	0	0	0
ENSG00000214279	0	0	7	6
ENSG00000214280	0	0	3	9
ENSG00000214281	0	0	0	0
ENSG00000214282	0	0	0	0
ENSG00000214283	0	0	0	0
ENSG00000214285	0	0	0	0
ENSG00000214286	0	0	0	0
ENSG00000214288	0	0	0	0
ENSG00000214289	0	0	0	0
ENSG00000214290	0	0	0	0
ENSG00000214293	12	12	15	14
ENSG00000214295	0	0	0	0
ENSG00000214297	0	0	0	0
ENSG00000214298	0	0	0	0
ENSG00000214300	0	0	0	0
ENSG00000214301	0	0	0	0
ENSG00000214305	0	0	0	0
ENSG00000214306	0	0	0	0
ENSG00000214309	1	0	1	1
ENSG00000214313	0	0	0	0
ENSG00000214314	0	0	0	0
ENSG00000214318	0	0	0	0
ENSG00000214319	0	0	0	0
ENSG00000214321	0	0	0	2
ENSG00000214322	0	0	0	0
ENSG00000214324	0	0	0	0
ENSG00000214325	0	0	0	0
ENSG00000214326	0	0	0	0
ENSG00000214329	0	0	0	0
ENSG00000214330	0	0	0	0
ENSG00000214331	0	42	50	62
ENSG00000214335	0	0	0	0
ENSG00000214336	0	0	0	0
ENSG00000214338	0	0	0	0
ENSG00000214342	0	0	0	0
ENSG00000214344	0	0	0	0
ENSG00000214347	0	0	0	0
ENSG00000214351	0	0	0	2
ENSG00000214353	1	0	0	6
ENSG00000214354	0	0	0	0
ENSG00000214357	21	36	97	84
ENSG00000214359	0	0	0	0
ENSG00000214360	0	0	5	0
ENSG00000214362	0	0	0	0

ENSG00000214366	0	0	1	0
ENSG00000214367	4	28	35	20
ENSG00000214369	0	0	0	0
ENSG00000214374	3	0	6	4
ENSG00000214376	0	0	0	0
ENSG00000214380	0	0	0	0
ENSG00000214381	0	0	0	0
ENSG00000214389	0	1	1	1
ENSG00000214391	0	0	1	0
ENSG00000214397	0	0	0	0
ENSG00000214401	32	43	41	208
ENSG00000214402	0	0	0	0
ENSG00000214405	0	0	0	0
ENSG00000214407	0	0	0	0
ENSG00000214413	51	396	409	367
ENSG00000214414	0	0	0	0
ENSG00000214415	0	0	0	0
ENSG00000214417	0	0	0	0
ENSG00000214424	0	0	0	0
ENSG00000214425	3	28	3	0
ENSG00000214428	0	0	0	0
ENSG00000214429	0	0	0	0
ENSG00000214432	0	0	0	0
ENSG00000214433	0	0	0	0
ENSG00000214434	0	0	0	0
ENSG00000214435	0	0	5	1
ENSG00000214439	0	52	119	96
ENSG00000214447	0	0	0	0
ENSG00000214455	0	0	0	0
ENSG00000214456	0	0	0	0
ENSG00000214457	0	0	0	0
ENSG00000214460	0	0	0	0
ENSG00000214465	0	0	0	0
ENSG00000214479	0	0	0	0
ENSG00000214484	0	0	3	0
ENSG00000214485	1	1	7	10
ENSG00000214487	0	0	0	0
ENSG00000214491	0	0	0	0
ENSG00000214510	0	0	0	0
ENSG00000214511	0	0	1	0
ENSG00000214513	0	0	0	0
ENSG00000214514	0	0	0	0
ENSG00000214517	5	70	33	42
ENSG00000214518	0	0	0	0
ENSG00000214525	0	0	0	0
ENSG00000214526	0	0	0	0

ENSG00000214530	12	42	10	37
ENSG00000214533	0	0	0	0
ENSG00000214534	0	16	9	23
ENSG00000214535	2	0	0	0
ENSG00000214541	0	0	0	0
ENSG00000214544	0	26	16	19
ENSG00000214546	0	0	0	0
ENSG00000214548	2	35	21	19
ENSG00000214549	0	0	0	0
ENSG00000214552	0	0	0	0
ENSG00000214553	0	0	0	0
ENSG00000214556	0	0	0	0
ENSG00000214558	0	0	0	3
ENSG00000214559	4	5	4	2
ENSG00000214560	0	0	0	0
ENSG00000214561	0	0	0	0
ENSG00000214562	1	0	0	6
ENSG00000214563	0	0	0	0
ENSG00000214566	0	0	0	0
ENSG00000214575	0	0	0	0
ENSG00000214578	0	0	0	0
ENSG00000214581	0	0	0	0
ENSG00000214584	0	0	0	0
ENSG00000214593	0	0	0	0
ENSG00000214595	20	114	133	222
ENSG00000214597	0	0	0	0
ENSG00000214602	0	0	0	0
ENSG00000214604	0	0	0	0
ENSG00000214607	0	0	0	0
ENSG00000214612	1	0	0	0
ENSG00000214614	0	0	0	0
ENSG00000214617	0	0	0	0
ENSG00000214626	0	0	0	0
ENSG00000214628	0	0	0	0
ENSG00000214629	0	0	0	0
ENSG00000214641	0	0	0	0
ENSG00000214642	0	0	0	0
ENSG00000214643	0	0	0	0
ENSG00000214645	1	0	0	0
ENSG00000214646	0	0	0	0
ENSG00000214650	0	0	0	0
ENSG00000214651	0	0	0	0
ENSG00000214652	0	0	0	0
ENSG00000214653	0	0	0	0
ENSG00000214654	0	2	3	5
ENSG00000214655	5	19	13	19

ENSG00000214657	0	0	0	0
ENSG00000214659	0	0	0	0
ENSG00000214660	0	0	0	0
ENSG00000214668	0	0	0	0
ENSG00000214669	0	0	0	0
ENSG00000214670	0	0	0	0
ENSG00000214671	0	0	2	0
ENSG00000214676	0	0	0	0
ENSG00000214681	0	0	0	0
ENSG00000214684	0	0	0	0
ENSG00000214686	0	0	0	0
ENSG00000214688	0	0	0	0
ENSG00000214691	0	0	0	0
ENSG00000214694	3	1	21	40
ENSG00000214695	0	0	0	0
ENSG00000214696	0	0	0	0
ENSG00000214700	0	0	0	0
ENSG00000214702	0	0	0	0
ENSG00000214706	1	0	1	0
ENSG00000214708	1	0	0	0
ENSG00000214711	0	0	1	1
ENSG00000214717	25	8	6	27
ENSG00000214719	6	0	2	3
ENSG00000214720	0	0	0	0
ENSG00000214725	0	0	0	0
ENSG00000214727	0	0	0	0
ENSG00000214732	0	0	0	0
ENSG00000214733	0	0	0	0
ENSG00000214736	0	0	0	0
ENSG00000214743	0	0	0	0
ENSG00000214745	0	0	0	0
ENSG00000214748	2	0	0	3
ENSG00000214753	91	125	81	79
ENSG00000214754	0	0	0	0
ENSG00000214756	3	0	0	7
ENSG00000214759	0	0	0	0
ENSG00000214760	0	0	0	0
ENSG00000214761	0	0	0	0
ENSG00000214765	20	127	459	486
ENSG00000214770	0	0	2	0
ENSG00000214772	0	0	2	0
ENSG00000214773	0	0	0	0
ENSG00000214776	25	76	193	368
ENSG00000214782	0	0	0	0
ENSG00000214783	26	77	58	127
ENSG00000214784	0	0	0	1

ENSG00000214787	0	0	0	0
ENSG00000214788	0	26	1	12
ENSG00000214794	0	0	0	0
ENSG00000214796	1	0	1	1
ENSG00000214797	0	0	0	0
ENSG00000214803	0	0	0	0
ENSG00000214807	0	0	0	0
ENSG00000214810	0	0	0	0
ENSG00000214812	0	0	0	0
ENSG00000214814	0	12	2	1
ENSG00000214815	0	0	0	0
ENSG00000214819	1	0	0	0
ENSG00000214820	0	0	1	0
ENSG00000214821	2	0	0	0
ENSG00000214822	0	0	0	0
ENSG00000214823	0	0	0	0
ENSG00000214825	0	0	0	0
ENSG00000214826	0	0	0	0
ENSG00000214827	0	0	1	7
ENSG00000214832	1	0	3	0
ENSG00000214835	0	0	0	0
ENSG00000214837	0	97	44	70
ENSG00000214841	0	0	0	0
ENSG00000214842	0	0	0	0
ENSG00000214843	0	0	0	0
ENSG00000214844	0	0	0	0
ENSG00000214846	0	0	0	0
ENSG00000214851	0	0	0	0
ENSG00000214853	0	0	0	0
ENSG00000214854	0	0	0	0
ENSG00000214855	0	0	0	0
ENSG00000214856	0	0	0	0
ENSG00000214857	0	0	0	0
ENSG00000214860	0	0	0	0
ENSG00000214866	0	0	0	0
ENSG00000214867	0	0	0	0
ENSG00000214869	0	0	0	0
ENSG00000214870	1	0	1	1
ENSG00000214871	0	0	0	0
ENSG00000214872	0	0	0	0
ENSG00000214875	0	0	0	0
ENSG00000214878	0	0	0	0
ENSG00000214880	0	0	0	0
ENSG00000214881	0	0	0	0
ENSG00000214883	0	0	0	0
ENSG00000214886	0	14	0	0

ENSG00000214888	0	0	0	0
ENSG00000214889	0	0	0	0
ENSG00000214890	0	0	0	0
ENSG00000214891	0	0	0	0
ENSG00000214892	1	1	2	14
ENSG00000214894	51	70	56	2
ENSG00000214896	0	0	0	0
ENSG00000214897	0	0	0	0
ENSG00000214900	2	0	0	0
ENSG00000214903	0	0	0	0
ENSG00000214904	0	0	0	0
ENSG00000214908	0	0	0	0
ENSG00000214914	0	0	0	0
ENSG00000214915	0	0	0	0
ENSG00000214917	0	0	0	0
ENSG00000214919	0	0	0	0
ENSG00000214921	0	0	0	0
ENSG00000214922	2	0	1	11
ENSG00000214925	0	0	0	0
ENSG00000214929	0	0	0	0
ENSG00000214930	0	0	0	0
ENSG00000214940	0	0	0	0
ENSG00000214941	236	768	775	676
ENSG00000214942	0	0	0	0
ENSG00000214943	0	0	0	0
ENSG00000214944	1	185	174	248
ENSG00000214946	0	0	0	0
ENSG00000214954	0	87	106	93
ENSG00000214955	1	0	0	0
ENSG00000214960	6	1737	2989	4484
ENSG00000214961	0	0	0	0
ENSG00000214967	0	0	3	0
ENSG00000214970	0	0	0	0
ENSG00000214973	0	0	0	0
ENSG00000214975	0	0	0	0
ENSG00000214976	0	0	0	0
ENSG00000214978	0	0	0	0
ENSG00000214980	0	0	0	0
ENSG00000214982	1	213	139	135
ENSG00000214985	0	0	0	0
ENSG00000214986	0	0	0	0
ENSG00000214988	0	0	0	0
ENSG00000214992	0	0	0	0
ENSG00000214998	0	0	0	0
ENSG00000214999	0	0	0	0
ENSG00000215000	0	0	0	0

ENSG00000215002	0	0	0	0
ENSG00000215003	0	0	1	0
ENSG00000215004	0	0	0	0
ENSG00000215005	0	0	0	0
ENSG00000215006	0	0	0	0
ENSG00000215007	0	0	0	0
ENSG00000215009	0	0	0	0
ENSG00000215012	4	0	0	0
ENSG00000215014	2	0	3	0
ENSG00000215016	0	0	0	0
ENSG00000215018	4	0	8	5
ENSG00000215019	0	0	0	0
ENSG00000215020	0	0	0	0
ENSG00000215021	88	6	17	30
ENSG00000215022	0	407	329	527
ENSG00000215023	0	0	0	0
ENSG00000215024	0	0	0	0
ENSG00000215029	0	0	0	0
ENSG00000215030	15	0	1	7
ENSG00000215032	0	0	0	0
ENSG00000215033	0	0	0	0
ENSG00000215034	0	0	0	0
ENSG00000215035	0	0	0	0
ENSG00000215037	0	0	0	0
ENSG00000215039	887	2383	1325	1313
ENSG00000215041	1	0	0	0
ENSG00000215043	0	0	0	0
ENSG00000215044	0	0	0	0
ENSG00000215045	0	0	2	1
ENSG00000215046	0	0	0	0
ENSG00000215048	0	0	0	0
ENSG00000215049	0	0	0	0
ENSG00000215054	0	0	0	0
ENSG00000215057	0	0	0	0
ENSG00000215063	0	0	0	0
ENSG00000215065	0	0	0	0
ENSG00000215066	0	0	0	0
ENSG00000215067	14	155	68	45
ENSG00000215068	1	0	2	7
ENSG00000215070	0	0	0	0
ENSG00000215077	0	0	0	0
ENSG00000215085	0	0	0	0
ENSG00000215086	0	0	0	0
ENSG00000215088	0	0	0	0
ENSG00000215089	0	0	0	0
ENSG00000215093	0	0	0	0

ENSG00000215094	0	0	0	0
ENSG00000215096	0	0	0	0
ENSG00000215097	0	0	0	0
ENSG00000215102	0	0	0	0
ENSG00000215104	5	84	51	82
ENSG00000215105	0	0	1	7
ENSG00000215110	0	0	0	0
ENSG00000215112	0	0	0	0
ENSG00000215113	0	0	0	0
ENSG00000215114	25	282	550	219
ENSG00000215115	0	0	0	0
ENSG00000215117	0	0	0	0
ENSG00000215120	0	0	0	0
ENSG00000215124	0	0	0	0
ENSG00000215126	0	0	8	4
ENSG00000215127	0	0	0	0
ENSG00000215131	0	0	0	0
ENSG00000215142	0	0	0	0
ENSG00000215146	0	10	0	6
ENSG00000215148	0	0	0	0
ENSG00000215149	0	0	0	0
ENSG00000215151	0	0	0	0
ENSG00000215154	4	0	0	1
ENSG00000215156	0	7	0	0
ENSG00000215158	1	77	76	130
ENSG00000215159	0	0	0	0
ENSG00000215160	0	0	0	0
ENSG00000215162	0	0	0	0
ENSG00000215165	0	0	0	0
ENSG00000215168	0	0	0	0
ENSG00000215174	0	0	0	0
ENSG00000215177	0	0	0	0
ENSG00000215179	0	0	0	2
ENSG00000215182	0	0	1	0
ENSG00000215183	0	0	1	4
ENSG00000215184	0	0	0	0
ENSG00000215186	0	0	0	0
ENSG00000215187	0	0	0	0
ENSG00000215190	2	8	8	22
ENSG00000215193	10	81	47	79
ENSG00000215196	1	0	0	0
ENSG00000215197	0	0	0	0
ENSG00000215198	0	0	0	0
ENSG00000215199	0	0	0	1
ENSG00000215203	0	0	0	0
ENSG00000215204	0	0	0	0

ENSG00000215206	0	0	0	0
ENSG00000215208	0	0	0	0
ENSG00000215210	0	0	0	0
ENSG00000215217	0	0	0	0
ENSG00000215218	0	0	0	0
ENSG00000215221	1	0	0	0
ENSG00000215223	0	0	0	0
ENSG00000215227	0	0	0	0
ENSG00000215231	0	0	0	0
ENSG00000215236	0	0	0	0
ENSG00000215237	2	13	4	4
ENSG00000215241	0	0	2	0
ENSG00000215244	2	0	0	0
ENSG00000215246	0	0	0	0
ENSG00000215251	110	406	1057	1021
ENSG00000215252	0	0	1	0
ENSG00000215256	94	220	304	365
ENSG00000215262	0	0	0	0
ENSG00000215263	0	0	0	0
ENSG00000215264	0	0	0	0
ENSG00000215267	0	74	18	29
ENSG00000215268	0	0	0	0
ENSG00000215269	0	0	0	0
ENSG00000215270	0	0	0	0
ENSG00000215271	7	19	15	28
ENSG00000215274	0	0	0	0
ENSG00000215277	58	132	119	453
ENSG00000215278	0	0	0	0
ENSG00000215283	0	0	0	0
ENSG00000215284	0	0	0	0
ENSG00000215286	0	0	0	0
ENSG00000215288	0	0	0	0
ENSG00000215296	0	0	0	0
ENSG00000215297	0	0	0	0
ENSG00000215298	0	0	0	0
ENSG00000215301	159	952	1668	771
ENSG00000215302	48	461	624	4211
ENSG00000215304	0	0	0	0
ENSG00000215305	10	0	3	8
ENSG00000215306	0	0	0	0
ENSG00000215307	0	0	0	0
ENSG00000215310	0	0	0	0
ENSG00000215311	0	0	0	0
ENSG00000215313	0	0	0	0
ENSG00000215317	0	0	0	0
ENSG00000215319	0	0	1	0

ENSG00000215323	0	0	0	0
ENSG00000215325	0	0	0	0
ENSG00000215326	0	0	0	1
ENSG00000215328	0	0	0	0
ENSG00000215333	0	0	0	0
ENSG00000215339	0	0	0	0
ENSG00000215343	0	0	0	0
ENSG00000215346	0	0	0	0
ENSG00000215347	0	0	0	0
ENSG00000215349	0	0	0	0
ENSG00000215351	0	0	0	0
ENSG00000215353	0	0	0	0
ENSG00000215354	0	0	0	0
ENSG00000215356	0	0	0	0
ENSG00000215357	0	0	0	0
ENSG00000215365	0	0	0	0
ENSG00000215367	0	0	0	0
ENSG00000215368	0	0	0	0
ENSG00000215369	0	0	0	0
ENSG00000215371	0	0	0	0
ENSG00000215372	0	0	0	0
ENSG00000215373	0	0	0	0
ENSG00000215374	0	0	0	0
ENSG00000215375	1	0	9	3
ENSG00000215378	0	0	0	0
ENSG00000215380	0	0	0	0
ENSG00000215381	0	0	0	0
ENSG00000215386	1	0	0	3
ENSG00000215388	0	0	0	1
ENSG00000215394	0	0	0	0
ENSG00000215397	0	0	0	0
ENSG00000215398	0	0	0	0
ENSG00000215399	0	0	0	0
ENSG00000215403	0	0	0	0
ENSG00000215405	0	0	0	0
ENSG00000215409	0	0	0	0
ENSG00000215414	0	0	0	0
ENSG00000215417	1	0	2	1
ENSG00000215418	0	0	0	0
ENSG00000215421	4	93	90	211
ENSG00000215424	1	15	0	4
ENSG00000215425	0	0	0	0
ENSG00000215428	0	0	0	0
ENSG00000215430	0	0	0	0
ENSG00000215431	0	0	0	0
ENSG00000215432	0	0	0	0

ENSG00000215440	2	0	0	0
ENSG00000215441	0	0	0	0
ENSG00000215444	0	0	0	0
ENSG00000215447	1	0	0	4
ENSG00000215448	0	0	0	0
ENSG00000215450	0	0	0	0
ENSG00000215452	0	0	1	4
ENSG00000215454	0	0	0	0
ENSG00000215455	0	0	0	0
ENSG00000215456	0	0	0	0
ENSG00000215457	0	0	0	0
ENSG00000215458	1	0	0	0
ENSG00000215462	0	0	0	0
ENSG00000215464	0	0	0	0
ENSG00000215467	0	0	0	0
ENSG00000215472	0	0	0	0
ENSG00000215474	0	0	0	0
ENSG00000215475	0	0	0	0
ENSG00000215477	0	0	0	0
ENSG00000215478	0	0	0	0
ENSG00000215480	3	4	2	0
ENSG00000215481	0	0	0	0
ENSG00000215482	0	0	0	0
ENSG00000215483	26	284	241	199
ENSG00000215486	0	0	0	0
ENSG00000215492	0	9	1	9
ENSG00000215493	0	0	0	0
ENSG00000215498	0	0	0	0
ENSG00000215504	3	0	0	3
ENSG00000215506	0	0	0	0
ENSG00000215507	0	0	0	0
ENSG00000215512	0	0	0	0
ENSG00000215513	0	0	0	0
ENSG00000215515	0	0	0	0
ENSG00000215520	0	0	0	0
ENSG00000215522	0	0	0	0
ENSG00000215523	0	0	0	0
ENSG00000215527	1	0	0	0
ENSG00000215529	0	2	7	3
ENSG00000215533	0	0	1	0
ENSG00000215537	0	0	0	0
ENSG00000215540	0	0	0	0
ENSG00000215544	0	0	0	0
ENSG00000215545	0	0	0	0
ENSG00000215546	0	0	0	0
ENSG00000215547	0	0	0	0

ENSG00000215548	0	7	13	51
ENSG00000215549	0	0	0	0
ENSG00000215553	0	0	0	0
ENSG00000215559	1	257	258	250
ENSG00000215560	0	0	0	0
ENSG00000215562	0	0	0	0
ENSG00000215565	1	0	0	0
ENSG00000215567	0	0	0	0
ENSG00000215568	0	0	0	0
ENSG00000215571	0	0	0	0
ENSG00000215572	0	0	0	0
ENSG00000215580	0	0	0	1
ENSG00000215583	0	0	0	0
ENSG00000215586	0	0	0	0
ENSG00000215589	0	0	0	0
ENSG00000215595	0	0	0	0
ENSG00000215601	0	0	0	0
ENSG00000215603	1	0	0	0
ENSG00000215604	0	0	0	0
ENSG00000215606	0	0	0	0
ENSG00000215608	1	0	0	0
ENSG00000215611	0	0	0	0
ENSG00000215612	0	0	0	0
ENSG00000215615	37	28	29	21
ENSG00000215616	0	0	0	0
ENSG00000215621	0	0	0	0
ENSG00000215630	4	30	26	43
ENSG00000215635	0	0	0	0
ENSG00000215641	0	0	0	0
ENSG00000215642	0	0	0	0
ENSG00000215644	0	0	0	0
ENSG00000215689	0	0	0	0
ENSG00000215692	0	0	0	0
ENSG00000215695	0	0	0	0
ENSG00000215699	2	0	1	11
ENSG00000215700	5	147	104	6
ENSG00000215704	0	0	0	0
ENSG00000215705	1	0	0	0
ENSG00000215712	9	148	106	87
ENSG00000215713	0	0	0	0
ENSG00000215717	202	1181	577	776
ENSG00000215719	0	0	0	0
ENSG00000215720	0	0	0	0
ENSG00000215734	0	0	0	0
ENSG00000215749	2	0	1	0
ENSG00000215750	28	8	0	8

ENSG00000215760	0	0	0	0
ENSG00000215764	2	0	0	0
ENSG00000215765	0	0	0	0
ENSG00000215769	2	0	0	0
ENSG00000215771	0	0	0	0
ENSG00000215781	6	1472	613	351
ENSG00000215784	2	0	1	0
ENSG00000215785	0	0	0	0
ENSG00000215788	0	0	0	0
ENSG00000215790	4	0	4	7
ENSG00000215791	0	0	0	0
ENSG00000215795	0	0	0	0
ENSG00000215796	0	0	0	0
ENSG00000215800	0	0	0	0
ENSG00000215802	0	0	0	0
ENSG00000215805	0	0	0	0
ENSG00000215807	0	0	0	0
ENSG00000215808	0	0	0	0
ENSG00000215811	1	0	0	0
ENSG00000215812	0	0	0	0
ENSG00000215817	0	6	40	31
ENSG00000215819	0	0	0	0
ENSG00000215833	0	0	0	0
ENSG00000215834	0	0	0	5
ENSG00000215835	0	0	0	0
ENSG00000215837	0	0	0	0
ENSG00000215838	0	0	0	0
ENSG00000215840	0	0	0	0
ENSG00000215841	0	0	0	0
ENSG00000215845	56	0	9	23
ENSG00000215846	0	0	0	0
ENSG00000215853	0	0	0	0
ENSG00000215859	0	0	0	0
ENSG00000215860	0	0	0	0
ENSG00000215861	0	0	0	2
ENSG00000215863	0	3	0	0
ENSG00000215864	0	0	0	2
ENSG00000215866	7	0	0	0
ENSG00000215867	0	0	0	0
ENSG00000215869	0	0	0	0
ENSG00000215871	0	0	0	0
ENSG00000215873	0	0	0	0
ENSG00000215874	0	0	0	0
ENSG00000215875	0	0	0	0
ENSG00000215878	0	0	0	0
ENSG00000215881	0	0	0	0

ENSG00000215883	1	12	3	0
ENSG00000215887	0	0	0	0
ENSG00000215889	0	0	0	0
ENSG00000215893	0	0	0	0
ENSG00000215895	0	0	0	0
ENSG00000215897	0	0	0	0
ENSG00000215899	0	0	0	0
ENSG00000215900	0	0	0	0
ENSG00000215902	0	0	0	0
ENSG00000215905	0	0	0	0
ENSG00000215906	0	0	0	0
ENSG00000215908	3	0	2	2
ENSG00000215909	0	0	0	0
ENSG00000215910	0	23	2	0
ENSG00000215912	0	0	0	0
ENSG00000215914	0	0	0	0
ENSG00000215915	1	16	0	0
ENSG00000215921	0	0	0	0
ENSG00000215922	0	0	0	0
ENSG00000215923	0	0	0	0
ENSG00000215924	0	0	0	0
ENSG00000215925	0	0	0	0
ENSG00000215930	0	0	0	0
ENSG00000215933	0	0	0	0
ENSG00000215934	0	0	0	0
ENSG00000215938	0	0	0	0
ENSG00000215939	0	0	0	0
ENSG00000215941	0	0	0	0
ENSG00000215942	0	0	0	0
ENSG00000215943	0	0	0	0
ENSG00000215944	0	0	0	0
ENSG00000215945	0	0	0	0
ENSG00000215946	0	0	0	0
ENSG00000215948	0	0	0	0
ENSG00000215952	0	0	0	0
ENSG00000215953	0	0	0	0
ENSG00000215954	0	0	0	0
ENSG00000215957	0	0	0	0
ENSG00000215958	0	0	0	0
ENSG00000215960	0	0	0	0
ENSG00000215961	0	0	0	0
ENSG00000215964	0	0	0	0
ENSG00000215966	0	0	0	0
ENSG00000215968	0	0	0	0
ENSG00000215972	0	0	0	0
ENSG00000215973	0	0	0	0

ENSG00000215976	0	0	0	0
ENSG00000215977	0	0	0	0
ENSG00000215979	0	0	0	0
ENSG00000215983	0	0	0	0
ENSG00000215984	0	0	0	0
ENSG00000215991	0	0	0	0
ENSG00000215993	0	0	0	0
ENSG00000215996	0	0	0	0
ENSG00000215998	0	0	0	0
ENSG00000215999	0	0	0	0
ENSG00000216001	0	0	0	0
ENSG00000216002	0	0	0	0
ENSG00000216004	0	0	0	0
ENSG00000216005	0	0	0	0
ENSG00000216009	0	0	0	0
ENSG00000216011	0	0	0	0
ENSG00000216014	0	0	0	0
ENSG00000216015	0	0	0	0
ENSG00000216017	0	0	0	0
ENSG00000216020	0	0	0	0
ENSG00000216022	0	0	0	0
ENSG00000216027	0	0	0	0
ENSG00000216031	0	0	0	0
ENSG00000216032	0	0	0	0
ENSG00000216035	0	0	0	0
ENSG00000216036	0	0	0	0
ENSG00000216037	0	0	0	0
ENSG00000216042	0	0	0	0
ENSG00000216045	0	0	0	0
ENSG00000216047	0	0	0	0
ENSG00000216054	0	0	0	0
ENSG00000216055	0	0	0	0
ENSG00000216056	0	0	0	0
ENSG00000216058	0	0	0	0
ENSG00000216060	0	0	0	0
ENSG00000216064	0	0	0	0
ENSG00000216067	0	0	0	0
ENSG00000216069	0	0	0	0
ENSG00000216070	0	0	0	0
ENSG00000216072	0	0	0	0
ENSG00000216073	0	0	0	0
ENSG00000216075	0	0	0	0
ENSG00000216076	0	0	0	0
ENSG00000216077	0	0	0	0
ENSG00000216081	0	0	0	0
ENSG00000216083	0	0	0	0

ENSG00000216084	0	0	0	0
ENSG00000216085	0	0	0	0
ENSG00000216088	0	0	0	0
ENSG00000216089	0	0	0	0
ENSG00000216090	0	0	0	0
ENSG00000216093	0	0	0	0
ENSG00000216095	0	0	0	0
ENSG00000216097	0	0	0	0
ENSG00000216098	0	0	0	0
ENSG00000216099	0	0	0	0
ENSG00000216100	0	0	0	0
ENSG00000216101	0	0	0	0
ENSG00000216102	0	0	0	0
ENSG00000216105	0	0	0	0
ENSG00000216109	0	0	0	0
ENSG00000216112	0	0	0	0
ENSG00000216113	0	0	0	0
ENSG00000216114	0	0	0	0
ENSG00000216115	0	0	0	0
ENSG00000216116	0	0	0	0
ENSG00000216123	0	0	0	0
ENSG00000216125	0	0	0	0
ENSG00000216127	0	0	0	0
ENSG00000216131	0	0	0	0
ENSG00000216133	0	0	0	0
ENSG00000216135	0	0	0	0
ENSG00000216136	0	0	0	0
ENSG00000216141	0	0	0	0
ENSG00000216154	0	0	0	0
ENSG00000216157	0	0	0	0
ENSG00000216162	0	0	0	0
ENSG00000216166	0	0	0	0
ENSG00000216168	0	0	0	0
ENSG00000216169	0	0	0	0
ENSG00000216171	0	0	0	0
ENSG00000216173	0	0	0	0
ENSG00000216179	0	0	0	0
ENSG00000216181	0	0	0	0
ENSG00000216182	0	0	0	0
ENSG00000216184	0	0	0	0
ENSG00000216191	0	0	0	0
ENSG00000216192	0	0	0	0
ENSG00000216193	0	0	0	0
ENSG00000216194	0	0	0	0
ENSG00000216195	0	0	0	0
ENSG00000216197	0	0	0	0

ENSG00000216204	0	0	0	0
ENSG00000216205	0	0	0	0
ENSG00000216206	0	0	0	0
ENSG00000216265	0	0	0	0
ENSG00000216285	0	0	3	0
ENSG00000216306	0	0	0	0
ENSG00000216307	0	0	0	0
ENSG00000216316	0	0	1	5
ENSG00000216324	0	0	0	0
ENSG00000216331	22	16	19	1
ENSG00000216347	0	0	0	0
ENSG00000216352	0	0	0	0
ENSG00000216359	0	0	0	0
ENSG00000216360	0	0	0	0
ENSG00000216364	0	0	0	0
ENSG00000216365	0	0	0	0
ENSG00000216368	0	0	0	0
ENSG00000216378	0	0	0	0
ENSG00000216412	0	0	0	0
ENSG00000216425	0	0	0	0
ENSG00000216436	1	0	0	0
ENSG00000216439	0	0	0	0
ENSG00000216444	0	0	0	0
ENSG00000216471	0	0	0	0
ENSG00000216475	0	0	0	0
ENSG00000216480	0	0	0	0
ENSG00000216490	0	0	0	0
ENSG00000216516	0	0	0	0
ENSG00000216518	0	0	0	0
ENSG00000216519	0	0	0	0
ENSG00000216522	0	0	0	0
ENSG00000216523	0	0	0	0
ENSG00000216548	0	0	0	0
ENSG00000216560	0	0	0	0
ENSG00000216588	0	0	0	0
ENSG00000216613	0	0	0	0
ENSG00000216616	0	0	0	0
ENSG00000216621	0	0	0	0
ENSG00000216624	0	0	0	0
ENSG00000216629	0	0	0	0
ENSG00000216636	0	0	0	0
ENSG00000216639	0	0	0	0
ENSG00000216642	0	0	0	0
ENSG00000216649	0	0	0	0
ENSG00000216657	0	0	0	0
ENSG00000216663	0	0	0	0

ENSG00000216671	0	0	0	0
ENSG00000216676	0	0	0	0
ENSG00000216687	0	0	0	0
ENSG00000216708	0	0	0	0
ENSG00000216710	0	0	0	0
ENSG00000216713	0	0	0	0
ENSG00000216718	0	0	0	0
ENSG00000216721	0	0	0	0
ENSG00000216723	0	0	0	0
ENSG00000216740	0	0	0	0
ENSG00000216753	0	0	0	0
ENSG00000216754	0	0	0	0
ENSG00000216762	0	0	0	0
ENSG00000216775	2	0	4	8
ENSG00000216777	0	0	0	0
ENSG00000216781	0	0	0	0
ENSG00000216802	0	0	0	0
ENSG00000216809	0	0	0	0
ENSG00000216811	0	0	0	0
ENSG00000216813	0	0	0	0
ENSG00000216817	0	0	0	0
ENSG00000216819	0	0	0	0
ENSG00000216824	0	0	0	0
ENSG00000216829	0	0	0	0
ENSG00000216835	0	0	0	0
ENSG00000216844	0	0	0	0
ENSG00000216853	0	0	0	0
ENSG00000216854	0	0	0	0
ENSG00000216859	0	0	0	2
ENSG00000216863	161	821	1173	864
ENSG00000216866	0	0	0	0
ENSG00000216867	0	0	0	0
ENSG00000216895	89	265	172	188
ENSG00000216901	0	0	0	0
ENSG00000216902	0	0	0	0
ENSG00000216904	0	0	0	0
ENSG00000216906	0	0	0	0
ENSG00000216913	0	0	0	0
ENSG00000216915	0	0	0	0
ENSG00000216917	0	0	0	0
ENSG00000216921	0	0	0	0
ENSG00000216937	41	4567	4177	6340
ENSG00000216938	0	0	0	0
ENSG00000216966	0	0	0	0
ENSG00000216977	0	0	0	0
ENSG00000216990	0	0	0	0

ENSG00000216998	0	0	0	0
ENSG00000217004	0	0	0	0
ENSG00000217026	0	0	0	0
ENSG00000217027	994	206	186	79
ENSG00000217030	0	0	0	0
ENSG00000217041	0	0	0	0
ENSG00000217044	0	0	0	0
ENSG00000217060	0	0	0	0
ENSG00000217067	0	0	0	0
ENSG00000217075	0	0	0	1
ENSG00000217078	0	0	0	0
ENSG00000217083	0	0	0	0
ENSG00000217085	0	0	0	0
ENSG00000217089	0	0	0	0
ENSG00000217094	1	1	0	0
ENSG00000217120	0	0	1	0
ENSG00000217128	9	7438	4884	8113
ENSG00000217130	0	0	0	0
ENSG00000217135	0	0	0	0
ENSG00000217139	0	0	0	0
ENSG00000217159	0	0	0	0
ENSG00000217160	0	0	0	0
ENSG00000217165	1	0	1	1
ENSG00000217169	0	0	0	0
ENSG00000217178	0	0	0	0
ENSG00000217179	0	0	0	0
ENSG00000217181	0	0	0	0
ENSG00000217195	0	0	0	0
ENSG00000217227	0	0	0	0
ENSG00000217228	14	47	11	61
ENSG00000217231	0	0	0	0
ENSG00000217236	0	0	0	0
ENSG00000217239	0	0	0	0
ENSG00000217241	0	3	3	1
ENSG00000217258	48	42	30	74
ENSG00000217261	0	0	0	0
ENSG00000217268	0	0	0	0
ENSG00000217272	0	0	0	0
ENSG00000217275	214	280	485	271
ENSG00000217281	0	0	0	0
ENSG00000217289	0	0	0	0
ENSG00000217314	0	0	0	0
ENSG00000217315	0	0	0	0
ENSG00000217325	0	0	0	0
ENSG00000217327	0	0	0	0
ENSG00000217330	0	0	0	0

ENSG00000217331	0	0	0	0
ENSG00000217334	0	0	0	0
ENSG00000217372	0	0	0	0
ENSG00000217377	0	0	0	0
ENSG00000217379	0	0	0	0
ENSG00000217385	0	0	0	0
ENSG00000217404	0	0	0	0
ENSG00000217408	0	0	0	0
ENSG00000217414	0	0	0	0
ENSG00000217416	0	0	0	0
ENSG00000217442	0	0	0	0
ENSG00000217447	0	0	0	0
ENSG00000217455	0	0	0	4
ENSG00000217477	0	0	0	0
ENSG00000217482	0	0	0	0
ENSG00000217483	0	0	0	0
ENSG00000217488	0	0	0	0
ENSG00000217495	0	0	0	0
ENSG00000217512	0	0	0	0
ENSG00000217514	0	0	0	0
ENSG00000217527	0	0	0	0
ENSG00000217539	0	0	0	0
ENSG00000217555	194	13	15	9
ENSG00000217557	0	0	0	0
ENSG00000217566	5	0	0	0
ENSG00000217576	1	0	14	3
ENSG00000217585	0	0	0	0
ENSG00000217612	0	0	0	2
ENSG00000217624	0	22	49	39
ENSG00000217631	0	0	0	0
ENSG00000217643	0	0	0	0
ENSG00000217644	2	0	0	0
ENSG00000217646	0	10	3	6
ENSG00000217648	1	14	17	18
ENSG00000217653	0	0	0	0
ENSG00000217680	0	0	0	0
ENSG00000217684	0	0	0	1
ENSG00000217686	0	0	0	0
ENSG00000217702	0	0	0	0
ENSG00000217707	0	0	0	0
ENSG00000217716	0	0	0	0
ENSG00000217718	0	0	0	0
ENSG00000217733	0	0	0	0
ENSG00000217746	0	0	0	0
ENSG00000217767	0	0	0	0
ENSG00000217769	0	1	3	0

ENSG00000217770	0	0	0	0
ENSG00000217776	0	0	0	0
ENSG00000217783	0	0	0	0
ENSG00000217786	0	0	0	0
ENSG00000217791	0	0	0	0
ENSG00000217792	0	0	0	0
ENSG00000217801	0	0	0	0
ENSG00000217805	0	0	0	0
ENSG00000217809	0	0	0	0
ENSG00000217811	0	0	0	0
ENSG00000217824	0	0	2	0
ENSG00000217825	0	0	0	0
ENSG00000217835	0	0	0	0
ENSG00000217862	4	0	10	3
ENSG00000217874	0	0	0	0
ENSG00000217878	0	0	0	0
ENSG00000217889	0	0	0	0
ENSG00000217896	0	0	0	0
ENSG00000217897	0	0	0	0
ENSG00000217929	0	0	0	0
ENSG00000217930	0	0	0	0
ENSG00000217950	0	0	0	0
ENSG00000218014	0	0	0	0
ENSG00000218016	0	0	0	0
ENSG00000218018	83	82	108	97
ENSG00000218020	0	0	0	0
ENSG00000218027	0	0	0	0
ENSG00000218029	0	0	0	0
ENSG00000218048	0	0	0	0
ENSG00000218049	0	0	0	0
ENSG00000218052	0	0	0	0
ENSG00000218069	0	0	0	0
ENSG00000218073	0	0	0	0
ENSG00000218089	0	0	0	0
ENSG00000218107	0	0	0	0
ENSG00000218109	0	0	0	0
ENSG00000218125	0	0	0	0
ENSG00000218143	0	0	0	0
ENSG00000218153	2	23	2	0
ENSG00000218173	0	0	0	0
ENSG00000218175	1	0	0	0
ENSG00000218180	0	0	0	0
ENSG00000218186	0	0	0	0
ENSG00000218187	0	0	0	0
ENSG00000218189	0	0	0	0
ENSG00000218194	0	0	0	0

ENSG00000218198	0	0	0	0
ENSG00000218208	2	0	0	0
ENSG00000218213	0	0	0	0
ENSG00000218226	0	0	0	0
ENSG00000218227	0	0	0	0
ENSG00000218233	0	0	0	0
ENSG00000218261	0	0	0	0
ENSG00000218265	0	0	0	0
ENSG00000218274	0	0	0	0
ENSG00000218281	0	0	0	0
ENSG00000218283	0	0	5	4
ENSG00000218297	0	0	0	0
ENSG00000218300	0	0	0	0
ENSG00000218305	1	3	4	3
ENSG00000218313	0	0	0	0
ENSG00000218336	1	0	0	0
ENSG00000218337	0	0	0	0
ENSG00000218346	0	0	0	0
ENSG00000218347	0	0	0	0
ENSG00000218350	0	0	0	0
ENSG00000218351	0	0	0	0
ENSG00000218357	0	0	0	0
ENSG00000218358	0	0	0	0
ENSG00000218359	0	0	0	0
ENSG00000218363	0	0	0	0
ENSG00000218410	0	0	0	0
ENSG00000218416	0	0	0	0
ENSG00000218418	0	39	51	17
ENSG00000218422	2	77	31	72
ENSG00000218424	0	0	0	0
ENSG00000218426	1	0	0	0
ENSG00000218428	0	0	0	0
ENSG00000218454	0	0	0	0
ENSG00000218459	0	0	2	0
ENSG00000218472	0	0	0	0
ENSG00000218475	0	0	0	0
ENSG00000218476	0	0	0	0
ENSG00000218483	0	0	0	0
ENSG00000218490	0	0	0	0
ENSG00000218497	0	0	0	0
ENSG00000218499	0	0	0	0
ENSG00000218502	0	0	0	0
ENSG00000218510	9	27	29	27
ENSG00000218512	0	0	0	0
ENSG00000218520	0	0	0	0
ENSG00000218521	0	0	0	0

ENSG00000218536	0	0	0	0
ENSG00000218537	0	0	2	0
ENSG00000218549	0	0	0	0
ENSG00000218561	0	0	0	0
ENSG00000218565	0	0	1	0
ENSG00000218574	0	0	0	0
ENSG00000218577	0	0	0	0
ENSG00000218582	0	0	0	0
ENSG00000218586	0	0	0	0
ENSG00000218596	0	0	2	0
ENSG00000218617	0	0	0	0
ENSG00000218631	0	0	0	2
ENSG00000218632	0	0	0	0
ENSG00000218643	0	0	0	0
ENSG00000218672	0	0	0	0
ENSG00000218676	0	0	1	2
ENSG00000218682	0	0	0	0
ENSG00000218689	0	0	0	0
ENSG00000218690	0	0	0	0
ENSG00000218698	0	0	0	0
ENSG00000218713	0	0	0	0
ENSG00000218716	0	0	0	0
ENSG00000218725	0	0	0	0
ENSG00000218728	0	0	0	0
ENSG00000218730	0	0	0	0
ENSG00000218732	0	0	0	0
ENSG00000218739	22	267	519	577
ENSG00000218748	0	0	0	0
ENSG00000218749	0	0	0	0
ENSG00000218754	0	0	0	0
ENSG00000218757	0	0	0	0
ENSG00000218766	0	0	0	0
ENSG00000218772	0	0	0	0
ENSG00000218776	0	0	0	0
ENSG00000218792	2	0	7	1
ENSG00000218793	0	0	0	0
ENSG00000218803	0	1	0	0
ENSG00000218806	0	0	0	0
ENSG00000218809	0	0	0	0
ENSG00000218813	0	0	0	0
ENSG00000218819	0	12	41	0
ENSG00000218823	0	0	3	0
ENSG00000218834	0	0	0	0
ENSG00000218839	0	0	0	0
ENSG00000218857	0	0	1	0
ENSG00000218868	0	0	0	0

ENSG00000218870	0	0	0	0
ENSG00000218872	0	0	0	0
ENSG00000218890	0	0	0	0
ENSG00000218891	0	0	0	0
ENSG00000218893	0	0	0	0
ENSG00000218896	0	0	0	0
ENSG00000218902	2	0	0	0
ENSG00000218965	0	0	0	0
ENSG00000218976	0	0	0	0
ENSG00000218980	5	0	0	0
ENSG00000218986	0	0	0	0
ENSG00000218991	0	0	0	0
ENSG00000218996	0	0	3	0
ENSG00000219016	0	0	0	0
ENSG00000219023	0	0	0	0
ENSG00000219027	0	0	0	0
ENSG00000219039	0	0	0	0
ENSG00000219041	0	0	0	0
ENSG00000219061	0	0	0	0
ENSG00000219073	0	0	0	0
ENSG00000219074	0	0	0	0
ENSG00000219085	0	0	0	4
ENSG00000219087	0	0	0	0
ENSG00000219088	0	0	0	0
ENSG00000219095	0	0	0	0
ENSG00000219102	0	0	0	0
ENSG00000219133	0	0	0	0
ENSG00000219135	0	0	0	0
ENSG00000219139	0	0	0	0
ENSG00000219146	0	0	0	0
ENSG00000219149	0	0	0	0
ENSG00000219150	0	0	0	0
ENSG00000219159	0	0	0	0
ENSG00000219163	0	0	0	2
ENSG00000219186	0	0	0	0
ENSG00000219188	0	0	0	0
ENSG00000219190	0	0	0	0
ENSG00000219200	2	3	0	1
ENSG00000219201	0	24	42	25
ENSG00000219222	1	0	0	0
ENSG00000219240	0	0	0	0
ENSG00000219249	0	0	0	0
ENSG00000219253	0	0	0	0
ENSG00000219257	0	0	0	0
ENSG00000219262	0	0	0	0
ENSG00000219273	0	0	0	0

ENSG00000219274	0	0	0	0
ENSG00000219280	0	0	0	0
ENSG00000219284	0	0	0	0
ENSG00000219294	0	0	0	0
ENSG00000219297	0	0	0	0
ENSG00000219298	0	0	0	0
ENSG00000219302	0	0	0	0
ENSG00000219314	0	0	0	0
ENSG00000219329	0	0	0	0
ENSG00000219332	0	0	0	0
ENSG00000219355	0	0	0	0
ENSG00000219361	0	0	0	0
ENSG00000219368	0	0	0	0
ENSG00000219374	0	0	0	0
ENSG00000219375	0	0	0	0
ENSG00000219384	0	0	0	0
ENSG00000219387	0	0	0	0
ENSG00000219391	0	0	0	0
ENSG00000219392	1	0	0	0
ENSG00000219395	0	0	0	0
ENSG00000219404	0	0	0	0
ENSG00000219409	0	0	0	0
ENSG00000219410	0	0	0	3
ENSG00000219430	0	0	0	0
ENSG00000219433	0	0	0	0
ENSG00000219435	0	0	0	0
ENSG00000219438	0	0	0	0
ENSG00000219445	0	0	0	0
ENSG00000219448	0	0	0	0
ENSG00000219451	0	0	0	0
ENSG00000219453	0	0	0	0
ENSG00000219463	0	0	0	1
ENSG00000219470	0	0	0	0
ENSG00000219481	11	18	58	52
ENSG00000219487	2	0	0	0
ENSG00000219491	0	0	0	0
ENSG00000219492	0	0	0	0
ENSG00000219500	0	0	0	0
ENSG00000219507	13	1917	1396	1579
ENSG00000219529	12	0	0	0
ENSG00000219532	0	0	0	0
ENSG00000219545	11	155	160	286
ENSG00000219547	0	0	0	0
ENSG00000219549	0	0	0	0
ENSG00000219553	0	0	0	0
ENSG00000219559	0	0	0	0

ENSG00000219565	0	0	0	0
ENSG00000219575	0	0	0	0
ENSG00000219582	0	0	0	0
ENSG00000219592	0	0	0	0
ENSG00000219604	0	0	0	0
ENSG00000219607	0	0	0	0
ENSG00000219608	0	0	0	0
ENSG00000219619	0	0	0	0
ENSG00000219622	0	0	0	0
ENSG00000219626	62	2663	2673	2060
ENSG00000219627	0	0	0	0
ENSG00000219642	0	0	0	0
ENSG00000219653	0	0	0	0
ENSG00000219665	102	130	138	166
ENSG00000219666	0	0	0	0
ENSG00000219669	0	0	0	0
ENSG00000219681	0	0	0	0
ENSG00000219682	0	0	2	3
ENSG00000219693	0	0	0	0
ENSG00000219699	0	0	0	0
ENSG00000219700	0	0	0	0
ENSG00000219702	0	0	0	0
ENSG00000219703	0	0	0	0
ENSG00000219712	0	0	0	0
ENSG00000219722	0	0	0	0
ENSG00000219736	0	0	0	0
ENSG00000219738	0	0	0	0
ENSG00000219747	0	0	0	0
ENSG00000219755	1	0	0	0
ENSG00000219757	0	0	0	0
ENSG00000219758	0	0	0	0
ENSG00000219770	0	0	0	0
ENSG00000219773	0	0	0	0
ENSG00000219776	0	0	0	0
ENSG00000219784	0	0	0	0
ENSG00000219790	0	0	0	0
ENSG00000219797	0	0	0	0
ENSG00000219806	0	0	0	0
ENSG00000219807	0	0	0	0
ENSG00000219814	0	0	0	0
ENSG00000219863	0	0	0	0
ENSG00000219867	0	0	0	0
ENSG00000219870	0	0	0	0
ENSG00000219881	0	0	0	0
ENSG00000219891	0	0	2	0
ENSG00000219902	0	0	0	0

ENSG00000219926	0	0	0	0
ENSG00000219928	0	0	0	0
ENSG00000219930	0	0	0	0
ENSG00000219932	0	0	0	0
ENSG00000219940	0	0	0	0
ENSG00000219941	0	0	0	0
ENSG00000219951	0	0	0	0
ENSG00000219986	0	0	0	0
ENSG00000219992	0	0	0	0
ENSG00000219993	0	0	0	0
ENSG00000220008	0	0	0	0
ENSG00000220023	35	13	103	70
ENSG00000220030	0	0	0	0
ENSG00000220032	0	0	0	0
ENSG00000220069	0	0	0	0
ENSG00000220076	0	0	0	0
ENSG00000220091	0	0	0	0
ENSG00000220105	0	0	0	0
ENSG00000220110	0	0	0	0
ENSG00000220113	0	0	0	0
ENSG00000220125	0	0	0	0
ENSG00000220130	0	0	0	0
ENSG00000220131	0	0	0	0
ENSG00000220132	0	0	0	0
ENSG00000220139	0	0	0	0
ENSG00000220154	0	0	0	0
ENSG00000220157	0	0	0	0
ENSG00000220161	11	0	4	0
ENSG00000220181	0	0	0	0
ENSG00000220184	0	0	0	0
ENSG00000220201	380	250	57	240
ENSG00000220204	0	0	0	0
ENSG00000220205	94	32	21	15
ENSG00000220212	0	0	0	0
ENSG00000220237	0	0	0	0
ENSG00000220240	0	0	0	0
ENSG00000220248	0	0	0	3
ENSG00000220256	0	0	0	0
ENSG00000220267	0	0	0	0
ENSG00000220291	0	0	0	0
ENSG00000220305	0	0	0	0
ENSG00000220311	0	0	0	0
ENSG00000220323	0	10	0	0
ENSG00000220326	0	0	0	0
ENSG00000220340	0	0	0	4
ENSG00000220343	0	0	0	0

ENSG00000220347	0	0	0	0
ENSG00000220349	0	0	0	0
ENSG00000220370	0	0	0	0
ENSG00000220377	0	0	0	0
ENSG00000220378	0	0	0	0
ENSG00000220379	0	0	0	0
ENSG00000220391	0	0	0	0
ENSG00000220392	0	0	0	0
ENSG00000220412	0	0	0	0
ENSG00000220418	0	0	0	0
ENSG00000220446	0	0	0	0
ENSG00000220447	0	0	0	0
ENSG00000220472	0	0	0	1
ENSG00000220483	0	0	0	0
ENSG00000220494	0	0	0	0
ENSG00000220505	0	0	0	0
ENSG00000220506	0	0	0	0
ENSG00000220514	0	0	0	0
ENSG00000220515	0	0	0	0
ENSG00000220517	26	35	118	23
ENSG00000220522	0	0	0	0
ENSG00000220537	0	0	0	0
ENSG00000220540	0	0	0	0
ENSG00000220541	0	0	0	0
ENSG00000220548	0	0	0	0
ENSG00000220550	0	0	0	0
ENSG00000220553	0	0	0	0
ENSG00000220556	0	0	0	0
ENSG00000220557	0	0	0	0
ENSG00000220563	0	0	0	0
ENSG00000220575	0	0	0	0
ENSG00000220581	0	0	0	0
ENSG00000220583	0	0	0	1
ENSG00000220585	0	0	0	0
ENSG00000220586	0	0	0	0
ENSG00000220598	0	0	0	0
ENSG00000220600	0	0	0	0
ENSG00000220614	0	0	0	0
ENSG00000220635	0	0	0	0
ENSG00000220643	0	0	0	0
ENSG00000220660	0	0	0	0
ENSG00000220666	0	0	0	0
ENSG00000220685	0	0	0	0
ENSG00000220693	1	0	0	0
ENSG00000220694	0	0	0	0
ENSG00000220695	0	0	0	0

ENSG00000220702	0	0	0	0
ENSG00000220721	0	0	0	0
ENSG00000220725	0	0	0	0
ENSG00000220730	0	0	0	0
ENSG00000220733	0	0	0	0
ENSG00000220734	0	0	0	0
ENSG00000220739	0	0	0	0
ENSG00000220744	0	0	0	0
ENSG00000220745	0	0	0	0
ENSG00000220748	0	0	0	0
ENSG00000220749	9	59	5	26
ENSG00000220758	0	0	0	0
ENSG00000220771	0	0	0	0
ENSG00000220773	0	0	0	0
ENSG00000220785	0	19	2	26
ENSG00000220793	1	0	0	0
ENSG00000220804	111	355	293	515
ENSG00000220823	0	0	0	0
ENSG00000220831	0	0	0	0
ENSG00000220842	7	0	1	4
ENSG00000220848	3	14	0	4
ENSG00000220867	0	0	0	0
ENSG00000220868	0	0	0	0
ENSG00000220871	0	0	0	0
ENSG00000220875	4	1	3	0
ENSG00000220884	0	0	0	0
ENSG00000220890	0	0	0	0
ENSG00000220891	0	0	0	0
ENSG00000220903	0	0	0	0
ENSG00000220908	0	0	0	0
ENSG00000220913	0	0	0	0
ENSG00000220918	0	0	0	0
ENSG00000220920	0	0	0	0
ENSG00000220924	0	0	0	0
ENSG00000220925	0	12	13	8
ENSG00000220937	0	0	1	0
ENSG00000220948	0	0	0	0
ENSG00000220949	0	0	0	0
ENSG00000220960	0	0	0	0
ENSG00000220978	0	0	0	0
ENSG00000220981	0	0	0	0
ENSG00000220982	0	0	0	0
ENSG00000220984	0	0	0	0
ENSG00000220986	0	0	0	0
ENSG00000220987	0	0	0	0
ENSG00000220988	0	0	0	0

ENSG00000220989	0	0	0	0
ENSG00000220990	0	0	0	0
ENSG00000220992	0	0	0	0
ENSG00000220993	0	0	0	0
ENSG00000220994	0	0	0	0
ENSG00000220996	0	0	0	0
ENSG00000220997	0	0	0	0
ENSG00000220999	0	0	0	0
ENSG00000221000	0	0	0	0
ENSG00000221002	0	0	0	0
ENSG00000221003	0	0	0	0
ENSG00000221005	0	0	0	0
ENSG00000221007	0	0	0	0
ENSG00000221008	0	0	0	0
ENSG00000221010	0	0	0	0
ENSG00000221011	0	0	0	0
ENSG00000221015	0	0	0	0
ENSG00000221016	0	0	0	0
ENSG00000221017	0	0	0	0
ENSG00000221018	0	0	0	0
ENSG00000221019	0	0	0	0
ENSG00000221020	0	0	0	0
ENSG00000221021	0	0	0	0
ENSG00000221023	0	0	0	0
ENSG00000221025	0	0	0	0
ENSG00000221026	0	0	0	0
ENSG00000221027	0	0	0	0
ENSG00000221028	0	0	0	0
ENSG00000221029	0	0	0	0
ENSG00000221030	0	0	0	0
ENSG00000221031	0	0	0	0
ENSG00000221032	0	0	0	0
ENSG00000221033	0	0	0	0
ENSG00000221035	0	0	0	0
ENSG00000221036	0	0	0	0
ENSG00000221037	0	0	0	0
ENSG00000221038	0	0	0	0
ENSG00000221039	0	0	0	0
ENSG00000221040	0	0	0	0
ENSG00000221041	0	0	0	0
ENSG00000221042	0	0	0	0
ENSG00000221043	0	0	0	0
ENSG00000221044	0	0	0	0
ENSG00000221046	0	0	0	0
ENSG00000221048	0	0	0	0
ENSG00000221050	0	0	0	0

ENSG00000221051	0	0	0	0
ENSG00000221052	0	0	0	0
ENSG00000221053	0	0	0	0
ENSG00000221055	0	0	0	0
ENSG00000221058	0	0	0	0
ENSG00000221059	0	0	0	0
ENSG00000221060	0	0	0	0
ENSG00000221062	0	0	0	0
ENSG00000221063	0	0	0	0
ENSG00000221065	0	0	0	0
ENSG00000221066	0	0	0	0
ENSG00000221067	0	0	0	0
ENSG00000221069	0	0	0	0
ENSG00000221070	0	0	0	0
ENSG00000221072	0	0	0	0
ENSG00000221073	0	0	0	0
ENSG00000221074	0	0	0	0
ENSG00000221075	0	0	0	0
ENSG00000221077	0	0	0	0
ENSG00000221079	0	0	0	0
ENSG00000221081	0	0	0	0
ENSG00000221082	0	0	0	0
ENSG00000221083	0	0	0	0
ENSG00000221084	0	0	0	0
ENSG00000221085	0	0	0	0
ENSG00000221087	0	0	0	0
ENSG00000221090	0	0	0	0
ENSG00000221091	0	0	0	0
ENSG00000221093	0	0	0	0
ENSG00000221094	0	0	0	0
ENSG00000221095	0	0	0	0
ENSG00000221096	0	0	0	0
ENSG00000221100	0	0	0	0
ENSG00000221101	0	0	0	0
ENSG00000221102	0	0	0	0
ENSG00000221104	0	0	0	0
ENSG00000221105	0	0	0	0
ENSG00000221108	0	0	0	0
ENSG00000221110	0	0	0	0
ENSG00000221111	0	0	0	0
ENSG00000221112	0	0	0	0
ENSG00000221113	0	0	0	0
ENSG00000221114	0	0	0	0
ENSG00000221115	0	0	0	0
ENSG00000221116	0	0	0	0
ENSG00000221118	0	0	0	0

ENSG00000221120	0	0	0	0
ENSG00000221121	0	0	0	0
ENSG00000221122	0	0	0	0
ENSG00000221123	0	0	0	0
ENSG00000221125	0	0	0	0
ENSG00000221126	0	0	0	0
ENSG00000221128	0	0	0	0
ENSG00000221130	0	0	0	0
ENSG00000221131	0	0	0	0
ENSG00000221132	0	0	0	0
ENSG00000221133	0	0	0	0
ENSG00000221134	0	0	0	0
ENSG00000221135	0	0	0	0
ENSG00000221136	0	0	0	0
ENSG00000221137	0	0	0	0
ENSG00000221139	0	0	0	0
ENSG00000221141	0	0	0	0
ENSG00000221142	0	0	0	0
ENSG00000221145	0	0	0	1
ENSG00000221147	0	0	0	0
ENSG00000221148	0	0	0	0
ENSG00000221156	0	0	0	0
ENSG00000221157	0	13	5	0
ENSG00000221159	0	0	0	0
ENSG00000221160	0	0	0	0
ENSG00000221163	0	0	0	0
ENSG00000221164	0	0	0	0
ENSG00000221165	0	0	0	0
ENSG00000221167	0	0	0	0
ENSG00000221169	0	0	0	0
ENSG00000221170	0	0	0	0
ENSG00000221172	0	0	0	0
ENSG00000221173	0	0	0	0
ENSG00000221174	0	0	0	0
ENSG00000221175	0	0	0	0
ENSG00000221176	0	0	0	0
ENSG00000221177	0	0	0	0
ENSG00000221178	0	0	0	0
ENSG00000221179	0	0	0	0
ENSG00000221180	0	0	0	0
ENSG00000221181	0	0	0	0
ENSG00000221182	0	0	0	0
ENSG00000221183	0	0	0	0
ENSG00000221184	0	0	0	0
ENSG00000221185	0	0	0	0
ENSG00000221186	0	0	0	0

ENSG00000221187	0	0	0	0
ENSG00000221189	0	0	0	0
ENSG00000221190	0	0	0	0
ENSG00000221191	0	0	0	0
ENSG00000221192	0	0	0	0
ENSG00000221195	0	0	0	2
ENSG00000221198	0	0	0	0
ENSG00000221199	0	0	0	0
ENSG00000221200	0	0	0	0
ENSG00000221201	0	0	0	0
ENSG00000221202	0	0	0	0
ENSG00000221203	0	0	0	0
ENSG00000221204	0	0	0	0
ENSG00000221206	0	0	0	0
ENSG00000221210	0	0	0	0
ENSG00000221211	0	0	0	0
ENSG00000221214	0	0	0	0
ENSG00000221216	0	0	0	0
ENSG00000221217	0	0	0	0
ENSG00000221218	0	0	0	0
ENSG00000221219	0	0	0	0
ENSG00000221220	0	0	0	0
ENSG00000221221	0	0	0	0
ENSG00000221222	0	0	0	0
ENSG00000221225	0	0	0	0
ENSG00000221226	0	0	0	0
ENSG00000221227	0	0	0	0
ENSG00000221230	0	0	0	0
ENSG00000221232	0	0	0	0
ENSG00000221233	0	0	0	0
ENSG00000221234	0	0	0	0
ENSG00000221235	0	0	0	0
ENSG00000221236	0	0	0	0
ENSG00000221237	0	0	0	0
ENSG00000221238	0	0	0	0
ENSG00000221240	0	0	0	0
ENSG00000221241	0	0	0	0
ENSG00000221244	0	0	0	0
ENSG00000221245	0	0	0	0
ENSG00000221246	0	0	0	0
ENSG00000221247	0	0	0	0
ENSG00000221249	0	0	0	0
ENSG00000221250	0	0	0	0
ENSG00000221251	0	0	0	0
ENSG00000221252	0	0	0	0
ENSG00000221254	0	0	0	0

ENSG00000221255	0	0	0	0
ENSG00000221257	0	0	0	0
ENSG00000221258	0	0	0	0
ENSG00000221259	0	0	0	0
ENSG00000221260	0	0	0	0
ENSG00000221261	0	0	0	0
ENSG00000221262	0	0	0	0
ENSG00000221263	0	0	0	0
ENSG00000221264	0	0	0	0
ENSG00000221265	0	0	0	0
ENSG00000221266	0	0	0	0
ENSG00000221267	0	0	0	0
ENSG00000221269	0	0	0	0
ENSG00000221270	0	0	0	0
ENSG00000221272	0	0	0	0
ENSG00000221273	0	0	0	0
ENSG00000221275	0	0	0	0
ENSG00000221278	0	0	0	0
ENSG00000221279	0	0	0	0
ENSG00000221280	0	0	0	0
ENSG00000221281	0	0	0	0
ENSG00000221286	0	0	0	0
ENSG00000221287	0	0	0	0
ENSG00000221288	0	0	0	0
ENSG00000221289	0	0	0	0
ENSG00000221290	0	0	0	0
ENSG00000221291	0	0	0	0
ENSG00000221292	0	0	0	0
ENSG00000221293	0	0	0	0
ENSG00000221294	0	0	0	0
ENSG00000221295	0	0	0	0
ENSG00000221296	0	0	0	2
ENSG00000221297	0	0	0	0
ENSG00000221299	0	0	0	0
ENSG00000221300	0	0	0	0
ENSG00000221301	0	0	0	0
ENSG00000221302	0	0	0	0
ENSG00000221303	0	0	0	0
ENSG00000221304	0	0	0	0
ENSG00000221305	0	0	0	0
ENSG00000221307	0	0	0	0
ENSG00000221309	0	0	0	0
ENSG00000221310	0	0	0	0
ENSG00000221311	0	0	0	0
ENSG00000221312	0	0	0	0
ENSG00000221313	0	0	0	0

ENSG00000221314	0	0	0	0
ENSG00000221315	0	0	0	0
ENSG00000221316	0	0	0	0
ENSG00000221317	0	0	0	0
ENSG00000221318	0	0	0	0
ENSG00000221319	0	0	0	0
ENSG00000221320	0	0	0	0
ENSG00000221321	0	0	0	0
ENSG00000221322	0	0	0	0
ENSG00000221323	0	0	0	0
ENSG00000221324	0	0	0	0
ENSG00000221325	0	0	0	0
ENSG00000221326	0	0	0	0
ENSG00000221328	0	0	0	0
ENSG00000221330	0	0	0	0
ENSG00000221331	0	0	0	0
ENSG00000221332	0	0	0	0
ENSG00000221333	0	0	0	0
ENSG00000221334	0	0	0	0
ENSG00000221335	0	0	0	0
ENSG00000221336	0	0	0	0
ENSG00000221337	0	0	0	0
ENSG00000221338	0	0	0	0
ENSG00000221340	0	0	0	0
ENSG00000221343	0	0	0	0
ENSG00000221344	0	0	0	0
ENSG00000221345	0	0	0	0
ENSG00000221346	0	0	0	0
ENSG00000221347	0	0	0	3
ENSG00000221348	0	0	0	0
ENSG00000221351	0	0	0	0
ENSG00000221352	0	0	0	0
ENSG00000221354	0	0	0	0
ENSG00000221355	0	0	0	0
ENSG00000221356	0	0	0	0
ENSG00000221357	0	0	0	0
ENSG00000221358	0	0	0	0
ENSG00000221359	0	0	0	0
ENSG00000221362	0	0	0	0
ENSG00000221363	0	0	0	0
ENSG00000221365	0	0	0	0
ENSG00000221366	0	0	0	0
ENSG00000221369	0	0	0	0
ENSG00000221371	0	0	0	0
ENSG00000221372	0	0	0	0
ENSG00000221373	0	0	0	0

ENSG00000221374	0	0	0	0
ENSG00000221375	0	0	0	0
ENSG00000221376	0	0	0	0
ENSG00000221377	0	0	0	0
ENSG00000221378	0	0	0	0
ENSG00000221379	0	0	0	0
ENSG00000221380	0	0	0	0
ENSG00000221381	0	0	0	0
ENSG00000221382	0	0	0	0
ENSG00000221383	0	0	0	0
ENSG00000221384	0	0	0	0
ENSG00000221385	0	0	0	0
ENSG00000221386	0	0	0	0
ENSG00000221387	0	0	0	0
ENSG00000221388	0	0	0	0
ENSG00000221389	0	0	0	0
ENSG00000221390	0	0	0	0
ENSG00000221391	0	0	0	0
ENSG00000221392	0	0	0	0
ENSG00000221393	0	0	0	0
ENSG00000221394	0	0	0	0
ENSG00000221395	0	0	0	0
ENSG00000221396	0	0	0	0
ENSG00000221397	0	0	0	0
ENSG00000221398	0	0	0	0
ENSG00000221399	0	0	0	0
ENSG00000221400	0	0	0	0
ENSG00000221401	0	0	0	0
ENSG00000221402	0	0	0	0
ENSG00000221403	0	0	0	0
ENSG00000221405	0	0	0	0
ENSG00000221406	0	0	0	0
ENSG00000221407	0	0	0	0
ENSG00000221409	0	0	0	0
ENSG00000221410	0	0	0	0
ENSG00000221411	0	0	0	0
ENSG00000221412	0	0	0	0
ENSG00000221413	0	0	0	0
ENSG00000221415	0	0	0	0
ENSG00000221416	0	0	0	0
ENSG00000221417	0	0	0	0
ENSG00000221418	0	0	0	0
ENSG00000221419	0	0	0	0
ENSG00000221420	0	0	0	0
ENSG00000221421	0	0	0	0
ENSG00000221423	0	0	0	0

ENSG00000221424	0	0	0	0
ENSG00000221425	0	0	0	0
ENSG00000221427	0	0	0	0
ENSG00000221428	0	0	0	0
ENSG00000221429	0	0	0	0
ENSG00000221430	0	0	0	0
ENSG00000221431	0	0	0	0
ENSG00000221432	0	0	0	0
ENSG00000221434	0	0	0	0
ENSG00000221436	0	0	0	0
ENSG00000221437	0	0	0	0
ENSG00000221438	0	0	0	0
ENSG00000221439	0	0	0	0
ENSG00000221440	0	0	0	0
ENSG00000221442	0	0	0	0
ENSG00000221443	0	0	0	0
ENSG00000221444	0	0	0	0
ENSG00000221445	0	0	0	0
ENSG00000221446	0	0	0	0
ENSG00000221447	0	0	0	0
ENSG00000221448	0	0	0	0
ENSG00000221449	0	0	0	0
ENSG00000221450	0	0	0	0
ENSG00000221451	0	0	0	0
ENSG00000221454	0	0	0	0
ENSG00000221455	0	0	0	0
ENSG00000221456	0	0	0	0
ENSG00000221457	0	0	0	0
ENSG00000221458	0	0	0	0
ENSG00000221459	0	0	0	0
ENSG00000221461	0	0	0	0
ENSG00000221462	0	0	0	0
ENSG00000221463	0	0	0	0
ENSG00000221464	0	0	0	0
ENSG00000221465	0	0	0	0
ENSG00000221466	0	0	0	0
ENSG00000221467	0	0	0	0
ENSG00000221468	0	0	0	0
ENSG00000221469	0	0	0	0
ENSG00000221470	0	0	0	0
ENSG00000221471	0	0	0	0
ENSG00000221473	0	0	0	0
ENSG00000221474	0	0	0	0
ENSG00000221475	0	0	0	0
ENSG00000221476	0	0	0	0
ENSG00000221477	0	0	0	0

ENSG00000221478	0	0	0	0
ENSG00000221479	0	0	0	0
ENSG00000221484	0	0	0	0
ENSG00000221485	0	0	0	0
ENSG00000221486	0	0	0	0
ENSG00000221487	0	0	0	0
ENSG00000221488	0	0	0	0
ENSG00000221489	0	0	0	0
ENSG00000221491	0	0	0	0
ENSG00000221492	0	0	0	0
ENSG00000221493	0	0	0	0
ENSG00000221494	0	0	0	0
ENSG00000221495	0	0	0	0
ENSG00000221496	0	0	0	0
ENSG00000221497	0	0	0	0
ENSG00000221498	1	0	1	0
ENSG00000221499	0	0	0	0
ENSG00000221500	0	0	0	0
ENSG00000221502	0	0	0	0
ENSG00000221504	0	0	0	0
ENSG00000221505	0	0	0	0
ENSG00000221506	0	0	0	0
ENSG00000221507	0	0	0	0
ENSG00000221510	0	0	0	0
ENSG00000221511	0	0	0	0
ENSG00000221514	0	0	0	0
ENSG00000221515	0	0	0	0
ENSG00000221516	0	0	0	0
ENSG00000221517	0	0	0	0
ENSG00000221518	0	0	0	0
ENSG00000221519	0	0	0	0
ENSG00000221520	0	0	0	0
ENSG00000221521	0	0	0	0
ENSG00000221523	0	0	0	0
ENSG00000221524	0	0	0	0
ENSG00000221525	0	0	0	0
ENSG00000221526	0	0	0	0
ENSG00000221527	0	0	0	0
ENSG00000221528	0	0	0	0
ENSG00000221529	0	0	0	0
ENSG00000221530	0	0	0	0
ENSG00000221531	0	0	0	0
ENSG00000221532	0	0	0	0
ENSG00000221533	0	0	0	0
ENSG00000221534	0	0	0	0
ENSG00000221535	0	0	0	0

ENSG00000221536	0	0	0	0
ENSG00000221537	0	0	0	0
ENSG00000221538	0	0	0	0
ENSG00000221539	0	0	0	0
ENSG00000221540	0	0	0	0
ENSG00000221541	0	0	0	0
ENSG00000221542	0	0	0	0
ENSG00000221544	0	0	0	0
ENSG00000221545	0	0	0	0
ENSG00000221547	0	0	0	0
ENSG00000221548	0	0	0	0
ENSG00000221550	0	0	0	0
ENSG00000221551	0	0	0	0
ENSG00000221552	0	0	0	0
ENSG00000221553	0	0	0	0
ENSG00000221555	0	0	0	0
ENSG00000221556	0	0	0	0
ENSG00000221558	0	0	0	0
ENSG00000221559	0	0	0	0
ENSG00000221560	0	0	0	0
ENSG00000221561	0	0	0	0
ENSG00000221562	0	0	0	0
ENSG00000221563	0	0	0	0
ENSG00000221564	0	0	0	0
ENSG00000221565	0	0	0	0
ENSG00000221566	0	0	0	0
ENSG00000221567	0	0	0	0
ENSG00000221568	0	0	0	0
ENSG00000221570	0	0	0	0
ENSG00000221571	0	0	0	1
ENSG00000221573	0	0	0	0
ENSG00000221574	0	0	0	0
ENSG00000221576	0	0	0	0
ENSG00000221578	0	0	0	0
ENSG00000221579	0	0	0	0
ENSG00000221580	0	0	0	0
ENSG00000221581	0	0	0	0
ENSG00000221583	0	0	0	0
ENSG00000221584	0	0	0	0
ENSG00000221585	0	0	0	0
ENSG00000221586	0	0	0	0
ENSG00000221589	0	0	0	0
ENSG00000221590	0	0	0	0
ENSG00000221591	0	0	0	0
ENSG00000221593	0	0	0	0
ENSG00000221594	0	0	0	0

ENSG00000221595	0	0	0	1
ENSG00000221597	0	0	0	0
ENSG00000221598	0	0	0	0
ENSG00000221599	0	0	0	0
ENSG00000221601	0	0	0	0
ENSG00000221602	0	0	0	0
ENSG00000221603	0	0	0	0
ENSG00000221604	0	0	0	0
ENSG00000221606	0	0	0	0
ENSG00000221607	0	0	0	0
ENSG00000221608	0	0	0	0
ENSG00000221610	0	0	0	0
ENSG00000221611	0	0	0	0
ENSG00000221612	0	0	0	0
ENSG00000221613	0	0	0	0
ENSG00000221614	0	0	0	0
ENSG00000221615	0	0	0	0
ENSG00000221616	0	0	0	0
ENSG00000221617	0	0	0	0
ENSG00000221618	0	0	0	0
ENSG00000221619	0	0	0	0
ENSG00000221621	0	0	0	0
ENSG00000221623	0	0	0	0
ENSG00000221625	3	0	0	2
ENSG00000221628	0	0	0	0
ENSG00000221629	0	0	0	0
ENSG00000221630	0	0	0	0
ENSG00000221631	0	0	0	0
ENSG00000221633	0	0	0	0
ENSG00000221634	0	0	0	0
ENSG00000221635	0	0	0	0
ENSG00000221637	0	0	0	0
ENSG00000221638	0	0	0	0
ENSG00000221639	0	0	0	0
ENSG00000221641	0	0	0	0
ENSG00000221643	0	0	0	0
ENSG00000221644	0	0	0	0
ENSG00000221645	0	0	0	0
ENSG00000221649	0	0	0	0
ENSG00000221650	0	0	0	0
ENSG00000221653	0	0	0	0
ENSG00000221654	0	0	0	0
ENSG00000221655	0	0	0	0
ENSG00000221656	0	0	0	0
ENSG00000221657	0	0	0	0
ENSG00000221659	0	0	0	0

ENSG00000221660	0	0	0	0
ENSG00000221661	0	0	0	0
ENSG00000221662	0	0	0	0
ENSG00000221664	0	0	0	0
ENSG00000221665	0	0	0	0
ENSG00000221666	0	0	0	0
ENSG00000221667	0	0	0	0
ENSG00000221669	0	0	0	0
ENSG00000221670	0	0	0	0
ENSG00000221672	0	0	0	0
ENSG00000221673	0	0	0	0
ENSG00000221676	0	35	9	13
ENSG00000221677	0	0	0	0
ENSG00000221679	0	0	0	0
ENSG00000221680	0	0	0	0
ENSG00000221681	0	0	0	0
ENSG00000221683	0	0	0	0
ENSG00000221684	0	0	0	0
ENSG00000221685	0	0	0	0
ENSG00000221686	0	0	0	0
ENSG00000221689	0	0	0	0
ENSG00000221690	0	0	0	0
ENSG00000221691	0	0	0	0
ENSG00000221692	0	0	0	0
ENSG00000221693	0	0	0	0
ENSG00000221695	0	0	0	0
ENSG00000221696	0	0	0	0
ENSG00000221697	0	0	0	0
ENSG00000221698	0	0	0	0
ENSG00000221699	0	0	0	0
ENSG00000221701	0	0	0	0
ENSG00000221702	0	0	0	0
ENSG00000221703	0	0	0	0
ENSG00000221704	0	0	0	0
ENSG00000221705	0	0	0	0
ENSG00000221706	0	0	0	0
ENSG00000221707	0	0	0	0
ENSG00000221708	0	0	0	0
ENSG00000221710	0	0	0	0
ENSG00000221711	0	0	0	0
ENSG00000221714	0	0	0	0
ENSG00000221716	0	0	0	0
ENSG00000221717	0	0	0	0
ENSG00000221718	0	0	0	0
ENSG00000221719	0	0	0	0
ENSG00000221720	0	0	0	0

ENSG00000221721	0	0	0	0
ENSG00000221723	0	0	0	0
ENSG00000221724	0	0	0	0
ENSG00000221725	0	0	0	0
ENSG00000221726	0	0	0	0
ENSG00000221729	0	0	0	0
ENSG00000221730	0	0	0	0
ENSG00000221731	0	0	0	0
ENSG00000221732	0	0	0	0
ENSG00000221733	0	0	0	0
ENSG00000221734	0	0	0	0
ENSG00000221736	0	0	0	0
ENSG00000221737	0	0	0	0
ENSG00000221739	0	0	0	0
ENSG00000221740	0	0	0	0
ENSG00000221741	0	0	0	0
ENSG00000221743	0	0	0	0
ENSG00000221744	0	0	0	0
ENSG00000221745	0	0	0	0
ENSG00000221746	0	0	0	0
ENSG00000221747	0	0	0	0
ENSG00000221748	0	0	0	0
ENSG00000221749	0	0	0	0
ENSG00000221750	0	0	0	0
ENSG00000221751	0	0	0	0
ENSG00000221753	0	0	0	0
ENSG00000221754	0	0	0	0
ENSG00000221755	0	0	0	0
ENSG00000221756	0	0	0	0
ENSG00000221760	0	0	0	0
ENSG00000221762	0	0	0	0
ENSG00000221763	0	0	0	0
ENSG00000221764	0	0	0	0
ENSG00000221766	0	0	0	0
ENSG00000221767	0	0	0	0
ENSG00000221768	0	0	0	0
ENSG00000221770	0	0	0	0
ENSG00000221771	0	0	0	0
ENSG00000221772	0	0	0	0
ENSG00000221774	0	0	0	0
ENSG00000221777	0	0	0	0
ENSG00000221778	0	0	0	0
ENSG00000221779	0	0	0	0
ENSG00000221781	0	0	0	0
ENSG00000221782	0	0	0	0
ENSG00000221783	0	0	0	0

ENSG00000221785	0	0	0	0
ENSG00000221786	0	0	0	0
ENSG00000221788	0	0	0	0
ENSG00000221789	0	0	0	0
ENSG00000221790	0	0	0	0
ENSG00000221792	0	458	202	207
ENSG00000221793	0	0	0	0
ENSG00000221795	0	0	0	0
ENSG00000221796	0	0	0	0
ENSG00000221798	0	0	0	0
ENSG00000221799	0	0	0	0
ENSG00000221801	0	0	0	0
ENSG00000221802	0	0	0	0
ENSG00000221803	0	0	0	0
ENSG00000221804	0	0	0	0
ENSG00000221806	0	0	0	0
ENSG00000221807	0	0	0	0
ENSG00000221808	0	0	0	0
ENSG00000221809	0	0	0	0
ENSG00000221810	0	0	0	0
ENSG00000221813	0	0	0	0
ENSG00000221817	15	54	57	41
ENSG00000221818	0	0	0	0
ENSG00000221819	0	0	0	0
ENSG00000221821	10	1	0	0
ENSG00000221823	530	18262	17965	16249
ENSG00000221826	0	0	0	0
ENSG00000221829	3	0	0	0
ENSG00000221836	0	0	0	0
ENSG00000221837	0	0	0	0
ENSG00000221838	5	42	13	25
ENSG00000221840	0	0	0	0
ENSG00000221843	0	0	0	0
ENSG00000221844	0	0	0	0
ENSG00000221845	0	0	0	0
ENSG00000221849	0	0	0	0
ENSG00000221852	0	0	0	0
ENSG00000221855	0	0	0	0
ENSG00000221857	0	0	0	0
ENSG00000221858	0	0	0	0
ENSG00000221859	0	0	0	0
ENSG00000221864	0	0	0	0
ENSG00000221866	20	45	74	366
ENSG00000221867	0	0	0	0
ENSG00000221869	3	0	1	4
ENSG00000221870	0	0	0	0

ENSG00000221874	0	0	0	0
ENSG00000221878	0	0	0	0
ENSG00000221879	0	0	0	0
ENSG00000221880	0	0	0	0
ENSG00000221882	0	0	0	0
ENSG00000221883	5	0	0	0
ENSG00000221886	1	0	6	9
ENSG00000221887	1	0	0	0
ENSG00000221888	0	0	0	0
ENSG00000221890	0	0	0	0
ENSG00000221891	0	0	0	0
ENSG00000221900	0	0	0	0
ENSG00000221909	3	25	14	54
ENSG00000221910	0	0	0	0
ENSG00000221914	20	404	567	638
ENSG00000221916	0	0	0	0
ENSG00000221923	11	157	44	219
ENSG00000221926	0	2	1	1
ENSG00000221930	0	4	1	0
ENSG00000221931	0	0	0	0
ENSG00000221932	0	0	0	0
ENSG00000221933	0	0	0	0
ENSG00000221937	0	0	0	0
ENSG00000221938	0	0	0	0
ENSG00000221944	2	13	7	7
ENSG00000221946	2	0	0	0
ENSG00000221947	1	39	14	13
ENSG00000221949	2	0	2	3
ENSG00000221953	0	0	0	0
ENSG00000221954	0	0	0	0
ENSG00000221955	0	0	0	0
ENSG00000221957	2	0	0	0
ENSG00000221961	0	0	0	0
ENSG00000221962	0	0	0	0
ENSG00000221963	21	0	18	6
ENSG00000221968	0	0	1	0
ENSG00000221970	0	0	0	0
ENSG00000221971	0	0	0	0
ENSG00000221972	0	0	0	0
ENSG00000221974	0	0	0	0
ENSG00000221977	0	0	0	0
ENSG00000221978	12	8	17	25
ENSG00000221983	4857	2620	1476	4240
ENSG00000221986	10	0	3	13
ENSG00000221988	40	21	18	5
ENSG00000221989	0	0	0	0

ENSG00000221990	0	0	0	0
ENSG00000221994	2	20	36	43
ENSG00000221995	0	16	1	9
ENSG00000221996	0	0	0	0
ENSG00000222000	0	0	0	0
ENSG00000222001	0	0	0	0
ENSG00000222004	0	0	0	0
ENSG00000222005	0	0	0	0
ENSG00000222007	0	0	0	0
ENSG00000222009	11	4	11	14
ENSG00000222011	1	354	725	754
ENSG00000222012	0	0	0	0
ENSG00000222014	0	0	0	0
ENSG00000222017	0	0	0	0
ENSG00000222018	0	48	0	1
ENSG00000222019	0	16	0	0
ENSG00000222020	4	0	0	5
ENSG00000222022	12	137	110	172
ENSG00000222024	0	0	0	0
ENSG00000222028	0	0	0	0
ENSG00000222030	0	0	0	0
ENSG00000222031	0	0	0	0
ENSG00000222032	3	129	109	234
ENSG00000222033	0	0	0	0
ENSG00000222035	0	0	0	0
ENSG00000222036	0	0	0	0
ENSG00000222037	0	0	0	0
ENSG00000222038	0	0	0	0
ENSG00000222040	0	0	0	0
ENSG00000222041	5341	3760	3629	4790
ENSG00000222042	0	0	0	0
ENSG00000222043	12	13	7	16
ENSG00000222044	0	0	0	0
ENSG00000222046	0	0	1	0
ENSG00000222047	0	0	0	0
ENSG00000222051	0	0	0	0
ENSG00000222054	0	0	0	0
ENSG00000222055	0	0	0	0
ENSG00000222057	1	0	0	0
ENSG00000222064	0	0	0	0
ENSG00000222066	0	0	0	0
ENSG00000222067	0	0	0	0
ENSG00000222068	0	0	0	0
ENSG00000222069	0	0	0	0
ENSG00000222070	0	0	0	0
ENSG00000222071	0	0	0	0

ENSG00000222072	0	0	0	0
ENSG00000222076	0	0	0	0
ENSG00000222078	0	0	0	0
ENSG00000222079	0	0	0	0
ENSG00000222080	0	0	0	0
ENSG00000222084	0	0	0	0
ENSG00000222085	0	0	0	0
ENSG00000222086	0	0	0	0
ENSG00000222087	0	0	0	0
ENSG00000222092	0	0	0	0
ENSG00000222094	0	0	0	0
ENSG00000222095	0	0	0	0
ENSG00000222096	0	0	0	0
ENSG00000222099	0	0	0	0
ENSG00000222100	0	0	0	0
ENSG00000222101	0	0	0	0
ENSG00000222102	0	0	0	0
ENSG00000222107	0	0	0	0
ENSG00000222108	0	0	0	0
ENSG00000222109	0	0	0	0
ENSG00000222111	0	0	0	0
ENSG00000222112	0	0	0	0
ENSG00000222114	0	0	0	0
ENSG00000222117	0	0	0	0
ENSG00000222118	0	0	0	0
ENSG00000222120	0	0	0	0
ENSG00000222121	0	0	0	0
ENSG00000222122	0	0	0	0
ENSG00000222123	0	0	0	0
ENSG00000222126	0	0	0	0
ENSG00000222129	0	0	0	0
ENSG00000222133	0	0	0	0
ENSG00000222139	0	0	0	0
ENSG00000222145	0	0	0	0
ENSG00000222146	0	0	0	0
ENSG00000222148	0	0	0	0
ENSG00000222150	0	0	0	0
ENSG00000222152	0	0	0	0
ENSG00000222154	0	0	0	0
ENSG00000222158	0	0	0	0
ENSG00000222160	0	0	0	0
ENSG00000222161	0	0	0	0
ENSG00000222162	0	0	0	0
ENSG00000222164	0	0	0	0
ENSG00000222168	0	0	0	0
ENSG00000222169	0	0	0	0

ENSG00000222170	0	0	0	0
ENSG00000222174	0	0	0	0
ENSG00000222177	0	0	0	0
ENSG00000222178	0	0	0	0
ENSG00000222179	0	0	0	0
ENSG00000222182	0	0	0	0
ENSG00000222185	0	0	0	0
ENSG00000222190	0	0	0	0
ENSG00000222192	0	0	0	0
ENSG00000222196	0	0	0	0
ENSG00000222197	0	0	0	0
ENSG00000222202	0	0	0	0
ENSG00000222203	0	0	0	0
ENSG00000222205	0	0	0	0
ENSG00000222206	0	0	0	0
ENSG00000222207	0	0	0	0
ENSG00000222208	0	0	0	0
ENSG00000222209	0	0	0	0
ENSG00000222210	0	0	0	0
ENSG00000222213	0	0	0	0
ENSG00000222219	0	0	0	0
ENSG00000222220	0	0	0	0
ENSG00000222222	0	0	0	0
ENSG00000222224	0	0	0	0
ENSG00000222225	0	0	0	0
ENSG00000222226	0	0	0	0
ENSG00000222230	0	0	0	0
ENSG00000222231	0	0	0	0
ENSG00000222232	0	0	0	0
ENSG00000222234	0	0	1	0
ENSG00000222235	0	0	0	0
ENSG00000222236	0	0	0	0
ENSG00000222238	0	0	0	0
ENSG00000222240	0	0	0	0
ENSG00000222244	0	0	0	0
ENSG00000222246	0	0	0	0
ENSG00000222248	0	0	0	0
ENSG00000222249	0	0	0	0
ENSG00000222251	0	0	0	0
ENSG00000222255	0	0	0	0
ENSG00000222257	0	0	0	0
ENSG00000222259	0	0	0	0
ENSG00000222262	0	0	0	0
ENSG00000222266	0	0	0	0
ENSG00000222267	0	0	0	0
ENSG00000222268	0	0	0	0

ENSG00000222274	0	0	0	0
ENSG00000222276	0	0	0	0
ENSG00000222281	0	0	0	0
ENSG00000222282	0	0	0	0
ENSG00000222285	0	0	0	0
ENSG00000222287	0	0	0	0
ENSG00000222293	0	0	0	0
ENSG00000222296	0	0	0	0
ENSG00000222297	0	0	0	0
ENSG00000222298	0	0	0	0
ENSG00000222299	0	0	0	0
ENSG00000222300	0	0	0	0
ENSG00000222301	0	0	0	0
ENSG00000222302	0	0	0	0
ENSG00000222303	0	0	0	0
ENSG00000222305	0	0	0	0
ENSG00000222306	0	0	0	0
ENSG00000222308	0	0	0	0
ENSG00000222309	0	0	0	0
ENSG00000222311	0	0	0	0
ENSG00000222312	0	0	0	0
ENSG00000222313	0	0	0	0
ENSG00000222314	0	0	0	0
ENSG00000222316	0	0	0	0
ENSG00000222317	0	0	0	0
ENSG00000222320	0	0	0	0
ENSG00000222321	0	0	0	0
ENSG00000222326	0	0	0	0
ENSG00000222327	0	0	0	0
ENSG00000222328	0	0	0	0
ENSG00000222329	0	0	0	0
ENSG00000222331	0	0	0	0
ENSG00000222333	0	0	0	1
ENSG00000222334	0	0	0	0
ENSG00000222335	0	0	0	0
ENSG00000222337	0	0	0	0
ENSG00000222338	0	0	0	0
ENSG00000222339	0	0	0	5
ENSG00000222343	0	0	0	0
ENSG00000222344	0	0	0	0
ENSG00000222345	0	0	0	0
ENSG00000222346	0	0	0	0
ENSG00000222348	0	0	0	0
ENSG00000222351	0	0	0	0
ENSG00000222352	0	0	0	0
ENSG00000222355	0	0	0	0

ENSG00000222356	0	0	0	0
ENSG00000222357	0	0	0	0
ENSG00000222359	0	0	0	0
ENSG00000222361	0	0	0	0
ENSG00000222363	0	0	0	0
ENSG00000222364	0	0	0	0
ENSG00000222365	0	0	0	0
ENSG00000222370	0	0	0	0
ENSG00000222371	0	0	0	0
ENSG00000222373	0	0	0	0
ENSG00000222374	0	0	0	0
ENSG00000222375	0	0	0	0
ENSG00000222376	0	0	0	0
ENSG00000222378	0	0	0	0
ENSG00000222383	0	0	0	0
ENSG00000222385	0	0	0	0
ENSG00000222386	0	0	0	0
ENSG00000222389	0	0	0	0
ENSG00000222391	0	0	0	0
ENSG00000222394	0	0	0	0
ENSG00000222395	0	0	0	0
ENSG00000222397	0	0	0	0
ENSG00000222398	0	0	0	2
ENSG00000222399	0	0	0	0
ENSG00000222404	0	0	0	0
ENSG00000222405	0	0	0	0
ENSG00000222406	0	0	0	0
ENSG00000222407	0	0	0	0
ENSG00000222408	0	0	0	0
ENSG00000222409	0	0	0	0
ENSG00000222411	0	0	0	0
ENSG00000222412	0	0	0	0
ENSG00000222413	0	0	0	0
ENSG00000222414	0	0	0	0
ENSG00000222416	0	0	0	0
ENSG00000222417	0	0	0	0
ENSG00000222418	0	0	0	0
ENSG00000222419	0	0	0	0
ENSG00000222421	0	0	0	0
ENSG00000222426	0	0	0	0
ENSG00000222427	0	0	0	0
ENSG00000222428	0	0	0	0
ENSG00000222429	0	0	0	0
ENSG00000222430	0	0	0	0
ENSG00000222431	0	0	0	0
ENSG00000222432	0	0	0	0

ENSG00000222436	0	0	0	0
ENSG00000222437	0	0	0	0
ENSG00000222438	0	0	0	0
ENSG00000222439	0	0	0	0
ENSG00000222440	0	0	0	0
ENSG00000222445	0	0	0	0
ENSG00000222448	0	0	0	0
ENSG00000222449	0	0	0	0
ENSG00000222451	0	0	0	0
ENSG00000222452	0	0	0	0
ENSG00000222455	0	0	0	0
ENSG00000222457	0	0	0	0
ENSG00000222459	0	0	0	0
ENSG00000222460	0	0	0	0
ENSG00000222463	0	0	0	0
ENSG00000222465	0	0	0	0
ENSG00000222466	0	0	0	0
ENSG00000222467	0	0	0	0
ENSG00000222468	0	0	0	0
ENSG00000222472	0	0	0	0
ENSG00000222477	0	0	0	0
ENSG00000222481	0	0	0	0
ENSG00000222482	0	0	0	0
ENSG00000222486	0	0	0	0
ENSG00000222488	0	0	0	0
ENSG00000222489	0	0	27	19
ENSG00000222490	0	0	0	0
ENSG00000222492	0	0	0	0
ENSG00000222493	0	0	0	0
ENSG00000222494	0	0	0	0
ENSG00000222496	0	0	0	0
ENSG00000222499	0	0	0	0
ENSG00000222500	0	0	0	0
ENSG00000222501	0	0	0	0
ENSG00000222503	0	0	0	0
ENSG00000222506	0	0	0	0
ENSG00000222509	0	0	0	0
ENSG00000222511	0	0	0	0
ENSG00000222512	0	0	0	0
ENSG00000222514	0	0	0	0
ENSG00000222515	0	0	0	0
ENSG00000222518	0	0	0	0
ENSG00000222520	0	0	0	0
ENSG00000222521	0	0	0	0
ENSG00000222522	0	0	0	0
ENSG00000222524	0	0	0	0

ENSG00000222526	0	0	0	0
ENSG00000222529	0	0	0	0
ENSG00000222532	0	0	0	0
ENSG00000222533	0	0	0	0
ENSG00000222536	0	0	0	0
ENSG00000222543	0	0	0	0
ENSG00000222544	0	0	0	0
ENSG00000222545	0	0	0	0
ENSG00000222546	0	0	0	0
ENSG00000222548	0	0	0	0
ENSG00000222551	0	0	0	0
ENSG00000222552	0	0	0	0
ENSG00000222558	0	0	0	0
ENSG00000222560	0	0	0	0
ENSG00000222561	0	0	0	0
ENSG00000222563	0	0	0	0
ENSG00000222567	0	0	0	0
ENSG00000222570	0	0	0	0
ENSG00000222574	0	0	0	0
ENSG00000222578	0	0	0	0
ENSG00000222579	0	0	0	0
ENSG00000222581	0	0	0	0
ENSG00000222582	0	0	0	0
ENSG00000222583	0	0	0	0
ENSG00000222585	0	0	0	0
ENSG00000222586	0	0	0	0
ENSG00000222588	0	0	0	0
ENSG00000222589	0	0	0	0
ENSG00000222591	0	0	0	0
ENSG00000222592	0	0	0	0
ENSG00000222594	0	0	0	0
ENSG00000222596	0	0	0	0
ENSG00000222598	0	0	0	0
ENSG00000222601	0	0	0	0
ENSG00000222602	0	0	0	0
ENSG00000222604	0	0	0	0
ENSG00000222607	0	0	0	0
ENSG00000222608	0	0	0	0
ENSG00000222609	0	0	0	0
ENSG00000222610	0	0	0	0
ENSG00000222611	0	0	0	0
ENSG00000222612	0	0	4	1
ENSG00000222613	0	0	0	0
ENSG00000222614	0	0	0	0
ENSG00000222615	0	0	0	0
ENSG00000222616	0	0	1	0

ENSG00000222617	0	0	0	0
ENSG00000222623	0	0	0	0
ENSG00000222624	0	0	0	0
ENSG00000222626	0	0	0	0
ENSG00000222627	0	0	0	0
ENSG00000222629	0	0	0	0
ENSG00000222630	0	0	0	0
ENSG00000222631	0	0	0	0
ENSG00000222635	0	0	0	0
ENSG00000222636	0	0	0	0
ENSG00000222640	0	0	0	0
ENSG00000222643	0	0	0	0
ENSG00000222644	0	0	0	0
ENSG00000222647	0	0	0	0
ENSG00000222649	0	0	0	0
ENSG00000222650	0	0	0	0
ENSG00000222651	0	0	0	0
ENSG00000222652	0	0	0	0
ENSG00000222658	0	0	0	0
ENSG00000222659	0	0	0	0
ENSG00000222663	0	0	0	0
ENSG00000222664	0	0	0	0
ENSG00000222666	0	0	0	0
ENSG00000222667	0	0	0	0
ENSG00000222670	0	0	0	0
ENSG00000222675	0	0	0	0
ENSG00000222677	0	0	0	0
ENSG00000222678	0	0	0	0
ENSG00000222679	0	0	0	0
ENSG00000222682	0	0	0	0
ENSG00000222683	0	0	0	0
ENSG00000222685	0	0	0	0
ENSG00000222686	0	0	0	0
ENSG00000222687	0	0	0	0
ENSG00000222690	0	0	0	0
ENSG00000222691	0	0	0	0
ENSG00000222692	0	0	0	0
ENSG00000222693	0	0	0	0
ENSG00000222698	0	0	0	0
ENSG00000222701	0	0	0	0
ENSG00000222704	0	0	0	0
ENSG00000222705	0	0	0	0
ENSG00000222706	0	0	0	0
ENSG00000222713	0	0	0	0
ENSG00000222714	0	0	0	0
ENSG00000222715	0	0	0	0

ENSG00000222717	0	0	0	0
ENSG00000222719	0	0	0	0
ENSG00000222720	0	0	0	0
ENSG00000222721	0	0	0	0
ENSG00000222722	0	0	0	0
ENSG00000222724	0	0	0	0
ENSG00000222726	1	0	2	0
ENSG00000222727	0	0	0	0
ENSG00000222729	0	0	0	0
ENSG00000222730	0	0	0	0
ENSG00000222732	0	0	0	0
ENSG00000222733	0	0	0	0
ENSG00000222736	0	0	0	0
ENSG00000222740	0	0	0	0
ENSG00000222741	0	0	0	0
ENSG00000222743	0	0	0	0
ENSG00000222744	0	0	0	0
ENSG00000222747	0	0	0	0
ENSG00000222749	0	0	0	0
ENSG00000222750	0	0	0	0
ENSG00000222755	0	0	0	0
ENSG00000222760	0	0	0	0
ENSG00000222761	0	0	0	0
ENSG00000222762	0	0	0	0
ENSG00000222764	6	69	76	47
ENSG00000222765	0	0	0	0
ENSG00000222767	0	0	0	0
ENSG00000222770	0	0	0	0
ENSG00000222774	0	0	0	0
ENSG00000222776	0	0	0	0
ENSG00000222777	0	0	0	0
ENSG00000222778	0	0	0	0
ENSG00000222783	0	0	0	0
ENSG00000222784	0	0	0	0
ENSG00000222787	0	0	0	0
ENSG00000222788	0	0	0	0
ENSG00000222790	0	0	0	0
ENSG00000222791	0	0	0	0
ENSG00000222792	0	0	0	0
ENSG00000222795	0	0	0	0
ENSG00000222796	0	0	0	0
ENSG00000222800	0	0	0	0
ENSG00000222805	0	0	0	0
ENSG00000222806	0	0	0	0
ENSG00000222808	1	0	0	0
ENSG00000222810	0	0	0	0

ENSG00000222821	0	0	0	0
ENSG00000222826	0	0	0	0
ENSG00000222831	0	0	0	0
ENSG00000222832	0	0	0	0
ENSG00000222833	0	0	0	0
ENSG00000222834	0	0	0	0
ENSG00000222835	0	0	0	0
ENSG00000222838	0	0	0	0
ENSG00000222842	0	0	0	0
ENSG00000222844	0	0	0	0
ENSG00000222845	0	0	0	0
ENSG00000222849	0	0	0	0
ENSG00000222852	0	0	0	0
ENSG00000222854	0	0	0	0
ENSG00000222858	0	0	0	0
ENSG00000222859	0	0	0	0
ENSG00000222861	0	0	0	0
ENSG00000222862	0	0	0	0
ENSG00000222864	0	0	0	0
ENSG00000222869	0	0	0	0
ENSG00000222870	0	0	0	0
ENSG00000222872	0	0	0	0
ENSG00000222874	0	0	0	0
ENSG00000222880	0	0	0	0
ENSG00000222881	0	0	0	0
ENSG00000222883	0	0	0	0
ENSG00000222884	0	0	0	0
ENSG00000222888	0	0	0	0
ENSG00000222889	0	0	0	0
ENSG00000222890	0	0	0	0
ENSG00000222894	1	0	0	1
ENSG00000222895	0	0	0	0
ENSG00000222898	0	0	3	0
ENSG00000222899	0	0	0	0
ENSG00000222909	0	0	0	0
ENSG00000222915	0	0	0	0
ENSG00000222920	0	0	0	0
ENSG00000222921	0	0	0	0
ENSG00000222922	0	0	0	0
ENSG00000222923	0	0	0	0
ENSG00000222924	0	0	0	0
ENSG00000222931	0	0	0	0
ENSG00000222932	0	0	0	0
ENSG00000222934	0	0	0	0
ENSG00000222937	0	0	0	0
ENSG00000222939	0	0	0	0

ENSG00000222940	0	0	2	0
ENSG00000222941	0	0	0	0
ENSG00000222942	0	0	0	0
ENSG00000222944	0	0	0	0
ENSG00000222945	0	0	0	0
ENSG00000222946	0	0	0	0
ENSG00000222950	0	0	0	0
ENSG00000222952	0	0	0	0
ENSG00000222954	0	0	0	0
ENSG00000222955	0	0	0	0
ENSG00000222958	0	0	0	0
ENSG00000222960	0	0	0	0
ENSG00000222961	0	0	0	0
ENSG00000222966	0	0	0	0
ENSG00000222969	0	0	0	0
ENSG00000222971	0	0	0	0
ENSG00000222972	0	0	0	0
ENSG00000222973	0	0	0	0
ENSG00000222974	0	0	0	0
ENSG00000222976	0	0	0	0
ENSG00000222978	0	0	0	0
ENSG00000222979	0	0	0	0
ENSG00000222982	0	0	0	0
ENSG00000222983	0	0	0	0
ENSG00000222985	28	0	12	1
ENSG00000222986	0	0	0	0
ENSG00000222987	0	0	0	0
ENSG00000222990	0	0	0	0
ENSG00000222994	0	0	0	0
ENSG00000222997	0	0	0	0
ENSG00000222998	0	0	0	0
ENSG00000222999	0	0	0	0
ENSG00000223001	0	0	0	0
ENSG00000223003	1	0	1	0
ENSG00000223004	0	0	0	0
ENSG00000223006	0	0	0	0
ENSG00000223007	0	0	0	0
ENSG00000223009	0	0	0	0
ENSG00000223010	0	0	0	0
ENSG00000223012	0	0	0	0
ENSG00000223013	0	0	0	0
ENSG00000223015	0	0	0	0
ENSG00000223018	0	0	0	0
ENSG00000223019	0	0	0	3
ENSG00000223023	0	0	0	0
ENSG00000223024	0	0	0	0

ENSG00000223026	0	0	0	0
ENSG00000223027	0	0	0	0
ENSG00000223029	0	0	0	0
ENSG00000223030	0	0	0	0
ENSG00000223036	0	0	0	0
ENSG00000223037	0	0	0	0
ENSG00000223039	0	0	0	0
ENSG00000223040	0	0	0	0
ENSG00000223042	0	0	0	0
ENSG00000223044	0	0	0	0
ENSG00000223046	0	0	0	0
ENSG00000223047	0	0	0	0
ENSG00000223052	0	0	0	0
ENSG00000223053	0	0	0	0
ENSG00000223056	0	0	0	0
ENSG00000223059	0	0	0	0
ENSG00000223060	0	0	0	0
ENSG00000223061	0	0	0	1
ENSG00000223062	0	0	0	0
ENSG00000223063	0	0	0	0
ENSG00000223064	0	0	0	0
ENSG00000223070	0	0	0	0
ENSG00000223075	0	0	0	0
ENSG00000223076	0	0	0	0
ENSG00000223078	0	0	0	0
ENSG00000223080	0	0	0	0
ENSG00000223082	0	0	0	0
ENSG00000223086	0	0	0	0
ENSG00000223087	0	0	0	0
ENSG00000223088	0	0	0	0
ENSG00000223091	0	0	0	0
ENSG00000223092	0	0	0	0
ENSG00000223096	0	0	0	0
ENSG00000223099	0	0	0	0
ENSG00000223101	0	0	0	0
ENSG00000223107	0	0	0	0
ENSG00000223109	0	0	0	1
ENSG00000223111	0	0	0	0
ENSG00000223113	0	0	0	0
ENSG00000223116	0	0	0	0
ENSG00000223117	0	0	0	0
ENSG00000223118	0	0	0	0
ENSG00000223119	0	0	0	0
ENSG00000223120	0	0	0	0
ENSG00000223123	0	0	0	0
ENSG00000223125	0	0	0	0

ENSG00000223126	0	0	0	0
ENSG00000223128	0	0	0	0
ENSG00000223131	0	0	0	0
ENSG00000223134	0	0	0	0
ENSG00000223136	0	0	0	0
ENSG00000223138	1	0	0	0
ENSG00000223142	0	0	0	0
ENSG00000223145	0	0	0	0
ENSG00000223148	0	0	0	0
ENSG00000223149	0	0	0	0
ENSG00000223152	0	0	0	0
ENSG00000223154	0	0	0	0
ENSG00000223156	0	0	0	0
ENSG00000223158	0	0	0	0
ENSG00000223162	0	0	0	0
ENSG00000223164	0	0	0	0
ENSG00000223168	0	0	0	0
ENSG00000223169	0	0	0	0
ENSG00000223174	0	0	0	0
ENSG00000223175	0	0	0	0
ENSG00000223177	0	0	0	0
ENSG00000223179	0	0	0	0
ENSG00000223180	0	0	0	0
ENSG00000223181	0	0	0	0
ENSG00000223182	0	0	0	0
ENSG00000223187	0	0	0	0
ENSG00000223188	0	0	0	0
ENSG00000223189	0	0	0	0
ENSG00000223190	0	0	0	0
ENSG00000223191	0	0	0	0
ENSG00000223192	0	0	0	0
ENSG00000223197	0	0	0	0
ENSG00000223198	0	0	0	0
ENSG00000223200	0	0	0	0
ENSG00000223201	0	0	0	0
ENSG00000223202	0	0	0	0
ENSG00000223203	0	0	0	0
ENSG00000223208	0	0	0	0
ENSG00000223211	0	0	0	0
ENSG00000223212	0	0	0	0
ENSG00000223213	0	0	0	0
ENSG00000223214	0	0	0	0
ENSG00000223215	0	0	0	0
ENSG00000223217	0	0	0	0
ENSG00000223220	0	0	0	0
ENSG00000223223	0	0	0	0

ENSG00000223224	0	0	0	0
ENSG00000223225	0	0	0	0
ENSG00000223229	0	0	0	0
ENSG00000223231	0	0	0	0
ENSG00000223235	0	0	0	0
ENSG00000223238	0	0	0	0
ENSG00000223243	0	0	0	0
ENSG00000223244	0	0	0	0
ENSG00000223245	0	0	0	0
ENSG00000223247	0	0	0	0
ENSG00000223249	0	0	0	0
ENSG00000223253	0	0	0	0
ENSG00000223254	0	0	0	0
ENSG00000223255	0	0	0	0
ENSG00000223256	0	0	0	0
ENSG00000223258	0	0	0	0
ENSG00000223259	0	0	0	0
ENSG00000223260	0	0	0	0
ENSG00000223262	0	0	0	0
ENSG00000223263	0	0	0	0
ENSG00000223265	0	0	0	0
ENSG00000223269	0	0	0	0
ENSG00000223271	0	0	0	0
ENSG00000223273	0	0	0	0
ENSG00000223274	0	0	0	0
ENSG00000223275	0	0	0	0
ENSG00000223279	0	0	11	2
ENSG00000223280	0	0	0	0
ENSG00000223281	0	0	0	0
ENSG00000223282	0	0	0	0
ENSG00000223284	0	0	0	0
ENSG00000223286	0	0	0	0
ENSG00000223287	0	0	0	0
ENSG00000223290	0	0	0	0
ENSG00000223291	0	0	0	0
ENSG00000223292	0	0	0	0
ENSG00000223293	0	0	0	0
ENSG00000223294	0	0	0	0
ENSG00000223297	0	0	0	0
ENSG00000223298	213	0	0	0
ENSG00000223299	0	0	0	0
ENSG00000223300	0	0	0	0
ENSG00000223302	0	0	0	0
ENSG00000223305	0	0	3	0
ENSG00000223306	0	0	0	0
ENSG00000223307	0	0	0	0

ENSG00000223308	0	0	0	0
ENSG00000223309	0	0	0	0
ENSG00000223313	0	0	0	0
ENSG00000223315	0	0	0	0
ENSG00000223316	0	0	0	0
ENSG00000223318	0	0	0	0
ENSG00000223321	0	0	0	3
ENSG00000223322	0	0	0	0
ENSG00000223324	0	0	0	0
ENSG00000223327	0	0	0	0
ENSG00000223330	0	0	0	0
ENSG00000223335	0	0	0	0
ENSG00000223336	0	0	2	9
ENSG00000223341	0	0	0	0
ENSG00000223342	0	0	0	0
ENSG00000223343	0	0	1	4
ENSG00000223344	0	0	0	0
ENSG00000223345	0	1	0	0
ENSG00000223349	0	0	0	0
ENSG00000223350	0	0	2	7
ENSG00000223351	0	0	0	0
ENSG00000223352	0	0	0	0
ENSG00000223353	0	0	0	0
ENSG00000223355	0	0	0	0
ENSG00000223356	0	0	0	5
ENSG00000223358	0	0	0	0
ENSG00000223359	0	0	0	0
ENSG00000223360	0	0	0	0
ENSG00000223361	20	15	38	23
ENSG00000223362	0	0	0	0
ENSG00000223364	0	0	0	0
ENSG00000223365	0	0	0	0
ENSG00000223367	0	0	0	0
ENSG00000223368	0	0	0	0
ENSG00000223372	0	0	0	0
ENSG00000223373	0	0	0	0
ENSG00000223374	0	27	99	25
ENSG00000223375	0	0	0	0
ENSG00000223376	0	0	0	0
ENSG00000223377	0	0	0	0
ENSG00000223379	0	0	0	0
ENSG00000223380	80	679	711	847
ENSG00000223381	0	0	0	0
ENSG00000223382	0	0	0	0
ENSG00000223383	0	0	0	0
ENSG00000223387	0	0	0	0

ENSG00000223389	0	0	0	0
ENSG00000223390	0	0	0	0
ENSG00000223391	0	0	0	0
ENSG00000223392	0	0	0	0
ENSG00000223393	2	11	3	5
ENSG00000223394	0	0	0	0
ENSG00000223395	0	0	0	0
ENSG00000223396	2	0	0	1
ENSG00000223398	0	0	0	0
ENSG00000223400	0	0	0	0
ENSG00000223401	0	0	2	0
ENSG00000223402	0	0	0	0
ENSG00000223403	1	0	0	0
ENSG00000223404	0	0	0	0
ENSG00000223405	0	0	0	0
ENSG00000223406	0	0	0	0
ENSG00000223407	0	0	0	0
ENSG00000223408	0	0	0	0
ENSG00000223409	0	0	0	0
ENSG00000223410	0	0	0	0
ENSG00000223412	0	0	0	0
ENSG00000223414	0	0	0	0
ENSG00000223416	1	0	0	0
ENSG00000223417	0	0	0	0
ENSG00000223418	0	0	0	0
ENSG00000223419	0	0	0	0
ENSG00000223421	0	0	0	0
ENSG00000223422	0	0	0	0
ENSG00000223427	0	0	0	0
ENSG00000223428	0	0	0	0
ENSG00000223429	0	0	0	0
ENSG00000223430	0	0	0	0
ENSG00000223431	0	0	0	0
ENSG00000223432	0	0	0	0
ENSG00000223433	0	0	0	0
ENSG00000223436	0	0	0	0
ENSG00000223437	0	0	0	0
ENSG00000223438	0	0	0	0
ENSG00000223439	0	0	0	0
ENSG00000223440	0	0	0	0
ENSG00000223441	0	0	0	0
ENSG00000223442	0	0	2	0
ENSG00000223443	0	0	0	0
ENSG00000223444	0	0	0	0
ENSG00000223445	0	0	0	0
ENSG00000223446	0	0	0	0

ENSG00000223447	0	0	0	0
ENSG00000223448	0	0	0	0
ENSG00000223449	0	0	0	0
ENSG00000223450	0	0	0	0
ENSG00000223452	0	0	0	0
ENSG00000223454	0	0	0	0
ENSG00000223455	0	0	0	0
ENSG00000223456	0	0	0	0
ENSG00000223457	0	0	0	0
ENSG00000223458	0	0	0	0
ENSG00000223459	0	0	0	0
ENSG00000223460	0	0	0	0
ENSG00000223461	1	0	2	0
ENSG00000223462	0	0	0	0
ENSG00000223463	0	0	0	0
ENSG00000223465	0	0	0	0
ENSG00000223466	0	0	0	0
ENSG00000223467	0	0	0	0
ENSG00000223469	0	0	0	0
ENSG00000223470	0	0	0	0
ENSG00000223473	1	0	2	6
ENSG00000223474	0	0	0	0
ENSG00000223475	0	0	0	0
ENSG00000223476	0	0	1	0
ENSG00000223477	0	0	0	0
ENSG00000223478	233	115	59	156
ENSG00000223479	0	0	0	0
ENSG00000223481	0	0	0	0
ENSG00000223482	890	1509	3356	2497
ENSG00000223484	0	0	0	0
ENSG00000223485	0	0	0	0
ENSG00000223486	48	3	19	36
ENSG00000223487	0	0	0	0
ENSG00000223488	0	0	0	0
ENSG00000223489	0	0	0	0
ENSG00000223490	0	0	0	0
ENSG00000223491	0	0	0	0
ENSG00000223492	0	0	0	0
ENSG00000223493	0	0	0	0
ENSG00000223494	0	0	0	0
ENSG00000223495	0	0	0	0
ENSG00000223496	8	0	1	2
ENSG00000223497	0	0	0	0
ENSG00000223498	0	0	0	0
ENSG00000223500	0	0	0	0
ENSG00000223501	1	22	35	71

ENSG00000223502	32	13	5	6
ENSG00000223503	0	0	0	0
ENSG00000223504	0	0	0	0
ENSG00000223505	0	0	0	0
ENSG00000223506	0	0	0	0
ENSG00000223508	3	137	92	165
ENSG00000223509	417	8848	10801	5084
ENSG00000223510	0	0	0	0
ENSG00000223511	0	0	0	14
ENSG00000223512	0	0	0	0
ENSG00000223513	0	0	0	0
ENSG00000223514	0	0	0	0
ENSG00000223516	0	0	0	0
ENSG00000223517	0	0	0	0
ENSG00000223518	0	0	1	0
ENSG00000223519	261	1736	1983	1868
ENSG00000223522	0	0	0	0
ENSG00000223523	0	0	1	11
ENSG00000223524	0	0	0	0
ENSG00000223525	4	1	6	7
ENSG00000223528	0	0	4	10
ENSG00000223529	0	0	0	0
ENSG00000223530	0	0	0	0
ENSG00000223531	0	0	0	0
ENSG00000223532	0	0	0	0
ENSG00000223533	0	0	0	0
ENSG00000223534	0	0	0	0
ENSG00000223535	0	0	0	0
ENSG00000223536	0	0	0	0
ENSG00000223537	0	0	0	0
ENSG00000223538	0	0	0	0
ENSG00000223539	0	0	0	0
ENSG00000223540	0	0	0	0
ENSG00000223542	0	0	0	0
ENSG00000223543	0	0	0	0
ENSG00000223544	0	0	0	0
ENSG00000223546	0	100	133	147
ENSG00000223547	9	36	92	64
ENSG00000223548	0	0	0	0
ENSG00000223549	0	0	0	0
ENSG00000223550	0	0	0	0
ENSG00000223551	2000	283	239	276
ENSG00000223552	3	18	5	0
ENSG00000223553	1270	4063	1169	27
ENSG00000223554	0	0	0	0
ENSG00000223555	0	0	0	0

ENSG00000223557	0	0	0	0
ENSG00000223558	0	0	0	0
ENSG00000223559	0	0	0	0
ENSG00000223560	0	0	0	0
ENSG00000223561	0	0	8	0
ENSG00000223562	0	0	0	0
ENSG00000223563	0	0	0	0
ENSG00000223564	0	0	0	0
ENSG00000223565	0	0	0	0
ENSG00000223566	0	0	0	0
ENSG00000223568	0	0	0	0
ENSG00000223569	0	0	0	0
ENSG00000223570	0	0	0	0
ENSG00000223571	0	0	0	0
ENSG00000223572	0	0	0	0
ENSG00000223573	1	0	0	0
ENSG00000223574	0	0	0	0
ENSG00000223575	0	0	0	0
ENSG00000223576	0	0	0	0
ENSG00000223577	0	0	0	0
ENSG00000223579	0	0	0	0
ENSG00000223581	0	0	0	0
ENSG00000223583	0	0	0	0
ENSG00000223584	0	0	0	0
ENSG00000223586	0	0	0	0
ENSG00000223587	0	0	0	0
ENSG00000223588	0	0	0	0
ENSG00000223589	0	0	0	0
ENSG00000223590	0	0	0	0
ENSG00000223591	0	0	0	0
ENSG00000223592	0	0	0	0
ENSG00000223593	0	0	0	0
ENSG00000223595	0	0	0	0
ENSG00000223597	0	0	0	0
ENSG00000223598	0	0	0	0
ENSG00000223599	1	0	15	6
ENSG00000223600	0	0	0	0
ENSG00000223601	0	0	0	0
ENSG00000223602	0	0	0	0
ENSG00000223603	0	0	0	0
ENSG00000223604	0	0	0	0
ENSG00000223605	0	0	0	0
ENSG00000223606	0	0	0	0
ENSG00000223608	0	0	0	0
ENSG00000223609	56	44	18	65
ENSG00000223611	0	0	0	0

ENSG00000223612	0	0	0	3
ENSG00000223614	0	0	0	0
ENSG00000223615	0	0	0	0
ENSG00000223617	0	0	0	0
ENSG00000223618	0	0	0	0
ENSG00000223619	0	0	3	0
ENSG00000223620	0	0	0	0
ENSG00000223621	0	0	0	0
ENSG00000223622	158	298	172	789
ENSG00000223623	0	0	0	0
ENSG00000223624	0	0	0	0
ENSG00000223625	0	0	0	0
ENSG00000223626	0	0	0	0
ENSG00000223627	0	0	0	0
ENSG00000223628	0	0	0	0
ENSG00000223629	0	0	0	0
ENSG00000223631	0	0	0	0
ENSG00000223633	0	0	0	0
ENSG00000223634	0	0	0	0
ENSG00000223635	8	0	3	9
ENSG00000223636	0	0	0	0
ENSG00000223637	0	0	0	0
ENSG00000223638	0	0	0	0
ENSG00000223639	0	0	0	0
ENSG00000223640	0	0	0	0
ENSG00000223641	0	0	0	0
ENSG00000223642	0	0	0	0
ENSG00000223643	0	0	0	0
ENSG00000223646	0	15	4	0
ENSG00000223647	0	0	1	1
ENSG00000223648	0	0	0	0
ENSG00000223649	0	0	0	0
ENSG00000223650	0	0	0	0
ENSG00000223651	0	0	0	0
ENSG00000223652	0	0	0	0
ENSG00000223653	7	10	5	9
ENSG00000223654	0	0	0	0
ENSG00000223655	0	0	0	0
ENSG00000223656	0	0	0	0
ENSG00000223657	0	0	0	0
ENSG00000223658	0	0	0	0
ENSG00000223659	0	0	0	0
ENSG00000223660	0	0	0	0
ENSG00000223661	0	0	0	0
ENSG00000223662	0	0	0	0
ENSG00000223663	0	0	0	0

ENSG00000223664	0	0	0	0
ENSG00000223665	1	0	0	0
ENSG00000223666	0	0	0	0
ENSG00000223668	0	0	0	0
ENSG00000223669	0	0	0	0
ENSG00000223671	0	0	0	0
ENSG00000223672	0	0	0	0
ENSG00000223675	0	0	0	0
ENSG00000223676	0	0	0	0
ENSG00000223677	0	0	0	0
ENSG00000223678	0	0	0	0
ENSG00000223679	0	0	0	0
ENSG00000223680	0	0	0	0
ENSG00000223683	0	0	0	0
ENSG00000223684	0	0	0	0
ENSG00000223685	0	15	42	115
ENSG00000223687	0	0	0	0
ENSG00000223688	0	0	0	0
ENSG00000223691	0	0	0	0
ENSG00000223692	0	0	2	1
ENSG00000223693	0	0	0	0
ENSG00000223694	0	0	0	0
ENSG00000223695	12	0	6	29
ENSG00000223696	0	0	0	0
ENSG00000223697	0	0	8	1
ENSG00000223698	0	0	0	0
ENSG00000223699	0	0	0	0
ENSG00000223700	0	0	0	0
ENSG00000223701	0	0	0	0
ENSG00000223702	0	0	0	0
ENSG00000223703	0	0	0	0
ENSG00000223704	0	0	2	6
ENSG00000223705	4	0	0	1
ENSG00000223707	0	0	0	0
ENSG00000223708	0	0	0	0
ENSG00000223709	1	0	2	0
ENSG00000223710	0	0	0	0
ENSG00000223711	0	0	0	0
ENSG00000223714	0	0	0	0
ENSG00000223715	0	0	1	12
ENSG00000223716	0	0	0	0
ENSG00000223717	0	0	0	0
ENSG00000223718	0	0	0	0
ENSG00000223719	0	0	0	0
ENSG00000223720	0	0	0	0
ENSG00000223721	0	0	0	0

ENSG00000223722	0	0	0	0
ENSG00000223723	0	0	0	0
ENSG00000223724	0	0	0	0
ENSG00000223725	2	24	7	15
ENSG00000223726	0	0	0	0
ENSG00000223727	1	0	3	1
ENSG00000223728	0	0	0	0
ENSG00000223729	0	0	0	0
ENSG00000223730	0	0	0	0
ENSG00000223731	0	0	0	0
ENSG00000223732	0	0	2	0
ENSG00000223733	0	0	0	0
ENSG00000223734	0	0	0	0
ENSG00000223735	0	0	0	0
ENSG00000223738	0	0	0	0
ENSG00000223739	0	0	0	0
ENSG00000223740	0	0	0	0
ENSG00000223741	0	0	0	0
ENSG00000223742	0	0	0	0
ENSG00000223744	0	0	0	0
ENSG00000223745	45	648	511	752
ENSG00000223746	0	0	0	0
ENSG00000223747	0	0	0	0
ENSG00000223749	1	0	0	0
ENSG00000223750	0	0	0	0
ENSG00000223751	0	0	0	0
ENSG00000223752	0	0	0	0
ENSG00000223753	2	0	0	0
ENSG00000223754	0	0	0	0
ENSG00000223756	1	6	1	6
ENSG00000223757	0	0	0	0
ENSG00000223758	0	0	0	0
ENSG00000223759	0	0	0	0
ENSG00000223760	0	0	0	0
ENSG00000223761	0	0	0	0
ENSG00000223764	0	0	0	0
ENSG00000223765	0	0	0	0
ENSG00000223766	0	0	0	0
ENSG00000223767	0	0	0	0
ENSG00000223768	0	0	1	0
ENSG00000223769	0	0	0	0
ENSG00000223770	0	0	3	2
ENSG00000223771	0	0	0	0
ENSG00000223772	0	0	0	0
ENSG00000223773	47	151	111	221
ENSG00000223774	0	0	0	0

ENSG00000223775	0	0	0	0
ENSG00000223776	0	13	0	0
ENSG00000223777	0	0	0	0
ENSG00000223779	0	0	1	10
ENSG00000223780	0	0	0	0
ENSG00000223781	0	0	0	0
ENSG00000223782	0	0	0	0
ENSG00000223783	0	0	0	0
ENSG00000223784	0	0	0	0
ENSG00000223786	0	0	0	0
ENSG00000223787	0	0	0	0
ENSG00000223788	0	0	0	0
ENSG00000223791	0	0	0	0
ENSG00000223793	0	0	0	0
ENSG00000223794	0	0	0	0
ENSG00000223795	0	0	0	0
ENSG00000223797	12	1	0	12
ENSG00000223798	0	0	0	0
ENSG00000223799	1	0	1	0
ENSG00000223800	0	0	0	0
ENSG00000223802	0	0	0	0
ENSG00000223803	0	0	0	0
ENSG00000223804	19	7	22	28
ENSG00000223806	0	0	0	0
ENSG00000223807	0	0	0	0
ENSG00000223808	0	0	0	0
ENSG00000223809	0	0	0	0
ENSG00000223810	0	0	0	0
ENSG00000223811	0	0	0	0
ENSG00000223812	0	0	0	0
ENSG00000223813	0	0	0	0
ENSG00000223814	0	0	0	0
ENSG00000223815	0	0	0	0
ENSG00000223816	0	0	0	0
ENSG00000223817	0	0	0	0
ENSG00000223819	0	0	0	0
ENSG00000223820	0	0	0	0
ENSG00000223821	0	0	0	0
ENSG00000223822	0	0	0	0
ENSG00000223823	0	0	0	0
ENSG00000223824	0	0	0	0
ENSG00000223825	0	0	0	1
ENSG00000223826	0	0	0	0
ENSG00000223827	0	0	0	0
ENSG00000223828	0	0	0	0
ENSG00000223829	0	0	0	0

ENSG00000223831	0	0	0	0
ENSG00000223833	0	0	0	0
ENSG00000223834	0	0	0	0
ENSG00000223836	0	0	0	0
ENSG00000223837	0	0	0	0
ENSG00000223838	0	0	17	31
ENSG00000223839	0	0	0	0
ENSG00000223840	0	0	0	0
ENSG00000223841	0	0	0	0
ENSG00000223842	0	0	0	0
ENSG00000223843	0	0	0	0
ENSG00000223844	0	0	0	0
ENSG00000223845	0	0	0	0
ENSG00000223847	0	0	0	0
ENSG00000223849	0	0	0	0
ENSG00000223850	0	0	0	0
ENSG00000223851	0	0	0	0
ENSG00000223852	0	0	0	0
ENSG00000223853	0	0	0	0
ENSG00000223855	3009	5793	3435	5738
ENSG00000223856	0	0	0	0
ENSG00000223858	0	0	0	0
ENSG00000223859	0	0	0	0
ENSG00000223860	0	0	0	0
ENSG00000223861	0	0	0	0
ENSG00000223863	0	0	1	3
ENSG00000223864	0	0	0	0
ENSG00000223865	375	266	227	152
ENSG00000223866	0	352	72	129
ENSG00000223867	0	0	0	0
ENSG00000223868	0	0	0	0
ENSG00000223869	0	0	0	0
ENSG00000223870	0	0	0	0
ENSG00000223872	0	0	0	0
ENSG00000223873	0	0	0	0
ENSG00000223874	0	0	0	0
ENSG00000223875	0	0	0	5
ENSG00000223876	0	0	0	0
ENSG00000223877	1	0	0	0
ENSG00000223878	0	0	3	0
ENSG00000223880	0	0	0	0
ENSG00000223881	0	0	0	0
ENSG00000223882	0	0	0	0
ENSG00000223883	0	0	0	0
ENSG00000223884	0	0	0	0
ENSG00000223885	0	0	0	0

ENSG00000223886	0	0	0	0
ENSG00000223887	0	0	0	0
ENSG00000223889	0	0	0	0
ENSG00000223890	0	0	0	0
ENSG00000223891	160	113	133	67
ENSG00000223892	0	0	0	0
ENSG00000223893	0	0	0	0
ENSG00000223894	0	0	0	0
ENSG00000223896	0	0	0	0
ENSG00000223897	0	0	0	0
ENSG00000223898	0	0	0	0
ENSG00000223899	0	0	0	0
ENSG00000223901	0	0	0	0
ENSG00000223903	0	0	0	0
ENSG00000223904	0	0	0	0
ENSG00000223905	0	0	0	0
ENSG00000223906	0	0	0	0
ENSG00000223907	0	0	0	0
ENSG00000223908	1	0	0	3
ENSG00000223910	0	0	0	0
ENSG00000223911	0	350	275	319
ENSG00000223912	0	0	1	0
ENSG00000223914	0	0	0	0
ENSG00000223915	0	0	0	0
ENSG00000223916	0	0	0	0
ENSG00000223917	0	0	0	0
ENSG00000223918	0	0	0	0
ENSG00000223919	0	0	0	0
ENSG00000223920	0	0	0	0
ENSG00000223921	0	0	0	0
ENSG00000223922	0	0	0	0
ENSG00000223923	0	0	0	0
ENSG00000223925	0	0	0	0
ENSG00000223927	0	0	0	0
ENSG00000223928	0	21	0	0
ENSG00000223929	0	0	3	2
ENSG00000223930	0	0	0	0
ENSG00000223931	0	0	0	0
ENSG00000223932	0	0	0	0
ENSG00000223933	0	0	0	0
ENSG00000223935	23	0	22	13
ENSG00000223939	0	0	0	0
ENSG00000223940	0	0	0	0
ENSG00000223941	0	0	0	1
ENSG00000223942	0	0	0	0
ENSG00000223944	0	0	0	0

ENSG00000223945	0	0	0	0
ENSG00000223946	1	7	0	1
ENSG00000223947	0	0	0	0
ENSG00000223948	0	0	0	0
ENSG00000223949	0	0	0	0
ENSG00000223951	0	0	0	0
ENSG00000223952	0	0	0	0
ENSG00000223953	0	0	0	0
ENSG00000223955	0	0	0	0
ENSG00000223956	0	0	0	0
ENSG00000223957	0	0	0	0
ENSG00000223958	0	0	0	0
ENSG00000223959	1	0	39	58
ENSG00000223960	0	25	19	9
ENSG00000223962	0	0	0	0
ENSG00000223963	0	0	0	0
ENSG00000223965	0	0	0	0
ENSG00000223966	0	0	0	0
ENSG00000223967	0	0	0	0
ENSG00000223968	0	0	0	0
ENSG00000223969	293	138	48	65
ENSG00000223970	0	0	0	0
ENSG00000223971	0	0	0	0
ENSG00000223972	28	96	123	572
ENSG00000223973	0	0	0	0
ENSG00000223974	0	0	0	0
ENSG00000223975	0	0	0	0
ENSG00000223976	0	0	0	0
ENSG00000223977	0	0	0	0
ENSG00000223978	0	0	0	0
ENSG00000223979	0	0	0	0
ENSG00000223980	0	0	0	0
ENSG00000223981	0	0	0	0
ENSG00000223982	0	0	0	0
ENSG00000223983	0	0	0	0
ENSG00000223984	12	110	148	68
ENSG00000223985	0	0	0	0
ENSG00000223986	0	0	0	0
ENSG00000223987	0	0	0	0
ENSG00000223989	0	0	6	1
ENSG00000223990	0	0	0	0
ENSG00000223991	0	0	0	0
ENSG00000223993	0	0	0	0
ENSG00000223995	0	0	0	0
ENSG00000223996	0	0	0	0
ENSG00000223997	0	0	0	0

ENSG00000223998	0	0	0	7
ENSG00000223999	0	0	0	0
ENSG00000224000	0	0	0	0
ENSG00000224001	0	0	0	0
ENSG00000224002	0	0	0	0
ENSG00000224003	0	0	5	0
ENSG00000224004	0	0	0	0
ENSG00000224005	0	0	0	0
ENSG00000224006	0	0	0	0
ENSG00000224007	0	0	0	0
ENSG00000224008	0	0	0	0
ENSG00000224011	0	0	0	0
ENSG00000224012	0	0	0	0
ENSG00000224014	0	0	0	0
ENSG00000224015	0	0	0	0
ENSG00000224016	0	0	2	4
ENSG00000224017	0	0	0	0
ENSG00000224018	0	0	0	0
ENSG00000224019	0	0	2	0
ENSG00000224020	0	0	0	0
ENSG00000224023	0	0	0	0
ENSG00000224025	0	0	0	0
ENSG00000224027	0	0	0	0
ENSG00000224028	0	0	0	0
ENSG00000224029	0	0	0	0
ENSG00000224031	0	0	0	0
ENSG00000224032	15	62	14	12
ENSG00000224033	0	0	0	0
ENSG00000224034	0	0	7	1
ENSG00000224035	0	0	0	0
ENSG00000224036	0	0	0	0
ENSG00000224037	0	0	0	0
ENSG00000224038	0	0	0	0
ENSG00000224039	0	0	0	0
ENSG00000224040	0	0	0	1
ENSG00000224041	0	0	0	0
ENSG00000224042	0	0	0	0
ENSG00000224043	0	0	7	14
ENSG00000224044	0	0	0	0
ENSG00000224045	0	0	0	0
ENSG00000224046	81	280	131	232
ENSG00000224048	0	0	0	0
ENSG00000224049	0	0	0	0
ENSG00000224050	0	0	0	0
ENSG00000224051	1	30	11	34
ENSG00000224052	0	0	0	0

ENSG00000224053	0	0	0	0
ENSG00000224054	0	0	0	0
ENSG00000224055	0	0	0	0
ENSG00000224056	0	0	0	0
ENSG00000224057	0	0	0	0
ENSG00000224058	0	0	0	0
ENSG00000224059	0	0	0	0
ENSG00000224060	0	0	0	0
ENSG00000224061	0	0	0	0
ENSG00000224062	0	0	0	0
ENSG00000224063	2	374	590	233
ENSG00000224064	0	0	0	0
ENSG00000224065	0	0	0	0
ENSG00000224066	0	0	0	4
ENSG00000224067	0	0	0	0
ENSG00000224069	0	0	0	0
ENSG00000224070	1	0	13	1
ENSG00000224071	0	0	0	0
ENSG00000224072	0	0	0	0
ENSG00000224073	0	0	0	0
ENSG00000224074	0	0	0	0
ENSG00000224075	0	0	0	0
ENSG00000224076	0	0	0	0
ENSG00000224077	0	0	0	0
ENSG00000224078	8	194	251	430
ENSG00000224079	0	0	0	0
ENSG00000224080	1	22	37	19
ENSG00000224081	6	0	0	0
ENSG00000224082	0	0	0	0
ENSG00000224083	0	0	2	0
ENSG00000224085	0	0	0	0
ENSG00000224086	0	0	1	2
ENSG00000224087	0	0	0	0
ENSG00000224089	0	0	0	0
ENSG00000224090	0	0	0	0
ENSG00000224091	4	10	14	10
ENSG00000224092	0	0	0	0
ENSG00000224093	0	12	6	2
ENSG00000224094	0	0	0	0
ENSG00000224095	0	0	0	0
ENSG00000224096	0	0	0	0
ENSG00000224097	0	0	0	0
ENSG00000224098	0	0	0	0
ENSG00000224099	1	0	0	0
ENSG00000224100	0	0	0	0
ENSG00000224101	0	1	8	12

ENSG00000224103	0	0	0	0
ENSG00000224104	0	0	0	0
ENSG00000224105	0	0	0	0
ENSG00000224106	0	0	0	0
ENSG00000224107	0	0	0	0
ENSG00000224108	0	0	0	0
ENSG00000224109	0	0	0	0
ENSG00000224110	0	0	0	0
ENSG00000224113	0	79	22	17
ENSG00000224114	14	0	0	0
ENSG00000224116	20	115	193	193
ENSG00000224117	0	0	0	0
ENSG00000224118	0	0	0	0
ENSG00000224119	0	0	0	0
ENSG00000224120	0	0	0	0
ENSG00000224121	0	0	0	0
ENSG00000224122	0	0	1	0
ENSG00000224124	55	0	0	0
ENSG00000224126	0	0	0	0
ENSG00000224127	0	0	0	0
ENSG00000224128	0	0	0	0
ENSG00000224129	0	0	0	0
ENSG00000224130	0	0	0	0
ENSG00000224131	0	0	0	0
ENSG00000224132	0	0	0	0
ENSG00000224133	0	0	0	0
ENSG00000224134	0	0	0	0
ENSG00000224136	0	0	0	0
ENSG00000224137	1	0	2	0
ENSG00000224138	0	0	0	0
ENSG00000224140	0	0	0	0
ENSG00000224141	0	0	0	0
ENSG00000224142	0	0	0	0
ENSG00000224143	0	0	0	0
ENSG00000224144	0	0	0	0
ENSG00000224145	0	0	0	0
ENSG00000224146	0	0	0	0
ENSG00000224149	0	0	0	0
ENSG00000224151	0	0	0	0
ENSG00000224152	19	93	109	112
ENSG00000224153	0	0	0	0
ENSG00000224155	0	0	0	0
ENSG00000224156	0	0	0	0
ENSG00000224157	7	0	11	2
ENSG00000224158	0	0	0	0
ENSG00000224159	0	0	0	0

ENSG00000224160	0	0	0	0
ENSG00000224161	0	0	0	0
ENSG00000224162	0	0	0	0
ENSG00000224163	0	0	0	0
ENSG00000224164	0	0	0	0
ENSG00000224165	72	16	37	14
ENSG00000224166	0	0	0	0
ENSG00000224167	0	0	0	0
ENSG00000224168	0	0	0	0
ENSG00000224169	0	0	0	0
ENSG00000224172	0	0	0	0
ENSG00000224173	0	0	0	0
ENSG00000224174	0	0	0	0
ENSG00000224177	3	0	1	0
ENSG00000224180	0	0	0	0
ENSG00000224183	0	0	0	0
ENSG00000224184	0	14	0	2
ENSG00000224185	0	0	0	0
ENSG00000224186	1	48	0	1
ENSG00000224187	0	0	0	0
ENSG00000224188	0	0	0	0
ENSG00000224189	0	0	0	0
ENSG00000224190	0	0	0	0
ENSG00000224192	0	0	0	0
ENSG00000224194	0	0	0	2
ENSG00000224195	0	0	4	0
ENSG00000224196	0	0	0	0
ENSG00000224197	0	0	0	0
ENSG00000224198	0	0	0	0
ENSG00000224199	0	0	0	0
ENSG00000224200	0	0	0	0
ENSG00000224201	0	0	0	0
ENSG00000224203	0	0	0	0
ENSG00000224204	0	0	0	0
ENSG00000224205	0	0	0	0
ENSG00000224207	0	0	6	3
ENSG00000224208	0	0	0	0
ENSG00000224209	0	0	0	0
ENSG00000224210	0	0	0	0
ENSG00000224212	0	0	0	0
ENSG00000224215	0	0	0	0
ENSG00000224216	0	12	0	3
ENSG00000224217	0	0	0	0
ENSG00000224218	0	0	0	0
ENSG00000224219	0	0	0	0
ENSG00000224220	0	0	0	0

ENSG00000224221	0	0	0	0
ENSG00000224222	0	0	0	0
ENSG00000224223	0	0	0	0
ENSG00000224224	0	0	0	0
ENSG00000224226	0	0	0	0
ENSG00000224227	4	54	243	61
ENSG00000224228	2	225	103	147
ENSG00000224229	0	0	0	0
ENSG00000224230	0	0	0	0
ENSG00000224231	0	0	0	5
ENSG00000224232	0	0	0	0
ENSG00000224233	0	0	0	0
ENSG00000224234	0	0	0	0
ENSG00000224235	0	0	0	0
ENSG00000224236	0	0	0	0
ENSG00000224237	0	0	0	0
ENSG00000224238	0	0	0	0
ENSG00000224239	0	0	0	0
ENSG00000224240	0	0	0	0
ENSG00000224241	0	0	0	0
ENSG00000224242	0	0	0	0
ENSG00000224243	0	0	0	0
ENSG00000224244	0	0	0	0
ENSG00000224245	0	0	0	0
ENSG00000224246	0	0	0	0
ENSG00000224247	0	0	0	0
ENSG00000224250	0	0	0	0
ENSG00000224251	0	0	0	10
ENSG00000224252	0	0	0	0
ENSG00000224253	0	0	0	0
ENSG00000224254	0	0	0	0
ENSG00000224255	0	0	0	0
ENSG00000224256	0	0	0	0
ENSG00000224257	0	0	0	0
ENSG00000224259	0	0	0	1
ENSG00000224260	0	0	0	0
ENSG00000224261	0	0	0	0
ENSG00000224263	0	0	0	0
ENSG00000224264	0	0	0	0
ENSG00000224265	0	0	0	0
ENSG00000224267	0	0	0	0
ENSG00000224269	0	0	0	0
ENSG00000224271	0	0	0	0
ENSG00000224272	0	0	0	0
ENSG00000224273	1	0	7	2
ENSG00000224274	0	0	0	0

ENSG00000224276	0	0	0	0
ENSG00000224277	0	0	0	0
ENSG00000224278	0	0	0	0
ENSG00000224279	0	0	0	0
ENSG00000224280	0	0	0	0
ENSG00000224281	7	1	39	45
ENSG00000224282	0	0	0	0
ENSG00000224286	0	0	0	2
ENSG00000224287	2	0	3	14
ENSG00000224288	0	0	0	0
ENSG00000224289	0	0	0	0
ENSG00000224290	0	0	0	0
ENSG00000224291	0	0	0	0
ENSG00000224292	0	0	0	0
ENSG00000224293	0	0	0	0
ENSG00000224294	0	0	0	0
ENSG00000224295	0	0	0	0
ENSG00000224296	0	0	0	0
ENSG00000224297	0	0	0	0
ENSG00000224298	0	0	6	0
ENSG00000224299	0	0	0	0
ENSG00000224300	0	0	0	0
ENSG00000224301	0	0	0	0
ENSG00000224302	0	0	0	0
ENSG00000224303	0	0	0	0
ENSG00000224305	0	0	0	0
ENSG00000224307	1	0	0	0
ENSG00000224308	0	0	0	0
ENSG00000224309	0	0	0	0
ENSG00000224310	0	0	0	0
ENSG00000224311	0	0	0	0
ENSG00000224312	0	0	0	0
ENSG00000224313	0	0	0	0
ENSG00000224314	0	0	0	0
ENSG00000224315	0	0	1	4
ENSG00000224316	0	0	12	18
ENSG00000224318	0	0	0	0
ENSG00000224319	0	0	0	0
ENSG00000224320	0	0	0	0
ENSG00000224321	0	0	0	0
ENSG00000224322	0	0	0	0
ENSG00000224323	0	0	0	0
ENSG00000224324	0	0	0	0
ENSG00000224326	0	0	0	0
ENSG00000224328	0	0	0	0
ENSG00000224329	0	0	0	0

ENSG00000224330	0	0	0	0
ENSG00000224331	0	0	0	0
ENSG00000224333	0	0	0	0
ENSG00000224334	0	0	1	0
ENSG00000224335	1	0	0	0
ENSG00000224336	0	0	0	0
ENSG00000224337	0	0	0	0
ENSG00000224338	0	0	0	0
ENSG00000224339	0	0	0	0
ENSG00000224340	0	0	0	0
ENSG00000224341	0	0	0	0
ENSG00000224342	1	0	2	0
ENSG00000224344	0	0	0	0
ENSG00000224345	6	49	29	7
ENSG00000224346	0	0	0	0
ENSG00000224347	0	0	0	0
ENSG00000224348	0	0	0	0
ENSG00000224349	0	0	0	0
ENSG00000224351	0	0	0	0
ENSG00000224352	0	0	0	0
ENSG00000224353	0	0	0	0
ENSG00000224354	0	0	0	0
ENSG00000224355	0	0	0	0
ENSG00000224356	5	0	0	0
ENSG00000224357	0	0	0	0
ENSG00000224358	0	0	0	0
ENSG00000224359	0	0	0	0
ENSG00000224360	0	0	0	0
ENSG00000224361	0	0	0	0
ENSG00000224362	0	0	0	0
ENSG00000224363	0	0	0	0
ENSG00000224365	0	0	6	0
ENSG00000224366	0	0	0	0
ENSG00000224367	0	0	0	0
ENSG00000224368	0	0	0	0
ENSG00000224370	0	0	0	0
ENSG00000224371	0	0	0	0
ENSG00000224372	0	0	0	0
ENSG00000224373	1	0	0	0
ENSG00000224374	0	0	3	5
ENSG00000224375	0	0	0	0
ENSG00000224376	3	0	0	0
ENSG00000224378	0	0	0	0
ENSG00000224379	0	0	0	0
ENSG00000224382	0	0	0	0
ENSG00000224383	32	115	65	10

ENSG00000224384	0	0	0	0
ENSG00000224387	0	0	0	0
ENSG00000224388	0	0	0	0
ENSG00000224389	0	0	0	0
ENSG00000224391	0	0	0	0
ENSG00000224392	0	0	0	0
ENSG00000224393	0	0	0	0
ENSG00000224394	0	0	0	0
ENSG00000224395	0	0	0	0
ENSG00000224396	0	0	0	0
ENSG00000224397	132	0	1	0
ENSG00000224398	0	0	0	0
ENSG00000224399	0	0	0	0
ENSG00000224400	0	0	0	0
ENSG00000224401	0	0	0	0
ENSG00000224402	0	0	0	0
ENSG00000224403	0	0	0	0
ENSG00000224404	0	0	0	0
ENSG00000224405	0	0	0	0
ENSG00000224406	0	0	0	3
ENSG00000224407	0	0	0	0
ENSG00000224408	0	0	0	0
ENSG00000224409	0	0	0	0
ENSG00000224410	0	0	0	0
ENSG00000224411	1	1	2	0
ENSG00000224412	0	0	0	0
ENSG00000224413	0	0	0	0
ENSG00000224414	0	0	0	0
ENSG00000224415	0	0	0	2
ENSG00000224416	0	0	0	0
ENSG00000224417	0	0	0	0
ENSG00000224418	1	12	11	17
ENSG00000224419	0	0	0	0
ENSG00000224420	0	0	0	0
ENSG00000224421	0	0	0	0
ENSG00000224424	0	0	0	1
ENSG00000224425	0	0	0	0
ENSG00000224426	0	0	0	0
ENSG00000224427	0	0	0	0
ENSG00000224429	2	0	0	0
ENSG00000224430	0	0	3	0
ENSG00000224431	0	0	2	0
ENSG00000224435	0	0	0	0
ENSG00000224436	0	0	0	0
ENSG00000224437	0	0	0	0
ENSG00000224438	0	0	0	0

ENSG00000224439	0	0	0	0
ENSG00000224440	0	0	0	0
ENSG00000224441	0	0	0	0
ENSG00000224442	0	0	0	0
ENSG00000224443	0	0	0	0
ENSG00000224444	0	0	0	0
ENSG00000224445	0	0	3	7
ENSG00000224446	0	0	0	0
ENSG00000224447	0	0	0	0
ENSG00000224448	0	0	0	0
ENSG00000224450	0	0	0	0
ENSG00000224451	0	0	0	0
ENSG00000224452	0	0	0	0
ENSG00000224455	0	0	0	0
ENSG00000224456	0	0	0	0
ENSG00000224458	0	0	0	0
ENSG00000224459	0	0	0	0
ENSG00000224460	0	0	7	0
ENSG00000224462	0	0	0	0
ENSG00000224463	0	0	0	0
ENSG00000224464	0	0	0	0
ENSG00000224465	0	0	0	0
ENSG00000224466	0	0	0	0
ENSG00000224467	0	0	0	0
ENSG00000224468	0	0	0	0
ENSG00000224469	0	0	0	0
ENSG00000224470	20	252	215	189
ENSG00000224471	0	0	0	0
ENSG00000224472	0	0	0	0
ENSG00000224473	0	0	0	0
ENSG00000224474	0	0	0	0
ENSG00000224475	0	0	0	0
ENSG00000224476	0	0	0	0
ENSG00000224477	0	0	0	0
ENSG00000224478	0	0	0	0
ENSG00000224479	0	0	4	1
ENSG00000224481	0	0	0	0
ENSG00000224482	1	0	0	0
ENSG00000224484	0	0	0	0
ENSG00000224485	0	0	0	0
ENSG00000224486	0	0	0	0
ENSG00000224487	0	0	0	0
ENSG00000224488	0	0	0	0
ENSG00000224490	0	11	0	0
ENSG00000224493	0	0	0	0
ENSG00000224494	0	0	0	0

ENSG00000224496	0	0	0	0
ENSG00000224497	0	0	0	0
ENSG00000224498	0	0	0	0
ENSG00000224500	0	0	0	0
ENSG00000224501	0	0	0	0
ENSG00000224503	0	0	0	0
ENSG00000224504	0	0	0	0
ENSG00000224505	3	34	35	24
ENSG00000224506	0	0	0	0
ENSG00000224507	0	0	0	0
ENSG00000224508	0	0	0	0
ENSG00000224509	0	0	0	0
ENSG00000224510	0	0	0	0
ENSG00000224511	0	0	0	0
ENSG00000224512	0	0	0	0
ENSG00000224513	0	0	0	0
ENSG00000224514	0	0	0	0
ENSG00000224515	0	0	0	0
ENSG00000224516	0	0	0	0
ENSG00000224517	0	0	0	0
ENSG00000224518	0	0	0	0
ENSG00000224519	0	0	0	0
ENSG00000224520	0	0	0	0
ENSG00000224521	0	0	0	1
ENSG00000224523	0	48	28	46
ENSG00000224524	0	0	0	0
ENSG00000224525	3	0	2	13
ENSG00000224526	0	0	0	0
ENSG00000224529	0	0	0	0
ENSG00000224530	0	0	0	0
ENSG00000224531	0	72	9	13
ENSG00000224532	0	0	1	0
ENSG00000224533	0	0	15	13
ENSG00000224535	0	0	0	0
ENSG00000224536	0	0	0	0
ENSG00000224537	0	0	0	0
ENSG00000224538	0	0	0	0
ENSG00000224539	0	0	0	0
ENSG00000224540	0	0	0	0
ENSG00000224541	3	0	0	0
ENSG00000224542	0	0	0	0
ENSG00000224543	0	0	0	0
ENSG00000224544	0	0	0	0
ENSG00000224545	0	0	0	0
ENSG00000224546	0	0	0	0
ENSG00000224547	0	0	0	0

ENSG00000224548	0	0	0	0
ENSG00000224549	0	0	0	0
ENSG00000224550	0	0	0	0
ENSG00000224551	0	0	0	0
ENSG00000224552	0	0	0	0
ENSG00000224553	5	0	4	1
ENSG00000224554	0	0	0	0
ENSG00000224555	0	0	0	0
ENSG00000224556	0	0	0	0
ENSG00000224557	1	0	0	0
ENSG00000224559	0	0	0	0
ENSG00000224560	0	0	0	0
ENSG00000224563	0	0	3	0
ENSG00000224564	0	0	0	0
ENSG00000224565	3	0	4	4
ENSG00000224566	0	0	0	0
ENSG00000224567	0	0	0	0
ENSG00000224568	0	0	0	0
ENSG00000224569	0	0	3	0
ENSG00000224570	0	0	0	0
ENSG00000224571	0	0	0	0
ENSG00000224573	0	0	0	0
ENSG00000224574	0	0	0	0
ENSG00000224577	0	0	0	0
ENSG00000224578	3	3	3	3
ENSG00000224579	0	0	0	0
ENSG00000224581	0	0	0	0
ENSG00000224582	0	0	0	0
ENSG00000224583	0	0	0	0
ENSG00000224584	0	0	0	0
ENSG00000224585	0	0	0	0
ENSG00000224586	0	0	0	0
ENSG00000224587	0	0	0	0
ENSG00000224589	0	0	0	0
ENSG00000224590	0	0	0	0
ENSG00000224592	0	0	0	0
ENSG00000224593	0	0	0	0
ENSG00000224594	0	0	0	0
ENSG00000224595	0	0	0	0
ENSG00000224596	0	22	1	3
ENSG00000224597	43	19	67	151
ENSG00000224598	0	0	0	0
ENSG00000224599	0	0	0	1
ENSG00000224600	0	0	0	0
ENSG00000224602	0	0	0	0
ENSG00000224603	0	0	0	0

ENSG00000224604	0	0	0	0
ENSG00000224605	0	0	0	0
ENSG00000224606	0	0	0	0
ENSG00000224607	0	0	0	0
ENSG00000224608	0	0	0	0
ENSG00000224609	0	0	0	0
ENSG00000224610	0	0	0	0
ENSG00000224611	0	0	0	0
ENSG00000224612	0	0	0	0
ENSG00000224613	0	0	1	0
ENSG00000224614	24	35	17	16
ENSG00000224615	0	0	0	0
ENSG00000224616	64	447	328	347
ENSG00000224617	0	0	0	0
ENSG00000224618	0	0	0	0
ENSG00000224619	0	0	0	0
ENSG00000224620	0	0	0	0
ENSG00000224621	0	0	0	0
ENSG00000224622	0	0	0	0
ENSG00000224623	0	0	0	0
ENSG00000224625	0	0	0	0
ENSG00000224626	0	0	0	0
ENSG00000224627	0	0	0	0
ENSG00000224628	0	0	0	0
ENSG00000224629	0	0	0	0
ENSG00000224630	0	0	0	0
ENSG00000224631	7	0	0	0
ENSG00000224632	0	0	0	0
ENSG00000224633	0	0	0	0
ENSG00000224634	0	0	0	0
ENSG00000224635	0	0	0	0
ENSG00000224637	0	0	0	0
ENSG00000224638	0	0	0	0
ENSG00000224639	0	0	0	0
ENSG00000224640	0	0	0	0
ENSG00000224641	0	0	0	0
ENSG00000224642	0	0	0	0
ENSG00000224643	3	11	32	3
ENSG00000224644	0	0	0	0
ENSG00000224645	0	0	0	0
ENSG00000224646	0	0	0	0
ENSG00000224647	0	0	0	0
ENSG00000224648	0	0	0	0
ENSG00000224649	13	46	2	0
ENSG00000224650	0	0	0	0
ENSG00000224652	0	0	0	0

ENSG00000224653	0	0	0	0
ENSG00000224654	0	0	0	0
ENSG00000224655	0	0	0	0
ENSG00000224656	0	0	0	0
ENSG00000224657	0	0	0	0
ENSG00000224658	0	0	0	0
ENSG00000224659	0	0	0	0
ENSG00000224660	7	35	13	23
ENSG00000224661	0	0	0	0
ENSG00000224662	0	0	0	0
ENSG00000224664	0	0	0	0
ENSG00000224666	0	0	0	0
ENSG00000224667	0	0	0	0
ENSG00000224668	0	0	0	0
ENSG00000224669	0	0	0	0
ENSG00000224670	0	0	0	0
ENSG00000224671	0	0	0	0
ENSG00000224672	0	0	1	0
ENSG00000224674	0	0	0	0
ENSG00000224675	0	0	0	0
ENSG00000224676	0	0	0	0
ENSG00000224677	0	0	0	0
ENSG00000224678	0	0	0	0
ENSG00000224679	0	0	0	0
ENSG00000224680	0	1	0	0
ENSG00000224681	0	0	0	0
ENSG00000224682	0	0	0	0
ENSG00000224683	0	0	0	0
ENSG00000224685	0	0	0	0
ENSG00000224686	0	0	0	0
ENSG00000224687	5	0	3	0
ENSG00000224688	0	0	0	0
ENSG00000224689	0	0	0	0
ENSG00000224690	0	0	0	0
ENSG00000224691	0	0	0	0
ENSG00000224692	0	0	0	0
ENSG00000224693	0	0	0	0
ENSG00000224695	0	0	0	0
ENSG00000224697	0	0	0	0
ENSG00000224698	0	0	0	0
ENSG00000224699	0	0	5	1
ENSG00000224700	0	0	0	0
ENSG00000224701	0	0	0	0
ENSG00000224702	0	0	0	0
ENSG00000224705	0	0	0	0
ENSG00000224706	0	0	0	0

ENSG00000224707	0	0	2	0
ENSG00000224708	0	0	0	0
ENSG00000224709	0	0	0	0
ENSG00000224710	0	0	0	0
ENSG00000224711	0	0	0	0
ENSG00000224712	0	0	0	0
ENSG00000224713	5	0	0	0
ENSG00000224714	0	0	0	0
ENSG00000224715	0	0	0	0
ENSG00000224717	0	0	0	0
ENSG00000224718	0	0	0	0
ENSG00000224719	0	0	0	0
ENSG00000224721	0	0	0	0
ENSG00000224722	0	0	0	0
ENSG00000224723	0	0	0	0
ENSG00000224725	0	0	0	0
ENSG00000224727	0	0	0	0
ENSG00000224728	0	0	0	0
ENSG00000224729	0	0	0	0
ENSG00000224730	0	0	0	0
ENSG00000224731	0	0	0	0
ENSG00000224732	0	0	0	0
ENSG00000224733	0	0	0	0
ENSG00000224735	0	0	0	0
ENSG00000224736	0	0	0	0
ENSG00000224738	1	0	0	0
ENSG00000224739	0	0	0	0
ENSG00000224740	0	0	0	0
ENSG00000224743	0	0	0	0
ENSG00000224744	0	0	0	0
ENSG00000224745	0	1028	940	1891
ENSG00000224746	5	22	31	15
ENSG00000224747	0	0	0	0
ENSG00000224748	0	0	0	0
ENSG00000224750	0	0	0	0
ENSG00000224751	0	0	0	0
ENSG00000224752	0	0	0	0
ENSG00000224753	0	0	0	0
ENSG00000224755	0	0	0	0
ENSG00000224758	0	0	0	0
ENSG00000224760	0	0	0	0
ENSG00000224761	0	8	6	15
ENSG00000224762	0	0	0	0
ENSG00000224763	0	0	0	0
ENSG00000224764	0	0	0	0
ENSG00000224765	0	0	0	0

ENSG00000224766	0	0	0	0
ENSG00000224769	0	0	0	0
ENSG00000224770	0	0	0	0
ENSG00000224771	0	0	0	0
ENSG00000224773	0	0	0	0
ENSG00000224774	0	0	0	0
ENSG00000224775	0	0	0	1
ENSG00000224776	1	0	3	0
ENSG00000224777	0	0	0	0
ENSG00000224778	0	0	0	0
ENSG00000224781	0	0	0	0
ENSG00000224782	0	0	0	0
ENSG00000224783	0	0	0	0
ENSG00000224784	0	0	0	0
ENSG00000224785	0	0	0	0
ENSG00000224786	5	22	15	40
ENSG00000224788	0	0	0	0
ENSG00000224789	0	0	1	0
ENSG00000224790	0	0	0	0
ENSG00000224791	0	0	0	0
ENSG00000224792	0	0	0	0
ENSG00000224793	0	0	0	0
ENSG00000224794	0	0	1	0
ENSG00000224795	0	0	0	0
ENSG00000224796	1	0	0	0
ENSG00000224797	0	0	0	0
ENSG00000224799	1	0	0	1
ENSG00000224800	0	0	0	0
ENSG00000224801	0	0	0	0
ENSG00000224802	0	0	0	0
ENSG00000224804	0	0	0	0
ENSG00000224805	3445	1253	963	1272
ENSG00000224806	0	0	0	0
ENSG00000224807	0	0	0	0
ENSG00000224808	0	0	0	0
ENSG00000224809	1	0	0	0
ENSG00000224810	1	0	1	0
ENSG00000224812	0	0	0	0
ENSG00000224813	0	0	0	0
ENSG00000224814	0	0	0	0
ENSG00000224815	0	0	0	0
ENSG00000224816	0	0	0	0
ENSG00000224817	0	0	0	0
ENSG00000224818	0	0	0	0
ENSG00000224819	0	0	0	0
ENSG00000224820	0	0	0	0

ENSG00000224821	0	0	0	0
ENSG00000224822	0	0	2	0
ENSG00000224825	0	0	0	0
ENSG00000224826	0	16	1	0
ENSG00000224827	0	0	0	0
ENSG00000224828	0	0	0	0
ENSG00000224829	0	0	0	0
ENSG00000224830	0	0	0	0
ENSG00000224831	0	0	7	7
ENSG00000224832	0	0	0	0
ENSG00000224834	0	0	0	0
ENSG00000224835	0	0	0	0
ENSG00000224836	0	0	0	0
ENSG00000224837	0	0	0	0
ENSG00000224838	4	0	0	0
ENSG00000224839	0	0	0	0
ENSG00000224841	0	0	0	0
ENSG00000224842	0	0	0	0
ENSG00000224843	0	0	0	0
ENSG00000224844	0	0	0	0
ENSG00000224846	1	946	793	2082
ENSG00000224848	3	23	39	40
ENSG00000224849	0	0	0	0
ENSG00000224850	1	0	0	4
ENSG00000224851	0	0	0	0
ENSG00000224852	0	0	0	0
ENSG00000224853	0	0	0	0
ENSG00000224854	0	0	0	0
ENSG00000224855	7	1	20	41
ENSG00000224856	0	0	0	0
ENSG00000224857	0	0	0	0
ENSG00000224858	1	0	0	0
ENSG00000224859	0	0	0	0
ENSG00000224860	0	0	0	0
ENSG00000224861	0	0	0	2
ENSG00000224862	0	0	0	0
ENSG00000224863	0	0	0	0
ENSG00000224864	0	0	0	0
ENSG00000224865	0	0	0	0
ENSG00000224866	0	0	0	0
ENSG00000224867	0	0	0	0
ENSG00000224869	0	0	0	0
ENSG00000224870	7	19	2	9
ENSG00000224871	0	0	0	0
ENSG00000224873	0	0	0	0
ENSG00000224874	0	0	0	0

ENSG00000224875	0	0	0	0
ENSG00000224876	0	0	0	0
ENSG00000224877	16	0	7	2
ENSG00000224879	0	0	0	0
ENSG00000224880	0	0	0	0
ENSG00000224881	0	0	1	0
ENSG00000224882	0	0	0	3
ENSG00000224883	0	0	0	0
ENSG00000224884	0	0	0	0
ENSG00000224885	0	0	1	3
ENSG00000224886	0	0	0	0
ENSG00000224887	0	0	0	0
ENSG00000224888	0	0	0	0
ENSG00000224889	0	0	0	0
ENSG00000224891	0	0	1	0
ENSG00000224892	5	57	29	41
ENSG00000224893	0	0	0	0
ENSG00000224894	0	0	0	0
ENSG00000224895	0	0	0	0
ENSG00000224896	0	0	0	0
ENSG00000224897	65	490	240	411
ENSG00000224899	0	0	0	0
ENSG00000224901	0	0	0	0
ENSG00000224902	0	0	0	0
ENSG00000224903	0	0	1	0
ENSG00000224904	0	0	0	0
ENSG00000224905	0	0	0	9
ENSG00000224906	1	0	0	0
ENSG00000224907	0	0	0	0
ENSG00000224908	0	0	0	0
ENSG00000224910	0	0	0	0
ENSG00000224911	0	0	0	0
ENSG00000224914	2	124	132	75
ENSG00000224916	0	0	0	0
ENSG00000224917	0	0	0	0
ENSG00000224918	0	0	0	0
ENSG00000224919	0	0	0	0
ENSG00000224920	0	0	0	0
ENSG00000224921	0	0	0	0
ENSG00000224922	0	0	0	0
ENSG00000224923	0	0	0	0
ENSG00000224924	0	0	0	0
ENSG00000224925	0	0	0	0
ENSG00000224927	0	0	15	4
ENSG00000224928	0	0	0	0
ENSG00000224929	0	0	0	0

ENSG00000224930	0	0	0	0
ENSG00000224931	0	0	0	0
ENSG00000224932	0	0	0	0
ENSG00000224933	0	0	0	0
ENSG00000224934	0	12	1	0
ENSG00000224935	0	0	0	0
ENSG00000224936	0	0	0	0
ENSG00000224937	0	0	0	0
ENSG00000224939	0	0	0	0
ENSG00000224940	0	0	0	0
ENSG00000224941	0	0	0	0
ENSG00000224942	0	0	0	0
ENSG00000224943	0	0	0	0
ENSG00000224944	0	0	0	0
ENSG00000224945	0	0	0	0
ENSG00000224946	0	0	0	0
ENSG00000224947	0	0	0	0
ENSG00000224948	0	0	0	0
ENSG00000224949	0	0	0	0
ENSG00000224950	1	0	0	2
ENSG00000224952	0	0	0	0
ENSG00000224953	0	0	0	0
ENSG00000224954	0	0	0	0
ENSG00000224955	0	0	0	0
ENSG00000224956	0	0	0	0
ENSG00000224957	0	0	0	0
ENSG00000224958	0	0	0	0
ENSG00000224959	27	42	14	20
ENSG00000224960	0	0	0	0
ENSG00000224961	0	0	0	8
ENSG00000224962	0	0	0	0
ENSG00000224963	0	0	0	0
ENSG00000224964	0	0	0	0
ENSG00000224965	2	0	0	0
ENSG00000224966	0	0	0	0
ENSG00000224967	0	5	0	0
ENSG00000224968	0	0	0	0
ENSG00000224969	0	0	0	0
ENSG00000224970	0	0	0	0
ENSG00000224971	0	0	0	0
ENSG00000224972	0	0	3	0
ENSG00000224973	0	0	0	0
ENSG00000224975	28	0	8	11
ENSG00000224976	0	0	0	0
ENSG00000224977	0	0	0	0
ENSG00000224978	0	0	0	0

ENSG00000224979	0	0	0	0
ENSG00000224980	0	0	0	0
ENSG00000224981	1	0	0	0
ENSG00000224982	5	14	6	1
ENSG00000224984	0	0	0	0
ENSG00000224985	0	0	1	0
ENSG00000224986	0	0	0	0
ENSG00000224987	0	0	0	0
ENSG00000224988	2	0	1	2
ENSG00000224989	0	0	0	0
ENSG00000224990	0	0	0	0
ENSG00000224992	0	0	1	0
ENSG00000224993	0	0	0	0
ENSG00000224994	0	0	0	0
ENSG00000224995	0	0	0	0
ENSG00000224997	0	0	0	0
ENSG00000224999	0	0	0	0
ENSG00000225000	0	0	0	0
ENSG00000225002	0	0	0	2
ENSG00000225003	0	0	0	0
ENSG00000225005	0	0	0	0
ENSG00000225006	0	0	0	0
ENSG00000225007	0	0	0	0
ENSG00000225008	0	0	0	0
ENSG00000225009	0	0	0	0
ENSG00000225010	0	0	0	0
ENSG00000225011	0	0	0	0
ENSG00000225012	0	0	0	0
ENSG00000225014	0	30	1	14
ENSG00000225015	0	0	0	0
ENSG00000225016	0	0	0	0
ENSG00000225017	0	0	0	0
ENSG00000225018	0	0	0	0
ENSG00000225022	0	1	0	0
ENSG00000225023	0	0	0	0
ENSG00000225024	0	0	0	0
ENSG00000225025	0	0	0	0
ENSG00000225026	0	0	0	0
ENSG00000225027	0	0	0	0
ENSG00000225028	0	0	0	0
ENSG00000225029	0	0	0	0
ENSG00000225030	0	0	0	0
ENSG00000225031	0	0	1	2
ENSG00000225032	0	0	0	0
ENSG00000225034	0	0	0	0
ENSG00000225036	0	0	0	0

ENSG00000225037	0	0	0	0
ENSG00000225038	0	0	0	0
ENSG00000225039	0	0	0	0
ENSG00000225043	0	0	0	0
ENSG00000225044	0	0	0	0
ENSG00000225045	0	0	0	0
ENSG00000225046	0	0	0	0
ENSG00000225048	0	0	0	0
ENSG00000225049	0	0	0	0
ENSG00000225050	0	0	0	0
ENSG00000225051	0	0	13	17
ENSG00000225053	0	0	0	0
ENSG00000225055	0	0	0	0
ENSG00000225056	0	0	0	0
ENSG00000225057	0	17	0	0
ENSG00000225058	0	0	0	0
ENSG00000225059	0	0	0	0
ENSG00000225060	0	0	0	0
ENSG00000225062	0	0	0	0
ENSG00000225063	0	0	0	0
ENSG00000225064	0	0	0	0
ENSG00000225066	0	0	0	0
ENSG00000225067	0	0	0	0
ENSG00000225069	0	0	0	0
ENSG00000225070	0	0	0	0
ENSG00000225071	1	0	0	0
ENSG00000225072	2	0	0	9
ENSG00000225073	0	0	0	0
ENSG00000225075	0	0	0	0
ENSG00000225076	0	0	0	0
ENSG00000225077	0	0	0	0
ENSG00000225078	0	0	0	0
ENSG00000225079	0	0	0	0
ENSG00000225080	0	0	0	0
ENSG00000225082	0	0	0	0
ENSG00000225083	0	0	0	0
ENSG00000225084	0	0	0	0
ENSG00000225085	0	0	0	0
ENSG00000225086	0	0	0	0
ENSG00000225087	0	0	0	6
ENSG00000225091	0	0	0	0
ENSG00000225092	0	7	0	0
ENSG00000225093	0	0	0	0
ENSG00000225094	0	0	0	0
ENSG00000225096	0	0	0	0
ENSG00000225098	0	0	0	0

ENSG00000225099	0	0	0	0
ENSG00000225100	0	0	0	0
ENSG00000225101	0	0	0	0
ENSG00000225102	0	0	0	0
ENSG00000225103	0	0	0	0
ENSG00000225105	0	0	0	0
ENSG00000225106	0	0	0	0
ENSG00000225107	0	0	0	0
ENSG00000225108	0	0	0	0
ENSG00000225109	0	0	0	0
ENSG00000225110	0	0	0	0
ENSG00000225111	0	0	0	0
ENSG00000225112	0	0	0	0
ENSG00000225113	1	60	2	4
ENSG00000225114	0	0	0	0
ENSG00000225116	0	0	0	0
ENSG00000225117	0	0	0	0
ENSG00000225118	0	0	0	0
ENSG00000225119	0	166	100	97
ENSG00000225120	0	0	0	0
ENSG00000225121	0	0	0	0
ENSG00000225122	0	0	0	0
ENSG00000225123	0	0	0	0
ENSG00000225124	0	0	0	0
ENSG00000225125	0	0	2	2
ENSG00000225126	0	0	0	0
ENSG00000225127	0	0	0	0
ENSG00000225128	0	26	3	2
ENSG00000225129	0	0	0	0
ENSG00000225130	0	0	0	0
ENSG00000225131	0	0	0	0
ENSG00000225132	0	0	0	0
ENSG00000225133	0	0	0	0
ENSG00000225135	11	71	126	132
ENSG00000225136	0	0	0	0
ENSG00000225137	0	0	1	5
ENSG00000225138	0	16	0	0
ENSG00000225140	0	0	0	0
ENSG00000225141	0	0	0	0
ENSG00000225142	0	0	0	0
ENSG00000225144	0	0	0	0
ENSG00000225145	0	0	0	0
ENSG00000225146	0	0	0	0
ENSG00000225147	0	0	0	0
ENSG00000225148	0	0	0	0
ENSG00000225150	0	0	0	0

ENSG00000225151	1	0	0	0
ENSG00000225152	0	0	0	0
ENSG00000225154	0	0	0	0
ENSG00000225155	0	0	0	0
ENSG00000225156	0	0	0	0
ENSG00000225157	0	0	0	0
ENSG00000225158	0	0	0	0
ENSG00000225159	0	0	0	1
ENSG00000225163	1	0	0	1
ENSG00000225164	0	0	0	0
ENSG00000225165	0	0	0	0
ENSG00000225166	0	0	0	0
ENSG00000225167	0	0	1	0
ENSG00000225169	0	0	0	0
ENSG00000225170	0	0	0	0
ENSG00000225171	0	0	0	1
ENSG00000225172	0	0	0	0
ENSG00000225173	0	0	5	0
ENSG00000225174	0	0	0	0
ENSG00000225175	0	0	0	0
ENSG00000225176	0	0	0	0
ENSG00000225177	1	0	6	0
ENSG00000225178	0	0	0	0
ENSG00000225179	0	0	1	0
ENSG00000225180	0	0	0	0
ENSG00000225181	0	0	0	0
ENSG00000225182	0	0	0	0
ENSG00000225183	0	0	1	6
ENSG00000225184	0	0	0	0
ENSG00000225185	0	0	0	0
ENSG00000225187	0	0	0	0
ENSG00000225188	0	0	0	0
ENSG00000225189	0	0	0	0
ENSG00000225190	6	29	39	109
ENSG00000225191	0	0	0	0
ENSG00000225192	0	0	0	0
ENSG00000225193	0	0	0	0
ENSG00000225194	0	0	0	0
ENSG00000225195	0	0	0	0
ENSG00000225196	0	0	0	0
ENSG00000225198	0	0	0	0
ENSG00000225200	0	0	0	0
ENSG00000225201	0	0	0	0
ENSG00000225203	0	0	0	0
ENSG00000225205	370	1920	1911	2467
ENSG00000225206	0	0	0	0

ENSG00000225208	0	0	0	0
ENSG00000225209	0	0	0	0
ENSG00000225210	0	0	3	19
ENSG00000225211	0	0	0	0
ENSG00000225212	0	0	0	0
ENSG00000225213	0	0	0	0
ENSG00000225214	0	0	0	0
ENSG00000225215	0	0	0	0
ENSG00000225216	0	0	0	0
ENSG00000225217	0	0	0	0
ENSG00000225218	0	0	0	0
ENSG00000225221	0	0	0	0
ENSG00000225222	0	0	0	0
ENSG00000225224	0	0	0	0
ENSG00000225225	1	0	0	0
ENSG00000225226	0	0	0	0
ENSG00000225228	0	0	0	0
ENSG00000225230	0	0	0	0
ENSG00000225231	3	35	307	15
ENSG00000225233	0	0	0	0
ENSG00000225234	0	0	0	0
ENSG00000225235	0	0	0	0
ENSG00000225236	0	0	3	7
ENSG00000225237	0	0	0	0
ENSG00000225238	0	0	0	0
ENSG00000225239	0	0	0	0
ENSG00000225240	0	0	0	0
ENSG00000225241	0	10	2	18
ENSG00000225242	0	0	0	0
ENSG00000225243	0	0	0	0
ENSG00000225244	0	0	0	0
ENSG00000225246	0	0	0	0
ENSG00000225247	0	0	0	0
ENSG00000225248	0	0	0	0
ENSG00000225249	0	0	0	0
ENSG00000225251	0	0	0	0
ENSG00000225253	0	0	0	0
ENSG00000225254	0	0	0	0
ENSG00000225255	0	0	0	0
ENSG00000225256	0	0	0	0
ENSG00000225258	0	0	0	0
ENSG00000225259	0	2	54	47
ENSG00000225261	1	0	0	0
ENSG00000225263	0	0	0	0
ENSG00000225264	0	0	0	1
ENSG00000225265	0	0	0	2

ENSG00000225267	0	0	0	0
ENSG00000225269	0	0	0	0
ENSG00000225270	0	0	0	0
ENSG00000225271	0	0	0	0
ENSG00000225272	0	0	0	0
ENSG00000225273	0	0	0	0
ENSG00000225274	0	0	0	0
ENSG00000225275	0	0	0	0
ENSG00000225276	0	0	0	0
ENSG00000225278	0	0	10	26
ENSG00000225279	0	0	0	0
ENSG00000225280	0	0	0	0
ENSG00000225282	0	0	0	0
ENSG00000225284	1	0	0	0
ENSG00000225285	0	0	0	0
ENSG00000225286	0	0	0	0
ENSG00000225287	0	0	0	0
ENSG00000225289	0	0	0	0
ENSG00000225291	0	0	0	0
ENSG00000225292	0	21	4	8
ENSG00000225293	0	0	0	0
ENSG00000225294	0	0	0	0
ENSG00000225295	0	0	0	0
ENSG00000225297	0	0	0	0
ENSG00000225298	0	0	0	0
ENSG00000225299	0	0	0	0
ENSG00000225300	1	0	1	0
ENSG00000225302	0	0	0	0
ENSG00000225303	0	0	0	0
ENSG00000225304	0	0	0	0
ENSG00000225305	0	0	0	0
ENSG00000225306	0	0	0	0
ENSG00000225308	0	0	0	0
ENSG00000225310	0	0	0	0
ENSG00000225311	0	0	0	0
ENSG00000225312	0	0	0	0
ENSG00000225313	0	0	0	0
ENSG00000225314	0	0	0	0
ENSG00000225315	9	0	0	3
ENSG00000225316	0	0	0	0
ENSG00000225317	0	0	0	0
ENSG00000225321	0	0	0	0
ENSG00000225322	0	0	0	0
ENSG00000225323	0	0	0	0
ENSG00000225325	0	0	3	0
ENSG00000225326	0	0	0	0

ENSG00000225327	0	0	0	0
ENSG00000225328	1	0	4	5
ENSG00000225329	0	0	0	0
ENSG00000225330	0	0	0	0
ENSG00000225331	1	0	0	0
ENSG00000225332	0	0	0	0
ENSG00000225333	0	0	0	0
ENSG00000225334	0	0	0	0
ENSG00000225335	2	0	10	10
ENSG00000225336	0	0	0	0
ENSG00000225337	0	0	0	0
ENSG00000225338	0	0	0	0
ENSG00000225339	333	56	108	58
ENSG00000225341	0	0	0	0
ENSG00000225342	0	0	0	0
ENSG00000225343	0	0	1	0
ENSG00000225344	0	0	0	0
ENSG00000225345	0	0	0	0
ENSG00000225346	0	0	0	0
ENSG00000225347	0	0	0	0
ENSG00000225349	0	0	0	0
ENSG00000225350	0	0	0	0
ENSG00000225351	0	0	0	0
ENSG00000225352	0	0	0	0
ENSG00000225353	0	0	0	0
ENSG00000225354	0	0	0	0
ENSG00000225355	0	0	0	0
ENSG00000225356	0	0	0	0
ENSG00000225357	0	0	0	0
ENSG00000225358	0	0	0	0
ENSG00000225359	0	0	0	0
ENSG00000225360	0	0	0	0
ENSG00000225361	1	0	1	4
ENSG00000225362	0	0	0	0
ENSG00000225364	0	0	0	0
ENSG00000225365	0	0	0	0
ENSG00000225366	0	0	0	0
ENSG00000225368	0	0	0	0
ENSG00000225369	0	0	0	0
ENSG00000225370	0	0	0	0
ENSG00000225371	0	0	0	0
ENSG00000225373	37	60	27	91
ENSG00000225376	0	0	0	0
ENSG00000225377	0	0	8	6
ENSG00000225378	0	0	0	0
ENSG00000225380	0	0	1	0

ENSG00000225381	0	0	0	0
ENSG00000225382	0	0	0	0
ENSG00000225383	0	0	0	0
ENSG00000225384	0	0	0	0
ENSG00000225385	0	0	0	0
ENSG00000225386	0	0	0	0
ENSG00000225387	0	0	0	0
ENSG00000225390	0	0	0	0
ENSG00000225391	5	0	0	0
ENSG00000225392	0	0	0	0
ENSG00000225393	2	0	4	23
ENSG00000225394	0	0	0	0
ENSG00000225396	0	0	0	0
ENSG00000225397	0	0	0	0
ENSG00000225398	0	0	3	0
ENSG00000225399	0	0	0	0
ENSG00000225400	1	0	3	0
ENSG00000225401	0	0	0	0
ENSG00000225402	0	0	0	0
ENSG00000225403	0	0	0	0
ENSG00000225404	0	0	0	0
ENSG00000225405	0	0	0	0
ENSG00000225406	0	0	0	0
ENSG00000225407	0	26	8	6
ENSG00000225408	0	18	8	6
ENSG00000225410	0	0	0	0
ENSG00000225411	0	0	0	0
ENSG00000225412	0	0	0	0
ENSG00000225413	0	0	0	0
ENSG00000225414	0	0	0	0
ENSG00000225415	0	0	0	0
ENSG00000225416	0	0	0	0
ENSG00000225417	0	0	0	0
ENSG00000225418	0	244	25	199
ENSG00000225419	0	0	0	0
ENSG00000225420	0	0	0	0
ENSG00000225421	0	0	0	0
ENSG00000225422	0	0	1	0
ENSG00000225423	0	0	0	0
ENSG00000225424	0	0	0	0
ENSG00000225426	0	0	0	0
ENSG00000225427	0	0	0	0
ENSG00000225428	0	0	0	0
ENSG00000225429	0	0	0	0
ENSG00000225431	0	0	0	0
ENSG00000225432	0	0	0	0

ENSG00000225433	0	0	0	0
ENSG00000225434	3	0	0	0
ENSG00000225437	0	0	0	0
ENSG00000225438	0	0	0	0
ENSG00000225439	114	369	185	128
ENSG00000225441	0	0	0	0
ENSG00000225442	0	0	0	0
ENSG00000225443	0	0	0	0
ENSG00000225444	0	0	0	0
ENSG00000225446	0	0	0	0
ENSG00000225447	1	0	2	13
ENSG00000225448	0	0	0	0
ENSG00000225449	0	0	0	0
ENSG00000225450	0	0	0	0
ENSG00000225451	0	0	0	0
ENSG00000225452	0	0	0	0
ENSG00000225453	0	0	0	0
ENSG00000225454	0	0	0	0
ENSG00000225455	0	0	0	0
ENSG00000225457	0	0	0	0
ENSG00000225458	0	0	0	0
ENSG00000225459	0	0	0	0
ENSG00000225460	4	0	4	10
ENSG00000225462	0	0	0	0
ENSG00000225463	0	0	0	0
ENSG00000225465	0	0	0	0
ENSG00000225466	0	0	0	0
ENSG00000225469	0	0	0	0
ENSG00000225470	62	35	75	82
ENSG00000225471	0	0	0	0
ENSG00000225472	0	0	0	0
ENSG00000225473	0	0	0	0
ENSG00000225475	0	0	0	0
ENSG00000225476	0	23	0	0
ENSG00000225477	0	0	0	0
ENSG00000225478	0	0	0	0
ENSG00000225479	0	0	0	0
ENSG00000225480	2	0	1	0
ENSG00000225482	0	0	0	0
ENSG00000225483	0	0	0	0
ENSG00000225484	34	104	239	171
ENSG00000225485	0	0	0	1
ENSG00000225486	0	0	0	0
ENSG00000225487	0	0	0	0
ENSG00000225488	0	0	0	0
ENSG00000225489	0	0	3	0

ENSG00000225490	0	0	0	0
ENSG00000225491	0	0	0	0
ENSG00000225492	1	0	0	0
ENSG00000225493	0	0	0	0
ENSG00000225496	0	0	0	0
ENSG00000225497	0	0	0	0
ENSG00000225498	0	0	0	0
ENSG00000225499	0	0	0	0
ENSG00000225501	0	0	0	0
ENSG00000225502	0	0	0	0
ENSG00000225504	0	0	0	0
ENSG00000225505	0	0	1	0
ENSG00000225506	11	53	57	70
ENSG00000225507	0	0	0	0
ENSG00000225508	0	0	0	0
ENSG00000225509	0	0	0	0
ENSG00000225510	0	0	0	0
ENSG00000225511	0	0	0	0
ENSG00000225512	0	0	0	0
ENSG00000225513	0	18	0	5
ENSG00000225514	0	0	0	0
ENSG00000225515	0	0	0	0
ENSG00000225516	0	0	0	0
ENSG00000225518	0	0	0	0
ENSG00000225519	0	0	0	0
ENSG00000225520	0	0	0	0
ENSG00000225521	0	0	0	0
ENSG00000225522	0	0	1	0
ENSG00000225523	0	0	0	0
ENSG00000225525	39	172	32	144
ENSG00000225526	0	0	0	0
ENSG00000225527	0	0	0	0
ENSG00000225528	520	4652	5358	3147
ENSG00000225529	0	0	0	0
ENSG00000225530	0	0	0	0
ENSG00000225531	0	0	2	1
ENSG00000225532	0	0	0	0
ENSG00000225533	0	0	0	0
ENSG00000225535	0	0	0	0
ENSG00000225536	0	0	0	0
ENSG00000225537	0	0	0	0
ENSG00000225538	0	0	0	0
ENSG00000225539	0	0	0	0
ENSG00000225540	0	0	0	0
ENSG00000225541	0	0	0	0
ENSG00000225542	0	0	0	0

ENSG00000225543	0	0	0	0
ENSG00000225544	0	0	0	0
ENSG00000225545	0	0	0	0
ENSG00000225546	4	48	16	3
ENSG00000225548	0	0	0	0
ENSG00000225549	0	0	0	0
ENSG00000225550	0	0	0	0
ENSG00000225551	0	0	0	0
ENSG00000225552	0	0	0	0
ENSG00000225553	0	0	0	0
ENSG00000225554	0	0	0	0
ENSG00000225555	0	0	0	0
ENSG00000225556	0	0	0	0
ENSG00000225557	0	0	0	0
ENSG00000225558	0	0	0	0
ENSG00000225559	1	0	0	0
ENSG00000225560	0	0	0	0
ENSG00000225561	2	0	66	19
ENSG00000225562	0	0	0	0
ENSG00000225563	0	0	0	0
ENSG00000225564	0	0	0	0
ENSG00000225567	0	0	0	0
ENSG00000225568	0	0	0	0
ENSG00000225569	0	0	0	2
ENSG00000225570	0	0	0	0
ENSG00000225571	0	0	0	0
ENSG00000225572	0	0	0	0
ENSG00000225573	0	2	0	0
ENSG00000225574	0	0	0	0
ENSG00000225578	0	0	0	0
ENSG00000225579	0	0	0	0
ENSG00000225580	0	0	0	0
ENSG00000225581	0	0	0	0
ENSG00000225582	0	0	0	0
ENSG00000225583	0	0	0	0
ENSG00000225585	0	0	0	0
ENSG00000225588	0	0	0	0
ENSG00000225589	0	0	0	0
ENSG00000225590	0	0	0	0
ENSG00000225591	0	0	2	4
ENSG00000225592	0	0	0	0
ENSG00000225594	0	0	0	0
ENSG00000225595	0	0	0	0
ENSG00000225597	1	0	0	0
ENSG00000225598	0	0	0	0
ENSG00000225602	0	0	0	0

ENSG00000225603	0	0	0	0
ENSG00000225604	0	0	0	0
ENSG00000225605	0	0	0	0
ENSG00000225606	0	0	0	0
ENSG00000225609	0	0	0	0
ENSG00000225610	0	0	0	0
ENSG00000225611	1	0	0	1
ENSG00000225612	1	0	0	0
ENSG00000225613	0	0	0	0
ENSG00000225614	0	0	0	0
ENSG00000225615	0	0	0	0
ENSG00000225616	1	0	0	0
ENSG00000225618	0	0	0	0
ENSG00000225619	0	0	0	0
ENSG00000225620	0	0	0	0
ENSG00000225622	0	0	0	0
ENSG00000225623	0	0	0	0
ENSG00000225624	0	0	0	0
ENSG00000225625	0	0	0	0
ENSG00000225626	1	0	0	0
ENSG00000225627	0	0	0	0
ENSG00000225629	0	0	0	0
ENSG00000225630	2227	4410	4305	778
ENSG00000225632	0	0	0	0
ENSG00000225635	0	0	0	0
ENSG00000225636	0	0	0	0
ENSG00000225637	0	0	0	0
ENSG00000225638	0	0	0	0
ENSG00000225639	0	0	0	0
ENSG00000225640	0	0	0	0
ENSG00000225642	0	0	0	0
ENSG00000225643	8	0	4	7
ENSG00000225644	0	0	0	0
ENSG00000225646	0	0	0	0
ENSG00000225647	0	0	0	0
ENSG00000225648	25	681	665	1033
ENSG00000225649	0	0	0	0
ENSG00000225650	0	0	0	0
ENSG00000225652	0	0	0	0
ENSG00000225653	0	0	0	0
ENSG00000225655	0	0	0	0
ENSG00000225656	0	0	0	0
ENSG00000225657	0	0	0	0
ENSG00000225658	0	0	0	0
ENSG00000225659	0	0	0	0
ENSG00000225660	0	0	0	0

ENSG00000225661	0	0	0	0
ENSG00000225662	0	0	0	0
ENSG00000225663	0	0	0	0
ENSG00000225664	0	0	0	0
ENSG00000225665	0	0	0	0
ENSG00000225666	0	0	1	0
ENSG00000225667	0	0	0	0
ENSG00000225669	0	0	0	0
ENSG00000225670	1	0	0	0
ENSG00000225671	0	0	0	0
ENSG00000225672	0	0	0	0
ENSG00000225673	0	0	0	0
ENSG00000225674	0	0	1	0
ENSG00000225675	0	0	0	0
ENSG00000225676	0	0	0	0
ENSG00000225678	0	0	0	0
ENSG00000225679	0	0	0	0
ENSG00000225680	0	0	0	0
ENSG00000225681	1	106	45	29
ENSG00000225682	0	0	0	0
ENSG00000225683	0	0	0	0
ENSG00000225684	0	0	0	0
ENSG00000225685	0	0	0	0
ENSG00000225689	0	0	0	0
ENSG00000225690	0	0	0	0
ENSG00000225691	0	0	0	0
ENSG00000225693	0	0	0	0
ENSG00000225695	0	0	0	0
ENSG00000225696	0	0	0	0
ENSG00000225697	1	0	2	3
ENSG00000225698	0	0	0	0
ENSG00000225701	0	0	0	0
ENSG00000225703	0	0	0	0
ENSG00000225704	0	0	0	0
ENSG00000225705	0	1	0	1
ENSG00000225706	0	0	0	0
ENSG00000225708	0	0	0	0
ENSG00000225710	0	0	0	0
ENSG00000225711	0	0	0	0
ENSG00000225712	0	0	0	0
ENSG00000225713	0	0	0	0
ENSG00000225714	0	0	0	0
ENSG00000225715	0	0	0	0
ENSG00000225716	0	0	0	0
ENSG00000225718	0	0	0	0
ENSG00000225719	0	0	4	0

ENSG00000225720	3	0	0	0
ENSG00000225721	0	0	3	0
ENSG00000225722	0	0	0	0
ENSG00000225723	0	0	0	0
ENSG00000225724	0	0	0	0
ENSG00000225725	0	0	0	0
ENSG00000225726	0	0	1	0
ENSG00000225727	0	0	0	0
ENSG00000225728	0	0	0	0
ENSG00000225730	0	0	0	0
ENSG00000225731	0	0	0	0
ENSG00000225732	0	0	0	0
ENSG00000225733	901	2141	3430	4438
ENSG00000225735	0	0	0	0
ENSG00000225736	0	0	0	0
ENSG00000225737	0	0	0	0
ENSG00000225738	0	0	0	0
ENSG00000225739	0	0	0	0
ENSG00000225740	0	0	0	0
ENSG00000225741	0	0	0	0
ENSG00000225742	0	0	0	0
ENSG00000225744	1	0	0	0
ENSG00000225745	0	0	0	0
ENSG00000225746	5	1690	777	1947
ENSG00000225748	0	0	0	0
ENSG00000225750	0	0	1	8
ENSG00000225751	0	0	0	7
ENSG00000225752	0	0	0	0
ENSG00000225755	0	0	0	0
ENSG00000225756	0	0	0	0
ENSG00000225758	0	0	0	0
ENSG00000225759	0	0	0	0
ENSG00000225760	0	0	0	1
ENSG00000225761	0	0	0	1
ENSG00000225762	155	143	172	112
ENSG00000225763	0	0	0	0
ENSG00000225764	10	0	2	9
ENSG00000225765	3	0	0	2
ENSG00000225766	0	0	0	2
ENSG00000225767	0	0	0	0
ENSG00000225768	0	0	0	0
ENSG00000225769	0	0	0	0
ENSG00000225770	0	0	0	0
ENSG00000225771	0	0	0	0
ENSG00000225773	0	0	0	0
ENSG00000225774	0	0	0	0

ENSG00000225775	0	0	0	0
ENSG00000225777	0	0	0	0
ENSG00000225778	4	10	21	31
ENSG00000225779	0	0	0	0
ENSG00000225781	0	0	0	0
ENSG00000225782	0	0	0	0
ENSG00000225783	0	24	0	3
ENSG00000225784	0	73	37	17
ENSG00000225785	0	0	0	0
ENSG00000225786	0	0	0	0
ENSG00000225787	0	0	0	0
ENSG00000225788	0	0	0	0
ENSG00000225790	0	0	0	0
ENSG00000225791	1	54	28	62
ENSG00000225792	0	0	3	0
ENSG00000225793	0	0	4	21
ENSG00000225794	0	0	0	0
ENSG00000225795	0	0	0	0
ENSG00000225796	0	0	0	0
ENSG00000225797	0	0	0	0
ENSG00000225798	0	0	3	0
ENSG00000225801	0	0	0	0
ENSG00000225802	0	0	0	0
ENSG00000225803	0	0	0	0
ENSG00000225805	0	0	0	0
ENSG00000225806	0	0	3	6
ENSG00000225807	0	0	0	3
ENSG00000225808	0	0	11	1
ENSG00000225809	0	0	0	0
ENSG00000225810	0	0	0	0
ENSG00000225811	0	0	0	0
ENSG00000225812	0	0	0	0
ENSG00000225813	0	0	0	0
ENSG00000225814	0	0	0	0
ENSG00000225815	0	0	0	0
ENSG00000225816	0	0	0	0
ENSG00000225818	0	0	0	0
ENSG00000225819	0	0	0	0
ENSG00000225822	0	0	0	1
ENSG00000225823	0	0	0	0
ENSG00000225824	0	0	0	0
ENSG00000225825	0	0	0	0
ENSG00000225826	0	0	0	0
ENSG00000225827	0	0	0	0
ENSG00000225828	3	56	14	10
ENSG00000225830	21	986	592	684

ENSG00000225831	0	0	0	0
ENSG00000225832	0	0	0	0
ENSG00000225833	0	0	0	0
ENSG00000225834	0	0	0	0
ENSG00000225836	0	0	0	0
ENSG00000225838	0	0	0	0
ENSG00000225839	0	0	0	0
ENSG00000225840	1	0	0	0
ENSG00000225842	0	0	0	0
ENSG00000225843	0	0	0	0
ENSG00000225845	0	0	0	0
ENSG00000225846	0	0	0	0
ENSG00000225849	0	0	0	0
ENSG00000225850	0	0	0	0
ENSG00000225851	0	0	0	0
ENSG00000225854	0	0	0	0
ENSG00000225855	1	0	2	0
ENSG00000225856	0	0	0	0
ENSG00000225857	0	0	0	0
ENSG00000225858	0	0	0	0
ENSG00000225859	0	0	0	0
ENSG00000225860	0	0	0	0
ENSG00000225864	1	1	2	4
ENSG00000225867	0	0	0	0
ENSG00000225868	0	0	0	0
ENSG00000225869	0	0	0	0
ENSG00000225870	0	0	0	0
ENSG00000225871	0	0	0	0
ENSG00000225872	0	0	0	0
ENSG00000225873	12	0	17	5
ENSG00000225876	0	0	0	0
ENSG00000225877	0	0	0	0
ENSG00000225878	0	0	0	0
ENSG00000225879	0	0	0	0
ENSG00000225880	4	0	0	1
ENSG00000225881	0	0	0	0
ENSG00000225882	0	0	0	0
ENSG00000225883	0	0	0	0
ENSG00000225884	0	0	0	9
ENSG00000225885	3	0	0	0
ENSG00000225886	0	0	0	0
ENSG00000225888	0	0	0	0
ENSG00000225889	17	7	2	25
ENSG00000225890	0	0	0	0
ENSG00000225891	0	0	11	29
ENSG00000225892	1	0	0	0

ENSG00000225893	0	0	0	0
ENSG00000225894	0	0	0	0
ENSG00000225895	0	0	0	0
ENSG00000225896	0	0	0	0
ENSG00000225898	0	0	0	0
ENSG00000225899	0	0	0	0
ENSG00000225900	0	0	0	0
ENSG00000225901	0	0	0	0
ENSG00000225903	0	0	0	0
ENSG00000225904	0	0	0	0
ENSG00000225905	0	0	0	0
ENSG00000225906	0	0	0	0
ENSG00000225907	0	13	0	1
ENSG00000225911	0	0	0	0
ENSG00000225912	0	0	0	0
ENSG00000225913	0	0	0	0
ENSG00000225914	37	406	452	302
ENSG00000225916	5	41	1	3
ENSG00000225918	0	0	0	0
ENSG00000225919	0	0	0	0
ENSG00000225920	2	0	13	9
ENSG00000225921	0	113	172	159
ENSG00000225922	0	0	0	0
ENSG00000225923	0	0	0	0
ENSG00000225924	0	0	2	0
ENSG00000225925	0	0	0	0
ENSG00000225926	0	0	0	0
ENSG00000225927	0	0	0	0
ENSG00000225928	0	0	0	0
ENSG00000225929	0	0	0	0
ENSG00000225930	0	0	0	0
ENSG00000225931	9	10	0	0
ENSG00000225932	0	0	0	0
ENSG00000225933	0	0	0	0
ENSG00000225934	0	0	0	0
ENSG00000225935	0	0	0	0
ENSG00000225936	416	3965	2977	3917
ENSG00000225937	1	0	0	0
ENSG00000225938	0	0	0	0
ENSG00000225939	0	0	0	0
ENSG00000225940	0	0	1	0
ENSG00000225942	0	0	0	0
ENSG00000225943	0	0	0	0
ENSG00000225944	0	0	0	0
ENSG00000225945	0	0	1	1
ENSG00000225946	0	0	0	0

ENSG00000225947	0	0	0	0
ENSG00000225948	1	0	23	34
ENSG00000225949	0	0	0	0
ENSG00000225950	0	0	0	0
ENSG00000225951	0	18	2	3
ENSG00000225952	0	0	0	0
ENSG00000225953	0	0	1	0
ENSG00000225954	0	0	0	0
ENSG00000225955	0	0	0	0
ENSG00000225956	0	0	3	0
ENSG00000225957	0	0	0	0
ENSG00000225959	0	0	0	0
ENSG00000225960	0	0	0	0
ENSG00000225962	0	0	0	0
ENSG00000225963	0	0	0	0
ENSG00000225964	0	0	0	0
ENSG00000225965	0	0	0	4
ENSG00000225967	0	0	0	0
ENSG00000225968	0	0	0	0
ENSG00000225969	0	0	0	2
ENSG00000225970	0	0	0	0
ENSG00000225971	1	0	0	0
ENSG00000225972	1573	205	108	251
ENSG00000225973	6	0	1	0
ENSG00000225974	0	0	0	0
ENSG00000225975	155	148	56	117
ENSG00000225976	0	0	0	0
ENSG00000225978	0	0	0	0
ENSG00000225979	0	0	0	0
ENSG00000225980	0	0	0	0
ENSG00000225981	10	26	23	20
ENSG00000225982	0	0	0	0
ENSG00000225984	1	0	0	0
ENSG00000225985	0	0	0	0
ENSG00000225986	1	0	0	0
ENSG00000225987	0	0	0	0
ENSG00000225988	0	0	0	0
ENSG00000225989	0	0	0	0
ENSG00000225990	0	0	0	0
ENSG00000225991	0	0	1	0
ENSG00000225992	0	0	0	0
ENSG00000225993	0	0	0	0
ENSG00000225994	0	0	0	0
ENSG00000225995	0	0	0	0
ENSG00000225996	0	0	0	0
ENSG00000225997	0	0	0	0

ENSG00000225998	0	0	0	0
ENSG00000225999	0	0	0	0
ENSG00000226000	0	0	0	0
ENSG00000226002	0	8	47	59
ENSG00000226003	0	0	0	0
ENSG00000226004	0	0	0	0
ENSG00000226005	0	0	0	0
ENSG00000226007	0	0	0	0
ENSG00000226008	0	0	0	0
ENSG00000226009	5	0	3	3
ENSG00000226010	0	0	0	0
ENSG00000226011	0	0	0	0
ENSG00000226012	0	0	0	0
ENSG00000226013	0	0	0	0
ENSG00000226014	0	0	0	0
ENSG00000226015	7	36	54	53
ENSG00000226016	0	0	0	0
ENSG00000226017	3	0	0	0
ENSG00000226020	0	0	0	0
ENSG00000226022	0	0	0	0
ENSG00000226023	0	0	0	0
ENSG00000226024	0	0	0	0
ENSG00000226025	0	0	0	0
ENSG00000226026	2	0	2	0
ENSG00000226027	0	0	0	0
ENSG00000226028	0	0	0	0
ENSG00000226029	0	0	1	0
ENSG00000226030	0	0	0	0
ENSG00000226031	0	0	0	0
ENSG00000226032	0	0	0	0
ENSG00000226033	0	0	0	0
ENSG00000226034	0	0	0	0
ENSG00000226035	0	0	0	0
ENSG00000226036	0	0	0	0
ENSG00000226037	0	0	0	0
ENSG00000226038	0	0	0	0
ENSG00000226040	0	0	0	0
ENSG00000226041	0	0	0	0
ENSG00000226042	0	0	0	0
ENSG00000226043	0	0	0	0
ENSG00000226044	0	0	0	0
ENSG00000226045	0	0	0	0
ENSG00000226046	0	0	0	0
ENSG00000226047	0	0	0	0
ENSG00000226048	0	0	0	0
ENSG00000226049	0	84	63	142

ENSG00000226050	0	0	0	0
ENSG00000226051	0	0	0	0
ENSG00000226053	0	0	0	0
ENSG00000226054	0	10	20	11
ENSG00000226055	0	0	0	0
ENSG00000226056	0	0	0	0
ENSG00000226057	0	0	0	0
ENSG00000226058	0	0	0	0
ENSG00000226059	0	0	0	0
ENSG00000226060	0	0	0	0
ENSG00000226061	0	0	0	0
ENSG00000226063	0	0	0	0
ENSG00000226064	0	0	0	0
ENSG00000226065	0	0	0	0
ENSG00000226066	0	0	0	0
ENSG00000226067	0	0	0	9
ENSG00000226068	0	0	0	0
ENSG00000226070	0	0	0	0
ENSG00000226071	0	0	0	0
ENSG00000226072	0	0	0	0
ENSG00000226074	0	0	0	0
ENSG00000226075	0	0	0	0
ENSG00000226078	0	0	0	0
ENSG00000226079	0	0	0	0
ENSG00000226080	0	0	0	0
ENSG00000226081	0	0	0	0
ENSG00000226082	0	0	0	0
ENSG00000226083	0	0	0	0
ENSG00000226084	2	0	0	2
ENSG00000226085	0	1	1	0
ENSG00000226086	0	0	0	0
ENSG00000226087	0	0	0	0
ENSG00000226088	0	0	0	0
ENSG00000226089	0	0	0	0
ENSG00000226091	2	0	1	8
ENSG00000226092	0	0	0	0
ENSG00000226093	0	0	0	0
ENSG00000226094	0	0	0	0
ENSG00000226096	0	0	0	0
ENSG00000226097	0	0	0	1
ENSG00000226098	0	0	0	0
ENSG00000226099	0	0	0	0
ENSG00000226101	0	0	0	0
ENSG00000226102	0	0	3	11
ENSG00000226103	0	0	0	0
ENSG00000226104	0	0	0	0

ENSG00000226107	0	0	0	0
ENSG00000226108	0	0	0	0
ENSG00000226109	0	0	0	0
ENSG00000226110	0	0	0	0
ENSG00000226111	0	0	0	0
ENSG00000226112	0	0	0	0
ENSG00000226113	0	0	0	0
ENSG00000226114	0	0	0	0
ENSG00000226115	0	0	0	0
ENSG00000226116	0	0	0	0
ENSG00000226117	0	0	0	0
ENSG00000226118	0	0	0	0
ENSG00000226119	0	0	0	0
ENSG00000226121	12	62	39	147
ENSG00000226122	0	0	0	0
ENSG00000226124	0	0	4	14
ENSG00000226125	1	0	0	2
ENSG00000226126	0	0	0	0
ENSG00000226127	0	0	0	0
ENSG00000226128	0	0	0	0
ENSG00000226129	0	0	0	0
ENSG00000226130	0	0	0	0
ENSG00000226131	0	0	0	0
ENSG00000226132	0	0	0	0
ENSG00000226133	0	0	0	0
ENSG00000226134	0	0	0	0
ENSG00000226137	88	37	35	64
ENSG00000226138	0	0	0	0
ENSG00000226139	0	0	0	0
ENSG00000226140	0	0	0	0
ENSG00000226141	0	0	0	0
ENSG00000226142	0	0	0	0
ENSG00000226143	0	1	0	0
ENSG00000226144	0	0	0	0
ENSG00000226145	0	0	0	0
ENSG00000226147	0	0	0	0
ENSG00000226148	0	0	0	0
ENSG00000226149	2	729	997	1298
ENSG00000226150	0	2	0	0
ENSG00000226152	0	0	0	0
ENSG00000226153	0	0	0	0
ENSG00000226155	3	7	5	5
ENSG00000226156	0	0	0	0
ENSG00000226157	0	0	0	0
ENSG00000226158	0	0	0	0
ENSG00000226159	0	0	0	0

ENSG00000226160	0	0	0	0
ENSG00000226161	0	0	0	0
ENSG00000226163	0	0	0	0
ENSG00000226164	0	0	0	0
ENSG00000226165	0	0	0	0
ENSG00000226166	0	0	0	0
ENSG00000226167	0	0	2	3
ENSG00000226168	0	0	0	0
ENSG00000226169	0	0	0	0
ENSG00000226170	0	0	0	0
ENSG00000226171	0	0	0	0
ENSG00000226172	0	0	0	0
ENSG00000226173	0	0	0	0
ENSG00000226174	0	0	1	0
ENSG00000226175	0	0	0	0
ENSG00000226176	0	0	0	0
ENSG00000226179	0	0	0	0
ENSG00000226180	0	0	0	0
ENSG00000226181	0	0	0	0
ENSG00000226182	0	0	0	0
ENSG00000226183	0	0	0	0
ENSG00000226185	0	0	0	0
ENSG00000226186	0	0	0	0
ENSG00000226187	0	0	0	0
ENSG00000226188	0	0	0	0
ENSG00000226191	2	18	61	32
ENSG00000226192	0	0	0	0
ENSG00000226193	0	0	0	0
ENSG00000226194	0	0	0	0
ENSG00000226196	0	0	0	0
ENSG00000226197	3	0	0	0
ENSG00000226199	0	0	0	0
ENSG00000226200	6	0	32	33
ENSG00000226201	0	0	0	0
ENSG00000226202	0	0	0	0
ENSG00000226203	0	0	0	0
ENSG00000226204	0	0	0	0
ENSG00000226205	0	0	0	0
ENSG00000226206	0	0	0	0
ENSG00000226207	0	0	0	0
ENSG00000226208	0	0	0	0
ENSG00000226209	0	0	0	0
ENSG00000226210	11	52	12	27
ENSG00000226211	0	0	0	0
ENSG00000226212	0	0	0	0
ENSG00000226213	0	0	0	0

ENSG00000226215	0	0	0	0
ENSG00000226216	0	0	0	0
ENSG00000226217	0	0	0	0
ENSG00000226218	0	0	0	0
ENSG00000226220	0	0	0	0
ENSG00000226221	0	0	0	0
ENSG00000226222	0	0	0	0
ENSG00000226223	0	0	0	0
ENSG00000226225	0	0	0	0
ENSG00000226226	0	0	0	0
ENSG00000226227	0	0	0	0
ENSG00000226228	0	0	0	0
ENSG00000226229	0	0	0	0
ENSG00000226230	0	0	0	0
ENSG00000226232	0	0	0	0
ENSG00000226233	0	0	0	0
ENSG00000226234	0	0	0	0
ENSG00000226235	0	0	0	0
ENSG00000226237	0	0	0	0
ENSG00000226238	0	0	0	0
ENSG00000226239	0	0	0	0
ENSG00000226240	0	0	0	0
ENSG00000226241	0	0	0	0
ENSG00000226242	0	0	0	0
ENSG00000226243	77	81	19	11
ENSG00000226245	0	0	1	0
ENSG00000226246	0	0	0	0
ENSG00000226247	0	0	0	0
ENSG00000226248	0	0	0	0
ENSG00000226249	0	0	0	0
ENSG00000226250	0	0	0	0
ENSG00000226251	3	0	3	10
ENSG00000226252	318	100	96	28
ENSG00000226253	0	0	0	0
ENSG00000226254	0	0	0	0
ENSG00000226255	0	0	0	0
ENSG00000226257	0	0	0	0
ENSG00000226258	0	0	0	2
ENSG00000226259	0	1	1	244
ENSG00000226260	0	0	0	0
ENSG00000226261	0	0	0	0
ENSG00000226262	0	0	0	0
ENSG00000226263	0	0	0	0
ENSG00000226264	0	0	0	0
ENSG00000226266	1	0	6	23
ENSG00000226267	0	0	0	0

ENSG00000226268	0	0	0	0
ENSG00000226270	0	0	0	0
ENSG00000226271	0	0	0	0
ENSG00000226272	0	0	0	0
ENSG00000226273	0	0	0	0
ENSG00000226275	0	0	0	0
ENSG00000226276	0	15	0	0
ENSG00000226277	0	0	0	0
ENSG00000226278	0	0	0	0
ENSG00000226279	0	0	0	0
ENSG00000226280	0	0	0	0
ENSG00000226281	0	0	0	0
ENSG00000226284	0	0	1	0
ENSG00000226285	0	0	0	0
ENSG00000226286	0	0	0	0
ENSG00000226287	1	0	3	2
ENSG00000226288	0	0	0	0
ENSG00000226289	0	0	0	4
ENSG00000226291	0	0	0	0
ENSG00000226292	0	0	0	0
ENSG00000226296	0	0	0	0
ENSG00000226297	0	0	0	0
ENSG00000226298	0	0	0	0
ENSG00000226299	0	0	0	0
ENSG00000226301	0	0	0	0
ENSG00000226302	0	0	0	0
ENSG00000226303	0	0	0	0
ENSG00000226304	0	0	0	0
ENSG00000226306	0	0	0	0
ENSG00000226307	0	0	0	0
ENSG00000226308	0	0	0	0
ENSG00000226309	0	0	1	5
ENSG00000226310	7	1	35	114
ENSG00000226312	0	52	45	12
ENSG00000226313	0	0	0	0
ENSG00000226314	0	0	6	1
ENSG00000226317	0	0	0	0
ENSG00000226318	0	14	3	6
ENSG00000226320	0	0	0	0
ENSG00000226321	0	0	0	0
ENSG00000226323	0	0	0	0
ENSG00000226324	0	0	0	0
ENSG00000226327	0	0	0	0
ENSG00000226328	0	0	6	6
ENSG00000226329	4	0	1	0
ENSG00000226330	0	0	0	0

ENSG00000226331	0	0	0	0
ENSG00000226332	1	1	0	0
ENSG00000226334	0	0	0	0
ENSG00000226335	0	0	0	0
ENSG00000226336	0	0	0	0
ENSG00000226337	0	0	0	0
ENSG00000226338	0	0	0	0
ENSG00000226339	0	0	0	0
ENSG00000226340	0	0	0	0
ENSG00000226341	0	0	0	0
ENSG00000226342	0	0	0	0
ENSG00000226343	0	0	0	0
ENSG00000226344	0	0	0	0
ENSG00000226345	0	0	0	0
ENSG00000226347	0	0	0	0
ENSG00000226348	0	0	0	0
ENSG00000226349	0	0	0	0
ENSG00000226352	0	0	0	0
ENSG00000226353	0	0	0	0
ENSG00000226354	0	0	0	0
ENSG00000226355	0	0	0	0
ENSG00000226356	0	0	0	0
ENSG00000226358	0	0	1	0
ENSG00000226359	0	0	0	0
ENSG00000226360	5	0	0	0
ENSG00000226361	0	0	0	0
ENSG00000226362	0	0	0	0
ENSG00000226363	0	0	0	0
ENSG00000226364	0	0	0	0
ENSG00000226365	0	0	0	0
ENSG00000226366	0	0	0	0
ENSG00000226367	0	84	52	37
ENSG00000226368	0	0	0	0
ENSG00000226369	0	0	0	0
ENSG00000226370	0	0	0	0
ENSG00000226372	0	0	0	0
ENSG00000226374	0	0	0	0
ENSG00000226375	0	0	13	4
ENSG00000226376	0	0	0	0
ENSG00000226377	1	0	0	0
ENSG00000226378	0	0	0	0
ENSG00000226379	0	0	0	0
ENSG00000226380	0	0	0	0
ENSG00000226381	12	130	103	71
ENSG00000226383	0	12	0	0
ENSG00000226384	0	0	0	0

ENSG00000226386	7	0	0	1
ENSG00000226387	0	0	0	0
ENSG00000226388	0	0	0	0
ENSG00000226389	0	0	0	0
ENSG00000226390	0	0	0	0
ENSG00000226392	0	0	0	0
ENSG00000226393	0	0	0	0
ENSG00000226394	0	0	0	0
ENSG00000226395	0	0	0	0
ENSG00000226396	0	0	0	1
ENSG00000226397	0	0	0	0
ENSG00000226398	0	0	0	0
ENSG00000226400	0	0	0	0
ENSG00000226401	0	0	0	0
ENSG00000226402	0	0	0	0
ENSG00000226403	4	0	0	0
ENSG00000226404	0	0	0	0
ENSG00000226405	0	0	0	0
ENSG00000226406	0	0	0	0
ENSG00000226407	0	0	0	0
ENSG00000226408	0	0	0	0
ENSG00000226409	0	0	0	0
ENSG00000226410	0	0	0	0
ENSG00000226411	0	0	0	0
ENSG00000226412	0	0	0	0
ENSG00000226413	0	0	0	0
ENSG00000226414	0	0	0	0
ENSG00000226415	0	0	1	0
ENSG00000226416	0	0	0	0
ENSG00000226417	0	0	0	0
ENSG00000226419	0	19	0	0
ENSG00000226420	0	0	0	0
ENSG00000226421	0	0	0	0
ENSG00000226422	0	0	0	0
ENSG00000226423	0	0	0	0
ENSG00000226425	0	0	0	0
ENSG00000226426	0	0	0	0
ENSG00000226427	0	0	0	0
ENSG00000226428	0	0	0	0
ENSG00000226429	0	0	0	0
ENSG00000226430	0	0	0	0
ENSG00000226431	0	0	0	0
ENSG00000226432	0	1	0	0
ENSG00000226433	0	0	0	0
ENSG00000226434	3	0	1	5
ENSG00000226435	36	377	145	1328

ENSG00000226436	1	0	0	1
ENSG00000226437	0	0	0	0
ENSG00000226438	0	0	1	3
ENSG00000226439	0	0	0	0
ENSG00000226440	0	0	0	0
ENSG00000226441	0	0	0	0
ENSG00000226442	0	0	0	0
ENSG00000226443	0	0	0	0
ENSG00000226444	0	0	0	0
ENSG00000226445	1	0	0	0
ENSG00000226446	1	0	0	2
ENSG00000226447	1	0	0	0
ENSG00000226448	0	0	0	0
ENSG00000226449	0	0	0	0
ENSG00000226450	0	0	0	0
ENSG00000226453	1	0	0	0
ENSG00000226454	0	0	0	0
ENSG00000226455	0	0	0	0
ENSG00000226457	0	0	0	0
ENSG00000226461	0	0	0	0
ENSG00000226462	0	0	0	0
ENSG00000226463	0	0	0	0
ENSG00000226465	2	0	6	1
ENSG00000226466	0	0	0	0
ENSG00000226467	0	0	0	0
ENSG00000226468	0	0	0	0
ENSG00000226469	0	0	0	0
ENSG00000226470	0	0	0	0
ENSG00000226471	21	53	4	4
ENSG00000226472	0	0	0	0
ENSG00000226473	0	0	3	2
ENSG00000226474	0	0	0	0
ENSG00000226476	9	84	39	81
ENSG00000226477	0	0	0	0
ENSG00000226478	0	0	2	2
ENSG00000226479	2	0	1	2
ENSG00000226480	0	0	0	0
ENSG00000226481	0	0	0	0
ENSG00000226482	0	0	0	0
ENSG00000226483	0	0	0	0
ENSG00000226484	0	0	0	0
ENSG00000226485	0	0	0	0
ENSG00000226486	0	0	0	0
ENSG00000226487	0	0	0	0
ENSG00000226488	0	0	0	0
ENSG00000226489	0	0	0	0

ENSG00000226490	0	0	0	0
ENSG00000226491	0	0	0	0
ENSG00000226492	0	0	0	0
ENSG00000226493	0	0	0	0
ENSG00000226494	0	0	0	0
ENSG00000226496	0	0	0	0
ENSG00000226497	0	0	0	0
ENSG00000226498	0	0	0	0
ENSG00000226499	0	0	0	0
ENSG00000226500	0	0	0	0
ENSG00000226501	0	0	0	0
ENSG00000226502	0	0	0	0
ENSG00000226504	0	0	0	0
ENSG00000226505	0	0	0	0
ENSG00000226506	0	0	0	0
ENSG00000226507	0	0	1	0
ENSG00000226508	0	0	0	0
ENSG00000226509	0	0	0	0
ENSG00000226510	13	27	16	13
ENSG00000226513	0	0	0	0
ENSG00000226515	0	0	0	0
ENSG00000226516	0	0	0	0
ENSG00000226517	0	0	0	0
ENSG00000226519	0	0	2	0
ENSG00000226520	0	0	0	0
ENSG00000226521	0	0	0	0
ENSG00000226522	0	0	0	0
ENSG00000226523	0	0	0	0
ENSG00000226524	0	175	51	104
ENSG00000226525	0	0	0	0
ENSG00000226526	0	0	0	0
ENSG00000226527	0	0	0	0
ENSG00000226529	0	0	0	0
ENSG00000226530	0	0	0	0
ENSG00000226531	0	0	0	0
ENSG00000226532	1	0	0	0
ENSG00000226533	0	0	0	0
ENSG00000226534	0	0	0	0
ENSG00000226535	0	0	0	0
ENSG00000226536	0	0	0	0
ENSG00000226537	0	0	0	0
ENSG00000226538	0	0	0	0
ENSG00000226539	0	0	0	0
ENSG00000226540	0	0	0	0
ENSG00000226541	0	0	0	0
ENSG00000226542	0	0	0	0

ENSG00000226543	0	0	0	0
ENSG00000226544	0	0	0	0
ENSG00000226545	0	0	0	0
ENSG00000226546	0	0	0	0
ENSG00000226547	0	0	0	0
ENSG00000226548	0	0	0	4
ENSG00000226549	0	0	0	0
ENSG00000226550	0	0	0	0
ENSG00000226552	0	0	0	0
ENSG00000226553	0	0	0	0
ENSG00000226554	0	0	0	0
ENSG00000226555	0	0	0	0
ENSG00000226556	0	0	0	4
ENSG00000226557	0	0	0	0
ENSG00000226558	0	0	0	0
ENSG00000226560	0	0	0	0
ENSG00000226562	0	0	0	0
ENSG00000226563	0	0	0	0
ENSG00000226564	14	32	25	34
ENSG00000226565	0	0	0	0
ENSG00000226566	0	0	0	0
ENSG00000226567	2	0	3	0
ENSG00000226570	0	0	0	0
ENSG00000226571	0	0	0	1
ENSG00000226572	0	0	0	0
ENSG00000226573	0	0	0	0
ENSG00000226574	0	0	0	0
ENSG00000226575	0	0	0	0
ENSG00000226576	0	0	0	0
ENSG00000226577	0	0	0	0
ENSG00000226578	0	0	1	0
ENSG00000226579	0	0	0	0
ENSG00000226580	0	0	0	0
ENSG00000226581	0	0	0	0
ENSG00000226582	0	0	0	0
ENSG00000226585	3	0	0	1
ENSG00000226586	0	0	0	0
ENSG00000226587	0	0	0	0
ENSG00000226589	0	0	0	0
ENSG00000226590	0	0	0	0
ENSG00000226591	0	0	0	0
ENSG00000226592	0	0	0	0
ENSG00000226593	0	0	0	0
ENSG00000226594	0	0	0	0
ENSG00000226595	0	0	0	0
ENSG00000226597	0	0	0	0

ENSG00000226598	0	0	0	0
ENSG00000226599	0	0	0	0
ENSG00000226600	0	0	0	0
ENSG00000226601	0	0	0	0
ENSG00000226603	0	0	0	0
ENSG00000226604	0	1	1	1
ENSG00000226605	0	46	9	6
ENSG00000226608	8	2	3	1
ENSG00000226609	1	0	0	0
ENSG00000226610	0	0	0	0
ENSG00000226611	0	0	0	0
ENSG00000226613	0	0	0	0
ENSG00000226614	0	0	0	0
ENSG00000226615	0	0	0	0
ENSG00000226616	0	0	0	0
ENSG00000226617	0	0	0	0
ENSG00000226618	0	0	0	0
ENSG00000226619	0	0	0	0
ENSG00000226620	0	0	0	0
ENSG00000226621	0	0	0	0
ENSG00000226622	0	0	0	0
ENSG00000226624	0	0	0	0
ENSG00000226625	0	0	0	0
ENSG00000226626	0	0	0	0
ENSG00000226627	0	0	0	0
ENSG00000226629	0	0	0	0
ENSG00000226631	0	0	0	0
ENSG00000226632	0	0	0	0
ENSG00000226633	0	0	0	0
ENSG00000226634	0	0	0	0
ENSG00000226636	0	0	0	0
ENSG00000226637	0	58	10	37
ENSG00000226640	0	0	0	0
ENSG00000226641	0	0	0	0
ENSG00000226642	0	0	0	0
ENSG00000226643	0	0	0	0
ENSG00000226644	0	0	0	0
ENSG00000226645	0	0	0	0
ENSG00000226646	0	0	0	0
ENSG00000226647	0	106	119	86
ENSG00000226648	0	131	118	73
ENSG00000226649	0	0	0	0
ENSG00000226650	0	0	0	0
ENSG00000226651	0	0	0	0
ENSG00000226652	0	0	0	0
ENSG00000226653	0	0	0	0

ENSG00000226655	0	0	0	0
ENSG00000226657	0	0	0	0
ENSG00000226658	0	0	0	0
ENSG00000226659	0	0	0	0
ENSG00000226660	0	0	0	0
ENSG00000226661	0	0	0	0
ENSG00000226662	0	0	0	0
ENSG00000226663	0	0	0	0
ENSG00000226664	0	0	0	0
ENSG00000226665	0	0	0	0
ENSG00000226666	0	0	0	0
ENSG00000226667	0	0	0	0
ENSG00000226668	0	0	0	0
ENSG00000226669	0	0	0	0
ENSG00000226670	0	0	0	0
ENSG00000226671	0	0	0	0
ENSG00000226673	0	0	0	0
ENSG00000226674	0	13	0	1
ENSG00000226675	0	0	0	0
ENSG00000226676	0	0	0	0
ENSG00000226677	0	0	0	0
ENSG00000226679	0	0	0	0
ENSG00000226680	0	0	0	0
ENSG00000226681	0	0	0	0
ENSG00000226683	0	0	0	1
ENSG00000226684	0	0	0	0
ENSG00000226685	0	0	0	0
ENSG00000226686	0	0	0	0
ENSG00000226687	0	0	0	0
ENSG00000226688	5	48	77	126
ENSG00000226690	0	0	0	1
ENSG00000226691	0	0	0	0
ENSG00000226693	0	0	0	0
ENSG00000226694	0	0	0	0
ENSG00000226695	0	0	0	0
ENSG00000226696	2	31	9	20
ENSG00000226698	1	0	1	0
ENSG00000226699	0	0	0	0
ENSG00000226700	0	0	0	0
ENSG00000226701	0	0	0	0
ENSG00000226702	0	0	0	0
ENSG00000226703	0	0	0	0
ENSG00000226704	0	0	0	0
ENSG00000226705	0	0	0	0
ENSG00000226706	0	0	0	0
ENSG00000226707	0	0	0	0

ENSG00000226708	11	34	31	9
ENSG00000226709	0	0	0	0
ENSG00000226710	0	0	0	0
ENSG00000226711	0	0	1	2
ENSG00000226713	0	0	0	0
ENSG00000226714	0	0	0	0
ENSG00000226715	0	0	0	0
ENSG00000226716	0	0	0	0
ENSG00000226717	0	0	0	0
ENSG00000226718	0	0	0	0
ENSG00000226721	0	0	0	2
ENSG00000226722	0	0	0	0
ENSG00000226723	0	0	0	2
ENSG00000226724	0	0	0	0
ENSG00000226725	0	0	0	0
ENSG00000226726	0	0	0	0
ENSG00000226727	0	0	0	0
ENSG00000226729	0	0	0	0
ENSG00000226732	0	0	0	0
ENSG00000226733	0	0	0	0
ENSG00000226734	0	0	0	0
ENSG00000226736	0	0	0	0
ENSG00000226738	0	0	0	0
ENSG00000226739	0	0	0	0
ENSG00000226740	0	0	0	0
ENSG00000226741	0	0	0	0
ENSG00000226742	21	37	8	19
ENSG00000226744	0	0	0	0
ENSG00000226745	0	0	0	0
ENSG00000226746	0	0	0	0
ENSG00000226747	0	0	0	0
ENSG00000226750	0	0	0	0
ENSG00000226751	0	0	1	0
ENSG00000226752	14	25	14	51
ENSG00000226753	0	0	0	0
ENSG00000226754	0	0	1	9
ENSG00000226755	0	0	0	0
ENSG00000226756	106	326	316	385
ENSG00000226757	0	0	0	0
ENSG00000226758	0	0	0	0
ENSG00000226759	0	0	0	0
ENSG00000226761	0	0	0	0
ENSG00000226762	5	0	0	0
ENSG00000226763	0	0	0	0
ENSG00000226764	0	0	0	0
ENSG00000226765	0	0	0	0

ENSG00000226766	0	0	0	0
ENSG00000226767	0	0	0	0
ENSG00000226768	0	0	0	0
ENSG00000226769	0	0	0	0
ENSG00000226770	1	0	0	0
ENSG00000226771	0	0	0	0
ENSG00000226772	0	0	0	0
ENSG00000226773	0	0	0	0
ENSG00000226774	0	0	0	0
ENSG00000226776	0	0	0	0
ENSG00000226777	155	240	255	251
ENSG00000226779	0	0	0	0
ENSG00000226780	0	0	0	0
ENSG00000226781	0	0	0	0
ENSG00000226782	0	0	0	0
ENSG00000226783	0	0	0	0
ENSG00000226784	0	1	0	1
ENSG00000226785	0	0	0	0
ENSG00000226786	0	0	0	0
ENSG00000226788	0	0	0	0
ENSG00000226789	0	0	0	0
ENSG00000226790	0	0	5	10
ENSG00000226791	1	0	0	0
ENSG00000226792	0	0	0	0
ENSG00000226794	0	0	0	0
ENSG00000226795	0	0	0	0
ENSG00000226796	0	0	0	0
ENSG00000226797	0	0	0	0
ENSG00000226798	0	0	0	0
ENSG00000226799	0	0	0	0
ENSG00000226800	4	0	3	11
ENSG00000226801	0	0	0	2
ENSG00000226802	0	0	0	0
ENSG00000226803	0	0	21	13
ENSG00000226804	0	0	0	0
ENSG00000226806	9	1	3	4
ENSG00000226807	0	0	0	0
ENSG00000226808	7	31	10	33
ENSG00000226810	0	0	0	0
ENSG00000226812	1	0	0	1
ENSG00000226813	0	0	0	0
ENSG00000226814	0	0	0	0
ENSG00000226816	1	21	0	3
ENSG00000226818	0	0	0	0
ENSG00000226819	3	5	6	1
ENSG00000226820	0	0	0	0

ENSG00000226822	39	51	257	226
ENSG00000226823	1	759	474	768
ENSG00000226824	47	155	93	88
ENSG00000226825	0	0	0	0
ENSG00000226826	0	0	0	0
ENSG00000226827	0	0	0	0
ENSG00000226828	1	0	0	0
ENSG00000226829	0	0	0	0
ENSG00000226830	0	0	0	0
ENSG00000226831	0	0	0	0
ENSG00000226832	0	0	0	0
ENSG00000226833	3	0	3	7
ENSG00000226834	0	0	0	0
ENSG00000226835	0	0	0	0
ENSG00000226836	0	0	0	0
ENSG00000226837	0	0	0	0
ENSG00000226838	0	0	0	0
ENSG00000226839	0	0	0	0
ENSG00000226840	0	0	0	0
ENSG00000226841	0	0	0	0
ENSG00000226842	0	0	0	0
ENSG00000226843	0	0	0	0
ENSG00000226844	0	0	0	0
ENSG00000226845	0	0	0	0
ENSG00000226846	2	0	0	0
ENSG00000226847	0	0	0	0
ENSG00000226849	0	0	1	0
ENSG00000226850	0	0	0	0
ENSG00000226851	0	0	0	0
ENSG00000226852	0	0	2	0
ENSG00000226853	4	0	1	1
ENSG00000226854	0	0	0	0
ENSG00000226855	0	0	0	0
ENSG00000226856	1	0	3	0
ENSG00000226857	0	0	0	0
ENSG00000226858	0	0	0	0
ENSG00000226859	0	0	0	0
ENSG00000226860	0	0	0	0
ENSG00000226861	0	0	0	0
ENSG00000226862	0	0	0	0
ENSG00000226863	0	0	0	0
ENSG00000226864	16	0	15	48
ENSG00000226867	0	0	0	0
ENSG00000226868	0	0	0	0
ENSG00000226869	0	0	0	2
ENSG00000226870	0	0	0	0

ENSG00000226871	0	0	0	0
ENSG00000226872	0	0	0	0
ENSG00000226873	0	0	0	0
ENSG00000226874	0	0	0	0
ENSG00000226875	0	0	0	0
ENSG00000226876	0	0	0	0
ENSG00000226877	0	0	0	0
ENSG00000226878	0	0	0	0
ENSG00000226879	0	0	0	0
ENSG00000226880	0	0	0	0
ENSG00000226881	1	0	0	0
ENSG00000226882	0	0	0	0
ENSG00000226883	0	0	0	0
ENSG00000226884	0	0	0	0
ENSG00000226885	0	0	0	0
ENSG00000226886	0	0	0	0
ENSG00000226887	0	0	0	0
ENSG00000226888	0	0	0	0
ENSG00000226889	0	0	0	0
ENSG00000226890	0	5	0	3
ENSG00000226891	0	69	29	25
ENSG00000226892	0	0	0	0
ENSG00000226893	0	0	0	0
ENSG00000226894	0	0	0	0
ENSG00000226896	0	0	0	0
ENSG00000226897	0	0	0	0
ENSG00000226898	0	0	0	0
ENSG00000226899	0	0	0	0
ENSG00000226900	127	88	4	20
ENSG00000226902	0	0	0	0
ENSG00000226903	0	0	0	0
ENSG00000226904	0	0	0	0
ENSG00000226905	0	0	0	0
ENSG00000226906	0	0	0	0
ENSG00000226907	0	0	0	0
ENSG00000226908	0	0	0	0
ENSG00000226912	0	0	0	0
ENSG00000226913	0	0	0	0
ENSG00000226914	0	0	0	0
ENSG00000226915	0	0	0	0
ENSG00000226916	0	0	0	0
ENSG00000226917	0	0	0	0
ENSG00000226918	0	0	0	0
ENSG00000226919	0	0	0	0
ENSG00000226920	2	0	0	0
ENSG00000226921	0	0	0	0

ENSG00000226923	0	0	0	0
ENSG00000226925	0	0	0	0
ENSG00000226926	0	0	0	0
ENSG00000226927	0	0	0	0
ENSG00000226928	0	4	0	0
ENSG00000226929	0	0	0	0
ENSG00000226930	0	0	0	0
ENSG00000226931	0	0	0	0
ENSG00000226932	0	0	0	0
ENSG00000226933	0	0	0	0
ENSG00000226935	0	0	0	0
ENSG00000226936	0	0	0	0
ENSG00000226937	0	0	7	2
ENSG00000226938	0	0	0	0
ENSG00000226939	0	0	0	0
ENSG00000226940	0	0	0	0
ENSG00000226941	0	0	0	0
ENSG00000226942	0	0	0	0
ENSG00000226943	1	0	0	2
ENSG00000226944	0	0	0	0
ENSG00000226945	0	0	0	0
ENSG00000226946	0	0	0	0
ENSG00000226947	0	0	0	0
ENSG00000226948	0	0	0	0
ENSG00000226949	0	0	0	0
ENSG00000226950	79	24	10	12
ENSG00000226952	0	0	0	0
ENSG00000226953	0	0	0	0
ENSG00000226954	1	0	0	0
ENSG00000226955	0	0	0	0
ENSG00000226956	0	0	0	0
ENSG00000226957	0	0	0	0
ENSG00000226958	660	1022	60	205
ENSG00000226960	0	0	0	0
ENSG00000226961	0	0	0	0
ENSG00000226962	0	0	0	0
ENSG00000226963	1	0	1	8
ENSG00000226964	0	0	0	0
ENSG00000226965	0	0	0	0
ENSG00000226967	0	0	0	0
ENSG00000226968	0	0	0	1
ENSG00000226969	0	0	0	0
ENSG00000226970	0	0	2	0
ENSG00000226971	0	0	0	0
ENSG00000226972	0	0	0	0
ENSG00000226973	0	0	0	0

ENSG00000226974	0	0	0	0
ENSG00000226975	0	0	0	0
ENSG00000226976	1	1	2	1
ENSG00000226977	0	0	0	0
ENSG00000226978	0	0	0	0
ENSG00000226979	80	55	26	62
ENSG00000226981	0	0	0	0
ENSG00000226982	0	0	0	0
ENSG00000226983	0	0	0	0
ENSG00000226984	0	0	0	0
ENSG00000226985	0	0	0	0
ENSG00000226986	0	0	0	0
ENSG00000226987	0	0	0	0
ENSG00000226989	0	0	0	0
ENSG00000226990	0	0	0	0
ENSG00000226991	0	0	0	0
ENSG00000226992	0	0	0	0
ENSG00000226993	0	0	0	0
ENSG00000226994	0	0	0	0
ENSG00000226995	0	0	0	0
ENSG00000226996	0	0	0	0
ENSG00000226998	0	0	0	0
ENSG00000226999	0	0	0	0
ENSG00000227000	0	0	0	0
ENSG00000227001	0	0	1	8
ENSG00000227002	0	0	0	0
ENSG00000227004	0	0	0	0
ENSG00000227005	0	0	0	0
ENSG00000227006	2	0	3	0
ENSG00000227007	0	0	1	0
ENSG00000227008	0	0	0	4
ENSG00000227009	0	0	0	0
ENSG00000227010	0	0	0	0
ENSG00000227011	0	0	0	0
ENSG00000227012	1	0	0	0
ENSG00000227013	0	0	0	0
ENSG00000227014	0	0	0	0
ENSG00000227015	0	0	0	0
ENSG00000227016	0	0	0	0
ENSG00000227017	0	0	0	0
ENSG00000227018	0	0	0	0
ENSG00000227019	0	0	0	0
ENSG00000227021	0	0	0	0
ENSG00000227023	0	0	0	0
ENSG00000227025	0	0	0	0
ENSG00000227027	0	0	0	0

ENSG00000227028	0	0	0	0
ENSG00000227029	0	0	0	0
ENSG00000227030	0	0	0	0
ENSG00000227032	0	0	0	0
ENSG00000227033	0	0	0	0
ENSG00000227034	0	0	0	0
ENSG00000227035	0	0	0	0
ENSG00000227036	0	0	0	0
ENSG00000227038	0	25	49	40
ENSG00000227039	6	0	0	0
ENSG00000227040	0	0	0	0
ENSG00000227042	0	68	30	68
ENSG00000227043	0	0	0	0
ENSG00000227044	0	0	0	0
ENSG00000227045	0	0	0	0
ENSG00000227046	0	0	0	0
ENSG00000227047	0	0	0	0
ENSG00000227048	0	0	0	0
ENSG00000227050	0	0	0	0
ENSG00000227051	0	0	0	0
ENSG00000227053	0	0	0	0
ENSG00000227054	0	0	0	0
ENSG00000227055	0	0	0	0
ENSG00000227056	0	0	0	0
ENSG00000227057	32	60	26	34
ENSG00000227058	0	0	0	0
ENSG00000227059	0	0	0	0
ENSG00000227060	13	90	7	10
ENSG00000227061	0	0	0	0
ENSG00000227062	0	0	0	0
ENSG00000227063	1	0	0	0
ENSG00000227064	0	0	0	0
ENSG00000227066	0	0	0	0
ENSG00000227067	0	0	0	0
ENSG00000227068	0	0	0	0
ENSG00000227069	0	0	0	0
ENSG00000227070	1	0	1	1
ENSG00000227071	6	47	6	5
ENSG00000227072	0	0	0	0
ENSG00000227073	0	0	0	0
ENSG00000227074	0	0	0	0
ENSG00000227075	0	0	0	0
ENSG00000227076	0	0	0	0
ENSG00000227077	0	0	0	0
ENSG00000227078	0	0	0	0
ENSG00000227080	0	0	0	0

ENSG00000227081	6	0	1	7
ENSG00000227082	0	0	0	0
ENSG00000227083	0	0	0	0
ENSG00000227084	0	0	0	0
ENSG00000227087	0	0	0	0
ENSG00000227088	0	0	0	0
ENSG00000227089	0	0	0	0
ENSG00000227090	0	0	0	0
ENSG00000227091	0	0	0	0
ENSG00000227094	0	0	0	0
ENSG00000227096	0	0	0	0
ENSG00000227097	339	24	5	37
ENSG00000227098	0	0	0	0
ENSG00000227099	0	0	0	0
ENSG00000227100	0	0	0	0
ENSG00000227101	0	13	0	0
ENSG00000227102	0	0	0	0
ENSG00000227104	0	0	0	0
ENSG00000227105	0	0	0	0
ENSG00000227107	0	0	0	0
ENSG00000227108	0	0	0	0
ENSG00000227109	0	0	0	0
ENSG00000227110	1	0	5	16
ENSG00000227112	0	0	0	0
ENSG00000227113	0	19	13	7
ENSG00000227114	0	0	1	6
ENSG00000227115	0	7	0	0
ENSG00000227116	0	0	0	0
ENSG00000227117	0	0	0	1
ENSG00000227118	0	0	2	0
ENSG00000227120	0	0	0	0
ENSG00000227121	0	0	0	0
ENSG00000227122	0	0	0	0
ENSG00000227123	0	0	0	0
ENSG00000227124	3	67	87	181
ENSG00000227125	0	0	0	0
ENSG00000227128	0	0	0	0
ENSG00000227129	0	0	0	0
ENSG00000227131	0	0	0	0
ENSG00000227132	0	0	0	0
ENSG00000227133	0	0	0	0
ENSG00000227134	0	0	0	0
ENSG00000227135	0	0	1	0
ENSG00000227136	0	0	0	0
ENSG00000227137	0	0	0	0
ENSG00000227138	0	0	0	0

ENSG00000227139	0	0	0	0
ENSG00000227140	0	0	0	0
ENSG00000227141	0	0	0	0
ENSG00000227143	0	0	0	0
ENSG00000227144	0	0	0	0
ENSG00000227145	0	0	0	0
ENSG00000227147	0	0	0	0
ENSG00000227148	0	0	0	0
ENSG00000227149	0	0	0	0
ENSG00000227150	0	0	0	0
ENSG00000227151	0	0	0	0
ENSG00000227152	0	0	0	0
ENSG00000227154	0	0	0	0
ENSG00000227155	0	0	0	0
ENSG00000227157	0	0	0	0
ENSG00000227158	0	0	0	0
ENSG00000227159	144	105	28	506
ENSG00000227160	0	0	0	0
ENSG00000227161	0	0	0	0
ENSG00000227163	0	0	0	0
ENSG00000227164	0	0	0	0
ENSG00000227165	3043	5028	5808	8354
ENSG00000227166	0	0	0	0
ENSG00000227167	0	0	0	0
ENSG00000227168	0	0	0	0
ENSG00000227169	0	0	0	0
ENSG00000227170	0	0	0	0
ENSG00000227171	0	0	0	0
ENSG00000227172	0	0	0	0
ENSG00000227173	0	0	0	0
ENSG00000227175	0	0	0	0
ENSG00000227176	0	0	0	0
ENSG00000227177	0	0	0	0
ENSG00000227178	0	0	0	0
ENSG00000227179	0	0	0	0
ENSG00000227180	0	0	0	0
ENSG00000227181	0	0	0	0
ENSG00000227182	0	0	0	0
ENSG00000227183	1	0	0	0
ENSG00000227184	0	0	0	0
ENSG00000227185	0	0	0	0
ENSG00000227186	0	0	0	0
ENSG00000227187	0	0	0	0
ENSG00000227188	0	0	0	0
ENSG00000227189	0	0	0	0
ENSG00000227191	8	0	1	0

ENSG00000227192	0	0	0	0
ENSG00000227193	0	0	0	0
ENSG00000227194	0	0	0	0
ENSG00000227195	0	0	0	0
ENSG00000227196	0	0	0	0
ENSG00000227197	0	0	0	0
ENSG00000227198	0	0	0	0
ENSG00000227199	49	74	84	54
ENSG00000227200	0	0	0	0
ENSG00000227201	0	0	0	0
ENSG00000227203	0	0	0	0
ENSG00000227204	0	0	0	0
ENSG00000227205	0	0	0	0
ENSG00000227206	0	0	0	0
ENSG00000227207	0	0	0	0
ENSG00000227208	0	0	0	0
ENSG00000227209	0	0	0	0
ENSG00000227210	0	0	0	0
ENSG00000227211	0	0	0	0
ENSG00000227212	0	0	0	0
ENSG00000227213	0	0	0	0
ENSG00000227214	5	0	0	0
ENSG00000227215	1	0	0	0
ENSG00000227216	0	0	0	0
ENSG00000227217	0	0	0	0
ENSG00000227218	0	0	0	0
ENSG00000227220	1	54	1	21
ENSG00000227222	0	0	0	0
ENSG00000227224	0	0	0	0
ENSG00000227225	0	0	0	0
ENSG00000227227	0	0	2	0
ENSG00000227228	0	0	0	0
ENSG00000227230	0	0	1	0
ENSG00000227231	0	0	0	0
ENSG00000227232	1	0	4	12
ENSG00000227233	0	0	0	0
ENSG00000227234	0	0	0	0
ENSG00000227236	0	0	0	0
ENSG00000227237	4	0	0	0
ENSG00000227238	0	0	0	0
ENSG00000227239	0	0	0	0
ENSG00000227240	0	0	0	4
ENSG00000227241	0	0	0	0
ENSG00000227242	0	2	0	0
ENSG00000227243	0	0	0	0
ENSG00000227244	0	0	1	0

ENSG00000227245	0	0	0	0
ENSG00000227246	0	0	0	0
ENSG00000227247	0	0	0	0
ENSG00000227248	0	0	0	0
ENSG00000227249	0	0	0	0
ENSG00000227251	0	0	0	0
ENSG00000227252	0	0	0	0
ENSG00000227253	0	0	0	0
ENSG00000227254	0	0	0	0
ENSG00000227255	0	0	0	0
ENSG00000227256	0	0	0	0
ENSG00000227257	0	0	0	0
ENSG00000227258	0	48	42	33
ENSG00000227259	0	0	0	0
ENSG00000227260	0	0	0	0
ENSG00000227261	0	0	0	0
ENSG00000227262	0	0	0	0
ENSG00000227264	0	0	0	0
ENSG00000227265	0	0	0	0
ENSG00000227267	0	0	0	0
ENSG00000227268	0	0	4	8
ENSG00000227269	0	0	0	0
ENSG00000227270	0	0	0	0
ENSG00000227271	0	0	0	0
ENSG00000227274	0	0	0	0
ENSG00000227275	0	0	0	0
ENSG00000227277	0	0	0	0
ENSG00000227278	0	0	0	0
ENSG00000227279	0	0	0	0
ENSG00000227280	0	0	0	0
ENSG00000227282	0	0	0	0
ENSG00000227283	0	0	0	0
ENSG00000227284	0	0	0	0
ENSG00000227285	1	0	0	0
ENSG00000227286	0	0	0	0
ENSG00000227288	0	1	0	0
ENSG00000227289	0	0	0	1
ENSG00000227290	0	0	1	0
ENSG00000227291	0	0	0	0
ENSG00000227292	0	0	0	0
ENSG00000227293	0	0	0	0
ENSG00000227294	0	0	0	0
ENSG00000227295	12	59	51	68
ENSG00000227296	0	0	0	0
ENSG00000227297	0	0	0	0
ENSG00000227298	0	0	0	0

ENSG00000227300	0	0	0	0
ENSG00000227301	0	0	0	0
ENSG00000227302	0	0	0	0
ENSG00000227303	0	0	0	0
ENSG00000227304	0	0	0	0
ENSG00000227305	0	0	0	0
ENSG00000227306	0	0	0	0
ENSG00000227307	0	0	0	0
ENSG00000227308	0	0	0	0
ENSG00000227309	0	0	0	0
ENSG00000227310	0	0	0	0
ENSG00000227311	0	0	0	0
ENSG00000227312	0	0	0	0
ENSG00000227313	0	0	0	0
ENSG00000227314	0	0	0	0
ENSG00000227315	0	0	0	0
ENSG00000227317	0	0	0	0
ENSG00000227318	1	14	29	14
ENSG00000227319	0	0	0	0
ENSG00000227321	0	0	0	0
ENSG00000227322	0	0	0	0
ENSG00000227325	0	0	0	0
ENSG00000227326	0	0	0	0
ENSG00000227329	0	0	0	0
ENSG00000227330	0	0	0	0
ENSG00000227331	0	0	6	3
ENSG00000227332	0	0	0	0
ENSG00000227333	0	0	0	0
ENSG00000227334	0	0	0	0
ENSG00000227335	0	0	0	0
ENSG00000227336	0	0	0	0
ENSG00000227337	0	0	0	0
ENSG00000227338	0	0	0	0
ENSG00000227339	0	0	0	0
ENSG00000227342	0	0	0	0
ENSG00000227343	0	0	0	0
ENSG00000227344	0	30	11	24
ENSG00000227345	5	62	64	64
ENSG00000227347	0	2	0	2
ENSG00000227348	0	0	0	0
ENSG00000227349	0	0	0	0
ENSG00000227350	0	0	0	0
ENSG00000227351	0	0	0	0
ENSG00000227352	0	0	0	0
ENSG00000227353	0	0	0	0
ENSG00000227354	2	0	3	11

ENSG00000227355	246	605	494	477
ENSG00000227356	0	0	0	0
ENSG00000227357	0	0	0	0
ENSG00000227358	0	0	0	0
ENSG00000227359	30	338	65	486
ENSG00000227360	0	0	0	0
ENSG00000227361	0	0	0	0
ENSG00000227363	0	0	0	0
ENSG00000227364	0	0	0	0
ENSG00000227365	0	0	0	0
ENSG00000227366	0	0	0	0
ENSG00000227367	0	0	0	0
ENSG00000227368	0	0	0	0
ENSG00000227370	0	0	2	0
ENSG00000227371	0	0	0	0
ENSG00000227372	4	15	8	17
ENSG00000227373	1	0	0	3
ENSG00000227374	0	0	0	0
ENSG00000227375	0	0	0	0
ENSG00000227376	19	6	9	4
ENSG00000227379	0	0	0	0
ENSG00000227380	0	0	0	0
ENSG00000227382	0	0	0	0
ENSG00000227383	0	0	0	0
ENSG00000227384	0	0	0	0
ENSG00000227386	0	0	0	0
ENSG00000227388	0	25	5	4
ENSG00000227389	0	0	0	0
ENSG00000227391	0	0	0	0
ENSG00000227392	0	0	0	0
ENSG00000227393	0	0	0	0
ENSG00000227394	0	0	0	0
ENSG00000227395	0	0	0	0
ENSG00000227397	0	0	0	0
ENSG00000227398	15	205	109	120
ENSG00000227399	0	0	0	0
ENSG00000227400	0	0	0	0
ENSG00000227401	0	0	0	0
ENSG00000227402	0	0	0	0
ENSG00000227403	1	0	11	17
ENSG00000227404	0	0	0	0
ENSG00000227405	0	0	0	0
ENSG00000227406	0	0	0	0
ENSG00000227407	0	0	0	0
ENSG00000227408	0	0	0	0
ENSG00000227409	0	158	187	230

ENSG00000227410	0	0	1	6
ENSG00000227411	0	0	0	0
ENSG00000227412	0	0	0	0
ENSG00000227413	0	0	0	0
ENSG00000227415	0	0	0	0
ENSG00000227416	0	0	0	0
ENSG00000227417	0	0	0	0
ENSG00000227418	0	0	0	0
ENSG00000227420	0	0	0	0
ENSG00000227421	0	0	0	0
ENSG00000227422	0	0	0	0
ENSG00000227423	0	0	0	0
ENSG00000227425	0	0	0	0
ENSG00000227426	0	0	0	0
ENSG00000227427	0	0	0	0
ENSG00000227429	0	0	0	0
ENSG00000227430	0	0	0	0
ENSG00000227431	0	0	0	0
ENSG00000227432	0	0	0	0
ENSG00000227433	0	0	0	0
ENSG00000227434	0	0	0	0
ENSG00000227435	0	0	0	0
ENSG00000227436	0	0	0	0
ENSG00000227437	0	0	0	0
ENSG00000227438	0	0	0	0
ENSG00000227439	0	0	0	0
ENSG00000227440	0	0	0	0
ENSG00000227442	0	0	0	0
ENSG00000227443	0	0	0	0
ENSG00000227444	0	0	0	0
ENSG00000227445	0	0	0	0
ENSG00000227446	0	0	0	0
ENSG00000227447	0	0	0	0
ENSG00000227448	0	0	0	0
ENSG00000227449	0	0	0	0
ENSG00000227450	0	0	0	0
ENSG00000227453	0	0	0	0
ENSG00000227454	0	0	0	0
ENSG00000227455	0	0	0	0
ENSG00000227456	0	0	0	0
ENSG00000227458	0	0	0	0
ENSG00000227459	0	0	0	0
ENSG00000227460	0	0	0	0
ENSG00000227462	0	0	0	0
ENSG00000227463	0	0	0	5
ENSG00000227465	0	0	0	0

ENSG00000227466	0	0	0	0
ENSG00000227467	2	0	0	1
ENSG00000227468	0	0	0	0
ENSG00000227469	0	0	0	0
ENSG00000227470	0	0	0	0
ENSG00000227471	0	0	0	0
ENSG00000227472	0	0	0	0
ENSG00000227473	0	0	0	0
ENSG00000227474	0	0	1	0
ENSG00000227475	0	0	0	0
ENSG00000227477	2	0	0	6
ENSG00000227479	0	0	0	0
ENSG00000227480	0	0	6	6
ENSG00000227481	0	0	0	0
ENSG00000227482	1	0	0	1
ENSG00000227484	0	0	0	0
ENSG00000227485	0	0	0	0
ENSG00000227486	1	0	21	38
ENSG00000227487	0	0	0	0
ENSG00000227488	0	0	0	0
ENSG00000227489	0	0	0	0
ENSG00000227490	0	0	0	0
ENSG00000227491	0	0	0	0
ENSG00000227492	0	0	0	0
ENSG00000227493	0	0	0	0
ENSG00000227494	0	0	0	0
ENSG00000227495	0	0	0	0
ENSG00000227496	0	0	1	34
ENSG00000227497	0	0	0	0
ENSG00000227498	0	0	0	0
ENSG00000227499	0	0	0	0
ENSG00000227500	0	35	1	2
ENSG00000227502	0	0	0	0
ENSG00000227505	0	0	0	0
ENSG00000227506	0	0	0	0
ENSG00000227507	45	3	5	1
ENSG00000227508	1	0	0	0
ENSG00000227509	0	0	0	0
ENSG00000227510	0	0	0	0
ENSG00000227511	0	0	0	0
ENSG00000227512	0	0	0	0
ENSG00000227513	0	0	0	0
ENSG00000227514	0	0	0	0
ENSG00000227515	0	0	0	0
ENSG00000227516	0	0	0	0
ENSG00000227517	12	0	16	38

ENSG00000227518	1	0	0	0
ENSG00000227519	0	0	0	0
ENSG00000227521	0	0	0	0
ENSG00000227523	0	0	0	0
ENSG00000227525	0	0	0	0
ENSG00000227526	0	0	0	0
ENSG00000227527	1	0	0	0
ENSG00000227528	0	0	1	3
ENSG00000227529	0	0	0	0
ENSG00000227531	0	0	1	6
ENSG00000227532	0	0	0	0
ENSG00000227533	0	4	3	4
ENSG00000227534	0	0	0	0
ENSG00000227535	0	0	0	0
ENSG00000227536	0	0	2	0
ENSG00000227537	0	0	0	0
ENSG00000227538	0	0	0	0
ENSG00000227540	0	0	2	0
ENSG00000227541	0	0	0	1
ENSG00000227542	0	0	0	0
ENSG00000227543	29	114	94	116
ENSG00000227544	3	0	1	2
ENSG00000227545	0	0	0	0
ENSG00000227547	0	0	0	0
ENSG00000227549	0	0	0	0
ENSG00000227550	82	355	247	114
ENSG00000227551	0	0	0	0
ENSG00000227552	0	0	0	0
ENSG00000227554	0	0	0	0
ENSG00000227555	0	0	1	0
ENSG00000227556	0	0	0	0
ENSG00000227557	0	0	0	0
ENSG00000227558	0	0	0	1
ENSG00000227559	0	0	0	0
ENSG00000227560	0	0	0	0
ENSG00000227562	0	0	0	0
ENSG00000227563	0	0	0	0
ENSG00000227564	0	0	0	0
ENSG00000227565	0	0	0	0
ENSG00000227567	0	0	0	0
ENSG00000227568	0	0	0	0
ENSG00000227569	0	0	0	0
ENSG00000227573	0	0	0	0
ENSG00000227574	0	0	0	0
ENSG00000227576	0	0	0	0
ENSG00000227577	0	0	0	0

ENSG00000227578	0	0	0	0
ENSG00000227579	0	0	0	0
ENSG00000227581	0	0	0	0
ENSG00000227582	0	0	1	6
ENSG00000227583	0	0	0	0
ENSG00000227584	0	0	0	0
ENSG00000227585	0	0	0	0
ENSG00000227586	0	0	0	0
ENSG00000227587	0	0	0	0
ENSG00000227588	0	0	0	0
ENSG00000227589	0	0	0	0
ENSG00000227590	0	0	0	0
ENSG00000227591	6	0	3	2
ENSG00000227592	0	0	0	0
ENSG00000227593	0	0	0	0
ENSG00000227596	0	0	0	0
ENSG00000227597	1	0	0	0
ENSG00000227598	0	0	0	4
ENSG00000227599	0	0	0	0
ENSG00000227600	0	0	0	0
ENSG00000227602	0	0	0	0
ENSG00000227603	1	0	1	5
ENSG00000227604	0	0	0	0
ENSG00000227606	0	0	0	0
ENSG00000227607	0	0	0	0
ENSG00000227608	0	0	0	0
ENSG00000227609	0	0	0	0
ENSG00000227610	0	0	0	0
ENSG00000227611	0	0	0	0
ENSG00000227613	1	0	3	0
ENSG00000227615	35	0	1	1
ENSG00000227616	0	0	0	0
ENSG00000227617	0	0	0	0
ENSG00000227619	0	0	0	0
ENSG00000227620	0	0	1	0
ENSG00000227621	0	0	0	0
ENSG00000227623	0	0	0	0
ENSG00000227624	0	0	0	0
ENSG00000227625	0	0	0	0
ENSG00000227626	0	0	0	0
ENSG00000227627	0	7	27	45
ENSG00000227628	0	0	0	0
ENSG00000227629	0	0	0	0
ENSG00000227630	2	0	0	0
ENSG00000227632	0	0	1	0
ENSG00000227633	0	0	0	0

ENSG00000227634	0	0	0	0
ENSG00000227635	0	0	0	0
ENSG00000227637	0	0	0	0
ENSG00000227638	0	0	0	0
ENSG00000227639	0	0	0	0
ENSG00000227640	2	37	18	2
ENSG00000227642	0	0	0	0
ENSG00000227644	0	0	0	0
ENSG00000227645	0	0	0	0
ENSG00000227646	0	0	0	0
ENSG00000227647	0	0	0	0
ENSG00000227649	0	0	0	0
ENSG00000227650	0	0	0	0
ENSG00000227652	0	0	0	0
ENSG00000227653	0	0	0	6
ENSG00000227655	0	0	0	0
ENSG00000227656	0	0	0	0
ENSG00000227657	0	0	0	0
ENSG00000227658	0	0	0	0
ENSG00000227659	0	0	0	0
ENSG00000227660	1	0	0	0
ENSG00000227661	0	0	0	0
ENSG00000227663	0	0	0	0
ENSG00000227665	0	0	0	0
ENSG00000227666	0	0	0	9
ENSG00000227667	0	0	0	0
ENSG00000227668	0	0	0	0
ENSG00000227669	0	0	0	0
ENSG00000227671	0	35	90	66
ENSG00000227672	0	0	0	0
ENSG00000227673	0	0	0	0
ENSG00000227674	0	191	10	21
ENSG00000227675	0	0	0	0
ENSG00000227676	0	0	0	0
ENSG00000227678	0	0	3	0
ENSG00000227679	0	0	0	0
ENSG00000227680	0	0	0	2
ENSG00000227681	0	0	0	0
ENSG00000227682	0	0	0	0
ENSG00000227683	1	0	16	11
ENSG00000227684	0	0	0	0
ENSG00000227685	0	0	0	0
ENSG00000227686	0	0	0	0
ENSG00000227687	0	0	0	0
ENSG00000227688	0	0	0	0
ENSG00000227689	0	0	0	0

ENSG00000227691	0	0	0	0
ENSG00000227692	2	0	0	0
ENSG00000227693	0	0	0	0
ENSG00000227694	0	0	0	0
ENSG00000227695	0	0	1	0
ENSG00000227698	0	0	0	0
ENSG00000227700	0	0	0	0
ENSG00000227702	0	0	0	0
ENSG00000227703	0	0	0	0
ENSG00000227704	0	0	0	0
ENSG00000227705	0	0	0	0
ENSG00000227706	2	0	0	0
ENSG00000227707	0	0	0	0
ENSG00000227708	0	0	0	0
ENSG00000227709	0	0	0	0
ENSG00000227710	0	0	0	0
ENSG00000227711	0	0	0	0
ENSG00000227712	0	0	0	0
ENSG00000227713	0	0	0	0
ENSG00000227714	0	0	0	0
ENSG00000227715	0	0	0	0
ENSG00000227716	0	0	0	0
ENSG00000227717	0	0	0	0
ENSG00000227718	0	0	0	0
ENSG00000227719	0	0	0	0
ENSG00000227720	0	0	0	0
ENSG00000227721	0	0	0	0
ENSG00000227722	0	0	1	0
ENSG00000227723	0	0	0	0
ENSG00000227725	0	0	0	0
ENSG00000227726	0	0	0	0
ENSG00000227728	0	0	0	0
ENSG00000227729	18	78	44	16
ENSG00000227730	0	0	0	0
ENSG00000227733	0	0	0	0
ENSG00000227734	0	0	0	0
ENSG00000227735	0	0	0	0
ENSG00000227736	0	0	0	0
ENSG00000227737	0	0	0	0
ENSG00000227738	0	0	0	0
ENSG00000227739	0	0	0	0
ENSG00000227740	0	0	2	4
ENSG00000227741	0	0	1	1
ENSG00000227742	0	0	1	1
ENSG00000227743	0	0	0	0
ENSG00000227744	0	0	0	0

ENSG00000227745	0	0	0	0
ENSG00000227746	0	0	0	0
ENSG00000227747	0	0	0	0
ENSG00000227748	0	0	0	0
ENSG00000227750	0	0	0	0
ENSG00000227751	0	0	7	1
ENSG00000227753	0	0	1	0
ENSG00000227754	0	0	0	0
ENSG00000227755	0	0	0	0
ENSG00000227757	0	0	0	0
ENSG00000227758	0	0	0	0
ENSG00000227759	0	0	0	0
ENSG00000227760	0	0	0	0
ENSG00000227761	0	0	0	0
ENSG00000227762	0	0	1	0
ENSG00000227764	0	0	0	2
ENSG00000227765	0	0	0	0
ENSG00000227766	0	2	80	52
ENSG00000227769	0	0	0	0
ENSG00000227770	0	0	0	0
ENSG00000227772	0	0	0	0
ENSG00000227773	0	15	11	27
ENSG00000227775	4	0	4	0
ENSG00000227776	0	0	0	0
ENSG00000227777	0	0	0	0
ENSG00000227778	0	0	0	0
ENSG00000227779	0	0	0	0
ENSG00000227780	0	0	0	0
ENSG00000227781	0	0	7	13
ENSG00000227782	0	0	4	0
ENSG00000227784	0	0	0	0
ENSG00000227785	0	0	0	0
ENSG00000227787	0	0	0	0
ENSG00000227788	0	0	0	0
ENSG00000227789	0	0	0	0
ENSG00000227790	0	0	0	0
ENSG00000227791	0	0	0	0
ENSG00000227792	0	0	0	0
ENSG00000227794	0	0	0	0
ENSG00000227795	0	0	0	0
ENSG00000227799	13	4	8	6
ENSG00000227800	0	0	0	0
ENSG00000227801	0	0	0	0
ENSG00000227802	0	0	0	0
ENSG00000227803	0	0	0	2
ENSG00000227804	0	0	0	0

ENSG00000227805	0	0	0	0
ENSG00000227806	0	0	0	0
ENSG00000227809	0	0	0	0
ENSG00000227811	168	2731	3029	1837
ENSG00000227813	2	9	2	0
ENSG00000227814	0	0	0	0
ENSG00000227815	0	0	10	7
ENSG00000227816	0	0	0	0
ENSG00000227817	0	0	0	0
ENSG00000227818	0	0	0	0
ENSG00000227821	0	0	0	0
ENSG00000227822	0	20	0	5
ENSG00000227824	0	0	0	0
ENSG00000227825	1	0	0	1
ENSG00000227826	0	0	0	0
ENSG00000227827	0	0	0	0
ENSG00000227830	0	0	0	0
ENSG00000227832	0	0	0	0
ENSG00000227834	0	0	0	0
ENSG00000227835	0	0	0	0
ENSG00000227836	0	0	0	0
ENSG00000227837	0	0	0	0
ENSG00000227838	0	0	0	0
ENSG00000227839	0	0	0	0
ENSG00000227840	0	0	0	0
ENSG00000227841	0	0	0	3
ENSG00000227842	0	0	0	0
ENSG00000227843	0	0	0	0
ENSG00000227844	1	0	0	1
ENSG00000227845	0	0	0	0
ENSG00000227846	0	0	0	0
ENSG00000227847	0	0	0	0
ENSG00000227848	0	0	0	0
ENSG00000227850	0	0	0	0
ENSG00000227851	0	0	0	0
ENSG00000227852	0	0	0	0
ENSG00000227853	0	0	0	0
ENSG00000227854	0	0	0	0
ENSG00000227855	0	0	14	10
ENSG00000227856	0	0	0	0
ENSG00000227857	0	0	0	0
ENSG00000227858	0	0	0	0
ENSG00000227859	0	0	0	0
ENSG00000227860	0	0	0	0
ENSG00000227861	0	0	0	0
ENSG00000227862	0	0	0	0

ENSG00000227863	0	0	0	0
ENSG00000227864	0	0	0	0
ENSG00000227867	0	0	0	0
ENSG00000227868	0	0	0	0
ENSG00000227869	0	0	0	0
ENSG00000227871	0	0	0	0
ENSG00000227873	0	0	0	0
ENSG00000227874	0	0	0	0
ENSG00000227875	0	0	0	0
ENSG00000227877	1	0	0	0
ENSG00000227878	0	0	0	0
ENSG00000227879	0	0	4	4
ENSG00000227880	0	0	0	0
ENSG00000227881	0	0	0	0
ENSG00000227882	0	0	0	0
ENSG00000227883	0	0	0	0
ENSG00000227884	0	0	0	0
ENSG00000227885	0	0	0	0
ENSG00000227886	0	0	0	0
ENSG00000227887	0	0	0	0
ENSG00000227888	0	0	0	0
ENSG00000227890	0	0	0	0
ENSG00000227892	0	0	0	0
ENSG00000227893	0	0	0	0
ENSG00000227895	1	0	0	0
ENSG00000227896	0	0	0	0
ENSG00000227899	0	0	0	0
ENSG00000227902	0	0	0	0
ENSG00000227905	1	0	0	0
ENSG00000227906	0	0	1	0
ENSG00000227907	0	0	0	0
ENSG00000227908	6	0	15	0
ENSG00000227910	0	0	5	0
ENSG00000227911	0	0	0	0
ENSG00000227912	0	0	0	0
ENSG00000227913	0	0	0	0
ENSG00000227914	0	0	0	0
ENSG00000227915	0	0	0	0
ENSG00000227917	0	0	0	0
ENSG00000227919	0	0	0	0
ENSG00000227920	0	0	0	0
ENSG00000227921	0	0	0	0
ENSG00000227922	0	0	0	0
ENSG00000227923	0	0	0	0
ENSG00000227924	0	0	0	0
ENSG00000227925	0	0	0	0

ENSG00000227927	0	6	6	1
ENSG00000227929	0	0	0	0
ENSG00000227930	0	0	0	0
ENSG00000227932	0	0	0	0
ENSG00000227933	0	0	0	0
ENSG00000227934	0	0	1	2
ENSG00000227935	0	0	0	0
ENSG00000227936	0	0	0	0
ENSG00000227937	0	0	0	0
ENSG00000227938	0	0	0	0
ENSG00000227939	3	0	0	0
ENSG00000227940	0	0	0	0
ENSG00000227941	0	0	0	0
ENSG00000227942	0	0	0	0
ENSG00000227945	0	0	0	0
ENSG00000227946	6	0	8	6
ENSG00000227947	0	0	0	0
ENSG00000227948	0	0	0	0
ENSG00000227949	0	0	0	0
ENSG00000227950	0	0	0	0
ENSG00000227952	0	0	0	0
ENSG00000227953	10	132	137	43
ENSG00000227954	0	0	0	0
ENSG00000227956	0	0	0	0
ENSG00000227958	0	0	0	0
ENSG00000227959	0	0	0	0
ENSG00000227960	0	0	0	0
ENSG00000227961	0	0	0	0
ENSG00000227962	0	0	0	0
ENSG00000227963	0	0	0	0
ENSG00000227964	0	0	0	0
ENSG00000227965	0	0	0	0
ENSG00000227966	0	0	0	0
ENSG00000227968	0	0	0	0
ENSG00000227969	0	0	0	0
ENSG00000227970	0	0	0	0
ENSG00000227971	0	0	0	0
ENSG00000227972	5	0	1	0
ENSG00000227973	0	0	0	0
ENSG00000227974	0	0	0	0
ENSG00000227979	0	0	0	0
ENSG00000227981	0	0	0	0
ENSG00000227982	0	0	0	0
ENSG00000227983	0	0	0	0
ENSG00000227986	0	0	4	0
ENSG00000227987	0	0	0	0

ENSG00000227988	0	0	0	0
ENSG00000227989	0	0	0	0
ENSG00000227992	22	48	89	49
ENSG00000227993	0	0	0	0
ENSG00000227994	0	0	0	0
ENSG00000227995	0	0	0	0
ENSG00000227997	0	0	0	0
ENSG00000227999	0	0	0	0
ENSG00000228000	0	0	0	0
ENSG00000228001	0	0	0	0
ENSG00000228002	0	0	0	0
ENSG00000228004	0	0	0	0
ENSG00000228005	0	0	0	0
ENSG00000228007	0	0	0	0
ENSG00000228008	0	0	0	5
ENSG00000228010	0	0	0	0
ENSG00000228011	0	0	0	0
ENSG00000228012	0	0	0	0
ENSG00000228013	3	0	5	1
ENSG00000228014	0	0	0	0
ENSG00000228015	0	0	0	0
ENSG00000228016	0	0	0	0
ENSG00000228019	0	0	0	0
ENSG00000228020	0	0	0	0
ENSG00000228021	0	0	0	0
ENSG00000228022	0	0	0	0
ENSG00000228024	0	0	0	0
ENSG00000228025	0	0	0	0
ENSG00000228027	0	0	0	0
ENSG00000228028	0	0	0	0
ENSG00000228029	0	0	0	0
ENSG00000228030	0	0	0	0
ENSG00000228031	0	20	6	13
ENSG00000228032	0	0	0	0
ENSG00000228033	0	0	0	0
ENSG00000228034	0	0	0	0
ENSG00000228035	0	0	0	0
ENSG00000228036	0	0	0	0
ENSG00000228037	21	29	22	14
ENSG00000228038	0	0	0	0
ENSG00000228039	0	0	0	0
ENSG00000228040	0	0	0	0
ENSG00000228041	0	0	0	0
ENSG00000228042	0	0	0	0
ENSG00000228043	0	0	1	21
ENSG00000228044	0	0	0	0

ENSG00000228045	0	0	0	0
ENSG00000228046	0	0	0	0
ENSG00000228048	0	0	0	0
ENSG00000228049	0	0	0	2
ENSG00000228050	0	0	0	0
ENSG00000228051	0	0	0	0
ENSG00000228053	1	0	0	0
ENSG00000228054	0	0	0	0
ENSG00000228055	3	11	13	5
ENSG00000228056	0	0	0	0
ENSG00000228057	0	0	1	1
ENSG00000228058	0	0	0	0
ENSG00000228060	0	0	0	0
ENSG00000228061	0	0	0	0
ENSG00000228063	473	6417	11372	3423
ENSG00000228064	0	0	0	0
ENSG00000228065	0	39	34	159
ENSG00000228067	0	0	0	0
ENSG00000228069	0	0	0	0
ENSG00000228071	0	0	0	0
ENSG00000228072	0	0	0	0
ENSG00000228073	0	0	0	0
ENSG00000228074	0	0	0	0
ENSG00000228075	0	0	0	0
ENSG00000228076	0	0	0	0
ENSG00000228078	0	0	0	0
ENSG00000228079	0	0	0	0
ENSG00000228080	0	0	0	0
ENSG00000228081	0	0	0	0
ENSG00000228082	0	0	0	0
ENSG00000228083	0	0	0	0
ENSG00000228084	0	0	0	0
ENSG00000228085	0	0	0	0
ENSG00000228086	0	125	131	102
ENSG00000228089	0	0	0	0
ENSG00000228090	0	0	0	0
ENSG00000228092	0	0	0	0
ENSG00000228095	0	0	0	0
ENSG00000228097	0	0	0	0
ENSG00000228098	0	0	0	0
ENSG00000228099	0	0	0	0
ENSG00000228100	0	0	0	0
ENSG00000228102	0	0	0	0
ENSG00000228104	0	0	0	0
ENSG00000228105	0	0	0	0
ENSG00000228106	17	97	155	376

ENSG00000228107	2	0	0	0
ENSG00000228108	0	0	0	0
ENSG00000228109	0	0	0	0
ENSG00000228110	0	0	1	0
ENSG00000228111	0	0	0	0
ENSG00000228112	0	0	0	0
ENSG00000228113	1	0	8	1
ENSG00000228115	0	0	0	0
ENSG00000228116	0	0	0	0
ENSG00000228118	0	0	0	0
ENSG00000228120	0	0	0	0
ENSG00000228121	0	0	0	0
ENSG00000228122	0	7	0	0
ENSG00000228123	0	0	0	0
ENSG00000228124	0	0	8	10
ENSG00000228125	0	0	0	0
ENSG00000228126	0	0	0	0
ENSG00000228127	0	0	2	3
ENSG00000228128	0	0	0	0
ENSG00000228130	0	0	0	0
ENSG00000228131	0	0	0	0
ENSG00000228132	0	0	0	0
ENSG00000228133	0	0	0	0
ENSG00000228134	0	0	0	0
ENSG00000228135	0	0	0	0
ENSG00000228136	0	0	0	0
ENSG00000228137	0	0	0	0
ENSG00000228139	0	0	0	0
ENSG00000228140	2	0	0	0
ENSG00000228141	0	0	0	0
ENSG00000228142	0	0	0	0
ENSG00000228143	0	0	0	0
ENSG00000228144	0	0	0	0
ENSG00000228146	1	0	0	0
ENSG00000228147	0	0	0	0
ENSG00000228149	0	0	0	0
ENSG00000228150	3	0	3	5
ENSG00000228151	0	0	0	0
ENSG00000228153	0	0	0	0
ENSG00000228154	0	0	0	0
ENSG00000228155	0	0	0	0
ENSG00000228156	0	0	0	0
ENSG00000228157	0	0	0	0
ENSG00000228158	0	0	0	0
ENSG00000228159	0	0	0	0
ENSG00000228160	0	0	0	0

ENSG00000228161	0	0	0	0
ENSG00000228162	0	0	0	0
ENSG00000228163	0	0	0	0
ENSG00000228164	0	0	0	0
ENSG00000228165	0	0	0	0
ENSG00000228166	0	0	0	0
ENSG00000228167	0	0	0	0
ENSG00000228168	0	0	0	0
ENSG00000228169	0	0	0	0
ENSG00000228170	0	0	0	0
ENSG00000228171	0	0	0	0
ENSG00000228172	0	0	9	0
ENSG00000228173	0	0	0	0
ENSG00000228174	0	0	0	0
ENSG00000228175	0	0	2	7
ENSG00000228176	0	0	0	0
ENSG00000228177	0	0	0	0
ENSG00000228179	0	0	0	0
ENSG00000228181	0	0	0	0
ENSG00000228182	0	0	0	0
ENSG00000228184	0	0	0	0
ENSG00000228186	0	0	0	0
ENSG00000228187	0	0	0	0
ENSG00000228189	0	0	0	0
ENSG00000228190	0	0	0	0
ENSG00000228191	0	0	0	0
ENSG00000228192	0	0	2	2
ENSG00000228193	0	0	0	0
ENSG00000228194	0	0	0	0
ENSG00000228195	0	0	0	0
ENSG00000228196	0	0	0	7
ENSG00000228197	0	0	0	0
ENSG00000228198	0	0	0	0
ENSG00000228199	0	0	0	0
ENSG00000228201	0	0	0	0
ENSG00000228202	0	0	0	0
ENSG00000228203	0	0	0	0
ENSG00000228204	0	0	0	0
ENSG00000228205	0	0	0	0
ENSG00000228206	0	0	0	0
ENSG00000228207	0	0	0	0
ENSG00000228208	0	0	10	0
ENSG00000228209	0	0	0	0
ENSG00000228210	0	0	0	0
ENSG00000228211	0	0	0	0
ENSG00000228212	0	0	0	0

ENSG00000228213	0	0	0	0
ENSG00000228214	0	0	0	0
ENSG00000228215	118	706	989	784
ENSG00000228216	0	0	0	0
ENSG00000228217	0	0	0	0
ENSG00000228218	7	0	1	1
ENSG00000228219	0	0	0	0
ENSG00000228220	0	0	0	0
ENSG00000228221	0	0	0	5
ENSG00000228222	0	0	4	0
ENSG00000228223	47	123	130	145
ENSG00000228224	0	0	0	0
ENSG00000228225	0	0	0	0
ENSG00000228226	0	0	0	0
ENSG00000228227	0	0	0	0
ENSG00000228229	0	0	0	0
ENSG00000228231	0	0	0	0
ENSG00000228232	0	0	0	0
ENSG00000228234	0	0	0	0
ENSG00000228235	0	0	0	0
ENSG00000228236	0	0	0	0
ENSG00000228237	0	1	12	9
ENSG00000228238	0	0	0	0
ENSG00000228239	0	0	0	0
ENSG00000228240	0	0	0	0
ENSG00000228241	0	0	0	0
ENSG00000228242	0	0	0	0
ENSG00000228243	0	0	1	1
ENSG00000228245	0	0	0	0
ENSG00000228247	0	0	0	0
ENSG00000228248	0	0	0	0
ENSG00000228250	0	0	0	0
ENSG00000228251	0	0	2	0
ENSG00000228252	0	0	4	0
ENSG00000228253	5660	7097	2714	6835
ENSG00000228254	0	0	0	0
ENSG00000228255	0	0	0	0
ENSG00000228257	0	0	0	0
ENSG00000228259	0	0	0	0
ENSG00000228260	0	0	0	0
ENSG00000228261	0	26	0	0
ENSG00000228262	0	0	0	0
ENSG00000228263	0	0	0	0
ENSG00000228264	0	0	0	0
ENSG00000228265	10	6	0	4
ENSG00000228267	0	0	0	0

ENSG00000228271	0	0	0	0
ENSG00000228272	0	0	0	0
ENSG00000228273	0	0	0	0
ENSG00000228274	0	0	0	0
ENSG00000228275	0	34	34	21
ENSG00000228277	0	0	0	0
ENSG00000228278	0	0	0	0
ENSG00000228280	0	0	0	1
ENSG00000228283	0	0	0	0
ENSG00000228284	0	0	0	0
ENSG00000228285	0	0	0	0
ENSG00000228286	0	0	0	0
ENSG00000228288	0	15	0	0
ENSG00000228289	0	0	0	0
ENSG00000228290	0	0	0	0
ENSG00000228291	0	0	0	0
ENSG00000228292	0	0	0	0
ENSG00000228293	0	0	0	0
ENSG00000228294	0	0	0	0
ENSG00000228295	0	0	0	0
ENSG00000228296	0	0	0	0
ENSG00000228299	0	0	0	0
ENSG00000228300	0	11	0	0
ENSG00000228301	0	0	0	0
ENSG00000228302	0	0	3	0
ENSG00000228303	0	0	0	0
ENSG00000228304	0	0	0	0
ENSG00000228305	0	0	0	0
ENSG00000228307	0	0	0	0
ENSG00000228308	0	0	0	0
ENSG00000228309	0	0	0	0
ENSG00000228312	0	0	0	0
ENSG00000228313	2	19	9	7
ENSG00000228314	0	0	2	1
ENSG00000228315	3	59	30	78
ENSG00000228316	0	0	0	0
ENSG00000228317	0	0	0	0
ENSG00000228318	0	0	0	0
ENSG00000228319	0	0	0	0
ENSG00000228321	0	0	0	0
ENSG00000228322	0	0	0	0
ENSG00000228323	0	0	0	0
ENSG00000228325	0	0	0	0
ENSG00000228326	0	0	0	0
ENSG00000228327	0	0	4	8
ENSG00000228328	0	0	0	0

ENSG00000228329	0	0	0	0
ENSG00000228330	0	0	0	0
ENSG00000228331	0	0	0	0
ENSG00000228333	0	0	0	0
ENSG00000228334	0	0	0	0
ENSG00000228335	1	8	50	68
ENSG00000228336	0	0	0	0
ENSG00000228337	0	0	0	0
ENSG00000228338	0	0	0	0
ENSG00000228339	1	0	4	5
ENSG00000228340	22	1020	1303	1420
ENSG00000228341	0	0	0	0
ENSG00000228343	1	0	0	0
ENSG00000228345	0	0	0	0
ENSG00000228347	0	0	0	0
ENSG00000228348	0	0	0	2
ENSG00000228349	0	0	0	0
ENSG00000228350	0	0	0	0
ENSG00000228351	0	0	0	0
ENSG00000228352	0	0	0	0
ENSG00000228353	0	0	0	0
ENSG00000228354	0	0	0	0
ENSG00000228355	0	0	0	0
ENSG00000228357	0	0	0	0
ENSG00000228358	0	0	0	0
ENSG00000228360	0	0	0	0
ENSG00000228361	0	0	0	0
ENSG00000228363	7	128	62	69
ENSG00000228364	0	0	0	0
ENSG00000228365	0	0	0	0
ENSG00000228366	0	0	0	0
ENSG00000228367	0	0	0	0
ENSG00000228368	0	0	0	0
ENSG00000228369	0	0	0	0
ENSG00000228370	0	0	0	0
ENSG00000228371	0	0	0	0
ENSG00000228372	0	0	0	0
ENSG00000228373	0	0	0	0
ENSG00000228374	0	0	1	0
ENSG00000228375	0	0	0	0
ENSG00000228376	0	0	0	0
ENSG00000228377	0	0	0	0
ENSG00000228379	0	0	0	0
ENSG00000228380	0	0	0	0
ENSG00000228382	1	0	0	0
ENSG00000228383	0	0	0	0

ENSG00000228384	0	0	0	0
ENSG00000228386	0	0	0	0
ENSG00000228389	6	1	7	4
ENSG00000228391	0	0	0	0
ENSG00000228392	0	0	0	0
ENSG00000228393	1	0	8	5
ENSG00000228395	0	10	8	15
ENSG00000228397	3	0	1	0
ENSG00000228398	0	0	0	0
ENSG00000228399	0	0	0	0
ENSG00000228400	0	0	0	0
ENSG00000228401	0	0	0	0
ENSG00000228403	0	0	0	0
ENSG00000228404	0	0	0	0
ENSG00000228405	0	0	0	0
ENSG00000228407	0	0	0	0
ENSG00000228408	0	0	0	0
ENSG00000228409	225	2895	1743	1828
ENSG00000228410	0	0	0	0
ENSG00000228411	0	0	0	0
ENSG00000228412	0	0	0	0
ENSG00000228413	0	0	0	0
ENSG00000228414	0	0	0	0
ENSG00000228415	0	0	0	0
ENSG00000228417	0	0	0	0
ENSG00000228418	0	0	3	5
ENSG00000228420	1	0	0	0
ENSG00000228421	0	0	0	0
ENSG00000228422	0	0	0	0
ENSG00000228423	0	0	0	0
ENSG00000228425	0	0	0	0
ENSG00000228426	0	0	0	0
ENSG00000228427	1	0	0	0
ENSG00000228429	0	0	0	0
ENSG00000228430	0	0	0	5
ENSG00000228431	0	0	0	0
ENSG00000228432	0	0	0	0
ENSG00000228433	0	0	0	0
ENSG00000228434	0	0	0	0
ENSG00000228435	0	0	0	0
ENSG00000228436	2	3	2	10
ENSG00000228437	0	3	2	3
ENSG00000228438	0	0	0	0
ENSG00000228439	2	311	330	482
ENSG00000228440	0	0	0	0
ENSG00000228444	0	0	0	0

ENSG00000228445	0	0	0	0
ENSG00000228446	0	0	0	0
ENSG00000228448	0	0	0	0
ENSG00000228450	0	0	0	0
ENSG00000228451	26	127	88	115
ENSG00000228452	1	0	0	0
ENSG00000228453	0	0	0	0
ENSG00000228454	0	0	0	0
ENSG00000228458	0	0	0	0
ENSG00000228459	0	0	0	0
ENSG00000228460	0	0	0	0
ENSG00000228461	0	0	0	0
ENSG00000228462	4	0	0	0
ENSG00000228463	8	419	139	965
ENSG00000228464	0	0	0	0
ENSG00000228465	0	0	0	0
ENSG00000228466	0	0	0	0
ENSG00000228467	0	0	0	0
ENSG00000228469	0	0	0	0
ENSG00000228470	0	0	0	0
ENSG00000228471	0	0	0	0
ENSG00000228473	0	0	0	0
ENSG00000228474	140467	71471	38914	70417
ENSG00000228476	0	0	0	0
ENSG00000228477	0	0	0	0
ENSG00000228478	0	0	0	0
ENSG00000228480	0	0	0	0
ENSG00000228481	0	0	0	0
ENSG00000228482	0	0	0	0
ENSG00000228483	0	0	0	0
ENSG00000228484	0	0	0	0
ENSG00000228485	3	13	10	6
ENSG00000228486	7	0	23	25
ENSG00000228487	0	0	0	0
ENSG00000228488	0	0	0	0
ENSG00000228489	0	0	0	0
ENSG00000228490	0	0	0	0
ENSG00000228491	0	0	0	0
ENSG00000228492	5	11	1	4
ENSG00000228494	0	0	0	0
ENSG00000228495	0	27	26	21
ENSG00000228496	0	0	0	3
ENSG00000228498	0	0	0	0
ENSG00000228499	0	0	0	0
ENSG00000228501	0	0	0	0
ENSG00000228502	2	0	0	0

ENSG00000228503	0	0	0	0
ENSG00000228504	0	0	0	0
ENSG00000228505	0	0	0	0
ENSG00000228506	0	29	19	0
ENSG00000228507	0	0	0	1
ENSG00000228509	0	0	3	2
ENSG00000228510	0	0	0	0
ENSG00000228512	0	0	0	0
ENSG00000228513	0	0	0	0
ENSG00000228515	0	0	0	0
ENSG00000228517	0	0	0	0
ENSG00000228518	0	0	0	0
ENSG00000228519	0	0	0	0
ENSG00000228520	0	0	0	0
ENSG00000228521	0	0	0	0
ENSG00000228522	0	0	0	0
ENSG00000228523	0	0	0	0
ENSG00000228524	0	0	0	0
ENSG00000228525	0	0	0	0
ENSG00000228526	0	0	0	0
ENSG00000228527	0	0	0	0
ENSG00000228528	0	0	0	0
ENSG00000228530	0	0	0	0
ENSG00000228532	0	0	0	0
ENSG00000228534	0	0	0	0
ENSG00000228536	2	0	1	3
ENSG00000228537	0	0	0	0
ENSG00000228538	0	0	1	0
ENSG00000228539	0	0	0	0
ENSG00000228540	0	0	0	3
ENSG00000228541	282	120	27	96
ENSG00000228543	0	0	0	0
ENSG00000228544	0	0	0	0
ENSG00000228546	1	31	22	28
ENSG00000228547	0	0	0	0
ENSG00000228548	0	0	0	0
ENSG00000228549	0	0	0	3
ENSG00000228550	0	0	0	0
ENSG00000228551	0	0	0	0
ENSG00000228553	0	0	0	0
ENSG00000228554	0	0	0	0
ENSG00000228557	0	0	0	0
ENSG00000228559	0	0	0	0
ENSG00000228560	0	0	0	0
ENSG00000228561	0	0	0	6
ENSG00000228563	0	0	0	0

ENSG00000228564	0	0	0	0
ENSG00000228566	0	0	0	0
ENSG00000228567	0	0	0	0
ENSG00000228568	0	0	0	0
ENSG00000228569	0	0	0	0
ENSG00000228570	0	0	0	0
ENSG00000228571	0	0	0	0
ENSG00000228572	0	0	0	0
ENSG00000228573	0	0	2	0
ENSG00000228575	0	0	0	0
ENSG00000228577	0	0	0	0
ENSG00000228578	0	0	0	0
ENSG00000228579	0	0	0	0
ENSG00000228581	0	0	0	0
ENSG00000228582	0	0	0	0
ENSG00000228583	0	0	0	0
ENSG00000228585	0	0	0	0
ENSG00000228586	0	0	0	0
ENSG00000228587	0	0	0	0
ENSG00000228589	2	0	8	0
ENSG00000228590	0	0	1	0
ENSG00000228592	0	0	0	0
ENSG00000228593	0	0	0	0
ENSG00000228594	10	0	1	1
ENSG00000228595	0	0	0	0
ENSG00000228596	0	0	0	0
ENSG00000228597	0	0	0	0
ENSG00000228598	0	0	0	0
ENSG00000228599	0	0	0	0
ENSG00000228600	0	0	0	0
ENSG00000228601	0	2	4	1
ENSG00000228604	0	0	0	0
ENSG00000228605	0	0	0	0
ENSG00000228606	22	67	43	27
ENSG00000228607	0	0	0	0
ENSG00000228609	1	0	2	2
ENSG00000228610	0	0	0	0
ENSG00000228611	0	0	0	0
ENSG00000228612	0	0	0	0
ENSG00000228613	0	0	0	0
ENSG00000228614	0	0	0	0
ENSG00000228615	0	0	0	0
ENSG00000228616	0	0	0	0
ENSG00000228617	0	0	0	0
ENSG00000228618	0	0	0	0
ENSG00000228620	0	0	0	0

ENSG00000228622	0	0	0	0
ENSG00000228623	1	0	0	0
ENSG00000228624	5	0	16	19
ENSG00000228625	0	0	0	0
ENSG00000228626	0	0	0	0
ENSG00000228627	0	0	0	0
ENSG00000228628	0	0	0	0
ENSG00000228629	0	0	0	0
ENSG00000228630	0	0	0	0
ENSG00000228634	0	0	0	0
ENSG00000228635	0	0	0	0
ENSG00000228636	0	0	0	0
ENSG00000228638	3	0	0	2
ENSG00000228639	0	0	0	0
ENSG00000228643	1	0	11	11
ENSG00000228645	0	0	0	0
ENSG00000228646	0	0	0	0
ENSG00000228648	0	0	0	0
ENSG00000228649	36	194	163	66
ENSG00000228650	0	0	0	0
ENSG00000228651	968	555	247	543
ENSG00000228652	0	0	0	0
ENSG00000228653	0	0	0	0
ENSG00000228655	0	0	0	0
ENSG00000228656	0	0	0	0
ENSG00000228657	0	0	0	0
ENSG00000228658	0	0	0	0
ENSG00000228659	0	0	0	0
ENSG00000228660	0	0	0	0
ENSG00000228661	1	0	0	1
ENSG00000228662	0	0	0	0
ENSG00000228663	0	0	0	0
ENSG00000228664	0	0	0	0
ENSG00000228665	1	0	0	0
ENSG00000228666	0	0	0	0
ENSG00000228667	0	0	0	0
ENSG00000228668	1	0	6	8
ENSG00000228669	0	0	0	0
ENSG00000228670	0	0	0	0
ENSG00000228672	0	0	0	0
ENSG00000228674	0	0	0	0
ENSG00000228675	0	0	0	0
ENSG00000228677	0	0	1	7
ENSG00000228679	0	0	0	0
ENSG00000228680	0	0	0	0
ENSG00000228681	0	0	0	0

ENSG00000228682	0	0	0	0
ENSG00000228683	0	0	0	0
ENSG00000228684	0	0	0	0
ENSG00000228685	0	0	0	0
ENSG00000228686	0	0	0	2
ENSG00000228687	0	0	0	0
ENSG00000228688	0	0	0	0
ENSG00000228689	0	0	0	0
ENSG00000228691	0	0	0	0
ENSG00000228692	1	0	0	0
ENSG00000228694	0	0	0	5
ENSG00000228695	0	0	0	0
ENSG00000228696	4	19	26	3
ENSG00000228697	0	0	0	0
ENSG00000228698	0	0	0	0
ENSG00000228700	0	0	0	0
ENSG00000228701	15	33	32	23
ENSG00000228702	0	0	0	0
ENSG00000228703	0	4	0	0
ENSG00000228704	0	0	0	0
ENSG00000228705	0	0	0	0
ENSG00000228706	0	0	0	0
ENSG00000228707	0	0	0	0
ENSG00000228708	0	0	0	0
ENSG00000228709	0	0	0	0
ENSG00000228711	0	0	0	0
ENSG00000228712	0	0	0	0
ENSG00000228713	0	0	0	0
ENSG00000228714	0	0	0	0
ENSG00000228716	32	25	28	29
ENSG00000228717	0	1	0	0
ENSG00000228718	0	0	0	0
ENSG00000228719	4	0	11	12
ENSG00000228721	0	0	0	0
ENSG00000228723	0	0	0	0
ENSG00000228725	0	0	0	0
ENSG00000228727	0	0	0	0
ENSG00000228728	0	0	0	0
ENSG00000228729	0	0	0	0
ENSG00000228730	0	0	0	0
ENSG00000228732	0	0	0	0
ENSG00000228733	0	0	0	0
ENSG00000228734	0	0	0	0
ENSG00000228735	0	0	0	0
ENSG00000228736	0	0	0	0
ENSG00000228737	1	0	6	15

ENSG00000228738	0	0	0	0
ENSG00000228739	0	0	0	0
ENSG00000228740	0	0	0	0
ENSG00000228741	0	0	3	0
ENSG00000228742	28	136	88	65
ENSG00000228744	0	0	0	0
ENSG00000228745	0	0	0	0
ENSG00000228748	0	0	0	0
ENSG00000228750	0	0	0	0
ENSG00000228751	0	0	0	0
ENSG00000228753	0	0	0	0
ENSG00000228754	0	0	0	0
ENSG00000228755	0	0	0	0
ENSG00000228757	0	0	0	0
ENSG00000228759	0	0	0	0
ENSG00000228760	0	0	0	0
ENSG00000228762	0	0	0	0
ENSG00000228763	0	0	0	0
ENSG00000228764	0	0	0	0
ENSG00000228765	0	0	0	0
ENSG00000228766	0	0	0	0
ENSG00000228767	0	0	0	0
ENSG00000228768	0	0	0	0
ENSG00000228769	0	0	0	0
ENSG00000228770	0	0	0	0
ENSG00000228771	0	0	0	0
ENSG00000228772	0	0	0	0
ENSG00000228775	1	0	7	1
ENSG00000228776	0	0	0	0
ENSG00000228777	0	0	0	0
ENSG00000228778	0	0	0	0
ENSG00000228779	0	0	0	0
ENSG00000228780	0	0	0	0
ENSG00000228781	0	0	0	0
ENSG00000228782	3	25	31	38
ENSG00000228783	0	0	0	0
ENSG00000228784	2	0	7	0
ENSG00000228785	0	0	0	0
ENSG00000228786	0	0	0	3
ENSG00000228787	0	0	0	0
ENSG00000228789	0	0	0	0
ENSG00000228790	0	0	0	0
ENSG00000228791	0	0	0	0
ENSG00000228792	0	0	0	0
ENSG00000228793	0	0	0	0
ENSG00000228794	3	16	8	9

ENSG00000228797	0	0	0	0
ENSG00000228798	0	0	0	0
ENSG00000228799	0	0	0	0
ENSG00000228800	0	0	0	1
ENSG00000228801	3	0	4	10
ENSG00000228802	0	0	0	0
ENSG00000228803	0	0	0	0
ENSG00000228804	0	0	0	0
ENSG00000228806	0	0	0	0
ENSG00000228807	0	0	0	0
ENSG00000228808	0	0	0	0
ENSG00000228809	0	0	0	0
ENSG00000228810	0	0	0	0
ENSG00000228812	0	0	0	0
ENSG00000228813	0	0	0	0
ENSG00000228814	0	0	0	0
ENSG00000228815	0	0	1	0
ENSG00000228816	0	0	0	0
ENSG00000228817	0	38	49	82
ENSG00000228818	0	0	0	0
ENSG00000228819	0	0	0	0
ENSG00000228820	0	0	0	0
ENSG00000228823	0	0	0	0
ENSG00000228824	0	0	2	0
ENSG00000228825	0	0	0	0
ENSG00000228826	0	0	0	0
ENSG00000228827	0	0	0	0
ENSG00000228828	0	0	0	1
ENSG00000228829	0	0	0	0
ENSG00000228830	0	16	1	0
ENSG00000228833	0	0	0	0
ENSG00000228834	0	0	0	0
ENSG00000228835	0	0	0	0
ENSG00000228836	0	0	0	0
ENSG00000228837	0	0	0	0
ENSG00000228838	0	0	0	0
ENSG00000228839	0	0	0	0
ENSG00000228840	0	0	0	0
ENSG00000228842	0	0	0	0
ENSG00000228843	0	0	0	0
ENSG00000228844	0	0	0	0
ENSG00000228845	0	0	0	0
ENSG00000228846	0	0	0	0
ENSG00000228847	0	0	0	0
ENSG00000228848	0	0	0	0
ENSG00000228849	0	0	0	0

ENSG00000228850	0	0	0	0
ENSG00000228851	0	0	0	0
ENSG00000228852	0	0	0	0
ENSG00000228853	0	0	0	0
ENSG00000228854	0	0	0	0
ENSG00000228855	0	0	0	0
ENSG00000228856	0	0	0	0
ENSG00000228857	10	0	23	15
ENSG00000228858	0	0	0	0
ENSG00000228859	0	0	0	0
ENSG00000228860	0	0	0	0
ENSG00000228861	0	0	0	0
ENSG00000228862	0	0	0	0
ENSG00000228863	0	1	0	0
ENSG00000228865	0	0	0	0
ENSG00000228867	0	0	0	0
ENSG00000228868	0	0	0	0
ENSG00000228869	0	0	0	0
ENSG00000228871	0	0	0	0
ENSG00000228872	0	0	0	0
ENSG00000228873	0	0	0	0
ENSG00000228874	0	0	0	0
ENSG00000228875	0	0	0	0
ENSG00000228876	0	0	0	0
ENSG00000228877	0	0	0	0
ENSG00000228878	24	155	213	338
ENSG00000228879	17	7	7	9
ENSG00000228881	0	0	0	0
ENSG00000228882	0	0	0	0
ENSG00000228883	0	0	0	0
ENSG00000228884	0	0	0	0
ENSG00000228885	0	0	0	0
ENSG00000228886	0	0	0	0
ENSG00000228887	0	0	0	0
ENSG00000228888	0	0	0	0
ENSG00000228889	40	43	18	41
ENSG00000228890	0	0	0	0
ENSG00000228891	0	0	0	0
ENSG00000228892	0	0	0	0
ENSG00000228894	0	0	0	0
ENSG00000228896	0	0	0	0
ENSG00000228897	0	0	0	0
ENSG00000228898	0	0	0	0
ENSG00000228900	0	0	0	0
ENSG00000228901	0	0	0	0
ENSG00000228902	0	0	0	0

ENSG00000228903	0	0	2	6
ENSG00000228904	0	0	0	0
ENSG00000228906	1	31	1	0
ENSG00000228907	0	0	0	0
ENSG00000228909	0	0	0	0
ENSG00000228912	0	0	0	0
ENSG00000228913	0	0	0	0
ENSG00000228914	0	0	0	0
ENSG00000228915	0	0	0	0
ENSG00000228917	0	0	0	0
ENSG00000228918	0	27	18	11
ENSG00000228919	0	0	0	0
ENSG00000228920	0	0	0	0
ENSG00000228921	0	0	0	0
ENSG00000228922	0	0	0	0
ENSG00000228923	0	0	0	0
ENSG00000228925	0	4	10	3
ENSG00000228926	0	0	0	0
ENSG00000228927	0	0	0	0
ENSG00000228928	0	0	0	0
ENSG00000228929	0	0	2	0
ENSG00000228930	0	0	0	0
ENSG00000228932	1	0	3	0
ENSG00000228933	0	0	0	0
ENSG00000228935	0	0	0	0
ENSG00000228937	0	0	0	0
ENSG00000228938	0	0	0	0
ENSG00000228939	0	0	0	0
ENSG00000228940	0	0	0	0
ENSG00000228941	0	0	0	0
ENSG00000228943	0	0	0	0
ENSG00000228944	0	0	0	0
ENSG00000228945	0	0	0	0
ENSG00000228948	0	0	1	0
ENSG00000228949	0	0	0	0
ENSG00000228950	0	0	0	0
ENSG00000228951	0	0	0	0
ENSG00000228952	0	0	0	0
ENSG00000228953	0	0	0	0
ENSG00000228954	0	0	0	0
ENSG00000228955	0	0	0	0
ENSG00000228956	2	207	149	238
ENSG00000228957	0	0	0	0
ENSG00000228958	0	0	0	0
ENSG00000228959	0	0	0	0
ENSG00000228960	0	0	0	0

ENSG00000228961	0	0	0	0
ENSG00000228962	6	0	59	71
ENSG00000228963	0	0	0	0
ENSG00000228964	0	0	0	0
ENSG00000228965	0	0	0	0
ENSG00000228966	0	0	0	0
ENSG00000228968	0	0	0	0
ENSG00000228970	0	0	0	0
ENSG00000228971	0	0	0	0
ENSG00000228972	0	0	0	0
ENSG00000228973	0	0	0	0
ENSG00000228974	0	0	0	0
ENSG00000228976	0	0	0	0
ENSG00000228977	0	0	0	0
ENSG00000228978	0	0	0	0
ENSG00000228979	0	0	0	0
ENSG00000228980	0	0	0	0
ENSG00000228981	0	0	0	0
ENSG00000228982	0	0	0	0
ENSG00000228983	0	0	0	0
ENSG00000228984	0	0	0	0
ENSG00000228985	0	0	0	0
ENSG00000228986	0	0	0	0
ENSG00000228987	0	0	0	0
ENSG00000228988	0	0	0	0
ENSG00000228989	1	0	11	17
ENSG00000228990	0	0	0	0
ENSG00000228991	0	0	0	0
ENSG00000228992	0	0	0	0
ENSG00000228993	0	0	0	0
ENSG00000228995	0	0	0	0
ENSG00000228997	0	0	0	0
ENSG00000228998	4	0	0	2
ENSG00000228999	0	0	0	0
ENSG00000229000	0	0	0	0
ENSG00000229001	0	0	0	0
ENSG00000229002	0	0	0	0
ENSG00000229005	1	34	34	45
ENSG00000229006	0	0	0	0
ENSG00000229007	0	0	1	0
ENSG00000229009	0	0	0	0
ENSG00000229010	0	0	0	0
ENSG00000229011	0	0	0	0
ENSG00000229012	0	0	0	0
ENSG00000229013	0	0	0	0
ENSG00000229014	0	0	0	0

ENSG00000229015	0	0	0	0
ENSG00000229016	0	0	0	0
ENSG00000229017	2	0	6	3
ENSG00000229018	1	150	80	107
ENSG00000229019	0	0	0	3
ENSG00000229020	0	0	0	0
ENSG00000229021	0	0	0	0
ENSG00000229022	0	0	0	0
ENSG00000229023	0	0	0	0
ENSG00000229025	0	0	0	0
ENSG00000229027	0	0	1	0
ENSG00000229028	0	0	0	0
ENSG00000229029	0	0	0	0
ENSG00000229030	0	0	0	0
ENSG00000229031	0	0	0	0
ENSG00000229032	0	0	0	0
ENSG00000229033	4	1	10	9
ENSG00000229034	0	0	0	0
ENSG00000229035	0	0	0	0
ENSG00000229036	2	1	6	22
ENSG00000229037	0	0	0	0
ENSG00000229038	0	0	0	0
ENSG00000229040	0	0	0	0
ENSG00000229042	0	0	0	0
ENSG00000229043	14	0	13	0
ENSG00000229044	0	94	132	70
ENSG00000229045	0	0	0	0
ENSG00000229046	0	0	0	0
ENSG00000229047	0	0	5	0
ENSG00000229048	0	0	0	0
ENSG00000229051	0	0	0	0
ENSG00000229052	0	0	0	3
ENSG00000229054	0	0	0	0
ENSG00000229055	0	0	0	0
ENSG00000229056	73	451	1088	2215
ENSG00000229057	0	0	0	0
ENSG00000229058	0	0	0	0
ENSG00000229060	0	0	2	0
ENSG00000229061	0	0	0	0
ENSG00000229063	0	0	0	0
ENSG00000229064	0	0	0	0
ENSG00000229065	0	0	0	0
ENSG00000229066	0	0	0	0
ENSG00000229067	0	0	0	0
ENSG00000229068	0	0	0	0
ENSG00000229069	0	0	0	0

ENSG00000229071	0	0	0	0
ENSG00000229072	0	0	0	0
ENSG00000229074	0	0	0	0
ENSG00000229075	0	0	0	0
ENSG00000229077	0	0	0	0
ENSG00000229079	0	0	0	0
ENSG00000229080	0	0	0	0
ENSG00000229081	0	0	0	0
ENSG00000229082	0	0	0	0
ENSG00000229083	0	0	0	0
ENSG00000229084	0	0	0	0
ENSG00000229086	0	0	0	0
ENSG00000229087	0	0	0	0
ENSG00000229088	0	0	0	0
ENSG00000229089	0	0	0	0
ENSG00000229090	0	0	0	0
ENSG00000229091	0	0	0	0
ENSG00000229092	0	0	0	0
ENSG00000229093	3	0	3	2
ENSG00000229094	0	0	0	0
ENSG00000229097	0	37	15	30
ENSG00000229099	0	0	0	0
ENSG00000229100	0	0	0	0
ENSG00000229101	0	0	0	0
ENSG00000229102	0	0	0	0
ENSG00000229103	0	0	0	0
ENSG00000229104	0	0	0	0
ENSG00000229105	0	0	0	0
ENSG00000229106	0	0	0	0
ENSG00000229107	0	0	1	0
ENSG00000229108	0	0	0	0
ENSG00000229109	1	0	1	1
ENSG00000229110	0	0	0	0
ENSG00000229111	0	41	27	13
ENSG00000229112	0	0	0	0
ENSG00000229115	0	0	0	0
ENSG00000229116	0	0	0	0
ENSG00000229117	10474	7695	2114	6601
ENSG00000229118	0	0	0	0
ENSG00000229119	1	0	1	0
ENSG00000229120	0	0	0	0
ENSG00000229122	0	0	0	0
ENSG00000229124	9832	39519	25409	19265
ENSG00000229125	0	0	0	0
ENSG00000229126	0	0	0	0
ENSG00000229127	0	134	111	134

ENSG00000229129	0	0	0	0
ENSG00000229131	0	0	0	0
ENSG00000229132	0	0	0	0
ENSG00000229133	0	0	0	0
ENSG00000229136	0	0	0	0
ENSG00000229137	1	0	0	0
ENSG00000229138	0	0	0	0
ENSG00000229140	0	42	9	10
ENSG00000229142	0	0	0	0
ENSG00000229143	0	0	0	0
ENSG00000229145	0	0	0	0
ENSG00000229146	0	0	0	0
ENSG00000229147	0	0	0	0
ENSG00000229149	0	0	0	0
ENSG00000229150	0	0	0	0
ENSG00000229151	0	0	0	0
ENSG00000229152	0	0	1	0
ENSG00000229153	17	1	42	53
ENSG00000229154	0	0	0	0
ENSG00000229155	0	0	0	0
ENSG00000229156	0	0	0	0
ENSG00000229159	0	0	0	0
ENSG00000229160	0	0	0	0
ENSG00000229161	0	0	0	0
ENSG00000229162	0	0	0	0
ENSG00000229163	0	0	0	0
ENSG00000229164	37	0	0	0
ENSG00000229165	0	0	0	0
ENSG00000229167	0	0	0	0
ENSG00000229168	0	0	0	0
ENSG00000229169	0	0	0	0
ENSG00000229170	0	0	0	0
ENSG00000229172	14	0	0	0
ENSG00000229173	0	0	0	0
ENSG00000229175	0	0	0	0
ENSG00000229176	0	0	0	0
ENSG00000229177	0	0	0	0
ENSG00000229178	0	0	0	0
ENSG00000229179	0	0	0	0
ENSG00000229180	8	8	6	3
ENSG00000229181	0	0	0	0
ENSG00000229182	0	0	0	0
ENSG00000229183	0	0	0	0
ENSG00000229184	0	0	0	0
ENSG00000229185	0	0	0	0
ENSG00000229186	3	0	4	11

ENSG00000229187	0	0	0	0
ENSG00000229188	0	0	0	0
ENSG00000229189	0	0	0	0
ENSG00000229190	0	59	13	3
ENSG00000229191	1	0	0	1
ENSG00000229192	0	0	0	0
ENSG00000229194	0	0	0	0
ENSG00000229195	0	0	3	0
ENSG00000229196	11	0	1	1
ENSG00000229197	0	0	0	0
ENSG00000229198	0	0	0	0
ENSG00000229199	0	0	0	0
ENSG00000229200	5	81	20	13
ENSG00000229201	0	0	0	0
ENSG00000229202	0	0	0	0
ENSG00000229203	0	0	0	0
ENSG00000229204	0	0	0	0
ENSG00000229205	0	0	0	0
ENSG00000229206	0	0	0	0
ENSG00000229207	0	0	0	0
ENSG00000229208	0	0	0	0
ENSG00000229209	0	0	0	0
ENSG00000229210	0	0	0	0
ENSG00000229211	0	0	0	0
ENSG00000229212	0	0	0	0
ENSG00000229213	0	0	0	0
ENSG00000229214	0	0	0	0
ENSG00000229215	0	0	0	0
ENSG00000229216	0	0	0	0
ENSG00000229217	0	0	0	0
ENSG00000229220	0	0	0	0
ENSG00000229221	0	0	0	0
ENSG00000229222	0	0	0	0
ENSG00000229223	0	0	0	0
ENSG00000229224	0	0	0	0
ENSG00000229225	0	0	0	0
ENSG00000229226	0	0	0	0
ENSG00000229227	1	0	0	0
ENSG00000229228	0	0	0	0
ENSG00000229229	0	0	0	0
ENSG00000229230	0	0	0	0
ENSG00000229231	0	0	0	0
ENSG00000229232	0	0	0	0
ENSG00000229233	0	0	0	0
ENSG00000229234	0	0	0	0
ENSG00000229235	0	0	0	3

ENSG00000229236	0	106	1	114
ENSG00000229237	0	0	0	0
ENSG00000229238	0	0	0	1
ENSG00000229239	0	0	0	0
ENSG00000229240	0	0	0	0
ENSG00000229241	0	0	0	0
ENSG00000229242	0	0	0	0
ENSG00000229243	0	0	0	0
ENSG00000229245	6	3	1	3
ENSG00000229246	0	0	0	0
ENSG00000229247	0	0	0	0
ENSG00000229248	0	0	0	0
ENSG00000229249	0	0	0	0
ENSG00000229250	0	0	0	0
ENSG00000229251	0	0	0	0
ENSG00000229252	0	0	0	0
ENSG00000229253	0	0	0	0
ENSG00000229254	0	0	0	0
ENSG00000229255	0	0	0	0
ENSG00000229256	0	0	0	0
ENSG00000229257	0	0	0	0
ENSG00000229258	0	0	0	0
ENSG00000229259	0	0	0	0
ENSG00000229261	0	0	0	0
ENSG00000229262	0	0	0	0
ENSG00000229263	0	0	0	0
ENSG00000229265	0	0	0	0
ENSG00000229266	0	0	0	0
ENSG00000229267	0	0	2	11
ENSG00000229268	0	0	0	0
ENSG00000229269	0	0	0	0
ENSG00000229271	0	0	0	0
ENSG00000229272	0	0	0	0
ENSG00000229273	0	0	0	0
ENSG00000229274	0	0	0	0
ENSG00000229275	0	0	0	0
ENSG00000229276	0	0	0	0
ENSG00000229278	0	0	5	1
ENSG00000229280	0	0	0	0
ENSG00000229281	0	0	0	0
ENSG00000229282	1	0	0	0
ENSG00000229283	0	0	0	0
ENSG00000229284	0	0	0	0
ENSG00000229286	0	0	0	0
ENSG00000229287	0	0	0	0
ENSG00000229288	0	0	0	0

ENSG00000229289	0	0	0	0
ENSG00000229291	0	0	0	0
ENSG00000229292	0	0	0	0
ENSG00000229293	0	0	0	0
ENSG00000229294	0	0	1	0
ENSG00000229295	0	0	0	0
ENSG00000229297	0	0	0	0
ENSG00000229298	0	0	0	0
ENSG00000229299	0	0	0	0
ENSG00000229300	0	0	0	0
ENSG00000229301	0	0	0	0
ENSG00000229302	0	0	0	0
ENSG00000229303	0	0	0	0
ENSG00000229305	0	0	0	0
ENSG00000229306	0	0	0	0
ENSG00000229307	0	0	0	0
ENSG00000229308	35	872	52	797
ENSG00000229309	0	0	0	0
ENSG00000229310	0	0	0	0
ENSG00000229311	0	0	0	0
ENSG00000229312	0	0	0	0
ENSG00000229313	0	27	4	0
ENSG00000229314	0	0	0	5
ENSG00000229315	0	0	0	0
ENSG00000229316	0	0	0	0
ENSG00000229320	13	45	37	33
ENSG00000229321	0	20	29	26
ENSG00000229322	0	0	0	0
ENSG00000229323	0	0	0	0
ENSG00000229324	0	0	0	0
ENSG00000229325	0	0	0	0
ENSG00000229326	0	0	0	0
ENSG00000229327	0	0	0	0
ENSG00000229328	0	0	0	0
ENSG00000229330	0	0	0	0
ENSG00000229331	0	0	0	0
ENSG00000229332	2	0	0	0
ENSG00000229334	1	0	1	1
ENSG00000229335	0	0	0	0
ENSG00000229336	0	0	0	0
ENSG00000229337	1	0	2	0
ENSG00000229338	0	0	0	0
ENSG00000229339	0	0	0	0
ENSG00000229341	0	0	0	0
ENSG00000229342	0	0	0	0
ENSG00000229343	0	0	0	0

ENSG00000229344	70	9	20	38
ENSG00000229345	0	0	0	0
ENSG00000229346	0	0	0	0
ENSG00000229347	0	0	0	0
ENSG00000229348	2	0	0	2
ENSG00000229349	0	17	0	0
ENSG00000229351	0	0	0	0
ENSG00000229352	0	0	0	0
ENSG00000229353	0	0	0	0
ENSG00000229356	0	0	0	0
ENSG00000229357	0	0	0	0
ENSG00000229358	2	2412	2489	1940
ENSG00000229359	0	0	0	0
ENSG00000229360	0	0	0	0
ENSG00000229361	0	0	0	0
ENSG00000229363	0	0	0	0
ENSG00000229364	0	0	0	0
ENSG00000229365	0	0	0	0
ENSG00000229367	0	0	0	0
ENSG00000229368	0	0	2	5
ENSG00000229369	0	0	0	0
ENSG00000229370	0	0	0	0
ENSG00000229372	0	0	0	0
ENSG00000229373	0	0	0	0
ENSG00000229375	0	0	0	0
ENSG00000229376	0	0	0	0
ENSG00000229379	0	0	0	0
ENSG00000229380	0	0	0	0
ENSG00000229381	0	0	0	0
ENSG00000229382	0	0	0	0
ENSG00000229383	0	0	0	0
ENSG00000229384	0	0	0	0
ENSG00000229385	0	0	0	0
ENSG00000229386	0	0	1	0
ENSG00000229387	0	0	0	0
ENSG00000229388	0	0	0	0
ENSG00000229389	0	0	0	0
ENSG00000229390	0	0	0	0
ENSG00000229391	3	0	0	0
ENSG00000229393	0	0	0	0
ENSG00000229394	0	0	0	0
ENSG00000229395	0	0	0	0
ENSG00000229396	0	0	0	0
ENSG00000229398	0	0	0	0
ENSG00000229399	0	0	0	0
ENSG00000229400	0	0	0	0

ENSG00000229401	0	0	0	0
ENSG00000229402	0	0	0	0
ENSG00000229403	0	0	0	0
ENSG00000229404	0	0	0	0
ENSG00000229405	0	0	0	0
ENSG00000229406	0	0	0	0
ENSG00000229407	0	0	0	0
ENSG00000229408	0	0	0	0
ENSG00000229409	0	0	0	0
ENSG00000229411	0	0	0	0
ENSG00000229412	0	0	0	0
ENSG00000229413	0	0	0	0
ENSG00000229414	0	0	0	0
ENSG00000229415	0	0	0	0
ENSG00000229416	0	0	0	0
ENSG00000229417	2	0	0	0
ENSG00000229418	0	0	0	0
ENSG00000229419	0	0	17	49
ENSG00000229420	0	0	0	0
ENSG00000229421	0	0	0	0
ENSG00000229422	0	0	0	0
ENSG00000229423	0	0	0	0
ENSG00000229424	0	0	0	0
ENSG00000229425	2	92	163	163
ENSG00000229427	0	0	0	0
ENSG00000229428	0	0	0	0
ENSG00000229430	0	0	0	0
ENSG00000229431	0	0	1	2
ENSG00000229432	0	0	0	0
ENSG00000229433	0	0	0	0
ENSG00000229434	0	0	0	0
ENSG00000229435	0	0	0	0
ENSG00000229436	1	0	0	0
ENSG00000229437	0	0	0	0
ENSG00000229438	0	0	0	0
ENSG00000229439	0	0	0	0
ENSG00000229440	0	0	0	0
ENSG00000229441	0	0	0	0
ENSG00000229442	0	0	0	0
ENSG00000229443	0	0	0	0
ENSG00000229444	0	0	0	0
ENSG00000229447	0	0	0	0
ENSG00000229452	0	0	0	0
ENSG00000229453	0	1	0	0
ENSG00000229454	0	0	0	0
ENSG00000229455	0	0	0	0

ENSG00000229456	0	0	0	0
ENSG00000229457	0	0	0	0
ENSG00000229458	0	0	0	0
ENSG00000229459	0	0	0	0
ENSG00000229461	0	0	0	0
ENSG00000229462	0	0	0	0
ENSG00000229463	0	0	0	2
ENSG00000229464	0	0	0	0
ENSG00000229465	0	0	0	0
ENSG00000229466	0	0	0	0
ENSG00000229467	0	0	0	0
ENSG00000229468	0	0	0	0
ENSG00000229469	0	0	0	0
ENSG00000229470	0	0	0	0
ENSG00000229471	0	0	0	0
ENSG00000229472	0	0	0	0
ENSG00000229473	2	0	3	3
ENSG00000229474	34	23	100	74
ENSG00000229477	0	0	0	0
ENSG00000229478	0	0	0	0
ENSG00000229481	0	0	0	0
ENSG00000229483	0	0	0	0
ENSG00000229484	1	0	0	0
ENSG00000229485	0	0	0	0
ENSG00000229486	0	0	0	0
ENSG00000229487	0	0	0	0
ENSG00000229488	0	0	0	0
ENSG00000229489	0	0	0	0
ENSG00000229491	0	0	0	0
ENSG00000229492	0	0	0	0
ENSG00000229493	0	0	0	0
ENSG00000229494	0	0	0	0
ENSG00000229495	0	0	0	0
ENSG00000229496	0	0	0	0
ENSG00000229497	0	0	0	0
ENSG00000229498	0	0	0	0
ENSG00000229499	0	0	0	0
ENSG00000229500	0	0	0	0
ENSG00000229501	0	0	0	0
ENSG00000229502	0	0	5	0
ENSG00000229503	1	0	0	0
ENSG00000229505	0	0	0	0
ENSG00000229508	0	0	0	0
ENSG00000229509	1	0	0	0
ENSG00000229510	0	0	1	0
ENSG00000229511	0	0	0	0

ENSG00000229512	0	0	0	0
ENSG00000229513	0	0	0	0
ENSG00000229515	0	0	0	0
ENSG00000229518	0	0	0	0
ENSG00000229519	0	0	0	0
ENSG00000229520	0	0	0	0
ENSG00000229521	0	0	0	0
ENSG00000229522	0	0	0	0
ENSG00000229523	0	0	0	0
ENSG00000229524	0	0	0	0
ENSG00000229525	1	0	0	0
ENSG00000229526	0	0	0	0
ENSG00000229528	0	0	0	0
ENSG00000229529	0	0	0	0
ENSG00000229530	0	0	0	0
ENSG00000229531	0	0	0	0
ENSG00000229532	0	0	0	0
ENSG00000229533	0	0	0	0
ENSG00000229534	0	0	0	0
ENSG00000229535	0	0	0	0
ENSG00000229536	0	0	0	0
ENSG00000229537	0	0	0	0
ENSG00000229539	1	0	0	0
ENSG00000229541	0	0	0	0
ENSG00000229542	0	0	0	0
ENSG00000229543	0	0	0	0
ENSG00000229544	0	0	0	0
ENSG00000229546	0	0	0	0
ENSG00000229547	0	0	0	0
ENSG00000229549	0	0	0	0
ENSG00000229550	0	0	0	0
ENSG00000229551	0	0	0	0
ENSG00000229552	0	0	0	0
ENSG00000229553	0	0	0	0
ENSG00000229554	0	0	0	0
ENSG00000229556	0	0	0	0
ENSG00000229557	0	0	0	0
ENSG00000229558	0	0	0	0
ENSG00000229559	0	0	0	0
ENSG00000229560	0	0	0	0
ENSG00000229562	0	0	0	0
ENSG00000229563	0	0	0	0
ENSG00000229565	0	0	0	0
ENSG00000229567	0	0	0	0
ENSG00000229568	0	0	0	0
ENSG00000229569	0	0	0	0

ENSG00000229570	0	0	0	0
ENSG00000229571	0	0	0	0
ENSG00000229572	0	0	0	0
ENSG00000229573	0	0	0	0
ENSG00000229575	0	0	0	0
ENSG00000229578	0	0	0	0
ENSG00000229579	0	0	0	0
ENSG00000229582	0	0	0	0
ENSG00000229583	0	0	0	0
ENSG00000229585	0	0	0	0
ENSG00000229586	0	0	0	0
ENSG00000229587	0	0	0	0
ENSG00000229588	0	0	0	0
ENSG00000229589	0	0	0	0
ENSG00000229590	0	0	1	6
ENSG00000229591	0	0	1	6
ENSG00000229593	0	0	0	0
ENSG00000229594	0	0	0	0
ENSG00000229595	0	0	0	0
ENSG00000229596	0	0	0	1
ENSG00000229597	0	0	0	0
ENSG00000229598	0	0	0	0
ENSG00000229599	0	0	0	0
ENSG00000229600	0	0	0	0
ENSG00000229601	0	0	0	0
ENSG00000229603	0	0	0	0
ENSG00000229604	1	0	0	0
ENSG00000229605	0	0	0	0
ENSG00000229606	0	0	0	0
ENSG00000229607	0	0	0	0
ENSG00000229608	0	0	0	0
ENSG00000229609	0	0	0	0
ENSG00000229611	3	0	0	0
ENSG00000229612	0	0	0	0
ENSG00000229613	0	0	0	0
ENSG00000229615	0	0	0	0
ENSG00000229616	0	0	0	0
ENSG00000229618	0	0	0	0
ENSG00000229619	0	947	901	594
ENSG00000229620	0	0	0	0
ENSG00000229621	0	0	0	0
ENSG00000229622	0	0	0	0
ENSG00000229623	0	0	0	0
ENSG00000229625	2	0	0	0
ENSG00000229626	0	0	0	0
ENSG00000229627	0	0	0	0

ENSG00000229628	0	0	0	0
ENSG00000229629	0	0	0	0
ENSG00000229630	0	0	0	0
ENSG00000229631	0	0	0	0
ENSG00000229635	0	0	0	0
ENSG00000229636	0	0	0	0
ENSG00000229637	0	0	0	0
ENSG00000229638	0	1	0	5
ENSG00000229639	0	0	0	0
ENSG00000229641	0	0	0	0
ENSG00000229642	2	0	3	6
ENSG00000229643	0	0	0	0
ENSG00000229644	1	25	9	5
ENSG00000229645	1	0	0	0
ENSG00000229646	1	0	4	6
ENSG00000229647	100	491	537	1035
ENSG00000229648	0	0	0	0
ENSG00000229649	0	0	0	0
ENSG00000229651	0	0	0	0
ENSG00000229652	0	0	0	0
ENSG00000229653	0	0	0	0
ENSG00000229654	0	0	0	0
ENSG00000229655	0	0	0	0
ENSG00000229656	2	0	11	23
ENSG00000229657	0	0	0	0
ENSG00000229658	0	0	0	0
ENSG00000229659	0	0	4	1
ENSG00000229660	0	0	0	0
ENSG00000229661	0	0	2	0
ENSG00000229662	0	0	0	0
ENSG00000229663	0	0	0	0
ENSG00000229664	0	0	0	0
ENSG00000229665	0	0	0	0
ENSG00000229666	148	963	963	1113
ENSG00000229667	0	0	0	0
ENSG00000229668	0	0	0	0
ENSG00000229670	0	34	0	0
ENSG00000229671	2	0	3	9
ENSG00000229672	0	0	1	0
ENSG00000229673	0	0	0	0
ENSG00000229674	0	0	0	0
ENSG00000229676	1	24	9	18
ENSG00000229677	0	0	0	0
ENSG00000229679	0	0	0	0
ENSG00000229680	0	0	0	0
ENSG00000229681	0	0	0	0

ENSG00000229682	0	0	0	0
ENSG00000229683	0	0	0	0
ENSG00000229684	0	0	0	0
ENSG00000229685	0	0	0	0
ENSG00000229686	0	0	0	0
ENSG00000229687	0	0	0	0
ENSG00000229688	0	0	0	0
ENSG00000229689	0	0	0	0
ENSG00000229690	0	0	0	0
ENSG00000229691	1	0	6	1
ENSG00000229692	1	3	3	6
ENSG00000229693	0	0	0	0
ENSG00000229694	0	0	0	0
ENSG00000229695	0	0	0	0
ENSG00000229696	0	0	0	0
ENSG00000229697	0	0	0	0
ENSG00000229698	0	0	0	0
ENSG00000229699	0	0	0	0
ENSG00000229700	9	16	11	29
ENSG00000229701	0	0	0	0
ENSG00000229702	0	0	0	0
ENSG00000229703	2	0	0	0
ENSG00000229704	0	0	0	0
ENSG00000229706	0	0	0	0
ENSG00000229707	0	0	0	0
ENSG00000229708	0	0	0	0
ENSG00000229709	0	0	0	0
ENSG00000229710	0	0	0	0
ENSG00000229711	0	0	0	0
ENSG00000229713	0	0	0	0
ENSG00000229714	0	0	0	0
ENSG00000229715	1	298	337	663
ENSG00000229716	0	0	0	0
ENSG00000229717	0	0	0	0
ENSG00000229719	0	0	0	0
ENSG00000229720	0	0	0	0
ENSG00000229721	0	0	0	0
ENSG00000229722	0	0	0	0
ENSG00000229723	0	0	0	0
ENSG00000229724	0	0	0	0
ENSG00000229725	0	0	0	0
ENSG00000229727	6	102	43	44
ENSG00000229728	11	0	3	14
ENSG00000229729	1	72	54	54
ENSG00000229730	0	0	0	0
ENSG00000229731	0	0	0	0

ENSG00000229732	0	0	0	0
ENSG00000229733	0	0	0	0
ENSG00000229735	0	0	0	0
ENSG00000229737	0	0	0	0
ENSG00000229738	0	0	0	0
ENSG00000229739	0	0	13	24
ENSG00000229740	0	8	1	0
ENSG00000229741	0	0	0	0
ENSG00000229742	0	0	0	0
ENSG00000229743	0	0	0	0
ENSG00000229744	0	0	0	0
ENSG00000229745	0	0	0	0
ENSG00000229746	0	0	0	0
ENSG00000229747	1	0	0	0
ENSG00000229749	0	0	0	0
ENSG00000229750	0	0	6	3
ENSG00000229751	0	0	0	0
ENSG00000229752	0	0	0	0
ENSG00000229753	0	0	0	0
ENSG00000229754	8596	26720	27590	31327
ENSG00000229755	0	0	0	0
ENSG00000229756	0	0	0	0
ENSG00000229757	0	0	0	0
ENSG00000229758	0	0	0	0
ENSG00000229759	0	0	0	0
ENSG00000229760	0	0	0	0
ENSG00000229761	0	0	0	0
ENSG00000229762	0	0	0	0
ENSG00000229765	0	0	0	0
ENSG00000229766	0	0	3	0
ENSG00000229767	0	0	0	0
ENSG00000229769	0	0	0	0
ENSG00000229770	0	0	0	0
ENSG00000229771	0	0	0	0
ENSG00000229773	0	0	0	0
ENSG00000229774	0	0	0	0
ENSG00000229775	0	0	0	0
ENSG00000229776	0	0	0	0
ENSG00000229779	0	0	0	0
ENSG00000229780	0	25	26	27
ENSG00000229781	1	0	0	0
ENSG00000229782	0	0	0	0
ENSG00000229785	0	0	0	0
ENSG00000229786	0	0	0	0
ENSG00000229788	0	0	0	0
ENSG00000229789	11	50	26	52

ENSG00000229791	0	0	0	0
ENSG00000229792	0	0	0	0
ENSG00000229794	0	0	0	0
ENSG00000229795	0	0	0	0
ENSG00000229796	0	0	0	0
ENSG00000229797	0	0	0	0
ENSG00000229798	0	0	0	0
ENSG00000229800	0	0	1	1
ENSG00000229801	2	28	5	20
ENSG00000229802	0	0	0	0
ENSG00000229805	0	0	0	0
ENSG00000229806	0	0	0	0
ENSG00000229807	0	0	103	0
ENSG00000229808	0	0	0	0
ENSG00000229809	14	116	55	103
ENSG00000229814	0	0	0	0
ENSG00000229815	0	0	0	0
ENSG00000229816	0	0	0	0
ENSG00000229817	0	0	0	0
ENSG00000229818	0	0	0	0
ENSG00000229819	0	0	10	0
ENSG00000229820	0	0	0	0
ENSG00000229821	0	0	0	0
ENSG00000229822	0	0	0	0
ENSG00000229823	0	0	0	0
ENSG00000229824	0	0	0	0
ENSG00000229825	0	0	0	0
ENSG00000229826	0	0	0	0
ENSG00000229827	0	0	0	0
ENSG00000229828	0	0	0	0
ENSG00000229829	0	0	0	0
ENSG00000229830	0	0	0	0
ENSG00000229831	0	0	0	0
ENSG00000229832	0	0	0	0
ENSG00000229833	133	34	37	24
ENSG00000229834	0	0	0	0
ENSG00000229835	0	0	0	0
ENSG00000229836	0	0	0	0
ENSG00000229839	0	11	2	0
ENSG00000229840	0	0	0	0
ENSG00000229841	1	0	4	0
ENSG00000229842	0	0	0	0
ENSG00000229846	0	0	0	0
ENSG00000229847	0	0	0	0
ENSG00000229848	0	0	0	0
ENSG00000229849	0	0	0	0

ENSG00000229851	0	0	0	0
ENSG00000229852	0	18	4	2
ENSG00000229853	0	0	0	0
ENSG00000229854	0	0	0	0
ENSG00000229855	0	0	3	0
ENSG00000229856	0	0	0	0
ENSG00000229857	0	0	0	0
ENSG00000229858	0	0	0	0
ENSG00000229859	1	0	0	0
ENSG00000229861	0	0	0	0
ENSG00000229862	0	0	0	0
ENSG00000229863	0	0	0	0
ENSG00000229865	0	0	0	0
ENSG00000229866	0	0	0	0
ENSG00000229867	0	0	1	0
ENSG00000229869	0	0	0	0
ENSG00000229870	0	0	0	0
ENSG00000229871	0	0	0	0
ENSG00000229873	0	0	0	0
ENSG00000229874	0	0	4	1
ENSG00000229875	0	0	0	0
ENSG00000229876	0	0	0	2
ENSG00000229877	0	0	0	0
ENSG00000229878	0	0	0	0
ENSG00000229880	0	0	0	0
ENSG00000229881	0	0	0	0
ENSG00000229882	0	0	0	0
ENSG00000229885	0	0	0	0
ENSG00000229886	0	0	0	0
ENSG00000229887	0	0	0	0
ENSG00000229889	0	0	0	0
ENSG00000229890	0	0	0	0
ENSG00000229891	1	0	0	0
ENSG00000229892	0	0	0	0
ENSG00000229893	0	0	4	0
ENSG00000229894	0	0	3	12
ENSG00000229895	0	0	0	0
ENSG00000229896	0	0	0	0
ENSG00000229897	0	0	0	0
ENSG00000229898	0	0	0	0
ENSG00000229899	0	0	0	0
ENSG00000229900	0	0	0	0
ENSG00000229901	0	0	0	0
ENSG00000229903	0	0	0	0
ENSG00000229905	0	0	0	0
ENSG00000229906	0	0	0	0

ENSG00000229907	0	0	0	0
ENSG00000229909	0	0	0	0
ENSG00000229910	0	0	0	0
ENSG00000229911	0	0	0	0
ENSG00000229912	0	0	0	0
ENSG00000229913	0	0	0	0
ENSG00000229914	1	0	0	0
ENSG00000229915	0	0	0	0
ENSG00000229917	0	0	0	0
ENSG00000229918	0	0	0	0
ENSG00000229919	0	0	0	0
ENSG00000229920	1	0	0	6
ENSG00000229921	0	0	0	0
ENSG00000229922	0	0	0	0
ENSG00000229923	0	0	1	0
ENSG00000229924	0	0	0	0
ENSG00000229925	0	0	0	0
ENSG00000229926	0	0	0	0
ENSG00000229927	0	0	0	0
ENSG00000229928	0	0	0	1
ENSG00000229929	0	0	0	0
ENSG00000229930	0	0	1	0
ENSG00000229931	0	0	3	0
ENSG00000229932	0	265	86	155
ENSG00000229935	0	0	0	0
ENSG00000229936	0	0	0	0
ENSG00000229937	0	0	0	0
ENSG00000229938	0	0	0	1
ENSG00000229939	0	0	0	0
ENSG00000229940	0	0	0	0
ENSG00000229941	68	77	152	67
ENSG00000229942	0	0	0	0
ENSG00000229943	0	0	0	0
ENSG00000229944	0	54	102	69
ENSG00000229946	0	0	0	0
ENSG00000229947	0	0	0	0
ENSG00000229948	0	0	0	0
ENSG00000229949	0	0	0	0
ENSG00000229950	0	0	1	0
ENSG00000229951	0	0	0	0
ENSG00000229952	0	0	0	0
ENSG00000229953	0	0	0	0
ENSG00000229954	0	0	0	0
ENSG00000229955	0	0	0	0
ENSG00000229956	0	0	12	12
ENSG00000229957	0	0	0	0

ENSG00000229959	0	0	0	0
ENSG00000229960	0	0	0	0
ENSG00000229961	2	0	0	0
ENSG00000229962	0	0	0	6
ENSG00000229964	0	0	0	0
ENSG00000229965	0	0	0	0
ENSG00000229967	0	0	0	0
ENSG00000229968	0	0	0	0
ENSG00000229969	0	0	0	0
ENSG00000229970	0	0	0	0
ENSG00000229971	0	0	0	0
ENSG00000229972	0	0	0	0
ENSG00000229974	0	0	30	2
ENSG00000229975	0	0	0	0
ENSG00000229976	0	0	0	0
ENSG00000229977	0	0	0	0
ENSG00000229978	0	0	0	0
ENSG00000229979	0	0	0	0
ENSG00000229980	2	0	11	2
ENSG00000229981	0	0	0	2
ENSG00000229982	0	0	0	0
ENSG00000229983	0	0	1	0
ENSG00000229985	0	0	0	0
ENSG00000229986	1	0	0	0
ENSG00000229988	10	48	20	53
ENSG00000229989	0	36	4	1
ENSG00000229990	0	0	0	0
ENSG00000229991	0	0	0	0
ENSG00000229992	0	0	0	0
ENSG00000229994	0	14	3	0
ENSG00000229995	0	0	0	0
ENSG00000229996	0	0	0	0
ENSG00000229998	0	0	0	0
ENSG00000229999	0	0	0	0
ENSG00000230000	0	0	0	1
ENSG00000230001	0	0	0	0
ENSG00000230002	0	0	0	0
ENSG00000230003	0	0	0	0
ENSG00000230004	0	0	0	0
ENSG00000230005	0	0	0	0
ENSG00000230006	37	2299	3867	1453
ENSG00000230009	0	0	0	0
ENSG00000230010	0	0	0	0
ENSG00000230011	0	0	0	0
ENSG00000230012	0	0	0	0
ENSG00000230013	0	0	0	0

ENSG00000230014	0	0	0	0
ENSG00000230015	0	0	0	0
ENSG00000230016	0	0	0	0
ENSG00000230017	0	0	0	0
ENSG00000230018	0	0	0	0
ENSG00000230019	0	0	0	0
ENSG00000230020	0	0	0	0
ENSG00000230021	1	0	0	0
ENSG00000230022	0	0	0	0
ENSG00000230023	0	0	0	0
ENSG00000230024	0	0	0	0
ENSG00000230025	0	0	0	0
ENSG00000230026	0	0	0	0
ENSG00000230027	0	0	0	0
ENSG00000230028	0	0	0	0
ENSG00000230029	0	0	0	0
ENSG00000230030	0	0	1	0
ENSG00000230031	0	0	0	0
ENSG00000230033	0	0	0	0
ENSG00000230034	0	0	0	0
ENSG00000230035	0	0	0	0
ENSG00000230036	0	0	0	0
ENSG00000230037	0	0	0	0
ENSG00000230039	0	0	0	0
ENSG00000230040	0	0	0	0
ENSG00000230042	0	0	0	4
ENSG00000230043	0	20393	30082	10641
ENSG00000230044	0	0	0	0
ENSG00000230045	0	0	0	0
ENSG00000230046	0	0	11	0
ENSG00000230047	0	0	0	0
ENSG00000230048	0	0	0	0
ENSG00000230051	0	0	0	0
ENSG00000230052	0	0	0	6
ENSG00000230053	0	0	0	0
ENSG00000230054	0	0	0	0
ENSG00000230055	23	0	0	0
ENSG00000230056	0	0	0	0
ENSG00000230058	6	31	6	5
ENSG00000230060	0	0	0	0
ENSG00000230061	0	0	0	0
ENSG00000230062	0	0	0	0
ENSG00000230063	0	0	0	0
ENSG00000230064	0	0	0	0
ENSG00000230065	0	0	0	0
ENSG00000230066	0	0	0	0

ENSG00000230067	0	0	0	0
ENSG00000230068	0	0	0	0
ENSG00000230069	1	0	49	78
ENSG00000230071	1	67	22	45
ENSG00000230072	0	0	0	0
ENSG00000230073	0	0	0	0
ENSG00000230074	1	0	6	2
ENSG00000230075	0	0	0	0
ENSG00000230076	1	0	0	0
ENSG00000230077	0	0	0	0
ENSG00000230079	0	0	0	0
ENSG00000230080	0	0	0	0
ENSG00000230081	0	0	0	0
ENSG00000230082	0	0	1	0
ENSG00000230083	0	0	0	0
ENSG00000230084	0	14	6	8
ENSG00000230086	0	0	0	0
ENSG00000230087	0	0	0	0
ENSG00000230088	0	0	0	0
ENSG00000230089	0	0	0	0
ENSG00000230090	0	4	9	5
ENSG00000230091	1	0	0	15
ENSG00000230092	1	0	5	0
ENSG00000230096	0	0	0	0
ENSG00000230097	0	0	0	0
ENSG00000230098	0	0	0	0
ENSG00000230099	7	27	23	14
ENSG00000230100	0	0	0	0
ENSG00000230101	0	0	0	0
ENSG00000230102	0	0	0	0
ENSG00000230104	0	0	0	0
ENSG00000230105	0	0	6	2
ENSG00000230106	0	0	0	0
ENSG00000230107	0	0	0	0
ENSG00000230108	0	0	0	0
ENSG00000230109	0	0	0	0
ENSG00000230110	0	0	0	0
ENSG00000230111	0	0	0	0
ENSG00000230112	0	0	0	0
ENSG00000230113	0	0	0	0
ENSG00000230114	0	0	0	0
ENSG00000230115	0	0	0	0
ENSG00000230116	0	0	0	0
ENSG00000230118	0	0	0	2
ENSG00000230119	0	0	0	0
ENSG00000230120	0	0	0	0

ENSG00000230121	0	0	0	1
ENSG00000230122	0	0	0	0
ENSG00000230123	0	0	0	0
ENSG00000230124	0	0	2	5
ENSG00000230125	0	0	0	0
ENSG00000230126	0	0	0	0
ENSG00000230128	0	0	0	0
ENSG00000230130	0	0	0	0
ENSG00000230131	1	0	0	3
ENSG00000230132	0	0	0	0
ENSG00000230133	0	0	0	0
ENSG00000230134	0	0	0	0
ENSG00000230136	0	0	0	0
ENSG00000230137	0	0	0	0
ENSG00000230138	1	0	0	0
ENSG00000230140	0	0	0	0
ENSG00000230141	0	0	0	0
ENSG00000230142	0	0	0	0
ENSG00000230143	0	0	0	0
ENSG00000230146	0	0	0	0
ENSG00000230147	0	0	0	0
ENSG00000230148	0	0	0	0
ENSG00000230149	0	0	0	0
ENSG00000230150	0	0	0	0
ENSG00000230153	0	0	0	0
ENSG00000230154	0	0	0	0
ENSG00000230155	4	0	6	8
ENSG00000230156	0	0	0	0
ENSG00000230157	0	0	0	0
ENSG00000230158	0	0	0	0
ENSG00000230159	0	0	0	0
ENSG00000230160	0	0	0	0
ENSG00000230161	0	0	0	0
ENSG00000230162	1	23	28	10
ENSG00000230163	0	0	0	0
ENSG00000230164	0	0	0	0
ENSG00000230165	0	0	0	0
ENSG00000230166	0	0	0	0
ENSG00000230169	0	0	0	0
ENSG00000230170	0	0	0	0
ENSG00000230171	0	0	0	0
ENSG00000230172	0	0	0	0
ENSG00000230173	0	0	0	0
ENSG00000230174	0	0	0	0
ENSG00000230175	0	0	0	0
ENSG00000230176	7	0	2	0

ENSG00000230177	2	40	46	55
ENSG00000230178	0	0	0	0
ENSG00000230180	0	0	0	0
ENSG00000230181	0	0	0	0
ENSG00000230182	0	0	0	0
ENSG00000230183	0	29	5	11
ENSG00000230184	0	0	0	0
ENSG00000230185	0	0	0	0
ENSG00000230186	0	0	0	0
ENSG00000230187	0	0	0	0
ENSG00000230188	0	0	0	0
ENSG00000230189	0	221	205	312
ENSG00000230190	0	0	0	0
ENSG00000230191	0	0	0	0
ENSG00000230192	0	0	0	0
ENSG00000230193	0	0	0	0
ENSG00000230194	0	0	0	0
ENSG00000230195	0	0	0	0
ENSG00000230196	0	0	0	0
ENSG00000230197	0	0	0	0
ENSG00000230198	0	0	0	0
ENSG00000230199	0	0	0	0
ENSG00000230201	0	0	0	0
ENSG00000230202	0	0	0	0
ENSG00000230203	0	0	0	0
ENSG00000230204	0	0	0	0
ENSG00000230205	0	0	0	0
ENSG00000230206	0	0	0	0
ENSG00000230207	0	0	1	0
ENSG00000230208	0	0	0	0
ENSG00000230209	0	0	0	0
ENSG00000230210	0	0	0	0
ENSG00000230211	0	0	0	0
ENSG00000230212	0	12	1	0
ENSG00000230213	0	0	0	0
ENSG00000230214	0	0	0	0
ENSG00000230215	2	0	0	0
ENSG00000230216	0	0	0	0
ENSG00000230219	0	0	0	0
ENSG00000230220	0	0	0	0
ENSG00000230221	0	0	0	0
ENSG00000230223	0	0	0	2
ENSG00000230224	0	0	0	0
ENSG00000230225	0	0	0	0
ENSG00000230226	0	0	0	0
ENSG00000230227	0	0	0	0

ENSG00000230228	1	0	0	0
ENSG00000230229	0	0	0	0
ENSG00000230230	0	0	0	0
ENSG00000230231	0	0	1	1
ENSG00000230233	0	0	0	0
ENSG00000230234	0	0	1	0
ENSG00000230237	0	0	0	0
ENSG00000230238	0	0	0	0
ENSG00000230239	0	0	0	0
ENSG00000230240	0	0	0	0
ENSG00000230241	0	0	0	0
ENSG00000230243	0	0	0	0
ENSG00000230244	0	0	0	0
ENSG00000230245	0	0	0	0
ENSG00000230246	0	0	0	0
ENSG00000230247	0	0	0	0
ENSG00000230248	0	0	0	0
ENSG00000230249	0	0	0	0
ENSG00000230250	0	0	0	0
ENSG00000230251	0	0	0	0
ENSG00000230252	0	0	0	0
ENSG00000230254	0	0	0	0
ENSG00000230255	0	0	0	0
ENSG00000230256	0	0	0	0
ENSG00000230257	23	20	32	37
ENSG00000230258	0	0	0	0
ENSG00000230259	0	0	0	0
ENSG00000230260	0	0	0	0
ENSG00000230261	0	0	0	0
ENSG00000230262	26	0	0	0
ENSG00000230265	0	0	0	0
ENSG00000230266	44	86	126	160
ENSG00000230267	0	26	10	39
ENSG00000230268	0	0	0	0
ENSG00000230269	0	0	0	0
ENSG00000230271	0	0	0	0
ENSG00000230272	0	0	1	0
ENSG00000230273	0	0	0	0
ENSG00000230274	0	0	0	0
ENSG00000230275	0	0	0	0
ENSG00000230276	0	0	0	0
ENSG00000230279	0	0	0	0
ENSG00000230280	0	0	0	0
ENSG00000230281	0	0	0	0
ENSG00000230282	0	0	0	0
ENSG00000230283	0	0	0	0

ENSG00000230284	0	0	0	0
ENSG00000230285	0	0	0	0
ENSG00000230286	0	0	0	0
ENSG00000230287	0	0	0	0
ENSG00000230288	0	0	0	0
ENSG00000230289	0	0	0	0
ENSG00000230290	0	0	0	0
ENSG00000230291	0	0	0	0
ENSG00000230292	0	0	0	0
ENSG00000230293	0	0	0	0
ENSG00000230294	1	0	0	0
ENSG00000230295	0	24	10	14
ENSG00000230298	0	0	0	0
ENSG00000230299	0	0	0	0
ENSG00000230300	0	0	0	0
ENSG00000230301	0	0	0	0
ENSG00000230302	0	0	0	0
ENSG00000230303	0	0	0	0
ENSG00000230304	0	0	0	0
ENSG00000230305	0	15	3	9
ENSG00000230306	0	0	0	0
ENSG00000230307	0	0	0	0
ENSG00000230308	0	0	0	0
ENSG00000230309	0	0	0	0
ENSG00000230310	0	23	5	0
ENSG00000230311	0	0	0	0
ENSG00000230312	0	0	0	0
ENSG00000230313	0	0	0	0
ENSG00000230314	0	0	0	0
ENSG00000230316	0	0	0	0
ENSG00000230317	0	0	0	0
ENSG00000230318	0	0	0	0
ENSG00000230319	0	0	0	0
ENSG00000230320	0	0	0	0
ENSG00000230321	0	0	0	0
ENSG00000230322	25	128	48	59
ENSG00000230323	0	0	0	0
ENSG00000230324	0	0	0	0
ENSG00000230325	0	0	0	0
ENSG00000230326	0	0	0	0
ENSG00000230327	0	0	0	0
ENSG00000230328	0	0	0	0
ENSG00000230330	0	0	1	0
ENSG00000230331	0	0	0	0
ENSG00000230332	0	0	0	0
ENSG00000230333	3	81	39	99

ENSG00000230335	0	0	0	0
ENSG00000230336	0	0	0	0
ENSG00000230337	0	0	0	0
ENSG00000230338	0	0	0	0
ENSG00000230339	0	0	0	0
ENSG00000230341	0	0	0	0
ENSG00000230342	0	0	0	0
ENSG00000230343	0	0	0	0
ENSG00000230345	0	0	0	0
ENSG00000230347	0	0	0	0
ENSG00000230349	0	0	0	0
ENSG00000230350	0	0	0	0
ENSG00000230352	0	0	0	0
ENSG00000230353	0	0	0	0
ENSG00000230354	0	0	0	0
ENSG00000230355	0	0	0	0
ENSG00000230356	0	0	0	0
ENSG00000230357	0	0	0	0
ENSG00000230358	0	0	0	0
ENSG00000230359	0	0	0	0
ENSG00000230360	0	0	0	0
ENSG00000230361	0	0	0	0
ENSG00000230362	0	0	0	0
ENSG00000230364	0	0	0	0
ENSG00000230365	0	0	0	0
ENSG00000230366	0	0	0	0
ENSG00000230368	4	18	3	1
ENSG00000230370	0	0	0	0
ENSG00000230371	0	0	0	0
ENSG00000230372	0	0	0	0
ENSG00000230373	1	48	30	4
ENSG00000230375	0	0	0	0
ENSG00000230376	0	0	0	1
ENSG00000230377	0	0	0	0
ENSG00000230379	0	0	0	0
ENSG00000230381	0	0	0	0
ENSG00000230383	0	0	0	0
ENSG00000230384	0	0	0	0
ENSG00000230385	0	818	1177	984
ENSG00000230386	0	0	0	0
ENSG00000230387	0	0	0	0
ENSG00000230389	0	0	0	0
ENSG00000230390	0	0	0	0
ENSG00000230391	0	0	0	0
ENSG00000230392	3	4	0	4
ENSG00000230393	0	0	0	0

ENSG00000230394	0	0	0	0
ENSG00000230395	0	0	0	0
ENSG00000230397	0	0	0	0
ENSG00000230398	0	0	0	0
ENSG00000230399	0	0	0	0
ENSG00000230400	0	0	0	0
ENSG00000230401	0	0	0	0
ENSG00000230402	0	0	0	0
ENSG00000230403	0	0	0	0
ENSG00000230404	0	0	0	0
ENSG00000230405	0	0	0	0
ENSG00000230406	0	0	0	0
ENSG00000230407	0	0	0	0
ENSG00000230408	0	14	8	2
ENSG00000230409	0	1	15	26
ENSG00000230410	0	0	0	0
ENSG00000230411	0	0	0	0
ENSG00000230412	0	0	0	0
ENSG00000230413	0	0	0	0
ENSG00000230414	0	0	0	0
ENSG00000230415	0	0	0	0
ENSG00000230416	0	0	0	0
ENSG00000230417	28	52	73	291
ENSG00000230418	0	0	0	0
ENSG00000230419	0	0	0	0
ENSG00000230422	0	0	0	0
ENSG00000230423	0	0	0	0
ENSG00000230424	3	0	0	0
ENSG00000230425	0	0	0	0
ENSG00000230426	0	0	0	0
ENSG00000230427	0	0	0	0
ENSG00000230428	0	0	0	0
ENSG00000230430	0	0	0	0
ENSG00000230432	0	0	0	0
ENSG00000230433	1	0	0	0
ENSG00000230434	0	0	0	0
ENSG00000230435	1	0	3	0
ENSG00000230437	0	0	0	0
ENSG00000230438	21	1	46	17
ENSG00000230439	0	0	0	0
ENSG00000230440	0	0	0	0
ENSG00000230441	0	0	0	0
ENSG00000230442	0	0	0	0
ENSG00000230444	0	0	0	0
ENSG00000230445	1	0	4	0
ENSG00000230446	0	0	0	0

ENSG00000230447	0	0	0	0
ENSG00000230448	0	0	0	0
ENSG00000230449	0	0	0	0
ENSG00000230450	0	0	0	0
ENSG00000230451	0	0	0	0
ENSG00000230452	0	0	0	0
ENSG00000230453	0	1	14	11
ENSG00000230454	0	0	0	0
ENSG00000230455	0	0	0	0
ENSG00000230456	0	0	0	0
ENSG00000230457	0	0	0	0
ENSG00000230458	0	0	0	0
ENSG00000230459	0	0	0	0
ENSG00000230461	0	0	0	0
ENSG00000230463	0	0	0	0
ENSG00000230465	0	0	0	0
ENSG00000230467	0	0	0	0
ENSG00000230468	0	0	0	0
ENSG00000230469	0	0	0	0
ENSG00000230470	0	0	0	0
ENSG00000230471	0	0	0	0
ENSG00000230472	0	0	0	0
ENSG00000230474	0	0	0	0
ENSG00000230475	0	0	0	0
ENSG00000230476	0	0	0	0
ENSG00000230477	0	0	0	0
ENSG00000230478	0	0	0	0
ENSG00000230479	0	0	0	0
ENSG00000230480	0	0	0	0
ENSG00000230481	0	0	0	3
ENSG00000230482	0	0	0	0
ENSG00000230483	0	0	0	0
ENSG00000230484	0	0	0	0
ENSG00000230485	0	0	0	0
ENSG00000230487	48	176	43	42
ENSG00000230489	8	34	9	14
ENSG00000230490	0	0	1	0
ENSG00000230491	0	0	0	0
ENSG00000230492	0	0	0	0
ENSG00000230493	0	0	0	0
ENSG00000230495	0	0	0	0
ENSG00000230496	0	0	0	0
ENSG00000230497	0	0	0	0
ENSG00000230498	0	0	0	0
ENSG00000230499	21	0	1	0
ENSG00000230500	0	0	0	0

ENSG00000230501	0	0	0	1
ENSG00000230502	0	0	0	0
ENSG00000230504	0	0	0	0
ENSG00000230505	0	0	0	0
ENSG00000230506	0	0	0	0
ENSG00000230507	0	0	0	0
ENSG00000230508	0	0	0	0
ENSG00000230509	0	0	0	0
ENSG00000230510	12	0	2	18
ENSG00000230511	0	0	0	0
ENSG00000230513	0	0	2	2
ENSG00000230514	0	0	0	0
ENSG00000230515	0	0	0	0
ENSG00000230516	0	0	0	0
ENSG00000230519	0	0	0	0
ENSG00000230520	0	0	0	0
ENSG00000230521	0	0	0	0
ENSG00000230522	0	0	0	0
ENSG00000230523	0	0	0	0
ENSG00000230524	0	0	0	0
ENSG00000230525	1	0	1	2
ENSG00000230526	0	1	0	8
ENSG00000230528	0	0	0	0
ENSG00000230529	0	0	0	0
ENSG00000230530	0	1	9	11
ENSG00000230531	0	0	0	0
ENSG00000230532	0	0	0	0
ENSG00000230533	0	0	0	0
ENSG00000230534	0	0	0	2
ENSG00000230535	0	0	0	0
ENSG00000230536	0	0	0	0
ENSG00000230537	0	0	0	0
ENSG00000230538	0	0	0	0
ENSG00000230539	0	0	2	10
ENSG00000230541	0	0	0	0
ENSG00000230542	0	0	0	0
ENSG00000230543	0	0	0	0
ENSG00000230544	0	0	0	0
ENSG00000230545	0	0	0	0
ENSG00000230546	0	0	0	0
ENSG00000230547	0	0	0	0
ENSG00000230548	0	0	0	0
ENSG00000230549	0	0	0	0
ENSG00000230550	0	0	0	0
ENSG00000230551	10	175	82	69
ENSG00000230552	0	0	0	0

ENSG00000230553	0	0	0	0
ENSG00000230555	13	0	0	1
ENSG00000230556	0	0	0	0
ENSG00000230558	0	0	0	0
ENSG00000230559	0	0	0	0
ENSG00000230561	0	0	0	0
ENSG00000230562	1	0	0	0
ENSG00000230563	0	0	0	0
ENSG00000230564	0	0	0	0
ENSG00000230565	0	0	0	0
ENSG00000230567	0	0	0	0
ENSG00000230568	0	0	0	0
ENSG00000230569	1	0	0	0
ENSG00000230570	0	0	0	0
ENSG00000230571	0	0	0	0
ENSG00000230572	0	0	0	0
ENSG00000230573	0	0	0	0
ENSG00000230574	0	0	0	0
ENSG00000230575	0	0	0	0
ENSG00000230576	4	14	45	20
ENSG00000230578	0	0	0	0
ENSG00000230580	0	0	0	0
ENSG00000230581	0	0	0	0
ENSG00000230582	0	0	0	0
ENSG00000230583	0	25	11	11
ENSG00000230584	0	0	0	0
ENSG00000230585	0	0	3	2
ENSG00000230587	3	0	0	0
ENSG00000230589	0	0	0	0
ENSG00000230590	20	271	621	355
ENSG00000230592	0	0	0	0
ENSG00000230593	2	0	0	0
ENSG00000230594	0	0	0	0
ENSG00000230595	0	0	0	0
ENSG00000230596	0	0	0	0
ENSG00000230597	0	0	0	0
ENSG00000230598	0	0	0	0
ENSG00000230599	0	0	0	0
ENSG00000230600	0	0	0	0
ENSG00000230601	0	0	0	0
ENSG00000230602	0	0	0	0
ENSG00000230603	0	0	0	0
ENSG00000230604	0	0	0	0
ENSG00000230605	0	0	0	0
ENSG00000230606	0	27	31	78
ENSG00000230607	0	0	0	0

ENSG00000230609	0	0	0	0
ENSG00000230610	0	0	0	0
ENSG00000230611	0	0	0	0
ENSG00000230612	0	0	0	0
ENSG00000230613	1	0	4	9
ENSG00000230614	0	0	0	0
ENSG00000230615	13	13	13	19
ENSG00000230616	0	0	0	0
ENSG00000230617	0	0	0	0
ENSG00000230618	0	0	0	0
ENSG00000230621	0	0	0	0
ENSG00000230622	1	0	0	0
ENSG00000230623	0	0	0	0
ENSG00000230624	0	0	0	0
ENSG00000230626	1	0	0	0
ENSG00000230627	0	0	0	0
ENSG00000230628	0	0	0	0
ENSG00000230629	1	0	0	0
ENSG00000230630	0	0	1	3
ENSG00000230631	0	0	0	0
ENSG00000230633	0	0	0	0
ENSG00000230634	0	0	0	0
ENSG00000230635	0	0	0	0
ENSG00000230636	0	0	0	0
ENSG00000230637	0	0	0	0
ENSG00000230638	0	0	0	0
ENSG00000230639	0	0	0	0
ENSG00000230641	4	21	20	17
ENSG00000230643	0	0	0	0
ENSG00000230645	0	0	0	0
ENSG00000230646	1	0	0	0
ENSG00000230647	0	0	0	0
ENSG00000230648	1	62	8	0
ENSG00000230649	1	0	4	3
ENSG00000230650	0	0	3	4
ENSG00000230651	0	0	0	0
ENSG00000230654	0	0	0	0
ENSG00000230655	0	0	0	0
ENSG00000230657	0	0	0	0
ENSG00000230658	1	0	0	4
ENSG00000230659	0	0	0	0
ENSG00000230660	0	0	0	0
ENSG00000230661	0	0	0	0
ENSG00000230662	0	0	0	0
ENSG00000230663	0	0	0	0
ENSG00000230665	0	13	0	0

ENSG00000230666	0	0	1	179
ENSG00000230667	0	0	0	0
ENSG00000230668	0	0	0	0
ENSG00000230669	0	0	0	0
ENSG00000230671	0	0	0	0
ENSG00000230672	0	0	0	0
ENSG00000230673	0	0	0	0
ENSG00000230674	0	0	0	0
ENSG00000230675	0	0	0	0
ENSG00000230676	0	0	0	0
ENSG00000230678	0	0	0	0
ENSG00000230679	2	31	2	7
ENSG00000230680	0	0	0	0
ENSG00000230681	0	0	0	0
ENSG00000230682	0	0	0	0
ENSG00000230683	0	0	0	0
ENSG00000230684	0	0	0	0
ENSG00000230685	0	0	0	0
ENSG00000230686	0	0	0	0
ENSG00000230687	0	0	0	0
ENSG00000230690	0	0	9	0
ENSG00000230691	0	0	0	0
ENSG00000230693	0	0	0	0
ENSG00000230694	0	0	0	0
ENSG00000230695	0	0	0	0
ENSG00000230696	0	0	0	0
ENSG00000230697	0	0	0	0
ENSG00000230698	0	0	0	0
ENSG00000230699	0	0	0	0
ENSG00000230700	0	0	0	0
ENSG00000230701	0	0	0	0
ENSG00000230702	0	0	0	0
ENSG00000230703	0	0	0	0
ENSG00000230704	0	0	0	0
ENSG00000230705	0	0	0	0
ENSG00000230706	0	0	0	0
ENSG00000230707	0	0	0	0
ENSG00000230708	0	0	0	0
ENSG00000230709	1	0	0	0
ENSG00000230710	0	0	0	0
ENSG00000230711	0	0	0	0
ENSG00000230712	0	0	0	0
ENSG00000230714	0	0	0	0
ENSG00000230715	0	0	0	0
ENSG00000230716	0	0	0	0
ENSG00000230718	0	0	0	0

ENSG00000230720	0	0	0	0
ENSG00000230721	0	0	0	0
ENSG00000230722	0	0	0	0
ENSG00000230724	1	86	141	186
ENSG00000230725	0	0	0	0
ENSG00000230726	0	0	0	0
ENSG00000230727	0	0	0	0
ENSG00000230728	0	0	0	0
ENSG00000230729	0	0	0	1
ENSG00000230730	0	0	0	0
ENSG00000230731	0	0	0	0
ENSG00000230732	0	0	0	0
ENSG00000230733	3	0	0	0
ENSG00000230734	4	0	0	0
ENSG00000230735	1	0	2	3
ENSG00000230736	0	0	0	0
ENSG00000230737	0	0	0	0
ENSG00000230739	0	0	0	0
ENSG00000230741	0	0	0	0
ENSG00000230742	0	0	0	0
ENSG00000230745	0	0	0	0
ENSG00000230746	0	0	0	0
ENSG00000230747	0	0	0	0
ENSG00000230748	0	0	0	0
ENSG00000230749	5	0	5	4
ENSG00000230750	0	0	0	0
ENSG00000230751	0	0	0	0
ENSG00000230752	0	0	0	0
ENSG00000230753	1	0	0	0
ENSG00000230754	0	0	0	0
ENSG00000230755	0	0	0	0
ENSG00000230756	0	0	0	4
ENSG00000230757	0	0	0	0
ENSG00000230758	0	0	0	0
ENSG00000230759	0	0	0	0
ENSG00000230761	19	0	2	23
ENSG00000230763	0	0	0	0
ENSG00000230764	0	0	0	0
ENSG00000230768	0	0	0	0
ENSG00000230769	0	0	0	0
ENSG00000230772	0	0	5	3
ENSG00000230773	132	510	503	798
ENSG00000230777	0	0	0	0
ENSG00000230778	0	0	0	0
ENSG00000230779	0	0	0	0
ENSG00000230780	0	0	0	0

ENSG00000230781	0	0	0	0
ENSG00000230782	0	0	0	0
ENSG00000230783	0	0	0	0
ENSG00000230785	0	0	0	0
ENSG00000230787	0	0	0	0
ENSG00000230788	0	0	0	0
ENSG00000230789	0	0	0	0
ENSG00000230790	0	0	0	0
ENSG00000230791	0	0	0	0
ENSG00000230793	0	0	0	0
ENSG00000230794	0	0	0	0
ENSG00000230795	0	0	1	0
ENSG00000230796	0	0	0	0
ENSG00000230797	0	0	0	0
ENSG00000230798	0	0	0	0
ENSG00000230799	0	0	0	0
ENSG00000230801	0	0	0	0
ENSG00000230803	0	0	0	0
ENSG00000230804	0	0	0	0
ENSG00000230805	0	0	0	0
ENSG00000230806	0	0	0	0
ENSG00000230807	0	0	0	0
ENSG00000230809	0	0	0	0
ENSG00000230812	0	0	0	0
ENSG00000230813	6	7	1	1
ENSG00000230814	0	0	0	0
ENSG00000230815	0	0	0	0
ENSG00000230816	0	0	0	0
ENSG00000230817	0	0	0	0
ENSG00000230818	0	0	0	0
ENSG00000230819	0	0	0	0
ENSG00000230820	0	0	0	0
ENSG00000230821	0	0	0	0
ENSG00000230823	0	0	0	0
ENSG00000230825	2	0	5	5
ENSG00000230826	0	0	0	0
ENSG00000230828	0	0	0	0
ENSG00000230829	0	0	0	0
ENSG00000230830	0	0	0	0
ENSG00000230831	0	0	3	0
ENSG00000230832	0	0	0	0
ENSG00000230833	0	0	0	0
ENSG00000230834	0	0	0	0
ENSG00000230835	0	0	0	0
ENSG00000230836	0	0	0	0
ENSG00000230837	0	0	0	0

ENSG00000230838	0	0	0	0
ENSG00000230839	0	0	0	0
ENSG00000230840	0	0	0	0
ENSG00000230841	0	0	0	0
ENSG00000230843	0	0	0	0
ENSG00000230844	6	56	38	15
ENSG00000230845	0	0	0	0
ENSG00000230846	0	0	0	0
ENSG00000230847	0	9	0	34
ENSG00000230848	0	0	0	0
ENSG00000230849	0	3	2	0
ENSG00000230850	4	12	26	25
ENSG00000230851	0	0	0	0
ENSG00000230852	0	0	0	0
ENSG00000230853	0	0	0	0
ENSG00000230854	0	0	0	0
ENSG00000230855	0	0	0	0
ENSG00000230856	0	0	0	0
ENSG00000230857	0	0	0	2
ENSG00000230859	0	0	0	0
ENSG00000230860	0	0	0	0
ENSG00000230863	0	0	0	0
ENSG00000230864	0	0	0	0
ENSG00000230865	0	0	0	0
ENSG00000230866	0	0	0	0
ENSG00000230867	0	0	0	0
ENSG00000230869	0	0	0	0
ENSG00000230870	0	0	2	0
ENSG00000230871	0	0	0	0
ENSG00000230872	0	0	0	3
ENSG00000230873	0	0	0	0
ENSG00000230874	0	0	0	0
ENSG00000230876	0	18	0	0
ENSG00000230878	0	0	0	0
ENSG00000230879	0	0	0	0
ENSG00000230880	0	0	26	19
ENSG00000230881	0	0	0	0
ENSG00000230882	0	0	0	0
ENSG00000230885	0	0	0	0
ENSG00000230886	0	0	0	0
ENSG00000230887	0	0	0	0
ENSG00000230888	0	0	0	0
ENSG00000230889	0	0	0	0
ENSG00000230891	0	0	0	0
ENSG00000230894	0	0	0	0
ENSG00000230896	0	0	0	0

ENSG00000230897	0	0	0	0
ENSG00000230898	0	0	0	0
ENSG00000230899	0	0	0	0
ENSG00000230900	0	0	0	0
ENSG00000230901	0	0	0	0
ENSG00000230902	0	0	0	0
ENSG00000230903	0	0	0	0
ENSG00000230904	0	0	0	0
ENSG00000230906	0	0	0	0
ENSG00000230907	0	0	0	0
ENSG00000230908	0	0	0	0
ENSG00000230910	0	0	0	0
ENSG00000230911	0	0	0	0
ENSG00000230912	0	0	0	0
ENSG00000230913	0	0	0	0
ENSG00000230914	0	0	0	0
ENSG00000230915	0	0	0	0
ENSG00000230916	0	0	0	0
ENSG00000230918	0	0	0	0
ENSG00000230920	0	0	0	0
ENSG00000230921	0	0	0	0
ENSG00000230922	0	0	0	0
ENSG00000230923	2	0	1	0
ENSG00000230925	0	0	0	0
ENSG00000230926	0	0	1	0
ENSG00000230927	0	0	0	4
ENSG00000230928	0	0	0	0
ENSG00000230929	0	0	0	0
ENSG00000230930	0	0	0	0
ENSG00000230931	0	0	0	0
ENSG00000230932	0	0	0	0
ENSG00000230934	0	0	0	0
ENSG00000230935	0	0	0	0
ENSG00000230936	0	0	0	0
ENSG00000230937	0	0	0	0
ENSG00000230939	13	0	3	4
ENSG00000230940	0	0	0	0
ENSG00000230941	0	0	0	0
ENSG00000230942	0	0	0	0
ENSG00000230943	0	0	0	0
ENSG00000230944	0	0	0	0
ENSG00000230945	0	0	0	0
ENSG00000230946	0	0	0	0
ENSG00000230947	0	0	0	0
ENSG00000230948	0	0	0	0
ENSG00000230949	0	0	0	0

ENSG00000230950	0	0	0	0
ENSG00000230951	0	0	0	0
ENSG00000230952	0	0	1	3
ENSG00000230953	0	0	0	0
ENSG00000230955	0	0	3	0
ENSG00000230956	0	0	0	0
ENSG00000230957	0	0	0	0
ENSG00000230958	0	0	0	0
ENSG00000230960	0	0	0	0
ENSG00000230961	0	0	0	0
ENSG00000230962	0	0	0	0
ENSG00000230964	0	0	1	6
ENSG00000230965	0	0	2	1
ENSG00000230967	0	0	0	0
ENSG00000230968	0	0	0	0
ENSG00000230969	0	0	0	0
ENSG00000230970	0	0	0	0
ENSG00000230971	0	0	0	0
ENSG00000230972	0	0	0	0
ENSG00000230973	0	0	0	0
ENSG00000230975	0	0	0	0
ENSG00000230977	0	0	0	0
ENSG00000230978	0	0	0	0
ENSG00000230979	2	0	0	0
ENSG00000230980	0	0	0	0
ENSG00000230981	1	0	0	0
ENSG00000230982	2	0	3	0
ENSG00000230983	0	0	0	0
ENSG00000230985	0	0	0	0
ENSG00000230986	0	0	0	0
ENSG00000230987	0	0	0	0
ENSG00000230988	0	0	0	0
ENSG00000230989	2342	4046	12727	11945
ENSG00000230990	0	0	0	0
ENSG00000230991	0	0	0	0
ENSG00000230992	0	0	1	10
ENSG00000230993	0	0	0	0
ENSG00000230994	0	0	0	0
ENSG00000230995	0	0	0	0
ENSG00000230997	0	0	0	0
ENSG00000230998	0	0	0	0
ENSG00000230999	0	0	0	0
ENSG00000231000	0	0	0	0
ENSG00000231001	0	0	0	0
ENSG00000231002	0	0	0	0
ENSG00000231003	0	0	0	0

ENSG00000231004	0	0	0	0
ENSG00000231005	0	0	0	0
ENSG00000231006	3	0	0	0
ENSG00000231007	0	0	0	0
ENSG00000231009	0	0	0	2
ENSG00000231010	0	0	0	0
ENSG00000231011	0	0	0	0
ENSG00000231013	0	0	4	0
ENSG00000231015	0	0	0	0
ENSG00000231017	0	0	0	0
ENSG00000231018	0	0	0	0
ENSG00000231019	0	0	0	0
ENSG00000231020	0	0	0	0
ENSG00000231021	0	0	0	0
ENSG00000231022	0	0	0	0
ENSG00000231023	0	0	0	0
ENSG00000231024	0	0	0	0
ENSG00000231025	64	218	290	236
ENSG00000231026	0	0	0	0
ENSG00000231027	10	0	0	0
ENSG00000231028	0	11	0	0
ENSG00000231029	0	0	0	0
ENSG00000231030	0	0	0	0
ENSG00000231031	0	0	0	2
ENSG00000231032	0	0	0	0
ENSG00000231034	1	0	0	0
ENSG00000231035	0	0	0	0
ENSG00000231037	0	0	0	0
ENSG00000231039	0	0	1	5
ENSG00000231040	0	0	0	0
ENSG00000231041	0	0	0	0
ENSG00000231043	0	0	0	0
ENSG00000231044	0	0	0	0
ENSG00000231046	0	0	0	0
ENSG00000231047	0	0	0	0
ENSG00000231048	0	0	0	0
ENSG00000231049	0	0	0	0
ENSG00000231050	0	0	0	0
ENSG00000231051	0	0	0	0
ENSG00000231052	0	0	0	0
ENSG00000231053	0	0	0	0
ENSG00000231054	0	0	0	0
ENSG00000231056	0	0	0	0
ENSG00000231057	0	0	0	0
ENSG00000231058	0	0	2	0
ENSG00000231060	0	0	0	0

ENSG00000231061	0	0	0	0
ENSG00000231062	0	0	0	0
ENSG00000231063	0	0	0	0
ENSG00000231064	0	0	0	0
ENSG00000231066	0	0	0	0
ENSG00000231067	0	0	0	0
ENSG00000231068	0	0	0	0
ENSG00000231069	0	0	0	0
ENSG00000231070	0	0	0	0
ENSG00000231071	0	0	0	0
ENSG00000231072	0	0	0	0
ENSG00000231073	9	0	2	4
ENSG00000231074	6	30	33	38
ENSG00000231078	0	0	0	0
ENSG00000231079	0	0	0	0
ENSG00000231080	0	0	0	0
ENSG00000231081	0	0	0	0
ENSG00000231082	0	0	0	0
ENSG00000231083	0	0	0	0
ENSG00000231084	0	0	0	0
ENSG00000231086	5	41	2	2
ENSG00000231087	0	0	0	0
ENSG00000231090	0	0	0	0
ENSG00000231091	0	0	0	0
ENSG00000231092	0	0	0	0
ENSG00000231093	0	0	0	0
ENSG00000231094	0	0	0	0
ENSG00000231095	0	0	0	0
ENSG00000231096	0	0	0	0
ENSG00000231098	0	0	0	0
ENSG00000231099	0	0	0	0
ENSG00000231100	0	0	0	0
ENSG00000231102	0	0	0	0
ENSG00000231103	0	0	0	0
ENSG00000231104	0	0	9	0
ENSG00000231105	0	0	0	0
ENSG00000231106	0	0	0	0
ENSG00000231107	0	0	0	0
ENSG00000231108	0	0	0	0
ENSG00000231110	0	0	0	0
ENSG00000231111	0	0	0	0
ENSG00000231112	0	0	0	0
ENSG00000231113	2	100	85	143
ENSG00000231114	0	0	0	0
ENSG00000231115	0	0	0	0
ENSG00000231116	0	0	0	0

ENSG00000231118	0	0	0	0
ENSG00000231119	18	0	13	5
ENSG00000231120	0	0	0	0
ENSG00000231121	0	0	8	0
ENSG00000231122	0	0	0	0
ENSG00000231123	0	0	0	0
ENSG00000231124	0	0	0	0
ENSG00000231125	0	0	0	0
ENSG00000231128	0	7	0	2
ENSG00000231129	0	0	0	0
ENSG00000231130	0	0	0	0
ENSG00000231131	2	8	73	137
ENSG00000231132	0	0	0	0
ENSG00000231133	0	0	0	0
ENSG00000231134	0	0	0	0
ENSG00000231135	0	0	0	0
ENSG00000231136	0	0	0	0
ENSG00000231137	0	0	0	0
ENSG00000231138	0	0	0	0
ENSG00000231139	0	0	0	0
ENSG00000231140	0	0	0	0
ENSG00000231141	0	0	0	0
ENSG00000231143	0	0	0	0
ENSG00000231144	0	0	0	0
ENSG00000231145	0	0	0	0
ENSG00000231147	0	0	0	0
ENSG00000231148	0	0	0	0
ENSG00000231149	0	0	0	0
ENSG00000231150	0	0	0	0
ENSG00000231152	0	0	0	0
ENSG00000231153	0	0	0	0
ENSG00000231154	0	0	1	0
ENSG00000231156	0	0	0	0
ENSG00000231157	0	0	0	0
ENSG00000231158	0	0	0	0
ENSG00000231159	0	0	0	0
ENSG00000231160	4	0	10	6
ENSG00000231162	0	0	0	0
ENSG00000231163	0	0	0	0
ENSG00000231164	0	0	0	4
ENSG00000231165	0	17	19	14
ENSG00000231166	0	0	0	0
ENSG00000231167	0	0	1	1
ENSG00000231169	0	0	0	0
ENSG00000231170	1	0	0	0
ENSG00000231171	0	0	0	0

ENSG00000231172	0	0	0	0
ENSG00000231173	0	0	0	0
ENSG00000231175	0	0	0	0
ENSG00000231177	0	0	0	0
ENSG00000231178	0	0	0	0
ENSG00000231179	0	0	0	0
ENSG00000231181	0	0	0	0
ENSG00000231182	0	0	0	0
ENSG00000231183	0	0	0	0
ENSG00000231184	0	0	0	0
ENSG00000231185	0	0	0	0
ENSG00000231187	0	0	0	0
ENSG00000231188	0	0	0	0
ENSG00000231189	0	0	0	0
ENSG00000231190	0	0	0	0
ENSG00000231192	0	0	0	0
ENSG00000231193	0	0	0	0
ENSG00000231194	0	0	0	0
ENSG00000231195	0	0	0	0
ENSG00000231196	0	0	0	0
ENSG00000231197	0	0	0	0
ENSG00000231198	0	0	0	0
ENSG00000231199	0	0	0	0
ENSG00000231200	0	0	0	0
ENSG00000231201	0	0	0	0
ENSG00000231202	0	0	0	0
ENSG00000231203	0	0	0	0
ENSG00000231204	0	0	0	0
ENSG00000231205	0	0	0	5
ENSG00000231206	0	0	1	0
ENSG00000231207	0	0	0	0
ENSG00000231208	0	0	0	0
ENSG00000231209	0	0	2	0
ENSG00000231210	0	0	0	0
ENSG00000231211	0	0	0	0
ENSG00000231212	0	8	3	25
ENSG00000231213	0	0	0	0
ENSG00000231216	0	0	0	0
ENSG00000231217	0	0	2	0
ENSG00000231219	0	0	0	0
ENSG00000231221	0	0	0	0
ENSG00000231222	0	0	0	0
ENSG00000231225	0	0	0	0
ENSG00000231226	0	0	0	0
ENSG00000231227	0	0	0	0
ENSG00000231228	0	0	1	0

ENSG00000231231	0	0	0	0
ENSG00000231232	0	0	0	0
ENSG00000231233	8	80	177	67
ENSG00000231234	1	20	11	31
ENSG00000231235	0	0	0	0
ENSG00000231236	0	0	0	0
ENSG00000231237	0	0	0	0
ENSG00000231238	0	0	0	0
ENSG00000231240	0	0	0	0
ENSG00000231241	0	0	0	0
ENSG00000231242	0	0	0	0
ENSG00000231243	0	0	0	0
ENSG00000231244	0	0	0	0
ENSG00000231245	1	0	3	0
ENSG00000231246	0	10	0	0
ENSG00000231247	0	0	0	0
ENSG00000231248	0	0	0	0
ENSG00000231249	8	4	13	2
ENSG00000231251	0	0	0	0
ENSG00000231252	2	13	4	21
ENSG00000231253	0	0	0	0
ENSG00000231254	0	0	0	0
ENSG00000231255	0	0	1	0
ENSG00000231256	13	55	120	124
ENSG00000231257	0	0	0	0
ENSG00000231258	0	0	10	23
ENSG00000231259	7	37	5	35
ENSG00000231261	0	0	0	0
ENSG00000231264	0	0	0	0
ENSG00000231265	0	0	0	0
ENSG00000231266	0	0	0	0
ENSG00000231267	0	0	0	0
ENSG00000231268	0	0	0	0
ENSG00000231270	0	0	0	0
ENSG00000231271	0	0	0	0
ENSG00000231272	0	0	0	0
ENSG00000231273	0	0	0	0
ENSG00000231274	0	0	0	0
ENSG00000231275	0	0	0	0
ENSG00000231276	0	0	0	0
ENSG00000231278	0	0	0	0
ENSG00000231279	0	0	0	0
ENSG00000231280	0	0	0	0
ENSG00000231281	0	0	0	0
ENSG00000231283	0	0	0	0
ENSG00000231286	0	0	0	0

ENSG00000231287	0	0	0	0
ENSG00000231288	0	0	0	0
ENSG00000231289	0	0	0	0
ENSG00000231290	2	0	5	17
ENSG00000231291	0	0	0	0
ENSG00000231292	0	0	0	0
ENSG00000231293	0	0	0	0
ENSG00000231294	2	0	0	0
ENSG00000231295	0	0	0	0
ENSG00000231296	0	0	0	5
ENSG00000231297	0	0	0	0
ENSG00000231298	0	0	4	6
ENSG00000231299	0	0	0	0
ENSG00000231300	0	0	0	0
ENSG00000231301	0	0	0	0
ENSG00000231302	0	0	0	0
ENSG00000231304	4	0	6	0
ENSG00000231305	0	1	1	1
ENSG00000231307	1	0	3	0
ENSG00000231309	0	0	0	0
ENSG00000231310	0	0	0	0
ENSG00000231311	0	0	0	0
ENSG00000231312	6	0	2	13
ENSG00000231313	0	0	2	0
ENSG00000231314	0	0	0	0
ENSG00000231315	0	0	0	0
ENSG00000231316	0	0	0	0
ENSG00000231317	0	0	0	0
ENSG00000231319	0	0	0	0
ENSG00000231320	0	0	0	0
ENSG00000231321	0	0	0	0
ENSG00000231322	0	0	0	5
ENSG00000231324	0	0	0	0
ENSG00000231325	0	0	0	0
ENSG00000231326	0	0	0	0
ENSG00000231327	8	3	0	0
ENSG00000231328	0	0	0	0
ENSG00000231329	0	21	1	0
ENSG00000231331	0	0	0	0
ENSG00000231332	0	0	0	0
ENSG00000231333	0	0	0	0
ENSG00000231334	0	0	0	0
ENSG00000231335	0	0	0	0
ENSG00000231336	0	0	0	0
ENSG00000231340	0	0	0	0
ENSG00000231341	0	0	0	0

ENSG00000231342	0	0	0	0
ENSG00000231343	0	0	0	0
ENSG00000231344	0	0	0	0
ENSG00000231345	0	0	1	0
ENSG00000231346	0	0	0	0
ENSG00000231349	0	0	0	0
ENSG00000231350	0	0	0	0
ENSG00000231351	0	0	1	0
ENSG00000231353	0	0	0	0
ENSG00000231355	0	0	0	0
ENSG00000231356	0	0	0	0
ENSG00000231357	0	0	0	0
ENSG00000231358	0	0	0	0
ENSG00000231359	0	0	0	0
ENSG00000231360	1	38	31	75
ENSG00000231361	0	0	0	0
ENSG00000231362	0	0	0	0
ENSG00000231363	0	0	0	0
ENSG00000231364	0	0	0	0
ENSG00000231365	1	0	21	24
ENSG00000231366	0	0	0	0
ENSG00000231367	0	0	0	0
ENSG00000231368	0	0	0	0
ENSG00000231369	0	0	0	0
ENSG00000231370	0	0	0	0
ENSG00000231371	0	0	0	0
ENSG00000231372	0	0	0	0
ENSG00000231373	0	0	0	4
ENSG00000231375	0	0	0	0
ENSG00000231376	0	0	0	0
ENSG00000231377	0	0	0	0
ENSG00000231378	0	0	0	0
ENSG00000231379	0	0	0	0
ENSG00000231381	0	0	0	0
ENSG00000231382	0	0	0	0
ENSG00000231383	0	0	0	0
ENSG00000231384	0	0	0	0
ENSG00000231385	0	0	0	0
ENSG00000231386	0	0	0	0
ENSG00000231388	0	0	0	0
ENSG00000231389	156	246	108	111
ENSG00000231390	0	0	0	0
ENSG00000231391	0	0	0	0
ENSG00000231392	0	0	0	0
ENSG00000231393	0	0	0	0
ENSG00000231394	0	0	0	0

ENSG00000231395	0	0	0	0
ENSG00000231396	0	0	0	0
ENSG00000231397	0	0	0	0
ENSG00000231398	0	0	0	0
ENSG00000231399	0	0	0	0
ENSG00000231401	0	0	0	0
ENSG00000231402	0	0	0	0
ENSG00000231403	0	0	0	0
ENSG00000231404	0	0	0	0
ENSG00000231405	0	0	0	0
ENSG00000231407	19	38	73	68
ENSG00000231408	0	0	0	0
ENSG00000231409	0	0	0	0
ENSG00000231411	0	0	0	0
ENSG00000231412	1	0	0	0
ENSG00000231413	0	0	0	0
ENSG00000231414	0	0	0	0
ENSG00000231416	0	0	0	0
ENSG00000231417	0	0	0	0
ENSG00000231418	0	0	0	0
ENSG00000231419	0	0	0	0
ENSG00000231420	0	0	0	0
ENSG00000231421	0	0	0	0
ENSG00000231422	0	0	0	0
ENSG00000231423	0	0	0	0
ENSG00000231424	0	0	0	0
ENSG00000231426	0	0	0	0
ENSG00000231427	0	0	0	0
ENSG00000231428	0	0	0	0
ENSG00000231429	0	0	0	0
ENSG00000231430	0	0	0	0
ENSG00000231431	0	0	0	0
ENSG00000231433	0	0	0	0
ENSG00000231434	0	0	0	0
ENSG00000231435	0	0	0	0
ENSG00000231436	0	0	0	0
ENSG00000231437	163	114	32	51
ENSG00000231439	2	0	0	0
ENSG00000231440	0	0	0	0
ENSG00000231441	0	0	0	0
ENSG00000231442	0	0	1	0
ENSG00000231443	1	0	0	0
ENSG00000231444	0	0	0	0
ENSG00000231445	0	0	0	0
ENSG00000231447	0	0	0	0
ENSG00000231448	0	0	0	7

ENSG00000231449	0	0	0	0
ENSG00000231450	0	0	0	0
ENSG00000231453	0	0	0	0
ENSG00000231454	0	0	0	0
ENSG00000231457	0	0	0	0
ENSG00000231458	0	0	0	0
ENSG00000231459	0	0	1	0
ENSG00000231460	1	0	0	0
ENSG00000231461	0	0	0	0
ENSG00000231464	0	0	0	0
ENSG00000231465	0	0	0	9
ENSG00000231466	0	0	0	0
ENSG00000231467	0	0	0	0
ENSG00000231468	0	0	0	0
ENSG00000231470	0	0	0	0
ENSG00000231471	0	0	0	0
ENSG00000231473	0	0	3	4
ENSG00000231474	0	0	0	0
ENSG00000231475	0	0	0	0
ENSG00000231476	0	0	0	0
ENSG00000231477	0	0	0	0
ENSG00000231478	0	0	0	0
ENSG00000231479	0	0	0	0
ENSG00000231480	0	0	0	0
ENSG00000231481	0	0	0	0
ENSG00000231482	0	0	0	0
ENSG00000231483	0	0	0	0
ENSG00000231484	0	0	0	0
ENSG00000231485	0	0	0	1
ENSG00000231486	2	0	2	0
ENSG00000231487	0	0	0	0
ENSG00000231488	0	0	0	0
ENSG00000231489	0	0	0	0
ENSG00000231490	0	0	0	0
ENSG00000231491	0	0	0	0
ENSG00000231492	0	0	0	0
ENSG00000231494	0	0	0	0
ENSG00000231495	0	0	0	0
ENSG00000231496	0	0	0	0
ENSG00000231500	1367	155	71	160
ENSG00000231501	0	0	0	0
ENSG00000231502	0	0	0	0
ENSG00000231503	0	0	0	0
ENSG00000231504	0	0	0	0
ENSG00000231505	0	0	0	0
ENSG00000231507	0	0	2	0

ENSG00000231508	0	0	0	0
ENSG00000231509	0	0	0	0
ENSG00000231510	0	0	0	0
ENSG00000231511	0	0	0	0
ENSG00000231512	0	0	0	3
ENSG00000231513	16	2053	1208	2103
ENSG00000231514	0	0	0	0
ENSG00000231515	0	0	0	0
ENSG00000231516	0	0	0	0
ENSG00000231517	0	0	0	0
ENSG00000231518	0	0	0	0
ENSG00000231519	0	0	0	0
ENSG00000231521	0	0	0	0
ENSG00000231522	0	0	0	0
ENSG00000231523	0	0	0	0
ENSG00000231525	0	0	0	0
ENSG00000231526	0	0	0	0
ENSG00000231527	0	0	0	0
ENSG00000231528	1	0	0	0
ENSG00000231529	0	0	0	0
ENSG00000231530	0	0	0	0
ENSG00000231531	0	0	0	0
ENSG00000231532	0	0	0	0
ENSG00000231533	0	0	1	5
ENSG00000231534	0	0	0	0
ENSG00000231535	2	0	0	16
ENSG00000231536	0	0	0	0
ENSG00000231537	0	0	0	0
ENSG00000231538	0	0	0	0
ENSG00000231539	1	4	0	1
ENSG00000231540	0	0	0	0
ENSG00000231541	0	0	0	0
ENSG00000231542	0	0	1	0
ENSG00000231543	0	0	0	0
ENSG00000231544	0	0	0	0
ENSG00000231546	0	0	0	0
ENSG00000231547	0	0	0	0
ENSG00000231548	0	0	0	0
ENSG00000231549	0	0	0	0
ENSG00000231550	0	0	0	0
ENSG00000231551	0	0	0	0
ENSG00000231552	0	0	0	0
ENSG00000231553	0	0	0	0
ENSG00000231555	0	0	0	0
ENSG00000231556	0	0	0	0
ENSG00000231557	0	0	0	0

ENSG00000231558	0	0	0	0
ENSG00000231559	0	0	0	0
ENSG00000231560	0	0	0	0
ENSG00000231561	0	0	0	0
ENSG00000231562	0	0	0	0
ENSG00000231563	0	0	0	0
ENSG00000231564	0	0	0	0
ENSG00000231565	0	0	0	0
ENSG00000231566	0	0	0	1
ENSG00000231567	0	0	0	0
ENSG00000231568	0	0	0	0
ENSG00000231569	0	0	0	0
ENSG00000231570	0	0	0	0
ENSG00000231573	0	0	0	0
ENSG00000231574	0	0	0	0
ENSG00000231575	0	0	0	0
ENSG00000231576	0	0	0	0
ENSG00000231577	0	0	0	0
ENSG00000231578	0	0	0	0
ENSG00000231579	0	0	0	0
ENSG00000231580	0	0	0	0
ENSG00000231581	10	0	9	11
ENSG00000231582	0	0	0	0
ENSG00000231583	1	0	0	0
ENSG00000231584	1	10	4	0
ENSG00000231585	0	0	0	0
ENSG00000231586	0	0	0	0
ENSG00000231587	0	0	0	0
ENSG00000231588	0	10	0	0
ENSG00000231589	0	0	0	0
ENSG00000231590	0	0	0	0
ENSG00000231591	0	0	0	0
ENSG00000231592	0	0	0	0
ENSG00000231593	0	0	0	0
ENSG00000231595	1	0	0	9
ENSG00000231596	0	0	0	0
ENSG00000231597	0	0	0	0
ENSG00000231599	0	0	5	7
ENSG00000231600	0	0	0	0
ENSG00000231601	0	0	0	0
ENSG00000231602	0	0	0	0
ENSG00000231603	0	0	0	0
ENSG00000231604	0	0	0	0
ENSG00000231605	0	0	0	0
ENSG00000231606	0	0	0	0
ENSG00000231607	446	6201	4794	7407

ENSG00000231608	0	0	0	0
ENSG00000231609	2	0	7	12
ENSG00000231610	0	0	0	0
ENSG00000231611	0	0	0	0
ENSG00000231612	0	0	0	0
ENSG00000231613	1	0	0	0
ENSG00000231615	0	0	0	0
ENSG00000231616	0	0	0	1
ENSG00000231617	0	0	0	0
ENSG00000231618	0	0	0	0
ENSG00000231619	0	0	0	0
ENSG00000231620	0	0	0	0
ENSG00000231621	6	0	0	0
ENSG00000231622	0	0	0	0
ENSG00000231623	0	0	0	0
ENSG00000231624	0	0	0	0
ENSG00000231625	0	0	0	0
ENSG00000231626	0	0	0	0
ENSG00000231628	0	0	0	0
ENSG00000231630	0	0	0	0
ENSG00000231631	0	0	0	0
ENSG00000231632	0	0	0	0
ENSG00000231633	0	0	0	0
ENSG00000231635	0	0	0	0
ENSG00000231636	7	0	0	2
ENSG00000231637	0	0	0	0
ENSG00000231638	0	0	0	0
ENSG00000231641	0	0	0	0
ENSG00000231643	0	0	2	2
ENSG00000231645	0	0	0	0
ENSG00000231646	0	27	7	27
ENSG00000231647	0	0	0	0
ENSG00000231648	0	0	0	0
ENSG00000231649	0	0	0	0
ENSG00000231650	0	0	0	0
ENSG00000231651	0	0	0	0
ENSG00000231652	0	119	31	151
ENSG00000231653	0	0	0	0
ENSG00000231654	0	0	0	0
ENSG00000231655	0	0	0	0
ENSG00000231656	0	0	0	0
ENSG00000231659	0	0	0	0
ENSG00000231660	0	0	0	0
ENSG00000231662	0	0	0	0
ENSG00000231663	9	3	1	0
ENSG00000231665	0	0	0	0

ENSG00000231666	0	0	0	1
ENSG00000231667	0	0	0	0
ENSG00000231668	0	0	0	0
ENSG00000231669	3621	2366	2914	2185
ENSG00000231670	0	0	0	0
ENSG00000231671	0	0	0	0
ENSG00000231672	0	9	14	26
ENSG00000231674	0	0	0	0
ENSG00000231675	0	0	0	0
ENSG00000231676	0	0	0	0
ENSG00000231677	0	0	0	0
ENSG00000231678	0	0	0	0
ENSG00000231679	0	0	0	0
ENSG00000231680	0	0	0	0
ENSG00000231681	0	0	0	0
ENSG00000231682	0	0	0	0
ENSG00000231683	0	0	0	0
ENSG00000231684	0	1	0	0
ENSG00000231686	0	0	0	0
ENSG00000231688	0	0	0	0
ENSG00000231689	0	0	0	0
ENSG00000231690	0	0	0	0
ENSG00000231691	0	0	0	0
ENSG00000231694	0	0	0	0
ENSG00000231697	0	0	0	6
ENSG00000231698	0	0	0	0
ENSG00000231699	0	0	0	0
ENSG00000231700	0	0	0	0
ENSG00000231701	0	0	0	0
ENSG00000231702	0	0	0	0
ENSG00000231703	5	0	1	0
ENSG00000231704	1	0	0	0
ENSG00000231705	0	0	0	0
ENSG00000231706	0	0	0	0
ENSG00000231707	0	0	0	0
ENSG00000231709	0	0	0	0
ENSG00000231710	0	0	0	0
ENSG00000231711	7	0	2	2
ENSG00000231712	0	0	0	0
ENSG00000231713	0	0	0	0
ENSG00000231714	0	0	0	0
ENSG00000231715	0	0	0	0
ENSG00000231716	0	0	0	0
ENSG00000231718	0	0	0	0
ENSG00000231719	0	0	0	0
ENSG00000231720	0	0	0	0

ENSG00000231721	60	240	166	145
ENSG00000231722	0	0	0	0
ENSG00000231723	0	0	0	0
ENSG00000231724	0	0	0	0
ENSG00000231725	28	386	361	500
ENSG00000231726	0	0	0	0
ENSG00000231728	3	8	2	1
ENSG00000231729	0	0	0	0
ENSG00000231731	0	0	0	0
ENSG00000231733	0	0	0	0
ENSG00000231734	0	0	9	5
ENSG00000231735	0	0	0	0
ENSG00000231737	0	0	0	0
ENSG00000231738	0	0	0	0
ENSG00000231739	0	0	0	0
ENSG00000231740	0	0	0	0
ENSG00000231741	0	0	0	0
ENSG00000231742	2	0	3	0
ENSG00000231743	1	0	1	5
ENSG00000231744	0	0	0	0
ENSG00000231745	0	0	0	0
ENSG00000231746	0	0	0	0
ENSG00000231747	3	0	0	0
ENSG00000231748	0	0	0	0
ENSG00000231749	0	0	0	0
ENSG00000231750	0	0	0	0
ENSG00000231752	0	0	3	2
ENSG00000231754	1	0	0	3
ENSG00000231755	10	14	6	24
ENSG00000231756	0	0	0	0
ENSG00000231757	0	0	0	0
ENSG00000231758	0	0	0	0
ENSG00000231759	0	0	0	0
ENSG00000231760	0	0	0	0
ENSG00000231762	0	0	0	0
ENSG00000231763	0	0	0	0
ENSG00000231764	0	0	0	0
ENSG00000231765	0	0	2	0
ENSG00000231766	0	0	0	0
ENSG00000231767	2	0	2	0
ENSG00000231768	0	0	0	1
ENSG00000231769	0	0	0	0
ENSG00000231770	11	0	11	27
ENSG00000231771	0	0	0	0
ENSG00000231772	0	0	0	0
ENSG00000231773	0	0	0	0

ENSG00000231774	0	0	0	0
ENSG00000231775	0	0	0	0
ENSG00000231776	0	0	0	0
ENSG00000231777	0	0	0	0
ENSG00000231780	0	0	0	0
ENSG00000231781	0	0	0	0
ENSG00000231782	0	0	0	0
ENSG00000231784	0	0	0	0
ENSG00000231785	0	0	0	0
ENSG00000231787	0	0	0	0
ENSG00000231788	0	0	0	0
ENSG00000231789	0	2	1	0
ENSG00000231791	0	0	0	0
ENSG00000231793	0	0	0	1
ENSG00000231794	0	0	0	0
ENSG00000231795	0	0	0	0
ENSG00000231799	1	0	0	1
ENSG00000231801	0	0	0	0
ENSG00000231802	0	0	0	0
ENSG00000231804	0	0	0	0
ENSG00000231806	0	0	0	0
ENSG00000231808	0	0	0	0
ENSG00000231809	0	0	0	0
ENSG00000231810	0	0	0	0
ENSG00000231811	0	0	0	0
ENSG00000231812	0	0	0	0
ENSG00000231813	0	0	0	0
ENSG00000231814	0	0	0	0
ENSG00000231815	0	0	0	0
ENSG00000231816	0	0	0	0
ENSG00000231817	0	0	0	0
ENSG00000231821	0	0	0	0
ENSG00000231822	0	0	0	0
ENSG00000231823	0	0	0	0
ENSG00000231824	0	0	0	0
ENSG00000231825	0	0	0	0
ENSG00000231826	0	0	0	0
ENSG00000231827	0	0	0	0
ENSG00000231829	0	0	3	3
ENSG00000231830	5	0	1	2
ENSG00000231831	1	0	0	0
ENSG00000231834	0	0	0	0
ENSG00000231836	0	0	0	0
ENSG00000231837	0	0	0	0
ENSG00000231838	0	0	0	0
ENSG00000231839	0	0	0	0

ENSG00000231840	0	0	0	3
ENSG00000231841	0	16	0	12
ENSG00000231842	0	0	0	0
ENSG00000231845	0	0	0	0
ENSG00000231846	0	0	0	0
ENSG00000231848	0	0	0	0
ENSG00000231849	0	0	0	0
ENSG00000231850	0	0	0	0
ENSG00000231851	0	0	0	0
ENSG00000231852	0	0	0	0
ENSG00000231854	0	0	0	0
ENSG00000231855	0	0	0	0
ENSG00000231856	0	0	0	0
ENSG00000231857	0	0	0	0
ENSG00000231858	0	0	1	0
ENSG00000231859	0	0	0	0
ENSG00000231860	0	0	0	0
ENSG00000231861	0	0	0	0
ENSG00000231862	0	0	0	0
ENSG00000231863	0	0	0	0
ENSG00000231864	0	0	0	0
ENSG00000231865	0	0	1	1
ENSG00000231866	0	0	0	0
ENSG00000231867	0	0	0	0
ENSG00000231868	0	0	0	0
ENSG00000231869	0	0	0	0
ENSG00000231870	0	0	0	0
ENSG00000231871	0	0	0	5
ENSG00000231873	0	0	0	0
ENSG00000231874	0	0	0	0
ENSG00000231875	0	19	5	19
ENSG00000231876	0	0	0	0
ENSG00000231877	0	0	1	0
ENSG00000231878	0	0	0	0
ENSG00000231879	0	0	0	0
ENSG00000231880	0	0	0	0
ENSG00000231881	0	0	0	0
ENSG00000231882	0	0	0	0
ENSG00000231883	0	0	0	0
ENSG00000231884	0	0	0	0
ENSG00000231887	0	0	0	0
ENSG00000231888	0	0	0	0
ENSG00000231889	4	36	40	19
ENSG00000231890	7	39	11	24
ENSG00000231892	0	0	0	0
ENSG00000231894	0	0	0	0

ENSG00000231895	0	0	0	0
ENSG00000231896	0	0	0	0
ENSG00000231897	0	0	0	0
ENSG00000231898	0	0	0	0
ENSG00000231900	0	3	23	20
ENSG00000231901	0	0	0	0
ENSG00000231902	0	0	0	0
ENSG00000231903	0	0	0	0
ENSG00000231904	0	0	0	0
ENSG00000231905	0	0	0	1
ENSG00000231906	0	0	0	0
ENSG00000231907	0	0	0	0
ENSG00000231908	1	0	0	0
ENSG00000231909	0	0	0	0
ENSG00000231910	0	0	0	0
ENSG00000231911	0	0	0	0
ENSG00000231912	0	0	0	0
ENSG00000231913	0	0	0	0
ENSG00000231914	0	0	0	0
ENSG00000231915	0	0	0	0
ENSG00000231916	0	0	0	0
ENSG00000231918	0	0	0	0
ENSG00000231919	0	0	0	0
ENSG00000231920	0	0	0	0
ENSG00000231922	0	0	0	0
ENSG00000231923	0	0	0	0
ENSG00000231924	0	0	0	0
ENSG00000231925	104	231	68	303
ENSG00000231926	0	0	0	0
ENSG00000231927	0	0	0	0
ENSG00000231928	0	0	0	0
ENSG00000231929	0	0	0	0
ENSG00000231930	0	0	0	0
ENSG00000231931	0	0	0	0
ENSG00000231933	0	0	2	0
ENSG00000231934	0	0	0	0
ENSG00000231937	1	0	2	0
ENSG00000231939	0	0	0	0
ENSG00000231940	1	0	0	0
ENSG00000231941	0	0	0	0
ENSG00000231942	0	0	0	0
ENSG00000231943	0	0	1	5
ENSG00000231944	0	0	0	0
ENSG00000231945	0	0	0	0
ENSG00000231947	0	0	0	0
ENSG00000231948	1	0	0	0

ENSG00000231949	0	0	0	0
ENSG00000231951	0	0	0	0
ENSG00000231952	0	309	174	330
ENSG00000231953	0	0	0	0
ENSG00000231954	0	0	0	0
ENSG00000231955	0	5	1	0
ENSG00000231956	0	0	0	0
ENSG00000231957	0	0	0	0
ENSG00000231960	0	0	0	0
ENSG00000231961	0	0	0	0
ENSG00000231962	0	0	0	0
ENSG00000231963	0	0	0	0
ENSG00000231964	0	0	0	0
ENSG00000231965	0	0	0	0
ENSG00000231966	0	0	0	0
ENSG00000231967	0	0	0	0
ENSG00000231968	0	0	0	0
ENSG00000231969	3	0	15	27
ENSG00000231970	0	0	0	0
ENSG00000231971	0	0	0	0
ENSG00000231973	0	0	0	0
ENSG00000231974	0	0	0	0
ENSG00000231975	0	0	0	0
ENSG00000231976	0	0	0	0
ENSG00000231977	0	0	0	0
ENSG00000231978	0	0	0	0
ENSG00000231979	0	0	0	0
ENSG00000231980	0	0	0	0
ENSG00000231981	0	0	0	0
ENSG00000231982	0	0	0	0
ENSG00000231983	0	0	0	0
ENSG00000231984	0	0	0	0
ENSG00000231985	0	0	0	0
ENSG00000231986	0	0	0	0
ENSG00000231987	0	0	0	0
ENSG00000231988	0	0	0	0
ENSG00000231989	0	0	2	1
ENSG00000231990	0	0	0	0
ENSG00000231991	0	0	0	1
ENSG00000231992	0	0	1	0
ENSG00000231993	0	0	0	0
ENSG00000231995	0	0	0	0
ENSG00000231996	0	0	0	0
ENSG00000231997	0	0	0	0
ENSG00000231998	0	0	0	0
ENSG00000231999	4	62	75	24

ENSG00000232000	0	1	1	2
ENSG00000232001	0	0	0	0
ENSG00000232002	0	0	0	1
ENSG00000232003	0	0	0	0
ENSG00000232004	0	0	0	0
ENSG00000232005	0	0	0	0
ENSG00000232006	0	0	0	0
ENSG00000232007	0	0	0	0
ENSG00000232009	0	0	0	0
ENSG00000232010	0	0	0	0
ENSG00000232011	0	0	0	0
ENSG00000232013	0	0	1	0
ENSG00000232014	0	0	0	0
ENSG00000232015	0	0	0	0
ENSG00000232017	0	0	0	0
ENSG00000232018	1	94	92	200
ENSG00000232019	1	0	0	0
ENSG00000232021	1	52	19	16
ENSG00000232022	1	0	0	0
ENSG00000232023	0	0	0	0
ENSG00000232024	0	0	3	0
ENSG00000232025	0	0	0	0
ENSG00000232026	0	0	0	0
ENSG00000232027	0	0	0	0
ENSG00000232028	0	0	0	0
ENSG00000232029	0	0	0	0
ENSG00000232030	0	0	0	0
ENSG00000232031	0	0	0	0
ENSG00000232032	0	0	0	0
ENSG00000232034	0	0	0	0
ENSG00000232035	0	0	0	0
ENSG00000232036	0	10	0	0
ENSG00000232037	0	0	0	0
ENSG00000232039	0	0	0	0
ENSG00000232040	0	0	0	0
ENSG00000232041	0	0	0	0
ENSG00000232042	0	0	0	0
ENSG00000232043	0	42	7	8
ENSG00000232044	0	0	0	0
ENSG00000232045	0	0	0	0
ENSG00000232046	0	24	22	40
ENSG00000232048	0	0	0	0
ENSG00000232049	0	0	0	0
ENSG00000232050	0	0	0	0
ENSG00000232053	1	0	5	6
ENSG00000232054	0	0	0	0

ENSG00000232056	0	0	0	0
ENSG00000232057	0	0	0	0
ENSG00000232058	0	0	0	0
ENSG00000232059	0	0	0	0
ENSG00000232060	0	0	0	0
ENSG00000232062	0	0	0	0
ENSG00000232063	0	0	0	0
ENSG00000232064	0	0	0	0
ENSG00000232065	220	36	104	230
ENSG00000232068	0	0	0	0
ENSG00000232069	0	0	0	0
ENSG00000232070	0	0	13	8
ENSG00000232072	0	0	0	0
ENSG00000232073	0	0	0	0
ENSG00000232075	0	0	0	0
ENSG00000232077	0	0	0	17
ENSG00000232079	1	0	0	0
ENSG00000232080	0	2	0	0
ENSG00000232081	0	0	0	0
ENSG00000232082	0	0	0	0
ENSG00000232083	0	0	0	0
ENSG00000232084	0	0	0	0
ENSG00000232085	0	0	2	0
ENSG00000232086	0	0	0	0
ENSG00000232087	0	0	0	0
ENSG00000232089	0	0	0	0
ENSG00000232090	0	0	0	0
ENSG00000232091	0	0	0	0
ENSG00000232093	1	0	1	2
ENSG00000232095	0	0	0	0
ENSG00000232096	0	0	0	0
ENSG00000232097	0	0	0	0
ENSG00000232098	194	50	11	23
ENSG00000232099	0	0	0	0
ENSG00000232100	0	0	0	0
ENSG00000232101	0	0	0	0
ENSG00000232102	0	0	0	0
ENSG00000232104	5	104	120	265
ENSG00000232105	0	0	0	0
ENSG00000232106	0	0	0	0
ENSG00000232109	0	0	0	0
ENSG00000232110	0	0	0	0
ENSG00000232111	0	0	0	0
ENSG00000232112	1791	2429	1285	2915
ENSG00000232113	0	0	0	0
ENSG00000232114	0	0	0	0

ENSG00000232115	0	0	0	0
ENSG00000232116	0	0	4	4
ENSG00000232117	0	0	0	0
ENSG00000232118	1	0	1	0
ENSG00000232119	43	31	38	37
ENSG00000232120	0	0	0	0
ENSG00000232121	0	0	0	0
ENSG00000232122	0	0	0	0
ENSG00000232124	0	0	0	0
ENSG00000232125	131	2207	2693	1243
ENSG00000232126	0	0	0	0
ENSG00000232127	0	0	0	0
ENSG00000232128	0	0	0	0
ENSG00000232129	0	0	0	0
ENSG00000232130	0	0	0	0
ENSG00000232131	0	0	0	0
ENSG00000232132	0	0	0	0
ENSG00000232133	0	0	16	2
ENSG00000232134	0	0	0	0
ENSG00000232135	0	0	0	0
ENSG00000232136	0	0	0	0
ENSG00000232138	0	0	0	0
ENSG00000232139	2	5	16	4
ENSG00000232140	0	0	0	0
ENSG00000232141	0	0	0	0
ENSG00000232142	0	0	0	0
ENSG00000232143	0	0	0	0
ENSG00000232144	0	0	0	0
ENSG00000232145	0	0	0	0
ENSG00000232146	0	0	0	0
ENSG00000232148	0	0	0	0
ENSG00000232149	0	17	2	0
ENSG00000232150	0	0	5	13
ENSG00000232151	0	12	0	1
ENSG00000232153	0	0	0	0
ENSG00000232154	0	0	0	0
ENSG00000232155	0	0	0	0
ENSG00000232156	0	0	0	0
ENSG00000232158	0	0	0	0
ENSG00000232159	0	0	0	0
ENSG00000232160	1	0	24	7
ENSG00000232161	0	0	0	0
ENSG00000232162	0	0	0	0
ENSG00000232163	0	0	0	0
ENSG00000232164	58	223	142	227
ENSG00000232165	0	0	0	0

ENSG00000232166	0	0	0	0
ENSG00000232167	0	0	0	0
ENSG00000232168	0	0	0	0
ENSG00000232169	0	0	0	0
ENSG00000232170	0	0	0	0
ENSG00000232172	0	0	0	0
ENSG00000232173	0	0	0	0
ENSG00000232174	0	0	0	0
ENSG00000232175	0	0	0	0
ENSG00000232176	0	0	2	0
ENSG00000232177	0	0	0	0
ENSG00000232178	0	0	0	0
ENSG00000232179	0	0	0	0
ENSG00000232180	0	0	0	0
ENSG00000232183	0	0	0	0
ENSG00000232184	0	0	1	10
ENSG00000232185	0	0	0	0
ENSG00000232186	0	0	0	0
ENSG00000232187	9	27	32	24
ENSG00000232188	0	0	0	0
ENSG00000232189	0	0	0	0
ENSG00000232190	0	0	0	0
ENSG00000232192	0	0	0	0
ENSG00000232193	0	0	0	0
ENSG00000232194	0	0	0	0
ENSG00000232195	0	0	0	0
ENSG00000232196	0	0	0	0
ENSG00000232197	0	0	0	0
ENSG00000232198	0	0	0	0
ENSG00000232199	0	0	0	0
ENSG00000232200	0	0	0	0
ENSG00000232202	0	0	0	0
ENSG00000232203	0	0	0	0
ENSG00000232204	0	0	0	0
ENSG00000232205	0	0	0	0
ENSG00000232208	0	0	0	0
ENSG00000232210	0	0	0	0
ENSG00000232211	0	0	0	0
ENSG00000232212	0	0	0	2
ENSG00000232215	0	13	0	2
ENSG00000232216	0	0	0	0
ENSG00000232217	0	0	0	0
ENSG00000232218	0	0	0	0
ENSG00000232220	0	0	0	0
ENSG00000232222	0	0	0	0
ENSG00000232223	0	0	0	0

ENSG00000232224	0	0	0	0
ENSG00000232225	0	0	0	0
ENSG00000232226	0	0	0	0
ENSG00000232227	0	0	0	0
ENSG00000232228	0	0	0	0
ENSG00000232229	0	0	0	0
ENSG00000232230	0	0	0	0
ENSG00000232232	0	0	0	0
ENSG00000232233	0	5	0	2
ENSG00000232234	0	0	0	0
ENSG00000232235	0	0	0	0
ENSG00000232236	0	0	0	0
ENSG00000232237	0	0	0	0
ENSG00000232238	0	0	0	0
ENSG00000232239	0	0	0	0
ENSG00000232240	0	0	0	0
ENSG00000232241	0	0	0	0
ENSG00000232242	0	0	0	0
ENSG00000232243	0	0	0	0
ENSG00000232244	0	0	0	0
ENSG00000232245	0	0	0	0
ENSG00000232249	0	0	0	0
ENSG00000232251	0	0	0	0
ENSG00000232252	0	0	0	0
ENSG00000232253	0	0	0	0
ENSG00000232254	0	0	0	0
ENSG00000232256	0	0	0	0
ENSG00000232258	0	0	0	0
ENSG00000232259	0	0	0	0
ENSG00000232260	0	0	0	0
ENSG00000232261	0	0	0	0
ENSG00000232263	0	0	0	0
ENSG00000232264	0	0	0	0
ENSG00000232265	55	0	19	233
ENSG00000232266	0	0	0	0
ENSG00000232267	0	0	0	0
ENSG00000232268	0	0	0	0
ENSG00000232270	0	0	0	2
ENSG00000232271	0	0	0	0
ENSG00000232272	0	0	0	0
ENSG00000232273	0	0	0	0
ENSG00000232274	0	1	4	1
ENSG00000232276	0	0	0	0
ENSG00000232277	0	0	0	0
ENSG00000232279	0	0	0	0
ENSG00000232280	0	0	0	0

ENSG00000232281	0	0	0	0
ENSG00000232282	0	0	0	0
ENSG00000232283	5	45	7	19
ENSG00000232284	0	16	3	8
ENSG00000232285	0	0	0	0
ENSG00000232286	0	0	0	0
ENSG00000232287	0	0	0	0
ENSG00000232289	0	0	0	0
ENSG00000232290	0	0	0	0
ENSG00000232292	0	0	0	0
ENSG00000232293	0	0	0	0
ENSG00000232294	0	0	0	0
ENSG00000232295	2	0	7	4
ENSG00000232296	0	0	0	0
ENSG00000232298	0	0	0	0
ENSG00000232299	0	0	0	0
ENSG00000232300	1	0	0	0
ENSG00000232301	0	0	0	0
ENSG00000232302	0	0	0	0
ENSG00000232303	0	0	0	0
ENSG00000232305	0	0	0	0
ENSG00000232306	0	0	0	0
ENSG00000232307	0	0	0	0
ENSG00000232308	0	0	0	0
ENSG00000232309	0	0	0	0
ENSG00000232310	0	0	0	0
ENSG00000232311	0	0	0	0
ENSG00000232312	0	0	0	0
ENSG00000232314	0	0	0	0
ENSG00000232316	0	0	0	0
ENSG00000232317	0	0	0	0
ENSG00000232320	0	0	0	0
ENSG00000232324	0	0	0	0
ENSG00000232325	0	0	0	0
ENSG00000232326	0	0	0	0
ENSG00000232327	0	0	0	0
ENSG00000232328	0	0	0	0
ENSG00000232331	0	0	0	0
ENSG00000232332	0	0	0	0
ENSG00000232333	0	0	0	0
ENSG00000232334	0	0	0	0
ENSG00000232335	0	33	5	0
ENSG00000232336	0	12	11	2
ENSG00000232337	0	0	0	0
ENSG00000232339	0	0	0	0
ENSG00000232341	0	0	0	0

ENSG00000232342	0	0	1	5
ENSG00000232343	0	0	0	0
ENSG00000232344	0	0	0	0
ENSG00000232345	0	0	0	0
ENSG00000232346	0	0	0	0
ENSG00000232347	1	13	27	36
ENSG00000232348	0	0	0	0
ENSG00000232349	0	0	0	0
ENSG00000232350	0	0	0	0
ENSG00000232351	0	0	0	0
ENSG00000232352	0	0	0	0
ENSG00000232353	0	0	0	0
ENSG00000232354	0	0	0	0
ENSG00000232355	0	0	0	0
ENSG00000232356	0	0	0	0
ENSG00000232357	0	0	0	0
ENSG00000232358	0	0	0	0
ENSG00000232359	0	0	0	0
ENSG00000232360	0	0	0	0
ENSG00000232361	0	0	0	0
ENSG00000232362	0	0	0	0
ENSG00000232363	0	0	0	0
ENSG00000232366	0	0	0	0
ENSG00000232367	0	0	0	0
ENSG00000232368	2	0	0	2
ENSG00000232369	0	0	0	0
ENSG00000232370	0	0	0	0
ENSG00000232372	0	0	0	0
ENSG00000232373	0	0	0	0
ENSG00000232374	0	0	0	0
ENSG00000232375	0	0	0	0
ENSG00000232376	0	0	0	0
ENSG00000232377	0	0	2	0
ENSG00000232378	0	0	0	0
ENSG00000232379	0	0	0	0
ENSG00000232380	0	19	0	0
ENSG00000232381	0	0	0	0
ENSG00000232382	0	0	0	0
ENSG00000232383	0	0	0	0
ENSG00000232384	0	0	0	0
ENSG00000232385	0	0	0	0
ENSG00000232386	0	0	0	0
ENSG00000232387	0	0	0	0
ENSG00000232388	48	3	6	6
ENSG00000232389	0	0	0	0
ENSG00000232390	0	0	0	0

ENSG00000232391	0	0	0	0
ENSG00000232392	0	0	5	14
ENSG00000232393	0	0	0	0
ENSG00000232394	0	0	0	0
ENSG00000232395	0	0	0	0
ENSG00000232396	0	0	0	0
ENSG00000232397	0	0	0	0
ENSG00000232398	0	0	0	0
ENSG00000232399	0	0	0	0
ENSG00000232400	0	0	0	0
ENSG00000232401	0	0	0	0
ENSG00000232403	0	0	0	0
ENSG00000232406	0	0	0	0
ENSG00000232407	0	0	0	0
ENSG00000232408	0	0	0	0
ENSG00000232409	0	0	0	0
ENSG00000232411	0	0	1	0
ENSG00000232412	28	0	23	18
ENSG00000232413	0	0	0	0
ENSG00000232414	0	0	0	0
ENSG00000232415	0	0	0	0
ENSG00000232416	0	0	0	0
ENSG00000232417	0	0	0	0
ENSG00000232418	0	0	0	0
ENSG00000232419	0	0	0	0
ENSG00000232420	0	0	0	0
ENSG00000232421	0	0	0	0
ENSG00000232422	0	0	0	0
ENSG00000232423	0	0	0	0
ENSG00000232424	0	0	0	0
ENSG00000232426	2	0	8	40
ENSG00000232429	0	0	0	0
ENSG00000232430	0	0	0	0
ENSG00000232431	0	0	0	0
ENSG00000232433	0	0	1	0
ENSG00000232434	0	0	0	0
ENSG00000232435	0	0	0	0
ENSG00000232436	0	0	0	0
ENSG00000232437	0	0	0	0
ENSG00000232438	0	0	0	0
ENSG00000232439	0	0	0	0
ENSG00000232441	0	0	0	0
ENSG00000232442	16	0	1	7
ENSG00000232444	0	10	7	4
ENSG00000232445	0	0	0	0
ENSG00000232446	0	0	0	0

ENSG00000232447	0	0	0	0
ENSG00000232448	0	0	0	0
ENSG00000232450	2	32	21	22
ENSG00000232451	0	0	0	0
ENSG00000232453	1	0	2	0
ENSG00000232454	0	0	0	0
ENSG00000232455	0	0	0	0
ENSG00000232456	0	0	0	0
ENSG00000232457	1	0	0	0
ENSG00000232458	0	0	0	0
ENSG00000232459	0	0	0	0
ENSG00000232460	0	0	0	0
ENSG00000232461	0	19	0	0
ENSG00000232462	0	0	0	0
ENSG00000232463	0	0	0	0
ENSG00000232464	0	0	0	0
ENSG00000232465	0	0	0	0
ENSG00000232466	0	0	0	0
ENSG00000232467	0	0	0	0
ENSG00000232469	0	0	0	0
ENSG00000232470	2	0	3	0
ENSG00000232471	0	0	0	0
ENSG00000232472	0	1	0	1
ENSG00000232473	0	0	0	0
ENSG00000232474	0	0	0	0
ENSG00000232475	0	0	0	0
ENSG00000232478	0	0	0	0
ENSG00000232479	0	3	1	4
ENSG00000232480	7	0	6	7
ENSG00000232482	0	0	0	0
ENSG00000232485	5	8	23	86
ENSG00000232486	0	0	0	0
ENSG00000232487	0	0	0	0
ENSG00000232489	0	0	0	0
ENSG00000232490	0	0	0	0
ENSG00000232491	0	0	0	0
ENSG00000232492	0	0	0	2
ENSG00000232493	0	0	0	0
ENSG00000232494	0	0	0	0
ENSG00000232495	0	0	0	0
ENSG00000232496	0	0	0	0
ENSG00000232497	0	0	0	0
ENSG00000232498	0	0	0	0
ENSG00000232499	0	0	0	0
ENSG00000232500	0	0	0	0
ENSG00000232502	0	0	0	0

ENSG00000232503	0	0	0	0
ENSG00000232504	0	0	0	0
ENSG00000232505	0	0	0	0
ENSG00000232508	0	0	0	0
ENSG00000232510	0	0	0	0
ENSG00000232511	0	0	0	0
ENSG00000232512	0	0	0	0
ENSG00000232514	0	0	0	0
ENSG00000232515	0	0	0	0
ENSG00000232516	0	0	0	0
ENSG00000232517	5	223	193	195
ENSG00000232518	0	0	0	0
ENSG00000232519	1	0	0	0
ENSG00000232520	5	0	0	2
ENSG00000232522	0	0	0	0
ENSG00000232523	0	0	0	0
ENSG00000232524	0	0	0	0
ENSG00000232525	0	0	0	0
ENSG00000232526	0	0	0	0
ENSG00000232527	2	0	0	0
ENSG00000232528	0	0	0	0
ENSG00000232529	0	0	0	0
ENSG00000232530	1	0	0	0
ENSG00000232531	0	0	0	0
ENSG00000232532	0	0	0	0
ENSG00000232533	164	110	227	507
ENSG00000232535	0	0	0	0
ENSG00000232536	0	0	0	0
ENSG00000232537	0	0	0	0
ENSG00000232539	0	0	0	0
ENSG00000232540	0	0	0	0
ENSG00000232541	0	0	0	0
ENSG00000232542	0	0	3	3
ENSG00000232543	0	0	0	0
ENSG00000232545	0	0	0	0
ENSG00000232546	0	5	31	32
ENSG00000232548	0	0	0	0
ENSG00000232549	0	0	0	0
ENSG00000232551	0	0	1	0
ENSG00000232553	0	0	0	0
ENSG00000232554	0	0	0	0
ENSG00000232555	0	0	4	1
ENSG00000232556	0	0	0	0
ENSG00000232557	0	0	0	0
ENSG00000232558	0	0	0	0
ENSG00000232559	6	225	83	81

ENSG00000232560	0	0	0	0
ENSG00000232561	1	58	57	15
ENSG00000232562	0	0	0	0
ENSG00000232563	0	0	0	0
ENSG00000232564	0	0	0	0
ENSG00000232565	0	0	0	0
ENSG00000232567	0	0	0	5
ENSG00000232568	0	0	0	1
ENSG00000232569	0	0	0	0
ENSG00000232571	0	0	0	0
ENSG00000232572	0	0	0	0
ENSG00000232573	14	0	6	7
ENSG00000232575	0	0	0	0
ENSG00000232576	0	0	0	0
ENSG00000232577	0	0	0	0
ENSG00000232578	0	0	0	0
ENSG00000232579	0	0	0	0
ENSG00000232581	0	3	32	17
ENSG00000232582	0	0	0	0
ENSG00000232583	0	0	0	0
ENSG00000232584	0	0	0	0
ENSG00000232585	0	0	0	0
ENSG00000232586	0	0	0	0
ENSG00000232587	0	0	2	4
ENSG00000232590	0	0	0	0
ENSG00000232591	0	0	0	0
ENSG00000232593	0	0	0	3
ENSG00000232594	0	0	0	0
ENSG00000232595	0	0	0	0
ENSG00000232596	0	0	0	0
ENSG00000232597	0	0	0	0
ENSG00000232598	0	0	0	0
ENSG00000232599	0	0	0	0
ENSG00000232600	0	0	1	2
ENSG00000232601	0	0	0	0
ENSG00000232603	0	0	0	0
ENSG00000232604	0	0	0	0
ENSG00000232605	0	0	0	0
ENSG00000232606	7	0	2	1
ENSG00000232608	0	0	0	0
ENSG00000232610	0	0	0	0
ENSG00000232611	0	0	2	0
ENSG00000232612	0	0	0	0
ENSG00000232613	2	0	0	0
ENSG00000232614	0	0	0	0
ENSG00000232615	0	0	0	0

ENSG00000232616	0	0	0	0
ENSG00000232617	0	0	0	0
ENSG00000232618	2	0	9	1
ENSG00000232620	0	0	0	0
ENSG00000232621	0	0	0	0
ENSG00000232622	0	0	0	0
ENSG00000232623	0	0	0	0
ENSG00000232624	6	0	3	0
ENSG00000232625	0	0	0	0
ENSG00000232626	0	0	0	0
ENSG00000232627	0	0	0	0
ENSG00000232628	1	0	2	0
ENSG00000232629	4	0	0	0
ENSG00000232630	0	0	3	0
ENSG00000232631	0	0	0	0
ENSG00000232632	0	0	0	0
ENSG00000232633	0	0	0	0
ENSG00000232634	0	0	0	0
ENSG00000232636	0	0	0	0
ENSG00000232637	3	0	0	0
ENSG00000232638	0	0	0	0
ENSG00000232639	0	0	0	0
ENSG00000232640	0	0	0	0
ENSG00000232641	0	0	0	0
ENSG00000232642	0	0	0	0
ENSG00000232643	0	0	0	0
ENSG00000232644	0	0	0	0
ENSG00000232645	1	0	1	0
ENSG00000232646	0	0	0	0
ENSG00000232647	0	0	0	0
ENSG00000232648	0	0	4	0
ENSG00000232649	0	0	0	0
ENSG00000232650	0	0	0	0
ENSG00000232652	0	0	0	0
ENSG00000232653	0	0	0	0
ENSG00000232654	0	0	0	0
ENSG00000232655	0	0	0	0
ENSG00000232656	0	0	0	0
ENSG00000232658	0	0	0	0
ENSG00000232659	0	0	0	0
ENSG00000232661	0	0	0	0
ENSG00000232662	0	0	0	0
ENSG00000232663	0	0	0	0
ENSG00000232664	0	0	0	0
ENSG00000232665	0	0	0	0
ENSG00000232667	0	0	0	0

ENSG00000232668	0	0	0	0
ENSG00000232671	2	0	1	0
ENSG00000232672	0	0	0	0
ENSG00000232675	0	0	5	9
ENSG00000232676	0	0	0	0
ENSG00000232677	1	0	2	0
ENSG00000232678	0	0	0	0
ENSG00000232679	0	0	1	3
ENSG00000232680	12	26	6	5
ENSG00000232682	0	0	5	0
ENSG00000232683	0	0	0	0
ENSG00000232684	0	0	0	0
ENSG00000232685	0	0	0	0
ENSG00000232686	0	0	0	0
ENSG00000232687	0	0	0	0
ENSG00000232688	0	0	0	0
ENSG00000232689	0	0	0	0
ENSG00000232690	0	0	0	0
ENSG00000232691	0	0	0	0
ENSG00000232692	0	38	5	10
ENSG00000232693	8	27	8	0
ENSG00000232694	0	0	0	0
ENSG00000232695	1	0	0	0
ENSG00000232696	0	0	0	0
ENSG00000232698	0	0	0	0
ENSG00000232699	0	0	0	0
ENSG00000232701	0	0	0	0
ENSG00000232702	0	0	0	0
ENSG00000232704	0	0	0	0
ENSG00000232705	0	0	0	0
ENSG00000232706	0	0	0	0
ENSG00000232707	0	0	0	0
ENSG00000232708	0	0	0	0
ENSG00000232709	0	0	0	0
ENSG00000232710	0	0	0	0
ENSG00000232712	0	0	0	0
ENSG00000232713	1	0	0	0
ENSG00000232714	0	0	0	0
ENSG00000232715	0	0	0	0
ENSG00000232716	0	0	0	0
ENSG00000232717	0	0	0	0
ENSG00000232718	0	0	0	0
ENSG00000232719	0	0	0	0
ENSG00000232721	0	0	0	0
ENSG00000232722	0	0	0	0
ENSG00000232723	0	0	0	0

ENSG00000232724	12	0	0	0
ENSG00000232725	22	50	5	43
ENSG00000232727	0	0	0	0
ENSG00000232728	0	0	0	0
ENSG00000232729	0	0	11	9
ENSG00000232730	0	0	0	0
ENSG00000232732	0	0	0	15
ENSG00000232733	0	0	0	0
ENSG00000232734	0	0	0	0
ENSG00000232735	0	0	0	0
ENSG00000232736	0	0	0	0
ENSG00000232738	0	0	0	0
ENSG00000232739	0	0	0	0
ENSG00000232740	0	0	0	0
ENSG00000232742	1	0	0	0
ENSG00000232743	0	0	0	0
ENSG00000232744	0	0	0	0
ENSG00000232745	0	5	8	30
ENSG00000232746	0	0	0	0
ENSG00000232747	0	0	0	0
ENSG00000232748	0	0	0	0
ENSG00000232749	0	0	0	0
ENSG00000232750	0	0	0	0
ENSG00000232751	0	0	0	0
ENSG00000232752	0	0	0	0
ENSG00000232753	0	0	0	0
ENSG00000232754	0	0	4	0
ENSG00000232756	0	0	0	0
ENSG00000232757	0	0	0	0
ENSG00000232758	0	0	0	0
ENSG00000232759	0	0	0	0
ENSG00000232760	0	0	0	0
ENSG00000232762	0	0	0	0
ENSG00000232764	0	0	0	0
ENSG00000232765	0	0	0	0
ENSG00000232766	0	0	0	0
ENSG00000232767	0	0	0	0
ENSG00000232768	0	0	0	0
ENSG00000232771	0	0	0	0
ENSG00000232772	0	0	0	0
ENSG00000232773	0	0	0	0
ENSG00000232774	3	67	73	49
ENSG00000232775	0	0	0	0
ENSG00000232777	0	0	0	0
ENSG00000232778	0	0	0	0
ENSG00000232780	0	0	0	0

ENSG00000232781	0	0	0	0
ENSG00000232783	1	0	0	39
ENSG00000232784	0	0	1	0
ENSG00000232786	0	0	0	0
ENSG00000232787	0	0	0	0
ENSG00000232788	590	1304	1003	1695
ENSG00000232789	0	0	0	0
ENSG00000232790	0	0	0	0
ENSG00000232791	0	0	0	0
ENSG00000232792	0	0	0	0
ENSG00000232793	0	0	0	0
ENSG00000232794	0	0	0	0
ENSG00000232795	0	0	0	0
ENSG00000232797	0	0	0	0
ENSG00000232798	0	0	0	0
ENSG00000232799	0	0	0	0
ENSG00000232800	0	0	0	0
ENSG00000232801	0	0	0	0
ENSG00000232802	0	0	0	0
ENSG00000232803	0	0	0	0
ENSG00000232804	0	0	0	0
ENSG00000232805	0	0	0	0
ENSG00000232806	0	0	0	0
ENSG00000232807	0	0	4	0
ENSG00000232808	0	0	0	0
ENSG00000232809	0	0	0	0
ENSG00000232810	1	0	0	0
ENSG00000232811	0	0	0	0
ENSG00000232812	0	0	0	0
ENSG00000232813	0	0	0	0
ENSG00000232814	0	0	2	0
ENSG00000232815	1	0	0	0
ENSG00000232816	0	0	0	0
ENSG00000232817	0	0	0	0
ENSG00000232818	0	0	0	0
ENSG00000232821	0	0	0	0
ENSG00000232823	0	0	0	0
ENSG00000232824	0	0	0	0
ENSG00000232825	0	0	0	0
ENSG00000232827	0	0	0	0
ENSG00000232828	0	28	2	7
ENSG00000232829	0	0	0	0
ENSG00000232830	0	0	0	0
ENSG00000232832	0	17	15	84
ENSG00000232833	0	0	0	0
ENSG00000232834	0	0	0	0

ENSG00000232835	0	0	0	0
ENSG00000232837	0	0	0	0
ENSG00000232838	0	0	0	0
ENSG00000232839	0	0	0	0
ENSG00000232841	0	0	0	0
ENSG00000232842	0	0	0	0
ENSG00000232843	0	0	0	0
ENSG00000232845	0	0	0	0
ENSG00000232846	0	0	0	0
ENSG00000232848	0	0	0	0
ENSG00000232849	0	0	0	0
ENSG00000232850	0	0	0	0
ENSG00000232851	0	0	0	0
ENSG00000232852	0	0	0	0
ENSG00000232853	0	0	0	0
ENSG00000232855	0	0	0	6
ENSG00000232857	0	0	0	0
ENSG00000232858	0	0	0	0
ENSG00000232859	46	22	40	41
ENSG00000232860	3	0	1	0
ENSG00000232862	0	0	0	0
ENSG00000232863	0	0	0	0
ENSG00000232864	0	0	0	0
ENSG00000232866	0	0	0	0
ENSG00000232867	0	0	0	0
ENSG00000232868	0	0	0	0
ENSG00000232869	2	0	0	0
ENSG00000232871	0	0	0	0
ENSG00000232872	0	0	0	0
ENSG00000232873	0	0	0	0
ENSG00000232874	0	0	0	0
ENSG00000232875	0	0	0	0
ENSG00000232876	0	33	0	0
ENSG00000232878	0	0	26	2
ENSG00000232879	0	0	0	0
ENSG00000232880	0	0	0	0
ENSG00000232881	0	0	0	0
ENSG00000232882	0	0	0	0
ENSG00000232883	0	0	0	0
ENSG00000232884	0	0	0	0
ENSG00000232885	0	0	0	0
ENSG00000232886	0	0	0	0
ENSG00000232887	0	0	0	0
ENSG00000232888	0	0	0	0
ENSG00000232889	0	0	0	0
ENSG00000232891	0	0	0	0

ENSG00000232892	0	0	0	0
ENSG00000232893	0	0	0	0
ENSG00000232894	0	0	2	2
ENSG00000232895	0	0	0	0
ENSG00000232896	0	0	0	0
ENSG00000232897	0	0	0	0
ENSG00000232899	0	0	0	0
ENSG00000232900	0	0	0	0
ENSG00000232901	0	1	0	9
ENSG00000232902	0	0	0	0
ENSG00000232903	0	0	0	0
ENSG00000232905	0	0	0	0
ENSG00000232906	0	0	0	0
ENSG00000232907	0	0	2	0
ENSG00000232908	3	0	0	0
ENSG00000232909	0	0	0	3
ENSG00000232910	0	0	0	0
ENSG00000232912	0	0	1	0
ENSG00000232913	0	0	0	0
ENSG00000232914	0	0	0	0
ENSG00000232915	0	0	0	0
ENSG00000232916	0	0	0	0
ENSG00000232917	0	0	0	0
ENSG00000232918	0	0	0	0
ENSG00000232920	0	0	0	0
ENSG00000232922	0	0	0	0
ENSG00000232924	0	0	0	0
ENSG00000232925	0	0	0	0
ENSG00000232926	0	0	0	0
ENSG00000232927	0	0	0	0
ENSG00000232928	0	0	0	0
ENSG00000232930	0	0	0	0
ENSG00000232931	0	12	9	6
ENSG00000232934	0	0	0	9
ENSG00000232935	0	0	0	0
ENSG00000232936	0	0	2	0
ENSG00000232937	0	0	0	0
ENSG00000232938	6	1	2	8
ENSG00000232939	0	0	0	0
ENSG00000232940	0	0	0	0
ENSG00000232941	0	215	472	67
ENSG00000232943	0	0	0	0
ENSG00000232944	0	0	0	0
ENSG00000232945	0	0	0	0
ENSG00000232946	0	0	0	0
ENSG00000232947	0	0	0	0

ENSG00000232948	0	0	0	0
ENSG00000232949	0	0	0	0
ENSG00000232950	0	0	0	0
ENSG00000232951	0	0	0	0
ENSG00000232952	0	0	0	0
ENSG00000232953	0	0	0	0
ENSG00000232954	0	0	0	0
ENSG00000232956	10	0	3	10
ENSG00000232957	0	0	0	0
ENSG00000232958	0	0	0	2
ENSG00000232959	0	115	66	42
ENSG00000232960	0	0	0	0
ENSG00000232962	0	0	0	0
ENSG00000232963	0	0	0	0
ENSG00000232964	0	0	0	0
ENSG00000232965	0	0	0	0
ENSG00000232968	0	0	0	0
ENSG00000232969	0	0	0	0
ENSG00000232970	0	0	0	0
ENSG00000232971	0	0	0	0
ENSG00000232973	1	0	6	10
ENSG00000232974	0	0	0	0
ENSG00000232975	0	0	0	0
ENSG00000232976	0	0	0	0
ENSG00000232977	0	0	0	0
ENSG00000232978	0	0	0	0
ENSG00000232979	0	0	0	0
ENSG00000232981	0	0	0	0
ENSG00000232982	0	0	0	0
ENSG00000232983	0	0	0	0
ENSG00000232984	0	0	0	0
ENSG00000232985	0	0	0	0
ENSG00000232986	0	0	0	0
ENSG00000232987	0	0	0	0
ENSG00000232988	0	0	0	0
ENSG00000232989	0	0	0	0
ENSG00000232990	0	0	0	0
ENSG00000232991	0	0	0	0
ENSG00000232992	0	0	0	0
ENSG00000232993	0	0	0	0
ENSG00000232994	0	0	0	0
ENSG00000232995	0	15	0	8
ENSG00000232997	0	0	0	0
ENSG00000232998	0	0	0	0
ENSG00000232999	0	0	0	0
ENSG00000233002	0	0	0	0

ENSG00000233003	0	0	0	0
ENSG00000233005	0	0	2	0
ENSG00000233006	0	0	0	0
ENSG00000233007	0	0	0	0
ENSG00000233008	1	0	0	0
ENSG00000233009	0	0	0	0
ENSG00000233010	0	0	0	0
ENSG00000233011	0	0	0	0
ENSG00000233012	0	0	0	0
ENSG00000233013	0	0	4	10
ENSG00000233014	0	0	0	0
ENSG00000233015	0	0	0	0
ENSG00000233016	74	383	104	97
ENSG00000233017	0	0	0	0
ENSG00000233018	0	0	0	0
ENSG00000233019	0	0	0	0
ENSG00000233020	0	0	0	0
ENSG00000233021	0	0	0	0
ENSG00000233022	0	0	0	0
ENSG00000233023	0	0	0	0
ENSG00000233024	0	0	0	0
ENSG00000233025	0	0	0	0
ENSG00000233026	0	0	0	0
ENSG00000233028	0	0	0	0
ENSG00000233029	0	0	0	0
ENSG00000233030	1	3	6	1
ENSG00000233031	0	0	0	0
ENSG00000233033	0	0	0	0
ENSG00000233035	0	0	0	0
ENSG00000233036	0	0	0	0
ENSG00000233037	0	0	0	0
ENSG00000233038	3	0	0	0
ENSG00000233039	0	0	0	0
ENSG00000233040	0	0	0	0
ENSG00000233041	0	0	0	0
ENSG00000233043	0	0	0	0
ENSG00000233044	0	0	0	0
ENSG00000233045	0	0	0	0
ENSG00000233046	0	0	0	0
ENSG00000233047	0	0	2	0
ENSG00000233048	0	0	0	0
ENSG00000233049	0	0	0	0
ENSG00000233050	0	0	0	0
ENSG00000233051	0	0	0	0
ENSG00000233052	0	0	0	0
ENSG00000233053	0	0	0	0

ENSG00000233054	0	0	0	0
ENSG00000233055	0	0	0	0
ENSG00000233056	0	0	0	0
ENSG00000233057	0	0	0	0
ENSG00000233058	0	0	0	0
ENSG00000233059	0	0	0	0
ENSG00000233060	0	0	0	0
ENSG00000233061	0	0	1	0
ENSG00000233063	0	0	0	0
ENSG00000233064	1	0	1	0
ENSG00000233067	0	0	0	0
ENSG00000233068	0	0	0	0
ENSG00000233069	0	0	0	0
ENSG00000233070	0	0	0	0
ENSG00000233071	0	0	0	0
ENSG00000233072	1	0	0	0
ENSG00000233073	0	0	0	0
ENSG00000233074	0	0	0	0
ENSG00000233075	0	0	0	0
ENSG00000233076	0	0	0	0
ENSG00000233077	0	0	0	0
ENSG00000233078	0	0	0	0
ENSG00000233079	0	0	0	0
ENSG00000233080	0	0	0	0
ENSG00000233081	0	0	0	0
ENSG00000233082	0	0	0	0
ENSG00000233083	0	0	0	0
ENSG00000233084	0	0	1	24
ENSG00000233085	0	0	0	0
ENSG00000233086	1	0	0	0
ENSG00000233087	0	0	6	1
ENSG00000233089	0	0	0	0
ENSG00000233090	0	0	0	0
ENSG00000233091	0	0	0	0
ENSG00000233093	678	1063	839	1558
ENSG00000233094	0	0	0	0
ENSG00000233095	0	0	0	0
ENSG00000233096	0	0	0	0
ENSG00000233098	0	33	10	1
ENSG00000233099	69	0	12	14
ENSG00000233101	0	0	0	0
ENSG00000233103	0	0	0	0
ENSG00000233105	0	0	0	0
ENSG00000233106	0	0	0	0
ENSG00000233107	0	0	0	0
ENSG00000233108	1	0	2	0

ENSG00000233109	0	0	0	0
ENSG00000233110	0	0	1	0
ENSG00000233111	0	0	0	0
ENSG00000233113	0	0	0	0
ENSG00000233114	0	0	0	0
ENSG00000233115	0	0	0	0
ENSG00000233117	0	0	1	1
ENSG00000233118	0	0	0	0
ENSG00000233119	0	0	0	0
ENSG00000233120	0	0	0	0
ENSG00000233121	0	0	0	0
ENSG00000233122	0	0	0	0
ENSG00000233123	0	0	0	0
ENSG00000233124	0	0	3	16
ENSG00000233125	0	0	0	0
ENSG00000233126	0	0	0	0
ENSG00000233128	0	0	0	0
ENSG00000233129	0	0	0	0
ENSG00000233131	0	0	0	0
ENSG00000233132	0	0	0	0
ENSG00000233133	0	0	0	0
ENSG00000233134	0	0	0	0
ENSG00000233135	0	0	0	0
ENSG00000233136	0	0	0	0
ENSG00000233137	22	0	31	23
ENSG00000233138	0	0	0	0
ENSG00000233139	0	0	0	0
ENSG00000233140	0	0	0	0
ENSG00000233141	0	0	0	0
ENSG00000233143	0	0	0	1
ENSG00000233144	0	18	64	52
ENSG00000233145	0	0	0	0
ENSG00000233146	0	0	0	0
ENSG00000233147	533	445	120	703
ENSG00000233148	0	0	0	0
ENSG00000233149	0	0	0	0
ENSG00000233151	0	0	0	0
ENSG00000233153	0	0	0	0
ENSG00000233154	0	0	0	10
ENSG00000233155	0	0	0	0
ENSG00000233156	0	0	0	0
ENSG00000233157	0	0	0	0
ENSG00000233158	0	0	0	0
ENSG00000233159	0	0	0	0
ENSG00000233160	0	0	0	0
ENSG00000233161	0	0	0	0

ENSG00000233162	0	0	0	0
ENSG00000233163	0	0	0	0
ENSG00000233164	0	0	0	0
ENSG00000233165	0	0	0	0
ENSG00000233167	0	0	0	0
ENSG00000233168	0	0	0	0
ENSG00000233169	0	0	0	0
ENSG00000233170	0	0	0	0
ENSG00000233171	0	0	0	0
ENSG00000233172	0	0	0	0
ENSG00000233173	0	0	0	0
ENSG00000233175	0	0	0	0
ENSG00000233176	0	0	0	0
ENSG00000233178	3	34	10	34
ENSG00000233179	0	0	0	0
ENSG00000233180	0	0	0	0
ENSG00000233182	0	0	0	0
ENSG00000233183	0	0	1	3
ENSG00000233184	0	0	11	26
ENSG00000233186	0	0	0	0
ENSG00000233189	0	0	0	0
ENSG00000233190	0	0	0	0
ENSG00000233191	0	0	0	0
ENSG00000233192	0	0	0	0
ENSG00000233193	0	0	0	0
ENSG00000233194	0	0	0	0
ENSG00000233196	0	0	0	0
ENSG00000233197	0	0	0	0
ENSG00000233198	0	0	0	0
ENSG00000233200	0	0	0	0
ENSG00000233202	0	0	0	0
ENSG00000233203	0	0	0	0
ENSG00000233204	0	0	0	0
ENSG00000233205	0	0	0	0
ENSG00000233206	0	0	0	0
ENSG00000233207	0	0	0	0
ENSG00000233208	29	3	41	32
ENSG00000233209	0	0	0	0
ENSG00000233210	0	0	0	0
ENSG00000233211	0	0	0	0
ENSG00000233213	0	0	0	0
ENSG00000233214	17	0	0	3
ENSG00000233215	0	0	0	0
ENSG00000233216	0	0	0	0
ENSG00000233217	0	0	0	0
ENSG00000233218	0	0	0	0

ENSG00000233219	0	0	0	0
ENSG00000233220	0	0	0	0
ENSG00000233221	0	0	0	0
ENSG00000233222	0	0	0	0
ENSG00000233223	0	20	15	14
ENSG00000233224	49	0	4	3
ENSG00000233225	0	0	0	0
ENSG00000233228	0	0	0	0
ENSG00000233229	0	0	0	0
ENSG00000233230	4	23	10	6
ENSG00000233231	0	0	0	0
ENSG00000233232	0	0	0	0
ENSG00000233234	0	0	0	0
ENSG00000233235	0	0	0	0
ENSG00000233236	0	0	0	0
ENSG00000233237	0	0	0	0
ENSG00000233238	0	0	0	0
ENSG00000233239	0	0	0	0
ENSG00000233240	0	0	0	0
ENSG00000233241	0	0	0	0
ENSG00000233242	0	0	0	0
ENSG00000233243	0	0	0	0
ENSG00000233244	0	0	0	0
ENSG00000233246	0	0	0	0
ENSG00000233247	0	1	1	1
ENSG00000233248	0	0	0	0
ENSG00000233250	0	0	0	0
ENSG00000233251	55	2	40	30
ENSG00000233252	0	0	0	0
ENSG00000233254	1	0	0	4
ENSG00000233255	0	0	1	4
ENSG00000233256	1	14	8	11
ENSG00000233258	0	0	0	0
ENSG00000233259	0	0	0	0
ENSG00000233260	0	0	0	0
ENSG00000233261	0	0	0	0
ENSG00000233262	0	0	0	0
ENSG00000233263	0	0	0	0
ENSG00000233264	0	0	1	0
ENSG00000233265	0	0	0	0
ENSG00000233266	8	0	8	5
ENSG00000233268	0	0	0	0
ENSG00000233270	1	0	0	0
ENSG00000233271	0	0	0	0
ENSG00000233272	0	0	0	0
ENSG00000233273	0	0	0	0

ENSG00000233275	0	0	0	0
ENSG00000233276	31121	22760	10757	31619
ENSG00000233277	0	0	0	0
ENSG00000233278	0	0	0	0
ENSG00000233280	3	0	10	24
ENSG00000233283	0	0	0	0
ENSG00000233285	0	0	0	0
ENSG00000233286	0	0	0	0
ENSG00000233287	0	0	0	0
ENSG00000233288	0	0	0	0
ENSG00000233290	0	0	0	0
ENSG00000233291	0	0	0	0
ENSG00000233292	0	0	0	0
ENSG00000233293	0	0	0	0
ENSG00000233295	0	0	0	0
ENSG00000233296	3	0	2	0
ENSG00000233297	0	0	0	0
ENSG00000233298	0	0	0	0
ENSG00000233299	0	0	0	0
ENSG00000233300	0	0	0	0
ENSG00000233301	0	0	0	0
ENSG00000233303	0	0	0	0
ENSG00000233304	0	0	0	1
ENSG00000233306	0	0	0	0
ENSG00000233307	0	0	0	0
ENSG00000233308	0	0	0	0
ENSG00000233309	0	0	0	0
ENSG00000233311	0	0	0	0
ENSG00000233312	0	0	0	0
ENSG00000233313	0	0	0	0
ENSG00000233314	0	0	0	0
ENSG00000233315	0	0	0	0
ENSG00000233316	0	0	0	0
ENSG00000233317	0	0	0	0
ENSG00000233318	0	0	0	0
ENSG00000233319	0	0	0	0
ENSG00000233320	0	0	0	0
ENSG00000233321	1	0	0	0
ENSG00000233323	0	0	0	0
ENSG00000233324	0	0	0	0
ENSG00000233325	0	0	0	0
ENSG00000233327	0	0	2	5
ENSG00000233328	0	0	0	0
ENSG00000233329	0	0	0	0
ENSG00000233330	1	0	0	0
ENSG00000233332	0	0	0	0

ENSG00000233333	0	0	0	0
ENSG00000233334	0	0	0	0
ENSG00000233335	0	0	0	0
ENSG00000233336	0	0	0	0
ENSG00000233337	0	0	0	0
ENSG00000233338	0	0	1	1
ENSG00000233339	0	0	0	0
ENSG00000233340	0	0	0	0
ENSG00000233342	0	0	0	0
ENSG00000233343	0	0	0	0
ENSG00000233345	0	0	0	0
ENSG00000233347	0	0	0	0
ENSG00000233348	0	0	0	0
ENSG00000233349	0	0	0	0
ENSG00000233350	0	0	0	0
ENSG00000233351	0	0	10	0
ENSG00000233352	0	0	0	0
ENSG00000233353	0	0	0	0
ENSG00000233354	2	0	0	0
ENSG00000233355	3	9	8	7
ENSG00000233357	0	0	0	0
ENSG00000233358	0	0	0	0
ENSG00000233359	0	0	0	0
ENSG00000233360	0	0	0	0
ENSG00000233363	0	0	0	0
ENSG00000233364	0	0	0	0
ENSG00000233365	0	0	0	0
ENSG00000233366	0	0	0	0
ENSG00000233367	0	0	0	0
ENSG00000233368	0	0	13	4
ENSG00000233369	4	178	94	49
ENSG00000233370	0	0	0	0
ENSG00000233372	0	0	0	0
ENSG00000233373	0	0	0	0
ENSG00000233376	0	0	0	0
ENSG00000233377	0	0	0	0
ENSG00000233378	0	0	0	0
ENSG00000233379	0	0	0	0
ENSG00000233380	0	0	0	0
ENSG00000233381	0	0	0	0
ENSG00000233382	5	8	9	41
ENSG00000233383	0	0	0	0
ENSG00000233384	0	0	0	0
ENSG00000233385	0	0	0	0
ENSG00000233387	0	0	0	0
ENSG00000233388	0	0	0	0

ENSG00000233392	0	0	0	0
ENSG00000233393	0	0	0	0
ENSG00000233394	0	0	0	0
ENSG00000233395	0	0	0	0
ENSG00000233396	0	0	0	0
ENSG00000233397	1	0	0	0
ENSG00000233399	0	0	0	0
ENSG00000233401	0	0	0	0
ENSG00000233402	0	0	0	0
ENSG00000233403	0	0	0	0
ENSG00000233404	58	166	216	84
ENSG00000233405	0	0	0	0
ENSG00000233406	0	0	2	0
ENSG00000233407	0	0	0	0
ENSG00000233408	0	0	0	0
ENSG00000233409	0	0	0	0
ENSG00000233410	0	0	0	0
ENSG00000233411	1	0	0	0
ENSG00000233412	0	0	0	0
ENSG00000233414	0	0	0	0
ENSG00000233415	0	0	0	0
ENSG00000233416	0	0	0	0
ENSG00000233417	0	0	0	0
ENSG00000233418	0	0	0	0
ENSG00000233419	0	0	0	0
ENSG00000233420	0	13	7	14
ENSG00000233421	0	0	0	0
ENSG00000233423	0	0	0	0
ENSG00000233424	0	0	0	0
ENSG00000233425	0	0	0	0
ENSG00000233426	0	0	3	0
ENSG00000233427	8	51	41	23
ENSG00000233428	0	0	0	0
ENSG00000233429	60	54	19	37
ENSG00000233430	0	0	0	0
ENSG00000233431	0	0	0	0
ENSG00000233432	0	0	0	0
ENSG00000233433	0	0	0	0
ENSG00000233434	0	0	0	0
ENSG00000233435	4	28	20	129
ENSG00000233436	0	0	0	0
ENSG00000233437	0	0	0	0
ENSG00000233438	0	0	1	0
ENSG00000233439	0	0	0	0
ENSG00000233440	0	0	0	0
ENSG00000233441	0	0	0	0

ENSG00000233442	0	0	0	0
ENSG00000233444	0	0	0	0
ENSG00000233445	0	0	0	0
ENSG00000233446	0	0	0	0
ENSG00000233447	0	0	0	0
ENSG00000233448	0	0	0	0
ENSG00000233449	0	0	0	0
ENSG00000233450	0	0	0	0
ENSG00000233451	0	0	0	0
ENSG00000233452	457	2521	2389	2303
ENSG00000233454	0	0	0	0
ENSG00000233455	0	0	0	0
ENSG00000233456	0	0	0	0
ENSG00000233457	0	0	0	0
ENSG00000233459	0	0	0	0
ENSG00000233460	0	0	0	0
ENSG00000233461	13	0	1	2
ENSG00000233462	0	0	0	0
ENSG00000233464	0	0	0	0
ENSG00000233465	0	0	0	0
ENSG00000233466	0	0	0	0
ENSG00000233467	0	0	0	0
ENSG00000233469	0	0	0	0
ENSG00000233470	0	0	0	0
ENSG00000233471	0	0	0	0
ENSG00000233472	0	0	0	0
ENSG00000233473	0	0	0	0
ENSG00000233476	0	3	9	8
ENSG00000233477	0	0	0	0
ENSG00000233478	0	0	0	0
ENSG00000233479	0	0	0	2
ENSG00000233480	0	0	0	0
ENSG00000233481	0	0	0	0
ENSG00000233482	0	0	0	0
ENSG00000233483	0	0	0	0
ENSG00000233484	0	0	0	0
ENSG00000233485	0	0	0	1
ENSG00000233486	0	0	0	0
ENSG00000233487	0	0	0	0
ENSG00000233488	24	5	17	21
ENSG00000233489	0	0	0	0
ENSG00000233490	0	0	0	0
ENSG00000233491	0	0	0	0
ENSG00000233492	0	0	0	0
ENSG00000233493	0	0	0	1
ENSG00000233494	0	0	0	0

ENSG00000233496	0	0	0	0
ENSG00000233497	0	0	0	0
ENSG00000233498	0	0	0	0
ENSG00000233499	0	0	0	0
ENSG00000233501	0	0	0	0
ENSG00000233502	0	0	0	0
ENSG00000233503	0	0	1	0
ENSG00000233504	0	0	0	0
ENSG00000233505	0	0	0	0
ENSG00000233506	0	0	0	0
ENSG00000233508	0	0	0	0
ENSG00000233509	0	0	0	0
ENSG00000233511	0	0	0	0
ENSG00000233513	0	0	0	0
ENSG00000233514	0	0	0	0
ENSG00000233515	0	0	0	2
ENSG00000233516	0	0	0	0
ENSG00000233517	0	0	0	0
ENSG00000233518	0	0	0	0
ENSG00000233519	0	5	0	0
ENSG00000233521	0	0	0	0
ENSG00000233522	0	0	0	0
ENSG00000233523	0	0	0	0
ENSG00000233524	0	0	0	0
ENSG00000233526	0	0	0	0
ENSG00000233527	218	196	206	279
ENSG00000233528	0	0	0	0
ENSG00000233529	0	0	0	0
ENSG00000233531	0	0	0	0
ENSG00000233532	0	0	0	0
ENSG00000233533	0	0	0	0
ENSG00000233534	0	0	0	0
ENSG00000233535	0	0	0	0
ENSG00000233536	0	0	0	0
ENSG00000233537	0	0	0	0
ENSG00000233538	0	0	0	0
ENSG00000233539	0	0	0	0
ENSG00000233540	0	108	38	32
ENSG00000233541	0	0	0	0
ENSG00000233542	1	0	1	0
ENSG00000233543	0	0	0	0
ENSG00000233544	0	0	0	0
ENSG00000233545	0	0	0	0
ENSG00000233546	0	0	0	0
ENSG00000233547	0	0	0	0
ENSG00000233548	0	0	0	0

ENSG00000233549	0	0	0	0
ENSG00000233550	0	0	0	0
ENSG00000233551	0	0	0	0
ENSG00000233553	0	0	0	0
ENSG00000233554	133	306	230	372
ENSG00000233555	0	0	0	0
ENSG00000233557	0	0	0	0
ENSG00000233558	0	0	2	0
ENSG00000233559	0	0	0	0
ENSG00000233560	0	0	0	0
ENSG00000233561	0	0	0	0
ENSG00000233562	0	0	0	0
ENSG00000233563	0	0	0	0
ENSG00000233564	0	0	0	0
ENSG00000233565	0	0	0	0
ENSG00000233566	0	0	0	0
ENSG00000233569	0	0	0	1
ENSG00000233570	0	0	0	0
ENSG00000233571	0	0	0	0
ENSG00000233573	0	0	0	0
ENSG00000233574	0	0	0	0
ENSG00000233575	0	0	0	0
ENSG00000233576	0	0	0	0
ENSG00000233577	0	0	0	0
ENSG00000233578	0	0	0	0
ENSG00000233579	0	0	0	0
ENSG00000233581	0	0	0	0
ENSG00000233583	0	0	0	0
ENSG00000233584	0	0	0	0
ENSG00000233585	6	0	22	16
ENSG00000233586	0	0	0	0
ENSG00000233587	0	0	0	0
ENSG00000233588	0	0	0	0
ENSG00000233589	0	24	8	0
ENSG00000233590	0	0	0	0
ENSG00000233592	0	0	0	0
ENSG00000233593	0	0	0	0
ENSG00000233594	0	0	0	0
ENSG00000233595	0	0	0	0
ENSG00000233597	0	0	0	0
ENSG00000233599	0	0	0	0
ENSG00000233601	0	0	0	0
ENSG00000233602	0	0	0	0
ENSG00000233603	0	0	0	0
ENSG00000233604	0	0	0	0
ENSG00000233605	0	0	0	0

ENSG00000233606	0	0	0	0
ENSG00000233607	0	0	0	0
ENSG00000233608	0	0	0	0
ENSG00000233609	0	0	0	0
ENSG00000233610	0	0	0	0
ENSG00000233611	0	0	0	0
ENSG00000233613	0	0	0	0
ENSG00000233614	2093	15552	3420	5284
ENSG00000233615	0	0	0	0
ENSG00000233616	0	0	0	0
ENSG00000233617	0	0	0	0
ENSG00000233618	0	0	0	0
ENSG00000233619	0	0	0	0
ENSG00000233620	0	0	0	0
ENSG00000233621	163	304	192	194
ENSG00000233622	28	0	8	17
ENSG00000233623	0	0	0	0
ENSG00000233625	0	0	0	0
ENSG00000233626	0	0	0	0
ENSG00000233627	0	0	0	0
ENSG00000233628	0	0	0	0
ENSG00000233630	0	0	0	0
ENSG00000233631	0	0	0	0
ENSG00000233632	0	0	0	0
ENSG00000233633	0	0	0	0
ENSG00000233634	0	0	0	0
ENSG00000233635	0	0	5	0
ENSG00000233636	0	0	0	0
ENSG00000233639	0	0	0	0
ENSG00000233640	0	0	0	0
ENSG00000233641	0	0	0	0
ENSG00000233642	0	0	0	0
ENSG00000233643	0	0	0	0
ENSG00000233644	0	0	0	0
ENSG00000233645	0	0	0	0
ENSG00000233646	0	0	0	0
ENSG00000233647	0	0	0	0
ENSG00000233648	0	0	0	0
ENSG00000233649	0	0	0	0
ENSG00000233650	0	0	0	0
ENSG00000233651	0	0	0	0
ENSG00000233652	0	0	0	0
ENSG00000233653	0	0	0	0
ENSG00000233654	2	0	20	37
ENSG00000233655	0	0	0	0
ENSG00000233656	0	0	0	0

ENSG00000233659	0	0	0	0
ENSG00000233661	0	0	2	0
ENSG00000233662	0	0	0	0
ENSG00000233663	0	0	0	0
ENSG00000233664	0	0	0	0
ENSG00000233665	0	0	0	0
ENSG00000233668	0	0	0	0
ENSG00000233670	0	0	0	0
ENSG00000233671	0	0	0	3
ENSG00000233672	0	13	12	42
ENSG00000233673	0	0	3	0
ENSG00000233674	0	0	0	0
ENSG00000233676	0	0	0	0
ENSG00000233677	0	0	0	0
ENSG00000233680	0	0	0	0
ENSG00000233681	0	0	0	3
ENSG00000233682	0	0	0	0
ENSG00000233683	0	0	0	0
ENSG00000233684	8	16	13	10
ENSG00000233685	0	0	0	0
ENSG00000233686	0	0	0	0
ENSG00000233687	0	0	0	0
ENSG00000233688	0	0	0	0
ENSG00000233689	0	0	0	0
ENSG00000233690	0	0	0	0
ENSG00000233691	0	0	0	0
ENSG00000233692	0	0	0	0
ENSG00000233693	1	0	0	0
ENSG00000233694	0	0	0	0
ENSG00000233695	0	0	0	0
ENSG00000233697	0	0	0	0
ENSG00000233699	0	0	0	0
ENSG00000233700	0	0	0	0
ENSG00000233701	0	0	0	0
ENSG00000233702	1	0	3	13
ENSG00000233703	0	0	0	0
ENSG00000233705	0	0	3	0
ENSG00000233706	0	14	6	0
ENSG00000233707	0	0	0	0
ENSG00000233708	0	0	0	0
ENSG00000233709	0	0	0	0
ENSG00000233710	1	0	0	0
ENSG00000233711	0	0	0	0
ENSG00000233712	1	0	0	0
ENSG00000233713	0	0	0	0
ENSG00000233714	0	0	0	0

ENSG00000233715	0	0	0	0
ENSG00000233716	0	0	1	0
ENSG00000233717	0	0	0	0
ENSG00000233718	81	84	29	76
ENSG00000233719	0	0	0	0
ENSG00000233720	0	0	0	0
ENSG00000233721	0	0	0	1
ENSG00000233723	2	0	2	0
ENSG00000233724	0	0	0	0
ENSG00000233725	0	0	0	1
ENSG00000233727	0	0	0	0
ENSG00000233728	0	0	0	0
ENSG00000233729	0	0	0	0
ENSG00000233730	7	42	5	14
ENSG00000233731	0	0	0	0
ENSG00000233732	0	0	0	0
ENSG00000233733	0	0	0	0
ENSG00000233734	0	0	0	0
ENSG00000233735	0	0	0	0
ENSG00000233737	0	0	0	0
ENSG00000233739	0	0	0	0
ENSG00000233740	0	0	0	0
ENSG00000233741	0	0	0	0
ENSG00000233742	0	0	0	0
ENSG00000233744	0	0	0	0
ENSG00000233745	0	0	0	0
ENSG00000233746	0	0	0	0
ENSG00000233747	1	0	1	3
ENSG00000233750	3	0	4	0
ENSG00000233751	0	0	0	0
ENSG00000233752	0	0	0	0
ENSG00000233754	0	0	0	0
ENSG00000233755	1	0	0	5
ENSG00000233756	0	0	0	0
ENSG00000233757	0	14	1	0
ENSG00000233760	0	0	0	0
ENSG00000233762	8	0	2	0
ENSG00000233764	0	0	0	0
ENSG00000233765	0	0	0	0
ENSG00000233766	14	981	1002	643
ENSG00000233767	0	0	0	0
ENSG00000233771	0	0	0	0
ENSG00000233772	0	0	0	0
ENSG00000233774	0	0	0	0
ENSG00000233775	0	0	0	0
ENSG00000233776	0	0	0	0

ENSG00000233778	0	0	0	0
ENSG00000233780	1	0	0	0
ENSG00000233782	0	0	0	0
ENSG00000233783	0	0	0	0
ENSG00000233785	4	61	7	15
ENSG00000233786	0	0	0	0
ENSG00000233787	0	0	1	0
ENSG00000233788	0	0	0	0
ENSG00000233791	0	0	3	2
ENSG00000233792	0	0	0	0
ENSG00000233795	0	0	0	0
ENSG00000233796	0	0	0	0
ENSG00000233797	1	0	11	9
ENSG00000233798	0	0	0	0
ENSG00000233799	2	0	4	0
ENSG00000233800	0	0	1	2
ENSG00000233801	0	0	0	0
ENSG00000233802	0	0	0	0
ENSG00000233803	0	0	0	0
ENSG00000233805	0	0	0	0
ENSG00000233806	0	3	3	0
ENSG00000233807	0	0	0	0
ENSG00000233810	0	0	0	0
ENSG00000233812	0	0	0	0
ENSG00000233813	0	0	0	0
ENSG00000233814	0	0	0	0
ENSG00000233816	0	0	0	0
ENSG00000233817	0	0	0	0
ENSG00000233818	0	0	0	0
ENSG00000233819	0	0	0	0
ENSG00000233820	0	0	0	0
ENSG00000233821	0	0	0	0
ENSG00000233822	929	2396	1347	1175
ENSG00000233823	0	0	0	0
ENSG00000233824	0	0	0	0
ENSG00000233825	0	0	0	0
ENSG00000233826	0	0	0	0
ENSG00000233827	0	0	0	0
ENSG00000233828	0	0	0	0
ENSG00000233829	0	0	0	0
ENSG00000233830	0	0	0	0
ENSG00000233831	0	0	0	0
ENSG00000233832	0	0	0	0
ENSG00000233833	0	0	0	0
ENSG00000233834	17	61	38	27
ENSG00000233835	0	0	0	0

ENSG00000233836	0	709	655	892
ENSG00000233837	0	0	0	0
ENSG00000233838	0	0	0	0
ENSG00000233839	0	0	0	0
ENSG00000233840	0	0	0	0
ENSG00000233841	0	0	0	0
ENSG00000233842	0	0	0	0
ENSG00000233843	0	0	0	0
ENSG00000233844	0	30	29	17
ENSG00000233845	0	0	0	0
ENSG00000233846	0	0	0	0
ENSG00000233847	0	0	0	0
ENSG00000233848	0	0	0	1
ENSG00000233849	0	0	0	0
ENSG00000233850	0	0	0	0
ENSG00000233851	0	0	0	0
ENSG00000233852	0	0	0	2
ENSG00000233853	0	0	0	0
ENSG00000233854	0	0	0	0
ENSG00000233857	0	0	0	0
ENSG00000233858	0	0	0	0
ENSG00000233859	0	1	0	0
ENSG00000233860	0	0	0	0
ENSG00000233861	0	0	0	0
ENSG00000233862	12	29	2	10
ENSG00000233863	0	0	0	0
ENSG00000233864	3	0	0	34
ENSG00000233866	0	0	0	0
ENSG00000233867	0	0	0	0
ENSG00000233868	0	0	0	0
ENSG00000233869	0	0	0	0
ENSG00000233870	0	0	0	0
ENSG00000233871	0	0	0	0
ENSG00000233872	0	0	0	0
ENSG00000233873	0	0	0	0
ENSG00000233874	0	0	0	0
ENSG00000233875	0	0	0	0
ENSG00000233876	0	0	0	0
ENSG00000233877	0	0	0	0
ENSG00000233878	5	0	0	0
ENSG00000233880	0	0	0	0
ENSG00000233882	0	0	0	0
ENSG00000233883	0	0	0	0
ENSG00000233884	0	0	0	0
ENSG00000233885	0	0	0	1
ENSG00000233887	0	0	0	0

ENSG00000233888	0	0	0	0
ENSG00000233889	0	0	0	0
ENSG00000233890	0	0	0	0
ENSG00000233891	1	0	0	0
ENSG00000233892	0	0	0	0
ENSG00000233893	0	0	0	0
ENSG00000233894	0	0	0	0
ENSG00000233895	2	100	28	32
ENSG00000233896	0	0	0	0
ENSG00000233899	1	0	0	0
ENSG00000233900	0	0	0	0
ENSG00000233901	10	0	0	3
ENSG00000233902	0	0	0	0
ENSG00000233903	13	0	7	14
ENSG00000233904	0	0	0	0
ENSG00000233905	0	0	0	0
ENSG00000233906	0	0	0	0
ENSG00000233907	0	0	0	0
ENSG00000233908	0	0	0	0
ENSG00000233909	0	0	0	0
ENSG00000233910	0	0	0	0
ENSG00000233911	0	0	0	0
ENSG00000233912	0	0	2	2
ENSG00000233913	1	0	0	0
ENSG00000233915	0	0	0	0
ENSG00000233916	2	0	7	14
ENSG00000233917	0	0	0	0
ENSG00000233918	0	0	0	0
ENSG00000233919	0	0	0	0
ENSG00000233920	0	0	0	0
ENSG00000233921	0	0	0	0
ENSG00000233922	0	0	0	0
ENSG00000233924	0	0	0	0
ENSG00000233926	0	0	0	0
ENSG00000233927	751	114	90	77
ENSG00000233928	0	0	0	0
ENSG00000233929	1	0	0	0
ENSG00000233930	0	0	0	0
ENSG00000233931	0	0	0	0
ENSG00000233932	0	0	0	0
ENSG00000233933	0	0	0	0
ENSG00000233934	0	0	0	0
ENSG00000233935	0	0	0	0
ENSG00000233936	0	0	0	0
ENSG00000233937	2	9	2	4
ENSG00000233938	0	0	0	0

ENSG00000233939	0	0	0	0
ENSG00000233940	0	0	0	0
ENSG00000233941	0	0	0	0
ENSG00000233942	1	0	1	1
ENSG00000233943	0	0	0	0
ENSG00000233944	0	0	0	0
ENSG00000233945	0	0	0	0
ENSG00000233946	0	0	0	0
ENSG00000233947	0	0	0	0
ENSG00000233948	0	0	0	0
ENSG00000233950	0	0	0	0
ENSG00000233951	0	0	0	0
ENSG00000233952	0	0	0	0
ENSG00000233953	0	0	0	0
ENSG00000233954	7	0	1	1
ENSG00000233955	0	0	0	0
ENSG00000233956	0	0	0	0
ENSG00000233960	0	0	0	0
ENSG00000233961	1	72	44	21
ENSG00000233962	0	0	0	0
ENSG00000233963	0	0	0	1
ENSG00000233964	0	0	0	0
ENSG00000233965	0	0	0	0
ENSG00000233966	0	0	0	0
ENSG00000233967	0	0	2	0
ENSG00000233968	1396	1992	3025	3977
ENSG00000233969	0	0	0	0
ENSG00000233970	0	0	0	0
ENSG00000233971	0	0	0	0
ENSG00000233972	0	0	0	0
ENSG00000233973	0	0	0	0
ENSG00000233974	0	0	0	0
ENSG00000233975	0	0	0	0
ENSG00000233977	0	0	0	0
ENSG00000233978	0	0	0	0
ENSG00000233979	0	0	0	0
ENSG00000233980	0	0	0	0
ENSG00000233981	0	0	0	0
ENSG00000233982	0	0	0	0
ENSG00000233983	0	0	0	0
ENSG00000233984	0	1	1	3
ENSG00000233985	1	0	0	0
ENSG00000233987	1	0	0	0
ENSG00000233988	0	0	0	0
ENSG00000233989	0	0	0	0
ENSG00000233990	0	0	0	0

ENSG00000233991	0	0	0	0
ENSG00000233993	0	0	0	0
ENSG00000233994	0	0	1	0
ENSG00000233995	0	0	0	0
ENSG00000233996	0	0	0	0
ENSG00000233997	1	0	0	0
ENSG00000233998	0	0	0	0
ENSG00000233999	0	0	0	0
ENSG00000234000	0	0	0	0
ENSG00000234001	0	0	0	0
ENSG00000234003	0	0	0	0
ENSG00000234004	0	0	0	0
ENSG00000234005	0	0	0	1
ENSG00000234006	0	0	0	0
ENSG00000234007	0	0	0	0
ENSG00000234008	0	0	0	0
ENSG00000234009	0	0	0	0
ENSG00000234011	0	0	0	0
ENSG00000234012	0	0	0	0
ENSG00000234015	0	0	0	0
ENSG00000234016	0	0	0	0
ENSG00000234017	0	0	0	0
ENSG00000234019	0	0	0	0
ENSG00000234020	0	0	0	0
ENSG00000234021	0	0	0	0
ENSG00000234022	2	23	15	25
ENSG00000234024	0	0	0	0
ENSG00000234025	0	0	0	0
ENSG00000234026	0	50	67	14
ENSG00000234028	13	35	31	52
ENSG00000234030	0	0	0	0
ENSG00000234031	0	90	60	155
ENSG00000234032	0	0	0	0
ENSG00000234033	0	0	0	0
ENSG00000234034	0	0	0	0
ENSG00000234035	0	0	0	0
ENSG00000234036	0	0	0	0
ENSG00000234037	0	0	0	0
ENSG00000234038	0	0	0	0
ENSG00000234039	0	0	0	0
ENSG00000234040	0	0	0	0
ENSG00000234041	0	0	0	0
ENSG00000234042	0	0	0	0
ENSG00000234043	4	45	28	52
ENSG00000234044	0	0	0	0
ENSG00000234046	0	0	0	0

ENSG00000234047	0	0	0	0
ENSG00000234048	0	0	0	0
ENSG00000234050	0	0	0	0
ENSG00000234052	0	0	0	0
ENSG00000234055	0	0	3	0
ENSG00000234056	0	0	0	0
ENSG00000234057	0	0	0	0
ENSG00000234058	0	0	0	0
ENSG00000234059	0	0	0	0
ENSG00000234060	0	0	0	0
ENSG00000234061	0	0	0	0
ENSG00000234062	0	1	4	6
ENSG00000234063	0	0	0	0
ENSG00000234064	0	0	0	0
ENSG00000234065	0	0	0	0
ENSG00000234066	0	0	0	0
ENSG00000234067	0	0	0	0
ENSG00000234068	0	0	0	0
ENSG00000234069	0	0	0	0
ENSG00000234070	0	0	0	0
ENSG00000234071	0	0	0	0
ENSG00000234072	0	0	6	4
ENSG00000234073	0	0	0	0
ENSG00000234074	0	0	0	0
ENSG00000234075	0	0	0	0
ENSG00000234076	5	0	0	0
ENSG00000234078	0	0	0	0
ENSG00000234079	0	0	0	0
ENSG00000234080	0	0	0	0
ENSG00000234081	0	0	0	0
ENSG00000234082	0	0	0	0
ENSG00000234083	0	0	0	0
ENSG00000234084	0	0	0	0
ENSG00000234085	0	0	0	0
ENSG00000234086	1	0	0	0
ENSG00000234087	0	0	0	0
ENSG00000234088	0	0	0	0
ENSG00000234089	0	0	0	0
ENSG00000234091	0	0	0	0
ENSG00000234092	0	0	0	0
ENSG00000234093	0	0	0	0
ENSG00000234095	0	0	0	0
ENSG00000234096	0	0	0	0
ENSG00000234099	0	0	0	0
ENSG00000234100	0	0	0	0
ENSG00000234101	0	0	0	0

ENSG00000234102	0	0	0	0
ENSG00000234103	0	0	0	0
ENSG00000234104	0	0	0	0
ENSG00000234105	1	0	0	0
ENSG00000234106	0	0	0	0
ENSG00000234107	0	0	0	0
ENSG00000234108	0	0	0	0
ENSG00000234109	0	0	0	0
ENSG00000234110	0	0	0	0
ENSG00000234111	0	0	0	0
ENSG00000234112	0	0	2	0
ENSG00000234113	0	0	0	0
ENSG00000234114	0	0	0	0
ENSG00000234115	0	0	0	0
ENSG00000234116	0	0	0	0
ENSG00000234117	40	98	213	116
ENSG00000234118	0	0	0	0
ENSG00000234119	0	0	0	0
ENSG00000234120	0	0	0	0
ENSG00000234121	0	0	0	0
ENSG00000234122	0	0	0	0
ENSG00000234123	0	0	0	0
ENSG00000234124	0	0	0	0
ENSG00000234125	0	0	0	0
ENSG00000234127	16	0	2	2
ENSG00000234129	0	100	117	299
ENSG00000234130	0	0	4	0
ENSG00000234131	0	0	0	0
ENSG00000234132	0	0	0	0
ENSG00000234133	0	0	0	0
ENSG00000234134	0	0	0	0
ENSG00000234135	0	0	0	0
ENSG00000234136	0	42	34	13
ENSG00000234137	0	0	0	0
ENSG00000234138	0	0	0	0
ENSG00000234139	0	0	3	0
ENSG00000234140	0	0	0	0
ENSG00000234141	1	13	45	19
ENSG00000234142	3	17	46	15
ENSG00000234143	0	0	0	0
ENSG00000234144	0	0	0	0
ENSG00000234145	0	0	1	0
ENSG00000234146	0	0	0	0
ENSG00000234147	0	0	5	2
ENSG00000234148	0	0	0	0
ENSG00000234149	0	0	0	0

ENSG00000234152	0	0	0	0
ENSG00000234154	0	0	0	0
ENSG00000234155	0	0	0	0
ENSG00000234156	0	22	0	0
ENSG00000234158	0	0	0	0
ENSG00000234159	0	0	0	0
ENSG00000234160	0	0	0	0
ENSG00000234161	0	121	14	42
ENSG00000234162	1	0	0	0
ENSG00000234163	0	0	0	0
ENSG00000234164	0	0	0	0
ENSG00000234165	0	0	0	0
ENSG00000234166	0	0	0	0
ENSG00000234167	0	0	0	0
ENSG00000234168	0	0	0	0
ENSG00000234169	0	0	0	0
ENSG00000234170	0	0	0	1
ENSG00000234171	5	6	44	83
ENSG00000234172	0	0	0	0
ENSG00000234173	0	0	0	0
ENSG00000234174	0	0	0	0
ENSG00000234175	0	0	0	0
ENSG00000234176	0	0	0	0
ENSG00000234177	0	0	0	0
ENSG00000234178	0	0	0	0
ENSG00000234179	0	0	0	0
ENSG00000234181	0	0	0	0
ENSG00000234182	0	0	0	0
ENSG00000234183	1	0	0	0
ENSG00000234184	13	0	0	0
ENSG00000234185	0	0	0	0
ENSG00000234186	0	0	0	0
ENSG00000234187	0	0	1	0
ENSG00000234188	0	0	0	0
ENSG00000234189	0	0	0	0
ENSG00000234190	0	0	0	0
ENSG00000234191	0	0	1	0
ENSG00000234192	0	0	0	0
ENSG00000234193	0	0	0	0
ENSG00000234195	0	0	0	0
ENSG00000234196	0	0	0	0
ENSG00000234197	0	0	0	0
ENSG00000234198	0	12	3	3
ENSG00000234199	1	0	0	2
ENSG00000234200	0	0	0	0
ENSG00000234201	0	0	0	6

ENSG00000234202	0	0	0	0
ENSG00000234203	0	0	2	0
ENSG00000234204	0	0	0	0
ENSG00000234206	0	0	0	0
ENSG00000234207	0	0	0	0
ENSG00000234208	0	0	0	0
ENSG00000234210	0	0	0	0
ENSG00000234211	0	0	0	0
ENSG00000234213	0	0	0	0
ENSG00000234214	0	0	0	0
ENSG00000234215	0	0	0	0
ENSG00000234216	0	0	0	0
ENSG00000234217	0	0	0	0
ENSG00000234218	0	0	0	0
ENSG00000234219	0	0	0	0
ENSG00000234222	0	18	7	5
ENSG00000234223	0	0	0	0
ENSG00000234224	0	0	0	0
ENSG00000234225	0	0	0	0
ENSG00000234226	0	0	0	0
ENSG00000234227	0	0	0	0
ENSG00000234228	0	0	0	0
ENSG00000234229	0	0	0	0
ENSG00000234230	0	0	0	0
ENSG00000234231	24	393	503	468
ENSG00000234232	0	0	0	0
ENSG00000234233	0	0	0	0
ENSG00000234234	0	0	0	0
ENSG00000234235	0	0	0	0
ENSG00000234237	0	0	0	0
ENSG00000234238	0	0	0	0
ENSG00000234239	0	0	0	0
ENSG00000234241	0	0	0	0
ENSG00000234242	0	0	0	0
ENSG00000234243	0	0	0	0
ENSG00000234244	0	0	0	0
ENSG00000234245	0	0	0	0
ENSG00000234247	0	0	0	0
ENSG00000234248	0	0	0	0
ENSG00000234249	0	0	0	0
ENSG00000234251	0	0	0	0
ENSG00000234252	0	0	0	0
ENSG00000234253	0	0	1	0
ENSG00000234255	1	0	0	0
ENSG00000234256	0	0	0	0
ENSG00000234257	0	0	0	0

ENSG00000234258	0	0	0	0
ENSG00000234259	0	0	0	0
ENSG00000234260	0	0	0	0
ENSG00000234261	0	0	0	0
ENSG00000234262	0	0	0	0
ENSG00000234263	2	0	0	0
ENSG00000234264	0	0	2	0
ENSG00000234265	0	0	0	0
ENSG00000234267	0	0	0	0
ENSG00000234268	2	0	1	0
ENSG00000234269	0	0	0	0
ENSG00000234270	0	0	0	0
ENSG00000234271	0	0	0	0
ENSG00000234272	0	0	0	0
ENSG00000234273	0	0	0	0
ENSG00000234274	0	0	0	0
ENSG00000234275	0	0	0	0
ENSG00000234276	0	0	0	0
ENSG00000234277	0	0	0	0
ENSG00000234278	0	0	0	0
ENSG00000234279	0	0	0	0
ENSG00000234280	0	0	0	0
ENSG00000234281	0	0	0	0
ENSG00000234282	0	0	0	0
ENSG00000234283	0	0	0	0
ENSG00000234284	1	2	6	4
ENSG00000234285	0	0	0	0
ENSG00000234286	0	0	0	0
ENSG00000234287	1	0	0	1
ENSG00000234288	0	0	0	0
ENSG00000234289	0	15	9	12
ENSG00000234290	0	0	0	0
ENSG00000234292	0	0	0	0
ENSG00000234293	0	0	0	0
ENSG00000234295	0	0	0	0
ENSG00000234296	0	0	0	0
ENSG00000234297	0	0	0	0
ENSG00000234298	0	0	0	0
ENSG00000234299	0	0	0	0
ENSG00000234300	0	0	0	0
ENSG00000234303	0	0	0	0
ENSG00000234304	0	0	0	0
ENSG00000234305	0	0	0	0
ENSG00000234306	0	0	0	0
ENSG00000234308	0	0	0	0
ENSG00000234309	0	0	0	0

ENSG00000234311	0	0	0	0
ENSG00000234312	0	0	0	0
ENSG00000234313	0	0	0	0
ENSG00000234315	0	0	0	0
ENSG00000234318	0	0	0	1
ENSG00000234319	0	0	0	0
ENSG00000234320	0	0	0	0
ENSG00000234322	0	0	0	0
ENSG00000234323	0	0	0	0
ENSG00000234324	0	0	0	0
ENSG00000234325	0	0	0	0
ENSG00000234327	2	0	5	0
ENSG00000234328	0	0	0	0
ENSG00000234329	0	0	0	0
ENSG00000234331	0	0	0	0
ENSG00000234332	0	8	11	6
ENSG00000234333	0	0	0	0
ENSG00000234335	0	0	0	0
ENSG00000234336	1	0	0	0
ENSG00000234337	0	7	4	5
ENSG00000234338	0	0	0	0
ENSG00000234340	0	0	0	0
ENSG00000234343	0	0	0	0
ENSG00000234344	0	0	0	0
ENSG00000234345	0	0	0	0
ENSG00000234347	0	0	0	0
ENSG00000234349	0	0	0	0
ENSG00000234350	0	0	0	0
ENSG00000234351	0	0	0	0
ENSG00000234352	0	0	0	0
ENSG00000234353	0	0	0	0
ENSG00000234354	1	0	0	0
ENSG00000234355	0	0	0	0
ENSG00000234356	0	0	0	0
ENSG00000234358	0	0	0	0
ENSG00000234359	0	0	0	0
ENSG00000234360	0	0	0	0
ENSG00000234361	1	0	0	0
ENSG00000234362	0	0	0	0
ENSG00000234363	0	0	0	0
ENSG00000234364	0	0	0	0
ENSG00000234365	0	0	0	0
ENSG00000234367	0	0	0	0
ENSG00000234369	0	0	0	0
ENSG00000234370	0	0	0	0
ENSG00000234371	0	0	0	0

ENSG00000234372	0	0	0	0
ENSG00000234373	0	0	0	0
ENSG00000234375	0	0	0	0
ENSG00000234376	0	0	0	0
ENSG00000234377	0	0	1	0
ENSG00000234378	0	0	0	0
ENSG00000234379	0	0	0	0
ENSG00000234380	0	0	0	0
ENSG00000234381	0	0	0	0
ENSG00000234382	0	0	0	0
ENSG00000234383	0	0	0	0
ENSG00000234384	0	0	0	0
ENSG00000234385	0	0	0	0
ENSG00000234386	0	0	0	0
ENSG00000234387	0	0	0	0
ENSG00000234388	0	0	0	0
ENSG00000234389	0	0	0	0
ENSG00000234390	1	0	0	0
ENSG00000234391	0	0	0	0
ENSG00000234393	0	0	0	0
ENSG00000234394	0	0	0	0
ENSG00000234396	0	0	0	0
ENSG00000234397	0	0	0	0
ENSG00000234398	0	0	0	0
ENSG00000234399	0	0	0	0
ENSG00000234402	0	0	0	0
ENSG00000234404	0	0	0	0
ENSG00000234405	0	0	4	0
ENSG00000234406	0	0	0	0
ENSG00000234407	0	0	0	0
ENSG00000234409	0	0	0	0
ENSG00000234411	0	0	0	0
ENSG00000234413	0	0	0	0
ENSG00000234414	0	0	0	0
ENSG00000234415	0	0	0	0
ENSG00000234416	0	0	0	0
ENSG00000234417	0	0	0	0
ENSG00000234418	0	0	0	1
ENSG00000234419	0	0	0	0
ENSG00000234420	0	0	24	13
ENSG00000234421	0	0	0	0
ENSG00000234422	0	0	0	0
ENSG00000234423	0	0	0	0
ENSG00000234424	0	0	0	0
ENSG00000234425	329	233	208	168
ENSG00000234426	0	0	0	0

ENSG00000234427	0	0	0	0
ENSG00000234428	3	0	0	1
ENSG00000234429	0	0	0	0
ENSG00000234430	0	0	0	0
ENSG00000234431	44	31	26	6
ENSG00000234432	0	0	5	0
ENSG00000234433	0	0	0	0
ENSG00000234435	0	0	0	0
ENSG00000234436	0	0	0	0
ENSG00000234437	0	0	0	0
ENSG00000234438	0	0	0	0
ENSG00000234439	0	0	0	0
ENSG00000234441	0	0	0	0
ENSG00000234442	0	0	0	0
ENSG00000234443	0	0	0	0
ENSG00000234444	1	40	6	26
ENSG00000234445	0	0	0	0
ENSG00000234446	0	0	0	0
ENSG00000234448	0	0	0	0
ENSG00000234449	0	34	404	10
ENSG00000234450	0	0	0	0
ENSG00000234451	0	0	1	0
ENSG00000234452	0	0	0	0
ENSG00000234453	0	0	0	0
ENSG00000234454	0	0	0	0
ENSG00000234455	0	0	0	0
ENSG00000234456	947	746	821	2119
ENSG00000234457	0	0	0	0
ENSG00000234458	0	0	0	0
ENSG00000234459	0	0	0	0
ENSG00000234460	0	0	0	0
ENSG00000234461	0	0	0	0
ENSG00000234463	0	0	0	0
ENSG00000234464	0	0	0	0
ENSG00000234465	60	101	21	70
ENSG00000234466	0	0	0	0
ENSG00000234467	0	0	0	0
ENSG00000234469	0	0	0	0
ENSG00000234470	0	0	0	0
ENSG00000234471	0	0	0	0
ENSG00000234473	0	0	0	0
ENSG00000234474	0	0	0	0
ENSG00000234475	0	0	0	0
ENSG00000234476	26	0	9	28
ENSG00000234477	0	0	0	0
ENSG00000234478	0	0	0	3

ENSG00000234479	0	0	0	0
ENSG00000234480	0	0	0	0
ENSG00000234481	0	0	0	0
ENSG00000234482	0	0	0	0
ENSG00000234484	0	0	0	0
ENSG00000234485	0	0	0	0
ENSG00000234487	0	0	0	0
ENSG00000234488	0	0	0	0
ENSG00000234489	0	0	0	0
ENSG00000234491	0	0	0	0
ENSG00000234492	0	0	14	23
ENSG00000234493	0	0	0	0
ENSG00000234494	3	0	2	0
ENSG00000234495	0	0	0	0
ENSG00000234496	0	0	0	0
ENSG00000234497	0	0	0	0
ENSG00000234498	0	0	0	0
ENSG00000234500	0	12	0	0
ENSG00000234502	0	0	0	0
ENSG00000234503	0	0	0	0
ENSG00000234504	0	0	0	0
ENSG00000234506	3	0	3	0
ENSG00000234507	0	0	0	0
ENSG00000234508	0	0	0	0
ENSG00000234509	1	18	9	0
ENSG00000234510	0	0	0	0
ENSG00000234511	1	1	6	10
ENSG00000234512	0	0	0	0
ENSG00000234513	2	0	0	0
ENSG00000234514	0	0	0	0
ENSG00000234515	9	30	1	2
ENSG00000234516	0	0	0	0
ENSG00000234518	0	0	0	0
ENSG00000234519	0	0	0	0
ENSG00000234520	0	0	0	1
ENSG00000234521	0	0	0	0
ENSG00000234522	0	0	0	0
ENSG00000234523	0	0	0	0
ENSG00000234524	0	0	0	0
ENSG00000234527	0	0	0	0
ENSG00000234528	0	0	0	0
ENSG00000234529	0	0	0	0
ENSG00000234530	0	0	0	0
ENSG00000234531	0	0	4	3
ENSG00000234534	0	0	0	0
ENSG00000234535	0	0	0	0

ENSG00000234536	0	0	0	0
ENSG00000234537	0	0	0	0
ENSG00000234538	0	0	0	0
ENSG00000234539	0	0	0	0
ENSG00000234540	0	0	0	0
ENSG00000234541	0	0	0	0
ENSG00000234542	0	0	0	0
ENSG00000234544	0	0	0	0
ENSG00000234545	356	2108	1338	2063
ENSG00000234546	0	0	0	0
ENSG00000234548	0	0	0	0
ENSG00000234549	0	0	0	0
ENSG00000234551	0	0	0	0
ENSG00000234553	0	0	0	0
ENSG00000234554	0	0	0	0
ENSG00000234556	0	0	0	4
ENSG00000234557	0	0	9	4
ENSG00000234558	0	0	0	0
ENSG00000234559	0	0	0	1
ENSG00000234560	0	0	0	0
ENSG00000234562	0	0	0	0
ENSG00000234564	0	0	0	0
ENSG00000234565	0	0	0	0
ENSG00000234566	0	0	0	0
ENSG00000234567	0	0	0	0
ENSG00000234568	2	0	0	0
ENSG00000234569	0	0	0	0
ENSG00000234570	0	0	0	0
ENSG00000234571	0	0	0	0
ENSG00000234572	0	0	0	0
ENSG00000234573	0	0	0	0
ENSG00000234574	0	0	0	0
ENSG00000234575	0	0	0	0
ENSG00000234576	0	0	0	0
ENSG00000234577	0	0	0	0
ENSG00000234578	0	0	0	0
ENSG00000234579	0	0	0	0
ENSG00000234580	0	0	0	0
ENSG00000234583	0	0	0	0
ENSG00000234584	2	14	15	6
ENSG00000234585	909	4187	3769	2594
ENSG00000234586	0	0	0	0
ENSG00000234587	0	0	0	0
ENSG00000234588	0	0	0	0
ENSG00000234589	0	0	0	0
ENSG00000234590	0	0	0	0

ENSG00000234592	0	0	0	0
ENSG00000234593	0	0	0	0
ENSG00000234594	0	0	0	0
ENSG00000234595	0	0	0	0
ENSG00000234596	0	0	0	0
ENSG00000234597	0	0	0	0
ENSG00000234599	0	0	0	0
ENSG00000234601	1	0	0	0
ENSG00000234602	0	0	0	0
ENSG00000234603	0	0	0	0
ENSG00000234604	0	0	0	0
ENSG00000234605	0	0	0	0
ENSG00000234607	0	0	0	0
ENSG00000234608	2	24	2	1
ENSG00000234610	0	0	0	0
ENSG00000234611	0	0	0	0
ENSG00000234612	0	0	0	0
ENSG00000234613	0	0	0	0
ENSG00000234614	3	44	9	7
ENSG00000234616	2	0	0	0
ENSG00000234617	0	0	3	0
ENSG00000234618	1	0	0	0
ENSG00000234619	0	0	0	0
ENSG00000234620	0	0	0	0
ENSG00000234622	0	0	0	0
ENSG00000234623	0	0	0	0
ENSG00000234624	0	0	0	0
ENSG00000234625	0	0	0	0
ENSG00000234626	1	0	0	0
ENSG00000234627	0	0	0	0
ENSG00000234629	0	0	0	0
ENSG00000234630	0	0	0	0
ENSG00000234631	0	0	0	0
ENSG00000234632	0	0	0	0
ENSG00000234634	0	0	0	0
ENSG00000234635	1	0	0	0
ENSG00000234636	8	14	14	13
ENSG00000234637	0	0	0	0
ENSG00000234638	0	0	0	0
ENSG00000234639	0	0	1	0
ENSG00000234640	0	0	0	0
ENSG00000234642	0	0	0	0
ENSG00000234643	0	0	0	0
ENSG00000234644	0	0	0	0
ENSG00000234645	0	0	0	0
ENSG00000234646	0	0	0	0

ENSG00000234647	1	0	0	0
ENSG00000234648	0	0	0	0
ENSG00000234650	0	0	0	0
ENSG00000234651	0	0	0	0
ENSG00000234652	0	0	0	0
ENSG00000234653	0	0	0	0
ENSG00000234654	0	0	0	0
ENSG00000234655	0	0	0	0
ENSG00000234658	0	0	0	0
ENSG00000234660	0	0	0	0
ENSG00000234661	0	0	0	0
ENSG00000234663	1	0	1	6
ENSG00000234664	0	0	0	0
ENSG00000234665	0	0	0	16
ENSG00000234667	0	0	0	0
ENSG00000234668	0	0	0	0
ENSG00000234669	0	0	0	0
ENSG00000234670	0	0	0	0
ENSG00000234671	0	0	0	0
ENSG00000234673	0	0	0	0
ENSG00000234674	0	0	0	0
ENSG00000234675	0	0	0	0
ENSG00000234676	9	40	20	18
ENSG00000234677	0	0	0	0
ENSG00000234678	0	0	0	0
ENSG00000234680	0	0	0	0
ENSG00000234681	0	0	0	0
ENSG00000234682	0	0	0	0
ENSG00000234683	0	0	0	0
ENSG00000234684	6	26	10	18
ENSG00000234685	0	0	0	0
ENSG00000234686	0	0	0	0
ENSG00000234688	0	0	0	0
ENSG00000234689	0	0	2	3
ENSG00000234690	0	0	0	0
ENSG00000234692	0	0	0	0
ENSG00000234693	0	0	0	0
ENSG00000234694	38	0	13	15
ENSG00000234695	3	28	0	0
ENSG00000234696	0	0	0	0
ENSG00000234698	2	0	9	3
ENSG00000234699	0	0	0	0
ENSG00000234700	0	0	0	0
ENSG00000234701	0	0	0	0
ENSG00000234702	0	0	0	0
ENSG00000234703	0	0	0	0

ENSG00000234704	0	0	0	0
ENSG00000234705	0	0	0	0
ENSG00000234706	0	0	0	0
ENSG00000234707	0	0	0	0
ENSG00000234709	0	0	0	0
ENSG00000234710	0	0	28	32
ENSG00000234711	0	0	0	0
ENSG00000234712	0	0	0	0
ENSG00000234713	0	0	0	0
ENSG00000234714	0	0	0	0
ENSG00000234715	0	0	0	0
ENSG00000234716	0	0	0	0
ENSG00000234717	0	0	0	0
ENSG00000234718	0	0	0	0
ENSG00000234719	0	0	1	1
ENSG00000234720	0	0	0	0
ENSG00000234721	0	0	0	0
ENSG00000234722	0	0	0	0
ENSG00000234723	0	0	0	0
ENSG00000234724	0	0	0	0
ENSG00000234725	0	0	0	0
ENSG00000234726	0	0	0	0
ENSG00000234728	0	0	0	0
ENSG00000234729	0	0	0	0
ENSG00000234730	0	0	0	0
ENSG00000234731	0	0	0	0
ENSG00000234732	0	0	0	0
ENSG00000234734	0	0	0	0
ENSG00000234735	0	0	0	0
ENSG00000234736	0	0	0	0
ENSG00000234737	0	0	0	0
ENSG00000234740	0	0	2	0
ENSG00000234741	163	41	142	226
ENSG00000234742	2	0	3	2
ENSG00000234743	0	1	0	0
ENSG00000234744	0	0	0	0
ENSG00000234745	12939	22368	28920	27480
ENSG00000234746	0	0	0	0
ENSG00000234748	0	0	0	0
ENSG00000234749	0	0	0	0
ENSG00000234750	0	0	0	0
ENSG00000234751	0	0	0	0
ENSG00000234752	0	0	0	0
ENSG00000234753	0	0	0	0
ENSG00000234754	0	0	0	0
ENSG00000234756	0	0	0	0

ENSG00000234757	0	0	0	0
ENSG00000234758	0	0	0	0
ENSG00000234761	0	0	0	0
ENSG00000234763	0	0	0	0
ENSG00000234764	0	0	0	0
ENSG00000234765	0	0	0	0
ENSG00000234766	0	0	0	0
ENSG00000234767	0	0	0	0
ENSG00000234768	0	0	0	0
ENSG00000234769	27	18	6	1
ENSG00000234770	0	0	0	0
ENSG00000234771	0	0	0	0
ENSG00000234772	3	0	20	18
ENSG00000234773	0	22	39	50
ENSG00000234775	0	0	0	0
ENSG00000234776	0	0	0	0
ENSG00000234777	0	0	0	0
ENSG00000234779	0	0	0	0
ENSG00000234780	0	0	0	0
ENSG00000234781	0	0	0	1
ENSG00000234782	0	0	0	0
ENSG00000234784	0	0	0	0
ENSG00000234785	0	0	0	0
ENSG00000234786	0	0	0	0
ENSG00000234787	0	0	0	0
ENSG00000234788	0	0	0	0
ENSG00000234789	0	0	0	0
ENSG00000234790	0	0	0	0
ENSG00000234791	0	0	0	0
ENSG00000234792	0	0	0	0
ENSG00000234793	0	0	0	0
ENSG00000234794	0	0	0	0
ENSG00000234795	0	0	0	0
ENSG00000234796	0	0	0	0
ENSG00000234797	1	10	4	29
ENSG00000234798	0	0	0	0
ENSG00000234799	0	0	0	0
ENSG00000234800	0	0	0	0
ENSG00000234801	0	0	0	0
ENSG00000234803	0	0	0	0
ENSG00000234804	0	0	0	0
ENSG00000234805	0	0	0	0
ENSG00000234806	0	0	0	0
ENSG00000234807	0	0	0	0
ENSG00000234809	0	0	0	0
ENSG00000234810	173	275	195	703

ENSG00000234811	0	0	0	0
ENSG00000234812	0	0	0	0
ENSG00000234813	0	0	0	0
ENSG00000234814	0	31	5	14
ENSG00000234816	0	0	4	0
ENSG00000234817	0	0	0	0
ENSG00000234818	0	0	0	5
ENSG00000234819	0	0	0	0
ENSG00000234821	0	0	0	0
ENSG00000234825	0	0	0	0
ENSG00000234826	0	0	0	0
ENSG00000234828	0	0	0	0
ENSG00000234829	0	0	0	0
ENSG00000234830	0	0	0	0
ENSG00000234831	0	0	0	0
ENSG00000234832	0	0	0	0
ENSG00000234834	0	0	0	0
ENSG00000234835	0	0	0	0
ENSG00000234836	0	0	0	0
ENSG00000234837	0	0	0	0
ENSG00000234838	0	0	0	0
ENSG00000234839	0	0	0	0
ENSG00000234840	0	21	3	0
ENSG00000234841	0	0	0	0
ENSG00000234842	0	0	0	0
ENSG00000234844	0	0	0	0
ENSG00000234846	0	0	0	0
ENSG00000234848	0	0	0	0
ENSG00000234849	0	0	0	0
ENSG00000234850	0	0	0	0
ENSG00000234851	0	11	1	8
ENSG00000234852	0	0	0	0
ENSG00000234853	0	0	1	0
ENSG00000234854	0	0	0	0
ENSG00000234855	0	0	0	0
ENSG00000234856	0	0	0	0
ENSG00000234857	0	0	0	0
ENSG00000234858	0	0	0	0
ENSG00000234859	0	0	0	0
ENSG00000234860	0	0	1	0
ENSG00000234861	0	0	0	0
ENSG00000234862	0	0	0	0
ENSG00000234863	0	0	0	0
ENSG00000234864	0	0	0	0
ENSG00000234865	0	0	0	0
ENSG00000234866	0	0	0	0

ENSG00000234869	0	0	0	0
ENSG00000234871	0	10	3	2
ENSG00000234872	0	0	0	0
ENSG00000234876	0	0	0	0
ENSG00000234877	0	0	0	0
ENSG00000234878	0	0	0	0
ENSG00000234880	0	0	0	0
ENSG00000234881	0	0	0	0
ENSG00000234882	2	0	0	0
ENSG00000234883	0	0	0	0
ENSG00000234884	7	41	24	30
ENSG00000234886	0	0	0	0
ENSG00000234888	0	0	0	0
ENSG00000234889	0	0	0	0
ENSG00000234890	0	0	0	0
ENSG00000234891	0	0	0	0
ENSG00000234892	0	0	0	0
ENSG00000234893	0	0	0	0
ENSG00000234894	0	0	0	0
ENSG00000234895	0	0	0	0
ENSG00000234896	1	0	0	0
ENSG00000234898	0	0	0	0
ENSG00000234899	0	0	4	0
ENSG00000234900	0	0	0	0
ENSG00000234901	0	0	0	0
ENSG00000234902	5	27	1	2
ENSG00000234903	0	0	0	0
ENSG00000234904	0	0	0	0
ENSG00000234906	0	0	0	0
ENSG00000234907	0	0	0	0
ENSG00000234910	0	0	0	0
ENSG00000234911	0	17	7	6
ENSG00000234912	269	354	374	89
ENSG00000234913	0	1	0	0
ENSG00000234915	0	0	0	0
ENSG00000234917	0	0	1	0
ENSG00000234918	0	0	0	0
ENSG00000234919	2	5	0	5
ENSG00000234920	0	0	0	0
ENSG00000234921	0	0	0	0
ENSG00000234922	0	0	0	0
ENSG00000234925	0	0	5	0
ENSG00000234927	0	0	0	0
ENSG00000234928	0	0	0	0
ENSG00000234929	0	0	0	0
ENSG00000234931	0	0	0	0

ENSG00000234932	0	0	0	0
ENSG00000234933	0	0	0	0
ENSG00000234934	0	0	0	0
ENSG00000234936	8	0	2	6
ENSG00000234937	0	0	0	0
ENSG00000234938	0	0	0	0
ENSG00000234939	0	0	0	0
ENSG00000234940	0	0	0	0
ENSG00000234941	0	0	0	0
ENSG00000234942	0	0	0	0
ENSG00000234943	0	0	0	0
ENSG00000234944	0	0	0	0
ENSG00000234945	0	0	0	0
ENSG00000234946	0	0	0	0
ENSG00000234947	0	0	0	0
ENSG00000234948	0	0	0	0
ENSG00000234949	0	1	0	0
ENSG00000234950	0	0	0	0
ENSG00000234951	0	0	0	0
ENSG00000234952	0	0	0	0
ENSG00000234953	0	0	0	0
ENSG00000234954	0	0	0	0
ENSG00000234956	1	0	0	0
ENSG00000234958	0	0	0	0
ENSG00000234961	0	14	8	11
ENSG00000234962	0	0	0	0
ENSG00000234964	0	0	0	0
ENSG00000234965	0	0	0	0
ENSG00000234966	0	0	0	0
ENSG00000234967	0	0	0	0
ENSG00000234968	0	0	0	0
ENSG00000234969	0	0	0	0
ENSG00000234970	0	0	0	0
ENSG00000234972	0	0	0	0
ENSG00000234973	4	0	1	0
ENSG00000234975	9	181	224	277
ENSG00000234977	0	0	0	0
ENSG00000234978	1	1	35	7
ENSG00000234979	0	0	0	0
ENSG00000234980	0	0	0	0
ENSG00000234981	0	0	0	0
ENSG00000234982	0	0	0	0
ENSG00000234983	0	0	1	2
ENSG00000234984	0	0	1	0
ENSG00000234985	0	0	0	0
ENSG00000234986	0	0	0	0

ENSG00000234988	0	0	0	0
ENSG00000234992	0	0	0	0
ENSG00000234993	0	5	2	3
ENSG00000234995	0	0	0	0
ENSG00000234996	0	0	0	0
ENSG00000234997	0	0	6	10
ENSG00000234998	0	0	0	0
ENSG00000234999	0	0	0	0
ENSG00000235001	0	0	0	0
ENSG00000235002	0	0	0	0
ENSG00000235003	0	0	0	0
ENSG00000235004	0	0	0	0
ENSG00000235005	22	30	48	28
ENSG00000235007	0	0	0	0
ENSG00000235008	0	0	0	1
ENSG00000235009	0	0	0	0
ENSG00000235010	0	0	0	0
ENSG00000235011	0	0	0	0
ENSG00000235012	0	0	0	0
ENSG00000235013	0	0	0	0
ENSG00000235014	0	0	0	0
ENSG00000235015	0	0	0	0
ENSG00000235016	0	0	0	0
ENSG00000235017	0	0	0	0
ENSG00000235018	0	0	0	0
ENSG00000235020	0	0	0	0
ENSG00000235021	0	0	0	0
ENSG00000235022	0	0	0	0
ENSG00000235023	0	0	0	0
ENSG00000235024	0	0	0	0
ENSG00000235025	0	0	0	0
ENSG00000235026	0	0	0	0
ENSG00000235027	0	19	2	0
ENSG00000235028	0	0	0	0
ENSG00000235029	0	0	0	0
ENSG00000235030	0	0	0	0
ENSG00000235032	0	0	0	0
ENSG00000235033	0	0	0	0
ENSG00000235034	0	0	1	0
ENSG00000235035	0	0	0	0
ENSG00000235036	0	0	1	4
ENSG00000235037	0	0	0	0
ENSG00000235038	0	0	0	0
ENSG00000235039	0	0	0	0
ENSG00000235040	0	0	0	0
ENSG00000235042	0	0	2	0

ENSG00000235043	0	0	0	3
ENSG00000235044	0	0	0	0
ENSG00000235045	0	0	0	0
ENSG00000235047	0	0	0	0
ENSG00000235049	0	0	0	0
ENSG00000235050	0	0	0	0
ENSG00000235051	0	0	0	0
ENSG00000235052	0	0	0	0
ENSG00000235054	0	0	0	0
ENSG00000235055	0	0	0	0
ENSG00000235056	0	0	0	0
ENSG00000235058	0	0	0	0
ENSG00000235059	0	0	0	0
ENSG00000235060	0	0	0	0
ENSG00000235061	0	0	0	0
ENSG00000235062	0	0	0	0
ENSG00000235064	0	0	0	0
ENSG00000235065	4	0	0	0
ENSG00000235066	0	0	0	0
ENSG00000235067	0	0	0	0
ENSG00000235068	0	0	0	0
ENSG00000235069	0	0	0	0
ENSG00000235070	4	35	14	19
ENSG00000235071	0	0	0	0
ENSG00000235072	0	0	0	0
ENSG00000235073	0	0	0	0
ENSG00000235074	0	0	0	0
ENSG00000235076	0	0	0	0
ENSG00000235077	0	0	0	2
ENSG00000235078	10	96	34	52
ENSG00000235079	0	0	3	8
ENSG00000235080	0	0	0	0
ENSG00000235081	0	0	0	0
ENSG00000235082	0	0	5	4
ENSG00000235083	0	0	0	0
ENSG00000235084	2	0	0	0
ENSG00000235085	0	0	0	0
ENSG00000235086	0	0	0	0
ENSG00000235088	0	0	0	0
ENSG00000235089	0	0	0	0
ENSG00000235090	0	0	0	0
ENSG00000235091	0	0	0	0
ENSG00000235092	4	30	5	7
ENSG00000235094	0	0	0	0
ENSG00000235095	0	0	0	1
ENSG00000235096	0	0	0	0

ENSG00000235097	0	0	0	0
ENSG00000235098	0	0	0	2
ENSG00000235099	0	0	0	0
ENSG00000235100	0	0	0	7
ENSG00000235101	1	22	0	0
ENSG00000235102	0	0	0	0
ENSG00000235105	0	0	0	6
ENSG00000235106	18	1	56	10
ENSG00000235107	0	0	0	0
ENSG00000235108	0	0	0	0
ENSG00000235109	0	0	6	2
ENSG00000235110	0	0	0	0
ENSG00000235111	0	0	0	1
ENSG00000235112	0	0	0	0
ENSG00000235113	0	0	0	0
ENSG00000235115	0	0	0	0
ENSG00000235116	0	0	0	0
ENSG00000235117	0	0	0	0
ENSG00000235118	1	0	0	0
ENSG00000235119	0	0	0	0
ENSG00000235120	0	0	0	0
ENSG00000235121	0	0	0	0
ENSG00000235122	0	0	0	0
ENSG00000235123	0	0	0	0
ENSG00000235124	0	0	0	0
ENSG00000235125	0	0	0	0
ENSG00000235126	0	0	0	0
ENSG00000235127	0	0	0	0
ENSG00000235128	0	0	0	0
ENSG00000235129	0	0	0	0
ENSG00000235130	0	0	0	0
ENSG00000235131	0	0	0	0
ENSG00000235132	0	0	0	0
ENSG00000235133	0	0	0	0
ENSG00000235134	0	0	0	0
ENSG00000235135	0	0	0	0
ENSG00000235136	0	0	0	0
ENSG00000235137	0	0	0	0
ENSG00000235138	0	0	0	0
ENSG00000235139	0	0	0	0
ENSG00000235140	0	0	0	0
ENSG00000235141	0	0	0	0
ENSG00000235142	0	0	0	0
ENSG00000235143	0	0	0	0
ENSG00000235144	0	0	0	0
ENSG00000235145	0	0	0	7

ENSG00000235146	0	0	0	0
ENSG00000235147	0	0	0	0
ENSG00000235148	0	0	0	0
ENSG00000235149	0	0	0	0
ENSG00000235150	0	0	0	0
ENSG00000235151	0	0	0	0
ENSG00000235152	0	0	0	0
ENSG00000235154	0	0	0	0
ENSG00000235155	0	0	0	0
ENSG00000235156	0	0	0	0
ENSG00000235158	0	0	0	0
ENSG00000235159	0	0	1	5
ENSG00000235160	0	0	2	0
ENSG00000235162	1268	5313	4770	8528
ENSG00000235163	0	0	0	0
ENSG00000235165	0	0	0	0
ENSG00000235166	0	0	0	0
ENSG00000235167	0	0	0	0
ENSG00000235168	0	0	0	0
ENSG00000235169	17	16	6	12
ENSG00000235170	0	0	0	0
ENSG00000235171	0	0	0	0
ENSG00000235172	1	0	0	3
ENSG00000235173	0	0	0	0
ENSG00000235174	0	0	0	0
ENSG00000235175	0	0	0	0
ENSG00000235176	0	0	0	0
ENSG00000235178	0	0	0	0
ENSG00000235180	1	0	0	0
ENSG00000235181	0	0	0	0
ENSG00000235182	0	0	0	0
ENSG00000235183	0	0	0	0
ENSG00000235184	0	0	0	0
ENSG00000235185	0	0	0	0
ENSG00000235186	0	0	0	0
ENSG00000235189	0	3	6	8
ENSG00000235190	0	0	0	0
ENSG00000235191	0	3	16	27
ENSG00000235192	1	0	0	0
ENSG00000235193	0	0	0	0
ENSG00000235194	39	75	25	56
ENSG00000235195	0	0	0	0
ENSG00000235196	0	0	0	0
ENSG00000235197	0	0	0	0
ENSG00000235198	0	0	0	0
ENSG00000235199	0	0	0	0

ENSG00000235200	0	0	0	0
ENSG00000235201	0	0	0	0
ENSG00000235202	0	0	0	0
ENSG00000235203	0	0	0	0
ENSG00000235204	0	0	0	0
ENSG00000235205	0	0	0	0
ENSG00000235207	0	0	0	0
ENSG00000235208	0	0	0	0
ENSG00000235209	0	0	0	0
ENSG00000235211	0	0	0	0
ENSG00000235213	0	0	0	4
ENSG00000235214	0	0	0	0
ENSG00000235215	0	0	0	0
ENSG00000235217	2	0	0	0
ENSG00000235218	0	0	0	0
ENSG00000235219	0	0	0	0
ENSG00000235220	0	0	0	0
ENSG00000235221	0	0	0	0
ENSG00000235222	0	0	0	0
ENSG00000235223	0	0	0	0
ENSG00000235224	0	0	0	0
ENSG00000235225	0	0	0	0
ENSG00000235226	0	0	0	0
ENSG00000235227	0	0	0	0
ENSG00000235228	0	0	0	0
ENSG00000235231	0	0	0	0
ENSG00000235232	0	0	0	0
ENSG00000235233	0	0	0	0
ENSG00000235235	0	0	0	0
ENSG00000235236	0	0	1	2
ENSG00000235237	0	0	0	0
ENSG00000235238	0	0	0	3
ENSG00000235239	0	0	0	1
ENSG00000235240	0	0	0	0
ENSG00000235241	0	0	0	0
ENSG00000235242	0	0	0	0
ENSG00000235243	0	0	0	0
ENSG00000235244	0	0	0	2
ENSG00000235245	0	0	0	1
ENSG00000235246	0	0	1	1
ENSG00000235248	0	0	0	0
ENSG00000235249	0	0	0	0
ENSG00000235250	0	0	0	0
ENSG00000235251	0	0	0	0
ENSG00000235253	0	0	0	0
ENSG00000235254	0	0	1	0

ENSG00000235256	0	0	0	0
ENSG00000235257	583	1640	1232	2396
ENSG00000235258	0	0	0	0
ENSG00000235259	0	0	0	0
ENSG00000235261	0	0	0	0
ENSG00000235262	0	0	0	0
ENSG00000235263	0	0	0	0
ENSG00000235264	0	0	0	0
ENSG00000235265	0	0	0	0
ENSG00000235266	0	0	0	0
ENSG00000235267	0	0	0	0
ENSG00000235268	0	0	0	0
ENSG00000235269	0	0	0	0
ENSG00000235271	0	0	0	0
ENSG00000235272	0	0	0	0
ENSG00000235273	0	0	0	0
ENSG00000235274	0	0	0	0
ENSG00000235275	0	0	0	0
ENSG00000235277	0	0	7	0
ENSG00000235278	0	0	0	0
ENSG00000235279	0	0	0	0
ENSG00000235280	0	0	0	0
ENSG00000235281	1	0	0	0
ENSG00000235282	0	0	0	0
ENSG00000235283	0	0	0	0
ENSG00000235284	0	0	0	0
ENSG00000235285	1	0	0	0
ENSG00000235286	0	0	0	0
ENSG00000235288	57	153	128	150
ENSG00000235289	0	0	0	0
ENSG00000235290	0	44	0	0
ENSG00000235291	0	0	0	0
ENSG00000235292	0	0	0	0
ENSG00000235293	0	0	0	0
ENSG00000235294	0	0	0	0
ENSG00000235295	0	0	0	0
ENSG00000235296	0	0	0	0
ENSG00000235297	0	0	0	0
ENSG00000235298	0	67	93	38
ENSG00000235299	0	0	0	0
ENSG00000235300	0	0	0	0
ENSG00000235301	0	0	0	0
ENSG00000235302	0	0	0	0
ENSG00000235303	0	0	0	0
ENSG00000235304	1	0	0	0
ENSG00000235305	0	0	0	0

ENSG00000235306	0	0	0	0
ENSG00000235307	0	0	0	0
ENSG00000235308	1	0	0	1
ENSG00000235309	0	0	0	0
ENSG00000235310	0	0	0	0
ENSG00000235312	0	0	0	0
ENSG00000235313	0	0	0	0
ENSG00000235314	2	0	2	0
ENSG00000235315	0	0	0	0
ENSG00000235316	0	0	0	0
ENSG00000235317	0	0	0	0
ENSG00000235318	0	0	0	0
ENSG00000235319	0	0	0	0
ENSG00000235321	0	0	0	0
ENSG00000235323	0	0	0	0
ENSG00000235324	0	0	0	0
ENSG00000235325	0	0	0	0
ENSG00000235326	1	98	56	134
ENSG00000235327	0	0	0	0
ENSG00000235328	0	0	0	0
ENSG00000235329	0	0	0	0
ENSG00000235330	0	0	0	0
ENSG00000235332	0	0	0	0
ENSG00000235333	0	0	0	0
ENSG00000235334	0	0	0	0
ENSG00000235335	0	0	0	0
ENSG00000235336	0	0	0	0
ENSG00000235337	0	0	0	0
ENSG00000235338	0	0	0	0
ENSG00000235339	0	0	0	0
ENSG00000235340	0	0	0	0
ENSG00000235342	0	0	0	0
ENSG00000235343	0	0	0	0
ENSG00000235344	0	0	0	0
ENSG00000235346	0	0	0	0
ENSG00000235347	0	1	16	7
ENSG00000235349	0	0	0	0
ENSG00000235350	0	0	0	0
ENSG00000235351	0	0	7	0
ENSG00000235352	0	0	0	0
ENSG00000235354	0	0	0	0
ENSG00000235355	0	0	0	0
ENSG00000235356	0	0	0	0
ENSG00000235357	0	0	0	0
ENSG00000235358	0	0	0	0
ENSG00000235360	0	0	0	0

ENSG00000235361	0	0	0	0
ENSG00000235363	1	0	0	0
ENSG00000235366	0	0	0	0
ENSG00000235368	0	0	0	0
ENSG00000235369	0	0	0	0
ENSG00000235370	0	0	0	0
ENSG00000235371	0	0	0	0
ENSG00000235372	0	0	0	0
ENSG00000235373	0	0	14	10
ENSG00000235374	3	22	2	2
ENSG00000235376	0	0	0	0
ENSG00000235377	0	0	0	0
ENSG00000235378	0	5	1	0
ENSG00000235379	0	0	0	0
ENSG00000235380	0	0	0	0
ENSG00000235381	0	0	0	0
ENSG00000235382	0	0	0	0
ENSG00000235385	0	0	0	0
ENSG00000235386	0	0	0	0
ENSG00000235387	0	0	0	0
ENSG00000235388	0	0	0	0
ENSG00000235389	0	0	0	0
ENSG00000235390	0	0	0	0
ENSG00000235391	0	0	0	0
ENSG00000235394	0	0	0	0
ENSG00000235396	0	0	0	0
ENSG00000235397	0	0	0	0
ENSG00000235398	3	0	1	0
ENSG00000235399	0	0	20	63
ENSG00000235400	0	0	0	0
ENSG00000235401	0	0	0	0
ENSG00000235403	0	0	0	0
ENSG00000235405	0	0	0	0
ENSG00000235407	0	0	0	0
ENSG00000235408	0	0	0	0
ENSG00000235410	0	0	0	0
ENSG00000235411	0	0	0	0
ENSG00000235412	0	0	0	0
ENSG00000235413	0	0	0	0
ENSG00000235414	0	0	0	0
ENSG00000235415	0	0	0	0
ENSG00000235416	0	0	0	0
ENSG00000235419	0	0	0	0
ENSG00000235420	0	0	0	0
ENSG00000235421	0	0	0	0
ENSG00000235422	0	0	0	0

ENSG00000235423	13	129	172	172
ENSG00000235424	0	0	0	0
ENSG00000235425	0	0	0	0
ENSG00000235426	0	0	0	0
ENSG00000235427	0	0	0	0
ENSG00000235428	0	0	0	0
ENSG00000235429	0	0	0	0
ENSG00000235430	0	0	0	0
ENSG00000235431	0	0	0	0
ENSG00000235432	0	0	0	0
ENSG00000235433	0	0	0	0
ENSG00000235434	0	0	0	0
ENSG00000235435	0	0	0	0
ENSG00000235436	0	0	0	0
ENSG00000235437	0	71	24	27
ENSG00000235438	0	0	0	0
ENSG00000235439	0	0	0	0
ENSG00000235440	0	0	0	0
ENSG00000235441	0	0	0	0
ENSG00000235443	0	0	0	0
ENSG00000235444	0	0	0	0
ENSG00000235445	0	0	0	0
ENSG00000235446	0	0	0	0
ENSG00000235447	0	0	0	0
ENSG00000235448	0	1	0	3
ENSG00000235449	0	0	0	0
ENSG00000235450	0	0	0	0
ENSG00000235451	0	0	0	0
ENSG00000235452	0	0	0	0
ENSG00000235453	457	379	415	866
ENSG00000235454	0	0	0	1
ENSG00000235455	0	0	0	0
ENSG00000235458	0	0	0	0
ENSG00000235459	0	0	0	0
ENSG00000235460	0	0	0	0
ENSG00000235461	0	0	0	0
ENSG00000235462	0	0	0	0
ENSG00000235463	0	0	0	0
ENSG00000235464	0	0	0	0
ENSG00000235467	0	0	0	0
ENSG00000235469	0	0	0	0
ENSG00000235470	0	0	0	0
ENSG00000235472	0	0	0	0
ENSG00000235475	0	0	3	3
ENSG00000235476	0	0	0	0
ENSG00000235477	0	10	4	10

ENSG00000235478	0	0	0	0
ENSG00000235479	0	0	0	0
ENSG00000235480	0	0	0	0
ENSG00000235481	0	0	0	0
ENSG00000235482	2	13	0	0
ENSG00000235483	0	0	0	0
ENSG00000235485	0	0	0	0
ENSG00000235486	0	0	0	0
ENSG00000235487	0	0	0	0
ENSG00000235488	31	17	40	23
ENSG00000235489	0	0	0	0
ENSG00000235491	0	0	0	0
ENSG00000235492	0	0	0	0
ENSG00000235493	0	103	47	33
ENSG00000235494	0	0	0	0
ENSG00000235495	0	0	0	0
ENSG00000235496	0	0	0	0
ENSG00000235497	0	0	0	0
ENSG00000235498	0	0	0	0
ENSG00000235499	0	0	0	0
ENSG00000235500	0	0	0	0
ENSG00000235501	0	0	0	0
ENSG00000235503	0	0	0	0
ENSG00000235504	0	0	0	0
ENSG00000235505	15	0	1	0
ENSG00000235506	0	0	0	0
ENSG00000235507	0	0	0	0
ENSG00000235508	2	0	0	0
ENSG00000235510	0	0	0	0
ENSG00000235511	0	0	0	0
ENSG00000235512	0	8	39	13
ENSG00000235513	181	756	416	545
ENSG00000235514	0	0	0	0
ENSG00000235516	0	0	0	0
ENSG00000235518	0	0	0	0
ENSG00000235519	0	0	0	0
ENSG00000235521	0	0	0	0
ENSG00000235522	0	37	32	23
ENSG00000235523	0	0	0	0
ENSG00000235524	0	0	0	0
ENSG00000235525	0	0	0	3
ENSG00000235526	0	0	0	0
ENSG00000235527	16	25	40	33
ENSG00000235528	0	0	0	0
ENSG00000235529	0	0	0	0
ENSG00000235530	1	0	0	1

ENSG00000235531	76	1647	972	905
ENSG00000235532	1	0	0	0
ENSG00000235533	0	0	0	0
ENSG00000235534	0	0	0	0
ENSG00000235535	0	0	0	0
ENSG00000235536	0	0	0	0
ENSG00000235537	0	0	0	0
ENSG00000235538	0	0	0	0
ENSG00000235539	0	0	0	0
ENSG00000235540	0	0	0	0
ENSG00000235541	0	0	0	0
ENSG00000235544	0	0	0	0
ENSG00000235545	1	0	4	4
ENSG00000235546	0	0	0	0
ENSG00000235547	0	0	0	0
ENSG00000235548	0	0	0	0
ENSG00000235549	0	0	0	0
ENSG00000235550	0	0	0	0
ENSG00000235552	5	12	1	3
ENSG00000235554	0	1	11	4
ENSG00000235555	0	0	0	0
ENSG00000235558	0	0	0	0
ENSG00000235559	0	0	0	0
ENSG00000235560	1	0	7	5
ENSG00000235561	0	0	0	0
ENSG00000235563	0	0	0	0
ENSG00000235564	0	0	0	0
ENSG00000235565	0	0	0	0
ENSG00000235566	0	0	0	0
ENSG00000235568	4	17	2	5
ENSG00000235569	0	0	0	0
ENSG00000235570	0	0	0	0
ENSG00000235571	0	0	0	0
ENSG00000235572	0	0	0	0
ENSG00000235573	0	0	0	0
ENSG00000235574	0	0	0	0
ENSG00000235575	0	0	0	0
ENSG00000235576	9	0	0	0
ENSG00000235577	0	0	0	0
ENSG00000235578	0	0	0	0
ENSG00000235579	0	0	0	0
ENSG00000235581	0	0	0	0
ENSG00000235582	0	0	0	0
ENSG00000235583	0	0	0	0
ENSG00000235584	0	0	0	0
ENSG00000235586	4	0	0	4

ENSG00000235587	0	0	0	0
ENSG00000235588	0	0	0	0
ENSG00000235589	0	0	0	0
ENSG00000235590	0	0	0	0
ENSG00000235592	0	0	0	0
ENSG00000235593	0	0	0	0
ENSG00000235594	0	0	0	0
ENSG00000235595	0	0	0	0
ENSG00000235596	0	0	0	0
ENSG00000235597	0	0	0	0
ENSG00000235598	0	0	0	0
ENSG00000235600	0	0	0	0
ENSG00000235601	0	0	0	0
ENSG00000235602	0	0	0	0
ENSG00000235604	0	0	0	0
ENSG00000235605	0	0	0	0
ENSG00000235606	0	0	0	0
ENSG00000235608	0	0	0	0
ENSG00000235609	93	1157	1379	1674
ENSG00000235610	0	0	0	0
ENSG00000235612	0	0	0	0
ENSG00000235613	1	0	0	1
ENSG00000235614	0	0	0	1
ENSG00000235615	0	0	0	1
ENSG00000235616	0	0	0	0
ENSG00000235617	0	0	0	0
ENSG00000235618	0	0	1	0
ENSG00000235619	0	0	0	0
ENSG00000235620	0	0	0	0
ENSG00000235621	0	0	0	0
ENSG00000235623	0	0	0	0
ENSG00000235625	0	0	0	0
ENSG00000235626	0	0	0	0
ENSG00000235627	0	0	0	0
ENSG00000235628	0	0	0	0
ENSG00000235629	0	0	0	0
ENSG00000235630	0	0	0	0
ENSG00000235631	0	0	3	0
ENSG00000235632	0	0	0	0
ENSG00000235635	0	0	1	0
ENSG00000235636	0	0	1	7
ENSG00000235637	0	0	0	0
ENSG00000235638	0	0	0	0
ENSG00000235639	0	0	0	0
ENSG00000235640	0	0	0	0
ENSG00000235641	0	0	0	0

ENSG00000235642	0	0	0	0
ENSG00000235643	0	0	0	0
ENSG00000235644	0	0	0	0
ENSG00000235645	0	0	0	0
ENSG00000235646	0	0	0	0
ENSG00000235647	0	0	0	0
ENSG00000235649	0	0	0	0
ENSG00000235650	0	0	0	0
ENSG00000235651	0	0	0	0
ENSG00000235652	2	33	88	71
ENSG00000235653	0	0	0	0
ENSG00000235655	727	9805	7126	6005
ENSG00000235656	0	0	0	0
ENSG00000235657	0	0	0	0
ENSG00000235658	0	0	0	0
ENSG00000235659	0	0	0	0
ENSG00000235660	0	0	0	0
ENSG00000235661	0	0	0	0
ENSG00000235663	0	0	0	0
ENSG00000235664	0	0	1	2
ENSG00000235665	0	0	1	0
ENSG00000235666	0	0	0	0
ENSG00000235667	0	0	0	0
ENSG00000235668	0	0	0	0
ENSG00000235669	0	0	0	0
ENSG00000235670	0	0	0	0
ENSG00000235672	0	0	0	0
ENSG00000235673	0	0	0	0
ENSG00000235674	0	1	3	0
ENSG00000235675	0	0	0	0
ENSG00000235676	0	0	0	0
ENSG00000235677	0	0	0	3
ENSG00000235678	0	0	0	0
ENSG00000235679	0	0	0	0
ENSG00000235680	0	0	0	0
ENSG00000235681	0	0	0	0
ENSG00000235683	0	0	0	0
ENSG00000235684	0	0	0	0
ENSG00000235685	0	0	0	0
ENSG00000235686	0	0	0	0
ENSG00000235687	0	0	0	0
ENSG00000235688	0	0	0	0
ENSG00000235689	0	0	0	0
ENSG00000235690	3	0	0	0
ENSG00000235691	0	0	0	0
ENSG00000235692	0	0	0	0

ENSG00000235695	0	0	0	0
ENSG00000235696	0	0	0	0
ENSG00000235697	0	0	0	0
ENSG00000235698	0	0	0	0
ENSG00000235699	0	0	0	0
ENSG00000235700	0	0	0	0
ENSG00000235701	0	0	0	0
ENSG00000235702	0	0	0	0
ENSG00000235703	7	14	17	42
ENSG00000235704	0	0	0	0
ENSG00000235705	0	0	0	0
ENSG00000235706	30	71	34	38
ENSG00000235707	0	0	0	0
ENSG00000235708	0	0	0	0
ENSG00000235710	0	0	0	0
ENSG00000235711	0	0	0	0
ENSG00000235712	0	0	0	0
ENSG00000235713	0	0	0	0
ENSG00000235714	0	0	0	0
ENSG00000235715	0	0	0	0
ENSG00000235716	0	0	0	0
ENSG00000235717	0	0	0	0
ENSG00000235718	0	0	0	0
ENSG00000235719	0	0	0	0
ENSG00000235720	0	0	5	9
ENSG00000235721	0	0	0	0
ENSG00000235722	0	0	0	0
ENSG00000235724	0	0	2	0
ENSG00000235725	0	0	0	0
ENSG00000235726	0	0	0	0
ENSG00000235727	0	0	0	0
ENSG00000235728	0	0	0	0
ENSG00000235729	0	0	0	0
ENSG00000235730	0	0	0	0
ENSG00000235731	0	0	0	0
ENSG00000235733	1	21	6	14
ENSG00000235734	0	0	0	0
ENSG00000235735	0	0	0	0
ENSG00000235736	0	0	0	0
ENSG00000235737	0	0	0	0
ENSG00000235738	0	0	0	0
ENSG00000235740	0	0	1	0
ENSG00000235741	0	0	0	0
ENSG00000235742	0	0	0	0
ENSG00000235743	0	22	0	10
ENSG00000235744	0	0	0	0

ENSG00000235746	0	0	0	0
ENSG00000235747	0	0	0	0
ENSG00000235748	0	0	0	1
ENSG00000235749	2	0	1	8
ENSG00000235750	0	0	0	0
ENSG00000235751	0	0	2	1
ENSG00000235754	0	0	0	0
ENSG00000235756	0	0	0	0
ENSG00000235757	0	0	0	0
ENSG00000235758	0	0	0	0
ENSG00000235759	0	0	0	0
ENSG00000235760	0	0	0	0
ENSG00000235761	0	0	0	0
ENSG00000235763	0	0	0	0
ENSG00000235764	0	0	0	0
ENSG00000235765	0	0	0	0
ENSG00000235768	0	0	0	0
ENSG00000235769	0	0	0	0
ENSG00000235770	4	0	0	27
ENSG00000235772	0	0	0	0
ENSG00000235773	0	0	0	0
ENSG00000235774	0	0	0	2
ENSG00000235775	0	0	0	0
ENSG00000235776	0	0	0	0
ENSG00000235777	0	0	0	2
ENSG00000235778	0	0	0	0
ENSG00000235779	0	0	0	0
ENSG00000235780	0	0	0	0
ENSG00000235781	46	728	1128	3026
ENSG00000235782	0	0	1	0
ENSG00000235784	0	0	0	0
ENSG00000235785	0	0	0	0
ENSG00000235786	0	0	0	0
ENSG00000235787	0	0	0	0
ENSG00000235788	0	0	0	0
ENSG00000235790	0	0	0	0
ENSG00000235794	0	0	0	0
ENSG00000235795	0	23	4	9
ENSG00000235797	0	0	0	0
ENSG00000235801	0	0	0	0
ENSG00000235802	23	138	46	39
ENSG00000235803	0	0	0	0
ENSG00000235804	0	0	0	0
ENSG00000235805	0	0	0	0
ENSG00000235806	1	0	2	0
ENSG00000235808	0	0	0	0

ENSG00000235809	0	0	0	0
ENSG00000235810	0	0	0	0
ENSG00000235811	0	0	0	0
ENSG00000235812	0	0	0	0
ENSG00000235813	0	0	0	0
ENSG00000235814	0	0	0	0
ENSG00000235815	0	0	0	0
ENSG00000235816	0	0	0	0
ENSG00000235817	0	0	0	0
ENSG00000235818	0	0	0	0
ENSG00000235819	0	0	3	6
ENSG00000235820	0	0	0	0
ENSG00000235821	0	0	0	0
ENSG00000235822	0	0	0	0
ENSG00000235823	1	0	0	0
ENSG00000235824	0	2	0	0
ENSG00000235825	0	0	0	0
ENSG00000235827	0	0	0	0
ENSG00000235828	0	0	0	0
ENSG00000235829	0	0	0	0
ENSG00000235830	0	0	0	0
ENSG00000235831	1	0	8	4
ENSG00000235832	0	0	0	0
ENSG00000235833	0	0	3	0
ENSG00000235834	0	0	0	0
ENSG00000235836	0	0	0	0
ENSG00000235837	0	0	3	0
ENSG00000235838	0	0	0	0
ENSG00000235840	0	0	0	0
ENSG00000235842	0	0	1	5
ENSG00000235843	0	0	0	0
ENSG00000235844	0	0	0	0
ENSG00000235845	0	0	0	0
ENSG00000235847	0	0	0	0
ENSG00000235848	0	142	33	9
ENSG00000235849	0	0	0	0
ENSG00000235852	0	0	1	0
ENSG00000235855	0	0	0	0
ENSG00000235857	0	0	0	0
ENSG00000235858	0	0	0	0
ENSG00000235859	44	46	43	55
ENSG00000235861	0	0	0	0
ENSG00000235862	0	0	0	0
ENSG00000235863	60	93	64	110
ENSG00000235864	0	0	0	0
ENSG00000235865	1	23	10	21

ENSG00000235868	0	0	0	0
ENSG00000235869	0	0	0	0
ENSG00000235871	0	0	0	0
ENSG00000235872	0	0	4	0
ENSG00000235875	0	0	0	0
ENSG00000235876	0	0	0	0
ENSG00000235877	0	0	0	0
ENSG00000235878	0	0	0	0
ENSG00000235879	0	0	0	0
ENSG00000235880	0	0	0	0
ENSG00000235881	0	0	0	0
ENSG00000235883	0	0	0	0
ENSG00000235884	2	0	0	0
ENSG00000235885	0	0	0	0
ENSG00000235886	0	0	0	0
ENSG00000235887	0	0	0	0
ENSG00000235888	5	0	0	0
ENSG00000235890	0	0	0	0
ENSG00000235892	0	0	0	0
ENSG00000235893	1	0	0	0
ENSG00000235895	0	0	0	0
ENSG00000235896	0	0	0	0
ENSG00000235897	0	0	3	4
ENSG00000235899	0	0	0	0
ENSG00000235901	0	0	0	0
ENSG00000235902	0	0	0	0
ENSG00000235903	0	0	0	0
ENSG00000235904	0	0	0	0
ENSG00000235905	0	0	0	0
ENSG00000235907	0	0	0	0
ENSG00000235908	0	0	0	0
ENSG00000235910	0	0	0	1
ENSG00000235911	0	0	0	0
ENSG00000235912	1	1	0	1
ENSG00000235914	0	0	0	0
ENSG00000235915	0	0	0	0
ENSG00000235916	0	0	0	0
ENSG00000235917	0	0	0	0
ENSG00000235919	5	18	7	13
ENSG00000235920	0	0	0	0
ENSG00000235922	0	0	0	0
ENSG00000235923	0	0	0	0
ENSG00000235924	0	0	0	0
ENSG00000235925	0	0	0	0
ENSG00000235926	0	0	0	0
ENSG00000235927	222	161	134	331

ENSG00000235929	0	0	0	0
ENSG00000235930	0	0	0	0
ENSG00000235931	0	0	0	0
ENSG00000235932	0	0	0	0
ENSG00000235933	0	0	0	0
ENSG00000235934	0	0	0	0
ENSG00000235937	0	0	0	0
ENSG00000235938	0	0	0	0
ENSG00000235939	0	0	0	0
ENSG00000235940	0	0	0	0
ENSG00000235941	0	0	0	0
ENSG00000235942	0	0	0	0
ENSG00000235943	0	0	0	0
ENSG00000235944	3	24	4	2
ENSG00000235945	0	0	0	0
ENSG00000235946	0	0	1	0
ENSG00000235947	0	0	0	0
ENSG00000235949	0	0	0	0
ENSG00000235950	0	0	0	0
ENSG00000235951	0	0	0	0
ENSG00000235952	0	0	0	0
ENSG00000235954	201	298	409	544
ENSG00000235955	0	0	0	0
ENSG00000235956	0	0	0	0
ENSG00000235957	0	0	0	0
ENSG00000235958	0	0	2	0
ENSG00000235959	0	0	0	0
ENSG00000235960	0	0	0	0
ENSG00000235961	0	0	0	0
ENSG00000235962	0	0	0	0
ENSG00000235963	0	0	0	0
ENSG00000235964	0	0	0	0
ENSG00000235965	0	0	0	0
ENSG00000235966	0	0	0	0
ENSG00000235967	1	0	0	0
ENSG00000235968	0	0	0	0
ENSG00000235969	0	0	0	0
ENSG00000235972	0	0	0	0
ENSG00000235974	0	0	0	0
ENSG00000235975	0	0	0	0
ENSG00000235976	0	0	0	0
ENSG00000235978	2	0	9	7
ENSG00000235979	0	0	0	0
ENSG00000235981	0	0	0	0
ENSG00000235982	0	0	0	0
ENSG00000235984	0	0	0	0

ENSG00000235985	0	0	0	0
ENSG00000235986	0	0	0	0
ENSG00000235988	0	0	0	0
ENSG00000235989	1	0	0	0
ENSG00000235990	0	0	0	0
ENSG00000235991	0	0	0	0
ENSG00000235992	0	0	0	0
ENSG00000235993	0	0	0	0
ENSG00000235994	0	0	0	0
ENSG00000235995	0	0	0	0
ENSG00000235996	0	0	1	4
ENSG00000235997	0	0	0	0
ENSG00000235998	0	0	0	0
ENSG00000235999	4	0	0	5
ENSG00000236002	0	0	0	0
ENSG00000236003	0	0	0	0
ENSG00000236004	0	0	0	0
ENSG00000236005	0	0	0	0
ENSG00000236006	0	0	0	0
ENSG00000236007	0	0	0	0
ENSG00000236008	23	125	202	198
ENSG00000236009	0	0	0	0
ENSG00000236011	0	0	0	0
ENSG00000236012	0	0	0	0
ENSG00000236013	0	0	0	0
ENSG00000236014	0	0	0	0
ENSG00000236015	0	0	0	0
ENSG00000236016	0	0	0	0
ENSG00000236017	0	15	0	0
ENSG00000236018	0	0	0	0
ENSG00000236019	0	0	0	0
ENSG00000236021	1	5	21	72
ENSG00000236022	0	0	0	0
ENSG00000236024	0	0	0	0
ENSG00000236025	0	0	0	0
ENSG00000236026	0	0	0	0
ENSG00000236027	0	0	0	0
ENSG00000236028	0	0	0	0
ENSG00000236029	0	0	0	0
ENSG00000236030	0	185	158	302
ENSG00000236031	0	11	71	24
ENSG00000236032	0	0	0	0
ENSG00000236033	0	0	0	0
ENSG00000236035	0	0	0	0
ENSG00000236036	0	0	0	0
ENSG00000236039	0	0	0	0

ENSG00000236040	0	0	0	0
ENSG00000236041	0	0	0	0
ENSG00000236042	0	0	0	0
ENSG00000236044	0	0	0	0
ENSG00000236045	0	0	0	0
ENSG00000236046	0	0	0	0
ENSG00000236047	0	0	0	0
ENSG00000236048	0	0	0	0
ENSG00000236049	0	5	0	0
ENSG00000236051	1	70	50	34
ENSG00000236052	0	0	0	0
ENSG00000236053	0	0	0	0
ENSG00000236054	0	0	0	0
ENSG00000236055	0	0	0	0
ENSG00000236056	1	5	0	0
ENSG00000236058	1	0	0	0
ENSG00000236060	0	0	0	1
ENSG00000236062	0	0	0	0
ENSG00000236063	0	0	0	0
ENSG00000236064	0	0	0	0
ENSG00000236065	0	0	0	0
ENSG00000236066	0	0	0	0
ENSG00000236068	0	0	0	0
ENSG00000236069	0	0	0	0
ENSG00000236070	0	0	0	0
ENSG00000236072	0	0	0	0
ENSG00000236073	0	27	38	137
ENSG00000236075	0	0	0	0
ENSG00000236076	0	0	1	0
ENSG00000236077	0	0	0	0
ENSG00000236078	18	26	26	39
ENSG00000236079	0	0	0	0
ENSG00000236080	0	0	0	0
ENSG00000236081	2	0	0	0
ENSG00000236082	0	0	0	0
ENSG00000236083	0	0	0	0
ENSG00000236085	0	0	0	0
ENSG00000236086	0	0	0	0
ENSG00000236088	1	17	20	18
ENSG00000236090	0	0	0	0
ENSG00000236091	0	0	0	0
ENSG00000236093	0	0	0	0
ENSG00000236094	0	0	0	0
ENSG00000236095	0	0	0	0
ENSG00000236097	0	0	0	0
ENSG00000236098	0	0	0	0

ENSG00000236099	0	0	0	0
ENSG00000236100	0	0	0	0
ENSG00000236101	0	0	0	0
ENSG00000236102	0	0	0	0
ENSG00000236104	2	0	9	6
ENSG00000236105	0	0	0	0
ENSG00000236106	0	0	0	0
ENSG00000236107	0	0	0	0
ENSG00000236108	0	0	0	0
ENSG00000236109	0	0	0	0
ENSG00000236110	0	0	0	0
ENSG00000236111	1	0	0	0
ENSG00000236113	0	0	0	0
ENSG00000236114	0	0	1	0
ENSG00000236115	0	0	0	0
ENSG00000236116	1	0	0	0
ENSG00000236117	0	0	0	0
ENSG00000236118	0	0	0	0
ENSG00000236119	0	0	0	0
ENSG00000236120	1	0	1	0
ENSG00000236121	0	0	0	0
ENSG00000236122	0	0	0	0
ENSG00000236123	0	0	0	0
ENSG00000236124	0	0	0	0
ENSG00000236125	0	0	0	0
ENSG00000236126	0	0	0	0
ENSG00000236129	0	0	0	0
ENSG00000236130	0	0	0	0
ENSG00000236131	0	0	0	0
ENSG00000236132	0	0	0	0
ENSG00000236133	0	0	0	0
ENSG00000236136	0	0	0	0
ENSG00000236137	1	0	4	6
ENSG00000236138	0	0	0	0
ENSG00000236140	0	0	0	0
ENSG00000236141	0	0	0	0
ENSG00000236144	0	0	0	0
ENSG00000236145	0	0	0	0
ENSG00000236146	0	0	0	0
ENSG00000236148	30	35	47	52
ENSG00000236149	0	0	0	0
ENSG00000236151	0	0	0	0
ENSG00000236152	0	0	0	0
ENSG00000236153	0	0	0	0
ENSG00000236154	0	0	0	0
ENSG00000236155	0	0	0	0

ENSG00000236156	0	0	0	0
ENSG00000236157	0	0	0	0
ENSG00000236158	0	0	0	0
ENSG00000236159	0	0	0	0
ENSG00000236160	0	0	0	0
ENSG00000236162	0	0	0	0
ENSG00000236164	0	0	0	0
ENSG00000236165	0	0	0	0
ENSG00000236166	1	434	298	554
ENSG00000236167	0	0	0	0
ENSG00000236168	0	0	0	0
ENSG00000236169	0	0	0	0
ENSG00000236170	0	0	0	0
ENSG00000236171	0	0	0	0
ENSG00000236172	0	0	0	0
ENSG00000236173	0	0	0	0
ENSG00000236175	0	0	0	0
ENSG00000236176	0	0	0	0
ENSG00000236177	0	0	0	0
ENSG00000236178	0	0	0	0
ENSG00000236179	0	0	0	0
ENSG00000236180	0	0	0	0
ENSG00000236182	0	0	0	0
ENSG00000236183	0	0	0	0
ENSG00000236184	0	22	0	1
ENSG00000236185	0	0	0	0
ENSG00000236187	0	0	0	0
ENSG00000236188	0	0	0	0
ENSG00000236189	0	0	0	0
ENSG00000236190	0	0	0	0
ENSG00000236191	0	0	0	0
ENSG00000236193	0	0	0	0
ENSG00000236194	0	0	3	18
ENSG00000236195	0	0	0	0
ENSG00000236196	0	0	0	0
ENSG00000236197	0	0	0	0
ENSG00000236198	0	0	0	0
ENSG00000236199	11	0	6	5
ENSG00000236200	9	33	27	0
ENSG00000236202	0	0	0	0
ENSG00000236203	0	0	0	0
ENSG00000236204	0	0	1	3
ENSG00000236205	0	0	0	0
ENSG00000236206	0	0	0	0
ENSG00000236208	0	0	0	0
ENSG00000236209	0	0	0	0

ENSG00000236210	0	0	0	0
ENSG00000236211	0	0	0	0
ENSG00000236212	0	0	0	0
ENSG00000236213	5	0	3	5
ENSG00000236216	0	0	0	0
ENSG00000236217	0	0	0	0
ENSG00000236221	0	0	0	0
ENSG00000236222	0	0	0	0
ENSG00000236225	0	0	0	0
ENSG00000236226	0	0	0	0
ENSG00000236227	0	0	0	0
ENSG00000236229	0	0	0	0
ENSG00000236230	2	0	0	0
ENSG00000236231	0	0	0	0
ENSG00000236232	0	0	0	0
ENSG00000236233	0	0	0	0
ENSG00000236234	0	0	0	0
ENSG00000236235	0	0	0	0
ENSG00000236236	0	0	0	0
ENSG00000236237	0	0	0	0
ENSG00000236238	0	0	0	0
ENSG00000236240	0	0	0	0
ENSG00000236241	0	0	0	0
ENSG00000236242	0	0	0	0
ENSG00000236243	0	0	0	0
ENSG00000236244	0	0	0	0
ENSG00000236246	0	0	0	0
ENSG00000236247	0	0	0	0
ENSG00000236248	0	0	0	0
ENSG00000236249	0	0	0	0
ENSG00000236250	0	0	0	0
ENSG00000236251	0	0	0	0
ENSG00000236252	0	0	0	0
ENSG00000236253	0	0	0	0
ENSG00000236254	0	0	1	2
ENSG00000236255	0	0	0	0
ENSG00000236256	0	0	0	0
ENSG00000236257	0	0	0	0
ENSG00000236258	0	0	0	0
ENSG00000236259	0	0	0	0
ENSG00000236261	0	0	0	0
ENSG00000236262	0	0	0	0
ENSG00000236263	0	0	0	0
ENSG00000236264	0	0	0	0
ENSG00000236265	0	0	0	0
ENSG00000236266	2	0	20	19

ENSG00000236267	0	0	0	0
ENSG00000236268	0	0	0	0
ENSG00000236269	0	0	0	1
ENSG00000236271	0	0	0	0
ENSG00000236272	0	0	0	0
ENSG00000236273	0	0	0	0
ENSG00000236274	0	0	0	0
ENSG00000236276	0	0	0	0
ENSG00000236277	0	0	0	0
ENSG00000236278	0	0	0	0
ENSG00000236279	50	206	141	167
ENSG00000236280	0	0	0	0
ENSG00000236281	0	0	0	0
ENSG00000236283	0	0	0	0
ENSG00000236284	0	0	0	0
ENSG00000236285	0	0	0	0
ENSG00000236287	75	142	152	168
ENSG00000236289	0	0	0	0
ENSG00000236290	0	0	0	0
ENSG00000236292	0	0	0	0
ENSG00000236294	0	0	0	0
ENSG00000236295	0	0	0	0
ENSG00000236296	0	0	11	22
ENSG00000236297	1	4	1	4
ENSG00000236299	0	0	0	0
ENSG00000236300	0	0	0	0
ENSG00000236301	0	0	0	0
ENSG00000236303	0	0	0	0
ENSG00000236304	17941	30231	19627	26910
ENSG00000236305	1	0	0	0
ENSG00000236306	0	0	0	0
ENSG00000236307	0	0	0	0
ENSG00000236308	0	0	0	0
ENSG00000236309	0	0	0	1
ENSG00000236310	0	0	1	3
ENSG00000236311	0	0	0	0
ENSG00000236312	0	0	0	0
ENSG00000236313	0	0	0	0
ENSG00000236315	0	0	0	0
ENSG00000236316	33	57	8	34
ENSG00000236317	0	0	0	0
ENSG00000236318	0	0	6	0
ENSG00000236319	0	0	0	0
ENSG00000236320	442	4632	5908	3310
ENSG00000236322	0	0	0	0
ENSG00000236323	0	0	0	0

ENSG00000236324	0	0	0	0
ENSG00000236325	0	0	0	0
ENSG00000236326	0	6	3	0
ENSG00000236327	0	0	0	0
ENSG00000236330	0	0	0	0
ENSG00000236332	0	0	0	0
ENSG00000236333	7	93	55	67
ENSG00000236334	0	0	0	0
ENSG00000236335	0	0	0	0
ENSG00000236336	0	0	0	0
ENSG00000236337	2	0	0	2
ENSG00000236338	0	2232	3657	1396
ENSG00000236339	0	0	0	0
ENSG00000236340	0	0	0	0
ENSG00000236341	0	0	0	0
ENSG00000236342	0	0	0	0
ENSG00000236343	0	0	0	0
ENSG00000236345	1	0	0	0
ENSG00000236346	0	0	0	0
ENSG00000236347	0	0	0	0
ENSG00000236348	0	0	0	0
ENSG00000236349	0	0	0	0
ENSG00000236352	0	0	2	0
ENSG00000236353	0	0	0	0
ENSG00000236354	0	0	0	0
ENSG00000236355	0	0	0	0
ENSG00000236356	0	0	0	0
ENSG00000236357	0	0	0	0
ENSG00000236358	0	0	0	0
ENSG00000236359	0	0	0	0
ENSG00000236360	0	0	0	1
ENSG00000236361	0	0	0	0
ENSG00000236362	0	0	0	0
ENSG00000236364	0	0	0	0
ENSG00000236365	0	0	0	0
ENSG00000236366	0	0	0	0
ENSG00000236369	0	0	0	0
ENSG00000236370	0	0	2	2
ENSG00000236371	0	0	0	0
ENSG00000236372	0	0	0	0
ENSG00000236373	0	0	0	0
ENSG00000236374	0	0	0	0
ENSG00000236375	0	0	0	0
ENSG00000236376	0	0	0	0
ENSG00000236377	1	0	0	0
ENSG00000236378	0	0	0	0

ENSG00000236379	0	0	0	0
ENSG00000236380	0	0	0	0
ENSG00000236382	0	0	0	0
ENSG00000236383	0	0	1	3
ENSG00000236384	0	0	0	0
ENSG00000236385	0	0	0	0
ENSG00000236386	0	0	0	0
ENSG00000236387	0	0	0	0
ENSG00000236388	0	0	0	0
ENSG00000236389	0	0	0	0
ENSG00000236390	0	0	0	0
ENSG00000236391	0	0	0	0
ENSG00000236392	0	0	0	0
ENSG00000236393	0	0	0	0
ENSG00000236394	0	0	0	0
ENSG00000236395	0	0	0	0
ENSG00000236396	0	0	0	0
ENSG00000236397	382	660	205	696
ENSG00000236398	0	0	0	0
ENSG00000236399	0	0	0	0
ENSG00000236400	0	0	0	0
ENSG00000236401	0	0	0	0
ENSG00000236403	0	0	0	0
ENSG00000236404	0	0	0	0
ENSG00000236405	0	0	0	0
ENSG00000236407	0	0	0	0
ENSG00000236408	0	0	0	0
ENSG00000236409	0	21	0	0
ENSG00000236411	24	136	75	126
ENSG00000236412	0	0	0	0
ENSG00000236413	0	0	0	0
ENSG00000236414	0	0	0	0
ENSG00000236415	0	0	0	0
ENSG00000236417	0	0	0	0
ENSG00000236418	0	0	0	0
ENSG00000236420	0	0	0	0
ENSG00000236422	0	0	0	0
ENSG00000236423	22	9	18	26
ENSG00000236424	0	0	0	0
ENSG00000236425	0	0	0	0
ENSG00000236426	2	0	0	0
ENSG00000236427	0	0	0	0
ENSG00000236428	0	0	0	0
ENSG00000236429	0	0	0	0
ENSG00000236430	0	0	0	0
ENSG00000236431	0	0	0	0

ENSG00000236432	0	0	2	2
ENSG00000236433	0	0	0	0
ENSG00000236434	0	0	0	0
ENSG00000236435	0	0	0	0
ENSG00000236436	0	0	0	0
ENSG00000236437	0	0	0	0
ENSG00000236438	10	26	27	24
ENSG00000236439	4	2	2	4
ENSG00000236440	0	0	0	0
ENSG00000236441	0	0	0	0
ENSG00000236442	0	0	0	0
ENSG00000236443	0	0	0	0
ENSG00000236444	0	0	0	1
ENSG00000236445	0	0	0	0
ENSG00000236446	0	0	0	0
ENSG00000236447	0	0	0	0
ENSG00000236449	0	0	0	0
ENSG00000236450	0	0	0	0
ENSG00000236451	0	0	0	0
ENSG00000236452	0	0	2	0
ENSG00000236453	0	21	5	2
ENSG00000236455	0	0	0	0
ENSG00000236456	0	0	0	0
ENSG00000236457	0	1	0	0
ENSG00000236459	0	0	0	0
ENSG00000236460	0	0	0	0
ENSG00000236461	0	0	0	0
ENSG00000236463	0	0	0	0
ENSG00000236464	0	0	0	0
ENSG00000236466	0	0	0	0
ENSG00000236467	0	0	0	0
ENSG00000236468	0	0	0	0
ENSG00000236469	0	0	0	0
ENSG00000236471	0	0	0	3
ENSG00000236472	0	0	0	0
ENSG00000236473	0	0	0	0
ENSG00000236474	0	0	0	0
ENSG00000236475	0	0	0	0
ENSG00000236476	0	0	0	0
ENSG00000236477	0	0	0	0
ENSG00000236478	0	0	0	0
ENSG00000236480	0	0	1	0
ENSG00000236481	0	0	0	0
ENSG00000236483	0	0	0	0
ENSG00000236484	0	0	0	0
ENSG00000236485	0	12	0	0

ENSG00000236487	0	0	0	0
ENSG00000236488	0	0	0	0
ENSG00000236489	0	0	0	0
ENSG00000236490	0	0	0	0
ENSG00000236491	0	0	0	0
ENSG00000236492	0	0	0	0
ENSG00000236493	1	12	6	3
ENSG00000236494	0	0	0	0
ENSG00000236495	0	0	0	0
ENSG00000236496	0	0	0	0
ENSG00000236497	0	0	0	0
ENSG00000236498	0	0	0	0
ENSG00000236499	0	0	0	0
ENSG00000236500	0	0	0	0
ENSG00000236501	0	0	3	9
ENSG00000236502	0	0	0	0
ENSG00000236503	0	0	0	0
ENSG00000236504	0	0	0	0
ENSG00000236505	0	0	0	0
ENSG00000236507	0	0	0	0
ENSG00000236508	8	118	19	66
ENSG00000236509	0	0	0	0
ENSG00000236510	0	0	0	0
ENSG00000236511	0	0	0	0
ENSG00000236512	1	0	0	0
ENSG00000236513	0	0	0	0
ENSG00000236514	0	0	0	0
ENSG00000236515	0	0	0	0
ENSG00000236516	0	0	0	0
ENSG00000236518	0	0	0	0
ENSG00000236519	1	0	0	0
ENSG00000236520	0	0	0	0
ENSG00000236521	0	0	0	0
ENSG00000236522	0	0	0	0
ENSG00000236523	0	0	0	0
ENSG00000236524	0	0	0	0
ENSG00000236525	0	0	0	0
ENSG00000236526	0	0	7	0
ENSG00000236527	0	0	0	0
ENSG00000236528	4	51	21	17
ENSG00000236529	0	0	0	0
ENSG00000236530	0	49	70	25
ENSG00000236531	0	0	0	0
ENSG00000236532	0	0	0	0
ENSG00000236533	0	0	0	0
ENSG00000236534	12	1	1	0

ENSG00000236535	0	0	0	0
ENSG00000236536	0	0	0	0
ENSG00000236537	4	11	21	31
ENSG00000236538	0	0	0	0
ENSG00000236539	0	0	0	0
ENSG00000236540	22	0	14	0
ENSG00000236541	0	0	0	0
ENSG00000236542	0	0	0	0
ENSG00000236543	0	0	0	0
ENSG00000236544	0	0	0	0
ENSG00000236545	0	0	0	0
ENSG00000236546	0	0	0	0
ENSG00000236547	0	0	0	0
ENSG00000236548	3	484	378	364
ENSG00000236549	0	0	0	0
ENSG00000236550	0	0	0	0
ENSG00000236552	8	0	0	3
ENSG00000236554	0	0	0	0
ENSG00000236555	0	0	0	0
ENSG00000236556	0	0	0	0
ENSG00000236557	1	8	0	2
ENSG00000236558	0	0	0	0
ENSG00000236559	0	0	0	0
ENSG00000236560	0	0	0	0
ENSG00000236561	0	0	0	0
ENSG00000236562	0	0	0	0
ENSG00000236564	0	0	0	0
ENSG00000236565	0	0	2	0
ENSG00000236567	0	0	0	0
ENSG00000236569	0	0	0	0
ENSG00000236570	0	0	0	0
ENSG00000236571	0	0	0	0
ENSG00000236572	0	0	0	0
ENSG00000236574	0	0	0	0
ENSG00000236575	0	0	0	0
ENSG00000236576	0	0	0	0
ENSG00000236577	0	0	0	0
ENSG00000236579	0	0	0	0
ENSG00000236580	0	0	0	0
ENSG00000236581	0	0	0	0
ENSG00000236582	0	0	0	0
ENSG00000236583	0	0	0	0
ENSG00000236584	0	0	0	0
ENSG00000236587	0	0	0	0
ENSG00000236590	0	0	0	0
ENSG00000236591	1	0	1	5

ENSG00000236592	0	0	0	0
ENSG00000236594	0	0	0	0
ENSG00000236595	0	0	0	0
ENSG00000236596	0	0	0	0
ENSG00000236597	0	0	0	0
ENSG00000236598	0	0	0	0
ENSG00000236599	0	0	0	0
ENSG00000236601	0	0	0	0
ENSG00000236603	0	0	0	0
ENSG00000236604	0	0	0	0
ENSG00000236605	0	0	0	0
ENSG00000236606	0	0	0	0
ENSG00000236607	0	0	0	0
ENSG00000236608	0	0	0	0
ENSG00000236609	3	0	0	0
ENSG00000236610	0	0	0	0
ENSG00000236611	0	0	0	0
ENSG00000236612	0	0	0	0
ENSG00000236614	0	0	0	0
ENSG00000236615	0	0	0	0
ENSG00000236616	0	0	0	0
ENSG00000236617	3	0	1	2
ENSG00000236618	0	0	1	0
ENSG00000236619	0	0	0	0
ENSG00000236620	0	0	0	0
ENSG00000236621	0	0	0	0
ENSG00000236624	1	0	5	7
ENSG00000236625	0	0	0	0
ENSG00000236626	0	0	0	0
ENSG00000236627	0	0	0	0
ENSG00000236629	0	0	0	0
ENSG00000236632	0	0	0	0
ENSG00000236634	0	0	0	0
ENSG00000236635	2	0	0	0
ENSG00000236636	0	0	0	0
ENSG00000236637	0	0	0	0
ENSG00000236638	0	0	0	0
ENSG00000236641	0	0	0	0
ENSG00000236642	0	0	0	0
ENSG00000236643	0	0	0	0
ENSG00000236646	0	0	0	0
ENSG00000236647	0	0	0	0
ENSG00000236648	0	0	0	0
ENSG00000236649	0	0	0	0
ENSG00000236650	0	0	0	0
ENSG00000236651	0	0	0	0

ENSG00000236653	41	187	86	279
ENSG00000236654	0	0	0	0
ENSG00000236655	0	0	0	0
ENSG00000236656	0	0	0	0
ENSG00000236657	0	0	0	0
ENSG00000236658	0	0	0	0
ENSG00000236660	0	0	0	0
ENSG00000236662	0	0	0	0
ENSG00000236663	0	0	0	0
ENSG00000236664	0	0	0	0
ENSG00000236665	0	0	0	0
ENSG00000236666	0	0	0	0
ENSG00000236667	0	0	0	0
ENSG00000236668	0	0	0	0
ENSG00000236669	0	0	0	0
ENSG00000236670	0	0	0	0
ENSG00000236671	3	146	90	127
ENSG00000236672	0	0	0	0
ENSG00000236673	0	0	1	0
ENSG00000236674	0	0	0	0
ENSG00000236675	0	0	0	0
ENSG00000236676	0	0	3	0
ENSG00000236677	0	0	0	0
ENSG00000236678	0	0	0	0
ENSG00000236679	0	0	2	0
ENSG00000236680	0	7	0	0
ENSG00000236681	0	0	0	0
ENSG00000236682	2	0	0	9
ENSG00000236683	0	0	0	0
ENSG00000236684	0	0	0	0
ENSG00000236686	0	0	0	0
ENSG00000236687	0	0	0	0
ENSG00000236688	0	0	0	0
ENSG00000236689	0	0	0	5
ENSG00000236690	0	0	0	0
ENSG00000236691	0	0	0	0
ENSG00000236692	0	0	0	0
ENSG00000236693	0	0	0	0
ENSG00000236695	0	0	0	0
ENSG00000236697	0	0	0	0
ENSG00000236698	0	0	1	0
ENSG00000236699	0	0	0	0
ENSG00000236700	0	0	0	0
ENSG00000236701	0	0	0	0
ENSG00000236703	0	0	2	0
ENSG00000236704	0	0	0	0

ENSG00000236706	0	0	0	0
ENSG00000236708	0	0	0	0
ENSG00000236709	0	0	0	0
ENSG00000236710	0	0	0	0
ENSG00000236711	0	0	0	0
ENSG00000236712	0	0	0	0
ENSG00000236713	0	0	0	3
ENSG00000236714	0	0	0	0
ENSG00000236716	0	0	0	0
ENSG00000236717	0	0	0	0
ENSG00000236718	0	0	0	0
ENSG00000236719	0	0	0	0
ENSG00000236720	0	0	0	0
ENSG00000236722	0	0	0	0
ENSG00000236723	0	0	0	0
ENSG00000236724	0	0	0	0
ENSG00000236727	0	0	0	0
ENSG00000236729	0	0	0	0
ENSG00000236731	0	0	0	0
ENSG00000236732	0	0	0	0
ENSG00000236733	0	0	1	0
ENSG00000236734	0	0	0	0
ENSG00000236735	0	0	0	0
ENSG00000236736	0	0	0	0
ENSG00000236737	0	0	0	0
ENSG00000236739	0	0	0	0
ENSG00000236740	1	0	0	0
ENSG00000236741	0	0	0	0
ENSG00000236742	0	0	0	0
ENSG00000236743	0	0	0	0
ENSG00000236744	0	0	4	0
ENSG00000236745	0	0	0	0
ENSG00000236746	0	0	0	0
ENSG00000236747	0	0	0	0
ENSG00000236748	0	0	0	0
ENSG00000236750	0	0	0	0
ENSG00000236751	0	0	2	0
ENSG00000236753	3	0	13	5
ENSG00000236754	0	0	0	1
ENSG00000236756	0	18	4	1
ENSG00000236757	0	0	0	0
ENSG00000236758	0	0	0	0
ENSG00000236759	0	0	0	0
ENSG00000236760	0	0	0	0
ENSG00000236761	0	0	0	0
ENSG00000236762	0	0	0	0

ENSG00000236763	0	0	0	0
ENSG00000236764	0	0	0	0
ENSG00000236765	0	0	0	0
ENSG00000236766	0	0	0	0
ENSG00000236768	0	0	0	0
ENSG00000236769	0	0	0	0
ENSG00000236770	0	0	0	0
ENSG00000236771	0	0	0	0
ENSG00000236772	0	0	0	5
ENSG00000236773	0	0	0	0
ENSG00000236775	0	0	0	0
ENSG00000236776	0	0	0	0
ENSG00000236777	0	0	0	0
ENSG00000236778	45	9	46	64
ENSG00000236779	0	0	0	0
ENSG00000236780	0	0	2	0
ENSG00000236782	0	0	0	0
ENSG00000236783	0	0	0	1
ENSG00000236785	0	0	0	0
ENSG00000236786	0	0	0	0
ENSG00000236787	0	0	0	0
ENSG00000236790	1	359	382	925
ENSG00000236791	0	0	0	0
ENSG00000236792	0	0	0	0
ENSG00000236794	0	0	0	0
ENSG00000236795	0	0	0	0
ENSG00000236796	0	0	0	0
ENSG00000236797	0	0	0	0
ENSG00000236798	0	0	0	0
ENSG00000236799	0	0	0	0
ENSG00000236800	0	0	0	0
ENSG00000236801	0	0	0	0
ENSG00000236802	0	0	0	0
ENSG00000236803	0	0	0	0
ENSG00000236804	0	0	0	0
ENSG00000236806	0	0	0	0
ENSG00000236807	0	0	0	0
ENSG00000236808	0	0	0	0
ENSG00000236809	0	0	1	0
ENSG00000236810	2	0	12	14
ENSG00000236811	0	0	0	0
ENSG00000236813	0	0	0	0
ENSG00000236814	0	0	0	0
ENSG00000236816	0	0	49	125
ENSG00000236817	0	0	17	1
ENSG00000236818	0	0	0	0

ENSG00000236819	0	0	0	0
ENSG00000236822	0	0	0	0
ENSG00000236823	13	0	8	0
ENSG00000236824	8	175	150	397
ENSG00000236825	0	0	0	0
ENSG00000236826	0	0	0	0
ENSG00000236827	0	0	0	0
ENSG00000236828	0	0	0	0
ENSG00000236829	0	0	0	0
ENSG00000236830	2	0	0	0
ENSG00000236831	0	0	0	0
ENSG00000236832	0	0	0	0
ENSG00000236833	0	0	0	0
ENSG00000236834	0	0	0	0
ENSG00000236835	0	0	0	0
ENSG00000236836	0	0	0	0
ENSG00000236837	0	0	0	0
ENSG00000236838	0	0	0	0
ENSG00000236839	0	0	0	0
ENSG00000236841	0	0	0	0
ENSG00000236842	0	0	0	0
ENSG00000236843	0	0	0	0
ENSG00000236844	0	0	0	0
ENSG00000236846	0	0	0	0
ENSG00000236847	0	0	0	0
ENSG00000236848	0	0	0	0
ENSG00000236849	0	0	0	0
ENSG00000236850	24	36	83	149
ENSG00000236851	0	0	0	0
ENSG00000236852	0	0	0	0
ENSG00000236853	13	0	15	12
ENSG00000236854	0	0	0	0
ENSG00000236856	0	0	0	0
ENSG00000236857	0	0	0	0
ENSG00000236858	0	0	0	0
ENSG00000236859	74	70	45	114
ENSG00000236860	0	0	0	0
ENSG00000236861	5	0	3	8
ENSG00000236862	0	0	0	0
ENSG00000236863	0	0	0	0
ENSG00000236864	0	0	0	0
ENSG00000236866	0	17	7	9
ENSG00000236867	0	0	0	0
ENSG00000236869	0	0	0	0
ENSG00000236870	0	0	0	0
ENSG00000236871	0	0	0	0

ENSG00000236872	0	0	0	0
ENSG00000236873	0	0	0	0
ENSG00000236874	0	0	0	0
ENSG00000236875	198	621	147	290
ENSG00000236876	21	14	13	20
ENSG00000236877	0	0	0	0
ENSG00000236878	0	0	0	0
ENSG00000236880	0	0	0	0
ENSG00000236881	0	0	0	0
ENSG00000236882	0	0	0	0
ENSG00000236883	0	0	0	0
ENSG00000236884	0	0	0	0
ENSG00000236885	0	0	0	0
ENSG00000236886	7	0	0	0
ENSG00000236887	0	0	0	0
ENSG00000236888	0	0	0	0
ENSG00000236889	0	0	0	0
ENSG00000236890	0	0	0	0
ENSG00000236892	0	0	0	0
ENSG00000236893	0	0	0	0
ENSG00000236894	0	0	0	0
ENSG00000236895	0	0	0	0
ENSG00000236896	0	0	0	0
ENSG00000236897	0	0	0	0
ENSG00000236898	0	0	0	0
ENSG00000236900	0	0	0	0
ENSG00000236901	0	0	1	0
ENSG00000236902	0	0	0	0
ENSG00000236903	0	0	0	0
ENSG00000236905	0	0	0	0
ENSG00000236907	0	0	0	0
ENSG00000236908	0	0	0	0
ENSG00000236909	0	0	0	0
ENSG00000236910	0	0	0	0
ENSG00000236911	0	12	10	30
ENSG00000236912	0	0	0	0
ENSG00000236913	0	0	0	0
ENSG00000236914	0	0	0	0
ENSG00000236915	0	38	1	20
ENSG00000236917	0	0	0	0
ENSG00000236918	0	0	0	0
ENSG00000236919	0	0	0	0
ENSG00000236920	0	0	0	0
ENSG00000236921	0	0	0	0
ENSG00000236922	0	0	0	0
ENSG00000236924	0	0	0	0

ENSG00000236925	0	0	0	0
ENSG00000236926	0	0	0	0
ENSG00000236927	0	0	0	0
ENSG00000236928	0	0	0	0
ENSG00000236929	0	0	0	0
ENSG00000236930	0	0	0	0
ENSG00000236931	0	0	0	0
ENSG00000236932	0	0	0	0
ENSG00000236933	0	0	7	0
ENSG00000236934	0	0	0	0
ENSG00000236935	9	0	2	0
ENSG00000236936	1214	1070	479	957
ENSG00000236937	0	0	0	0
ENSG00000236938	0	0	0	0
ENSG00000236939	0	0	0	0
ENSG00000236940	0	0	0	0
ENSG00000236941	0	0	0	0
ENSG00000236942	0	0	0	0
ENSG00000236943	0	72	92	244
ENSG00000236944	0	0	0	0
ENSG00000236946	0	0	0	0
ENSG00000236947	0	0	0	0
ENSG00000236948	0	0	0	0
ENSG00000236949	0	0	0	0
ENSG00000236950	0	0	0	0
ENSG00000236951	0	0	0	0
ENSG00000236953	0	0	0	0
ENSG00000236956	0	0	0	0
ENSG00000236957	0	0	0	0
ENSG00000236958	0	0	0	0
ENSG00000236959	0	0	0	0
ENSG00000236960	0	0	0	0
ENSG00000236961	0	0	0	0
ENSG00000236963	0	0	0	0
ENSG00000236965	0	0	0	0
ENSG00000236966	0	0	0	0
ENSG00000236967	0	0	0	0
ENSG00000236968	77	39	64	57
ENSG00000236969	0	0	0	0
ENSG00000236971	0	0	0	0
ENSG00000236972	0	0	0	0
ENSG00000236973	0	0	0	0
ENSG00000236975	0	0	0	0
ENSG00000236976	0	0	0	0
ENSG00000236977	0	0	0	0
ENSG00000236978	0	0	0	0

ENSG00000236979	0	0	0	0
ENSG00000236980	0	0	0	0
ENSG00000236981	0	0	0	0
ENSG00000236982	0	0	0	0
ENSG00000236983	0	0	0	0
ENSG00000236984	0	0	0	0
ENSG00000236985	0	0	0	0
ENSG00000236986	0	6	0	9
ENSG00000236987	0	0	0	0
ENSG00000236988	0	0	0	0
ENSG00000236989	0	0	0	0
ENSG00000236990	1	0	0	0
ENSG00000236991	0	0	1	3
ENSG00000236992	0	0	1	0
ENSG00000236993	0	0	0	0
ENSG00000236994	0	0	0	0
ENSG00000236996	0	0	0	0
ENSG00000236998	0	0	0	0
ENSG00000236999	0	0	0	0
ENSG00000237000	0	0	0	0
ENSG00000237001	0	0	0	0
ENSG00000237002	0	0	0	0
ENSG00000237003	0	0	0	0
ENSG00000237004	0	0	0	0
ENSG00000237005	0	0	0	0
ENSG00000237006	0	0	0	0
ENSG00000237007	0	0	0	0
ENSG00000237008	0	0	0	0
ENSG00000237009	0	0	0	0
ENSG00000237010	0	0	0	0
ENSG00000237011	0	0	0	0
ENSG00000237012	0	0	0	0
ENSG00000237013	0	0	0	0
ENSG00000237014	0	0	0	0
ENSG00000237015	0	0	0	0
ENSG00000237016	0	0	0	0
ENSG00000237017	0	0	0	0
ENSG00000237019	0	0	1	0
ENSG00000237020	0	0	0	0
ENSG00000237021	0	0	0	1
ENSG00000237022	0	0	0	0
ENSG00000237023	1	0	0	0
ENSG00000237024	0	0	0	0
ENSG00000237025	0	0	0	0
ENSG00000237026	0	0	0	0
ENSG00000237027	0	0	0	0

ENSG00000237030	0	0	0	0
ENSG00000237031	0	0	0	0
ENSG00000237032	0	0	0	0
ENSG00000237033	0	0	0	0
ENSG00000237034	0	0	0	0
ENSG00000237035	3	0	0	0
ENSG00000237036	0	0	0	0
ENSG00000237037	0	0	0	11
ENSG00000237038	0	0	0	0
ENSG00000237039	0	0	0	0
ENSG00000237040	0	0	0	0
ENSG00000237041	0	0	0	0
ENSG00000237042	0	0	0	0
ENSG00000237043	0	0	0	0
ENSG00000237046	0	0	0	0
ENSG00000237048	0	0	0	0
ENSG00000237049	0	0	0	0
ENSG00000237050	0	0	0	0
ENSG00000237051	0	0	0	0
ENSG00000237052	0	0	0	0
ENSG00000237053	0	0	0	0
ENSG00000237054	0	25	8	1
ENSG00000237055	0	0	0	0
ENSG00000237056	0	0	0	0
ENSG00000237057	0	0	0	0
ENSG00000237058	0	0	0	0
ENSG00000237061	0	0	0	0
ENSG00000237062	0	0	0	0
ENSG00000237063	0	0	0	0
ENSG00000237064	0	0	0	0
ENSG00000237065	0	0	0	0
ENSG00000237068	0	0	0	0
ENSG00000237069	0	0	0	0
ENSG00000237070	0	0	0	0
ENSG00000237071	0	0	0	0
ENSG00000237072	0	0	0	0
ENSG00000237073	12	0	10	10
ENSG00000237074	0	0	0	0
ENSG00000237075	0	0	0	0
ENSG00000237076	0	0	0	0
ENSG00000237077	0	0	0	0
ENSG00000237078	0	0	0	0
ENSG00000237080	0	0	0	2
ENSG00000237081	2	0	0	0
ENSG00000237082	0	0	0	0
ENSG00000237083	0	0	0	0

ENSG00000237085	0	0	0	0
ENSG00000237086	0	0	0	0
ENSG00000237087	0	0	0	0
ENSG00000237088	0	0	0	0
ENSG00000237089	0	0	0	0
ENSG00000237090	0	0	0	0
ENSG00000237092	0	0	0	0
ENSG00000237093	0	0	0	0
ENSG00000237094	1	140	62	112
ENSG00000237095	0	0	0	0
ENSG00000237096	6	0	0	13
ENSG00000237098	0	0	0	0
ENSG00000237099	0	0	0	0
ENSG00000237100	0	0	0	0
ENSG00000237101	0	0	0	0
ENSG00000237102	0	0	0	0
ENSG00000237103	0	0	0	0
ENSG00000237104	0	0	0	0
ENSG00000237105	0	0	0	0
ENSG00000237106	0	0	0	0
ENSG00000237107	0	0	0	0
ENSG00000237108	0	0	0	0
ENSG00000237109	0	0	0	0
ENSG00000237110	0	0	0	0
ENSG00000237111	0	0	0	0
ENSG00000237112	0	0	0	0
ENSG00000237114	0	0	0	0
ENSG00000237115	0	0	0	0
ENSG00000237118	28	0	25	51
ENSG00000237119	0	9	0	0
ENSG00000237120	0	0	0	0
ENSG00000237121	0	0	0	0
ENSG00000237122	0	0	0	0
ENSG00000237123	0	0	0	0
ENSG00000237124	0	0	0	0
ENSG00000237125	0	0	0	0
ENSG00000237126	0	0	3	0
ENSG00000237127	0	0	0	0
ENSG00000237128	0	0	0	0
ENSG00000237129	0	0	0	0
ENSG00000237130	0	0	0	0
ENSG00000237131	0	0	0	0
ENSG00000237132	0	0	0	0
ENSG00000237133	1	0	7	8
ENSG00000237135	0	0	0	0
ENSG00000237136	0	0	0	0

ENSG00000237137	0	0	0	0
ENSG00000237138	0	0	0	0
ENSG00000237139	0	0	0	0
ENSG00000237140	0	0	0	0
ENSG00000237141	0	0	0	0
ENSG00000237145	0	0	0	0
ENSG00000237148	0	0	0	0
ENSG00000237149	0	0	0	0
ENSG00000237152	0	0	0	0
ENSG00000237153	0	0	0	0
ENSG00000237154	0	0	0	0
ENSG00000237155	0	0	0	0
ENSG00000237156	0	0	0	0
ENSG00000237158	0	0	0	0
ENSG00000237159	0	0	0	0
ENSG00000237160	0	0	0	0
ENSG00000237161	1	0	0	7
ENSG00000237162	0	0	0	0
ENSG00000237163	0	0	0	0
ENSG00000237164	3	6	1	0
ENSG00000237165	0	0	0	0
ENSG00000237166	7	0	0	0
ENSG00000237167	0	0	0	0
ENSG00000237168	0	0	1	0
ENSG00000237169	2	0	0	0
ENSG00000237170	0	0	0	0
ENSG00000237171	0	0	0	0
ENSG00000237172	0	0	0	0
ENSG00000237173	0	0	0	0
ENSG00000237174	0	0	0	0
ENSG00000237175	0	0	0	0
ENSG00000237176	0	0	0	0
ENSG00000237178	0	0	0	0
ENSG00000237179	0	0	0	0
ENSG00000237180	0	0	0	0
ENSG00000237181	203	453	311	278
ENSG00000237182	0	0	0	0
ENSG00000237183	0	0	0	0
ENSG00000237184	0	0	0	0
ENSG00000237185	0	0	0	0
ENSG00000237186	0	0	0	0
ENSG00000237187	0	0	0	1
ENSG00000237188	5	66	9	26
ENSG00000237189	0	0	0	0
ENSG00000237190	32	26	6	22
ENSG00000237191	0	0	0	0

ENSG00000237192	0	0	0	0
ENSG00000237193	0	0	0	0
ENSG00000237194	0	0	0	0
ENSG00000237195	0	0	0	0
ENSG00000237196	0	0	0	0
ENSG00000237197	0	0	0	0
ENSG00000237198	0	0	0	0
ENSG00000237200	0	0	5	5
ENSG00000237202	0	0	0	0
ENSG00000237205	0	0	0	0
ENSG00000237206	0	0	0	0
ENSG00000237207	0	0	0	0
ENSG00000237208	0	0	0	0
ENSG00000237210	0	0	0	0
ENSG00000237211	0	0	0	0
ENSG00000237212	0	0	0	0
ENSG00000237213	0	0	0	0
ENSG00000237214	0	0	0	0
ENSG00000237215	0	0	0	0
ENSG00000237216	0	0	0	0
ENSG00000237217	0	0	0	0
ENSG00000237218	0	0	0	0
ENSG00000237220	0	0	0	0
ENSG00000237221	0	0	0	0
ENSG00000237222	0	0	0	0
ENSG00000237223	0	0	0	0
ENSG00000237224	0	0	0	0
ENSG00000237225	0	0	0	0
ENSG00000237226	0	0	0	0
ENSG00000237227	0	0	0	0
ENSG00000237230	0	0	0	0
ENSG00000237232	0	0	0	0
ENSG00000237233	0	0	0	0
ENSG00000237234	0	0	0	0
ENSG00000237235	0	0	0	0
ENSG00000237236	0	0	0	0
ENSG00000237238	1	331	53	51
ENSG00000237240	0	0	0	0
ENSG00000237242	0	0	0	0
ENSG00000237243	0	0	2	0
ENSG00000237247	0	0	0	0
ENSG00000237248	0	0	76	38
ENSG00000237249	0	0	2	0
ENSG00000237250	0	0	0	0
ENSG00000237251	0	0	0	0
ENSG00000237252	0	0	0	0

ENSG00000237253	0	0	0	0
ENSG00000237254	0	0	0	0
ENSG00000237256	0	0	0	0
ENSG00000237257	0	0	0	0
ENSG00000237258	0	0	0	0
ENSG00000237259	0	0	0	0
ENSG00000237260	0	0	0	0
ENSG00000237261	0	0	0	0
ENSG00000237262	0	0	0	0
ENSG00000237263	0	0	0	0
ENSG00000237264	1	125	133	132
ENSG00000237265	0	0	0	0
ENSG00000237266	0	0	0	0
ENSG00000237267	0	0	0	0
ENSG00000237268	0	0	6	2
ENSG00000237269	0	0	0	0
ENSG00000237271	0	0	0	0
ENSG00000237272	0	0	0	0
ENSG00000237273	0	0	0	0
ENSG00000237274	0	0	0	0
ENSG00000237275	0	0	0	0
ENSG00000237276	10	29	8	12
ENSG00000237278	0	0	0	0
ENSG00000237279	0	0	0	0
ENSG00000237280	0	0	0	0
ENSG00000237281	11	0	6	11
ENSG00000237282	0	0	0	0
ENSG00000237283	0	0	0	0
ENSG00000237284	0	0	0	0
ENSG00000237285	0	0	4	3
ENSG00000237286	0	0	0	0
ENSG00000237289	0	0	0	0
ENSG00000237290	0	0	0	0
ENSG00000237291	0	0	0	1
ENSG00000237292	0	0	0	2
ENSG00000237293	0	0	1	0
ENSG00000237294	0	0	0	0
ENSG00000237295	0	0	0	0
ENSG00000237296	0	56	38	26
ENSG00000237297	0	0	0	0
ENSG00000237298	2	371	535	544
ENSG00000237299	0	0	0	0
ENSG00000237300	0	0	0	0
ENSG00000237301	0	0	0	0
ENSG00000237302	0	0	0	0
ENSG00000237303	0	0	0	0

ENSG00000237306	0	0	0	0
ENSG00000237307	0	0	0	0
ENSG00000237308	0	0	0	0
ENSG00000237309	0	0	0	0
ENSG00000237310	50	24	9	12
ENSG00000237311	0	0	4	0
ENSG00000237312	0	0	0	0
ENSG00000237313	0	0	0	0
ENSG00000237314	0	0	0	0
ENSG00000237316	1	0	0	0
ENSG00000237317	0	0	0	0
ENSG00000237319	0	0	0	0
ENSG00000237320	0	0	0	0
ENSG00000237321	0	0	2	0
ENSG00000237322	0	0	0	0
ENSG00000237323	0	0	0	0
ENSG00000237324	0	0	0	0
ENSG00000237325	0	0	0	0
ENSG00000237326	0	0	0	2
ENSG00000237327	0	0	0	0
ENSG00000237328	0	0	0	0
ENSG00000237329	0	0	0	0
ENSG00000237330	0	0	0	0
ENSG00000237331	0	0	0	2
ENSG00000237332	0	0	0	0
ENSG00000237333	0	0	0	0
ENSG00000237334	0	0	0	0
ENSG00000237335	0	0	0	0
ENSG00000237336	0	0	0	0
ENSG00000237337	0	0	0	0
ENSG00000237338	0	0	0	0
ENSG00000237339	0	0	0	0
ENSG00000237341	0	0	0	0
ENSG00000237342	0	0	0	0
ENSG00000237343	0	0	0	0
ENSG00000237344	0	0	0	0
ENSG00000237345	0	0	0	0
ENSG00000237346	2	2	0	5
ENSG00000237347	0	0	0	0
ENSG00000237348	0	0	0	0
ENSG00000237349	0	0	0	0
ENSG00000237350	1	136	82	163
ENSG00000237351	0	0	0	0
ENSG00000237352	0	0	0	0
ENSG00000237353	0	0	0	0
ENSG00000237354	0	0	0	0

ENSG00000237356	0	0	1	6
ENSG00000237357	0	0	0	0
ENSG00000237358	0	0	0	0
ENSG00000237359	0	186	371	534
ENSG00000237360	0	0	0	0
ENSG00000237361	2	0	0	3
ENSG00000237363	0	0	0	0
ENSG00000237365	0	0	0	0
ENSG00000237366	0	0	0	0
ENSG00000237369	0	0	0	0
ENSG00000237370	0	0	0	0
ENSG00000237371	0	0	0	0
ENSG00000237372	1	0	0	0
ENSG00000237373	0	0	0	0
ENSG00000237374	0	0	0	0
ENSG00000237375	0	0	0	0
ENSG00000237377	0	0	0	0
ENSG00000237378	0	0	0	0
ENSG00000237379	0	0	0	0
ENSG00000237380	0	0	0	0
ENSG00000237381	0	0	0	0
ENSG00000237382	0	0	0	0
ENSG00000237383	0	0	0	0
ENSG00000237385	0	0	0	0
ENSG00000237387	1	0	0	0
ENSG00000237388	0	0	0	0
ENSG00000237389	0	0	0	0
ENSG00000237390	2	0	0	0
ENSG00000237393	0	0	0	0
ENSG00000237395	0	0	0	0
ENSG00000237396	0	0	0	0
ENSG00000237398	0	0	0	0
ENSG00000237399	0	0	4	4
ENSG00000237400	0	0	0	0
ENSG00000237401	0	0	0	0
ENSG00000237402	0	0	0	0
ENSG00000237403	0	0	0	0
ENSG00000237404	0	0	0	0
ENSG00000237405	0	0	0	0
ENSG00000237406	0	0	0	0
ENSG00000237407	0	0	0	0
ENSG00000237408	0	0	0	0
ENSG00000237409	0	0	0	0
ENSG00000237410	0	0	0	0
ENSG00000237412	0	0	0	0
ENSG00000237413	0	0	0	0

ENSG00000237414	0	0	2	2
ENSG00000237415	0	0	0	0
ENSG00000237416	0	0	0	0
ENSG00000237417	0	0	0	0
ENSG00000237418	0	0	0	0
ENSG00000237419	310	590	164	898
ENSG00000237421	0	0	0	0
ENSG00000237422	2	0	0	0
ENSG00000237423	0	0	0	0
ENSG00000237424	30	0	33	19
ENSG00000237425	0	0	0	0
ENSG00000237426	0	0	0	0
ENSG00000237427	0	0	0	0
ENSG00000237428	0	0	0	0
ENSG00000237429	27	9	5	33
ENSG00000237432	0	0	0	0
ENSG00000237433	0	0	0	0
ENSG00000237434	0	0	0	0
ENSG00000237435	0	26	10	11
ENSG00000237436	8	0	3	3
ENSG00000237437	0	0	0	0
ENSG00000237438	0	0	1	0
ENSG00000237439	0	0	0	0
ENSG00000237440	0	183	249	441
ENSG00000237441	3	0	2	11
ENSG00000237442	0	0	0	0
ENSG00000237443	0	0	0	0
ENSG00000237444	0	0	0	0
ENSG00000237445	0	0	0	0
ENSG00000237446	0	0	0	0
ENSG00000237447	0	0	0	0
ENSG00000237448	0	0	0	0
ENSG00000237449	0	0	0	0
ENSG00000237450	0	0	0	0
ENSG00000237451	0	0	0	1
ENSG00000237452	0	0	0	0
ENSG00000237453	0	0	0	0
ENSG00000237455	0	0	0	0
ENSG00000237456	0	0	0	0
ENSG00000237457	0	0	0	0
ENSG00000237458	0	0	0	0
ENSG00000237459	0	0	0	0
ENSG00000237460	0	0	0	0
ENSG00000237461	0	55	42	92
ENSG00000237462	0	0	0	0
ENSG00000237463	0	0	0	0

ENSG00000237464	0	0	0	0
ENSG00000237466	0	0	0	0
ENSG00000237467	0	0	0	0
ENSG00000237468	1	0	0	0
ENSG00000237469	0	0	0	0
ENSG00000237470	0	0	0	0
ENSG00000237471	0	0	0	0
ENSG00000237472	0	0	0	0
ENSG00000237473	169	363	652	351
ENSG00000237474	0	0	0	0
ENSG00000237475	0	0	0	0
ENSG00000237476	4	33	6	7
ENSG00000237477	0	0	0	0
ENSG00000237478	0	0	0	0
ENSG00000237479	0	0	0	0
ENSG00000237480	0	0	0	0
ENSG00000237481	0	28	0	0
ENSG00000237482	0	0	0	0
ENSG00000237483	0	0	0	0
ENSG00000237484	13	488	709	657
ENSG00000237485	2	0	12	13
ENSG00000237487	0	0	0	0
ENSG00000237489	2	11	3	0
ENSG00000237490	0	0	0	0
ENSG00000237491	0	0	5	31
ENSG00000237492	0	0	0	0
ENSG00000237493	0	0	0	1
ENSG00000237494	0	0	0	0
ENSG00000237495	0	0	0	0
ENSG00000237498	0	0	0	0
ENSG00000237499	18	75	53	85
ENSG00000237500	0	0	0	0
ENSG00000237501	0	0	0	0
ENSG00000237502	0	0	0	0
ENSG00000237503	0	0	0	0
ENSG00000237505	1	43	55	113
ENSG00000237506	0	1	6	2
ENSG00000237508	0	0	0	0
ENSG00000237510	0	0	0	0
ENSG00000237512	0	0	0	0
ENSG00000237513	0	1	1	0
ENSG00000237514	0	0	0	0
ENSG00000237515	35	49	43	31
ENSG00000237517	0	0	0	0
ENSG00000237520	0	0	0	0
ENSG00000237521	0	0	0	0

ENSG00000237522	0	0	0	0
ENSG00000237523	0	0	0	0
ENSG00000237524	0	0	0	0
ENSG00000237525	0	0	1	0
ENSG00000237527	0	0	0	0
ENSG00000237528	0	0	0	0
ENSG00000237529	2	0	3	9
ENSG00000237530	0	0	0	0
ENSG00000237531	0	0	0	0
ENSG00000237532	0	0	0	0
ENSG00000237533	0	0	0	0
ENSG00000237534	0	0	0	0
ENSG00000237536	0	0	0	0
ENSG00000237539	0	0	0	0
ENSG00000237540	0	0	0	0
ENSG00000237541	19	0	0	0
ENSG00000237542	0	0	0	0
ENSG00000237543	0	16	0	0
ENSG00000237544	0	0	0	0
ENSG00000237545	0	0	0	0
ENSG00000237546	0	0	0	0
ENSG00000237547	0	0	0	0
ENSG00000237548	0	0	0	0
ENSG00000237549	0	0	0	0
ENSG00000237550	3	0	0	0
ENSG00000237551	0	0	0	0
ENSG00000237552	0	0	0	0
ENSG00000237553	0	0	0	0
ENSG00000237554	0	0	0	0
ENSG00000237555	0	0	0	0
ENSG00000237556	0	0	0	0
ENSG00000237557	0	0	0	0
ENSG00000237558	0	0	0	0
ENSG00000237559	0	0	0	0
ENSG00000237560	0	0	0	0
ENSG00000237563	0	0	0	0
ENSG00000237565	0	0	0	0
ENSG00000237566	0	0	0	0
ENSG00000237567	0	0	0	0
ENSG00000237568	0	0	0	0
ENSG00000237569	0	0	0	0
ENSG00000237571	0	0	0	0
ENSG00000237572	0	0	0	0
ENSG00000237574	0	0	0	0
ENSG00000237575	2	0	0	0
ENSG00000237576	9	0	1	4

ENSG00000237577	0	0	0	0
ENSG00000237578	0	0	0	0
ENSG00000237579	0	0	0	0
ENSG00000237580	0	0	1	4
ENSG00000237581	0	0	0	0
ENSG00000237582	0	0	0	0
ENSG00000237583	0	0	0	0
ENSG00000237584	0	0	0	0
ENSG00000237585	1	0	3	3
ENSG00000237586	0	0	0	0
ENSG00000237587	0	0	0	0
ENSG00000237588	0	0	0	0
ENSG00000237589	0	0	0	0
ENSG00000237590	0	0	0	0
ENSG00000237591	0	0	0	0
ENSG00000237592	0	0	0	0
ENSG00000237593	0	0	0	0
ENSG00000237594	0	0	0	0
ENSG00000237595	13	16	3	26
ENSG00000237596	0	0	0	0
ENSG00000237597	0	0	0	0
ENSG00000237599	0	0	0	0
ENSG00000237601	0	0	0	0
ENSG00000237603	0	0	0	0
ENSG00000237604	0	0	0	0
ENSG00000237605	0	0	0	0
ENSG00000237606	0	0	0	0
ENSG00000237608	0	0	0	0
ENSG00000237609	0	0	0	0
ENSG00000237610	0	0	0	0
ENSG00000237611	2	0	0	0
ENSG00000237612	0	0	0	0
ENSG00000237613	0	0	0	0
ENSG00000237614	0	0	0	0
ENSG00000237616	0	0	0	0
ENSG00000237617	0	0	0	0
ENSG00000237618	0	0	9	0
ENSG00000237619	0	0	0	0
ENSG00000237620	0	0	0	3
ENSG00000237621	0	0	0	0
ENSG00000237622	0	0	0	0
ENSG00000237623	0	0	0	0
ENSG00000237624	0	0	0	0
ENSG00000237625	0	0	0	0
ENSG00000237626	1	0	0	0
ENSG00000237628	1	0	0	0

ENSG00000237629	16	34	29	16
ENSG00000237630	0	0	0	0
ENSG00000237631	0	0	3	2
ENSG00000237632	0	0	0	0
ENSG00000237633	0	0	0	0
ENSG00000237634	0	0	0	0
ENSG00000237635	0	0	0	0
ENSG00000237636	0	0	0	0
ENSG00000237637	0	0	0	0
ENSG00000237638	0	0	0	0
ENSG00000237639	0	0	0	0
ENSG00000237640	0	0	0	0
ENSG00000237641	0	0	0	0
ENSG00000237642	0	0	0	0
ENSG00000237643	0	0	0	0
ENSG00000237645	0	0	0	0
ENSG00000237646	0	0	0	0
ENSG00000237647	0	0	0	0
ENSG00000237649	1	0	0	8
ENSG00000237650	0	0	0	0
ENSG00000237651	0	0	0	0
ENSG00000237653	0	0	0	0
ENSG00000237654	0	0	0	0
ENSG00000237655	0	0	1	1
ENSG00000237658	0	0	0	0
ENSG00000237659	0	0	0	0
ENSG00000237661	0	0	0	0
ENSG00000237662	0	0	0	0
ENSG00000237663	0	0	0	0
ENSG00000237664	0	0	0	0
ENSG00000237665	0	0	0	0
ENSG00000237666	0	0	0	0
ENSG00000237667	0	0	0	0
ENSG00000237668	0	0	0	0
ENSG00000237669	0	0	0	0
ENSG00000237670	0	0	0	0
ENSG00000237671	0	0	0	0
ENSG00000237672	0	0	0	2
ENSG00000237674	0	0	0	0
ENSG00000237675	0	0	0	0
ENSG00000237676	0	0	0	0
ENSG00000237677	0	0	0	0
ENSG00000237679	0	0	0	0
ENSG00000237682	0	0	0	0
ENSG00000237683	7	0	2	3
ENSG00000237684	0	0	0	0

ENSG00000237685	0	0	0	0
ENSG00000237686	91	0	4	11
ENSG00000237687	0	0	0	0
ENSG00000237689	0	0	0	0
ENSG00000237690	0	0	0	0
ENSG00000237691	0	0	0	0
ENSG00000237693	1	7	1	5
ENSG00000237697	0	0	0	0
ENSG00000237698	0	0	0	0
ENSG00000237699	0	0	0	0
ENSG00000237700	0	0	0	0
ENSG00000237701	0	0	0	0
ENSG00000237702	0	0	0	0
ENSG00000237703	0	0	0	0
ENSG00000237704	0	0	0	0
ENSG00000237705	2	0	1	14
ENSG00000237706	0	0	0	0
ENSG00000237707	0	0	0	0
ENSG00000237708	0	0	0	0
ENSG00000237709	0	0	0	0
ENSG00000237710	0	0	0	0
ENSG00000237711	0	0	1	3
ENSG00000237713	0	0	0	0
ENSG00000237714	0	0	0	0
ENSG00000237716	0	0	0	0
ENSG00000237717	0	0	0	0
ENSG00000237718	0	0	0	0
ENSG00000237719	0	0	0	0
ENSG00000237720	0	0	0	0
ENSG00000237721	0	0	0	0
ENSG00000237722	0	0	0	0
ENSG00000237723	0	0	0	0
ENSG00000237724	0	0	0	0
ENSG00000237725	0	0	0	0
ENSG00000237726	0	0	0	0
ENSG00000237727	0	0	0	0
ENSG00000237728	0	0	0	0
ENSG00000237729	2	0	0	0
ENSG00000237730	0	0	0	0
ENSG00000237731	0	0	0	0
ENSG00000237732	2	0	0	0
ENSG00000237733	0	0	0	0
ENSG00000237734	0	0	0	0
ENSG00000237735	0	0	0	0
ENSG00000237736	0	0	0	0
ENSG00000237737	0	0	2	0

ENSG00000237738	4	0	0	6
ENSG00000237740	0	7	1	0
ENSG00000237741	0	0	0	0
ENSG00000237742	0	0	0	0
ENSG00000237743	0	0	0	6
ENSG00000237745	0	0	0	0
ENSG00000237746	0	0	0	0
ENSG00000237747	0	0	0	0
ENSG00000237748	0	0	0	0
ENSG00000237749	3	0	0	1
ENSG00000237750	0	0	0	0
ENSG00000237751	0	0	0	0
ENSG00000237752	0	0	0	0
ENSG00000237753	48	32	62	56
ENSG00000237754	0	0	0	0
ENSG00000237756	0	0	0	0
ENSG00000237757	0	0	0	1
ENSG00000237758	0	0	0	0
ENSG00000237759	0	0	0	0
ENSG00000237760	0	0	0	0
ENSG00000237761	0	0	0	0
ENSG00000237763	0	0	0	0
ENSG00000237764	0	23	0	0
ENSG00000237765	214	259	268	465
ENSG00000237766	1	0	3	0
ENSG00000237767	0	0	0	0
ENSG00000237768	0	0	13	4
ENSG00000237770	0	0	0	0
ENSG00000237772	0	0	0	0
ENSG00000237773	10	31	10	19
ENSG00000237774	0	0	0	0
ENSG00000237775	0	0	0	0
ENSG00000237777	0	0	0	0
ENSG00000237781	0	0	0	2
ENSG00000237782	0	0	0	0
ENSG00000237783	0	0	0	0
ENSG00000237784	0	0	0	0
ENSG00000237786	0	0	2	1
ENSG00000237787	7	0	1	7
ENSG00000237788	0	0	0	0
ENSG00000237790	0	0	0	0
ENSG00000237792	0	0	0	0
ENSG00000237793	0	0	0	0
ENSG00000237796	0	0	0	0
ENSG00000237797	0	0	0	0
ENSG00000237798	0	103	13	7

ENSG00000237799	0	0	0	0
ENSG00000237801	0	0	1	0
ENSG00000237802	0	0	0	0
ENSG00000237803	297	849	645	670
ENSG00000237804	0	0	0	0
ENSG00000237805	118	72	149	133
ENSG00000237806	0	0	0	0
ENSG00000237807	0	0	0	0
ENSG00000237808	0	0	0	0
ENSG00000237810	0	0	1	0
ENSG00000237811	0	0	0	0
ENSG00000237812	0	0	0	0
ENSG00000237813	0	0	9	2
ENSG00000237814	0	0	0	0
ENSG00000237815	0	0	0	0
ENSG00000237816	0	0	0	0
ENSG00000237818	0	0	4	0
ENSG00000237819	23	2	10	31
ENSG00000237821	0	1	2	1
ENSG00000237822	0	0	0	0
ENSG00000237823	0	0	0	0
ENSG00000237824	0	0	0	0
ENSG00000237825	0	0	0	0
ENSG00000237827	0	0	1	0
ENSG00000237828	0	0	0	0
ENSG00000237829	0	0	0	0
ENSG00000237831	0	0	0	0
ENSG00000237832	0	0	0	0
ENSG00000237833	0	0	0	0
ENSG00000237834	0	0	0	0
ENSG00000237835	0	0	0	0
ENSG00000237836	0	15	2	1
ENSG00000237837	0	0	0	0
ENSG00000237838	0	0	0	0
ENSG00000237840	0	15	12	9
ENSG00000237841	0	0	0	0
ENSG00000237842	0	0	0	0
ENSG00000237843	0	0	0	0
ENSG00000237844	0	0	0	0
ENSG00000237845	0	4	0	0
ENSG00000237846	4	0	0	0
ENSG00000237847	0	0	0	0
ENSG00000237848	0	0	0	0
ENSG00000237849	0	0	0	0
ENSG00000237850	0	0	0	0
ENSG00000237851	0	0	0	0

ENSG00000237852	62	66	18	69
ENSG00000237853	0	0	0	0
ENSG00000237854	92	546	397	973
ENSG00000237856	0	0	0	0
ENSG00000237857	0	0	0	0
ENSG00000237859	0	0	0	0
ENSG00000237860	0	0	0	0
ENSG00000237861	0	0	0	0
ENSG00000237862	0	0	0	0
ENSG00000237863	0	0	0	0
ENSG00000237864	0	0	0	0
ENSG00000237865	0	0	0	0
ENSG00000237868	0	0	0	0
ENSG00000237869	0	0	0	0
ENSG00000237870	1	0	9	12
ENSG00000237871	0	0	0	0
ENSG00000237872	0	0	0	0
ENSG00000237873	0	0	0	0
ENSG00000237874	0	0	0	0
ENSG00000237875	0	0	0	0
ENSG00000237876	0	0	0	0
ENSG00000237877	1	388	552	683
ENSG00000237879	0	0	0	0
ENSG00000237880	0	0	0	0
ENSG00000237881	0	0	0	0
ENSG00000237882	0	0	0	0
ENSG00000237883	0	0	7	0
ENSG00000237884	0	0	0	0
ENSG00000237885	0	0	0	0
ENSG00000237886	0	0	0	0
ENSG00000237887	0	0	0	0
ENSG00000237888	0	0	0	0
ENSG00000237889	0	0	0	0
ENSG00000237890	0	0	0	0
ENSG00000237891	0	0	0	0
ENSG00000237892	0	0	6	4
ENSG00000237894	0	0	0	0
ENSG00000237895	0	0	0	0
ENSG00000237896	0	0	0	0
ENSG00000237897	0	0	0	0
ENSG00000237898	0	0	0	0
ENSG00000237899	0	0	0	4
ENSG00000237900	0	0	0	0
ENSG00000237901	0	0	0	0
ENSG00000237902	0	0	0	0
ENSG00000237903	0	0	0	0

ENSG00000237904	0	0	0	0
ENSG00000237905	0	0	0	0
ENSG00000237906	0	0	0	0
ENSG00000237907	0	0	0	0
ENSG00000237910	0	0	0	0
ENSG00000237911	0	0	0	0
ENSG00000237913	0	0	0	0
ENSG00000237914	0	0	0	0
ENSG00000237916	0	0	0	0
ENSG00000237917	0	0	0	0
ENSG00000237918	0	0	0	0
ENSG00000237919	0	0	0	0
ENSG00000237920	0	0	0	0
ENSG00000237921	0	0	0	0
ENSG00000237922	0	0	0	0
ENSG00000237923	0	0	0	0
ENSG00000237924	0	0	0	0
ENSG00000237926	0	0	0	0
ENSG00000237927	0	0	0	0
ENSG00000237928	37	61	26	169
ENSG00000237929	0	0	0	0
ENSG00000237930	0	0	0	0
ENSG00000237931	0	0	0	0
ENSG00000237934	0	0	0	0
ENSG00000237936	0	0	0	0
ENSG00000237937	0	0	0	0
ENSG00000237938	0	0	0	0
ENSG00000237939	0	0	0	0
ENSG00000237940	0	0	0	5
ENSG00000237941	0	0	0	0
ENSG00000237943	51	166	117	92
ENSG00000237945	52	29	73	87
ENSG00000237947	0	0	0	0
ENSG00000237948	0	0	0	0
ENSG00000237949	0	0	0	0
ENSG00000237950	0	0	0	0
ENSG00000237951	0	0	0	0
ENSG00000237952	0	0	0	0
ENSG00000237953	0	0	0	0
ENSG00000237954	0	0	0	0
ENSG00000237955	0	0	0	0
ENSG00000237956	0	0	0	0
ENSG00000237957	0	0	0	0
ENSG00000237958	0	0	0	0
ENSG00000237959	0	0	0	0
ENSG00000237961	0	0	0	0

ENSG00000237962	0	0	0	0
ENSG00000237963	0	0	0	0
ENSG00000237964	0	0	0	0
ENSG00000237968	0	0	0	0
ENSG00000237970	0	5	0	0
ENSG00000237971	0	0	0	0
ENSG00000237972	0	0	0	0
ENSG00000237973	502	663	949	1331
ENSG00000237974	0	0	0	0
ENSG00000237975	0	0	3	10
ENSG00000237976	2	0	0	0
ENSG00000237977	0	0	4	0
ENSG00000237978	0	9	5	6
ENSG00000237979	0	0	0	0
ENSG00000237980	0	0	0	0
ENSG00000237982	0	0	0	0
ENSG00000237984	44	226	516	348
ENSG00000237986	0	0	0	0
ENSG00000237987	0	0	0	0
ENSG00000237988	0	0	0	0
ENSG00000237989	0	0	0	0
ENSG00000237990	59	451	231	37
ENSG00000237991	0	0	0	0
ENSG00000237992	0	0	0	0
ENSG00000237993	0	0	0	0
ENSG00000237994	0	0	0	0
ENSG00000237997	0	0	0	0
ENSG00000237998	0	0	0	0
ENSG00000237999	0	0	0	0
ENSG00000238000	0	0	0	0
ENSG00000238001	0	0	0	0
ENSG00000238002	0	0	0	0
ENSG00000238003	0	0	0	0
ENSG00000238004	0	0	0	0
ENSG00000238005	9	4	6	3
ENSG00000238007	0	0	0	0
ENSG00000238008	0	0	0	0
ENSG00000238009	2	16	10	28
ENSG00000238010	0	0	0	0
ENSG00000238012	0	0	1	2
ENSG00000238013	0	0	0	0
ENSG00000238015	0	3	2	0
ENSG00000238017	0	0	0	0
ENSG00000238018	0	0	0	0
ENSG00000238019	0	0	0	0
ENSG00000238020	0	0	0	0

ENSG00000238021	0	0	0	0
ENSG00000238022	0	0	0	0
ENSG00000238024	0	0	0	0
ENSG00000238025	0	0	0	0
ENSG00000238026	0	0	0	0
ENSG00000238027	0	0	0	0
ENSG00000238029	0	0	0	0
ENSG00000238031	0	0	0	0
ENSG00000238032	0	0	0	0
ENSG00000238033	0	0	0	0
ENSG00000238034	0	0	0	0
ENSG00000238035	0	0	0	0
ENSG00000238037	0	0	0	0
ENSG00000238038	0	0	0	0
ENSG00000238039	0	0	0	0
ENSG00000238040	0	0	0	0
ENSG00000238041	0	0	0	0
ENSG00000238042	0	14	0	1
ENSG00000238043	0	0	0	0
ENSG00000238045	0	40	64	11
ENSG00000238046	0	0	0	0
ENSG00000238047	0	0	0	0
ENSG00000238048	0	0	0	0
ENSG00000238049	0	0	0	0
ENSG00000238051	0	0	0	0
ENSG00000238052	0	0	0	0
ENSG00000238053	0	0	0	0
ENSG00000238054	0	0	0	0
ENSG00000238055	0	12	1	16
ENSG00000238056	0	0	0	0
ENSG00000238057	11	377	295	238
ENSG00000238058	0	0	1	4
ENSG00000238059	0	0	0	0
ENSG00000238061	4	0	5	1
ENSG00000238062	0	0	0	0
ENSG00000238063	0	0	0	0
ENSG00000238064	0	0	0	0
ENSG00000238065	0	0	0	0
ENSG00000238066	0	0	0	0
ENSG00000238067	0	0	0	0
ENSG00000238068	0	0	0	0
ENSG00000238069	0	0	0	0
ENSG00000238070	0	0	0	0
ENSG00000238071	0	0	0	0
ENSG00000238072	0	0	0	0
ENSG00000238073	0	0	0	0

ENSG00000238074	0	0	0	0
ENSG00000238075	0	0	0	0
ENSG00000238076	0	0	0	0
ENSG00000238077	0	0	0	0
ENSG00000238078	0	0	0	0
ENSG00000238079	0	0	0	0
ENSG00000238081	0	0	0	0
ENSG00000238082	0	0	0	0
ENSG00000238083	29	53	35	102
ENSG00000238084	0	0	0	1
ENSG00000238085	0	0	0	0
ENSG00000238086	0	0	0	0
ENSG00000238087	0	0	0	0
ENSG00000238088	0	0	0	0
ENSG00000238090	0	0	0	0
ENSG00000238091	0	0	0	0
ENSG00000238092	0	0	0	0
ENSG00000238094	0	0	0	0
ENSG00000238097	0	0	3	0
ENSG00000238098	2	0	0	3
ENSG00000238099	0	0	0	0
ENSG00000238100	0	0	0	0
ENSG00000238101	0	0	0	0
ENSG00000238102	0	0	0	0
ENSG00000238103	1	0	0	0
ENSG00000238104	0	0	0	0
ENSG00000238105	4	19	9	12
ENSG00000238106	0	0	0	0
ENSG00000238107	0	0	0	0
ENSG00000238108	0	0	0	0
ENSG00000238109	0	0	0	0
ENSG00000238110	0	0	0	0
ENSG00000238111	0	0	0	0
ENSG00000238113	6	21	7	3
ENSG00000238114	0	0	0	0
ENSG00000238116	0	0	0	0
ENSG00000238117	0	0	0	0
ENSG00000238118	0	0	0	0
ENSG00000238120	5	0	11	0
ENSG00000238121	0	0	0	0
ENSG00000238122	0	0	0	0
ENSG00000238123	0	0	0	0
ENSG00000238124	0	0	0	0
ENSG00000238125	1	2	2	0
ENSG00000238126	0	0	0	0
ENSG00000238127	0	0	0	0

ENSG00000238129	0	0	0	0
ENSG00000238130	0	0	0	0
ENSG00000238131	0	0	0	0
ENSG00000238132	0	0	0	0
ENSG00000238133	0	0	1	0
ENSG00000238134	0	0	0	0
ENSG00000238135	0	0	0	0
ENSG00000238137	0	0	0	0
ENSG00000238138	0	0	0	0
ENSG00000238139	0	0	0	0
ENSG00000238140	3	0	0	0
ENSG00000238141	0	0	1	5
ENSG00000238142	1	0	0	0
ENSG00000238143	0	0	0	0
ENSG00000238145	0	0	0	0
ENSG00000238148	0	0	0	0
ENSG00000238149	0	0	0	0
ENSG00000238150	0	0	0	0
ENSG00000238151	0	0	0	0
ENSG00000238152	0	0	0	0
ENSG00000238153	0	0	0	0
ENSG00000238154	0	0	0	0
ENSG00000238156	0	0	0	0
ENSG00000238158	0	0	0	0
ENSG00000238160	43	212	259	306
ENSG00000238161	0	0	0	0
ENSG00000238162	0	0	0	0
ENSG00000238164	18	62	49	37
ENSG00000238165	0	0	0	0
ENSG00000238166	0	0	0	0
ENSG00000238168	0	0	0	0
ENSG00000238169	0	0	0	0
ENSG00000238170	0	0	0	0
ENSG00000238171	0	0	0	0
ENSG00000238172	0	0	0	0
ENSG00000238173	0	0	0	0
ENSG00000238176	0	0	0	0
ENSG00000238178	1	0	1	0
ENSG00000238179	0	0	1	0
ENSG00000238180	0	0	0	0
ENSG00000238181	0	0	0	0
ENSG00000238183	0	0	0	0
ENSG00000238184	0	0	0	0
ENSG00000238185	0	0	0	0
ENSG00000238186	0	0	0	0
ENSG00000238188	0	0	0	0

ENSG00000238189	0	0	0	0
ENSG00000238190	0	0	0	0
ENSG00000238191	0	0	0	0
ENSG00000238192	0	0	0	0
ENSG00000238193	0	0	0	0
ENSG00000238194	0	0	0	0
ENSG00000238195	0	0	0	0
ENSG00000238196	0	0	0	0
ENSG00000238197	3	12	5	20
ENSG00000238198	1	0	0	0
ENSG00000238199	0	0	0	0
ENSG00000238200	0	0	0	0
ENSG00000238201	2143	6866	8255	11526
ENSG00000238202	0	0	0	0
ENSG00000238203	0	0	0	0
ENSG00000238205	0	0	0	0
ENSG00000238206	15	22	0	0
ENSG00000238207	0	0	0	0
ENSG00000238208	0	0	0	0
ENSG00000238210	0	0	0	0
ENSG00000238211	0	0	0	0
ENSG00000238212	0	0	0	0
ENSG00000238213	0	0	0	0
ENSG00000238215	0	0	0	0
ENSG00000238216	0	0	0	0
ENSG00000238217	0	0	0	0
ENSG00000238220	0	0	0	0
ENSG00000238221	3	216	155	106
ENSG00000238222	0	0	0	0
ENSG00000238223	0	0	0	0
ENSG00000238224	0	0	0	0
ENSG00000238225	0	0	1	0
ENSG00000238226	0	0	0	0
ENSG00000238227	21	69	48	61
ENSG00000238228	0	0	0	0
ENSG00000238230	0	0	0	0
ENSG00000238231	0	0	0	0
ENSG00000238232	0	0	1	0
ENSG00000238234	0	0	0	0
ENSG00000238235	0	0	0	0
ENSG00000238236	0	0	0	0
ENSG00000238238	0	0	0	0
ENSG00000238240	0	0	0	0
ENSG00000238241	0	0	0	0
ENSG00000238242	0	0	3	0
ENSG00000238243	187	103	323	270

ENSG00000238244	0	0	0	0
ENSG00000238245	0	0	0	0
ENSG00000238246	0	0	0	0
ENSG00000238247	0	0	2	0
ENSG00000238248	0	0	0	0
ENSG00000238249	0	0	0	0
ENSG00000238250	0	0	0	0
ENSG00000238251	0	0	0	0
ENSG00000238254	0	0	0	0
ENSG00000238255	0	0	0	0
ENSG00000238256	0	0	0	0
ENSG00000238257	0	0	0	0
ENSG00000238258	1	0	0	0
ENSG00000238259	0	0	0	0
ENSG00000238260	0	0	0	0
ENSG00000238261	0	0	9	12
ENSG00000238262	0	0	0	0
ENSG00000238263	0	0	0	0
ENSG00000238265	0	0	0	0
ENSG00000238266	0	0	0	0
ENSG00000238267	0	0	0	0
ENSG00000238268	0	0	0	0
ENSG00000238269	0	0	2	0
ENSG00000238270	0	0	0	0
ENSG00000238271	0	0	0	0
ENSG00000238272	0	0	0	0
ENSG00000238273	0	0	0	0
ENSG00000238274	60	311	283	599
ENSG00000238275	0	0	0	0
ENSG00000238276	0	0	0	0
ENSG00000238277	0	0	0	0
ENSG00000238278	2	52	14	44
ENSG00000238279	0	0	0	0
ENSG00000238280	0	0	0	1
ENSG00000238282	0	0	0	0
ENSG00000238283	0	0	0	0
ENSG00000238284	0	0	0	0
ENSG00000238285	0	0	0	0
ENSG00000238286	0	0	0	4
ENSG00000238287	0	0	0	0
ENSG00000238288	0	0	0	0
ENSG00000238289	0	0	0	0
ENSG00000238290	0	0	0	0
ENSG00000238291	0	0	0	0
ENSG00000238294	0	0	0	0
ENSG00000238295	0	0	0	0

ENSG00000238296	0	0	0	0
ENSG00000238297	0	0	0	0
ENSG00000238298	0	0	0	0
ENSG00000238300	0	0	0	0
ENSG00000238301	0	0	0	0
ENSG00000238302	0	0	0	0
ENSG00000238303	0	0	0	0
ENSG00000238304	0	0	0	0
ENSG00000238305	0	0	0	0
ENSG00000238306	0	0	0	0
ENSG00000238309	0	0	0	1
ENSG00000238310	0	0	0	0
ENSG00000238311	0	0	0	0
ENSG00000238312	0	0	0	0
ENSG00000238313	0	0	0	0
ENSG00000238314	0	0	0	1
ENSG00000238315	0	0	0	0
ENSG00000238316	0	0	0	0
ENSG00000238317	0	0	0	0
ENSG00000238318	0	0	0	0
ENSG00000238319	0	0	0	0
ENSG00000238320	0	0	0	0
ENSG00000238321	0	0	0	0
ENSG00000238322	0	0	0	0
ENSG00000238323	0	0	0	0
ENSG00000238324	0	0	0	0
ENSG00000238325	0	0	0	0
ENSG00000238326	0	0	0	0
ENSG00000238327	0	0	0	0
ENSG00000238328	0	0	0	0
ENSG00000238329	0	0	0	0
ENSG00000238330	0	0	0	0
ENSG00000238331	0	0	0	0
ENSG00000238333	0	0	0	0
ENSG00000238334	0	0	0	0
ENSG00000238335	0	0	0	0
ENSG00000238336	0	0	0	0
ENSG00000238337	0	0	0	0
ENSG00000238339	0	0	0	0
ENSG00000238340	0	0	0	0
ENSG00000238341	0	0	0	0
ENSG00000238342	0	0	0	0
ENSG00000238343	0	0	0	0
ENSG00000238344	0	0	0	0
ENSG00000238345	0	0	0	0
ENSG00000238347	0	0	0	0

ENSG00000238348	0	0	0	0
ENSG00000238349	0	0	0	0
ENSG00000238350	0	0	0	0
ENSG00000238351	0	0	0	0
ENSG00000238352	0	0	0	0
ENSG00000238353	0	0	0	0
ENSG00000238354	0	0	0	0
ENSG00000238355	0	0	0	0
ENSG00000238357	0	0	0	0
ENSG00000238358	0	0	0	0
ENSG00000238359	0	0	0	0
ENSG00000238361	0	0	0	0
ENSG00000238362	0	0	0	0
ENSG00000238363	0	0	0	0
ENSG00000238364	0	0	0	0
ENSG00000238365	0	0	0	0
ENSG00000238366	0	0	0	0
ENSG00000238367	0	0	0	0
ENSG00000238368	0	0	0	0
ENSG00000238369	0	0	0	0
ENSG00000238370	0	0	0	0
ENSG00000238371	0	0	0	0
ENSG00000238372	0	0	0	0
ENSG00000238374	0	0	0	0
ENSG00000238375	0	0	0	0
ENSG00000238376	0	0	0	0
ENSG00000238377	0	0	0	0
ENSG00000238379	0	0	0	0
ENSG00000238380	0	0	0	0
ENSG00000238382	0	0	0	0
ENSG00000238383	0	0	0	0
ENSG00000238384	0	0	0	0
ENSG00000238385	0	0	0	0
ENSG00000238386	0	0	0	0
ENSG00000238387	0	0	0	0
ENSG00000238388	0	0	0	0
ENSG00000238389	0	0	0	0
ENSG00000238390	0	0	0	0
ENSG00000238391	0	0	0	0
ENSG00000238392	0	0	0	0
ENSG00000238393	0	0	0	0
ENSG00000238394	0	0	0	0
ENSG00000238395	0	0	0	0
ENSG00000238397	0	0	0	0
ENSG00000238398	0	0	0	0
ENSG00000238399	0	0	0	0

ENSG00000238400	0	0	0	0
ENSG00000238401	0	24	0	0
ENSG00000238402	0	0	0	0
ENSG00000238403	0	0	0	0
ENSG00000238405	0	0	0	0
ENSG00000238406	0	0	0	0
ENSG00000238407	0	0	0	0
ENSG00000238408	0	0	0	0
ENSG00000238409	0	0	0	0
ENSG00000238410	0	0	0	0
ENSG00000238411	0	0	0	0
ENSG00000238412	0	0	0	0
ENSG00000238413	0	0	0	0
ENSG00000238414	0	0	0	0
ENSG00000238415	0	0	0	0
ENSG00000238416	0	0	0	0
ENSG00000238417	0	0	0	0
ENSG00000238418	0	0	0	0
ENSG00000238419	1	0	0	0
ENSG00000238420	0	0	0	0
ENSG00000238421	3	0	0	1
ENSG00000238422	0	0	0	0
ENSG00000238423	0	0	0	0
ENSG00000238424	0	0	0	0
ENSG00000238425	0	0	0	0
ENSG00000238426	0	0	0	0
ENSG00000238427	0	0	0	0
ENSG00000238428	0	0	0	0
ENSG00000238430	0	0	0	0
ENSG00000238431	0	0	0	0
ENSG00000238432	0	0	0	0
ENSG00000238433	0	0	0	0
ENSG00000238436	0	0	0	0
ENSG00000238437	0	0	0	0
ENSG00000238438	0	0	0	0
ENSG00000238439	0	0	0	0
ENSG00000238440	0	0	0	0
ENSG00000238441	0	0	0	0
ENSG00000238442	0	0	0	0
ENSG00000238443	0	0	0	0
ENSG00000238444	0	0	0	0
ENSG00000238445	0	0	0	0
ENSG00000238446	0	0	0	0
ENSG00000238447	0	0	0	0
ENSG00000238448	0	0	0	0
ENSG00000238449	0	0	0	0

ENSG00000238450	0	0	0	0
ENSG00000238451	0	0	0	0
ENSG00000238452	0	0	0	0
ENSG00000238453	0	0	0	0
ENSG00000238455	0	0	0	0
ENSG00000238456	0	0	0	0
ENSG00000238457	0	0	0	0
ENSG00000238458	0	0	0	0
ENSG00000238459	0	0	0	0
ENSG00000238460	0	0	0	0
ENSG00000238461	0	0	0	0
ENSG00000238462	0	0	0	0
ENSG00000238463	0	0	0	0
ENSG00000238464	0	0	0	0
ENSG00000238465	0	0	0	0
ENSG00000238466	0	0	0	0
ENSG00000238468	0	0	0	0
ENSG00000238469	0	0	0	0
ENSG00000238470	0	0	0	0
ENSG00000238471	0	0	0	0
ENSG00000238472	0	0	0	0
ENSG00000238473	0	0	0	0
ENSG00000238474	0	0	0	0
ENSG00000238475	0	0	0	0
ENSG00000238476	0	0	0	0
ENSG00000238477	0	0	0	0
ENSG00000238478	0	0	0	0
ENSG00000238480	0	0	0	0
ENSG00000238481	0	0	0	0
ENSG00000238482	0	0	0	0
ENSG00000238483	0	0	0	0
ENSG00000238484	0	0	0	0
ENSG00000238485	0	0	0	0
ENSG00000238486	0	0	0	0
ENSG00000238487	0	0	0	0
ENSG00000238488	0	0	0	0
ENSG00000238489	0	0	0	0
ENSG00000238490	0	0	0	0
ENSG00000238491	0	0	0	0
ENSG00000238492	0	0	0	0
ENSG00000238493	0	0	0	0
ENSG00000238494	0	0	0	0
ENSG00000238496	0	0	0	0
ENSG00000238498	0	0	0	0
ENSG00000238500	0	0	0	0
ENSG00000238501	0	0	0	0

ENSG00000238502	0	0	0	0
ENSG00000238503	0	0	0	0
ENSG00000238504	0	0	0	0
ENSG00000238505	0	0	0	0
ENSG00000238506	0	0	0	0
ENSG00000238507	0	0	0	0
ENSG00000238508	0	0	0	0
ENSG00000238509	0	0	0	0
ENSG00000238511	0	0	0	0
ENSG00000238512	0	0	0	0
ENSG00000238513	0	0	0	0
ENSG00000238514	0	0	0	0
ENSG00000238515	0	0	0	0
ENSG00000238516	0	0	0	0
ENSG00000238517	0	0	0	0
ENSG00000238518	0	0	0	0
ENSG00000238519	0	0	0	0
ENSG00000238520	0	0	0	0
ENSG00000238521	0	0	0	0
ENSG00000238522	0	0	0	0
ENSG00000238523	0	0	0	0
ENSG00000238524	0	0	0	0
ENSG00000238525	0	0	0	0
ENSG00000238526	0	0	0	0
ENSG00000238527	0	0	0	0
ENSG00000238528	0	0	0	5
ENSG00000238529	0	0	0	0
ENSG00000238530	0	0	0	0
ENSG00000238531	0	0	0	0
ENSG00000238532	0	0	0	0
ENSG00000238533	0	0	0	0
ENSG00000238534	0	0	0	0
ENSG00000238535	0	0	0	0
ENSG00000238536	0	0	0	0
ENSG00000238537	0	0	0	0
ENSG00000238538	0	0	0	0
ENSG00000238540	0	0	0	0
ENSG00000238541	0	0	0	0
ENSG00000238542	0	0	0	0
ENSG00000238543	0	0	0	0
ENSG00000238544	0	0	0	0
ENSG00000238545	0	0	0	0
ENSG00000238546	0	0	0	0
ENSG00000238547	0	0	0	0
ENSG00000238549	0	0	0	0
ENSG00000238551	0	0	0	0

ENSG00000238552	0	0	0	0
ENSG00000238553	0	0	0	0
ENSG00000238554	0	0	0	0
ENSG00000238556	0	0	0	0
ENSG00000238557	0	0	0	0
ENSG00000238558	0	0	0	0
ENSG00000238559	0	0	0	0
ENSG00000238560	0	0	0	0
ENSG00000238561	0	0	0	0
ENSG00000238562	0	0	0	0
ENSG00000238563	0	0	0	0
ENSG00000238564	0	0	0	0
ENSG00000238565	0	0	0	0
ENSG00000238566	0	0	0	0
ENSG00000238567	0	0	0	0
ENSG00000238568	0	0	0	0
ENSG00000238569	0	0	0	0
ENSG00000238570	0	0	0	0
ENSG00000238571	0	0	0	0
ENSG00000238572	0	0	0	0
ENSG00000238575	0	0	0	0
ENSG00000238576	0	0	0	0
ENSG00000238577	0	0	0	0
ENSG00000238578	0	0	0	0
ENSG00000238579	0	0	0	0
ENSG00000238581	0	0	0	0
ENSG00000238582	0	0	0	0
ENSG00000238583	0	0	0	0
ENSG00000238584	0	0	0	0
ENSG00000238585	0	0	0	0
ENSG00000238587	0	0	0	0
ENSG00000238588	0	0	0	0
ENSG00000238590	0	0	0	0
ENSG00000238591	0	0	0	0
ENSG00000238592	0	0	0	0
ENSG00000238593	0	0	0	0
ENSG00000238594	0	0	0	0
ENSG00000238595	0	0	0	0
ENSG00000238596	0	0	0	0
ENSG00000238597	0	0	0	0
ENSG00000238598	0	0	0	0
ENSG00000238600	0	0	0	0
ENSG00000238601	0	0	0	0
ENSG00000238602	0	0	0	0
ENSG00000238603	0	0	0	0
ENSG00000238604	0	0	0	0

ENSG00000238605	0	0	0	0
ENSG00000238606	0	0	0	0
ENSG00000238607	0	0	0	0
ENSG00000238608	0	0	0	0
ENSG00000238609	0	0	0	0
ENSG00000238610	0	0	0	0
ENSG00000238611	0	0	0	0
ENSG00000238612	0	0	0	0
ENSG00000238615	0	0	0	0
ENSG00000238616	0	0	0	0
ENSG00000238617	0	0	0	0
ENSG00000238618	0	0	0	0
ENSG00000238619	0	0	0	0
ENSG00000238620	0	0	0	0
ENSG00000238621	0	0	0	0
ENSG00000238622	0	0	0	0
ENSG00000238623	0	0	0	0
ENSG00000238624	0	0	0	0
ENSG00000238625	0	0	0	0
ENSG00000238626	0	0	0	0
ENSG00000238627	0	0	0	0
ENSG00000238628	0	0	0	0
ENSG00000238629	0	0	0	0
ENSG00000238630	0	13	0	0
ENSG00000238631	0	0	0	0
ENSG00000238632	0	0	0	0
ENSG00000238634	0	0	0	0
ENSG00000238636	0	0	0	0
ENSG00000238638	0	0	0	0
ENSG00000238639	0	0	0	0
ENSG00000238641	0	0	0	0
ENSG00000238642	0	0	0	0
ENSG00000238643	0	0	0	0
ENSG00000238644	0	0	0	0
ENSG00000238645	0	0	0	0
ENSG00000238646	0	0	0	0
ENSG00000238648	0	0	0	0
ENSG00000238649	0	0	0	0
ENSG00000238650	0	0	0	0
ENSG00000238651	0	0	0	0
ENSG00000238652	0	0	0	0
ENSG00000238653	0	0	0	0
ENSG00000238654	0	0	0	0
ENSG00000238656	0	0	0	0
ENSG00000238657	0	0	0	0
ENSG00000238658	0	0	0	0

ENSG00000238660	0	0	0	0
ENSG00000238661	0	0	0	0
ENSG00000238662	0	0	0	0
ENSG00000238663	0	0	0	0
ENSG00000238664	0	0	0	0
ENSG00000238665	0	0	0	0
ENSG00000238666	0	0	0	0
ENSG00000238667	0	0	0	0
ENSG00000238669	0	0	0	0
ENSG00000238670	0	0	0	0
ENSG00000238671	0	0	0	0
ENSG00000238672	0	0	0	0
ENSG00000238673	0	0	0	0
ENSG00000238674	0	0	0	0
ENSG00000238676	0	0	0	0
ENSG00000238677	0	0	0	0
ENSG00000238678	0	0	0	0
ENSG00000238679	0	0	0	0
ENSG00000238680	0	0	0	0
ENSG00000238682	0	0	0	0
ENSG00000238683	0	0	0	0
ENSG00000238684	0	0	0	0
ENSG00000238685	0	0	0	0
ENSG00000238686	0	0	0	0
ENSG00000238687	0	0	0	0
ENSG00000238688	0	0	0	0
ENSG00000238689	0	0	0	0
ENSG00000238690	0	0	0	0
ENSG00000238691	0	0	0	0
ENSG00000238692	0	0	0	0
ENSG00000238693	0	0	0	0
ENSG00000238694	0	0	0	0
ENSG00000238695	0	0	0	0
ENSG00000238696	0	0	0	0
ENSG00000238697	0	0	0	0
ENSG00000238698	0	0	0	0
ENSG00000238699	0	0	0	0
ENSG00000238701	0	0	0	0
ENSG00000238702	0	0	0	0
ENSG00000238703	0	0	0	0
ENSG00000238704	0	0	0	0
ENSG00000238705	0	0	0	0
ENSG00000238707	0	0	0	0
ENSG00000238708	0	0	0	0
ENSG00000238709	0	0	0	0
ENSG00000238711	0	0	0	0

ENSG00000238712	0	0	0	0
ENSG00000238713	0	0	0	0
ENSG00000238714	0	0	0	0
ENSG00000238715	0	0	0	0
ENSG00000238716	0	0	0	0
ENSG00000238717	0	0	0	0
ENSG00000238718	0	0	0	0
ENSG00000238719	0	0	0	0
ENSG00000238720	0	0	0	0
ENSG00000238721	0	0	0	0
ENSG00000238722	0	0	0	0
ENSG00000238723	0	0	0	0
ENSG00000238724	0	0	0	0
ENSG00000238726	0	0	0	0
ENSG00000238727	0	0	0	0
ENSG00000238728	0	0	0	0
ENSG00000238729	0	0	0	0
ENSG00000238730	0	0	1	0
ENSG00000238731	0	0	0	0
ENSG00000238732	0	0	0	0
ENSG00000238733	0	0	0	0
ENSG00000238734	0	0	0	0
ENSG00000238735	0	0	0	0
ENSG00000238736	18	0	8	0
ENSG00000238737	0	0	0	0
ENSG00000238738	0	0	0	0
ENSG00000238739	0	0	0	0
ENSG00000238740	0	0	0	0
ENSG00000238741	1	0	19	19
ENSG00000238742	0	0	0	0
ENSG00000238744	0	0	0	0
ENSG00000238745	0	0	0	0
ENSG00000238746	0	0	0	0
ENSG00000238747	0	0	0	0
ENSG00000238748	0	0	0	0
ENSG00000238749	0	0	0	0
ENSG00000238750	0	0	0	0
ENSG00000238752	0	0	0	0
ENSG00000238753	0	0	0	0
ENSG00000238754	0	0	0	0
ENSG00000238755	0	0	3	2
ENSG00000238756	0	0	0	0
ENSG00000238758	0	0	0	0
ENSG00000238759	0	0	0	0
ENSG00000238760	0	0	0	0
ENSG00000238761	0	0	0	0

ENSG00000238762	0	0	0	0
ENSG00000238763	0	0	0	0
ENSG00000238764	0	0	0	0
ENSG00000238765	0	0	0	0
ENSG00000238766	0	0	0	0
ENSG00000238767	0	0	0	0
ENSG00000238768	0	0	0	0
ENSG00000238769	0	0	0	0
ENSG00000238770	0	0	0	0
ENSG00000238771	0	0	0	0
ENSG00000238772	0	0	0	0
ENSG00000238773	0	0	0	0
ENSG00000238774	0	0	0	0
ENSG00000238775	0	0	1	0
ENSG00000238776	0	0	0	0
ENSG00000238777	0	0	0	0
ENSG00000238778	0	0	0	0
ENSG00000238779	0	0	0	0
ENSG00000238781	0	0	0	0
ENSG00000238782	0	0	0	0
ENSG00000238783	0	0	0	0
ENSG00000238785	0	0	0	0
ENSG00000238787	0	0	0	0
ENSG00000238788	0	0	0	0
ENSG00000238789	0	0	0	0
ENSG00000238790	0	0	0	0
ENSG00000238791	0	0	0	0
ENSG00000238792	0	0	0	0
ENSG00000238793	0	0	0	0
ENSG00000238795	0	0	0	0
ENSG00000238796	0	0	0	0
ENSG00000238797	0	0	0	0
ENSG00000238798	0	0	0	0
ENSG00000238799	0	0	0	0
ENSG00000238800	0	0	0	0
ENSG00000238801	0	0	0	0
ENSG00000238802	0	0	0	0
ENSG00000238803	0	0	0	0
ENSG00000238804	0	0	0	0
ENSG00000238805	0	0	0	0
ENSG00000238806	0	0	0	0
ENSG00000238807	0	0	0	0
ENSG00000238808	0	0	0	0
ENSG00000238809	0	0	0	0
ENSG00000238810	0	0	0	0
ENSG00000238811	0	0	0	0

ENSG00000238812	0	0	0	0
ENSG00000238813	0	0	0	0
ENSG00000238815	0	0	0	0
ENSG00000238816	0	0	0	0
ENSG00000238818	0	0	0	0
ENSG00000238819	0	0	0	0
ENSG00000238820	0	0	0	0
ENSG00000238821	0	0	0	0
ENSG00000238822	0	0	0	0
ENSG00000238823	0	0	0	0
ENSG00000238824	0	0	0	0
ENSG00000238825	0	0	0	0
ENSG00000238826	0	0	0	0
ENSG00000238827	0	0	0	0
ENSG00000238829	0	0	0	0
ENSG00000238830	0	0	0	0
ENSG00000238831	0	0	0	2
ENSG00000238832	0	0	1	0
ENSG00000238833	0	0	0	0
ENSG00000238834	0	0	0	0
ENSG00000238835	0	0	0	0
ENSG00000238836	0	0	0	0
ENSG00000238837	0	0	0	0
ENSG00000238838	0	0	0	0
ENSG00000238840	0	0	0	0
ENSG00000238841	0	0	0	0
ENSG00000238842	0	0	0	0
ENSG00000238843	0	0	0	0
ENSG00000238844	0	0	0	0
ENSG00000238845	0	0	0	0
ENSG00000238849	0	0	0	0
ENSG00000238850	0	0	0	0
ENSG00000238851	0	0	0	0
ENSG00000238852	0	0	0	0
ENSG00000238853	0	0	0	0
ENSG00000238854	0	0	0	0
ENSG00000238855	0	0	0	0
ENSG00000238856	0	0	0	0
ENSG00000238857	0	0	0	0
ENSG00000238858	0	0	0	0
ENSG00000238859	0	0	0	0
ENSG00000238860	0	0	0	0
ENSG00000238861	0	0	0	0
ENSG00000238862	0	0	0	0
ENSG00000238863	1	0	0	0
ENSG00000238864	0	0	0	0

ENSG00000238865	0	0	0	0
ENSG00000238867	0	0	0	0
ENSG00000238868	0	0	0	0
ENSG00000238869	0	0	0	0
ENSG00000238870	0	0	0	0
ENSG00000238871	0	0	0	0
ENSG00000238872	0	0	0	0
ENSG00000238874	0	0	1	0
ENSG00000238875	0	0	0	0
ENSG00000238876	0	0	0	0
ENSG00000238878	0	0	0	0
ENSG00000238880	0	0	0	0
ENSG00000238881	0	0	0	0
ENSG00000238882	0	0	0	0
ENSG00000238883	0	0	0	0
ENSG00000238884	0	0	0	0
ENSG00000238885	0	0	0	0
ENSG00000238886	0	0	0	0
ENSG00000238887	0	0	0	0
ENSG00000238888	0	0	0	0
ENSG00000238889	0	0	0	0
ENSG00000238890	0	0	0	0
ENSG00000238891	0	0	0	0
ENSG00000238892	0	0	0	0
ENSG00000238893	0	0	0	0
ENSG00000238895	0	0	0	0
ENSG00000238896	0	0	0	0
ENSG00000238898	0	0	0	0
ENSG00000238899	0	0	0	0
ENSG00000238900	0	0	0	0
ENSG00000238901	1	414	387	263
ENSG00000238902	0	0	0	0
ENSG00000238904	0	0	0	0
ENSG00000238905	0	0	0	0
ENSG00000238906	0	0	0	0
ENSG00000238907	0	0	0	0
ENSG00000238908	0	0	0	0
ENSG00000238909	0	0	0	0
ENSG00000238910	0	0	0	0
ENSG00000238911	0	0	0	0
ENSG00000238912	0	0	0	0
ENSG00000238913	0	0	0	0
ENSG00000238914	0	0	0	0
ENSG00000238917	0	0	0	0
ENSG00000238918	0	0	0	0
ENSG00000238919	0	0	1	0

ENSG00000238920	0	0	0	0
ENSG00000238921	0	0	0	0
ENSG00000238922	0	0	0	0
ENSG00000238923	0	0	0	0
ENSG00000238924	0	0	0	0
ENSG00000238925	0	0	0	0
ENSG00000238926	0	0	0	0
ENSG00000238929	0	0	0	0
ENSG00000238930	0	0	0	0
ENSG00000238931	0	0	0	0
ENSG00000238932	0	0	0	0
ENSG00000238933	0	0	0	0
ENSG00000238934	0	0	0	0
ENSG00000238935	0	0	0	0
ENSG00000238936	0	0	0	0
ENSG00000238938	1	0	0	0
ENSG00000238939	0	0	0	0
ENSG00000238940	0	0	0	0
ENSG00000238941	0	0	0	0
ENSG00000238942	0	0	0	0
ENSG00000238943	0	0	0	0
ENSG00000238944	0	0	0	0
ENSG00000238945	0	0	0	0
ENSG00000238946	0	0	0	0
ENSG00000238947	0	0	0	0
ENSG00000238948	0	0	0	0
ENSG00000238949	0	0	0	0
ENSG00000238950	0	0	0	0
ENSG00000238951	0	0	0	0
ENSG00000238952	0	0	0	0
ENSG00000238953	0	0	0	0
ENSG00000238954	0	0	0	0
ENSG00000238957	0	0	0	0
ENSG00000238958	0	0	0	0
ENSG00000238959	0	0	0	0
ENSG00000238960	0	0	0	0
ENSG00000238961	0	0	0	0
ENSG00000238962	0	0	0	0
ENSG00000238963	0	0	0	0
ENSG00000238964	0	0	0	0
ENSG00000238965	0	0	0	0
ENSG00000238966	0	0	0	0
ENSG00000238968	0	0	0	0
ENSG00000238969	0	0	0	0
ENSG00000238970	0	0	0	0
ENSG00000238971	0	0	0	0

ENSG00000238972	0	0	0	0
ENSG00000238974	0	0	0	0
ENSG00000238975	0	0	0	0
ENSG00000238976	0	0	0	0
ENSG00000238977	0	0	0	0
ENSG00000238978	0	0	0	0
ENSG00000238979	0	0	0	0
ENSG00000238980	0	0	0	0
ENSG00000238981	0	0	0	0
ENSG00000238982	0	0	0	0
ENSG00000238983	0	0	0	0
ENSG00000238984	0	0	0	0
ENSG00000238985	0	0	0	0
ENSG00000238986	0	0	0	0
ENSG00000238987	0	0	0	0
ENSG00000238988	0	0	0	0
ENSG00000238991	0	0	0	0
ENSG00000238992	0	0	0	0
ENSG00000238993	0	0	0	0
ENSG00000238994	0	0	0	0
ENSG00000238995	0	0	0	0
ENSG00000238996	0	0	0	0
ENSG00000238997	0	0	0	0
ENSG00000238998	0	0	0	0
ENSG00000238999	0	0	0	0
ENSG00000239000	0	0	0	0
ENSG00000239001	0	0	0	0
ENSG00000239002	0	0	0	0
ENSG00000239003	0	0	0	0
ENSG00000239004	0	0	0	0
ENSG00000239005	0	0	0	0
ENSG00000239006	0	0	0	0
ENSG00000239007	0	0	0	0
ENSG00000239008	0	0	0	0
ENSG00000239010	0	0	0	0
ENSG00000239011	0	0	0	0
ENSG00000239012	0	0	0	0
ENSG00000239013	0	0	0	0
ENSG00000239014	0	0	11	32
ENSG00000239015	0	0	0	0
ENSG00000239017	0	0	0	0
ENSG00000239018	0	0	0	0
ENSG00000239020	0	0	0	0
ENSG00000239021	0	0	0	0
ENSG00000239022	0	0	0	0
ENSG00000239023	0	0	0	0

ENSG00000239024	0	0	0	0
ENSG00000239025	0	0	0	0
ENSG00000239026	0	0	0	0
ENSG00000239027	0	0	0	0
ENSG00000239028	1	0	0	0
ENSG00000239030	0	0	0	0
ENSG00000239031	0	0	0	0
ENSG00000239033	0	0	0	0
ENSG00000239034	0	0	0	0
ENSG00000239035	0	0	0	0
ENSG00000239036	0	0	0	0
ENSG00000239037	0	0	0	0
ENSG00000239038	0	0	0	0
ENSG00000239039	0	0	0	0
ENSG00000239040	0	0	0	0
ENSG00000239041	0	0	0	0
ENSG00000239043	0	0	0	0
ENSG00000239044	0	0	0	0
ENSG00000239045	0	0	0	0
ENSG00000239046	0	0	0	0
ENSG00000239048	0	0	0	0
ENSG00000239049	0	0	0	0
ENSG00000239050	0	0	0	0
ENSG00000239051	0	0	0	0
ENSG00000239052	0	0	0	0
ENSG00000239053	0	0	0	0
ENSG00000239054	0	0	0	0
ENSG00000239055	0	0	0	0
ENSG00000239056	0	0	0	0
ENSG00000239057	0	0	0	0
ENSG00000239058	0	0	0	0
ENSG00000239059	0	0	0	0
ENSG00000239060	0	0	0	0
ENSG00000239061	0	0	0	0
ENSG00000239062	0	0	0	0
ENSG00000239063	0	0	0	0
ENSG00000239064	0	0	0	0
ENSG00000239065	0	0	0	0
ENSG00000239066	0	0	0	0
ENSG00000239067	0	0	0	0
ENSG00000239068	0	0	0	0
ENSG00000239069	0	0	0	0
ENSG00000239070	0	0	0	0
ENSG00000239071	0	0	0	0
ENSG00000239072	0	0	0	0
ENSG00000239073	0	0	0	0

ENSG00000239075	0	0	0	0
ENSG00000239077	0	0	0	0
ENSG00000239078	1	0	0	0
ENSG00000239079	0	0	0	0
ENSG00000239080	0	0	0	0
ENSG00000239081	0	0	0	0
ENSG00000239082	0	0	0	0
ENSG00000239083	0	0	0	0
ENSG00000239084	0	0	0	0
ENSG00000239086	0	0	0	0
ENSG00000239087	0	0	0	0
ENSG00000239088	0	0	0	0
ENSG00000239089	0	0	0	0
ENSG00000239090	0	0	0	0
ENSG00000239091	0	0	0	0
ENSG00000239093	0	0	0	0
ENSG00000239094	0	0	0	0
ENSG00000239095	0	0	0	0
ENSG00000239096	0	0	0	0
ENSG00000239098	0	0	0	0
ENSG00000239099	0	0	0	0
ENSG00000239100	0	0	0	0
ENSG00000239101	0	0	0	0
ENSG00000239102	0	0	0	0
ENSG00000239103	0	0	0	0
ENSG00000239105	0	0	0	0
ENSG00000239106	0	0	0	0
ENSG00000239107	0	0	0	0
ENSG00000239108	0	0	0	0
ENSG00000239111	0	0	0	0
ENSG00000239112	0	0	0	0
ENSG00000239114	0	0	0	0
ENSG00000239115	0	0	0	0
ENSG00000239116	0	0	0	0
ENSG00000239117	0	0	0	0
ENSG00000239118	0	0	0	0
ENSG00000239119	0	0	0	0
ENSG00000239120	0	0	0	0
ENSG00000239121	0	0	0	0
ENSG00000239122	0	0	0	0
ENSG00000239123	0	0	0	0
ENSG00000239124	0	0	0	0
ENSG00000239125	0	0	0	0
ENSG00000239126	0	0	0	0
ENSG00000239127	0	0	0	0
ENSG00000239128	0	0	0	0

ENSG00000239129	0	0	0	0
ENSG00000239130	0	0	0	0
ENSG00000239131	0	0	0	0
ENSG00000239132	0	0	0	0
ENSG00000239133	0	0	0	0
ENSG00000239134	0	0	0	0
ENSG00000239135	0	0	0	0
ENSG00000239136	0	0	0	0
ENSG00000239137	0	0	0	0
ENSG00000239138	0	0	0	0
ENSG00000239140	0	0	0	0
ENSG00000239141	0	0	0	0
ENSG00000239142	0	0	0	0
ENSG00000239143	0	0	0	0
ENSG00000239144	0	0	0	0
ENSG00000239145	0	0	0	0
ENSG00000239146	0	0	0	0
ENSG00000239148	0	0	0	0
ENSG00000239149	0	0	0	0
ENSG00000239150	0	0	0	0
ENSG00000239151	0	0	0	0
ENSG00000239152	0	0	0	0
ENSG00000239153	0	0	0	0
ENSG00000239154	0	0	0	0
ENSG00000239155	0	0	0	0
ENSG00000239156	0	0	0	0
ENSG00000239157	0	0	0	0
ENSG00000239158	0	0	0	0
ENSG00000239159	0	0	0	0
ENSG00000239160	0	0	0	0
ENSG00000239161	0	0	0	0
ENSG00000239163	0	0	0	0
ENSG00000239164	0	0	0	0
ENSG00000239166	0	0	0	0
ENSG00000239167	0	0	0	0
ENSG00000239168	0	0	0	0
ENSG00000239169	0	0	0	0
ENSG00000239170	0	0	0	0
ENSG00000239171	0	0	0	0
ENSG00000239172	0	0	0	0
ENSG00000239173	0	0	0	0
ENSG00000239175	0	0	0	0
ENSG00000239176	0	0	0	0
ENSG00000239178	0	0	0	0
ENSG00000239179	0	0	0	0
ENSG00000239180	0	0	0	0

ENSG00000239181	0	0	0	0
ENSG00000239182	0	0	0	0
ENSG00000239183	0	0	0	0
ENSG00000239184	0	0	0	0
ENSG00000239185	0	0	0	0
ENSG00000239186	0	0	0	0
ENSG00000239187	0	0	0	0
ENSG00000239188	0	0	1	0
ENSG00000239189	0	0	0	0
ENSG00000239190	0	0	0	0
ENSG00000239191	0	0	0	0
ENSG00000239192	0	0	0	0
ENSG00000239193	0	0	0	0
ENSG00000239194	0	0	0	0
ENSG00000239195	0	0	0	0
ENSG00000239196	0	0	0	0
ENSG00000239197	0	0	0	0
ENSG00000239198	0	0	0	0
ENSG00000239199	0	0	0	0
ENSG00000239200	0	0	0	0
ENSG00000239201	0	0	0	0
ENSG00000239202	0	0	0	0
ENSG00000239203	0	0	0	0
ENSG00000239204	0	0	0	0
ENSG00000239205	0	0	0	0
ENSG00000239207	0	0	0	0
ENSG00000239210	0	0	0	0
ENSG00000239211	0	0	0	0
ENSG00000239212	0	0	0	0
ENSG00000239213	311	415	451	596
ENSG00000239215	0	0	0	0
ENSG00000239216	0	0	0	0
ENSG00000239218	0	0	0	0
ENSG00000239219	6	0	7	7
ENSG00000239221	0	0	0	0
ENSG00000239223	0	0	0	0
ENSG00000239224	0	0	0	0
ENSG00000239225	0	0	0	0
ENSG00000239226	0	0	0	0
ENSG00000239227	0	0	0	0
ENSG00000239228	0	0	0	0
ENSG00000239230	0	0	0	0
ENSG00000239235	63	162	255	607
ENSG00000239238	0	0	0	0
ENSG00000239246	0	0	0	0
ENSG00000239247	0	0	0	0

ENSG00000239249	0	0	0	0
ENSG00000239250	0	0	0	0
ENSG00000239253	0	0	0	0
ENSG00000239254	0	0	0	0
ENSG00000239255	0	0	0	0
ENSG00000239256	0	0	0	0
ENSG00000239257	0	0	0	0
ENSG00000239261	0	0	0	0
ENSG00000239263	0	0	0	0
ENSG00000239264	1	26	78	14
ENSG00000239265	1	0	0	0
ENSG00000239268	0	0	0	0
ENSG00000239269	0	0	0	0
ENSG00000239272	0	0	0	0
ENSG00000239275	0	0	0	0
ENSG00000239279	0	0	0	0
ENSG00000239280	0	0	0	0
ENSG00000239281	0	0	0	0
ENSG00000239282	0	0	0	0
ENSG00000239285	0	0	0	0
ENSG00000239288	0	0	1	0
ENSG00000239291	0	0	0	0
ENSG00000239293	0	0	0	0
ENSG00000239300	0	0	0	1
ENSG00000239304	0	0	0	0
ENSG00000239305	25	210	324	82
ENSG00000239306	28	115	33	30
ENSG00000239311	0	0	0	0
ENSG00000239314	0	0	0	0
ENSG00000239315	0	0	0	0
ENSG00000239316	0	0	0	0
ENSG00000239317	0	0	0	0
ENSG00000239319	0	0	0	0
ENSG00000239320	0	0	0	0
ENSG00000239322	0	0	0	0
ENSG00000239323	0	0	0	0
ENSG00000239327	0	0	0	0
ENSG00000239329	0	0	0	0
ENSG00000239332	2	0	1	0
ENSG00000239333	0	0	0	0
ENSG00000239334	0	0	0	0
ENSG00000239335	0	0	0	0
ENSG00000239337	0	0	0	0
ENSG00000239344	0	0	0	0
ENSG00000239345	0	0	0	0
ENSG00000239350	0	0	0	0

ENSG00000239351	0	0	0	0
ENSG00000239353	0	0	0	0
ENSG00000239354	0	0	0	0
ENSG00000239356	0	0	0	0
ENSG00000239365	0	0	0	0
ENSG00000239367	1	0	0	0
ENSG00000239372	0	0	0	2
ENSG00000239373	0	0	0	0
ENSG00000239374	0	0	0	0
ENSG00000239377	0	0	0	1
ENSG00000239381	0	0	0	0
ENSG00000239382	1	0	7	1
ENSG00000239383	0	0	0	0
ENSG00000239388	2	0	3	5
ENSG00000239389	0	0	0	0
ENSG00000239390	0	0	0	0
ENSG00000239392	0	0	0	0
ENSG00000239393	0	0	0	0
ENSG00000239395	0	0	0	0
ENSG00000239396	0	0	0	0
ENSG00000239397	0	0	0	0
ENSG00000239398	0	0	0	0
ENSG00000239402	0	0	0	0
ENSG00000239405	1	30	23	17
ENSG00000239407	2	228	170	280
ENSG00000239408	0	0	0	0
ENSG00000239412	0	0	0	0
ENSG00000239413	0	0	0	0
ENSG00000239415	0	0	0	0
ENSG00000239419	0	0	0	0
ENSG00000239421	0	0	0	0
ENSG00000239426	0	0	0	0
ENSG00000239428	0	0	0	0
ENSG00000239429	0	0	0	0
ENSG00000239432	0	0	0	0
ENSG00000239435	4	3	2	7
ENSG00000239437	0	0	0	0
ENSG00000239438	0	0	0	0
ENSG00000239439	0	0	0	0
ENSG00000239440	0	0	1	0
ENSG00000239443	0	0	0	0
ENSG00000239445	27	47	166	178
ENSG00000239446	0	0	0	0
ENSG00000239453	0	0	0	0
ENSG00000239454	0	0	0	0
ENSG00000239455	0	0	0	0

ENSG00000239457	0	0	0	0
ENSG00000239462	0	0	0	0
ENSG00000239463	0	0	0	0
ENSG00000239464	0	0	0	0
ENSG00000239465	0	0	0	0
ENSG00000239466	0	0	0	0
ENSG00000239467	0	0	0	0
ENSG00000239468	0	0	0	0
ENSG00000239470	3	0	0	0
ENSG00000239471	0	0	0	0
ENSG00000239472	0	0	0	0
ENSG00000239473	2	0	5	2
ENSG00000239474	0	0	0	1
ENSG00000239475	0	0	0	0
ENSG00000239480	2	0	1	3
ENSG00000239481	0	0	0	0
ENSG00000239482	0	0	1	0
ENSG00000239483	0	0	0	0
ENSG00000239486	5	0	0	0
ENSG00000239490	0	0	0	0
ENSG00000239492	0	0	0	0
ENSG00000239494	0	0	0	0
ENSG00000239497	0	0	0	0
ENSG00000239498	0	0	0	0
ENSG00000239500	0	0	0	0
ENSG00000239501	0	0	0	0
ENSG00000239503	0	0	0	0
ENSG00000239504	0	0	0	0
ENSG00000239508	0	0	0	0
ENSG00000239510	0	0	0	0
ENSG00000239511	0	0	0	0
ENSG00000239513	0	0	0	0
ENSG00000239516	0	0	0	0
ENSG00000239517	0	0	0	0
ENSG00000239519	0	0	0	1
ENSG00000239520	0	0	0	0
ENSG00000239521	8	0	7	3
ENSG00000239523	3	109	93	52
ENSG00000239524	0	0	0	0
ENSG00000239525	0	0	0	0
ENSG00000239527	0	0	0	0
ENSG00000239528	0	0	0	0
ENSG00000239532	0	0	0	0
ENSG00000239533	0	0	0	0
ENSG00000239539	0	0	0	0
ENSG00000239542	0	0	0	0

ENSG00000239544	0	0	0	0
ENSG00000239545	0	0	0	0
ENSG00000239547	0	0	0	0
ENSG00000239552	0	0	0	0
ENSG00000239553	0	0	0	0
ENSG00000239555	0	0	0	0
ENSG00000239556	0	0	0	2
ENSG00000239557	0	0	0	0
ENSG00000239559	2	0	0	0
ENSG00000239560	0	0	0	0
ENSG00000239568	0	0	0	0
ENSG00000239569	109	0	8	2
ENSG00000239570	0	0	3	8
ENSG00000239571	0	0	0	0
ENSG00000239572	0	0	0	1
ENSG00000239576	0	0	0	0
ENSG00000239577	0	0	0	0
ENSG00000239579	0	0	0	0
ENSG00000239580	0	0	0	0
ENSG00000239581	0	0	0	0
ENSG00000239586	0	0	0	0
ENSG00000239587	0	0	0	0
ENSG00000239589	0	0	0	0
ENSG00000239590	0	0	0	1
ENSG00000239593	1	0	0	3
ENSG00000239594	0	0	0	0
ENSG00000239595	0	0	0	0
ENSG00000239600	0	0	0	0
ENSG00000239602	0	0	0	0
ENSG00000239605	0	0	0	0
ENSG00000239607	0	0	0	0
ENSG00000239608	0	0	0	0
ENSG00000239610	0	0	0	0
ENSG00000239614	0	0	2	0
ENSG00000239615	0	0	0	0
ENSG00000239617	0	0	0	0
ENSG00000239618	0	0	0	0
ENSG00000239620	0	0	0	0
ENSG00000239622	0	0	0	0
ENSG00000239624	0	0	0	0
ENSG00000239625	0	0	0	0
ENSG00000239626	0	0	0	0
ENSG00000239627	0	0	0	0
ENSG00000239628	0	0	0	0
ENSG00000239632	0	0	0	0
ENSG00000239636	0	0	1	0

ENSG00000239640	0	0	0	0
ENSG00000239641	0	0	0	0
ENSG00000239642	0	0	0	0
ENSG00000239648	0	0	0	0
ENSG00000239649	0	0	0	0
ENSG00000239650	0	0	7	24
ENSG00000239652	0	0	0	0
ENSG00000239653	0	38	6	33
ENSG00000239659	0	0	0	0
ENSG00000239661	0	0	0	0
ENSG00000239664	0	0	0	0
ENSG00000239665	1	0	3	4
ENSG00000239666	0	0	0	0
ENSG00000239670	2	0	0	0
ENSG00000239671	0	0	0	0
ENSG00000239672	14	0	4	5
ENSG00000239674	0	0	0	0
ENSG00000239675	0	0	0	0
ENSG00000239677	0	0	0	0
ENSG00000239679	0	0	0	0
ENSG00000239683	0	0	0	0
ENSG00000239684	0	0	0	0
ENSG00000239685	0	0	0	0
ENSG00000239686	0	0	0	0
ENSG00000239688	0	0	0	0
ENSG00000239689	0	0	0	0
ENSG00000239690	0	0	0	0
ENSG00000239694	0	0	0	0
ENSG00000239696	0	0	0	0
ENSG00000239697	17	0	1	0
ENSG00000239699	0	0	0	0
ENSG00000239701	0	0	0	0
ENSG00000239702	0	0	0	0
ENSG00000239703	0	0	0	0
ENSG00000239704	0	0	0	0
ENSG00000239705	1	0	0	0
ENSG00000239706	0	0	0	0
ENSG00000239708	0	0	0	0
ENSG00000239710	0	0	0	0
ENSG00000239713	68	14	19	17
ENSG00000239715	0	0	0	0
ENSG00000239716	0	0	0	0
ENSG00000239718	0	27	1	0
ENSG00000239719	0	0	0	0
ENSG00000239722	0	0	0	0
ENSG00000239726	0	0	0	0

ENSG00000239731	0	0	0	0
ENSG00000239732	0	0	0	0
ENSG00000239736	0	0	0	2
ENSG00000239739	0	0	0	0
ENSG00000239741	0	0	0	0
ENSG00000239742	0	0	0	0
ENSG00000239744	0	0	0	0
ENSG00000239745	0	0	0	0
ENSG00000239748	0	0	0	0
ENSG00000239754	0	0	0	0
ENSG00000239756	0	0	0	0
ENSG00000239763	1	0	2	2
ENSG00000239767	0	0	0	0
ENSG00000239769	0	0	0	0
ENSG00000239774	0	0	0	4
ENSG00000239775	0	0	0	0
ENSG00000239776	0	0	0	0
ENSG00000239779	2	1	1	22
ENSG00000239780	0	0	0	2
ENSG00000239783	0	0	0	0
ENSG00000239784	0	0	0	0
ENSG00000239789	7	0	0	0
ENSG00000239791	0	0	0	0
ENSG00000239792	0	0	0	0
ENSG00000239793	0	0	0	0
ENSG00000239794	0	0	0	0
ENSG00000239797	0	0	0	0
ENSG00000239799	0	0	0	0
ENSG00000239800	0	0	0	0
ENSG00000239801	27	47	11	33
ENSG00000239804	0	0	0	0
ENSG00000239805	0	0	0	0
ENSG00000239808	0	0	0	0
ENSG00000239809	0	0	0	0
ENSG00000239810	0	0	0	0
ENSG00000239815	0	0	0	0
ENSG00000239819	0	0	0	0
ENSG00000239820	0	0	0	0
ENSG00000239821	0	0	0	0
ENSG00000239822	0	0	0	0
ENSG00000239823	0	0	0	0
ENSG00000239825	0	0	0	0
ENSG00000239827	1	58	94	166
ENSG00000239828	0	20	19	86
ENSG00000239829	0	0	0	0
ENSG00000239830	0	0	0	0

ENSG00000239831	0	0	0	0
ENSG00000239835	0	0	0	0
ENSG00000239836	0	0	0	0
ENSG00000239839	27	2	0	30
ENSG00000239840	0	0	0	5
ENSG00000239855	0	0	0	0
ENSG00000239856	0	0	0	0
ENSG00000239857	0	0	0	0
ENSG00000239859	0	0	0	0
ENSG00000239861	0	0	0	0
ENSG00000239862	0	0	0	0
ENSG00000239865	0	0	0	0
ENSG00000239867	0	0	0	0
ENSG00000239868	1	0	0	0
ENSG00000239870	0	0	0	0
ENSG00000239872	0	0	0	0
ENSG00000239873	0	0	0	0
ENSG00000239877	0	0	2	0
ENSG00000239880	0	0	0	0
ENSG00000239881	0	0	0	0
ENSG00000239882	0	0	0	0
ENSG00000239883	0	0	0	10
ENSG00000239884	0	0	0	0
ENSG00000239886	0	0	0	0
ENSG00000239887	0	0	0	1
ENSG00000239888	0	0	0	0
ENSG00000239891	0	0	0	0
ENSG00000239893	0	0	0	0
ENSG00000239898	0	0	0	0
ENSG00000239899	0	71	39	168
ENSG00000239900	22	126	70	117
ENSG00000239903	0	0	0	0
ENSG00000239906	0	3	11	11
ENSG00000239908	0	0	0	2
ENSG00000239910	0	0	0	0
ENSG00000239911	2	0	0	0
ENSG00000239912	0	0	1	0
ENSG00000239917	0	0	0	0
ENSG00000239919	0	0	0	0
ENSG00000239920	0	0	2	0
ENSG00000239921	0	0	0	0
ENSG00000239922	0	0	0	0
ENSG00000239923	0	0	0	0
ENSG00000239924	0	0	0	0
ENSG00000239926	0	0	0	0
ENSG00000239927	0	0	0	0

ENSG00000239930	4	3	5	0
ENSG00000239932	0	0	0	0
ENSG00000239939	0	0	0	0
ENSG00000239940	0	0	0	0
ENSG00000239941	24	0	6	16
ENSG00000239942	0	0	0	0
ENSG00000239944	0	0	6	6
ENSG00000239945	0	0	0	0
ENSG00000239946	0	0	0	0
ENSG00000239948	0	0	0	0
ENSG00000239951	1	0	0	0
ENSG00000239953	1	0	0	0
ENSG00000239955	0	0	0	0
ENSG00000239958	0	0	0	0
ENSG00000239959	1	0	0	0
ENSG00000239961	3	0	0	0
ENSG00000239964	0	0	0	0
ENSG00000239967	0	0	0	0
ENSG00000239968	0	0	0	0
ENSG00000239969	0	0	0	3
ENSG00000239974	0	0	0	0
ENSG00000239975	0	0	0	0
ENSG00000239978	0	0	0	0
ENSG00000239981	0	0	0	0
ENSG00000239983	0	0	0	0
ENSG00000239984	0	0	0	0
ENSG00000239985	0	0	0	0
ENSG00000239986	0	0	0	0
ENSG00000239988	0	0	0	0
ENSG00000239989	0	0	0	0
ENSG00000239991	0	0	0	0
ENSG00000239992	0	0	0	0
ENSG00000239994	0	0	0	0
ENSG00000239995	0	0	0	0
ENSG00000239997	0	0	0	1
ENSG00000239998	15	0	0	0
ENSG00000240002	0	0	0	0
ENSG00000240003	2	0	10	25
ENSG00000240005	164	29	14	67
ENSG00000240006	0	0	0	0
ENSG00000240007	0	0	0	0
ENSG00000240008	0	0	0	0
ENSG00000240010	0	0	0	0
ENSG00000240012	0	0	0	0
ENSG00000240014	0	0	0	0
ENSG00000240015	0	0	0	0

ENSG00000240021	0	0	0	0
ENSG00000240023	0	0	0	0
ENSG00000240024	4	22	6	27
ENSG00000240027	0	0	0	0
ENSG00000240031	0	0	0	0
ENSG00000240032	1	0	26	24
ENSG00000240033	0	0	0	0
ENSG00000240034	0	0	0	0
ENSG00000240036	1	0	0	1
ENSG00000240038	0	17	31	52
ENSG00000240040	0	0	0	0
ENSG00000240041	0	0	0	0
ENSG00000240042	0	0	0	0
ENSG00000240043	0	0	0	0
ENSG00000240045	0	0	0	0
ENSG00000240047	0	0	0	0
ENSG00000240048	0	0	0	0
ENSG00000240050	0	0	0	0
ENSG00000240051	0	0	0	0
ENSG00000240052	0	0	0	0
ENSG00000240053	0	0	0	0
ENSG00000240056	0	0	0	0
ENSG00000240057	0	0	3	5
ENSG00000240058	0	0	0	0
ENSG00000240063	3	0	1	0
ENSG00000240065	683	673	219	364
ENSG00000240068	0	0	0	0
ENSG00000240069	0	0	0	0
ENSG00000240074	0	0	0	0
ENSG00000240083	0	0	0	0
ENSG00000240084	0	0	0	0
ENSG00000240086	0	0	0	0
ENSG00000240087	0	2	2	0
ENSG00000240089	0	0	0	0
ENSG00000240093	0	0	0	0
ENSG00000240095	0	0	0	0
ENSG00000240096	0	0	0	0
ENSG00000240097	0	0	0	0
ENSG00000240098	0	0	0	0
ENSG00000240100	0	0	0	0
ENSG00000240103	0	0	0	0
ENSG00000240106	0	0	0	0
ENSG00000240107	0	0	0	0
ENSG00000240108	0	0	0	0
ENSG00000240110	0	0	0	0
ENSG00000240111	0	0	0	0

ENSG00000240115	0	0	0	0
ENSG00000240116	0	0	0	0
ENSG00000240118	0	0	0	0
ENSG00000240121	0	0	0	0
ENSG00000240122	0	0	0	1
ENSG00000240125	0	0	0	0
ENSG00000240126	0	0	0	0
ENSG00000240127	0	0	0	0
ENSG00000240128	0	0	0	0
ENSG00000240130	0	0	0	0
ENSG00000240131	0	0	0	0
ENSG00000240132	0	0	0	0
ENSG00000240135	0	0	0	0
ENSG00000240137	1	0	4	11
ENSG00000240138	0	0	0	0
ENSG00000240143	0	0	0	0
ENSG00000240151	0	0	0	0
ENSG00000240152	0	0	0	0
ENSG00000240156	0	0	0	0
ENSG00000240159	0	0	0	0
ENSG00000240160	0	0	0	0
ENSG00000240163	0	0	0	0
ENSG00000240165	0	0	0	0
ENSG00000240167	0	0	0	0
ENSG00000240173	0	0	0	0
ENSG00000240174	0	0	0	0
ENSG00000240175	0	0	0	0
ENSG00000240179	0	0	0	0
ENSG00000240180	0	0	0	0
ENSG00000240183	0	0	0	0
ENSG00000240184	0	0	0	0
ENSG00000240186	0	0	0	0
ENSG00000240189	0	0	0	0
ENSG00000240194	0	0	0	0
ENSG00000240197	0	0	0	0
ENSG00000240198	0	0	0	0
ENSG00000240199	0	0	0	0
ENSG00000240200	0	1	4	1
ENSG00000240201	0	0	0	0
ENSG00000240202	0	0	0	0
ENSG00000240203	0	0	0	0
ENSG00000240204	8	16	17	32
ENSG00000240205	0	0	0	0
ENSG00000240207	50	492	171	353
ENSG00000240210	0	0	0	0
ENSG00000240211	6	1	6	7

ENSG00000240215	2	20	6	5
ENSG00000240216	1	0	0	0
ENSG00000240219	0	0	1	0
ENSG00000240221	0	0	0	0
ENSG00000240224	0	0	0	0
ENSG00000240225	1039	6573	6959	5238
ENSG00000240230	34	0	3	0
ENSG00000240231	0	0	0	0
ENSG00000240233	0	0	0	0
ENSG00000240235	0	0	0	0
ENSG00000240236	0	0	0	0
ENSG00000240237	0	0	0	0
ENSG00000240238	0	0	0	0
ENSG00000240240	0	0	2	0
ENSG00000240241	0	0	0	0
ENSG00000240244	0	0	0	0
ENSG00000240246	0	0	0	0
ENSG00000240247	0	0	0	0
ENSG00000240250	0	0	0	0
ENSG00000240253	0	0	0	0
ENSG00000240254	0	0	5	0
ENSG00000240255	0	0	0	0
ENSG00000240256	0	0	0	0
ENSG00000240257	0	0	0	0
ENSG00000240258	0	0	0	0
ENSG00000240265	0	0	0	0
ENSG00000240268	0	0	0	0
ENSG00000240270	0	0	0	0
ENSG00000240271	0	0	0	0
ENSG00000240279	0	0	0	0
ENSG00000240280	0	0	0	0
ENSG00000240281	0	0	0	0
ENSG00000240283	2	1	5	9
ENSG00000240284	0	0	0	0
ENSG00000240286	0	0	0	0
ENSG00000240288	0	0	0	0
ENSG00000240289	0	0	0	0
ENSG00000240291	14	12	5	2
ENSG00000240292	0	0	0	0
ENSG00000240293	0	0	0	0
ENSG00000240294	0	0	0	0
ENSG00000240295	0	0	0	0
ENSG00000240296	0	0	0	0
ENSG00000240298	0	0	0	0
ENSG00000240299	0	0	0	0
ENSG00000240302	0	0	0	0

ENSG00000240303	0	0	0	1
ENSG00000240305	0	0	0	0
ENSG00000240306	0	0	0	0
ENSG00000240309	0	0	0	0
ENSG00000240310	0	0	0	0
ENSG00000240311	0	0	0	0
ENSG00000240317	0	0	0	0
ENSG00000240320	0	0	0	0
ENSG00000240322	0	0	0	0
ENSG00000240327	0	0	0	0
ENSG00000240328	0	0	0	0
ENSG00000240338	0	0	0	0
ENSG00000240341	0	0	0	0
ENSG00000240342	0	12	0	0
ENSG00000240344	16	201	167	333
ENSG00000240347	0	0	0	0
ENSG00000240350	4	20	5	1
ENSG00000240354	0	0	0	0
ENSG00000240355	0	0	0	0
ENSG00000240356	1843	13051	2964	16712
ENSG00000240359	0	0	0	0
ENSG00000240361	0	0	0	0
ENSG00000240364	0	0	0	0
ENSG00000240366	3	0	1	0
ENSG00000240368	0	17	9	6
ENSG00000240370	36	11	11	41
ENSG00000240371	0	0	0	0
ENSG00000240373	0	0	0	0
ENSG00000240374	0	0	0	0
ENSG00000240375	0	0	0	0
ENSG00000240376	0	0	0	0
ENSG00000240379	0	0	0	0
ENSG00000240382	0	0	0	0
ENSG00000240385	0	0	0	0
ENSG00000240386	0	0	0	0
ENSG00000240388	0	0	0	0
ENSG00000240389	0	0	0	0
ENSG00000240392	0	0	0	0
ENSG00000240393	0	0	0	0
ENSG00000240395	0	0	0	0
ENSG00000240396	0	0	0	0
ENSG00000240399	0	0	1	0
ENSG00000240401	0	22	18	15
ENSG00000240403	2	0	0	0
ENSG00000240404	0	0	0	0
ENSG00000240405	0	0	0	6

ENSG00000240409	11	0	1	1
ENSG00000240411	0	0	0	0
ENSG00000240412	0	0	0	0
ENSG00000240416	0	0	0	0
ENSG00000240418	0	0	0	0
ENSG00000240419	0	0	0	0
ENSG00000240423	0	0	0	0
ENSG00000240424	0	0	0	0
ENSG00000240426	0	0	0	0
ENSG00000240427	0	0	0	0
ENSG00000240428	2	13	1	13
ENSG00000240429	2	0	1	8
ENSG00000240430	0	0	0	0
ENSG00000240432	0	0	0	0
ENSG00000240433	0	0	0	0
ENSG00000240435	0	0	0	0
ENSG00000240436	0	0	0	0
ENSG00000240438	0	0	0	0
ENSG00000240439	0	0	0	0
ENSG00000240440	0	0	0	1
ENSG00000240441	0	0	0	0
ENSG00000240443	0	0	0	0
ENSG00000240445	0	0	3	0
ENSG00000240449	0	0	0	0
ENSG00000240450	0	0	0	0
ENSG00000240452	0	0	0	0
ENSG00000240453	0	0	0	0
ENSG00000240454	0	1	0	0
ENSG00000240455	0	0	0	0
ENSG00000240456	4	0	1	1
ENSG00000240457	0	0	0	0
ENSG00000240458	0	0	0	0
ENSG00000240459	0	0	0	0
ENSG00000240463	1	0	0	0
ENSG00000240466	0	0	0	0
ENSG00000240468	0	0	0	0
ENSG00000240470	0	0	0	0
ENSG00000240471	0	0	1	0
ENSG00000240474	0	0	0	0
ENSG00000240475	0	0	0	0
ENSG00000240476	0	0	0	0
ENSG00000240477	0	0	0	0
ENSG00000240478	0	0	0	0
ENSG00000240480	0	0	0	0
ENSG00000240481	0	0	0	0
ENSG00000240484	0	0	0	0

ENSG00000240486	0	0	0	0
ENSG00000240487	0	0	0	0
ENSG00000240489	0	2	0	0
ENSG00000240490	0	0	0	0
ENSG00000240494	0	0	0	0
ENSG00000240497	1159	1042	1093	1279
ENSG00000240498	0	0	0	3
ENSG00000240499	0	0	0	0
ENSG00000240502	0	0	0	0
ENSG00000240505	2	0	0	0
ENSG00000240508	0	0	0	0
ENSG00000240509	1	0	0	0
ENSG00000240510	0	0	0	0
ENSG00000240511	0	0	0	0
ENSG00000240513	0	0	0	0
ENSG00000240518	0	0	0	0
ENSG00000240519	0	0	0	0
ENSG00000240520	0	0	0	0
ENSG00000240521	0	0	0	0
ENSG00000240522	0	0	0	0
ENSG00000240524	0	27	22	14
ENSG00000240527	0	0	0	0
ENSG00000240529	0	0	1	6
ENSG00000240531	0	0	0	0
ENSG00000240533	0	0	0	0
ENSG00000240534	0	0	0	0
ENSG00000240535	2	0	0	0
ENSG00000240540	0	0	0	0
ENSG00000240541	1	0	0	1
ENSG00000240542	0	0	0	0
ENSG00000240545	0	0	0	0
ENSG00000240549	0	0	0	0
ENSG00000240553	0	0	0	0
ENSG00000240554	0	0	0	0
ENSG00000240556	0	0	0	0
ENSG00000240562	0	0	0	0
ENSG00000240563	4	0	0	0
ENSG00000240566	0	0	0	0
ENSG00000240567	0	0	0	0
ENSG00000240568	0	0	0	0
ENSG00000240571	0	33	29	15
ENSG00000240572	0	0	0	0
ENSG00000240573	0	0	0	0
ENSG00000240577	0	0	0	0
ENSG00000240578	0	0	0	0
ENSG00000240579	0	0	0	0

ENSG00000240583	42	112	73	182
ENSG00000240584	0	0	0	0
ENSG00000240589	0	0	0	0
ENSG00000240590	0	0	0	0
ENSG00000240591	0	0	1	4
ENSG00000240592	0	0	0	0
ENSG00000240596	0	0	0	0
ENSG00000240601	0	0	0	0
ENSG00000240602	0	0	0	0
ENSG00000240606	0	0	0	0
ENSG00000240611	0	0	0	0
ENSG00000240613	0	0	0	0
ENSG00000240616	0	0	0	0
ENSG00000240618	0	0	5	5
ENSG00000240620	0	0	0	0
ENSG00000240621	0	0	0	0
ENSG00000240622	2	0	0	0
ENSG00000240624	0	0	0	0
ENSG00000240625	0	0	0	0
ENSG00000240626	0	0	0	0
ENSG00000240627	0	0	0	0
ENSG00000240631	0	0	0	0
ENSG00000240632	0	0	0	0
ENSG00000240634	0	0	0	0
ENSG00000240637	0	0	0	0
ENSG00000240639	0	0	0	0
ENSG00000240641	0	0	0	0
ENSG00000240647	0	0	0	0
ENSG00000240651	0	1	0	0
ENSG00000240652	0	0	0	0
ENSG00000240654	0	0	0	1
ENSG00000240661	0	0	0	0
ENSG00000240663	0	0	0	0
ENSG00000240665	0	0	0	0
ENSG00000240666	0	0	0	0
ENSG00000240667	0	0	0	0
ENSG00000240668	0	0	0	0
ENSG00000240669	0	0	0	0
ENSG00000240671	0	0	0	0
ENSG00000240673	0	0	0	0
ENSG00000240674	2	0	0	1
ENSG00000240677	0	0	0	0
ENSG00000240680	0	0	0	0
ENSG00000240682	57	23	16	28
ENSG00000240684	0	0	0	0
ENSG00000240685	6	25	7	14

ENSG00000240687	0	0	3	0
ENSG00000240688	0	0	0	0
ENSG00000240692	0	0	0	0
ENSG00000240694	0	0	0	0
ENSG00000240695	1	0	9	14
ENSG00000240698	0	0	0	0
ENSG00000240704	0	0	0	0
ENSG00000240707	0	0	0	0
ENSG00000240708	0	0	0	0
ENSG00000240710	0	0	0	0
ENSG00000240712	0	0	0	0
ENSG00000240713	0	0	0	0
ENSG00000240718	0	0	0	0
ENSG00000240720	0	0	0	0
ENSG00000240721	0	0	0	0
ENSG00000240723	0	0	0	0
ENSG00000240724	0	0	0	0
ENSG00000240729	2	0	0	0
ENSG00000240730	0	0	0	0
ENSG00000240731	0	0	0	0
ENSG00000240733	0	0	0	0
ENSG00000240738	0	0	0	0
ENSG00000240739	0	0	0	0
ENSG00000240742	0	0	0	0
ENSG00000240747	0	0	1	0
ENSG00000240750	0	0	0	0
ENSG00000240751	0	0	0	0
ENSG00000240752	0	0	0	0
ENSG00000240754	0	0	0	0
ENSG00000240755	0	0	0	0
ENSG00000240758	0	0	0	0
ENSG00000240759	2	0	0	0
ENSG00000240760	0	0	0	0
ENSG00000240761	0	0	0	0
ENSG00000240764	0	0	0	0
ENSG00000240766	0	0	0	0
ENSG00000240767	0	0	0	0
ENSG00000240770	0	0	0	0
ENSG00000240771	0	0	1	0
ENSG00000240772	0	0	0	0
ENSG00000240774	0	0	0	0
ENSG00000240775	0	0	0	0
ENSG00000240776	0	0	0	0
ENSG00000240777	0	0	0	0
ENSG00000240779	0	0	0	0
ENSG00000240785	0	0	0	0

ENSG00000240787	0	0	0	0
ENSG00000240789	0	0	0	0
ENSG00000240790	0	0	0	0
ENSG00000240791	0	0	0	0
ENSG00000240792	0	0	0	0
ENSG00000240793	0	0	0	0
ENSG00000240796	0	0	0	0
ENSG00000240800	0	0	0	0
ENSG00000240801	0	0	0	0
ENSG00000240803	0	0	0	0
ENSG00000240804	0	0	0	0
ENSG00000240808	0	0	0	0
ENSG00000240809	0	0	2	0
ENSG00000240813	0	0	0	0
ENSG00000240820	0	0	0	0
ENSG00000240821	0	0	0	0
ENSG00000240823	0	0	0	0
ENSG00000240824	0	0	0	0
ENSG00000240825	0	0	0	0
ENSG00000240827	0	0	0	0
ENSG00000240828	0	0	0	0
ENSG00000240833	0	0	0	0
ENSG00000240834	0	0	0	0
ENSG00000240837	0	0	0	0
ENSG00000240842	0	0	0	0
ENSG00000240845	0	0	0	0
ENSG00000240846	0	0	0	0
ENSG00000240847	0	0	0	0
ENSG00000240849	520	956	1079	1178
ENSG00000240853	0	0	0	0
ENSG00000240854	0	0	0	0
ENSG00000240857	3	0	6	9
ENSG00000240859	0	0	0	0
ENSG00000240861	0	0	0	0
ENSG00000240863	0	0	0	0
ENSG00000240864	0	0	0	0
ENSG00000240868	0	0	0	0
ENSG00000240869	0	5	1	7
ENSG00000240870	0	0	0	0
ENSG00000240871	0	0	0	0
ENSG00000240873	0	0	0	0
ENSG00000240874	0	0	0	0
ENSG00000240875	1	0	13	27
ENSG00000240877	0	0	0	0
ENSG00000240881	0	0	0	0
ENSG00000240882	0	0	0	0

ENSG00000240888	0	0	0	0
ENSG00000240889	0	0	0	0
ENSG00000240890	0	0	0	0
ENSG00000240891	0	0	0	1
ENSG00000240893	0	0	1	0
ENSG00000240895	0	0	0	0
ENSG00000240898	0	0	0	0
ENSG00000240902	0	0	0	0
ENSG00000240905	0	0	0	0
ENSG00000240906	3	0	3	1
ENSG00000240907	0	0	0	0
ENSG00000240912	0	0	0	0
ENSG00000240913	0	0	0	0
ENSG00000240914	0	0	0	0
ENSG00000240915	0	0	0	0
ENSG00000240919	0	0	0	4
ENSG00000240922	0	0	0	0
ENSG00000240924	0	0	0	0
ENSG00000240925	0	0	0	0
ENSG00000240927	0	0	0	0
ENSG00000240929	0	0	1	0
ENSG00000240934	0	0	0	0
ENSG00000240935	3	21	1	0
ENSG00000240936	0	0	0	0
ENSG00000240940	0	0	0	0
ENSG00000240950	0	0	0	0
ENSG00000240951	0	0	0	0
ENSG00000240954	0	0	0	0
ENSG00000240957	0	0	0	0
ENSG00000240959	0	0	0	0
ENSG00000240960	0	0	0	0
ENSG00000240961	0	0	0	0
ENSG00000240962	0	0	0	0
ENSG00000240963	0	0	0	0
ENSG00000240964	0	0	0	0
ENSG00000240966	0	0	0	0
ENSG00000240970	0	0	0	0
ENSG00000240972	0	0	8	5
ENSG00000240973	0	0	0	0
ENSG00000240974	0	0	0	0
ENSG00000240975	0	0	0	0
ENSG00000240977	0	0	0	0
ENSG00000240979	0	0	0	0
ENSG00000240980	0	0	0	0
ENSG00000240983	0	0	0	0
ENSG00000240990	0	0	0	0

ENSG00000240991	0	0	0	0
ENSG00000240992	0	0	0	0
ENSG00000240993	0	0	0	0
ENSG00000240994	0	0	0	0
ENSG00000240995	0	0	0	0
ENSG00000240996	1	0	0	1
ENSG00000240997	0	0	0	0
ENSG00000241000	0	0	0	0
ENSG00000241002	0	0	0	0
ENSG00000241003	0	0	0	0
ENSG00000241007	0	0	0	0
ENSG00000241008	0	0	0	0
ENSG00000241011	0	0	0	0
ENSG00000241014	4	0	3	2
ENSG00000241015	11	7	2	16
ENSG00000241018	0	0	0	0
ENSG00000241020	0	0	0	0
ENSG00000241022	0	0	0	0
ENSG00000241026	0	0	0	0
ENSG00000241030	0	0	0	0
ENSG00000241032	0	0	0	0
ENSG00000241034	0	0	0	0
ENSG00000241035	0	0	0	0
ENSG00000241037	0	0	0	0
ENSG00000241042	0	0	0	0
ENSG00000241043	10	12	14	24
ENSG00000241045	0	0	0	0
ENSG00000241048	0	0	0	0
ENSG00000241052	0	0	0	0
ENSG00000241053	0	0	0	0
ENSG00000241054	0	0	0	0
ENSG00000241057	0	0	0	0
ENSG00000241058	15	795	755	1926
ENSG00000241059	0	0	0	0
ENSG00000241061	0	0	0	0
ENSG00000241064	0	0	0	0
ENSG00000241067	0	0	0	0
ENSG00000241069	0	0	0	0
ENSG00000241073	0	0	0	0
ENSG00000241074	0	0	0	0
ENSG00000241075	0	0	0	0
ENSG00000241081	0	0	0	0
ENSG00000241082	0	0	0	0
ENSG00000241084	0	0	0	0
ENSG00000241088	0	0	0	0
ENSG00000241095	0	0	4	0

ENSG00000241097	0	0	0	0
ENSG00000241098	0	0	0	0
ENSG00000241101	0	0	0	0
ENSG00000241102	0	0	0	0
ENSG00000241103	0	0	0	0
ENSG00000241104	0	0	0	0
ENSG00000241105	0	0	0	0
ENSG00000241106	2	0	0	0
ENSG00000241107	0	0	0	4
ENSG00000241111	0	2	1	0
ENSG00000241112	0	9	1	1
ENSG00000241114	0	0	1	0
ENSG00000241119	0	0	0	0
ENSG00000241120	0	0	0	0
ENSG00000241123	1	0	0	0
ENSG00000241124	0	0	0	0
ENSG00000241125	0	0	0	0
ENSG00000241127	2	0	0	3
ENSG00000241128	0	0	0	0
ENSG00000241129	0	0	0	0
ENSG00000241130	0	0	0	0
ENSG00000241131	0	0	0	0
ENSG00000241132	0	0	0	0
ENSG00000241134	0	0	0	0
ENSG00000241135	0	0	0	0
ENSG00000241136	0	0	0	0
ENSG00000241143	0	0	0	0
ENSG00000241144	0	0	0	0
ENSG00000241145	0	0	0	0
ENSG00000241146	0	0	0	0
ENSG00000241149	0	0	0	0
ENSG00000241150	0	0	0	0
ENSG00000241151	0	0	0	0
ENSG00000241152	0	0	0	0
ENSG00000241155	2	0	1	11
ENSG00000241156	0	0	0	0
ENSG00000241157	0	0	1	0
ENSG00000241158	0	0	0	0
ENSG00000241159	0	0	0	0
ENSG00000241162	0	0	0	0
ENSG00000241163	4	0	0	0
ENSG00000241166	0	0	0	0
ENSG00000241167	0	0	0	0
ENSG00000241168	0	0	2	6
ENSG00000241169	0	0	0	0
ENSG00000241170	1	0	1	2

ENSG00000241172	0	0	0	0
ENSG00000241174	0	0	0	0
ENSG00000241175	0	0	0	0
ENSG00000241177	0	0	0	0
ENSG00000241179	0	0	0	0
ENSG00000241180	0	0	0	0
ENSG00000241181	0	0	0	0
ENSG00000241183	0	0	0	0
ENSG00000241185	0	0	0	7
ENSG00000241186	0	0	0	0
ENSG00000241187	0	0	0	0
ENSG00000241188	0	0	0	0
ENSG00000241190	0	0	0	0
ENSG00000241198	0	0	0	0
ENSG00000241200	0	0	0	0
ENSG00000241202	0	0	0	0
ENSG00000241203	0	0	0	0
ENSG00000241204	0	0	0	0
ENSG00000241207	0	0	0	0
ENSG00000241211	0	0	0	0
ENSG00000241213	0	0	0	0
ENSG00000241216	0	0	0	0
ENSG00000241217	0	0	0	0
ENSG00000241218	3	0	0	0
ENSG00000241219	0	0	0	0
ENSG00000241220	2	0	1	12
ENSG00000241221	0	0	0	0
ENSG00000241223	0	0	0	0
ENSG00000241224	0	0	0	0
ENSG00000241225	0	0	0	0
ENSG00000241226	0	0	0	0
ENSG00000241227	0	0	0	0
ENSG00000241228	0	0	0	0
ENSG00000241229	0	0	0	0
ENSG00000241230	0	0	0	0
ENSG00000241231	0	0	0	0
ENSG00000241233	0	0	0	0
ENSG00000241238	0	0	0	0
ENSG00000241241	0	0	0	0
ENSG00000241243	0	0	0	0
ENSG00000241244	0	0	0	0
ENSG00000241246	0	0	0	0
ENSG00000241247	0	31	15	13
ENSG00000241248	0	0	0	0
ENSG00000241250	0	0	0	0
ENSG00000241251	0	0	0	0

ENSG00000241253	0	0	0	0
ENSG00000241254	0	0	0	0
ENSG00000241255	0	0	1	0
ENSG00000241257	0	0	0	0
ENSG00000241258	18	3	16	21
ENSG00000241261	0	0	0	0
ENSG00000241266	0	0	0	0
ENSG00000241269	0	0	0	0
ENSG00000241273	0	0	0	0
ENSG00000241278	0	0	0	0
ENSG00000241280	2	0	0	0
ENSG00000241281	0	0	0	0
ENSG00000241282	0	0	0	0
ENSG00000241286	0	0	0	0
ENSG00000241287	0	0	0	0
ENSG00000241288	0	0	2	4
ENSG00000241291	0	0	0	0
ENSG00000241293	0	0	0	0
ENSG00000241294	0	0	0	0
ENSG00000241295	0	0	0	0
ENSG00000241296	0	0	0	0
ENSG00000241305	0	0	0	0
ENSG00000241307	0	0	0	0
ENSG00000241313	1	0	0	0
ENSG00000241316	1	0	0	2
ENSG00000241317	0	0	0	0
ENSG00000241318	0	0	0	0
ENSG00000241319	0	0	0	0
ENSG00000241322	0	0	0	0
ENSG00000241324	0	0	0	0
ENSG00000241326	0	0	0	0
ENSG00000241328	0	0	0	0
ENSG00000241333	0	0	0	0
ENSG00000241334	0	0	0	0
ENSG00000241335	0	0	0	0
ENSG00000241336	0	0	0	0
ENSG00000241340	0	0	0	0
ENSG00000241341	0	0	0	0
ENSG00000241343	19	21	39	23
ENSG00000241344	0	0	0	0
ENSG00000241345	0	0	0	0
ENSG00000241346	0	0	0	0
ENSG00000241347	0	0	0	0
ENSG00000241350	0	0	1	0
ENSG00000241351	0	0	0	0
ENSG00000241352	0	0	0	0

ENSG00000241353	0	0	0	0
ENSG00000241354	0	0	0	0
ENSG00000241356	0	0	0	0
ENSG00000241357	0	0	2	0
ENSG00000241358	0	0	0	0
ENSG00000241359	0	0	0	0
ENSG00000241360	0	0	0	0
ENSG00000241361	0	0	0	4
ENSG00000241362	0	0	0	0
ENSG00000241367	0	0	0	0
ENSG00000241369	0	0	4	0
ENSG00000241370	0	0	0	0
ENSG00000241381	0	0	0	0
ENSG00000241383	0	0	0	0
ENSG00000241385	0	0	1	0
ENSG00000241386	0	0	0	0
ENSG00000241388	0	0	0	0
ENSG00000241391	0	0	0	0
ENSG00000241392	0	0	0	0
ENSG00000241394	0	0	0	0
ENSG00000241395	0	0	0	0
ENSG00000241397	0	0	0	0
ENSG00000241399	16	0	15	17
ENSG00000241400	0	0	0	0
ENSG00000241404	1	0	0	0
ENSG00000241406	0	0	0	0
ENSG00000241408	0	0	0	0
ENSG00000241409	0	1	8	2
ENSG00000241411	0	0	0	0
ENSG00000241413	0	0	0	0
ENSG00000241416	0	0	0	0
ENSG00000241418	0	0	0	0
ENSG00000241420	0	0	0	0
ENSG00000241423	0	0	0	0
ENSG00000241429	0	0	0	0
ENSG00000241431	0	0	0	0
ENSG00000241434	0	0	0	0
ENSG00000241438	0	0	0	0
ENSG00000241439	0	0	0	0
ENSG00000241449	0	0	0	0
ENSG00000241451	0	0	0	0
ENSG00000241456	0	0	0	0
ENSG00000241457	0	0	0	0
ENSG00000241458	0	0	0	0
ENSG00000241459	0	0	0	0
ENSG00000241461	0	0	0	0

ENSG00000241462	0	0	0	0
ENSG00000241464	0	0	0	0
ENSG00000241465	0	0	0	0
ENSG00000241468	470	54	60	142
ENSG00000241469	21	118	47	86
ENSG00000241471	0	0	0	0
ENSG00000241472	0	0	0	1
ENSG00000241473	0	0	0	0
ENSG00000241475	0	0	0	0
ENSG00000241476	0	0	0	0
ENSG00000241478	0	0	0	0
ENSG00000241479	0	0	0	0
ENSG00000241481	0	0	0	0
ENSG00000241484	0	0	0	0
ENSG00000241487	0	0	0	0
ENSG00000241489	0	0	0	0
ENSG00000241490	1	0	0	0
ENSG00000241493	0	0	0	0
ENSG00000241494	0	0	0	0
ENSG00000241499	0	0	0	0
ENSG00000241500	0	0	0	0
ENSG00000241505	0	0	0	0
ENSG00000241506	0	0	2	2
ENSG00000241511	0	0	0	0
ENSG00000241520	5	19	2	24
ENSG00000241522	0	0	0	0
ENSG00000241524	0	0	0	0
ENSG00000241525	0	12	16	12
ENSG00000241526	0	0	0	0
ENSG00000241527	0	0	0	0
ENSG00000241528	0	0	0	0
ENSG00000241529	2	2	2	12
ENSG00000241532	16	1	104	528
ENSG00000241534	0	0	0	0
ENSG00000241535	0	0	0	0
ENSG00000241536	0	0	0	0
ENSG00000241537	0	0	0	0
ENSG00000241539	0	0	0	0
ENSG00000241541	0	0	0	0
ENSG00000241542	0	0	0	0
ENSG00000241544	0	0	0	0
ENSG00000241546	0	0	0	0
ENSG00000241547	0	0	2	10
ENSG00000241549	1	58	13	18
ENSG00000241550	0	0	0	0
ENSG00000241552	0	0	0	0

ENSG00000241553	2352	1159	725	993
ENSG00000241556	0	0	0	0
ENSG00000241558	0	0	0	0
ENSG00000241559	0	0	0	0
ENSG00000241560	0	0	0	2
ENSG00000241562	0	0	0	0
ENSG00000241563	0	0	0	0
ENSG00000241566	0	0	0	0
ENSG00000241568	0	0	0	0
ENSG00000241570	1	0	0	0
ENSG00000241571	0	0	0	0
ENSG00000241572	0	0	0	0
ENSG00000241573	0	0	0	0
ENSG00000241577	0	0	0	0
ENSG00000241582	0	0	0	0
ENSG00000241587	0	0	0	0
ENSG00000241588	0	0	0	0
ENSG00000241590	0	0	0	0
ENSG00000241592	0	0	0	0
ENSG00000241593	0	0	6	6
ENSG00000241595	0	0	0	0
ENSG00000241596	0	0	0	0
ENSG00000241597	0	0	0	0
ENSG00000241598	0	0	0	0
ENSG00000241599	0	0	0	0
ENSG00000241601	0	0	0	0
ENSG00000241604	0	0	0	0
ENSG00000241607	0	0	0	0
ENSG00000241609	0	0	0	0
ENSG00000241612	1	0	0	0
ENSG00000241613	0	0	0	0
ENSG00000241621	0	0	0	0
ENSG00000241622	0	0	4	0
ENSG00000241624	0	0	0	0
ENSG00000241625	0	0	0	0
ENSG00000241626	0	0	0	0
ENSG00000241627	0	0	0	0
ENSG00000241631	0	0	0	0
ENSG00000241634	0	0	0	0
ENSG00000241635	0	0	0	0
ENSG00000241636	0	0	0	0
ENSG00000241640	0	0	0	0
ENSG00000241641	0	0	0	0
ENSG00000241644	5	0	2	3
ENSG00000241648	0	0	0	0
ENSG00000241651	0	0	0	0

ENSG00000241652	0	0	0	0
ENSG00000241654	0	0	0	0
ENSG00000241656	0	0	0	0
ENSG00000241657	0	0	0	0
ENSG00000241661	0	0	0	0
ENSG00000241665	0	0	0	0
ENSG00000241666	0	0	0	0
ENSG00000241667	0	0	0	0
ENSG00000241668	0	0	0	0
ENSG00000241669	2	0	8	0
ENSG00000241670	0	0	0	0
ENSG00000241671	0	0	0	0
ENSG00000241673	0	0	0	0
ENSG00000241674	0	0	0	0
ENSG00000241678	0	0	0	0
ENSG00000241679	0	0	15	1
ENSG00000241680	0	0	2	12
ENSG00000241684	0	0	0	0
ENSG00000241685	218	683	810	1340
ENSG00000241690	0	0	0	0
ENSG00000241693	0	0	0	0
ENSG00000241695	0	0	0	0
ENSG00000241696	0	0	0	0
ENSG00000241697	0	0	0	0
ENSG00000241701	0	0	0	0
ENSG00000241708	0	0	0	0
ENSG00000241709	0	0	0	0
ENSG00000241710	0	0	0	0
ENSG00000241713	0	0	0	0
ENSG00000241717	0	2	9	6
ENSG00000241720	0	0	3	0
ENSG00000241721	0	0	0	0
ENSG00000241722	0	0	0	0
ENSG00000241723	0	0	0	0
ENSG00000241728	0	0	0	0
ENSG00000241732	116	1096	2211	890
ENSG00000241735	17	36	18	22
ENSG00000241737	0	0	0	0
ENSG00000241738	0	0	0	0
ENSG00000241739	0	0	0	0
ENSG00000241741	0	0	0	0
ENSG00000241743	0	0	3	0
ENSG00000241744	0	0	0	0
ENSG00000241745	0	0	0	0
ENSG00000241746	0	0	0	0
ENSG00000241749	0	0	0	0

ENSG00000241754	0	0	0	0
ENSG00000241755	0	16	1	0
ENSG00000241756	0	0	0	0
ENSG00000241757	0	0	0	0
ENSG00000241764	47	32	30	29
ENSG00000241765	0	0	0	0
ENSG00000241767	0	0	0	0
ENSG00000241769	3	0	4	10
ENSG00000241770	0	0	0	0
ENSG00000241772	0	0	0	0
ENSG00000241774	0	0	0	0
ENSG00000241776	0	0	0	0
ENSG00000241777	0	0	0	0
ENSG00000241778	0	0	0	0
ENSG00000241779	0	0	0	0
ENSG00000241781	0	0	0	0
ENSG00000241782	0	0	0	0
ENSG00000241784	0	0	1	0
ENSG00000241785	0	0	0	0
ENSG00000241787	0	0	0	0
ENSG00000241788	0	0	0	0
ENSG00000241789	0	0	0	0
ENSG00000241790	0	0	0	0
ENSG00000241791	0	0	0	0
ENSG00000241792	0	0	0	0
ENSG00000241794	0	0	0	0
ENSG00000241804	0	0	0	0
ENSG00000241807	0	0	0	0
ENSG00000241808	0	0	0	0
ENSG00000241809	0	0	0	0
ENSG00000241810	0	0	1	0
ENSG00000241811	0	0	0	0
ENSG00000241815	0	0	0	0
ENSG00000241818	0	0	0	0
ENSG00000241821	0	0	0	0
ENSG00000241822	0	0	0	0
ENSG00000241825	0	0	0	0
ENSG00000241828	0	0	0	0
ENSG00000241829	0	0	0	0
ENSG00000241831	0	0	0	0
ENSG00000241832	0	0	0	0
ENSG00000241834	0	0	0	0
ENSG00000241837	133	27	12	26
ENSG00000241838	0	0	0	0
ENSG00000241839	210	187	160	95
ENSG00000241840	0	0	0	0

ENSG00000241846	0	0	0	0
ENSG00000241847	0	0	13	3
ENSG00000241850	0	0	0	0
ENSG00000241852	4	0	1	2
ENSG00000241853	0	0	0	0
ENSG00000241859	3	97	1	123
ENSG00000241860	1	55	45	95
ENSG00000241861	0	0	0	0
ENSG00000241863	0	0	0	0
ENSG00000241866	0	0	0	0
ENSG00000241868	0	0	0	0
ENSG00000241869	0	0	0	0
ENSG00000241870	0	0	0	0
ENSG00000241874	0	0	0	0
ENSG00000241877	0	0	0	0
ENSG00000241878	18	59	22	42
ENSG00000241879	0	0	0	0
ENSG00000241880	0	0	0	0
ENSG00000241881	0	0	0	0
ENSG00000241882	0	0	0	0
ENSG00000241884	0	0	0	0
ENSG00000241886	4	100	41	114
ENSG00000241888	0	0	0	0
ENSG00000241889	0	0	0	0
ENSG00000241890	0	0	0	0
ENSG00000241891	0	0	0	0
ENSG00000241899	0	0	0	0
ENSG00000241905	0	0	0	0
ENSG00000241907	0	0	0	0
ENSG00000241910	0	0	0	0
ENSG00000241911	0	0	0	0
ENSG00000241912	1	0	2	5
ENSG00000241913	0	0	0	0
ENSG00000241916	0	0	0	0
ENSG00000241917	0	0	0	0
ENSG00000241921	0	0	0	0
ENSG00000241923	0	0	5	0
ENSG00000241926	0	0	0	0
ENSG00000241929	0	0	0	0
ENSG00000241932	0	0	0	0
ENSG00000241933	0	0	0	0
ENSG00000241935	11	0	0	0
ENSG00000241939	0	0	0	0
ENSG00000241941	0	0	0	0
ENSG00000241942	0	0	0	0
ENSG00000241943	0	0	0	0

ENSG00000241945	1	0	0	7
ENSG00000241946	0	0	0	0
ENSG00000241947	0	0	0	0
ENSG00000241950	0	0	0	0
ENSG00000241954	0	0	0	0
ENSG00000241956	8	3	7	10
ENSG00000241959	0	0	0	0
ENSG00000241961	0	0	0	0
ENSG00000241962	4	38	3	2
ENSG00000241963	0	0	0	0
ENSG00000241964	0	0	0	0
ENSG00000241965	0	0	0	0
ENSG00000241973	66	562	260	608
ENSG00000241975	0	0	0	0
ENSG00000241978	0	0	2	0
ENSG00000241981	0	0	0	0
ENSG00000241983	0	0	0	0
ENSG00000241984	0	0	0	0
ENSG00000241985	0	0	0	0
ENSG00000241990	37	0	5	4
ENSG00000241991	0	0	0	0
ENSG00000241992	0	0	0	0
ENSG00000241993	0	0	0	0
ENSG00000241997	0	0	0	0
ENSG00000242001	0	0	0	0
ENSG00000242009	0	23	53	14
ENSG00000242010	2	0	0	0
ENSG00000242012	0	0	0	0
ENSG00000242013	6	0	0	1
ENSG00000242014	0	0	0	0
ENSG00000242017	0	0	0	0
ENSG00000242019	0	0	0	0
ENSG00000242020	0	0	0	0
ENSG00000242021	0	0	0	0
ENSG00000242022	0	0	0	0
ENSG00000242026	0	0	0	0
ENSG00000242028	0	0	0	0
ENSG00000242029	0	0	0	1
ENSG00000242034	0	0	0	0
ENSG00000242036	0	0	0	0
ENSG00000242037	0	0	0	0
ENSG00000242038	0	0	0	0
ENSG00000242039	0	0	0	0
ENSG00000242041	0	0	0	0
ENSG00000242042	0	0	0	0
ENSG00000242044	0	0	0	0

ENSG00000242048	1	0	0	0
ENSG00000242049	0	0	0	0
ENSG00000242052	0	0	0	0
ENSG00000242058	0	0	0	2
ENSG00000242060	0	0	0	0
ENSG00000242061	0	0	0	0
ENSG00000242062	0	0	0	0
ENSG00000242065	0	0	0	0
ENSG00000242066	0	0	0	0
ENSG00000242067	0	0	0	0
ENSG00000242068	0	0	0	0
ENSG00000242070	0	0	0	0
ENSG00000242071	0	1	0	2
ENSG00000242072	0	0	0	0
ENSG00000242073	0	0	0	0
ENSG00000242076	0	0	0	0
ENSG00000242078	0	0	0	0
ENSG00000242079	0	0	0	0
ENSG00000242080	0	0	0	0
ENSG00000242082	0	0	1	0
ENSG00000242083	0	0	0	0
ENSG00000242085	0	0	0	0
ENSG00000242086	2	76	340	1056
ENSG00000242087	0	0	0	0
ENSG00000242088	1	0	0	0
ENSG00000242092	0	0	0	0
ENSG00000242094	0	0	0	4
ENSG00000242095	0	0	0	0
ENSG00000242097	0	0	0	0
ENSG00000242100	0	0	0	0
ENSG00000242101	0	0	0	0
ENSG00000242102	0	0	0	0
ENSG00000242103	0	0	0	0
ENSG00000242104	0	0	0	0
ENSG00000242107	0	0	0	0
ENSG00000242109	0	0	0	0
ENSG00000242110	0	0	2	0
ENSG00000242111	0	0	0	0
ENSG00000242113	0	0	0	0
ENSG00000242114	0	0	0	0
ENSG00000242115	0	0	0	0
ENSG00000242118	0	0	0	0
ENSG00000242119	0	0	0	0
ENSG00000242120	0	0	0	0
ENSG00000242121	0	0	0	0
ENSG00000242123	0	0	0	0

ENSG00000242125	100	89	71	89
ENSG00000242128	0	0	0	0
ENSG00000242134	0	0	0	0
ENSG00000242135	0	0	0	0
ENSG00000242136	0	0	0	0
ENSG00000242137	0	0	0	0
ENSG00000242138	0	0	0	0
ENSG00000242140	0	0	0	0
ENSG00000242142	0	0	0	0
ENSG00000242145	0	0	0	0
ENSG00000242147	0	0	0	0
ENSG00000242150	0	0	0	0
ENSG00000242151	0	0	0	0
ENSG00000242152	0	0	1	0
ENSG00000242153	0	0	0	0
ENSG00000242154	0	0	0	0
ENSG00000242156	0	0	0	0
ENSG00000242158	0	0	0	0
ENSG00000242159	0	0	0	0
ENSG00000242162	0	0	0	0
ENSG00000242163	0	0	13	4
ENSG00000242165	0	0	0	0
ENSG00000242169	0	0	0	0
ENSG00000242170	0	0	0	0
ENSG00000242173	0	0	0	0
ENSG00000242175	0	0	0	0
ENSG00000242176	0	0	0	0
ENSG00000242178	0	0	0	0
ENSG00000242180	0	0	0	0
ENSG00000242182	0	0	0	0
ENSG00000242185	0	0	0	0
ENSG00000242186	0	0	0	0
ENSG00000242188	0	0	0	0
ENSG00000242190	0	0	0	0
ENSG00000242193	51	243	108	205
ENSG00000242194	0	0	0	0
ENSG00000242195	0	0	0	0
ENSG00000242197	0	0	0	0
ENSG00000242198	0	0	0	0
ENSG00000242199	0	0	0	0
ENSG00000242201	0	0	0	0
ENSG00000242206	0	0	0	0
ENSG00000242207	0	0	0	0
ENSG00000242208	0	0	0	0
ENSG00000242209	0	0	0	0
ENSG00000242214	0	0	0	0

ENSG00000242216	0	0	0	0
ENSG00000242218	0	0	0	0
ENSG00000242220	0	0	0	0
ENSG00000242221	0	0	0	0
ENSG00000242222	0	0	0	0
ENSG00000242227	0	0	0	0
ENSG00000242229	0	0	0	0
ENSG00000242236	0	0	0	0
ENSG00000242241	0	0	0	0
ENSG00000242242	0	0	0	0
ENSG00000242244	0	0	0	0
ENSG00000242246	0	0	0	0
ENSG00000242247	56	836	764	789
ENSG00000242248	0	0	0	1
ENSG00000242251	0	0	0	0
ENSG00000242252	0	0	0	0
ENSG00000242255	0	0	0	0
ENSG00000242256	0	0	0	0
ENSG00000242258	2	0	0	15
ENSG00000242259	200	258	312	485
ENSG00000242261	0	0	0	0
ENSG00000242262	0	0	1	0
ENSG00000242265	1	0	2	0
ENSG00000242266	0	0	0	0
ENSG00000242267	0	0	0	0
ENSG00000242268	0	0	0	2
ENSG00000242272	0	0	0	0
ENSG00000242276	0	0	0	0
ENSG00000242278	0	0	0	0
ENSG00000242279	0	0	0	0
ENSG00000242280	0	0	0	0
ENSG00000242281	0	0	0	0
ENSG00000242282	49	53	7	19
ENSG00000242284	0	0	0	0
ENSG00000242285	0	0	0	0
ENSG00000242286	0	0	0	0
ENSG00000242288	0	2	38	36
ENSG00000242289	0	0	0	0
ENSG00000242290	0	0	0	0
ENSG00000242291	0	0	0	0
ENSG00000242292	0	0	0	0
ENSG00000242293	0	0	0	0
ENSG00000242294	2	0	0	0
ENSG00000242295	0	0	0	0
ENSG00000242296	0	0	0	0
ENSG00000242299	1	0	3	1

ENSG00000242300	0	0	0	0
ENSG00000242301	0	0	0	0
ENSG00000242307	0	0	0	0
ENSG00000242308	0	0	0	0
ENSG00000242311	0	0	0	0
ENSG00000242314	0	0	0	0
ENSG00000242315	0	0	0	0
ENSG00000242317	0	0	0	0
ENSG00000242318	0	0	0	0
ENSG00000242320	0	0	0	0
ENSG00000242321	0	0	0	0
ENSG00000242324	0	0	0	0
ENSG00000242325	0	0	0	0
ENSG00000242326	0	0	0	0
ENSG00000242327	0	0	0	0
ENSG00000242329	0	0	0	0
ENSG00000242330	0	0	0	0
ENSG00000242335	0	0	0	0
ENSG00000242337	3	0	6	1
ENSG00000242338	0	0	0	0
ENSG00000242339	0	0	0	0
ENSG00000242341	0	0	0	0
ENSG00000242348	0	0	0	0
ENSG00000242349	402	159	66	300
ENSG00000242352	0	0	0	0
ENSG00000242353	0	0	0	0
ENSG00000242357	0	0	0	0
ENSG00000242358	0	0	0	0
ENSG00000242360	0	0	0	0
ENSG00000242361	0	0	0	0
ENSG00000242362	0	0	0	0
ENSG00000242364	0	0	0	0
ENSG00000242365	0	0	0	0
ENSG00000242366	0	0	0	0
ENSG00000242370	0	0	0	0
ENSG00000242371	0	0	0	0
ENSG00000242372	31	4	2	3
ENSG00000242375	0	0	0	0
ENSG00000242379	0	0	0	0
ENSG00000242381	0	0	0	0
ENSG00000242384	0	0	0	0
ENSG00000242385	0	0	0	0
ENSG00000242386	0	0	0	0
ENSG00000242387	0	0	0	0
ENSG00000242389	0	0	0	0
ENSG00000242390	0	0	0	0

ENSG00000242391	0	0	0	0
ENSG00000242393	0	0	0	0
ENSG00000242396	0	0	0	0
ENSG00000242398	0	0	1	0
ENSG00000242399	0	0	0	0
ENSG00000242405	0	0	0	0
ENSG00000242407	0	0	0	0
ENSG00000242411	0	0	0	0
ENSG00000242412	0	0	0	0
ENSG00000242417	0	0	0	0
ENSG00000242419	0	0	0	0
ENSG00000242421	0	0	0	0
ENSG00000242423	0	0	0	0
ENSG00000242425	0	0	0	0
ENSG00000242428	0	0	3	0
ENSG00000242429	0	0	0	0
ENSG00000242430	0	0	0	0
ENSG00000242431	0	0	0	0
ENSG00000242435	0	0	0	0
ENSG00000242436	0	0	0	0
ENSG00000242439	0	0	0	0
ENSG00000242440	1	0	0	0
ENSG00000242441	0	0	0	0
ENSG00000242443	0	0	0	0
ENSG00000242444	0	0	0	0
ENSG00000242445	1	0	0	0
ENSG00000242456	0	0	0	0
ENSG00000242457	0	0	0	0
ENSG00000242461	0	0	0	0
ENSG00000242463	0	0	0	0
ENSG00000242472	0	0	0	0
ENSG00000242473	0	0	0	0
ENSG00000242474	3	0	0	1
ENSG00000242477	0	0	0	0
ENSG00000242479	0	0	0	0
ENSG00000242480	0	0	0	0
ENSG00000242482	0	0	0	0
ENSG00000242483	0	0	0	0
ENSG00000242485	57	92	49	73
ENSG00000242486	0	0	0	0
ENSG00000242488	0	0	0	0
ENSG00000242493	0	0	0	0
ENSG00000242498	3	0	0	0
ENSG00000242500	0	0	0	0
ENSG00000242502	0	0	0	0
ENSG00000242507	0	0	0	0

ENSG00000242509	0	0	0	0
ENSG00000242510	0	0	0	0
ENSG00000242511	0	0	0	0
ENSG00000242512	0	6	0	0
ENSG00000242515	0	0	0	0
ENSG00000242516	2	12	5	61
ENSG00000242518	0	0	0	0
ENSG00000242520	0	0	0	0
ENSG00000242522	2	0	0	0
ENSG00000242524	0	0	0	0
ENSG00000242525	0	0	0	0
ENSG00000242527	0	0	0	0
ENSG00000242529	0	0	0	0
ENSG00000242531	0	0	0	0
ENSG00000242534	0	0	0	0
ENSG00000242536	0	0	0	1
ENSG00000242537	0	0	0	0
ENSG00000242539	0	2	19	1
ENSG00000242540	0	0	0	0
ENSG00000242542	0	0	0	0
ENSG00000242545	0	0	0	0
ENSG00000242547	0	0	1	1
ENSG00000242550	3	3	0	12
ENSG00000242551	0	0	0	4
ENSG00000242552	0	0	0	0
ENSG00000242553	0	0	0	0
ENSG00000242557	0	0	5	3
ENSG00000242559	0	0	0	0
ENSG00000242560	0	0	0	0
ENSG00000242561	0	0	0	0
ENSG00000242562	0	0	0	0
ENSG00000242565	0	0	0	0
ENSG00000242568	0	15	0	0
ENSG00000242569	1	15	5	13
ENSG00000242571	0	0	0	0
ENSG00000242573	0	0	0	0
ENSG00000242574	4	0	3	0
ENSG00000242575	0	0	0	0
ENSG00000242576	0	0	0	0
ENSG00000242578	0	0	0	0
ENSG00000242580	0	0	0	0
ENSG00000242583	0	0	0	0
ENSG00000242586	0	0	0	0
ENSG00000242588	24	1646	1378	1743
ENSG00000242590	0	0	0	0
ENSG00000242593	0	0	0	0

ENSG00000242595	0	0	0	0
ENSG00000242598	0	0	0	0
ENSG00000242599	0	0	0	0
ENSG00000242600	0	0	0	0
ENSG00000242602	0	0	0	0
ENSG00000242607	0	0	0	0
ENSG00000242608	0	0	0	0
ENSG00000242609	0	0	0	0
ENSG00000242610	0	0	0	0
ENSG00000242611	88	82	17	340
ENSG00000242612	0	0	0	0
ENSG00000242613	0	0	0	0
ENSG00000242614	0	0	0	0
ENSG00000242615	3	5	13	8
ENSG00000242616	0	0	0	0
ENSG00000242618	0	0	2	0
ENSG00000242620	0	0	0	0
ENSG00000242622	3	22	24	11
ENSG00000242628	1	0	0	0
ENSG00000242629	130	375	369	521
ENSG00000242631	0	0	0	0
ENSG00000242634	0	0	0	0
ENSG00000242635	0	0	0	0
ENSG00000242636	0	0	0	0
ENSG00000242638	0	0	0	0
ENSG00000242639	0	0	0	0
ENSG00000242640	0	0	0	0
ENSG00000242641	0	0	0	2
ENSG00000242650	0	0	0	0
ENSG00000242651	0	0	0	0
ENSG00000242653	0	0	0	0
ENSG00000242654	0	0	0	0
ENSG00000242657	0	0	0	0
ENSG00000242659	0	0	0	1
ENSG00000242660	0	0	0	0
ENSG00000242661	0	0	0	0
ENSG00000242663	0	0	0	0
ENSG00000242667	0	0	0	0
ENSG00000242668	0	0	0	0
ENSG00000242670	0	0	0	0
ENSG00000242671	0	0	0	0
ENSG00000242673	0	0	0	0
ENSG00000242675	0	0	0	0
ENSG00000242676	0	0	0	0
ENSG00000242683	0	0	0	0
ENSG00000242685	0	0	0	0

ENSG00000242686	0	0	0	0
ENSG00000242687	7	36	15	21
ENSG00000242688	0	0	0	0
ENSG00000242689	0	0	12	2
ENSG00000242692	0	0	0	0
ENSG00000242696	0	0	0	0
ENSG00000242697	0	0	0	0
ENSG00000242699	0	0	0	0
ENSG00000242703	0	0	0	0
ENSG00000242705	0	0	0	0
ENSG00000242706	0	0	0	0
ENSG00000242707	0	0	0	0
ENSG00000242709	0	0	0	0
ENSG00000242711	0	0	0	0
ENSG00000242712	0	0	0	0
ENSG00000242715	0	0	1	1
ENSG00000242716	0	0	0	0
ENSG00000242719	0	0	0	0
ENSG00000242726	0	0	0	0
ENSG00000242727	0	0	0	0
ENSG00000242728	0	0	0	0
ENSG00000242729	0	0	0	0
ENSG00000242731	1	0	5	3
ENSG00000242732	1	0	1	0
ENSG00000242735	0	0	0	0
ENSG00000242736	0	0	0	0
ENSG00000242737	0	0	0	0
ENSG00000242741	0	0	0	0
ENSG00000242747	0	0	0	0
ENSG00000242748	0	0	0	0
ENSG00000242752	0	0	0	0
ENSG00000242753	0	0	0	0
ENSG00000242756	0	1	7	33
ENSG00000242757	0	0	0	0
ENSG00000242759	1	10	0	11
ENSG00000242764	0	0	0	0
ENSG00000242766	0	0	0	0
ENSG00000242767	0	0	0	0
ENSG00000242768	0	0	0	0
ENSG00000242769	0	0	0	0
ENSG00000242770	1	3	0	0
ENSG00000242771	0	0	0	0
ENSG00000242774	0	0	0	0
ENSG00000242775	0	0	0	4
ENSG00000242779	6	36	42	47
ENSG00000242781	0	0	7	0

ENSG00000242790	0	0	0	0
ENSG00000242791	1	0	0	0
ENSG00000242793	0	0	0	0
ENSG00000242794	0	0	0	0
ENSG00000242795	0	0	7	2
ENSG00000242797	0	0	0	0
ENSG00000242798	0	0	0	0
ENSG00000242802	2	0	1	3
ENSG00000242807	0	0	0	0
ENSG00000242808	0	0	1	0
ENSG00000242810	0	0	0	0
ENSG00000242811	0	0	0	0
ENSG00000242814	0	0	0	0
ENSG00000242816	0	0	0	0
ENSG00000242818	0	0	0	0
ENSG00000242822	0	0	0	0
ENSG00000242828	0	0	18	0
ENSG00000242829	0	0	0	0
ENSG00000242834	0	0	0	0
ENSG00000242836	0	0	0	0
ENSG00000242837	0	0	0	0
ENSG00000242841	0	0	0	0
ENSG00000242848	0	0	0	0
ENSG00000242849	0	0	0	0
ENSG00000242850	0	0	0	0
ENSG00000242852	0	0	0	1
ENSG00000242853	1	0	0	7
ENSG00000242854	0	0	0	0
ENSG00000242855	0	0	0	0
ENSG00000242856	0	0	0	0
ENSG00000242858	0	0	0	0
ENSG00000242860	0	0	0	0
ENSG00000242861	11	24	13	9
ENSG00000242863	0	0	0	0
ENSG00000242865	0	0	0	0
ENSG00000242866	0	0	0	0
ENSG00000242870	0	22	2	15
ENSG00000242871	0	0	0	0
ENSG00000242874	0	0	0	0
ENSG00000242875	0	0	0	0
ENSG00000242876	0	0	0	0
ENSG00000242879	0	0	0	0
ENSG00000242880	0	0	6	27
ENSG00000242882	0	0	0	0
ENSG00000242887	0	0	0	0
ENSG00000242888	0	0	0	0

ENSG00000242889	0	0	0	0
ENSG00000242893	0	0	0	0
ENSG00000242894	0	0	0	0
ENSG00000242899	0	0	0	0
ENSG00000242902	0	0	0	0
ENSG00000242908	0	0	0	0
ENSG00000242911	0	0	0	0
ENSG00000242912	0	0	0	0
ENSG00000242915	0	0	0	0
ENSG00000242922	0	0	0	0
ENSG00000242924	0	0	0	0
ENSG00000242925	0	0	0	0
ENSG00000242926	0	0	0	0
ENSG00000242928	0	0	0	0
ENSG00000242931	0	2	0	0
ENSG00000242936	0	0	0	0
ENSG00000242941	0	0	0	0
ENSG00000242943	0	0	0	0
ENSG00000242945	0	0	0	0
ENSG00000242948	0	0	0	0
ENSG00000242950	0	0	0	0
ENSG00000242951	0	0	0	0
ENSG00000242952	0	0	0	1
ENSG00000242953	0	0	1	3
ENSG00000242958	0	0	0	0
ENSG00000242960	2	3	7	17
ENSG00000242962	0	0	0	0
ENSG00000242963	0	0	0	0
ENSG00000242968	0	0	1	0
ENSG00000242970	0	0	0	0
ENSG00000242971	0	0	0	0
ENSG00000242973	0	0	0	0
ENSG00000242976	0	0	0	0
ENSG00000242978	0	0	0	0
ENSG00000242979	0	0	0	0
ENSG00000242983	0	0	0	0
ENSG00000242985	0	0	0	0
ENSG00000242986	0	0	0	0
ENSG00000242989	0	0	0	0
ENSG00000242990	0	0	0	0
ENSG00000242991	0	0	0	0
ENSG00000242992	1	0	0	0
ENSG00000242993	0	0	0	0
ENSG00000242995	0	0	0	0
ENSG00000242998	0	0	0	0
ENSG00000242999	0	0	0	0

ENSG00000243000	0	0	0	0
ENSG00000243003	0	0	0	0
ENSG00000243004	27	87	24	105
ENSG00000243005	0	0	0	0
ENSG00000243007	0	0	0	0
ENSG00000243008	0	0	0	0
ENSG00000243009	0	0	0	0
ENSG00000243011	0	0	0	0
ENSG00000243012	7	48	11	5
ENSG00000243014	1	0	0	0
ENSG00000243015	1	0	0	0
ENSG00000243016	0	0	0	0
ENSG00000243018	0	0	0	0
ENSG00000243020	0	0	0	0
ENSG00000243022	0	0	0	0
ENSG00000243023	0	0	0	0
ENSG00000243024	0	0	0	0
ENSG00000243025	0	0	0	0
ENSG00000243027	0	0	0	0
ENSG00000243029	0	0	0	0
ENSG00000243033	0	0	0	0
ENSG00000243035	0	0	0	0
ENSG00000243038	0	0	0	0
ENSG00000243039	0	0	0	0
ENSG00000243040	0	0	0	0
ENSG00000243044	0	0	0	0
ENSG00000243048	0	0	0	0
ENSG00000243049	0	0	0	0
ENSG00000243050	0	0	0	0
ENSG00000243051	0	0	0	0
ENSG00000243053	0	0	0	0
ENSG00000243055	0	0	1	3
ENSG00000243056	0	0	0	0
ENSG00000243058	0	0	0	0
ENSG00000243059	0	0	0	0
ENSG00000243062	0	0	0	0
ENSG00000243063	0	0	0	0
ENSG00000243064	1	0	3	0
ENSG00000243066	0	0	0	0
ENSG00000243067	0	0	0	0
ENSG00000243069	14	40	120	103
ENSG00000243071	1	0	0	0
ENSG00000243072	0	0	0	0
ENSG00000243073	0	0	0	0
ENSG00000243075	0	0	0	0
ENSG00000243077	0	0	0	0

ENSG00000243081	1	0	0	0
ENSG00000243083	0	0	0	0
ENSG00000243085	0	0	0	0
ENSG00000243088	0	0	0	0
ENSG00000243089	0	0	0	3
ENSG00000243094	0	0	0	0
ENSG00000243095	0	0	0	0
ENSG00000243099	0	0	0	0
ENSG00000243101	0	0	0	0
ENSG00000243103	0	0	0	0
ENSG00000243104	0	0	0	0
ENSG00000243107	0	0	0	0
ENSG00000243113	0	0	0	0
ENSG00000243115	0	0	0	0
ENSG00000243116	0	0	0	0
ENSG00000243122	0	0	0	0
ENSG00000243124	0	0	0	0
ENSG00000243125	0	0	0	0
ENSG00000243129	0	0	0	0
ENSG00000243130	0	0	0	0
ENSG00000243133	0	0	0	0
ENSG00000243135	0	0	0	0
ENSG00000243136	0	0	0	0
ENSG00000243137	0	0	0	0
ENSG00000243141	0	0	0	0
ENSG00000243144	0	0	0	0
ENSG00000243147	54	4	18	13
ENSG00000243149	0	0	1	0
ENSG00000243150	0	0	0	0
ENSG00000243151	0	0	0	0
ENSG00000243153	0	0	1	0
ENSG00000243154	0	0	0	0
ENSG00000243155	0	0	0	0
ENSG00000243156	2	128	73	63
ENSG00000243160	0	0	0	0
ENSG00000243164	0	0	0	0
ENSG00000243165	0	0	0	0
ENSG00000243167	0	0	0	0
ENSG00000243171	0	0	0	0
ENSG00000243173	0	0	0	0
ENSG00000243175	0	0	0	0
ENSG00000243176	3	76	75	77
ENSG00000243179	0	0	0	0
ENSG00000243181	2	0	0	0
ENSG00000243187	0	0	0	0
ENSG00000243188	0	0	0	0

ENSG00000243189	0	0	0	0
ENSG00000243193	0	0	0	0
ENSG00000243195	0	0	0	0
ENSG00000243197	0	0	0	0
ENSG00000243199	2	0	0	0
ENSG00000243207	0	0	0	0
ENSG00000243215	0	0	0	0
ENSG00000243220	0	0	0	0
ENSG00000243224	3	0	0	0
ENSG00000243225	0	0	0	0
ENSG00000243227	0	0	0	0
ENSG00000243230	0	0	0	0
ENSG00000243232	0	0	0	0
ENSG00000243234	0	0	0	0
ENSG00000243236	0	0	0	0
ENSG00000243238	0	0	0	0
ENSG00000243243	0	0	3	0
ENSG00000243244	105	1281	1414	673
ENSG00000243245	0	0	0	0
ENSG00000243250	0	0	0	0
ENSG00000243251	0	0	0	0
ENSG00000243252	0	0	0	0
ENSG00000243254	0	0	0	0
ENSG00000243256	0	0	0	0
ENSG00000243257	0	0	0	0
ENSG00000243260	0	0	0	0
ENSG00000243264	0	0	0	0
ENSG00000243265	0	0	0	0
ENSG00000243267	0	0	0	0
ENSG00000243268	0	0	0	0
ENSG00000243273	0	0	0	0
ENSG00000243274	0	0	0	0
ENSG00000243276	0	0	0	0
ENSG00000243279	51	9	5	20
ENSG00000243280	0	0	0	0
ENSG00000243284	0	0	0	2
ENSG00000243286	0	0	0	0
ENSG00000243287	0	0	0	0
ENSG00000243289	0	0	0	0
ENSG00000243290	0	0	0	0
ENSG00000243295	0	0	0	0
ENSG00000243296	0	0	0	0
ENSG00000243297	0	0	3	0
ENSG00000243300	0	0	0	0
ENSG00000243302	1	0	0	4
ENSG00000243303	0	0	0	0

ENSG00000243304	0	0	0	0
ENSG00000243305	0	0	5	0
ENSG00000243307	0	0	0	0
ENSG00000243312	0	0	0	0
ENSG00000243313	0	0	0	0
ENSG00000243314	0	0	0	0
ENSG00000243316	0	0	0	0
ENSG00000243317	7815	18628	16469	14464
ENSG00000243319	0	0	0	0
ENSG00000243321	0	0	0	0
ENSG00000243322	0	0	0	0
ENSG00000243323	0	0	0	0
ENSG00000243328	0	0	0	0
ENSG00000243333	0	0	0	0
ENSG00000243335	56	103	79	21
ENSG00000243336	0	0	0	0
ENSG00000243338	0	0	0	0
ENSG00000243339	0	0	1	1
ENSG00000243345	0	0	0	0
ENSG00000243346	0	0	0	0
ENSG00000243347	0	0	0	0
ENSG00000243349	0	0	0	0
ENSG00000243350	0	0	0	0
ENSG00000243352	0	0	0	0
ENSG00000243353	0	0	0	0
ENSG00000243355	0	0	0	0
ENSG00000243359	0	0	0	0
ENSG00000243364	0	0	0	0
ENSG00000243365	0	0	0	0
ENSG00000243366	0	0	0	0
ENSG00000243368	0	0	0	0
ENSG00000243370	0	0	0	0
ENSG00000243373	0	0	0	0
ENSG00000243374	0	0	0	0
ENSG00000243378	0	0	0	0
ENSG00000243382	0	0	0	0
ENSG00000243383	0	0	0	0
ENSG00000243384	0	0	0	0
ENSG00000243385	0	0	0	0
ENSG00000243388	0	0	0	0
ENSG00000243389	0	0	0	0
ENSG00000243396	0	0	0	0
ENSG00000243398	0	0	0	0
ENSG00000243402	0	0	0	0
ENSG00000243403	3	0	0	0
ENSG00000243404	0	0	0	0

ENSG00000243406	0	0	0	0
ENSG00000243410	0	0	0	0
ENSG00000243414	0	0	0	0
ENSG00000243415	0	0	0	0
ENSG00000243416	0	0	0	0
ENSG00000243417	0	0	0	0
ENSG00000243420	0	0	0	0
ENSG00000243422	14	107	28	29
ENSG00000243423	0	0	0	0
ENSG00000243424	0	0	0	0
ENSG00000243426	0	0	0	0
ENSG00000243429	0	0	0	0
ENSG00000243430	0	0	0	0
ENSG00000243431	0	0	0	0
ENSG00000243433	0	0	0	0
ENSG00000243437	0	0	0	0
ENSG00000243438	0	0	0	0
ENSG00000243439	0	0	0	0
ENSG00000243440	0	67	5	24
ENSG00000243441	0	0	0	0
ENSG00000243444	0	0	0	0
ENSG00000243445	0	0	0	0
ENSG00000243446	0	0	0	0
ENSG00000243449	3	0	0	0
ENSG00000243452	0	24	1	0
ENSG00000243453	0	0	0	0
ENSG00000243455	0	0	0	0
ENSG00000243464	0	0	0	0
ENSG00000243466	0	0	0	0
ENSG00000243467	0	0	0	0
ENSG00000243468	0	0	0	0
ENSG00000243469	0	0	0	0
ENSG00000243473	0	0	0	0
ENSG00000243477	5	0	0	3
ENSG00000243478	1	0	0	3
ENSG00000243479	0	0	0	0
ENSG00000243480	0	0	0	0
ENSG00000243483	0	0	0	0
ENSG00000243485	0	0	0	0
ENSG00000243486	0	0	0	0
ENSG00000243488	0	0	0	0
ENSG00000243489	0	0	0	0
ENSG00000243491	42	263	125	50
ENSG00000243492	0	0	0	0
ENSG00000243494	0	0	0	0
ENSG00000243495	0	0	0	0

ENSG00000243496	0	0	0	0
ENSG00000243497	0	0	0	0
ENSG00000243498	0	0	0	0
ENSG00000243499	0	0	0	0
ENSG00000243500	0	0	0	0
ENSG00000243501	0	0	0	0
ENSG00000243503	0	0	0	0
ENSG00000243504	0	0	0	0
ENSG00000243505	0	0	0	0
ENSG00000243507	0	0	0	0
ENSG00000243508	0	0	0	0
ENSG00000243509	0	0	0	0
ENSG00000243510	0	0	0	0
ENSG00000243514	0	0	0	0
ENSG00000243516	0	0	0	0
ENSG00000243517	1	0	0	0
ENSG00000243518	0	0	0	0
ENSG00000243519	0	0	0	0
ENSG00000243521	0	0	0	0
ENSG00000243531	0	0	0	0
ENSG00000243532	0	0	0	0
ENSG00000243533	0	0	0	0
ENSG00000243536	0	0	0	0
ENSG00000243537	0	0	0	0
ENSG00000243538	0	0	0	0
ENSG00000243539	0	0	0	0
ENSG00000243540	0	0	0	0
ENSG00000243541	0	0	0	0
ENSG00000243543	0	0	0	0
ENSG00000243544	0	0	0	0
ENSG00000243546	0	0	0	0
ENSG00000243547	0	0	0	0
ENSG00000243548	0	0	0	0
ENSG00000243549	0	0	0	0
ENSG00000243550	0	0	0	0
ENSG00000243554	0	30	29	30
ENSG00000243558	0	0	0	0
ENSG00000243560	0	0	0	0
ENSG00000243562	0	0	0	0
ENSG00000243565	0	0	0	0
ENSG00000243566	0	0	0	0
ENSG00000243568	0	0	0	0
ENSG00000243570	0	0	0	0
ENSG00000243572	0	0	0	0
ENSG00000243574	0	0	0	0
ENSG00000243581	0	0	0	0

ENSG00000243583	0	0	0	0
ENSG00000243584	1	0	0	0
ENSG00000243587	0	1	34	49
ENSG00000243591	0	0	0	0
ENSG00000243592	1	28	10	10
ENSG00000243593	0	0	0	0
ENSG00000243594	0	0	0	0
ENSG00000243596	0	0	0	0
ENSG00000243601	0	0	0	5
ENSG00000243604	0	0	0	0
ENSG00000243607	0	0	0	0
ENSG00000243609	0	0	0	0
ENSG00000243612	0	0	0	0
ENSG00000243613	0	0	0	0
ENSG00000243620	0	0	0	0
ENSG00000243621	0	0	0	0
ENSG00000243627	0	0	0	0
ENSG00000243628	0	0	0	0
ENSG00000243629	0	0	0	0
ENSG00000243633	0	0	0	0
ENSG00000243635	0	0	0	0
ENSG00000243636	0	0	0	0
ENSG00000243637	70	205	70	68
ENSG00000243641	0	0	0	0
ENSG00000243642	0	0	0	0
ENSG00000243643	0	0	0	0
ENSG00000243644	0	0	0	0
ENSG00000243646	21	32	32	10
ENSG00000243648	0	0	1	0
ENSG00000243649	0	0	0	0
ENSG00000243650	0	0	1	3
ENSG00000243654	0	0	0	0
ENSG00000243655	0	0	0	0
ENSG00000243658	0	0	0	0
ENSG00000243659	1	0	0	0
ENSG00000243660	9	19	4	12
ENSG00000243661	0	0	0	0
ENSG00000243663	0	0	0	0
ENSG00000243664	0	0	0	0
ENSG00000243667	2	0	1	0
ENSG00000243669	0	0	0	0
ENSG00000243671	0	0	0	0
ENSG00000243672	0	0	0	0
ENSG00000243674	0	0	0	0
ENSG00000243675	0	0	0	0
ENSG00000243678	0	0	0	0

ENSG00000243679	2	0	0	0
ENSG00000243680	0	0	0	0
ENSG00000243686	0	0	0	0
ENSG00000243687	0	0	0	0
ENSG00000243694	0	0	0	0
ENSG00000243695	0	0	0	0
ENSG00000243696	0	0	0	0
ENSG00000243697	0	0	0	0
ENSG00000243698	0	0	0	0
ENSG00000243700	0	0	0	0
ENSG00000243701	186	766	403	442
ENSG00000243702	0	0	0	0
ENSG00000243704	0	0	0	0
ENSG00000243705	0	0	0	0
ENSG00000243708	0	0	0	1
ENSG00000243709	48	70	52	50
ENSG00000243710	0	0	2	3
ENSG00000243711	0	0	0	0
ENSG00000243714	0	0	0	0
ENSG00000243715	0	0	0	0
ENSG00000243716	2	23	14	21
ENSG00000243719	0	0	0	0
ENSG00000243720	0	0	0	0
ENSG00000243721	0	0	2	0
ENSG00000243723	0	0	0	0
ENSG00000243725	1	0	0	0
ENSG00000243729	0	0	0	0
ENSG00000243730	0	0	0	0
ENSG00000243733	0	0	0	0
ENSG00000243736	0	0	0	0
ENSG00000243738	0	0	0	0
ENSG00000243742	0	0	0	0
ENSG00000243744	0	0	0	0
ENSG00000243745	0	6	0	3
ENSG00000243746	0	0	0	0
ENSG00000243749	0	0	0	0
ENSG00000243753	36	0	0	0
ENSG00000243754	11	67	9	21
ENSG00000243758	0	0	0	0
ENSG00000243759	0	0	3	1
ENSG00000243761	0	0	0	0
ENSG00000243762	0	0	0	0
ENSG00000243766	0	0	0	0
ENSG00000243768	0	0	0	0
ENSG00000243770	0	0	0	0
ENSG00000243771	0	0	0	0

ENSG00000243772	3	0	0	0
ENSG00000243775	0	0	0	0
ENSG00000243777	0	0	0	0
ENSG00000243779	0	0	0	0
ENSG00000243780	0	0	0	0
ENSG00000243781	0	0	0	0
ENSG00000243782	0	0	0	0
ENSG00000243788	0	19	2	8
ENSG00000243789	1	0	0	0
ENSG00000243790	0	0	0	0
ENSG00000243792	0	0	0	0
ENSG00000243794	0	0	0	0
ENSG00000243795	0	0	0	0
ENSG00000243797	9	115	224	101
ENSG00000243799	0	0	0	0
ENSG00000243801	0	0	0	0
ENSG00000243802	0	0	0	0
ENSG00000243804	0	0	0	0
ENSG00000243806	0	0	2	2
ENSG00000243810	0	0	0	0
ENSG00000243811	9	0	9	14
ENSG00000243813	0	0	0	0
ENSG00000243817	0	0	0	0
ENSG00000243818	0	0	0	0
ENSG00000243819	0	0	0	0
ENSG00000243822	0	0	0	0
ENSG00000243824	0	0	0	0
ENSG00000243828	0	0	0	0
ENSG00000243829	0	0	0	0
ENSG00000243830	0	0	0	0
ENSG00000243831	0	0	0	0
ENSG00000243832	0	0	0	0
ENSG00000243835	0	0	0	0
ENSG00000243836	1	0	0	0
ENSG00000243838	0	0	0	0
ENSG00000243840	0	0	0	0
ENSG00000243844	0	0	0	0
ENSG00000243845	0	0	0	0
ENSG00000243847	0	0	1	0
ENSG00000243849	0	0	1	1
ENSG00000243853	0	0	0	0
ENSG00000243854	0	0	0	0
ENSG00000243855	0	0	0	0
ENSG00000243856	0	0	0	0
ENSG00000243859	0	0	0	0
ENSG00000243861	0	0	0	0

ENSG00000243864	0	0	0	0
ENSG00000243869	0	0	0	0
ENSG00000243870	0	0	0	0
ENSG00000243871	0	0	0	0
ENSG00000243872	0	0	0	0
ENSG00000243873	0	0	0	0
ENSG00000243877	0	0	0	0
ENSG00000243883	0	0	0	0
ENSG00000243885	0	0	0	0
ENSG00000243886	0	0	0	0
ENSG00000243888	0	0	0	0
ENSG00000243889	0	0	0	0
ENSG00000243894	0	0	0	0
ENSG00000243896	0	0	0	0
ENSG00000243897	0	0	0	0
ENSG00000243899	0	0	0	0
ENSG00000243900	0	0	0	0
ENSG00000243902	0	0	0	0
ENSG00000243903	0	0	0	0
ENSG00000243904	0	0	0	0
ENSG00000243905	0	0	0	0
ENSG00000243910	2	2	0	3
ENSG00000243911	0	0	0	0
ENSG00000243914	0	7	1	0
ENSG00000243915	0	7	1	3
ENSG00000243916	0	0	0	0
ENSG00000243918	0	0	0	0
ENSG00000243920	0	0	0	0
ENSG00000243922	0	0	0	0
ENSG00000243925	0	0	0	0
ENSG00000243926	2	39	4	9
ENSG00000243927	89	256	212	152
ENSG00000243928	0	0	0	0
ENSG00000243929	0	0	0	0
ENSG00000243930	0	0	0	0
ENSG00000243935	0	0	0	0
ENSG00000243939	0	0	0	0
ENSG00000243943	7	27	6	3
ENSG00000243944	0	0	0	0
ENSG00000243945	0	0	0	0
ENSG00000243951	0	0	0	0
ENSG00000243952	0	0	0	0
ENSG00000243953	0	0	0	0
ENSG00000243954	0	0	0	0
ENSG00000243955	791	429	205	240
ENSG00000243957	0	0	0	0

ENSG00000243958	0	0	0	0
ENSG00000243959	0	0	0	0
ENSG00000243960	0	0	0	0
ENSG00000243961	0	0	0	0
ENSG00000243964	0	0	2	3
ENSG00000243967	0	0	0	0
ENSG00000243968	0	0	0	0
ENSG00000243969	0	0	0	0
ENSG00000243970	0	0	0	0
ENSG00000243974	0	0	0	0
ENSG00000243976	0	0	0	0
ENSG00000243977	1	0	1	0
ENSG00000243978	0	0	8	34
ENSG00000243979	0	0	0	0
ENSG00000243980	0	0	0	0
ENSG00000243981	0	0	0	0
ENSG00000243986	0	0	0	0
ENSG00000243988	0	0	0	0
ENSG00000243989	0	0	0	0
ENSG00000243991	0	0	0	0
ENSG00000243993	0	0	0	0
ENSG00000243995	0	0	0	0
ENSG00000244000	0	0	0	0
ENSG00000244002	0	0	0	0
ENSG00000244003	0	0	0	0
ENSG00000244004	0	0	0	0
ENSG00000244005	2	24	4	10
ENSG00000244006	0	0	0	0
ENSG00000244008	0	0	0	0
ENSG00000244009	0	0	0	0
ENSG00000244018	0	0	0	0
ENSG00000244019	0	0	0	0
ENSG00000244020	0	0	0	0
ENSG00000244021	1	0	0	0
ENSG00000244024	0	0	0	0
ENSG00000244025	0	0	0	0
ENSG00000244026	5	0	3	1
ENSG00000244030	0	0	0	0
ENSG00000244031	0	0	0	0
ENSG00000244033	0	0	0	0
ENSG00000244034	0	0	0	0
ENSG00000244036	0	0	3	0
ENSG00000244038	27	15	36	73
ENSG00000244039	0	0	0	0
ENSG00000244040	0	0	3	0
ENSG00000244041	1968	2020	1884	1217

ENSG00000244043	0	0	0	0
ENSG00000244044	0	0	0	0
ENSG00000244045	9	0	8	2
ENSG00000244048	0	0	0	0
ENSG00000244050	0	0	0	0
ENSG00000244052	0	0	0	0
ENSG00000244053	0	0	0	0
ENSG00000244054	0	0	0	0
ENSG00000244055	0	860	390	967
ENSG00000244056	0	0	0	0
ENSG00000244057	0	0	0	0
ENSG00000244060	0	0	0	0
ENSG00000244061	0	0	0	0
ENSG00000244062	0	0	0	0
ENSG00000244063	10	0	17	12
ENSG00000244065	0	0	0	0
ENSG00000244066	0	0	0	0
ENSG00000244067	4	0	0	30
ENSG00000244071	0	0	0	0
ENSG00000244073	0	0	0	0
ENSG00000244076	0	0	0	0
ENSG00000244078	0	0	0	0
ENSG00000244080	0	0	0	0
ENSG00000244081	0	0	0	0
ENSG00000244083	0	0	0	0
ENSG00000244086	0	0	0	0
ENSG00000244088	0	0	0	0
ENSG00000244089	0	0	3	0
ENSG00000244091	0	0	0	0
ENSG00000244094	0	0	0	0
ENSG00000244097	0	7	3	10
ENSG00000244099	0	0	0	0
ENSG00000244101	0	0	0	4
ENSG00000244104	0	0	0	0
ENSG00000244107	0	0	1	0
ENSG00000244112	0	0	0	0
ENSG00000244113	0	0	0	0
ENSG00000244115	0	0	0	0
ENSG00000244116	0	0	0	0
ENSG00000244117	0	0	0	0
ENSG00000244119	0	0	0	0
ENSG00000244122	0	0	0	0
ENSG00000244124	0	0	0	0
ENSG00000244125	0	0	0	0
ENSG00000244128	0	0	0	0
ENSG00000244130	0	0	0	0

ENSG00000244131	0	0	0	0
ENSG00000244134	0	0	0	0
ENSG00000244137	0	0	0	0
ENSG00000244139	0	0	0	0
ENSG00000244142	0	0	0	0
ENSG00000244144	0	0	0	0
ENSG00000244146	0	0	0	0
ENSG00000244150	0	0	0	0
ENSG00000244151	0	0	0	0
ENSG00000244152	0	0	0	0
ENSG00000244153	0	0	0	0
ENSG00000244155	0	0	0	0
ENSG00000244157	0	0	0	0
ENSG00000244158	10	113	70	36
ENSG00000244159	0	0	0	0
ENSG00000244161	0	0	0	0
ENSG00000244162	0	0	0	0
ENSG00000244165	0	0	1	0
ENSG00000244167	0	0	0	0
ENSG00000244169	0	0	0	0
ENSG00000244171	0	0	0	3
ENSG00000244176	0	0	0	0
ENSG00000244179	0	0	0	0
ENSG00000244180	0	0	0	0
ENSG00000244183	0	0	0	0
ENSG00000244184	0	0	0	0
ENSG00000244186	0	0	0	0
ENSG00000244187	75	7	6	51
ENSG00000244192	0	0	0	0
ENSG00000244193	0	0	0	0
ENSG00000244194	0	0	0	0
ENSG00000244196	0	0	0	0
ENSG00000244197	0	0	0	0
ENSG00000244198	0	0	0	0
ENSG00000244199	0	0	0	0
ENSG00000244202	0	0	0	0
ENSG00000244203	0	0	0	0
ENSG00000244207	0	0	0	0
ENSG00000244211	0	0	0	0
ENSG00000244213	0	0	0	0
ENSG00000244214	0	0	0	0
ENSG00000244215	0	0	0	0
ENSG00000244217	0	0	0	0
ENSG00000244218	0	0	0	0
ENSG00000244219	1	0	0	8
ENSG00000244222	0	0	0	0

ENSG00000244226	0	0	0	0
ENSG00000244227	1	0	0	0
ENSG00000244229	0	0	0	0
ENSG00000244230	0	0	0	0
ENSG00000244231	0	0	0	0
ENSG00000244232	0	0	0	0
ENSG00000244234	0	0	0	0
ENSG00000244235	0	0	0	0
ENSG00000244236	0	0	0	0
ENSG00000244237	0	0	0	0
ENSG00000244239	0	0	0	0
ENSG00000244242	1	0	0	0
ENSG00000244244	0	0	0	0
ENSG00000244245	0	0	0	0
ENSG00000244246	0	0	0	0
ENSG00000244247	0	0	0	0
ENSG00000244249	0	0	0	0
ENSG00000244251	0	0	0	0
ENSG00000244252	0	0	0	0
ENSG00000244253	0	0	0	0
ENSG00000244255	0	0	0	0
ENSG00000244256	1	0	0	0
ENSG00000244257	0	0	0	0
ENSG00000244259	0	0	0	0
ENSG00000244260	0	0	0	0
ENSG00000244264	0	0	0	0
ENSG00000244265	0	21	12	16
ENSG00000244266	0	0	0	0
ENSG00000244267	0	0	0	0
ENSG00000244268	12	0	12	11
ENSG00000244270	0	0	0	0
ENSG00000244273	140	70	277	323
ENSG00000244274	0	0	0	0
ENSG00000244278	0	0	0	0
ENSG00000244280	0	0	0	0
ENSG00000244281	0	0	0	0
ENSG00000244283	0	0	0	0
ENSG00000244286	0	0	0	0
ENSG00000244289	0	0	0	0
ENSG00000244291	5	0	10	0
ENSG00000244292	0	0	0	0
ENSG00000244294	0	0	0	0
ENSG00000244295	0	0	0	0
ENSG00000244296	0	0	0	0
ENSG00000244297	0	0	0	0
ENSG00000244300	1	22	22	6

ENSG00000244301	0	0	0	4
ENSG00000244302	0	0	0	0
ENSG00000244306	3	0	1	52
ENSG00000244307	0	0	0	0
ENSG00000244308	0	0	0	0
ENSG00000244310	0	0	0	0
ENSG00000244313	3	0	0	0
ENSG00000244314	0	0	0	0
ENSG00000244318	0	0	0	0
ENSG00000244321	0	0	0	0
ENSG00000244324	0	0	0	0
ENSG00000244326	1	0	0	0
ENSG00000244327	0	0	0	0
ENSG00000244328	0	0	0	0
ENSG00000244329	0	0	0	0
ENSG00000244331	0	0	0	0
ENSG00000244332	0	0	0	0
ENSG00000244335	1	0	0	0
ENSG00000244337	0	0	0	0
ENSG00000244342	0	0	0	0
ENSG00000244345	0	0	0	0
ENSG00000244346	0	0	0	0
ENSG00000244349	0	0	0	0
ENSG00000244351	0	0	0	0
ENSG00000244355	1	0	0	0
ENSG00000244356	0	0	0	0
ENSG00000244357	0	0	0	0
ENSG00000244358	0	0	0	0
ENSG00000244361	0	0	0	0
ENSG00000244362	0	0	0	0
ENSG00000244363	0	0	0	0
ENSG00000244365	0	0	6	7
ENSG00000244371	0	0	0	0
ENSG00000244372	0	0	0	0
ENSG00000244376	0	0	0	1
ENSG00000244378	0	0	0	0
ENSG00000244380	0	15	3	7
ENSG00000244381	0	0	0	0
ENSG00000244382	0	0	0	0
ENSG00000244383	0	20	0	1
ENSG00000244384	0	0	0	0
ENSG00000244389	0	0	0	0
ENSG00000244390	0	0	0	0
ENSG00000244391	0	0	0	0
ENSG00000244392	0	0	0	0
ENSG00000244393	0	0	4	0

ENSG00000244395	0	0	0	0
ENSG00000244398	5	67	170	360
ENSG00000244399	0	0	0	0
ENSG00000244400	0	0	0	0
ENSG00000244402	0	0	0	0
ENSG00000244404	0	0	0	0
ENSG00000244405	5	29	17	21
ENSG00000244411	0	0	0	0
ENSG00000244413	0	0	0	0
ENSG00000244414	0	0	0	0
ENSG00000244422	0	0	0	0
ENSG00000244424	0	0	0	0
ENSG00000244425	0	0	0	0
ENSG00000244427	0	0	0	0
ENSG00000244429	0	0	0	0
ENSG00000244432	0	0	0	0
ENSG00000244433	0	0	0	0
ENSG00000244436	0	0	0	0
ENSG00000244437	0	0	0	0
ENSG00000244438	0	0	0	0
ENSG00000244439	0	0	0	0
ENSG00000244441	0	0	0	0
ENSG00000244444	0	0	0	0
ENSG00000244451	0	0	0	0
ENSG00000244456	0	0	0	0
ENSG00000244457	0	0	0	0
ENSG00000244459	292	49	13	28
ENSG00000244461	0	0	0	0
ENSG00000244462	11	399	898	969
ENSG00000244464	0	0	0	1
ENSG00000244468	0	0	1	0
ENSG00000244470	0	29	60	68
ENSG00000244471	0	0	0	0
ENSG00000244472	0	0	0	0
ENSG00000244474	0	0	0	0
ENSG00000244476	0	0	0	0
ENSG00000244479	10	18	2	1
ENSG00000244480	0	0	0	0
ENSG00000244482	0	0	0	0
ENSG00000244484	0	0	0	0
ENSG00000244485	0	0	0	0
ENSG00000244486	0	0	0	0
ENSG00000244490	1	0	0	0
ENSG00000244491	0	0	0	0
ENSG00000244493	0	17	7	0
ENSG00000244498	0	0	0	0

ENSG00000244501	0	0	0	0
ENSG00000244502	0	0	0	0
ENSG00000244503	0	0	0	0
ENSG00000244509	800	183	119	182
ENSG00000244510	0	0	0	0
ENSG00000244512	0	0	0	0
ENSG00000244513	0	49	31	11
ENSG00000244514	0	0	0	0
ENSG00000244515	0	0	0	0
ENSG00000244521	0	0	0	0
ENSG00000244522	0	0	0	1
ENSG00000244527	0	0	0	0
ENSG00000244528	0	0	0	0
ENSG00000244531	0	0	0	0
ENSG00000244532	0	0	0	0
ENSG00000244534	0	0	0	0
ENSG00000244535	0	0	0	0
ENSG00000244537	0	0	0	0
ENSG00000244538	0	0	0	0
ENSG00000244540	0	0	0	0
ENSG00000244541	0	0	0	0
ENSG00000244544	0	0	0	0
ENSG00000244545	0	0	0	0
ENSG00000244550	0	0	0	0
ENSG00000244551	0	0	0	0
ENSG00000244556	1	0	0	0
ENSG00000244558	0	0	0	0
ENSG00000244559	0	0	0	0
ENSG00000244560	0	0	0	0
ENSG00000244561	0	0	0	0
ENSG00000244563	0	0	0	0
ENSG00000244564	0	0	0	0
ENSG00000244565	0	0	0	0
ENSG00000244567	4	0	4	0
ENSG00000244568	0	0	0	0
ENSG00000244571	0	0	0	0
ENSG00000244573	5	0	6	7
ENSG00000244575	0	0	0	0
ENSG00000244576	0	0	0	0
ENSG00000244578	0	0	0	0
ENSG00000244582	55	53	20	28
ENSG00000244585	0	0	0	0
ENSG00000244586	0	0	0	0
ENSG00000244588	1	0	8	1
ENSG00000244593	0	0	0	0
ENSG00000244599	0	0	0	0

ENSG00000244604	1	0	0	0
ENSG00000244607	0	0	7	4
ENSG00000244609	0	0	0	0
ENSG00000244610	0	0	0	0
ENSG00000244612	0	0	0	0
ENSG00000244615	0	0	0	0
ENSG00000244617	0	0	0	0
ENSG00000244618	0	0	0	0
ENSG00000244619	0	0	0	0
ENSG00000244620	0	17	0	13
ENSG00000244621	0	0	0	0
ENSG00000244623	0	0	0	0
ENSG00000244624	0	0	0	0
ENSG00000244625	24	29	50	122
ENSG00000244627	2	4	7	9
ENSG00000244630	0	0	0	0
ENSG00000244632	0	0	0	0
ENSG00000244640	0	0	0	0
ENSG00000244641	0	0	0	0
ENSG00000244642	0	16	11	44
ENSG00000244646	0	0	0	0
ENSG00000244649	0	0	0	0
ENSG00000244650	0	0	0	0
ENSG00000244652	0	0	0	0
ENSG00000244653	0	0	0	0
ENSG00000244656	0	0	0	0
ENSG00000244657	0	0	0	0
ENSG00000244661	0	0	0	0
ENSG00000244662	0	0	0	0
ENSG00000244666	0	0	0	0
ENSG00000244668	0	0	0	0
ENSG00000244669	0	0	0	0
ENSG00000244671	0	0	0	0
ENSG00000244672	0	0	0	0
ENSG00000244674	0	0	0	0
ENSG00000244675	2	0	0	0
ENSG00000244676	0	0	0	2
ENSG00000244677	0	0	0	0
ENSG00000244681	0	0	0	0
ENSG00000244682	0	0	0	0
ENSG00000244684	0	0	0	0
ENSG00000244687	8	1	44	30
ENSG00000244691	0	0	0	0
ENSG00000244692	0	0	0	0
ENSG00000244693	0	0	0	0
ENSG00000244694	1	0	0	0

ENSG00000244695	0	0	0	0
ENSG00000244697	0	0	0	0
ENSG00000244699	0	17	4	1
ENSG00000244701	8	0	7	0
ENSG00000244703	0	12	30	74
ENSG00000244705	0	0	0	0
ENSG00000244706	0	0	0	0
ENSG00000244708	0	0	0	0
ENSG00000244710	0	0	0	0
ENSG00000244712	0	0	0	0
ENSG00000244716	0	0	0	5
ENSG00000244717	0	0	0	0
ENSG00000244720	0	0	0	3
ENSG00000244721	0	0	0	0
ENSG00000244722	0	0	0	0
ENSG00000244723	0	0	0	0
ENSG00000244730	0	0	0	0
ENSG00000244731	0	0	0	0
ENSG00000244732	0	0	0	0
ENSG00000244733	3	23	44	4
ENSG00000244734	7847	7351	3171	3030
ENSG00000244736	0	0	0	0
ENSG00000244738	0	0	0	3
ENSG00000244740	0	0	0	0
ENSG00000244743	0	0	0	0
ENSG00000244748	0	0	0	0
ENSG00000244752	0	0	0	0
ENSG00000244753	0	0	0	0
ENSG00000244754	144	28786	25292	33257
ENSG00000244756	0	0	0	0
ENSG00000244757	26	130	62	133
ENSG00000244791	0	0	0	0
ENSG00000244879	80	160	100	119
ENSG00000244921	0	0	0	0
ENSG00000244926	0	0	1	0
ENSG00000244932	0	0	0	0
ENSG00000244945	1	31	93	49
ENSG00000244952	0	0	0	0
ENSG00000244953	0	0	0	0
ENSG00000244968	0	0	0	0
ENSG00000244998	2	0	1	0
ENSG00000245008	112	347	652	392
ENSG00000245017	4	0	13	21
ENSG00000245025	0	0	0	0
ENSG00000245059	0	0	0	0
ENSG00000245060	21	131	70	97

ENSG00000245067	0	0	0	0
ENSG00000245080	0	0	0	0
ENSG00000245105	0	42	6	11
ENSG00000245112	0	0	1	0
ENSG00000245146	15	69	68	107
ENSG00000245148	0	0	0	0
ENSG00000245149	3	187	57	22
ENSG00000245156	0	47	60	37
ENSG00000245164	25	0	2	0
ENSG00000245205	1	0	1	0
ENSG00000245213	1	52	64	71
ENSG00000245248	0	0	0	0
ENSG00000245261	0	0	0	0
ENSG00000245275	5	0	4	6
ENSG00000245281	7	23	35	37
ENSG00000245293	0	0	0	0
ENSG00000245311	0	0	17	13
ENSG00000245317	1	0	0	0
ENSG00000245322	0	11	12	22
ENSG00000245330	0	0	0	0
ENSG00000245384	3	11	5	2
ENSG00000245385	0	0	0	0
ENSG00000245466	0	0	0	0
ENSG00000245468	0	0	0	0
ENSG00000245479	0	0	0	0
ENSG00000245482	0	0	26	4
ENSG00000245498	0	0	0	1
ENSG00000245522	0	0	0	0
ENSG00000245526	0	0	0	2
ENSG00000245532	2025	6934	5123	6697
ENSG00000245534	1	6	21	15
ENSG00000245552	132	158	183	102
ENSG00000245556	6	0	1	2
ENSG00000245562	1	2	0	0
ENSG00000245571	0	0	3	0
ENSG00000245573	0	0	14	21
ENSG00000245598	1	0	0	0
ENSG00000245614	1	22	2	4
ENSG00000245648	0	0	0	1
ENSG00000245651	0	0	0	0
ENSG00000245662	1	0	5	0
ENSG00000245667	0	0	0	0
ENSG00000245680	6	21	13	17
ENSG00000245685	0	33	15	28
ENSG00000245688	0	0	0	0
ENSG00000245694	11	66	34	61

ENSG00000245711	0	0	0	0
ENSG00000245719	0	0	0	0
ENSG00000245729	0	0	0	0
ENSG00000245748	0	0	0	12
ENSG00000245750	1	0	2	6
ENSG00000245768	0	35	3	0
ENSG00000245812	0	0	0	0
ENSG00000245832	0	0	0	0
ENSG00000245848	0	0	0	0
ENSG00000245849	36	40	33	15
ENSG00000245857	0	0	0	0
ENSG00000245864	0	40	64	15
ENSG00000245869	0	8	0	0
ENSG00000245870	0	0	0	0
ENSG00000245888	0	0	0	0
ENSG00000245904	3	0	2	0
ENSG00000245910	3625	5587	4252	3905
ENSG00000245928	0	0	0	0
ENSG00000245937	11	8	49	87
ENSG00000245954	1	0	0	0
ENSG00000245958	4	83	396	161
ENSG00000245970	1	26	13	6
ENSG00000245975	0	0	0	0
ENSG00000246016	0	0	0	0
ENSG00000246022	0	0	0	0
ENSG00000246067	33	130	125	142
ENSG00000246082	1	0	0	0
ENSG00000246084	2	0	19	13
ENSG00000246089	1	0	2	2
ENSG00000246090	4	34	130	80
ENSG00000246095	0	0	0	0
ENSG00000246100	1	0	0	0
ENSG00000246115	0	0	0	0
ENSG00000246130	0	0	0	0
ENSG00000246139	0	0	0	0
ENSG00000246145	0	0	0	0
ENSG00000246174	1	0	0	0
ENSG00000246203	0	0	0	3
ENSG00000246211	0	0	0	0
ENSG00000246214	0	0	0	0
ENSG00000246223	3	0	0	0
ENSG00000246225	9	32	40	30
ENSG00000246228	6	0	8	17
ENSG00000246250	0	0	5	0
ENSG00000246263	4	0	3	1
ENSG00000246273	0	0	0	0

ENSG00000246283	0	13	1	0
ENSG00000246308	3	0	10	9
ENSG00000246316	0	0	0	0
ENSG00000246323	0	0	1	4
ENSG00000246331	0	0	0	0
ENSG00000246334	65	0	43	48
ENSG00000246339	0	0	0	0
ENSG00000246350	0	0	0	4
ENSG00000246363	3	0	3	2
ENSG00000246366	5	25	66	26
ENSG00000246375	0	0	1	2
ENSG00000246379	0	0	0	0
ENSG00000246394	0	0	0	0
ENSG00000246422	0	0	0	0
ENSG00000246430	0	0	0	0
ENSG00000246448	823	2887	2179	3157
ENSG00000246451	0	0	0	0
ENSG00000246465	0	0	0	0
ENSG00000246477	0	0	0	7
ENSG00000246523	0	0	0	0
ENSG00000246526	2	0	3	3
ENSG00000246528	0	0	0	0
ENSG00000246541	0	0	0	0
ENSG00000246548	0	0	0	2
ENSG00000246560	17	0	22	19
ENSG00000246575	0	0	0	0
ENSG00000246582	0	0	0	0
ENSG00000246596	0	52	23	45
ENSG00000246627	0	0	0	0
ENSG00000246640	0	0	0	0
ENSG00000246662	4	55	3	2
ENSG00000246695	1	0	0	0
ENSG00000246705	715	3044	1996	1564
ENSG00000246731	0	0	0	0
ENSG00000246740	0	0	0	0
ENSG00000246763	0	0	0	0
ENSG00000246774	0	0	0	0
ENSG00000246777	0	0	0	0
ENSG00000246790	0	0	0	0
ENSG00000246792	4	17	17	1
ENSG00000246820	0	0	0	0
ENSG00000246851	0	1	1	3
ENSG00000246859	1	46	55	25
ENSG00000246863	0	0	0	0
ENSG00000246876	0	0	0	0
ENSG00000246877	0	0	0	0

ENSG00000246889	139	310	205	327
ENSG00000246898	1	0	3	12
ENSG00000246922	0	0	0	2
ENSG00000246982	0	6	15	11
ENSG00000246985	47	111	84	67
ENSG00000247011	0	0	0	0
ENSG00000247033	0	0	1	0
ENSG00000247049	0	0	0	0
ENSG00000247077	8	13	0	2
ENSG00000247081	0	18	55	122
ENSG00000247092	121	84	36	86
ENSG00000247095	0	0	0	0
ENSG00000247121	29	82	53	66
ENSG00000247130	0	0	0	0
ENSG00000247131	1	0	0	0
ENSG00000247134	1	78	38	70
ENSG00000247137	3	21	16	40
ENSG00000247151	0	0	0	0
ENSG00000247157	10	0	2	6
ENSG00000247193	0	0	0	0
ENSG00000247199	0	0	0	0
ENSG00000247213	0	0	0	0
ENSG00000247228	0	0	3	0
ENSG00000247240	23	1	49	54
ENSG00000247270	0	0	0	0
ENSG00000247271	459	1249	1609	1363
ENSG00000247287	0	0	0	0
ENSG00000247311	0	0	0	0
ENSG00000247315	0	0	0	0
ENSG00000247317	0	0	0	0
ENSG00000247324	0	0	0	0
ENSG00000247345	58	57	97	214
ENSG00000247363	12	22	11	7
ENSG00000247372	0	0	0	0
ENSG00000247373	2	0	3	7
ENSG00000247381	0	0	0	0
ENSG00000247400	37	63	102	70
ENSG00000247402	0	0	0	0
ENSG00000247416	0	0	0	0
ENSG00000247473	0	0	1	0
ENSG00000247498	52	853	1619	380
ENSG00000247516	4	0	0	6
ENSG00000247556	831	1634	2220	3365
ENSG00000247570	0	0	0	0
ENSG00000247572	2	0	8	5
ENSG00000247595	12	0	2	0

ENSG00000247596	84	72	19	31
ENSG00000247624	1	99	59	144
ENSG00000247626	0	0	1	0
ENSG00000247627	34	54	57	9
ENSG00000247629	0	0	0	0
ENSG00000247675	109	28	14	13
ENSG00000247679	0	0	0	0
ENSG00000247699	14	21	22	4
ENSG00000247700	0	0	0	0
ENSG00000247708	0	0	4	3
ENSG00000247728	0	9	5	0
ENSG00000247732	0	0	0	3
ENSG00000247735	9	141	81	97
ENSG00000247746	2	0	3	0
ENSG00000247763	0	0	0	0
ENSG00000247765	14	0	17	21
ENSG00000247774	112	13	18	4
ENSG00000247775	7	62	95	58
ENSG00000247796	0	0	3	2
ENSG00000247809	0	0	0	0
ENSG00000247810	0	0	0	0
ENSG00000247828	13	151	206	559
ENSG00000247844	0	0	0	0
ENSG00000247853	0	0	4	2
ENSG00000247867	0	0	0	0
ENSG00000247872	0	0	0	0
ENSG00000247877	0	0	4	0
ENSG00000247903	14	0	0	1
ENSG00000247911	0	0	0	0
ENSG00000247925	0	0	0	0
ENSG00000247934	2	0	4	22
ENSG00000247950	0	0	4	4
ENSG00000247970	0	0	0	0
ENSG00000247982	6	0	0	0
ENSG00000247993	0	0	0	0
ENSG00000248008	1	0	0	0
ENSG00000248015	0	0	0	0
ENSG00000248019	0	0	3	0
ENSG00000248027	0	0	0	0
ENSG00000248029	0	0	0	0
ENSG00000248044	5	55	22	25
ENSG00000248049	10	187	235	391
ENSG00000248050	0	0	1	0
ENSG00000248079	0	0	0	2
ENSG00000248092	43	39	55	55
ENSG00000248098	0	0	0	0

ENSG00000248099	0	0	2	2
ENSG00000248100	0	0	0	0
ENSG00000248101	0	0	0	0
ENSG00000248102	0	0	0	0
ENSG00000248103	0	0	0	0
ENSG00000248104	0	0	0	0
ENSG00000248105	0	0	0	0
ENSG00000248106	0	0	0	0
ENSG00000248107	0	0	0	0
ENSG00000248109	0	0	0	0
ENSG00000248112	0	0	0	0
ENSG00000248113	0	0	0	0
ENSG00000248114	0	0	0	0
ENSG00000248115	0	0	0	0
ENSG00000248117	0	0	0	0
ENSG00000248118	0	0	0	0
ENSG00000248120	0	0	0	0
ENSG00000248121	0	0	0	0
ENSG00000248122	0	0	0	0
ENSG00000248124	2	512	281	412
ENSG00000248125	0	0	2	2
ENSG00000248126	0	0	0	0
ENSG00000248127	0	0	0	0
ENSG00000248128	0	0	0	0
ENSG00000248131	0	0	0	0
ENSG00000248132	0	0	0	0
ENSG00000248133	0	0	0	0
ENSG00000248134	0	0	0	0
ENSG00000248136	0	0	0	0
ENSG00000248137	0	0	0	0
ENSG00000248138	0	0	0	4
ENSG00000248139	0	0	0	0
ENSG00000248143	0	0	0	0
ENSG00000248144	0	0	0	0
ENSG00000248145	0	0	0	0
ENSG00000248146	0	0	0	0
ENSG00000248147	0	0	0	0
ENSG00000248148	0	0	0	0
ENSG00000248150	0	22	3	0
ENSG00000248152	0	0	0	0
ENSG00000248155	0	0	0	0
ENSG00000248156	0	0	0	0
ENSG00000248159	0	0	0	0
ENSG00000248160	0	0	0	0
ENSG00000248161	6	39	17	15
ENSG00000248162	0	0	0	0

ENSG00000248165	0	0	0	0
ENSG00000248166	1	0	0	0
ENSG00000248167	0	0	0	0
ENSG00000248170	0	0	0	0
ENSG00000248172	0	0	0	0
ENSG00000248173	0	0	2	0
ENSG00000248174	7	0	3	1
ENSG00000248175	1	0	4	9
ENSG00000248176	0	0	0	2
ENSG00000248180	0	0	1	0
ENSG00000248182	0	0	0	0
ENSG00000248184	0	0	0	0
ENSG00000248185	0	0	0	0
ENSG00000248187	0	0	5	0
ENSG00000248188	0	0	0	0
ENSG00000248191	0	0	0	0
ENSG00000248192	0	0	0	0
ENSG00000248195	0	0	0	0
ENSG00000248196	0	0	0	0
ENSG00000248197	0	0	0	0
ENSG00000248199	0	0	0	0
ENSG00000248200	0	0	0	0
ENSG00000248201	0	0	1	0
ENSG00000248202	0	0	0	0
ENSG00000248203	0	0	0	0
ENSG00000248205	0	0	0	0
ENSG00000248206	0	0	0	0
ENSG00000248208	0	0	0	0
ENSG00000248209	0	0	0	0
ENSG00000248210	0	0	0	0
ENSG00000248211	0	0	0	0
ENSG00000248213	0	0	0	0
ENSG00000248215	0	0	0	0
ENSG00000248216	0	0	0	0
ENSG00000248221	0	0	0	0
ENSG00000248222	0	0	0	0
ENSG00000248223	0	4	33	10
ENSG00000248227	0	0	0	0
ENSG00000248228	0	9	0	0
ENSG00000248229	0	0	0	0
ENSG00000248230	0	0	0	0
ENSG00000248231	0	0	0	0
ENSG00000248234	0	0	0	0
ENSG00000248235	0	0	0	0
ENSG00000248236	0	0	0	0
ENSG00000248237	0	0	0	0

ENSG00000248238	0	0	0	0
ENSG00000248240	0	0	0	3
ENSG00000248242	130	2132	1859	2065
ENSG00000248243	0	0	11	25
ENSG00000248245	7	0	1	0
ENSG00000248249	0	0	0	0
ENSG00000248254	0	0	0	0
ENSG00000248256	0	4	4	3
ENSG00000248257	0	0	0	0
ENSG00000248259	0	0	0	0
ENSG00000248261	0	0	0	0
ENSG00000248262	0	0	0	0
ENSG00000248265	0	0	0	0
ENSG00000248266	0	0	0	0
ENSG00000248268	0	0	0	0
ENSG00000248271	0	0	0	0
ENSG00000248275	479	153	128	216
ENSG00000248278	0	0	0	0
ENSG00000248279	0	0	0	0
ENSG00000248280	0	0	1	0
ENSG00000248281	0	0	0	0
ENSG00000248282	0	0	0	0
ENSG00000248283	0	0	0	0
ENSG00000248285	0	0	0	0
ENSG00000248286	0	0	0	0
ENSG00000248287	0	0	0	0
ENSG00000248288	0	0	0	0
ENSG00000248290	1	0	3	4
ENSG00000248293	0	0	0	0
ENSG00000248294	0	0	0	5
ENSG00000248295	0	0	0	0
ENSG00000248296	0	0	0	0
ENSG00000248300	0	0	0	0
ENSG00000248302	0	0	0	0
ENSG00000248305	0	0	0	0
ENSG00000248306	0	0	0	0
ENSG00000248307	0	0	0	0
ENSG00000248308	0	0	0	0
ENSG00000248309	273	2671	2769	3487
ENSG00000248311	0	0	0	0
ENSG00000248313	0	0	0	0
ENSG00000248315	0	0	0	0
ENSG00000248317	0	0	0	0
ENSG00000248318	0	0	3	0
ENSG00000248319	12	0	3	37
ENSG00000248320	0	0	0	0

ENSG00000248322	0	0	0	0
ENSG00000248323	20	31	57	53
ENSG00000248325	0	0	1	0
ENSG00000248327	0	0	0	0
ENSG00000248328	0	0	0	0
ENSG00000248329	0	0	0	0
ENSG00000248330	0	0	0	0
ENSG00000248332	0	0	0	0
ENSG00000248333	17	73	20	19
ENSG00000248334	2603	14152	14004	16964
ENSG00000248335	0	0	0	0
ENSG00000248336	0	0	0	0
ENSG00000248337	0	0	0	0
ENSG00000248338	0	0	0	1
ENSG00000248339	0	0	0	0
ENSG00000248340	0	0	0	0
ENSG00000248341	0	0	0	0
ENSG00000248343	0	0	0	0
ENSG00000248345	0	0	0	0
ENSG00000248346	0	0	0	0
ENSG00000248347	0	0	0	0
ENSG00000248349	0	0	0	0
ENSG00000248350	0	0	0	0
ENSG00000248351	0	0	0	0
ENSG00000248352	0	0	0	0
ENSG00000248354	0	0	0	0
ENSG00000248355	0	0	0	0
ENSG00000248356	0	0	0	0
ENSG00000248358	0	0	0	0
ENSG00000248359	0	0	0	0
ENSG00000248360	99	590	1353	765
ENSG00000248362	0	0	0	0
ENSG00000248363	0	0	0	0
ENSG00000248364	0	0	0	0
ENSG00000248365	0	0	0	0
ENSG00000248366	0	0	0	0
ENSG00000248367	0	0	0	0
ENSG00000248369	0	0	0	0
ENSG00000248370	0	0	0	0
ENSG00000248371	1	0	3	0
ENSG00000248373	87	78	154	48
ENSG00000248374	0	0	0	0
ENSG00000248375	0	0	0	0
ENSG00000248376	0	0	0	0
ENSG00000248377	0	0	0	0
ENSG00000248378	0	0	0	0

ENSG00000248381	0	0	0	0
ENSG00000248383	0	0	0	0
ENSG00000248385	0	0	0	3
ENSG00000248387	0	0	0	0
ENSG00000248388	0	0	0	0
ENSG00000248391	0	0	0	0
ENSG00000248393	0	0	0	0
ENSG00000248394	0	0	0	0
ENSG00000248395	0	20	0	7
ENSG00000248396	0	0	0	0
ENSG00000248397	0	0	0	2
ENSG00000248399	0	0	0	0
ENSG00000248400	0	0	0	0
ENSG00000248401	0	0	0	0
ENSG00000248403	0	0	0	0
ENSG00000248405	0	0	0	0
ENSG00000248406	0	0	0	0
ENSG00000248408	75	0	5	2
ENSG00000248409	0	0	0	0
ENSG00000248415	0	0	0	0
ENSG00000248416	0	0	0	0
ENSG00000248417	0	0	0	0
ENSG00000248418	0	0	0	0
ENSG00000248419	0	0	0	0
ENSG00000248420	0	0	0	0
ENSG00000248421	0	0	0	0
ENSG00000248422	0	0	0	0
ENSG00000248423	0	0	0	0
ENSG00000248424	0	0	0	0
ENSG00000248425	0	0	0	0
ENSG00000248426	0	0	0	0
ENSG00000248428	0	0	0	0
ENSG00000248429	1	0	0	7
ENSG00000248430	0	0	0	0
ENSG00000248431	3	0	10	12
ENSG00000248432	0	0	0	0
ENSG00000248434	0	0	0	0
ENSG00000248439	0	0	0	0
ENSG00000248440	0	0	1	0
ENSG00000248441	0	0	0	0
ENSG00000248442	0	0	0	0
ENSG00000248443	0	0	0	0
ENSG00000248444	0	0	0	0
ENSG00000248445	0	0	1	0
ENSG00000248447	0	0	0	0
ENSG00000248448	0	0	0	0

ENSG00000248449	0	0	0	0
ENSG00000248451	0	0	0	0
ENSG00000248452	0	0	0	0
ENSG00000248453	0	0	0	0
ENSG00000248455	0	0	0	0
ENSG00000248456	0	0	0	0
ENSG00000248457	0	0	0	0
ENSG00000248458	5	1	13	42
ENSG00000248459	0	0	0	0
ENSG00000248461	0	0	0	0
ENSG00000248462	0	0	0	0
ENSG00000248464	0	0	0	0
ENSG00000248466	0	0	0	0
ENSG00000248467	0	0	0	0
ENSG00000248468	0	0	0	0
ENSG00000248469	0	0	0	3
ENSG00000248471	0	0	0	0
ENSG00000248472	20	49	30	209
ENSG00000248473	0	0	0	0
ENSG00000248474	0	0	0	0
ENSG00000248475	156	173	202	489
ENSG00000248476	0	0	15	17
ENSG00000248477	0	0	0	0
ENSG00000248478	0	0	0	0
ENSG00000248479	0	0	0	0
ENSG00000248480	0	0	0	0
ENSG00000248482	0	0	0	0
ENSG00000248483	0	0	0	0
ENSG00000248484	0	0	0	0
ENSG00000248485	0	0	0	0
ENSG00000248486	0	0	0	0
ENSG00000248487	10	0	1	2
ENSG00000248488	0	0	0	0
ENSG00000248489	12	107	61	94
ENSG00000248490	0	0	0	0
ENSG00000248491	0	0	0	0
ENSG00000248492	0	0	2	0
ENSG00000248493	0	0	0	0
ENSG00000248494	3	0	9	0
ENSG00000248496	0	0	0	0
ENSG00000248498	0	0	0	0
ENSG00000248503	1	1	16	8
ENSG00000248505	0	0	0	0
ENSG00000248506	0	0	0	0
ENSG00000248507	0	0	0	0
ENSG00000248508	1	0	3	1

ENSG00000248510	11	47	194	23
ENSG00000248511	0	0	0	0
ENSG00000248514	0	0	0	0
ENSG00000248515	0	0	0	0
ENSG00000248516	57	904	786	728
ENSG00000248517	0	0	0	0
ENSG00000248518	0	0	0	0
ENSG00000248521	0	0	0	0
ENSG00000248522	0	0	0	0
ENSG00000248525	0	0	0	0
ENSG00000248527	26530	12250	19763	105
ENSG00000248528	0	0	0	1
ENSG00000248529	0	0	0	0
ENSG00000248530	0	0	0	0
ENSG00000248531	0	0	0	0
ENSG00000248532	0	0	0	0
ENSG00000248533	0	0	0	0
ENSG00000248537	0	0	0	0
ENSG00000248538	1	8	0	9
ENSG00000248539	0	0	0	0
ENSG00000248540	0	0	0	0
ENSG00000248542	0	0	0	0
ENSG00000248543	0	0	0	0
ENSG00000248544	0	0	0	0
ENSG00000248545	0	0	0	0
ENSG00000248546	0	0	0	0
ENSG00000248547	0	0	0	0
ENSG00000248548	0	0	0	0
ENSG00000248550	0	0	0	0
ENSG00000248551	0	0	0	0
ENSG00000248552	0	0	0	0
ENSG00000248553	0	0	0	0
ENSG00000248554	0	0	0	0
ENSG00000248555	0	0	0	1
ENSG00000248557	0	0	0	0
ENSG00000248559	0	0	0	0
ENSG00000248560	0	0	0	0
ENSG00000248563	0	0	0	0
ENSG00000248564	0	0	0	0
ENSG00000248565	0	0	0	0
ENSG00000248567	0	0	0	0
ENSG00000248568	0	0	0	0
ENSG00000248569	0	0	0	0
ENSG00000248571	0	0	0	0
ENSG00000248572	0	0	0	0
ENSG00000248573	0	0	0	0

ENSG00000248574	0	0	0	0
ENSG00000248576	0	0	0	0
ENSG00000248577	0	0	0	0
ENSG00000248578	0	0	0	0
ENSG00000248583	0	0	0	0
ENSG00000248585	1	0	0	0
ENSG00000248586	1	0	0	0
ENSG00000248587	0	0	0	0
ENSG00000248588	0	0	0	0
ENSG00000248590	0	0	0	0
ENSG00000248591	0	0	0	0
ENSG00000248592	0	0	0	0
ENSG00000248593	69	115	112	156
ENSG00000248594	0	0	0	0
ENSG00000248596	0	0	1	0
ENSG00000248597	0	0	0	0
ENSG00000248599	4	19	14	1
ENSG00000248600	0	0	0	0
ENSG00000248601	0	0	0	0
ENSG00000248603	0	0	0	0
ENSG00000248605	0	0	0	0
ENSG00000248607	0	0	0	0
ENSG00000248608	0	0	0	0
ENSG00000248610	0	0	0	0
ENSG00000248611	0	0	0	0
ENSG00000248613	0	0	0	0
ENSG00000248616	0	0	0	0
ENSG00000248618	0	0	0	1
ENSG00000248621	0	0	0	0
ENSG00000248624	0	0	2	0
ENSG00000248625	0	0	0	0
ENSG00000248626	0	0	0	0
ENSG00000248627	0	0	0	0
ENSG00000248629	0	0	0	0
ENSG00000248631	0	0	0	0
ENSG00000248632	0	0	1	0
ENSG00000248633	0	0	0	0
ENSG00000248634	1	0	0	0
ENSG00000248635	0	0	0	0
ENSG00000248636	180	138	103	195
ENSG00000248637	0	0	0	0
ENSG00000248639	0	0	0	0
ENSG00000248640	0	0	0	0
ENSG00000248641	0	0	0	0
ENSG00000248642	0	0	0	0
ENSG00000248643	0	0	0	0

ENSG00000248645	0	0	0	0
ENSG00000248646	0	0	0	0
ENSG00000248647	0	0	0	0
ENSG00000248648	0	0	0	0
ENSG00000248651	0	0	0	0
ENSG00000248652	0	0	0	0
ENSG00000248654	0	0	0	0
ENSG00000248655	0	0	0	0
ENSG00000248656	0	0	0	0
ENSG00000248659	0	0	0	0
ENSG00000248660	0	0	0	0
ENSG00000248663	0	0	0	0
ENSG00000248664	0	23	92	72
ENSG00000248666	0	0	0	0
ENSG00000248667	0	0	0	0
ENSG00000248668	0	0	0	0
ENSG00000248669	0	0	0	0
ENSG00000248671	0	0	0	0
ENSG00000248672	0	0	0	0
ENSG00000248673	1	0	0	0
ENSG00000248674	0	0	0	0
ENSG00000248676	0	0	0	0
ENSG00000248677	1	1	5	1
ENSG00000248682	0	0	0	0
ENSG00000248684	0	23	0	0
ENSG00000248685	0	0	0	0
ENSG00000248686	0	0	0	0
ENSG00000248687	0	0	0	0
ENSG00000248690	0	0	0	0
ENSG00000248692	0	0	0	0
ENSG00000248693	0	0	3	0
ENSG00000248694	0	0	0	0
ENSG00000248696	0	0	0	0
ENSG00000248697	0	0	0	0
ENSG00000248698	0	0	11	26
ENSG00000248699	0	0	0	0
ENSG00000248701	0	0	0	0
ENSG00000248702	1	0	0	0
ENSG00000248703	0	0	0	0
ENSG00000248704	0	0	0	0
ENSG00000248705	0	0	0	0
ENSG00000248708	0	0	0	0
ENSG00000248709	0	0	0	0
ENSG00000248710	0	0	0	0
ENSG00000248711	0	0	0	0
ENSG00000248712	14	0	14	12

ENSG00000248713	0	12	4	1
ENSG00000248714	0	0	0	0
ENSG00000248715	0	0	0	0
ENSG00000248716	0	0	0	0
ENSG00000248717	0	0	0	0
ENSG00000248719	0	0	0	0
ENSG00000248720	0	0	0	0
ENSG00000248721	0	0	0	0
ENSG00000248722	0	0	0	0
ENSG00000248724	0	0	1	5
ENSG00000248725	0	0	0	0
ENSG00000248727	0	0	0	0
ENSG00000248729	0	0	0	0
ENSG00000248730	0	0	0	0
ENSG00000248731	1	0	1	0
ENSG00000248733	0	0	0	0
ENSG00000248734	0	0	5	5
ENSG00000248735	0	0	0	0
ENSG00000248736	0	0	0	0
ENSG00000248738	0	0	0	0
ENSG00000248739	0	0	0	0
ENSG00000248740	0	0	0	0
ENSG00000248744	0	0	0	0
ENSG00000248745	0	0	0	0
ENSG00000248746	0	0	0	0
ENSG00000248747	0	0	0	0
ENSG00000248748	0	0	0	0
ENSG00000248749	0	16	1	0
ENSG00000248750	0	0	0	0
ENSG00000248751	0	0	0	0
ENSG00000248752	0	0	4	2
ENSG00000248753	0	0	0	0
ENSG00000248755	0	0	0	0
ENSG00000248757	0	0	0	0
ENSG00000248758	0	0	0	0
ENSG00000248760	0	0	0	0
ENSG00000248761	0	0	0	0
ENSG00000248762	0	0	0	0
ENSG00000248763	0	0	0	0
ENSG00000248764	0	0	0	0
ENSG00000248765	0	0	0	0
ENSG00000248766	0	0	0	0
ENSG00000248767	0	0	0	0
ENSG00000248769	0	0	0	0
ENSG00000248770	0	0	0	0
ENSG00000248771	2	0	0	0

ENSG00000248772	0	0	0	0
ENSG00000248773	0	0	0	0
ENSG00000248774	0	0	2	7
ENSG00000248775	0	0	0	0
ENSG00000248777	0	0	0	0
ENSG00000248778	0	0	0	0
ENSG00000248779	0	0	0	0
ENSG00000248780	0	0	0	0
ENSG00000248781	0	0	0	0
ENSG00000248783	0	0	0	0
ENSG00000248785	0	0	0	0
ENSG00000248787	0	0	0	0
ENSG00000248789	0	0	0	0
ENSG00000248790	0	0	0	0
ENSG00000248791	0	0	0	0
ENSG00000248792	0	0	0	0
ENSG00000248794	0	0	0	0
ENSG00000248795	0	0	0	0
ENSG00000248796	0	0	0	0
ENSG00000248799	0	0	0	0
ENSG00000248801	0	0	0	0
ENSG00000248802	0	0	0	0
ENSG00000248803	0	0	0	0
ENSG00000248807	0	0	0	0
ENSG00000248809	0	0	0	0
ENSG00000248810	3	28	50	15
ENSG00000248813	0	0	0	0
ENSG00000248816	0	0	0	0
ENSG00000248817	0	0	0	0
ENSG00000248820	0	0	0	0
ENSG00000248821	0	0	0	0
ENSG00000248822	0	0	0	0
ENSG00000248824	0	0	6	0
ENSG00000248826	0	0	0	0
ENSG00000248827	0	0	0	0
ENSG00000248828	16	757	577	643
ENSG00000248830	0	0	0	0
ENSG00000248831	0	0	0	0
ENSG00000248832	0	0	0	0
ENSG00000248834	0	0	0	0
ENSG00000248835	1	0	2	0
ENSG00000248837	0	0	0	8
ENSG00000248838	0	0	0	0
ENSG00000248839	0	0	0	0
ENSG00000248840	0	0	0	0
ENSG00000248842	0	0	0	0

ENSG00000248843	0	0	0	0
ENSG00000248844	0	0	0	0
ENSG00000248846	0	0	0	0
ENSG00000248847	0	0	0	0
ENSG00000248848	21	21	18	0
ENSG00000248849	0	0	0	0
ENSG00000248850	0	0	0	0
ENSG00000248851	0	0	0	0
ENSG00000248853	0	0	0	0
ENSG00000248854	0	0	0	0
ENSG00000248858	0	21	12	1
ENSG00000248859	0	0	0	0
ENSG00000248861	0	0	0	0
ENSG00000248863	0	0	0	6
ENSG00000248864	0	0	0	0
ENSG00000248865	0	0	1	0
ENSG00000248866	0	0	0	3
ENSG00000248867	0	0	0	0
ENSG00000248869	0	0	0	0
ENSG00000248870	0	0	0	0
ENSG00000248871	0	0	0	0
ENSG00000248872	0	0	0	0
ENSG00000248873	0	0	0	0
ENSG00000248874	0	27	14	13
ENSG00000248876	0	0	0	0
ENSG00000248877	0	0	0	0
ENSG00000248878	0	0	0	0
ENSG00000248880	0	0	0	0
ENSG00000248881	0	1	2	0
ENSG00000248883	0	0	0	0
ENSG00000248884	6	0	17	14
ENSG00000248885	0	0	0	0
ENSG00000248886	0	0	0	0
ENSG00000248890	0	0	0	0
ENSG00000248891	0	0	0	0
ENSG00000248893	0	0	0	0
ENSG00000248895	0	0	0	0
ENSG00000248896	0	0	0	0
ENSG00000248898	1	16	6	49
ENSG00000248901	0	0	0	0
ENSG00000248903	0	0	0	0
ENSG00000248905	7	1	5	16
ENSG00000248906	0	0	0	0
ENSG00000248907	0	0	0	0
ENSG00000248908	0	0	0	0
ENSG00000248909	0	0	0	0

ENSG00000248911	0	0	0	0
ENSG00000248912	0	0	0	0
ENSG00000248913	0	0	0	0
ENSG00000248915	0	0	0	0
ENSG00000248916	0	0	0	0
ENSG00000248918	0	0	0	0
ENSG00000248919	0	0	0	0
ENSG00000248920	0	0	0	0
ENSG00000248921	0	0	0	0
ENSG00000248923	2	0	0	1
ENSG00000248924	0	0	0	0
ENSG00000248925	1	13	4	20
ENSG00000248926	0	0	0	0
ENSG00000248927	1	0	3	5
ENSG00000248928	0	0	0	0
ENSG00000248929	0	0	0	0
ENSG00000248930	0	0	0	0
ENSG00000248931	0	0	0	0
ENSG00000248932	0	35	25	64
ENSG00000248933	0	0	0	0
ENSG00000248935	0	0	0	0
ENSG00000248936	1	0	0	0
ENSG00000248939	0	0	0	0
ENSG00000248942	0	0	0	0
ENSG00000248943	0	0	0	0
ENSG00000248944	0	0	0	0
ENSG00000248946	0	0	0	0
ENSG00000248949	0	0	0	0
ENSG00000248950	0	0	0	0
ENSG00000248951	0	0	0	0
ENSG00000248954	0	0	0	0
ENSG00000248955	0	0	0	0
ENSG00000248956	0	0	0	0
ENSG00000248958	0	0	0	0
ENSG00000248962	0	0	0	0
ENSG00000248964	0	0	0	0
ENSG00000248965	0	0	0	0
ENSG00000248966	0	0	0	0
ENSG00000248967	0	0	1	0
ENSG00000248968	0	0	0	0
ENSG00000248969	0	0	0	5
ENSG00000248971	1	1	26	22
ENSG00000248973	0	0	0	0
ENSG00000248975	0	0	0	0
ENSG00000248977	0	0	0	0
ENSG00000248978	0	0	0	0

ENSG00000248979	0	0	0	0
ENSG00000248980	0	0	18	9
ENSG00000248984	0	0	0	0
ENSG00000248986	0	0	0	0
ENSG00000248987	0	0	0	0
ENSG00000248988	0	0	0	0
ENSG00000248989	0	0	0	0
ENSG00000248990	0	0	0	0
ENSG00000248991	0	0	0	0
ENSG00000248993	0	0	0	0
ENSG00000248994	0	0	0	0
ENSG00000248995	0	0	0	0
ENSG00000248996	0	0	0	0
ENSG00000248998	0	0	0	0
ENSG00000248999	0	0	0	0
ENSG00000249000	0	0	0	0
ENSG00000249001	0	0	0	0
ENSG00000249002	0	0	0	0
ENSG00000249003	0	0	0	0
ENSG00000249004	0	0	0	0
ENSG00000249005	0	0	0	0
ENSG00000249006	0	0	0	0
ENSG00000249007	0	0	0	0
ENSG00000249008	0	0	0	0
ENSG00000249012	0	1	0	0
ENSG00000249013	0	0	5	4
ENSG00000249014	0	0	0	0
ENSG00000249016	0	0	0	0
ENSG00000249017	0	0	0	0
ENSG00000249018	0	0	0	0
ENSG00000249019	0	0	0	0
ENSG00000249020	0	0	0	0
ENSG00000249021	0	0	0	0
ENSG00000249022	0	0	0	0
ENSG00000249023	0	0	0	0
ENSG00000249025	0	0	0	0
ENSG00000249026	0	0	0	0
ENSG00000249028	0	0	0	0
ENSG00000249030	0	0	0	0
ENSG00000249031	0	0	0	0
ENSG00000249032	0	0	0	0
ENSG00000249034	0	0	0	0
ENSG00000249035	0	279	436	248
ENSG00000249036	0	0	0	0
ENSG00000249038	0	0	0	0
ENSG00000249041	0	0	0	4

ENSG00000249042	1	0	0	6
ENSG00000249044	0	0	1	3
ENSG00000249045	0	0	0	0
ENSG00000249047	0	0	0	0
ENSG00000249048	0	0	0	0
ENSG00000249049	0	0	0	0
ENSG00000249050	0	0	0	0
ENSG00000249051	0	0	0	0
ENSG00000249052	0	0	0	0
ENSG00000249053	0	0	0	0
ENSG00000249054	0	0	0	0
ENSG00000249055	0	0	0	0
ENSG00000249056	0	0	0	0
ENSG00000249057	0	0	0	0
ENSG00000249061	0	0	8	14
ENSG00000249064	0	0	0	0
ENSG00000249065	0	0	0	0
ENSG00000249066	0	0	0	0
ENSG00000249068	0	0	0	3
ENSG00000249069	1	0	1	3
ENSG00000249071	0	0	0	0
ENSG00000249072	0	0	0	0
ENSG00000249073	0	0	0	0
ENSG00000249074	0	0	0	0
ENSG00000249076	0	0	0	0
ENSG00000249077	0	0	0	0
ENSG00000249079	0	0	0	0
ENSG00000249081	0	0	0	0
ENSG00000249082	0	0	0	0
ENSG00000249084	0	0	0	0
ENSG00000249085	0	0	0	0
ENSG00000249086	0	0	0	0
ENSG00000249087	0	6	0	4
ENSG00000249089	0	0	0	0
ENSG00000249091	0	0	0	0
ENSG00000249092	0	0	0	0
ENSG00000249094	0	0	0	0
ENSG00000249096	5	0	0	0
ENSG00000249098	0	0	0	0
ENSG00000249099	0	0	0	0
ENSG00000249100	0	0	0	0
ENSG00000249101	0	0	0	0
ENSG00000249102	0	0	0	0
ENSG00000249103	0	0	0	0
ENSG00000249104	0	0	0	0
ENSG00000249105	0	0	0	0

ENSG00000249106	0	0	0	0
ENSG00000249109	0	0	0	0
ENSG00000249111	0	0	0	0
ENSG00000249112	0	0	0	0
ENSG00000249114	0	0	0	0
ENSG00000249115	3	0	6	0
ENSG00000249116	0	0	0	0
ENSG00000249119	1	5	3	7
ENSG00000249122	0	0	0	0
ENSG00000249123	0	0	0	0
ENSG00000249125	0	0	0	0
ENSG00000249127	0	0	0	0
ENSG00000249128	0	0	0	0
ENSG00000249129	0	0	1	0
ENSG00000249131	0	0	0	0
ENSG00000249135	0	0	0	0
ENSG00000249138	0	0	0	0
ENSG00000249139	0	0	0	0
ENSG00000249140	2	8	7	0
ENSG00000249141	0	0	0	0
ENSG00000249142	0	0	0	0
ENSG00000249145	0	0	0	0
ENSG00000249148	0	0	0	0
ENSG00000249149	0	0	0	0
ENSG00000249150	0	0	0	0
ENSG00000249152	0	0	0	0
ENSG00000249153	0	0	0	0
ENSG00000249156	0	0	0	0
ENSG00000249157	0	0	0	0
ENSG00000249158	0	0	0	0
ENSG00000249159	0	0	0	0
ENSG00000249160	0	0	0	0
ENSG00000249162	0	0	0	0
ENSG00000249163	0	0	0	0
ENSG00000249166	0	0	0	0
ENSG00000249167	0	0	0	3
ENSG00000249169	0	0	0	0
ENSG00000249170	0	0	0	0
ENSG00000249171	0	0	0	0
ENSG00000249173	0	0	0	0
ENSG00000249174	0	0	0	0
ENSG00000249175	2	18	2	0
ENSG00000249176	0	0	0	0
ENSG00000249177	0	0	0	0
ENSG00000249179	19	27	91	11
ENSG00000249180	0	14	6	2

ENSG00000249183	0	0	0	0
ENSG00000249184	0	0	0	0
ENSG00000249186	0	0	0	0
ENSG00000249188	0	0	4	1
ENSG00000249189	0	0	0	0
ENSG00000249191	0	0	0	0
ENSG00000249192	0	0	0	0
ENSG00000249193	0	0	0	0
ENSG00000249194	0	0	0	0
ENSG00000249196	0	0	0	0
ENSG00000249197	0	0	0	0
ENSG00000249198	0	0	0	0
ENSG00000249199	0	0	0	0
ENSG00000249200	0	0	0	0
ENSG00000249201	0	0	0	0
ENSG00000249203	107	0	0	0
ENSG00000249206	0	0	0	0
ENSG00000249207	0	0	42	18
ENSG00000249209	0	0	0	0
ENSG00000249210	0	0	0	0
ENSG00000249212	0	0	4	1
ENSG00000249213	0	0	0	0
ENSG00000249214	0	0	0	0
ENSG00000249215	0	0	0	0
ENSG00000249216	0	0	0	0
ENSG00000249219	0	0	0	0
ENSG00000249222	1	0	0	0
ENSG00000249225	0	0	0	0
ENSG00000249226	0	0	0	0
ENSG00000249228	1	0	0	5
ENSG00000249229	0	0	0	0
ENSG00000249230	0	0	0	0
ENSG00000249231	0	0	0	0
ENSG00000249234	0	0	0	0
ENSG00000249235	0	0	0	0
ENSG00000249236	1	0	0	0
ENSG00000249237	0	0	0	0
ENSG00000249238	0	0	0	0
ENSG00000249239	0	0	0	0
ENSG00000249240	0	0	0	0
ENSG00000249241	1	0	0	0
ENSG00000249242	11	28	32	124
ENSG00000249244	0	0	0	0
ENSG00000249245	0	0	0	1
ENSG00000249247	0	0	0	0
ENSG00000249249	1	30	10	17

ENSG00000249251	0	0	0	0
ENSG00000249252	0	20	4	0
ENSG00000249253	0	0	0	0
ENSG00000249255	0	0	0	0
ENSG00000249256	0	0	0	0
ENSG00000249257	0	0	0	0
ENSG00000249258	0	0	1	1
ENSG00000249259	0	0	0	0
ENSG00000249261	0	0	0	0
ENSG00000249262	0	0	0	0
ENSG00000249263	0	0	0	0
ENSG00000249264	0	0	0	0
ENSG00000249265	0	0	0	0
ENSG00000249266	0	0	0	0
ENSG00000249267	0	0	0	3
ENSG00000249269	0	0	0	0
ENSG00000249270	0	0	0	0
ENSG00000249271	0	0	0	0
ENSG00000249272	0	0	0	0
ENSG00000249274	1	23	13	38
ENSG00000249275	0	0	0	0
ENSG00000249276	0	0	0	0
ENSG00000249277	0	0	0	0
ENSG00000249278	0	0	0	0
ENSG00000249279	0	0	0	0
ENSG00000249282	0	0	0	0
ENSG00000249283	0	0	0	0
ENSG00000249284	0	0	0	0
ENSG00000249285	0	0	0	0
ENSG00000249286	0	0	3	13
ENSG00000249287	0	0	0	0
ENSG00000249288	0	0	0	0
ENSG00000249289	0	0	0	0
ENSG00000249290	0	0	0	0
ENSG00000249293	0	0	0	0
ENSG00000249295	0	0	0	0
ENSG00000249297	0	0	0	0
ENSG00000249301	0	0	0	0
ENSG00000249302	0	0	0	0
ENSG00000249304	0	0	0	0
ENSG00000249305	0	0	0	0
ENSG00000249306	0	0	0	0
ENSG00000249307	716	1354	1304	1698
ENSG00000249309	0	0	0	0
ENSG00000249310	0	0	0	0
ENSG00000249311	0	0	0	0

ENSG00000249312	0	0	0	0
ENSG00000249316	0	0	0	0
ENSG00000249317	0	0	0	0
ENSG00000249318	0	0	0	0
ENSG00000249319	0	0	0	0
ENSG00000249320	0	0	0	0
ENSG00000249321	0	0	0	0
ENSG00000249326	0	0	0	0
ENSG00000249328	0	0	0	0
ENSG00000249329	0	0	0	0
ENSG00000249330	0	0	0	0
ENSG00000249332	0	0	0	0
ENSG00000249333	0	0	0	0
ENSG00000249334	0	0	0	3
ENSG00000249335	0	0	0	0
ENSG00000249336	0	0	0	0
ENSG00000249337	0	0	0	0
ENSG00000249338	0	0	0	0
ENSG00000249339	0	0	0	0
ENSG00000249341	2	0	1	2
ENSG00000249343	0	0	0	0
ENSG00000249345	0	0	0	0
ENSG00000249346	0	0	0	0
ENSG00000249347	0	0	0	0
ENSG00000249348	92	674	786	738
ENSG00000249349	1	0	0	0
ENSG00000249351	0	0	0	0
ENSG00000249352	0	0	0	1
ENSG00000249353	0	0	1	1
ENSG00000249356	0	0	2	0
ENSG00000249357	0	0	0	0
ENSG00000249358	0	0	0	0
ENSG00000249359	0	0	0	1
ENSG00000249360	0	0	0	0
ENSG00000249362	0	0	0	0
ENSG00000249363	0	0	0	4
ENSG00000249364	2	0	0	0
ENSG00000249365	0	0	0	0
ENSG00000249367	0	0	0	0
ENSG00000249368	0	0	0	1
ENSG00000249372	0	0	0	0
ENSG00000249373	0	0	0	0
ENSG00000249375	0	0	0	0
ENSG00000249376	0	0	0	0
ENSG00000249378	0	0	0	0
ENSG00000249379	0	0	0	0

ENSG00000249380	0	0	0	0
ENSG00000249381	0	0	0	0
ENSG00000249382	0	0	0	0
ENSG00000249383	0	0	0	0
ENSG00000249386	0	0	0	0
ENSG00000249387	0	0	0	0
ENSG00000249388	0	0	0	0
ENSG00000249391	0	0	0	1
ENSG00000249392	0	0	0	0
ENSG00000249393	0	0	0	0
ENSG00000249395	0	0	1	0
ENSG00000249396	0	0	0	0
ENSG00000249400	0	0	0	0
ENSG00000249403	0	0	0	0
ENSG00000249404	0	0	0	0
ENSG00000249405	0	0	0	0
ENSG00000249406	0	0	0	0
ENSG00000249407	0	0	0	0
ENSG00000249409	0	0	3	3
ENSG00000249410	0	0	0	0
ENSG00000249411	0	0	0	0
ENSG00000249412	0	0	0	0
ENSG00000249413	0	0	0	0
ENSG00000249415	0	0	0	0
ENSG00000249416	0	0	0	0
ENSG00000249417	0	0	0	0
ENSG00000249418	0	0	0	0
ENSG00000249419	0	0	0	0
ENSG00000249421	0	0	0	0
ENSG00000249423	0	0	0	0
ENSG00000249425	0	0	0	0
ENSG00000249426	0	0	0	0
ENSG00000249427	0	0	0	0
ENSG00000249428	0	0	0	0
ENSG00000249429	0	0	0	0
ENSG00000249430	0	0	0	0
ENSG00000249433	0	0	0	0
ENSG00000249434	0	0	0	0
ENSG00000249435	0	0	0	0
ENSG00000249436	2	23	4	21
ENSG00000249437	2	0	12	14
ENSG00000249438	0	0	0	0
ENSG00000249439	0	0	0	0
ENSG00000249441	0	0	0	0
ENSG00000249443	0	0	0	0
ENSG00000249444	0	0	0	0

ENSG00000249446	0	0	0	0
ENSG00000249448	0	0	0	0
ENSG00000249449	0	0	0	0
ENSG00000249451	0	0	0	0
ENSG00000249452	0	0	0	0
ENSG00000249453	0	0	0	0
ENSG00000249454	0	0	0	0
ENSG00000249456	0	0	0	0
ENSG00000249458	0	0	0	2
ENSG00000249459	0	0	9	24
ENSG00000249460	0	0	0	0
ENSG00000249462	0	0	0	0
ENSG00000249463	0	0	0	0
ENSG00000249464	5	0	7	5
ENSG00000249465	0	0	0	0
ENSG00000249467	0	0	0	0
ENSG00000249471	0	0	2	0
ENSG00000249472	0	0	0	0
ENSG00000249474	0	0	0	0
ENSG00000249476	0	0	0	0
ENSG00000249478	3	0	6	11
ENSG00000249479	0	0	0	0
ENSG00000249480	0	0	0	0
ENSG00000249481	0	0	0	0
ENSG00000249482	0	0	0	0
ENSG00000249483	1	0	3	6
ENSG00000249484	0	0	0	0
ENSG00000249485	0	0	0	0
ENSG00000249486	0	0	0	0
ENSG00000249487	0	0	0	0
ENSG00000249488	0	0	0	0
ENSG00000249489	0	0	0	0
ENSG00000249490	0	0	0	0
ENSG00000249491	0	0	0	0
ENSG00000249492	0	0	1	0
ENSG00000249493	0	0	0	0
ENSG00000249494	0	0	0	0
ENSG00000249495	0	0	0	0
ENSG00000249497	0	0	0	0
ENSG00000249500	0	0	0	0
ENSG00000249501	0	0	0	0
ENSG00000249502	2	0	4	11
ENSG00000249503	0	0	0	0
ENSG00000249504	0	0	0	0
ENSG00000249505	0	0	0	0
ENSG00000249506	0	0	0	0

ENSG00000249509	0	0	0	0
ENSG00000249510	0	0	0	0
ENSG00000249513	0	0	0	0
ENSG00000249514	0	0	0	0
ENSG00000249515	0	0	0	0
ENSG00000249516	0	0	0	0
ENSG00000249518	0	0	0	0
ENSG00000249519	0	0	0	0
ENSG00000249520	0	0	0	0
ENSG00000249521	0	0	0	0
ENSG00000249522	0	0	0	0
ENSG00000249525	0	0	0	0
ENSG00000249526	0	0	1	0
ENSG00000249531	0	0	0	0
ENSG00000249532	0	0	5	0
ENSG00000249534	0	0	2	0
ENSG00000249539	0	0	0	0
ENSG00000249540	0	0	0	0
ENSG00000249541	0	0	0	0
ENSG00000249542	0	0	0	0
ENSG00000249545	0	0	0	0
ENSG00000249547	0	0	0	0
ENSG00000249550	0	0	0	0
ENSG00000249551	0	0	0	0
ENSG00000249553	0	0	0	0
ENSG00000249555	0	0	0	0
ENSG00000249557	0	0	0	0
ENSG00000249558	0	0	0	0
ENSG00000249562	0	0	0	0
ENSG00000249563	0	0	0	0
ENSG00000249564	0	0	0	0
ENSG00000249565	0	0	1	0
ENSG00000249568	0	0	0	0
ENSG00000249571	0	0	0	0
ENSG00000249572	0	0	2	0
ENSG00000249574	0	0	1	3
ENSG00000249577	0	0	0	0
ENSG00000249579	0	0	0	0
ENSG00000249580	0	0	0	0
ENSG00000249581	0	0	0	0
ENSG00000249582	0	0	0	0
ENSG00000249584	0	0	0	0
ENSG00000249588	0	0	0	0
ENSG00000249590	0	0	0	0
ENSG00000249592	10	8	7	5
ENSG00000249593	0	0	0	0

ENSG00000249595	0	0	0	0
ENSG00000249599	0	0	0	0
ENSG00000249600	0	0	0	0
ENSG00000249601	2	0	0	4
ENSG00000249602	0	0	2	0
ENSG00000249604	0	0	0	0
ENSG00000249605	0	0	0	0
ENSG00000249606	0	0	0	0
ENSG00000249607	0	0	0	0
ENSG00000249609	0	0	0	0
ENSG00000249610	0	0	0	0
ENSG00000249612	0	0	0	0
ENSG00000249613	0	0	0	0
ENSG00000249614	188	674	690	506
ENSG00000249615	0	0	8	1
ENSG00000249616	0	0	0	0
ENSG00000249617	0	0	0	0
ENSG00000249618	0	0	0	0
ENSG00000249619	0	0	0	0
ENSG00000249620	0	0	0	0
ENSG00000249621	0	0	0	0
ENSG00000249623	0	0	0	0
ENSG00000249624	0	0	2	0
ENSG00000249626	0	0	0	0
ENSG00000249627	0	0	0	3
ENSG00000249628	1	0	0	0
ENSG00000249631	0	0	0	0
ENSG00000249633	0	0	0	0
ENSG00000249634	0	0	0	0
ENSG00000249635	0	0	0	5
ENSG00000249637	1	0	0	1
ENSG00000249638	0	0	0	0
ENSG00000249639	0	0	14	2
ENSG00000249641	0	0	0	0
ENSG00000249642	0	0	0	0
ENSG00000249643	0	0	0	0
ENSG00000249645	0	0	0	3
ENSG00000249646	778	40	66	47
ENSG00000249647	0	0	0	0
ENSG00000249649	0	0	0	0
ENSG00000249650	0	0	0	0
ENSG00000249654	0	0	0	0
ENSG00000249655	0	0	0	0
ENSG00000249658	0	0	0	0
ENSG00000249661	0	0	0	0
ENSG00000249662	1	0	0	0

ENSG00000249663	0	0	0	0
ENSG00000249664	0	0	0	0
ENSG00000249666	0	0	0	0
ENSG00000249667	0	0	0	0
ENSG00000249668	0	0	0	0
ENSG00000249669	0	0	0	0
ENSG00000249673	11	61	49	69
ENSG00000249675	0	4	0	0
ENSG00000249677	0	0	0	0
ENSG00000249678	0	0	0	0
ENSG00000249679	0	0	0	0
ENSG00000249680	0	0	0	0
ENSG00000249681	0	0	0	0
ENSG00000249684	46	173	23	28
ENSG00000249685	1	0	16	35
ENSG00000249686	0	0	0	0
ENSG00000249688	0	0	0	0
ENSG00000249689	0	0	0	0
ENSG00000249690	0	0	0	0
ENSG00000249691	0	0	0	0
ENSG00000249692	0	0	0	0
ENSG00000249693	0	0	2	3
ENSG00000249694	0	0	1	7
ENSG00000249695	0	0	0	0
ENSG00000249697	0	0	0	0
ENSG00000249698	0	0	0	0
ENSG00000249699	1	3	1	3
ENSG00000249700	1	0	4	6
ENSG00000249704	0	0	0	0
ENSG00000249706	0	0	0	0
ENSG00000249708	0	0	0	0
ENSG00000249709	0	1	0	3
ENSG00000249710	0	0	0	0
ENSG00000249713	0	0	1	0
ENSG00000249715	0	0	2	1
ENSG00000249717	0	0	0	0
ENSG00000249719	0	0	0	0
ENSG00000249721	1	40	2	0
ENSG00000249722	0	0	1	0
ENSG00000249725	0	0	0	0
ENSG00000249726	0	0	0	0
ENSG00000249727	0	0	0	0
ENSG00000249729	0	0	1	0
ENSG00000249730	0	0	0	0
ENSG00000249731	14	163	210	204
ENSG00000249734	0	0	0	0

ENSG00000249735	0	0	0	0
ENSG00000249736	9	6	1	0
ENSG00000249737	0	0	0	0
ENSG00000249738	0	0	0	0
ENSG00000249740	0	0	0	0
ENSG00000249741	0	0	0	0
ENSG00000249742	0	0	0	0
ENSG00000249743	0	0	0	0
ENSG00000249744	0	0	0	0
ENSG00000249745	0	0	0	0
ENSG00000249746	0	0	0	0
ENSG00000249747	0	0	0	0
ENSG00000249748	0	0	0	0
ENSG00000249751	2	0	1	5
ENSG00000249752	0	0	0	0
ENSG00000249753	0	0	0	0
ENSG00000249754	0	0	0	0
ENSG00000249755	0	0	0	0
ENSG00000249758	0	784	559	233
ENSG00000249761	0	0	0	0
ENSG00000249762	0	0	0	0
ENSG00000249763	0	0	0	0
ENSG00000249764	0	0	0	0
ENSG00000249766	0	0	0	0
ENSG00000249767	1	0	3	0
ENSG00000249768	0	0	0	0
ENSG00000249770	0	0	0	0
ENSG00000249771	0	0	0	0
ENSG00000249772	0	0	1	0
ENSG00000249773	0	0	0	0
ENSG00000249774	0	0	0	0
ENSG00000249776	0	0	0	0
ENSG00000249777	0	0	0	0
ENSG00000249778	0	0	0	0
ENSG00000249779	0	0	0	0
ENSG00000249780	0	0	0	0
ENSG00000249781	0	0	0	0
ENSG00000249782	0	0	0	0
ENSG00000249784	0	0	0	0
ENSG00000249785	0	0	0	0
ENSG00000249786	0	0	23	1
ENSG00000249787	0	0	0	0
ENSG00000249790	4	33	12	39
ENSG00000249791	0	0	0	0
ENSG00000249792	0	0	0	0
ENSG00000249795	0	0	0	0

ENSG00000249797	0	0	4	0
ENSG00000249798	0	0	0	0
ENSG00000249799	0	0	0	0
ENSG00000249803	0	0	0	0
ENSG00000249806	0	0	11	14
ENSG00000249807	0	0	0	0
ENSG00000249808	0	0	0	0
ENSG00000249811	0	0	0	0
ENSG00000249814	0	0	0	0
ENSG00000249815	0	0	0	0
ENSG00000249816	14	52	41	52
ENSG00000249818	0	0	0	0
ENSG00000249819	0	0	0	0
ENSG00000249820	0	0	0	0
ENSG00000249823	0	0	0	0
ENSG00000249825	0	0	0	0
ENSG00000249828	0	0	0	0
ENSG00000249829	0	0	0	0
ENSG00000249830	0	0	0	0
ENSG00000249831	0	0	0	0
ENSG00000249833	0	0	0	0
ENSG00000249834	0	0	0	0
ENSG00000249835	0	0	0	0
ENSG00000249837	0	0	0	0
ENSG00000249838	0	0	0	0
ENSG00000249839	0	0	0	0
ENSG00000249840	0	0	0	0
ENSG00000249842	0	0	0	0
ENSG00000249843	0	0	0	0
ENSG00000249844	0	0	0	0
ENSG00000249846	0	0	1	1
ENSG00000249847	0	0	0	0
ENSG00000249848	0	0	0	0
ENSG00000249849	0	0	0	0
ENSG00000249850	0	0	3	0
ENSG00000249851	0	0	0	0
ENSG00000249852	0	0	0	0
ENSG00000249853	0	0	0	0
ENSG00000249854	0	0	0	0
ENSG00000249855	0	0	0	1
ENSG00000249856	0	43	11	28
ENSG00000249857	0	0	0	0
ENSG00000249858	0	0	0	0
ENSG00000249859	2	70	31	25
ENSG00000249860	0	0	0	0
ENSG00000249861	0	0	0	0

ENSG00000249863	0	0	3	5
ENSG00000249864	1	0	0	1
ENSG00000249865	0	0	0	0
ENSG00000249866	0	0	0	0
ENSG00000249867	0	0	0	0
ENSG00000249868	0	0	0	0
ENSG00000249869	0	0	0	0
ENSG00000249870	0	0	0	0
ENSG00000249873	0	0	0	0
ENSG00000249875	0	0	0	0
ENSG00000249876	0	0	0	0
ENSG00000249877	0	0	0	0
ENSG00000249878	0	0	0	0
ENSG00000249880	0	0	0	0
ENSG00000249881	0	0	0	0
ENSG00000249882	0	0	0	0
ENSG00000249883	0	0	0	0
ENSG00000249884	0	0	0	0
ENSG00000249885	0	0	0	0
ENSG00000249887	0	0	0	0
ENSG00000249888	0	0	0	0
ENSG00000249889	0	0	0	0
ENSG00000249890	0	0	0	0
ENSG00000249891	0	0	0	0
ENSG00000249892	0	0	0	0
ENSG00000249893	0	0	0	0
ENSG00000249894	0	1	3	0
ENSG00000249896	0	0	0	0
ENSG00000249898	182	2046	923	1239
ENSG00000249899	4	14	35	28
ENSG00000249901	0	0	0	0
ENSG00000249904	0	0	0	0
ENSG00000249906	0	0	0	0
ENSG00000249908	0	0	0	0
ENSG00000249909	0	0	0	0
ENSG00000249910	0	0	0	0
ENSG00000249911	0	0	0	0
ENSG00000249912	0	0	0	0
ENSG00000249913	0	0	1	2
ENSG00000249915	136	248	164	287
ENSG00000249916	0	0	0	0
ENSG00000249917	0	0	0	8
ENSG00000249919	0	0	0	0
ENSG00000249920	0	0	0	0
ENSG00000249921	0	0	0	0
ENSG00000249923	1	0	0	0

ENSG00000249924	0	0	0	0
ENSG00000249926	0	0	0	0
ENSG00000249927	0	0	0	0
ENSG00000249928	0	0	0	0
ENSG00000249930	0	0	0	0
ENSG00000249931	0	0	0	0
ENSG00000249932	0	0	0	0
ENSG00000249934	0	0	0	0
ENSG00000249936	2	4	9	17
ENSG00000249937	2	0	0	12
ENSG00000249941	0	0	0	0
ENSG00000249942	0	0	0	0
ENSG00000249943	0	0	0	0
ENSG00000249944	0	0	0	0
ENSG00000249945	0	0	0	0
ENSG00000249947	0	0	0	0
ENSG00000249948	1	0	0	0
ENSG00000249950	0	0	0	0
ENSG00000249951	0	0	0	0
ENSG00000249955	0	20	101	45
ENSG00000249956	0	0	0	0
ENSG00000249958	0	0	0	0
ENSG00000249959	0	0	0	0
ENSG00000249960	0	0	0	0
ENSG00000249961	0	56	70	89
ENSG00000249962	0	0	0	0
ENSG00000249963	0	0	0	0
ENSG00000249965	0	0	0	0
ENSG00000249966	0	0	0	0
ENSG00000249967	0	0	0	0
ENSG00000249970	0	0	0	0
ENSG00000249971	0	0	0	0
ENSG00000249973	0	0	0	0
ENSG00000249975	0	0	0	0
ENSG00000249976	0	0	0	0
ENSG00000249977	0	0	0	0
ENSG00000249978	0	0	0	4
ENSG00000249981	0	0	0	0
ENSG00000249982	0	0	0	0
ENSG00000249984	0	0	0	0
ENSG00000249985	0	0	0	0
ENSG00000249986	0	0	0	0
ENSG00000249987	0	0	0	0
ENSG00000249988	0	0	1	0
ENSG00000249990	0	0	0	0
ENSG00000249992	782	677	544	467

ENSG00000249993	0	0	0	0
ENSG00000249994	0	0	0	0
ENSG00000249995	0	0	0	0
ENSG00000249996	0	0	0	0
ENSG00000249997	0	0	0	0
ENSG00000249998	0	0	0	0
ENSG00000250001	0	0	0	0
ENSG00000250003	0	0	0	0
ENSG00000250006	0	0	2	0
ENSG00000250007	0	0	0	0
ENSG00000250011	0	0	0	0
ENSG00000250012	0	0	0	0
ENSG00000250013	0	0	0	0
ENSG00000250015	0	0	0	0
ENSG00000250016	0	0	0	0
ENSG00000250017	3	0	0	0
ENSG00000250020	0	0	0	0
ENSG00000250021	0	0	0	0
ENSG00000250024	0	0	0	0
ENSG00000250025	0	0	0	0
ENSG00000250026	0	0	0	0
ENSG00000250027	0	0	0	0
ENSG00000250030	0	0	0	0
ENSG00000250031	0	0	0	0
ENSG00000250032	0	0	0	0
ENSG00000250033	1	20	0	1
ENSG00000250034	0	0	0	0
ENSG00000250036	0	0	0	0
ENSG00000250037	0	0	0	0
ENSG00000250038	0	0	1	9
ENSG00000250039	0	0	0	24
ENSG00000250040	0	0	0	0
ENSG00000250041	0	0	0	0
ENSG00000250042	0	0	0	0
ENSG00000250043	0	0	0	0
ENSG00000250045	0	0	0	0
ENSG00000250046	0	0	0	0
ENSG00000250047	0	0	0	0
ENSG00000250048	0	0	0	4
ENSG00000250049	0	0	0	0
ENSG00000250050	0	0	0	0
ENSG00000250053	0	0	0	1
ENSG00000250055	0	0	0	0
ENSG00000250056	0	0	0	0
ENSG00000250057	1	0	2	8
ENSG00000250060	0	0	0	0

ENSG00000250061	2	15	10	14
ENSG00000250062	0	0	3	0
ENSG00000250064	0	0	0	0
ENSG00000250066	0	0	0	0
ENSG00000250067	0	0	0	0
ENSG00000250068	0	0	0	0
ENSG00000250069	0	0	0	0
ENSG00000250071	0	0	0	0
ENSG00000250072	8	320	172	9
ENSG00000250073	229	304	358	164
ENSG00000250074	0	0	0	0
ENSG00000250075	0	2	3	3
ENSG00000250076	0	0	0	0
ENSG00000250078	0	0	0	0
ENSG00000250079	0	0	0	0
ENSG00000250080	0	0	0	0
ENSG00000250081	0	0	0	0
ENSG00000250082	0	0	0	0
ENSG00000250084	0	0	0	0
ENSG00000250088	0	0	0	0
ENSG00000250090	0	0	0	0
ENSG00000250091	47	111	20	76
ENSG00000250092	0	0	0	0
ENSG00000250094	0	0	0	0
ENSG00000250095	0	0	0	0
ENSG00000250098	0	0	0	0
ENSG00000250099	0	0	0	0
ENSG00000250100	0	0	0	0
ENSG00000250101	0	0	3	1
ENSG00000250102	0	0	0	1
ENSG00000250103	0	0	0	0
ENSG00000250105	0	0	0	0
ENSG00000250106	0	0	0	0
ENSG00000250107	0	0	0	0
ENSG00000250111	0	0	1	0
ENSG00000250113	0	0	0	0
ENSG00000250114	0	0	0	0
ENSG00000250115	0	0	0	0
ENSG00000250116	0	0	0	0
ENSG00000250118	0	0	0	0
ENSG00000250120	0	0	0	0
ENSG00000250122	0	0	0	0
ENSG00000250124	0	0	0	0
ENSG00000250125	0	16	0	10
ENSG00000250126	0	0	0	0
ENSG00000250127	2	16	0	2

ENSG00000250129	0	0	0	0
ENSG00000250130	0	0	0	0
ENSG00000250131	0	0	8	9
ENSG00000250132	1	19	10	9
ENSG00000250133	0	0	0	0
ENSG00000250135	0	0	0	0
ENSG00000250136	0	0	0	0
ENSG00000250137	0	0	0	0
ENSG00000250138	0	0	0	0
ENSG00000250140	0	0	0	0
ENSG00000250141	0	0	0	0
ENSG00000250144	0	0	0	0
ENSG00000250145	0	0	0	0
ENSG00000250147	0	0	0	0
ENSG00000250148	0	0	0	4
ENSG00000250149	0	0	0	0
ENSG00000250150	0	0	0	0
ENSG00000250151	0	0	0	0
ENSG00000250154	0	0	0	0
ENSG00000250155	12	143	59	364
ENSG00000250156	0	0	0	0
ENSG00000250158	0	0	0	0
ENSG00000250159	0	5	20	20
ENSG00000250161	0	0	0	0
ENSG00000250162	0	0	0	0
ENSG00000250163	0	0	1	8
ENSG00000250164	0	0	0	4
ENSG00000250166	0	0	0	0
ENSG00000250167	0	0	0	0
ENSG00000250169	0	0	0	0
ENSG00000250170	0	0	0	0
ENSG00000250173	0	0	0	0
ENSG00000250174	0	58	21	11
ENSG00000250180	0	0	0	0
ENSG00000250182	0	0	1	0
ENSG00000250183	0	0	0	0
ENSG00000250185	0	0	0	0
ENSG00000250186	0	0	0	0
ENSG00000250189	0	0	0	0
ENSG00000250190	0	0	0	0
ENSG00000250191	0	5	0	0
ENSG00000250192	0	0	0	0
ENSG00000250193	0	0	0	0
ENSG00000250194	0	0	0	0
ENSG00000250195	0	0	2	4
ENSG00000250197	0	0	0	0

ENSG00000250198	1	4	0	0
ENSG00000250200	0	0	0	0
ENSG00000250202	0	0	0	0
ENSG00000250204	0	0	0	0
ENSG00000250205	0	0	0	0
ENSG00000250206	0	0	0	0
ENSG00000250207	0	0	0	0
ENSG00000250208	0	0	0	0
ENSG00000250210	0	0	0	0
ENSG00000250213	0	0	0	0
ENSG00000250214	0	0	0	0
ENSG00000250215	0	0	0	0
ENSG00000250218	0	0	0	0
ENSG00000250219	0	0	0	0
ENSG00000250220	0	54	17	7
ENSG00000250221	0	0	0	0
ENSG00000250222	1	0	1	1
ENSG00000250223	0	0	9	6
ENSG00000250227	0	0	0	0
ENSG00000250229	0	0	0	0
ENSG00000250230	0	0	0	0
ENSG00000250231	0	0	0	0
ENSG00000250232	0	0	0	0
ENSG00000250234	0	0	0	0
ENSG00000250237	0	0	0	1
ENSG00000250238	0	0	0	0
ENSG00000250240	0	0	1	1
ENSG00000250241	0	0	0	0
ENSG00000250242	0	0	0	0
ENSG00000250243	0	0	0	0
ENSG00000250244	0	0	0	0
ENSG00000250247	0	0	0	0
ENSG00000250249	0	0	0	0
ENSG00000250250	0	0	0	0
ENSG00000250251	0	3	0	2
ENSG00000250252	0	0	0	0
ENSG00000250253	0	0	0	0
ENSG00000250254	0	0	0	0
ENSG00000250256	0	0	0	0
ENSG00000250257	0	0	0	0
ENSG00000250258	0	0	0	0
ENSG00000250259	0	0	0	0
ENSG00000250260	0	0	0	0
ENSG00000250261	5	16	1	0
ENSG00000250263	0	0	0	0
ENSG00000250264	0	0	0	0

ENSG00000250266	0	0	0	0
ENSG00000250267	0	0	0	0
ENSG00000250268	0	0	0	0
ENSG00000250269	0	0	0	0
ENSG00000250271	0	0	1	0
ENSG00000250272	0	0	13	1
ENSG00000250273	0	0	0	0
ENSG00000250274	0	0	0	0
ENSG00000250277	0	0	0	0
ENSG00000250280	0	0	0	0
ENSG00000250282	0	0	0	0
ENSG00000250284	0	0	0	0
ENSG00000250286	0	0	0	0
ENSG00000250289	0	0	0	0
ENSG00000250290	1	0	1	3
ENSG00000250292	0	0	0	0
ENSG00000250293	0	0	0	0
ENSG00000250294	0	0	0	0
ENSG00000250295	0	0	0	0
ENSG00000250296	0	0	0	0
ENSG00000250298	0	0	0	0
ENSG00000250299	0	0	0	0
ENSG00000250300	0	0	0	0
ENSG00000250302	0	0	0	0
ENSG00000250303	1	0	3	2
ENSG00000250304	0	0	0	0
ENSG00000250305	1	0	0	2
ENSG00000250306	0	0	0	0
ENSG00000250307	0	0	0	0
ENSG00000250308	0	0	0	0
ENSG00000250309	0	0	0	0
ENSG00000250310	0	0	0	0
ENSG00000250312	12	298	419	351
ENSG00000250313	0	0	0	0
ENSG00000250315	0	0	0	0
ENSG00000250316	0	0	0	0
ENSG00000250317	21	0	57	35
ENSG00000250318	1	0	0	0
ENSG00000250319	0	0	0	0
ENSG00000250320	0	0	0	0
ENSG00000250321	1	0	0	0
ENSG00000250322	0	0	0	0
ENSG00000250324	0	0	0	0
ENSG00000250325	0	0	0	0
ENSG00000250326	0	0	0	0
ENSG00000250327	0	0	0	0

ENSG00000250328	0	0	0	0
ENSG00000250329	0	0	0	0
ENSG00000250330	0	0	0	2
ENSG00000250331	0	0	5	0
ENSG00000250332	0	0	0	0
ENSG00000250333	0	0	0	0
ENSG00000250334	4011	75043	82753	71201
ENSG00000250337	1	0	0	0
ENSG00000250338	0	0	0	0
ENSG00000250339	0	0	0	0
ENSG00000250340	0	0	0	0
ENSG00000250341	0	0	0	0
ENSG00000250342	0	0	0	0
ENSG00000250343	0	0	0	0
ENSG00000250344	0	0	0	0
ENSG00000250345	0	0	1	0
ENSG00000250346	0	0	0	0
ENSG00000250347	0	0	0	0
ENSG00000250348	88	460	269	387
ENSG00000250349	0	0	0	0
ENSG00000250350	0	0	0	0
ENSG00000250351	0	0	0	0
ENSG00000250354	0	0	0	0
ENSG00000250355	0	0	0	0
ENSG00000250356	0	0	0	0
ENSG00000250357	0	0	0	0
ENSG00000250358	0	0	0	0
ENSG00000250359	0	0	0	0
ENSG00000250360	0	0	0	0
ENSG00000250361	3	0	4	3
ENSG00000250362	0	0	0	0
ENSG00000250363	0	0	0	0
ENSG00000250364	0	0	0	0
ENSG00000250365	1	0	6	2
ENSG00000250366	1	0	0	0
ENSG00000250371	0	0	0	0
ENSG00000250372	0	0	0	0
ENSG00000250374	0	0	0	0
ENSG00000250375	0	0	0	0
ENSG00000250376	0	0	0	0
ENSG00000250377	0	0	0	0
ENSG00000250378	0	0	0	0
ENSG00000250379	0	0	0	0
ENSG00000250381	0	0	0	0
ENSG00000250383	0	0	0	0
ENSG00000250384	0	0	0	0

ENSG00000250385	0	0	0	0
ENSG00000250386	0	0	0	0
ENSG00000250387	1	0	0	3
ENSG00000250389	0	0	0	0
ENSG00000250390	0	0	1	20
ENSG00000250391	0	0	0	0
ENSG00000250392	0	0	0	0
ENSG00000250393	0	0	0	0
ENSG00000250397	0	0	0	0
ENSG00000250398	0	0	0	0
ENSG00000250400	0	0	0	6
ENSG00000250402	0	0	0	0
ENSG00000250403	0	0	0	0
ENSG00000250404	0	0	0	0
ENSG00000250405	0	0	0	0
ENSG00000250406	0	0	0	0
ENSG00000250407	0	0	42	0
ENSG00000250409	0	0	1	0
ENSG00000250410	0	0	0	0
ENSG00000250411	0	0	0	0
ENSG00000250412	0	0	0	0
ENSG00000250413	0	0	1	0
ENSG00000250415	0	0	0	0
ENSG00000250416	0	0	0	0
ENSG00000250417	0	0	0	0
ENSG00000250418	0	0	0	0
ENSG00000250420	0	0	0	0
ENSG00000250421	2	84	11	4
ENSG00000250422	0	0	0	0
ENSG00000250423	0	0	0	0
ENSG00000250424	0	0	0	0
ENSG00000250425	0	0	0	0
ENSG00000250426	0	0	0	0
ENSG00000250427	0	0	0	0
ENSG00000250428	0	0	0	0
ENSG00000250430	0	0	0	0
ENSG00000250431	0	0	0	0
ENSG00000250432	0	0	0	0
ENSG00000250433	0	0	0	0
ENSG00000250436	0	0	0	0
ENSG00000250437	0	0	0	0
ENSG00000250438	0	0	0	0
ENSG00000250439	0	0	0	0
ENSG00000250441	0	0	0	0
ENSG00000250442	0	0	0	0
ENSG00000250443	0	0	0	0

ENSG00000250444	0	0	0	0
ENSG00000250446	0	0	0	0
ENSG00000250447	0	0	2	2
ENSG00000250448	1	0	0	5
ENSG00000250451	0	0	0	0
ENSG00000250453	0	0	0	0
ENSG00000250455	0	0	0	0
ENSG00000250456	0	0	0	0
ENSG00000250458	0	0	0	0
ENSG00000250461	0	2	0	0
ENSG00000250462	0	0	0	0
ENSG00000250463	0	0	0	0
ENSG00000250464	0	0	0	0
ENSG00000250467	0	0	0	0
ENSG00000250470	0	0	0	0
ENSG00000250471	0	0	2	0
ENSG00000250472	0	0	2	0
ENSG00000250473	0	0	0	0
ENSG00000250474	2	0	2	0
ENSG00000250475	0	0	0	0
ENSG00000250476	0	0	0	0
ENSG00000250479	20	0	2	5
ENSG00000250480	0	0	0	0
ENSG00000250481	0	0	0	0
ENSG00000250482	0	0	0	0
ENSG00000250483	0	0	0	0
ENSG00000250484	0	0	0	0
ENSG00000250485	0	0	0	0
ENSG00000250486	0	0	1	0
ENSG00000250487	0	0	0	0
ENSG00000250488	0	0	0	0
ENSG00000250490	0	1	5	2
ENSG00000250492	0	0	0	3
ENSG00000250493	0	0	0	0
ENSG00000250494	0	0	0	0
ENSG00000250496	0	0	0	0
ENSG00000250497	0	0	0	0
ENSG00000250500	0	0	0	0
ENSG00000250501	0	1	77	21
ENSG00000250503	0	0	0	0
ENSG00000250504	0	0	0	0
ENSG00000250505	0	0	0	0
ENSG00000250506	0	0	1	2
ENSG00000250507	0	0	0	0
ENSG00000250508	24	64	150	63
ENSG00000250509	0	0	0	0

ENSG00000250510	0	0	0	0
ENSG00000250511	0	0	0	0
ENSG00000250513	0	0	0	0
ENSG00000250514	0	0	0	0
ENSG00000250515	0	0	0	0
ENSG00000250516	0	0	0	0
ENSG00000250517	0	0	0	0
ENSG00000250519	0	0	0	0
ENSG00000250522	0	0	0	0
ENSG00000250523	0	0	0	0
ENSG00000250524	0	0	0	0
ENSG00000250526	0	0	0	0
ENSG00000250529	0	0	0	0
ENSG00000250530	0	0	0	0
ENSG00000250532	0	0	0	0
ENSG00000250534	22	51	19	8
ENSG00000250535	121	15	4	3
ENSG00000250536	0	0	1	0
ENSG00000250537	0	0	0	0
ENSG00000250538	0	0	0	0
ENSG00000250539	0	0	0	0
ENSG00000250540	0	0	0	3
ENSG00000250541	0	0	0	0
ENSG00000250543	0	0	0	0
ENSG00000250544	0	0	0	0
ENSG00000250546	0	0	0	0
ENSG00000250547	0	0	0	0
ENSG00000250548	10	0	29	12
ENSG00000250550	1	0	17	66
ENSG00000250551	0	13	1	0
ENSG00000250555	0	0	0	0
ENSG00000250556	0	0	0	0
ENSG00000250558	0	0	0	0
ENSG00000250559	0	0	0	0
ENSG00000250560	0	0	0	0
ENSG00000250561	0	0	0	0
ENSG00000250562	0	0	0	0
ENSG00000250563	0	0	0	0
ENSG00000250564	0	0	0	0
ENSG00000250565	4	18	13	35
ENSG00000250566	0	0	0	0
ENSG00000250567	0	0	0	0
ENSG00000250568	0	0	1	0
ENSG00000250569	0	0	0	2
ENSG00000250571	0	0	0	0
ENSG00000250572	0	0	0	0

ENSG00000250573	0	0	0	0
ENSG00000250574	0	0	0	0
ENSG00000250575	0	0	0	0
ENSG00000250576	0	0	0	0
ENSG00000250577	0	0	0	0
ENSG00000250579	0	0	0	0
ENSG00000250580	0	0	0	0
ENSG00000250582	0	0	0	0
ENSG00000250583	0	0	0	0
ENSG00000250584	0	0	0	0
ENSG00000250585	1	0	0	0
ENSG00000250587	0	0	0	0
ENSG00000250588	0	0	2	0
ENSG00000250590	0	0	0	0
ENSG00000250591	0	0	0	0
ENSG00000250592	0	0	0	0
ENSG00000250594	0	0	0	0
ENSG00000250595	0	0	0	0
ENSG00000250596	0	0	0	0
ENSG00000250597	0	0	0	0
ENSG00000250599	0	0	0	0
ENSG00000250600	3	41	18	2
ENSG00000250602	0	0	0	0
ENSG00000250603	2	0	0	0
ENSG00000250604	0	0	0	0
ENSG00000250606	0	0	0	0
ENSG00000250608	0	0	0	0
ENSG00000250609	0	0	0	0
ENSG00000250610	0	0	0	0
ENSG00000250611	0	0	0	0
ENSG00000250612	0	0	0	0
ENSG00000250613	0	0	0	0
ENSG00000250614	0	0	0	0
ENSG00000250615	0	0	0	0
ENSG00000250616	1	0	0	0
ENSG00000250618	0	0	0	0
ENSG00000250619	0	0	0	0
ENSG00000250620	0	0	0	0
ENSG00000250622	0	0	0	0
ENSG00000250623	0	0	0	0
ENSG00000250624	0	0	0	0
ENSG00000250625	0	0	0	0
ENSG00000250626	0	0	0	0
ENSG00000250627	0	0	0	0
ENSG00000250629	0	0	0	0
ENSG00000250630	0	0	0	0

ENSG00000250632	0	0	0	0
ENSG00000250634	4	68	76	28
ENSG00000250635	0	0	0	0
ENSG00000250636	0	0	0	0
ENSG00000250637	0	0	0	0
ENSG00000250640	0	0	0	0
ENSG00000250641	1	0	3	4
ENSG00000250642	0	0	0	0
ENSG00000250643	0	19	19	11
ENSG00000250644	0	0	0	0
ENSG00000250645	0	0	0	0
ENSG00000250646	0	0	0	0
ENSG00000250650	0	0	0	0
ENSG00000250651	0	0	0	0
ENSG00000250654	0	0	1	0
ENSG00000250655	0	0	0	0
ENSG00000250656	0	0	0	0
ENSG00000250657	0	5	6	0
ENSG00000250658	0	22	5	0
ENSG00000250659	0	0	0	0
ENSG00000250661	14	13	12	11
ENSG00000250662	0	0	0	0
ENSG00000250665	0	0	0	0
ENSG00000250666	0	0	0	0
ENSG00000250667	0	0	0	0
ENSG00000250668	0	0	0	0
ENSG00000250669	0	0	2	0
ENSG00000250670	10	30	26	1
ENSG00000250672	0	0	0	0
ENSG00000250673	0	0	0	0
ENSG00000250674	0	0	0	0
ENSG00000250677	0	0	0	0
ENSG00000250678	0	0	0	0
ENSG00000250681	0	0	0	0
ENSG00000250682	0	0	1	1
ENSG00000250684	0	0	0	0
ENSG00000250685	0	0	0	0
ENSG00000250686	0	104	109	160
ENSG00000250687	0	0	0	2
ENSG00000250688	0	0	0	0
ENSG00000250692	0	0	0	0
ENSG00000250693	0	0	0	0
ENSG00000250694	0	0	0	0
ENSG00000250696	0	0	8	17
ENSG00000250697	0	0	0	0
ENSG00000250698	0	0	0	0

ENSG00000250699	0	0	0	0
ENSG00000250703	0	0	0	0
ENSG00000250704	0	0	0	0
ENSG00000250705	0	0	0	0
ENSG00000250706	0	0	0	1
ENSG00000250708	0	0	0	0
ENSG00000250709	0	0	0	0
ENSG00000250710	0	0	0	0
ENSG00000250711	0	0	0	0
ENSG00000250712	0	0	0	0
ENSG00000250714	0	0	0	0
ENSG00000250715	0	0	0	0
ENSG00000250716	0	0	0	0
ENSG00000250719	0	0	0	0
ENSG00000250721	0	0	0	0
ENSG00000250722	0	0	4	19
ENSG00000250723	0	0	0	0
ENSG00000250725	0	0	0	2
ENSG00000250726	0	0	0	0
ENSG00000250727	0	0	0	0
ENSG00000250728	0	0	0	0
ENSG00000250730	0	0	0	0
ENSG00000250731	0	0	0	0
ENSG00000250732	0	0	0	0
ENSG00000250733	0	0	0	0
ENSG00000250734	0	0	0	0
ENSG00000250735	0	0	0	0
ENSG00000250739	0	0	0	0
ENSG00000250740	0	0	0	0
ENSG00000250741	0	0	0	0
ENSG00000250742	0	0	0	0
ENSG00000250745	0	0	0	0
ENSG00000250746	0	0	0	0
ENSG00000250747	0	0	0	0
ENSG00000250748	0	0	0	0
ENSG00000250749	0	0	0	0
ENSG00000250750	0	0	0	0
ENSG00000250751	0	0	0	0
ENSG00000250752	0	0	0	0
ENSG00000250753	0	0	0	0
ENSG00000250754	0	0	0	0
ENSG00000250756	0	0	0	0
ENSG00000250760	0	0	0	0
ENSG00000250761	0	0	1	0
ENSG00000250762	0	0	0	0
ENSG00000250763	1	34	44	45

ENSG00000250764	0	0	0	2
ENSG00000250765	0	0	0	0
ENSG00000250767	0	0	0	0
ENSG00000250768	0	0	0	0
ENSG00000250769	0	0	0	0
ENSG00000250770	0	0	0	0
ENSG00000250771	17	174	124	229
ENSG00000250772	0	0	0	0
ENSG00000250775	0	0	0	0
ENSG00000250777	0	0	0	0
ENSG00000250778	0	0	0	0
ENSG00000250781	0	0	0	0
ENSG00000250782	0	0	0	0
ENSG00000250784	0	0	0	0
ENSG00000250786	0	0	0	0
ENSG00000250787	1	0	0	5
ENSG00000250788	0	0	0	0
ENSG00000250790	11	13	27	28
ENSG00000250791	0	0	0	0
ENSG00000250794	0	0	0	0
ENSG00000250796	0	0	0	0
ENSG00000250799	0	0	0	0
ENSG00000250801	0	0	0	0
ENSG00000250802	0	85	132	102
ENSG00000250803	0	0	0	0
ENSG00000250804	0	0	0	0
ENSG00000250806	0	0	0	0
ENSG00000250807	0	0	0	0
ENSG00000250808	0	0	0	0
ENSG00000250810	0	0	0	0
ENSG00000250812	0	0	0	0
ENSG00000250813	0	0	0	0
ENSG00000250814	0	0	0	0
ENSG00000250815	0	0	0	0
ENSG00000250816	0	0	0	0
ENSG00000250819	0	0	0	0
ENSG00000250820	0	0	0	0
ENSG00000250821	0	0	0	0
ENSG00000250822	0	0	0	0
ENSG00000250825	0	0	0	0
ENSG00000250826	0	0	0	0
ENSG00000250827	0	0	0	0
ENSG00000250828	0	0	0	0
ENSG00000250829	0	0	0	0
ENSG00000250830	0	0	0	0
ENSG00000250831	0	0	0	1

ENSG00000250833	0	0	0	0
ENSG00000250834	0	0	0	0
ENSG00000250835	0	0	0	0
ENSG00000250838	0	0	0	0
ENSG00000250839	0	0	0	0
ENSG00000250842	0	0	2	16
ENSG00000250844	0	0	0	0
ENSG00000250846	0	0	0	0
ENSG00000250847	0	0	0	0
ENSG00000250848	0	0	1	0
ENSG00000250850	0	0	0	0
ENSG00000250852	0	0	0	0
ENSG00000250853	0	0	0	0
ENSG00000250855	0	0	0	0
ENSG00000250857	0	0	0	0
ENSG00000250858	0	0	0	0
ENSG00000250859	0	0	0	0
ENSG00000250860	0	0	0	0
ENSG00000250862	1	0	1	0
ENSG00000250863	0	0	0	0
ENSG00000250865	0	0	0	0
ENSG00000250866	0	0	0	0
ENSG00000250867	0	0	0	0
ENSG00000250868	0	0	0	0
ENSG00000250869	0	0	0	0
ENSG00000250874	0	0	0	0
ENSG00000250877	0	0	0	0
ENSG00000250878	0	0	1	13
ENSG00000250882	0	0	0	0
ENSG00000250884	0	0	0	0
ENSG00000250885	0	0	0	0
ENSG00000250886	0	0	0	0
ENSG00000250887	0	0	0	0
ENSG00000250888	0	0	0	0
ENSG00000250889	0	0	0	6
ENSG00000250890	0	0	0	0
ENSG00000250891	0	0	0	3
ENSG00000250892	0	0	0	2
ENSG00000250893	0	0	0	0
ENSG00000250894	0	0	0	0
ENSG00000250895	0	0	0	0
ENSG00000250896	0	0	1	0
ENSG00000250897	0	0	0	0
ENSG00000250899	0	0	0	0
ENSG00000250900	2	0	0	0
ENSG00000250902	0	0	10	11

ENSG00000250903	17	236	179	275
ENSG00000250905	0	0	0	0
ENSG00000250906	0	0	0	0
ENSG00000250908	0	0	0	0
ENSG00000250909	0	0	4	9
ENSG00000250910	0	0	1	0
ENSG00000250913	0	0	0	0
ENSG00000250914	0	0	0	0
ENSG00000250915	0	0	0	0
ENSG00000250917	0	0	0	0
ENSG00000250918	0	0	0	0
ENSG00000250919	0	0	0	2
ENSG00000250920	0	0	0	0
ENSG00000250921	0	0	0	0
ENSG00000250922	0	0	0	0
ENSG00000250923	0	0	0	0
ENSG00000250927	0	0	0	0
ENSG00000250928	0	0	0	0
ENSG00000250929	0	0	0	0
ENSG00000250930	0	0	0	0
ENSG00000250933	0	0	0	0
ENSG00000250934	0	0	0	0
ENSG00000250938	0	0	1	0
ENSG00000250939	0	0	0	0
ENSG00000250940	0	0	0	0
ENSG00000250942	0	0	0	2
ENSG00000250945	0	0	0	0
ENSG00000250946	0	0	0	0
ENSG00000250947	0	0	0	0
ENSG00000250948	0	0	0	0
ENSG00000250949	0	0	0	0
ENSG00000250950	0	0	0	0
ENSG00000250951	0	0	0	0
ENSG00000250954	0	0	0	0
ENSG00000250955	0	0	0	0
ENSG00000250956	0	0	0	0
ENSG00000250957	0	0	0	0
ENSG00000250958	0	0	0	0
ENSG00000250959	0	0	1	0
ENSG00000250961	13	88	167	601
ENSG00000250962	0	0	0	0
ENSG00000250963	0	0	0	0
ENSG00000250966	31	1	1	1
ENSG00000250968	0	0	0	0
ENSG00000250969	0	0	0	0
ENSG00000250970	0	0	0	1

ENSG00000250971	0	0	0	0
ENSG00000250972	0	0	0	0
ENSG00000250973	1	0	0	0
ENSG00000250974	0	0	0	0
ENSG00000250976	0	0	0	0
ENSG00000250977	0	0	0	0
ENSG00000250978	0	0	0	0
ENSG00000250979	0	0	0	0
ENSG00000250980	0	0	0	0
ENSG00000250981	0	0	0	0
ENSG00000250982	0	0	0	0
ENSG00000250983	0	0	0	0
ENSG00000250984	0	0	0	0
ENSG00000250986	0	0	0	0
ENSG00000250988	3	7	1	0
ENSG00000250989	0	0	0	0
ENSG00000250990	0	0	2	1
ENSG00000250991	0	0	0	0
ENSG00000250992	0	0	0	0
ENSG00000250993	0	0	0	0
ENSG00000250994	0	0	0	0
ENSG00000250995	0	0	0	0
ENSG00000250997	0	0	0	0
ENSG00000250999	0	0	0	0
ENSG00000251000	2	0	0	0
ENSG00000251001	0	0	9	0
ENSG00000251002	0	0	1	0
ENSG00000251003	0	0	1	0
ENSG00000251005	0	0	0	0
ENSG00000251007	0	0	0	0
ENSG00000251008	0	0	0	0
ENSG00000251009	1	7	0	0
ENSG00000251010	0	0	0	0
ENSG00000251011	0	0	1	0
ENSG00000251012	0	0	0	0
ENSG00000251013	0	0	0	0
ENSG00000251014	0	0	0	0
ENSG00000251015	0	0	6	0
ENSG00000251017	0	0	0	0
ENSG00000251018	0	0	10	2
ENSG00000251019	0	0	0	0
ENSG00000251022	34	316	223	405
ENSG00000251023	1	0	0	4
ENSG00000251024	0	0	0	0
ENSG00000251025	0	0	0	0
ENSG00000251026	0	0	0	0

ENSG00000251027	0	0	0	0
ENSG00000251031	0	0	0	0
ENSG00000251032	0	0	0	0
ENSG00000251033	0	0	0	0
ENSG00000251034	2	0	1	0
ENSG00000251035	0	0	0	0
ENSG00000251037	0	0	0	0
ENSG00000251038	0	0	0	0
ENSG00000251039	0	0	0	0
ENSG00000251040	0	0	0	0
ENSG00000251041	0	0	0	0
ENSG00000251044	0	0	0	0
ENSG00000251045	0	0	0	0
ENSG00000251046	0	1	0	1
ENSG00000251048	0	0	0	0
ENSG00000251049	0	0	0	0
ENSG00000251050	0	0	0	0
ENSG00000251051	0	0	0	0
ENSG00000251054	0	0	0	0
ENSG00000251055	0	0	0	0
ENSG00000251056	0	0	2	1
ENSG00000251058	0	0	0	0
ENSG00000251059	0	0	0	0
ENSG00000251060	26317	8643	30367	37121
ENSG00000251061	0	0	0	0
ENSG00000251062	0	0	0	0
ENSG00000251063	0	0	0	0
ENSG00000251066	0	0	1	0
ENSG00000251070	0	0	0	2
ENSG00000251072	0	0	0	0
ENSG00000251073	0	0	0	0
ENSG00000251074	0	0	0	0
ENSG00000251075	0	0	0	0
ENSG00000251076	0	0	0	0
ENSG00000251078	0	0	0	0
ENSG00000251079	0	0	0	3
ENSG00000251080	0	0	0	0
ENSG00000251081	0	0	0	2
ENSG00000251085	0	0	0	0
ENSG00000251087	0	0	0	0
ENSG00000251088	0	0	0	0
ENSG00000251090	0	0	0	0
ENSG00000251093	0	0	0	0
ENSG00000251095	0	335	539	160
ENSG00000251099	0	0	0	0
ENSG00000251101	0	0	0	0

ENSG00000251102	0	0	0	0
ENSG00000251105	0	0	0	0
ENSG00000251106	0	0	0	0
ENSG00000251107	0	0	0	0
ENSG00000251108	0	0	0	0
ENSG00000251111	0	0	0	0
ENSG00000251112	0	0	0	0
ENSG00000251113	0	0	0	0
ENSG00000251117	0	0	0	0
ENSG00000251118	13	0	0	1
ENSG00000251122	0	0	0	0
ENSG00000251123	0	0	0	0
ENSG00000251125	0	0	0	0
ENSG00000251126	0	0	0	0
ENSG00000251127	0	0	0	0
ENSG00000251128	3	0	1	0
ENSG00000251129	0	117	10	244
ENSG00000251131	0	0	3	4
ENSG00000251132	0	0	0	0
ENSG00000251135	0	0	0	0
ENSG00000251136	11	531	268	775
ENSG00000251137	0	0	0	0
ENSG00000251138	0	22	25	21
ENSG00000251139	0	0	0	0
ENSG00000251141	1	17	10	9
ENSG00000251142	0	0	0	0
ENSG00000251143	0	0	2	0
ENSG00000251144	0	0	0	0
ENSG00000251147	0	0	0	0
ENSG00000251148	0	0	0	0
ENSG00000251149	0	0	0	1
ENSG00000251151	0	0	0	0
ENSG00000251152	0	0	0	0
ENSG00000251154	0	0	0	0
ENSG00000251155	0	0	6	8
ENSG00000251158	0	0	0	0
ENSG00000251159	0	0	0	0
ENSG00000251160	0	0	2	0
ENSG00000251161	0	0	0	0
ENSG00000251162	0	0	0	0
ENSG00000251163	0	0	0	0
ENSG00000251164	0	0	0	0
ENSG00000251165	0	0	0	0
ENSG00000251166	0	0	0	0
ENSG00000251168	0	0	0	0
ENSG00000251169	0	0	2	0

ENSG00000251170	0	0	0	0
ENSG00000251171	0	0	0	0
ENSG00000251172	0	0	0	0
ENSG00000251173	0	0	0	0
ENSG00000251174	0	0	0	0
ENSG00000251175	0	0	0	0
ENSG00000251176	0	0	0	0
ENSG00000251177	0	0	0	0
ENSG00000251178	0	0	0	0
ENSG00000251179	0	0	0	0
ENSG00000251180	0	0	0	0
ENSG00000251182	0	0	0	0
ENSG00000251183	0	0	0	0
ENSG00000251184	0	0	0	0
ENSG00000251185	0	0	0	0
ENSG00000251186	0	0	0	0
ENSG00000251187	0	0	0	0
ENSG00000251188	0	0	0	0
ENSG00000251189	0	0	0	0
ENSG00000251191	3	20	2	3
ENSG00000251192	1	28	25	51
ENSG00000251193	0	0	0	0
ENSG00000251194	0	0	0	0
ENSG00000251195	0	0	0	0
ENSG00000251196	14	0	1	4
ENSG00000251197	0	0	0	0
ENSG00000251199	0	0	0	0
ENSG00000251200	0	0	0	0
ENSG00000251201	0	0	0	0
ENSG00000251203	0	0	0	0
ENSG00000251204	0	0	0	0
ENSG00000251205	0	0	0	0
ENSG00000251206	0	0	0	0
ENSG00000251209	17	4	25	3
ENSG00000251210	0	0	0	0
ENSG00000251211	0	0	0	0
ENSG00000251212	0	0	0	0
ENSG00000251213	0	0	0	0
ENSG00000251214	0	0	0	0
ENSG00000251215	0	11	5	13
ENSG00000251216	0	0	0	0
ENSG00000251218	0	0	0	0
ENSG00000251219	0	0	6	1
ENSG00000251220	0	0	0	0
ENSG00000251221	0	0	0	0
ENSG00000251223	0	0	0	0

ENSG00000251224	0	0	2	1
ENSG00000251226	0	0	0	0
ENSG00000251228	0	0	0	0
ENSG00000251229	0	0	0	0
ENSG00000251230	2	0	0	0
ENSG00000251234	0	0	0	0
ENSG00000251235	0	0	2	0
ENSG00000251236	0	0	0	0
ENSG00000251237	0	0	0	0
ENSG00000251239	0	0	0	0
ENSG00000251243	0	0	0	0
ENSG00000251244	0	0	0	2
ENSG00000251246	0	0	0	0
ENSG00000251247	18	95	12	15
ENSG00000251248	0	0	0	0
ENSG00000251249	1	0	0	0
ENSG00000251250	0	0	0	0
ENSG00000251252	0	0	4	2
ENSG00000251253	0	0	0	0
ENSG00000251254	0	0	0	0
ENSG00000251256	0	0	0	0
ENSG00000251257	0	0	0	0
ENSG00000251258	0	0	0	0
ENSG00000251259	55	237	115	162
ENSG00000251260	0	0	0	0
ENSG00000251261	0	0	2	0
ENSG00000251264	0	0	0	3
ENSG00000251266	0	0	0	0
ENSG00000251267	0	0	0	0
ENSG00000251270	0	0	0	0
ENSG00000251271	0	0	0	0
ENSG00000251273	0	0	3	0
ENSG00000251274	0	0	0	0
ENSG00000251275	0	0	0	0
ENSG00000251276	0	0	0	0
ENSG00000251278	0	0	0	0
ENSG00000251279	0	55	35	10
ENSG00000251281	0	0	0	0
ENSG00000251283	0	0	0	0
ENSG00000251284	0	0	0	0
ENSG00000251285	0	0	0	0
ENSG00000251286	0	0	0	0
ENSG00000251287	2	5	14	16
ENSG00000251288	0	0	2	0
ENSG00000251289	0	0	0	0
ENSG00000251291	0	0	0	0

ENSG00000251292	3	0	16	6
ENSG00000251293	0	0	0	0
ENSG00000251294	0	0	0	0
ENSG00000251296	1	12	0	6
ENSG00000251297	0	0	0	0
ENSG00000251298	0	0	0	0
ENSG00000251299	0	0	0	0
ENSG00000251300	0	0	0	0
ENSG00000251301	973	1069	1143	1326
ENSG00000251303	0	0	0	0
ENSG00000251304	0	0	0	0
ENSG00000251306	0	0	0	0
ENSG00000251307	0	0	0	2
ENSG00000251308	0	0	0	0
ENSG00000251309	2	0	6	0
ENSG00000251310	0	0	0	0
ENSG00000251311	0	0	0	0
ENSG00000251312	0	0	0	0
ENSG00000251313	0	0	0	0
ENSG00000251314	0	17	4	12
ENSG00000251317	0	0	0	0
ENSG00000251320	0	0	0	0
ENSG00000251321	1	0	0	0
ENSG00000251322	0	0	4	5
ENSG00000251323	0	0	8	7
ENSG00000251324	0	0	0	0
ENSG00000251325	0	0	0	0
ENSG00000251326	0	0	0	0
ENSG00000251329	0	0	0	0
ENSG00000251330	0	0	0	0
ENSG00000251331	0	0	0	0
ENSG00000251332	0	0	0	0
ENSG00000251333	0	0	0	0
ENSG00000251334	0	0	0	0
ENSG00000251336	0	0	0	0
ENSG00000251338	0	0	0	0
ENSG00000251339	0	0	1	0
ENSG00000251340	0	0	0	0
ENSG00000251342	0	0	0	0
ENSG00000251345	0	0	0	0
ENSG00000251347	0	0	0	0
ENSG00000251348	0	0	2	0
ENSG00000251349	0	0	0	0
ENSG00000251350	0	0	0	0
ENSG00000251352	0	0	0	0
ENSG00000251353	0	0	0	0

ENSG00000251354	0	0	0	0
ENSG00000251356	0	10	0	0
ENSG00000251357	0	0	0	0
ENSG00000251359	0	0	0	0
ENSG00000251360	0	0	0	0
ENSG00000251361	0	0	0	0
ENSG00000251363	0	0	1	0
ENSG00000251364	0	0	1	5
ENSG00000251365	0	0	0	0
ENSG00000251366	0	0	0	0
ENSG00000251367	0	0	0	0
ENSG00000251368	0	0	0	0
ENSG00000251369	0	0	2	15
ENSG00000251370	0	0	1	0
ENSG00000251371	0	0	1	0
ENSG00000251372	0	0	0	0
ENSG00000251373	0	0	0	0
ENSG00000251374	0	0	0	0
ENSG00000251376	0	0	0	0
ENSG00000251377	0	0	6	9
ENSG00000251378	0	0	0	0
ENSG00000251379	1	18	24	28
ENSG00000251380	0	0	0	0
ENSG00000251381	0	0	0	0
ENSG00000251383	0	0	0	0
ENSG00000251385	0	0	0	0
ENSG00000251387	0	0	0	0
ENSG00000251388	0	0	0	0
ENSG00000251389	0	0	0	0
ENSG00000251390	149	163	75	57
ENSG00000251391	0	0	0	1
ENSG00000251393	0	0	0	0
ENSG00000251394	0	0	0	0
ENSG00000251395	0	0	0	0
ENSG00000251396	0	0	0	0
ENSG00000251398	0	0	0	0
ENSG00000251399	72	384	542	702
ENSG00000251400	0	0	0	0
ENSG00000251401	0	0	0	0
ENSG00000251402	0	0	0	0
ENSG00000251405	0	0	0	0
ENSG00000251407	0	0	0	0
ENSG00000251408	0	0	0	0
ENSG00000251409	0	0	0	0
ENSG00000251410	0	0	0	0
ENSG00000251411	0	0	0	0

ENSG00000251412	0	0	0	0
ENSG00000251413	0	0	0	0
ENSG00000251414	0	0	0	0
ENSG00000251416	0	0	0	0
ENSG00000251417	4	0	0	0
ENSG00000251418	0	0	0	0
ENSG00000251419	0	0	0	0
ENSG00000251421	0	0	0	0
ENSG00000251423	0	0	0	0
ENSG00000251424	0	0	0	0
ENSG00000251426	0	0	0	0
ENSG00000251427	0	0	0	0
ENSG00000251429	0	0	1	4
ENSG00000251430	0	0	0	0
ENSG00000251431	0	0	0	0
ENSG00000251432	1	79	120	192
ENSG00000251433	0	0	0	0
ENSG00000251434	0	0	0	0
ENSG00000251435	5	95	155	158
ENSG00000251436	0	0	0	0
ENSG00000251437	0	0	0	0
ENSG00000251438	0	0	0	0
ENSG00000251439	0	0	0	0
ENSG00000251440	0	0	0	0
ENSG00000251441	0	0	2	0
ENSG00000251442	3	59	27	14
ENSG00000251443	0	0	0	0
ENSG00000251445	1	0	1	0
ENSG00000251446	0	0	0	0
ENSG00000251447	0	0	0	0
ENSG00000251448	0	0	0	0
ENSG00000251449	0	0	0	0
ENSG00000251450	0	0	0	0
ENSG00000251451	0	18	11	7
ENSG00000251452	0	0	0	0
ENSG00000251453	0	41	0	0
ENSG00000251454	0	0	0	0
ENSG00000251455	0	0	0	0
ENSG00000251456	0	0	0	0
ENSG00000251458	0	0	0	0
ENSG00000251459	0	0	0	0
ENSG00000251460	0	0	0	0
ENSG00000251461	0	0	0	0
ENSG00000251463	0	0	0	0
ENSG00000251464	0	0	0	0
ENSG00000251467	0	0	0	0

ENSG00000251468	0	0	0	0
ENSG00000251470	0	0	0	0
ENSG00000251471	0	0	0	0
ENSG00000251473	0	0	0	0
ENSG00000251474	7	24	5	6
ENSG00000251476	0	0	0	0
ENSG00000251477	0	0	0	0
ENSG00000251478	0	0	0	0
ENSG00000251479	0	0	0	0
ENSG00000251482	0	0	0	0
ENSG00000251483	0	0	0	0
ENSG00000251484	0	0	0	0
ENSG00000251485	0	0	0	0
ENSG00000251487	0	0	0	0
ENSG00000251488	0	0	0	0
ENSG00000251489	0	0	0	0
ENSG00000251490	0	0	0	0
ENSG00000251491	0	0	0	0
ENSG00000251492	0	0	0	0
ENSG00000251493	0	0	0	0
ENSG00000251495	0	0	0	0
ENSG00000251497	0	0	0	0
ENSG00000251498	0	0	0	0
ENSG00000251500	0	0	0	0
ENSG00000251501	0	0	0	0
ENSG00000251503	0	0	0	0
ENSG00000251504	0	0	0	0
ENSG00000251506	0	0	0	0
ENSG00000251508	0	0	0	0
ENSG00000251510	0	0	0	0
ENSG00000251511	0	0	0	4
ENSG00000251513	1	17	11	9
ENSG00000251515	0	0	0	0
ENSG00000251516	0	0	0	0
ENSG00000251517	0	0	0	0
ENSG00000251518	0	0	0	0
ENSG00000251520	0	0	0	0
ENSG00000251521	0	0	0	0
ENSG00000251523	0	0	1	0
ENSG00000251525	0	0	0	0
ENSG00000251526	0	0	4	0
ENSG00000251527	0	0	0	0
ENSG00000251529	0	0	0	0
ENSG00000251532	1	0	3	0
ENSG00000251533	0	0	0	0
ENSG00000251535	0	0	1	2

ENSG00000251536	0	0	0	0
ENSG00000251537	0	0	0	0
ENSG00000251538	0	0	0	0
ENSG00000251539	0	0	0	0
ENSG00000251540	0	0	0	0
ENSG00000251542	0	0	0	0
ENSG00000251543	0	0	0	0
ENSG00000251544	0	0	0	0
ENSG00000251545	0	0	0	0
ENSG00000251546	0	0	0	0
ENSG00000251547	0	0	0	0
ENSG00000251548	0	0	0	0
ENSG00000251549	0	0	0	0
ENSG00000251550	0	0	0	0
ENSG00000251552	0	0	0	0
ENSG00000251553	0	0	0	0
ENSG00000251554	0	0	0	0
ENSG00000251555	0	0	0	7
ENSG00000251556	1	0	0	0
ENSG00000251557	0	0	0	0
ENSG00000251562	3153	15704	13350	14331
ENSG00000251563	0	0	0	0
ENSG00000251564	0	0	0	0
ENSG00000251566	0	0	0	0
ENSG00000251567	0	0	0	0
ENSG00000251568	0	0	0	0
ENSG00000251569	0	0	0	0
ENSG00000251571	0	0	0	0
ENSG00000251572	0	0	0	0
ENSG00000251573	0	0	0	0
ENSG00000251574	0	0	0	0
ENSG00000251575	0	0	0	0
ENSG00000251576	0	0	0	0
ENSG00000251577	0	0	0	0
ENSG00000251578	2	0	0	0
ENSG00000251579	0	0	0	0
ENSG00000251580	12	66	3	24
ENSG00000251581	0	0	0	0
ENSG00000251582	1	0	0	0
ENSG00000251583	0	0	0	0
ENSG00000251584	0	0	2	1
ENSG00000251585	0	0	0	0
ENSG00000251586	2	0	0	1
ENSG00000251587	0	0	0	0
ENSG00000251588	0	0	0	0
ENSG00000251590	0	0	0	0

ENSG00000251591	0	0	0	0
ENSG00000251593	0	0	1	0
ENSG00000251594	0	0	0	0
ENSG00000251595	0	0	0	0
ENSG00000251596	0	0	0	0
ENSG00000251597	0	0	0	0
ENSG00000251598	0	0	0	0
ENSG00000251599	0	0	0	0
ENSG00000251600	67	1045	1530	1639
ENSG00000251601	0	0	0	0
ENSG00000251602	4	0	0	0
ENSG00000251603	0	0	0	0
ENSG00000251604	0	0	0	0
ENSG00000251605	0	0	0	0
ENSG00000251606	1	63	11	32
ENSG00000251608	0	0	0	0
ENSG00000251609	0	0	0	0
ENSG00000251610	0	0	0	0
ENSG00000251611	0	0	1	0
ENSG00000251613	0	0	0	0
ENSG00000251614	0	0	0	0
ENSG00000251615	0	0	3	0
ENSG00000251616	0	0	0	0
ENSG00000251617	0	0	0	0
ENSG00000251618	0	0	0	0
ENSG00000251619	0	0	0	0
ENSG00000251620	0	0	0	0
ENSG00000251621	0	0	0	0
ENSG00000251623	0	0	0	0
ENSG00000251624	0	0	0	0
ENSG00000251627	0	0	0	0
ENSG00000251628	0	0	0	0
ENSG00000251629	0	0	0	0
ENSG00000251630	0	0	0	0
ENSG00000251632	0	0	0	0
ENSG00000251633	1	0	0	0
ENSG00000251634	0	0	2	1
ENSG00000251635	0	0	0	0
ENSG00000251636	1	114	387	494
ENSG00000251637	0	0	0	0
ENSG00000251638	0	0	0	0
ENSG00000251639	0	0	0	0
ENSG00000251642	0	0	0	0
ENSG00000251643	0	0	0	1
ENSG00000251644	0	0	0	0
ENSG00000251646	0	0	0	0

ENSG00000251647	0	0	0	0
ENSG00000251648	1	0	0	0
ENSG00000251649	0	0	0	0
ENSG00000251652	0	0	0	0
ENSG00000251654	0	0	0	0
ENSG00000251655	0	0	0	0
ENSG00000251656	0	0	0	0
ENSG00000251659	0	0	0	0
ENSG00000251660	0	0	0	0
ENSG00000251661	0	0	0	0
ENSG00000251662	0	0	0	0
ENSG00000251663	0	0	0	0
ENSG00000251664	0	0	0	0
ENSG00000251665	0	0	0	0
ENSG00000251666	0	0	0	0
ENSG00000251667	0	2	0	0
ENSG00000251668	0	0	0	0
ENSG00000251669	0	0	0	0
ENSG00000251670	0	0	0	0
ENSG00000251675	0	0	0	0
ENSG00000251676	0	0	0	0
ENSG00000251678	0	0	0	0
ENSG00000251679	0	0	1	0
ENSG00000251680	0	0	5	0
ENSG00000251682	0	0	0	0
ENSG00000251685	0	0	0	0
ENSG00000251686	0	0	0	0
ENSG00000251687	0	0	0	0
ENSG00000251688	0	0	1	0
ENSG00000251689	0	0	0	0
ENSG00000251691	0	0	0	0
ENSG00000251692	0	0	0	1
ENSG00000251694	0	0	0	0
ENSG00000251695	0	0	0	0
ENSG00000251696	0	0	0	0
ENSG00000251697	0	0	0	0
ENSG00000251698	0	0	0	0
ENSG00000251699	0	0	0	0
ENSG00000251700	0	0	0	0
ENSG00000251702	0	0	0	0
ENSG00000251703	0	0	0	0
ENSG00000251704	0	0	0	0
ENSG00000251705	0	0	0	0
ENSG00000251706	0	0	0	0
ENSG00000251707	0	0	0	0
ENSG00000251708	0	0	0	0

ENSG00000251709	0	0	0	0
ENSG00000251710	0	0	0	0
ENSG00000251711	0	0	0	0
ENSG00000251712	0	0	0	0
ENSG00000251713	0	0	0	0
ENSG00000251714	0	0	0	0
ENSG00000251715	0	0	0	0
ENSG00000251716	0	0	0	0
ENSG00000251717	0	0	0	0
ENSG00000251718	0	0	0	0
ENSG00000251719	0	0	0	0
ENSG00000251720	0	0	0	0
ENSG00000251721	0	0	1	0
ENSG00000251722	0	0	0	0
ENSG00000251723	0	0	0	0
ENSG00000251724	0	0	0	0
ENSG00000251725	0	0	0	0
ENSG00000251726	0	0	0	0
ENSG00000251727	0	0	0	0
ENSG00000251728	0	0	0	0
ENSG00000251729	0	0	0	0
ENSG00000251730	0	0	0	0
ENSG00000251731	0	0	0	0
ENSG00000251732	0	0	0	0
ENSG00000251733	0	0	0	0
ENSG00000251735	0	0	0	0
ENSG00000251736	0	0	0	0
ENSG00000251737	0	0	0	0
ENSG00000251738	0	0	0	0
ENSG00000251739	0	0	0	0
ENSG00000251740	0	0	0	0
ENSG00000251741	0	0	0	0
ENSG00000251742	0	0	0	0
ENSG00000251744	0	0	0	0
ENSG00000251745	0	0	0	0
ENSG00000251746	0	0	0	0
ENSG00000251747	0	0	0	0
ENSG00000251748	0	0	0	0
ENSG00000251749	0	0	0	0
ENSG00000251750	0	0	0	0
ENSG00000251751	0	0	0	0
ENSG00000251752	0	0	0	0
ENSG00000251753	0	0	0	0
ENSG00000251754	0	0	0	0
ENSG00000251755	0	0	0	0
ENSG00000251756	0	0	0	0

ENSG00000251757	0	0	0	0
ENSG00000251758	0	0	0	0
ENSG00000251759	0	0	0	0
ENSG00000251760	0	0	0	0
ENSG00000251761	0	0	0	0
ENSG00000251762	0	0	0	0
ENSG00000251763	0	0	0	0
ENSG00000251764	0	0	0	0
ENSG00000251766	0	0	0	0
ENSG00000251767	0	0	0	0
ENSG00000251768	0	0	0	0
ENSG00000251769	0	0	0	0
ENSG00000251770	0	0	0	0
ENSG00000251771	0	0	0	0
ENSG00000251772	0	0	0	0
ENSG00000251773	0	0	0	0
ENSG00000251774	0	0	1	2
ENSG00000251775	0	0	0	0
ENSG00000251776	0	0	0	0
ENSG00000251777	0	0	0	0
ENSG00000251778	0	0	0	0
ENSG00000251779	0	0	0	0
ENSG00000251780	0	0	0	0
ENSG00000251781	0	0	0	0
ENSG00000251783	0	0	0	0
ENSG00000251784	0	0	0	0
ENSG00000251785	0	0	0	0
ENSG00000251786	0	0	0	0
ENSG00000251787	0	0	0	0
ENSG00000251788	0	0	0	0
ENSG00000251789	0	0	0	0
ENSG00000251790	0	0	0	0
ENSG00000251791	0	0	0	0
ENSG00000251792	0	0	0	0
ENSG00000251793	0	0	0	0
ENSG00000251794	0	0	0	0
ENSG00000251795	0	0	0	0
ENSG00000251796	0	0	0	0
ENSG00000251797	0	0	0	0
ENSG00000251798	0	0	0	0
ENSG00000251799	0	0	0	0
ENSG00000251800	0	0	0	0
ENSG00000251801	0	0	0	0
ENSG00000251802	0	0	0	0
ENSG00000251803	0	0	0	0
ENSG00000251804	0	0	0	0

ENSG00000251805	0	0	0	0
ENSG00000251806	0	0	0	0
ENSG00000251807	0	0	0	0
ENSG00000251808	0	0	0	0
ENSG00000251809	0	0	0	0
ENSG00000251810	0	0	0	0
ENSG00000251811	0	0	0	0
ENSG00000251812	0	0	0	0
ENSG00000251813	0	0	0	0
ENSG00000251814	0	0	0	0
ENSG00000251815	0	0	0	0
ENSG00000251816	0	0	0	0
ENSG00000251817	0	0	0	0
ENSG00000251818	0	0	0	1
ENSG00000251819	0	10	0	0
ENSG00000251820	0	0	0	0
ENSG00000251821	0	0	0	2
ENSG00000251822	0	0	0	0
ENSG00000251823	0	0	0	0
ENSG00000251824	0	0	0	0
ENSG00000251825	0	0	0	0
ENSG00000251827	0	0	0	0
ENSG00000251828	0	0	0	0
ENSG00000251829	0	0	0	0
ENSG00000251830	0	0	0	0
ENSG00000251831	0	0	0	0
ENSG00000251833	0	0	0	0
ENSG00000251834	0	0	0	0
ENSG00000251835	0	0	0	0
ENSG00000251836	0	0	0	0
ENSG00000251837	0	0	0	0
ENSG00000251838	0	0	0	0
ENSG00000251839	0	0	0	0
ENSG00000251840	0	0	0	0
ENSG00000251841	0	0	0	0
ENSG00000251842	0	0	0	0
ENSG00000251843	0	0	0	0
ENSG00000251844	0	0	0	0
ENSG00000251845	0	0	0	0
ENSG00000251846	0	0	0	0
ENSG00000251847	0	0	0	0
ENSG00000251848	0	0	0	0
ENSG00000251849	0	0	0	0
ENSG00000251850	0	0	0	0
ENSG00000251851	0	0	0	0
ENSG00000251852	0	0	0	0

ENSG00000251854	0	0	0	0
ENSG00000251856	0	0	0	0
ENSG00000251857	0	0	0	0
ENSG00000251858	0	0	0	0
ENSG00000251859	0	0	0	0
ENSG00000251860	0	0	0	0
ENSG00000251861	0	0	0	0
ENSG00000251862	0	0	0	0
ENSG00000251863	0	0	0	0
ENSG00000251864	0	0	0	0
ENSG00000251865	0	0	0	0
ENSG00000251866	0	0	0	0
ENSG00000251867	0	0	0	0
ENSG00000251868	0	0	0	0
ENSG00000251869	0	0	0	0
ENSG00000251870	0	0	0	0
ENSG00000251871	0	0	0	0
ENSG00000251872	0	0	0	0
ENSG00000251873	0	0	0	0
ENSG00000251874	0	0	0	0
ENSG00000251875	0	0	0	0
ENSG00000251876	0	0	0	0
ENSG00000251877	0	0	0	0
ENSG00000251878	0	0	0	0
ENSG00000251879	0	0	0	0
ENSG00000251880	0	0	0	0
ENSG00000251881	0	0	0	0
ENSG00000251882	0	0	0	0
ENSG00000251883	0	0	0	0
ENSG00000251884	0	0	0	0
ENSG00000251885	0	0	0	0
ENSG00000251886	0	0	0	0
ENSG00000251887	0	0	0	0
ENSG00000251888	0	0	0	0
ENSG00000251889	0	0	0	0
ENSG00000251890	0	0	0	0
ENSG00000251891	0	0	0	0
ENSG00000251892	0	0	0	0
ENSG00000251893	0	0	0	0
ENSG00000251894	0	0	0	0
ENSG00000251895	0	0	0	0
ENSG00000251896	0	0	0	0
ENSG00000251897	0	0	0	0
ENSG00000251898	0	0	0	0
ENSG00000251899	0	0	0	0
ENSG00000251900	0	0	0	0

ENSG00000251901	0	0	0	0
ENSG00000251902	0	0	0	0
ENSG00000251904	0	0	0	0
ENSG00000251905	0	0	0	0
ENSG00000251906	0	0	0	0
ENSG00000251907	0	0	0	0
ENSG00000251908	0	0	0	0
ENSG00000251909	0	0	0	0
ENSG00000251911	0	0	0	0
ENSG00000251912	0	0	0	0
ENSG00000251913	0	0	0	0
ENSG00000251914	0	0	0	0
ENSG00000251915	0	0	0	0
ENSG00000251916	0	0	0	0
ENSG00000251917	0	0	0	0
ENSG00000251918	0	0	0	0
ENSG00000251919	0	0	0	0
ENSG00000251920	0	0	1	0
ENSG00000251922	0	0	0	0
ENSG00000251923	0	0	0	0
ENSG00000251924	0	0	0	0
ENSG00000251925	0	0	0	0
ENSG00000251926	0	0	0	0
ENSG00000251927	0	0	0	0
ENSG00000251928	0	0	0	0
ENSG00000251929	0	0	0	0
ENSG00000251930	0	0	0	0
ENSG00000251931	0	0	0	0
ENSG00000251932	0	0	0	0
ENSG00000251933	0	0	0	0
ENSG00000251934	0	0	0	0
ENSG00000251935	0	0	0	0
ENSG00000251936	0	0	0	0
ENSG00000251937	0	0	0	0
ENSG00000251938	0	0	0	0
ENSG00000251939	0	0	0	0
ENSG00000251940	0	0	0	0
ENSG00000251941	0	0	0	0
ENSG00000251942	0	0	0	0
ENSG00000251943	0	0	0	0
ENSG00000251944	0	0	0	0
ENSG00000251945	0	0	0	0
ENSG00000251946	0	0	0	0
ENSG00000251947	0	0	0	0
ENSG00000251949	0	0	0	0
ENSG00000251950	0	0	0	0

ENSG00000251951	0	0	0	0
ENSG00000251952	0	0	0	0
ENSG00000251953	0	0	0	0
ENSG00000251954	0	0	0	0
ENSG00000251955	0	0	0	0
ENSG00000251956	0	0	0	0
ENSG00000251957	0	0	0	0
ENSG00000251958	0	0	0	0
ENSG00000251959	0	0	0	0
ENSG00000251960	0	0	0	0
ENSG00000251961	0	0	0	0
ENSG00000251962	0	0	0	0
ENSG00000251963	0	0	0	0
ENSG00000251964	0	0	0	0
ENSG00000251965	0	0	0	0
ENSG00000251966	0	0	0	0
ENSG00000251967	0	0	0	0
ENSG00000251968	0	0	0	0
ENSG00000251969	0	0	0	0
ENSG00000251970	0	0	0	0
ENSG00000251971	0	0	0	0
ENSG00000251972	0	0	0	0
ENSG00000251973	0	0	0	0
ENSG00000251974	0	0	0	0
ENSG00000251975	0	0	0	0
ENSG00000251976	0	0	0	0
ENSG00000251977	0	0	0	0
ENSG00000251978	0	0	0	0
ENSG00000251979	0	0	0	0
ENSG00000251980	0	0	0	0
ENSG00000251981	0	0	0	0
ENSG00000251982	0	0	0	0
ENSG00000251983	0	0	0	0
ENSG00000251985	0	0	0	0
ENSG00000251986	0	0	0	0
ENSG00000251987	0	0	0	0
ENSG00000251988	0	0	0	0
ENSG00000251989	0	0	0	0
ENSG00000251990	0	0	0	0
ENSG00000251991	0	0	0	0
ENSG00000251992	0	0	0	0
ENSG00000251993	0	0	0	0
ENSG00000251994	0	0	0	0
ENSG00000251995	0	0	0	0
ENSG00000251996	0	0	0	0
ENSG00000251997	0	0	0	0

ENSG00000251998	0	0	0	0
ENSG00000251999	0	0	0	0
ENSG00000252000	0	0	0	0
ENSG00000252001	0	0	0	0
ENSG00000252002	0	0	0	0
ENSG00000252003	0	0	0	0
ENSG00000252004	0	0	0	0
ENSG00000252005	0	0	0	0
ENSG00000252006	0	0	0	0
ENSG00000252008	0	0	0	0
ENSG00000252009	0	0	0	0
ENSG00000252010	0	0	0	0
ENSG00000252011	0	0	0	0
ENSG00000252012	0	0	0	0
ENSG00000252013	0	0	0	0
ENSG00000252014	0	0	0	0
ENSG00000252015	0	0	0	0
ENSG00000252016	0	0	0	0
ENSG00000252017	0	0	0	0
ENSG00000252018	0	0	0	0
ENSG00000252019	0	0	0	0
ENSG00000252020	0	0	0	0
ENSG00000252021	0	0	0	0
ENSG00000252022	0	0	0	0
ENSG00000252023	0	0	0	0
ENSG00000252024	0	0	0	0
ENSG00000252025	0	0	0	0
ENSG00000252026	0	0	0	0
ENSG00000252027	0	0	0	0
ENSG00000252028	0	0	0	0
ENSG00000252029	0	0	0	0
ENSG00000252030	0	0	0	0
ENSG00000252031	0	0	0	0
ENSG00000252032	0	0	0	0
ENSG00000252033	0	0	0	0
ENSG00000252034	0	0	0	0
ENSG00000252035	0	0	0	0
ENSG00000252036	0	0	0	0
ENSG00000252037	0	0	0	0
ENSG00000252038	0	0	0	0
ENSG00000252039	0	0	0	0
ENSG00000252040	0	0	0	0
ENSG00000252041	0	0	0	0
ENSG00000252042	0	0	0	0
ENSG00000252043	0	0	0	0
ENSG00000252045	0	0	0	0

ENSG00000252046	0	0	0	0
ENSG00000252047	0	0	0	0
ENSG00000252048	0	0	0	0
ENSG00000252049	0	0	0	0
ENSG00000252050	0	0	0	0
ENSG00000252051	0	0	0	0
ENSG00000252053	0	0	0	0
ENSG00000252054	0	0	0	0
ENSG00000252055	0	0	0	0
ENSG00000252056	0	0	0	0
ENSG00000252057	0	0	0	0
ENSG00000252058	0	0	0	0
ENSG00000252059	0	0	0	0
ENSG00000252060	0	0	0	0
ENSG00000252061	0	0	1	0
ENSG00000252062	0	0	3	1
ENSG00000252063	0	0	0	0
ENSG00000252064	0	0	0	0
ENSG00000252065	0	0	0	0
ENSG00000252066	0	0	0	0
ENSG00000252067	0	0	0	0
ENSG00000252068	0	0	0	0
ENSG00000252069	0	0	0	0
ENSG00000252070	0	0	0	0
ENSG00000252071	0	0	0	0
ENSG00000252072	0	0	0	0
ENSG00000252073	0	0	0	0
ENSG00000252074	0	0	0	0
ENSG00000252075	0	0	0	0
ENSG00000252076	0	0	0	0
ENSG00000252077	0	0	0	0
ENSG00000252078	0	0	0	0
ENSG00000252079	0	0	0	0
ENSG00000252080	0	0	0	0
ENSG00000252081	0	0	0	0
ENSG00000252082	0	0	0	0
ENSG00000252083	0	0	0	0
ENSG00000252084	0	0	0	0
ENSG00000252085	0	0	0	0
ENSG00000252086	0	0	0	0
ENSG00000252087	0	0	0	0
ENSG00000252088	0	0	0	0
ENSG00000252089	0	0	0	0
ENSG00000252091	0	0	0	0
ENSG00000252092	0	0	0	0
ENSG00000252093	0	0	0	0

ENSG00000252094	0	0	0	0
ENSG00000252096	0	0	0	0
ENSG00000252097	0	0	0	0
ENSG00000252098	0	0	0	0
ENSG00000252099	0	0	0	0
ENSG00000252100	0	0	0	0
ENSG00000252101	0	0	0	0
ENSG00000252102	0	0	0	0
ENSG00000252103	0	0	0	0
ENSG00000252104	0	0	0	0
ENSG00000252105	0	0	0	0
ENSG00000252106	0	0	0	0
ENSG00000252107	0	0	0	0
ENSG00000252108	0	0	0	0
ENSG00000252109	0	0	0	0
ENSG00000252110	0	0	0	0
ENSG00000252111	0	0	0	0
ENSG00000252112	0	0	0	0
ENSG00000252113	0	0	0	0
ENSG00000252114	0	0	0	0
ENSG00000252115	0	0	0	0
ENSG00000252116	0	0	0	0
ENSG00000252117	0	0	0	0
ENSG00000252118	0	0	0	0
ENSG00000252119	0	0	0	0
ENSG00000252120	0	0	0	0
ENSG00000252121	0	0	0	0
ENSG00000252122	0	0	0	0
ENSG00000252123	0	0	0	0
ENSG00000252124	0	0	0	0
ENSG00000252125	0	0	0	0
ENSG00000252126	0	0	0	0
ENSG00000252127	0	0	0	0
ENSG00000252128	0	0	0	0
ENSG00000252129	0	0	0	0
ENSG00000252130	0	0	0	0
ENSG00000252131	0	0	0	0
ENSG00000252132	0	0	0	0
ENSG00000252133	0	0	0	0
ENSG00000252134	0	0	0	0
ENSG00000252135	0	0	0	0
ENSG00000252136	0	0	0	0
ENSG00000252137	0	0	0	0
ENSG00000252138	0	0	0	0
ENSG00000252139	0	0	0	0
ENSG00000252140	0	0	0	0

ENSG00000252141	0	0	0	0
ENSG00000252142	0	0	0	0
ENSG00000252143	0	0	0	0
ENSG00000252144	0	0	0	0
ENSG00000252145	0	0	0	0
ENSG00000252146	0	0	0	0
ENSG00000252147	0	0	0	0
ENSG00000252148	0	0	0	0
ENSG00000252149	0	0	0	0
ENSG00000252150	0	0	0	1
ENSG00000252151	0	0	0	0
ENSG00000252153	0	0	0	0
ENSG00000252154	0	0	0	0
ENSG00000252155	0	0	0	0
ENSG00000252156	0	0	0	0
ENSG00000252157	0	0	0	0
ENSG00000252158	0	0	0	0
ENSG00000252159	0	0	0	0
ENSG00000252161	0	0	0	0
ENSG00000252162	0	0	0	0
ENSG00000252163	0	0	0	0
ENSG00000252164	0	0	0	0
ENSG00000252166	0	0	0	0
ENSG00000252167	0	0	0	0
ENSG00000252169	0	0	0	0
ENSG00000252170	0	0	0	0
ENSG00000252171	0	0	0	0
ENSG00000252172	0	0	0	0
ENSG00000252173	0	0	0	0
ENSG00000252174	0	0	0	0
ENSG00000252175	0	0	0	0
ENSG00000252176	0	0	0	0
ENSG00000252177	0	0	0	0
ENSG00000252178	0	0	0	0
ENSG00000252179	1	0	0	0
ENSG00000252180	0	0	0	0
ENSG00000252181	0	0	0	0
ENSG00000252182	0	0	0	0
ENSG00000252183	0	0	0	0
ENSG00000252184	0	0	0	0
ENSG00000252185	0	0	0	0
ENSG00000252186	0	0	0	0
ENSG00000252187	0	0	0	0
ENSG00000252188	0	0	0	0
ENSG00000252189	0	0	0	0
ENSG00000252190	0	0	0	0

ENSG00000252191	0	0	0	0
ENSG00000252192	0	0	0	0
ENSG00000252193	0	0	0	0
ENSG00000252196	0	0	0	0
ENSG00000252198	0	0	0	0
ENSG00000252199	0	0	0	0
ENSG00000252200	0	0	0	0
ENSG00000252201	0	0	0	0
ENSG00000252202	0	0	0	0
ENSG00000252203	0	0	0	0
ENSG00000252204	0	0	0	0
ENSG00000252205	0	0	0	0
ENSG00000252206	0	0	0	0
ENSG00000252207	0	0	0	0
ENSG00000252209	0	0	0	0
ENSG00000252210	0	0	0	0
ENSG00000252211	1	0	0	0
ENSG00000252212	0	0	0	0
ENSG00000252213	0	0	0	0
ENSG00000252214	0	0	0	0
ENSG00000252215	0	0	0	0
ENSG00000252216	0	0	0	0
ENSG00000252217	0	0	0	0
ENSG00000252218	0	0	0	0
ENSG00000252219	0	0	0	0
ENSG00000252220	0	0	0	0
ENSG00000252222	0	0	0	0
ENSG00000252223	0	0	0	0
ENSG00000252224	0	0	0	0
ENSG00000252225	0	0	0	0
ENSG00000252226	0	0	0	0
ENSG00000252227	0	0	0	0
ENSG00000252228	0	0	0	0
ENSG00000252230	0	0	0	0
ENSG00000252231	0	0	0	0
ENSG00000252233	0	0	1	0
ENSG00000252234	0	0	0	0
ENSG00000252235	0	0	0	0
ENSG00000252236	0	0	0	0
ENSG00000252237	0	0	0	0
ENSG00000252238	0	0	0	0
ENSG00000252239	0	0	0	0
ENSG00000252240	0	0	0	0
ENSG00000252241	0	0	0	0
ENSG00000252242	0	0	0	0
ENSG00000252243	0	0	0	0

ENSG00000252244	0	0	0	0
ENSG00000252245	0	0	0	0
ENSG00000252246	0	0	0	0
ENSG00000252247	0	0	0	0
ENSG00000252249	0	0	0	0
ENSG00000252250	0	0	0	0
ENSG00000252251	0	0	0	0
ENSG00000252252	0	0	0	0
ENSG00000252254	0	0	0	0
ENSG00000252255	0	0	0	0
ENSG00000252256	0	0	0	0
ENSG00000252257	0	0	0	0
ENSG00000252258	0	0	0	0
ENSG00000252259	0	0	0	0
ENSG00000252260	0	0	0	0
ENSG00000252261	0	0	0	0
ENSG00000252262	0	0	0	0
ENSG00000252263	0	0	0	0
ENSG00000252264	0	0	0	0
ENSG00000252265	0	0	0	0
ENSG00000252266	0	0	0	0
ENSG00000252267	0	0	0	0
ENSG00000252268	0	0	0	0
ENSG00000252269	0	0	0	0
ENSG00000252270	0	0	0	0
ENSG00000252271	0	0	0	0
ENSG00000252272	0	0	0	0
ENSG00000252273	0	0	0	0
ENSG00000252274	0	0	0	0
ENSG00000252275	0	0	0	0
ENSG00000252277	0	0	0	0
ENSG00000252278	0	0	0	0
ENSG00000252279	0	0	0	0
ENSG00000252280	0	0	0	0
ENSG00000252281	0	0	0	0
ENSG00000252282	0	0	0	0
ENSG00000252283	0	0	0	0
ENSG00000252284	0	0	0	0
ENSG00000252285	0	0	0	0
ENSG00000252287	0	0	0	0
ENSG00000252288	0	0	0	0
ENSG00000252289	0	0	0	0
ENSG00000252290	0	0	0	0
ENSG00000252291	0	0	0	0
ENSG00000252292	0	0	0	0
ENSG00000252293	0	0	0	0

ENSG00000252294	0	0	0	0
ENSG00000252295	0	0	0	0
ENSG00000252296	0	0	0	0
ENSG00000252297	0	0	0	0
ENSG00000252298	0	0	0	0
ENSG00000252299	0	0	0	0
ENSG00000252300	0	0	0	0
ENSG00000252301	0	0	0	0
ENSG00000252302	0	0	0	0
ENSG00000252303	0	0	0	0
ENSG00000252305	0	0	0	0
ENSG00000252306	0	0	0	0
ENSG00000252307	0	0	0	0
ENSG00000252308	0	0	0	0
ENSG00000252309	0	0	0	0
ENSG00000252310	0	0	0	0
ENSG00000252311	0	0	0	0
ENSG00000252312	0	0	0	0
ENSG00000252313	0	0	0	0
ENSG00000252314	0	0	0	0
ENSG00000252315	0	0	0	0
ENSG00000252316	0	0	6	1
ENSG00000252317	0	0	0	0
ENSG00000252319	0	0	0	0
ENSG00000252320	0	0	0	0
ENSG00000252321	0	0	0	0
ENSG00000252322	0	0	0	0
ENSG00000252323	0	0	0	0
ENSG00000252324	0	0	0	0
ENSG00000252325	0	0	0	0
ENSG00000252326	0	0	0	0
ENSG00000252327	0	0	0	0
ENSG00000252328	0	0	0	0
ENSG00000252329	0	0	0	0
ENSG00000252330	0	0	0	0
ENSG00000252332	0	0	0	0
ENSG00000252333	0	0	0	0
ENSG00000252334	0	0	0	0
ENSG00000252335	0	0	0	1
ENSG00000252336	0	0	0	0
ENSG00000252337	0	0	0	0
ENSG00000252338	0	0	0	0
ENSG00000252339	0	0	0	0
ENSG00000252341	0	0	0	0
ENSG00000252342	0	0	0	0
ENSG00000252343	0	0	0	0

ENSG00000252346	0	0	0	0
ENSG00000252347	0	0	0	0
ENSG00000252348	0	0	0	0
ENSG00000252349	0	0	0	0
ENSG00000252350	0	0	0	0
ENSG00000252351	0	0	0	0
ENSG00000252352	0	0	0	0
ENSG00000252353	0	0	0	0
ENSG00000252354	0	0	0	0
ENSG00000252355	0	0	0	0
ENSG00000252356	0	0	0	0
ENSG00000252357	0	0	0	0
ENSG00000252358	0	0	0	0
ENSG00000252359	0	0	0	0
ENSG00000252360	0	0	0	0
ENSG00000252361	0	0	0	0
ENSG00000252362	0	0	0	0
ENSG00000252363	0	0	0	0
ENSG00000252364	0	0	0	0
ENSG00000252365	0	0	0	0
ENSG00000252366	0	0	0	0
ENSG00000252367	0	0	0	0
ENSG00000252368	0	0	0	0
ENSG00000252369	0	0	0	0
ENSG00000252370	0	0	0	0
ENSG00000252371	0	0	0	0
ENSG00000252372	0	0	0	0
ENSG00000252373	0	0	0	0
ENSG00000252374	0	0	0	0
ENSG00000252375	0	0	0	0
ENSG00000252376	0	0	0	0
ENSG00000252377	0	0	0	0
ENSG00000252378	0	0	0	0
ENSG00000252379	0	0	0	0
ENSG00000252380	0	0	0	0
ENSG00000252382	0	0	0	0
ENSG00000252383	0	0	0	0
ENSG00000252384	0	0	0	0
ENSG00000252385	0	0	0	0
ENSG00000252386	0	0	0	0
ENSG00000252387	0	0	0	0
ENSG00000252388	0	0	0	0
ENSG00000252389	0	0	0	0
ENSG00000252390	0	0	0	0
ENSG00000252391	0	0	0	0
ENSG00000252392	0	0	0	0

ENSG00000252393	0	0	0	0
ENSG00000252394	0	0	0	0
ENSG00000252395	0	0	0	0
ENSG00000252396	0	0	0	0
ENSG00000252397	0	0	0	0
ENSG00000252398	0	0	0	0
ENSG00000252399	0	0	0	0
ENSG00000252400	0	0	0	0
ENSG00000252401	0	0	0	0
ENSG00000252402	0	0	0	0
ENSG00000252403	0	0	0	0
ENSG00000252404	0	0	0	0
ENSG00000252405	0	0	0	0
ENSG00000252406	0	0	0	0
ENSG00000252407	0	0	0	0
ENSG00000252408	0	0	0	0
ENSG00000252409	0	0	0	0
ENSG00000252410	0	0	0	0
ENSG00000252411	0	0	0	0
ENSG00000252412	0	0	0	0
ENSG00000252413	0	0	0	0
ENSG00000252414	0	0	0	0
ENSG00000252415	0	0	0	0
ENSG00000252416	0	0	0	0
ENSG00000252417	0	0	0	0
ENSG00000252418	0	0	0	0
ENSG00000252419	0	0	0	0
ENSG00000252420	0	0	0	0
ENSG00000252421	0	0	0	0
ENSG00000252422	0	0	0	0
ENSG00000252423	0	0	0	0
ENSG00000252424	0	0	0	0
ENSG00000252425	0	0	0	0
ENSG00000252426	0	0	0	0
ENSG00000252427	0	0	0	0
ENSG00000252428	0	0	0	0
ENSG00000252429	0	0	0	0
ENSG00000252430	0	0	0	0
ENSG00000252431	0	0	0	0
ENSG00000252432	0	0	0	0
ENSG00000252433	0	0	0	0
ENSG00000252434	0	0	0	0
ENSG00000252435	0	0	0	7
ENSG00000252436	0	0	0	0
ENSG00000252437	0	0	0	0
ENSG00000252438	0	0	0	0

ENSG00000252439	0	0	0	0
ENSG00000252440	0	0	0	0
ENSG00000252441	0	0	0	0
ENSG00000252442	0	0	0	0
ENSG00000252443	0	0	0	0
ENSG00000252444	0	0	0	0
ENSG00000252445	0	0	0	0
ENSG00000252446	0	0	0	0
ENSG00000252447	0	0	0	0
ENSG00000252448	0	0	0	0
ENSG00000252449	0	0	0	0
ENSG00000252450	0	0	0	0
ENSG00000252451	0	0	0	0
ENSG00000252452	0	0	0	0
ENSG00000252453	0	0	0	0
ENSG00000252454	0	0	0	0
ENSG00000252455	0	0	0	0
ENSG00000252456	0	0	0	0
ENSG00000252457	0	0	0	0
ENSG00000252458	0	0	0	0
ENSG00000252459	0	0	0	0
ENSG00000252460	0	0	0	0
ENSG00000252461	0	0	0	0
ENSG00000252462	0	0	0	0
ENSG00000252463	0	0	0	0
ENSG00000252464	0	0	0	0
ENSG00000252465	0	0	0	0
ENSG00000252466	0	0	0	0
ENSG00000252467	0	0	0	0
ENSG00000252468	0	0	0	0
ENSG00000252469	0	0	0	0
ENSG00000252470	0	0	0	0
ENSG00000252471	0	0	0	0
ENSG00000252472	0	0	0	0
ENSG00000252473	0	0	1	0
ENSG00000252474	0	0	0	0
ENSG00000252475	0	0	0	0
ENSG00000252476	0	0	0	0
ENSG00000252477	0	0	0	0
ENSG00000252478	0	0	0	0
ENSG00000252479	0	0	0	0
ENSG00000252480	0	0	0	0
ENSG00000252481	0	0	4	2
ENSG00000252482	0	0	0	0
ENSG00000252483	0	0	0	0
ENSG00000252484	0	0	0	0

ENSG00000252485	0	0	0	0
ENSG00000252486	0	0	0	0
ENSG00000252487	0	0	0	0
ENSG00000252489	0	0	0	0
ENSG00000252490	0	0	0	0
ENSG00000252491	0	0	0	0
ENSG00000252494	0	0	0	0
ENSG00000252495	0	0	0	0
ENSG00000252496	0	0	0	0
ENSG00000252497	0	0	0	0
ENSG00000252498	0	0	0	0
ENSG00000252499	0	0	0	0
ENSG00000252500	0	0	0	0
ENSG00000252501	0	0	0	0
ENSG00000252502	1	0	0	0
ENSG00000252503	0	0	0	0
ENSG00000252504	0	0	0	0
ENSG00000252505	0	0	0	0
ENSG00000252506	0	0	0	0
ENSG00000252507	0	0	0	0
ENSG00000252508	0	0	0	0
ENSG00000252509	0	0	0	0
ENSG00000252510	0	0	0	0
ENSG00000252511	0	0	0	0
ENSG00000252512	0	0	0	0
ENSG00000252513	0	0	0	0
ENSG00000252514	0	0	0	0
ENSG00000252515	0	0	0	0
ENSG00000252516	0	0	0	0
ENSG00000252517	0	0	0	0
ENSG00000252518	0	0	0	0
ENSG00000252519	0	0	0	0
ENSG00000252521	0	0	0	0
ENSG00000252522	0	0	0	0
ENSG00000252523	0	0	0	0
ENSG00000252524	0	0	0	0
ENSG00000252525	0	0	0	0
ENSG00000252526	0	0	0	0
ENSG00000252527	0	0	0	0
ENSG00000252529	0	0	0	0
ENSG00000252530	0	0	0	0
ENSG00000252531	0	0	0	0
ENSG00000252532	0	0	0	0
ENSG00000252533	0	0	0	0
ENSG00000252534	0	0	0	0
ENSG00000252535	0	0	0	0

ENSG00000252536	1	0	0	0
ENSG00000252537	0	0	0	0
ENSG00000252538	0	0	0	0
ENSG00000252539	0	0	0	0
ENSG00000252540	0	0	0	0
ENSG00000252541	0	0	0	0
ENSG00000252542	0	0	0	0
ENSG00000252543	0	0	0	0
ENSG00000252544	0	0	0	0
ENSG00000252545	0	0	0	0
ENSG00000252546	0	0	0	0
ENSG00000252547	0	0	0	0
ENSG00000252548	0	0	0	0
ENSG00000252549	0	0	0	0
ENSG00000252550	0	0	0	0
ENSG00000252552	1	0	0	0
ENSG00000252553	0	0	0	0
ENSG00000252554	0	0	0	0
ENSG00000252555	0	0	0	0
ENSG00000252556	0	0	0	0
ENSG00000252557	0	0	0	0
ENSG00000252558	0	0	0	0
ENSG00000252559	0	0	0	0
ENSG00000252560	0	0	0	0
ENSG00000252561	0	0	0	0
ENSG00000252562	0	0	0	0
ENSG00000252563	0	0	0	0
ENSG00000252564	0	0	0	0
ENSG00000252565	0	0	0	0
ENSG00000252566	0	0	0	0
ENSG00000252568	0	0	0	0
ENSG00000252569	1	0	0	0
ENSG00000252570	0	0	0	0
ENSG00000252571	0	0	0	0
ENSG00000252572	0	0	0	0
ENSG00000252574	0	0	0	0
ENSG00000252575	0	0	0	0
ENSG00000252576	0	0	0	0
ENSG00000252577	0	0	0	0
ENSG00000252578	0	0	0	0
ENSG00000252579	0	0	0	0
ENSG00000252580	0	0	0	0
ENSG00000252581	0	0	0	0
ENSG00000252582	0	0	0	0
ENSG00000252583	0	0	0	0
ENSG00000252584	0	0	0	0

ENSG00000252585	0	0	0	0
ENSG00000252586	0	0	0	0
ENSG00000252587	0	0	0	0
ENSG00000252589	0	0	0	0
ENSG00000252590	0	0	0	0
ENSG00000252591	0	0	0	0
ENSG00000252592	0	0	0	0
ENSG00000252593	0	0	0	0
ENSG00000252594	0	0	0	0
ENSG00000252595	0	0	0	0
ENSG00000252596	0	0	0	0
ENSG00000252597	0	0	0	0
ENSG00000252598	0	0	0	0
ENSG00000252599	0	0	0	0
ENSG00000252601	0	0	0	0
ENSG00000252602	0	0	0	0
ENSG00000252603	0	0	0	0
ENSG00000252604	0	0	0	0
ENSG00000252605	0	0	0	0
ENSG00000252606	0	0	0	0
ENSG00000252607	0	0	0	0
ENSG00000252608	0	0	0	0
ENSG00000252609	0	0	0	0
ENSG00000252611	0	0	0	0
ENSG00000252612	0	0	0	0
ENSG00000252613	0	0	0	0
ENSG00000252614	0	0	0	0
ENSG00000252615	0	0	0	0
ENSG00000252616	0	0	0	0
ENSG00000252617	0	0	0	0
ENSG00000252618	0	0	0	0
ENSG00000252619	0	0	0	0
ENSG00000252620	0	0	0	0
ENSG00000252621	0	0	0	0
ENSG00000252622	0	0	0	0
ENSG00000252623	0	0	0	0
ENSG00000252624	0	0	0	0
ENSG00000252625	0	0	0	0
ENSG00000252626	0	0	0	0
ENSG00000252627	0	0	0	0
ENSG00000252628	0	0	0	0
ENSG00000252629	0	0	0	0
ENSG00000252633	0	0	0	0
ENSG00000252634	0	0	0	0
ENSG00000252635	0	0	0	0
ENSG00000252636	0	0	0	0

ENSG00000252637	0	0	0	0
ENSG00000252638	0	0	0	0
ENSG00000252639	0	0	0	0
ENSG00000252640	0	0	0	0
ENSG00000252641	0	0	0	0
ENSG00000252642	0	0	0	0
ENSG00000252643	0	0	0	0
ENSG00000252644	0	0	0	0
ENSG00000252645	0	0	0	0
ENSG00000252646	0	0	0	0
ENSG00000252647	0	0	0	0
ENSG00000252648	0	0	0	0
ENSG00000252649	0	0	0	0
ENSG00000252650	0	0	0	0
ENSG00000252651	0	0	0	0
ENSG00000252652	0	0	0	0
ENSG00000252653	0	0	0	0
ENSG00000252654	0	0	0	0
ENSG00000252655	0	0	0	0
ENSG00000252656	0	0	0	0
ENSG00000252657	0	0	0	0
ENSG00000252658	0	0	0	0
ENSG00000252659	0	0	0	0
ENSG00000252660	0	0	0	0
ENSG00000252661	0	0	0	0
ENSG00000252662	0	0	0	0
ENSG00000252663	0	0	0	0
ENSG00000252664	0	0	0	0
ENSG00000252665	0	0	0	0
ENSG00000252667	0	0	0	0
ENSG00000252668	0	0	0	0
ENSG00000252669	0	0	0	0
ENSG00000252670	0	0	0	0
ENSG00000252671	0	0	0	0
ENSG00000252672	0	0	0	0
ENSG00000252673	0	0	0	0
ENSG00000252674	0	0	0	0
ENSG00000252675	0	0	0	0
ENSG00000252676	0	0	0	0
ENSG00000252677	0	0	0	0
ENSG00000252678	0	0	0	0
ENSG00000252679	0	0	0	0
ENSG00000252680	0	0	0	0
ENSG00000252681	0	0	0	0
ENSG00000252682	0	0	0	0
ENSG00000252683	0	0	0	0

ENSG00000252684	0	0	0	0
ENSG00000252685	0	0	0	0
ENSG00000252686	0	0	0	0
ENSG00000252687	0	0	0	0
ENSG00000252688	0	0	0	0
ENSG00000252689	0	0	0	0
ENSG00000252690	0	0	0	0
ENSG00000252691	0	0	0	0
ENSG00000252692	0	0	0	0
ENSG00000252693	0	0	0	0
ENSG00000252694	0	0	0	0
ENSG00000252695	0	0	0	0
ENSG00000252696	0	0	0	0
ENSG00000252697	0	0	0	0
ENSG00000252698	0	0	0	0
ENSG00000252699	0	0	0	0
ENSG00000252700	0	0	0	0
ENSG00000252701	0	0	0	0
ENSG00000252702	0	0	0	0
ENSG00000252703	0	0	0	0
ENSG00000252704	0	0	0	0
ENSG00000252705	0	0	1	0
ENSG00000252706	0	0	0	0
ENSG00000252707	0	0	0	0
ENSG00000252709	0	0	0	0
ENSG00000252710	0	0	0	0
ENSG00000252711	0	0	0	0
ENSG00000252712	0	0	0	0
ENSG00000252713	0	0	0	0
ENSG00000252714	0	0	0	0
ENSG00000252715	0	0	0	0
ENSG00000252716	0	0	0	0
ENSG00000252717	0	0	0	0
ENSG00000252718	0	0	0	0
ENSG00000252719	0	0	0	0
ENSG00000252720	0	0	0	0
ENSG00000252721	0	0	0	0
ENSG00000252722	0	0	0	0
ENSG00000252723	0	0	0	0
ENSG00000252724	0	0	0	0
ENSG00000252725	0	0	0	0
ENSG00000252726	0	0	0	0
ENSG00000252727	0	0	0	0
ENSG00000252728	0	0	0	0
ENSG00000252729	0	0	0	0
ENSG00000252730	0	0	0	0

ENSG00000252731	0	0	0	0
ENSG00000252732	0	0	0	0
ENSG00000252733	0	0	0	0
ENSG00000252734	0	0	0	0
ENSG00000252735	0	0	0	0
ENSG00000252736	0	0	0	0
ENSG00000252738	0	0	0	0
ENSG00000252739	0	0	0	0
ENSG00000252740	0	0	0	0
ENSG00000252742	0	0	0	0
ENSG00000252743	0	0	0	0
ENSG00000252744	0	0	0	0
ENSG00000252745	0	0	0	0
ENSG00000252746	0	0	0	0
ENSG00000252747	0	0	0	0
ENSG00000252748	0	0	0	0
ENSG00000252749	0	0	0	0
ENSG00000252750	0	0	0	0
ENSG00000252751	0	0	0	0
ENSG00000252752	0	0	0	0
ENSG00000252753	0	0	0	0
ENSG00000252754	0	0	0	0
ENSG00000252755	0	0	0	0
ENSG00000252756	0	0	0	0
ENSG00000252757	0	0	0	0
ENSG00000252758	0	0	0	0
ENSG00000252759	0	0	0	0
ENSG00000252760	0	0	0	0
ENSG00000252761	0	0	0	0
ENSG00000252762	0	0	0	0
ENSG00000252763	0	0	0	0
ENSG00000252764	0	0	0	0
ENSG00000252765	0	0	0	0
ENSG00000252766	0	0	0	0
ENSG00000252767	0	0	0	0
ENSG00000252768	0	0	0	0
ENSG00000252769	0	0	0	0
ENSG00000252770	0	0	0	0
ENSG00000252771	0	0	0	0
ENSG00000252772	0	0	0	0
ENSG00000252774	0	0	0	0
ENSG00000252776	0	0	0	0
ENSG00000252777	0	0	0	0
ENSG00000252778	0	0	0	0
ENSG00000252779	0	0	0	0
ENSG00000252780	0	0	0	0

ENSG00000252782	0	0	0	0
ENSG00000252783	0	0	0	0
ENSG00000252784	0	0	0	0
ENSG00000252785	0	0	0	0
ENSG00000252786	0	0	0	0
ENSG00000252787	0	0	0	0
ENSG00000252789	0	0	0	0
ENSG00000252790	0	0	0	0
ENSG00000252791	0	0	0	0
ENSG00000252792	0	0	0	0
ENSG00000252793	0	0	0	0
ENSG00000252794	0	0	0	0
ENSG00000252795	0	0	0	0
ENSG00000252796	0	0	0	0
ENSG00000252797	3	0	1	0
ENSG00000252798	0	0	0	0
ENSG00000252799	0	0	0	0
ENSG00000252800	0	0	0	0
ENSG00000252801	0	0	0	0
ENSG00000252802	0	0	0	0
ENSG00000252803	0	0	0	0
ENSG00000252804	0	0	0	0
ENSG00000252805	0	0	0	0
ENSG00000252806	0	0	0	0
ENSG00000252807	0	0	0	0
ENSG00000252808	0	0	0	0
ENSG00000252809	0	0	0	0
ENSG00000252810	0	0	0	0
ENSG00000252811	0	0	0	0
ENSG00000252812	1	0	0	0
ENSG00000252813	0	0	0	0
ENSG00000252814	0	0	0	0
ENSG00000252815	0	0	0	0
ENSG00000252816	0	0	0	0
ENSG00000252817	0	0	0	0
ENSG00000252818	0	0	0	0
ENSG00000252819	0	0	0	0
ENSG00000252820	0	0	0	0
ENSG00000252821	0	0	0	0
ENSG00000252822	0	0	0	0
ENSG00000252823	0	0	0	0
ENSG00000252824	0	0	0	0
ENSG00000252825	0	0	0	0
ENSG00000252826	0	0	0	0
ENSG00000252827	0	0	0	0
ENSG00000252828	0	0	0	0

ENSG00000252829	0	0	0	0
ENSG00000252830	0	0	0	0
ENSG00000252831	0	0	0	0
ENSG00000252832	0	0	0	0
ENSG00000252833	0	0	0	0
ENSG00000252834	0	0	0	0
ENSG00000252835	0	20	10	23
ENSG00000252836	0	0	0	0
ENSG00000252837	0	0	0	0
ENSG00000252838	0	0	0	0
ENSG00000252839	0	0	0	0
ENSG00000252840	0	0	0	0
ENSG00000252841	0	0	0	0
ENSG00000252842	0	0	0	0
ENSG00000252844	0	0	0	0
ENSG00000252845	0	0	0	0
ENSG00000252846	0	0	0	0
ENSG00000252847	0	0	0	0
ENSG00000252848	0	0	0	0
ENSG00000252849	0	0	0	0
ENSG00000252850	0	0	0	0
ENSG00000252852	0	0	0	0
ENSG00000252853	0	0	0	0
ENSG00000252854	0	0	0	0
ENSG00000252855	0	0	0	0
ENSG00000252856	0	0	0	0
ENSG00000252857	0	0	0	0
ENSG00000252858	0	0	0	0
ENSG00000252859	0	0	0	0
ENSG00000252860	0	0	0	0
ENSG00000252861	0	0	0	0
ENSG00000252863	0	0	0	0
ENSG00000252864	0	0	0	0
ENSG00000252865	0	0	0	0
ENSG00000252866	0	0	0	0
ENSG00000252867	0	0	0	0
ENSG00000252868	0	0	0	0
ENSG00000252869	0	0	0	0
ENSG00000252870	0	0	0	0
ENSG00000252872	0	0	0	0
ENSG00000252873	0	0	0	0
ENSG00000252874	0	0	0	0
ENSG00000252876	0	0	0	0
ENSG00000252877	0	0	0	0
ENSG00000252878	0	0	0	0
ENSG00000252879	0	0	0	0

ENSG00000252880	0	0	0	0
ENSG00000252881	0	0	0	0
ENSG00000252882	0	0	0	0
ENSG00000252883	0	0	0	0
ENSG00000252884	0	0	0	0
ENSG00000252886	0	0	0	0
ENSG00000252887	0	0	0	0
ENSG00000252888	0	0	0	0
ENSG00000252889	0	0	0	0
ENSG00000252890	0	0	0	0
ENSG00000252891	0	0	0	0
ENSG00000252892	0	0	0	0
ENSG00000252893	0	0	0	0
ENSG00000252894	0	0	0	0
ENSG00000252895	0	0	0	0
ENSG00000252896	0	0	0	0
ENSG00000252897	0	0	0	0
ENSG00000252898	0	0	0	0
ENSG00000252900	0	0	0	0
ENSG00000252901	0	0	0	0
ENSG00000252902	0	0	0	0
ENSG00000252903	0	0	0	0
ENSG00000252904	0	0	0	0
ENSG00000252905	0	0	0	0
ENSG00000252906	0	0	0	0
ENSG00000252908	0	0	0	0
ENSG00000252909	0	0	0	0
ENSG00000252910	0	0	0	0
ENSG00000252912	0	0	0	0
ENSG00000252913	0	0	0	0
ENSG00000252914	0	8	0	0
ENSG00000252915	0	0	0	0
ENSG00000252916	0	0	0	0
ENSG00000252917	0	0	0	0
ENSG00000252918	0	0	0	0
ENSG00000252919	1	0	0	0
ENSG00000252920	0	0	0	0
ENSG00000252921	0	0	0	0
ENSG00000252922	0	0	0	0
ENSG00000252923	0	0	0	0
ENSG00000252925	0	0	0	0
ENSG00000252927	0	0	0	0
ENSG00000252928	0	0	0	0
ENSG00000252929	0	0	0	0
ENSG00000252930	0	0	0	0
ENSG00000252931	0	0	0	0

ENSG00000252932	0	0	0	0
ENSG00000252933	0	0	0	0
ENSG00000252934	0	0	0	0
ENSG00000252935	0	0	0	0
ENSG00000252936	0	0	0	0
ENSG00000252937	0	0	0	0
ENSG00000252940	0	0	0	0
ENSG00000252941	0	0	0	0
ENSG00000252942	0	0	0	0
ENSG00000252943	0	0	0	0
ENSG00000252944	0	0	0	0
ENSG00000252945	0	0	0	0
ENSG00000252946	0	0	0	0
ENSG00000252947	0	0	0	0
ENSG00000252948	0	0	0	0
ENSG00000252949	0	0	0	0
ENSG00000252950	0	0	0	0
ENSG00000252951	0	0	0	0
ENSG00000252952	0	0	0	0
ENSG00000252953	0	0	0	0
ENSG00000252955	0	0	0	0
ENSG00000252956	0	0	0	0
ENSG00000252957	0	0	0	0
ENSG00000252958	0	0	0	0
ENSG00000252959	0	0	0	0
ENSG00000252960	0	0	0	0
ENSG00000252961	0	0	0	0
ENSG00000252962	0	0	0	0
ENSG00000252963	0	0	0	0
ENSG00000252964	0	0	0	0
ENSG00000252965	1	0	0	0
ENSG00000252966	0	0	0	0
ENSG00000252968	0	0	0	0
ENSG00000252969	0	0	0	0
ENSG00000252970	0	0	0	0
ENSG00000252971	0	0	0	0
ENSG00000252972	0	0	0	0
ENSG00000252973	0	0	0	0
ENSG00000252974	0	0	0	0
ENSG00000252975	0	0	0	0
ENSG00000252976	0	0	0	0
ENSG00000252977	0	0	0	0
ENSG00000252978	0	0	0	0
ENSG00000252979	0	0	0	0
ENSG00000252980	0	0	0	0
ENSG00000252981	0	0	0	0

ENSG00000252982	0	0	0	0
ENSG00000252983	0	0	0	0
ENSG00000252984	0	0	0	0
ENSG00000252985	0	0	3	0
ENSG00000252986	0	0	0	0
ENSG00000252987	0	0	0	0
ENSG00000252988	0	0	0	0
ENSG00000252989	0	0	0	0
ENSG00000252990	0	0	0	0
ENSG00000252991	0	0	0	0
ENSG00000252992	0	0	0	0
ENSG00000252993	0	0	0	0
ENSG00000252994	0	0	0	0
ENSG00000252995	0	0	0	0
ENSG00000252996	0	0	0	0
ENSG00000252997	0	0	0	0
ENSG00000252998	0	0	0	0
ENSG00000252999	0	0	0	0
ENSG00000253000	0	0	0	0
ENSG00000253001	0	0	0	0
ENSG00000253002	0	0	0	0
ENSG00000253003	0	0	0	0
ENSG00000253004	0	0	0	0
ENSG00000253005	0	0	0	0
ENSG00000253006	0	0	0	0
ENSG00000253007	0	0	0	0
ENSG00000253008	0	0	0	0
ENSG00000253009	0	0	0	0
ENSG00000253010	0	0	0	0
ENSG00000253011	0	0	0	0
ENSG00000253012	0	0	0	0
ENSG00000253013	0	0	0	0
ENSG00000253014	0	0	0	0
ENSG00000253015	0	0	0	0
ENSG00000253016	0	0	0	0
ENSG00000253017	0	0	0	0
ENSG00000253018	0	0	0	0
ENSG00000253019	0	0	0	0
ENSG00000253020	0	0	0	0
ENSG00000253021	0	0	0	0
ENSG00000253022	0	0	0	0
ENSG00000253023	0	0	0	0
ENSG00000253024	0	0	0	0
ENSG00000253025	0	0	0	0
ENSG00000253026	0	0	0	0
ENSG00000253027	0	0	0	0

ENSG00000253028	0	0	0	0
ENSG00000253029	0	0	0	0
ENSG00000253030	0	0	0	0
ENSG00000253031	0	0	0	0
ENSG00000253032	0	0	3	7
ENSG00000253033	0	0	0	0
ENSG00000253035	0	0	0	0
ENSG00000253036	0	0	0	0
ENSG00000253037	0	0	0	0
ENSG00000253038	0	0	0	0
ENSG00000253039	0	0	0	0
ENSG00000253040	0	0	0	0
ENSG00000253041	0	0	0	0
ENSG00000253042	0	0	0	0
ENSG00000253043	0	0	0	0
ENSG00000253044	0	0	0	0
ENSG00000253045	0	0	0	0
ENSG00000253046	0	0	0	0
ENSG00000253047	0	3	0	0
ENSG00000253048	0	0	0	0
ENSG00000253049	0	0	0	0
ENSG00000253050	0	0	0	0
ENSG00000253051	0	0	6	6
ENSG00000253052	0	0	0	0
ENSG00000253053	0	0	0	0
ENSG00000253054	0	0	0	0
ENSG00000253055	0	0	0	0
ENSG00000253056	0	0	0	0
ENSG00000253057	0	0	0	0
ENSG00000253058	12	0	0	0
ENSG00000253059	0	0	0	0
ENSG00000253060	0	0	0	0
ENSG00000253062	0	0	0	0
ENSG00000253063	0	0	0	0
ENSG00000253064	0	0	0	0
ENSG00000253065	0	0	0	0
ENSG00000253066	0	0	0	0
ENSG00000253067	0	0	0	0
ENSG00000253068	0	0	0	0
ENSG00000253069	0	0	0	0
ENSG00000253070	0	0	0	0
ENSG00000253071	0	0	0	0
ENSG00000253072	0	0	0	0
ENSG00000253073	0	0	0	0
ENSG00000253074	0	0	0	0
ENSG00000253075	0	0	0	0

ENSG00000253076	0	0	0	0
ENSG00000253077	0	0	0	0
ENSG00000253078	0	0	0	0
ENSG00000253079	0	0	0	0
ENSG00000253080	0	0	0	0
ENSG00000253081	0	0	0	0
ENSG00000253082	0	0	0	0
ENSG00000253083	0	0	0	0
ENSG00000253084	0	0	0	0
ENSG00000253085	0	0	0	0
ENSG00000253086	0	0	0	0
ENSG00000253087	0	0	0	0
ENSG00000253088	0	0	0	0
ENSG00000253089	0	0	0	0
ENSG00000253090	0	0	0	0
ENSG00000253091	0	0	0	0
ENSG00000253092	0	0	0	0
ENSG00000253093	0	0	0	0
ENSG00000253094	0	0	0	0
ENSG00000253095	0	0	0	0
ENSG00000253096	0	0	0	0
ENSG00000253097	0	0	0	0
ENSG00000253098	0	0	0	0
ENSG00000253099	0	0	0	0
ENSG00000253100	0	0	0	0
ENSG00000253102	0	0	0	0
ENSG00000253103	3	128	234	125
ENSG00000253104	0	0	0	0
ENSG00000253105	0	0	0	0
ENSG00000253106	1	0	10	3
ENSG00000253107	0	0	0	0
ENSG00000253108	0	0	0	0
ENSG00000253109	0	0	0	0
ENSG00000253110	0	0	0	0
ENSG00000253111	0	0	0	0
ENSG00000253112	0	0	0	0
ENSG00000253114	0	0	0	0
ENSG00000253115	0	0	0	0
ENSG00000253116	0	24	2	0
ENSG00000253117	0	0	0	0
ENSG00000253118	0	0	0	0
ENSG00000253119	0	0	0	0
ENSG00000253120	0	0	0	0
ENSG00000253121	0	0	0	0
ENSG00000253122	0	0	0	0
ENSG00000253123	0	0	0	0

ENSG00000253124	0	0	0	0
ENSG00000253125	0	0	0	0
ENSG00000253126	0	0	0	0
ENSG00000253127	0	0	0	0
ENSG00000253128	0	0	0	0
ENSG00000253130	0	0	0	0
ENSG00000253131	0	0	0	0
ENSG00000253132	0	0	0	0
ENSG00000253133	0	0	1	0
ENSG00000253134	0	0	0	0
ENSG00000253135	0	0	0	0
ENSG00000253137	0	0	0	0
ENSG00000253138	0	0	0	0
ENSG00000253139	0	0	0	0
ENSG00000253140	1	0	0	0
ENSG00000253141	0	0	0	0
ENSG00000253142	0	0	0	0
ENSG00000253143	0	0	0	0
ENSG00000253144	0	9	0	0
ENSG00000253146	0	0	0	0
ENSG00000253147	1	0	3	13
ENSG00000253148	0	0	0	0
ENSG00000253149	0	0	0	0
ENSG00000253151	0	0	0	0
ENSG00000253152	0	0	0	0
ENSG00000253153	0	0	0	0
ENSG00000253154	1	0	0	7
ENSG00000253155	0	0	0	0
ENSG00000253156	0	0	0	0
ENSG00000253158	0	0	0	0
ENSG00000253159	0	0	0	0
ENSG00000253160	0	0	0	0
ENSG00000253161	0	0	0	0
ENSG00000253162	0	0	0	0
ENSG00000253163	0	0	0	0
ENSG00000253164	0	0	0	0
ENSG00000253165	0	0	0	0
ENSG00000253166	0	0	0	0
ENSG00000253167	0	0	0	0
ENSG00000253168	0	0	0	0
ENSG00000253169	0	0	0	0
ENSG00000253170	0	0	0	0
ENSG00000253171	0	0	0	0
ENSG00000253172	0	0	0	0
ENSG00000253173	0	0	0	0
ENSG00000253174	0	0	0	0

ENSG00000253175	0	0	0	0
ENSG00000253176	0	0	0	0
ENSG00000253177	1	0	0	0
ENSG00000253178	0	0	0	0
ENSG00000253179	0	0	0	0
ENSG00000253180	0	0	0	0
ENSG00000253181	0	0	0	0
ENSG00000253182	0	0	0	0
ENSG00000253183	0	0	0	0
ENSG00000253184	0	0	0	0
ENSG00000253186	0	0	0	0
ENSG00000253187	0	0	0	0
ENSG00000253188	0	0	0	0
ENSG00000253189	0	0	0	0
ENSG00000253190	0	22	3	4
ENSG00000253191	0	0	0	0
ENSG00000253193	0	0	0	0
ENSG00000253194	0	0	0	1
ENSG00000253195	0	0	0	0
ENSG00000253196	0	0	0	0
ENSG00000253197	0	0	6	5
ENSG00000253198	0	0	0	0
ENSG00000253199	1	20	0	0
ENSG00000253200	0	0	0	0
ENSG00000253202	0	0	0	0
ENSG00000253203	9	0	9	5
ENSG00000253204	0	0	0	0
ENSG00000253205	0	0	0	0
ENSG00000253206	0	0	0	0
ENSG00000253207	0	0	0	0
ENSG00000253208	0	0	0	0
ENSG00000253209	0	0	0	0
ENSG00000253210	14	13	41	45
ENSG00000253213	0	0	0	0
ENSG00000253214	0	0	0	0
ENSG00000253215	0	0	0	0
ENSG00000253216	0	0	0	0
ENSG00000253217	0	0	0	5
ENSG00000253218	0	0	0	0
ENSG00000253219	0	0	0	0
ENSG00000253220	11	0	1	2
ENSG00000253222	0	0	0	0
ENSG00000253223	0	0	4	0
ENSG00000253224	0	0	0	0
ENSG00000253225	0	0	0	0
ENSG00000253226	0	0	0	0

ENSG00000253227	23	66	40	20
ENSG00000253228	0	0	0	0
ENSG00000253229	0	0	0	0
ENSG00000253230	0	0	0	0
ENSG00000253231	0	0	0	0
ENSG00000253232	0	0	0	1
ENSG00000253233	0	0	0	0
ENSG00000253234	0	0	0	0
ENSG00000253235	0	0	0	0
ENSG00000253236	0	0	0	0
ENSG00000253237	0	0	0	0
ENSG00000253238	0	0	0	0
ENSG00000253239	0	0	0	0
ENSG00000253240	0	0	0	0
ENSG00000253241	0	0	0	0
ENSG00000253242	0	0	0	0
ENSG00000253244	0	0	0	0
ENSG00000253245	0	0	0	0
ENSG00000253247	0	0	0	0
ENSG00000253248	0	0	0	0
ENSG00000253250	0	55	35	25
ENSG00000253251	3	44	19	23
ENSG00000253252	0	0	0	0
ENSG00000253256	0	0	0	0
ENSG00000253257	0	14	0	0
ENSG00000253258	0	0	0	0
ENSG00000253259	0	0	0	0
ENSG00000253260	0	0	0	0
ENSG00000253261	0	0	0	0
ENSG00000253262	0	0	0	0
ENSG00000253263	0	0	0	0
ENSG00000253264	0	0	0	0
ENSG00000253265	0	0	0	0
ENSG00000253266	0	0	0	0
ENSG00000253267	0	0	0	0
ENSG00000253269	0	0	0	0
ENSG00000253270	0	0	0	0
ENSG00000253271	0	0	0	0
ENSG00000253273	0	0	0	0
ENSG00000253274	0	0	0	0
ENSG00000253275	0	0	0	0
ENSG00000253276	73	850	1927	756
ENSG00000253278	0	0	0	0
ENSG00000253279	0	0	3	0
ENSG00000253280	0	0	0	0
ENSG00000253281	0	0	0	0

ENSG00000253282	0	0	0	0
ENSG00000253283	0	0	0	0
ENSG00000253284	0	0	5	19
ENSG00000253286	0	0	0	0
ENSG00000253287	0	0	0	0
ENSG00000253288	0	0	0	0
ENSG00000253290	0	0	0	0
ENSG00000253291	0	0	0	0
ENSG00000253292	0	0	0	0
ENSG00000253293	1	0	0	0
ENSG00000253294	0	0	0	0
ENSG00000253295	0	0	0	0
ENSG00000253296	0	0	0	0
ENSG00000253297	0	0	0	0
ENSG00000253298	0	0	0	0
ENSG00000253299	0	0	0	0
ENSG00000253300	0	0	0	0
ENSG00000253301	0	0	0	0
ENSG00000253302	1	0	0	0
ENSG00000253303	0	0	0	0
ENSG00000253304	0	15	24	0
ENSG00000253305	0	0	0	0
ENSG00000253307	1	54	17	2
ENSG00000253308	0	0	0	0
ENSG00000253309	5	17	16	15
ENSG00000253310	0	0	0	0
ENSG00000253311	0	0	0	0
ENSG00000253312	0	0	0	0
ENSG00000253313	0	0	0	0
ENSG00000253314	0	0	0	0
ENSG00000253315	1	0	0	0
ENSG00000253317	0	0	0	0
ENSG00000253318	2	0	0	0
ENSG00000253319	0	0	0	0
ENSG00000253320	2	72	70	42
ENSG00000253321	0	0	0	0
ENSG00000253322	0	0	0	0
ENSG00000253324	0	0	0	0
ENSG00000253325	0	0	0	0
ENSG00000253326	0	0	0	0
ENSG00000253327	1	0	3	0
ENSG00000253328	0	0	0	0
ENSG00000253329	0	0	0	0
ENSG00000253330	0	0	0	0
ENSG00000253331	0	0	0	0
ENSG00000253332	0	0	0	0

ENSG00000253333	0	0	0	0
ENSG00000253334	0	19	6	1
ENSG00000253335	0	0	0	0
ENSG00000253336	0	0	0	0
ENSG00000253338	0	0	0	0
ENSG00000253339	0	0	0	0
ENSG00000253340	0	0	0	0
ENSG00000253341	0	0	0	0
ENSG00000253342	0	0	0	0
ENSG00000253343	0	0	0	0
ENSG00000253344	0	0	0	0
ENSG00000253345	0	0	0	0
ENSG00000253346	0	0	0	0
ENSG00000253347	0	63	111	118
ENSG00000253348	0	0	0	0
ENSG00000253349	0	0	0	0
ENSG00000253350	4	0	4	20
ENSG00000253351	0	0	0	0
ENSG00000253352	378	2397	3367	2586
ENSG00000253354	0	0	0	0
ENSG00000253355	2	94	67	63
ENSG00000253356	0	42	30	36
ENSG00000253357	0	0	0	0
ENSG00000253358	0	18	134	44
ENSG00000253359	0	0	0	0
ENSG00000253361	0	0	0	0
ENSG00000253362	0	0	0	0
ENSG00000253363	0	0	0	0
ENSG00000253364	0	0	0	0
ENSG00000253365	0	0	0	0
ENSG00000253366	0	0	0	0
ENSG00000253367	0	0	0	0
ENSG00000253368	0	0	0	0
ENSG00000253369	0	0	0	0
ENSG00000253370	0	0	0	0
ENSG00000253371	0	0	0	0
ENSG00000253372	0	0	1	11
ENSG00000253373	0	0	0	0
ENSG00000253374	0	0	0	0
ENSG00000253376	0	0	0	1
ENSG00000253377	0	0	3	0
ENSG00000253379	1	0	0	0
ENSG00000253380	0	0	0	0
ENSG00000253381	0	0	0	0
ENSG00000253382	0	0	0	0
ENSG00000253383	0	0	0	0

ENSG00000253384	1	0	0	1
ENSG00000253385	0	0	0	0
ENSG00000253386	0	0	0	0
ENSG00000253387	0	0	0	0
ENSG00000253388	0	0	0	0
ENSG00000253389	0	0	0	0
ENSG00000253390	0	0	0	0
ENSG00000253391	0	0	0	0
ENSG00000253392	0	19	8	3
ENSG00000253393	0	0	0	0
ENSG00000253394	266	1383	1638	522
ENSG00000253395	17	43	32	65
ENSG00000253396	0	0	0	0
ENSG00000253397	0	0	0	0
ENSG00000253398	0	0	0	0
ENSG00000253399	0	0	0	0
ENSG00000253400	0	0	0	0
ENSG00000253401	0	0	0	0
ENSG00000253403	0	0	0	0
ENSG00000253404	0	0	0	0
ENSG00000253405	0	0	0	0
ENSG00000253406	0	21	36	23
ENSG00000253407	0	0	0	0
ENSG00000253408	0	0	0	0
ENSG00000253409	181	234	344	101
ENSG00000253410	0	0	0	0
ENSG00000253412	0	0	0	0
ENSG00000253413	0	0	0	0
ENSG00000253414	0	0	0	0
ENSG00000253415	0	0	0	0
ENSG00000253416	0	0	0	0
ENSG00000253417	0	0	0	0
ENSG00000253418	0	0	0	0
ENSG00000253420	0	0	0	0
ENSG00000253421	0	0	0	0
ENSG00000253422	0	0	0	0
ENSG00000253423	0	0	0	0
ENSG00000253424	1	53	33	25
ENSG00000253425	0	0	0	0
ENSG00000253426	0	0	1	0
ENSG00000253427	0	0	0	0
ENSG00000253428	0	0	0	0
ENSG00000253429	0	0	0	0
ENSG00000253430	0	0	0	0
ENSG00000253431	0	0	0	0
ENSG00000253432	0	0	0	0

ENSG00000253433	0	0	0	0
ENSG00000253434	0	0	0	0
ENSG00000253435	0	0	0	0
ENSG00000253437	0	0	0	0
ENSG00000253438	0	0	0	0
ENSG00000253439	0	0	0	0
ENSG00000253440	0	0	0	0
ENSG00000253441	0	0	0	0
ENSG00000253444	0	0	0	0
ENSG00000253445	0	4	0	0
ENSG00000253447	0	0	0	0
ENSG00000253448	0	0	0	0
ENSG00000253449	2	0	5	13
ENSG00000253451	0	0	0	0
ENSG00000253452	0	0	0	0
ENSG00000253454	0	0	0	0
ENSG00000253455	0	0	0	0
ENSG00000253456	0	0	0	0
ENSG00000253457	0	0	0	0
ENSG00000253458	0	0	0	0
ENSG00000253459	4	0	0	0
ENSG00000253460	0	0	0	0
ENSG00000253461	0	0	0	0
ENSG00000253462	0	0	0	0
ENSG00000253463	0	0	9	21
ENSG00000253464	0	0	0	0
ENSG00000253465	0	0	0	0
ENSG00000253467	0	0	0	0
ENSG00000253468	0	0	0	0
ENSG00000253469	0	0	0	0
ENSG00000253470	0	0	0	0
ENSG00000253471	0	0	0	0
ENSG00000253472	0	0	0	0
ENSG00000253474	3	10	9	10
ENSG00000253475	0	0	0	0
ENSG00000253476	0	0	0	0
ENSG00000253477	0	0	0	0
ENSG00000253479	0	0	0	0
ENSG00000253480	0	0	0	0
ENSG00000253481	0	0	0	0
ENSG00000253482	0	0	0	0
ENSG00000253483	0	0	0	0
ENSG00000253484	0	0	0	0
ENSG00000253485	0	0	0	1
ENSG00000253486	0	0	0	0
ENSG00000253487	0	0	0	0

ENSG00000253488	0	0	0	0
ENSG00000253489	0	0	0	0
ENSG00000253490	0	0	0	0
ENSG00000253491	0	0	0	0
ENSG00000253492	0	0	0	0
ENSG00000253493	0	0	0	0
ENSG00000253495	0	0	0	0
ENSG00000253496	0	0	0	0
ENSG00000253497	0	0	0	0
ENSG00000253499	0	0	0	0
ENSG00000253500	1	0	0	0
ENSG00000253501	0	0	0	0
ENSG00000253502	0	0	0	0
ENSG00000253503	0	0	0	0
ENSG00000253504	0	0	0	0
ENSG00000253505	0	0	0	0
ENSG00000253506	0	0	0	0
ENSG00000253507	0	0	0	0
ENSG00000253508	0	0	0	0
ENSG00000253509	0	0	0	0
ENSG00000253510	0	0	0	0
ENSG00000253512	0	0	0	0
ENSG00000253513	0	0	0	0
ENSG00000253515	0	0	0	0
ENSG00000253516	0	0	0	0
ENSG00000253517	0	0	0	0
ENSG00000253519	0	0	0	0
ENSG00000253520	59	270	187	116
ENSG00000253521	0	0	0	2
ENSG00000253522	46	233	175	173
ENSG00000253523	0	0	1	0
ENSG00000253524	0	0	0	0
ENSG00000253525	0	0	0	0
ENSG00000253526	2	0	0	6
ENSG00000253527	0	0	0	0
ENSG00000253528	0	0	0	0
ENSG00000253530	0	0	0	0
ENSG00000253531	0	0	0	0
ENSG00000253532	0	0	0	0
ENSG00000253534	0	0	0	0
ENSG00000253535	0	0	0	0
ENSG00000253536	0	0	0	0
ENSG00000253537	0	0	0	0
ENSG00000253538	0	0	0	0
ENSG00000253539	0	0	0	1
ENSG00000253540	0	0	0	2

ENSG00000253541	0	0	0	0
ENSG00000253542	0	0	0	0
ENSG00000253543	0	0	0	0
ENSG00000253544	0	0	1	0
ENSG00000253545	0	0	0	0
ENSG00000253546	0	0	0	0
ENSG00000253547	0	0	0	0
ENSG00000253548	0	0	0	0
ENSG00000253549	57	269	182	257
ENSG00000253550	0	0	0	0
ENSG00000253551	0	0	0	0
ENSG00000253552	0	0	0	0
ENSG00000253553	0	0	0	0
ENSG00000253554	0	0	0	0
ENSG00000253555	0	0	0	0
ENSG00000253556	0	0	0	0
ENSG00000253557	0	0	0	0
ENSG00000253558	0	0	0	1
ENSG00000253559	0	0	3	0
ENSG00000253560	0	0	0	0
ENSG00000253561	0	0	0	0
ENSG00000253562	0	0	0	0
ENSG00000253563	0	0	0	0
ENSG00000253564	0	0	0	0
ENSG00000253567	0	0	0	0
ENSG00000253568	0	0	0	0
ENSG00000253569	0	0	0	0
ENSG00000253570	0	0	0	0
ENSG00000253571	0	0	0	0
ENSG00000253572	0	0	0	0
ENSG00000253573	0	0	0	0
ENSG00000253574	0	0	0	0
ENSG00000253576	0	0	0	0
ENSG00000253577	0	0	0	0
ENSG00000253578	0	0	0	0
ENSG00000253579	0	0	0	0
ENSG00000253580	0	0	0	0
ENSG00000253581	0	0	0	0
ENSG00000253582	0	13	6	0
ENSG00000253583	0	0	0	0
ENSG00000253584	0	0	0	0
ENSG00000253585	0	0	0	0
ENSG00000253586	0	0	0	0
ENSG00000253587	0	0	0	0
ENSG00000253588	0	0	0	0
ENSG00000253590	0	0	0	0

ENSG00000253591	0	0	0	0
ENSG00000253592	0	0	0	0
ENSG00000253593	0	0	0	0
ENSG00000253595	0	0	0	0
ENSG00000253596	0	0	0	0
ENSG00000253597	0	0	0	0
ENSG00000253598	0	28	5	6
ENSG00000253600	0	0	0	0
ENSG00000253602	0	0	0	0
ENSG00000253603	0	0	0	0
ENSG00000253604	0	0	0	0
ENSG00000253605	0	0	0	0
ENSG00000253606	0	0	0	0
ENSG00000253607	0	0	13	10
ENSG00000253608	0	0	0	0
ENSG00000253610	0	0	0	0
ENSG00000253611	0	0	0	0
ENSG00000253612	0	0	0	0
ENSG00000253613	0	0	0	0
ENSG00000253614	0	0	0	0
ENSG00000253615	0	0	0	0
ENSG00000253616	0	0	0	0
ENSG00000253617	0	0	0	0
ENSG00000253618	0	0	1	2
ENSG00000253619	0	0	0	1
ENSG00000253620	0	0	0	0
ENSG00000253621	1	0	0	0
ENSG00000253622	0	0	0	0
ENSG00000253623	0	0	0	0
ENSG00000253625	0	0	0	0
ENSG00000253626	0	0	0	0
ENSG00000253627	0	0	0	0
ENSG00000253628	0	0	0	0
ENSG00000253629	0	0	0	0
ENSG00000253630	0	0	0	0
ENSG00000253631	0	0	0	0
ENSG00000253632	0	0	0	0
ENSG00000253633	5	0	7	11
ENSG00000253634	0	0	0	0
ENSG00000253635	0	0	0	0
ENSG00000253636	1	0	0	6
ENSG00000253637	0	0	0	0
ENSG00000253638	0	0	0	0
ENSG00000253639	0	0	0	0
ENSG00000253640	0	0	0	0
ENSG00000253641	0	0	4	0

ENSG00000253642	0	0	0	0
ENSG00000253643	0	0	0	0
ENSG00000253644	0	0	0	0
ENSG00000253645	0	0	9	0
ENSG00000253646	0	0	0	0
ENSG00000253647	0	0	0	2
ENSG00000253648	0	0	0	0
ENSG00000253649	0	0	0	0
ENSG00000253650	0	0	0	0
ENSG00000253651	0	0	0	0
ENSG00000253652	0	0	0	0
ENSG00000253653	0	0	0	0
ENSG00000253654	0	0	0	0
ENSG00000253655	0	0	0	0
ENSG00000253656	1	0	0	0
ENSG00000253657	0	0	0	0
ENSG00000253658	0	0	0	0
ENSG00000253659	0	0	0	0
ENSG00000253660	0	0	0	0
ENSG00000253661	0	0	0	0
ENSG00000253663	0	0	0	0
ENSG00000253664	0	0	0	0
ENSG00000253665	0	0	0	0
ENSG00000253666	0	0	0	0
ENSG00000253667	0	0	0	0
ENSG00000253668	0	0	0	0
ENSG00000253669	22	0	6	4
ENSG00000253670	0	0	0	0
ENSG00000253671	0	0	0	0
ENSG00000253672	0	0	0	0
ENSG00000253673	0	0	0	0
ENSG00000253674	0	0	0	0
ENSG00000253675	0	0	0	0
ENSG00000253676	1	101	66	139
ENSG00000253677	0	0	0	0
ENSG00000253678	0	0	0	0
ENSG00000253679	0	0	0	0
ENSG00000253680	0	0	0	0
ENSG00000253681	0	0	0	0
ENSG00000253682	0	0	0	0
ENSG00000253683	1	0	1	2
ENSG00000253684	0	0	0	0
ENSG00000253685	0	0	0	0
ENSG00000253686	1	0	0	2
ENSG00000253687	0	0	0	0
ENSG00000253688	0	0	0	0

ENSG00000253689	0	0	0	0
ENSG00000253690	0	0	0	0
ENSG00000253691	0	0	0	0
ENSG00000253692	0	0	0	0
ENSG00000253693	0	0	0	0
ENSG00000253695	0	0	0	0
ENSG00000253696	0	0	0	0
ENSG00000253697	0	0	0	0
ENSG00000253698	0	0	0	0
ENSG00000253699	0	0	0	0
ENSG00000253701	2	0	0	0
ENSG00000253702	0	0	0	0
ENSG00000253703	0	0	0	0
ENSG00000253704	0	0	0	0
ENSG00000253705	0	0	0	0
ENSG00000253706	0	0	1	0
ENSG00000253707	0	0	0	0
ENSG00000253708	0	0	0	0
ENSG00000253709	0	0	0	0
ENSG00000253710	3	156	191	200
ENSG00000253711	0	0	0	0
ENSG00000253712	1	0	0	0
ENSG00000253713	0	0	0	0
ENSG00000253714	0	30	0	0
ENSG00000253715	0	0	0	0
ENSG00000253716	14	135	53	48
ENSG00000253717	0	0	0	0
ENSG00000253718	0	0	0	0
ENSG00000253719	127	1001	946	1090
ENSG00000253720	0	0	0	0
ENSG00000253721	0	0	2	0
ENSG00000253722	0	0	0	0
ENSG00000253723	0	0	0	0
ENSG00000253725	0	0	0	0
ENSG00000253726	0	0	0	0
ENSG00000253728	0	0	0	0
ENSG00000253729	40	2292	1943	2916
ENSG00000253730	0	0	0	1
ENSG00000253731	0	0	0	0
ENSG00000253732	0	0	0	0
ENSG00000253733	0	0	0	0
ENSG00000253734	0	0	2	0
ENSG00000253735	0	0	0	0
ENSG00000253736	1	0	10	4
ENSG00000253737	25	217	91	78
ENSG00000253738	364	1086	1095	1580

ENSG00000253739	0	0	0	0
ENSG00000253740	0	0	0	0
ENSG00000253741	0	0	0	3
ENSG00000253742	0	0	0	0
ENSG00000253743	0	0	0	0
ENSG00000253744	0	0	1	0
ENSG00000253745	0	0	0	0
ENSG00000253746	0	0	0	0
ENSG00000253747	0	0	0	0
ENSG00000253748	0	0	0	0
ENSG00000253749	0	0	0	0
ENSG00000253750	0	0	0	0
ENSG00000253752	0	0	0	0
ENSG00000253753	0	0	0	0
ENSG00000253754	0	0	0	0
ENSG00000253755	1	0	0	0
ENSG00000253756	0	0	0	0
ENSG00000253759	0	0	0	0
ENSG00000253760	0	0	0	0
ENSG00000253762	0	0	0	0
ENSG00000253763	0	0	0	0
ENSG00000253764	0	0	0	0
ENSG00000253765	0	0	0	0
ENSG00000253766	0	0	0	0
ENSG00000253767	0	0	0	0
ENSG00000253768	0	0	0	0
ENSG00000253770	0	0	0	0
ENSG00000253771	6	0	11	2
ENSG00000253772	0	0	0	6
ENSG00000253773	17	27	36	48
ENSG00000253774	0	0	0	0
ENSG00000253775	0	0	0	0
ENSG00000253776	0	0	0	0
ENSG00000253777	0	0	0	0
ENSG00000253778	0	0	0	0
ENSG00000253779	0	0	0	0
ENSG00000253780	0	0	0	0
ENSG00000253781	0	0	0	0
ENSG00000253782	0	0	0	0
ENSG00000253783	0	0	0	0
ENSG00000253784	0	0	0	0
ENSG00000253785	0	0	0	0
ENSG00000253786	0	0	0	0
ENSG00000253787	0	0	0	0
ENSG00000253789	0	0	1	0
ENSG00000253790	0	0	0	0

ENSG00000253792	0	0	0	0
ENSG00000253793	0	0	0	0
ENSG00000253794	0	0	0	0
ENSG00000253795	0	0	0	0
ENSG00000253796	153	135	188	239
ENSG00000253797	43	73	62	37
ENSG00000253798	0	0	0	0
ENSG00000253799	0	0	0	2
ENSG00000253800	0	0	0	0
ENSG00000253801	0	0	0	0
ENSG00000253802	0	0	0	0
ENSG00000253803	0	0	0	0
ENSG00000253805	0	0	0	0
ENSG00000253806	0	0	0	0
ENSG00000253807	0	0	0	0
ENSG00000253808	0	0	0	0
ENSG00000253809	0	0	0	0
ENSG00000253810	0	0	0	0
ENSG00000253811	0	0	0	0
ENSG00000253813	0	0	0	0
ENSG00000253814	0	0	0	0
ENSG00000253816	0	0	0	7
ENSG00000253817	0	0	0	0
ENSG00000253818	0	0	0	0
ENSG00000253819	88	4225	4288	6905
ENSG00000253820	0	0	0	0
ENSG00000253821	0	0	0	0
ENSG00000253822	0	0	0	0
ENSG00000253823	0	0	0	0
ENSG00000253824	0	0	0	0
ENSG00000253825	0	0	0	1
ENSG00000253826	0	0	0	0
ENSG00000253828	0	0	0	0
ENSG00000253829	0	0	0	0
ENSG00000253830	0	0	0	0
ENSG00000253831	0	0	0	0
ENSG00000253832	0	0	0	0
ENSG00000253833	0	0	0	0
ENSG00000253834	0	0	0	0
ENSG00000253836	0	0	0	0
ENSG00000253837	0	0	0	0
ENSG00000253838	0	0	0	0
ENSG00000253839	0	0	1	0
ENSG00000253840	0	0	0	0
ENSG00000253841	0	0	0	0
ENSG00000253842	0	0	0	0

ENSG00000253843	0	0	0	0
ENSG00000253844	0	0	0	0
ENSG00000253845	0	0	0	0
ENSG00000253846	0	0	2	0
ENSG00000253848	15	29	7	9
ENSG00000253849	0	0	0	0
ENSG00000253851	0	0	1	0
ENSG00000253852	0	0	0	0
ENSG00000253853	1	0	12	0
ENSG00000253854	0	0	0	0
ENSG00000253855	0	0	0	0
ENSG00000253857	0	0	0	0
ENSG00000253858	0	0	0	0
ENSG00000253859	0	0	0	0
ENSG00000253860	0	0	0	0
ENSG00000253861	0	0	0	0
ENSG00000253862	0	0	0	0
ENSG00000253864	0	0	0	0
ENSG00000253865	1	13	2	2
ENSG00000253866	0	0	0	0
ENSG00000253868	0	0	0	0
ENSG00000253869	0	0	0	0
ENSG00000253870	0	0	0	0
ENSG00000253871	0	0	0	0
ENSG00000253872	0	0	0	1
ENSG00000253873	0	0	0	0
ENSG00000253874	0	0	0	0
ENSG00000253875	0	0	0	0
ENSG00000253877	0	0	0	0
ENSG00000253878	3	22	0	0
ENSG00000253879	0	0	0	0
ENSG00000253880	1	0	0	0
ENSG00000253881	0	0	0	0
ENSG00000253882	0	0	5	3
ENSG00000253883	0	0	0	0
ENSG00000253884	0	0	0	0
ENSG00000253885	0	0	0	0
ENSG00000253886	0	0	0	0
ENSG00000253887	0	0	0	0
ENSG00000253888	0	0	0	0
ENSG00000253889	0	0	0	0
ENSG00000253891	0	0	0	0
ENSG00000253892	0	0	0	0
ENSG00000253893	0	18	1	1
ENSG00000253894	0	0	0	0
ENSG00000253895	0	0	0	0

ENSG00000253896	0	0	0	0
ENSG00000253897	0	0	0	0
ENSG00000253898	1	35	29	80
ENSG00000253899	0	0	0	0
ENSG00000253900	0	0	0	0
ENSG00000253901	0	0	0	0
ENSG00000253903	0	0	0	0
ENSG00000253906	0	0	0	0
ENSG00000253907	0	0	0	0
ENSG00000253908	0	0	0	0
ENSG00000253910	0	0	0	0
ENSG00000253911	0	0	0	0
ENSG00000253912	0	0	0	0
ENSG00000253913	0	0	0	0
ENSG00000253915	0	0	0	0
ENSG00000253916	0	0	0	0
ENSG00000253917	0	0	0	0
ENSG00000253919	0	1	3	8
ENSG00000253920	0	0	0	0
ENSG00000253921	0	0	0	0
ENSG00000253923	0	0	0	0
ENSG00000253924	0	0	0	0
ENSG00000253925	0	0	0	0
ENSG00000253926	0	0	0	0
ENSG00000253927	0	0	0	0
ENSG00000253929	0	0	0	0
ENSG00000253930	0	0	0	0
ENSG00000253931	0	0	0	0
ENSG00000253932	0	0	0	0
ENSG00000253934	0	0	0	0
ENSG00000253935	0	0	0	0
ENSG00000253936	0	0	0	0
ENSG00000253937	0	0	0	0
ENSG00000253939	0	0	0	0
ENSG00000253940	0	0	0	0
ENSG00000253941	0	0	0	0
ENSG00000253942	0	0	0	0
ENSG00000253943	0	0	0	0
ENSG00000253944	0	0	0	0
ENSG00000253945	0	16	0	0
ENSG00000253946	0	0	0	0
ENSG00000253947	0	0	0	0
ENSG00000253948	8	0	8	8
ENSG00000253949	0	0	0	0
ENSG00000253950	3	0	0	0
ENSG00000253951	0	0	0	0

ENSG00000253952	0	0	0	0
ENSG00000253953	0	0	0	0
ENSG00000253954	2	0	1	0
ENSG00000253955	0	0	0	0
ENSG00000253956	0	0	0	0
ENSG00000253957	0	0	0	0
ENSG00000253958	0	0	0	0
ENSG00000253959	0	0	0	0
ENSG00000253960	0	0	0	0
ENSG00000253961	0	0	0	0
ENSG00000253963	0	0	0	0
ENSG00000253964	0	0	0	0
ENSG00000253965	0	0	0	0
ENSG00000253966	0	0	0	0
ENSG00000253967	0	0	0	0
ENSG00000253968	0	0	0	0
ENSG00000253970	0	0	4	0
ENSG00000253971	0	0	0	0
ENSG00000253972	0	0	0	0
ENSG00000253973	0	0	0	0
ENSG00000253974	0	0	0	0
ENSG00000253975	0	0	0	0
ENSG00000253976	0	0	0	0
ENSG00000253977	0	0	0	0
ENSG00000253978	0	0	0	0
ENSG00000253979	0	0	0	0
ENSG00000253980	0	0	0	0
ENSG00000253981	0	0	0	0
ENSG00000253982	732	945	658	473
ENSG00000253983	0	0	0	0
ENSG00000253985	0	0	0	0
ENSG00000253986	0	0	0	0
ENSG00000253988	0	0	0	0
ENSG00000253989	0	0	0	0
ENSG00000253991	0	0	0	0
ENSG00000253992	0	0	0	0
ENSG00000253993	0	0	0	0
ENSG00000253994	0	0	0	0
ENSG00000253995	0	0	0	0
ENSG00000253996	0	0	0	0
ENSG00000253997	0	0	0	0
ENSG00000253998	0	0	0	0
ENSG00000253999	0	0	0	0
ENSG00000254000	0	0	0	0
ENSG00000254001	0	0	0	0
ENSG00000254002	0	0	0	0

ENSG00000254003	0	0	0	1
ENSG00000254004	23	183	168	150
ENSG00000254006	1	0	1	0
ENSG00000254007	0	0	0	0
ENSG00000254008	0	0	0	0
ENSG00000254009	0	0	0	0
ENSG00000254010	0	0	0	0
ENSG00000254011	0	0	0	0
ENSG00000254012	0	0	0	0
ENSG00000254013	0	0	0	0
ENSG00000254014	0	0	0	0
ENSG00000254015	0	0	0	0
ENSG00000254016	0	0	0	0
ENSG00000254017	0	0	0	0
ENSG00000254018	0	0	0	0
ENSG00000254019	0	96	46	87
ENSG00000254020	0	0	0	0
ENSG00000254021	0	0	0	0
ENSG00000254023	0	0	0	0
ENSG00000254024	0	0	0	0
ENSG00000254025	0	0	0	0
ENSG00000254026	1	0	1	1
ENSG00000254027	0	0	0	0
ENSG00000254028	0	0	0	0
ENSG00000254029	0	0	0	0
ENSG00000254030	1	0	0	0
ENSG00000254031	0	0	0	0
ENSG00000254033	0	0	0	0
ENSG00000254034	0	0	0	0
ENSG00000254035	0	0	0	0
ENSG00000254036	0	0	0	0
ENSG00000254037	0	0	0	0
ENSG00000254038	0	0	0	0
ENSG00000254039	0	0	0	0
ENSG00000254040	0	0	0	0
ENSG00000254041	0	0	0	0
ENSG00000254042	0	0	0	0
ENSG00000254043	0	0	0	0
ENSG00000254044	0	0	0	0
ENSG00000254045	0	0	0	0
ENSG00000254046	0	0	0	0
ENSG00000254047	0	0	0	0
ENSG00000254048	0	0	0	0
ENSG00000254049	0	0	0	0
ENSG00000254050	0	0	0	0
ENSG00000254051	0	0	0	0

ENSG00000254052	0	0	0	0
ENSG00000254053	0	0	0	0
ENSG00000254054	0	0	0	0
ENSG00000254055	0	0	0	0
ENSG00000254056	0	0	0	0
ENSG00000254057	0	0	0	0
ENSG00000254060	0	0	0	0
ENSG00000254061	0	0	0	0
ENSG00000254063	0	0	0	0
ENSG00000254064	0	0	0	0
ENSG00000254065	0	0	0	0
ENSG00000254066	0	0	0	0
ENSG00000254067	0	0	0	0
ENSG00000254069	0	0	0	0
ENSG00000254070	0	0	0	0
ENSG00000254073	0	0	0	0
ENSG00000254075	0	0	0	0
ENSG00000254076	1	0	0	0
ENSG00000254077	0	0	0	0
ENSG00000254079	0	0	0	0
ENSG00000254080	0	0	0	0
ENSG00000254081	0	0	0	0
ENSG00000254082	0	0	0	0
ENSG00000254083	0	0	0	0
ENSG00000254084	0	0	0	0
ENSG00000254086	0	0	0	0
ENSG00000254087	1781	10438	12777	12592
ENSG00000254088	0	0	1	0
ENSG00000254089	0	0	0	0
ENSG00000254090	0	0	0	0
ENSG00000254091	0	0	0	0
ENSG00000254092	3	0	3	0
ENSG00000254093	6	0	3	6
ENSG00000254094	0	0	0	0
ENSG00000254095	0	0	0	0
ENSG00000254097	0	0	0	0
ENSG00000254098	0	0	0	0
ENSG00000254099	0	0	37	2
ENSG00000254100	0	0	0	0
ENSG00000254101	0	0	4	0
ENSG00000254102	0	0	0	0
ENSG00000254103	0	0	0	0
ENSG00000254104	0	0	0	0
ENSG00000254105	0	0	0	0
ENSG00000254106	0	0	0	0
ENSG00000254109	0	0	0	0

ENSG00000254111	0	0	0	0
ENSG00000254112	0	0	0	0
ENSG00000254113	0	0	0	0
ENSG00000254114	0	0	0	0
ENSG00000254115	0	0	0	0
ENSG00000254117	0	0	0	0
ENSG00000254118	0	0	0	0
ENSG00000254119	6	80	16	1
ENSG00000254120	0	0	0	0
ENSG00000254122	0	0	0	0
ENSG00000254123	0	0	0	0
ENSG00000254124	0	0	0	0
ENSG00000254126	0	0	0	0
ENSG00000254127	0	0	0	0
ENSG00000254129	0	0	2	0
ENSG00000254130	0	0	0	0
ENSG00000254131	0	0	0	0
ENSG00000254132	0	0	0	0
ENSG00000254133	0	0	0	0
ENSG00000254134	0	0	0	0
ENSG00000254135	0	0	0	0
ENSG00000254136	0	0	0	0
ENSG00000254138	40	450	113	424
ENSG00000254139	0	0	0	2
ENSG00000254140	0	0	0	0
ENSG00000254141	1	0	2	16
ENSG00000254142	0	0	0	0
ENSG00000254143	0	0	0	0
ENSG00000254144	0	0	0	0
ENSG00000254145	0	0	0	0
ENSG00000254146	0	0	0	0
ENSG00000254148	0	0	0	0
ENSG00000254150	0	0	0	0
ENSG00000254151	0	0	0	0
ENSG00000254152	0	0	0	2
ENSG00000254153	0	0	0	4
ENSG00000254154	0	25	4	8
ENSG00000254156	0	0	0	0
ENSG00000254157	0	0	0	0
ENSG00000254158	0	0	0	0
ENSG00000254160	0	0	0	0
ENSG00000254161	0	0	0	0
ENSG00000254162	0	0	0	0
ENSG00000254163	0	0	0	0
ENSG00000254164	0	0	0	0
ENSG00000254165	0	0	0	0

ENSG00000254166	0	0	0	0
ENSG00000254167	0	0	0	0
ENSG00000254170	0	0	0	0
ENSG00000254171	0	0	0	0
ENSG00000254172	0	0	0	0
ENSG00000254173	0	0	0	0
ENSG00000254174	0	0	0	0
ENSG00000254175	0	0	0	0
ENSG00000254176	0	0	0	0
ENSG00000254177	0	0	0	0
ENSG00000254178	0	0	0	0
ENSG00000254180	3	16	38	47
ENSG00000254181	0	0	0	0
ENSG00000254182	0	0	0	0
ENSG00000254183	0	0	0	0
ENSG00000254184	0	267	169	384
ENSG00000254185	0	0	1	0
ENSG00000254186	0	0	0	10
ENSG00000254187	0	0	0	0
ENSG00000254189	0	0	0	0
ENSG00000254190	0	0	0	0
ENSG00000254191	0	0	0	1
ENSG00000254192	0	0	0	0
ENSG00000254193	0	0	0	0
ENSG00000254194	0	0	0	0
ENSG00000254195	0	0	0	0
ENSG00000254197	0	0	0	0
ENSG00000254198	0	0	1	0
ENSG00000254199	0	0	0	0
ENSG00000254200	0	0	0	0
ENSG00000254201	0	0	0	0
ENSG00000254202	0	0	0	0
ENSG00000254203	0	0	0	0
ENSG00000254204	0	0	0	0
ENSG00000254205	0	0	0	0
ENSG00000254206	0	6	10	4
ENSG00000254207	0	0	0	0
ENSG00000254208	0	0	0	0
ENSG00000254209	0	0	0	0
ENSG00000254210	0	0	0	0
ENSG00000254211	0	0	0	0
ENSG00000254212	0	0	0	0
ENSG00000254213	0	0	0	0
ENSG00000254214	0	0	0	0
ENSG00000254215	0	0	2	0
ENSG00000254216	0	0	0	0

ENSG00000254219	2	0	0	1
ENSG00000254220	0	0	0	0
ENSG00000254221	0	0	0	0
ENSG00000254222	0	0	0	0
ENSG00000254223	0	0	0	0
ENSG00000254224	0	21	6	18
ENSG00000254225	0	0	0	0
ENSG00000254226	0	0	2	4
ENSG00000254227	0	0	0	0
ENSG00000254228	0	0	0	0
ENSG00000254229	0	0	0	0
ENSG00000254230	0	0	0	0
ENSG00000254231	3	0	26	20
ENSG00000254233	1	0	0	2
ENSG00000254235	0	0	0	0
ENSG00000254236	0	0	5	0
ENSG00000254237	0	0	0	0
ENSG00000254238	0	0	0	0
ENSG00000254239	0	0	0	0
ENSG00000254240	0	0	0	0
ENSG00000254241	0	0	0	0
ENSG00000254242	0	0	0	0
ENSG00000254244	0	0	0	0
ENSG00000254245	0	0	0	0
ENSG00000254246	0	0	0	0
ENSG00000254247	0	0	0	0
ENSG00000254248	0	0	2	0
ENSG00000254249	0	0	0	0
ENSG00000254251	0	0	0	3
ENSG00000254252	0	0	0	0
ENSG00000254253	0	0	0	0
ENSG00000254254	2	0	2	0
ENSG00000254255	0	0	0	0
ENSG00000254256	0	0	0	0
ENSG00000254258	3	0	3	5
ENSG00000254260	0	0	0	0
ENSG00000254261	0	0	0	0
ENSG00000254262	0	0	0	0
ENSG00000254263	0	0	0	0
ENSG00000254264	0	0	0	0
ENSG00000254265	0	0	0	0
ENSG00000254266	1	0	0	7
ENSG00000254267	0	0	0	0
ENSG00000254268	0	0	0	0
ENSG00000254269	0	0	0	0
ENSG00000254270	0	0	0	0

ENSG00000254271	0	0	0	0
ENSG00000254272	0	0	3	0
ENSG00000254273	0	0	0	0
ENSG00000254274	0	0	0	0
ENSG00000254275	1	1	0	0
ENSG00000254276	0	0	0	0
ENSG00000254277	0	0	0	0
ENSG00000254278	0	0	0	0
ENSG00000254279	0	0	0	0
ENSG00000254281	0	0	0	3
ENSG00000254282	0	0	0	0
ENSG00000254283	0	0	0	0
ENSG00000254284	0	0	0	0
ENSG00000254285	0	0	0	0
ENSG00000254286	0	0	0	0
ENSG00000254287	1	0	0	0
ENSG00000254288	0	0	0	0
ENSG00000254289	0	0	0	0
ENSG00000254290	0	0	0	0
ENSG00000254291	0	0	0	0
ENSG00000254292	0	0	0	0
ENSG00000254293	0	0	0	0
ENSG00000254294	0	0	0	0
ENSG00000254295	0	0	0	0
ENSG00000254297	0	0	0	0
ENSG00000254298	0	0	0	0
ENSG00000254299	0	0	0	0
ENSG00000254300	0	0	0	0
ENSG00000254302	0	0	0	0
ENSG00000254303	2	0	3	16
ENSG00000254305	0	0	0	0
ENSG00000254306	0	0	0	0
ENSG00000254307	0	0	0	0
ENSG00000254308	0	0	0	0
ENSG00000254309	0	0	0	0
ENSG00000254310	0	0	0	0
ENSG00000254311	0	0	0	0
ENSG00000254312	0	0	0	0
ENSG00000254313	0	0	0	0
ENSG00000254314	0	0	0	0
ENSG00000254315	0	0	0	0
ENSG00000254316	0	0	0	0
ENSG00000254317	0	0	0	0
ENSG00000254318	0	0	0	0
ENSG00000254319	2	0	16	6
ENSG00000254320	0	0	0	0

ENSG00000254321	0	0	0	0
ENSG00000254324	0	0	0	0
ENSG00000254325	0	0	0	0
ENSG00000254326	0	0	0	0
ENSG00000254328	1	0	1	2
ENSG00000254329	0	0	0	0
ENSG00000254330	0	0	1	0
ENSG00000254331	0	0	0	0
ENSG00000254332	0	1	1	5
ENSG00000254333	0	0	1	2
ENSG00000254334	0	0	0	0
ENSG00000254335	0	0	0	0
ENSG00000254336	0	0	0	0
ENSG00000254337	1	0	0	0
ENSG00000254338	0	0	0	0
ENSG00000254339	0	0	0	0
ENSG00000254340	1	0	0	0
ENSG00000254341	0	0	0	0
ENSG00000254342	0	0	0	0
ENSG00000254343	1	0	0	0
ENSG00000254344	0	0	0	0
ENSG00000254345	0	0	0	0
ENSG00000254346	0	0	0	0
ENSG00000254347	0	0	0	0
ENSG00000254348	0	0	0	0
ENSG00000254349	0	0	0	1
ENSG00000254350	0	0	0	0
ENSG00000254351	0	0	0	0
ENSG00000254352	0	0	1	0
ENSG00000254353	0	0	0	0
ENSG00000254355	0	0	0	0
ENSG00000254357	0	0	0	0
ENSG00000254358	0	0	0	0
ENSG00000254360	0	16	2	0
ENSG00000254361	0	0	0	0
ENSG00000254362	0	0	0	0
ENSG00000254363	0	0	0	3
ENSG00000254364	0	0	0	0
ENSG00000254365	0	0	0	0
ENSG00000254366	0	0	0	0
ENSG00000254367	0	0	0	0
ENSG00000254369	0	0	0	0
ENSG00000254370	0	0	0	0
ENSG00000254372	0	0	0	0
ENSG00000254373	0	0	2	0
ENSG00000254374	0	0	1	0

ENSG00000254376	0	0	0	0
ENSG00000254377	0	0	0	0
ENSG00000254380	0	0	0	0
ENSG00000254381	0	0	0	0
ENSG00000254383	0	0	1	0
ENSG00000254384	0	0	0	0
ENSG00000254387	0	0	0	0
ENSG00000254388	0	0	0	0
ENSG00000254389	0	0	0	0
ENSG00000254391	0	0	0	0
ENSG00000254392	0	0	0	0
ENSG00000254394	0	0	0	0
ENSG00000254395	0	0	0	0
ENSG00000254396	3	1	9	11
ENSG00000254397	0	0	0	0
ENSG00000254398	0	0	0	0
ENSG00000254399	0	6	0	0
ENSG00000254400	0	3	13	8
ENSG00000254401	0	0	0	0
ENSG00000254402	0	0	0	0
ENSG00000254403	0	0	0	0
ENSG00000254404	0	0	0	0
ENSG00000254405	0	0	0	0
ENSG00000254406	0	0	0	0
ENSG00000254407	0	0	0	0
ENSG00000254408	0	0	0	0
ENSG00000254409	0	0	1	0
ENSG00000254411	0	0	0	0
ENSG00000254412	0	0	0	0
ENSG00000254413	0	0	0	0
ENSG00000254414	0	0	1	5
ENSG00000254415	4	0	0	0
ENSG00000254416	0	0	0	0
ENSG00000254417	0	0	0	0
ENSG00000254418	0	0	0	0
ENSG00000254419	0	0	0	0
ENSG00000254420	0	0	1	0
ENSG00000254422	0	0	0	0
ENSG00000254423	0	0	0	0
ENSG00000254424	0	0	0	0
ENSG00000254425	0	0	0	0
ENSG00000254427	0	0	0	0
ENSG00000254428	0	0	0	0
ENSG00000254429	141	4	8	0
ENSG00000254430	0	0	0	0
ENSG00000254431	0	0	0	0

ENSG00000254432	0	0	0	0
ENSG00000254433	0	0	1	2
ENSG00000254434	0	0	0	0
ENSG00000254436	0	0	0	0
ENSG00000254437	0	0	0	0
ENSG00000254438	2	0	0	4
ENSG00000254440	0	0	0	0
ENSG00000254441	0	0	0	0
ENSG00000254442	0	0	0	0
ENSG00000254443	0	0	0	0
ENSG00000254444	0	0	0	0
ENSG00000254445	0	0	0	0
ENSG00000254447	0	0	0	0
ENSG00000254449	0	0	0	0
ENSG00000254450	0	0	0	0
ENSG00000254451	0	0	0	0
ENSG00000254452	0	210	124	50
ENSG00000254453	0	0	0	0
ENSG00000254454	0	0	0	0
ENSG00000254455	0	0	0	0
ENSG00000254456	0	0	0	0
ENSG00000254457	0	0	0	0
ENSG00000254458	0	0	0	0
ENSG00000254459	0	0	0	0
ENSG00000254460	0	0	0	0
ENSG00000254461	0	0	0	0
ENSG00000254462	0	0	0	0
ENSG00000254463	0	0	2	0
ENSG00000254464	0	0	0	0
ENSG00000254465	0	0	0	0
ENSG00000254466	0	0	0	0
ENSG00000254467	0	0	0	0
ENSG00000254468	0	0	0	0
ENSG00000254469	5	0	3	13
ENSG00000254470	3	0	0	0
ENSG00000254471	0	0	0	0
ENSG00000254472	0	0	0	0
ENSG00000254473	10	38	30	91
ENSG00000254474	0	0	0	0
ENSG00000254475	0	0	0	0
ENSG00000254477	0	0	0	0
ENSG00000254478	0	0	0	0
ENSG00000254479	0	0	0	0
ENSG00000254480	0	0	0	0
ENSG00000254481	1309	2400	2649	2739
ENSG00000254482	0	0	0	6

ENSG00000254483	0	0	0	0
ENSG00000254484	0	0	0	0
ENSG00000254485	0	0	0	1
ENSG00000254486	0	0	0	0
ENSG00000254487	0	0	0	0
ENSG00000254488	1	0	0	0
ENSG00000254489	0	0	0	0
ENSG00000254490	0	0	0	0
ENSG00000254491	0	0	0	0
ENSG00000254492	0	0	0	0
ENSG00000254495	1	0	0	0
ENSG00000254496	0	0	0	0
ENSG00000254497	0	0	0	0
ENSG00000254498	0	0	0	0
ENSG00000254499	0	0	0	0
ENSG00000254500	0	0	0	0
ENSG00000254501	0	0	0	0
ENSG00000254502	0	0	0	0
ENSG00000254503	1	0	0	0
ENSG00000254504	0	0	0	0
ENSG00000254505	2	0	2	0
ENSG00000254506	3	73	30	34
ENSG00000254507	0	0	0	0
ENSG00000254508	0	0	0	0
ENSG00000254509	0	0	0	0
ENSG00000254510	2	0	6	0
ENSG00000254511	0	0	0	0
ENSG00000254512	0	0	0	0
ENSG00000254514	0	0	0	0
ENSG00000254515	0	0	0	0
ENSG00000254516	0	0	0	0
ENSG00000254517	0	0	0	0
ENSG00000254518	0	0	0	0
ENSG00000254519	0	0	0	0
ENSG00000254521	14	0	2	2
ENSG00000254522	0	0	0	0
ENSG00000254524	0	0	0	0
ENSG00000254526	0	0	0	0
ENSG00000254527	0	0	0	0
ENSG00000254528	0	0	0	0
ENSG00000254529	0	0	0	0
ENSG00000254530	0	0	0	0
ENSG00000254531	5	0	4	0
ENSG00000254532	0	0	0	0
ENSG00000254533	0	0	0	0
ENSG00000254534	0	0	1	0

ENSG00000254535	0	0	0	0
ENSG00000254536	0	0	0	0
ENSG00000254537	0	0	0	0
ENSG00000254538	0	0	0	7
ENSG00000254539	0	0	0	0
ENSG00000254540	0	0	0	0
ENSG00000254541	0	0	0	0
ENSG00000254542	0	0	0	0
ENSG00000254543	0	0	0	0
ENSG00000254544	0	0	0	0
ENSG00000254545	0	0	5	0
ENSG00000254546	0	0	0	0
ENSG00000254547	0	0	0	0
ENSG00000254548	0	0	0	0
ENSG00000254550	0	0	0	0
ENSG00000254551	0	0	0	0
ENSG00000254552	0	0	0	0
ENSG00000254553	0	0	0	0
ENSG00000254554	0	0	0	0
ENSG00000254555	0	0	0	0
ENSG00000254556	0	0	0	0
ENSG00000254557	0	0	0	0
ENSG00000254558	0	0	0	0
ENSG00000254559	0	0	1	2
ENSG00000254560	0	0	0	0
ENSG00000254561	0	0	0	0
ENSG00000254562	0	0	0	0
ENSG00000254563	0	0	0	0
ENSG00000254564	0	0	0	0
ENSG00000254565	0	0	0	0
ENSG00000254566	0	0	0	0
ENSG00000254567	0	0	0	0
ENSG00000254568	0	0	0	0
ENSG00000254569	0	0	0	0
ENSG00000254571	0	0	9	13
ENSG00000254572	0	0	0	0
ENSG00000254573	0	0	0	0
ENSG00000254574	0	0	0	0
ENSG00000254575	0	0	0	0
ENSG00000254576	0	0	0	0
ENSG00000254577	0	0	0	0
ENSG00000254578	0	0	0	0
ENSG00000254579	0	0	0	0
ENSG00000254580	0	0	0	0
ENSG00000254581	0	0	0	0
ENSG00000254582	0	0	0	0

ENSG00000254583	0	0	0	0
ENSG00000254584	0	0	0	0
ENSG00000254585	0	0	0	0
ENSG00000254586	0	0	0	0
ENSG00000254587	0	0	0	0
ENSG00000254588	0	0	0	0
ENSG00000254589	0	0	2	0
ENSG00000254590	0	0	0	0
ENSG00000254591	0	0	0	0
ENSG00000254592	0	0	0	0
ENSG00000254593	0	0	0	0
ENSG00000254594	0	0	0	0
ENSG00000254595	0	0	0	0
ENSG00000254596	0	0	0	0
ENSG00000254597	0	0	0	0
ENSG00000254598	0	0	0	2
ENSG00000254599	0	0	0	0
ENSG00000254601	0	0	0	0
ENSG00000254602	0	0	2	0
ENSG00000254603	0	0	0	0
ENSG00000254604	2	0	2	3
ENSG00000254605	0	0	0	0
ENSG00000254606	0	0	0	0
ENSG00000254607	0	0	0	1
ENSG00000254608	0	0	0	0
ENSG00000254609	0	0	0	0
ENSG00000254610	0	0	0	16
ENSG00000254612	0	4	7	4
ENSG00000254613	0	0	0	0
ENSG00000254614	3177	7260	2538	7111
ENSG00000254615	43	78	22	18
ENSG00000254616	0	0	0	0
ENSG00000254617	0	0	0	0
ENSG00000254618	0	0	0	0
ENSG00000254619	0	0	0	0
ENSG00000254620	0	0	0	0
ENSG00000254621	0	0	0	0
ENSG00000254622	0	0	0	0
ENSG00000254623	0	0	0	0
ENSG00000254624	0	0	0	0
ENSG00000254625	0	0	0	0
ENSG00000254626	0	0	0	0
ENSG00000254627	0	0	0	0
ENSG00000254629	0	0	0	0
ENSG00000254630	0	0	0	0
ENSG00000254631	1	0	0	2

ENSG00000254632	0	0	0	0
ENSG00000254633	0	0	0	0
ENSG00000254634	1	66	77	45
ENSG00000254635	186	164	144	305
ENSG00000254636	0	0	0	0
ENSG00000254637	0	0	0	0
ENSG00000254638	0	0	0	0
ENSG00000254639	0	0	0	0
ENSG00000254640	0	0	0	0
ENSG00000254641	0	0	0	0
ENSG00000254642	0	0	0	0
ENSG00000254644	0	0	0	0
ENSG00000254645	0	0	0	0
ENSG00000254646	0	0	1	1
ENSG00000254647	0	0	0	0
ENSG00000254648	0	0	0	0
ENSG00000254649	0	0	0	0
ENSG00000254650	0	0	0	0
ENSG00000254651	0	0	0	0
ENSG00000254652	0	0	0	0
ENSG00000254653	0	0	0	0
ENSG00000254654	0	0	0	0
ENSG00000254655	0	0	0	0
ENSG00000254656	0	0	0	0
ENSG00000254658	0	0	0	0
ENSG00000254659	1	76	104	97
ENSG00000254660	0	0	0	0
ENSG00000254661	0	0	0	0
ENSG00000254662	0	0	0	0
ENSG00000254663	0	0	0	0
ENSG00000254664	0	0	0	0
ENSG00000254665	0	0	0	0
ENSG00000254667	0	0	0	0
ENSG00000254668	0	0	0	0
ENSG00000254669	0	0	1	0
ENSG00000254670	0	0	0	0
ENSG00000254671	0	0	0	8
ENSG00000254672	0	0	0	0
ENSG00000254673	0	0	0	0
ENSG00000254674	0	0	0	0
ENSG00000254675	0	0	0	0
ENSG00000254676	0	0	0	0
ENSG00000254677	0	0	0	0
ENSG00000254678	0	0	0	0
ENSG00000254680	0	0	0	0
ENSG00000254681	0	0	0	0

ENSG00000254682	44	56	4	59
ENSG00000254683	0	0	0	0
ENSG00000254684	0	0	0	0
ENSG00000254685	13	107	166	158
ENSG00000254686	0	0	0	0
ENSG00000254687	0	19	56	30
ENSG00000254688	0	0	0	0
ENSG00000254689	0	0	0	0
ENSG00000254690	0	0	0	0
ENSG00000254691	0	0	0	0
ENSG00000254692	0	0	0	0
ENSG00000254693	0	2	20	1
ENSG00000254694	0	0	0	0
ENSG00000254695	0	0	0	0
ENSG00000254696	0	0	0	0
ENSG00000254697	0	0	0	0
ENSG00000254698	0	0	0	0
ENSG00000254699	0	0	0	0
ENSG00000254700	0	0	0	0
ENSG00000254701	0	150	20	30
ENSG00000254702	0	0	0	0
ENSG00000254703	432	649	278	606
ENSG00000254704	0	0	0	0
ENSG00000254705	0	0	0	0
ENSG00000254706	0	0	0	0
ENSG00000254707	0	0	0	0
ENSG00000254708	0	0	0	0
ENSG00000254709	0	0	0	0
ENSG00000254710	0	0	0	0
ENSG00000254712	0	0	0	0
ENSG00000254713	0	0	0	0
ENSG00000254714	0	0	0	0
ENSG00000254715	0	0	0	0
ENSG00000254717	336	364	405	368
ENSG00000254718	115	383	311	209
ENSG00000254719	0	0	0	0
ENSG00000254720	0	0	0	0
ENSG00000254721	0	0	7	0
ENSG00000254722	0	0	0	0
ENSG00000254723	0	0	0	0
ENSG00000254724	0	0	0	0
ENSG00000254725	0	0	1	0
ENSG00000254726	0	0	0	0
ENSG00000254727	0	0	0	0
ENSG00000254728	0	0	0	0
ENSG00000254729	0	0	0	0

ENSG00000254730	0	0	0	0
ENSG00000254731	0	0	0	0
ENSG00000254732	0	0	0	0
ENSG00000254733	0	0	39	0
ENSG00000254734	0	0	0	0
ENSG00000254735	5	0	0	1
ENSG00000254736	0	0	0	0
ENSG00000254737	0	0	0	0
ENSG00000254738	0	0	0	0
ENSG00000254739	0	0	0	0
ENSG00000254740	0	0	0	0
ENSG00000254741	0	0	0	6
ENSG00000254743	0	0	0	0
ENSG00000254744	1	0	0	0
ENSG00000254746	0	0	0	0
ENSG00000254747	0	0	0	0
ENSG00000254748	0	0	0	0
ENSG00000254749	0	0	0	0
ENSG00000254750	0	0	0	0
ENSG00000254751	0	0	0	0
ENSG00000254752	0	0	0	0
ENSG00000254753	0	0	0	0
ENSG00000254754	0	0	0	0
ENSG00000254755	0	0	0	0
ENSG00000254756	0	0	0	0
ENSG00000254757	0	0	0	0
ENSG00000254758	0	0	0	0
ENSG00000254759	0	0	1	0
ENSG00000254760	0	0	0	0
ENSG00000254761	0	0	0	0
ENSG00000254762	0	0	0	0
ENSG00000254764	0	0	0	0
ENSG00000254765	0	0	0	0
ENSG00000254767	1	0	0	0
ENSG00000254768	0	0	0	0
ENSG00000254769	0	0	0	0
ENSG00000254770	0	0	0	0
ENSG00000254771	0	0	0	0
ENSG00000254772	0	0	0	0
ENSG00000254774	0	0	0	0
ENSG00000254775	0	0	0	0
ENSG00000254776	0	0	0	0
ENSG00000254777	1	0	3	3
ENSG00000254778	0	0	0	0
ENSG00000254779	0	0	0	0
ENSG00000254780	0	0	0	0

ENSG00000254781	0	0	0	0
ENSG00000254782	0	0	0	0
ENSG00000254783	1	0	1	9
ENSG00000254784	0	0	0	0
ENSG00000254785	0	0	0	0
ENSG00000254786	345	557	826	1015
ENSG00000254787	0	0	0	0
ENSG00000254788	0	0	0	0
ENSG00000254789	0	0	0	0
ENSG00000254790	0	0	0	0
ENSG00000254791	0	0	0	0
ENSG00000254792	0	0	0	0
ENSG00000254793	0	0	0	2
ENSG00000254794	0	0	0	0
ENSG00000254795	0	0	0	0
ENSG00000254796	0	0	0	0
ENSG00000254798	0	0	3	4
ENSG00000254799	0	0	0	0
ENSG00000254800	0	0	0	0
ENSG00000254801	0	0	0	0
ENSG00000254802	41	25	28	70
ENSG00000254803	0	0	0	0
ENSG00000254804	0	0	0	0
ENSG00000254805	0	0	0	0
ENSG00000254806	0	0	0	0
ENSG00000254807	0	0	0	0
ENSG00000254810	392	858	1119	1041
ENSG00000254811	0	0	0	0
ENSG00000254812	0	0	0	0
ENSG00000254813	0	0	0	0
ENSG00000254814	0	0	0	0
ENSG00000254815	0	0	0	0
ENSG00000254816	0	0	0	0
ENSG00000254817	0	0	0	0
ENSG00000254818	0	0	0	0
ENSG00000254819	0	0	0	0
ENSG00000254820	0	0	0	0
ENSG00000254821	0	0	0	0
ENSG00000254822	0	0	0	0
ENSG00000254823	0	0	0	0
ENSG00000254824	0	0	0	0
ENSG00000254825	0	0	0	0
ENSG00000254826	0	0	0	0
ENSG00000254827	0	0	0	0
ENSG00000254828	0	0	0	0
ENSG00000254829	0	0	0	0

ENSG00000254830	0	0	0	0
ENSG00000254831	0	0	0	0
ENSG00000254832	0	0	0	0
ENSG00000254833	0	0	0	0
ENSG00000254834	0	0	0	0
ENSG00000254835	0	0	0	0
ENSG00000254836	1	0	0	0
ENSG00000254837	6	43	36	27
ENSG00000254838	13	30	36	26
ENSG00000254839	4	0	12	8
ENSG00000254840	0	0	0	0
ENSG00000254841	0	0	0	0
ENSG00000254842	0	0	4	0
ENSG00000254843	0	0	0	0
ENSG00000254844	0	0	0	0
ENSG00000254845	0	0	0	0
ENSG00000254846	0	0	0	0
ENSG00000254847	0	0	0	0
ENSG00000254848	0	0	0	0
ENSG00000254849	0	0	0	0
ENSG00000254850	0	0	0	0
ENSG00000254851	0	0	0	0
ENSG00000254852	0	0	0	0
ENSG00000254853	0	0	0	0
ENSG00000254854	0	0	0	0
ENSG00000254855	0	0	0	0
ENSG00000254856	0	0	0	0
ENSG00000254857	0	0	0	0
ENSG00000254858	3	0	0	3
ENSG00000254859	1	0	0	1
ENSG00000254860	0	0	2	3
ENSG00000254861	0	0	0	0
ENSG00000254862	1	0	3	0
ENSG00000254863	2	0	0	0
ENSG00000254864	0	0	0	0
ENSG00000254865	0	0	0	0
ENSG00000254866	0	0	0	0
ENSG00000254867	0	0	0	0
ENSG00000254869	0	0	0	0
ENSG00000254870	0	0	0	0
ENSG00000254871	0	0	0	0
ENSG00000254872	0	0	0	0
ENSG00000254873	0	67	70	34
ENSG00000254874	0	0	0	0
ENSG00000254875	0	0	0	0
ENSG00000254876	0	0	1	3

ENSG00000254877	199	189	140	123
ENSG00000254878	0	0	0	0
ENSG00000254879	0	0	0	0
ENSG00000254880	0	0	0	0
ENSG00000254883	0	0	0	0
ENSG00000254884	0	0	0	0
ENSG00000254885	0	0	1	0
ENSG00000254886	0	0	0	0
ENSG00000254887	1	14	2	0
ENSG00000254888	0	0	0	0
ENSG00000254889	0	0	0	0
ENSG00000254890	0	0	0	0
ENSG00000254891	0	0	0	0
ENSG00000254892	0	0	0	0
ENSG00000254893	0	1616	1356	2355
ENSG00000254894	0	0	0	0
ENSG00000254895	0	0	0	0
ENSG00000254896	0	0	0	0
ENSG00000254897	0	0	0	0
ENSG00000254898	0	0	5	1
ENSG00000254899	0	0	0	0
ENSG00000254900	0	0	0	0
ENSG00000254901	2	20	1	0
ENSG00000254902	0	0	0	0
ENSG00000254903	0	0	0	0
ENSG00000254905	0	0	0	0
ENSG00000254906	0	0	0	0
ENSG00000254907	0	0	23	30
ENSG00000254909	0	0	1	0
ENSG00000254910	0	0	0	0
ENSG00000254911	0	0	0	0
ENSG00000254912	39	638	380	2915
ENSG00000254913	0	0	0	0
ENSG00000254914	0	0	0	0
ENSG00000254915	0	0	0	0
ENSG00000254916	0	0	0	0
ENSG00000254917	0	0	0	0
ENSG00000254918	5	0	7	17
ENSG00000254919	0	0	0	1
ENSG00000254920	0	0	0	0
ENSG00000254921	0	0	0	0
ENSG00000254923	0	0	0	0
ENSG00000254924	0	0	0	0
ENSG00000254925	0	0	0	0
ENSG00000254926	0	0	0	0
ENSG00000254927	0	0	0	0

ENSG00000254928	0	11	1	0
ENSG00000254929	0	0	0	0
ENSG00000254930	0	0	0	0
ENSG00000254931	0	0	0	0
ENSG00000254932	0	0	0	0
ENSG00000254933	0	4	22	67
ENSG00000254934	0	0	0	0
ENSG00000254935	0	0	0	0
ENSG00000254936	0	0	0	0
ENSG00000254937	0	0	0	0
ENSG00000254938	0	0	0	0
ENSG00000254939	0	0	0	0
ENSG00000254940	0	0	0	0
ENSG00000254941	0	0	0	0
ENSG00000254942	0	0	0	0
ENSG00000254943	0	0	0	0
ENSG00000254944	0	0	0	0
ENSG00000254946	0	0	0	0
ENSG00000254947	0	0	0	0
ENSG00000254948	0	0	0	0
ENSG00000254949	0	0	0	0
ENSG00000254951	0	0	0	0
ENSG00000254952	1	0	0	0
ENSG00000254953	0	0	0	0
ENSG00000254954	0	0	0	0
ENSG00000254957	0	0	0	0
ENSG00000254958	0	0	0	0
ENSG00000254959	0	0	0	0
ENSG00000254960	0	0	0	0
ENSG00000254961	0	0	0	0
ENSG00000254962	0	0	0	0
ENSG00000254963	0	0	11	4
ENSG00000254964	0	0	0	0
ENSG00000254965	1	58	36	56
ENSG00000254966	0	0	0	0
ENSG00000254967	0	0	0	0
ENSG00000254968	0	0	0	0
ENSG00000254971	0	0	0	0
ENSG00000254972	0	0	0	0
ENSG00000254973	0	0	0	0
ENSG00000254974	0	19	9	8
ENSG00000254975	85	38	5	8
ENSG00000254976	0	0	0	0
ENSG00000254977	0	0	0	0
ENSG00000254978	0	0	0	0
ENSG00000254979	0	0	0	0

ENSG00000254980	0	0	0	0
ENSG00000254981	0	0	0	0
ENSG00000254982	0	0	1	5
ENSG00000254983	0	0	0	0
ENSG00000254984	0	0	0	0
ENSG00000254985	0	0	0	0
ENSG00000254986	7	1	7	4
ENSG00000254987	0	0	0	0
ENSG00000254988	0	15	6	12
ENSG00000254989	0	0	0	0
ENSG00000254990	0	0	0	0
ENSG00000254991	0	0	0	0
ENSG00000254992	0	0	0	0
ENSG00000254993	0	0	0	0
ENSG00000254994	0	0	0	0
ENSG00000254995	0	0	0	0
ENSG00000254996	0	0	0	1
ENSG00000254997	0	0	0	0
ENSG00000254998	0	0	0	0
ENSG00000254999	3672	4197	3869	4285
ENSG00000255000	0	0	0	0
ENSG00000255001	0	0	0	0
ENSG00000255002	268	26	34	27
ENSG00000255003	0	0	0	0
ENSG00000255004	0	0	0	0
ENSG00000255005	0	0	0	0
ENSG00000255006	0	0	0	0
ENSG00000255007	67	0	14	23
ENSG00000255008	0	0	0	0
ENSG00000255009	0	0	0	0
ENSG00000255010	0	0	0	0
ENSG00000255011	0	0	0	0
ENSG00000255012	0	0	0	0
ENSG00000255013	0	0	0	0
ENSG00000255014	0	0	0	0
ENSG00000255015	0	0	0	0
ENSG00000255016	0	0	0	0
ENSG00000255018	0	0	0	0
ENSG00000255019	0	0	0	0
ENSG00000255020	0	0	0	0
ENSG00000255021	4	0	1	0
ENSG00000255022	0	0	0	0
ENSG00000255025	0	0	0	0
ENSG00000255026	0	0	0	0
ENSG00000255027	0	0	0	0
ENSG00000255028	38	624	1041	1227

ENSG00000255029	0	0	0	0
ENSG00000255030	0	0	0	0
ENSG00000255031	0	0	0	0
ENSG00000255032	0	0	0	0
ENSG00000255033	0	0	0	0
ENSG00000255035	0	0	0	0
ENSG00000255036	0	54	16	47
ENSG00000255037	0	0	0	0
ENSG00000255038	0	0	2	2
ENSG00000255039	0	0	0	0
ENSG00000255040	0	0	0	0
ENSG00000255041	0	0	0	0
ENSG00000255042	0	0	0	0
ENSG00000255043	0	0	0	0
ENSG00000255045	5	201	122	107
ENSG00000255046	5	0	0	0
ENSG00000255047	0	0	0	0
ENSG00000255048	0	0	0	0
ENSG00000255050	0	0	0	0
ENSG00000255051	1	0	2	16
ENSG00000255052	0	0	0	0
ENSG00000255053	0	0	0	0
ENSG00000255054	0	0	0	0
ENSG00000255055	0	0	0	0
ENSG00000255057	0	0	0	0
ENSG00000255058	1	0	12	12
ENSG00000255059	2	0	1	0
ENSG00000255060	0	0	0	0
ENSG00000255062	1	0	1	0
ENSG00000255063	0	0	0	0
ENSG00000255065	0	0	0	0
ENSG00000255067	0	0	0	0
ENSG00000255069	0	0	0	0
ENSG00000255070	0	0	0	0
ENSG00000255071	0	0	0	0
ENSG00000255072	0	0	0	0
ENSG00000255073	0	0	0	0
ENSG00000255074	0	0	0	0
ENSG00000255075	0	0	0	0
ENSG00000255076	0	0	0	0
ENSG00000255077	0	0	0	0
ENSG00000255078	0	0	0	0
ENSG00000255079	306	469	186	233
ENSG00000255080	0	13	8	8
ENSG00000255081	0	0	0	0
ENSG00000255082	0	12	0	0

ENSG00000255083	0	0	0	0
ENSG00000255084	0	0	0	0
ENSG00000255085	0	0	0	0
ENSG00000255086	0	0	0	0
ENSG00000255087	0	0	0	0
ENSG00000255088	0	0	0	0
ENSG00000255089	0	0	0	0
ENSG00000255090	0	0	0	0
ENSG00000255091	0	0	0	0
ENSG00000255092	0	0	0	0
ENSG00000255093	0	0	0	0
ENSG00000255094	0	0	1	0
ENSG00000255095	0	0	0	0
ENSG00000255096	0	0	0	0
ENSG00000255097	0	0	0	0
ENSG00000255098	0	0	0	0
ENSG00000255099	0	0	0	0
ENSG00000255100	0	0	0	0
ENSG00000255101	0	0	0	0
ENSG00000255102	0	22	0	1
ENSG00000255103	0	0	2	6
ENSG00000255104	0	0	0	0
ENSG00000255105	0	0	0	0
ENSG00000255106	0	0	0	0
ENSG00000255107	0	0	0	0
ENSG00000255108	0	0	0	3
ENSG00000255109	0	0	0	0
ENSG00000255110	0	0	0	0
ENSG00000255111	0	0	0	0
ENSG00000255112	152	1332	1259	1160
ENSG00000255113	0	0	0	0
ENSG00000255114	0	0	0	0
ENSG00000255115	0	0	0	0
ENSG00000255116	0	0	0	0
ENSG00000255117	0	0	0	0
ENSG00000255118	0	0	0	0
ENSG00000255119	0	0	0	0
ENSG00000255120	0	0	0	0
ENSG00000255121	3	0	0	0
ENSG00000255122	0	0	2	0
ENSG00000255123	0	0	0	0
ENSG00000255124	0	0	0	0
ENSG00000255125	0	0	0	0
ENSG00000255126	0	51	44	112
ENSG00000255127	0	0	0	0
ENSG00000255128	0	0	0	0

ENSG00000255129	0	0	0	0
ENSG00000255130	0	0	0	0
ENSG00000255131	0	0	0	0
ENSG00000255132	0	0	0	0
ENSG00000255133	0	0	0	0
ENSG00000255134	0	0	0	0
ENSG00000255135	44	20	8	31
ENSG00000255136	0	0	0	0
ENSG00000255137	0	0	0	0
ENSG00000255138	0	0	0	0
ENSG00000255139	0	3	11	25
ENSG00000255140	0	0	0	0
ENSG00000255141	0	0	0	0
ENSG00000255142	0	0	0	0
ENSG00000255143	0	0	0	0
ENSG00000255144	0	0	0	0
ENSG00000255145	15	13	45	53
ENSG00000255146	0	0	0	0
ENSG00000255147	0	0	0	0
ENSG00000255148	0	0	0	0
ENSG00000255149	0	0	0	0
ENSG00000255150	1	22	1	2
ENSG00000255151	0	0	0	0
ENSG00000255152	0	0	0	0
ENSG00000255153	31	46	53	14
ENSG00000255154	0	0	0	0
ENSG00000255155	0	0	0	0
ENSG00000255156	0	0	0	0
ENSG00000255157	0	0	0	0
ENSG00000255158	0	0	0	0
ENSG00000255159	0	7	0	0
ENSG00000255160	0	0	0	0
ENSG00000255161	0	0	0	0
ENSG00000255162	0	0	0	0
ENSG00000255163	0	0	0	0
ENSG00000255164	0	0	0	0
ENSG00000255165	0	0	0	0
ENSG00000255166	0	0	0	0
ENSG00000255167	0	0	0	0
ENSG00000255168	0	0	0	0
ENSG00000255169	0	0	0	0
ENSG00000255170	0	0	0	0
ENSG00000255171	0	0	0	0
ENSG00000255172	0	0	0	0
ENSG00000255173	0	0	0	0
ENSG00000255174	0	0	0	0

ENSG00000255175	0	0	0	0
ENSG00000255176	0	0	0	1
ENSG00000255177	0	0	0	0
ENSG00000255178	0	0	0	0
ENSG00000255179	0	0	0	0
ENSG00000255181	0	0	0	0
ENSG00000255182	0	0	0	0
ENSG00000255183	0	0	0	0
ENSG00000255184	0	0	0	0
ENSG00000255185	6	20	5	10
ENSG00000255186	0	0	0	0
ENSG00000255187	0	0	0	1
ENSG00000255188	0	0	0	0
ENSG00000255189	85	24	103	38
ENSG00000255190	0	0	0	0
ENSG00000255191	0	0	0	0
ENSG00000255192	0	0	0	0
ENSG00000255193	0	0	0	0
ENSG00000255194	0	0	0	0
ENSG00000255196	0	0	0	0
ENSG00000255197	4	0	0	1
ENSG00000255198	208	9	5	11
ENSG00000255199	0	0	0	0
ENSG00000255200	831	545	437	563
ENSG00000255201	0	0	0	0
ENSG00000255202	0	0	0	0
ENSG00000255203	0	0	0	1
ENSG00000255204	0	0	0	0
ENSG00000255205	0	0	0	0
ENSG00000255206	0	0	0	0
ENSG00000255207	0	0	0	0
ENSG00000255208	0	0	0	0
ENSG00000255209	0	0	0	0
ENSG00000255210	0	0	0	0
ENSG00000255211	0	0	0	0
ENSG00000255213	0	0	0	0
ENSG00000255214	0	0	0	0
ENSG00000255215	0	0	0	0
ENSG00000255216	0	0	0	0
ENSG00000255217	0	0	0	0
ENSG00000255218	0	0	0	0
ENSG00000255219	0	0	0	0
ENSG00000255220	0	0	0	0
ENSG00000255221	0	0	0	0
ENSG00000255222	1	0	0	0
ENSG00000255223	0	0	0	0

ENSG00000255224	2	10	9	4
ENSG00000255225	0	0	0	0
ENSG00000255226	0	0	0	0
ENSG00000255227	0	0	0	0
ENSG00000255229	0	0	1	0
ENSG00000255230	0	0	0	0
ENSG00000255231	0	0	0	0
ENSG00000255232	0	0	0	0
ENSG00000255233	0	0	0	0
ENSG00000255234	0	0	0	0
ENSG00000255235	0	0	0	0
ENSG00000255236	0	0	0	0
ENSG00000255237	14	0	0	0
ENSG00000255238	0	0	0	0
ENSG00000255239	0	0	2	0
ENSG00000255240	1737	2546	1402	3004
ENSG00000255241	0	0	0	0
ENSG00000255242	0	0	0	0
ENSG00000255243	0	0	0	0
ENSG00000255244	0	0	0	0
ENSG00000255245	0	0	0	0
ENSG00000255246	0	0	0	0
ENSG00000255247	0	0	0	0
ENSG00000255248	0	0	0	0
ENSG00000255250	0	0	0	0
ENSG00000255251	0	0	0	0
ENSG00000255252	0	0	0	0
ENSG00000255253	0	0	0	0
ENSG00000255254	0	0	0	0
ENSG00000255255	0	0	0	0
ENSG00000255256	0	0	0	0
ENSG00000255257	0	0	0	0
ENSG00000255258	0	0	0	0
ENSG00000255259	0	0	0	0
ENSG00000255260	0	0	0	0
ENSG00000255261	0	0	0	0
ENSG00000255262	0	0	0	0
ENSG00000255263	0	0	0	0
ENSG00000255265	0	0	0	0
ENSG00000255266	0	0	0	0
ENSG00000255267	0	0	0	0
ENSG00000255268	0	0	0	0
ENSG00000255269	0	0	0	0
ENSG00000255270	0	0	0	0
ENSG00000255271	0	0	0	0
ENSG00000255272	0	0	0	0

ENSG00000255273	0	0	0	0
ENSG00000255274	0	0	0	0
ENSG00000255275	0	0	0	0
ENSG00000255276	0	0	0	0
ENSG00000255277	0	0	0	0
ENSG00000255279	0	0	0	0
ENSG00000255280	0	0	0	0
ENSG00000255281	0	0	0	0
ENSG00000255282	0	0	1	0
ENSG00000255283	0	0	0	0
ENSG00000255284	0	0	0	2
ENSG00000255285	0	0	0	0
ENSG00000255286	0	0	0	0
ENSG00000255287	0	0	0	0
ENSG00000255288	0	0	0	0
ENSG00000255289	0	0	0	0
ENSG00000255291	0	0	0	0
ENSG00000255292	0	0	0	0
ENSG00000255293	0	0	0	0
ENSG00000255294	0	0	0	0
ENSG00000255295	0	0	0	0
ENSG00000255296	0	0	0	0
ENSG00000255297	0	0	0	0
ENSG00000255298	0	0	0	0
ENSG00000255299	0	0	0	0
ENSG00000255300	0	0	0	0
ENSG00000255301	0	0	0	0
ENSG00000255302	675	1676	1466	2148
ENSG00000255303	0	0	0	0
ENSG00000255304	0	0	0	0
ENSG00000255305	0	0	0	0
ENSG00000255306	0	0	0	0
ENSG00000255307	0	0	0	0
ENSG00000255308	0	0	2	0
ENSG00000255309	0	0	0	0
ENSG00000255310	4	0	43	26
ENSG00000255311	0	0	0	0
ENSG00000255312	0	0	0	0
ENSG00000255313	0	0	0	0
ENSG00000255314	0	0	0	0
ENSG00000255315	0	0	0	0
ENSG00000255316	0	0	0	0
ENSG00000255317	0	0	0	0
ENSG00000255318	1	0	0	0
ENSG00000255319	2	0	22	5
ENSG00000255320	44	14	2	0

ENSG00000255321	0	0	0	0
ENSG00000255322	1	134	62	124
ENSG00000255323	0	0	0	0
ENSG00000255325	136	324	311	370
ENSG00000255326	1	25	15	13
ENSG00000255327	0	0	0	0
ENSG00000255328	1	0	0	0
ENSG00000255329	0	0	0	0
ENSG00000255330	0	0	0	0
ENSG00000255331	0	0	0	0
ENSG00000255332	0	0	0	0
ENSG00000255333	0	0	0	0
ENSG00000255334	0	0	0	0
ENSG00000255335	0	0	0	0
ENSG00000255336	0	0	0	0
ENSG00000255337	0	0	0	0
ENSG00000255338	0	0	0	0
ENSG00000255339	0	0	0	0
ENSG00000255340	0	0	0	1
ENSG00000255341	0	0	0	0
ENSG00000255342	0	0	0	0
ENSG00000255343	0	0	0	0
ENSG00000255344	0	0	0	0
ENSG00000255345	0	0	0	0
ENSG00000255346	0	0	1	0
ENSG00000255347	0	0	0	0
ENSG00000255348	0	0	0	0
ENSG00000255349	0	0	0	0
ENSG00000255350	0	0	0	0
ENSG00000255351	0	0	0	2
ENSG00000255352	0	0	0	0
ENSG00000255353	0	0	0	0
ENSG00000255354	0	0	0	0
ENSG00000255355	0	0	0	0
ENSG00000255356	0	0	0	0
ENSG00000255357	0	0	0	0
ENSG00000255358	0	0	0	0
ENSG00000255359	0	0	0	0
ENSG00000255360	0	0	0	0
ENSG00000255361	0	0	0	0
ENSG00000255362	0	0	0	0
ENSG00000255363	34	83	48	70
ENSG00000255364	61	952	531	1388
ENSG00000255365	0	0	0	0
ENSG00000255366	0	0	0	0
ENSG00000255367	1	91	46	133

ENSG00000255368	0	0	0	0
ENSG00000255369	0	0	0	0
ENSG00000255370	0	0	0	0
ENSG00000255371	0	0	0	0
ENSG00000255372	0	0	0	0
ENSG00000255373	0	0	0	0
ENSG00000255374	0	0	0	2
ENSG00000255375	0	0	0	0
ENSG00000255376	0	0	0	0
ENSG00000255377	0	0	0	0
ENSG00000255378	0	0	0	0
ENSG00000255379	0	0	0	0
ENSG00000255380	0	0	0	0
ENSG00000255381	0	0	0	0
ENSG00000255382	0	0	0	0
ENSG00000255384	1	0	0	0
ENSG00000255385	0	0	0	0
ENSG00000255386	0	0	0	0
ENSG00000255387	0	0	2	0
ENSG00000255388	0	0	0	0
ENSG00000255389	1	0	2	3
ENSG00000255390	0	0	0	0
ENSG00000255391	0	0	0	0
ENSG00000255393	0	0	0	0
ENSG00000255394	0	0	0	0
ENSG00000255395	0	0	0	0
ENSG00000255396	0	0	0	0
ENSG00000255397	0	0	0	0
ENSG00000255398	1	0	0	0
ENSG00000255399	0	0	0	0
ENSG00000255400	0	0	0	0
ENSG00000255401	0	0	0	0
ENSG00000255402	0	0	0	0
ENSG00000255403	0	0	0	0
ENSG00000255404	0	0	0	0
ENSG00000255406	0	0	0	0
ENSG00000255408	0	0	0	0
ENSG00000255409	0	0	0	0
ENSG00000255410	0	0	0	0
ENSG00000255411	0	0	0	0
ENSG00000255413	0	0	0	0
ENSG00000255414	0	0	0	0
ENSG00000255415	0	0	0	0
ENSG00000255416	0	0	0	0
ENSG00000255417	0	0	0	0
ENSG00000255418	5	302	306	426

ENSG00000255419	0	0	0	0
ENSG00000255420	0	0	0	0
ENSG00000255421	0	0	0	0
ENSG00000255422	0	0	0	0
ENSG00000255423	1	0	0	0
ENSG00000255424	0	0	0	0
ENSG00000255425	0	0	0	0
ENSG00000255426	0	0	0	0
ENSG00000255427	0	0	0	0
ENSG00000255428	0	0	0	0
ENSG00000255429	0	0	0	0
ENSG00000255430	0	0	0	0
ENSG00000255431	0	0	0	0
ENSG00000255432	0	0	0	0
ENSG00000255433	0	0	0	0
ENSG00000255434	1	0	0	0
ENSG00000255435	0	0	1	0
ENSG00000255436	0	0	0	0
ENSG00000255437	0	0	0	0
ENSG00000255438	0	0	0	0
ENSG00000255439	0	0	0	0
ENSG00000255440	0	0	1	0
ENSG00000255441	0	0	0	0
ENSG00000255442	0	0	0	0
ENSG00000255443	0	0	2	4
ENSG00000255444	0	0	0	0
ENSG00000255445	0	0	0	0
ENSG00000255446	0	0	0	0
ENSG00000255447	0	0	0	0
ENSG00000255448	0	0	0	0
ENSG00000255449	0	0	0	0
ENSG00000255450	0	0	0	0
ENSG00000255451	0	40	15	14
ENSG00000255452	0	0	0	0
ENSG00000255454	0	0	0	0
ENSG00000255455	6	23	29	0
ENSG00000255456	0	0	0	0
ENSG00000255457	0	0	0	0
ENSG00000255458	0	0	0	0
ENSG00000255459	0	0	0	0
ENSG00000255460	0	0	0	0
ENSG00000255461	0	0	0	0
ENSG00000255462	0	0	0	1
ENSG00000255463	0	0	0	0
ENSG00000255464	0	0	0	0
ENSG00000255465	1	27	2	4

ENSG00000255466	0	0	0	0
ENSG00000255467	0	0	2	0
ENSG00000255468	0	0	0	0
ENSG00000255469	0	0	0	0
ENSG00000255470	0	0	0	0
ENSG00000255471	0	0	1	0
ENSG00000255472	0	0	0	0
ENSG00000255474	0	0	0	0
ENSG00000255475	0	0	0	0
ENSG00000255476	0	0	0	0
ENSG00000255477	0	0	0	0
ENSG00000255478	4	212	227	178
ENSG00000255479	87	119	54	75
ENSG00000255480	3	0	3	18
ENSG00000255481	0	0	0	0
ENSG00000255482	0	0	0	0
ENSG00000255483	0	0	0	0
ENSG00000255484	0	0	0	0
ENSG00000255485	0	0	0	0
ENSG00000255486	0	0	0	0
ENSG00000255487	0	0	0	0
ENSG00000255489	0	0	0	0
ENSG00000255490	0	0	0	0
ENSG00000255491	0	0	0	6
ENSG00000255492	0	0	0	0
ENSG00000255493	0	0	0	0
ENSG00000255494	0	0	0	0
ENSG00000255495	0	0	0	2
ENSG00000255496	0	1	0	3
ENSG00000255497	0	0	0	0
ENSG00000255498	0	0	0	0
ENSG00000255499	0	0	0	0
ENSG00000255500	0	0	0	1
ENSG00000255501	0	0	0	0
ENSG00000255502	0	0	0	0
ENSG00000255503	1	0	0	0
ENSG00000255504	0	0	0	0
ENSG00000255505	0	0	0	0
ENSG00000255506	0	0	0	0
ENSG00000255507	5	0	2	5
ENSG00000255508	0	0	0	0
ENSG00000255509	0	0	0	0
ENSG00000255510	0	0	0	0
ENSG00000255511	0	0	0	0
ENSG00000255512	0	0	0	0
ENSG00000255513	0	0	0	0

ENSG00000255514	0	1	0	7
ENSG00000255515	0	0	0	0
ENSG00000255516	0	0	0	0
ENSG00000255517	22	0	8	4
ENSG00000255518	0	0	0	0
ENSG00000255519	0	0	0	0
ENSG00000255520	0	0	0	0
ENSG00000255521	0	0	0	0
ENSG00000255522	0	0	0	0
ENSG00000255523	0	0	0	2
ENSG00000255524	0	0	1	2
ENSG00000255525	0	0	0	0
ENSG00000255526	0	0	0	0
ENSG00000255527	0	0	0	0
ENSG00000255528	0	0	0	0
ENSG00000255529	36	391	444	277
ENSG00000255530	0	0	0	0
ENSG00000255531	0	0	2	2
ENSG00000255532	0	0	0	0
ENSG00000255533	0	0	0	0
ENSG00000255534	0	0	0	0
ENSG00000255535	0	0	0	0
ENSG00000255536	0	0	0	0
ENSG00000255537	0	0	0	0
ENSG00000255538	0	0	0	0
ENSG00000255539	0	0	1	0
ENSG00000255540	0	0	0	0
ENSG00000255541	0	0	0	0
ENSG00000255542	0	0	0	0
ENSG00000255543	0	0	0	0
ENSG00000255544	0	0	0	0
ENSG00000255545	0	0	0	0
ENSG00000255546	0	0	0	0
ENSG00000255547	0	0	0	0
ENSG00000255548	0	0	0	0
ENSG00000255549	0	0	0	0
ENSG00000255550	0	0	0	0
ENSG00000255551	0	0	0	0
ENSG00000255552	59	35	34	20
ENSG00000255553	0	0	0	0
ENSG00000255554	0	0	0	0
ENSG00000255555	0	0	0	0
ENSG00000255556	0	0	0	0
ENSG00000255557	0	0	0	0
ENSG00000255558	0	0	0	0
ENSG00000255559	0	0	0	0

ENSG00000255560	0	0	0	0
ENSG00000255561	0	19	0	0
ENSG00000255562	0	0	0	0
ENSG00000255563	0	0	0	0
ENSG00000255565	0	0	0	0
ENSG00000255566	0	0	0	0
ENSG00000255568	0	0	0	1
ENSG00000255569	0	0	0	0
ENSG00000255571	2	0	0	0
ENSG00000255572	0	0	0	0
ENSG00000255575	0	0	0	0
ENSG00000255580	0	0	0	0
ENSG00000255581	0	0	0	4
ENSG00000255582	0	0	0	0
ENSG00000255583	0	0	0	0
ENSG00000255585	0	0	0	0
ENSG00000255587	0	0	0	0
ENSG00000255591	0	0	0	0
ENSG00000255594	0	0	0	0
ENSG00000255595	0	0	0	0
ENSG00000255599	0	0	0	0
ENSG00000255605	0	0	0	0
ENSG00000255606	2	0	0	0
ENSG00000255608	0	0	0	0
ENSG00000255618	0	0	0	0
ENSG00000255619	0	0	0	0
ENSG00000255621	3	44	31	10
ENSG00000255622	0	0	0	0
ENSG00000255624	2	35	22	42
ENSG00000255627	0	0	0	0
ENSG00000255628	0	0	0	0
ENSG00000255629	0	0	0	0
ENSG00000255633	40	0	0	0
ENSG00000255637	0	0	0	0
ENSG00000255638	0	0	0	0
ENSG00000255639	0	0	0	0
ENSG00000255641	0	0	0	0
ENSG00000255642	0	0	0	0
ENSG00000255644	0	0	0	0
ENSG00000255647	0	0	0	0
ENSG00000255648	0	0	0	0
ENSG00000255649	0	0	0	0
ENSG00000255650	0	0	0	0
ENSG00000255651	0	0	0	0
ENSG00000255652	0	0	0	0
ENSG00000255653	0	0	0	0

ENSG00000255655	0	0	6	9
ENSG00000255660	0	0	0	0
ENSG00000255661	0	0	1	14
ENSG00000255663	0	0	1	6
ENSG00000255664	0	0	0	0
ENSG00000255666	0	0	0	5
ENSG00000255669	0	0	0	0
ENSG00000255670	0	0	0	2
ENSG00000255671	0	0	0	0
ENSG00000255672	0	0	0	0
ENSG00000255674	0	0	0	0
ENSG00000255679	1	0	0	2
ENSG00000255680	3	87	129	65
ENSG00000255681	0	0	0	0
ENSG00000255686	0	0	0	0
ENSG00000255689	0	0	0	0
ENSG00000255690	0	0	0	0
ENSG00000255692	0	0	0	0
ENSG00000255693	0	0	0	0
ENSG00000255698	0	0	0	0
ENSG00000255700	0	0	0	0
ENSG00000255701	0	0	0	0
ENSG00000255703	0	0	0	0
ENSG00000255704	0	0	0	0
ENSG00000255707	0	0	0	0
ENSG00000255710	0	0	0	0
ENSG00000255713	0	0	0	0
ENSG00000255714	0	0	0	0
ENSG00000255717	11	60	14	25
ENSG00000255723	0	0	0	0
ENSG00000255725	0	0	0	0
ENSG00000255726	0	0	0	0
ENSG00000255727	1	0	0	0
ENSG00000255729	0	0	0	0
ENSG00000255730	0	0	0	0
ENSG00000255733	0	0	0	0
ENSG00000255734	0	0	0	0
ENSG00000255735	0	0	0	0
ENSG00000255737	0	0	2	0
ENSG00000255738	0	0	0	0
ENSG00000255740	0	0	0	0
ENSG00000255741	0	0	0	0
ENSG00000255743	0	0	0	0
ENSG00000255745	0	0	0	0
ENSG00000255746	0	0	0	0
ENSG00000255748	0	0	0	0

ENSG00000255749	0	0	0	0
ENSG00000255750	0	0	0	0
ENSG00000255752	0	0	0	0
ENSG00000255753	0	0	0	0
ENSG00000255757	0	0	0	0
ENSG00000255760	0	0	0	0
ENSG00000255763	0	0	0	0
ENSG00000255767	0	0	0	0
ENSG00000255769	0	0	0	0
ENSG00000255772	0	0	0	0
ENSG00000255774	0	0	0	0
ENSG00000255775	45	12	0	0
ENSG00000255776	0	0	0	0
ENSG00000255778	0	0	0	0
ENSG00000255780	0	0	0	0
ENSG00000255782	0	0	0	0
ENSG00000255783	0	0	0	0
ENSG00000255786	0	0	0	0
ENSG00000255787	0	0	0	0
ENSG00000255790	0	0	0	0
ENSG00000255794	0	14	9	5
ENSG00000255798	0	0	2	0
ENSG00000255800	0	0	4	23
ENSG00000255801	0	0	0	0
ENSG00000255804	0	0	0	0
ENSG00000255807	0	0	0	0
ENSG00000255808	0	0	0	0
ENSG00000255811	0	8	4	15
ENSG00000255813	0	0	0	0
ENSG00000255814	0	0	0	0
ENSG00000255815	0	0	0	0
ENSG00000255817	0	0	0	0
ENSG00000255819	0	0	0	0
ENSG00000255823	42	0	1	0
ENSG00000255824	0	0	0	0
ENSG00000255825	0	0	0	0
ENSG00000255829	0	0	0	0
ENSG00000255830	0	0	0	0
ENSG00000255831	0	0	0	0
ENSG00000255833	4	0	1	0
ENSG00000255835	0	0	0	0
ENSG00000255836	7	0	3	3
ENSG00000255837	0	17	5	11
ENSG00000255838	0	0	0	0
ENSG00000255839	0	0	0	0
ENSG00000255840	0	0	0	0

ENSG00000255843	0	0	0	0
ENSG00000255845	0	0	0	0
ENSG00000255847	0	0	0	0
ENSG00000255850	0	0	0	0
ENSG00000255851	0	0	0	0
ENSG00000255853	0	0	0	0
ENSG00000255854	0	0	0	0
ENSG00000255855	0	0	0	0
ENSG00000255856	0	0	0	0
ENSG00000255857	4	0	0	0
ENSG00000255858	0	0	0	0
ENSG00000255860	0	0	0	0
ENSG00000255863	0	0	0	0
ENSG00000255864	0	0	0	0
ENSG00000255866	0	0	0	0
ENSG00000255867	0	0	0	0
ENSG00000255870	0	0	0	0
ENSG00000255871	0	0	0	0
ENSG00000255872	0	0	0	0
ENSG00000255874	43	45	79	43
ENSG00000255875	0	0	0	0
ENSG00000255882	0	0	0	0
ENSG00000255883	0	0	0	0
ENSG00000255885	0	0	0	0
ENSG00000255886	0	0	0	0
ENSG00000255892	0	0	0	0
ENSG00000255893	0	0	2	1
ENSG00000255896	0	0	2	2
ENSG00000255899	0	0	0	0
ENSG00000255900	0	0	0	0
ENSG00000255902	0	0	0	0
ENSG00000255903	0	0	0	0
ENSG00000255905	0	0	0	0
ENSG00000255906	1	0	0	0
ENSG00000255909	0	0	0	0
ENSG00000255910	0	0	3	0
ENSG00000255913	0	0	0	0
ENSG00000255915	0	0	0	0
ENSG00000255916	0	0	0	0
ENSG00000255919	0	0	0	0
ENSG00000255920	0	0	0	0
ENSG00000255921	0	0	0	4
ENSG00000255923	0	0	0	0
ENSG00000255927	0	0	0	0
ENSG00000255928	0	0	0	0
ENSG00000255929	0	1	1	0

ENSG00000255931	0	0	0	0
ENSG00000255933	0	0	0	0
ENSG00000255940	0	0	0	0
ENSG00000255944	0	0	0	0
ENSG00000255945	0	0	0	0
ENSG00000255946	0	0	0	0
ENSG00000255947	0	0	0	0
ENSG00000255949	0	0	1	5
ENSG00000255951	0	0	0	0
ENSG00000255958	0	0	0	0
ENSG00000255959	0	0	0	0
ENSG00000255960	0	0	0	0
ENSG00000255963	0	0	0	0
ENSG00000255964	0	0	0	0
ENSG00000255965	0	0	0	0
ENSG00000255966	1	0	3	0
ENSG00000255967	0	0	0	0
ENSG00000255968	0	0	0	0
ENSG00000255970	0	0	0	4
ENSG00000255972	0	0	0	0
ENSG00000255973	0	0	0	0
ENSG00000255974	0	0	0	3
ENSG00000255976	0	0	0	0
ENSG00000255977	0	0	0	0
ENSG00000255980	0	0	0	0
ENSG00000255982	0	0	0	0
ENSG00000255983	0	0	0	0
ENSG00000255986	0	0	0	0
ENSG00000255987	0	0	2	0
ENSG00000255988	0	0	0	0
ENSG00000255989	0	0	0	0
ENSG00000255990	0	0	0	0
ENSG00000255991	0	0	0	0
ENSG00000255992	0	0	0	0
ENSG00000255993	0	0	0	0
ENSG00000255994	0	0	0	0
ENSG00000255995	0	0	0	0
ENSG00000255996	0	0	0	0
ENSG00000255998	0	0	0	0
ENSG00000256001	0	0	1	0
ENSG00000256004	0	0	0	0
ENSG00000256005	0	0	0	0
ENSG00000256006	0	0	0	0
ENSG00000256007	0	0	1	1
ENSG00000256008	0	0	0	0
ENSG00000256010	0	0	0	0

ENSG00000256011	0	0	0	0
ENSG00000256013	0	0	0	0
ENSG00000256014	0	0	0	0
ENSG00000256018	0	0	0	0
ENSG00000256019	0	0	4	0
ENSG00000256020	0	0	0	0
ENSG00000256021	0	0	0	0
ENSG00000256022	0	0	0	1
ENSG00000256024	0	0	0	0
ENSG00000256025	0	0	0	0
ENSG00000256028	0	0	0	0
ENSG00000256029	0	0	0	0
ENSG00000256030	0	0	0	0
ENSG00000256031	0	0	0	0
ENSG00000256034	0	17	23	23
ENSG00000256035	0	0	0	0
ENSG00000256037	0	0	0	0
ENSG00000256039	3	0	0	0
ENSG00000256040	0	0	0	0
ENSG00000256041	0	0	0	0
ENSG00000256043	4	0	1	1
ENSG00000256044	0	0	0	0
ENSG00000256045	0	0	0	0
ENSG00000256049	0	0	0	0
ENSG00000256050	167	2851	265	2014
ENSG00000256053	0	0	1	0
ENSG00000256056	0	0	0	0
ENSG00000256060	23	21	18	6
ENSG00000256061	1	0	0	0
ENSG00000256062	0	0	0	0
ENSG00000256064	0	0	0	0
ENSG00000256066	0	0	0	0
ENSG00000256069	1	0	0	0
ENSG00000256070	0	0	0	0
ENSG00000256071	0	0	0	0
ENSG00000256072	0	0	1	0
ENSG00000256073	24	24	14	11
ENSG00000256075	0	0	0	0
ENSG00000256077	0	0	0	0
ENSG00000256079	0	0	0	0
ENSG00000256081	0	0	0	0
ENSG00000256083	0	0	0	0
ENSG00000256084	0	0	0	0
ENSG00000256085	0	0	0	0
ENSG00000256087	19	9	26	36
ENSG00000256091	0	0	0	0

ENSG00000256092	418	109	92	110
ENSG00000256093	0	0	0	0
ENSG00000256097	0	0	0	0
ENSG00000256098	0	0	0	0
ENSG00000256100	0	0	0	0
ENSG00000256101	4	0	0	1
ENSG00000256103	0	0	0	0
ENSG00000256108	0	0	0	0
ENSG00000256115	0	0	0	0
ENSG00000256116	0	0	0	1
ENSG00000256120	0	0	0	0
ENSG00000256121	0	0	0	0
ENSG00000256122	0	0	0	0
ENSG00000256124	0	0	0	0
ENSG00000256125	0	0	0	0
ENSG00000256128	11	0	0	0
ENSG00000256134	0	0	0	0
ENSG00000256136	0	0	0	0
ENSG00000256137	0	0	0	0
ENSG00000256138	0	0	0	0
ENSG00000256139	0	0	0	1
ENSG00000256142	0	0	0	0
ENSG00000256143	0	0	0	3
ENSG00000256146	0	0	0	0
ENSG00000256148	0	1	1	1
ENSG00000256149	0	0	0	0
ENSG00000256150	0	0	0	0
ENSG00000256151	0	0	0	0
ENSG00000256152	0	0	36	6
ENSG00000256155	0	0	0	0
ENSG00000256157	0	0	0	0
ENSG00000256159	0	0	0	0
ENSG00000256162	0	0	0	0
ENSG00000256164	0	0	0	0
ENSG00000256166	0	0	0	0
ENSG00000256167	0	0	0	10
ENSG00000256171	0	0	0	0
ENSG00000256172	0	0	0	0
ENSG00000256176	0	0	0	0
ENSG00000256178	0	0	0	0
ENSG00000256181	0	0	0	0
ENSG00000256182	0	0	0	0
ENSG00000256185	15	68	67	90
ENSG00000256186	0	0	0	0
ENSG00000256188	0	53	34	48
ENSG00000256189	0	0	0	0

ENSG00000256192	0	0	0	0
ENSG00000256193	0	0	11	3
ENSG00000256195	0	0	0	0
ENSG00000256196	0	0	0	0
ENSG00000256197	0	0	0	0
ENSG00000256199	0	0	0	0
ENSG00000256204	0	0	0	0
ENSG00000256206	0	0	0	0
ENSG00000256209	0	0	0	0
ENSG00000256210	0	0	1	0
ENSG00000256211	0	0	0	0
ENSG00000256212	0	0	0	0
ENSG00000256218	0	0	0	0
ENSG00000256220	0	0	0	0
ENSG00000256221	0	0	0	0
ENSG00000256222	0	0	0	0
ENSG00000256223	0	13	7	18
ENSG00000256225	0	0	0	0
ENSG00000256226	0	0	0	0
ENSG00000256229	3	15	31	58
ENSG00000256232	0	0	0	0
ENSG00000256234	0	0	4	2
ENSG00000256235	3	56	165	36
ENSG00000256237	0	0	0	0
ENSG00000256238	0	0	0	0
ENSG00000256239	0	0	0	0
ENSG00000256240	0	0	0	0
ENSG00000256243	0	0	0	0
ENSG00000256248	0	0	0	0
ENSG00000256249	0	0	0	0
ENSG00000256250	0	0	0	0
ENSG00000256252	0	0	0	0
ENSG00000256254	0	0	0	0
ENSG00000256256	0	0	0	0
ENSG00000256257	0	0	0	0
ENSG00000256258	0	0	0	0
ENSG00000256259	0	0	0	0
ENSG00000256261	0	0	0	0
ENSG00000256262	2	0	0	0
ENSG00000256263	2	86	54	98
ENSG00000256268	0	0	0	0
ENSG00000256269	7	0	3	0
ENSG00000256271	0	0	0	0
ENSG00000256273	0	0	0	0
ENSG00000256274	0	0	1	2
ENSG00000256276	0	0	0	0

ENSG00000256278	0	0	0	0
ENSG00000256280	0	0	0	0
ENSG00000256281	0	0	0	0
ENSG00000256282	0	0	0	0
ENSG00000256283	0	0	0	1
ENSG00000256285	0	0	0	0
ENSG00000256286	0	0	0	0
ENSG00000256287	0	0	0	0
ENSG00000256288	0	0	0	0
ENSG00000256292	0	0	0	0
ENSG00000256293	0	0	1	4
ENSG00000256294	0	0	2	1
ENSG00000256295	0	0	0	0
ENSG00000256298	0	0	0	0
ENSG00000256299	0	0	0	0
ENSG00000256301	0	0	0	0
ENSG00000256304	1	0	1	4
ENSG00000256305	0	0	0	0
ENSG00000256306	0	0	0	0
ENSG00000256309	0	0	0	0
ENSG00000256310	0	0	0	0
ENSG00000256311	0	0	0	0
ENSG00000256312	0	0	0	0
ENSG00000256314	0	0	0	0
ENSG00000256315	0	0	0	0
ENSG00000256316	7	81	22	61
ENSG00000256321	0	0	0	0
ENSG00000256323	0	0	0	0
ENSG00000256325	0	0	0	0
ENSG00000256329	0	0	0	0
ENSG00000256331	213	209	157	246
ENSG00000256338	11	18	3	6
ENSG00000256339	0	0	0	0
ENSG00000256340	0	0	0	1
ENSG00000256341	0	0	0	0
ENSG00000256342	0	0	0	0
ENSG00000256343	0	0	0	0
ENSG00000256344	0	0	0	0
ENSG00000256346	0	0	0	0
ENSG00000256347	0	0	0	0
ENSG00000256349	0	0	0	0
ENSG00000256350	0	0	0	0
ENSG00000256351	0	0	0	0
ENSG00000256352	0	0	0	0
ENSG00000256353	0	0	0	0
ENSG00000256354	0	0	0	0

ENSG00000256355	0	0	0	0
ENSG00000256356	0	0	0	0
ENSG00000256357	0	0	0	0
ENSG00000256358	0	0	0	0
ENSG00000256360	0	0	0	0
ENSG00000256361	0	0	0	0
ENSG00000256362	0	0	0	0
ENSG00000256364	0	0	18	22
ENSG00000256371	0	0	0	0
ENSG00000256372	0	0	0	0
ENSG00000256373	0	0	0	0
ENSG00000256374	0	0	0	0
ENSG00000256377	1	0	2	0
ENSG00000256378	0	0	0	0
ENSG00000256379	0	0	2	0
ENSG00000256381	0	0	0	0
ENSG00000256383	0	15	0	0
ENSG00000256385	0	0	0	0
ENSG00000256386	0	0	0	0
ENSG00000256389	0	0	0	0
ENSG00000256390	0	0	0	0
ENSG00000256391	0	0	0	0
ENSG00000256392	1	0	0	0
ENSG00000256393	0	0	0	0
ENSG00000256394	0	0	0	0
ENSG00000256399	0	0	0	0
ENSG00000256400	0	0	0	0
ENSG00000256403	0	0	0	0
ENSG00000256404	0	0	0	0
ENSG00000256407	0	0	0	0
ENSG00000256417	0	0	1	0
ENSG00000256419	0	0	0	0
ENSG00000256420	0	0	0	0
ENSG00000256422	0	0	0	0
ENSG00000256424	0	0	0	0
ENSG00000256425	0	0	0	0
ENSG00000256427	0	0	0	2
ENSG00000256428	0	0	0	0
ENSG00000256429	0	0	0	0
ENSG00000256433	0	0	0	0
ENSG00000256436	1	1	14	9
ENSG00000256439	0	0	0	0
ENSG00000256440	0	0	0	0
ENSG00000256441	0	0	0	0
ENSG00000256442	1	0	0	0
ENSG00000256443	0	0	0	0

ENSG00000256448	0	3	0	0
ENSG00000256450	0	0	0	0
ENSG00000256452	0	0	0	0
ENSG00000256453	0	0	0	0
ENSG00000256456	0	0	0	0
ENSG00000256458	0	3	0	7
ENSG00000256462	0	0	0	0
ENSG00000256463	0	0	0	0
ENSG00000256464	0	0	2	13
ENSG00000256465	0	0	0	0
ENSG00000256466	0	0	0	0
ENSG00000256469	0	0	2	0
ENSG00000256473	0	0	0	0
ENSG00000256474	0	0	0	0
ENSG00000256480	0	0	0	0
ENSG00000256481	0	0	0	0
ENSG00000256482	0	0	3	0
ENSG00000256484	0	0	0	0
ENSG00000256490	0	0	0	0
ENSG00000256492	0	0	0	0
ENSG00000256494	0	0	0	0
ENSG00000256496	0	0	0	0
ENSG00000256498	0	0	0	0
ENSG00000256499	0	0	0	0
ENSG00000256500	0	0	0	0
ENSG00000256502	0	0	0	0
ENSG00000256504	0	0	0	0
ENSG00000256508	0	0	0	0
ENSG00000256512	0	0	1	0
ENSG00000256513	0	0	0	0
ENSG00000256514	0	0	0	0
ENSG00000256515	123	0	0	23
ENSG00000256518	0	0	0	0
ENSG00000256521	0	0	0	0
ENSG00000256525	3	8	114	174
ENSG00000256527	0	0	0	0
ENSG00000256528	0	0	0	0
ENSG00000256530	0	0	0	0
ENSG00000256533	0	0	0	0
ENSG00000256537	0	50	29	31
ENSG00000256538	1	0	3	12
ENSG00000256540	0	0	0	0
ENSG00000256542	0	0	0	0
ENSG00000256545	0	0	0	0
ENSG00000256546	1	10	0	2
ENSG00000256551	0	0	0	0

ENSG00000256552	0	0	0	0
ENSG00000256553	0	0	0	0
ENSG00000256557	0	0	0	0
ENSG00000256560	0	0	0	0
ENSG00000256563	0	0	0	0
ENSG00000256564	0	0	0	0
ENSG00000256566	0	0	0	0
ENSG00000256568	0	0	0	0
ENSG00000256569	0	0	0	1
ENSG00000256571	0	0	0	0
ENSG00000256574	0	0	0	0
ENSG00000256576	0	0	0	0
ENSG00000256577	0	0	0	0
ENSG00000256579	0	0	0	0
ENSG00000256581	0	0	0	0
ENSG00000256582	0	0	5	2
ENSG00000256586	0	0	0	0
ENSG00000256588	0	0	0	0
ENSG00000256589	0	0	0	0
ENSG00000256590	0	0	0	0
ENSG00000256591	1	0	4	0
ENSG00000256594	12	53	13	23
ENSG00000256596	0	0	0	0
ENSG00000256597	0	0	0	3
ENSG00000256599	0	0	0	0
ENSG00000256603	0	0	0	0
ENSG00000256604	0	8	1	0
ENSG00000256609	0	0	0	0
ENSG00000256612	0	12	5	3
ENSG00000256614	0	0	0	0
ENSG00000256615	0	0	0	0
ENSG00000256616	0	0	0	0
ENSG00000256618	32	2	4	0
ENSG00000256619	0	0	0	0
ENSG00000256625	0	0	16	36
ENSG00000256626	0	0	0	0
ENSG00000256627	0	0	0	0
ENSG00000256628	0	42	13	17
ENSG00000256629	0	0	0	0
ENSG00000256630	0	0	0	0
ENSG00000256631	0	0	0	0
ENSG00000256632	0	0	0	0
ENSG00000256633	3	17	5	3
ENSG00000256637	0	0	0	0
ENSG00000256640	0	0	0	0
ENSG00000256642	0	0	0	0

ENSG00000256643	0	0	0	0
ENSG00000256646	0	0	1	0
ENSG00000256650	0	0	0	0
ENSG00000256651	3	0	2	6
ENSG00000256654	0	0	0	0
ENSG00000256655	0	0	0	0
ENSG00000256657	0	0	0	0
ENSG00000256658	0	0	0	0
ENSG00000256659	0	0	0	0
ENSG00000256660	2	77	16	12
ENSG00000256661	0	0	0	0
ENSG00000256663	0	0	0	0
ENSG00000256664	0	0	0	0
ENSG00000256667	10	77	244	198
ENSG00000256670	0	0	0	0
ENSG00000256671	0	0	0	0
ENSG00000256672	13	135	26	27
ENSG00000256673	9	15	14	20
ENSG00000256674	0	0	0	0
ENSG00000256678	0	0	0	0
ENSG00000256681	0	0	0	0
ENSG00000256682	0	0	0	0
ENSG00000256683	10	298	184	308
ENSG00000256684	0	0	0	0
ENSG00000256686	0	0	0	0
ENSG00000256690	0	0	0	1
ENSG00000256691	0	0	0	0
ENSG00000256692	0	0	0	0
ENSG00000256694	0	0	0	0
ENSG00000256695	0	0	0	0
ENSG00000256699	0	0	0	0
ENSG00000256703	0	0	0	0
ENSG00000256704	0	0	0	0
ENSG00000256705	1	0	0	0
ENSG00000256706	0	0	0	0
ENSG00000256708	0	0	0	0
ENSG00000256709	0	0	0	0
ENSG00000256712	0	0	1	0
ENSG00000256713	0	0	0	0
ENSG00000256714	0	0	0	0
ENSG00000256715	0	0	0	0
ENSG00000256717	0	0	0	0
ENSG00000256720	0	0	0	0
ENSG00000256721	0	0	0	0
ENSG00000256723	0	0	0	0
ENSG00000256725	0	0	0	0

ENSG00000256731	0	0	1	0
ENSG00000256732	0	0	0	0
ENSG00000256733	0	0	0	0
ENSG00000256734	13	0	2	0
ENSG00000256737	0	0	0	0
ENSG00000256739	0	0	0	0
ENSG00000256742	0	16	2	0
ENSG00000256745	1	0	0	0
ENSG00000256746	1	0	0	0
ENSG00000256747	0	0	0	0
ENSG00000256748	0	0	0	0
ENSG00000256750	0	0	0	0
ENSG00000256751	0	0	0	3
ENSG00000256752	0	0	0	0
ENSG00000256756	0	0	0	0
ENSG00000256757	0	0	0	0
ENSG00000256761	0	0	0	0
ENSG00000256762	0	0	0	0
ENSG00000256763	0	0	0	0
ENSG00000256769	0	0	0	0
ENSG00000256771	0	41	38	44
ENSG00000256774	0	0	0	0
ENSG00000256777	0	0	1	0
ENSG00000256779	0	0	0	0
ENSG00000256783	0	0	0	0
ENSG00000256786	0	0	0	0
ENSG00000256789	0	45	22	3
ENSG00000256790	0	0	0	0
ENSG00000256797	1	0	0	0
ENSG00000256799	0	0	0	0
ENSG00000256802	0	0	0	0
ENSG00000256803	0	1	0	0
ENSG00000256804	0	0	0	0
ENSG00000256806	0	0	0	0
ENSG00000256810	0	0	0	0
ENSG00000256811	0	0	0	0
ENSG00000256812	0	0	0	0
ENSG00000256813	0	0	0	0
ENSG00000256814	0	0	0	0
ENSG00000256815	0	0	0	0
ENSG00000256817	0	0	0	0
ENSG00000256818	0	0	0	0
ENSG00000256822	0	0	0	0
ENSG00000256824	0	0	0	0
ENSG00000256825	0	0	0	0
ENSG00000256826	0	0	0	0

ENSG00000256827	0	0	0	0
ENSG00000256835	0	0	0	0
ENSG00000256837	0	0	0	0
ENSG00000256840	0	0	0	0
ENSG00000256843	0	0	0	0
ENSG00000256844	0	0	0	0
ENSG00000256845	0	0	0	0
ENSG00000256847	0	0	0	0
ENSG00000256849	0	0	0	0
ENSG00000256850	0	0	0	0
ENSG00000256851	0	0	0	0
ENSG00000256852	0	0	0	0
ENSG00000256861	0	0	0	0
ENSG00000256862	1	0	0	0
ENSG00000256863	0	0	0	0
ENSG00000256868	0	0	0	0
ENSG00000256870	0	0	0	0
ENSG00000256872	1	0	0	0
ENSG00000256873	0	0	0	0
ENSG00000256874	0	0	0	0
ENSG00000256875	0	0	0	0
ENSG00000256878	0	0	0	0
ENSG00000256879	0	3	16	15
ENSG00000256882	0	0	0	0
ENSG00000256884	0	0	0	0
ENSG00000256885	0	0	0	0
ENSG00000256888	0	0	0	0
ENSG00000256889	0	0	0	0
ENSG00000256892	0	0	0	0
ENSG00000256894	0	0	0	0
ENSG00000256895	0	0	0	0
ENSG00000256896	0	0	0	0
ENSG00000256897	0	0	0	0
ENSG00000256898	0	0	0	0
ENSG00000256902	0	0	0	0
ENSG00000256904	8	0	0	0
ENSG00000256905	0	0	0	0
ENSG00000256906	0	0	0	0
ENSG00000256912	1	0	0	0
ENSG00000256913	0	0	0	0
ENSG00000256914	0	0	0	0
ENSG00000256915	0	0	0	0
ENSG00000256916	3	0	1	0
ENSG00000256917	0	0	0	0
ENSG00000256920	0	0	0	0
ENSG00000256922	0	0	0	6

ENSG00000256923	0	0	0	0
ENSG00000256925	0	0	0	0
ENSG00000256927	0	0	0	0
ENSG00000256928	0	0	0	0
ENSG00000256929	0	0	0	0
ENSG00000256937	0	0	0	0
ENSG00000256940	0	0	9	14
ENSG00000256943	0	0	0	0
ENSG00000256944	0	0	0	0
ENSG00000256947	0	0	1	0
ENSG00000256948	0	0	0	0
ENSG00000256950	0	0	0	0
ENSG00000256951	0	0	0	0
ENSG00000256953	0	0	0	2
ENSG00000256955	0	0	0	0
ENSG00000256956	0	0	0	0
ENSG00000256963	0	0	0	0
ENSG00000256966	0	0	0	0
ENSG00000256967	0	0	0	0
ENSG00000256968	0	0	0	0
ENSG00000256969	0	0	0	0
ENSG00000256971	0	0	0	15
ENSG00000256972	0	0	0	0
ENSG00000256973	0	0	0	0
ENSG00000256974	0	0	0	0
ENSG00000256975	0	0	0	0
ENSG00000256977	0	0	0	0
ENSG00000256980	0	0	0	0
ENSG00000256981	0	0	0	5
ENSG00000256982	0	0	0	0
ENSG00000256984	0	0	0	0
ENSG00000256986	0	0	0	0
ENSG00000256987	0	0	0	0
ENSG00000256988	0	0	0	0
ENSG00000256989	0	0	0	0
ENSG00000256994	0	0	0	0
ENSG00000256995	0	0	3	0
ENSG00000257000	0	0	0	0
ENSG00000257002	0	0	0	0
ENSG00000257004	0	0	0	0
ENSG00000257005	0	0	0	0
ENSG00000257008	0	0	0	0
ENSG00000257009	0	0	0	0
ENSG00000257010	0	0	0	0
ENSG00000257012	0	0	0	0
ENSG00000257016	0	0	0	0

ENSG00000257017	0	0	0	1
ENSG00000257019	0	0	0	0
ENSG00000257020	0	0	0	0
ENSG00000257021	0	0	0	0
ENSG00000257022	0	0	0	0
ENSG00000257023	0	0	0	0
ENSG00000257025	0	0	0	0
ENSG00000257026	0	0	0	0
ENSG00000257027	7	22	0	6
ENSG00000257028	0	0	0	0
ENSG00000257033	0	0	0	0
ENSG00000257034	0	0	0	0
ENSG00000257035	0	0	0	0
ENSG00000257037	0	0	0	0
ENSG00000257038	0	0	0	0
ENSG00000257040	0	0	0	0
ENSG00000257042	0	0	0	0
ENSG00000257043	1	0	0	0
ENSG00000257045	0	0	0	0
ENSG00000257046	0	0	0	0
ENSG00000257048	0	0	0	0
ENSG00000257052	0	0	0	0
ENSG00000257053	0	0	0	0
ENSG00000257056	0	0	0	0
ENSG00000257057	0	0	0	0
ENSG00000257058	0	0	0	0
ENSG00000257060	0	0	0	0
ENSG00000257061	0	0	0	0
ENSG00000257062	0	0	0	0
ENSG00000257065	0	0	0	0
ENSG00000257067	0	0	0	0
ENSG00000257069	0	0	0	0
ENSG00000257070	0	0	0	0
ENSG00000257072	0	0	0	0
ENSG00000257073	0	0	0	0
ENSG00000257074	0	0	0	0
ENSG00000257075	0	0	0	0
ENSG00000257078	0	0	0	0
ENSG00000257083	0	0	0	0
ENSG00000257084	0	0	0	0
ENSG00000257086	0	18	2	11
ENSG00000257087	0	0	0	0
ENSG00000257088	0	0	0	0
ENSG00000257093	73	113	453	188
ENSG00000257094	0	0	0	0
ENSG00000257095	0	0	0	0

ENSG00000257096	0	0	1	0
ENSG00000257097	0	0	1	1
ENSG00000257101	0	0	0	0
ENSG00000257103	569	2417	1927	2631
ENSG00000257105	0	0	0	0
ENSG00000257108	0	0	0	0
ENSG00000257109	0	0	0	0
ENSG00000257110	0	0	0	0
ENSG00000257113	0	0	0	0
ENSG00000257114	0	0	0	0
ENSG00000257115	0	0	0	0
ENSG00000257119	0	0	0	0
ENSG00000257120	0	0	0	0
ENSG00000257121	2	0	0	0
ENSG00000257122	92	112	76	125
ENSG00000257123	0	0	0	0
ENSG00000257124	0	0	0	0
ENSG00000257125	0	0	0	0
ENSG00000257126	0	0	0	0
ENSG00000257127	0	0	0	0
ENSG00000257128	0	0	0	0
ENSG00000257129	0	0	0	0
ENSG00000257135	30	34	78	73
ENSG00000257137	0	0	0	0
ENSG00000257138	0	0	0	0
ENSG00000257139	0	0	0	0
ENSG00000257141	0	0	0	0
ENSG00000257142	0	0	0	0
ENSG00000257146	0	0	0	0
ENSG00000257147	0	0	0	0
ENSG00000257148	0	0	0	0
ENSG00000257149	0	0	0	0
ENSG00000257150	0	0	0	0
ENSG00000257151	2	0	8	11
ENSG00000257152	0	0	0	0
ENSG00000257153	0	0	0	0
ENSG00000257154	0	0	0	0
ENSG00000257155	0	0	0	0
ENSG00000257156	0	0	2	3
ENSG00000257157	0	0	0	0
ENSG00000257158	0	0	0	0
ENSG00000257159	0	0	0	0
ENSG00000257160	0	0	0	0
ENSG00000257162	0	0	0	0
ENSG00000257164	0	0	0	0
ENSG00000257165	0	0	0	0

ENSG00000257167	47	23	22	20
ENSG00000257168	0	0	0	0
ENSG00000257169	0	0	3	2
ENSG00000257170	0	0	0	0
ENSG00000257171	0	0	0	0
ENSG00000257172	0	0	0	0
ENSG00000257173	0	0	0	0
ENSG00000257175	0	0	0	2
ENSG00000257176	2	112	55	51
ENSG00000257177	0	0	0	0
ENSG00000257178	0	0	0	0
ENSG00000257179	0	0	0	0
ENSG00000257180	0	0	0	0
ENSG00000257181	0	17	30	0
ENSG00000257182	0	0	0	0
ENSG00000257183	0	0	0	0
ENSG00000257184	0	0	0	0
ENSG00000257185	0	0	0	0
ENSG00000257187	0	0	0	0
ENSG00000257188	0	0	0	0
ENSG00000257189	0	0	0	0
ENSG00000257190	0	0	0	0
ENSG00000257191	0	0	0	0
ENSG00000257192	0	0	0	0
ENSG00000257193	0	0	0	0
ENSG00000257194	0	0	0	0
ENSG00000257195	0	0	0	0
ENSG00000257196	0	23	0	4
ENSG00000257198	0	0	0	0
ENSG00000257199	0	0	0	0
ENSG00000257202	0	0	0	0
ENSG00000257206	0	0	0	0
ENSG00000257207	0	0	2	1
ENSG00000257210	1	0	0	0
ENSG00000257211	0	0	0	0
ENSG00000257212	0	0	0	0
ENSG00000257213	0	0	0	0
ENSG00000257215	0	0	0	0
ENSG00000257216	0	0	0	0
ENSG00000257217	0	0	0	0
ENSG00000257218	8	0	2	5
ENSG00000257219	0	0	0	0
ENSG00000257220	0	0	0	0
ENSG00000257221	0	0	0	0
ENSG00000257222	0	0	2	0
ENSG00000257223	0	0	0	0

ENSG00000257224	0	0	0	0
ENSG00000257225	0	27	1	0
ENSG00000257226	0	0	0	0
ENSG00000257227	0	0	0	0
ENSG00000257228	0	0	0	0
ENSG00000257230	0	0	0	0
ENSG00000257231	0	0	0	1
ENSG00000257232	0	0	0	0
ENSG00000257233	0	0	0	0
ENSG00000257235	0	0	0	0
ENSG00000257236	0	0	0	0
ENSG00000257237	0	0	1	0
ENSG00000257238	0	0	0	0
ENSG00000257239	0	0	5	6
ENSG00000257240	0	0	0	0
ENSG00000257241	0	0	0	0
ENSG00000257242	1	0	0	0
ENSG00000257243	0	0	0	0
ENSG00000257245	0	0	0	0
ENSG00000257246	0	0	0	9
ENSG00000257247	0	0	0	0
ENSG00000257248	0	0	0	0
ENSG00000257249	0	0	0	0
ENSG00000257251	0	0	0	0
ENSG00000257252	1	16	2	0
ENSG00000257253	0	0	0	0
ENSG00000257254	0	0	0	0
ENSG00000257255	0	0	0	0
ENSG00000257256	0	0	0	0
ENSG00000257258	0	0	4	7
ENSG00000257259	0	0	0	0
ENSG00000257260	0	0	0	0
ENSG00000257261	346	596	420	1065
ENSG00000257262	0	0	0	0
ENSG00000257263	0	0	0	0
ENSG00000257264	0	0	0	7
ENSG00000257265	0	0	0	0
ENSG00000257266	0	0	0	0
ENSG00000257267	4975	8836	9304	7970
ENSG00000257268	0	0	0	0
ENSG00000257270	0	0	0	0
ENSG00000257271	0	0	0	2
ENSG00000257272	0	0	0	0
ENSG00000257273	0	0	0	0
ENSG00000257275	3	0	1	0
ENSG00000257276	0	0	0	0

ENSG00000257277	0	0	0	0
ENSG00000257278	0	0	0	0
ENSG00000257279	3	0	0	6
ENSG00000257281	0	0	0	0
ENSG00000257283	0	0	0	0
ENSG00000257284	0	0	0	0
ENSG00000257285	0	0	1	1
ENSG00000257286	0	0	0	0
ENSG00000257287	0	0	0	0
ENSG00000257288	0	0	0	0
ENSG00000257289	0	0	0	3
ENSG00000257292	0	0	0	0
ENSG00000257294	0	0	1	0
ENSG00000257295	0	0	0	0
ENSG00000257296	0	0	0	0
ENSG00000257298	0	0	0	0
ENSG00000257299	0	0	0	0
ENSG00000257300	0	24	0	0
ENSG00000257301	0	0	0	0
ENSG00000257302	0	0	0	0
ENSG00000257303	0	0	0	0
ENSG00000257305	0	0	0	0
ENSG00000257307	0	0	0	0
ENSG00000257308	0	0	0	0
ENSG00000257309	0	0	0	0
ENSG00000257310	0	0	0	0
ENSG00000257311	0	0	0	0
ENSG00000257313	0	0	0	0
ENSG00000257314	0	0	0	0
ENSG00000257315	72	1183	1224	672
ENSG00000257316	0	0	0	0
ENSG00000257318	0	0	0	0
ENSG00000257319	0	0	0	0
ENSG00000257320	0	0	0	0
ENSG00000257321	0	0	0	0
ENSG00000257322	2	0	0	0
ENSG00000257323	0	0	0	0
ENSG00000257324	0	0	0	0
ENSG00000257325	0	0	0	0
ENSG00000257326	0	0	0	0
ENSG00000257327	1	0	0	9
ENSG00000257328	0	0	0	0
ENSG00000257329	0	0	0	0
ENSG00000257331	0	0	0	0
ENSG00000257332	0	0	0	0
ENSG00000257333	0	0	0	0

ENSG00000257334	0	0	0	0
ENSG00000257335	3	0	1	0
ENSG00000257336	0	0	0	0
ENSG00000257337	0	0	12	13
ENSG00000257338	0	0	0	0
ENSG00000257339	0	0	0	0
ENSG00000257340	0	0	0	0
ENSG00000257341	0	0	0	0
ENSG00000257342	0	0	6	11
ENSG00000257343	0	0	0	0
ENSG00000257345	0	0	0	0
ENSG00000257346	0	0	0	0
ENSG00000257348	0	0	0	0
ENSG00000257350	0	0	0	0
ENSG00000257351	0	0	0	0
ENSG00000257354	5	22	39	29
ENSG00000257355	0	0	0	0
ENSG00000257356	0	0	0	0
ENSG00000257357	0	0	0	0
ENSG00000257359	1	0	0	0
ENSG00000257360	0	0	0	0
ENSG00000257361	0	0	0	0
ENSG00000257362	0	0	0	0
ENSG00000257363	0	0	0	0
ENSG00000257364	0	0	0	0
ENSG00000257365	1	0	14	21
ENSG00000257366	0	0	0	0
ENSG00000257368	1	0	0	0
ENSG00000257369	0	0	0	0
ENSG00000257373	0	0	0	0
ENSG00000257374	0	0	0	0
ENSG00000257376	0	0	0	0
ENSG00000257377	0	0	0	0
ENSG00000257378	0	0	0	0
ENSG00000257379	0	0	0	0
ENSG00000257381	0	0	0	0
ENSG00000257382	0	0	0	0
ENSG00000257383	0	0	0	0
ENSG00000257384	0	0	0	0
ENSG00000257386	0	0	13	16
ENSG00000257389	0	0	0	0
ENSG00000257390	0	0	1	3
ENSG00000257391	0	0	2	0
ENSG00000257392	0	0	0	0
ENSG00000257393	0	0	0	0
ENSG00000257394	0	0	0	0

ENSG00000257395	0	0	0	0
ENSG00000257396	0	0	0	0
ENSG00000257398	0	0	0	0
ENSG00000257399	0	0	0	0
ENSG00000257400	0	0	0	0
ENSG00000257402	0	0	0	0
ENSG00000257403	0	308	156	250
ENSG00000257404	0	0	0	0
ENSG00000257405	0	0	0	0
ENSG00000257407	0	0	0	0
ENSG00000257408	0	0	0	0
ENSG00000257410	1	0	0	0
ENSG00000257411	0	0	0	0
ENSG00000257412	0	0	0	0
ENSG00000257413	0	0	0	0
ENSG00000257414	0	0	0	0
ENSG00000257415	0	0	0	0
ENSG00000257416	0	0	0	0
ENSG00000257418	0	0	0	0
ENSG00000257420	0	0	0	0
ENSG00000257421	0	0	0	0
ENSG00000257423	0	0	0	0
ENSG00000257424	0	1	0	0
ENSG00000257425	0	0	0	0
ENSG00000257426	0	0	0	0
ENSG00000257427	0	0	0	0
ENSG00000257428	0	0	0	0
ENSG00000257429	0	0	0	0
ENSG00000257431	0	0	0	0
ENSG00000257432	0	0	0	0
ENSG00000257433	28	47	21	64
ENSG00000257434	0	0	0	0
ENSG00000257435	0	0	0	0
ENSG00000257436	0	0	0	0
ENSG00000257437	0	0	0	0
ENSG00000257438	0	0	0	0
ENSG00000257440	0	0	0	0
ENSG00000257443	0	0	0	0
ENSG00000257444	0	0	0	0
ENSG00000257446	0	0	1	0
ENSG00000257449	0	0	0	0
ENSG00000257451	0	0	0	0
ENSG00000257452	0	0	0	0
ENSG00000257453	0	0	0	0
ENSG00000257454	0	0	0	0
ENSG00000257456	0	0	0	0

ENSG00000257457	0	0	0	0
ENSG00000257458	0	0	0	0
ENSG00000257461	0	0	0	0
ENSG00000257464	0	0	0	0
ENSG00000257465	0	0	0	0
ENSG00000257467	0	0	0	0
ENSG00000257468	0	0	0	0
ENSG00000257469	0	0	0	0
ENSG00000257470	0	0	0	0
ENSG00000257472	0	0	0	0
ENSG00000257473	0	0	0	0
ENSG00000257474	0	0	0	0
ENSG00000257475	0	0	0	0
ENSG00000257476	0	0	0	0
ENSG00000257477	0	0	0	0
ENSG00000257478	0	0	0	0
ENSG00000257480	0	0	0	0
ENSG00000257482	0	0	0	0
ENSG00000257485	0	0	0	0
ENSG00000257488	0	0	0	0
ENSG00000257489	0	1	2	10
ENSG00000257493	0	0	0	0
ENSG00000257494	0	0	2	0
ENSG00000257495	0	0	0	0
ENSG00000257496	0	0	0	0
ENSG00000257497	13	0	3	0
ENSG00000257499	4	0	0	0
ENSG00000257500	0	0	0	0
ENSG00000257501	0	0	0	0
ENSG00000257502	0	0	0	0
ENSG00000257503	0	0	0	0
ENSG00000257504	0	0	0	0
ENSG00000257506	0	0	0	0
ENSG00000257507	0	0	10	14
ENSG00000257509	0	0	0	0
ENSG00000257510	0	0	0	0
ENSG00000257511	3	0	3	3
ENSG00000257512	1	0	0	0
ENSG00000257513	0	0	0	0
ENSG00000257514	0	0	0	0
ENSG00000257515	0	0	0	0
ENSG00000257516	0	0	0	0
ENSG00000257517	0	0	0	0
ENSG00000257519	0	0	0	0
ENSG00000257520	0	0	0	0
ENSG00000257522	0	0	0	0

ENSG00000257523	0	0	0	0
ENSG00000257524	0	0	0	0
ENSG00000257526	0	0	2	0
ENSG00000257527	0	0	0	0
ENSG00000257528	0	0	0	0
ENSG00000257529	0	0	0	0
ENSG00000257530	0	24	6	1
ENSG00000257531	0	0	0	0
ENSG00000257534	0	0	0	0
ENSG00000257535	0	0	0	0
ENSG00000257536	0	0	0	0
ENSG00000257537	0	0	0	0
ENSG00000257538	0	0	0	0
ENSG00000257539	1	0	0	0
ENSG00000257541	0	0	0	0
ENSG00000257542	0	0	0	0
ENSG00000257543	0	0	0	0
ENSG00000257544	0	0	0	0
ENSG00000257545	0	0	0	0
ENSG00000257548	0	0	0	0
ENSG00000257549	0	0	0	0
ENSG00000257550	0	9	4	0
ENSG00000257551	0	0	0	0
ENSG00000257553	0	1	0	1
ENSG00000257554	0	0	0	0
ENSG00000257556	0	0	0	0
ENSG00000257557	30	34	49	38
ENSG00000257558	0	0	0	0
ENSG00000257559	0	0	0	0
ENSG00000257560	0	0	0	0
ENSG00000257561	0	0	0	0
ENSG00000257562	0	0	0	0
ENSG00000257563	0	0	0	0
ENSG00000257564	0	0	0	0
ENSG00000257565	0	0	0	0
ENSG00000257568	0	0	0	0
ENSG00000257569	0	0	0	0
ENSG00000257570	0	0	0	0
ENSG00000257571	0	0	0	0
ENSG00000257572	0	0	0	0
ENSG00000257573	0	0	0	1
ENSG00000257575	0	0	0	0
ENSG00000257576	0	0	0	0
ENSG00000257578	0	0	0	0
ENSG00000257579	0	0	0	0
ENSG00000257580	0	0	0	0

ENSG00000257582	0	0	0	0
ENSG00000257583	0	0	0	0
ENSG00000257585	0	0	0	0
ENSG00000257586	0	0	0	0
ENSG00000257587	0	0	0	0
ENSG00000257588	0	0	0	0
ENSG00000257589	0	0	0	0
ENSG00000257590	0	0	0	0
ENSG00000257591	0	0	1	4
ENSG00000257593	0	0	0	0
ENSG00000257594	0	0	7	0
ENSG00000257595	0	0	0	0
ENSG00000257596	0	0	0	0
ENSG00000257599	0	66	15	31
ENSG00000257600	0	0	0	0
ENSG00000257601	0	0	0	0
ENSG00000257602	0	0	0	0
ENSG00000257603	0	0	0	0
ENSG00000257604	0	0	0	0
ENSG00000257605	2	0	1	0
ENSG00000257607	0	0	0	0
ENSG00000257608	1	0	0	0
ENSG00000257609	0	0	0	0
ENSG00000257611	0	0	0	0
ENSG00000257612	0	0	0	0
ENSG00000257613	3	63	38	109
ENSG00000257614	0	0	0	0
ENSG00000257615	0	0	0	0
ENSG00000257616	0	0	0	0
ENSG00000257618	0	0	0	0
ENSG00000257621	271	936	1048	869
ENSG00000257622	0	0	0	0
ENSG00000257624	0	0	0	0
ENSG00000257626	0	0	0	0
ENSG00000257629	0	0	0	0
ENSG00000257630	0	0	0	0
ENSG00000257632	0	0	0	0
ENSG00000257634	0	0	0	0
ENSG00000257635	0	0	0	0
ENSG00000257636	0	0	0	0
ENSG00000257637	0	0	0	0
ENSG00000257638	0	0	0	0
ENSG00000257639	0	20	8	28
ENSG00000257640	0	0	0	0
ENSG00000257642	0	0	0	0
ENSG00000257643	0	0	0	0

ENSG00000257644	0	0	0	0
ENSG00000257645	0	0	0	0
ENSG00000257646	0	0	0	0
ENSG00000257647	0	0	0	0
ENSG00000257648	0	0	0	0
ENSG00000257649	0	0	0	0
ENSG00000257651	0	0	0	0
ENSG00000257652	0	0	0	0
ENSG00000257653	0	0	3	2
ENSG00000257654	0	0	0	0
ENSG00000257656	0	0	0	0
ENSG00000257657	0	0	1	0
ENSG00000257658	0	0	0	0
ENSG00000257659	0	0	0	0
ENSG00000257660	5	0	3	6
ENSG00000257661	0	0	0	0
ENSG00000257662	0	0	0	0
ENSG00000257663	0	0	0	0
ENSG00000257664	0	0	0	0
ENSG00000257665	0	0	0	0
ENSG00000257666	0	0	0	0
ENSG00000257668	0	0	0	0
ENSG00000257670	0	0	0	0
ENSG00000257671	0	0	0	0
ENSG00000257672	0	0	0	0
ENSG00000257674	0	0	0	0
ENSG00000257675	0	0	0	0
ENSG00000257677	0	0	0	0
ENSG00000257679	0	0	0	0
ENSG00000257680	0	0	0	0
ENSG00000257681	0	0	1	0
ENSG00000257682	0	0	0	0
ENSG00000257683	0	0	0	0
ENSG00000257686	0	0	0	0
ENSG00000257687	0	0	0	0
ENSG00000257690	0	0	0	0
ENSG00000257691	0	0	0	0
ENSG00000257692	0	0	0	0
ENSG00000257693	0	0	0	0
ENSG00000257696	0	0	0	0
ENSG00000257697	0	0	0	0
ENSG00000257698	7	0	2	7
ENSG00000257700	0	0	0	0
ENSG00000257701	0	0	0	0
ENSG00000257702	0	16	1	9
ENSG00000257703	0	0	0	0

ENSG00000257704	17	0	5	7
ENSG00000257705	0	0	0	0
ENSG00000257707	0	0	0	0
ENSG00000257709	0	0	0	0
ENSG00000257711	1	0	0	0
ENSG00000257712	0	0	0	0
ENSG00000257713	0	0	0	0
ENSG00000257715	0	0	1	2
ENSG00000257717	0	0	0	0
ENSG00000257718	1	0	4	1
ENSG00000257720	0	0	0	0
ENSG00000257721	0	0	0	0
ENSG00000257723	0	0	0	0
ENSG00000257725	0	0	0	0
ENSG00000257726	0	0	0	0
ENSG00000257727	0	0	2	8
ENSG00000257729	0	0	0	0
ENSG00000257730	0	0	0	0
ENSG00000257731	0	0	0	0
ENSG00000257732	0	0	0	0
ENSG00000257733	0	0	0	0
ENSG00000257735	0	17	0	0
ENSG00000257736	0	0	0	0
ENSG00000257737	0	0	0	0
ENSG00000257738	0	0	0	0
ENSG00000257739	0	0	0	0
ENSG00000257740	3	0	0	0
ENSG00000257741	0	0	0	0
ENSG00000257742	4	4	13	12
ENSG00000257743	2	0	5	7
ENSG00000257744	0	0	0	0
ENSG00000257745	0	0	0	0
ENSG00000257746	0	0	0	0
ENSG00000257747	0	0	0	0
ENSG00000257748	0	0	0	0
ENSG00000257749	0	0	2	0
ENSG00000257750	0	0	0	0
ENSG00000257751	0	0	0	0
ENSG00000257752	0	0	0	0
ENSG00000257754	0	0	0	0
ENSG00000257756	0	0	0	0
ENSG00000257757	0	0	0	0
ENSG00000257758	0	0	0	0
ENSG00000257759	0	0	0	0
ENSG00000257760	0	0	0	0
ENSG00000257761	0	0	0	0

ENSG00000257762	3	0	0	0
ENSG00000257763	0	0	0	2
ENSG00000257764	0	0	6	7
ENSG00000257766	0	0	0	0
ENSG00000257767	0	0	0	0
ENSG00000257769	0	0	0	0
ENSG00000257770	0	0	0	0
ENSG00000257771	4	0	0	0
ENSG00000257773	0	0	0	0
ENSG00000257774	0	0	0	0
ENSG00000257775	0	0	0	0
ENSG00000257777	0	0	0	0
ENSG00000257778	0	0	0	0
ENSG00000257779	0	0	0	0
ENSG00000257780	0	0	0	0
ENSG00000257781	0	0	0	0
ENSG00000257782	0	0	0	0
ENSG00000257783	0	0	0	0
ENSG00000257784	0	0	0	0
ENSG00000257786	0	0	0	0
ENSG00000257787	0	0	0	0
ENSG00000257790	0	0	0	0
ENSG00000257791	0	0	0	0
ENSG00000257792	0	0	0	0
ENSG00000257793	0	0	0	0
ENSG00000257794	0	0	0	0
ENSG00000257796	0	0	0	0
ENSG00000257797	0	0	0	0
ENSG00000257798	0	0	0	0
ENSG00000257800	3	8	6	5
ENSG00000257802	0	0	0	0
ENSG00000257803	0	0	0	0
ENSG00000257805	0	0	0	0
ENSG00000257806	0	0	0	0
ENSG00000257807	0	0	0	0
ENSG00000257808	0	0	0	0
ENSG00000257809	0	0	2	0
ENSG00000257811	0	0	0	0
ENSG00000257813	0	0	0	0
ENSG00000257814	0	0	0	0
ENSG00000257815	315	596	350	385
ENSG00000257817	0	0	0	0
ENSG00000257818	0	0	0	0
ENSG00000257819	0	0	0	0
ENSG00000257820	0	0	0	0
ENSG00000257822	0	0	0	0

ENSG00000257823	0	0	0	0
ENSG00000257824	2	0	0	0
ENSG00000257825	0	0	0	0
ENSG00000257826	0	0	0	0
ENSG00000257829	0	0	0	0
ENSG00000257830	0	0	0	0
ENSG00000257831	0	0	1	8
ENSG00000257832	0	0	0	0
ENSG00000257834	0	0	0	0
ENSG00000257835	0	0	0	0
ENSG00000257837	0	0	0	0
ENSG00000257838	0	1	0	0
ENSG00000257839	0	0	2	0
ENSG00000257841	0	0	0	0
ENSG00000257842	0	0	0	0
ENSG00000257843	0	0	0	0
ENSG00000257844	0	0	0	0
ENSG00000257845	0	0	0	0
ENSG00000257846	0	0	0	0
ENSG00000257847	0	0	0	0
ENSG00000257848	0	0	0	0
ENSG00000257849	0	0	0	0
ENSG00000257850	0	0	0	0
ENSG00000257851	0	0	0	0
ENSG00000257852	0	0	0	0
ENSG00000257853	0	0	0	0
ENSG00000257854	0	0	0	0
ENSG00000257855	0	0	0	0
ENSG00000257856	0	0	0	0
ENSG00000257858	0	0	0	0
ENSG00000257859	0	0	0	0
ENSG00000257860	0	0	0	0
ENSG00000257862	0	0	0	0
ENSG00000257863	0	0	0	0
ENSG00000257864	0	0	0	0
ENSG00000257865	0	0	0	0
ENSG00000257867	0	0	0	0
ENSG00000257868	0	0	0	0
ENSG00000257869	0	0	0	0
ENSG00000257870	0	0	35	70
ENSG00000257872	0	0	0	0
ENSG00000257873	0	0	0	0
ENSG00000257874	0	0	0	0
ENSG00000257875	0	0	0	1
ENSG00000257877	0	0	0	0
ENSG00000257878	0	0	0	0

ENSG00000257879	0	0	0	0
ENSG00000257880	0	0	0	0
ENSG00000257882	0	0	0	0
ENSG00000257883	0	0	0	0
ENSG00000257884	0	0	0	0
ENSG00000257885	0	0	0	0
ENSG00000257886	0	0	0	0
ENSG00000257887	0	0	0	0
ENSG00000257888	0	0	0	0
ENSG00000257889	0	0	0	0
ENSG00000257890	0	0	0	0
ENSG00000257891	0	0	0	0
ENSG00000257892	0	0	0	0
ENSG00000257893	0	0	0	0
ENSG00000257894	1	0	9	4
ENSG00000257895	0	0	0	0
ENSG00000257896	0	0	2	1
ENSG00000257897	0	0	0	0
ENSG00000257899	0	0	0	0
ENSG00000257900	0	0	0	0
ENSG00000257901	0	0	0	0
ENSG00000257902	0	0	0	0
ENSG00000257904	0	0	0	0
ENSG00000257905	0	0	0	0
ENSG00000257906	0	0	0	0
ENSG00000257907	0	0	0	0
ENSG00000257910	0	0	0	0
ENSG00000257911	0	0	0	0
ENSG00000257912	0	0	0	0
ENSG00000257913	0	0	0	0
ENSG00000257914	0	0	0	0
ENSG00000257915	0	0	0	0
ENSG00000257916	0	0	0	0
ENSG00000257917	0	0	0	0
ENSG00000257918	1	0	7	0
ENSG00000257920	0	0	0	2
ENSG00000257921	0	0	0	0
ENSG00000257922	0	0	0	0
ENSG00000257923	1006	7380	7393	9897
ENSG00000257924	0	0	0	0
ENSG00000257925	0	0	0	0
ENSG00000257927	0	0	0	0
ENSG00000257931	0	0	0	0
ENSG00000257932	0	0	0	0
ENSG00000257933	0	0	0	0
ENSG00000257934	0	0	0	0

ENSG00000257935	0	0	0	0
ENSG00000257936	0	0	0	0
ENSG00000257937	0	0	0	0
ENSG00000257938	0	0	0	0
ENSG00000257939	0	0	0	0
ENSG00000257940	0	0	0	0
ENSG00000257941	0	0	7	20
ENSG00000257943	0	0	0	0
ENSG00000257944	0	0	0	0
ENSG00000257945	0	0	0	0
ENSG00000257947	0	0	0	0
ENSG00000257948	0	0	0	0
ENSG00000257949	0	0	0	0
ENSG00000257950	0	18	1	0
ENSG00000257951	0	0	0	0
ENSG00000257953	5	0	39	55
ENSG00000257954	0	52	23	16
ENSG00000257955	0	0	0	0
ENSG00000257956	0	0	0	0
ENSG00000257957	0	0	0	0
ENSG00000257958	0	0	0	0
ENSG00000257959	0	0	0	0
ENSG00000257961	0	0	0	0
ENSG00000257962	0	0	0	0
ENSG00000257964	0	0	0	0
ENSG00000257966	0	0	0	0
ENSG00000257967	0	0	0	0
ENSG00000257972	0	0	0	0
ENSG00000257973	74	92	99	78
ENSG00000257975	0	0	0	0
ENSG00000257976	0	0	0	0
ENSG00000257977	0	0	0	0
ENSG00000257978	0	0	0	0
ENSG00000257979	0	0	0	0
ENSG00000257980	0	0	0	0
ENSG00000257981	0	0	0	0
ENSG00000257984	0	0	0	0
ENSG00000257985	0	0	0	0
ENSG00000257986	0	0	0	0
ENSG00000257987	6	0	7	12
ENSG00000257989	0	0	0	0
ENSG00000257990	0	0	0	0
ENSG00000257991	0	0	0	0
ENSG00000257993	0	0	0	0
ENSG00000257994	0	0	0	0
ENSG00000257995	0	0	0	0

ENSG00000257997	0	0	0	0
ENSG00000257998	0	0	0	0
ENSG00000257999	0	0	0	0
ENSG00000258001	0	0	0	0
ENSG00000258002	0	0	0	0
ENSG00000258003	0	0	0	0
ENSG00000258004	0	0	0	0
ENSG00000258007	0	0	0	0
ENSG00000258008	0	0	0	0
ENSG00000258009	0	0	0	0
ENSG00000258010	0	0	0	0
ENSG00000258011	0	0	0	0
ENSG00000258012	0	0	0	0
ENSG00000258013	0	0	0	0
ENSG00000258014	0	0	0	0
ENSG00000258015	0	0	0	0
ENSG00000258016	0	0	0	0
ENSG00000258017	0	332	298	186
ENSG00000258018	0	0	0	0
ENSG00000258020	0	0	0	0
ENSG00000258021	0	0	0	0
ENSG00000258022	0	0	0	0
ENSG00000258023	0	0	0	0
ENSG00000258024	0	0	0	0
ENSG00000258025	0	0	0	0
ENSG00000258026	0	15	1	1
ENSG00000258027	0	0	0	1
ENSG00000258028	0	0	0	0
ENSG00000258030	0	0	0	0
ENSG00000258032	0	0	0	0
ENSG00000258033	0	0	0	0
ENSG00000258034	0	0	1	2
ENSG00000258035	0	0	0	0
ENSG00000258036	0	0	0	0
ENSG00000258037	0	0	0	0
ENSG00000258038	0	0	1	0
ENSG00000258039	0	0	0	0
ENSG00000258040	0	0	0	0
ENSG00000258044	0	0	0	0
ENSG00000258045	0	0	0	0
ENSG00000258048	0	0	0	0
ENSG00000258049	0	0	0	0
ENSG00000258050	0	0	0	0
ENSG00000258051	2	23	24	44
ENSG00000258052	0	0	0	0
ENSG00000258053	0	0	0	0

ENSG00000258054	0	0	0	0
ENSG00000258056	3	0	0	3
ENSG00000258057	2	0	0	0
ENSG00000258058	0	0	0	0
ENSG00000258059	0	0	0	0
ENSG00000258060	0	0	0	0
ENSG00000258062	0	0	0	0
ENSG00000258064	0	0	0	0
ENSG00000258065	0	0	0	0
ENSG00000258066	0	0	0	0
ENSG00000258067	0	0	0	0
ENSG00000258068	0	0	0	0
ENSG00000258069	0	0	0	0
ENSG00000258070	0	0	0	0
ENSG00000258071	0	0	0	0
ENSG00000258072	0	0	1	0
ENSG00000258073	0	0	0	0
ENSG00000258074	0	0	0	0
ENSG00000258075	0	0	0	0
ENSG00000258076	0	0	0	0
ENSG00000258077	0	0	0	0
ENSG00000258080	0	0	0	0
ENSG00000258081	0	0	0	0
ENSG00000258082	0	0	0	0
ENSG00000258083	0	0	0	0
ENSG00000258084	0	0	0	0
ENSG00000258085	0	0	0	0
ENSG00000258086	1	0	0	0
ENSG00000258088	0	0	0	1
ENSG00000258090	0	0	0	1
ENSG00000258091	0	0	0	0
ENSG00000258092	0	0	0	0
ENSG00000258093	0	0	0	0
ENSG00000258094	0	0	0	0
ENSG00000258096	4	0	0	0
ENSG00000258097	0	0	0	0
ENSG00000258098	0	0	0	0
ENSG00000258099	0	0	1	1
ENSG00000258100	0	0	0	0
ENSG00000258101	0	0	0	0
ENSG00000258102	2	0	1	0
ENSG00000258103	0	0	0	0
ENSG00000258104	0	0	0	0
ENSG00000258107	0	0	0	0
ENSG00000258108	0	0	0	0
ENSG00000258109	0	0	2	0

ENSG00000258110	0	0	0	0
ENSG00000258111	0	0	0	0
ENSG00000258112	0	0	0	0
ENSG00000258113	0	0	0	0
ENSG00000258114	0	0	0	0
ENSG00000258115	0	0	0	0
ENSG00000258116	0	0	0	0
ENSG00000258117	0	0	0	0
ENSG00000258118	0	0	0	0
ENSG00000258119	0	0	0	0
ENSG00000258120	0	0	0	0
ENSG00000258121	0	0	0	0
ENSG00000258122	13	0	12	18
ENSG00000258123	4	31	10	9
ENSG00000258124	0	0	0	0
ENSG00000258125	0	0	0	0
ENSG00000258126	0	0	0	0
ENSG00000258128	0	0	0	0
ENSG00000258130	0	0	0	0
ENSG00000258131	0	0	0	0
ENSG00000258133	0	0	0	0
ENSG00000258134	0	0	0	0
ENSG00000258135	0	0	0	0
ENSG00000258136	0	0	11	0
ENSG00000258137	0	0	0	0
ENSG00000258138	0	0	0	0
ENSG00000258140	0	0	0	0
ENSG00000258141	0	0	0	0
ENSG00000258142	0	0	0	0
ENSG00000258143	0	0	0	0
ENSG00000258144	0	0	0	0
ENSG00000258145	0	0	0	0
ENSG00000258146	0	0	0	0
ENSG00000258147	0	0	0	0
ENSG00000258148	0	0	0	0
ENSG00000258149	0	0	0	0
ENSG00000258150	0	0	0	0
ENSG00000258151	0	0	0	0
ENSG00000258153	0	0	0	2
ENSG00000258154	0	0	0	0
ENSG00000258155	0	0	0	0
ENSG00000258158	0	0	0	0
ENSG00000258159	0	0	0	0
ENSG00000258160	0	0	0	0
ENSG00000258162	0	0	0	0
ENSG00000258164	0	0	0	0

ENSG00000258165	0	0	0	0
ENSG00000258166	0	0	0	0
ENSG00000258167	0	0	0	0
ENSG00000258168	0	0	1	3
ENSG00000258169	0	0	0	0
ENSG00000258170	0	0	0	0
ENSG00000258171	0	0	0	0
ENSG00000258172	0	0	0	0
ENSG00000258173	0	0	0	0
ENSG00000258175	0	0	0	0
ENSG00000258177	7	51	51	23
ENSG00000258178	0	0	0	0
ENSG00000258179	0	0	0	0
ENSG00000258181	1	0	4	11
ENSG00000258182	0	0	0	0
ENSG00000258183	0	0	0	0
ENSG00000258184	0	0	0	0
ENSG00000258185	0	0	0	0
ENSG00000258186	0	0	0	0
ENSG00000258188	0	0	0	0
ENSG00000258191	0	0	0	0
ENSG00000258192	0	0	0	0
ENSG00000258193	0	0	0	0
ENSG00000258194	0	0	0	0
ENSG00000258195	0	0	0	0
ENSG00000258196	0	0	0	0
ENSG00000258197	0	0	0	0
ENSG00000258198	0	0	0	0
ENSG00000258199	4	0	0	0
ENSG00000258200	0	0	0	0
ENSG00000258202	0	0	0	0
ENSG00000258203	0	0	0	0
ENSG00000258204	0	0	0	0
ENSG00000258205	0	0	0	0
ENSG00000258206	0	0	1	0
ENSG00000258207	0	0	0	0
ENSG00000258208	0	0	0	0
ENSG00000258209	0	0	0	0
ENSG00000258210	0	0	0	0
ENSG00000258211	0	0	0	0
ENSG00000258212	0	0	0	0
ENSG00000258213	0	0	0	0
ENSG00000258214	0	0	0	0
ENSG00000258215	0	0	0	0
ENSG00000258216	0	0	1	0
ENSG00000258218	0	0	0	0

ENSG00000258219	0	0	0	0
ENSG00000258220	0	0	0	0
ENSG00000258221	0	0	0	0
ENSG00000258223	0	0	0	0
ENSG00000258224	0	0	0	0
ENSG00000258225	0	0	0	0
ENSG00000258226	0	0	0	0
ENSG00000258227	0	0	2	0
ENSG00000258228	0	0	0	0
ENSG00000258230	0	0	0	0
ENSG00000258231	0	0	0	0
ENSG00000258232	0	185	159	112
ENSG00000258233	0	0	0	0
ENSG00000258234	0	0	0	0
ENSG00000258235	0	0	0	3
ENSG00000258238	0	0	0	0
ENSG00000258239	0	0	0	0
ENSG00000258240	0	0	0	0
ENSG00000258241	0	0	0	0
ENSG00000258243	0	0	0	0
ENSG00000258244	0	0	0	0
ENSG00000258245	0	0	0	0
ENSG00000258247	0	0	0	0
ENSG00000258248	0	0	0	0
ENSG00000258249	0	0	0	0
ENSG00000258251	0	0	0	0
ENSG00000258252	0	0	0	0
ENSG00000258253	0	0	0	0
ENSG00000258254	0	0	0	0
ENSG00000258256	6	0	2	5
ENSG00000258259	0	0	0	0
ENSG00000258260	0	0	0	0
ENSG00000258261	0	0	0	0
ENSG00000258262	0	0	0	0
ENSG00000258263	0	0	0	0
ENSG00000258264	0	0	0	0
ENSG00000258265	0	0	0	0
ENSG00000258268	0	0	0	0
ENSG00000258269	0	0	0	0
ENSG00000258270	0	0	0	0
ENSG00000258271	0	0	0	0
ENSG00000258272	0	0	0	0
ENSG00000258273	0	0	0	0
ENSG00000258274	0	0	0	0
ENSG00000258275	0	0	0	0
ENSG00000258276	0	0	0	0

ENSG00000258278	0	0	0	0
ENSG00000258279	0	0	0	0
ENSG00000258280	0	0	0	0
ENSG00000258281	0	0	0	0
ENSG00000258282	0	0	0	0
ENSG00000258283	0	0	0	0
ENSG00000258284	0	0	0	0
ENSG00000258285	0	0	0	0
ENSG00000258286	0	0	0	0
ENSG00000258287	0	0	0	0
ENSG00000258288	0	0	0	0
ENSG00000258289	517	1663	2589	3283
ENSG00000258290	0	0	0	0
ENSG00000258291	0	0	0	0
ENSG00000258292	0	0	0	0
ENSG00000258294	7	10	46	55
ENSG00000258296	0	0	0	0
ENSG00000258297	7	38	77	48
ENSG00000258298	0	0	0	0
ENSG00000258299	0	0	0	0
ENSG00000258300	0	0	0	0
ENSG00000258301	0	0	0	4
ENSG00000258302	0	0	5	6
ENSG00000258303	0	0	0	0
ENSG00000258304	0	0	0	0
ENSG00000258308	0	0	0	0
ENSG00000258311	0	0	0	0
ENSG00000258312	0	0	0	0
ENSG00000258313	0	0	0	0
ENSG00000258314	0	0	0	0
ENSG00000258315	0	0	0	0
ENSG00000258316	0	0	0	0
ENSG00000258317	0	0	2	0
ENSG00000258318	0	0	0	0
ENSG00000258320	0	0	0	0
ENSG00000258321	0	0	0	0
ENSG00000258322	0	0	0	0
ENSG00000258323	0	0	0	0
ENSG00000258324	0	0	0	0
ENSG00000258325	0	0	0	0
ENSG00000258326	0	0	0	0
ENSG00000258330	0	0	0	0
ENSG00000258331	0	0	0	0
ENSG00000258332	0	0	0	0
ENSG00000258333	0	0	0	0
ENSG00000258334	0	0	0	0

ENSG00000258336	0	0	0	0
ENSG00000258337	0	0	0	0
ENSG00000258338	0	0	0	0
ENSG00000258342	0	0	0	0
ENSG00000258343	0	0	0	0
ENSG00000258344	0	0	0	0
ENSG00000258345	0	0	2	0
ENSG00000258346	0	0	0	0
ENSG00000258348	0	0	0	0
ENSG00000258349	0	0	0	0
ENSG00000258350	0	0	0	0
ENSG00000258352	2	0	0	0
ENSG00000258353	0	0	0	0
ENSG00000258354	0	0	0	0
ENSG00000258355	0	0	0	0
ENSG00000258356	0	0	0	0
ENSG00000258357	0	0	0	0
ENSG00000258358	0	0	0	0
ENSG00000258359	0	0	5	0
ENSG00000258360	0	0	0	0
ENSG00000258361	0	0	0	0
ENSG00000258363	0	0	0	0
ENSG00000258364	0	0	0	0
ENSG00000258365	0	0	3	3
ENSG00000258366	0	0	0	0
ENSG00000258367	0	0	0	0
ENSG00000258368	0	0	0	0
ENSG00000258369	0	0	0	0
ENSG00000258370	0	0	0	0
ENSG00000258372	0	0	0	0
ENSG00000258373	0	0	0	0
ENSG00000258374	0	0	0	0
ENSG00000258375	0	0	0	0
ENSG00000258376	3	0	0	0
ENSG00000258377	0	0	4	4
ENSG00000258378	0	0	0	0
ENSG00000258379	0	0	0	0
ENSG00000258380	0	0	0	0
ENSG00000258381	0	0	0	0
ENSG00000258382	0	0	0	0
ENSG00000258383	0	0	0	0
ENSG00000258384	0	0	2	5
ENSG00000258385	0	0	0	0
ENSG00000258386	0	0	0	0
ENSG00000258387	0	0	0	0
ENSG00000258388	0	263	213	83

ENSG00000258389	0	0	0	0
ENSG00000258390	0	0	0	0
ENSG00000258391	0	0	0	0
ENSG00000258392	0	0	0	0
ENSG00000258393	0	0	0	0
ENSG00000258394	0	0	0	0
ENSG00000258395	0	0	0	0
ENSG00000258396	0	0	0	0
ENSG00000258397	0	0	0	0
ENSG00000258398	0	0	3	0
ENSG00000258399	0	83	53	78
ENSG00000258400	0	0	0	0
ENSG00000258401	0	0	0	0
ENSG00000258402	0	0	0	0
ENSG00000258403	0	0	0	0
ENSG00000258404	0	0	0	0
ENSG00000258405	1	0	2	10
ENSG00000258406	0	0	0	0
ENSG00000258407	0	0	0	0
ENSG00000258408	0	0	0	0
ENSG00000258410	2	388	187	184
ENSG00000258411	0	0	0	0
ENSG00000258412	0	0	0	0
ENSG00000258413	0	12	0	0
ENSG00000258414	0	0	1	0
ENSG00000258415	0	0	0	0
ENSG00000258416	0	0	0	0
ENSG00000258417	0	0	0	0
ENSG00000258418	10	121	168	112
ENSG00000258419	0	0	0	0
ENSG00000258420	0	0	0	0
ENSG00000258421	0	0	0	0
ENSG00000258422	12	127	153	233
ENSG00000258423	0	0	0	0
ENSG00000258424	0	83	71	26
ENSG00000258425	0	0	0	0
ENSG00000258426	0	0	0	0
ENSG00000258427	0	0	1	0
ENSG00000258428	0	0	0	0
ENSG00000258429	1	0	0	0
ENSG00000258430	0	0	7	0
ENSG00000258431	0	0	0	0
ENSG00000258433	0	0	0	0
ENSG00000258434	0	0	0	0
ENSG00000258435	0	0	0	0
ENSG00000258436	0	0	0	0

ENSG00000258437	0	0	0	0
ENSG00000258438	0	0	0	0
ENSG00000258439	0	0	0	0
ENSG00000258440	1	0	0	0
ENSG00000258441	2	0	4	0
ENSG00000258442	0	0	0	0
ENSG00000258443	0	0	0	0
ENSG00000258444	0	0	0	0
ENSG00000258445	0	0	2	0
ENSG00000258446	2	0	0	0
ENSG00000258447	0	0	0	0
ENSG00000258448	0	0	0	0
ENSG00000258449	0	3	17	19
ENSG00000258450	0	0	0	0
ENSG00000258451	0	0	0	0
ENSG00000258452	0	0	0	0
ENSG00000258453	0	0	0	0
ENSG00000258454	0	0	2	0
ENSG00000258455	0	0	0	0
ENSG00000258456	0	0	0	0
ENSG00000258457	0	0	0	4
ENSG00000258458	0	0	0	0
ENSG00000258459	0	0	1	0
ENSG00000258460	0	0	0	0
ENSG00000258461	5	0	0	0
ENSG00000258462	0	0	0	0
ENSG00000258463	0	0	0	0
ENSG00000258464	0	0	0	0
ENSG00000258465	4	0	0	0
ENSG00000258466	0	0	1	3
ENSG00000258467	0	0	0	0
ENSG00000258468	0	0	0	0
ENSG00000258469	0	0	0	0
ENSG00000258471	0	0	0	0
ENSG00000258472	1	0	0	1
ENSG00000258473	2	0	0	0
ENSG00000258474	0	0	0	0
ENSG00000258475	0	0	0	0
ENSG00000258476	2	0	0	0
ENSG00000258477	0	0	0	0
ENSG00000258478	0	0	0	0
ENSG00000258479	0	0	0	0
ENSG00000258480	4	86	188	105
ENSG00000258481	0	0	0	0
ENSG00000258482	0	0	0	0
ENSG00000258483	0	0	0	0

ENSG00000258484	0	0	1	0
ENSG00000258485	0	0	0	0
ENSG00000258486	0	0	0	0
ENSG00000258487	0	0	0	0
ENSG00000258488	0	0	0	0
ENSG00000258489	0	0	0	0
ENSG00000258490	0	0	0	0
ENSG00000258491	0	0	0	0
ENSG00000258493	0	0	0	0
ENSG00000258494	0	0	0	0
ENSG00000258496	0	0	0	0
ENSG00000258497	0	0	0	0
ENSG00000258498	0	0	0	0
ENSG00000258499	0	0	0	0
ENSG00000258500	0	0	0	0
ENSG00000258501	0	0	0	0
ENSG00000258502	0	0	0	0
ENSG00000258503	0	0	0	0
ENSG00000258504	0	0	0	0
ENSG00000258505	0	0	0	0
ENSG00000258506	0	0	0	0
ENSG00000258507	0	0	0	0
ENSG00000258509	0	0	0	0
ENSG00000258510	0	0	0	0
ENSG00000258511	0	0	0	0
ENSG00000258512	1	0	0	0
ENSG00000258513	0	0	0	0
ENSG00000258514	0	0	0	0
ENSG00000258515	0	0	1	0
ENSG00000258516	0	0	0	0
ENSG00000258517	0	0	0	0
ENSG00000258518	0	0	0	0
ENSG00000258519	0	0	0	0
ENSG00000258520	0	0	0	0
ENSG00000258521	0	0	0	0
ENSG00000258522	0	0	0	0
ENSG00000258524	0	0	0	0
ENSG00000258525	0	0	6	4
ENSG00000258526	0	0	5	6
ENSG00000258527	0	0	0	0
ENSG00000258528	0	0	0	0
ENSG00000258529	0	0	0	0
ENSG00000258530	0	0	0	0
ENSG00000258531	0	0	0	0
ENSG00000258532	0	0	0	0
ENSG00000258533	0	0	0	0

ENSG00000258534	0	0	0	0
ENSG00000258535	0	0	0	0
ENSG00000258536	0	0	0	0
ENSG00000258537	0	0	0	0
ENSG00000258538	0	0	0	0
ENSG00000258539	0	0	0	0
ENSG00000258540	0	0	0	0
ENSG00000258541	0	0	0	0
ENSG00000258542	0	0	0	0
ENSG00000258543	0	0	0	0
ENSG00000258544	0	0	0	0
ENSG00000258545	0	0	0	0
ENSG00000258546	0	0	0	0
ENSG00000258548	0	0	0	0
ENSG00000258549	0	0	0	0
ENSG00000258550	0	0	0	0
ENSG00000258551	2	0	2	32
ENSG00000258552	0	0	0	0
ENSG00000258553	0	0	14	10
ENSG00000258554	0	0	3	1
ENSG00000258555	0	0	0	0
ENSG00000258556	0	13	0	0
ENSG00000258557	0	0	0	0
ENSG00000258558	0	0	0	0
ENSG00000258559	0	0	0	0
ENSG00000258560	0	0	0	0
ENSG00000258561	0	0	0	0
ENSG00000258562	0	0	0	0
ENSG00000258563	0	0	0	0
ENSG00000258564	0	0	0	0
ENSG00000258565	0	0	0	0
ENSG00000258566	0	0	0	0
ENSG00000258567	0	0	0	0
ENSG00000258568	0	0	0	6
ENSG00000258569	0	0	0	0
ENSG00000258570	0	0	0	0
ENSG00000258571	0	0	0	0
ENSG00000258572	3	0	0	0
ENSG00000258573	0	0	0	0
ENSG00000258576	0	0	0	0
ENSG00000258577	0	0	0	0
ENSG00000258578	0	0	0	0
ENSG00000258579	0	0	2	0
ENSG00000258580	0	0	0	0
ENSG00000258581	0	0	0	0
ENSG00000258582	0	0	0	0

ENSG00000258583	0	0	4	0
ENSG00000258584	0	0	0	0
ENSG00000258585	0	0	0	0
ENSG00000258586	1	0	0	0
ENSG00000258587	0	0	0	0
ENSG00000258588	0	0	0	0
ENSG00000258589	0	0	0	0
ENSG00000258590	2	246	210	353
ENSG00000258591	1	0	0	0
ENSG00000258592	0	0	0	0
ENSG00000258593	0	0	0	0
ENSG00000258594	0	0	0	0
ENSG00000258595	0	0	0	0
ENSG00000258597	0	0	0	0
ENSG00000258598	0	0	0	0
ENSG00000258599	0	0	0	0
ENSG00000258600	0	0	0	0
ENSG00000258601	0	0	0	0
ENSG00000258602	0	0	0	0
ENSG00000258603	0	17	0	3
ENSG00000258604	0	0	0	0
ENSG00000258605	0	0	0	0
ENSG00000258607	5	0	5	1
ENSG00000258608	0	0	0	0
ENSG00000258609	2	0	0	0
ENSG00000258610	0	0	0	0
ENSG00000258611	0	0	0	0
ENSG00000258612	0	0	0	0
ENSG00000258613	0	0	0	0
ENSG00000258615	1	14	3	0
ENSG00000258616	0	0	0	0
ENSG00000258617	0	0	0	0
ENSG00000258618	0	0	0	0
ENSG00000258619	0	0	0	0
ENSG00000258620	0	0	0	0
ENSG00000258622	0	0	0	0
ENSG00000258623	0	0	0	0
ENSG00000258624	0	0	0	0
ENSG00000258625	0	0	0	0
ENSG00000258626	2	0	0	0
ENSG00000258627	0	0	0	0
ENSG00000258628	0	0	0	0
ENSG00000258629	0	0	0	0
ENSG00000258630	0	0	0	0
ENSG00000258631	0	0	0	0
ENSG00000258632	0	0	0	0

ENSG00000258633	0	0	0	0
ENSG00000258634	2	0	0	2
ENSG00000258635	0	0	0	0
ENSG00000258636	0	0	0	0
ENSG00000258637	0	0	0	0
ENSG00000258638	0	0	0	0
ENSG00000258639	0	0	0	0
ENSG00000258640	0	0	0	0
ENSG00000258641	0	0	0	0
ENSG00000258642	0	0	0	0
ENSG00000258643	0	0	0	0
ENSG00000258644	0	0	0	0
ENSG00000258645	0	0	0	0
ENSG00000258646	0	0	4	3
ENSG00000258647	0	0	0	0
ENSG00000258648	0	0	0	0
ENSG00000258649	0	0	0	0
ENSG00000258650	0	0	0	0
ENSG00000258651	0	0	0	0
ENSG00000258652	0	0	0	0
ENSG00000258653	0	0	0	0
ENSG00000258654	0	0	0	0
ENSG00000258655	15	0	15	47
ENSG00000258656	0	0	0	0
ENSG00000258657	0	0	0	0
ENSG00000258658	0	0	0	0
ENSG00000258659	0	0	0	0
ENSG00000258660	0	0	0	0
ENSG00000258661	0	0	0	0
ENSG00000258662	0	0	0	0
ENSG00000258663	0	0	0	0
ENSG00000258664	0	0	0	0
ENSG00000258665	0	0	0	0
ENSG00000258666	0	0	0	0
ENSG00000258667	1	0	1	10
ENSG00000258668	0	0	0	0
ENSG00000258670	0	0	0	0
ENSG00000258671	0	0	0	0
ENSG00000258672	0	0	0	0
ENSG00000258673	0	0	0	0
ENSG00000258674	0	0	0	0
ENSG00000258675	0	0	0	0
ENSG00000258676	0	0	0	0
ENSG00000258677	0	0	0	0
ENSG00000258678	1	0	0	0
ENSG00000258679	0	0	0	0

ENSG00000258680	0	0	0	0
ENSG00000258681	0	0	0	0
ENSG00000258682	0	0	0	0
ENSG00000258683	0	0	0	0
ENSG00000258684	0	0	0	0
ENSG00000258685	0	0	0	0
ENSG00000258686	0	0	0	0
ENSG00000258687	0	0	0	0
ENSG00000258689	0	0	3	1
ENSG00000258690	0	0	0	0
ENSG00000258691	0	0	0	0
ENSG00000258692	0	0	0	0
ENSG00000258693	0	0	0	0
ENSG00000258694	63	64	56	110
ENSG00000258695	0	0	0	0
ENSG00000258696	0	0	0	0
ENSG00000258697	0	0	0	0
ENSG00000258698	0	0	0	0
ENSG00000258699	0	0	0	0
ENSG00000258700	0	0	0	0
ENSG00000258701	0	0	0	0
ENSG00000258702	0	0	0	0
ENSG00000258703	0	0	0	0
ENSG00000258704	18	586	263	885
ENSG00000258705	0	0	0	0
ENSG00000258706	0	0	0	0
ENSG00000258707	0	0	0	0
ENSG00000258708	0	0	0	0
ENSG00000258710	0	0	0	0
ENSG00000258711	5	0	0	1
ENSG00000258712	0	0	0	0
ENSG00000258713	0	0	0	0
ENSG00000258714	0	0	3	0
ENSG00000258716	0	0	0	0
ENSG00000258717	1	0	1	2
ENSG00000258718	0	0	0	0
ENSG00000258719	0	0	0	0
ENSG00000258721	0	0	0	0
ENSG00000258722	0	0	0	0
ENSG00000258723	0	0	0	0
ENSG00000258724	0	0	0	0
ENSG00000258725	0	0	7	3
ENSG00000258726	0	0	0	0
ENSG00000258727	0	0	1	0
ENSG00000258728	0	0	0	0
ENSG00000258729	0	0	0	0

ENSG00000258730	0	0	0	0
ENSG00000258731	0	0	13	21
ENSG00000258732	1	0	0	5
ENSG00000258733	1	9	10	16
ENSG00000258734	0	0	0	0
ENSG00000258735	0	0	0	0
ENSG00000258736	0	0	0	0
ENSG00000258737	0	0	0	0
ENSG00000258738	97	371	362	277
ENSG00000258739	0	0	0	0
ENSG00000258740	0	0	0	0
ENSG00000258741	0	0	0	0
ENSG00000258742	0	0	0	0
ENSG00000258743	0	0	3	0
ENSG00000258744	0	0	1	0
ENSG00000258745	0	13	34	24
ENSG00000258746	0	0	0	0
ENSG00000258747	0	0	0	0
ENSG00000258748	0	0	0	0
ENSG00000258749	0	0	0	0
ENSG00000258750	0	0	0	0
ENSG00000258751	0	0	0	0
ENSG00000258752	0	0	0	0
ENSG00000258753	0	0	0	0
ENSG00000258754	0	0	0	0
ENSG00000258755	0	0	0	0
ENSG00000258756	0	0	3	0
ENSG00000258757	0	0	0	0
ENSG00000258758	0	0	0	0
ENSG00000258759	0	0	0	1
ENSG00000258760	0	0	0	0
ENSG00000258761	0	8	1	0
ENSG00000258762	0	0	0	0
ENSG00000258763	0	0	0	0
ENSG00000258764	0	0	0	0
ENSG00000258765	0	17	23	22
ENSG00000258766	0	0	0	0
ENSG00000258767	0	0	0	0
ENSG00000258768	3	0	8	6
ENSG00000258769	0	0	0	0
ENSG00000258770	0	0	0	0
ENSG00000258771	0	0	0	0
ENSG00000258772	0	0	0	0
ENSG00000258773	0	0	0	0
ENSG00000258774	0	0	0	0
ENSG00000258776	0	0	0	0

ENSG00000258777	0	0	2	0
ENSG00000258778	0	0	0	0
ENSG00000258779	0	0	0	0
ENSG00000258780	0	0	0	0
ENSG00000258781	0	0	0	0
ENSG00000258782	0	5	4	0
ENSG00000258783	0	0	0	0
ENSG00000258784	0	0	0	0
ENSG00000258785	0	0	0	0
ENSG00000258786	0	0	0	0
ENSG00000258787	0	0	0	0
ENSG00000258788	0	0	0	0
ENSG00000258789	0	0	0	0
ENSG00000258790	0	0	0	0
ENSG00000258791	77	159	60	244
ENSG00000258792	0	0	0	0
ENSG00000258793	0	0	0	0
ENSG00000258794	0	0	0	0
ENSG00000258795	0	0	0	0
ENSG00000258796	0	0	0	0
ENSG00000258797	0	0	0	0
ENSG00000258798	3	0	0	8
ENSG00000258799	0	0	0	0
ENSG00000258800	0	0	0	0
ENSG00000258801	0	0	0	0
ENSG00000258802	0	0	0	0
ENSG00000258803	3	19	9	22
ENSG00000258804	0	0	0	0
ENSG00000258805	0	0	0	0
ENSG00000258806	0	0	0	0
ENSG00000258807	0	0	0	0
ENSG00000258808	0	0	0	0
ENSG00000258809	0	0	0	0
ENSG00000258810	0	0	0	0
ENSG00000258811	0	0	0	0
ENSG00000258812	0	0	0	0
ENSG00000258813	0	0	2	0
ENSG00000258814	0	0	0	0
ENSG00000258815	0	0	0	3
ENSG00000258816	0	0	0	0
ENSG00000258817	0	0	0	0
ENSG00000258818	0	0	0	0
ENSG00000258819	0	0	0	3
ENSG00000258820	0	0	0	0
ENSG00000258821	0	0	0	0
ENSG00000258822	0	0	0	0

ENSG00000258823	0	0	0	0
ENSG00000258824	1	0	0	0
ENSG00000258825	0	0	0	0
ENSG00000258826	0	0	0	2
ENSG00000258827	0	0	0	0
ENSG00000258828	0	0	0	0
ENSG00000258829	0	0	0	0
ENSG00000258830	0	0	0	0
ENSG00000258831	0	0	0	0
ENSG00000258834	0	0	0	0
ENSG00000258835	0	0	0	0
ENSG00000258836	0	0	0	0
ENSG00000258837	0	0	0	0
ENSG00000258838	0	0	0	0
ENSG00000258839	0	0	0	0
ENSG00000258841	0	0	0	0
ENSG00000258842	0	0	0	0
ENSG00000258843	2	43	6	15
ENSG00000258844	0	0	0	0
ENSG00000258845	0	0	0	0
ENSG00000258846	0	0	0	0
ENSG00000258847	0	0	0	0
ENSG00000258848	0	0	0	0
ENSG00000258849	0	0	0	0
ENSG00000258850	0	0	0	0
ENSG00000258851	0	0	0	0
ENSG00000258852	0	0	0	0
ENSG00000258853	0	0	0	0
ENSG00000258854	0	0	0	0
ENSG00000258855	0	0	0	0
ENSG00000258856	0	0	0	0
ENSG00000258857	0	63	4	4
ENSG00000258858	0	0	0	0
ENSG00000258859	0	0	0	0
ENSG00000258860	29	54	21	14
ENSG00000258861	0	0	0	0
ENSG00000258863	0	0	0	0
ENSG00000258864	0	0	0	0
ENSG00000258865	0	0	0	0
ENSG00000258866	0	0	0	0
ENSG00000258867	0	0	0	0
ENSG00000258868	0	0	0	0
ENSG00000258869	0	0	0	0
ENSG00000258870	0	0	0	0
ENSG00000258871	8	6	3	0
ENSG00000258872	0	0	0	0

ENSG00000258873	0	0	0	0
ENSG00000258874	0	0	0	0
ENSG00000258875	1	0	0	0
ENSG00000258876	0	0	0	0
ENSG00000258877	0	0	0	0
ENSG00000258878	0	0	0	0
ENSG00000258879	0	0	1	0
ENSG00000258880	0	0	0	0
ENSG00000258881	0	0	0	0
ENSG00000258882	0	0	0	0
ENSG00000258883	0	0	0	0
ENSG00000258884	1	0	0	7
ENSG00000258885	0	0	0	0
ENSG00000258886	0	0	0	0
ENSG00000258887	0	0	0	0
ENSG00000258888	0	0	0	0
ENSG00000258889	1	0	0	0
ENSG00000258890	12	207	213	259
ENSG00000258891	0	0	9	0
ENSG00000258892	0	0	0	0
ENSG00000258893	0	0	0	0
ENSG00000258894	0	0	0	0
ENSG00000258895	0	0	2	0
ENSG00000258896	0	0	0	0
ENSG00000258897	0	0	0	0
ENSG00000258899	0	0	0	0
ENSG00000258900	1	0	0	0
ENSG00000258901	0	0	0	0
ENSG00000258902	0	0	0	0
ENSG00000258903	0	0	0	0
ENSG00000258904	0	0	0	0
ENSG00000258905	0	0	0	0
ENSG00000258906	0	0	0	0
ENSG00000258907	0	0	0	0
ENSG00000258908	0	21	134	49
ENSG00000258909	0	0	0	0
ENSG00000258910	0	0	0	0
ENSG00000258911	0	0	0	0
ENSG00000258912	0	0	0	0
ENSG00000258913	0	0	0	0
ENSG00000258914	0	0	0	0
ENSG00000258915	0	0	0	0
ENSG00000258916	0	0	0	0
ENSG00000258917	0	0	0	0
ENSG00000258918	0	0	3	5
ENSG00000258919	0	0	0	0

ENSG00000258920	2	0	0	0
ENSG00000258921	0	0	0	0
ENSG00000258922	0	0	0	0
ENSG00000258923	0	0	0	0
ENSG00000258924	0	0	0	0
ENSG00000258925	0	0	0	0
ENSG00000258926	0	0	0	0
ENSG00000258927	0	0	0	0
ENSG00000258928	0	0	0	0
ENSG00000258929	1	0	1	0
ENSG00000258930	0	0	0	0
ENSG00000258931	0	0	0	0
ENSG00000258932	0	0	2	1
ENSG00000258933	0	0	0	0
ENSG00000258934	0	0	0	0
ENSG00000258935	1	0	0	0
ENSG00000258938	0	0	1	0
ENSG00000258939	0	0	0	0
ENSG00000258940	0	26	1	1
ENSG00000258941	0	0	0	0
ENSG00000258942	0	0	0	0
ENSG00000258943	0	0	0	0
ENSG00000258944	0	0	1	1
ENSG00000258945	0	0	0	0
ENSG00000258946	0	0	0	0
ENSG00000258947	0	0	0	0
ENSG00000258948	0	0	0	0
ENSG00000258949	1	0	0	0
ENSG00000258950	0	0	0	0
ENSG00000258951	0	0	0	0
ENSG00000258952	0	0	0	0
ENSG00000258953	0	0	2	0
ENSG00000258954	0	0	0	0
ENSG00000258955	0	0	0	0
ENSG00000258956	0	0	0	0
ENSG00000258957	0	0	0	0
ENSG00000258958	0	0	0	0
ENSG00000258959	0	0	4	7
ENSG00000258960	0	0	0	0
ENSG00000258962	0	0	0	0
ENSG00000258963	0	0	0	0
ENSG00000258964	0	0	0	0
ENSG00000258965	0	0	0	0
ENSG00000258966	0	0	0	0
ENSG00000258967	0	0	0	0
ENSG00000258968	0	0	0	0

ENSG00000258969	0	0	0	0
ENSG00000258970	0	0	0	0
ENSG00000258971	0	0	0	0
ENSG00000258972	0	0	0	0
ENSG00000258973	0	0	0	0
ENSG00000258975	0	0	0	0
ENSG00000258976	0	0	0	0
ENSG00000258977	0	0	0	0
ENSG00000258978	0	0	0	0
ENSG00000258979	0	0	1	0
ENSG00000258980	0	0	0	0
ENSG00000258981	0	0	0	0
ENSG00000258982	0	0	0	0
ENSG00000258983	0	0	0	0
ENSG00000258984	1	0	0	0
ENSG00000258985	0	0	0	0
ENSG00000258986	0	0	0	0
ENSG00000258987	0	0	0	0
ENSG00000258988	0	0	0	0
ENSG00000258989	0	0	0	0
ENSG00000258990	0	0	0	0
ENSG00000258991	0	0	0	0
ENSG00000258992	0	0	0	0
ENSG00000258993	0	0	0	0
ENSG00000258994	0	0	0	0
ENSG00000258995	0	0	0	0
ENSG00000258996	0	0	0	0
ENSG00000258997	0	0	0	0
ENSG00000258998	0	0	0	0
ENSG00000258999	53	694	644	820
ENSG00000259000	0	0	0	0
ENSG00000259001	0	0	0	0
ENSG00000259002	0	0	0	0
ENSG00000259003	0	0	0	0
ENSG00000259004	2	0	0	0
ENSG00000259005	0	0	1	0
ENSG00000259006	0	0	0	0
ENSG00000259007	0	0	0	0
ENSG00000259008	0	0	0	0
ENSG00000259009	0	0	0	0
ENSG00000259010	0	0	0	0
ENSG00000259011	0	0	0	0
ENSG00000259012	0	0	0	0
ENSG00000259013	0	0	0	0
ENSG00000259014	0	0	0	0
ENSG00000259015	0	0	0	0

ENSG00000259016	0	0	0	0
ENSG00000259017	0	0	1	0
ENSG00000259018	0	0	0	0
ENSG00000259019	0	0	0	0
ENSG00000259020	2	0	0	0
ENSG00000259021	0	0	0	0
ENSG00000259022	1	0	2	0
ENSG00000259023	0	0	0	0
ENSG00000259024	0	0	0	0
ENSG00000259025	0	0	0	0
ENSG00000259026	0	0	0	0
ENSG00000259027	0	0	0	0
ENSG00000259028	0	0	0	0
ENSG00000259029	0	0	0	0
ENSG00000259030	0	0	0	0
ENSG00000259031	0	0	0	0
ENSG00000259032	0	0	0	0
ENSG00000259033	0	0	12	9
ENSG00000259034	0	0	0	0
ENSG00000259035	0	0	0	0
ENSG00000259036	0	0	0	0
ENSG00000259037	0	0	0	0
ENSG00000259038	0	0	0	0
ENSG00000259039	0	0	2	0
ENSG00000259040	0	0	0	0
ENSG00000259041	0	0	0	0
ENSG00000259042	0	0	0	0
ENSG00000259043	0	0	0	0
ENSG00000259044	0	0	0	0
ENSG00000259045	0	0	0	0
ENSG00000259046	0	0	0	0
ENSG00000259047	0	0	0	0
ENSG00000259048	1	0	0	0
ENSG00000259049	0	0	2	0
ENSG00000259050	0	0	0	0
ENSG00000259051	0	0	0	0
ENSG00000259052	0	0	3	11
ENSG00000259053	0	0	0	0
ENSG00000259054	1	0	0	0
ENSG00000259055	0	0	0	0
ENSG00000259056	0	0	0	0
ENSG00000259057	0	0	0	0
ENSG00000259058	13	0	0	0
ENSG00000259059	0	0	0	0
ENSG00000259060	0	0	0	0
ENSG00000259061	0	0	0	0

ENSG00000259062	61	11	9	36
ENSG00000259063	0	0	0	0
ENSG00000259064	0	0	0	0
ENSG00000259065	30	15	7	8
ENSG00000259066	0	0	0	0
ENSG00000259067	0	0	0	0
ENSG00000259068	0	0	0	0
ENSG00000259069	0	0	0	0
ENSG00000259070	3	0	1	8
ENSG00000259071	76	175	215	83
ENSG00000259072	0	0	0	0
ENSG00000259073	0	0	0	0
ENSG00000259074	0	0	0	0
ENSG00000259075	1	0	0	0
ENSG00000259076	0	0	0	0
ENSG00000259077	0	0	0	0
ENSG00000259078	0	0	1	0
ENSG00000259079	0	0	0	0
ENSG00000259080	0	0	0	0
ENSG00000259081	0	0	0	0
ENSG00000259082	0	0	0	0
ENSG00000259083	0	0	0	0
ENSG00000259084	0	0	0	0
ENSG00000259086	0	0	0	2
ENSG00000259087	0	0	0	0
ENSG00000259088	1	0	0	17
ENSG00000259089	0	0	0	0
ENSG00000259090	0	0	0	0
ENSG00000259091	0	0	0	0
ENSG00000259092	0	0	0	0
ENSG00000259093	0	0	0	0
ENSG00000259094	0	0	0	0
ENSG00000259095	0	0	0	1
ENSG00000259096	0	0	0	0
ENSG00000259097	0	0	0	0
ENSG00000259098	0	0	0	0
ENSG00000259099	0	0	0	0
ENSG00000259100	0	0	0	0
ENSG00000259101	0	0	0	0
ENSG00000259102	0	0	0	0
ENSG00000259103	0	0	0	0
ENSG00000259104	0	0	0	0
ENSG00000259105	0	0	0	0
ENSG00000259106	0	0	0	0
ENSG00000259107	0	0	0	0
ENSG00000259108	0	0	0	0

ENSG00000259109	0	0	0	0
ENSG00000259110	0	0	0	0
ENSG00000259111	0	0	0	0
ENSG00000259112	0	0	0	0
ENSG00000259113	1	37	43	30
ENSG00000259114	0	0	0	0
ENSG00000259115	0	0	0	0
ENSG00000259116	0	0	2	0
ENSG00000259117	0	0	0	0
ENSG00000259118	0	580	807	487
ENSG00000259119	0	0	0	0
ENSG00000259120	29	118	1	3
ENSG00000259121	0	0	0	0
ENSG00000259122	0	0	0	0
ENSG00000259123	0	0	0	0
ENSG00000259124	0	0	0	0
ENSG00000259125	1	0	0	0
ENSG00000259126	0	0	0	0
ENSG00000259128	0	0	0	0
ENSG00000259129	0	0	0	0
ENSG00000259130	0	0	0	0
ENSG00000259131	0	0	0	0
ENSG00000259132	0	0	0	0
ENSG00000259133	0	0	1	1
ENSG00000259134	2	0	0	2
ENSG00000259135	0	0	0	0
ENSG00000259136	0	0	0	0
ENSG00000259137	0	0	0	0
ENSG00000259138	0	0	0	0
ENSG00000259139	0	0	0	0
ENSG00000259140	0	0	0	0
ENSG00000259141	0	0	0	0
ENSG00000259142	0	0	0	0
ENSG00000259143	2124	0	6	7
ENSG00000259144	0	0	0	0
ENSG00000259145	0	0	0	0
ENSG00000259146	0	0	0	3
ENSG00000259148	0	0	0	0
ENSG00000259149	0	0	0	0
ENSG00000259150	0	0	0	0
ENSG00000259151	0	0	0	0
ENSG00000259152	0	0	0	0
ENSG00000259153	0	0	0	0
ENSG00000259154	0	0	0	0
ENSG00000259155	0	0	0	0
ENSG00000259156	0	0	0	0

ENSG00000259157	0	0	0	0
ENSG00000259158	0	0	0	0
ENSG00000259159	0	0	0	0
ENSG00000259160	0	0	0	0
ENSG00000259161	0	0	0	0
ENSG00000259162	10	43	49	62
ENSG00000259163	0	47	18	14
ENSG00000259164	0	0	0	0
ENSG00000259165	55	127	108	136
ENSG00000259166	0	0	0	0
ENSG00000259167	0	0	0	0
ENSG00000259168	0	0	0	0
ENSG00000259169	0	0	0	0
ENSG00000259170	0	0	0	0
ENSG00000259171	0	0	0	0
ENSG00000259172	15	62	139	105
ENSG00000259173	0	0	0	0
ENSG00000259174	697	205	150	56
ENSG00000259175	0	0	0	0
ENSG00000259176	0	0	0	0
ENSG00000259177	0	0	0	0
ENSG00000259178	0	0	0	0
ENSG00000259179	0	0	0	0
ENSG00000259180	0	0	0	0
ENSG00000259181	0	0	0	0
ENSG00000259182	0	0	0	0
ENSG00000259183	0	0	0	0
ENSG00000259184	0	0	0	0
ENSG00000259185	0	31	55	12
ENSG00000259186	0	0	0	0
ENSG00000259187	0	0	0	0
ENSG00000259188	1	0	0	2
ENSG00000259190	0	0	0	0
ENSG00000259191	0	0	0	0
ENSG00000259192	0	0	0	0
ENSG00000259193	0	0	0	0
ENSG00000259194	0	0	0	0
ENSG00000259195	1	0	0	0
ENSG00000259196	0	0	0	0
ENSG00000259197	0	0	0	0
ENSG00000259198	3	0	0	0
ENSG00000259199	0	0	0	0
ENSG00000259200	0	0	0	0
ENSG00000259201	0	262	184	119
ENSG00000259202	0	0	0	0
ENSG00000259203	0	0	0	0

ENSG00000259204	0	0	0	0
ENSG00000259205	0	0	0	0
ENSG00000259206	0	0	0	0
ENSG00000259207	2638	38394	51674	37741
ENSG00000259208	0	0	0	0
ENSG00000259209	0	0	0	0
ENSG00000259211	0	0	0	2
ENSG00000259212	0	0	0	0
ENSG00000259213	0	0	0	0
ENSG00000259214	0	0	0	0
ENSG00000259215	0	0	0	0
ENSG00000259216	0	0	0	0
ENSG00000259217	0	0	0	0
ENSG00000259218	0	0	0	0
ENSG00000259219	0	0	0	0
ENSG00000259220	0	0	0	0
ENSG00000259221	0	0	0	0
ENSG00000259222	0	0	0	0
ENSG00000259223	0	0	0	0
ENSG00000259224	0	0	0	0
ENSG00000259225	9	0	0	0
ENSG00000259227	0	0	0	0
ENSG00000259228	0	0	0	0
ENSG00000259229	0	0	0	0
ENSG00000259230	0	0	0	0
ENSG00000259231	0	0	0	0
ENSG00000259232	0	0	0	0
ENSG00000259233	0	0	0	0
ENSG00000259234	0	0	0	0
ENSG00000259235	0	0	0	0
ENSG00000259236	0	0	0	0
ENSG00000259237	0	0	0	0
ENSG00000259238	3	40	66	17
ENSG00000259239	0	0	0	0
ENSG00000259240	0	0	0	0
ENSG00000259241	0	0	2	0
ENSG00000259242	0	0	0	0
ENSG00000259243	0	0	0	0
ENSG00000259244	0	0	0	0
ENSG00000259245	0	0	0	0
ENSG00000259246	0	0	0	0
ENSG00000259247	0	0	0	0
ENSG00000259248	0	12	57	45
ENSG00000259250	4	0	1	0
ENSG00000259251	0	0	0	0
ENSG00000259252	0	0	0	0

ENSG00000259254	0	0	1	1
ENSG00000259255	0	0	0	0
ENSG00000259256	0	0	0	0
ENSG00000259257	0	0	0	0
ENSG00000259258	0	0	0	0
ENSG00000259259	0	0	0	0
ENSG00000259260	0	6	25	1
ENSG00000259261	0	0	1	2
ENSG00000259262	0	0	0	0
ENSG00000259264	0	0	0	0
ENSG00000259265	0	0	0	0
ENSG00000259266	0	0	0	0
ENSG00000259267	1	0	0	0
ENSG00000259268	0	0	1	0
ENSG00000259269	0	0	0	0
ENSG00000259270	0	0	0	0
ENSG00000259271	0	0	0	0
ENSG00000259273	0	0	0	0
ENSG00000259274	1	0	2	0
ENSG00000259275	0	0	0	0
ENSG00000259276	0	0	0	0
ENSG00000259277	0	0	0	0
ENSG00000259278	1	527	241	269
ENSG00000259279	0	74	157	84
ENSG00000259280	0	0	0	0
ENSG00000259281	0	0	0	0
ENSG00000259282	0	0	0	0
ENSG00000259283	0	0	0	0
ENSG00000259284	0	0	0	0
ENSG00000259285	0	0	0	0
ENSG00000259286	0	0	0	0
ENSG00000259287	0	0	0	0
ENSG00000259288	0	0	0	0
ENSG00000259289	0	0	0	0
ENSG00000259290	0	0	0	0
ENSG00000259291	2	0	3	0
ENSG00000259293	4	0	6	3
ENSG00000259294	0	0	0	0
ENSG00000259295	0	0	0	0
ENSG00000259296	0	0	0	0
ENSG00000259297	0	0	0	0
ENSG00000259298	0	0	0	0
ENSG00000259299	0	0	2	0
ENSG00000259300	0	0	0	0
ENSG00000259301	0	0	0	0
ENSG00000259302	0	0	0	0

ENSG00000259303	0	0	0	0
ENSG00000259304	0	0	0	0
ENSG00000259305	2	0	0	0
ENSG00000259306	0	0	0	4
ENSG00000259307	0	0	0	0
ENSG00000259308	0	0	0	0
ENSG00000259309	0	0	0	0
ENSG00000259310	0	0	0	0
ENSG00000259312	0	0	0	0
ENSG00000259314	0	0	0	0
ENSG00000259315	0	0	0	0
ENSG00000259316	0	0	6	0
ENSG00000259317	0	0	0	0
ENSG00000259318	0	0	0	0
ENSG00000259319	0	0	0	0
ENSG00000259320	0	0	0	0
ENSG00000259321	0	0	0	0
ENSG00000259322	0	0	0	0
ENSG00000259323	0	0	0	0
ENSG00000259324	0	0	0	0
ENSG00000259325	0	0	0	0
ENSG00000259326	0	0	2	0
ENSG00000259327	2	13	16	28
ENSG00000259328	0	0	0	0
ENSG00000259329	0	0	0	0
ENSG00000259330	877	3794	3339	4135
ENSG00000259331	0	0	0	0
ENSG00000259332	0	0	2	4
ENSG00000259333	0	0	0	0
ENSG00000259334	0	0	0	0
ENSG00000259335	0	0	0	0
ENSG00000259336	0	0	1	7
ENSG00000259337	0	0	0	0
ENSG00000259338	1	0	0	0
ENSG00000259339	0	0	0	0
ENSG00000259340	0	0	0	0
ENSG00000259341	0	0	0	0
ENSG00000259342	0	0	0	0
ENSG00000259343	0	0	1	5
ENSG00000259344	6	53	10	47
ENSG00000259345	111	456	432	431
ENSG00000259346	0	0	0	0
ENSG00000259347	7	21	1	6
ENSG00000259348	0	0	0	0
ENSG00000259349	1	0	0	2
ENSG00000259350	0	0	0	0

ENSG00000259351	0	0	0	0
ENSG00000259352	0	0	0	0
ENSG00000259353	0	0	4	0
ENSG00000259354	0	0	0	0
ENSG00000259356	0	0	0	0
ENSG00000259357	0	561	464	212
ENSG00000259358	0	0	0	0
ENSG00000259359	0	0	0	0
ENSG00000259360	0	0	0	0
ENSG00000259361	0	0	0	0
ENSG00000259362	0	0	0	0
ENSG00000259363	2	0	1	0
ENSG00000259364	0	0	0	0
ENSG00000259365	0	0	0	0
ENSG00000259366	0	0	3	9
ENSG00000259367	0	0	0	0
ENSG00000259368	0	0	0	0
ENSG00000259369	0	0	0	0
ENSG00000259370	0	0	0	0
ENSG00000259371	0	0	0	0
ENSG00000259372	0	0	0	0
ENSG00000259374	0	0	0	0
ENSG00000259375	0	0	0	0
ENSG00000259376	3	0	2	1
ENSG00000259377	0	0	0	0
ENSG00000259378	0	0	3	0
ENSG00000259379	0	0	0	0
ENSG00000259380	60	694	721	704
ENSG00000259381	0	0	0	0
ENSG00000259382	0	0	0	0
ENSG00000259383	0	0	0	0
ENSG00000259384	1	0	0	0
ENSG00000259385	0	0	0	0
ENSG00000259387	0	0	0	0
ENSG00000259388	0	0	0	0
ENSG00000259389	0	0	0	0
ENSG00000259390	5	0	8	6
ENSG00000259392	0	0	0	0
ENSG00000259393	0	0	0	0
ENSG00000259394	0	0	0	0
ENSG00000259395	0	0	0	0
ENSG00000259396	0	0	0	0
ENSG00000259397	0	0	0	0
ENSG00000259398	0	0	0	0
ENSG00000259399	0	0	0	0
ENSG00000259400	0	0	0	0

ENSG00000259401	0	0	0	0
ENSG00000259402	0	0	0	0
ENSG00000259403	0	0	0	0
ENSG00000259404	0	0	0	6
ENSG00000259405	0	0	0	0
ENSG00000259407	0	19	1	0
ENSG00000259408	0	0	0	0
ENSG00000259409	0	0	0	0
ENSG00000259410	0	0	0	0
ENSG00000259411	0	0	0	0
ENSG00000259413	0	0	0	0
ENSG00000259414	0	0	0	0
ENSG00000259415	0	0	0	0
ENSG00000259416	0	0	0	0
ENSG00000259417	0	0	0	0
ENSG00000259418	0	0	0	0
ENSG00000259419	0	0	0	0
ENSG00000259420	0	0	0	0
ENSG00000259421	0	0	0	0
ENSG00000259422	0	0	0	0
ENSG00000259423	13	54	23	52
ENSG00000259424	0	0	0	0
ENSG00000259425	0	0	0	0
ENSG00000259426	0	0	0	0
ENSG00000259427	0	0	0	0
ENSG00000259428	0	0	0	0
ENSG00000259429	0	0	0	4
ENSG00000259430	0	0	0	0
ENSG00000259431	2	0	1	8
ENSG00000259432	0	0	0	0
ENSG00000259433	0	0	0	0
ENSG00000259434	0	0	0	0
ENSG00000259435	0	0	0	0
ENSG00000259436	2	0	2	0
ENSG00000259437	0	0	0	0
ENSG00000259438	0	14	1	1
ENSG00000259439	0	0	0	0
ENSG00000259440	0	0	0	0
ENSG00000259441	0	0	0	0
ENSG00000259442	0	0	0	0
ENSG00000259443	0	0	0	0
ENSG00000259444	0	0	0	0
ENSG00000259445	0	0	2	7
ENSG00000259446	0	0	0	0
ENSG00000259447	0	0	2	5
ENSG00000259448	4	0	1	0

ENSG00000259449	0	0	0	0
ENSG00000259450	9	0	36	15
ENSG00000259451	0	0	0	0
ENSG00000259452	0	0	0	0
ENSG00000259453	0	0	0	0
ENSG00000259454	0	0	0	0
ENSG00000259455	0	0	0	0
ENSG00000259456	1	0	7	13
ENSG00000259457	0	0	0	0
ENSG00000259458	0	0	0	0
ENSG00000259459	0	0	0	0
ENSG00000259460	0	0	0	0
ENSG00000259461	0	0	0	0
ENSG00000259462	0	0	0	0
ENSG00000259463	0	0	0	0
ENSG00000259464	0	0	0	0
ENSG00000259465	0	0	0	0
ENSG00000259466	0	0	0	0
ENSG00000259467	0	0	0	0
ENSG00000259468	0	0	1	0
ENSG00000259469	0	0	2	2
ENSG00000259470	0	0	0	0
ENSG00000259471	8	37	6	3
ENSG00000259472	0	0	0	0
ENSG00000259473	0	0	0	0
ENSG00000259474	0	0	0	0
ENSG00000259475	0	2	0	1
ENSG00000259476	5	0	8	8
ENSG00000259477	0	0	0	0
ENSG00000259478	0	0	0	0
ENSG00000259479	0	0	0	0
ENSG00000259480	0	0	2	8
ENSG00000259481	0	0	0	0
ENSG00000259482	0	0	0	0
ENSG00000259483	1316	1795	2911	3643
ENSG00000259484	4	11	7	12
ENSG00000259485	0	0	0	0
ENSG00000259488	0	50	17	11
ENSG00000259489	0	0	0	0
ENSG00000259490	0	0	0	0
ENSG00000259493	0	0	0	0
ENSG00000259494	4	0	0	0
ENSG00000259495	0	0	0	0
ENSG00000259496	0	17	0	0
ENSG00000259497	0	0	0	0
ENSG00000259498	3	30	37	41

ENSG00000259499	0	0	0	0
ENSG00000259500	0	0	0	0
ENSG00000259501	0	0	0	0
ENSG00000259502	0	0	0	0
ENSG00000259503	0	0	0	0
ENSG00000259504	0	0	1	0
ENSG00000259505	0	0	0	0
ENSG00000259507	0	0	0	0
ENSG00000259508	0	0	0	0
ENSG00000259509	0	0	0	0
ENSG00000259511	1	0	0	0
ENSG00000259512	0	0	0	0
ENSG00000259513	0	0	3	0
ENSG00000259514	0	0	0	0
ENSG00000259515	0	0	2	0
ENSG00000259516	0	0	0	0
ENSG00000259517	0	0	0	0
ENSG00000259518	0	0	0	0
ENSG00000259519	0	0	0	0
ENSG00000259520	0	0	0	0
ENSG00000259521	0	0	2	0
ENSG00000259522	0	0	0	0
ENSG00000259523	0	0	0	0
ENSG00000259524	0	0	0	0
ENSG00000259525	0	0	0	0
ENSG00000259526	0	0	0	0
ENSG00000259527	0	0	0	0
ENSG00000259528	0	0	0	0
ENSG00000259529	0	0	0	0
ENSG00000259530	0	0	0	0
ENSG00000259531	0	0	0	4
ENSG00000259532	0	0	0	0
ENSG00000259533	0	0	0	0
ENSG00000259534	0	0	0	0
ENSG00000259535	0	0	0	0
ENSG00000259536	0	0	2	0
ENSG00000259538	0	0	0	0
ENSG00000259539	0	0	0	0
ENSG00000259540	0	0	0	0
ENSG00000259541	0	0	0	0
ENSG00000259542	0	0	0	0
ENSG00000259543	0	0	0	0
ENSG00000259544	0	0	1	0
ENSG00000259545	0	0	0	0
ENSG00000259546	0	0	0	0
ENSG00000259547	0	0	0	0

ENSG00000259548	0	0	0	0
ENSG00000259549	0	0	0	0
ENSG00000259550	0	0	0	0
ENSG00000259551	0	0	0	0
ENSG00000259553	1	0	3	0
ENSG00000259554	0	0	0	0
ENSG00000259555	0	0	0	0
ENSG00000259556	1	6	18	38
ENSG00000259557	0	0	0	0
ENSG00000259558	0	0	0	0
ENSG00000259560	0	0	0	0
ENSG00000259561	0	0	0	0
ENSG00000259562	0	18	2	22
ENSG00000259563	0	0	0	0
ENSG00000259564	0	0	0	0
ENSG00000259565	0	0	0	0
ENSG00000259567	0	0	0	0
ENSG00000259568	0	0	0	0
ENSG00000259569	0	0	0	0
ENSG00000259570	0	0	0	0
ENSG00000259571	0	0	0	0
ENSG00000259572	0	0	0	0
ENSG00000259573	0	0	0	0
ENSG00000259575	0	0	0	0
ENSG00000259577	49	286	161	748
ENSG00000259579	0	0	0	0
ENSG00000259580	0	0	0	0
ENSG00000259581	0	0	2	0
ENSG00000259582	0	0	0	0
ENSG00000259583	6	6	14	18
ENSG00000259584	0	0	0	0
ENSG00000259585	0	0	3	1
ENSG00000259586	0	0	0	0
ENSG00000259587	0	0	0	0
ENSG00000259588	0	0	0	0
ENSG00000259589	0	92	25	10
ENSG00000259590	0	0	0	0
ENSG00000259591	0	0	0	0
ENSG00000259592	0	0	0	0
ENSG00000259593	0	0	0	0
ENSG00000259594	0	0	1	0
ENSG00000259595	0	16	59	27
ENSG00000259596	0	0	0	0
ENSG00000259597	0	0	0	0
ENSG00000259598	0	0	0	0
ENSG00000259599	0	0	1	1

ENSG00000259600	0	0	0	0
ENSG00000259601	0	0	0	0
ENSG00000259602	0	0	0	0
ENSG00000259603	0	0	0	0
ENSG00000259604	0	0	0	0
ENSG00000259605	0	0	0	0
ENSG00000259606	0	0	0	0
ENSG00000259607	0	0	0	0
ENSG00000259608	0	0	0	0
ENSG00000259609	0	0	0	0
ENSG00000259610	0	0	2	0
ENSG00000259611	0	0	0	0
ENSG00000259612	0	0	0	0
ENSG00000259613	0	0	0	0
ENSG00000259614	0	0	0	0
ENSG00000259615	0	0	0	0
ENSG00000259616	0	0	0	0
ENSG00000259617	0	0	0	0
ENSG00000259618	0	0	1	6
ENSG00000259619	0	0	0	0
ENSG00000259620	0	0	0	0
ENSG00000259621	0	0	0	0
ENSG00000259622	0	0	0	0
ENSG00000259623	0	0	0	0
ENSG00000259624	0	0	0	0
ENSG00000259626	0	0	0	0
ENSG00000259627	0	118	145	64
ENSG00000259628	1	0	0	0
ENSG00000259630	0	0	0	0
ENSG00000259631	0	0	0	0
ENSG00000259632	0	0	0	0
ENSG00000259633	0	0	0	0
ENSG00000259634	0	0	0	0
ENSG00000259635	0	0	0	0
ENSG00000259636	0	0	0	0
ENSG00000259637	0	0	0	0
ENSG00000259638	0	0	0	0
ENSG00000259639	2	0	0	3
ENSG00000259640	0	0	0	0
ENSG00000259641	2	0	0	0
ENSG00000259642	1	0	0	2
ENSG00000259644	0	0	0	0
ENSG00000259645	0	0	0	0
ENSG00000259646	0	0	0	0
ENSG00000259647	0	0	0	0
ENSG00000259648	0	0	0	0

ENSG00000259649	0	0	0	0
ENSG00000259650	0	0	0	0
ENSG00000259651	0	0	0	0
ENSG00000259652	0	0	0	0
ENSG00000259654	0	0	0	0
ENSG00000259655	0	0	0	0
ENSG00000259656	0	0	0	0
ENSG00000259657	1	0	0	0
ENSG00000259658	13	0	2	8
ENSG00000259659	0	0	0	0
ENSG00000259660	0	0	0	7
ENSG00000259661	0	54	24	14
ENSG00000259663	0	0	0	0
ENSG00000259664	0	0	0	0
ENSG00000259665	0	0	0	0
ENSG00000259666	0	0	0	0
ENSG00000259668	0	0	1	0
ENSG00000259669	0	0	0	0
ENSG00000259670	0	26	1	0
ENSG00000259671	0	0	0	0
ENSG00000259672	0	0	0	0
ENSG00000259673	0	0	1	2
ENSG00000259674	4	0	3	0
ENSG00000259675	0	0	0	0
ENSG00000259676	0	0	0	0
ENSG00000259677	0	0	0	0
ENSG00000259678	0	0	0	0
ENSG00000259679	0	0	0	0
ENSG00000259680	0	0	0	0
ENSG00000259681	0	0	0	0
ENSG00000259682	0	0	0	0
ENSG00000259683	0	0	0	0
ENSG00000259684	0	0	0	0
ENSG00000259685	0	0	0	0
ENSG00000259686	0	0	0	0
ENSG00000259687	1	0	0	0
ENSG00000259688	0	0	0	0
ENSG00000259689	0	0	0	0
ENSG00000259690	0	0	0	4
ENSG00000259691	0	0	0	0
ENSG00000259692	0	0	0	1
ENSG00000259694	0	0	0	0
ENSG00000259695	0	0	0	0
ENSG00000259696	0	0	0	0
ENSG00000259697	0	0	0	0
ENSG00000259698	0	0	0	0

ENSG00000259699	2	0	0	0
ENSG00000259700	0	0	0	0
ENSG00000259701	0	0	0	0
ENSG00000259702	0	0	0	0
ENSG00000259703	0	0	0	0
ENSG00000259704	23	0	6	2
ENSG00000259705	0	0	0	0
ENSG00000259706	0	0	1	0
ENSG00000259707	0	0	0	0
ENSG00000259708	0	0	0	0
ENSG00000259709	1	12	17	17
ENSG00000259710	0	0	0	0
ENSG00000259711	0	0	0	0
ENSG00000259712	0	0	0	0
ENSG00000259713	0	0	0	0
ENSG00000259714	0	0	0	0
ENSG00000259715	0	0	0	0
ENSG00000259716	0	344	161	108
ENSG00000259717	0	0	0	0
ENSG00000259719	203	2699	2529	4748
ENSG00000259720	0	0	0	0
ENSG00000259721	0	0	0	0
ENSG00000259722	0	0	0	0
ENSG00000259723	0	0	0	0
ENSG00000259724	0	0	0	0
ENSG00000259725	0	0	0	0
ENSG00000259726	0	0	0	0
ENSG00000259727	2	0	3	0
ENSG00000259728	5	9	37	3
ENSG00000259729	0	0	0	0
ENSG00000259730	0	0	0	0
ENSG00000259731	0	0	0	0
ENSG00000259732	0	0	0	0
ENSG00000259734	0	0	0	0
ENSG00000259735	0	0	0	0
ENSG00000259736	4	561	628	923
ENSG00000259737	0	0	1	0
ENSG00000259738	0	0	0	0
ENSG00000259739	0	0	0	0
ENSG00000259740	0	0	0	0
ENSG00000259742	0	0	0	0
ENSG00000259743	0	0	0	0
ENSG00000259744	0	0	0	0
ENSG00000259745	0	0	0	0
ENSG00000259746	0	0	0	3
ENSG00000259747	0	0	0	0

ENSG00000259749	0	0	0	0
ENSG00000259750	0	0	0	0
ENSG00000259751	0	14	4	2
ENSG00000259752	0	0	0	0
ENSG00000259753	0	0	0	0
ENSG00000259754	0	0	0	0
ENSG00000259755	0	0	5	0
ENSG00000259756	0	0	0	0
ENSG00000259757	0	0	0	0
ENSG00000259758	50	454	523	341
ENSG00000259759	0	0	0	0
ENSG00000259760	0	0	0	0
ENSG00000259761	0	0	0	0
ENSG00000259762	0	0	0	0
ENSG00000259763	0	0	0	0
ENSG00000259764	0	6	0	0
ENSG00000259767	0	0	0	0
ENSG00000259768	1	0	2	11
ENSG00000259769	0	0	0	0
ENSG00000259770	0	0	0	0
ENSG00000259771	0	0	0	0
ENSG00000259772	2	0	0	0
ENSG00000259773	0	29	23	16
ENSG00000259774	0	0	0	0
ENSG00000259775	5	8	2	1
ENSG00000259776	0	0	0	0
ENSG00000259777	0	0	0	0
ENSG00000259778	0	0	0	0
ENSG00000259779	0	0	0	0
ENSG00000259780	0	0	0	0
ENSG00000259781	4	670	947	1047
ENSG00000259782	0	0	0	0
ENSG00000259783	0	0	0	0
ENSG00000259784	0	0	0	0
ENSG00000259785	0	0	0	0
ENSG00000259786	0	0	0	22
ENSG00000259787	0	0	0	0
ENSG00000259788	0	0	0	0
ENSG00000259789	0	0	0	2
ENSG00000259790	1	0	0	0
ENSG00000259791	0	0	0	0
ENSG00000259792	0	0	0	0
ENSG00000259793	4	0	0	0
ENSG00000259795	0	0	0	0
ENSG00000259796	0	0	0	0
ENSG00000259797	0	0	0	0

ENSG00000259798	0	0	0	0
ENSG00000259799	0	0	0	0
ENSG00000259800	0	0	0	0
ENSG00000259801	0	0	0	0
ENSG00000259802	8	0	7	8
ENSG00000259803	0	0	1	1
ENSG00000259804	10	0	4	0
ENSG00000259805	0	0	0	0
ENSG00000259806	4	0	0	1
ENSG00000259807	0	0	0	0
ENSG00000259808	0	0	0	0
ENSG00000259809	0	0	0	0
ENSG00000259810	0	0	0	0
ENSG00000259811	0	0	0	0
ENSG00000259812	0	0	3	0
ENSG00000259813	0	0	1	0
ENSG00000259814	0	0	0	0
ENSG00000259815	0	0	0	0
ENSG00000259816	0	0	0	0
ENSG00000259817	0	0	0	0
ENSG00000259818	0	0	0	0
ENSG00000259819	0	41	0	0
ENSG00000259820	0	0	1	0
ENSG00000259821	0	0	0	0
ENSG00000259822	0	0	0	0
ENSG00000259823	0	0	0	0
ENSG00000259824	0	0	0	0
ENSG00000259825	0	0	0	0
ENSG00000259826	3	0	13	12
ENSG00000259827	0	0	0	0
ENSG00000259828	0	0	0	0
ENSG00000259829	0	0	0	0
ENSG00000259831	0	0	0	0
ENSG00000259832	0	0	0	0
ENSG00000259833	0	0	0	0
ENSG00000259834	491	1712	4062	1770
ENSG00000259836	0	0	0	0
ENSG00000259837	0	0	0	0
ENSG00000259838	0	0	0	0
ENSG00000259839	0	0	0	0
ENSG00000259840	0	0	0	0
ENSG00000259841	0	0	0	0
ENSG00000259842	0	0	0	0
ENSG00000259843	0	0	0	0
ENSG00000259844	0	0	0	0
ENSG00000259845	0	0	0	0

ENSG00000259846	0	0	65	0
ENSG00000259847	0	0	0	0
ENSG00000259848	8	0	0	0
ENSG00000259849	0	0	0	0
ENSG00000259850	14	43	33	81
ENSG00000259851	0	0	0	0
ENSG00000259852	0	0	0	0
ENSG00000259853	0	0	0	0
ENSG00000259854	0	0	0	0
ENSG00000259855	0	0	0	0
ENSG00000259856	0	0	0	0
ENSG00000259857	0	0	0	0
ENSG00000259858	0	0	0	0
ENSG00000259859	0	0	0	0
ENSG00000259861	0	0	0	0
ENSG00000259862	0	0	0	0
ENSG00000259863	0	0	0	0
ENSG00000259864	0	0	0	0
ENSG00000259865	1	0	14	17
ENSG00000259866	0	0	7	5
ENSG00000259867	0	19	0	0
ENSG00000259868	0	0	0	0
ENSG00000259869	105	265	68	220
ENSG00000259870	0	0	0	0
ENSG00000259871	0	0	0	0
ENSG00000259872	0	0	0	0
ENSG00000259873	0	0	0	0
ENSG00000259874	0	0	0	0
ENSG00000259875	0	0	0	0
ENSG00000259876	0	0	0	0
ENSG00000259877	3	0	23	24
ENSG00000259878	0	0	0	0
ENSG00000259879	0	0	0	0
ENSG00000259880	0	0	0	0
ENSG00000259881	0	0	0	0
ENSG00000259882	0	0	0	0
ENSG00000259883	0	0	0	0
ENSG00000259884	0	0	0	0
ENSG00000259885	0	0	0	0
ENSG00000259886	0	0	0	0
ENSG00000259887	0	0	0	0
ENSG00000259888	0	0	0	0
ENSG00000259889	0	0	0	0
ENSG00000259890	0	0	0	0
ENSG00000259891	1	0	0	0
ENSG00000259892	0	0	0	0

ENSG00000259893	0	0	0	0
ENSG00000259894	0	0	0	0
ENSG00000259895	0	0	0	0
ENSG00000259896	0	0	0	0
ENSG00000259897	0	0	0	0
ENSG00000259898	0	0	0	0
ENSG00000259899	0	0	0	0
ENSG00000259900	0	0	0	0
ENSG00000259901	1	0	4	0
ENSG00000259902	0	0	0	0
ENSG00000259903	0	0	0	0
ENSG00000259904	0	0	0	0
ENSG00000259905	0	0	0	3
ENSG00000259906	0	0	0	0
ENSG00000259907	4	0	2	0
ENSG00000259908	0	0	0	0
ENSG00000259909	0	0	0	0
ENSG00000259910	0	0	0	0
ENSG00000259912	0	0	0	0
ENSG00000259913	0	0	0	0
ENSG00000259914	0	0	0	0
ENSG00000259915	0	0	2	0
ENSG00000259916	0	0	0	0
ENSG00000259917	0	0	0	0
ENSG00000259918	0	1	2	0
ENSG00000259919	0	0	0	0
ENSG00000259920	8	0	11	1
ENSG00000259921	0	0	0	0
ENSG00000259922	0	0	0	0
ENSG00000259923	0	0	0	0
ENSG00000259924	0	88	23	42
ENSG00000259925	0	0	1	0
ENSG00000259926	0	0	0	0
ENSG00000259927	0	0	0	0
ENSG00000259928	0	0	0	0
ENSG00000259929	0	0	0	0
ENSG00000259930	0	0	0	0
ENSG00000259931	0	0	0	0
ENSG00000259932	0	0	0	0
ENSG00000259933	0	0	0	0
ENSG00000259934	0	0	0	0
ENSG00000259935	0	0	0	0
ENSG00000259937	0	0	0	0
ENSG00000259938	0	0	0	0
ENSG00000259939	0	0	0	0
ENSG00000259940	0	1	0	3

ENSG00000259941	0	1	58	7
ENSG00000259942	0	0	0	0
ENSG00000259943	36	177	98	165
ENSG00000259944	0	0	0	0
ENSG00000259945	0	0	0	0
ENSG00000259946	0	0	0	0
ENSG00000259947	0	0	0	0
ENSG00000259948	0	0	0	3
ENSG00000259950	0	0	0	0
ENSG00000259951	0	0	0	0
ENSG00000259952	0	0	5	4
ENSG00000259953	113	433	442	628
ENSG00000259954	0	0	0	0
ENSG00000259955	0	0	0	0
ENSG00000259956	0	0	0	0
ENSG00000259957	0	0	0	0
ENSG00000259958	0	0	0	0
ENSG00000259959	0	0	0	0
ENSG00000259960	0	0	0	0
ENSG00000259961	0	0	0	0
ENSG00000259962	0	0	0	0
ENSG00000259963	0	0	0	0
ENSG00000259964	0	15	0	0
ENSG00000259965	0	0	0	0
ENSG00000259966	0	0	0	0
ENSG00000259967	0	0	0	0
ENSG00000259968	0	0	0	0
ENSG00000259969	0	19	1	0
ENSG00000259970	0	0	0	0
ENSG00000259971	0	0	0	0
ENSG00000259972	0	0	0	0
ENSG00000259973	0	0	0	0
ENSG00000259974	0	0	0	0
ENSG00000259975	0	0	0	0
ENSG00000259976	1	0	0	5
ENSG00000259977	0	0	0	0
ENSG00000259978	0	0	0	0
ENSG00000259979	0	0	0	0
ENSG00000259980	0	0	0	0
ENSG00000259981	0	0	0	0
ENSG00000259982	0	0	0	0
ENSG00000259983	0	0	0	0
ENSG00000259984	0	0	0	0
ENSG00000259985	2	0	16	27
ENSG00000259986	4	0	11	9
ENSG00000259987	0	0	0	0

ENSG00000259988	0	0	0	0
ENSG00000259989	0	0	0	0
ENSG00000259990	0	0	0	0
ENSG00000259991	0	0	0	0
ENSG00000259992	0	0	0	0
ENSG00000259993	0	0	0	0
ENSG00000259994	0	8	0	0
ENSG00000259995	0	0	0	0
ENSG00000259996	0	0	0	0
ENSG00000259997	0	0	0	0
ENSG00000259998	0	0	0	0
ENSG00000259999	0	0	0	0
ENSG00000260000	0	28	6	13
ENSG00000260001	0	0	0	0
ENSG00000260002	0	0	0	0
ENSG00000260003	0	0	0	0
ENSG00000260004	0	0	0	0
ENSG00000260005	0	0	0	0
ENSG00000260006	5	10	32	26
ENSG00000260007	0	0	0	0
ENSG00000260008	1	0	0	0
ENSG00000260009	0	0	0	0
ENSG00000260010	0	0	0	0
ENSG00000260011	0	0	0	0
ENSG00000260012	0	0	0	0
ENSG00000260013	0	0	0	0
ENSG00000260014	0	0	0	0
ENSG00000260015	0	0	0	0
ENSG00000260016	0	0	0	0
ENSG00000260017	0	15	6	0
ENSG00000260018	0	0	0	0
ENSG00000260019	63	120	138	214
ENSG00000260020	0	0	0	0
ENSG00000260021	0	0	0	0
ENSG00000260022	0	0	0	0
ENSG00000260023	0	0	0	0
ENSG00000260024	0	0	0	0
ENSG00000260025	0	0	0	0
ENSG00000260026	0	0	0	0
ENSG00000260027	3	0	0	0
ENSG00000260028	0	0	0	0
ENSG00000260029	0	0	0	0
ENSG00000260030	1	0	0	0
ENSG00000260031	0	0	0	0
ENSG00000260032	9469	62432	76691	66644
ENSG00000260033	0	0	0	0

ENSG00000260034	0	66	8	17
ENSG00000260035	0	0	0	0
ENSG00000260036	0	0	0	0
ENSG00000260037	0	0	1	0
ENSG00000260038	0	0	0	0
ENSG00000260039	0	0	0	0
ENSG00000260040	0	0	0	0
ENSG00000260041	0	0	0	0
ENSG00000260042	0	0	0	0
ENSG00000260043	0	0	0	0
ENSG00000260045	0	0	0	0
ENSG00000260046	0	0	0	0
ENSG00000260047	0	0	0	0
ENSG00000260048	0	0	0	0
ENSG00000260049	0	0	0	0
ENSG00000260050	0	0	0	0
ENSG00000260051	0	0	0	0
ENSG00000260052	0	0	0	0
ENSG00000260053	0	0	0	0
ENSG00000260054	2	0	0	0
ENSG00000260055	0	0	0	0
ENSG00000260056	0	0	0	0
ENSG00000260057	0	0	0	0
ENSG00000260058	0	0	0	0
ENSG00000260059	0	0	0	0
ENSG00000260060	0	0	0	0
ENSG00000260061	0	0	0	0
ENSG00000260062	0	0	0	0
ENSG00000260063	0	0	4	0
ENSG00000260064	0	0	0	0
ENSG00000260065	175	1221	1283	1410
ENSG00000260066	0	0	0	0
ENSG00000260067	0	0	0	0
ENSG00000260068	0	0	0	0
ENSG00000260069	0	0	0	0
ENSG00000260070	0	0	0	0
ENSG00000260071	0	0	0	0
ENSG00000260072	1	0	0	0
ENSG00000260073	0	0	0	0
ENSG00000260074	0	0	0	0
ENSG00000260075	0	0	0	2
ENSG00000260076	0	0	0	0
ENSG00000260077	763	442	395	490
ENSG00000260078	5	0	6	0
ENSG00000260079	0	0	0	0
ENSG00000260080	0	0	0	0

ENSG00000260081	0	0	0	0
ENSG00000260082	0	0	0	0
ENSG00000260083	0	0	0	0
ENSG00000260084	0	0	0	0
ENSG00000260086	3	0	2	0
ENSG00000260087	0	0	0	0
ENSG00000260088	1	0	0	0
ENSG00000260089	0	0	0	0
ENSG00000260090	0	0	0	0
ENSG00000260091	0	0	0	0
ENSG00000260092	0	0	0	0
ENSG00000260093	0	0	0	0
ENSG00000260094	0	0	0	0
ENSG00000260095	0	0	0	0
ENSG00000260096	0	0	0	0
ENSG00000260097	0	0	0	0
ENSG00000260099	0	0	0	0
ENSG00000260100	0	0	0	0
ENSG00000260101	34	33	88	71
ENSG00000260102	0	0	0	0
ENSG00000260103	8	20	34	31
ENSG00000260104	0	0	0	0
ENSG00000260105	0	0	0	0
ENSG00000260106	0	0	0	0
ENSG00000260107	0	0	0	0
ENSG00000260108	0	0	0	0
ENSG00000260109	0	0	0	0
ENSG00000260111	0	0	0	0
ENSG00000260112	0	0	0	0
ENSG00000260113	0	0	1	0
ENSG00000260114	0	0	5	12
ENSG00000260115	0	0	0	0
ENSG00000260116	0	0	0	0
ENSG00000260117	0	0	0	0
ENSG00000260118	0	0	0	2
ENSG00000260119	0	0	0	0
ENSG00000260120	0	0	0	0
ENSG00000260121	0	0	0	0
ENSG00000260122	0	0	0	0
ENSG00000260123	0	0	0	0
ENSG00000260124	0	0	0	0
ENSG00000260125	0	0	0	0
ENSG00000260126	0	0	0	0
ENSG00000260128	0	1	4	5
ENSG00000260129	0	0	0	0
ENSG00000260130	0	0	0	0

ENSG00000260131	0	0	0	0
ENSG00000260132	0	0	0	0
ENSG00000260133	65	87	34	129
ENSG00000260134	0	0	0	0
ENSG00000260135	0	0	0	0
ENSG00000260136	0	0	0	0
ENSG00000260137	0	0	0	0
ENSG00000260138	0	0	0	0
ENSG00000260139	0	0	0	0
ENSG00000260141	0	26	2	3
ENSG00000260142	0	0	0	0
ENSG00000260143	0	0	0	0
ENSG00000260144	0	0	7	2
ENSG00000260145	0	0	0	0
ENSG00000260146	0	0	0	0
ENSG00000260147	0	0	0	0
ENSG00000260148	0	0	0	0
ENSG00000260150	0	0	0	0
ENSG00000260151	0	0	0	0
ENSG00000260152	0	0	0	0
ENSG00000260153	0	0	0	0
ENSG00000260155	0	0	0	0
ENSG00000260156	0	0	1	0
ENSG00000260157	0	0	0	0
ENSG00000260158	0	0	1	0
ENSG00000260159	0	0	0	0
ENSG00000260160	1	0	0	2
ENSG00000260161	0	0	0	0
ENSG00000260162	0	0	0	0
ENSG00000260163	0	0	0	0
ENSG00000260164	0	0	0	0
ENSG00000260165	0	0	0	0
ENSG00000260166	0	0	0	0
ENSG00000260167	0	0	0	0
ENSG00000260168	0	0	0	0
ENSG00000260169	0	0	0	0
ENSG00000260170	0	0	0	0
ENSG00000260171	0	0	0	0
ENSG00000260172	0	0	0	0
ENSG00000260173	0	0	0	0
ENSG00000260174	0	0	0	0
ENSG00000260175	0	0	0	0
ENSG00000260176	0	0	0	0
ENSG00000260177	0	0	0	0
ENSG00000260178	0	0	0	0
ENSG00000260179	0	0	0	0

ENSG00000260181	0	0	0	0
ENSG00000260182	0	0	0	0
ENSG00000260183	0	0	0	0
ENSG00000260184	0	0	0	0
ENSG00000260185	0	0	2	4
ENSG00000260186	0	0	0	0
ENSG00000260187	0	0	1	0
ENSG00000260188	1	0	0	0
ENSG00000260189	0	0	0	0
ENSG00000260190	0	0	0	0
ENSG00000260191	0	0	0	0
ENSG00000260192	0	0	0	0
ENSG00000260193	0	0	0	0
ENSG00000260194	0	0	0	0
ENSG00000260195	0	0	0	0
ENSG00000260196	0	0	0	0
ENSG00000260197	0	12	0	2
ENSG00000260198	0	0	0	0
ENSG00000260199	0	0	0	0
ENSG00000260200	0	0	0	0
ENSG00000260201	0	0	0	0
ENSG00000260202	0	0	0	0
ENSG00000260204	0	0	0	0
ENSG00000260205	0	0	0	0
ENSG00000260206	0	0	0	0
ENSG00000260207	0	0	0	0
ENSG00000260208	0	0	0	0
ENSG00000260209	0	0	0	0
ENSG00000260211	0	0	0	0
ENSG00000260212	0	0	0	0
ENSG00000260213	0	0	0	0
ENSG00000260214	3	0	6	15
ENSG00000260215	0	0	0	0
ENSG00000260216	0	0	0	0
ENSG00000260217	0	0	0	0
ENSG00000260218	0	0	0	0
ENSG00000260219	0	0	0	0
ENSG00000260220	0	0	0	0
ENSG00000260221	0	0	0	0
ENSG00000260222	0	0	0	0
ENSG00000260223	0	0	0	0
ENSG00000260224	0	0	0	0
ENSG00000260225	0	0	0	0
ENSG00000260226	0	0	0	0
ENSG00000260227	0	0	0	0
ENSG00000260228	2	0	0	0

ENSG00000260229	0	0	0	0
ENSG00000260230	1	0	0	10
ENSG00000260231	229	376	336	154
ENSG00000260232	0	0	0	0
ENSG00000260233	2	0	0	0
ENSG00000260234	0	0	0	0
ENSG00000260235	0	0	0	0
ENSG00000260236	1	0	0	0
ENSG00000260237	0	0	0	0
ENSG00000260238	0	0	0	0
ENSG00000260239	0	0	0	0
ENSG00000260240	0	0	0	0
ENSG00000260241	0	0	0	0
ENSG00000260242	0	0	0	0
ENSG00000260244	307	888	2421	2834
ENSG00000260245	0	0	0	0
ENSG00000260246	0	0	0	0
ENSG00000260247	0	0	0	0
ENSG00000260248	0	0	0	0
ENSG00000260249	49	35	23	83
ENSG00000260250	0	0	0	0
ENSG00000260251	0	0	0	0
ENSG00000260252	0	0	7	0
ENSG00000260253	0	0	0	0
ENSG00000260254	0	0	1	0
ENSG00000260255	0	0	0	0
ENSG00000260256	0	0	0	0
ENSG00000260257	9	0	35	26
ENSG00000260258	0	0	0	0
ENSG00000260259	0	0	0	0
ENSG00000260260	35	15	3	11
ENSG00000260261	0	0	0	0
ENSG00000260262	0	0	0	0
ENSG00000260263	3	0	2	4
ENSG00000260264	0	0	0	0
ENSG00000260265	0	0	0	2
ENSG00000260266	1	0	0	0
ENSG00000260267	0	0	3	3
ENSG00000260268	0	0	0	0
ENSG00000260269	1	1	1	0
ENSG00000260271	0	0	0	0
ENSG00000260272	0	0	0	0
ENSG00000260273	2	0	2	0
ENSG00000260274	2	17	11	13
ENSG00000260275	0	0	0	0
ENSG00000260276	0	1	1	0

ENSG00000260277	0	0	0	0
ENSG00000260278	1	0	0	0
ENSG00000260279	0	0	0	0
ENSG00000260280	0	0	0	0
ENSG00000260281	0	42	89	31
ENSG00000260282	0	0	0	0
ENSG00000260283	0	0	0	0
ENSG00000260284	0	0	0	0
ENSG00000260285	0	0	0	0
ENSG00000260286	0	0	0	0
ENSG00000260287	0	0	0	0
ENSG00000260288	2	0	0	0
ENSG00000260289	0	0	0	0
ENSG00000260290	0	0	0	0
ENSG00000260291	0	0	0	0
ENSG00000260293	0	0	0	0
ENSG00000260294	0	0	0	0
ENSG00000260295	0	0	0	0
ENSG00000260296	0	0	0	0
ENSG00000260297	0	0	0	0
ENSG00000260298	0	0	0	0
ENSG00000260299	0	0	0	0
ENSG00000260300	0	0	0	0
ENSG00000260301	0	0	0	0
ENSG00000260302	0	0	0	0
ENSG00000260303	0	0	0	0
ENSG00000260304	0	0	0	0
ENSG00000260305	0	0	0	0
ENSG00000260306	0	0	0	0
ENSG00000260307	0	0	0	0
ENSG00000260308	0	0	0	0
ENSG00000260309	0	0	0	0
ENSG00000260310	0	0	0	0
ENSG00000260311	0	0	0	0
ENSG00000260312	0	0	0	0
ENSG00000260313	0	0	0	0
ENSG00000260314	0	0	0	0
[truncated: 487,161 more chars]
